# Litteratura Coleopterologica (1758–1900): a guide to selected books related to the taxonomy of Coleoptera with publication dates and notes

**DOI:** 10.3897/zookeys.583.7084

**Published:** 2016-04-25

**Authors:** Yves Bousquet

**Affiliations:** 1 Agriculture and Agri-Food Canada, Central Experimental Farm, Ottawa, Ontario K1A 0C6, Canada

**Keywords:** Coleoptera, beetles, literature, dates of publication, biographies

## Abstract

Bibliographic references to works pertaining to the taxonomy of Coleoptera published between 1758 and 1900 in the non-periodical literature are listed. Each reference includes the full name of the author, the year or range of years of the publication, the title in full, the publisher and place of publication, the pagination with the number of plates, and the size of the work. This information is followed by the date of publication found in the work itself, the dates found from external sources, and the libraries consulted for the work. Overall, more than 990 works published by 622 primary authors are listed. For each of these authors, a biographic notice (if information was available) is given along with the references consulted.

## Introduction

One of the fundamental principles of the *International Code of Zoological Nomenclature* (hereafter called “*Code*”) is the Principle of Priority (Article 23.1). The valid name of a taxon is the oldest available name applied to it, unless that name has been invalidated or another name is given precedence by a provision of the *Code* or by a ruling of the International Commission on Zoological Nomenclature (herein “the Commission”). Although considered of trivial importance by some zoologists, dates of publication of works that contain new scientific names are important in establishing priority.

The scope of this work focuses mainly on determining dates of publication, as precisely as possible, of works, such as books, pamphlets, dissertations, catalogues, dictionaries and school programs, relating to the taxonomy and nomenclature of Coleoptera from 1 January 1758, the starting date of zoological nomenclature (ICZN 1999: Article 3), to the end of 1900. Works published in periodical literature, such as journals and proceedings of meetings, are not listed nor are reprints of articles unless the reprint version appeared first or is the version most often cited in the literature. Works issued in journals with separate pagination or as supplements are included in this bibliography because they are usually bound separately from the journal issues. Particular attention has been given to works containing new taxa (at the family, generic and species levels, including fossils), new synonymies and type species designations for genus-group taxa. Besides the dates of publication, this bibliography provides exact titles, publisher’s names if listed, places of publication, paginations (including number of plates) and sizes for all works included. Brief biographic notes of the authors are included if information was available.

## Format

The format used in this book is essentially similar to that used by Evenhuis (1997a, b). For each publication listed, the following information is provided.

Author Name. The name of the author of the publication, or section of the work, is given in full. Variations in orthography are listed in square brackets. If more than one author is responsible for the publication, their names are listed in square brackets before the title of the work.

Biographical Note. This section includes biographical notes about the author. First, the places and dates of birth and death are given in parentheses if available^1^. Then a brief biographical sketch follows, usually including the current location of the author’s collection if known^2^. The section concludes with a limited selection of *references* pertaining to the biography of the author.

Literature Citation. The following information is provided for every work cited.

*Year(s)*. The actual year of publication or range of years if the work was issued in parts is listed. If the year is uncertain, it is given in brackets.

*Title*. The full title of the work is listed taken from the title page. Alternative titles from additional title pages are indicated in the remarks section.

*Imprint*. The publisher(s)^3^ and place(s) of publication of the work are listed in the original orthography.

*Pagination and plates.* Complete pagination of the work or section of a work is given. Unnumbered pages are counted and the number given in square brackets. The number of plates included in the book or associated with it is provided.

*Size*. The signature size of the work is listed in parentheses using the standard denotations folio, 4to (quarto), 8vo (octavo), 12mo (duodecimo), etc. This information was gathered mainly from Hagen (1862, 1863), Derksen and Scheiding (1963) and Derksen and Scheiding-Göllner (1965, 1968, 1972).


 Date of publication [DP]. This section, usually given after the signature size or for works issued in parts in the remarks section, lists dates of publication found during this study. The first one is the date (usually indicated only by the year) on the title page^4^. It is followed in parentheses by the date listed in the preliminaries, such as the preface or the dedication, or on the last printed text-page (colophon) if it exists, which is cited here simply to denote that a particular work could not have been published before that date. This is followed chronologically by the dates found in accounts of society meetings, in journals or in other works. If the date on the title page is the earliest known date, then this date is to be adopted as the date of publication of the work unless there is evidence that the work, or part of the work, was indeed published later. If the earliest date known is the year only, then it is assumed that the work was published on the last day of that year, as stated in article 21.3.2 of the *Code* (ICZN 1999).

The date of publication is defined in the *Code* (ICZN 1999: 102) as “the date on which copies of the work become available by purchase or free distribution.” Unless listed on the work itself, most dates found in this study are not the actual dates of publication but dates on which works are demonstrated to be in existence as published works. Since most dates found are from published lists of works received by societies or institutions, from contemporary reviews in journals, or from national registers of publications, such as the *Bibliographie de la France* or the *Allgemeine Bibliographie für Deutschland*, there is sometimes a significant gap between the actual date of publication and that reported. This is particularly true for journals publishing reviews of works. Nevertheless, the earliest dates reported in this study, if earlier than on the title pages, should be considered as dates of publication until better dating can be found.

Source. The libraries consulted for each particular work treated in this publication are listed in abbreviated form (see “source” section below).

Remarks. This section provides information relevant to the work such as alternate title pages, subsequent editions, translations, Commission actions, etc. Books issued in parts are often detailed, with the date of publication of each part, in this section. Subsequent editions that contained new or significant taxonomic information may be cited separately.

## Sources

A number of libraries were visited during this study to consult books in order to provide accurate title and other relevant information. In addition, several books were consulted using digital databases available on the Internet. They are referred to in the text by the following abbreviations.

### Libraries

AMNH
 American Museum of Natural History Library, New York, New York, USA 


ANSP
 The Ewell Sale Stewart Library, Academy of Natural Sciences of Drexel University, Philadelphia, Pennsylvania, USA 


ANXL
 Library Annex, Cornell University, Ithaca, New York, USA 


BOT
 Botany Libraries, Harvard University, Cambridge, Massachusetts, USA 


BYU
 Harold B. Lee Library, Brigham Young University, Provo, Utah, USA 


CAKL
 Carl A. Kroch Library, Cornell University, Ithaca, New York, USA 


CMNL
 Canadian Museum of Nature Library, Gatineau, Quebec, Canada 


CMLE
 Comstock Memorial Library of Entomology, Cornell University, Ithaca, New York, USA [content has now been transferred to various libraries at Cornell University] 


CNC
 Canadian Agriculture Library (Entomology), Canadian National Collection of Insects, Arachnids and Nematodes, Agriculture and Agri-Food Canada, Ottawa, Ontario, Canada


HOU
 Houghton Library, Harvard University, Cambridge, Massachusetts, USA 


LAC
 Library and Archives Canada, Ottawa, Ontario, Canada 


MANL
 Mann Library, Cornell University, Ithaca, New York, USA 


MCZ
 Ernst Mayr Library, Museum of Comparative Zoology, Harvard University, Cambridge, Massachusetts, USA 


NAL
 National Agricultural Library, Beltsville, Maryland, USA 


NCSU
 D.H. Hill Library, North Carolina State University, Raleigh, North Carolina, USA 


OLIL
 Olin Library, Cornell University, Ithaca, New York, USA 


SME
 Aleš Smetana personal library, Ottawa, Ontario, Canada 


URIL
 Uris Library, Cornell University, Ithaca, New York, USA 


WID
 Widener Library, Harvard University, Cambridge, Massachusetts, USA 


YB
 Yves Bousquet personal library, Ottawa, Ontario, Canada 


### Digital libraries^5^

ARC
 Internet Archive 


BDH
 Biblioteca Digital Hispánica 


BHL
 Biodiversity Heritage Library 


BNDC
 Biblioteca Nacional Digital de Chile 


CSIC
 Red de bibliotecas del Consejo Superior de Investigaciones Científicas de España 


DFG
 Deutsche Forschungsgemeinschaft 


DORIA
 National Library of Finland 


EUR
 Europeana 


GAL
 Gallica 


GB
 Google Books 


GDZ
 Göttinger Digitalisierungszentrum 


HATH
 Hathi Trust Digital Library 


MDZ
 Münchener Digitalisierungszentrum 


SBB
 Staatsbibliothek zu Berlin 


SDL
 Swiss Digital Library [e-rara.ch] 


UFM
 Digital Sammlungen Universität Frankfurt am Main 


ULBD
 Universitäts- und Landesbibliothek Düsseldorf 


ULBM
 Univ- und Landesbibliothek Münster 


Dates of publication were obtained from the works themselves or from secondary sources, such as published contemporary notices or reviews, advertisements in recording journals or other books, and published lists of works received by societies or institutions. Dates found from secondary sources are followed by the abbreviated title of the journal or the society name. The abbreviations used are:

### Societies^6^

*Acad Imp Sci Lyon*: Académie Impériale des Sciences, Belles-Lettres & Arts de Lyon (Séance publique).

*Acad Imp Sci St-Pétersb*: Académie Impériale des Sciences de St.-Pétersbourg (Bulletin Scientifique; Bulletin de la Classe historico-philologique).

*Acad Nat Sci Phila*: Academy of Natural Sciences, Philadelphia (Proceedings).

*Acad R Sci Belg*: Académie Royale des Sciences, des Lettres et des Beaux-Arts de Belgique [Brussels] (Bulletin). Previously as “Académie Royale des Sciences et Belles-Lettres de Bruxelles” [*q.v.*].

*Acad R Sci Brux*: Académie Royale des Sciences et Belles-Lettres de Bruxelles [Brussels] (Bulletins). Subsequently as “Académie Royale des Sciences, des Lettres et des Beaux-Arts de Belgique” [*q.v.*].

*Acad R Sci Turin*: Académie Royale des Sciences [Turin] (Mémoires historiques).

*Acad Sci Fr*: Académie des Sciences [Paris] (Comptes-Rendus des séances; Procès-verbaux des séances).

*Accad Lincei*: Accademia dei Lincei [Rome] (Rendiconti).

*Amer Ent Soc*: American Entomological Society [Philadelphia] (Proceedings).

*Amer Phil Soc*: American Philosophical Society [Philadelphia] (Proceedings).

*Ashmolean Soc*: Ashmolean Society [Oxford] (Proceedings).

*Asia Soc Beng*: Asiatic Society of Bengal [Calcutta] (Proceedings).

*Boston Soc Nat Hist*: Boston Society of Natural History (Proceedings).

*Brook Ent Soc*: Brooklyn Entomological Society (Bulletin).

*Českos Akad Věd*: Československé Akademie Věd [Prague] (Věstník).

*Ent Soc Lond*: Entomological Society of London (Proceedings).

*Ent Soc Phila*: Entomological Society of Philadelphia (Proceedings).

*Ent Ver Stettin*: Entomologischer Verein zu Stettin (Vereinsangelegenheiten).

*Freun Naturwiss*: Freunden der Naturwissenschaften in Wien (Berichte über die Mittheilungen).

*Geol Soc Lond*: Geological Society of London (Transactions).

*Gesell Erdk Berl*: Gesellschaft für Erdkunde zu Berlin (Verhandlungen).

*Gesell Naturfors Freun Berl*: Gesellschaft Naturforschender Freunde zu Berlin (Sitzungsberichte).

*Ist Ven Sci*: Istituto Veneto di Scienze, Lettere ed Arti (Atti delle adunanze).

*Kaiser Akad Wiss*: Kaiserliche Akademie der Wissenschaften [Vienna] (Sitzungsberichte).

*Kaiser Geol Reichs*: Kaiserlich-Königliche Geologische Reichsanstalt [Vienna] (Verhandlungen).

*Königl Akad Wiss Berl*: Königlich-Preussische Akademie der Wissenschaften zu Berlin (Bericht über die Bekanntmachung geeigneten Verhandlungen).

*Königl Akad Wiss Münc*: Königlich-Bayerische Akademie der Wissenschaften zu München (Sitzungsberichte).

*Linn Soc NS Wales*: Linnean Society of New South Wales [Sydney] (Proceedings).

*Nat Inst Sci*: National Institution for the Promotion of Science [Washington DC] (Proceedings).

*Naturfors Gesell Zürich*: Naturforschende Gesellschaft in Zürich (Vierteljahrsschrift).

*Naturfors Ver Brünn*: Naturforschender Verein in Brünn (Verhandlungen).

*Naturfors Ver Riga*: Naturforschender Verein zu Riga (Correspondenzblatt).

*Naturhist Ver Rhein West*: Naturhistorischer Verein der preussischen Rheinlande und Westfalens [Bonn] (Correspondenzblatt).

*Naturwiss Gesell Isis*: Naturwissenschaftliche Gesellschaft Isis Dresden (Sitzungs-Berichte).

*Naturwiss Ver Bremen*: Naturwissenschaftlicher Verein zu Bremen (Jahresbericht).

*Naturwiss Ver Sach Thür*: Naturwissenschaftlicher Verein für die Provinz Sachsen und Thüringen [Halle] (Correspondenzblatt).

*Nederl Ent Ver*: Nederlandsche Entomologische Vereniging [Leiden] (Inhouds-Opgave van Werken).

*Nied Gesell Natur Heil*: Niederrheinische Gesellschaft für Natur- und Heilkunde zu Bonn (Sitzungsberichte).

*Phys Gesell Berl*: Physikalische Gesellschaft in Berlin (Verhandlungen).

*Phys-Ökon Gesell König*: Physikalisch-Ökonomische Gesellschaft zu Königsberg (Bericht über die in den Sitzungen).

*R Accad Sci Torino*: Reale Accademia delle Scienze di Torino (Memorie).

*R Astr Soc*: Royal Astronomical Society [London] (Memoirs).

*R Geog Soc*: Royal Geographical Society [London] (Proceedings).

*R Soc Edinb*: Royal Society of Edinburgh (Transactions).

*R Soc Lond*: Royal Society of London (Philosophical Transactions; Proceedings).

*R Soc Victoria*: Royal Society of Victoria [Melbourne] (Proceedings).

*Schweiz Naturfors Gesell*: Schweizerische Naturforschende Gesellschaft [Bern] (Verhandlungen).

*Sieben Ver*: Siebenbürgischer Verein für Naturwissenschaften zu Hermannstadt (Verhandlungen und Mittheilungen).

*Soc Agr Fr*: Société Centrale d’Agriculture de France / Société Nationale d’Agriculture de France [Paris] (Bulletin des Séances).

*Soc Agr Hist Nat Arts Lyon*: Société d’Agriculture, Histoire Naturelle et Arts utiles de Lyon (Procès-verbaux).

*Soc Amis Sci Nat Rouen*: Société des Amis des Sciences Naturelles de Rouen (Bulletin).

*Soc Bot Fr*: Société Botanique de France [Paris] (Bulletin).

*Soc Encou Ind Nat*: Société d’Encouragement pour l’Industrie Nationale [Paris] (Bulletin).

*Soc Ent Belg*: Société Entomologique de Belgique [Brussels] (Comptes rendus des Séances).

*Soc Ent Fr*: Société Entomologique de France [Paris] (Bulletin).

*Soc Espa Hist Nat*: Sociedad Española de Historia Natural [Madrid] (Actas).

*Soc Fauna Flora Fenn*: Societas pro Fauna et Flora Fennica [Helsinki] (Meddelanden).

*Soc Géog Fr*: Société de Géographie [Paris] (Compte Rendu des séances; Bulletin).

*Soc Géol Fr*: Société Géologique de France [Paris] (Bulletin).

*Soc Hist Nat Autun*: Société d’Histoire naturelle d’Autun (Procès-verbaux des séances).

*Soc Imp Nat Moscou*: Société Impériale des Naturalistes de Moscou (Bulletin).

*Soc Imp Zool Acclim*: Société Impériale Zoologique d’Acclimatation [Paris] (Procès-Verbaux).

*Soc Linn Nord Fr*: Société Linnéenne du nord de la France [Amiens] (Bulletin).

*Soc Linn Norm*: Société Linnéenne de Normandie [Caen] (Bulletin).

*Soc Philom*: Société Philomatique [Paris] (Bulletin des Sciences; Nouveau Bulletin des Sciences).

*Soc R Belg Géog*: Société Royale Belge de Géographie [Brussels] (Bulletin).

*Soc R Bot Belg*: Société Royale de Botanique de Belgique [Brussels] (Bulletin).

*Soc R Malac Belg*: Société Royale Malacologique de Belgique [Brussels] (Bulletin des Séances).

*Soc Rur Argent*: Sociedad Rural Argentina [Buenos Aires] (Anales).

*Soc Sci Méd Ouest*: Société Scientifique et Médicale de l’Ouest [Rennes] (Bulletin).

*Soc Stat Mars*: Société de Statistique de Marseille (Répertoire des Travaux).

*Soc Zool Fr*: Société Zoologique de France [Paris] (Procès-verbaux).

*Ver Natur Presburg*: Verein für Naturkunde zu Presburg (Sitzungsberichte).

*Ver Natur Zwickau*: Verein für Naturkunde zu Zwickau (Jahresbericht).

*Ver Sieb Land*: Verein für Siebenbürgische Landeskunde [Hermannstadt] (Korrespondenzblatt).

*Vetens Akad*: Kungliga Vetenskaps-Akademien [Stockholm] (Förhandlingar).

*Zool-Bot Gesell Wien*: Zoologisch-Botanische Gesellschaft in Wien (Verhandlungen).

### Journals

*Abeille*: L’Abeille. Mémoires d’Entomologie [Paris: 1864–1900+]. As “L’Abeille. Journal d’Entomologie” (1875–1900+).

*Acad Rev*: Academische Revue [Munich: 1894–1897].

*Academy*: The Academy. A Weekly Review of Literature, Science, and Art [London: 1869–1902].

*Affic Lyon*: Affiches de Lyon [Lyon: 1750–1821].

*Album*: L’Album. Journal des Arts, des Modes et des Théatres [Paris: 1821–1823].

*Alg Konst Letter*: Algemeene Konst- en Letter-Bode [Haarlem: 1788–1862].

*Allg Anz*: Allgemeiner Anzeiger und Nationalzeitung der Deutschen [Gotha: 1791–1848]. As “Allgemeiner Anzeiger der Deutschen” (1814–1829).

*Allg Bericht Bücher*: Allgemeiner Bericht von neuen Büchern, Landkarten, Musikalien und andern Kunstartikeln [Heidelberg: 1812–1821].

*Allg Bibl*: Allgemeine Bibliographie. Monatliches Verzeichniss der wichtigern neuen Erscheinungen der Deutschen und Ausländischen Literatur [Leipzig: 1856–1900+].

*Allg Bibl Deutsch*: Allgemeine Bibliographie für Deutschland [Leipzig: 1836–1892]. Preceded by “Bibliographie von Deutschland.”

*Allg Forst Jagd Ztg*: Allgemeine Forst- und Jagd-Zeitung [Frankfurt am Main: 1825–1900+].

*Allg Lit Anz*: Allgemeiner Litterarischer Anzeiger [Leipzig: 1796–1801].

*Allg Lit Ztg*: Allgemeine Literatur-Zeitung [Jena/Halle: 1785–1849]. Succeeded by “Literarisches Centralblatt für Deutschland” [*q.v.*].

*Allg Lit Ztg Kath*: Allgemeine Literatur-Zeitung, Zunächst fūr das Katholische Deutschland [Vienna: 1854–1873].

*Allg Monats Deutsch*: Allgemeiner Monats-Bericht für Deutschland [Weimar: 1811–1837]. Preceded by “Monats-Bericht des F.S. privil. Landes-Industrie-Comptoirs” [*q.v.*].

*Allg Press Ztg*: Allgemeine Press-Zeitung [Leipzig: 1840–1845].

*Allg Schul Ztg*: Allgemeine Schul-Zeitung [Darmstadt: 1824–1881].

*Allg Verz Bücher*: Allgemeines Verzeichniss neuer Bücher mit kurzen Anmerkungen [Leipzig: 1776–1784].

*Allg Ztg*: Allgemeine Zeitung [Tübingen/Stuttgart/Augsburg/Munich: 1798–1900+].

*Amer Ent*: The American Entomologist [St. Louis/New York: 1868–1870, 1880].

*Amer J Sci Arts*: The American Journal of Science and Arts [New Haven: 1818–1900+].

*Amer Monthly Mag*: The American Monthly Magazine and Critical Review [New York: 1817–1819].

*Amer Nat*: The American Naturalist [Salem/Philadelphia/Boston: 1867–1900+].

*Amer Publ Circ*: American Publishers’ Circular and Literary Gazette [New York/Philadelphia: 1855–1872]. As “American Literary Gazette and Publishers’ Circular” (1863–1872).

*Amer Quart Obs*: The American Quarterly Observer [Boston: 1833–1834].

*Amts Reg Bromberg*: Amtsblatt der Königlichen Regierung zu Bromberg [Bromberg: 1815–1900+].

*Amts Reg Frankfurt*: Amtsblatt der Königlich Preussischen Regierung zu Frankfurt a.O. [Frankfurt an der Oder: 1816–1900+].

*Amts Reg Koblenz*: Amtsblatt der Preußischen Regierung zu Koblenz [Coblenz: 1816–1900+].

*Amts Reg Liegnitz*: Amtsblatt der Königlichen Regierung zu Liegnitz [Liegnitz: 1811–1900+].

*Amts Reg Minden*: Amtsblatt der Königlichen Regierung zu Minden [Minden: 1816–1900+].

*Amts Reg Postdam*: Amtsblatt der Königlichen Regierung zu Potsdam [Potsdam: 1816–1900+]. As “Amtsblatt der Königlichen Regierung zu Potsdam und der Stadt Berlin” (1822–1900+).

*An Lit*: L’Année Littéraire [Paris: 1754–1790].

*Analec Mag*: The Analectic Magazine [Philadelphia: 1813–1820].

*Anales Cir Méd Argent*: Anales del Circulo Médico Argentino [Buenos Aires: 1877–1900+].

*Analyst*: The Analyst; a Quarterly Journal of Science, Literature, Natural History, and the Fine Arts [London: 1834–1840].

*Analyt Rev*: The Analytical Review [London: 1788–1799].

*Ann Civ Reg Sicile*: Annali Civili del Regno delle due Sicilie [Napoli: 1833–1860].

*Ann Geog*: Annali di Geographica, e di Statistica [Genoa: 1802].

*Ann Mag Nat Hist*: The Annals and Magazine of Natural History [London: 1838–1900+].

*Ann Öst Lit*: Annalen der Österreichischen Literatur [Regensburg/Vienna: 1802–1812]. Title varies over years. Published as “Annalen der Literatur und Kunst in dem Oesterreichischen Kaiserthume” (1811–1812).

*Ann Phil*: The Annals of Philosophy [London: 1813–1826].

*Ann Typogr*: Annales Typographiques, ou Notice du Progrès des Connoissances Humaines [Paris: 1759–1763].

*Anti-Jac Rev Mag*: The Anti-Jacobin Review and Magazine [London: 1798–1810].

*Arch Med Erf*: Archiv für Medizinische Erfahrung [Berlin: 1801–1836].

*Athenaeum*: The Athenaeum. Journal of Literature, Science, and the Fine Arts [London: 1828–1900+].

*Athenaeum–Prague*: Athenaeum. Listy pro Literaturu a Kritiku Vědeckou [Prague: 1883–1893].

*Athenaeum Fr*: L’Athenaeum Français [Paris: 1852–1856].

*Ausland*: Das Ausland [Munich/Tübingen/Augsburg/Stuttgart: 1828–1893].

*Author*: The Author. The Organ of the Society of Authors (Incorporated) [London: 1890–1900+].

*Bayer Ann*: Bayerische Annalen [Munich: 1833–1835].

*Bayer Landbote*: Der Bayerische Landbote [Munich: 1825–1878].

*Beil Aarau Ztg*: Beilage zur Aarauer Zeitung. Issued in conjunction with “Aarauer Zeitung” [Aarau: 1814–1821].

*Beil Allg Ztg*: Beilage zur Allgemeinen Zeitung; Ausserordentliche Beilage zur Allgemeinen Zeitung. Issued in conjunction with “Allgemeine Zeitung” [*q.v.*].

*Beil Illustr Ztg*: Beilage zur Illustrirten Zeitung. Issued in conjunction with “Illustrirte Zeitung” [Leipzig: 1843–1900+].

*Beil Ztg Frankfurt*: Beilage zu der Zeitung des Grossherzogthums Frankfurt. Issued in conjunction with “Zeitung des Grossherzogthums Frankfurt” [Frankfurt am Main: 1811–1814].

*Berg J*: Bergmännisches Journal [Freyberg and Annaberg: 1788–1794].

*Berl Ent Zeit*: Berliner Entomologische Zeitschrift [Berlin: 1857–1900+]. As “Deutsche Entomologische Zeitschrift” (1875–1880).

*Bibl Alsacien*: Le Bibliographe Alsacien [Strasbourg: 1863–1869].

*Bibl Belg*: Bibliographie de la Belgique [Brussels: 1838–1868, 1875–1900+].

*Bibl Blätt*: Bibliographische Blätter der Press-Zeitung [Leipzig: 1840].

*Bibl Cathol*: Bibliographie Catholique, Revue Critique des Ouvrages de Religion, de Philosophie, d’Histoire, de Littérature, d’Éducation, etc. [Paris: 1841–1889].

*Bibl Deutsch*: Bibliographie von Deutschland [Leipzig: 1826–1835]. Succeeded by “Allgemeine Bibliographie für Deutschland” [*q.v.*].

*Bibl Fr*: Bibliographie de la France [Paris: 1811–1900+]. As “Journal général de l’Imprimerie et de la Librairie” (1810–1811).

*Bibl Ital-1*: Bibliografia Italiana [Milan: 1835–1846].

*Bibl Ital-2*: Bibliografia Italiana [Florence/Milan: 1867–1903].

*Bibl Lit Anz*: Bibliographischer und Literarischer Anzeiger. Issued in conjunction with “Allgemeine Monatschrift für Literatur” [Halle: 1850–1854].

*Bibl Monat*: Bibliographischer Monatsbericht [Leipzig: 1889–1900+].

*Bibl Univers*: Bibliographie Universelle. Journal du Libraire et de l’Amateur de Livres [Paris: 1848].

*Bibl Zool*: Bibliographia Zoologica [Leipzig: 1896–1900+].

*Bibliologue*: Le Bibliologue [Paris: 1833].

*Bibliot Genève*: Bibliothèque Universelle de Genève [Geneva: 1836–1857].

*Bibliot Geo-Stat*: Bibliotheca Geographico-Statistica et Oeconomico-Politica [Göttingen: 1853–1874] . As “Bibliotheca Historico-Geographica” (1853–1861) and “Bibliotheca Geographica” (1870–1874).

*Bibliot Hist-Nat*: Bibliotheca Historico-Naturalis, Physico-Chemica et Mathematica [Göttingen: 1851–1888].

*Bibliot Ital*: Biblioteca Italiana o sia Giornale di Letteratura, Scienze ed Arti [Milan: 1816–1840].

*Bibliot Philo*: Bibliotheca Philologica [Göttingen: 1848–1897].

*Biol Centr*: Biologisches Centralblatt [Leipzig: 1881–1900+].

*Blackw Mag*: Blackwood’s Edinburgh Magazine [London: 1817–1900+].

*Blätt Lit Unterhalt*: Blätter für Literarische Unterhaltung [Leipzig: 1826–1896].

*Bookseller*: The Bookseller, a Newspaper of British and Foreign Literature [London: 1858–1900+].

*Börsen Deutsch Buch*: Börsenblatt für den Deutschen Buchhandel [Leipzig: 1834–1900+].

*Braunsch Mag*: Braunschweigisches Magazin [Brunswick: 1788–1868].

*Bremer Ztg*: Bremer Zeitung [Bremen: 1742–1848].

*British Critic*: The British Critic [London: 1793–1843].

*British Rev*: The British Review, and London Critical Journal [London: 1811–1825].

*Bull Bibl*: Bulletin Bibliographique des Ouvrages Publiés en France sur les Sciences Médicales, Naturelles et Physiques [Paris: 1835–1836].

*Bull Litt Étr*: Bulletin de la Littérature Étrangère [Paris: 1801–1841]. First published under the title “Journal général de la Littérature Étrangère” (1801–1830), it was annexed to the “Journal général de la Littérature de France” [*q.v.*].

*Bull Mens*: Bulletin Mensuel des Récentes Publications Françaises [Paris: 1882–1900+].

*Bull Sci Nat Géol*: Bulletin des Sciences Naturelles et de Géologie [Paris: 1824–1831].

*Bull Soc Sav*: Bulletin des Société Savantes, Missions Scientifiques et Littéraire [Paris: 1854–1855].

*Can Ent*: The Canadian Entomologist [Toronto/London/Ottawa: 1868–1900+].

*Can Mag*: The Canadian Magazine, and Literary Repository [Montreal: 1823–1825].

*Čas Mus Král České*: Časopis Musea Království Českého [Prague: 1854–1900+].

*Cat Hebd*: Catalogue Hebdomadaire ou Liste Alphabétique des Livres, tant Nationaux qu’Étrangers [Paris: 1763–1789].

*Cat Lib Fr*: Catalogue Mensuel de la Librairie Française [Paris: 1876–1900+].

*Cat Muquardt*: Catalogue de Nouveautés de la Littérature Allemande Publié par la Librairie Allemande et Étrangère de C. Muquardt [Bruxelles: 1849–1856].

*Centr Forst*: Centralblatt für das gesamte Forstwesen [Vienna: 1875–1900+].

*Chem Centr*: Chemisches Central-Blatt [Leipzig: 1830–1900+]. Title varies over time.

*Christ Exam*: The Christian Examiner and Religious Miscellany [Boston: 1824–1869].

*Christ Obs*: The Christian Observer [London: 1802–1874].

*Constit*: Le Constitutionnel [Paris: 1815–1900+].

*Cosmos*: Cosmos [Paris: 1852–1900+].

*Courrier Lib*: Courrier de la Librairie [Paris: 1851–1858].

*Crit Rev*: The Critical Review or, Annals of Literature [London: 1756–1817].

*Cultiv Count Gent*: The Cultivator & Country Gentleman [Albany: 1866–1897].

*Décade*: La Décade Philosophique, Littéraire et Politique [Paris: 1794–1804]. Succeeded by “La Revue Philosophique, Littéraire et Politique” [*q.v.*].

*Deutsch Ent Zeit*: Deutsche Entomologische Zeitschrift [Berlin: 1881–1900+].

*Deutsch Forst-Ztg*: Deutsche Forst-Zeitung [Neudamm: 1886–1900+].

*Deutsch Litt*: Deutsche Litteraturzeitung [Berlin: 1880–1900+].

*Deutsch Ztg*: Deutsche Zeitung [Heidelberg: 1847–1850].

*Donns-Blatt*: Donnstags-Blatt [Zürich: 1781–1800].

*Dresdner Anz*: Dresdner Anzeigen [Dresden: 1795–1847].

*Dublin Phil J*: The Dublin Philosophical Journal, and Scientific Review [Dublin: 1825–1826].

*Dublin Univ Mag*: The Dublin University Magazine [Dublin: 1833–1882].

*Echo Monde Sav*: L’Écho du Monde Savant [Paris: 1834–1845].

*Eclectic Rev*: The Eclectic Review [London: 1805–1868].

*Edinb Mag*: The Edinburgh Magazine [Edinburgh: 1817–1900+]. Succeeded “The Scots Magazine” [*q.v.*].

*Edinb Rev*: The Edinburgh Review or Critical Journal [Edinburgh: 1802–1900+].

*English Cat Books*: The English Catalogue of Books [London: 1864–1900+]. A retrospective volume published in 1914 by Peddie and Waddington covers the years 1801–1835. The first volume covers the years 1835–1864.

*Ent Amer*: Entomologica Americana [Brooklyn: 1885–1890, 1926+].

*Ent Litt*: Entomologische Litteraturblätter [Berlin: 1901–1914].

*Ent Mag*: The Entomological Magazine [London: 1832–1838].

*Ent Monats*: Entomologische Monatsblätter [Berlin: 1876–1880].

*Ent Monthly Mag*: The Entomologist’s Monthly Magazine [London: 1864–1900+].

*Ent Nachr*: Entomologische Nachrichten [Putbus/Berlin: 1875–1900].

*Ent News*: Entomological News [Philadelphia: 1890–1900+].

*Ent Record*: The Entomologist’s Record and Journal of Variation [London: 1890–1900+].

*Ent Weekly Intel*: The Entomologist’s Weekly Intelligencer [London: 1856–1861].

*Ent Zeit*: Entomologische Zeitschrift [Frankfurt a.M.: 1887–1900+].

*Ent Ztg*: Entomologische Zeitung [Stettin: 1840–1900+]. As “Stettiner Entomologische Zeitung” (1897–1900+).

*Entomologist*: The Entomologist [London: 1840–1900+].

*Erlang Anmer Nachr*: Erlangische gelehrte Anmerkungen und Nachrichten [Erlangen: 1746–1798]. As “Erlangische gelehrte Zeitung” (1790–1798).

*Esp Méd*: La España Médica [Madrid: 1856–1866].

*Esprit J*: L’Esprit des Journaux, François et Étrangers [Paris: 1772–1818]. As “Le Nouvel Esprit des Journaux, Français et Étrangers” (1803–1804).

*European Mag*: The European Magazine and London Review [London: 1782–1826].

*Feuil Comm*: Feuilleton Commercial du Journal général de l’Imprimerie et de la Librairie. Issued in conjunction with “Bibliographie de la France” [*q.v.*].

*Feuil J Lib*: Feuilleton du Journal de la Librairie. Issued in conjunction with “Bibliographie de la France” [*q.v.*].

*Feuille Jeunes Nat*: Feuille des Jeunes Naturalistes [Paris: 1870–1900+].

*Foreign Quart Rev*: The Foreign Quarterly Review [London: 1827–1846].

*Frän Merkur*: Fränkischer Merkur [Bamberg: 1810–1848].

*Frank Anz*: Frankfurter Gelehrte Anzeigen [Frankfurt am Main: 1772–1790].

*Gal Lit Gaz*: Galignani’s Literary Gazette, or Sunday Messenger [Paris: 1821–1823].

*Gal Weekly Repert*: Galignani’s Weekly Repertory, or Literary Gazette, and Journal of the Belles Lettres [Paris: 1818–1821].

*Garden*: The Garden: an Illustrated Weekly Journal of Horticulture in all its Branches [London: 1872–1900+].

*Gaz Fr*: Gazette de France [Paris: 1631–1900+].

*Gaz Litt*: Gazette Littéraire [Paris: 1830–1831].

*Gaz Litt Eur-Amsterdam*: Gazette Littéraire de l’Europe [Amsterdam: 1764–1785].

*Gaz Litt Eur-Paris*: Gazette Littéraire de l’Europe [Paris: 1764–1766]. Preceded by “Journal Étranger” [*q.v.*].

*Gaz Tirol*: Gazzetta del Tirolo Italiano [Trento: 1850–1856].

*Gemein Wochen*: Gemeinnützige Wochenschrift [Würzburg: 1851–1897].

*Gent Mag*: The Gentleman’s Magazine [London: 1731–1900+].

*Geog J*: The Geographical Journal [London: 1893–1900+].

*Germ Mus*: The German Museum, or Monthly Repository of the Literature of Germany, the North and the Continent in General [London: 1800–1801].

*Gior Bibl Univers*: Giornale Bibliografico Universale [Milan: 1807–1811].

*Gior Fis-Med*: Giornale Fisico-Medico [Pavia: 1792–1796].

*Gior Lib*: Giornale della Libreria, della Tipografia e delle Arti e Industrie affini [Milan: 1888–1900+].

*Globe*: Le Globe, Journal Philosophique et Littéraire [Paris: 1824–1832].

*Goth Ztg*: Gothaische Gelehrte Zeitungen [Gotha: 1774–1804].

*Gött Anz*: Göttingische Gelehrte Anzeigen [Göttingen: 1739–1900+]. As “Göttingische Anzeigen von Gelehrten Sachen” (1753–1802).

*Hall Ztg*: Hallische Gelehrte Zeitungen [Halle: 1766–1792].

*Handlungs-Ztg*: Handlungs-Zeitung oder wöchentliche Nachrichten von Handel, Manufakturwesen, Künsten und neuen Erfindungen [Gotha: 1784–1799]. As “Handlungs-Zeitung oder wöchentliche Nachrichten von Handel, Manufakturwesen und Oekonomie” (1784–1788).

*Humboldt*: Humboldt. Monatsschrift für die gesamten Naturwissenschaften [Stuttgart: 1882–1890].

*Illustration*: L’Illustration, Journal Universel [Paris: 1843–1900+].

*Insekten-Börse*: Insekten-Börse [Leipzig: 1884–1900+].

*Intell Allg Lit Ztg*: Intelligenzblatt der Allgemeinen Literatur-Zeitung. Published in conjunction with “Allgemeine Literatur-Zeitung” [*q.v.*].

*Intell Jena Lit Ztg*: Intelligenzblatt der Jenaischen Allgemeine Literatur-Zeitung. Published in conjunction with “Jenaischen Allgemeine Literatur-Zeitung” [*q.v.*].

*Intell Serapeum*: Intelligenz-Blatt zum Serapeum. Published in conjunction with “Serapeum” [Leipzig: 1840–1870].

*Intell Teutsch Merkur*: Intelligenzblatt zum Neuen Teutschen Merkur. Issued in conjunction with “Der Teutschen Merkur” [*q.v.*].

*Intell Ztg Welt*: Intelligenzblatt der Zeitung für die Elegante Welt. Published in conjunction with “Zeitung für die Elegante Welt” [*q.v.*].

*Isis*: Isis von Oken^7^ [Leipzig: 1818–1848].

*Isis Lieb*: Isis. Zeitschrift für alle Naturwissenschaftlichen Liebhabereien [Magdeburg: 1876–1889].

*Isis Natuur*: Isis. Tijdschrift voor Natuurwetenschap [Haarlem: 1872–1879]. As “Isis. Maandschrift voor Natuurwetenschap” (1875–1879).

*J Débats*: Journal des Débats [Paris: 1789–1900+]. Title varies over time.

*J Encycl*: Journal Encyclopédique ou Universel [Liège/Bouillon: 1756–1793].

*J Étrang*: Journal Étranger [Paris: 1754–1763]. Succeeded by “Gazette Littéraire de l’Europe” [*q.v.*].

*J Fr*: Journal général de France [Paris: 1785–1792].

*J Frank Inst*: Journal of the Franklin Institute [Philadelphia: 1826–1900+].

*J Hist Lit*: Journal Historique et Littéraire [Luxembourg/Liège: 1773–1794].

*J Instr Publ*: Journal général de l’Instruction Publique [Paris: 1831–1870, 1879–1882].

*J Lit Fr*: Journal général de la Littérature de France [Paris: 1798–1841]. Included the “Bulletin de la Littérature étrangère” [*q.v.*].

*J Méd*: Journal de Médecine, Chirurgie, Pharmacie, &c. [Paris: 1754–1794, 1801–1817].

*J Oeco*: Journal Oeconomique [Paris: 1751–1772].

*J Paris*: Journal de Paris [Paris: 1777–1840].

*J Pharm*: Journal de Pharmacie et de Chimie [Paris: 1815–1900+]. As “Journal de Pharmacie et des Sciences Accessoires” (1815–1841).

*J Phys*: Journal de Physique, de Chimie et d’Histoire Naturelle [Paris: 1793–1823].

*J Polit*: Journal Politique, ou Gazette des Gazettes [Bouillon: 1764–1793]. Title varies over time.

*J Polit Litt*: Journal de Politique et de Littérature [Brussels: 1774–1783].

*J Pract Heil*: Journal der Practischen Heilkunde [Jena/Berlin: 1795–1843].

*J Sav*: Journal des Savants [Paris: 1665–1792, 1816–1900+]. As “Le Journal des Sçavans” (1665–1792).

*J Soc Pharm*: Journal de la Société des Pharmaciens de Paris [Paris: 1797–1799].

*J Typogr Bibl*: Journal Typographique et Bibliographique [Paris: 1797–1810].

*Jena Lit Ztg*: Jenaischen Allgemeine Literatur-Zeitung [Jena: 1804–1841]. Succeeded by “Neue Jenaischen Allgemeine Literatur-Zeitung” [*q.v.*].

*Jena Ztg*: Jenaische Zeitungen von Gelehrten Sachen [Jena: 1765–1786].

*Jenaer Lit*: Jenaer Literaturzeitung [Leipzig: 1874–1879].

*Ladies Monthly Mus*: The Ladies’ Monthly Museum [London: 1798–1832].

*Lancet*: The Lancet [London: 1823–1900+].

*Land Centr Deutsch*: Landwirthschaftliches Centralblatt für Deutschland [Leipzig/Berlin: 1853–1876].

*Law Times*: The Law Times [London: 1843–1900+].

*Leip Intell-Blatt*: Leipziger Intelligenz-Blatt [Leipzig: 1763–1824].

*Leip Lit Ztg*: Leipziger Literatur-Zeitung [Leipzig: 1812–1834]. Preceded by “Neue Leipziger Literaturzeitung” [*q.v.*].

*Leip Reper*: Leipziger Repertorium der Deutschen und Ausländischen Literatur [Leipzig: 1843–1860]. Preceded by “Repertorium der Gesammten Deutschen Literatur” [*q.v.*].

*Leip Ztg*: Leipziger Zeitung [Leipzig: 1810–1900+].

*Leopoldina*: Leopoldina [Jena/Halle: 1859–1900+].

*Lit-Blatt*: Literatur-Blatt [Stuttgart/Tübingen: 1817–1849].

*Lit Centr Deutsch*: Literarisches Centralblatt für Deutschland [Leipzig: 1850–1900+]. Preceded by “Allgemeine Literatur-Zeitung” [*q.v.*].

*Lit Deutsch Natur Ztg*: Literatur-Blatt der Allgemeinen Deutschen Naturhistorischen Zeitung. Published in conjunction with “Allgemeine Deutschen Naturhistorischen Zeitung” [Dresden: 1846–1847, 1855–1857].

*Lit Gaz*: The Literary Gazette, and Journal of the Belles Lettres, Arts, Sciences [London: 1817–1862]. Sometimes as “The London Literary Gazette.”

*Lit J*: The Literary Journal, and Weekly Register of Science and the Arts [Providence: 1833–1834].

*Lit Panor*: The Literary Panorama [London: 1806–1819]. As “The Literary Panorama, and National Register” (1814–1819)

*Lit World-Lond*: The Literary World [London: 1868–1900+].

*Lit World-NY*: The Literary World [New York: 1847–1853].

*Lit Ztg*: Literarische Zeitung [Berlin: 1834–1849].

*Litt Ztg*: Litteratur Zeitung [Erlangen: 1799–1802].

*Lond Mag*: The London Magazine or, Gentleman’s Monthly Intelligencer [London: 1732–1785].

*Lond Med Phys J*: The London Medical and Physical Journal [London: 1799–1833].

*Lond Rev*: The London Review of Politics, Society, Literature, Art, and Science [London: 1860–1869].

*Luz Orient List*: Luzac’s Oriental List [London: 1890–1900+].

*Mag Encycl*: Magasin Encyclopédique, ou Journal des Sciences, des Lettres et des Arts [Paris: 1792–1816].

*Mag Lit Ausl*: Magazin für die Literatur des Auslandes [Berlin: 1832–1900+].

*Mag Nat Hist*: The Magazine of Natural History and Journal of Zoology, Botany, Mineralogy, Geology, and Meteorology [London: 1828–1840].

*Med-Chir Ztg*: Medicinisch-Chirurgische Zeitung [Salzburg/Innsbruck/Munich: 1790–1839].

*Med Corr-Blatt*: Medicinisches Correspondenz-Blatt Bayerischer Aerzte [Erlangen: 1840–1850].

*Med Times Gaz*: The Medical Times and Gazette [London: 1852–1885].

*Mém Encyc*: Mémorial Encyclopédique et Progressif des Connaissances Humaines [Paris: 1831–1846]. As “Mémorial; Revue Encyclopédique des Connaissances Humaines” (1840–1844).

*Merc Fr*: Mercure de France [Paris: 1724–1823].

*Minerva*: Minerva, et Maanedsskrivt [Copenhagen: 1785–1808].

*Misc Ent*: Miscellanea Entomologica [Narbonne: 1892–1900+].

*Monatl Anz*: Monathliche Historisch-Litterarisch-Artistische Anzeigen [Nürnberg: 1797–1802].

*Monatl Bericht Bücher*: Monatlicher Bericht über alle neu erschienenen Bücher. Published in conjunction with “Preußische Provinzial-Blätter” [Königsberg: 1829–1834].

*Monatl Corresp*: Monatliche Correspondenz zur Beförderung der Erd- und Himmels-Kunde [Gotha: 1800–1813].

*Monats-Bericht Landes*: Monats-Bericht des F.S. privil. Landes-Industrie-Comptoirs [Weimer: 1805–1810]. Succeeded by “Allgemeiner Monats-Bericht für Deutschland” [*q.v.*].

*Monit Lib*: Le Moniteur de la Librairie [Paris: 1841–1846].

*Monit Univers*: Le Moniteur Universel [Paris: 1789–1901]. As “Gazette Nationale ou le Moniteur Universel” (1789–1810).

*Monit Zool Ital*: Monitore Zoologico Italiano [Florence: 1890–1900+].

*Monthly Epit*: The Monthly Epitome [London: 1797–1804].

*Monthly List Muquardt*: A Monthly List of all New Books Published in Great Britain, sold by Mr. C. Muquardt [London: 1844–1855].

*Monthly Lit Adv*: The Monthly Literary Advertiser [London: 1805–1860]. Also as “Bent’s Monthly Literary Advertiser” or “Bent’s Literary Advertiser.”

*Monthly Mag*: The Monthly Magazine [London: 1796–1843].

*Monthly Rev*: The Monthly Review [London: 1749–1844].

*Nat Nov*: Naturae Novitates [Berlin: 1879–1900+].

*Nat Sci*: Natural Science: A Monthly Review of Scientific Progress [London: 1892–1899].

*Nation*: The Nation. A Weekly Journal Devoted to Politics, Literature, Science, & Arts [New York: 1865–1900+].

*Natur Can*: Le Naturaliste Canadien [Quebec City: 1868–1900+].

*Natur Offen*: Natur und Offenbarung [Münster: 1855–1900+].

*Naturaliste*: Le Naturaliste [Paris: 1879–1900+].

*Nature*: Nature. A Weekly Illustrated Journal of Science [London: 1869–1900+].

*Naturwiss Rund*: Naturwissenschaftliche Rundschau [Brunswick: 1886–1900+].

*Naturwiss Wochen*: Naturwissenschaftliche Wochenschrift [Berlin/Jena: 1887–1900+].

*Nederl Bibl*: Nederlandsche Bibliographie [’s Gravenhage: 1856–1900+].

*Nederl Spectator*: Nederlandsche Spectator [‘s Gravenhage: 1856–1900+].

*Neue Hall Ztg*: Neue Hallische Gelehrte Zeitungen [Halle: 1766–1792].

*Neue Helvetia*: Neue Helvetia. Eine Schweizerische Monatsschrift [Zürich: 1843–1844].

*Neue Intell Lit Kunst*: Neues allgemeines Intelligenzblatt für Literatur und Kunst. Published in conjunction with “Neue Leipziger Literaturzeitung” [*q.v.*].

*Neue Jena Lit Ztg*: Neue Jenaischen Allgemeine Literatur-Zeitung [Leipzig: 1842–1848]. Preceded by “Jenaischen Allgemeine Literatur-Zeitung” [*q.v.*].

*Neue Leip Lit*: Neue Leipziger Literaturzeitung [Leipzig: 1803–1811]. Succeeded by “Leipziger Literatur-Zeitung” [*q.v.*].

*Neue Leip Ztg*: Neue Leipziger Gelehrte Zeitungen [Leipzig: 1715–1797]. Title varies over time.

*Neue Münch Ztg*: Neue Münchener Zeitung [Munich: 1848–1862].

*Neue Spey Ztg*: Neue Speyerer Zeitung [Speyer: 1816–1853].

*Neue Würz Ztg*: Neue Würzburger Zeitung [Würzburg: 1804–1900+].

*Neue Ztg*: Neue Zeitungen von Gelehrten Sachen [Leipzig: 1715–1784].

*Neuer Anz Bibl*: Neuer Anzeiger für Bibliographie und Bibliothekwissenschaft [Dresden/Berlin and Stuttgart: 1840–1886].

*New Eng Farmer*: The New England Farmer [Boston: 1822–1846].

*New Monthly Mag*: The New Monthly Magazine [London: 1814–1884].

*New Rev*: A New Review; with Literary Curiosities and Literary Intelligence [London: 1782–1786].

*New Zeal Parl Debates*: New Zealand. Parliamentary Debates [Wellington: 1854–1900+].

*North Amer Rev*: The North American Review [Boston/New York: 1815–1900+].

*Norton Lit Gaz*: Norton’s Literary Gazette and Publishers’ Circular [New York: 1851–1855]. Superseded by the “American Publishers’ Circular and Literary Gazette” [*q.v.*].

*Not Geb Natur*: Notizen aus dem Gebiete der Natur- und Heilkunde [Erfurt/Weimar: 1821–1849]. As “Neue Notizen aus dem Gebiete der Natur- und Heilkunde” (1837–1846).

*Not Geb Pharm*: Notizen aus dem Gebiete der Practischen Pharmacie [Krefeld: 1837–1863].

*Nouv Litt*: Le Nouvelliste Littéraire, des Sciences et des Arts [Paris: 1796–1801].

*Nouv Rev Encycl*: Nouvelle Revue Encyclopédique [Paris: 1846–1847].

*Nouv Rev Germ*: Nouvelle Revue Germanique [Paris: 1829–1837]. As “ Revue Germanique” (1835–1837).

*Nouveauté*: La Nouveauté, Journal du Commerce, des Sciences, de la Littérature, des Théatres et des Arts [Paris: 1825–1827].

*Nürnb Ztg*: Nürnbergische Gelehrte Zeitung [Nürnberg: 1777–1789].

*Ober Lit*: Oberdeutsche Allgemeine Litteraturzeitung [Salzburg/Munich: 1788–1808].

*Observ Phys*: Observations sur la Physique, sur l’Histoire Naturelle et sur les Arts [Paris: 1752–1757, 1771–1793].

*Observ Spec*: L’Observateur des Spectacles, de la Littérature et des Arts [Paris: 1802].

*Oest Med Wochen*: Oesterreichische Medicinische Wochenschrift [Vienna: 1841–1848].

*Oest Monat Orient*: Oesterreichische Monatsschrift für den Orient [Vienna: 1875–1900+].

*Ohio Cult*: Ohio Cultivator [Columbus: 1845–1866].

*Oppos-Blatt*: Oppositions-Blatt oder Weimarische Zeitung [Weimar: 1817–1820].

*Orient Herald*: The Oriental Herald, and Journal of General Literature [London: 1824–1829].

*Pal Expl Fund*: Palestine Exploration Fund, Quaterly Statement [London: 1870–1900+].

*Pet Nouv Ent*: Petites Nouvelles Entomologiques [Paris: 1869–1879].

*Pharm Centr*: Pharmaceutisches Central-Blatt [Leipzig: 1830–1849].

*Phil Mag*: The Philosophical Magazine [London: 1798–1900+]. As “The Philosophical Magazine and Journal” (1814–1826) and “The London, Edinburgh, and Dublin Philosophical Magazine and Journal of Science” (1840–1900+).

*Poligrafo*: Poligrafo. Giornale di Scienze, Lettere ed Arti [Verona: 1830–1845].

*Polit J*: Politisches Journal nebst Anzeige von Gelehrten und Andern Sachen [Hamburg: 1781–1840].

*Polybiblion*: Polybiblion. Revue Bibliographique Universelle [Paris: 1868–1900+].

*Pop Sci Monthly*: The Popular Science Monthly [New York: 1872–1900+].

*Presse*: La Presse [Paris: 1836–1900+].

*Psyche*: Psyche [Cambridge (Massachusetts): 1874–1900+].

*Publ Circ*: The Publishers’ Circular [London: 1837–1900+].

*Publ Weekly*: The Publishers’ Weekly [New York: 1872–1900+].

*Punch*: Punch, or the London Charivari [London: 1841–1900+].

*Quart J Sci*: The Quarterly Journal of Science [London: 1864–1878].

*Quart Rev*: The Quarterly Review [London: 1809–1900+].

*Regens Morgen*: Regensburger Morgenblatt [Regensburg: 1861–1900+].

*Regens Wochen*: Regensburger Wochenblatt [Regensburg: 1815–1900+].

*Reichs-Anz*: Der Reichs-Anzeiger [Gotha: 1793–1806]. The title of each number reads “Kaiserlich Privilegirter Reichs-Anzeiger.”

*Réper Bibl Lib Fr*: Répertoire Bibliographique de la Librairie Française [Paris: 1900–1904].

*Reper Deutsch Lit*: Repertorium der Gesammten Deutschen Literatur [Leipzig: 1834–1842]. Succeeded by “Leipziger Repertorium der Deutschen und Ausländischen Literatur” [*q.v.*].

*Rev Bibl-Brus*: Revue Bibliographique du Royaume des Pays-Bas et de l’Étranger [Brussels: 1822–1830].

*Rev Bibl-Par*: Revue Bibliographique. Journal de Bibliologie, d’Histoire Littéraire, d’Imprimerie et de Librairie [Paris: 1839].

*Rev Clin Thérap*: Revue générale de Clinique et de Thérapeutique [Paris: 1887–1900+].

*Rev Encycl*: Revue Encyclopédique [Paris: 1819–1835].

*Rev Ent*: Revue d’Entomologie [Caen: 1882–1900+].

*Rev Gen Sci*: Revue générale des Sciences Pures et Appliquées [Paris: 1890–1900+].

*Rev Germ*: Revue Germanique [Strasbourg/Paris: 1858–1865]. As “Revue Germanique et Française” (1862–1865).

*Rev Instr Publ*: Revue de l’Instruction Public de la Littérature et des Sciences en France et dans les Pays Étrangers [Paris: 1842–1870].

*Rev Mag Zool*: Revue et Magasin de Zoologie Pure et Appliquée [Paris: 1849–1879]. Preceded by “ Revue Zoologique ” [*q.v.*].

*Rev Mar Colon*: Revue Maritime et Coloniale [Paris: 1861–1896].

*Rev Phil*: La Revue Philosophique, Littéraire et Politique [Paris: 1804–1807]. Preceded by “La Décade Philosophique, Littéraire et Politique” [*q.v.*].

*Rev Sci*: Revue Scientifique (Revue Rose) [Paris: 1863–1900+]. As “La Revue Scientifique de la France et de l’Étranger” (1871–1883).

*Rev Sci Lim*: La Revue Scientifique du Limousin [Limoges: 1893–1900+].

*Rev Tunis*: Revue Tunisienne [Tunis: 1894–1900+].

*Rev Zool*: Revue Zoologique [Paris: 1838–1848]. Succeeded by “Revue et Magasin de Zoologie Pure et Appliquée” [*q.v.*].

*Säch Berg-Ztg*: Sächsische Bergwerks-Zeitung [Freiberg: 1851–1854].

*Salzb Intell*: Salzburger Intelligenzblatt [Salzburg: 1784–1799]. As “Salzburger Intelligenzblatt, oder Wöchentliche Nachrichten zum Allgemeine Nutzen, und zur Erbauung” (1786–1787).

*Sat Rev*: The Saturday Review of Politics, Literature, Science, and Art [London: 1855–1900+].

*Schwäb Merk*: Schwäbischer Merkur [Stuttgart: 1785–1900+].

*Schweizer-Bote*: Der Aufrichtige und Wohlerfahrene Schweizer-Bote [Aarau: 1798–1800, 1804–1836].

*Science*: Science: an Illustrated Journal [Cambridge (Mass.)/New York: 1883–1897].

*Science-Gossip*: Hardwicke’s Science-Gossip [London: 1865–1893].

*Scots Mag*: The Scots Magazine [Edinburgh: 1739–1817]. Preceded by “The Edinburgh Magazine” [*q.v.*].

*Semeur*: Le Semeur [Paris: 1831–1850].

*Siglo Médico*: El Siglo Médico [Madrid: 1854–1900+].

*Soc Ent*: Societas Entomologica [Zürich: 1886–1900+].

*Spectator*: The Spectator [London: 1828–1900+].

*Substitute*: The Substitute; or Entomological Exchange Facilitator, and Entomologist’s Fire-side Companion [London: 1856–1857].

*Suite Clef*: Suite de la Clef, ou Journal Historique sur les Matières du Tems [Paris: 1717–1776].

*Svensk Bokh*: Svensk Bokhandels-Tidning [Stockholm: 1863–1900+].

*Swensk Bibl*: Swensk Bibliographi [Stockholm: 1828–1865].

*Télé Litt*: Le Télégraphe Littéraire, ou le Correspondant de la Librairie [Paris: 1802–1810].

*Term Közl*: Természettudományi Közlöny [Budapest: 1869–1900+].

*Teutsch Merkur*: Der Teutsche Merkur [Weimar: 1773–1810]. As “Der Neuen Teutschen Merkur” (1790–1810).

*Torch Col Book Circ*: The Torch and Colonial Book Circular [London: 1887–1892].

*Town Count Mag*: The Town and Country Magazine [London: 1769–1796].

*Trüb Amer Orient Lit Rec*: Trübner’s American and Oriental Literary Record [London: 1865–1891].

*Univers Cat*: The Universal Catalogue [London: 1772–1774].

*Univers Mag*: The Universal Magazine of Knowledge and Pleasure [London: 1747–1814]. As “The Universal Magazine” (1804–1814).

*US Lit Gaz*: The United States Literary Gazette [Boston: 1824–1826].

*Vas Ujs*: Vasảrnapi Ujsảg [Budapest: 1854–1900+].

*Verz Bücher Landkarten*: Verzeichniss der Bücher, Landkarten &c [Leipzig: 1776–1900+]. Title varies over time. As “*Verzeichniss neuer Bücher*” (1785–1822).

*Verz Derj Bücher*: Verzeichnis Derjenigen Bücher [Munich: 1831].

*Wien Ent Monats*: Wiener Entomologische Monatschrift [Vienna: 1857–1864].

*Wien Ent Ztg*: Wiener Entomologische Zeitung [Vienna: 1882–1900+].

*Wien Kirch*: Wiener Kirchenzeitung [Vienna: 1848–1874].

*Wien Obst Gart*: Wiener Obst- und Garten-Zeitung [Vienna: 1876–1900+].

*Wöchentl Lit Anz*: Wöchentliches Literarisches Anzeige-Blatt. Issued in conjunction with “Allgemeine Bibliographie für Deutschland” [*q.v.*].

*Wöchentl Nachr*: Wöchentliche Nachrichten von neuen Landcharten, Geographischen, Statistischen und Historischen Büchern und Sachen [Berlin: 1773–1787].

*Zeit Forst Jagd*: Zeitschrift für Forst- und Jagdwesen [Berlin: 1869–1900+].

*Zeit Math Naturwiss Unter*: Zeitschrift für Mathematischen und Naturwissenschaftlichen Unterricht [Leipzig: 1870–1900+].

*Zeit Naturwiss*: Zeitschrift für die Gesammten Naturwissenschaften [Halle/Berlin: 1853–1881].

*Zool Anz*: Zoologischer Anzeiger [Leipzig: 1878–1900+].

*Zool Centr*: Zoologisches Centralblatt [Leipzig: 1894–1900+].

*Zool Gart*: Der Zoologische Garten [Frankfurt a.M.: 1859–1900+].

*Zoologist*: The Zoologist: A Monthly Journal of Natural History [London: 1843–1900+].

*Ztg Welt*: Zeitung für die Elegante Welt [Leipzig: 1801–1859].

*Zug Gött Anz*: Zugabe zu den Göttingischen Anzeigen von Gelehrten Sachen. Issued in conjunction with “Göttingische Gelehrte Anzeigen” [*q.v.*].

The search for publication dates through these journals was done mainly using Google Books. This process has its limitations. For example, some pages are poorly scanned and unreadable, others are simply missing, and sometimes whole issues of the journal are missing. Nevertheless, this digital database has many rare or difficult-to-find journals useful for dating books.

## List of abbreviations used

ca
 circa 


cf.

*confer* [to be compared to] 


Code

*International Code of Zoological Nomenclature*
 (ICZN 1999)

DP date of publication


e.g. exempli gratia [for example]


et al.

*et alii* [and others] 


fasc. fasciculus


ICZN International Commission on Zoological Nomenclature


i.e.
*id est* [that is]


JD Julian date


livr. livraison


mf microfiches


n.d. no date


no number


nos numbers


n.v.

*non vide* [not seen] 


P
 portrait 


p. page


ph photocopy


pl. plate


pls plates


pp. pages


q.v.

*quod vide* [see] 


vol. volume


vols volumes


## Acknowledgments

I thank Neal L. Evenhuis, Miguel A. Alonso-Zarazaga, Patrice Bouchard, Anthony Davies and Martyn E.Y. Low for providing information relevant to this project and for reviewing the manuscript. Aleš Smetana is acknowledged for translating German texts and access to his personal library. I am indebted to Jiří Háva for providing a PDF of Precht’s work. The cooperation of Patricia Madaire and Tania Durr of the Canadian Agricultural Library (Entomology) in Ottawa, Dana Fischer of the Ernst Mayr Library at the Museum of Comparative Zoology in Cambridge, Massachusetts, Eileen Mathias and Ria Capone of The Ewell Sale Stewart Library at the Academy of Natural Sciences of Drexel University in Philadelphia, and Marty Schlabach at the Comstock Memorial Library of Entomology at Cornell University in Ithaca, New York, for locating works is greatly appreciated.

## Taxonomic literature

### Books (other than dictionaries)

#### [Anonymous]

**1790**. *Handbuch der deutschen Thiergeschichte für Schulen.* Giesen [= Giessen]. 247 pp. (8vo) [DP: 1790 (title page), 3 October 1790^8^] GB, MDZ

This book was published anonymously. Bertau (2014: 491) credited it to Johann Samuel Halle [1727–1810], a Prussian historian and toxicologist, professor of history at the Royal Prussian Corps des Cadets in Berlin.

The Coleoptera are on pages 166–190.

#### Abeille de Perrin, Elzéar Emmanuel Arène (Marseille, Bouches-du-Rhône, France: 3 January 1843 – 9 October 1910: Marseille, France). French entomologist; studied law but soon after turned to his real passion, entomology; collected mostly in Europe, particularly in caves, but also in Syria (1874, 1879), Algeria (1893), and Tunisia (1894); described about a thousand species, mostly Coleoptera but also Hymenoptera; his collection was donated to the Muséum National d’Histoire Naturelle in Paris by his widow. References. Caillol (1912); Gouillard (2004: 50–51); Cambefort (2006: 103, P).

**1872**. *Études sur les coléoptères cavernicoles suivies de la description de 27 coléoptères nouveaux français.* Marius Olive, Marseille. 40 + [1] pp. (4to) [DP: April 1872 (title page), 8 May 1872 (*Soc Ent Fr*), 1 June 1872 (*Pet Nouv Ent*), 5 April 1873 (*Bibl Fr*)] Online

The half-title page reads “*Insectes cavernicoles de l’Ariège*.” The new species are described by Abeille de Perrin, Félicien de Saulcy, and Louis Pandellé; their latin diagnoses were reproduced in 1872 in the *Annales de la Société Entomologique de France* (cinquième série) 2: xviii–xxii, xli–xliv.

**1874**. *Essai monographique sur les cisides européens & circaméditerranéens.* E. Camoin, Marseille. [1] + 99 + [1] pp. (8vo) [DP: 1874 (title page; *introduction* dated 15 August 1874), 1 February 1875 (*Pet Nouv Ent*), 1 June 1875 (*Feuille Jeunes Nat*)] CNC, GB

#### Acerbi, Joseph [Giuseppe] (Mantua, Lombardy, Italy: 3 May 1773 – 25 August 1846: Mantua, Italy). Italian naturalist, explorer and composer; Austrian consul in Egypt (1825–1834). References. Wurzbach (1856: 3); Anonymous (1876a, P); Gabrieli (1971, P); Navarrini (2002: vii–xiii).

**1802**. *Travels through Sweden, Finland, and Lapland, to the North Cape, in the years 1798 and 1799. In two volumes. Illustrated with seventeen elegant engravings. Vol. II.* Joseph Mawman, London. viii + 380 pp. + 9 pls. (4to) [DP: 1802 (title page; dedication in first volume dated 1 February 1802), May 1802 (Peddie and Waddington 1914: 3), June 1802 (*Ann Geog*), July 1802 (*Anti-Jac Rev Mag*), 2 August 1802 (*Gött Anz*), October 1802 (*Crit Rev*; *Edinb Rev*), November 1802 (*Monthly Rev*), 2 December 1802 (*Allg Lit Ztg*)] GB

Section XV of the second volume (pp. 245–256 + 3 pls), entitled “Of the insects and testaceous animals of Lapland,” includes a list of insects collected; some species of Coleoptera are briefly described and illustrated but none of them are new to science.

A German translation was published in Berlin, 1803, under the title “*Reise durch Schweden und Finnland, bis an die äussersten Gränzen von Lappland, in den Jahren 1798 und 1799. Von Joseph Acerbi. Aus dem Englischen übersetzt von Ch. Weyland*” [GB]. A French translation was issued in three volumes (8vo) in Paris, 1803–1804, under the title “*Voyage au Cap-Nord, par la Suede, la Finlande et la Laponie, par Joseph Acerbi. Traduction d’après l’original anglais, revue sous les yeux de l’auteur, par Joseph Lavallée*” [GB].

#### Ackermann, Carl [Karl] Christian (Fulda, Hesse, Germany: 2 March 1841 – 23 April 1903: Cassel, Hesse, Germany). German educator and botanist; professor in secondary schools in Hersfeld (1865–1875) and later in Cassel (1875–1895) where he acceded to the post of director in 1888. Reference. Fennel (1903, P).

**1871.** *Die Kaefer. Zum Gebrauche beim Unterrichte und zum Selbstbestimmen.* Böttrich & Hoehl, Hersfeld [&] Rotenburg. vi + 110 pp. (8vo) [DP: 1871 (title page; *Vorwort* dated January 1870), January–June 1871 (*Bibliot Hist-Nat*)] GB

The section on Pentamera and Heteromera (pp. 1–66) was first published in 1870 in *Programm der höheren Bürgerschule zu Hersfeld* (*n.v.*) and recorded on January–June 1870 (*Bibliot Hist-Nat*).

#### Acloque, Alexandre Noël Charles (Auxi-le-Château, Pas-de-Calais, France: 12 January 1871 – 1908/1941^9^). French adman and naturalist, expert on lichens; popularizer of natural history.

**1895**. *Faune de France contenant la description de toutes les espèces indigènes disposées en tableaux analytiques et illustrée de figures représentant les types caractéristiques des genres et des sous-genres. Avec une préface par Edmond Perrier. Coléoptères. Avec 1052 figures.* J.-B. Baillière et Fils, Paris. 466 pp. (12mo) [DP: 1896 (title page; *avant-propos* dated 1 November 1895), 30 December 1895 (*Acad Sci Fr*), 4 January 1896 (*Cosmos*), 8 January 1896 (*Soc Ent Fr*), 11 January 1896 (*Rev Sci*), 15 January 1896 (*Misc Ent*), January 1896 (*Nat Nov*), 15 February 1896 (*Rev Sci Lim*), 11 April 1896 (*Bibl Fr*)] CNC

The entire series consists of four volumes, 1893–1900, treating the vertebrates and the Insecta. Despite the title, it is evident from the *avant-propos* that Acloque did not mean to designate type species for the genus-group taxa treated in the sense of the *Code*.

The following genus-group names are made available in this work: *Eudromius* (p. 49), *Eubrachinus* (p. 50), *Euditomus* (p. 53), *Pseudelaphrus* (p. 81), *Euphilhydrus* (p. 94), *Euberosus* (p. 94), and *Pseudhydraena* (p. 96). The new family-group names Ilybii (p. 83) and Pholidini (p. 145) are also made available for the first time.

#### Adams, Arthur (Gosport, Hampshire, United Kingdom: 1820 – 16 October 1878: London, United Kingdom). British navy physician and naturalist particularly interested in molluscs. Reference. Crosse and Fischer (1879: 91–93).

**1848**. Notes from a journal of research into the natural history of the countries visited during the voyage of H.M.S. *Samarang*, under the command of Captain Sir E. Belcher, C.B. Pp. 225–532 *in*: *Narrative of the voyage of H.M.S.* Samarang, *during the years 1843–46; employed surveying the islands of the eastern archipelago; accompanied by a brief vocabulary of the principal languages. Published under the authority of the Lords Commissioners of the Admiralty. By Captain Sir Edward Belcher, R.N., C.B., F.R.A.S., F.G.S., &c. commander of the expedition. With notes on the natural history of the islands, by Arthur Adams, assistant-surgeon, R.N. In two volumes. Vol. II.* Reeve, Benham, and Reeve, London. 574 + [2] pp. (4to) [DP: 1848 (title page), 22 January 1848 (*Athenaeum*), January 1848 (Low 1853: 25), 1 February 1848 (*Publ Circ*; *Monthly List Muquardt*), 10 February 1848 (*Monthly Lit Adv*)] GB, BHL

The entire work was published in two volumes, issued simultaneously. Two new species of beetles are described in the second volume, *Bolboceras koreensis* (p. 462) and *Platyrhynchus waltonianus* (p. 468).

As noted by Petit (2007: 91–92) there was a lengthly review of this book in *The Athenaeum* for 25 December 1847 by W.H. Dixon. Several quotations from the work are included but the title given differs slightly from that published and the publisher is listed as “Reeve & Co.” a firm that ceased to exist in November 1847. Clearly Dixon was furnished with a review copy.

**1854**. [Adams, A., Baikie, W.B. and Barron, C.] *A manual of natural history, for the use of travellers; being a description of the families of the animal and vegetable kingdoms: with remarks on the practical study of geology and meteorology. To which are appended directions for collecting and preserving.* John van Voorst, London. viii + 749 pp. (8vo) [DP: 1854 (title page; preface dated January 1854), 22 April 1854 (*Athenaeum*; *Lit Gaz*), 1 May 1854 (*Publ Circ*), 1 June 1854 (*Monthly List Muquardt*)] GB

The Coleoptera are on pages 174–206.

#### Agassiz, Jean Louis Rodolphe (Môtier [currently part of Haut-Vully], Switzerland: 28 May 1807 – 14 December 1873: Cambridge, Massachusetts, USA). Swiss biologist, geologist, paleontologist, and nomenclatorist; received his medical degree from the University of Erlangen in 1830 and the following year moved to Paris to study under Cuvier; returning to Switzerland, he taught for 13 years at the Lyceum of Neuchatel where he worked on many projects, including glaciology, and developed his theory of a great ice age that once gripped the earth; in 1846, travelled to the United States and settled there; soon after was appointed professor of zoology and geology at Harvard University; was instrumental in the establishment of the Museum of Comparative Zoology and served as the museum’s first director until his death. References. Ratzeburg (1874: 1–4); Agassiz (1885, P); Greenwood (1886: 94–117); Bolton (1889: 302–346, P); Holder (1893, P); Forsee (1958); Adler (1989: 39–40, P).

**1846**. *Nomenclator zoologicus, continens nomina systematica generum animalium tam viventium quam fossilium, secundum ordinem alphabeticum disposita, adjectis auctoribus, libris in quibus reperiuntur, anno editionis, etymologia et familiis, ad quas pertinent, in variis classibus*. *Fasciculus XI. Continens Coleoptera.* Jent et Gassmann, Soloduri [= Solothurn]. xi + [1] + 170 pp. (4to) [DP: 1846 (title page), 9 October 1846 (*Ent Ver Stettin*)] CNC, GB

An additional title page reads “*Nomina systematica generum Coleopterorum, tam viventium quam fossilium, secundum ordinem alphabeticum disposita, adjectis auctoribus, libris in quibus reperiuntur, anno editionis, etymologia et familiis ad quas pertinent*. *Recognovit permultisque nominibus locupletavit Guil. F. Erichson.*”

The entire series consists of 12 fascicles, 1842–1846. The first 11 pertain to classes or orders, the 11th being on Coleoptera; the 12th is the *Index Universalis* (see next entry).

**1846**. *Nomenclatoris zoologici. Index universalis, continens nomina systematica classium, ordinum, familiarum et generum animalium omnium, tam viventium quam fossilium, secundum ordinem alphabeticum unicum disposita, adjectis homonymiis plantarum, nec non variis adnotationibus et emendationibus.* Jent et Gassmann, Soloduri [= Solothurn]. viii + 393 pp. (4to) [DP: 1846 (title page; *praefatio* dated December 1845), 29 December 1846 (Evenhuis 1997a: 50), 10 July 1847 (*Ent Ver Stettin*)] MCZ, McG, GB

This volume is the 12th and last fascicle of Agassiz’s *Nomenclator zoologicus*. Another edition, 12mo, was published in 1848 and contains 1135 pages. According to Evenhuis (1997a: 52), there are no apparent differences between the two editions other than page size and pagination. The work includes numerous unjustified emendations of generic names.

#### Ahrens, Johann Friedrich August (Ballenstedt, Saxony-Anhalt, Germany: 18 August 1780 – 28 November 1841: Hettstedt, Saxony-Anhalt, Germany). German stage-actor (of the national theater of Magdeburg), private scholar and entomologist. References. Germar (1842); Eckstein (1844: 283); Carus (1875a).

**1812**. *Fauna Insectorum Europae. Fasciculus primus.* Car. Aug. Kümmel, Halae [= Halle]. [2] + [25] pp. + 25 pls. (12mo) [DP: n.d. (title page; preliminaries dated 1 March 1812), 27 March 1812 (*Leip Lit Ztg*), 31 March 1812 (*Allg Lit Ztg*), 8 April 1812 (*Intell Jena Lit Ztg*)] CNC (mf), GB, ANSP

This work was intended to supplement Panzer’s *Faunae Insectorum Germanicae initia*. Ahrens published the first fascicle, Germar and Kaulfuss the third one, 1816 [*q.v.*], and Germar the second (1814) and last 21 (1822–1847) [*q.v.*]. Rautenberg (1957: 615–625) gave a list of all species illustrated on the plates for the 24 fascicles.

#### Allard, Ernest (1820 – 1900). French coleopterist; head clerk at Bordeaux railway; part of his collection was bought by René Oberthür [*q.v.*]. Reference. Cambefort (2006: 104).

**1869**. *Révision des curculionides byrsopsides*. C.C. Meinhold & fils, Dresde [= Dresden]. 28 pp. + 1 pl. (8vo) [DP: 1869 (title page), 1 December 1869 (*Pet Nouv Ent*)] USNM

This booklet was issued subsequently in Heyden’s *Entomologische Reise nach dem südlichen Spanien* published in 1870 [*q.v.*]. “Révision du genre *Sphenophorus*” begins on page 25.

**1886**. *Description de six nouvelles espèces de coléoptères hétéromères.* [Paris]. [6] pp. (8vo) [DP: January 1886 (title page), 10 November 1886 (*Soc Ent Fr*), 20 December 1886 (*Wien Ent Ztg* 5: 352)] YB (ph)

The publisher and place of publication are not listed on this booklet and the pages not numbered. The species described, all Tenebrionidae, are: *Trigonoscelis elegans* (Turkestan) [p. 1], *Trigonoscelis cordata* (Lenkoran) [p. 2], *Thriptera bedeli* (Algeria) [p. 3], *Ocnera leprieuri* (Egypt) [p. 4], *Asida lostiae* (Sardinia) [p. 5], and *Blapimorpha davidea* (Pekin) [p. 6].

#### Alluaud, Charles (Limoges, Haute-Vienne, France: 4 May 1861 – 12 December 1949: Crozant, Creuse, France). French explorer and coleopterist of independent means until WWII left him penniless; made 24 expeditions to Africa and several islands in the Atlantic and Indian oceans, including the Canaries, Seychelles, Mascareignes, and Madagascar; one of the first to collect on Kilimanjaro; founded the journal *Afra* devoted to the study of Afrotropical Geadephaga and authored most of the papers that would be published in the 11 fascicles of the journal, 1930–1936; married, he had one son, who accidentally killed himself at the age of 14 while playing with a gun; published about 165 papers, most of them short notes of a few pages on the family Carabidae; most of his material is now in the Muséum National d’Histoire Naturelle in Paris. References. Jeannel (1952, P); Chevallier (1996); Gouillard (2004: 99–100); Cambefort (2006: 104–106).

**1902**. *Histoire physique, naturelle et politique de Madagascar publié par Alfred Grandidier. Volume XXI. Histoire naturelle des Coléoptères. Tome I – Texte – 1^re^ partie. Liste des insectes coléoptères de la région Malgache.* Imprimerie Nationale, Paris. viii + 509 pp. (4to) [DP: 1 April 1900 (title page), 6 September 1902 (*Bibl Fr*), November 1902 (*Polybiblion*)] CMLE, BHL

The *Histoire naturelle des Coléoptères* was published in three parts, forming two volumes, 1887–1902. The second volume was authored by Kunckel d’Herculais, 1887–1891 [*q.v.*]. According to Friedländer (1902: 209), the first volume was published only in 1902 despite the date on the title page; this seems to be confirmed by the entries in the two recording journals cited above.

The entire *Histoire physique, naturelle et politique de Madagascar* was issued in 57 fascicles, forming 39 volumes, 1875–1954, first under the direction of Alfred Grandidier, 1875–1913, then Alfred and Guillaume Grandidier, 1914–1932, and finally Guillaume Grandidier, 1942–1954. Alluaud’s contribution is the 52nd fascicle of the series.

This work contains a list of beetles, with localities, known from Madagascar and nearby islands. One new replacement name, *Eutelonotus* (p. 456) to be attributed to Fairmaire, is proposed for *Eutelus* Solier, a junior homonym of *Eutelus* Walker.

#### Altmann, L.

**1837**. *Kurzer Abriss der Entomologie mit besonderer Rücksicht auf Deutschlands Käfer nach den neueren Benennungen geordnet.* Ch. G. Kayser, Leipzig. iv + 124 pp. (8vo) [DP: 1837 (title page; *Vorwort* dated 14 May 1837), 22 September 1837 (*Allg Bibl Deutsch*), 27 September 1837 (*Lit Ztg*), 30 October 1837 (*Reper Deutsch Lit*), July–December 1837 (*Verz Bücher Landkarten*)] YB

**1844**. *Die nützlichen und schädlichen Forstkäfer für Forstbeamte. Nebst einem Anhange über das Vorkommen der Käfer auf den Baum- und Strauch-Arten.* Hermann Neubürger, Dessau. 60 pp. (8vo) [DP: 1844 (title page; *Vorrede* dated September 1844), 28 November 1844 (*Allg Bibl Deutsch*), 11 December 1844 (*Lit Ztg*), July–December 1844 (*Verz Bücher Landkarten*)] GB

#### Altum, Johann Bernard Theodor (Münster, North Rhine-Westphalia, Germany: 31 January/December 1824^10^ – 1 February 1900: Eberswalde, Brandenburg, Germany). German catholic priest and zoologist; lecturer at the University of Münster; later, professor of zoology at the Royal Academy of Forestry in Eberswalde. References. Anonymous (1900b: 45–46); Schalow (1919: 555–569, P); Eidmann (1953).

**1881**. *Forstzoologie. III. Insecten. 1. Abth. Allgemeines und Käfer. Zweite verbesserte und vermehrte Auflage. Mit 55 meist Original-Figuren in Holzschnitt.* Julius Springer, Berlin. vii + 380 pp. (4to) [DP: 1881 (title page; *Vorrede* dated 12 July 1881), 8 October 1881 (*Lit Centr Deutsch*), 17 October 1881 (*Zool Anz*), October 1881 (*Nat Nov*), November 1881 (*Allg Forst Jagd Ztg*), July–December 1881 (*Bibliot Hist-Nat*; *Verz Bücher Landkarten*)] GB, BHL

The series was issued in three volumes, 1876–1881, the third one in two parts (*Abtheilungen*). The first edition was published 1872–1875 [GB] and the first part of the third volume, containing the beetles, was recorded on October 1874 (*Allg Bibl*).

#### Ambrosi, Francesco (Borgo Valsugana, Trentino-Alto Adige, Italy: 17 November 1821 – 9 April 1897: Trento, Trentino-Alto Adige, Italy). Italian librarian and naturalist; appointed director of the library and of the adjoining municipal museum of Trento in 1864. References. Saccardo (1898); Festi (1998, P).

**1852**. VI. Animali. Prospetto delle specie zoologiche conosciute nel Trentino. Pp. 262–346 *in*: *Statistica del Trentino compilata da Agostino Perini. Volume I.* Fratelli Perini, Trento. (4to) [DP: 1852 (title page), 31 May 1853 (*Gaz Tirol*), July–December 1853 (*Bibliot Geo-Stat*)] GB, MDZ

Ambrosi’s contribution consists of an annotated list of the animals found in the province of Trentino in northern Italy; the Coleoptera are on pages 299–323. Despite the date on the title page, this volume may have been issued only in 1853.

#### Amor y Mayor, Fernando (Madrid, Spain: *ca* 1823 – 21 October 1863: San Francisco, California, USA). Spanish biologist; professor of natural history at the Institute of Córdoba as well as director and professor at the Elementary School of Agriculture; collected in Morocco in 1859; participated to the Scientific Commission to the Pacific (1862–1866), headed by naturalist Patricio M. Paz y Membiela, where he became sick and was admitted in a San Francisco hospital where he died. References. Olmedilla y Puig (1872); Roldán Guerrero and Herrero Hinojo (1953, P); Perejón (2012, P).

**1860**. *Memoria sobre los insectos epispásticos de algunas provincias de España. Presentada al Colegio de Farmacéuticos de Madrid.* Manuel Alvarez, Madrid. 36 pp. (8vo) [DP: 1860 (title page; last page dated 21 August 1860), 2 September 1860 (*Siglo Médico*), 15 September 1860 (*Esp Méd*)] GB

This work was also issued the same year in *El Restaurador Farmacéutico* 16: 97–99 [DP: 10 September 1860], 101–103 [DP: 20 September 1860], 105–107 [DP: 30 September 1860], 109–111 [DP: 10 October 1860], 113–115 [DP: 20 October 1860], 117–119 [DP: 31 October 1860].

#### Andersch, Johann David (Königsberg [currently Kaliningrad], Russia: 7 September 1768 – 17 October 1847: Tilsit [currently Sovetsk], Russia]). Prussian physician and entomologist.

**1797**. Entomologische Bemerkungen. Pp. 152–166 *in*: *Entomologisches Taschenbuch für die Anfänger und Liebhaber dieser Wissenschaft auf das Jahr 1797*. *Herausgegeben von David Heinrich Hoppe*. Montag und Weiss, Regensburg. [2] + 252 pp. (8vo) [DP: n.d. (title page), 23 August 1797 (*Allg Lit Ztg*)] CMLE, MDZ

The new beetle species *Cetonia affinis* (p. 154), *Cetonia aenea* (p. 157), *Cetonia albiguttata* (p. 158), *Cetonia obscura* (p. 161) and *Calopus testaceus* (p. 166) are described and the new genus *Boleticola* (p. 166) is made available in this work.

#### Angas, George French (Newcastle upon Tyne, Tyne and Wear, United Kingdom: 25 April 1822 – 4 October 1886: London, United Kingdom). British explorer, naturalist and painter; sailed to Australia in 1843; visited New Zealand in 1844; after his return to England in 1846, visited South Africa and then went back to Australia; appointed secretary to the Australian Museum in Sydney (1853–1860); returned to London with his family in February 1863. References. Mennell (1892: 14–15); Tregenza (1980).

**1846–1847**. *South Australia illustrated.* Thomas McLean, London. iv + 61 pls + text. (Folio) AMNH

This book was published in ten parts, 1846–1847 (Sherborn 1922a: xviii). It was completed by 11 December 1847 (*Monthly Lit Adv*). The cover is dated 1846 and the preface 1 July 1846; however, the title page of the book and the wrappers are dated 1847. Collation and dates of publication for each part are needed.

The Coleoptera are illustrated on plate 50 as follows: 1. *Stigmodera fortnumii*; 2. *Stigmodera variabilis*; 3. *Stigmodera variabilis*; 4. *Stigmodera*. New species. Port Lincoln; 5. *Buprestis scalaris*; 6. *Stigmodera*. New species; 7. *Cyria imperialis*; 8. *Stigmodera suturalis*; 9. *Stigmodera macularia*; 10. *Diphucrania*. New species, inhabiting the grass-tree (*Xanthorrhaea*); 11. *Stigmodera erythrura*; 12. *Anthaxia*. New species; 13. *Stigmodera*. New species; 14. *Pachnoda*. New species; very similar to the African one; 15. *Diaphonia frontalis*; 16. *Rhipicera mystacina*. From the stringy bark forests; 17. Purpuricenus (Cyclodera). New species; 18. *Orthorinus cylindrirostris*; 19. *Chrysolopus spectabilis*; 20. *Amycterus*. New species; 21. *Phoracantha gigas*. New species; 22. *Prionus*; 23. *Catadromus lacordairei*. Rapid Bay; 24. *Scarites silenus*; 25. *Paropsis*. New species; 26. *Paropsis*. New species; 27. *Anoplognathus viridi-aeneus*; 28. *Helaeus gigas*; 29. *Ptomaphila lachrymosa*; 30. *Mordella*; 31. *Silphomorpha*. Although several new species are illustrated, a single one is named, *Phoracantha gigas*. Sherborn (1926b: 2701) gave the date of 1847 for this species.

A facsimile edition, limited to 1000 copies, was published in Sydney, 1967, by A.H. & A.W. Reed [BOT].

#### Apetz, Johann Heinrich Gottfried (Altenburg, Thuringia, Germany: 24 February 1794 – 8 November 1857: Altenburg, Germany). German orientalist and entomologist; deacon in Lurka and from 1830, professor at the Friedrich-Gymnasium in Altenburg. References. Siegfried (1875); Striedl (1953).

**1854**. *De coleopteris, quae Oscarus et Alfredus Brehm in Africa legerunt.* Typographeo Aulico, Altenburgi. 15 pp. (4to) [DP: 1854 (title page)] MCZ, BHL

This work is attached to a 20 page school newsletter entitled “*Sieben und vierzigste Nachricht von dem Friedrichs-Gymnasium zu Altenburg auf das Schuljahr Ostern 1853 bis dahin 1854. Als Einladungsschrift zu den vom 3. bis 7. April 1854 in der Aula des Josephinum stattfindenden Schulfeierlichkeiten von Schulrath D. Heinrich Eduard Foss, Director. Beigefügt ist eine Abhandlung von Prof. J.H. Apetz: De coleopteris, quae Oscarus et Alfredus Brehm in Africa legerunt*.”

The work treats the families Cicindelidae, Carabidae, Dytiscidae, Gyrinidae and Histeridae. Several new species are described.

#### Aragona, Luigi [Aloysius] [d’] Italian physician in Pavia. Reference. Goidanich (1975: 73–74).

**1830**. *De quibusdam coleopteris Italiae novis aut rarioribus tentamen inaugurale quod annuentibus magnifico domino rectore illustrissimo facultatis directore spectabili d. decano ac clarissimis D. D. professoribus auspice J.M. Zendrini historiae naturalis spec. prof. ord. pro doctoris medicinae laurea rite capessenda Aloysius Aragona Ticinensis in celebratiss. I. R. Archigymnasio Ticinensi publicae disquisitioni submittit. Mense Aprilis MDCCCXXX in thesis adnexas disputabitur in Universitatis Aedibus.* Bizzoni, Ticini Regii [= Pavia]. 31 pp. (8vo) [DP: April 1830 (title page)] CNC, GB

This thesis includes notes on some species of Coleoptera and descriptions of several new species from Italy.

#### Ashmead, William Harris (Philadelphia, Pennsylvania, USA: 19 September 1855 – 17 October 1908: Washington DC, USA). American entomologist who published on many groups of insects but particularly Hymenoptera; formed his own publishing house devoted to agriculture in Florida and launched the *Florida Dispatch*, an agricultural weekly magazine; from 1895 on, worked as assistant curator in the Department of Entomology of the U.S. National Museum. References. Howard (1908); Anonymous (1909, P); Essig (1931: 539–542, P); Mallis (1971: 355–357, P).

**1880**. *Orange insects. A treatise on the injurious and beneficial insects found on the orange trees of Florida.* Ashmead Bros., Jacksonville. xv + 78 pp. (8vo) [DP: 1880 (title page), 11 December 1880 (*Publ Weekly*), February 1881 (*Pop Sci Monthly*), 11 March 1881 (*Acad Nat Sci Phila*), March 1881 (*Nat Nov*; *Amer Nat*)] GB

One new beetle species, the coccinellid *Hyperaspidius coccidivora* (pp. 10–11), is described.

#### Asso y del Río, Ignacio Jordán Claudio de (Saragossa, Aragon, Spain: 4 June 1742 – 21 May 1814: Saragossa, Spain). Spanish jurist, diplomat and naturalist, which sometimes used the pseudonym of “Melchor de Azagra”; practicing lawyer (1765–1776); consul in Dunkirk (1776), Amsterdam (1776–1787) and Bordeaux (1787–1791); later, director of the departments of Chemistry and Botany at the Royal Economic Society of Aragon (1797–1802). References. Latassa y Ortin (1802: 94–109); Mora (1972); Anaya Revuelta (1998: 142–144); Villar (2010).

**1784**. *Introductio in oryctographiam, et zoologiam Aragoniae. Accedit enumeratio stirpium in eadem regione noviter detectarum.* 192 pp. + 7 pls. (8vo) [DP: 1784 (title page), April 1785 (*New Rev*)] GB, BHL, MDZ

The publisher and place of publication are not indicated. Although published anonymously, the work is credited to Asso y del Río (e.g., Dryander 1798: 240; Agassiz 1848: 148). It contains an annotated list of the animals of Aragon in northeast Spain. The Coleoptera are on pages 96–109; the known species are named but those not recognized by the author are described but not named.

#### Aubé, Charles Nicolas (Paris, France: 6 May 1802 – 15 October 1869: Crépy-en-Laonnois, Aisne, France). French pharmacist, physician, and entomologist; after working a few years as a pharmacist, studied medicine and defended his thesis on scabies and the mite which causes it; published mainly on families of small and neglected beetles; founding member of the *Société Entomologique de France*; his collection, who included almost all his types, was left to August Grenier after his death, then passed into the hands of Albert Léveillé in 1890, to the *Société Entomologique de France* in 1901, and finally to the Muséum National d’Histoire Naturelle, Paris, in 1930. References. Laboulbène (1870, P); Gouillard (2004: 39–40); Cambefort (2006: 108).

**1836–1838**. *Iconographie et histoire naturelle des coléoptères d’Europe. Tome cinquième. Hydrocanthares.* Méquignon-Marvis Père et Fils, Paris. xi + pp. 13–415 + [1] pp. + 46 pls. (4to) CNC, GB

This work was published in ten livraisons, corresponding to livraisons 47–56 of the entire series started by Dejean and Boisduval, 1829–1836 [*q.v.*]. The first livraison was noticed in the *Annales de la Société Entomologique de France* (p. ciii) as published between 1 October 1836 and 31 December 1836 and recorded by the *Bibliographie de la France* on 25 February 1837 (three *feuilles* and five plates). I have not been able to find any dates of publication regarding the other livraisons. The title page is dated 1836, the preface 12 November 1836. Guignot (1932: 548) researched the dates of publication and indicated that pages 1–64 were distributed in November or early December 1836, pages 65–224 between April and December 1837, and pages 225–415 in 1838. At the request of Frank Balfour-Browne, F.J. Griffin, then registrar of the Royal Entomological Society of London, found that pages 1–48 of this work were issued between 1 October and 1 December 1836 (Balfour-Browne 1936: 105). This corroborates the *Bibliographie de la France* entry which noticed three *feuilles* of text (48 pp.). At this time, the dates of publication that should be retained are: (pp. 1–48) 1 December 1836; (pp. 49–224) 31 December 1837; (pp. 225–415) 31 December 1838.

**1838**. *Species général des hydrocanthares et gyriniens; pour faire suite au species général des coléoptères de la collection de M. le comte Dejean.* Méquignon Père et Fils, Paris. xvi + 804 pp. (8vo) [DP: 1838 (title page; preface dated 1 August 1838), 29 September 1838 (*Bibl Fr*), 12 October 1838 (*Allg Bibl Deutsch*), 17 October 1838 (*Lit Ztg*)] CNC, GB, GAL

#### Aubert, Louis Jules Antoine (Saint-Nazaire [today Sanary-sur-Mer], Var, France: 25 August 1854 – ?). French naval physician.

**1887**. *Étude sur les insectes vésicants en général et essai sur quelques espèces exotiques en particulier.* Boehm et Fils, Montpellier. 80 pp. (8vo) [DP: 1887 (title page), 22 September 1887 (*Rev Clin Thérap* 1: 496), 29 October 1887 (*Bibl Fr*), November 1887 (*Nat Nov*), 9 January 1888 (*Zool Anz*), January 1888 (*Polybiblion*)] GAL

This work was first presented as a thesis at the *Faculté de médecine* of the *Académie de Montpellier* in France.

#### Aucher-Éloy, Pierre Martin Remi (Blois, Loir-et-Cher, France: 2 October 1792 – 6 October 1838: near Ispahan, Iran). French printer, pharmacist and botanist; operated a bookstore and print shop in Blois; lived in Istanbul from 1830 and collected plants through Asia Minor. References. Prévost (1948); Marshall (1994: 25).

**1826**. *L’entomologie, ou l’histoire naturelle des insectes enseignée en 15 leçons. Ouvrage contenant: les principes élémentaires de cette science, l’histoire des mœurs et des métamorphoses des insectes, la méthode de classification de Geoffroy, et une méthode analytique a l’aide de laquelle on peut seul, et en quelques minutes connaître le nom générique de tous les insectes connus; orné de 75 figures en taille douce.* Aucher-Eloy et Comp^ie^, Paris. ii + 437 + [1 (Table des matières)] pp. + 9 pls. (12mo) [DP: 1826 (title page), 26 July 1826 (*Bibl Fr*), 8 August 1826 (*Nouveauté*), August 1826 (*Rev Encycl*), 2 September 1826 (*Globe*)] BHL

The book was published under the initials “R.A.E.” The Coleoptera are on pages 78–151 and 377–390.

The work was also issued in 1827 [GB] by the same publisher under the same title except that the sentence “*ouvrage dans lequel on a suivi la méthode de classification de M. Duméril*” was added before “*orné de 75 figures en taille douce*.”

#### Audinet-Serville, Jean-Guillaume (Paris, France: 11 November 1775 – 27 March 1858: La Ferté-sous-Jouarre, Seine-et-Marne, France). French entomologist known mostly for his work on Orthoptera although he published important works on beetles, including the first classification of the family Cerambycidae; founding member of the *Société Entomologique de France* and its first president in 1832; his beetle collection was purchased by Léon Dufour [*q.v.*]. References. Amyot (1858); Gouillard (2004: 20–21); Cambefort (2006: 108–109); Aguilar (2004, P); Delfosse (2014, P).

**1821**. *Faune française, ou histoire naturelle, générale et particulière des animaux qui se trouvent en France, constamment ou passagèrement, à la surface du sol, dans les eaux qui le baignent, et dans le littoral des mers qui le bornent.* F.-G. Levrault, Paris. 96 pp. + 20 pls. (8vo) [DP: n.d. (cover), 31 March 1821 (*Bibl Fr*), April 1821 (*Rev Encycl*), August 1821 (*Not Geb Natur*)] HOU

This section constitutes livraisons 1 and 2 (but registered as livraison 1) of the *Faune Française* which was issued in 29 livraisons, 1821–1830. There is no date on the cover of the copy I have seen but Bedel (1877: lxxxvi), who commented on all new species described in these two livraisons, mentioned that it was published in 1821. This is corroborated by the entry in the *Bibliographie de la France*. A new edition of these two livraisons was issued in 1829 [*q.v.*] along with livraison 24.

The *Faune Française* was published with the plates available colored or uncolored; a few copies were available in 4to.

**1829–1830**. *Faune française, ou histoire naturelle, générale et particulière, des animaux qui se trouvent en France, constamment ou passagèrement, à la surface du sol, dans les eaux qui le baignent, et dans le littoral des mers qui le bornent. Coléoptères.* Plassan, Paris. 240 pp. + 22 pls. (8vo) ANSP

This portion of the *Faune Française* was issued in two livraisons: **24**: (pp. 1–160) 17 October 1829 (*Bibl Fr*), 30 November 1829 (*Rev Bibl-Brus*); **27**: (pp. 161–240) 8 May 1830 (*Bibl Fr*), 31 July 1830 (*Rev Bibl-Brus*). Pages 1–80 constitute a new edition of the 1821 version (Sherborn and Woodward 1901b: 493).

#### Audouin, Jean-Victor (Paris, France: 27 April 1797 – 9 November 1841: Paris, France). French educator and invertebrate zoologist; assistant librarian at the Muséum National d’Histoire Naturelle, Paris, in 1823; obtained his doctorate in 1826; succeeded Latreille as assistant naturalist of the Crustaceans and Insects chair at the Muséum in 1830 and three year later as professor of the same chair; died of apoplexy; co-founder of the *Annales des Sciences Naturelles* and a founding member of the *Société Entomologique de France*; had no insect collection. References. Duponchel (1842); Milne-Edwards (1851); Théodoridès (1978); Mearns and Mearns (1988: 31–33, P); Jaussaud (2004: 48–49, P); Adler (2012: 84–85, P).

**1836–1849**. [Audouin, J.V., Blanchard, E., Doyère, L. and Milne-Edwards, H.] *Le règne animal distribué d’après son organisation, pour servir de base à l’histoire naturelle des animaux, et d’introduction à l’anatomie comparée, par Georges Cuvier. Edition accompagnée de planches gravées, représentant les types de tous les genres, les caractères distinctifs des divers groupes et les modifications de structure sur lesquelles repose cette classification; par une réunion de disciples de Cuvier, MM. Audouin, Blanchard, Deshayes, Alcide d’Orbigny, Doyère, Dugès, Duvernoy, Laurillard, Milne Edwards, Roulin et Valenciennes. / Les insectes. Avec un atlas. Myriapodes, thysanoures, parasites, suceurs et coléoptères. Texte* [I]. Fortin, Masson et C^ie^, Paris. xii + 557 pp. (4to) CNC, BHL

This is the third (also called the Disciples’) edition of Cuvier’s *Le Règne animal*. It was published in 262 livraisons, each containing a mixture of text and plates for each class. Completed sets of livraisons were subsequently bound into 20 volumes divided into sections. The insect section comprised two volumes, each with two parts (text and atlas [*vide infra*]).

The Coleoptera are on pages 81–538 of the first volume, issued in the following livraisons: **112**: (pp. 81–88) January 1841; **123**: (pp. 89–96) July 1841; **126**: (pp. 97–104) August 1841; **132**: (pp. 105–112) November 1841; **138**: (pp. 113–120) February 1842; **155**: (pp. 121–128) October 1842^11^; **159**: (pp. 129–136) December 1842; **162**: (pp. 137–144 [pages 145–146 were not printed]) January 1843, 9 February 1843 (Sherborn 1922b: 555); **171**: (pp. 147–154) 11 May 1843 (Sherborn 1922b: 555), May 1843; **175**: (pp. 155–162) July 1843; **181**: (pp. 163–202) 10 August 1843 (Sherborn 1922b: 555), September 1843; **183**: (203–242) October 1843; **186**: (pp. 243–282) November 1843; **189**: (pp. 283–322) January 1844; **193**: (pp. 323–362) March 1844; **198**: (pp. 363–402) May 1844; **201**: (pp. 403–442) 26 July 1844 (Sherborn 1922b: 555), July 1844; **205**: (pp. 443–450) September 1844; **207**: (pp. 451–490) November 1844; **209**: (pp. 491–530) December 1844; **210**: (pp. 531–555) 17 January 1845 (Sherborn 1922b: 555), January 1845. The dates of publication are from Cowan (1976: 55–56) unless otherwise noted.

**1836–1849**. [Audouin, J.V., Blanchard, E., Doyère, L. and Milne-Edwards, H.] *Le règne animal distribué d’après son organisation, pour servir de base à l’histoire naturelle des animaux, et d’introduction à l’anatomie comparée, par Georges Cuvier. Edition accompagnée de planches gravées, représentant les types de tous les genres, les caractères distinctifs des divers groupes et les modifications de structure sur lesquelles repose cette classification; par une réunion de disciples de Cuvier, MM. Audouin, Blanchard, Deshayes, Alcide d’Orbigny, Doyère, Dugès, Duvernoy, Laurillard, Milne Edwards, Roulin et Valenciennes. / Les insectes. Avec un atlas. Myriapodes, thysanoures, parasites, suceurs et coléoptères. Atlas* [I]. Fortin, Masson et C^ie^, Paris. 83 pls. (4to) CNC, BHL

The Coleoptera are on plates 15–75 (including 22*bis*, 39*bis*, 40*bis*, 44*bis*, 53*bis*, 66*bis*, and 74*bis*). The publication dates of these plates, as noted by Cowan (1976: 55–56) unless otherwise noted, are as follows: **Plate 15**: September 1846; **16**: August 1839; **17**: March 1839; **18–19**: May 1845; **20**: August 1841; **21–22**: February 1842; **22*bis*–25**: November 1841; **26–27**: February 1842; **28–31**: October 1842; **32–35**: December 1842; **36–37**: 11 May 1843 (Evenhuis 1997a: 179), May 1843; **38 [39*bis*]–40**: January 1843, 10 February 1843 (Evenhuis 1997a: 179); **40*bis***: July 1841; **41**: 11 May 1843 (Evenhuis 1997a: 179), May 1843; **42–43**: July 1843; **44**: 10 August 1843 (Evenhuis 1997a: 179), September 1843; **45**: July 1843; **45*bis***: 10 August 1843 (Evenhuis 1997a: 179), September 1843; **46–47**: July 1841; **48–49**: January 1844; **50–51**: March 1844; **52**: May 1844; **53–53*bis***: September 1844; **54**: August 1841; **55**: January 1841; **56**: November 1843; **57**: November 1844; **58**: September 1844; **59–60**: December 1844; **61–62**: November 1843; **63**: October 1843; **64**: August 1841; **65**: 10 August 1843 (Evenhuis 1997a: 179), September 1843; **66–66*bis***: October 1843; **67–69**: January 1841; **70**: August 1840; **71**: July 1841; **72**: August 1841; **73**: January 1844; **74**: March 1844; **74*bis*–75**: May 1844.

The names of the insects depicted on the plates are considered type-species designations as indicated in the title of the work. Plates 16–17 are signed “L.D. [= L. Doyère] et V. [= probably Valenciennes]” and plates 18–75 “Em. Bl.” or “Emile Bl. [= Emile Blanchard]” to whom type-species designations should be attributed. Plate 15 shows beetle larvae (and a pupa) of various families. The species depicted on plates 16–75 are listed in Appendix 1.

#### Austin, Edward Payson. American engineer, botanist and coleopterist; worked as an assistant engineer for the United States Lake Survey (1859–1863); employed by the Office of the Nautical Almanac in Washington DC and by the US War Department of Engineers; later, worked as assistant at the astronomical observatory connected with Harward University. Reference. Pringle (1989: 15–16).

**1880**. *Supplement to the check list of the Coleoptera of America, north of Mexico.* S.E. Cassino, Boston. 67 pp. (8vo) [DP: 1880 (title page; preface dated August 1880), 12 November 1880 (*Amer Ent Soc*), December 1880 (*Amer Ent* 3: 295), January 1881 (*Nat Nov*)] MCZ, GB

This publication is a supplement to Crotch’s *Check list of the Coleoptera of America, north of Mexico*, 1874 [*q.v.*].

#### Bach, Michael (Boppard, Rhineland-Palatinate, Germany: 19 March 1808 – 17 April 1878: Boppard, Germany). German educator, botanist, and entomologist in Boppard; received a doctorate *honoris causa* from the University of Bonn; his interest in natural history was directed first to botany and later to entomology, particularly beetles; his collection is at the Philipps-Universität Marburg. References. Kehrein (1868: 10); Anonymous (1878a); Soukup and Schober (2009: 23).

**1849–1867**. *Käferfauna für Nord- und Mitteldeutschland mit besonderer Rücksicht auf die preußischen Rheinlande.* J. Hölscher, Coblenz. (8vo) CNC, SME, GB, MDZ

*Erster Band.* [6] + 413 + [1] pp. This volume was published in two *Lieferungen*: **1**: (pp. 1–336) 5 July 1849 (*Ent Ver Stettin*), 15 October 1849 (*Intell Serapeum*), 5 November 1849 (*Ent Soc Lond*), July–December 1849 (*Verz Bücher Landkarten*); **2**: (pp. 337–413 + [6]) 1851 (title page), 29 July 1851 (*Börsen Deutsch Buch*), 31 July 1851 (*Allg Bibl Deutsch*), 23 August 1851 (*Lit Centr Deutsch*), 31 August 1851 (*Intell Serapeum*), July–December 1851 (*Bibliot Hist-Nat*).

*Zweiter Band.* [6] + 392 pp. This volume was published in two *Lieferungen*: **3**: (pp. 1–148) 1852 (colophon on p. 148), 20 September 1852 (*Börsen Deutsch Buch*), 23 September 1852 (*Allg Bibl Deutsch*), July–September 1852 (*Cat Muquardt*), July–December 1852 (*Bibliot Hist-Nat*), January 1853 (*Ent Ver Stettin*); **4**: (pp. 149–392) 1854 (title page), 16 March 1854 (*Allg Bibl Deutsch*), January–March 1854 (*Cat Muquardt*), January–June 1854 (*Bibliot Hist-Nat*), 2 October 1854 (*Ent Soc Lond*).

*Dritter Band.* 317 pp. This volume was published in two *Lieferungen*: **5**: (pp. 1–142) 1856 (title page), 6 December 1855 (*Allg Bibl Deutsch*), 31 January 1856 (*Intell Serapeum*), January–June 1856 (*Bibliot Hist-Nat*); **6**: (pp. 143–317) 21 October 1858 (*Allg Bibl Deutsch*), 31 October 1858 (*Intell Serapeum*), November 1858 (*Allg Bibl*), July–December 1858 (*Bibliot Hist-Nat*), 1859 (Hagen 1862: 23; Horn and Schenkling 1928a: 32).

*Vierter Band.* [2] + 292 pp. [DP: 1860 (title page), 16 February 1860 (*Allg Bibl Deutsch*), March 1860 (*Allg Bibl*), January–June 1860 (*Bibliot Hist-Nat*)].

*Schlußlieferung. Enthält Nachträge zu Band 1–4 nebst System. Verzeichniß.* [1] + pp. 415–523 + 393–493 + 319–364 + 293–308. [DP: 1867 (title page), 24 April 1867 (*Börsen Deutsch Buch*), April 1867 (*Allg Bibl*), 2 May 1867 (*Allg Bibl Deutsch*), January–June 1867 (*Bibliot Hist-Nat*), 31 August 1867 (*Intell Serapeum*)]. This part contains five sections: *Nachträge, Zusätze und Verbesserungen zum 1. Bande der Käferfauna*, paginated 415–523, *Nachträge, Zusätze und Verbesserungen zum 2. Bande der Käferfauna*, paginated 393–493, *Nachträge, Zusätze und Verbesserungen zum 3. Bande der Käferfauna*, paginated 319–364, *Zusätze und Verbesserungen zum vierten Bande der Käferfauna*, paginated 293–299, and *Alphabetisches Verzeichniß der Käfergattungen aller vier Bände*, paginated 300–308.

The title on the cover of the *Lieferungen*, at least for the first one, was “*Käferfauna der preussischen Rheinlande mit besonderer Rücksicht auf Nord- und Mitteldeutschland*.”

**1866**. *Systematisches Verzeichniss der Käfer Deutschlands, gehörend zur Käferfauna.* J. Hölscher, Coblenz. lxiv pp. (8vo) [DP: 1866 (title page), 21 December 1866 (*Börsen Deutsch Buch*)] SME

#### Baczko, Ludwig Franz Josef Adolf von (Lyck [currently Ełk], Poland: 8 June 1756 – 27 March 1823: Königsberg [currently Kaliningrad], Russia). Prussian educator and writer; became completely blind at 21 years of age following an attack of smallpox; professor at the Königsberg Artillery Academy; in 1816 appointed head of the Königsberg Institute for the blind. References. Doering (1831: 33–36); Wegele (1875); Brümmer (1876: 30–31).

**1800**. *Reise durch einen Theil Preussens. Erstes Bändchen.* Gottfried Vollmer, Hamburg und Altona. [2] + 238 pp. (8vo) [DP: 1800 (title page), 4 May 1800 (*Germ Mus* 2: 79)^12^, May 1800 (*Polit J*)] GB

Baczko’s journey through part of Prussia was issued in two volumes, 1800. It was also issued under the title “*Nankes Wanderungen durch Preussen.*” One new beetle species, *Opatrum campestre* (p. 21), is described in the first volume; the species is not recorded in Sherborn’s *Index Animalium*.

#### Baldassini, Francesco (Marquis) (Pesaro, Marche, Italy: 15 November 1785 – 13 January 1857: Pesaro, Italy). Italian naturalist and translator. Reference. Strafforello and Treves (1878: 171).

**1834**. *Storia naturale degli animali invertebrati del Sig. Cavaliere de Lamarck compendiata ed arricchita di note.* Annesio Nobili, Pesaro. 391 + [5] pp. (8vo) [DP: 1834 (title page; p. [392] dated 4 October 1834), January 1835 (*Bibl Ital-1*), June 1835 (*Bibliot Ital*)] GB

This work is an emended Italian resumé of Lamarck’s *Histoire naturelle des animaux sans vertèbres*, 1815–1822 [*q.v.*]. The Coleoptera are on pages 186–215.

#### Baly, Joseph Sugar (Warwick, Warwickshire, United Kingdom: 1816 – 25 March 1890: Warwick, United Kingdom). British physician and entomologist, specialist on phytophagous Coleoptera; surgeon at Kentish Town and Camden Town dispensaries in London, later (1868–90) at Warwick; most of his beetle specimens, including his types, are at the Natural History Museum in London. References. Sharp (1890); Staines and Staines (1999).

**1858**. *Catalogue of Hispidae in the collection of the British Museum. Part I.* Printed by order of the Trustees, London. x + 172 + [11] pp. + 9 pls. (8vo) [DP: 1858 (title page; preface dated 1 November 1858), January 1859 (Anonymous 1859d: 3), 7 February 1859 (*Ent Soc Lond*), 12 February 1859 (Sherborn 1934: 310)] CNC, BHL, GB

No other parts were published.

**1864**. *Descriptions of new genera and species of Phytophaga.* [London]. 16 pp. (8vo) [DP: 19 April 1864 (p. 1), 2 May 1864 (*Ent Soc Lond*)] GB

**1885–1886**. Fam. Hispidae. Pp. 1–124 + pls 1–4 *in*: *Biologia Centrali-Americana. Insecta. Coleoptera. Vol. VI. Part 2. Phytophaga (part). Hispidae by J.S. Baly, with an appendix by G.C. Champion. Cassididae by C.G. Champion.* Taylor and Francis, London. x + 249 pp. + 13 pls. (4to) CNC

This section was published in eight parts as follows (see Lyal 2011: 88): (pp. 1–16 + pl. 1) January 1885; (pp. 17–24 + pl. 2) March 1885; (pp. 25–40) April 1885; (pp. 41–56 + pl. 3) May 1885; (pp. 57–72) November 1885; (pp. 73–96) February 1886; (pp. 97–112 + pl. 4) April 1886; (pp. 113–124) May 1886.

**1890**. Phytophaga. Pp. 25–36 *in*: *Scientific results of the second Yarkand mission; based upon the collections and notes of the late Ferdinand Stoliczka, Ph.D. Coleoptera.* Office of Superintendent of Government printing, Calcutta. 79 pp. + 2 pls. (4to) [DP: 1890 (title page; colophon dated 13 November 1890), 4 March 1891 (*Asia Soc Beng*), 10 June 1891 (*Vetens Akad*), 21 June 1891 (*Accad Lincei*), 6 August 1891 (*Nature*), 19 September 1891 [JD] (*Soc Imp Nat Moscou*)] CAKL, GB

The “*Scientific results of the second Yarkand mission*” was issued in several parts, 1878–1891.

#### Barbut, James [Jaques] (*ca* 1711 – 31 May 1791: London, United Kingdom). British painter, naturalist, and bank officer. References. Evenhuis (1997a: 68); Damkaer (2002: 96–98).

**1781**. *The genera insectorum of Linnaeus exemplified by various specimens of English insects drawn from nature.* J. Sewell, London. [2] + xvii + [1] + 371 + [20] pp. + 22 pls. (4to) [DP: 1781 (title page), August 1781 (*Univers Mag*)] GDZ, CNC (mf), GB

The text is written in English and French, mostly in parallel columns. The French title page reads “*Les genres des insectes de Linné; constatés par divers échantillons d’insectes d’Angleterre, copiés d’après nature.*” The Coleoptera are on pages 10–95 and plates 1–7.

Engelmann (1846: 521), Graesse (1859: 290) and other bibliographers mentioned a second edition, essentially a reissue of the first edition with a change of title page (Evenhuis 1997a: 70), published in 1783 (*n.v.*).

#### Barthélemy Lapommeraye, Christophe-Jérôme (Marseille, Bouches-du-Rhönes, France: 13 April 1796 – 4 October 1869: Marseille, France). French naturalist; director of the museum of natural history and of the zoological garden in Marseille; Knight of the Legion of Honor. Reference. Saurel (1877: 107).

**1846**. *Carabe d’Agassiz*, Carabus Agassizi. Barlatier & Co., Marseille. 4 pp. (8vo) [DP: n.d. (title page), 28 October 1846 (*Soc Ent Fr*), 2 November 1846 (*Soc Géol Fr*)] MCZ

This small work, containing the description of a fossil *Carabus* found at Aix in France, was originally presented at the *Congrès Scientifique de France* on 10 September 1846 and reprinted as a separate pamphlet before the Congrès proceedings [GB] were published in May 1847. It is usually dated 1850 (Guérin-Méneville 1851: 203; Hagen 1862: 30; Horn and Schenkling 1928a: 44).

#### Bates, Frederick (Leicester, Leicestershire, United Kingdom: 18 November 1829 – 6 October 1903: London, United Kingdom). British naturalist and director of a brewery in Leicester; worked mainly on beetles, particularly *Heteromera*, but also interested in fresh-water algae and orchids; younger brother of Henry Walter Bates [*q.v.*]; his collection of British beetles passed on to Horace Donisthorpe, while his collection of *Heteromera* of the world, comprising 22400 specimens representing 7200 species, was purchased by the British Museum (Natural History), now the Natural History Museum, in 1881 and 1897. References. Donisthorpe (1903); Harvey et al. (1996: 14).

**1890**. Heteromera. Pp. 55–79 + 1 pl. *in*: *Scientific results of the second Yarkand mission; based upon the collections and notes of the late Ferdinand Stoliczka, Ph.D. Coleoptera. Published by order of the government of India.* Office of Superintendent of Government printing, Calcutta. 79 pp. + 2 pls. (4to) [DP: 1890 (title page; colophon dated 13 November 1890), 4 March 1891 (*Asia Soc Beng*), 10 June 1891 (*Vetens Akad*), 21 June 1891 (*Accad Lincei*), 6 August 1891 (*Nature*), 19 September 1891 [JD] (*Soc Imp Nat Moscou*)] CAKL, GB

The “*Scientific results of the second Yarkand mission*” was issued in several parts, 1878–1891.

#### Bates, Henry Walter (Leicester, Leicestershire, United Kingdom: 8 February 1825 – 16 February 1892: London, United Kingdom). British naturalist with a passion for Lepidoptera and Coleoptera (particularly Carabidae and Cerambycidae); at the age of 23, embarked on a collecting expedition to the Amazon Valley with his friend Alfred Russel Wallace; returned to London 11 years later and spent the next few years writing taxonomic works on butterflies and beetles of the Amazon and his book, *The Naturalist on the River Amazons*; developed his theory of mimicry; from 1864 until his death, assistant secretary at the Royal Geographical Society of London; his personal collections of Endomychidae and Elateridae are now in the Natural History Museum in London while the remaining groups were acquired by René Oberthür and are now in the Muséum National d’Histoire Naturelle in Paris. References. Bouchard (1892, P); Papavero (1973: 256–261); Dickenson (1992); O’Hara (1995); Dickenson (2004a, P); Cambefort (2006: 118–119).

**1879–1885**. Tribe Longicornia. Pp. 1–436 + pls 1–25 *in*: *Biologia Centrali-Americana. Insecta. Coleoptera. Vol. V. Longicornia by Henry Walter Bates, F.R.S. Bruchides by David Sharp, M.B.* Taylor and Francis, London. xii + 525 pp. + 26 pls. (4to) CNC

This work was issued in several parts. The dates listed at the bottom of the first page of each sheet are: (pp. 1–16 + pls 1, 2) November 1879; (pp. 17–40 + pls 3, 4) February 1880; (pp. 41–64 + pl. 5) April 1880; (pp. 65–88 + pl. 6) June 1880; (pp. 89–128 + pls 7–9) August 1880; (pp. 129–152 + pls 10, 11) October 1880; (pp. 153–168 + pl. 12) February 1881; (pp. 169–192 + pl. 13) April 1881; (pp. 193–224 + pls 14, 15) June 1881. *Supplement*. (pp. 225–248 + pl. 16) December 1884; (pp. 249–272 + pl. 17) January 1885; (pp. 273– 312 + pls 18, 19) March 1885; (pp. 313–328 + pl. 20) April 1885; (pp. 329–352 + pl. 21) May 1885; (pp. 353–384 + pl. 22) July 1885; (pp. 385–408) August 1885; (pp. 409–436 + pls 23–25) October 1885. The collation for the plates is from Lyal (2011: 86).

**1881–1884**. *Biologia Centrali-Americana. Insecta. Coleoptera. Vol. I. Part 1.* Taylor and Francis, London. x + 316 pp. + 13 pls. (4to) CNC

This work was published in several parts. The dates listed at the bottom of the first page of each sheet are: (pp. 1–16 + pl. 1) October 1881; (pp. 17–40 + pl. 2) December 1881; (pp. 41–72 + pl. 3) February 1882; (pp. 73–88) April 1882; (pp. 89–112 + pl. 4) June 1882; (pp. 113–136 + pl. 5) August 1882; (pp. 137–152) October 1882; (pp. 153–168 + pl. 6) January 1883; (pp. 169–216 + pl. 7) March 1883; (pp. 217–240 + pls 8, 9) September 1883; (pp. 241–256 + pls 10–12) December 1883; (pp. 257–296 + pl. 13) July 1884; (pp. 297–312) August 1884; (pp. 313–316 + [i]–x) December 1884. The collation for the plates is from Lyal (2011: 79). The volume covers the Cicindelidae and Carabidae. The title page is dated 1881–1884.

**1886–1890**. *Biologia Centrali-Americana. Insecta. Coleoptera. Vol. II. Part 2. Pectinicornia and Lamellicornia.* Taylor and Francis, London. xii + 432 pp. + 24 pls. (4to) CNC

This work was published in several parts. The dates listed at the bottom of the first page of each sheet are: (pp. 1–24 + pl. 1) May 1886; (pp. 25–64 + pls 2, 3) May 1887; (pp. 65–80 + pl. 4) June 1887; (pp. 81–88 + pl. 5) August 1887; (pp. 89–104 + pl. 6) September 1887; (pp. 105–120 + pl. 7) October 1887; (pp. 121–136) November 1887; (pp. 137–160 + pl. 8) December 1887; (pp. 161–176 + pl. 9) January 1888; (pp. 177–200) February 1888; (pp. 201–216 + pls 10, 11) April 1888; (pp. 217–248 + pls 12, 13) June 1888; (pp. 249–296 + pls 14, 15) August 1888; (pp. 297–312 + pl. 16) October 1888; (pl. 17) November 1888; (pp. 313–336) December 1888; (pl. 18) February 1889; (pp. 337–376 + pls 19–21) March 1889; (pls 22, 23) April 1889; (pp. 377–384) October 1889; (pp. 385–416 + pl. 24) December 1889; (pp. 417–432 + [1]–xii) January 1890. The collation for the plates is from Lyal (2011: 81). The volume covers the families Lucanidae (pp. 1–2), Passalidae (pp. 2–24), Copridae (pp. 25–83), Aphodiidae (pp. 83–104), Orphnidae (pp. 105–107), Hybosoridae (pp. 107–108), Geotrupidae (pp. 108–115), Trogidae (pp. 116–129), Aclopidae (pp. 129–130), Chasmatopteridae (p. 130), Melolonthidae (pp. 130–215), Rutelidae (pp. 216–296), Dynastidae (pp. 296–342), Cetoniidae (pp. 343–376), Trichiidae (pp. 377–381). It also includes a supplement (pp. 382–416). The title page is dated 1886–1890.

**1890**. Geodephaga and Longicornia. Pp. 1–23 + 1 pl. *in*: *Scientific results of the second Yarkand mission; based upon the collections and notes of the late Ferdinand Stoliczka, Ph.D. Coleoptera. Published by order of the government of India.* Office of Superintendent of Government printing, Calcutta. 79 pp. + 2 pls. (4to) [DP: 1890 (title page; colophon dated 13 November 1890), 4 March 1891 (*Asia Soc Beng*), 10 June 1891 (*Vetens Akad*), 21 June 1891 (*Accad Lincei*), 6 August 1891 (*Nature*), 19 September 1891 [JD] (*Soc Imp Nat Moscou*)] CAKL, GB

**1890**. List of Coleoptera collected by Mr. Jameson on the Aruwimi. Pp. 423–425 *in*: *Story of the rear column of the Emin Pasha relief expedition. By the late James S. Jameson, naturalist to the expedition. Edited by Mrs. J.S. Jameson. With portrait, map, and illustrations from the author’s sketches.* R.H. Porter, London. xxii + [1 (Errata)] + 455 pp. (8vo) [DP: 1890 (title page; editor’s note dated 12 December 1890), 27 December 1890 (*Athenaeum*; *Spectator*), 24 January 1891 (*Publ Weekly*), January 1891 (*English Cat Books*; *Nat Nov*), 5 February 1891 (*Nation*)] McG

A note below Bates’ name on page 423 reads “*The Curculionidae have been named and described by Mr. H.W. Simpson, who has paid especial attention to this family*.” Therefore the following new taxa are to be credited to Simpson: *Xenostes* (Lithinidae), n. gen. (p. 424), *Xenostes jamesoni*, n.sp. (p. 424) and *Oxyopisthen pygidiale*, n.sp. (p. 425). The only new species described by Bates is *Tefflus jamesoni* Bates, n.sp. (p. 423).

An “authorized edition” was issued in New York [ARC] and in Toronto in 1891 [BHL].

**1892**. Coleoptera. Pp. 7–39 *in*: *Supplementary appendix to travels amongst the Great Andes of the Equator by Edward Whymper with contributions by H.W. Bates. T.G. Bonney. G.A. Boulenger. Peter Cameron. F. Day. W.L. Distant. A.E. Eaton, F.D. Godman. H.S. Gorham. Martin Jacoby. E.J. Miers. A. Sidney Olliff. O. Salvin. David Sharp. T.R.R. Stebbing. Illustrated.* John Murray, London. xxii + [1 (Addenda)] + 147 pp. + 14 pls. (4to) [DP: 1891 (title page), 2 March 1892 (Sharp 1892: 61), 18 March 1892 (*Science*), 26 March 1892 (*Athenaeum*), 9 April 1892 (*Publ Circ*), April 1892 (*Nat Nov*), 16 June 1892 (*Nature*), 13 July 1892 (*Soc Ent Fr*)] CNC, GB

According to Sharp (1892: 61), the printing was done in 1891 but the work was not available for purchase until 2 March 1892.

**1893**. On new genera and species of coleopterous insects from Mount Kina Balu, North Borneo. Pp. 289–295 *in*: *Exploration of Mount Kina Balu, North Borneo. By John Whitehead. With coloured plates and original illustrations.* Gurney and Jackson, London. x + [2] + 317 pp. + 32 pls. (Folio) [DP: 1893 (title page; introduction dated 10 May 1893), 22 July 1893 (*Athenaeum*), July 1893 (*English Cat Books*), August 1893 (*Nat Nov*)] MCZ, GB

Bates’ contribution was first published in the *Proceedings of the Zoological Society of London* 1889: 383–393.

#### Batsch, August Johann Georg Karl (Jena, Thuringia, Germany: 28 October 1761 – 29 September 1802: Jena, Germany). German naturalist, known for his contribution on mushrooms; professor at the University of Jena. References. Jessen (1875); Ziegenspeck (1953).

**1789**. *Versuch einer Anleitung, zur Kenntniss und Geschichte der Thiere und Mineralien, für akademische Vorlesungen entworfen, und mit den nöthigsten Abbildungen versehen. Zweyter Theil. Besondre Geschichte der Insekten, Gewürme und Mineralien. Mit zwey Kupfertafeln.* Jena. Pp. 529–890 [last pages numbered 661–690] + [2 (Druckfehler)] pp. + 2 pls. (8vo) [DP: 1789 (title page), 1 May 1790 (*Goth Ztg*)] GB

The entire work was issued in two volumes, 1788–1789, continuously paginated. The Coleoptera are on pages 549–581 of the second volume.

#### Bau, Alexander (Berlin, Germany: 31 January 1853 – 2 July 1926: Lochau, Vorarlberg, Austria). German pyrotechnician, entertainment club manager in Berlin, entomologist and ornithologist. References. Hinrichsen (1887: 28); Schalow (1919: 589–594, P).

**1888**. *Handbuch für Käfer-Sammler. Beschreibung der in Deutschland, Oesterreich-Ungarn und der Schweiz vorkommenden Coleopteren. Mit 144 naturgetreuen Zeichnungen im Text*. Creutz, Magdeburg. iv + 494 pp. (8vo) [DP: 1888 (title page; *Vorwort* dated May 1888), 1 July 1888 (*Soc Ent*; *Ent Zeit* 2: 44), 19 July 1888 (*Isis Lieb*), July 1888 (*Nat Nov*; *Humboldt* 7: 364), 20 September 1888 (*Wien Ent Ztg* 7: 252), September 1888 (*Ent Nachr* 14: 271)] CMLE, GB

#### Baudet-Lafarge, Mathieu Jean (Maringues, Puy-de-Dôme, France: 8 November 1765 – 2 May 1837: Maringues, France). French administrator, general councilor, magistrate, deputy of Puy-de-Dôme at the *Conseil des Cinq-Cents*, and coleopterist; founding member of the *Société Entomologique de France*; built a relatively large collection of beetles which he bequeathed to the Faculté des Sciences de Clermont^13^. References. Tailhand (1844); Robert and Cougny (1889: 200–201).

**1809**. *Essai sur l’entomologie du Département du Puy-de-Dôme. Monographie des lamelli-antennes*. Landriot, Clermont. xvi + 86 + [1 (Errata)] pp. (8vo) [DP: 1809 (title page; preface dated 12 September 1809)] CNC

[**1838**]. *Essai sur l’entomologie du Département de Puy-de-Dôme, monographie des carabiques.* Thibaud-Landriot, Clermont-Ferrand. 127 [last two pages numbered 226 and 227] pp. (8vo) [DP: 1836 (title page), 7 February 1838 (*Soc Ent Fr*), 10 March 1838 (*Bibl Fr*)] CNC, GB

The first 69 pages of this work were also published in the *Annales scientifiques, littéraires et industrielles de l’Auvergne* 9 [1836]: 535–570 and 10 [1837]: 160–192. Despite the date on the title page, this work may have been published late 1837 or early 1838. Quérard (1842: 189), Engelmann (1846: 521) and Gonod (1849: 62) dated the book 1838.

#### Baudi di Selve, Flaminio (Savigliano, Piedmont, Italy: 7 July 1821 – 26 June 1901: Genoa, Piedmont, Italy). Italian entomologist who specialised on Coleoptera and Heteroptera; cavalier; municipal councillor of the city of Genoa; his Coleoptera collection is at the Museo di Zoologia dell’Università di Torino. Reference. Camerano (1901).

**1886**. *Rassegna delle specie della famiglia dei Milabridi (Bruchidi degli autori) viventi in Europa e regioni finitime.* Virzi, Palermo. 136 + [2] pp. (4to) GB

This work was issued, with separate pagination, in *Il Naturalista Siciliano* **5** [10]: 1–?12 [DP: 1 July 1886]; [11]: ?13–?32 [DP: 1 August 1886]; [12]: ?33–52 [DP: 1 September 1886]; **6** [1]: 53–72 [DP: 1 October 1886]; [2]: 73–92 [DP: 1 November 1886]; [3]: 93–112 [DP: 1 December 1886]; [4–5]: 113–136 + [2] [DP: 1 February 1887]. The cover of the separate [GDZ] reads “*Rassegna dei Milabridi (Bruchidi Lin.) della fauna Europea e regioni finitime.*” A supplement to this work was published in 1890 in *Il Naturalista Siciliano* 9 [1889–90]: 205–215.

A modify Latin version of this work was issued under the title “*Mylabridum seu Bruchidum (Lin. Schön. All.) europeae et finitimarum regionum Faunae recensitio*” in *Deutsche Entomologische Zeitschrift* **30**: 385–416 [DP: December 1886] and **31**: 33–80 [DP: June 1887], 449–494 [DP: November 1887].

The keys, adjusted to the French fauna, were translated in French and published in 1888 by Abeille de Perrin under the title “*Tableau synoptique des bruchides et urodonides français d’après M. Fl. Baudi de Selve*” in Revue d’Entomologie 7: 77–90.

**1889**. *Catalogo dei coleotteri del Piemonte.* Camilla e Bertolero, Torino. 226 pp. (8vo) [DP: 1889 (title page), 28 February 1890 (*Monit Zool Ital*)] GB, ARC

This work was also issued in *Annali della R. Accademia d’Agricoltura di Torino* 32 [1889]: 51–274 [DP: 1890 (title page), 15 May 1890 (*Bibl Ital-2*)]. A few new taxa are described in this work.

#### Beauregard, Henri Emmanuel (Le Havre, Seine-Maritime, France: 6 December 1851 – 25 March 1900: Grasse, Alpes-Maritimes, France). French pharmacist and physician; lecturer at *École de pharmacie* of Paris early in his adulthood and later in his life; assistant of comparative anatomy at the Muséum National d’Histoire Naturelle in Paris in 1883 and received his tenure two years later; worked mainly on sperm whales and vesicant beetles. References. Coutière (1900); Petit (1900); Jaussaud (2004: 60–61).

**1890**. *Les insectes vésicants. Avec 34 planches en lithographie hors texte et 44 figures dans le texte.* Félix Alcan, Paris. xv + 544 pp. + 19 pls. (8vo) [DP: 1890 (title page; *introduction* dated 20 October 1889), 15 February 1890 (*Rev Gen Sci*), February 1890 (*Nat Nov*), 12 March 1890 (*Soc Ent Fr*), 20 February 1892 (*Bibl Fr*)] GB, GAL

#### Bechstein, Johann Matthäus (Waltershausen, Thuringia, Germany: 11 July 1757 – 23 February 1822: Dreissigacker, Thuringia, Germany). German naturalist, forester and wildlife conservationist, well-known ornithologist; studied theology at the University of Jena; taught science and mathematics at a school for boys in Schnepfenthal (1784–1794); opened a training school of forestry at Waltershausen in 1795; named director of the forestry school in Dreissigacker in 1801. References. Bechstein (1855, P); Ratzeburg (1874: 30–35); Carus (1875b); Hildebrandt (1933, P); Evenhuis (1997a: 72, P); Pfauch (1998); Adler (2007: 33–34, P).

**1794**. *Kurzgefasste gemeinnützige Naturgeschichte des In- und Auslandes für Schulen und häuslichen Unterricht. Ersten Bandes zweyte Abtheilung. Fische, Insecten und Würmer. Mit einer Kupfertafel.* Siegfried Leberecht Crusius, Leipzig. viii + pp. 613–1352 + 1 pl. (8vo) [DP: 1794 (title page; *Vorrede* dated 21 March 1794), 16 July 1794 (*Intell Allg Lit Ztg*), 23 August 1794 (*Gött Anz*)] BHL, GB, MDZ

Another title page reads “*Kurzgefasste gemeinnützige Naturgeschichte des In- und Auslandes. Ein Lehrbuch zum Unterricht und Hülfsmittel zum Gebrauch bey andern Wissenschaften. Mit Kupfern.*”

The entire work was issued in two *Bande*, each consisting of two *Abtheilungen*, 1792–1797. The Coleoptera are on pages 805–894 of the second *Abtheilung* of the first *Band*.

**1804–1805**. [Bechstein, J.M. and Scharfenberg, G.L.] *Vollständige Naturgeschichte der schädlichen Forstinsekten. Ein Handbuch für Forstmänner, Cameralisten und Oekonomen.* Carl Friedrich Knoch Richter, Leipzig. (4to) BHL, SDL

*Erster Theil. Mit drey illuminirten Kupfertafeln.* viii + 292 pp. + 3 pls. [DP: 1804 (title page; *Vorbericht* dated 10 July 1803), 22 April 1804^14^, 13 September 1804 (*Dresdner Anz*), July–December 1804 (*Verz Bücher Landkarten*), 18 January 1805 (*Schweizer-Bote*), 2 March 1805 (*Intell Allg Lit Ztg*)]. Gaedike and Smetana (1978: 341) and Evenhuis (1997a: 73) listed the publication date of this part as 1803. I have found no indication to corroborate this date. Hagen (1862: 36), Horn and Schenkling (1928a: 55), Sherborn (1922a: xxii) and Nissen (1969: 36) dated this part 1804.

*Zweyter Theil. Mit illuminirten Kupfertafeln*. Pp. 295–602 + pls 4–9. [DP: 1805 (title page), January–June 1805 (*Verz Bücher Landkarten*)]. Evenhuis (1997a : 73) dated this part 1804 but the title page on the copy in BHL is dated 1805. I have seen an advertisement in the 2 March 1805 issue of *Intelligenzblatt der Allgemeinen Literatur-Zeitung* stating that the book will be presented at the Leipzig Easter Book Fairs, which was held in 1805 on 5 May (Evenhuis 2014a: 4).

*Dritter Theil. Mit illuminirten Kupfertafeln*. Pp. 605–1042 + [4] pp. + pls 10–13. [DP: 1805 (title page), January–June 1805 (*Verz Bücher Landkarten*), 24 August 1805 (*Intell Allg Lit Ztg*), 31 August 1805 (*Intell Ztg Welt*), 24 October 1805 (*Dresdner Anz*)]. The half-title page reads “*Vollständige Naturgeschichte der schädlichen Forstinsekten. Dritte und letzte Abtheilung. Nebst dem Anhang von den nützlichen Forstinsekten.*”

This entire work was published in three *Theilen*. The Coleoptera are in the first (pp. 55–243) and third (pp. 880–888, 895–927) parts. Two new species, *Bostrichus pinastri* Bechstein (p. 93) and *Bostrichus fraxini* Bechstein (p. 107), are described in the first part and one, *Bostrichus mali* Bechstein (p. 882) in the third one. A title page, dated 1805, was issued later which reads “*Vollständige Naturgeschichte der schädlichen Forstinsekten, nebst einem Nachtrag der schonenswerthen Insekten, welche die schädlichen vertilgen helfen. Ein Handbuch für Forstmänner, Cameralisten und Oekonomen. In drey Theilen. Mit xiii fein illuminirten Quartkupfern von J. Sturm gezeichnet und gestochen.*”

**1818**. *Forstinsectologie oder Naturgeschichte der für den Wald schädlichen und nützlichen Insecten nebst Einleitung in die Insectenkunde überhaupt, für angehende und ausübende Forstmänner und Cameralisten. Mit vier illuminirten Kupfertafeln.* Hennings, Gotha. xii + 558 + [2] pp. + 4 pls. (8vo) [DP: 1818 (title page; *Vorbericht* dated 1 April 1817), 20 July 1818 (*Oppos-Blatt*), August 1818 (*Allg Lit Ztg*), July–December 1818 (*Verz Bücher Landkarten*)] BHL, GB

An additional title page reads “*Die Forst- und Jagdwissenschaft nach allen ihren Theilen für angehende und ausübende Forstmänner und Jäger. Vierter Theil. Forstschutz. Zweyter Band. Beschreibung der schädlichen Forstinsecten nebst ihren Verhütungs- und Vertilgungsmitteln*.” A second edition was published in two volumes, the first one issued in 1829 by Daniel Ernst Müller and the second one in 1835 by Anton F.A. Desberger [*q.v.*].

The Coleoptera are on pages 69–93, 167–250 and 469–482. One new species of beetle, *Bostrichus abietiperda* (p. 74), is described in this work.

#### Beck [Beck von Mannagetta und Lerchenau], Günther (Pressburg [currently Bratislava], Slovakia: 25 August 1856 – 23 June 1931: Prague, Czech Republic). Austrian botanist; professor at the University of Vienna (1894–1899); later, professor of systematic botany at the German Charles University in Prague (1899–1921). Reference. Frahm and Eggers (2001: 32).

**1886**. [Beck, G. and Ganglbauer, L.] Coleoptera, Käfer. Pp. 606–632 *in*: *Hernstein in Niederösterreich. Sein Gutsgebiet und das Land im weiteren Umkreise. Mit Unterstützung seiner kaiserlichen Hoheit des durchlauchtigsten Herrn Erzherzogs Leopold herausgegeben von M.A. Becker. I. Band. Die geologischen Verhältnisse, Flora und Fauna.* Alfred Hölder, Wien. xii + 711 pp. + 11 pls. (8vo) [DP: 1886 (title page; *Vortwort* dated 24 November 1885), March 1886 (*Nat Nov*), 31 May 1886 (*Zool Anz*), January–June 1886 (*Bibliot Hist-Nat*)] GB

An additional title page reads “*I. Die geologischen Verhältnisse. Bearbeitet von Dr. Alexander Bittner. II. Flora des Gebietes. Bearbeitet von Dr. Günther Beck. III. Fauna des Gebietes. Unter Mitwirkung von D^r.^ E. Becher, D^r.^ F. Brauer, L. Ganglbauer, Fr. Kohl, C. Koelbel, D^r.^ R. Latzel, D^r.^ F. und P. Loew, J. Mann, D^r.^ G. Mayr, J. Mik und A. Rogenhofer zusammengestellt von D^r.^ Günther Beck. Mit 5 Karten, 11 Tafeln und 37 Illustrationen im Texte.*” The third part was also issued separately the same year and recorded on July 1886 (*Nat Nov*).

The entire work consists of three volumes, 1886–1888. Beck and Ganglbauer’s contribution consists of a list of Coleoptera from the region of Hernstein in Lower Austria with short annotations for some.

#### Beck, Joseph Maximilian (Augsburg, Bavaria, Germany: 11 February 1775 – 22 July 1826: Zusmarshausen, Bavaria, Germany). State judge (“Landrichter”) in Zusmarshausen. Reference. Fischer (1970: 131–136).

**1817**. *Beiträge zur baierischen Insektenfaune, oder Beschreibung und Abbildung neuentdeckter Käfer, mit angehängtem Namensverzeichnisse der Eleuteraten des Landgerichtbezirks Zusmeshausen.* J. Wolff, Augsburg. [4] + 7–45 + [2 (Ankündigung)] pp. + 7 pls. (8vo) [DP: 1817 (title page; *Ankündigung* dated January 1817), 25 March 1817 (*Allg Ztg*), 7 May 1817 (*Beil Allg Ztg*)] MCZ, GB

This work was published anonymously. Percheron (1837: 20), Hagen (1862: 37) and Horn and Schenkling (1928a: 55) credited the book to “L. von Beck” but it was authored by Joseph Maximilian [Max] Beck (Fischer 1970). It includes the descriptions of 40 new species of beetles (pp. 7–26) and a catalogue of the beetles of Zusmeshausen in Bavaria, Germany (pp. 27–45).

#### Beckmann, Johann (Hoya, Lower Saxony, Germany: 4 June 1739 – 3 February 1811: Göttingen, Lower Saxony, Germany). German scientist; professor of physics and natural history at a Lutheran Gymnasium in St. Petersburg (1763–1764); later, professor of philosophy and economics at the University of Göttingen. References. Hansen (1811); Karmarsch (1875); Bayerl and Beckmann (1999, P).

**1767**. *Anfangsgründe der Naturgeschichte.* Georg Ludewig Förster, Göttingen und Bremen. [30] + 302 pp. (8vo) [DP: 1767 (title page; *Vorrede* dated 3 September 1766), 23 February 1767 (*Hall Ztg*), 23 March 1767 (*Jena Ztg*)] GB

The Coleoptera are on pages 89–99. A second edition was issued in 1777 in Frankfurt and Leipzig (*n.v.*); a reprint of the second edition appeared in 1785 [GB]. Hagen (1862: 37) mentioned that a new, improved edition by S. Scholz was published in Breslau, 1813 (*n.v.*).

**1772**. *Caroli a Linné Systema naturae ex editione duodecima in epitomen redactum et praelectionibus academicis accommodatum. Tomus I. Regnum animale.* Vandenhoeck, Gottingae. [4] + 240 + [10 (Index)] pp. (8vo) [DP: 1772 (title page; *praefatio* dated 28 April 1772), 18 September 1772 (*Jena Ztg*), 21 September 1772 (*Gött Anz*), 26 December 1772 (*Erlang Anmer Nachr*)] GB, BHL

This is a textual abridged version of the first tome of Linnaeus’ 12th edition of his *Systema Naturae*. The Coleoptera are on pages 132–146. There are no new species in this work (Sherborn 1902: xiii). The entire work consists of two volumes (*Regnum animale* and *Regnum vegetabile*) issued in 1772.

#### Bedel, Louis Ernest Marie (Nantes, Loire-Atlantique, France: 16 May 1849 – 26 January 1922: Paris, France). French coleopterist of independent means; made several collecting expeditions in France and northern Africa; his large collection was bequeathed to the Muséum National d’Histoire Naturelle in Paris except for some specimens (doubles) which were broken up by drawing lots among members of the Entomological Society of France. References. Sainte-Claire Deville (1922, P); Gouillard (2004: 94–95, P); Cambefort (2006: 121); Aguilar (2012, P).

**1879–1886**. *Faune des coléoptères du Bassin de la Seine.* Société Entomologique de France, Paris. (8vo) CNC

*Première partie. Tome 1er avec planche.* xxiv + 359 + [1] pp. + 1 pl. This volume was issued in 11 parts, 1879–1881, as follows: **1**: (pp. 1–32) 25 June 1879; **2**: (pp. 33–80) 27 August 1879; **3**: (pp. 81–128) 12 November 1879; **4**: (pp. 129–160) 21 March 1880; **5**: (pp. 161–192) 9 June 1880; **6**: (pp. 193–224) 25 August 1880; **7**: (pp. 225–256) 8 December 1880; **8**: (pp. 257–288) 13 April 1881; **9**: (pp. 289– 304) 22 July 1881; **10**: (pp. 305–336) 12 October 1881; **11**: (pp. 337–359 + [1] + i–xxiv) 28 December 1881. The preface is dated November 1881 (p. vii). On page xxiii, Bedel lists the genera and species described in the volume and gives, for the first time, the type species of his new genus-group taxa as follows: *Bembidion* subg. *Bracteon* (type: *Bracteon littorale* Ol.), *Orescius* (type: *Carabus Hoffmannseggi* Pz), *Crenitis* (type: *Hydr. punctatostriatus* Letz.), and *Cymbiodyta* (type: *Hydr. marginellus* Fabr.).

*Tome V.* 423 pp. This volume was issued in 16 parts, 1889–1901, as follows: (pp. 1–32) 11 September 1889; (pp. 33–64) 31 October 1889; (pp. 65–104) 22 January 1890; (pp. 105–120) 22 September 1891 (pp. 121–136) 23 December 1891; (pp. 137–152) 12 October 1892; (pp. 153–160) 28 December 1892; (pp. 161–180) 28 April 1897; (pp. 181–212) 26 October 1898; (pp. 213–228) 28 December 1898; (pp. 229–260) 10 May 1899; (pp. 261–276) 12 July 1899; (pp. 277–308) 13 June 1900; (pp. 309–340) 13 March 1901; (pp. 341–356) 25 June 1901; (pp. 357–423) 23 October 1901. This volume treats the Cerambycidae and Chrysomelidae.

*Tome VI.* 442 + [2 (Errata, Explication de la planche)] + 1 pl. This volume was issued in 20 parts, 1882–1888, as follows: (pp. 1–16) 27 December 1882; (pp. 17–32) 30 May 1883; (pp. 33–48) 8 August 1883; (pp. 49–64) 31 October 1883; (pp. 65–80) 31 January 1884; (pp. 81–96) 28 May 1884; (97–112) 31 July 1884; (113–128) 8 October 1884; (pp. 129–144) 24 December 1884; (pp. 145–160) 13 May 1885; (pp. 161–176) 15 July 1885; (pp. 177–200) 28 December 1885; (pp. 201–216) 15 July 1886; (pp. 217–248) 15 October 1886; (pp. 249–280) 30 December 1886; (pp. 281–312) 9 March 1887; (pp. 313–328) 25 May 1887; (pp. 329–360) 15 August 1887; (pp. 361–384) 28 December 1887; (pp. 385–442 + [2]) 11 April 1888. This volume deals with the Curculionidae Rhynchophora.

This work was issued as part of the *Annales de la Société Entomologique de France*, except for pages i–xxiv of the first volume, with special pagination. The dates of publication are those of the issues of the *Annales* where the corresponding pages were included. The following volumes were published: 1, 2, 4, 5, 6, 6*bis.* Volume 2 on Staphylinoidea was authored by J. Sainte-Claire Deville, 1906–1910. Volume 4 dealing with Scarabaeidae, Buprestidae, and Elateridae, 1911–1930, was authored by Bedel. Volume 6*bis* is a supplement to the Curculionidae Rhynchophora, 1924, by Sainte-Claire Deville.

**1895–1925**. *Catalogue raisonné des coléoptères du nord de l’Afrique (Maroc, Algérie, Tunisie et Tripolitaine) avec notes sur la faune des îles Canaries et de Madère.* Société Entomologique de France, Paris. 402 pp. (8vo) GB, BHL

The entire work appeared from 1895 to 1914 and in 1925; the first 320 pages were published in connection with *L’Abeille*, with separate title-page and pagination. The dates listed at the bottom of first page of each sheet are: (pp. 1–16) March 1895; (pp. 17–32) June 1895; (pp. 33–48) September 1895; (pp. 49–68) March 1896; (pp. 69–84) October 1896; (pp. 85–100) February 1897; (pp. 101–108) March 1897; (pp. 109–124) August 1897; (pp. 125–136) March 1898; (pp. 137–152) February 1899; (pp. 153–160) March 1899; (pp. 161–168) June 1899; (pp. 169–200) October 1899; (pp. 201–208) May 1900; (pp. 209–220) November 1902; (pp. 221–228) May 1904; (pp. 229–252) August 1905; (pp. 253–264) 1906; (pp. 265–280) 1907; (pp. 281–296) 1913; (pp. 297–320) 1914; (pp. 321–402) 1925.

The last part (pp. 321–402) was published posthumously and contains additions, placed in square brackets, by Paul de Peyerimhoff.

**1900**. *Exploration scientifique de la Tunisie. Catalogue raisonné des coléoptères de Tunisie comprenant tous les documents déjà publiés ou obligeamment communiqués et spécialement le résultat des voyages de MM. Valéry Mayet et Maurice Sedillot membres de la Mission de l’Exploration Scientifique de la Tunisie. Première partie. Cicindelidae–Staphylinidae.* Imprimerie Nationale, Paris. xiv + 130 pp. (8vo) [DP: 1900 (title page), 27 February 1901 (*Soc Ent Fr*), 9 March 1901 (*Bibl Fr*), April 1901 (*Rev Tunis*), Mai 1901 (*Nat Nov*)] GB

The half-title page reads “*Exploration scientifique de la Tunisie publiée sous les auspices du Ministère de l’Instruction Publique. Zoologie.–Coléoptères.*” Two new family-group names are proposed in this work.

#### Belon, Paul Marie Joseph (Angers, Maine-et-Loire, France: 30 January 1839 – 27 December 1912: Rijckholt [currently Eijsden-Margraten], Limburg, Netherlands). French Dominican monk, professor at the *Institut catholique de Lyon* for 28 years and coleopterist in his spare time with a special interest for small groups, like the latridiids; expelled from France in 1902 following the law on religious congregations, he left his cerambycid collection to Albert Argod-Vallon and his latridiid collection to Maurice Pic [*q.v.*]. References. Argod-Vallon (1914, P); Gouillard (2004: 50); Cambefort (2006: 123).

**1881–1884**. *Histoire naturelle des coléoptères de France par E. Mulsant. Famille des lathridiens.* A. Storck [vol. 1] / Pitrat ainé [vol. 2], Lyon. (8vo) CMLE, GAL, GB

*1re partie.* 209 pp. [DP: 1881 (title page), 27 July 1881 (*Soc Ent Fr*), 3 September 1881 (*Bibl Fr*), September 1881 (*Nat Nov*), November 1881 (*Polybiblion*), 17 December 1881 (*Zool Anz*)].

*Deuxième partie.* 152 pp. [DP: 1884 (title page), 25 February 1885 (*Soc Ent Fr*), 18 April 1885 (*Bibl Fr*), May 1885 (*Nat Nov*), 10 June 1885 (*Wien Ent Ztg* 4: 159), June 1885 (*Polybiblion*), January–June 1885 (*Bibliot Hist-Nat*), 27 July 1885 (*Zool Anz*)].

This work was also issued in the *Annales de la Société Linnéenne de Lyon* (Nouvelle Série) 26 [1879]: 157–365 [DP: 1879 (title page), 13 August 1881 (*Bibl Fr*), 24 August 1881 (*Soc Ent Fr*), September 1881 (*Nat Nov*)] and 31 [1884]: 61–212 [DP: 1885 (title page), 31 October 1885 (*Bibl Fr*), December 1885 (*Nat Nov*)].

**1889**. *Supplément à la monographie des lathridiens de France.* Pitrat ainé, Lyon. 17 pp. (8vo) [DP: 1889 (title page), 20 July 1889 (*Bibl Fr*), July 1889 (*Nat Nov*), 26 February 1890 (*Soc Ent Fr*)] NAL

This work was also issued in the *Annales de la Société Linnéenne de Lyon* (Nouvelle Série) 35 [1888]: 75–91 [DP: 1889 (title page), 30 November 1889 (*Bibl Fr*), December 1889 (*Nat Nov*), 8 October 1890 (*Soc Ent Fr*)].

**1899**. *Récapitulation des Lathridiidae de l’Amérique méridionale.* Lyon. 56 pp. (8vo) [DP: 13 December 1899 (*Soc Ent Fr*), February 1900 (*Nat Nov*)] (*n.v.*).

This work was also published in *Annales de la Société Linnéenne de Lyon* (Nouvelle Série) 46 [1899]: 137–192 [DP: 1900 (title page), 20 January 1900 (*Bibl Fr*), April 1900 (*Nat Nov*), 23 May 1900 (*Soc Ent Fr*)].

#### Bennet, Jan [Jean] Arnold [Aarnoud] (Breda, North Brabant, Netherlands: 20 December 1758 – 3 September 1828: Leiden, South Holland, Netherlands). Dutch physician in Amsterdam and later in Leiden; appointed professor of rural economy at the University of Leiden in 1815. Reference. Van der Aa (1854: 317–318).

**1825**. [Bennet, J.A. and Olivier, G. van] *Natuurkundige verhandelingen van de Hollandsche Maatschappij der Wetenschappen te Haarlem. Veertiende deel.* A. Loosjes, Te Haarlem. 521 pp. [DP: 1825 (title page)] GB

The author names are not listed on the title page. They appeared on the second page which reads also “*Verhandeling ter beantwoording der vrage: bij welke verlangd wordt “eene naauwkeurige naamlijst der visschen en insecten de natuurlijke en niet slechts van elders verdwaalde inwoners dezer landen zijn, of die niet wijd van onze stranden in zee wonen, met bijvoeging van derzelver verschillende namen, in verschillende Nederlandsche gewesten, en de geslachts- en soortskenmerken, 200 veel doenlijk volgens het Linneaansch stelsel, doch ook met aanhaling van latere stelsels zeer kort gesteld, en met aanwijzing van één of meer der beste afbeeldingen van elk dier.*” The first page (p. [9]) following the preface is entitled “*Naamlijst van Nederlandsche insecten*.” The book treats the insects of the Netherlands; the Coleoptera are on pages 9–155.

The entire series was issued in 24 volumes, 1799–1844; Bennet and Gerrit van Olivier’s [1759–1827] work forms the fourteenth volume. The first six volumes of the series were issued under different titles. For example, the title of the first three volumes is “*Natuurkundige verhandelingen van de Bataafsche Maatschappy der Wetenschappen te Haarlem.*” A second, in 25 volumes 1841–1868, and third editions, incomplete in nine volumes 1872–1922, were published.

#### Berg, Friedrich Wilhelm Karl [Carlos] (Tukums, Latvia: 21 March 1843 – 19 January 1902: Buenos Aires, Argentina). Argentine naturalist of Baltic origin; curator of the entomology department at the Museum of Natural History in Riga and then at the Riga Technical University; moved to Argentina in 1873 at the invitation of Karl Hermann Burmeister; professor of natural history at the National College of Buenos Aires; director of the Natural History Museum in Montevideo (1890–1892); later, director of the National Museum in Buenos Aires. References. Gallardo (1902, P); Adler (1989: 64).

**1881**. Insectos. Pp. 77–115 *in*: *Informe oficial de la comision científica agregada al Estado Mayor General de la Expedicion al Rio Negro (Patagonia) realizada en los meses de Abril, Mayo y Junio de 1879, bajo las órdenes del General D. Julio A. Roca (con 16 láminas). Entrega I. – Zoología (con 4 láminas).* Ostwald y Martinez, Buenos Aires. xxiv + 168 + [1 (Errores tipográficos)] pp. + 4 pls. (4to) [DP: 1881 (title page), 1 November 1881 (*Anales Cir Méd Argent* 5: 171), 30 November 1881 (*Soc Rur Argent* [*Anales* 15: 339])] McG, GB, BHL

The report of the Commission was published in three parts (*entregas*), 1881–1882. The Coleoptera are on pages 94–111; one new genus and several species-group taxa are made available for the first time in this publication through descriptions and/or illustrations (plate 2).

#### Berge, Karl Friedrich Wilhelm (Stuttgart, Baden-Württemberg, Germany: 11 December 1811 – 19 September 1883: Stuttgart, Germany). German naturalist who worked mainly on birds and insects; involved in construction as an assistant. References. Calmbach (1921); Rebel (1923: 61).

**1843–1844**. *Käferbuch. Allgemeine und specielle Naturgeschichte der Käfer, mit vorzüglicher Rücksicht auf die europäischen Gattungen. Nebst der Anweisung, sie zu sammeln, zuzubereiten und aufzubewahren. Mit 1315 colorirten Abbildungen.* Hoffmann, Stuttgart. 268 pp. + 36 pls. (4to) MCZ

This work was issued in ten *Lieferungen* (Engelmann 1846: 471): **1**: (pp. 1–24 + 4 pls) 12 October 1843 (*Allg Bibl Deutsch*), 25 October 1843 (*Allg Anz*), 28 October 1843 (*Lit Ztg*), October 1843 (*Allg Lit Ztg*), 22 December 1843 (*Leip Reper*), October–December 1843 (*Foreign Quart Rev*), July–December 1843 (*Verz Bücher Landkarten*); **2**: (pp. 25–48 + pls 5–8) 14 December 1843 (*Allg Bibl Deutsch*), 31 December 1843 (*Intell Serapeum*), December 1843 (*Allg Lit Ztg*); **3–10**: (pp. 49–268 + pls 9–36) 18 January 1844 (*Allg Bibl Deutsch*), 31 January 1844 (*Lit Ztg*), 9 February 1844 (*Leip Reper*), 29 June 1844 (*Allg Anz*), January–June 1844 (*Verz Bücher Landkarten*). The title page is dated 1844.

**1847**. *Taschenbuch für Käfer- und Schmetterlings-Sammler, oder praktische Anweisung, Käfer und Schmetterlinge zu sammeln, zu erziehen, zuzubereiten und aufzubewahren. Nebst einer namentlichen Aufzählung der europäischen Gattungen in systematischer Ordnung. Mit instruktiven Abbildungen der Fanginstrumente und sonstiger Geräthschaften.* Hoffmann, Stuttgart. ix + 360 pp. + 2 pls. (8vo) [DP: 1847 (title page; *Vorwort* dated April 1847), 11 May 1847 (*Allg Anz*), 13 May 1847 (*Allg Bibl Deutsch*), 11 June 1847 (*Leip Reper*)] GB, MDZ

The work comprises a systematic list of the European Coleoptera including a list of non-european genera with number of species (pp. 65–277).

#### Bergsträsser, Johann Andreas Benignus (Idstein, Hesse, Germany: 21 December 1732 – 24 December 1812: Hannover, Lower Saxony, Germany). German scholar and writer; professor and rector of the Evangelical Lutheran Lyceum in Hannover. References. Strieder (1781: 361–370); Rose (1848a: 122); Larousse (1865: 586).

**1777–1778**. *Nomenclatur und Beschreibung der Insecten in der Grafschaft Hanau-Münzenberg wie auch der Wetterau und der angränzenden Nachbarschaft dies und jenseits des Mains mit erleuchteten Kupfern. Erster Jahrgang.* Hanau. [6] + 88 pp. + 14 pls. (4to) CAKL, GDZ, GB

This work was issued in parts. I have seen the following notices: (pp. 1–48 + pls 1–6) 29 August 1777 (*Jena Ztg*), 17 September 1777 (*Goth Ztg*); (pls 7–12) 14 November 1777 (*Jena Ztg*); (pp. 49–72 + pls 13–18^15^) 5 January 1778 (*Jena Ztg*). By 21 August 1778 (*Jena Ztg*) the first *Jahrgang* was completed. The title page is dated 1778 and the *Vorrede* 5 February 1778.

Bergsträsser’s work consists of descriptions of the insects in the county of Hanau-Münzenberg in old Germany as well as the Wetterau and the surrounding areas on both sides of the Main. A few new species of beetles, such as *Dytiscus bistriatus* (p. 42) and *Dytiscus frischii* (p. 43), are described. The other three *Jahrgänge* pertain to Lepidoptera.

#### Berkenhout, John (Leeds, West Yorkshire, United Kingdom: 8 July 1726 – 3 April 1791: Besselsleigh, Oxfordshire, United Kingdom). British physician and naturalist; studied in Germany; joined the Prussian army and rose to the grade of captain; later, studied medicine in Edinburgh and Leiden and practiced in Isleworth. References. Chalmers (1812: 70–73); Rose (1848a: 132–133); Anonymous (1853: 184–185); Lisney (1960: 192–196, P).

**1769**. *Outlines of the natural history of Great Britain and Ireland. Containing a systematic arrangement and concise description of all the animals, vegetables, and fossiles which have hitherto been discovered in these kingdoms. In three volumes. Vol. I. Comprehending the animal kingdom.* P. Elmsly, London. xiii + 233 pp. (8vo) [DP: 1769 (title page), March 1769 (*Gent Mag*), April 1769 (*Lond Mag*), May 1769 (*Monthly Rev*)] BHL, GDZ, GB

The entire work consists of three volumes, 1769–1772. The beetles are on pages 85–113 of the first volume. Second and third editions were published in 1789 [*q.v.*] and 1795.

**1789**. *Synopsis of the natural history of Great-Britain and Ireland. Containing a systematic arrangement and concise description of all the animals, vegetables, and fossils, which have hitherto been discovered in these kingdoms. Being a second edition of the Outlines, &c. Corrected and considerably enlarged. Vol. I. Comprehending the animal and fossil kingdoms.* T. Cadell, London. xii + 13–334 + [1 (Errata)] pp. (8vo) [DP: 1789 (title page), March 1789 (*Analyt Rev*), April 1789 (*Crit Rev*), May 1789 (*Town Count Mag*), July 1789 (*Monthly Rev*; *Esprit J*), 26 August 1789 (*Intell Allg Lit Ztg*)] GDZ

Berkenhout’s work was published in two volumes issued simultaneously. The Coleoptera are on pages 86–110 of the first volume. A third edition was published in 1795 [GB] with no change in pagination to the first volume of this edition.

#### Bernard, Pons-Joseph (Trans-en-Provence, Var, France: 14 July 1748 – 29 July 1816: Trans-en-Provence, France). French mathematician, naturalist and geologist; professor of mathematics and philosophy at the Oratoriens; later, assistant director of the Marseille Observatory. References. Weiss (1835); Le Bas (1840: 431).

**1788**. *Mémoires pour servir a l’histoire naturelle de la Provence. Tome II.* Didot Fils aîné, Paris. 559 + [4] + 7 [Supplément] + [1 (Errata)] + 3 pls. (12mo) [DP: 1788 (title page), 2 December 1788 (*J Fr*), 20 December 1788 (*Merc Fr*)] GDZ

The entire work consists of two volumes issued simultaneously. Two well-known beetle pests are made available in the second volume, *Byrrhus taranio* (p. 270) and *Scolytus scaraboeoides* (p. 271). A preliminary version of the natural history part (without Latin names for the taxa) appeared in 1783 (Miguel A. Alonso-Zarazaga, personal communication) in the *Recueil de l’Académie des Belles Lettres, Sciences et Arts de Marseille, du 25 août 1782* (*n.v.*).

#### Bernhardt, Gustav

**1870**. *Kleines Käferbuch. Eine Anleitung zur Kenntniss der Käfer im Allgemeinen wie auch zur Anleitung und zweckmässigen Einrichtung von Käfersammlungen. Mit 72 illuminirten Abbildungen auf 6 Tafeln. Vierte verbesserte Auflage.* Otto Hendel, Halle. [2] + 140 pp. + 6 pls. (8vo) [DP: 1870 (title page; *Vorwort* dated January 1870), 3 March 1870 (*Börsen Deutsch Buch*), 10 March 1870 (*Allg Bibl Deutsch*), March 1870 (*Natur Offen*), January–June 1870 (*Bibliot Hist-Nat*; *Verz Bücher Landkarten*)] BHL

Five subsequent editions were issued between 1876 and 1888. The third edition, with a slight change in the title, was published in 1862 [SBB] and recorded on 11 December 1862 (*Allg Bibl Deutsch*). The second edition (*n.v.*) was issued in 1856 and recorded on 13 March 1856 (*Allg Bibl Deutsch*). No information was found for the original edition.

#### Berthold, Arnold Adolph (Soest, North Rhine-Westphalia, Germany: 26 February 1803 – 3 February 1861: Göttingen, Lower Saxony, Germany). German naturalist and physician, mostly known for his work on endocrinology; studied medicine at the University of Göttingen and obtained his degree at the age of 20; in the following months, visited many hospitals in Berlin and Paris; in 1825 returned to Göttingen where he practiced medicine for many years and gave courses in physiology, comparative anatomy, and zoology at the Faculty of Sciences of the University before becoming associate professor at the age of 32; obtained his tenure the following year. References. Vapereau (1858: 185–186); Larousse (1865: 616–617); Adler (2007: 69, P); Soukup and Schober (2009: 40–41).

**1827**. *Latreille’s Natürliche Familien des Thierreichs. Aus dem Französischen. Mit Anmerkungen und Zusätzen.* Landes-Industrie-Comptoirs, Weimar. x + 606 pp. (8vo) [DP: 1827 (title page; *Vorrede* dated 30 August 1826), 22 September 1827 (*Bibl Deutsch*), September 1827 (*Allg Monats Deutsch*), 8 November 1827 (*Gött Anz*), July–December 1827 (*Verz Bücher Landkarten*), January 1828 (*Not Geb Natur*)] CNC, BHL

This is a German translation of Latreille’s (1825) *Familles naturelles du règne animal* [*q.v.*] with notes and additions; it contains latinizations of most vernacular names proposed by Latreille. Authorship of these names should be credited to Berthold (Evenhuis 1997a: 86). The Coleoptera are on pages 321–404.

**1845**. *Lehrbuch der Zoologie.* Vandenhoeck und Ruprecht, Göttingen. v + [1] + 591 + [1] pp. (8vo) [DP: 1845 (title page; *Vorrede* dated 31 December 1844), 10 February 1845 (*Gött Anz*), 10 April 1845 (*Allg Bibl Deutsch*), April 1845 (*Intell Allg Lit Ztg*), 16 May 1845 (*Leip Reper*), January–June 1845 (*Verz Bücher Landkarten*)] GB

The Coleoptera are on pages 408–437.

#### Bertolini, Stefano (Civezzana, Trentino-Alto Adige, Italy: 30 June 1832 – 16 April 1905: Madrano near Pergine Valsugana, Trentino-Alto Adige, Italy). Italian court employee, who retired with the rank of Commissioner, and amateur entomologist; his collection is at the Museo delle Scienze in Trento. References. Bargagli (1905); Conci (1975: 848); Conci and Poggi (1996: 186, P); Gobbi et al. (2012, P).

**1872–1878**. *Catalogo sinonimico e topografico dei coleotteri d’Italia.* Cenniniana, Firenze. 263 pp. (8vo) USNM, GB, BHL

This work was issued in several parts as supplements to the *Bullettino della Società Entomologica Italiana* for volumes **4** (pp. 1–44) 1872; **5** (pp. 45–92) 1873; **6** (pp. 93–156) 1874; **7** (pp. 157–204) 1875; **8** (pp. 205–236) 1876; **10** (pp. 237–263) 1878. The last part (pp. 237–254) is the *Supplemento contenente le specie scoperte o descritte di recente od ommesse nel «Catalogo sinonimico e topografico dei coleotteri d’Italia.*» The *Société Entomologique de Belgique* received pages 1–12 on 1 June 1872, 45–60 on 7 June 1873, 61–76 on 6 September 1873, 77–92 on 8 November 1873. The title page is dated 1872 and the preliminaries December 1871.

#### Betta, Virginio. Italian physician born in Cles, in the province of Trento.

**1847**. *De quibusdam coleopteris agri Ticinensis. Dissertatio inauguralis quam annuentibus perillustri d. facultatis medicae directore spectabili d. decano ac clarissimis d.d. professoribus auspice Doct. Bartholomaeo Panizza anatom. Human. P.o. coronae ferreae equite ad medicinae lauream rite assequendam in c.r. Universitate Ticinensi Mense Martii 1847 publicae disquisitioni offerebat una cum thesibus defendendis Betta Virginius Tirolensis e Cles.* Fusi et Socii, Ticini Regii [= Pavia]. 33 pp. (8vo) [DP: March 1847 (title page)] GB

This thesis treats some of the Coleoptera found in the fields of Pavia. The new species described are: *Clivina binotata* (p. 11), *Clivina similata* (p. 12), *Bledius marginalis* (p. 17), *Bledius rufus* (p. 18), *Agrilus comolli* (p. 19), *Agrilus auricollis* (p. 20), *Malachius glabrellus* (p. 23), *Scydmaenus ullrichi* (p. 24), *Cercyon elegans* (p. 25), *Lagria villosula* (p. 27), *Ripiphorus maurus* (p. 28), *Ripiphorus suturalis* (p. 28), *Sitaris flavescens* (p. 29), *Sitaris fuliginosa* (p. 30), *Tropideres pictus* (p. 30), *Apion tonsuratum* (p. 31), *Gymnetron vittatus* (p. 32), *Agathidium festivum* (p. 33). None of these species were recorded by Sherborn in his *Index Animalium*.

#### Bielz, Eduard [Ede] Albert (Sibiu, Romania: 4 February 1827 – 26 May 1898: Sibiu, Romania). Transylvanian public servant, statistician and naturalist; moved in 1869 to Budapest to work for the Ministry of Commerce in the State Statistical Bureau; sent to Sibiu in 1873 as school inspector; retired in 1878 after an eye injury which left him gradually blind; his collection of beetles is at the Museum of Natural History in Sibiu. References. Anonymous (1899, P); Capesius (1899, 1902); Csiki (1900, P).

**1857**. *Handbuch der Landeskunde Siebenbürgens: eine physikalisch-statistisch-topographische Beschreibung dieses Landes.* S. Filtsch, Hermannstadt. viii + 613 + [1] pp. (8vo) [DP: 1857 (title page; *Vorwort* dated September 1856), 30 April 1857 (*Allg Bibl Deutsch*), 15 June 1857 (*Intell Serapeum*), July 1857 (*Allg Bibl*)] GB

The work contains a section on the fauna of Transylvania; a list of beetle species (pp. 118–123), some with short annotations, is part of it.

#### Billberg, Gustaf Johan (Karlskrona, Blekinge, Sweden: 14 June 1772 – 26 November 1844: Stockholm, Sweden). Swedish lawyer, zoologist and botanist; councilor to the Swedish royal court (1812–1836); his collection burned in 1822 and he subsequently bought the collection of Forsstroem (particularly strong with insects from the West Indies) and built a second collection which was deposited at the Natural History Museum in London via John George Children. References. Anonymous (1859a); Hofberg (1876a: 92–93).

**1813**. *Monographia mylabridum.* Caroli Delén, Holmiae [= Stockholm]. 74 + [6] pp. + 7 pls. (8vo) [DP: 1813 (p. [78])] MCZ

Percheron (1837: 30) and Engelmann (1846: 472) mentioned another edition, issued in 1818, but according to Sherborn (1922a: xxiv) the edition does not exist. Agassiz (1848: 290) listed the publication date of this booklet as 1812.

**1820**. *Enumeratio insectorum in museo Gust. Joh. Billberg.* Gadelianis, [Stockholm]. [2] + 138 pp. (4to) [DP: 1820 (title page)] MCZ, BHL

This is a list of arthropods in Billberg’s collection. A number of new family-group names of Coleoptera and several new genus-group names are made available by the inclusion of one or more available specific names under them.

#### Blackburn, Thomas (Islington near London, United Kingdom: 16 March 1844 – 28 May 1912: Woodville near Adelaide, South Australia, Australia). British priest and entomologist; ordained deacon in 1869; at the age of 32, sent to Honolulu and for the next six years collected insects on the Hawaiian islands during his spare time; transferred in 1882 to Port Lincoln in South Australia and four years later to Adelaide where he remained for the rest of his life; despite his heavy load of clerical work, collected and published extensively during his 30 years in Australia; sold a “first selection” of his collection to the British Museum (Natural History) in London in 1888 including 334 types; most of the remainder of his collection was given to the British Museum in 1910 but some of his types are located in the South Australian Museum. References. Lea (1912, P); Zimmerman (1993: 471, P); Smetana and Herman (2001: 45–46, P).

**1896**. Coleoptera (exclusive of the Carabidae). Pp. 254–308 *in*: *Report on the work of the Horn scientific expedition to central Australia. Part II. – Zoology. Edited by Baldwin Spencer.* Dulau and Co., London [&] Melville, Mullen and Slade, Melbourne. iv + 431 pp. + 29 pls. (4to) [DP: February 1896 (title page), June 1896 (*Nat Nov*), 16 July 1896 (*Nature*), 5 September 1896 (*Athenaeum*)] McG, GB

This expedition, fitted by William Austin Horn, was set in 1894 to investigate the part of Australia between Oodnadatta and Macdonnell Ranges (Musgrave 1932: 155). The entire report was published in four parts. The Carabidae of the expedition were treated by Sloane [*q.v.*].

#### Blake, John Frederick (Stoke near Guildford, Surrey, United Kingdom: 3 April 1839 – 7 July 1906: London, United Kingdom). British priest and geologist; professor of mathematics and assistant chaplain at St. Peter’s School in York (1865–1873); lecturer of comparative anatomy at Charing Cross Hospital in London (1876–1880); later, professor of natural sciences at University College in Nottingham (1881–1888). References. Anonymous (1906a); Venn and Venn (1940: 288); Oldroyd (1990: 185).

**1876**. Insecta. P. 426 + pl. XVI *in*: *The Yorkshire Lias by Ralph Tate and J.F. Blake.* John van Voorst, London. viii + [1] + 475 + xii pp. + 19 pls. (8vo) [DP: 1876 (title page), 31 August 1876 (*Nature*), 2 September 1876 (*Academy*; *Spectator*; *Athenaeum*), 5 October 1876 (*Bookseller*)] GB

One new beetle fossil, *Buprestites bractoides*, is made available by an illustration.

#### Blanchard, Charles Émile (Paris, France: 6 March 1819 – 11 February 1900: Paris, France). French naturalist and educator; spent almost all his adulthood associated with the Muséum National d’Histoire Naturelle in Paris, first as a temporary assistant at the age of 14 under the direction of Jean-Victor Audouin [*q.v.*] and Henri Milne-Edwards [*q.v.*], then as adjunct assistant naturalist (1838), assistant naturalist (1841), and finally as professor of the entomology chair (1862) the year after being elected to the *Académie des Sciences*; his reluctance to new ideas brought him to take position against Darwin; progressively lost his sight from the age of 40 and became completely blind by 1890. References. Gaudry (1900); Jaussaud (2004: 81–82); Cambefort (2006: 124–126); Adler (2012: 114–115, P).

**1835–1847**. Insectes de l’Amérique méridionale. Recueillis par Alcide d’Orbigny et décrits par Emile Blanchard et Auguste Brullé. Pp: 57–222 *in*: *Voyage dans l’Amérique méridionale (le Brésil, la République orientale de l’Uruguay, la République Argentine, la Patagonie, la République du Chili, la République de Bolivie, la République du Pérou), exécuté pendant les années 1826, 1827, 1828, 1829, 1830, 1831, 1832 et 1833 par Alcide d’Orbigny. Ouvrage dédié au Roi, et publié sous les auspices de M. le Ministre de l’Instruction publique (commencé sous le ministère de M. Guizot). Tome sixième. 2.^e^ Partie: Insectes.* P. Bertrand, Paris [&] V. Levrault, Strasbourg. [4] + 222 pp. [+ 32 associated pls]. (4to–text; Folio–plates) CNC, GB, BHL

The text of this volume and the associated plates were issued in 32 livraisons from 1835 to 1847 (Evenhuis 1997b: 572) although the title page of the text volume is dated 1837–1843. The first 56 pages were authored by Gaspard Auguste Brullé [*q.v.*], and the remaining ones by Blanchard. The Coleoptera are on pages 1–214 and plates 1–25. Pages 57–222 were issued in the following livraisons: **56**: (pp. 57–72) 1841 (wrapper), 24 January 1842 (*Soc Géol Fr*), 17 June 1842 (*Soc Géog Fr*); **57**: (pp. 73–80) 1842 (wrapper), 17 June 1842 (*Soc Géog Fr*); **63**: (pp. 81–88) 1842 (wrapper), 12 May 1843 (*Soc Géog Fr*), 19 June 1843 (*Soc Géol Fr*); **68**: (pp. 89–96) 1842 (wrapper), 15 December 1843 (*Soc Géog Fr*), 8 July 1844 (*Acad Sci Fr*); **77**: (pp. 97–112) 1844 (wrapper), 20 December 1844 (*Soc Géog Fr*); **78**: (pp. 113–152) 1845 (wrapper), 2 May 1845 (*Soc Géog Fr*); **79**: (pp. 153–168) 1845 (wrapper); **80**: (pp. 169–184) 1845 (wrapper); **90**: (pp. 185–222) 1847 (wrapper), after 17 April 1847 (record date of livr. 89 in *Feuil J Lib*).

The dates of the Coleoptera plates are as follows: **Plate 1** [livr. 15]: 1835 (wrapper), 1 August 1836 (*Acad Sci Fr*), September 1836 (*J Sav*); **Plate 2** [livr. 18]: 1836 (wrapper), 7 November 1836 (*Acad Sci Fr*), 30 December 1836 (*Allg Bibl Deutsch*); **Plate 3** [livr. 22]: 1836 (wrapper), 27 February 1837 (*Acad Sci Fr*), 14 April 1837 (*Allg Bibl Deutsch*), January–June 1837 (*Verz Bücher Landkarten*); **Plate 4** [livr. 17^16^]: 1836 (wrapper), 3 October 1836 (*Acad Sci Fr*), 2 December 1836 (*Allg Bibl Deutsch*); **Plate 5** [livr. 21]: 1836 (wrapper), 16 January 1837 (*Soc Géol Fr*), 14 April 1837 (*Allg Bibl Deutsch*), January–June 1837 (*Verz Bücher Landkarten*); **Plates 6–7** [livr. 53]: 1841 (wrapper), 8 November 1841 (*Soc Géol Fr*), 14 February 1842 (*Acad Sci Fr*); **Plate 8** [livr. 54]: 1841 (wrapper), 8 November 1841 (*Soc Géol Fr*), 14 February 1842 (*Acad Sci Fr*); **Plate 9** [livr. 52]: 1841 (wrapper), 22 May 1841 (*Feuil J Lib*), 8 November 1841 (*Soc Géol Fr*), 15 November 1841 (*Acad Sci Fr*); **Plate 10** [livr. 54]: 1841 (wrapper), 8 November 1841 (*Soc Géol Fr*), 14 February 1842 (*Acad Sci Fr*); **Plate 11** [livr. 61]: 1842 (wrapper), 19 June 1843 (*Soc Géol Fr*); **Plate 12** [livr. 58]: 1842 (wrapper), 17 June 1842 (*Soc Géog Fr*); **Plate 13** [livr. 59]: 1842 (wrapper), 17 June 1842 (*Soc Géog Fr*), 18 June 1842 (*Feuil J Lib*); **Plate 14** [livr. 60]: 1842 (wrapper), 19 June 1843 (*Soc Géol Fr*); **Plate 15** [livr. ??]: ??; **Plate 16** [livr. 68]: 1842 (wrapper); **Plate 17** [livr. 69]: 1843 (wrapper), 8 July 1844 (*Acad Sci Fr*); **Plate 18** [livr. 71]: 1843 (wrapper), 8 July 1844 (*Acad Sci Fr*); **Plate 19** [livr. 60]: 1842 (wrapper), 19 June 1843 (*Soc Géol Fr*); **Plate 20** [livr. 56]: 1841 (wrapper), 24 January 1842 (*Soc Géol Fr*), 17 June 1842 (*Soc Géog Fr*); **Plate 21** [livr. 57]: 1842 (wrapper), 17 June 1842 (*Soc Géog Fr*); **Plate 22** [livr. 65]: 1842 (wrapper), 12 May 1843 (*Soc Géog Fr*), 19 June 1843 (*Soc Géol Fr*); **Plate 23** [livr. 66]: 1842 (wrapper), 12 May 1843 (*Soc Géog Fr*), 19 June 1843 (*Soc Géol Fr*); **Plate 24** [livr. 68]: 1842 (wrapper), 15 December 1843 (*Soc Géog Fr*); **Plate 25** [livr. ??]: ?? The collation is from Sherborn and Griffin (1934) who had access to a copy with original wrappers. The list of species illustrated on the plates is given in Appendix 5.

The entire expedition report was published in seven volumes of text and two volumes of plates^17^, 1835–1849, issued in 90 livraisons (Dickinson et al. 2011: 88). A facsimile edition was published by Junk, in Lochem, 1972. Graesse (1863: 35) mentioned that a new edition was published in 1852, Paris. However, although labelled “*nouvelle édition*,” it is for the most part supplementary information published in 1852 and 1853 under the title “*Voyage dans les deux Amériques, augmenté de renseignements exacts jusqu’en 1853 sur les différents états du Nouveau Monde*.” A Spanish version in four volumes was published in 1945, in Buenos Aires, without the zoology section, under the title “*Viaje a la América meridional – Brasil – República del Uruguay – República Argentina – La Patagonia – República de Chile – República de Bolivia – República del Perú – realizado de 1826 a 1833 por Alcides d’Orbigny. Prólogo de Ernesto Morales*” [OLIL].

**1842**. [*Catalogue des cétonides de la collection du Muséum d’Histoire Naturelle à Paris*]. Poussielgue, Paris. 23 pp. (8vo) [DP: November 1842 (p. 1)] YB (ph)

There is no title page on this booklet and the author is not listed. However, the work is credited to Émile Blanchard by several bibliographers, including Hagen (1862: 55), Horn and Schenkling (1928a: 86) and Sherborn (1922a: xcix). The last page reads “Extrait du Catalogue des collections Entomologiques du Museum d’hist. nat. de Paris. No. 1.”

**1842–1854**. *Voyage au Pole Sud et dans l’Océanie sur les corvettes l*’Astrolabe *et la* Zélée; *exécuté par ordre du roi pendant les années 1837–1838–1839–1840, sous le commandement de M.J. Dumont-d’Urville, Capitaine de vaisseau; publié par ordre du gouvernement, sous la direction supérieure de M. Jacquinot, Capitaine de vaisseau, commandant de la* Zélée. *Zoologie Atlas.* Paris. 140 plates. (Folio) McG

The *Zoologie Atlas* contains 140 colored plates, published in 28 livraisons between 25 March 1842 and 24 February 1854. The Coleoptera are depicted on 19 plates (numbered 1–19) representing 325 species of which 241 were considered to be new and indicated by *Nob[is].* Furthermore a number of new genera were made available from the plates. The dates of publication of these 19 plates, as discussed by Emberson (1992) (see also Clark and Crosnier 2000) unless otherwise noted, are: **Plate 1** [livr. 3]: 1 December 1842; **Plate 2** [livr. 4]: 2 February 1843; **Plate 3** [livr. 5]: 4 May 1843; **Plate 4** [livr. 7]: 26 August 1843; **Plate 5** [livr. 11]: 19 July 1844; **Plates 6–7** [livr. 12]: 20 December 1844 (*Soc Géog Fr*), 10 January 1845; **Plate 8** [livr. 21]: 5 November 1846; **Plate 9** [livr. 17]: 6 July 1846; **Plate 10** [livr. 21]: 5 November 1846; **Plate 11** [livr. 22]: 6 April 1847; **Plate 12** [livr. 21]: 5 November 1846; **Plate 13** [livr. 20]: 6 July 1846; **Plate 14** [livr. 22]: 6 April 1847; **Plate 15** [livr. 21]: 5 November 1846; **Plates 16–17** [livr. 18]: 6 July 1846; **Plate 18** [livr. 17]: 6 July 1846; **Plate 19** [livr. 20]: 6 July 1846. A list of the Coleoptera depicted on the plates is given in Appendix 2. The text was published in 1853 [*q.v.*].

The entire expedition report was divided into seven parts: *Histoire du voyage*; *Anthropologie*; *Botanique*; *Géologie, Minérologie et géographie physique*; *Hydrographie*; *Physique*; and *Zoologie*. Most of the zoological material of this expedition was collected around the Straits of Magellan, on various southern Pacific Islands, including New Zealand, in Tasmania, along the north coast of Australia and in the Indo-Malaysian archipelago from New Guinea to Singapore (Emberson 1992: 251).

**1844**. Zoologie. Insectes recueillis à l’Himalaya, par Victor Jacquemont, et décrits. Pp. 13–31 [second section] *in*: *Voyage dans l’Inde, par Victor Jacquemont, pendant les années 1828 à 1832, publié sous les auspices de M. Guizot, ministre de l’Instruction publique. Description des collections par MM. Isidore Geoffroy Saint-Hilaire. – Milne Edwards. – Émile Blanchard. Valenciennes. – Cambessedes. – J. Decaisne. Tome quatrième.* Firmin Didot Frères, Paris. iii + 90 (mammifères, oiseaux) + [2 (Index)] + 31 (crustacés, insectes) + 183 (botanique) + [1 (avis au relieur)] pp. (4to) [DP: 1844 (title page)] McG, GB, MDZ

The entire report of Jacquemont’s expedition was published in four volumes of text and two volumes of plates issued in 80 livraisons, 1835–1844. The date of 1844 listed on the title page of volume 4 probably refers to the completion of the volume. Collation and dates of publication of each livraison are desired. Overall, the atlases contain 290 plates and four maps. Three of these plates refer to entomology and all of them pertain to Lepidoptera only.

The Coleoptera are on pages 25–28 of the crustacea and insects section and the new species described are *Mylabris jacquemontii* (p. 26), *Diacanthus fuscipennis* (p. 27), and *Bruchus biguttatus* (p. 27).

**1845**. *Histoire des insectes, traitant de leurs moeurs et de leurs métamorphoses en général et comprenant une nouvelle classification fondée sur leurs rapports naturels.* Firmin Didot Frères, Paris. (8vo) MCZ, GAL, BHL

*Hyménoptères et Coléoptères.* v + 398 pp. + pls 1–10. [DP: 1845 (title page), 11 June 1845 (*Soc Ent Fr*), 28 June 1845 (*Bibl Fr*), 30 June 1845 (*Acad Sci Fr*), 5 July 1845 (*Feuil J Lib*), 9 July 1845 (*Lit Ztg*), 18 July 1845 (*Leip Reper*)].

*Coléoptères, Orthoptères, Thysanoptères, Nevroptères, Lépidoptères, Hémiptères, Aphaniptères, Strepsiptères, Diptères, Anoplures et Thysanures.* 524 pp. + pls 11–20. [DP: 1845 (title page), 11 June 1845 (*Soc Ent Fr*), 28 June 1845 (*Bibl Fr*), 5 July 1845 (*Feuil J Lib*), 9 July 1845 (*Lit Ztg*), 18 July 1845 (*Leip Reper*)].

These volumes were also issued the same year by the publisher F. Savy, under the title “*Histoire naturelle des insectes: leurs moeurs, leurs métamorphoses et leur classification ou traité élémentaire d’entomologie. Avec 20 planches représentant 218 figures*” [CNC]. The two volumes of the Didot set constitute volumes 8 and 9 of the 13–volume series “*Traité complet d’histoire naturelle*” under the direction of Achille Joseph Comte.

[**1851**]. [Insectos. Coleopteros.] Pp. 285–564 *in*: *Historia fisica y politica de Chile segun documentos adquiridos en esta republica durante doce años de residencia en ella y publicada bajo los auspicios del supremo gobierno por Claudio Gay. Zoologia. Tomo quinto.* Autor, Paris [&] Museo de Historia Natural de Santiago, Chile. 564 + 3 (numbered [561]–563) pp. (8vo) [DP: 1851 (title page)] CNC, BHL

The entire *Historia fisica y politica de Chile* of Claude Gay [1800–1873] consists of 28 text-volumes and two atlas-volumes, 1844–1871. The *Zoologia* section forms eight volumes published between 1847 and 1854 based on the title-page dates. The text and plates were issued in 87 parts (Evenhuis 2015c: 40). Collation and dates of publication for the parts are desired.

The Coleoptera are in volumes 4 and 5. There are 32 plates depicting beetles, with scientific names listed at the bottom. These are included in the second volume of “*Atlas de la historia fisica y politica de Chile*” dated 1854 on the title page [GB]. Stuardo Ortiz (1954) provided a list of all botanical and zoological taxa illustrated on the plates.

The remaining pages of volume 5 related to Coleoptera were authored by Solier, 1851 [*q.v.*].

**1853**. *Voyage au Pole Sud et dans l’Océanie sur les corvettes l*’Astrolabe *et la* Zélée; *exécuté par ordre du Roi pendant les années 1837–1838–1839–1840, sous le commandement de M.J. Dumont-d’Urville, Capitaine de vaisseau; publié par ordre du gouvernement, sous la direction supérieure de M. Jacquinot, Capitaine de vaisseau, commandant de la* Zélée. *Zoologie par MM. Hombron et Jacquinot. Tome quatrième.* Gide et J. Baudry, Paris. 422 pp. (4to) [DP: 1853 (title page), 3 March 1854 (Clark and Crosnier 2000: 414)] McG, GB, GAL

The second page is entitled “*Zoologie. Description des insectes par Émile Blanchard.*” The Coleoptera are on pages 1–347.

The entire expedition report was published in 22 volumes and several atlases totaling about 500 plates. The zoology section appeared in three volumes (vol. III: 1853; vol. IV: 1853; vol. V: 1854) and one atlas of 140 colored plates (Clark and Crosnier 2000). Most of the plates were published before the text (see Blanchard, 1842–1854) and the new taxa depicted on them date from the plates.

#### Blandford, Walter Fielding Holloway (London, United Kingdom: 28 December 1864^18^ – 23 January 1952: London, United Kingdom). British entomologist, solicitor, musicologist, and horn player; lecturer in entomology at the Royal Indian Engineering College at Coopers Hill; switched to law and became a solicitor in 1901; manager at Wyke House, Isleworth, a mental institution (1923–1932). Reference. Pegge (1953).

**1895–1905**. Fam. Scolytidae. Pp. 81–298 + pls 4–9 *in*: *Biologia Centrali-Americana. Insecta. Coleoptera. Vol. IV. Part 6. By David Sharp; W.F.H. Blandford; and Karl Jordan.* Taylor and Francis, London. [4] + 396 pp. + 14 pls. (4to) CNC

This work was published in several parts, 1895–1905. The dates listed at the bottom of the first page of each sheet are: (pp. 81–96) December 1895; (pp. 97–112 + pl. 4) January 1896; (pp. 113–120) March 1896; (pp. 121–128 + pl. 5) May 1896; (pp. 129–144) June 1896; (pp. 145–152) August 1897; (pp. 153–168 + pl. 6) September 1897; (pp. 169–176) November 1897; (pp. 177–184) December 1897; (pp. 185–216) January 1898; (pp. 217–224) April 1898; (pl. 7) November 1901; (pp. 225–248) June 1904; (pp. 249–280 + pl. 8) November 1904; (pp. 281–298 + pl. 9) June 1905. The collation for the plates is from Lyal (2011: 85).

#### Block, Peter Ludwig Heinrich von (Baron) (Dresden, Saxony, Germany: 25 February 1764 – 1818: Dresden, Germany). German councilor and natural history and art collector; travelled with Prince Bariatinsky through Germany, northern Italy and Switzerland in 1790; inspector at the historic museum “Grünen Gewölbes” in Dresden (1802–1817); dismissed from his museum job and jailed for embezzlement in 1817. References. Möbius (1944: 3); Gaedike and Smetana (1978: 349); Phillips (2012: 66–67).

**1799**. Verzeichnis der merkwürdigsten Insecten welche in Plauischen Grunde gefunden werden. Pp. 95–120 + 4 pls *in*: *Der Plauische Grund bei Dresden, mit Hinsicht auf Naturgeschichte und schöne Gartenkunst. Herausgegeben von W.G. Becker. Mit fünf und zwanzig Kupferblättern. Zweiter Teil.* Stiebnerianis, Norimbergae [= Nürnberg]. 120 + [1 (Verzeichniss einiger Druckfehler)] pp. + 8 pls. (4to) [DP: 1799 (title page; dedication dated 1 May 1799), 29 May 1800 (*Litt Ztg*), 27 September 1800 (*Allg Lit Ztg*), September 1800 (*Intell Teutsch Merkur*)] MCZ, GB, ARC

Becker’s work consists of two volumes, issued simultaneously. The first one was authored by Wilhelm Gottlieb Becker alone. The second one was authored by several persons. There are 25 plates overall. Pages 115–120 are entitled “*Monographiae Insectorum XVIII. In valle Plauensi delectorum*” in which the following Coleoptera species are described: *Staphylinus edentulus* (p. 115), *Staphylinus mordax* (p. 115), *Staphylinus fuscipennis* (p. 116), *Staphylinus tetracarinatus* (p. 116), *Staphylinus spinipes* (p. 116), *Staphylinus opacus* (p. 117), *Staphylinus multipunctatus* (p. 117), *Staphylinus nigrophthalmus* (p. 117), *Staphylinus bicornis* (p. 118). A few new species are also described in the list of species (pp. 99–113), such as *Carabus chlorophanus* (p. 100), *Gyrinus oblongus* (p. 100), *Mordella elegans* (p. 102) and *Cerambyx atratus* (p. 103).

#### Blumenbach, Johann Friedrich (Gotha, Thuringia, Germany: 11 May 1752 – 22 January 1840: Göttingen, Lower Saxony, Germany). German physician, naturalist, physiologist and anthropologist; appointed professor of medicine at the University of Göttingen in 1776. References. Traill (1854); Marx (1865); Kleinschmidt (1955: 329–330).

**1779**. *Handbuch der Naturgeschichte. Mit Kupfern.* Johann Christian Dieterich, Göttingen. [11] + 448 pp. + 1 pl. (8vo) [DP: 1779 (title page; *Vorrede* dated 24 April 1779), 23 June 1779 (*Goth Ztg*), 30 September 1779 (*Gött Anz*)] GB

This work was published in two volumes, 1779–1780. The Coleoptera are on pages 324–343 of the first volume.

Twelve editions of this work, some of them updated, were issued between 1779 and 1830 (see Kroke 2010: 25–29). Danish, 1793 in Copenhagen [GB], Russian (three volumes), 1797 in Saint Petersburg [GDZ], Dutch, 1802 in Leiden [GB], French, 1803 in Paris [GB], English, 1825 in London [GB], and Italian translations, 1825 in Lugano (*n.v.*) and 1826–1830 in Milan (six volumes) [GB] were published.

#### Bodemeyer, August Rudolf Eduard von (Reindörfel [currently Nieszków], Poland: 2 April 1854 – 21 November 1918: Berlin, Germany). German captain and entomologist; served in the army for 24 years; most specimens of his collection are at the Deutsches Entomologisches Institut in Müncheberg and at the Zoologisches Museum der Humboldt-Universität in Berlin. Reference. Bodemeyer (1928, P).

**1900**. *Quer durch Klein-Asien in den Bulghar-Dagh. Eine naturwissenschaftliche Studien-Reise.* Die Druck- und Verlags-Aktien-Gesellschaft Vormals Dölter, Emmendingen. v + 169 pp. (8vo) [DP: 1900 (title page; *Vorwort* dated 1 May 1900), 5 November 1900 (*Wien Ent Ztg* 19: 252), 22 November 1900 (*Insekten-Börse*), January 1901 (*Nat Nov*), 20 July 1901 (*Bibl Zool*)] USNM, NCSU, BHL, GB

This work includes a section entitled *Coleopterologisches* (pp. 93–169) where several new species-group taxa are described by Max Bernhauer, Jules Bourgeois, Ernst Brenske, Ludwig Ganglbauer, Maurice Pic, Edmund Reitter, and Julius Weise.

#### Boheman, Carl Heinrich (Jönköping, Sweden: 10 July 1796 – 2 November 1868: Stockholm, Sweden). Swedish soldier and coleopterist; entered the army at the age of 17 and retired, with the rank of captain, at 48; in the 1840s, accepted the position of curator of the entomological section at the Museum of Natural History in Stockholm where he deposited his collection; elected honorary member of the Entomological Societies of London, France, Stettin, and Netherlands. References. Stål (1869a, 1869b).

**1848–1857**. *Insecta Caffraria annis 1838–1845 a J. A. Wahlberg collecta.* Officina Norstedtiana, Holmiae [= Stockholm]. (8vo) CNC, MCZ, GB

*Pars. I. Fascic. I. Coleoptera. (Carabici, Hydrocanthari, Gyrinii et Staphylinii).* viii + 297 pp. [DP: 1848 (title page; preliminaries dated 10 July 1848), 10 October 1848 (*Vetens Akad*), 19 October 1848 (*Lit Ztg*), 26 October 1848 (*Allg Bibl Deutsch*), 15 November 1848 (*Intell Serapeum*; *Publ Circ*), November 1848 (*Isis*, Heft XI: [2]), 4 December 1848 (*Ent Soc Lond*)].

*Pars. I. Fascic. II. Cum Tab. II aen. Coleoptera. (Buprestides, Elaterides, Cebrionites, Rhipicerides, Cyphonides, Lycides, Lampyrides, Telephorides, Melyrides, Clerii, Terediles, Ptiniores, Palpatores, Silphales, Histeres, Scaphidilia, Nitidulariae, Cryptophagidae, Byrrhii, Dermestini, Parnidae, Hydrophilidae).* [1] + pp. 299–626 + 2 pls. [DP: 1851 (title page), 13 November 1851 (*Allg Bibl Deutsch*; *Vetens Akad*), 30 November 1851 (*Intell Serapeum*), 4 December 1851 (*Ent Ver Stettin*), 5 January 1852 (*Ent Soc Lond*), January–June 1852 (*Bibliot Hist-Nat*)]. The title pages of this and subsequent fascicles read “*Insecta Caffraria annis 1838–1845 a J. A. Wahlberg collecta, amici auxilio suffultus*.”

*Pars. II. Cum Tab. I. Coleoptera. (Scarabaeides.).* [2] + 395 pp. + 1 pl. [DP: 1857 (title page), 8 November 1857 (*Ent Ver Stettin*), 11 November 1857 (*Vetens Akad*), 14 January 1858 (*Allg Bibl Deutsch*), March 1858 (*Allg Bibl*), 15 April 1858 (*Intell Serapeum*)].

This work contains Latin descriptions of the Coleoptera collected by Johan August Wahlberg [1810–1856] in the region of Kaffraria, in present-day South Africa, during the years 1838–1845.

Supplements to this work were published by Olof Immanuel Fåhraeus, 1870–1872, in volumes 27, 28, and 29 of *Öfversigt af Kongl. Svenka Vetenskaps–Akademiens Förhandlingar*.

**1850–1862**. *Monographia cassididarum.* Officina Norstedtiana, Holmiae [= Stockholm]. (8vo) CNC, GB

*Tomus primus cum Tab. IV.* [4] + 452 pp. + 4 pls. [DP: 1850 (title page; preliminaries dated 10 July 1850), 11 September 1850 (*Vetens Akad*), 3 October 1850 (*Allg Bibl Deutsch*), 15 October 1850 (*Intell Serapeum*; *Bibl Lit Anz*), 3 February 1851 (*Ent Soc Lond*)].

*Tomus secundus cum Tab. II.* 506 pp. + pls 5–6. [DP: 1854 (title page), 10 May 1854 (*Vetens Akad*), 3 July 1854 (*Ent Soc Lond*)].

*Tomus tertius cum Tab. I.* 543 pp. + pl. 7. [DP: 1855 (title page), 10 October 1855 (*Vetens Akad*), 24 November 1855 (*Feuil J Lib*), February 1856 (*Ent Ztg* 17: 64), 1 October 1856 (*Zool-Bot Gesell Wien*), 2 March 1857 (*Ent Soc Lond*), January–June 1857 (*Bibliot Hist-Nat*)].

*Tomus quartus. (Supplementum).* vi + 504 pp. [DP: 1862 (title page; preface dated 14 April 1862), 14 May 1862 (*Vetens Akad*), 7 July 1862 (*Ent Soc Lond*)].

**1856**. *Catalogue of coleopterous insects in the collection of the British Museum. Part IX. Cassididae.* The Trustees, London. [2] + 225 pp. (8vo) [DP: 1856 (title page; preface dated November 1856), 14 February 1857 (Sherborn 1934: 310)] McG, GB

**1858–1859**. Coleoptera. Species novas descripsit. Pp. 1–217 + pls 1–2 *in*: *Kongliga Svenska Fregatten* Eugenies *Resa omkring jorden under befäl af C.A. Virgin, Åren 1851–1853. Vetenskapliga Iakttagelser på H.M. Konung Oscar den Förstes befallning utgifna af K. Svenska Vetenskaps Akademien. Andra delen. Zoologi. 1. Insecta.* P.A. Norstedt & Söner, Stockholm. [1] + 617 pp. + 9 pls. (4to) CNC, BHL

The Coleoptera section was published in two parts, corresponding to *Häfte* 4 and 6 of the whole work (see Linnström 1883: 385): (pp. 1–112) 1858; (pp. 113–218) 1859, August 1859 (*Swensk Bibl*). Persson (1971) presented information regarding the voyage and the insect collection. The book was reissued in 1910 by Alqvist & Wiksells in Uppsala & Stockholm, with Latin text [McG].

This voyage was the first Swedish circumnavigation expedition, undertaken during the years 1851–1853 under the command of Chistian Adolf Virgin [1797–1870]. The crew visited South America, Panama, Galapagos Islands, California, Hawaii, Tahiti, Australia, Hong Kong, Canton, the Philippines, Mauritius and the Cape. The entire expedition report was published in three sections, botany, zoology, and physics, in five volumes, 1857–1910. The first official account of the expedition was published in 1851–1854 by C.J.A. Skogman as “Fregatten *Eugenies* Resa omkring jorden.”

#### Boisduval, Jean Baptiste Alphonse Déchauffour de (Ticheville, Normandy, France: 17 June 1799 – 30 December 1879: Ticheville, France). French physician, horticulturist and entomologist, primarily known for his work on Lepidoptera; first worked in drug stores in various cities of France and received his medical degree in 1828; founding member of the *Société Entomologique de France* in 1832; the same year, received the Honor Cross from the French government for his devotion to the sick during the cholera epidemic of 1830 in Paris; worked for the General Dejean [*q.v.*] in Paris for some years as curator of his collection. References. Girard (1880); Oberthür (1880); Letacq (1889 : 250–255); Gouillard (2004: 58–59); Girardin (2010, P).

**1835**. *Voyage de découvertes de l*’Astrolabe *exécuté par ordre du Roi, pendant les années 1826–1827–1828–1829, sous le commandement de M. J. Dumont d’Urville. Faune entomologique de l’Océan Pacifique, avec l’illustration des insectes nouveaux recueillis pendant le voyage. Deuxième partie. Coléoptères et autres ordres.* J. Tastu, Paris. vii + 716 pp. (8vo) [DP: 1835 (title page; *avertissement* dated February 1835), 27 March 1835 (*Soc Géog Fr*), 9 May 1835 (*Bibl Fr*), 20 May 1835 (*Lit Ztg*), May 1835 (Guérin-Méneville 1838: 271), 3 June 1835 (*Soc Ent Fr*), June 1835 (*Bull Bibl*)] MCZ, GB

The expedition report was divided into five sections: *Histoire du voyage*; *Botanique*; *Observations nautiques et de physique*; *Philologie*; and *Zoologie*. The *Faune entomologique de l’Océan Pacifique*, part of the *Zoologie* section, was issued into two volumes, both written by Boisduval. The first one, dealing with Lepidoptera, was published in 1832; the second one, dealt with here, comprises livraisons 36–38 of the zoology portion (Evenhuis 1997a: 104). Twelve plates were issued for the entomology part, including four on Coleoptera (pls 6–9). I found no dates of publication for the plates except that Crotch (1874: 32) reported that the first livraison of plates was issued in 1832. Since only French vernacular names are provided for the species illustrated on the plates, they are not nomenclaturally significant.

This work was also published the same year under the title “*Faune entomologique de l’Océanie comprenant les coléoptères, les hémiptères, les orthoptères, les névroptères, les hyménoptères et les diptères*” by Roret, Paris [CNC, BHL].

**1835**. [Boisduval, J.B.A. and Lacordaire, J.T.] *Faune entomologique des environs de Paris; ou species général des insectes qui se trouvent dans un rayon de quinze à vingt lieues aux alentours de Paris. Tome premier.* Méquignon-Marvis, Père et Fils, Paris. [2] + 17 + 696 [numbered i–cii, 103–696] pp. + 3 pls. (8vo) [DP: September 1835 (title page; *préface* dated 25 August 1835), 26 September 1835 (*Bibl Fr*), September 1835 (*Bull Bibl*), 7 October 1835 (*Soc Ent Fr*)] CNC, SME, GB, GAL

The preface along with the list of works (pp. 1–17) and generalities (i–107) are attributed to both authors. As indicated in the preface (p. 9), Lacordaire is the sole author of the text relating to the orders, including the Coleoptera (pp. 133–677), treated in this volume. According to the publisher, the entire work was to consist of three volumes but only the first one was issued.

#### Boitard, Pierre (Mâcon, Saône-et-Loire, France: 27 April 1789 – 25 August 1859: Montrouge, Hauts-de-Seine, France). French naturalist, agronomist, technologist and popularizer of natural history; field officer in the irregular force during the last period of Napoleon’s reign (les Cent-Jours); founded the *Journal des Jardins* and the *Journal de Flore*; mostly known for the numerous manuals on botany, natural history, and rural economy he published, often under pseudonyms such as Vérardi, A. Poiteau, and Ragonnot-Godefroy. References. Vapereau (1861: 225); Anonymous (1862b).

**1828**. *Manuel d’entomologie, ou histoire naturelle des insectes, contenant la synonymie et la description de la plus grande partie des espèces d’Europe et des espèces exotiques les plus remarquables.* Roret, Paris. (18mo) GB, GAL

*Tome premier.* [1] + 435 + [1] pp. [DP: 1828 (title page), 29 March 1828 (*Bibl Fr*), January–April 1828 (*Foreign Quart Rev*), 21 May 1828 (*Rev Bibl-Brus*), August 1828 (*Rev Encycl*)].

*Tome second.* 417 + [1] pp. [DP: 1828 (title page), 29 March 1828 (*Bibl Fr*), January–April 1828 (*Foreign Quart Rev*), 21 May 1828 (*Rev Bibl-Brus*), August 1828 (*Rev Encycl*)].

The Coleoptera are in the first (pp. 87–435) and second (pp. 1–89) volumes. An atlas of 110 plates, either colored or uncolored, was published under the title “*Atlas des insectes, composé de 110 planches, représentant la plupart des insectes décrits dans le Manuel d’Histoire Naturelle et dans le Manuel d’Entomologie*” [BHL]. There is no date on the title page but it was noticed on 6 July 1844 (*Bibl Fr*) and 20 July 1844 (*Monit Lib*).

A second edition of this work was published, 1843, in three volumes, by Roret, Paris, under the title “*Nouveau manuel complet d’entomologie ou histoire naturelle des insectes et des myriapodes, contenant la synonymie et la description de la plus grande partie des espèces d’Europe et des espèces exotiques les plus remarquables. Nouvelle édition, revue et considérablement augmentée*” [GB]; it was noticed on 29 May 1843 (*Bibl Fr*) and 7 June 1843 (*Soc Ent Fr*).

#### Bonelli, François André (Cuneo, Piedmont, Italy: 10 November 1784 – 18 November 1830: Turin, Piedmont, Italy). Italian zoologist; made a one-year stay in Paris in 1810–1811 and upon his return was appointed professor of zoology and director of the zoological museum at the University of Turin; gave his collection to the University of Turin. References. Gené (1834); Passerin d’Entreves and Sella Gentile (1985); Mearns and Mearns (1988: 83–85, P); Casale and Giachino (1998, P).

**1810**. *Observations entomologiques. Première partie (cicindélètes et portion des carabiques).* Félix Galletti, Turin. 58 pp. + 1 table (“*Tabula synoptica exhibens genera carabicorum in sectiones et stirpes disposita*”). (4to) [DP: 1810 (Madge 1978: 11), April 1810 (*Mag Encycl*, p. 379^19^)] (*n.v.*).

The text was reissued in 1811, without the “*Tabula synoptica*,” in *Memorie della Reale Accademia della Scienze di Torino* 18 [1809–1810]: 21–78 (Madge 1978: 10). A facsimile of the *Tabula synoptica* was published in Gaskin and Lewis (1956).

In Opinion 1226 (ICZN 1982) the Commission ruled that Bonelli’s *Observations entomologiques*, première partie, including the *Tabula synoptica exhibens genera carabicorum in sectiones et stirpes disposita* is an available work and that it was published in 1810.

#### Bonsdorff, Gabriel von (Porvoo, Uusimaa, Finland: 6 November 1762 – 22 November 1831: Turku, Turku ja Pori, Finland). Finnish physician and naturalist; professor of natural history and later of surgery and anatomy at the University of Åbo (Turku) (1786–1823); president of the Medical College in 1815 and 1819. References. Anonymous (1859b); Schrader and Hering (1863: 49–50); Hjelt (1884).

**1785**. *Historia naturalis curculionum Sveciae.* [2 theses]. Johan Edman, Upsaliae [= Uppsala]. (4to) MCZ, GDZ, GB

*Cujus partem primam, consent. experient. facult. med. examini offerunt auctor Gabriel Bonsdorff, et respondens Laurentius G. Borgström, stipendiarius regius. Fennones. In audit. Gust. Maj. d. XXV Junii, an. MDCCLXXXV. H.a.m.s.* 18 pp. [DP: 25 June 1785 (title page)].

*Cujus partem secundam, consent. experient. facult. med. examini offerunt auctor Gabriel Bonsdorff, et respondens Petrus Ant. Norlin, Stockholmiensis. In audit. Gust. Maj. d. XXVII Junii, an. MDCCLXXXV. H.a.m.s.* Pp. 19–42 + 1 pl. [DP: 27 June 1785 (title page)].

#### Bonvouloir, Henry [Henri] Achard de (Viscount) (Paris, France: 25 May 1839 – 13 July 1914: Paris, France). French entomologist, specialist of Coleoptera and particularly the families Throscidae and Eucnemidae; for many years took care of the archives and library of the *Société Entomologique de France*; his collection, rich in exotic and cave-dwelling species, was purchased by René Oberthür [*q.v.*] who kept a first choice and dispersed the other specimens. References. Rabaud (1914); Cambefort (2006: 128–129, P).

**1859**. *Essai monographique sur la famille des throscides.* A. Deyrolle, Paris. xviii + 144 pp. + 5 pls. (8vo) [DP: 1859 (title page), 30 April 1859 (*Bibl Fr*), January–June 1859 (*Bibliot Hist-Nat*), July 1859 (*Allg Bibl*)] CNC, GB

**1871–1875**. *Monographie de la famille des eucnémides.* Félix Malteste et C^ie^, Paris. 907 pp. + 42 pls. (4to) ANSP, BHL, GB

This work was issued in four *cahiers* as supplements to the *Annales de la Société Entomologique de France*: **1**: (pp. 1–288 + pls 1–?21) 1871 (wrapper), 15 August 1871 (*Pet Nouv Ent*); **2**: (pp. 289–448 + pls ?22–?28) 15 July 1872 (wrapper), 1 August 1872 (*Pet Nouv Ent*); **3**: (pp. 449–560 + pls ?29–36) 31 December 1872 (wrapper); **4**: (pp. 561–907 + pls 37–42) 28 April 1875 (wrapper). All plates have the inscription “Annales de la Société entomologique de France 4^e^ Série. Tome X. 2^e^ Partie (1870).”

#### Borkhausen [Borckhausen], Moritz Balthasar (Giessen, Hesse, Germany: 3 December 1760 – 30 November 1806: Darmstadt, Hesse, Germany). German naturalist and forester; assessor for the water and forest management in Darmstadt. References. Jourdan (1820); Hess (1876); Weidner (1981: 111–112).

**1797**. *Epitome entomologiae Fabricianae sive nomenclator entomologicvs emendatvs sistens Fabriciani systematis cvm Linneano comparationem adiectis characteribvs ordinvm et genervm, speciebvs novis aliorvm entomologorvm, insectorvm habitationibvs nominibvs Germanorvm Francogallorvm Anglorvm. Cvm indicibvs et bibliotheca Fabriciana.* Gottlob Feind, Lipsiae [= Leipzig]. xvi + 224 pp. (8vo) [DP: 1797 (title page; *lectori ervdito s.d. editor* dated 14 June 1797), 24 November 1797 (*Allg Lit Ztg*)] GB, GAL

This work was published anonymously but some bibliographers (Engelmann 1846: 477; Hagen 1862: 109; Horn and Schenkling 1928a: 73–74; Evenhuis 1997a: 108) attributed it to Borkhausen; Ersch (1821: 586) credited it to Christian Friedrich Ludwig; Oudemans (1929: 1091) to Friedrich Weber. This is an index of the insect names in Fabricius’ *Entomologia systematica* (1792–1794) [*q.v.*]. An “editio nova” was published in 1810 by I.C. Hinrichs, Lipsiae [USNM, BHL]; it is essentially a reissue of the original.

#### Börner, Immanuel [Emanuel] Karl [Carl] Heinrich (Klobikau [currently part of Bad Lauchstädt], Saxony-Anhalt, Germany: 10 June 1745 – 13 April 1807: Breslau [currently Wrocław], Poland). German landscape counsel; court tutor for Count of Manteuffel in Livland; from 1775, landscape legal adviser in Wrocław and secretary of the Silesian Patriotic Society. Reference. Straubel (2009: 101).

**1774**. *Sammlungen aus der Naturgeschichte, Oekonomie-Polizey-Kameral- und Finanzwissenschaft. Erster Theil. Mit Kupfern.* Hilscher, Dresden. xii + [2] + 567 pp. + 1 pl. (8vo) [DP: 1774 (title page; *Vorrede* dated 25 April 1774), 12 August 1774 (*Jena Ztg*), 22 April 1775 (*Gött Anz*)] GB, SDL

The Coleoptera on pages 458–479.

#### Bouché, Peter Friedrich (Berlin, Germany: 15 February 1785 – 3 April 1856: Berlin, Germany). German horticulturist and entomologist; along with his brother Peter Carl carried on their father’s horticulture business; part of his insect collection is at the Deutsches Entomologisches Institut in Müncheberg. References. Anonymous (1859e: 117–122); Ratzeburg (1874: 70–72); Weidner (1981: 113–114).

**1833**. *Naturgeschichte der schädlichen und nützlichen Garten-Inskecten und die bewährtesten Mittel zur Vertilgung der ersteren.* Nicolai, Berlin. vi + 176 pp. (8vo) [DP: 1833 (title page), 16 June 1833 (*Bibl Deutsch*), January–June 1833 (*Verz Bücher Landkarten*), 20 August 1833 (*Intell Ztg Welt*)] GB

The Coleoptera are on pages 15–33 and 139–146.

**1834**. *Naturgeschichte der Insekten, besonders in Hinsicht ihrer ersten Zustände als Larven und Puppen. Erste Lieferung. Mit 10 Kupfertafeln.* Nicolai, Berlin. v + [3] + 216 pp. + 10 pls. (8vo) [DP: 1834 (title page), January–June 1834 (*Verz Bücher Landkarten*), 13 July 1834 (*Allg Bibl*), 16 July 1834 (*Lit Ztg*), September 1834 (*Intell Allg Lit Ztg*; *Nouv Rev Germ*), 15 October 1834 (*Allg Forst Jagd Ztg*)] GB, MDZ

This is the only part published. The Coleoptera are on pages 179–206 and plates 7–10.

#### Bourgeois, Jules (Sainte-Marie-aux-Mines, Haut-Rhin, France: 31 May 1847 – 18 July 1911: Sainte-Marie-aux-Mines, France). French fabric merchant and coleopterist, specialist of the families Lycidae and Cantharidae; his collection was bequeathed to the Muséum National d’Histoire Naturelle in Paris. References. Constantin (1992: 17–18); Cambefort (2006: 131).

**1884–1893**. *Faune gallo-rhénane ou species des insectes qui habitent la France, la Belgique, la Hollande, le Luxembourg, la Prusse Rhénane, le Nassau et le Valais avec tableaux synoptiques et planches gravées. Coléoptères. Tome quatrième.* F. Le Blanc-Hardel, Caen. 208 + 34 + x pp. (8vo) CMLE

This volume, the fourth of the *Faune gallo-rhénane*, covers the cébrionides (pp. 1–8), dascillides (pp. 9–45), and malacodermes (pp. 46–208). It was issued in parts as supplements to *Revue d’Entomologie*. The dates of publication are listed on page i and at the bottom of the first page of the gatherings for the supplement: (pp. [1] + 1–60) 1884; (pp. 61–100) 1885; (pp. 101–132) 1886; (pp. 133–156) 1887; (pp. 157–172) 1888; (pp. 173–208) 1892; (pp. 1–16 [supplement]) June 1893; (pp. 17–34 [supplement]) July 1893; (pp. i–x) December 1893. The preface is dated 14 April 1884. The first three parts of the *Faune gallo-rhénane* were authored by Fauvel [*q.v.*] and the fifth one by Buysson [*q.v.*].

#### Bracciforti, Alberto. Professor of mathematics and natural sciences at the technical school of Viadana in northern Italy. Reference. Conci (1975: 860).

**1873**. *L’entomologia degli stagni ossia catalogo sistematico degli insetti che vivono nelle acque e sulle plante che in esse si trovano.* Remagni Nicodemo, Viadana. 93 + [1 (Errata)] pp. (8vo) [DP: 1873 (title page)] GB

The Coleoptera are on pages 7–32 and 90 (Appendice); many species are commented, some described but none are new to science.

#### Brahm, Nikolaus Joseph (Mainz, Rhineland-Palatinate, Germany: 18 May 1754 – 29 June 1821: Mainz, Germany). German lawyer and naturalist in Mainz. Reference. Niehuis (2011).

**1790**. *Insektenkalender für Sammler und Oekonomen. Erster Theil.* Kurfürstl. Privileg. Universitätsbuchhandlung, Mainz. lxlii + 248 pp. (8vo) [DP: 1790 (title page; *Vorbericht* dated 20 December 1789), 12 May 1790 (*Ober Lit*), 30 June 1790 (*Allg Lit Ztg*), 16 October 1790 (*Gött Anz*)] CMLE, GB

The entire work consists of two volumes. Only the first one has parts related to Coleoptera. The second *Theil*, issued in 1791, pertains to Lepidoptera.

**1797**. Verzeichniss der, von der Gattung *Clerus* Fabr. in den Gegenben von Mainz und Aschaffenburg einheimischen Arten. Pp. 133–139 *in*: *Entomologisches Taschenbuch für die Anfänger und Liebhaber dieser Wissenschaft auf das Jahr 1797*. *Herausgegeben von David Heinrich Hoppe*. Montag und Weiss, Regensburg. [2] + 252 pp. (8vo) [DP: n.d. (title page), 23 August 1797 (*Allg Lit Ztg*)] CMLE, MDZ

One new species is described, *Clerus rufipes* (p. 136).

#### Brancsik, Károly [Karl, Carl, Karel] (O-Besztercze [currently Stará Bystrica], Slovakia: 13 March 1842 – 18 November 1915: Trenčín, Slovakia). Hungarian physician and zoologist; a large part of his beetle collection was acquired in 1955 with the collection of Eduard Knirsch by the Field Museum in Chicago while the Chrysomelidae, the exotic Cerambycidae, and Curculionidae are in Museum Georg Frey in Tutzing, Germany. References. Jenő (1916, P); Hetschko (1932, P); Kiss (2015).

**1871**. *Die Käfer der Steiermark. Systematisch zusammengestellt.* Paul Cieslar, Graz. [2] + 114 pp. (8vo) [DP: 1871 (title page; *Vorrede* dated May 1871)] MCZ, GB

This work is an annotated catalogue of the beetles of Styria in Austria.

#### Brandt, Johann Friedrich von (Jüterbog, Brandenburg, Germany: 25 May 1802 – 15 July 1879: Meriküla, Estonia). German physician and naturalist; associate professor at the University of Berlin; later, professor in Saint Petersburg, Russia, and director of the zoological department at the Academy of Sciences; made several collecting trips in the Russian empire. References. Ratzeburg (1874: 72–76); Strauch (1889: 5–28); Stieda (1903); Adler (2012: 105–106, P).

**1830–1833**. [Brandt, J.F. and Ratzeburg, J.T.C.] *Medizinische Zoologie oder getreue Darstellung und Beschreibung der Thiere, die in der Arzneimittellehre in Betracht kommen, in systematischer Folge. Zweiter Band. Mit 39 Kupfertafeln unter XXXVI nummern.* A. Hirschwald, Berlin. iv + 364 pp. + 39 pls. (4to) GB, MDZ

This work consists of two volumes, the first one issued 1827–1829 (Hagen 1862: 82) and the second one 1830–1833. The second volume was issued in eight parts recorded as follows: **1–2**: (pp. 1–52 + 10 pls) 2 October 1830 (*Bibl Deutsch*); **3–4**: (pp. 53–124 + 10 pls) 24 September 1831 (*Pharm Centr*), 8 October 1831 (*Bibl Deutsch*), July–December 1831 (*Verz Derj Bücher*); **5**: (pp. 125–196 + 5 pls) 11 August 1832 (*Bibl Deutsch*); **6–8**: (pp. iv + 197–364 + 14 pls) 10 December 1833 (*Bibl Deutsch*), January–June 1834 (*Verz Bücher Landkarten*). The title page is dated 1833, the *Vorrede* August 1833. Sherborn (1922a: xxix) gave the following publication dates: **1–3**: 1831; **4–6**: 1832; **7–8**: 1833. The Coleoptera are treated in the second volume (pp. 100–142). At least two new species are described, *Meloe reticulatus* (p. 108) and *Lytta violacea* (p. 123). Sherborn (1932a: 6915) dated the second species from 1832; the first one was omitted. Based on this study, both species should be dated 1831.

#### Brême [Breme], François de [Ferdinando Arborio Gattinara di] (Marquis) (Milan, Lombardy, Italy: 30 April 1807 – 23 January 1869: Florence, Tuscany, Italy). Italian senator of independent means, naturalist and etcher; director of the Albertina Academy; his collection is at the Zoology Museum of the University of Turin. References. Barbiellini Amidei (1943); Conci (1975: 833); Goidanich (1975: 147–163); Conci and Poggi (1996: 198).

**1842**. *Monographie de quelques genres de coléoptères hétéromères, appartenant à la tribu des blapsides.* Lachèze, Paris. 25 + [1] pp. + 1 pl. (12mo) [DP: 1842 (title page), 28 May 1842 (*Bibl Fr*), 1 June 1842 (*Soc Ent Fr*), 8 June 1842 (*Lit Ztg*), 15 June 1842 (*Monit Lib*), 15 October 1842 [JD] (*Soc Imp Nat Moscou*)] CMLE

The text of this publication was also issued, almost integrally, in *Revue Zoologique* 1842: 81–83 [March], 106–114 [April] under the titles “Note monographique sur le genre *Misolampus*” (pp. 81–83) and “Monographie des *Sphaerotus* et de quelques autres genres appartenant au premier groupe de la tribu des blapsides (famille des Coléoptères hétéromères)” (pp. 106–114). I have noticed the following two changes in the spelling of the scientific names between the two publications: *Misolampus gondotii* (p. 11) is spelled *Misolampus goudotii* (p. 82) in the journal and *Sphoerotus loevigatus* (p. 15) is spelled *Sphoerotus levigatus* (p. 108).

**1842–1846**. *Essai monographique et iconographique de la tribu des cossyphides*. Lachèze [vol. 1] / Victor Masson [vol. 2], Paris. (8vo) CMLE, GB

*Première partie.* 72 pp. + 7 pls. [DP: 1842 (title page; *avertissement* dated 15 September 1842), 22 October 1842 (*Bibl Fr*), 2 November 1842 (*Lit Ztg*), 7 November 1842 (*Acad Sci Fr*), 10 November 1842 (*Monthly Lit Adv*), 15 November 1842 (*Monit Lib*), 7 December 1842 (*Soc Ent Fr*)].

*Deuxième partie. (Cossyphus proprement dit.).* 31 + [1] pp. + 3 pls. [DP: 1846 (title page), 22 April 1846 (*Soc Ent Fr*), June 1846 (*Rev Zool*)].

#### Bremi-Wolf, Johann Jacob (Dübendorf, Switzerland: 25 May 1791 – 27 February 1857: Zürich, Switzerland). Swiss entomologist and turner in Zürich; lost his hearing due to illness at the age of 11. References. Menzel (1858, P); Bremi-Wolf (1871, P).

**1856**. *Catalog der schweizerischen Coleopteren, als Vorläufer der Beiträge für schweizerische Entomologie.* Friedrich Schulthess, Zürich. vi + 78 pp. (8vo) [DP: 1856 (title page), 27 August 1856 (*Schweiz Naturfors Gesell*), 28 August 1856 (*Allg Bibl Deutsch*), 30 September 1856 (*Intell Serapeum*), September 1856 (*Allg Bibl*), July–September 1856 (*Cat Muquardt*), 1 October 1856 (*Zool-Bot Gesell Wien*), July–December 1856 (*Bibliot Hist-Nat*)] CMLE (mf), GB

#### Brodie, Peter Bellinger (London, United Kingdom: 1815 – 1 November 1897: Rowington, Warwickshire, United Kingdom). British geologist and clergyman; rector of Down Hatherley in Gloucestershire; later, vicar of Rowington in Warwickshire. References. Woodward (1897, P); Anonymous (1910d).

**1845**. *A history of the fossil insects in the Secondary rocks of England. Accompanied by a particular account of the strata in which they occur, and of the circumstances connected with their preservation.* John van Voorst, London. xviii + 130 pp. + 11 pls. (8vo) [DP: 1845 (title page), 21 June 1845 (*Athenaeum*; *Lit Gaz*), June 1845 (Low 1853: 44), 1 July 1845 (*Publ Circ*; *Monthly List Muquardt*), 8 August 1845 (*Leip Reper*)] GB

Several new fossil species of beetles, including *Carabus elongatus*, *Coccinella wittsii* and *Elater vetustus*, are named and made available by illustrations.

A section entitled “Introductory observations by Mr. Westwood” (pp. xi–xviii) suggests that J.O. Westwood should be regarded as the author of the new species illustrated on plates 2–6. Evenhuis (1997a: 128) believed so while Sherborn, in his *Index Animalium*, credited the new species to Brodie.

#### Broun, Thomas (Edinburgh, Scotland, United Kingdom: 15 July 1838 – 24 August 1919: Auckland, New Zealand). British military, educator and entomologist; entered the army at the age of 16 and participated to the Crimean War (1853–1856); afterward stationed in Burma and later in India; retired from the army in 1862 at the age of 24 and the next year moved to New Zealand; appointed Captain and served during the Maori War and eventually raised to the rank of Major; from 1876 to 1888, worked as a teacher in several places in New Zealand; in 1894, appointed government entomologist in Auckland; his collection of New Zealand beetles is at the Natural History Museum in London. References. Zimmerman (1993: 486, P); Smetana and Herman (2001: 49, P); Cambefort (2006: 135–136).

**1880–1893**. *Manual of the New Zealand Coleoptera.* Colonial Museum & Geological Survey Department [parts 1–IV] / New Zealand Institute [parts V–VII], Wellington. (8vo) CNC, BHL

[*Part I*]. xix + 651 pp. [DP: 1880 (title page; preface dated June 1880), 12 August 1880 (*New Zeal Parl Debates* 37: 293), January 1881 (*Nat Nov*)].

*Part II*. viii + pp. 653–744 + xxi–xxiii. [DP: 1881 (title page; preface dated 20 May 1881), May 1882 (*Nat Nov*)].

*Parts III. and IV.* xvii + pp. 745–973. [DP: 1886 (title page; preface dated April 1886), February 1887 (*Nat Nov*)].

*Parts V., VI., and VII.* xvii + pp. 975–1504. [DP: 1893 (title page; preface dated 14 January 1893), 28 September 1893 (*Nature*), November 1893 (*Nat Nov*)].

The text of *Part III* (species number 1322–1450) was first published in *The New Zealand Journal of Science* 1: 215–227, 287–304, 367–382, 430–441, 487–499, issued in 1882–1883, of which a reprint (79 pp.) was made available by Wilkie in 1883. The nomenclatural changes noted between the journal (1882–83) and book (1886) printings are: *Somemus rectus*, n.sp. (p. 303) and *Somemus vittatus*, n.sp. (p. 304) are changed in the book to *Lomemus rectus*, n.s. (p. 774) and *Lomemus vittatus*, n.s. (p. 775) respectively; *Ancistropterus prasinus*, n.sp. (p. 435) to *Amylopterus prasinus*, n.s. (p. 797); *Pentarthrum erenatum*, n.sp. (p. 489) to *Pentarthrum crenatum*, n.s. (p. 804); and *Eudontus*, n.gen. (p. 492) to *Euodontus*, nov. gen. (p. 808). Several species noted as new in *Part IV* were first described in *The New Zealand Journal of Science* 2: 226–228, 238–239, 384–387, issued in 1884–1885.

#### Brown, Edwin (*ca* 1819 – 1 September 1876: Tenby, Wales, United Kingdom). British banker and naturalist; manager of the Burton, Uttoxeter and Ashbourne Union Bank in Burton-on-Trent; his collection was sold at auction in March 1877. Reference. Anonymous (1876c).

**1863**. Fauna of the neighbourhood of Burton-on-Trent. Pp. 84–230 *in*: *The natural history of Tutbury. By Sir Oswald Mosley, Bart., D.C.L., F.L.S. Together with the fauna and flora of the district surrounding Tutbury and Burton-on-Trent by Edwin Brown. With an appendix.* John Van Voorst, London. xiii + [1] + 408 pp. + 9 pls. (8vo) [DP: 1863 (title page; preface dated 28 July 1863), 19 December 1863 (*Athenaeum*), January–June 1864 (*Bibliot Hist-Nat*)] GB

The work includes a list of the Coleoptera of the district around Burton-on-Trent in east Staffordshire, England (pp. 140–163).

#### Brown, Peter (? – 1799: London, United Kingdom). English (Danish-born) naturalist and natural history illustrator in London. References. Bryan (1886: 188); Raat (2010: 550–551).

**1775–1776**. *Nouvelles illustrations de zoologie, contenant cinquante planches enluminées d’oiseaux curieux, et qui non étés jamais descrits, et quelques de quadrupedes, de reptiles et d’insectes, avec de courtes descriptions systematiques. / New illustrations of zoology, containing fifty coloured plates of new, curious, and non-descript birds, with a few quadrupeds, reptiles and insects. Together with a short and scientific description of the same.* B. White, London. [2] + 136 pp. + 50 pls. (4to) [DP: 1776 (title page; preface dated 9 May 1776)] McG, BHL, GB

The text is written in French and English. The Coleoptera described in the text are figured on plates **49**: *Cerambyx punctatus* of Cape of Good Hope, fig. 1; *Scarabaeus oblongus* of Africa, fig. 4; *Scarabaeus serratipes* of Africa, fig. 5; *Curculio pulcher* of Madeira and Jamaica, fig. 6; *Curculio lividus* of South Carolina, fig. 8; and **50**: *Cerambyx regalis* of Africa, fig. 1; *Cerambyx unicolor* of Africa, fig. 4. In the preface the author mentioned “je dois encore toutes les descriptions des insectes à M. Yeats” [I owed all the insect descriptions to Mr. Yeats (taken here to be Thomas Pattinson Yeats [*q.v.*])]. The new species should be credited to Yeats. The plates are dated 1775 but no names are listed on them.

#### Browne, Patrick (Claremorris, County Mayo, Ireland: *ca* 1720 – 29 August 1790: Claremorris, Ireland). Irish physician and naturalist; spent a year in the Caribbean before studying botany and medicine in Paris (1738–1742) and Leiden; practiced medicine in London for two years; travelled several times to the West Indies where he practiced medicine, mostly in Antigua and Jamaica, and collected natural history specimens; retired to Ireland after 1781. References. Anonymous (1793); Chalmers (1813: 132–135); Nelson (1997, 2004).

**1789**. *The civil and natural history of Jamaica. Containing I. An accurate description of that island, its situation, and soil; with a brief account of its former and present state, government, revenues, produce, and trade. II. An history of the natural productions, including the various sorts of native fossils; perfect and imperfect vegetables; quadrupeds, birds, fishes, reptiles, and insects; with their properties and uses in mechanics, diet, and physic. Illustrated with forty-nine copper plates; in which the most curious productions are represented of their natural sizes, and delineated immediately from the objects, by George Dionysius Ehret. There are now added complete Linnaean indexes, and a large and accurate map of the island.* B. White and Son, London. viii + 503 + [46] pp. + 49 pls. (Folio) [DP: 1789 (title page), 16 November 1789 (Anonymous 1793: 4)] MCZ

This is a second edition of a work first published in March 1756. Differences between the two editions are noted by Nelson (1997). This work was placed on the *Official Index of Rejected and Invalid Works in Zoological Nomenclature* (Direction 32, ICZN 1956a).

The Coleoptera are on pages 428–433.

#### Brullé, Gaspard Auguste (Paris, France: 7 April 1809 – 21 January 1873: Dijon, Côte-d’Or, France). French naturalist, best known in his early years for his participation in the scientific expedition of Jean-Baptiste Bory de Saint-Vincent to Morea in Greece; founding member of the *Société Entomologique de France*; assistant to Jean-Victor Audouin [*q.v.*] at the Museum of Paris by 1833 where he worked mainly on Coleoptera; at the age of 30 defended his doctoral dissertation of 28 pages about the genus and species limits in zoology; eventually accepted a position of professor at the Faculty of Dijon where he was named dean of the science department in 1861; not known to own a personal collection of beetles. References. Desmarest (1873); Jaussaud and Brygoo (2004: 111–112); Cambefort (2006: 136).

**1832–1836**. IV^e^ classe. Insectes. Pp. 64–395 *in*: *Expédition scientifique de Morée. Section des sciences physiques. Tome III. – 1.^re^ partie. Zoologie. Deuxième section. – Des animaux articulés. Par M. Brullé; les crustacés par M. Guérin.* F.G. Levrault, Paris [&] Strasbourg. [2] + 400 + [2 (errata)] pp. [+ 27 associated pls, numbered 27–53]. (4to–text; Folio–plates) CMLE (mf), MCZ, GB

The text for this section of the work was published in eight livraisons: **1–2**: (pp. 1–96) April–June 1832 (*Soc Ent Fr* [*Annales*, p. 243]), 5 September 1832 (*Soc Ent Fr*), 24 September 1832 (*Acad Sci Fr*); **3–4**: (pp. 97–192) July–September 1832 (*Soc Ent Fr* [*Annales*, p. 334]); **5–6**: (pp. 193–288) 9 January 1833 (*Soc Ent Fr*); **7**: (pp. 289–336) 20 March 1833 (*Soc Ent Fr*); **8**: (pp. 337–400 + [2]) 3 April 1833 (*Soc Ent Fr*). The whole volume was received on 18 March 1833 by the *Académie des Sciences*. The Coleoptera are on pages 114–274 (livraisons 3–6) and plates 33–44. Dates of publication of some of the plates are known (see Evenhuis 1997a: 137) and are indicated in Appendix 11 along with the list of species illustrated. Brullé is the author of the entire book except for pages 30–63 which deal with *crustacés*.

The publication dates provided by Evenhuis (1997a: 136) for the fifth and sixth livraisons could not be confirmed. No entries were found in the *Bibliographie de la France* for the year 1832 regarding livraisons of text; all those recorded concerned livraisons of plates.

The scientific expedition to Morea was linked to the French military mission to the Peloponnese from January 1829 to July 1833. Members of the mission reached Greece by March 1829 but by early 1830 most of them, exhausted by diseases and deprivation, had returned to France. Nevertheless, a large quantity of botanical and zoological specimens had been collected during these few months.

**1834–[1838**]. *Histoire naturelle des insectes, traitant de leur organisation et de leurs moeurs en général, par M. V. Audouin, et comprenant leur classification et la description des espèces, par M.A. Brullé. Le tout accompagné de planches gravées sur acier, d’après des peintures exécutées pour cette édition sur la collection du Muséum de Paris.* F.D. Pillot, Paris. (8vo) MCZ, GAL, GB

[*Tome IV. Coléoptères. I*]. viii + 479 pp. + 18 pls. [DP: 1834 (title page; *avertissement* dated October 1834)]. The text of this volume was issued in two livraisons: **1**: (pp. 1–240) 2 August 1834 (*Bibl Fr*), 9 August 1834 (*Feuil J Lib*); **2**: (pp. 241–479 + i–viii) February 1835 (*Bull Bibl*).

[*Tome V. Coléoptères. II*]. 436 pp. + 16 pls. [DP: 1835 (title page)]. The text of this volume was issued in two livraisons: **3** (pp. 1–224) and **4** (pp. 225–436) 11 February 1837 (*Bibl Fr*), 22 February 1837 (*Lit Ztg*), 24 February 1837 (*Allg Bibl Deutsch*).

[*Tome VI. Coléoptères. III*]. 448 pp. + pls 18, 19, 19*bis*, 20, 25. [DP: 1837 (title page)]. The text of this volume was issued in two livraisons: **7**: (pp. 1–224) 1837 (Brockhaus and Avenarius 1838: 57), 30 March 1839 (*Bibl Fr*), 12 April 1839 (*Allg Bibl Deutsch*), 17 April 1839 (*Lit Ztg*); **8**: (pp. 225–448) 1838 (Brockhaus and Avenarius 1839: 73).

The two livraisons for volume 5 have been recorded by the *Bibliographie de la France* only in 1837 suggesting that the dates on the title pages (e.g., 1835) could be incorrect. However the entire volume was noted by Burmeister (1836: 294) in his review of the entomological literature for 1835.

According to the prospectus (*Feuilleton du Journal de la Librairie* of 9 August 1834), the entire series was to comprise 12 volumes and 240 plates (either colored or uncolored) issued in 24 livraisons of text and 30 *cahiers* of plates. Only volumes 4–6 on Coleoptera and 9 dealing with Orthoptera and Hemiptera were published and all four were authored by Brullé alone.

**1836–1840**. Insectes. Pp. 53–95 *in*: *Histoire naturelle des Iles Canaries, par MM. P. Barker-Webb et Sabin Berthelot; ouvrage publié sous les auspices de M. Guizot, ministre de l’Instruction publique. Tome deuxième. Deuxième partie. Contenant la zoologie.* Béthune, Paris. 12 + 48 + 109 + [1] + 119 + 152 pp. [+ 52 associated pls]. (4to–text; Folio–plates) McG, BHL

The *zoologie* section of the *Histoire naturelle des Iles Canaries*, 1836–1851, was issued in several livraisons. The entomology part, entitled “*Animaux articulés recueillis aux Îles Canaries par MM. Webb et Berthelot et décrits par Messieurs: Brullé (pour les crustacés et la plus grande partie des insectes.) Lucas (pour les arachnides et les myriapodes.) Macquart (pour les insectes diptères.)*” and containing 119 pages and seven associated plates, was issued in the following livraisons: **livr. 13**: (pl. 2) 7 November 1836 (*Acad Sci Fr*); **livr. 28**: (pl. 5) 26 February 1838 (*Acad Sci Fr*), March 1838 (Stearn 1937: 55); **livr. 40**: (pp. 1–40) 24 June 1839 (*Acad Sci Fr*); **livr. 41**: (pp. 41–56 + pls 6–7) 12 August 1839 (*Acad Sci Fr*); **livr. 42**: (pp. 57–72 + pl. 4) 16 September 1839 (*Acad Sci Fr*); **livr. 43**: (pp. 73–88) 30 September 1839 (*Acad Sci Fr*), October 1839 (Stearn 1937: 55); **livr. 44**: (pp. 89–119) 28 October 1839 (*Acad Sci Fr*), November 1839 (Stearn 1937: 55); **livr. 50**: (pls 1, 3) 27 July 1840 (*Acad Sci Fr*). The title page of the book is dated 1836–1844. The collation is taken from Stearn (1937: 55).

The Coleoptera are on pages 55–74 and plates 1–2 of the “*Insectes*.” The Coleoptera species figured are: **Plate 1**: 1. *Melolontha fuscipennis* Br[ullé]; 2. *Melolontha obscura* Br.; 3. Cerambyx (Monochamus) annulicornis Br.; 4. Cerambyx (Monochamus) albidus Br.; 5. *Lamia gibba* Br.; 6. *Callidium roridum* Br.; 7. *Erodius curtus* Br.; 8. *Zophosis plicata* Br.; 9. *Hegeter glaber* Br.; 10. *Pimelia verrucosa* Br.; 11. *Pimelia lutaria* Br. (*lusaria* in the description on page 68); 12. *Pimelia levigata* Br.; 13. *Dytilus concolor* Br.; 14. *Tylodes scaber* Br.; 15. *Omias tessallatus* Br.; 16. *Mononyx variegatus* Br.; 17. *Hispa occator* Br. **Plate 2**: 1. *Calathus depressus* Br.; 2. *Carabus coarctatus* Br.; 3. *Carabus faustus* Br.; 4. *Feronia glabra* Br.; 5. *Chlaenius canariensis* Dej[ean]; 6. *Scarites dimidiatus* Br.; 7. *Nebria dilatata* Dej[ean]; 8. *Sphodrus alternans* Dej[ean]; 9. *Bembidion laetum* Br.; 10. *Silpha tenuicornis* Br.; 11. *Silpha costata* Br.; 12. *Buprestis bertheloti* Br. As such all taxa figured on plate 2 were made available by the illustrations issued on 7 November 1836 and those on plate 1 by the text issued from August to October 1839. The names *Silpha costata* Br. (pl. 2, fig. 11) and *Silpha tenuicornis* Br. (pl. 2, fig. 10) are changed to *Silpha figurata* Br. (No 59, p. 59) and *Silpha simplicicornis* Br. (No 60, p. 59) respectively in the text. *Buprestis bertheloti* Br. on plate 2 (fig. 12) is not described in the text.

**1836–1837**. Insectes de l’Amérique méridionale. Recueillis par Alcide d’Orbigny et décrits par Emile Blanchard et Auguste Brullé. Pp. 1–56 *in*: *Voyage dans l’Amérique méridionale (le Brésil, la République orientale de l’Uruguay, la République Argentine, la Patagonie, la République du Chili, la République de Bolivie, la République du Pérou), exécuté pendant les années 1826, 1827, 1828, 1829, 1830, 1831, 1832 et 1833 par Alcide d’Orbigny. Ouvrage dédié au Roi, et publié sous les auspices de M. le Ministre de l’Instruction publique (commencé sous le ministère de M. Guizot). Tome sixième. 2.e Partie: Insectes.* P. Bertrand, Paris [&] V. Levrault, Strasbourg. [4] + 222 pp. [+ 32 associated pls]. (4to–text; Folio–plates) CNC

This volume was issued in 32 livraisons, 1836–1847. The first 56 pages were authored by Brullé, and the remaining ones by Émile Blanchard [*q.v.*]. The Coleoptera are on pages 1–214 and plates 1–25. Pages 1–56 were issued in the following livraisons: **23**: (p. 1–16) 1836 (wrapper), 3 April 1837 (*Acad Sci Fr*), 7 April 1837 (*Soc Géog Fr*), 2 June 1837 (*Allg Bibl Deutsch*), January–June 1837 (*Verz Bücher Landkarten*); **25**: (p. 17–32) 1837 (wrapper), 19 June 1837 (*Acad Sci Fr*), 25 August 1837 (*Allg Bibl Deutsch*); **28**: (p. 33–56) 1837 (wrapper), October 1837 (Evenhuis 1997b: 573), 1 December 1837 (*Soc Géog Fr*). See under Blanchard 1841–1847 for the dates of publication of the plates.

#### Brünnich, Morten Thrane (Copenhagen, Denmark: 30 September 1737 – 19 September 1827: Copenhagen, Denmark). Danish zoologist and mineralogist; lecturer in natural history and economy at the University of Copenhagen (1765–1772); later, superintendent of silver mines in Norway. References. Erslew (1843: 241–243); Mearns and Mearns (1988: 93–96, P); Evenhuis (1997a: 137, P).

**1761**. *Prodromus insectologiae Siaellandicae, qvem dissertationis loco publico examini submittit Martinus Thrane Brünniche Hafnia-Danus, defendentis spartam ornante praestantissimo et doctissimo urbano Bruun Aascow, philosoph. baccalaur. et medic. studioso in auditorio Collegii Regii. Die 23. Aprilis MCCLXI.* [sic!] *H.ad.m.f.* Andreae Hartvig Godiche, Hafniae [= Copenhagen]. 31 pp. (8vo) [DP: 23 April 1761 (title page)] MDZ

This dissertation, defended by Bruun Aascow, consists of a list of insects, including notes for some, of the insects of the island of Zealand in Denmark. The Coleoptera are on pages 5–11.

**1764**. *Entomologia, sistens insectorum tabulas systematicas, cum introductione et iconibus. – Insektlaere, indeholdende insekternes systematiske tavler, samt indledning og figurer.* Andreas Hartvig Godiche, Kiøbenhavn [= Copenhagen]. [8] + 85 + [3] pp. + 1 pl. (8vo) [DP: 1764 (title page), 23 August 1764 (Evenhuis 1997a: 138), 1 December 1764 (*J Encycl*)] GB, BHL

The text is in Latin and Danish. The Coleoptera are on pages 44–56.

**1771**. *Zoologiae fundamenta praelectionibus academicis accommodata. Grunde i dyrelaeren.* Frider. Christ. Pelt, Hafniae et Lipsiae [= Copenhagen & Leipzig]. 253 + [1] pp. (8vo) [DP: 1772 (title page), 10 January 1772 (*Jena Ztg*), 29 August 1772 (*Gött Anz*)] BHL, GB

The text is in Latin and Danish. The Coleoptera are on pages 176–177, 186–193. The work includes what seems to be the first modern keys for identification of genera.

Copies of this work dated 1771 on their title pages are known (see Winther 1829: 6) and the Commission ruled that the book should in the future be attributed to the year 1771 (ICZN 1950a: 307).

#### Buchoz [Buc’hoz], Pierre Joseph (Metz, Moselle, France: 27 January 1731 – 30 January 1807: Paris, France). French lawyer, physician, botanist and compiler. References. Feller (1821: 120–121); Monfalcon (1821); Dezeimeris et al. (1831: 551–553).

**1775**. *Dictionnaire vétérinaire, et des animaux domestiques. Contenant leurs mœurs, leurs caracteres, leurs descriptions anatomiques, la maniere de les nourrir, de les élever, & de les gouverner, les alimens qui leur sont propres, les maladies auxquelles ils sont sujets, & leurs propriétés, tant pour la médecine & la nourriture de l’homme, que pour tous les différens usages de la société civile; auquel on a joint un Fauna Gallicus. Orne’ de 60 planches, grave’es en taille-douce. Tome sixieme.* Brunet, Paris. clxxiv + 506 pp. (8vo) [DP: 1775 (title page), July 1775 (*Merc Fr*)] GB

This volume is divided in two parts, the first one (pp. v–clxxiv) is the last section of the *Dictionnaire vétérinaire, et des animaux domestiques* and the second is the *Fauna Gallicus* where the animals of France are described. The Coleoptera are on pages 123–185.

The entire *Dictionnaire vétérinaire* was issued in six volumes, 1770–1775, first by J.-P. Costard and later by Brunet. A “Nouvelle édition” was issued in 1775 by Brunet.

#### Buckland, William (Axminster, Devon, United Kingdom: 12 March 1784 – 14 August 1856: Islip, Oxfordshire, United Kingdom). British geologist, palaeontologist and priest; lecturer in mineralogy at Corpus Christi College in Oxford; appointed dean of Westminster in 1845; first professor of geology and mineralogy at the University of Oxford; wrote the first full account of a fossil dinosaur, which he named *Megalosaurus*. References. Anonymous (1859c); Gordon (1894, P); Spencer (1997); Haile (2004, P).

**1836**. *Geology and mineralogy considered with reference to natural theology. Vol. II.* William Pickering, London. vii + 128 pp. + 87 pls. (8vo) [DP: 1836 (title page), 24 September 1836 (*Athenaeum*), September 1836 (Peddie and Waddington 1914: 82), 12 October 1836 (*Lit Ztg*), October 1836 (*British Critic*), 7 November 1836 (*Soc Géol Fr*), 12 November 1836 (*Lit Gaz*), December 1836 (*Dublin Univ Mag*)] GB

This work was published as part 6 of “The Bridgewater treatises on the power wisdom and goodness of God as manifested in the creation” and consists of two volumes, issued in 1836. It was reissued in 1837 by the same publisher (*n.v.*) and by Carey, Lea and Blanchard in Philadelphia [BHL, CAKL]. “A new edition. With supplementary notes” was issued by Lea & Blanchard, Philadelphia, in 1841 [CAKL]. “A new edition, with additions, by Professor Owen, Professor Phillips, Mr. Robert Brown and memoir of the author. Edited by Francis T. Buckland” was published by George Routledge & Co., London in 1858 [CAKL]. The work was reprinted in 1980 by Arno Press, New York as part of their “History of paleontology.” A German translation with notes and additions by Louis Agassiz was published in Bern, Chur and Leipzig, 1838 (vol. 2) –1839 (vol. 1), under the title “*Geologie und Mineralogie in Beziehung zur natürlichen Theologie*” [GB]. A French translation by L. Doyère was issued in Paris in two volumes under the title “*La géologie et la minéralogie dans leurs rapports avec la théologie naturelle.*”

Two new fossils, attributed to the family Curculionidae, are described and illustrated, *Curculioides ansticii* (p. 76; pl. 46”) and *Curculioides prestvicii* (p. 77; pl. 46”). However, these were subsequently shown to be arachnids, the first of the order Ricinulei and the second of the extinct order Trigonotarbida.

#### Burchell, William John (London, United Kingdom: 23 July 1781 – 23 March 1863: London, United Kingdom). British explorer, naturalist, artist, and author; appointed schoolmaster and superintendent of the botanical garden at the British island of Saint Helena (1805–1810); travelled to South Africa between 1810 and 1815 and to Brazil between 1825 and 1830 where he collected intensively; spent the last three decades of his life in England cataloguing his enormous collections; after his death by suicide, his insect collection went to Oxford University Museum. References. Chichester (1886); Poulton (1907, P); Papavero (1973: 221–229); Dickenson (2004b, P).

**1822**. *Travels in the interior of southern Africa. Volume I. With an entirely new map, and numerous engravings.* Longman, Hurst, Rees, Orme, and Brown, London. viii + [4] + 582 pp. + 10 pls. (4to) [DP: 1822 (title page; preface dated February 1822), 2 March 1822 (*Lit Gaz*), March 1822 (*Blackw Mag*; Peddie and Waddington 1914: 3), 1 April 1822 (*New Monthly Mag*), April 1822 (*European Mag*; *British Critic*), June 1822 (*Eclectic Rev*), September 1822 (*Monthly Rev*)] ANSP, GB

This work consists of two volumes, 1822–1823. A few new species of beetles, such as *Cetonia bachapinica* (p. 464), are described in footnotes in the first volume. The work was reprinted in 1953 by I. Schapera, London. A German translation was published in two volumes, 1822–1825, in Weimar under the title “*Reisen in das Innere von Süd-Afrika*” [GB]. An extended review of the book was published in *The Monthly Review or Literary Journal* 99 [1822]: 1–16, 143–151.

#### Burmeister, Hermann Carl Conrad (Stralsund, Mecklenburg-West Pomerania, Germany: 15 January 1807 – 2 May 1892: Buenos Aires, Argentina). German entomologist; professor at the University of Berlin (1834–1837) and later at the University of Halle; travelled to South America (1850–1852; 1856–1860); emigrated in 1862 to Argentina to become the founding director of the Museo Público de Buenos Aires, currently the Museo Argentino de Ciencias Naturales; most specimens of his collection prior to 1861 are in the Zoologisches Museum, Martin-Luther-Universität, in Halle, those after 1861 are in Museo Argentino de Ciencias Naturales in Buenos Aires. References. Ratzeburg (1874: 101–104); Berg (1895, P); Ulrich (1972, P); Schneider (2006, P); Bragg (2007a, P).

**1829**. *De insectorum systemate naturali. Dissertatio inauguralis quam consensu Facultatis Medicae Halensis ut doctoris utriusque medicinae gradum rite adipiscatur die IV. Novembris MDCCCXXIX publice defendet.* Grunertorum Patris filiique, Halis Saxonum [= Halle an der Saale]. 40 + [2 (*curriculum vitae*)] pp. (8vo) [DP: 4 November 1829 (title page)] CNC, GB, BHL, MDZ

Evenhuis (1997a: 141) mentioned that this work was also issued in 1829 as a separate version by Anton in Copenhagen (*n.v.*).

**1830**. *Lehrbuch der Naturgeschichte.* Anton und Gelbcke, Halle. x + 594 pp. (8vo) [DP: 1830 (title page), February 1830 (*Isis*, Heft II: [2]), 20 March 1830 (*Bibl Deutsch*), 18 May 1830 (*Intell Ztg Welt*), January–June 1830 (*Verz Bücher Landkarten*)] GB

The Coleoptera are on pages 417–430.

**1836**. *Handbuch der Naturgeschichte. Zum Gebrauch bei Vorlesungen. Zweite Abtheilung. Zoologie.* Theod. Chr. Friedr. Enslin, Berlin. xii + pp. 369–858. (8vo) [DP: 1837 (title page), 23 December 1836 (*Allg Bibl Deutsch*), January–June 1837 (*Verz Bücher Landkarten*)] GB

This work was issued in 1836 in two *Abtheilungen* continuously paginated; the first one pertains to mineralogy and botany. The Coleoptera are on pages 632–662 in the second *Abtheilung*.

**1837**. *Zur Naturgeschichte der Gattung Calandra, nesbt Beschreibung einer neuen Art*: Calandra sommeri. *Mit 1 Kupfertafel.* Burmeister und Stange, Berlin. 24 pp. + 1 pl. (4to) [DP: 1837 (title page), 28 April 1837 (*Allg Bibl Deutsch*), 3 May 1837 (*Lit Ztg*; *Soc Ent Fr*), 5 June 1837 (*Ent Soc Lond*), April–June 1837 (*Foreign Quart Rev*), January–June 1837 (*Verz Bücher Landkarten*)] CNC, GB

This publication was also issued in “*Programm des Real-Gymnasiums in Berlin*” (*n.v.*) and recorded on 29 March 1837 (*Lit Ztg*).

**1840–1846**. *Genera quaedam insectorum. Iconibus illustravit et descripsit. Volumen I. Continet tabulas XL.* A. Burmeister, Berolini [= Berlin]. viii + [2] + [120] pp. + 40 pls. (8vo) CNC

[*Heft 5*]. [DP: 1840 (Sherborn 1909: 72), 29 May 1840 (*Allg Bibl Deutsch*)]. This section includes the treatment of various genera of insects including the beetle genus *Acropis*.

[*Heft 6*]. [DP: 1840 (Sherborn 1909: 72), 9 October 1840 (*Allg Bibl Deutsch*), 15 October 1840 (*Intell Serapeum*), October–December 1840 (*Foreign Quart Rev*)]. This section includes the treatment of the beetle genera *Eudinopus* (2 pp. + 1 pl.), *Platygenia* (2 pp. + 1 pl.) and *Hypselogenia* (2 pp. + 1 pl.).

[*Heft 7*]. [DP: 1841 (Sherborn 1909: 72), 25 March 1842 (*Allg Bibl Deutsch*), April–June 1842 (*Foreign Quart Rev*), January–June 1842 (*Verz Bücher Landkarten*)]. This section includes the treatment of the “Familia Xylophila” (16 pp. + 2 pls), including the genera *Chalcosoma*, *Megalosoma*, *Xylotrupes* and *Augosoma*.

[*Heft 8*]. [DP: 1845 (Sherborn 1909: 72), 15 April 1845 (*Intell Serapeum*), 24 April 1845 (*Allg Bibl Deutsch*), 10 May 1845 (*Lit Ztg*), 16 May 1845 (*Leip Reper*), January–June 1845 (*Verz Bücher Landkarten*)]. This section includes the treatment of the beetle genera *Euchirus* (2 pp. + 1 pl.) and *Uloptera* (2 pp. + 1 pl. with the name spelled *Ulopterus* on the plate).

[*Heft 9*]. [DP: 1845 (Sherborn 1909: 72), July–December 1845 (*Verz Bücher Landkarten*), 12 February 1846 (*Allg Bibl Deutsch*), 3 April 1846 (*Leip Reper*)]. This section includes the treatment of the Cremastochilidae (8 pp. + 4 pls), including the genera *Cremastochilus*, *Psilocnemis*, *Cyclidius*, *Coenochilus*, *Trichoplus*, *Hoplostomus*, *Centrognathus*, *Rhagopteryx* and *Ptychophorus*.

[*Heft 10*]. [DP: 1846 (Sherborn 1909: 72; P[ost] S[criptum] dated July 1846), 26 November 1846 (*Allg Bibl Deutsch*), 18 December 1846 (*Leip Reper*), July–December 1846 (*Verz Bücher Landkarten*)]. This section includes the treatment of the Trichiadae (8 pp. + 4 pls) including the genera *Clastocnemis*, *Trigonopeltastes*, *Copris* and *Pelidnota*.

This entire work was published in 10 *Hefte*, 1838–1846, each consisting of one or one-and-a-half sheet of text, with unnumbered pages, and four plates (Hagen 1862: 107). The half-title page reads “*Genera insectorum. Vol. I.*” The title on each *Heft* is “*Genera insectorum. Iconibus illustravit et descripsit. Volumen I. Rhynchota*”. The Coleoptera are found in *Hefte* 5–10.

**1842–1855**. *Handbuch der Entomologie.* Theod. Chr. Friedr. Enslin, Berlin. (8vo) CNC, GB

*Dritter Band. Besondere Entomologie, Fortsetzung. ColeopteraLamellicornia Melitophila.* xxii + 828 + [1 (Drucksehler)] pp. [DP: 1842 (title page; *Vorrede* dated September 1842), 6 December 1842 (*Allg Bibl Deutsch*), 28 December 1842 (*Lit Ztg*), 18 January 1843 (*Soc Ent Fr*), January–June 1843 (*Verz Bücher Landkarten*)].

*Vierter Band. Besondere Entomologie, Fortsetzung. Erste Abtheilung. ColeopteraLamellicornia Anthobia et Phyllophaga systellochela.* xii + 587 + [1 (Drucksehler)] pp. [DP: 1844 (title page), 25 July 1844 (*Allg Bibl Deutsch*), 3 August 1844 (*Lit Ztg*), 16 August 1844 (*Leip Reper*), July–December 1844 (*Verz Bücher Landkarten*)].

*Vierter Band. Besondere Entomologie, Fortsetzung. Zweite Abtheilung. ColeopteraLamellicorniaPhyllophaga chaenochela.* x + 569 + [1 (Drucksehler)] pp. [DP: 1855 (title page; *Vorrede* dated 20 May 1855), 12 July 1855 (*Allg Bibl Deutsch*), 30 July 1855 (*Intell Serapeum*), July 1855 (*Lit Deutsch Natur Ztg*), July–September 1855 (*Cat Muquardt*), 28 December 1855 [JD] (*Soc Imp Nat Moscou*)].

*Fünfter Band. Besondere Entomologie, Fortsetzung. ColeopteraLamellicornia Xylophila et Pectinicornia.* viii + 584 pp. [DP: 1847 (title page; preface dated February 1847), 9 June 1847 (*Lit Ztg*), 17 June 1847 (*Allg Bibl Deutsch*), 30 June 1847 (*Intell Serapeum*), June 1847 (*Intell Allg Lit Ztg*), 16 July 1847 (*Leip Reper*), 16 August 1847 (*Publ Circ*), 17 August 1847 (*Ent Ver Stettin*), 16 October 1847 [JD] (*Soc Imp Nat Moscou*), 27 October 1847 (*Soc Ent Fr*), 3 January 1848 (*Ent Soc Lond*)].

The entire work consists of seven volumes, divided in five *Bande*, 1832–1855. The first three volumes (*Bande* 1 and 2 published 1832–1839) do not deal with Coleoptera.

**1861**. *Reise durch die La Plata-Staaten, mit besonderer Rücksicht auf die physische Beschaffenheit und den Culturzustand der Argentinischen Republik. Ausgeführt in den Jahren 1857, 1858, 1859 und 1860.* H.W. Schmidt, Halle. (8vo) CNC, GB

*Erster Band. Die südlichen Provinzen umfassend. Mit einer Charte und einem Titel-Bilde.* vi + 503 + [1] pp. [DP: 1861 (title page; *Vorrede* dated 4 February 1861), 16 May 1861 (*Allg Bibl Deutsch*), 31 May 1861 (*Intell Serapeum*)].

*Zweiter Band. Die nordwestlichen Provinzen und die Cordilleren zwischen Catamarca und Copiapo umfassend. Nebst einer systematischen Uebersicht der beobachteten Rückgratthiere. Mit einer Charte der bereisten Gegenden.* iv + 538 + [2] pp. [DP: 1861 (title page; *Vorrede* dated 10 July 1861), 15 August 1861 (*Allg Bibl Deutsch*), 15 September 1861 (*Intell Serapeum*)].

The Coleoptera are on pages 311–314 in the first volume; at least one new species is described in the second volume (*Amphionycha petronae*, p. 166).

#### Buysson, Henri Julien Charles du (Vernet, Allier, France: 4 June 1856 – 28 July 1927: Pergola, Vichy, France). French naturalist, specialist on the taxonomy of click beetles (Elateridae); studied law in Paris; his collection of Elateridae was bequeathed to the Muséum National d’Histoire Naturelle in Paris while the remaining of his collection was dispersed; his father was the botanist François du Buysson. References. Olivier (1927, P); Cambefort (2006: 160–161).

**[1892]–1906**. *Faune gallo-rhénane ou species des insectes qui habitent la France, la Belgique, la Hollande, le Luxembourg, la Prusse Rhénane, le Nassau et le Valais avec tableaux synoptiques et planches gravées. Coléoptères. Tome cinquième.* E. Adeline, Caen. 494 pp. (8vo) ARC

This volume, the fifth one of the *Faune gallo-rhénane*, covers the family Elateridae. It was issued in parts as supplements to *Revue d’Entomologie*. The dates of publication are listed at the bottom of the first page of each sheet (except the first one): (pp. 1–8) December 1892–January 1893; (pp. 9–16) February 1893; (pp. 17–32) March 1893; (pp. 33–40) May 1893; (pp. 41–48) September 1893; (pp. 49–64) October 1893; (pp. 65–72) November 1893; (pp. 73–80) February 1894; (pp. 81–96) March 1894; (pp. 97–112) April 1894; (pp. 113–128) May 1894; (pp. 129–136) June 1894; (pp. 137–144) July 1894; (pp. 145–152) February 1896; (pp. 153–160) March 1896; (pp. 161–176) June 1896; (pp. 177–208) September 1896; (pp. 209–216) October 1896; (pp. 217–224) November 1896; (pp. 225–240) January 1900; (pp. 241–256) February 1900; (pp. 257–264) March 1900; (pp. 265–280) January 1902; (pp. 281–312) May 1902; (pp. 313–336) June 1902; (pp. 337–368) September 1905; (pp. 369–384) October 1905; (pp. 385–400) November 1905; (pp. 401–416) December 1905; (pp. 417–448) January 1906; (pp. 449–464) February 1906; (pp. 465–494) March 1906. The title page is dated 1906 and the preface December 1892. The first three parts of the series were authored by Fauvel [*q.v.*] and the fourth one by Bourgeois [*q.v.*].

#### Cadolini, Luigi [Aloysius]. Italian physician; his collection, including the types, were in the hands of [Jos. de] Brambilla in Milan. Reference. Hagen (1862: 110).

**1830**. *Enumeratio Carabicorum Ticinensium sistens insecta hujus familiae in agro Ticinensi hucusque inventa. Dissertatio inauguralis quam annuentibus magnifico domino rectore illustrissimo facultatis directore spectabili d. decano ac clarissimis d.d. professoribus auspice J. Maria Zendrini pro doctoris medicinae laurea rite capessenda Aloysius Cadolini Mediolanensis. In celebratiss. i.r. archigymnasio Ticinensi publicae disquisitioni submittit mense Augusti MDCCCXXX in theses adnexas disputabitur in universitatis aedibus.* Bizzoni, Ticini [= Pavia]. 36 pp. (8vo) [DP: August 1830 (title page)] GB

This thesis consists of a list of the carabid species of Pavia, Italy, with habitat data. One new species, listed under the name “*Demetrias ruficeps*. Gené MS” (p. 9), is described; the taxon should be attributed to Carlo Giuseppe Gené [1800–1847].

Agassiz (1848: 400) listed a work entitled “*Enumeratio Caraborum Ticinensium*” published in 1830 by someone named “Brambilla.” There is no such work and the reference certainly pertains to Cadolini’s thesis. Hagen (1863: 303) listed this thesis under Cadolini and Giovanni Maria Zendrini [1783–1857].

#### Calwer, Carl Gustav (Stuttgart, Baden-Württemberg, Germany: 11 November 1821 – 19 August 1874: Stuttgart, Germany). German forester, ornithologist and entomologist, with a special interest in beetles. Reference. Schenkling (1933: 26–27).

**1854–1855**. *Die Thierwelt Deutschlands und der Schweiz. Mit 12 nach der Natur und den besten Hülfsmitteln gezeichneten, lithographirten und mit Farbendruck behandelten Tafeln von G.M. Kirn.* Chr. Belser, Stuttgart [&] William Radde, New York. iv + 596 pp. [+ 12 associated pls]. (8vo- text; Folio-plates) GB

This work was issued in six *Lieferungen*: **1**: (pp. 1–96 + 2 pls) 1854 (wrapper), 2 April 1854 (*Schwäb Merk*), 13 April 1854 (*Allg Bibl Deutsch*), 31 May 1854 (*Intell Serapeum*), January–June 1854 (*Bibliot Hist-Nat*); **2**: (pp. 97–176 + 2 pls) 1854 (wrapper), 2 November 1854 (*Allg Bibl Deutsch*), July–December 1854 (*Bibliot Hist-Nat*); **3**: (pp. 177–272 + 2pls) 1855 (wrapper), 15 March 1855 (*Allg Bibl Deutsch*); **4**: (pp. 273–352 + 2 pls) 1855 (wrapper), 22 July 1855 (*Schwäb Merk*), 2 August 1855 (*Allg Bibl Deutsch*); **5**: (pp. 353–432 + 2 pls) 1855 (wrapper), 27 September 1855 (*Allg Bibl Deutsch*); **6**: (pp. iv + 433–596 + 2 pls) 1855 (wrapper), 10 January 1856 (*Allg Bibl Deutsch*). The title page is dated 1856 and the *Vorrede* 8 December 1855. The Coleoptera are on pages 360–424.

**1857**. *Käferbuch. Allgemeine und specielle Naturgeschichte der Käfer Europa’s. Nebst der Anweisung sie zu sammeln und aufzubewahren. Mit 1 schwarzen und 48 colorirten Tafeln.* Krais & Hoffmann, Stuttgart. xxviii + 788 pp. + 49 pls. (8vo) [DP: 1858 (title page), 5 November 1857 (*Allg Bibl Deutsch*), December 1857 (*Allg Bibl*; *Lit Deutsch Natur Ztg*), July–December 1857 (*Bibliot Hist-Nat*), 2 March 1858 (*Acad Nat Sci Phila*)] SME, GB, MDZ

Five subsequent editions of this work were published: in 1869 by Calwer and Gustav Jäger (xvi + 626 pp. + 49 pls), in 1877 edited by G. Jäger (lx + 700 pp. + 50 pls), in 1884 edited by G. Jäger (lvi + 667 + [1] pp. + 50 pls), in 1893 by Wilhelm Gustav Stierlin (lix + [1] + 715 pp. + 50 pls), and in 1907–1916 by Camillo Schaufuss (lxxxviii + 1390 pp. + 51 pls). A Dutch edition was also issued in 1930 with 1330 pages and 50 plates.

#### Candèze, Ernest Charles Auguste (Liège, Belgium: 27 February 1827 – 2 July 1898: Liège, Belgium). Belgian physician, entomologist, and photographer; physician and director of a hospital for the mentally ill; best known for his work on the family Elateridae; a first collection of Elateridae (up to 1869) went to the Natural History Museum in London via F.D. Godman and O. Salvin, while a second collection of Elateridae and other families went to the Institut Royal des Sciences Naturelles de Belgique in Brussels. References. Ratzeburg (1874: 104–105); Lameere (1898, P); Fraipont (1899, P); Selys-Longchamps (1900, P); Essig (1931: 563–564, P).

**1857–1863**. *Monographie des élatérides.* H. Dessain, Liége. (8vo) CNC, GB

*Tome premier.* viii + 400 pp. + 7 pls. [DP: May 1857 (title page; preface dated April 1857), 29 June 1857 (*Acad Sci Fr*), July–December 1857 (*Bibliot Hist-Nat*)].

*Tome second.* 543 pp. + 7 pls. [DP: May 1859 (title page), October 1859 (*Allg Bibl*)].

*Tome troisième.* 512 pp. + 5 pls. [DP: July 1860 (title page)].

*Tome quatrième.* 534 pp. + 6 pls. [DP: May 1863 (title page), 7 September 1863 (*Ent Soc Lond*)].

This work was also issued as volumes 12 [1857], 14 [1859], 15 [1860], and 17 [1863] of the *Mémoires de la Société Royale des Sciences de Liège*.

**1881–1896**. *Élatérides nouveaux.* Hayez, Bruxelles. (8vo) CNC

*Troisième fascicule.* ii + 117 pp. [DP: 1881 (title page; *avant-propos* dated October 1881), 2 March 1882 (*Acad R Sci Belg*), 8 March 1882 (*Soc Ent Fr*), March 1882 (*Nat Nov*)].

*Cinquième fascicule.* 76 pp. [DP: 1893 (title page; *avant-propos* dated November 1892), 22 February 1893 (*Soc Ent Fr*), 28 February 1893 (*Soc Zool Fr*)].

*Sixième fascicule.* 88 pp. [DP: 1896 (title page; *avant-propos* dated December 1895), September 1896 (*Nat Nov*)].

These fascicles were also issued, with separate pagination, as part of the *Mémoires de la Société Royale des Sciences de Liège* (2e Série) 9: 1–117 [DP: 20 February 1882 (title page)], 18 (No 4): 1–76 [DP: July 1895 (title page)], 19: 1–88 [DP: January 1897 (title page)]. The entire work consists of seven fascicles. The first one was published in the *Mémoires de l’Académie de Belgique* 17 [1865]: 1–63; the second, fourth, and seventh ones in the *Annales de la Société Entomologique de Belgique* 21 [1878]: li–lxi, lxxv–lxxxv, cxxxv–cxliii, clxi–clxxii, clxxxix–cxcix; 33 [1889]: 67–123; and 44 [1900]: 77–101.

**1891**. *Catalogue méthodique des élatérides connus en 1890.* H. Vaillant-Carmanne, Liége. xii + 246 pp. (8vo) [DP: 1891 (title page; *avant-propos* dated December 1890), March 1891 (*Nat Nov*), August 1891 (*Bibl Belg*)] CNC, GB

A supplement containing additions to this catalogue was published by Ewald Bergroth in the *Annales de la Société Entomologique de Belgique* 35 [1891]: ccxxxiii–ccxxxvii.

#### Cardona y Orfila, Francisco (Mahón, Minorca Island, Spain: November 1833 – 17 January 1892: Mahón, Spain). Spanish clergyman and naturalist; professor of natural history and director of the Institute of Mahón; later director of the civic hospital. References. Anonymous (1892, P); Barber Barceló (1978, P).

**1872**. *Catálogo metódico de los Coleópteros de Menorca. Acompañado de numerosos datos y varias listas alfabéticas. O sea: guia del coleccionista entomólogo en dicha Balear.* Fábregues hermanos, Mahon. viii + 120 + [2] pp. (8vo) [DP: 1872 (title page; preliminaries dated 31 March 1872)] CSIC

This work is an annotated catalogue of the beetles of Minorca. Two supplements were published in 1875 [*q.v.*] and 1878 [*q.v.*].

**1875**. *Doscientos coleópteros mas de Menorca, por el autor del catalogo de estos insectos.* M. Parpal, Mahon. 23 pp. (8vo) [DP: 1875 (title page; preliminaries dated 1 January 1875)] CSIC

**1878**. *Otros cien coleópteros de Menorca por el autor del catálogo de estos insectos y del cuaderno doscientos coleópteros mas de Menorca.* M. Parpal, Mahon. 14 + [3] pp. (8vo) [DP: 1878 (title page; preliminaries dated 1 May 1878)] CSIC

#### Carpenter, William Benjamin (Exeter, Devon, United Kingdom: 29 October 1813 – 19 November 1885: London, United Kingdom). British physician, university administrator, invertebrate zoologist and physiologist; practiced medicine for a few years in Bristol before being appointed Fullerian professor of physiology at the Royal Institution in London in 1844; later, registrar of the University of London (1856–1879). References. Duncan (1872, P); Anonymous (1886a, P); Charton (2003: 32–33); Smith (2004, P); Murray (2012: 161, P).

**1849**. [Carpenter, W.B. and Westwood, J.O.] *The animal kingdom, arranged after its organization, forming a natural history of animals, and an introduction to comparative anatomy. By the late Baron Cuvier. Translated and adapted to the present state of science. Mammalia, birds, and reptiles, by Edward Blyth. The fishes and radiata, by Robert Mudie. The molluscous animals, by George Johnston. The articulated animals, by J.O. Westwood. A new edition, with additions by W.B. Carpenter and J.O. Westwood. Illustrated by three hundred engravings on wood and thirty-four on steel.* Wm. S. Orr and Co., London. vii + [4] + 718 + [2 (List of plates)] pp. + 34 pls. (4to) [DP: 1849 (title page), 1 February 1849 (*Monthly List Muquardt*), 10 February 1849 (*Athenaeum*), 15 February 1849 (*Publ Circ*), February 1849 (Low 1853: 86), 8 March 1849 (*Lit Ztg*)] GB, BHL

The Coleoptera are on pages 491–556 and 678–685. Separate printings of this edition (without change in pagination) were issued in 1851 [GB] and 1854 [GB, BHL]. Another edition of this translation was published in 1859 (*n.v.*) by Fullarton, London (Evenhuis 1997a: 180) and in 1863 [MCZ, BHL] by Henry G. Bohn, London, with pagination as xxii + 706 pages and 44 plates.

This work forms a new edition of “*Cuvier’s animal kingdom, arranged according to its organization; forming the basis for a natural history of animals, and an introduction to comparative anatomy. Mammalia, birds, and reptiles, by Edward Blyth. The fishes and radiata, by Robert Mudie. The molluscous animals, by George Johnston. The articulated animals, by J.O. Westwood. Illustrated by three hundred engravings on wood*” issued in 1840 by the same publisher with pagination as vii + 670 pages [GB, BHL]. It was noticed on 1 October 1840 (*Publ Circ*). Type species designations are listed for some generic names.

#### Casey, Thomas Lincoln (West Point, New York, USA: 19 February 1857 – 3 February 1925: Washington DC, USA). American military officer, engineer, entomologist, conchologist, and astronomer, well known for his controversial concept of species where little room was left for variation; graduated in 1879 from West Point Military Academy and until his retirement in 1912, at the age of 55 with the grade of Colonel, served in the Engineer Corps of the Army, working largely on river and harbor improvements; participated in 1882 to the astronomical expedition to Cape of Good Hope in South Africa; travelled to California and Texas in 1885–86, to Rhode Island in 1888, to New York in 1889–1893, to Virginia in 1895–1899, and to Missouri in 1902–1906; spent the last 18 years of his life in Washington DC; over 40 years published more than 8000 pages of beetle descriptions, without or with very few drawings; built an impressive collection of more than 19200 “species” and 117,000 specimens through collecting and purchases which was deposited at the National Museum of Natural History in Washington DC. References. Leng (1925); Schwarz and Mann (1925, P); Hatch (1926); Essig (1931: 565–567, P); Mallis (1971: 260–264); Smetana and Herman (2001: 53–54, P).

**1884**. *Revision of the Stenini of America north of Mexico. Insects of the family Staphylinidae, order Coleoptera.* Collins Printing House, Philadelphia. 206 pp. + 1 pl. (8vo) [DP: November 1884 (last page; preliminaries dated 10 August 1884), 8 December 1884 (*Amer Ent Soc*), 19 December 1884 (*Science*), 1 February 1885 (*Wien Ent Ztg* 4: 63), February 1885 (*Nat Nov*)] CNC, ARC

**1884–1885**. *Contributions to the descriptive and systematic coleopterology of North America.* Collins Printing House, Philadelphia. (8vo) CNC, BHL

*Part. I.* 60 pp. + 1 pl. [DP: August 1884 (bottom of several pages)].

*Part. II.* Pp. 61–198. This part was issued in two sections: **1**: (pp. 61–124) December 1884 (bottom of several pages); **2**: (pp. 125–198) January 1885 (bottom of several pages).

This work was received by the Academy of Natural Sciences in Philadelphia on 26 January 1885 and by the *Société Entomologique de France* on 25 March 1885.

#### Cederhjelm [Cederhielm], Johann [Johannes]. Russian naturalist.

**1798**. *Favnae Ingricae prodromvs exhibens methodicam descriptionem insectorvm agri Petropolensis praemissa mammalivm, avivm, amphibiorvm et piscivm envmeratione. Cum tabulis III. pictis.* Iohann. Fried. Hartknoch, Lipsiae [= Leipzig]. xviii + [2] + 348 pp. + 3 pls. (8vo) [DP: 1798 (title page; *praefatio* dated 20 November 1797), 18 July 1798 (*Allg Lit Ztg*), 22 October 1798 (*Mag Encycl*), 21 December 1798–19 January 1799 (*J Lit Fr*)] MCZ, GB, BHL

This work is essentially a list, with descriptions, of the insects (*sensu lato*) of Ingria, now a part of metropolitan Saint Petersburg. The Coleoptera are treated on pages 1–122 and 335–336 (additamentum). A few new species are described.

#### Champion, George Charles (London, United Kingdom: 29 April 1851 – 8 August 1927: Horsell, Surrey, United Kingdom). British entomologist; hired by Frederick Du Cane Godman and Osbert Salvin as collector and left England in 1879 for Guatemala, Panama, and Colombia; returned to his native country four years later where he served as secretary and chief assistant to Godman and Salvin, the editors of the *Biologia Centrali-Americana*; most of the specimens he collected are in the Natural History Museum in London. References. Calvert (1927); Walker (1927, P); Zimmerman (1993: 498, P); Smetana and Herman (2001: 54–55, P); Jackson (2004).

**1884–1893**. *Biologia Centrali-Americana. Insecta. Coleoptera. Vol. IV.* Taylor and Francis, London. (4to) CNC

*Part 1. Heteromera (part).* xxxiv + 572 pp. + 23 pls. This work was published in several parts, 1884–1893. The dates indicated at the bottom of the first page of each sheet are: (pp. 1–24) July 1884; (pp. 25– 56 + pl. 1) August 1884; (pp. 57– 72 + pl. 2) October 1884; (pp. 73– 88 + pl. 3) December 1884; (pp. 89– 96 + pl. 4) January 1885; (pp. 97–120 + pls 5, 6) July 1885; (pp. 121–136) October 1885; (pp. 137–152) April 1886; (pp. 153–168 + pl. 7) May 1886; (pp. 169–184) June 1886; (pp. 185–208) July 1886; (pp. 209–216 + pls 8, 9) August 1886; (pp. 217–224) October 1886; (pp. 225–240) November 1886; (pp. 241–264 + pl. 10) December 1886; (pp. 265–272 + pl. 11) January 1887; (pp. 273–296) June 1887; (pp. 297–320 + pl. 12) August 1887; (pl. 13) September 1887; (pp. 321–328) October 1887; (pp. 329–352 + pl. 14) December 1887; (pp. 353–368 + pl. 15) January 1888; (pp. 369–384) February 1888; (pl. 16) April 1888; (pp. 385–408 + pl. 17) August 1888; (pp. 409–424 + pl. 18) October 1888; (pp. 425–464 + pl. 19) November 1888; (pp. 465–476 + pl. 20) December 1888; (pl. 21) April 1889; (pp. 477–524) November 1892; (pp. 525–564 + pl. 22) January 1893; (pp. 565–572 + i–xxxiv + pl. 23) March 1893. The collation for the plates is from Lyal (2011: 83). The following families are treated in this volume: Tenebrionidae (pp. 1–384, 477–563), Cistelidae (pp. 385–465, 563–570), Othniidae (pp. 465–469), Nilionidae (pp. 470–472), and Monommidae (pp. 472–476, 570–572).

*Part 2. Heteromera (part).* x + 494 pp. + 21 pls. This work was published in several parts, 1889–1893. The dates listed at the bottom of some pages are: (pp. 1–32) April 1889; (pp. 33–72 + pls 1–3) May 1889; (pp. 73–96) July 1889; (pp. 97–104 + pl. 4) August 1889; (pp. 105–120 + pl. 5) December 1889; (pp. 121–160 + pls 6, 7) January 1890; (pp. 161–184) June 1890; (pp. 185–216 + pls 8, 9) August 1890; (pp. 217–248) October 1890; (pl. 10) November 1890; (pp. 249–256) December 1890; (pp. 257–304 + pl. 11) July 1891; (pls 12, 13) September 1891; (pp. 305–344) October 1891; (pp. 345–360 + pls 14, 15) November 1891; (pp. 361–368 + pl. 16) December 1891; (pp. 369–392) February 1892; (pl. 17) March 1892; (pp. 393–400 + pl. 18) May 1892; (pp. 401–448 + pl. 19) September 1892; (pl. 20) November 1892; (pp. 449–464 + pl. 21) March 1893; (pp. 465–494, i–x) April 1893. The collation for the plates is from Lyal (2011: 83). The volume covers the Lagriidae (pp. 1–74, 451–452), Melandryidae (pp. 75–103, 452), Pythidae (pp. 103–109), Oedemeridae (pp. 109–165, 453–455), Xylophilidae (pp. 166–190, 455–461), Anthicidae (pp. 190–250, 461–462), Mordellidae (pp. 250–350, 462), Rhipidophoridae (pp. 350–364), and Meloidae (pp. 364–450, 462–464).

**1893–1894**. Fam. Cassididae [pp. 125–232 + pls 5–13] Fam. Hispidae. Appendix [pp. 234–242] *in*: *Biologia Centrali-Americana. Insecta. Coleoptera. Vol. VI. Part 2. Phytophaga (part). Hispidae by J.S. Baly, with an appendix by G.C. Champion. Cassididae by C.G. Champion.* Taylor and Francis, London. x + 249 pp. + 13 pls. (4to) CNC

These sections were issued in seven parts as follows (see Lyal 2011: 88): (pp. 125–164) November 1893; (pp. 165–180 + pl. 5) January 1894; (pls 6, 7) February 1894; (pp. 181–232 + pls 8, 9) April 1894; (pp. 234–236) April 1894; (pp. 237–242) July 1894; (pls 10–13) July 1894.

**1894–1897**. Fam. Elateridae [pp. 258–556 + pls 11–24] Fam. Cebrionidae [pp. 557–573 + pl. 25] Fam. Rhipidoceridae [pp. 574–586 + pl. 24] Fam. Dascillidae [pp. 586–662 + pls 26–27]. Appendix. Fam. Throscidae [and] Fam. Eucnemidae [pp. 667–670] *in*: *Biologia Centrali-Americana. Insecta. Coleoptera. Vol. III. Part 1. Serricornia.* Taylor and Francis, London. xv + [1 (Errata et corrigenda)] + 690 pp. + 27 pls. (8vo) CNC

This work was published in parts. The dates listed at the bottom of the first page of each sheet are: (pp. 258–264) October 1894; (pp. 265–296) November 1894; (pp. 297–312 + pls 11, 12) January 1895; (pp. 313–336 + pl. 13) March 1895; (pp. 337–360 + pl. 14) May 1895; (pp. 361–376 + pls 15, 16) July 1895; (pp. 377–400 + pl. 17) October 1895; (pp. 401–440 + pls 18, 19) December 1895; (pp. 441–472 + pl. 20) January 1896; (pp. 473–496) March 1896; (pp. 497–528 + pls 21, 22) May 1896; (pp. 529–552 + pl. 23) June 1896; (pp. 553–584 + pl. 24) October 1896; (pl. 25) December 1896; (pp. 585–608 + pl. 26) February 1897; (pp. 609–624) March 1897; (pp. 625–656) May 1897; (pp. 657–662, 667–670 + pl. 27) August 1897. The collation for the plates is from Lyal (2011: 82).

#### Chapuis, Félicien (Verviers, Belgium: 29 April 1824 – 30 September 1879: Heusy near Verviers, Belgium). Belgian physician and entomologist, particularly interested in Coleoptera; practiced medicine in his native town; part of his collection (Chrysomelidae, Ipidae and Platypodidae) was acquired by the Institut Royal des Sciences Naturelles de Belgique in Brussels while the rest went to the Natural History Museum in London. References. Ratzeburg (1874: 111); Candèze (1880a, 1880b); Lameere (1956).

**1865**. *Monographie des platypides.* H. Dessain, Liége. vi + 7–344 pp. + 24 pls. (8vo) [DP: 1865 (title page; *préface* dated November 1864), 2 April 1866 (*Ent Soc Lond*), 7 April 1866 (*Acad R Sci Belg*)] CNC, BHL

This work was also issued as volume 20 of the *Mémoires de la Société Royale des Sciences de Liège* [DP: 1866 (title page), October–November 1866 (*Bibl Belg*)].

**1869**. *Synopsis des scolytides (prodrome d’un travail monographique).* J. Desoer, Liége. 61 pp. (8vo) [DP: 1869 (title page), 5 June 1869 (*Soc Ent Belg*), 1 July 1869 (*Pet Nouv Ent*), 14 July 1869 (*Naturfors Ver Brünn*), 12 October 1869 (*Amer Ent Soc*), 1 November 1869 (*Ent Soc Lond*)] CMLE, GB

This work was also published, under the title “*Synopsis des scolytides*,” in *Mémoires de la Société Royale des Sciences de Liège* (Deuxième Série) 3: 213–269 [DP: 1873 (title page), 7 June 1873 (*Soc Ent Belg*)].

**1874–1876**. *Histoire naturelle des insectes. Genera des coléoptères ou exposé méthodique et critique de tous les genres proposées jusqu’ici dans cet ordre d’insectes. Par Mm. Th. Lacordaire et F. Chapuis.* Roret, Paris. (8vo) CNC, SME, GB

*Tome dixième. Famille des phytophages.* iv + 455 pp. [DP: 1874 (title page; *avant-propos* dated February 1873), 1 July 1874 (*Pet Nouv Ent*), 12 September 1874 (*Bibl Fr*), October 1874 (*Polybiblion*)].

*Tome onzième. Famille des phytophages.* 420 pp. [DP: 1875 (title page), 15 September 1875 (*Pet Nouv Ent*), 16 October 1875 (*Bibl Fr*), 11 December 1875 (*Lit Centr Deutsch*), July–December 1875 (*Bibliot Hist-Nat*)].

*Tome douzième. Famille des érotyliens, des endomychides et des coccinellides.* 424 pp. [DP: 1876 (title page; *épilogue* dated March 1876), 1 May 1876 (*Pet Nouv Ent*), 17 June 1876 (*Bibl Fr*), 2 August 1876 (*Ent Soc Lond*), September 1876 (*Polybiblion*)].

The title on the wrappers is “*Collection des suites a Buffon formant avec les oeuvres de cet auteur un cours complet d’histoire naturelle publiées avec la collaboration de membres de l’Institut de France, de professeurs du Muséum d’Histoire naturelle de Paris, et de diverses facultés, de membres de la Société Entomologique de France, etc. Insectes Coléoptères.*” The first nine volumes of the *Histoire naturelle des insectes* were authored by Jean Théodore Lacordaire, 1854–1872 [*q.v.*].

#### Charpentier, Toussaint von [de] (Freiberg, Saxony, Germany: 22 November 1779 – 4 March 1847: Brieg [currently Brzeg], Poland). German geologist and entomologist; worked as mine inspector in Breslau and Dortmund; his main collection went to the Zoologisches Museum der Humboldt-Universität in Berlin. References. Anonymous (1903b); Weidner (1981: 119); Anonymous (2005b).

**1825**. *Horae entomologicae, adjectis tabulis novem coloratis.* A. Gosohorsky, Wratislaviae [=Wrocław]. xvi + 255 + [5 (Tabularum explicatio; Corrigenda)] pp. + 9 pls. (4to) [DP: 1825 (title page; preliminaries dated March 1825), 20 September 1825 (*Intell Ztg Welt*), December 1825 (*Allg Lit Ztg*), July–December 1825 (*Verz Bücher Landkarten*), February 1826 (*Not Geb Natur*), 29 April 1826 (*Gött Anz*)] CNC, GB, BHL

#### Chaudoir, Maximilien de (Baron) (Ivnitza, Ukraine: 12 September 1816 – 6 May 1881: Amélie-les-Bains-Palalda, Pyrénées-Orientales, France). Russian entomologist of independent means, who specialized early in life in the study of Carabidae; at 15, left for Dorpat (Tartu) to study law but never obtained his degree; made the sole extensive collecting trip of his life, a 40 day-journey to the Caucasus in company of M.H. Hochhuth in 1845; settled in his native town, Ivnitza, in Ukraine but moved in 1864 to Paris and then to Amélie-les-Bains-Palalda, France; made several trips to various cities in Europe to study type specimens in museums and private collections; his collection, certainly one of the best of his time (if not the best) on the family Carabidae, passed in the hands of René Oberthür after Chaudoir’s death and eventually ended up at the Muséum National d’Histoire Naturelle in Paris. References. Putzeys (1881); Sallé (1881); Ball and Erwin (1983, P); Basilewsky (1983); Cambefort (2006: 142–143).

**1846**. [Chaudoir, M. de and Hochhuth, M.H.] *Enumération des carabiques et hydrocanthares, recueillis pendant un voyage au Caucase et dans les provinces transcaucasiennes par le Baron M. de Chaudoir et le Baron A. de Gotsch. Carabiques. Par le Baron M. de Chaudoir. Hydrochanthares. Par M. H. Hochhuth.* J. Wallner, Kiew. 268 pp. (8vo) [DP: June 1846 (title page), 3 September 1846 (*Ent Ver Stettin*), 19 September 1846 [JD] (*Soc Imp Nat Moscou*), 27 January 1847 (*Lit Ztg*), 11 February 1847 (*Allg Bibl Deutsch*), 28 February 1847 (*Intell Serapeum*), 5 March 1847 (*Leip Reper*)] MCZ, GB, GAL

The carabiques are on pages 49–209 and the hydrocanthares on pages 213–225. Pages 1–46 form the *Introduction*, written by Chaudoir, pages 228–234 are entited “*Carabiques nouveaux de la Crimée*,” written by Chaudoir, and pages 235–268 is the “*Catalogue*.”

**1847**. *Observations*. Kieff. 13 pp. (8vo) [DP: February 1847 (last page), 17 August 1847 (*Ent Ver Stettin*), 18 September 1847 [JD] (*Soc Imp Nat Moscou*)] CNC

This opuscule includes taxonomic information on some of the carabid species described by Chaudoir in the *Bulletin de la Société Impériale des Naturalistes de Moscou* in 1843, 1844 and 1846. A few new species are described.

**1865**. *Catalogue de la collection de cicindélètes de M. le Baron de Chaudoir.* J. Nys, Bruxelles. 64 pp. (4to) [DP: March 1865 (title page), 16 June–31 August 1865 (*Leopoldina* 5: 70)] CNC

The appendix (pp. 49–64), entitled “*Description d’espèces nouvelles*,” includes the descriptions of several new species and genus-group taxa.

#### Chevrolat, Louis Alexandre August (Paris, France: 29 March 1799 – 16 December 1884: Paris, France). French public servant in the Ministry of Finance and coleopterist; founding member of the *Société Entomologique de France*; his collection, one of the richest of his time, was scattered during his lifetime and by his heirs. References. Reiche (1885); Zimmerman (1993: 500); Gouillard (2004: 39, P); Cambefort (2006: 143–145, P).

**1833–1836**. *Coléoptères du Mexique.* G. Silbermann, Strasbourg. (8vo) CNC, GAL

[*1er*] *Fascicule*. vii + [48] pp. [DP: 1834 (wrapper), 18 December 1833 (*Soc Ent Fr* [*Annales*, pp. lxxv, lxxviii]), 8 March 1834 (*Feuil J Lib*), 2 April 1834 (*Soc Ent Fr*), 26 April 1834 (*Bibl Fr*), 13 August 1834 (*Lit Ztg*)]. Most pages end with the notation “Janvier 1833” or “Juin 1833.” Contains the descriptions of 24 species and the *Introduction*.

[*2e*] *Fascicule.* [48] pp. [DP: 1834 (wrapper), 6 August 1834 (*Soc Ent Fr*), 16 August 1834 (*Feuil J Lib*), 27 September 1834 (*Bibl Fr*)]. Some pages end with the notation “Juin 1833” or “Mars 1834.” Contains the descriptions of 22 species.

*3e Fascicule*. [48] pp. [DP: 1835 (wrapper), 7 March 1835 (*Feuil J Lib*), 15 April 1835 (*Soc Ent Fr*)]. Most pages end with the notation “1834,” “Octobre 1834” or “Novembre 1834.” Contains the descriptions of 24 species.

*4e Fascicule.* [70] pp. [DP: 1835 (wrapper), 18 July 1835 (*Feuil J Lib*), 5 August 1835 (*Soc Ent Fr*)]. Most pages end with the notation “1834,” “Novembre 1834” or “Janvier 1835.” Contains the descriptions of 31 species, a “*Table de la première centurie des coléoptères du Mexique*”, and “*Errata et addenda pour la première centurie*.”

*5e Fascicule.* [48] pp. [DP: 1835 (wrapper), 10 October 1835 (*Feuil J Lib*)]. The *signature* on each species treated is dated “Janvier 1835,” except for no. 102 which has “2e fascicule. Mars 1834.” Contains the descriptions of 24 species, numbered 101–124.

*6e Fascicule.* [48] pp. [DP: 1835 (wrapper), 2 December 1835 (*Soc Ent Fr*), 9 January 1836 (*Feuil J Lib*)]. The *signature* on each species treated is dated “Juin 1835.” Contains the descriptions of 23 species, numbered 125–147.

*7e Fascicule.* [48] pp. [DP: 1835 (wrapper), 6 April 1836 (*Soc Ent Fr*)]. The *signature* on each species treated is dated “Juillet 1835.” Contains the descriptions of 23 species, numbered 148–170.

*8e Fascicule.* [65] pp. [DP: 1835 (wrapper), May 1836 (Guyot de Fère 1837: 102)]. The *signature* on each species treated is dated “Septembre 1835.” Contains the descriptions of 30 species, numbered 171–200, a “*Liste des espèces dans la deuxième centurie des coléoptères du Mexique, classées d’après l’arrangement du Catalogue de Dejean*” and “*Observations pour la deuxième centurie*.”

Despite the date on the wrappers, the last two fascicles were apparently published only in 1836.

The work was continued by the descriptions of more species in *Magasin de Zoologie* (Deuxième Série) 3 [1841]: pls 55–59 (+ 16 pp.) and pls 64–65 (+ 16 pp.) and 5 [1843]: pls 107–113 (+ 37 pp.).

**1838**. *Centurie de buprestides.* G. Silbermann, Strasbourg. 78 pp. (8vo) [DP: 1838 (title page), February 1839 (*Rev Zool*)] BHL (pp. 1–72 only)

The first 72 pages of this work were also issued in *Revue Entomologique* 5: 41–110 [DP: 21 November 1838 (Nagel and Schmidlin 2014: 97)]. Pages 73–78, entitled “*Du Necydalis major de Linné, Molorchus abbreviatus de Fabricius*,” were not issued previously and contained the original description of *Molorchus ulmi* Chevrolat (Nagel and Schmidlin 2014: 99; see also Guérin-Méneville 1839).

**1876**. *Mémoire sur la famille des clérites.* Lucien Buquet, Paris. 51 pp. (8vo) [DP: 15 March 1876 (title page), 13 May 1876 (*Bibl Fr*), January–June 1876 (*Bibliot Hist-Nat*), July 1876 (*Polybiblion*), 14 February 1877 (*Soc Ent Fr*)] CMLE

#### Cirillo [Cyrillo, Cyrillus], Dominicus Maria Leone (Grumo Nevano, Campania, Italy: 10 April 1739 – 29 October 1799: Naples, Campania, Italy). Italian naturalist and court physician; professor of botany and medicine at the University of Naples; publicly executed by Napoleon’s troops in Naples. References. D’Ayala (1870); Krauss (1920); Conci (1975: 879); Evenhuis (1997a: 184–185, P); D’Errico (2001, P).

**1787–1792**. *Entomologiae Neapolitanae specimen primum.* Neapoli. [1 (*praefatio*)] + [12] pp. + 12 pls. (Folio) GDZ

Hagen (1862: 153) and other bibliographers mentioned that this work contains 12 plates and eight pages. However, the copy on GDZ has 12 written pages, one for each plate, where the species illustrated are named and described. According to Krauss (1920: 33), the plates were issued in three parts as follows: **1**: (pls 1–4) 1787; **2**: (pls 5–8) 1791; **3**: (pls 9–12) 1792. The title page is dated 1787.

Only one new species of beetle, *Mylabris 12-punctata*, is described and illustrated on plate 5. All new species in this work have been dated 1787 in Sherborn’s *Index Animalium*.

The text pertaining to the new species described in this work was extracted by Meyer (1794: 270–282).

#### Clairville, Joseph Philippe de (southern France: 1742 – 31 July 1830: Winterthur near Zürich, Switzerland). Swiss entomologist and botanist of French origin; studied medicine and natural history at Montpellier; moved to Winterthur in 1782 but during the French occupation was forced to leave Switzerland and fled to Erlangen, Germany; co-founder of the *Schweizer naturforschenden Gesellschaft*; mainly interested in Odonata, Diptera, and Coleoptera; his collection of beetles is located at the Natural History Museum in Basel. References. Geilinger (1932); Evenhuis (2014b, P); Schaefer (2015).

**1798–1806**. *Entomologie helvétique ou catalogue des insectes de la Suisse rangés d’après une nouvelle méthode. Avec descriptions et figures.* / *Helvetische Entomologie oder Verzeichniss der schweizerischen Insekten nach einer neuen Methode geordnet. Mit Beschreibungen und Abbildungen.* Orell, Fussli et Compagnie, Zuric. (8vo) CMLE, GB

*Vol. I.* / *Erster Theil.* 149 + [4 (errata)] pp. + 16 pls. [DP: 1798 (title page), 14 November 1798 (*Allg Lit Ztg*), 20 April–19 May 1799 (*Soc Philom*)].

*Vol. II.* / *Zweiter Theil.* xliii + 247 + [4] pp. + 32 pls. [DP: 1806 (title page; *préface* dated August 1806), 19 April 1807 (*Allg Ztg*)].

These volumes were published anonymously and although some authors attributed the work to Johann Ulrich Schellenberg [*q.v.*], Clairville, as indicated on the title page of his 1811 work “*Manuel d’herborisation en Suisse et en Valais...*,” is the author. Méquignon (1940) pointed out that Clairville wrote the text and Johann Ulrich Schellenberg did the illustrations. This was confirmed by Kerzhner (1991: 118). The text is both in French (verso pages) and German (recto pages). Engelmann’s (1846: 477) entry suggests that separate French and German versions were also issued but this could not be corroborated. Latreille (1799, 1810) wrote a short review of the first volume and an extended one of the second volume.

#### Clark, Hamlet (Navenby, Lincolnshire, United Kingdom: 30 March 1823 – 10 June 1867: Rhyl, Wales, United Kingdom). British cleric and entomologist with an interest in Coleoptera; made collecting trips to Cape Verde Islands and Brazil among others; his collections of Hydradephaga, Phytophaga and Elateridae were purchased by the British Museum (Natural History) in London but his collections of British Coleoptera and Lepidoptera were sold at auction in 1865. References. Newman (1867); Staines (2002).

**1860**. *Catalogue of Halticidae in the collection of the British Museum. Physapodes and Oedipodes. Part I.* The Trustees, London. xii + 301 pp. + 10 pls. (8vo) [DP: 1860 (title page; preface dated 15 August 1860), 7 January 1861 (*Ent Soc Lond*), 12 January 1861 (Sherborn 1934: 310)] McG, GB

**1866**. *A catalogue of Phytophaga. [Coleoptera, Pseudotetramera]. Part I. I. Sagridae. II. Donacidae. III. Crioceridae. IV. Megalopidae. With an appendix, containing descriptions of new species, by H.W. Bates, and Rev. H. Clark.* Williams and Norgate, London & Edinburgh. 50 + 88 pp. (8vo) [DP: 1866 (title page; dedication dated October 1865), 14 April 1866 (*Athenaeum*), 4 June 1866 (*Ent Soc Lond*), June 1866 (*Ent Monthly Mag* 3: 23), September 1866 (*Ann Mag Nat Hist* (3)18: 248), July–December 1866 (*Bibliot Hist-Nat*)] AMNH, GB

The second section has a title page reading “Descriptions of new species of Phytophaga by H.W. Bates and Rev. H. Clark. Being the appendix to the catalogue of Phytophaga by the Rev. Hamlet Clark.” The page is dated October 1865.

#### Comolli, Antonio

**1837**. *De coleopteris novis ac rarioribus minusve cognitis provinciae Novocomi.* Fusi et Socii, Ticini [= Pavia]. [2] + pp. 7–54. (8vo) [DP: 1837 (title page), 6 November 1837 (*Ent Soc Lond*), 18 April 1838 (*Soc Ent Fr*)] CNC

This work was also issued the same year by the same publisher under the title “*De coleopteris novis ac rarioribus minusve cognitis provinciae Novocomi. Dissertatio inauguralis quam consensu magnifici domini rectoris, perillustris facultatis directoris, spectabilis decani nec non cl. D. D. professorum auspice Doct. Josepho Moretti botanicae professore publico ordinario. Una cum adnexis thesibus defendendis publicae disquisitioni offerebat in C. R. Archigymnasio Ticinensi an. 1837. Lauream medicam assecuturus Comelli Antonius ex Novocomo*” [GB]. It provides a list, with comments, of the beetles from the Province of Como in the Lombardy region of Italy. Several new species are described.

#### Companyo, Baudile-Jean-Louis (Céret, Pyrénées Orientales, France: 16 December 1781 – 10 September 1871: Perpignan, Pyrénées Orientales, France). French physician and naturalist; director of the Musée d’Histoire Naturelle of Perpignan from 1840 to 1871; wrote his major work, *Histoire naturelle du Département des Pyrénées-Orientales*, being over eighty years old. References. Fabre (1872); Capeille (1914: 135–136); Orousset (1996: 20–22).

**1862**. *Histoire naturelle du département des Pyrénées-Orientales*. *Tome troisième.* J.-B. Alzine, Perpignan. 942 pp. + 1 pl. [DP: 1863 (title page), 20 September 1862 (*Feuil J Lib*), September 1862 (*Allg Bibl*), 4 October 1862 (*Bibl Fr*)] McG, BHL, GAL

The entire work consists of three volumes, 1861–1864. The third one, which was published before the second volume, deals with the animal kingdom; the Coleoptera are on pages 521–828. Although dated 1863 on the title page, this tome was issued in 1862.

#### Comte, Achille Joseph (Grenoble, Isère, France: 29 September 1802 – 17 January 1866: Nantes, Loire-Atlantique, France). French physician and naturalist; professor of natural history at the Lycée Charlemagne in Paris; head clerk at the state education ministry; later, director of school in Nantes. References. Figuier (1867: 479–480); Larousse (1869a: 821); Knight (1872: 401).

**1832–1840**. *Règne animal disposé en tableaux méthodiques. Ouvrage adopté par le conseil royal de l’instruction publique pour l’enseignement des colléges et autres établissements de l’université.* Fortin-Masson et C^ie^, Paris. [2]^20^ pp. + 91 pls. (Folio) MCZ

This work consists of 91 plates, each corresponding to a livraison, with about 5000 illustrations depicting various genera and subgenera of animals. According to the prospectus, each plate depicts the type of a genus or subgenus in 77 orders of animals. The Coleoptera are on plates 74, 75, 78, 80, 82–88. Plate 83 was recorded on March 1840 in *Revue Zoologique* and plates 82–85 on 10 July 1841 in the *Bibliographie de la France*. All the species names of beetles are in vernacular form. Each plate is entitled “*Règne animal de Mr le Baron Cuvier, disposé en tableaux méthodiques.*”

The title cited by Engelmann (1846: 319) and Evenhuis (1997a: 152) is “*Règne animal de Cuvier, disposé en tableaux méthodiques, ouvrage adopté par le conseil royal de l’instruction publique, pour l’enseignement de l’histoire naturelle dans les établissements de l’université. Titre orné d’un portr. de Cuvier, et suivi d’un rapport fait à l’institut.*” This may have been the title on the wrappers.

#### Contarini, Nicolò Bertucci (Count) (Venice, Veneto, Italy: 26 September 1780 – 16 April 1849: Venice, Italy). Italian naturalist of independent means; had special interest in botany, ornithology and entomology; his collections, including the insects, and library were bequeathed to the Museo Corner of Venice. References. Venanzio (1850: 92–99); Conci (1975: 883).

**1832**. *Sopra il* Macronychus quadrituberculatus *del Müller.* Baseggio, Bassano. 24 pp. + 1 pl. (8vo) [DP: 1832 (title page), June 1833 (*Poligrafo*), 3 July 1833 (*Soc Ent Fr* [see p. xlv])] GB

Hagen (1862: 136) and Horn and Schenkling (1928a: 204) incorrectly dated this work 1822. A facsimile of Contarini’s booklet was published by Goidanich (1969).

**1843**. *Cataloghi degli uccelli e degli insetti delle provincie di Padova e Venezia.* Baseggio, Bassano. 42 pp. (4to) [DP: 1843 (title page), 26 November 1843 (*Ist Ven Sci*), November–December 1843 (*Bibliot Ital*)] GB

This is a catalogue of birds and insects of the provinces of Padova and Venezia in Italy. The Coleoptera are on pages 17–27. No new species-group of beetles are described in this work.

**1847**. Notizie sulla fauna terrestre e particolarmente sulla ornitologia del Veneto estuario con cenni sul passaggio degli uccelli e sulla caccia. Pp. 157–191 *in*: *Venezia e le sue lagune. Volume secondo.* Antonelli, Venezia. 594 pp. (8vo) [DP: 1847 (title page), 24 February 1848 (*Bibl Univers*)] GB

The entire work consists of two volumes issued simultaneously in four parts; it was published anonymously. The section on pages 157–191 is credited to Contarini because his name is listed after the description of the new species on pages 189–191. Three new species of beetles are described: *Lixus roseus* (p. 189), *Chrysomela santonici* (p. 189), and *Coccinella villosa* (p. 190).

#### Coquebert [de Montbret], Antoine Jean (Paris, France: 6 March 1753 – 6 April 1825: Paris, France). French statesman and naturalist; knight, lord of Montbret; junior official at the Court of Accounts; adviser to the Royal Court of Amiens and of Marie-Henriette Coquebert de Romain in Rheims. References. Larousse (1869b: 81); Saint-Allais (1874: 42–43).

**1804**. *Illustratio iconographica insectorum quae in Musaeis parisinis observavit et in lucem edidit Joh. Christ. Fabricius, praemissis ejusdem descriptionibus; accedunt species plurimae, vel minus aut nondum cognitae. Tabularum decas tertia.* Petri Didot, Parisiis. [4] + pp. 91–142 + pls 21–30. (4to) [DP: “Anno XII (1804)” (title page)] CNC, BHL

The entire work consists of three *decas*, 1799–1804. The plates were available in both colored and uncolored versions. The Coleoptera are on pages 123–134 and plates 28–30 of the third *deca*.

#### Coquerel, Jean Charles (Amsterdam, Netherlands: 2 December 1822 – 12 April 1867: Salasie, island of Réunion). French (Dutch-born) surgeon in the navy and entomologist; made several maritime campaigns in the Mediterranean and the Indian Ocean; collected in many places including Madagascar, the island of Réunion and Martinique; his collection of African insects went to the Muséum National d’Histoire Naturelle in Paris via Léon Fairmaire [*q.v.*]. References. Fairmaire (1868); Papavero (1971: 194–195); Gouillard (2004: 42).

**1865**. Liste des Coléoptères, Hémiptères et Diptères de Madagascar. Pp. 1–24 (Annexes C–E) *in*: *Voyage a Madagascar au couronnement de Radama II par Auguste Vinson.* Librairie encyclopédique de Roret, Paris. [3] + 575 + 7 (Table des matières) + 4 (Annexe A) + 6 (Annexe B) + 48 (Annexes C–F) pp. + 7 pls. (8vo) [DP: 1865 (title page), 23 September 1865 (*Bibl Fr*)] GB, GAL

The work was available with the plates colored or not.

#### Costa, Achille (Alessano, Puglia, Italy: 10 August 1823 – 17 November 1898: Rome, Italy). Italian entomologist and explorer; son of Oronzio Gabriele Costa [*q.v.*]; professor of zoology at the University of Naples and director of the Zoological Museum of the city; made collecting expeditions to southern Italy and Sardinia; his collection is at the Zoological Museum of the University of Naples Federico II. References. De Stefani (1899); Conci and Poggi (1996: 218, P); Pantaleoni (2012, P).

**1849–1859**. *Fauna del Regno di Napoli ossia enumerazione di tutti gli animali che abitano le diverse regioni di questo regno e le acque che le bagnano contenente la descrizione de’ nuovi o poco esattamente conosciuti con figure ricavate da originali viventi e dipinte al naturale di Oronzio-Gabriele Costa. Coleotteri.* Gaetano Sautto, Napoli. (4to) CNC

*Parte I.^a^ con XXIV tavole in rame.* xiii + 44 + 4 + 8 + 14 + 10 + 32 + 16 + 12 + 32 + 36 + 12 + 112 + 15 + [1] + 4 [Alcune addizioni e correzioni] + 12 [Indice] pp. This volume was published in 47 parts. The dates of publication were discussed by Sherborn (1937a: 42–43) and D’Erasmo (1949: 30–32) and some minor differences appear between the two authors. The dates found by Sherborn are given here. *Prefacione* (xiii pp.), November 1854; *Coleotteri pentameri. Famiglia degli Scarabeidei – Scarabaeidea* (44 pp.), as follows: (pp. 1–8) August 1853; (pp. 9–16) 2 September 1853; (pp. 17–24) 5 September 1853; (pp. 25–32) 7 September 1853; (pp. 33–40) 9 September 1853; (pp. 41–44) 18 September 1853; *Coleotteri pentameri. Famiglia degli Agestidei – Agestidea* (4 pp.), 5 November 1853; *Coleotteri pentameri. Famiglia degli Pachipidei – Pachypidea* (8 pp.), 11 October 1853; *Coleotteri pentameri. Famiglia de’ Geotrupidei – Geotrupidea* (14 pp.), as follows: (pp. 1–8) 1 December 1853; (pp. 9–14) 3 January 1854; *Coleotteri pentameri. Famiglia de’ Trogidei – Trogidea* (10 pp.), as follows: (pp. 1–2) 3 January 1854; (pp. 3–10) 18 January 1854; *Coleotteri pentameri. Famiglia de’ Cetoniidei – Cetoniidea* (32 pp.), as follows: (pp. 1–24) 1 October 1852; (pp. 25–32) 6 October 1852; *Coleotteri pentameri. Famiglia de’ Trichiidei – Trichiidea* (16 pp.), as follows: (pp. 1–8) 13 December 1852; (pp. 9–16) 14 December 1852; *Coleotteri pentameri. Famiglia de’ Lucanidei – Lucanidea* (12 pp.), as follows: (pp. 1–8) 2 October 1852; (pp. 9–12) 2 October 1853; *Coleotteri eteromeri. Famiglia de’ Mordellidei – Mordellidea* (32 pp.), as follows: (pp. 1–8) 1 December 1853; (pp. 9–16) 1854; (pp. 17–24) September 1854; (pp. 25–32) 10 December 1854; *Coleotteri eteromeri. Famiglia degli Eodemeridei – Oedemeridea* (36 pp.), as follows: (pp. 1–8) June 1852; (9–24) 1852; (pp. 25–32) 12 June 1852; (pp. 33–36) 13 June 1852; *Coleotteri eteromeri. Famiglia de’ Salpingidei – Salpingidea* (12 pp.), 1 July 1852; *Coleotteri trimeri. Famiglia de’ Coccinellidei – Coccinellidea* (112 pp.), as follows: (pp. 1–8) August 1849; (pp. 9–16) 5 September 1849; (pp. 17–24) 20 September 1849; (pp. 25–32) September 1849; (pp. 33–48) 21 September 1849; (pp. 49–56) 4 October 1849; (pp. 57–64) 12 October 1849; (pp. 65–72) 19 October 1849; (pp. 73–80) November 1849; (pp. 81–88) 7 November 1849; (pp. 89–96) 10 November 1849; (pp. 97–112) November 1849; *Coleotteri trimeri. Famiglia degli Endomichidei – Endomychidea* (15 + [1] pp.), as follows: (pp. 1–8) April 1850; (pp. 9–15 + [1]) 11 April 1850; *Alcune addizioni e correzioni* (4 pp.), probably November 1854; *Indice* (12 pp.), probably November 1854.

[*Parte 2.^a^*]. 12 + 40 + 68 + 12 + 24 pp. This volume was published in 21 parts. The dates of publication were discussed by Sherborn (1937a: 42–43) and D’Erasmo (1949: 32) and those noted by Sherborn are reported here. *Coleotteri pentameri. Famiglia de’ Cicindelidei; Cicindelidea* (12 pp.), 1 May 1857; *Coleotteri pentameri. Famiglia de’ Carabidei; Carabidea* (40 pp.), as follows: (pp. 1–16) 4 August 1857; (pp. 17–24) 1 September 1857; (pp. 25–32) 1 December 1857; (pp. 33–40) 15 December 1857; *Coleotteri tetrameri. Sezione de’ Longicorni* (68 pp.), as follows: (pp. 1–8) 1 August 1854; (pp. 9–16) 5 August 1854; (pp. 17–24) 10 August 1854; (pp. 25–32) 1 March 1855; (pp. 33–40) 12 March 1855; (pp. 41–48) 16 March 1855; (pp. 49–56) 1855; (pp. 57–64) September 1855; (pp. 65–68) 18 December 1855; *Coleotteri pentameri. Sezione dei Malacodermi. Famiglia de’ Licidei; Lycidea* (12 pp.), 1 August 1866; *Supplementi alle famiglie di Coleotteri* [unfinished] (24 pp.), as follows: (pp. 1–8) 2 October 1858; (pp. 9–16) 28 September 1859; (pp. 17–24) 1 October 1859.

The entire *Fauna del Regno di Napoli* was issued in sets of pages, 1829–1886 (Ruggiero 2002: 67), by Oronzio Gabriele Costa and his son Achille Costa. The entomology section was divided into six parts, each corresponding to an insect order. The parts dealing with Lepidoptera was written by O.G. Costa, those dealing with Coleoptera, Neuroptera and Hymenoptera by A. Costa, and those dealing with Orthoptera and Hemiptera by both O.G. and A. Costa (D’Erasmo 1949).

**1858**. *Ricerche entomologiche sopra i monti Partenii nel Principato Ulteriore. Con una tavola in rame.* Stamperia e Calcografia, Napoli. 29 + [1 (Spiegazione della tavola)] pp. + 1 pl. (8vo) [DP: 1858 (title page; preface dated 15 December 1857), 14 March 1859 (*Ist Ven Sci*)] CNC

Several new species of beetles are described in this work from mount Partenio in the Italian region of Campania.

#### Costa, Giuseppe. Italian naturalist and professor; son of Oronzio Gabriele Costa [*q.v.*].

**1871–1874**. *Fauna Salentina ossia enumerazione di tutti gli animali che trovansi nelle diverse contrade della provincia di Terra d’Otranto e nelle acque de’ due mari che la bagnano contenente la descrizione de’ nuovi o poco esattamente conosciuti.* Editrice Salentina, Lecce. vii + pp. 9–624. (16mo) GDZ

[*Vol. I*]. vii + pp. 9–336. [DP: 1871 (title page)].

[*Vol. II*]. Pp. 337–624. [DP: 1874 (title page), 31 March 1874 (*Bibl Ital-2*), May 1874 (*Allg Bibl*; *Polybiblion*), January–June 1874 (*Bibliot Hist-Nat*)].

These volumes pertain to the Terra d’Otranto fauna in the Salentina Peninsula, southern Italy. They form tomes 20 and 21 of “*Collana di opere scelte edite ed inedite di scrittori di Terra d’Otranto*” directed by Salvatore Grande. The Coleoptera are on pages 153–458.

#### Costa, Oronzio Gabriele (Alessano, Puglia, Italy: 26 August 1787 – 7 November 1867: Naples, Campania, Italy). Italian physician, naturalist and paleontologist; professor of natural history at the University of Naples (1836–1849) and director of the Zoological Museum of the city (1839–1849); founded in 1841 the *Accademia degli Aspiranti Naturalisti*, a school educating young volunteers to become naturalists. References. Salfi (1956: 93–95); Conci (1975: 887–888); Conci and Poggi (1996: 218).

[**1835**]. *Cenni zoologici ossia descrizione sommaria delle specie nuove di animali discoperti in diverse contrade del Regno nell’anno 1834. Con illustrazioni sopra talune altre meno ovvie.* Azzolino e Comp., Napoli. xii + pp. 13–90 (8vo) [DP: 1834 (title page; dedication dated 30 December 1834)] GB

A list of Coleoptera are included on pages 69–71 and 80 (Addenda) under a section entitled “*Enumeratio insectorum rariorum quae per apuliam, agrum puteolanum, insulas prochitae et pithecusiae, ac in monte Vesevo Perlegi, anno 1834.*” New species are named but not described. Considering the date of the dedication, this book was probably issued in 1835 despite the 1834 date on the title page.

**1839**. *Corrispondenza zoologica destinata a diffondere nel Regno delle Due Sicilie tutto ciò che si va discuoprendo entro e fuori Europa (e vice-versa) risguardante la zoologia in generale. Anno I.* Azzolino e Compagno, Napoli. 194 pp. + 12 pls. (8vo) [DP: 1839 (title page), March–April 1839 (*Ann Civ Reg Sicile* 19: 152)] CNC (mf)

A few new beetle species are described in this work, including *Carabus variolatus* (p. 6), *Carabus dragonetti* (p. 8), *Cicindela sicula* (p. 40), *Hylesinus prestae* (p. 101), *Bagous oleae* (p. 103), and *Coccinella olivetorum* (p. 104).

#### Cox, Herbert Edward (London, United Kingdom: early 1838? – 10 December 1914: Jamaica). British entomologist; lived for many years in London but moved to Jamaica where he held a position in the local government; a large part of his collection was presented to the Hope Museum of the University of Oxford by his widow. Reference. MacKechnie-Jarvis (1976: 96–97).

**1874**. *A handbook of the Coleoptera or beetles, of Great Britain and Ireland.* E.W. Janson, London. (8vo) MCZ, BHL

*Vol. I.* [1] + viii + 527 pp. [DP: 1874 (title page; preface dated November 1874), 7 December 1874 (*Ent Soc Lond*)].

*Vol. II.* 366 pp. [DP: 1874 (title page), 7 December 1874 (*Ent Soc Lond*)].

#### Creutzer, Christian. Secretary of Field Marshal Franz Moritz Grafen von Lacy in Vienna; his collection was sold, after his death, to Franz Gerl in 1827. Reference. Fitzinger (1868: 1089).

**1799**. *Entomologische Versuche. Mit drey ausgemahlten Kupfertafeln von Herrn Sturm.* Karl Schaumburg und Comp., Wien. 142 + [10] pp. + 3 pls. (8vo) [DP: 1799 (title page; *Wiederruf und Abbitte* at the end of the book dated 18 September 1799), 16 October 1799 (*Intell Allg Lit Ztg*)] MCZ, GB

The entire work pertains to Coleoptera except for a small section related to *Gryllus* (p. 129–131). Several new species are described.

#### Cristofori, Giuseppe de (Milan, Lombardy, Italy: 11 October 1803 – 27 December 1837: Milan, Italy). Italian naturalist of independent means; his collections, merged with those of Giorgio Jan in 1832, formed the basis of the Museo Civico di Storia Naturali of Milan; visited Russia, Finland, Norway, and Sweden in 1836 where he collected for the museum. References. Villa (1838); Longhena (1838); Conci (1975: 894–895).

**1832**. [Cristofori, G. de and Jan, G.] *Cataloghi sistematici e descrittivi degli oggetti di storia naturale esistenti nel museo di Giuseppe De Cristofori e Prof. Gioegio Jan contenenti il prodromo della fauna, della flora e della descrizione orittognostico-geognostica dell’Italia superiore. Sectio III.^a^ entomologia. Pars I.^a^ conspectus methodicus insectorum. Fasc. I.^us^Coleoptera.* Giovanni Pirotta, Milano. 3 + 111 + [2] + 16 pp. (8vo) [DP: 1832 (title page), April–June 1832 (*Bibliot Ital*), 7 November 1832 (*Soc Ent Fr*)] MCZ

#### Crotch, George Robert (Somerset, United Kingdom: 1842 – 16 June 1874: Philadelphia, Pennsylvania, USA). British entomologist; studied at Cambridge University; collected in various places in Europe; came to America in 1872 and collected in British Columbia, Oregon and California in 1873; worked as assistant to Hermann August Hagen at the Museum of Comparative Zoology; his collections of Erotylidae, Coccinellidae and European Coleoptera were bequeathed to the Museum of Comparative Zoology in Cambridge, Massachusetts. References. Newman (1874a); Sharp (1874); Edwards (1875); Essig (1931: 598–600, P); Smart and Wager (1977).

**1863**. *A catalogue of British Coleoptera.* Cambridge. 45 pp. (8vo) [DP: 1863 (title page; preliminaries dated July 1863), 2 November 1863 (*Ent Soc Lond*)] CNC, GB

[**1866**]. *Catalogue of British Coleoptera. Second edition.* [Cambridge]. [1] + 17 + [1] pp. (8vo) [DP: n.d. (title page)] CMLE

There is no date on the title page. Harting (1884: 71), Woodward (1903: 403) and Derksen and Scheiding (1963: 549) listed it as published in 1871. However, the *Société Entomologique de France* received the catalogue during the period including the year 1866 and the first three months of 1867 (*Soc Ent Fr* [*Annales* (4) 6: lxxvi]) and the *Zoological Record* recorded it in the year 1866.

**1870**. Coleoptera. Pp. 45–99 *in*: *Natural history of the Azores, or Western Islands. By Frederick Du Cane Godman.* John van Voorst, London. v + [1] + 358 pp. (8vo) [DP: 1870 (title page; preface dated September 1870), 29 October 1870 (*Athenaeum*), 1 November 1870 (*Publ Circ*), 24 December 1870 (*Spectator*), 29 December 1870 (*Nature*)] GB

As mentioned on page 45 [footnote] this section is a “reprint, with alterations,” of a paper published by the author in the *Proceedings of the Zoological Society of London for the year 1867* (pp. 359–391 + pl. 23). However there are nomenclatural acts, such as new synonyms, proposed in the 1870 version.

**1871**. *Synopsis coleopterorum Europae et confinium anno 1868 descriptorum.* Williams and Norgate, London & Edinburgh. [1] + 68 pp. (8vo) [DP: 1871 (title page; preface dated 1 September 1870), 11 February 1871 (*Athenaeum*), 1 March 1871 (*Pet Nouv Ent*), 6 March 1871 (*Ent Soc Lond*), March 1871 (*Ent Monthly Mag* 7: 236), 1 April 1871 (*Publ Circ*), 13 May 1871 (*Lit Centr Deutsch*), January–June 1871 (*Bibliot Hist-Nat*)] NCSU

This work consists mainly of a notification of all species of Coleoptera described in the year 1868 with reproduction or abridgment of the diagnoses.

**1871**. *List of Coccinellidae.* Cambridge. 8 pp. (8vo) [DP: 27 April 1871 (last page), 3 June 1871 (*Soc Ent Belg*)] MCZ

**1871**. *List of all the Coleoptera described A.D. 1758–1821 referred to their modern genera*. Cambridge. [1] + 24 pp. (8vo) [DP: 1871 (title page; preface dated 15 August 1871)] CNC

This booklet covers only the families Cicindelidae, Carabidae, and Dytiscidae.

[**1872**]. Part 2. List of the Coleoptera found during the progress of the survey. Pp. 263–268 *in*: *Ordnance survey of the Peninsula of Sinai. Made with the sanction of the Right Hon: Sir John Pakincton, Bart: secretary of state for war, by Captains C. W. Wilson, and H. S. Palmer. Under the direction of Colonel Sir Henry James, R.E: F.R.S. &c. director-general of the ordnance survey. [Part I].* Lords Commissioners of Her Majesty’s Treasury, Southampton. iv + 323 pp. + 20 pls. (Folio) [DP: 1869 (title page; preface dated 31 October 1871), 23 April 1872 (Alonso-Zarazaga and Lyal 1999: 219), 25 May 1872 (*Sat Rev*), 17 August 1872 (*Athenaeum*)] HOU

The second page is entitled “*Ordnance survey of the Peninsula of Sinai. In three parts. Part I. Account of the survey (with illustrations). Part II. Maps, plans, and sections. Part III. Photographic views (in 3 vols.). Vol. I., Suez to Mount Sinai (Jebel Músá). Vol. II., Wády Feirán and Mount Serbál. Vol. III., Sinaitic and Egyptian inscriptions.*”

The official account of the survey, made in 1868–1869 by a team of Royal Engineers under the leadership of Captain Charles Wilson, was published in five volumes. Three volumes of photographs were published in 1869 and the volume of text with illustrations and that of maps were issued more than two years later.

The species described by Crotch in this work are: *Saprinus sinaiticus* (p. 266), *Pachydema sinaitica* (p. 266), *Pachydema israelitica* (p. 266), *Pachydema nocturna* (p. 266), *Pachydema ? sancta* (p. 267), *Erodius sinaiticus* (p. 267), *Erodius ecostatus* (p. 267), *Adesmia drakii* (p. 267), *Adesmia sinaitica* (p. 267), *Tentyria palmeri* (p. 267), *Sepidium korah* (p. 268), *Sepidium dathan* (p. 268), *Sepidium abiram* (p. 268), *Tychius sinaiticus* (p. 268) and *Chrysomela sanguineo-cincta* (p. 268).

**1874**. *Check list of the Coleoptera of America, north of Mexico.* Naturalists’ Agency, Salem. [1] + 136 pp. (8vo) [DP: 1873 (title page; preface dated 1 June 1873), 1874 (cover), May 1874 (*Amer Nat* 8: 303), 1 September 1874 (*Pet Nouv Ent*), 9 October 1874 (*Psyche* 1: 24)] CNC, BHL

The book was reissued in 1880 [BHL], along with the supplement of Austin [*q.v.*], by the same publisher.

**1874**. *A revision of the coleopterous family Coccinellidae.* E.W. Janson, London. xv + [1] + 311 pp. (8vo) [DP: 1874 (title page; letter in the preface dated 1 September 1874)] CNC, GB

This posthumous work was edited by Edward W. Janson.

#### Cuní y Martorell, Miguel (Calella, Catalonia, Spain: 1827 – 14 May 1902: Barcelona, Spain). Spanish merchant and entomologist. References. Longinos Navás (1902); Vives Noguera (2011: 53–54).

**1876**. [Cuní y Martorell, M. and Martorell y Peña, M.] *Catálogo metódico y razonado de los coleópteros observados en Cataluña.* Tomas Gorchs y Comp., Barcelona. viii + 360 pp. (8vo) [DP: 1876 (title page), 5 April 1876 (*Soc Espa Hist Nat*)] NCSU, GB, BDH

This work is an annotated catalogue of the Coleoptera of Catalonia, in northeastern Spain.

#### Curtis, John (Norwich, Norfolk, United Kingdom: 3 September 1791 – 6 October 1862: London, United Kingdom). British naturalist and illustrator who made his living mostly from his paintings and publications; from 1840 took an active interest in economic entomology and published many articles in the *Gardener’s Chronicle*, under the pseudonym Ruricola, and in the *Journal of the Royal Agricultural Society of England*; his British Coleoptera were purchased from his widow by the Museum of Victoria in Abbotsford, Australia, while his collections of Cerambycidae, Elateridae and Melasidae are at the Natural History Museum in London. References. Smith (1862: 122–125); Westwood (1863); Smetana and Herman (2001: 56–58, P); Hooper (2004); Gilbert (2005: 37, P).

**1824–1839**. *British entomology; being illustrations and descriptions of the genera of insects found in Great Britain and Ireland: containing coloured figures from nature of the most rare and beautiful species, and in many instances of the plants upon which they are found.* London. 769 plates + text. (4to) CNC

This work was published in 192 parts. Each part contained five (later four) plates with two unnumbered pages of text for each plate. Type species are indicated for all genera depicted in the work, several of them being the first valid ones. Two title pages were issued, one dated 1824–39 for a numeric arrangement in 16 volumes, the second dated 1823–40, for a systematic arrangement in eight volumes.

The dates of publication of each volume are as follows: *Vol. I*: (pls 1–50) 1824; *Vol. II*: (pls 51–98) 1825; *Vol. III*: (pls 99–146) 1826; *Vol. IV*: (pls 147–194) 1827; *Vol. V*: (pls 195–241 + 205*) 1828; *Vol. VI*: (pls 242–289) 1829; *Vol. VII*: (pls 290–337) 1830; *Vol. VIII*: (pls 338–385) 1831; *Vol. IX*: (pls 386–433) 1832; *Vol. X*: (pls 434–481) 1833; *Vol. XI*: (pls 482–529) 1834; *Vol. XII*: (pls 530–577) 1835; *Vol. XIII*: (pls 578–625) 1836; *Vol. XIV*: (pls 626–673) 1837; *Vol. XV*: (pls 674–721) 1838; *Vol. XVI*: (pls 722–769) 1839. Specific dates are indicated on the plates themselves. A list of Coleoptera depicted in this work is included in Appendix 3 along with the type species listed for each genus.

**1829–1840**. *British entomology; being illustrations and descriptions of the genera of insects found in Great Britain and Ireland: containing coloured figures from nature of the most rare and beautiful species, and in many instances of the plants upon which they are found.* [Second edition]. London. 122 plates + text (4to) (*n.v.*).

This work was published in 30 parts as follows (see Blackwelder 1947: 3 and Sherborn and Durrant 1911: 85): **1**: (pls 1–5) 1 January 1829; **2**: (pls 6–10) 1830 (after July); **3**: (pls 11–14) March 1834; **4**: (pls 15–18) 1835; **5**: (pls 19–22) 1835; **6**: (pls 23–26) 1839; **7**: (pls 27–30) 1840; **8**: (pls 31–34) 1840; **9–30**: (pls 35–122) 1840. This second edition had no new plates relating to Coleoptera but the text for some plates in the first eight parts were rewritten and often enlarged, particularly the text for plates 1, 6, 11, 15, 19, 23, 27, and 31 (see Blackwelder 1947: 3–4). The plates and text of parts 9–30 are simply reprints of the original edition. The nomenclatural changes between the two editions as related to Coleoptera is that the generic names on plates 19 (*Rhipiphorus*) and 23 (*Siagonum*) are emended in the second edition and the species depicted on plate 11 is changed from *Molorchus minor* to *Necydalis minor* (see Blackwelder 1947: 3–4). A reprint of the second edition was issued in 1862 (Sherborn and Durrant 1911: 85).

**1829–1831**. *A guide to an arrangement of British insects; being a catalogue of all the named species hitherto discovered in Great Britain and Ireland.* Richard Taylor, London. vi pp. + 256 columns (8vo) CMLE

This work was published in eight parts, each consisting of 16 pages with two columns per page, as follows (Sherborn 1922a: xl): **1**: (pp. i–vi, cols 1–32) 1829; **2–4**: (cols 33–128) 1829–1830; **5**: (cols 129–160) 1830; **6**: (cols 161–192) 1831; **7**: (cols 193–224) 1831; **8**: (cols 225–256) 1831. The preface is dated March 1829. Evenhuis (1997a: 167) recorded columns 1–128 as being published on 1 June 1829 based on Stephens’ (1846: iv) comment. However, Stephens remarked that “the first 20 columns only of the Guide…were published on the 1st of June, 1829.”

Curtis’ work was also issued printed on one side for the purpose of labelling cabinets. Some of the undescribed genera and species listed in this work were described in *The Entomological Magazine* 1 [1833]: 186–199. A second edition was published in 1837 [*q.v.*].

**1835**. Descriptions, &c. of the insects brought home by Commander James Clark Ross, R.N., F.R.S., &c. Pp. lix–lxxx + 1 pl. *in*: *Appendix to the narrative of a second voyage in search of a North-West passage, and of a residence in the Arctic regions during the years 1829, 1830, 1831, 1832, 1833. By Sir John Ross, C.B., K.S.A., K.C.S. &c. &c. Including the reports of Commander, now Captain, James Clark Ross, R.N., F.R.S., F.L.S., &c. and the discovery of the northern magnetic pole.* A.W. Webster, London. xii + 120 + [i]–cxliv + [i]–cii + [1 (Omissions. Errata)] pp. (4to) [DP: 1835 (title page), 21 November 1835 (*Athenaeum*), 28 November 1835 (*Lit Gaz*), November 1835 (Peddie and Waddington 1914: 503), 11 December 1835 (*R Astr Soc*), 21 December 1835 (*Acad Sci Fr*)] CNC, BHL

The only Coleoptera mentioned in this work is *Colymbetes moestus* (p. lx), a new species.

**1837**. *A guide to an arrangement of British insects; being a catalogue of all the named species hitherto discovered in Great Britain and Ireland. Second edition, greatly enlarged.* J. Pigot and Co., London. vi pp. + 294 columns. (8vo) [DP: 1837 (title page; preface dated June 1837), August 1837 (*Phil Mag*)] CNC, GB, ARC, BHL

A statement in the preface suggests that Curtis intended to select type species for the genera listed in this work. However, the Commission (ICZN 1957b) ruled in Opinion 488 that Curtis did not select type species.

**1858**. *The genera of British Coleoptera, transferred from the original figures, in 256 plates of “British Entomology.*” Lovell Reeve, London. viii pp. + 29 pls. (4to) [DP: 1858 (title page), 1 June 1858 (Anonymous 1858: 1), 26 June 1858 (*Lit Gaz*), 24 July 1858 (*Athenaeum*)] GB

This work consits of a list of the species of Coleoptera illustrated in Curtis’ British Entomology, 1824–1839 [*q.v.*] following by 29 plates, each with eight or nine illustrations of beetles extracted from the plates of the same work.

#### Curtis, Thomas. British educator; principal of Grove House school, Islington in London.

**1829**. Entomology. Pp. 433–509 + 6 pls *in*: *The London encyclopaedia, or universal dictionary of science, art, literature, and practical mechanics, comprising a popular view of the present state of knowledge. Illustrated by numerous engravings, a general atlas, and appropriate diagrams. By the original editor of the Encyclopaedia Metropolitana, assisted by eminent professional and other gentlemen. In twenty-two volumes. Vol. VIII.* Thomas Tegg, London. 776 pp. + 18 pls. (4to) [DP: 1829 (title page; publisher’s address in volume 1 dated 23 August 1829)] GB

Thomas Curtis was the editor of the *London encyclopaedia* which comprises 22 volumes, issued in 1829. Regarding the article on entomology, he acknowledged the valuable assistance of John Curtis [*q.v.*]. Curtis’ *London Encyclopaedia* was reissued in 1837 [HATH].

#### Curtis, William (Alton, Hampshire, United Kingdom: 11 January 1746 – 7 July 1799: London, United Kingdom). British botanist, entomologist and publisher; apothecary and demonstrator of practical botany at medical schools in London; established botanic gardens in England; his insect collection apparently passed to Adrian Hardy Haworth whose collection was auctioned in 1834. References. Ratzeburg (1874: 125); Curtis (1941); Lisney (1960: 204–206, P); Hunt (2004); Darby (2016).

**1772**. *Fundamenta entomologiae: or, an introduction to the knowledge of insects. Being a translation of the Fundamenta Entomologiae of Linnaeus, farther illustrated with copper plates and additions.* G. Pearch, London. viii + 90 pp. + 2 pls. (8vo) [DP: 1772 (title page), October 1772 (*Univers Cat*), November 1772 (*Gent Mag*; *J Sav*)] CMLE, GB, BHL

This is an English translation, with short remarks and additions, of Linnaeus’ *Fundamenta Entomologiae* [*q.v.*] published in 1767.

#### Cuvier, Georges Léopold Chrétien Frédéric Dagobert (Baron) (Montbéliard, Doubs, France: 23 August 1769 – 13 May 1832: Paris, France). French naturalist, anatomist and paleontologist; tutor to the son of the Comte d’Héricy in Normandy (1788–1794); professor of natural history at the Collège de France in Paris in 1799; professor of comparative anatomy at the Muséum National d’Histoire Naturelle in Paris from 1802 on; Grand Officer of the Legion of Honour and pair de France. References. Audouin (1832); Duvernoy (1833); Bolton (1889: 65–80, P); Adler (1989: 17–19); Smith (1993: 9–12, P); Jaussaud (2004: 157–160); Taquet (2006, P).

**1797**. *Tableau élémentaire de l’histoire naturelle des animaux*. Baudouin, Paris. xvi + 710 pp. + 14 pls. (8vo) [DP: “An 6” (title page), 24 December 1797 (*J Typogr Bibl*), 26 December 1797 (*Acad Sci Fr*), 21 December 1797–19 January 1798 (*Soc Philom*), 19 January 1798 (*Merc Fr*), 3 February 1798 (*J Soc Pharm*), April 1798 (*J Phys*)] CNC, GB, GAL

The Coleoptera are on pages 512–564. According to Smith (1993: 196), this work was translated into Danish (1801–1803), German (1800), Portuguese (1815) and Spanish (1834).

#### Czenpiński [Czempiński], Paweł [Paul] (1755 – 8 August 1793). Polish physician, educator and naturalist; employee of the Society for Elementary Books in Warsaw; made a scientific expedition to the Carpathian Mountains in 1782; co-founded the Warsaw School of Anatomy and Surgery. Reference. Anonymous (2015a).

**1778**. *Dissertatio inauguralis zoologico-medica, sistens totius regni animalis genera, in classes et ordines Linnaeana methodo digesta, praefixa cuilibet classi terminorum explicatione, quam annuente inclyta facultate medica in antiquissima ac celeberrima Universitate Vindobonensi publicae disquisitioni submittit Paulus de Czenpinski, nobilis polonus Varsoviensis. Disputabitur in Universitatis Palatio. Die mensis Aprilis anno 1778.* Joan. Thom. nob. de Trattnern, Viennae. [14] + 122 + [2] pp. (8vo) [DP: April 1778 (title page), July 1778 (*Allg Verz Bücher*)] MCZ, GDZ, GB

This thesis contains a list of animal genera with short descriptions; the Coleoptera are on pages 49–54. A new genus and species of beetle, *Scotias psylloides*, are described (pp. 51–52).

#### Dahl, Georg (Mosbach, Baden-Württemburg, Germany: 24 December 1769 – 1 January 1831: Währing near Vienna, Austria). German naturalist; insect dealer in Währing; travelled to several countries in Europe to collect; his personal collection of Coleoptera is at the Hungarian Natural History Museum in Budapest. References. Anonymous (1837: 413–414); Wurzbach (1858: 129–130).

**1823**. *Coleoptera und Lepidoptera. Ein systematisches Verzeichniss, mit beygesetzten Preisen der Vorräthe.* J.E. Akkermann, Wien. iv + 105 pp. (8vo) [DP: 1823 (title page; *Einleitung* dated 21 January 1823)] CNC, GB

This work is a catalogue of the Coleoptera and Lepidoptera, most from Austria and Hungaria, in the hands of the author with their prices. The book was suppressed in Opinion 710 by the ICZN (1964) for the purposes of nomenclature.

#### Dahlbom, Anders Gustav (Herrberga parish, Östergötland, Sweden: 3 March 1806 – 3 May 1859: Lund, Sweden). Swedish entomologist, specialist of Hymenoptera; professor of natural history and director of the entomological museum at the University of Lund. Reference. Anonymous (1906b).

**1837**. *Kort underrättelse om Skandinaviska insekters allmännare skada och nytta i hushållningen. Em handbok för landtbrukare och naturforskare.* Berling, Lund. vi + xxxvi + [2] + 340 + [12] pp. + 2 pls. (8vo) [DP: 1837 (title page; *Företal* dated midsummer 1837), October 1837 (*Swensk Bibl*)] GB

The Coleoptera are on pages 1–99.

#### Dale, Charles William (London, United Kingdom: 15 May 1851 – 20 February 1906: Sherborne, Dorset, United Kingdom). British naturalist of independent means who spent his life studying natural history, and particularly the insect fauna, of Glanvilles Wootton in southern England; his collection, which included that of his father James Charles Dale, was bequeathed to the Hope Museum at Oxford University. References. Bankes (1906); Salmon (2000: 139–141, P).

**1878**. *The history of Glanville’s Wootton, in the county of Dorset, including its zoology and botany.* Hatchards, London. viii + 392 pp. + 2 pls. (8vo) [DP: 1878 (title page), 24 August 1878 (*Athenaeum*), 31 August 1878 (*Garden*)] GB

The Coleoptera, essentially a list of species from the parish of Glanville’s Wootton, are on pages 79–126.

#### Dallinger, Franz Xaver Prosper (Eggenfelden, Bavaria, Germany: 3 July 1763 – 6 October 1826: Wasserburg, Bavaria, Germany). German preacher and entomologist. References. Callisen (1839: 209); Scheglmann (1904: 292–293).

**1798**. *Vollständige Geschichte des Borkenkäfers, Fichtenkreb’s, oder sogenannten schwarzen Wurms. Mit Vorschlägen und Mitteln seiner höchstschädlichen Bevölkerung zu steuern. Den Förstern und Jägern, vorzüglich in Baiern. Nebst einer ausgemalten Kupfertafel.* Gebrüder Jacobi, Weissenburg in Franken. 72 pp. + 1 pl. (8vo) [DP: 1798 (title page; *Vorbericht* dated October 1797), 15 January 1798 (*Ober Lit*), 17 February 1798 (*Reichs-Anz*)] GB

#### Dalman, Johan [Johann] Wilhelm (Hinseberg, Västmanland, Sweden: 4 November 1787 – 11 July 1828: Stockholm, Sweden). Swedish physician and naturalist; librarian and later professor at the Royal Swedish Academy of Sciences in Stockholm; also professor of botany at the Karolinska Institutet in Stockholm. References. Anonymous (1829a); Mohnike (1831: 57–70); Evenhuis (1997a: 189, P).

**1823**. *Analecta entomologica. Cum tabulis IV aeneis.* J.P. Lindh, Holmiae [= Stockholm]. vii + 104 + [4 (Index)] + 4 pls. (4to) [DP: 1823 (title page), 7 September 1823 (Evenhuis 1997a: 190), September 1823 (*Not Geb Natur*), December 1823 (*Isis*, Heft XII: [2])] CNC, GB

Apparently another printing was issued, also in Holmiae [= Stockholm], in 1824 (*n.v.*) as noticed in the *Journal général de la Littérature étrangère* 24: 321.

**1824**. *Ephemerides entomologicae. I.* P.A. Norstedt, Holmiae [= Stockholm]. [2] + 36 pp. (8vo) [DP: 1824 (title page)] CNC, GB

The entire work was reissued in 1828 in *Entomologisches Archiv* 1: 80–89.

#### Daniel, Karl (Munich, Bavaria, Germany: 1862 – 31 March 1930: Munich, Germany). German chemist and coleopterist; his collection of Palaearctic Coleoptera is at the Zoologische Staatssammlung München. Reference. Scherer (1992: 67).

**1891–1898**. [Daniel, K. and Daniel, J.] *Coleopteren-Studien.* C. Wolf & Sohn, München. (8vo) MCZ, ARC, GB

*I.* [3] + 64 pp. [DP: 17 May 1891 (title page), 14 December 1892 (*Soc Ent Fr*)].

*II.* [1] + 88 pp. [DP: 28 December 1898 (title page), 22 February 1899 (*Soc Ent Fr*)].

#### Dawson, John Frederic (1802 – 16 October 1870: Clapham, Bedfordshire, United Kingdom). British priest and coleopterist; curate of St. Pauls Church in Bedford, then rector of Toynton St Peter and vicar of Toynton All Saints in the district of Lincolnshire, England.

**1854**. *Geodephaga Britannica. A monograph of the carnivorous ground-beetles indigenous to the British Isles.* John van Voorst, London. xix + 224 pp. + 3 pls. (8vo) [DP: 1854 (title page; preliminary observations dated 6 April 1854), 6 May 1854 (*Athenaeum*), 15 May 1854 (*Publ Circ*), 1 June 1854 (*Monthly List Muquardt*), 30 June 1854 (*Intell Serapeum*), January–June 1854 (*Bibliot Hist-Nat*)] MCZ, BHL, GB

An extensive review of this book was published in *The Natural History Review* 1 [1854]: 105–112.

**1856**. [Dawson, J.F. and Clark, H.] *A rearrangement of the nomenclature and synonymy of those species of British Coleoptera which are comprised under the sections Geodephaga, Hydradephaga, and part of Philhydrida, being the first portion of a general British catalogue.* Taylor and Francis, London. 12 pp. (8vo) [DP: 1856 (title page), 12 April 1856 (*Ent Weekly Intel*), May 1856 (*Ann Mag Nat Hist* (2)17: 419)] YB (ph)

This work is essentially a synonymic list of British Cicindelidae, Carabidae, Dytiscidae, Gyrinidae, Heteroceridae, Parnidae, Georyssidae and Hydrophilidae. No further parts were published.

#### DeGeer, Carl (Finspång, Östergötland, Sweden: 10 February 1720 – 8 March 1778: Stockholm, Sweden). Swedish naturalist of independent means; Baron of Leutsta, Marshal of the Court of Sweden, Knight of the Polar Star, and Commander of the Order of Vasa; pupil of Linnaeus in Uppsala; inspired by the work of the celebrated French scientist and naturalist René Antoine Ferschault de Réaumur [1683–1757], thence his nickname of the *Swedish Réaumur*; burned most of the stock of the first volume of his *Mémoires pour servir à l’histoire des insectes* out of pique of the poor interest it raised; his collection is at the Naturhistoriska Riksmuseet in Stockholm. References. Walckenaer (1830: 145–147); Duncan (1840: 59–66); Ratzeburg (1874: 143–144); Ferrer (2011).

**1774–1778**. *Mémoires pour servir a l’histoire des insectes.* Pierre Hesselberg, Stockholm. (4to) GAL, GB

*Tome quatrième.* xii + 456 pp. + 19 pls. [DP: 1774 (title page), 4 February 1775 (*Erlang Anmer Nachr*)].

*Tome cinquième.* vii + [1 (Errata)] + 448 pp. + 16 pls. [DP: 1775 (title page), 5 April 1775 (*J Polit Litt*)].

*Tome septième. Ouvrage posthume.* xii + 950 pp. + 49 pls. [DP: 1778 (title page)].

The entire work consists of seven volumes, 1752–1778. The Coleoptera are in volumes 4 and 5. Volume 7 contains a section entitled “*Dixième mémoire. Supplément aux mémoires des volumes précédens, ou description de quelques insectes ailés, suivie de celle de plusieurs insectes du Cap-de-Bonne Espérance*” (pp. 591–666) where several new species of Coleoptera are described (pp. 622–666).

A German translation of DeGeer’s work was issued by J.A.E. Goeze, 1777–1783 [*q.v.*].

Welter-Schultes and Wieland (2012) pointed out, in their application to the Commission, that DeGeer did not consistently follow strict binominal rules in this work when describing new species. Because many of the specific names proposed by DeGeer are used today, they proposed that the Commission ruled the work available while suppressing several polynominal names. The Coleoptera polynominal names recommended for suppression are: *Lampyris noctiluca communis* (vol. 4, p. 31), *Elater fuscus major* (vol. 4, p. 146), *Elater fuscus minor* (vol. 4, p. 146), *Elater aeneus rufipes* (vol. 4, p. 149), *Elater fuscus flavipes* (vol. 4, p. 151), *Silpha nigra major* (vol. 4, p. 173), *Leptura aquatica spinosa* (vol. 5, p. 140), *Leptura aquatica mutica* (vol. 5, p. 142), *Leptura aquatica fasciata* (vol. 5, p. 142), *Leptura aquatica aenea* (vol. 5, p. 143), *Chrysomela marginella ranunculi* (vol. 5, p. 304), *Chrysomela viridis alni* (vol. 5, p. 306), *Chrysomela coerulea betulae* (vol. 5, p. 317), *Chrysomela coerulea salicis* (vol. 5, p. 318), *Chrysomela grisea alni* (vol. 5, p. 325), *Chrysomela cylindrica 4-punctata* (vol. 5, p. 329), *Chrysomela rubra liliorum* (vol. 5, p. 339) and *Chrysomela 22-punctata obscura* (vol. 5, p. 380). In Opinion 2333 (ICZN 2014) the Commission suppressed these polynominal names and placed them on the *Official Index of Rejected and Invalid Specific Names in Zoology*. In the same Opinion, volumes 3–7 (1773–1778) of DeGeer’s *Mémoires pour servir a l’histoire des insectes* have been placed on the *Official List of Works Approved as Available for Zoological Nomenclature*.

#### Dei, Giovanni Angelo Apelle Crespino (Siena, Tuscany, Italy: 17 December 1819 – 2 January 1903: Siena, Italy). Italian ornithologist and entomologist; curator at the “Gabinetto di Anatomia Comparata” of the University of Siena and at the “Museo dell’Accademia dei Fisiocritici” of the same city where his collection is deposited. References. Conci (1975: 896); Baccetti (1979); Conci and Poggi (1996: 226).

**1862**. *Insecta Senarum in agro suburbano atque in ipsa civitate lecta et in Museo Fisiocriticorum Academiae servata*. A. Moschini, Senis [= Siena]. 16 pp. (4to) [DP: 1862 (title page)] GB

This is a catalogue of the insects found in the vicinity of Siena in Italy.

#### Dejean, Pierre François Marie Auguste (Count) (Amiens, Somme, France: 10 August 1780 – 17 March 1845: Paris, France). French military officer and entomologist, specialized on Coleoptera; joined the army at the age of 15 and moved on to become general of division and aide-de-camp to Napoleon Bonaparte; collected insects in several countries, including Spain, Portugal, Germany, and Russia, where his military activity took him; went in exile in June 1815 and for the next three years collected in the eastern parts of the Austrian empire; returned in Paris at the end of 1818 and devoted the rest of his life mostly to entomology and politics, as he became a member of the *Chambre des Pairs* in 1824; his collection, the largest private beetle collection of his time, was sold in lots during 1840. References. Boisduval (1846, P); Fleutiaux (1894); Haythornthwaite (1998: 92); Bousquet (2004: 34–36, P); Gouillard (2004: 36–37); Cambefort (2006: 152–154).

**1802**. *Catalogue des coléoptères de la collection d’Auguste Dejean: classés suivant le Systema Eleutheratorum Fabricii.* Imprimerie de la République, Paris. [2] + 11 pp. (8vo) [DP: “An XI. –1802 v[ieux] st[yle]” (last page), 21 September–31 December 1802 (Madge 1988: 318)] HOU

Overall 910 species are mentioned in this catalogue. No new taxa are proposed in it. Apparently the publication was not for sale (Boisduval 1846: 501), but since it was distributed to friends and colleagues, it is available. A facsimile was printed in Paris, 1929 [CNC].

**1821**. *Catalogue de la collection de coléoptères de M. le Baron Dejean.* Crevot, Paris. viii + 136 + [2 (Errata)] pp. (8vo) [DP: 1821 (title page), 12 May 1821 (*Bibl Fr*), June 1821(*Rev Encycl*), August 1821 (*Not Geb Natur*)] CNC, BHL, GB

The catalogue recorded 6,692 species. Several new genus-group taxa were made available by inclusion of one or more available species under them.

**1825–1831**. *Species général des coléoptères, de la collection de M. le Comte Dejean.* Crevot [vols 1–2] / Méquignon-Marvis [vols 3–5], Paris. (8vo) CNC, YB, GB, GAL

*Tome premier.* xxx + 463 pp. [DP: 1825 (title page), 5 September 1825 (*Acad Sci Fr*), 10 September 1825 (*Bibl Fr*), 30 September 1825 (*Rev Bibl-Brus*)].

*Tome second.* viii + 501 pp. [DP: 1826 (title page), 17 April 1826 (*Acad Sci Fr*), 22 April 1826 (*Bibl Fr*), May 1826 (*Rev Bibl-Brus*), July 1826 (*Not Geb Natur*)].

*Tome troisième.* vii + 556 pp. [DP: 1828 (title page), 24 November 1828 (*Acad Sci Fr*), 29 November 1828 (*Bibl Fr*), September–December 1828 (*Foreign Quart Rev*), 31 January 1829 (*Rev Bibl-Brus*)].

*Tome quatrième.* vii + 520 pp. [DP: 1829 (title page), 23 November 1829 (*Acad Sci Fr*), 5 December 1829 (*Bibl Fr*), 10 December 1829 (*Gaz Litt*), 31 January 1830 (*Rev Bibl-Brus*)].

*Tome cinquième.* viii + 883 pp. [DP: 1831 (title page), 11 July 1831 (*Acad Sci Fr*), 23 July 1831 (*Bibl Fr*)].

**1829–1836**. [Dejean, P.F.M.A. and Boisduval, J.B.A.] *Iconographie et histoire naturelle des coléoptères d’Europe.* Méquignon-Marvis, Paris. (8vo) CNC, BHL, GAL

*Tome premier.* xiv + 400 pp. + 60 pls. This volume was published in 12 livraisons: **1**: (pp. i–xiv, 1–32, pls 1–5) 26 September 1829; **2**: (pp. 33–80, pls 6–10) 7 November 1829; **3**: (pp. 81–112, pls 11–15) 5 December 1829; **4**: (pp. 113–144, pls 16–20) 30 January 1830; **5**: (pp. 145–176, pls 21–25) 20 February 1830; **6**: (pp. 177–208, pls 26–30) 20 March 1830; **7**: (pp. 209–240, pls 31–35) 24 April 1830; **8**: (pp. 241–272, pls 36–40) 29 May 1830; **9**: (pp. 273–304, pls 41–45) 12 June 1830; **10**: (pp. 305–336, pls 46–50) 24 July 1830; **11**: (pp. 337–368, pls 51–55) 18 September 1830; **12**: (369–400, pls 56–60) 16 October 1830. The title page is dated 1829. The book was reprinted in 1837.

*Tome second.* 407 pp. + pls 61–125. This volume was issued in 14 livraisons: **1**: (pp. 1–32, pls 61–65) 13 November 1830; **2**: (pp. 33–48, pls 66–70) 18 December 1830; **3**: (pp. 49–64, pls 71–75) 5 February 1831; **4**: (pp. 65–80, pls 76–80) 9 April 1831; **5**: (pp. 81–96, pls 81–85) 10 April–15 July 1831; **6**: (pp. 97–128, pls 86–90) 16 July 1831; **7**: (pp. 129–160, pls 91–95) 3 September 1831; **8**: (pp. 161–192, pls 96–100) 26 November 1831; **9**: (pp. 193–224, pls 101–105) 7 January 1832; **10**: (pp. 225–256, pls 106–110) 11 February 1832; **11**: (pp. 257–288, pls 111–115) 17 March 1832; **12**: (pp. 289–320, pls 116–120) 12 May 1832; **13**: (pp. 321–352, pls 121–125) 14 July 1832; **14**: (pp. 353–407, pls 126–130) 13 October 1832. The title page is dated 1830. The book was reprinted in 1837.

*Tome troisième.* 326 pp. + pls 126–171. This volume was issued in eight livraisons: **1**: (pp. 1–48, pls 131–135) 8 December 1832; **2**: (pp. 49–96, pls 136–140) 25 February 1833 (*Bibliologue*), 2 March 1833; **3**: (pp. 97–128, pls 141–145) 4 May 1833; **4**: (pp. 129–176, pls 146–150) 29 June 1833; **5**: (pp. 177–208, pls 151–155) 27 July 1833; **6**: (pp. 209–240, pls 156–160) 21 September 1833; **7**: (pp. 241–272, pls 161–165) 26 October 1833; **8**: (pp. 273–326, pls 166–170) 11 January 1834. The title page is dated 1832. This volume was reprinted in 1840.

*Tome quatrième.* 486 pp. + pls 172–223. This volume was issued in 12 livraisons: **1**: (pp. 1–32, pls 171–175) 22 March 1834; **2**: (pp. 33–64, pls 176–179) 17 May 1834; **3**: (pp. 65–96, pls 180–184) 26 July 1834; **4**: (pp. 97–128, pls 185–188) 9 August 1834; **5**: (pp. 129–160, pls 189–193) 11 October 1834; **6**: (pp. 161–192, pls 194–198) October–December 1834; **7**: (pp. 193–224, pls 199–203) October–December 1834; **8**: (pp. 225–256, pls 204–208) January–March 1835; **9**: (pp. 257–320, pls 209–213) April–June 1835; **10**: (pp. 321–384, pls 214–218) October–December 1835; **11**: (pp. 385–432, pls 219–223) July–September 1836; **12**: (pp. 433–486) July–September 1836. The title page is dated 1834.

The dates of publication and collation are from Bousquet (2004: 43). All new taxa proposed in this work are to be attributed to Dejean alone (Bousquet 2004: 42). A fifth volume in the series was authored by Aubé, 1836–1838 [*q.v.*].

**1833–1836**. *Catalogue des coléoptères de la collection de M. le Comte Dejean.* Méquignon-Marvis, Paris. 443 pp. (8vo) CNC, BHL, ARC, GAL

This catalogue was issued in five livraisons: **1**: (pp. 1–96) 15 January 1833 (*Bibliologue*), 19 January 1833 (*Bibl Fr*), 18 March 1833 (*Acad Sci Fr*); **2**: (pp. 97–176)^21^ 27 July 1833 (*Bibl Fr*), 9 August 1833 (*J Débats*); **3**: (pp. 177–256) April–June 1834 (*Soc Ent Fr* [*Annales*, p. xxxv]); **4**: (pp. 257–360) 22 August 1835 (*Bibl Fr*), August 1835 (*Bull Bibl*); **5**: (pp. 361–443) end of 1836 (Madge 1988: 318). This edition lists 20,909 species. Many new genus-group taxa are made available by the inclusion of one or more available species under them. As discussed by Bousquet (2004: 38), comments made by Dejean in the *avertissement* of the third edition [*q.v.*] indicate that Chevrolat should be credited for the new genus-group taxa of the *chrysomélines* (pp. 383–455) and *trimères* (pp. 456–464) sections attributed to Chevrolat. The other new taxa available are to be credited to Dejean even though some of the names are attributed to other authors in the catalogue. All new genus-group names introduced in this work have been commented upon by Bousquet and Bouchard (2013a).

**1836–1837**. *Catalogue des coléoptères de la collection de M. le Comte Dejean. Troisième édition, revue, corrigée et augmentée*. Méquignon-Marvis, Paris. xiv + 503 pp. (8vo) CNC, GB

This catalogue was published in five livraisons, the first four issued together: **1–4**: (pp. 1–384) 30 July 1836 (*Bibl Fr*), 10 August 1836 (*Lit Ztg*), August 1836 (*J Lit Fr*), 1 March 1837 (*Soc Ent Fr*); **5**: (pp. xiv + 385–503) 27 May 1837 (*Bibl Fr*), 7 June 1837 (*Lit Ztg*), 5 July 1837 (*Soc Ent Fr*). The title page is dated 1837 and the *avertissement* April 1837. This edition lists 22,399 species. Some new genus-group taxa are made available for the first time. All new genus-group names of this work have been discussed by Bousquet and Bouchard (2013b).

#### Delegorgue, Louis Adulphe Joseph (Courcelles-les-Lens, Pas-de Calais, France: 13 November 1814 – 30 May 1850: at sea, off the West African coast). French traveler, collector and sportsman, nicknamed the “elephant killer;” joined the merchant navy at the age of sixteen; travelled to the West Indies and Africa; Knight of the Legion of Honour. References. Berthoud (1850); Anonymous (1861: 207); Frixon (1892); Guellaën-Vaast (1989, P).

**1847**. *Voyage dans l’Afrique australe notamment dans le territoire de Natal, dans celui des Cafres Amazoulous et Makatisses et jusqu’au tropique du Capricorne exécuté durant les années 1838, 1839, 1840, 1841, 1842, 1843 & 1844. Accompagné de dessins et cartes. Avec une introduction par M. Albert-Montémont. II.* A. René et C^e^, Paris. 622 + [2 (Errata)] pp. + 4 pls. (8vo) [DP: 1847 (title page), 31 July 1847 (*Bibl Fr*), 10 August 1847 (*Monthly Lit Adv*), 11 August 1847 (*Lit Ztg*), 16 August 1847 (*Publ Circ*), 20 August 1847 (*Soc Géog Fr*), 30 October 1847 (*Illustration*)] GB, GAL

This volume contains a “Catalogue des insectes coléoptères” (pp. 603–613). The work was published in two volumes, both issued in 1847.

#### Denny, Henry (Norwich, Norfolk, United Kingdom: 1803 – 7 March 1871: Leeds, West Yorkshire, United Kingdom). British botanist and entomologist, especially interested in Anoplura; curator of the museum and assistant secretary of the Leeds Literary and Philosophical Society for 45 years. References. Harrison (1888); Denny (1889).

**1825**. *Monographia pselaphidarum et scydmaenidarum Britanniae: or an essay on the British species of the genera Pselaphus, of Herbst, and Scydmaenus, of Latreille: in which those genera are subdivided, and all the species hitherto discovered in Great Britain are accurately described and arranged, with an indication of the situations in which they are usually found: each species illustrated by a highly magnified figure.* S. Wilkin, Norwich. vi + 74 pp. + 14 pls. (8vo) [DP: 1825 (title page)] CNC, GB

#### Dent, Hasting Charles (London, United Kingdom: 23 June 1855 – 6 March 1909: Godstone, Surrey, United Kingdom). British civil engineer and collector of entomological (particularly Coleoptera and Lepidoptera) and botanical specimens; the whereabouts of his collections are unknown. Reference. Jackson (1909).

**1886**. *A year in Brazil with notes on the abolition of slavery, the finances of the empire, religion, meteorology, natural history, etc. With ten full-page illustrations and two maps.* Kegan Paul, Trench & Co., London. xvii + [1 (List of illustrations)] + 444 pp. + 10 pls. (8vo) [DP: 1886 (title page; preface dated March 1886), 29 May 1886 (*Athenaeum*), 26 June 1886 (*Academy*), 1 July 1886 (*Nation*), 17 July 1886 (*Deutsch Litt*)] GB, BHL

A list of beetles collected during the voyage is included on page 389–397 followed by “Note on a few of the above Coleoptera” (pp. 397–399) where one new species (*Compsocerus jucundus*, p. 397) is briefly described.

#### Desberger [Dessberger], Anton Friedrich A. (Munich, Bavaria, Germany: 8 December 1789 – ?). German military physician. Reference. Hahn (1883).

**1834**. *Dr. Johann Matthäus Bechstein’s Forstinsectologie oder Naturgeschichte der für den Wald schädlichen und nützlichen Insecten nebst Einleitung in die Insectenkunde überhaupt, für angehende und ausübende Forstmänner und Cammeralisten. Neu bearbeitet. Zweiter Theil. Beschreibende Forst-Insectenkunde. Mit vier illuminirten und einer schwarzen Kupfertafel.* Hennings, Gotha. iv + 400 pp. + 5 pls. (8vo) [DP: 1835 (title page), 20 September 1834 (*Bibl Deutsch*), November 1834 (*Nouv Rev Germ*), 13 December 1834 (*Bayer Ann*), July–December 1834 (*Verz Bücher Landkarten*), 28 January 1835 (*Allg Forst Jagd Ztg*)] SDL

Another title page reads “*Die Forst- und Jagdwissenschaft nach allen ihren Theilen für angehende und ausübende Forstmänner und Jäger. Ausgearbeitet von einer Gesellschaft und herausgegeben von Dr. Johann Matthäus Bechstein. Vierter Theil. Forstschutz. Zweiten Bandes erste Abtheilung. Beschreibung der Forst-Insekten nebst ihren Verhütungs- und Vertilgungs-Mitteln. Zweite Auflage. Mit vier illuminirten und einer schwarzen Kupfertafel.*” The first edition was issued in 1818 by Johann M. Bechstein [*q.v.*].

Two new beetles taxa are described in this work, *Curculio pomorum rubromaculatus* (p. 64) and *Curculio pomorum rubellus* (p. 64); these are not listed in Sherborn’s *Index Animalium*.

#### Desbrochers des Loges, Jules (Béthune, Pas-de-Calais, France: 1836 – 10 August 1913: Tours, Indre-et-Loire, France). French coleopterist; civil servant at the Ministry of Finance; natural history dealer at Tours; founded the entomological journal *Le Frelon*; his collection was sold in part during his lifetime and the remaining (mainly Curculionidae and Chrysomelidae) by his daughter after his death and are now at the Muséum National d’Histoire Naturelle in Paris. References. Sainte-Claire Deville (1914); Cambefort (2006: 156).

**1867**. *Notice sur l’entomologie du Bourbonnais suivie de la description de trois espèces nouvelles.* C. Desrosiers, Moulins. [1] + 44 pp. (8vo) [DP: 1867 (title page), January–March 1867 (*Soc Ent Fr* [*Bulletin bibliographique*, p. lxxvi])^22^] CMLE, GB

This publication was apparently also published in the *Bulletin des Assises scientifiques du Bourbonnais* 1866 (*n.v.*), on pages 122–165 (see Bouchard 1872: 71).

The three new species described are: *Apion tibiale* (p. 40), *Apion conspicuum* (p. 42), and *Harpalus reichei* (p. 42).

**1875**. *Opuscules entomologiques (coléoptères). 1^er^ cahier 1874–1875.* A. Gaudon, Gannat. [1] + 56 pp. (8vo) CMLE, GB

The dates listed at the bottom of some of the pages are 28 November 1874 (pp. 1–8), 7 January 1875 (pp. 9–12), 10 January 1875 (pp. 13–20), 7 April 1875 (pp. 21–36), and 5 September 1875 (pp. 37–56). However, as discussed by Desbrochers des Loges (1892: 88), pages 1–36 were issued on 13 April 1875 and pages 37–56 on 12 September 1875.

#### Deshayes, Gérard Paul (Nancy, Meurthe-et-Moselle, France: 15 May 1795 – 9 June 1875: Boran-sur-Oise, Oise, France). French geologist and malacologist; participated to the Algeria mission under the direction of Jean-Baptiste Bory de Saint-Vincent; at 70 years old, obtained the post of professor of natural history at the Muséum National d’Histoire Naturelle in Paris. References. Crosse and Fischer (1876); Evans (1876: 80–82); Jaussaud (2004: 184–185).

**1835**. [Deshayes, G.P. and Milne-Edwards, H.] *Histoire naturelle des animaux sans vertèbres, présentant les caractères généraux et particuliers de ces animaux, leur distribution, leurs classes, leurs familles, leurs genres, et la citation des principales espèces qui s’y rapportent; précédée d’une introduction offrant la détermination des caractères essentiels de l’animal, sa distinction du végétal et des autres corps naturels; enfin, l’exposition des principes fondamentaux de la zoologie par J.B.P.A. de Lamarck. Deuxième édition, revue et augmentée de notes présentant les faits nouveaux dont la science s’est enrichie jusqu’à ce jour. Tome quatrième. Histoire des insectes.* J.B. Baillière, Paris. 787 [last two numbered 586 and 587] pp. (8vo) [DP: 1835 (title page), December 1835 (Sherborn 1922a: lxxvii), 2 January 1836 (*Bibl Fr*), January 1836 (*Bull Bibl*)] BHL, GB

This edition of Lamarck’s work was issued in 11 volumes, 1835–1845. The Coleoptera are on pages 466–773 of volume 4. A third edition of Lamarck’s work was published in three volumes in Brussels, 1837–1839, under the same title with the addition of the words “*Troisième édition, revue et augmentée de notes présentant les faits nouveaux dont la science s’est enrichie jusqu’a ce jour; par MM. G.P. Deshayes et H. Milne Edwards*” [ANSP, GB]; the Coleoptera are on pages 160–264 of the second tome issued in 1839. According to Iredale (1922: 84–85) the third edition “must have been published in livraisons” as there are annotations first published in 1843 in the third volume dated 1839.

#### Desmarest, Eugène Anselme Sébastien Léon (Paris, France: 31 March 1816 – 25 December 1889: Paris, France). French naturalist; preparator for the chair of comparative anatomy and, 37 years later, keeper of the comparative anatomy and anthropology galleries at the Muséum National d’Histoire Naturelle in Paris; secretary of the *Société Entomologique de France* for more than 30 years. Reference. Jaussaud and Brygoo (2004: 185).

[**1850].** (Coleoptères.). Pp. 291–328 *in*: *Voyage autour du monde exécuté pendant les années 1836 et 1837 sur la corvette* La Bonite *commandée par M. Vaillant capitaine de vaisseau. Publié par ordre du Gouvernement sous les auspices du département de la marine. Zoologie par MM. Eydoux et Souleyet, médecins de l’expédition. Tome premier.* Arthus Bertrand, Paris. iv + xxxix + 334 pp. (8vo) CNC, CAKL, BHL, GB

This volume was published in two parts: **1**: (iv + xxxix + pp. 1–132) 18 December 1841 (*Bibl Fr*), 17 June 1842 (*Soc Géog Fr*); **2**: (pp. 135–334) after March 1848, probably early 1850 (Bauchot et al. 1982: 63). The title page is dated 1841 and the *avant-propos* November 1841. Sherborn and Woodward (1901a: 391) dated the second part 1842 but Bauchot et al. (1982) found conclusive evidence that the reptile (pp. 133–154) and fish texts (pp. 155–216) were published after March 1848 and most likely in the early part of 1850. Since the insect section was not published separately, the insect names should be dated 1850.

There are two plates in the atlas illustrating insects, the second one (Insectes. Coléoptères Pl. 2) beetles. I have found no date of publication for the plates. All the names on the plates are in vernacular form.

Some of the new species of Coleoptera collected on this expedition were described earlier by F. Eydoux and L.F.A. Souleyet in *Revue Zoologique, par la Société Cuvérienne* 1839: 264–267 under the title “*Descriptions sommaires de quelques Coléoptères nouveaux, provenant de Manille et destinés à être publiés dans le voyage autour du monde de la corvette la Bonite*.”

The entire report of the expedition was published in 15 volumes of text and four atlases, 1840–1866. The zoology section consists of two volumes and an atlas of 101 engraved plates issued in several livraisons. The Vaillant expedition sailed via Cape Horn, and visited Brazil, Chile, Bolivia, Peru, and Colombia. The crew stayed a month in Hawaii, visiting both Kealakekua Bay and Honolulu, and then continued on to the Philippines and Canton. Cochin-China, Singapore, India, and finally St. Helena were also visited.

**1850–[1851**]. *Encyclopédie d’histoire naturelle ou traité complet de cette science d’après les travaux des naturalistes les plus éminents de tous les pays et de toutes les époques; Buffon, Daubenton, Lacépède, G. Cuvier, F. Cuvier, Geoffroy Saint-Hilaire, Latreille, de Jussieu, Brongniart, etc., etc. Ouvrage résumant les observations des auteurs anciens et comprenant toutes les découvertes modernes jusqu’à nos jours. Par le D^r^ Chenu. Coléoptères cicindelètes, carabiques, dytisciens, hydrophiliens, sylphales et nitidulaires, avec la collaboration de M.E. Desmarest, secrétaire de la Société Entomologique.* Marescq et Compagnie, Paris. [2] + 312 pp. + 28 pls. (4to) [DP: n.d. (title page)] CNC, GB, BHL

The entire *Encyclopédie d’histoire naturelle* consists of 31 volumes, 22 of text, illustrated with more than 8,000 figures and nine of tables (written by Eugène Desmarest), 1850–1861, under the direction of Jean Charles Chenu [1808–1879]. The Coleoptera section forms three volumes, 1850–1860, and one of tables, 1861 (Cowan 1971a). Although there is clear evidence that all three Coleoptera volumes were issued in series of livraisons, the publication dates of these cannot be established at this time. The first series of livraisons (3½ *feuilles* and 6 plates) was noticed on 21 September 1850 (*Bibl Fr*). No further livraisons were recorded in the *Bibliographie de la France*. The date of 1851 should be accepted for nomenclatural purposes. All three Coleoptera volumes contain type species designations for many genera.

Despite that the title pages of the three volumes of the *Encyclopédie d’Histoire naturelle* dealing with Coleoptera list Jean Charles Chenu as the author, the wrappers of the livraisons indicated that Desmarest was the author (see Bouchard et al. 2011: 12).

**1851–1852**. *Encyclopédie d’histoire naturelle ou traité complet de cette science d’après les travaux des naturalistes les plus éminents de tous les pays et de toutes les époques; Buffon, Daubenton, Lacépède, G. Cuvier, F. Cuvier, Geoffroy Saint-Hilaire, Latreille, de Jussieu, Brongniart, etc., etc. Ouvrage résumant les observations des auteurs anciens et comprenant toutes les découvertes modernes jusqu’à nos jours. Par le D^r^ Chenu. Coléoptères staphyliniens, psélaphiens, dermestiens, ptiniens, clériens, malachiens, etc. Avec la collaboration de M.E. Desmarest, du Muséum d’Histoire naturelle, secrétaire de la Société Entomologique de France. Deuxième partie.* Marescq et Compagnie, Paris. [2] + 312 pp. + 40 pls. (4to) [DP: n.d. (title page), 10 April 1852 (*Feuil J Lib*), 23 September 1852 (*Presse*), 10 December 1852 (*J Débats*)] CNC, GB, BHL

The first three livraisons were published in 1851 (see *Soc Ent Fr* [*Annales* (2) 9: cxxv]). The date of 1852 should be accepted for nomenclatural purposes. Horn and Schenkling (1928a: 187) dated this volume 1857.

**1860**. *Encyclopédie d’histoire naturelle ou traité complet de cette science d’après les travaux des naturalistes les plus éminents de tous les pays et de toutes les époques; Buffon, Daubenton, Lacépède, G. Cuvier, F. Cuvier, Geoffroy Saint-Hilaire, Latreille, de Jussieu, Brongniart, etc., etc. Ouvrage résumant les observations des auteurs anciens et comprenant toutes les découvertes modernes jusqu’à nos jours. Par le D^r^ Chenu. Coléoptères buprestiens, scarabéiens, piméliens, curculioniens, scolytiens, chrysoméliens, etc. Avec la collaboration de M.E. Desmarest, du Muséum d’Histoire naturelle, secrétaire de la Société Entomologique de France, etc. Troisième partie.* Marescq et Compagnie, Paris. [3] + 360 pp. + 48 pls. (4to) [DP: n.d. (title page; preface dated 31 January 1860)] CNC

Cowan (1971a: 14, 17), without qualification, dated this volume “1859–1860.” The three volumes on Coleoptera were reissued in 1870 and 1884 by Firmin Didot.

#### Detharding, Georg Christoph (Güstrow, Mecklenburg-West Pomerania, Germany: 10 April 1699 – 9 October 1784: Bützow, Mecklenburg-West Pomerania, Germany). German physician; professor of medicine and mathematics at the University of Rostock (1733–1760); later, professor of medicine at the University of Bützow (1760–1784). References. Meusel (1803: 340–342); Fromm (1877); Hahn (1883).

**1763**. *Dispvtatio physico-medica inavgvralis, de insectis Coleopteris Danicis. Qvam, consensv gratiosae facvltatis medicae, in Academia Fridericiana Bvetzoviensi, svb praesidio, Dn. Georg. Christoph. Dethardingii, sereniss. dvc. regn. megapol. consil. avlic. facvlt. med. senioris et decani, ann. MDCCLXIII. die ivl. pro gradv doctoris legitime impetrando h.l.q.c. Pvblico ervditorvm examini svbmittit Ioannes Pavli, Neocopiensis Falstrorvm.* Iohann. Gotth. Fritzii, Bvetzovii [= Bützow]. [4] + 34 pp. (4to) [DP: July 1763 (title page)] GB

This thesis contains a list of Coleoptera (including *Gryllus* and *Forficula*) of Denmark with explanatory comments. The genera are briefly described. There is a section (pp. 29–34) on the use of certain beetles in medicine. There are no new species described.

#### Deyrolle, Achille (Lille, Nord, France: 2 October 1813 – 31 December 1865: Paris, France). French entomologist, publisher, and dealer of natural history specimens in Paris; as a young man went to Brussels to help his father at the natural history museum of the city; participated in a scientific mission to Brazil for five months; afterwards moved to Paris; his collection was scattered by his son Émile. References. Grenier (1866); Cambefort (2006: 157).

**1862**. Annexe H de l’ouvrage intitulé: Notes sur l’ile de la Réunion par L. Maillard. Coléoptères. Pp. 1–21 + pl. 20 *in*: *Notes sur l’île de la Réunion (Bourbon) par L. Maillard.* Dentu, Paris. 344 + 4 (Annexe à la liste des oiseaux) + 7 (Echinides et stellerides) + [1 (Cnidiaires)] + 32 (Faune ichthyologique) + 8 (Ethnologie) + 2 (Mollusques) + 16 (Faune carcinologique) + 72 (Lépidoptères) + 40 (Coléoptères, Orthoptères, etc.) + 25 (Algues) + 5 (Botanique phanérogamique) + 4 (Table des matières) + 2 (Errata des annexes) pp. + 27 pls. (8vo) [DP: 1862 (title page; dedication dated 22 January 1862), 21 March 1863 (*Bibl Fr*), March 1863 (*Allg Bibl*), April 1863 (*Rev Mar Colon* 7: 785)] MCZ, GB, BHL

Maillard’s work consists of two parts, each with their own title page: the first one consists of 344 pages of text; the second one (“*Seconde partie. Annexes*”) consists of faunal lists arranged into “annexes” A–R. Some annexes are separately paginated, others are grouped. Annexes H–M are continuously paginated and include all the insects, except Lepidoptera.

A second edition was published in 1863 under the title “*Notes sur l’île de la Réunion (Bourbon). Deuxième édition revue et corrigée*” [GB] by the same publisher. There is no difference between the two editions regarding the Coleoptera section (Annexe H).

#### Diaz y Lizana, Rafael (1820 – 1873). Pharmaceutical subdelegate of the city of Talavera de la Reine, Toledo province, Spain.

**1864**. *Discurso leido en la Universidad Central por el licenciado Don Rafael Diaz y Lizana, en el acto solemne de recibir la investidura de Doctor en la Facultad de Farmacia.* Manuel Tello, Madrid. 42 pp. (8vo) [DP: 1864 (title page; last page dated 17 January 1864)] GB

The half-title page reads “*Consideraciones filosófico-naturales sobre los insectos, seguidas de una monografia de los epispásticos.*”

#### Dieck, Georg (Zöschen, Saxony-Anhalt, Germany: 28 April 1847 – 21 October 1925: Zöschen, Germany). German botanist, entomologist, and explorer; studied natural history at the University of Jena where he was a pupil and assistant to Ernst Haeckel; open a nursery in 1874 at Zöschen; made expeditions to the Rocky Mountains (1888), Caucasus (1891) and Spain (1892) where he collected mostly beetles, plants and mosses; travelled also to France, Italy, Sicily, Morocco, the Balkans and Turkey; his collection is at the Zoologisches Museum, Martin-Luther-Universität in Halle. References. Frahm and Egger (2001: 76–77, P); Mantzsch (2005); Kümmel and Kiehne (2009, P).

**1869**. *Diagnosen neuer blinder Käfer aus Süd-Europa und von der Nordküste Maroccos.* Merseburg. [8] pp. (4to) [DP: n.d. (title page; last page dated 5 June 1869), 1 August 1869 (*Pet Nouv Ent*)] YB (ph)

This booklet includes short descriptions of one new genus and 21 new species-group taxa. These, except for *Amaurorhinus andalusicus*, were redescribed in length afterwards in *Berliner Entomologische Zeitschrift* 13 [1869]: 337–360 [DP: January 1870].

#### Dillwyn, Lewis Weston (London, United Kingdom: 21 August 1778 – 31 August 1855: Swansea, Wales, United Kingdom). British naturalist, porcelain manufacturer, and member of parliament for the county of Glamorgan; the whereabouts of his beetle collection are unknown. References. Williams (1855: 287–304); Anonymous (1857: xxxvi–xxxix); Walker (2003, P).

**1829**. *Memoranda relating to coleopterous insects, found in the neighbourhood of Swansea. Not published.* W.C. Murray and D. Rees, Swansea. [2] + 75 pp. (8vo) [DP: n.d. (title page; preface dated 11 November 1829), 1829 (Dillwyn 1848: 18)] CMLE (mf), CNC (mf)

This work was published anonymously.

#### Dimmock, George (Springfield, Massachusetts, USA: 17 May 1852 – 17 May 1930: Springfield, Massachusetts, USA). American zoologist, particularly interested in entomology and genealogy; studied at Harward, Leipzig and Paris; secretary and librarian of the Cambridge Entomological Club; edited the entomological journal *Psyche* from 1877 to 1890; his collection of Coleoptera is at the National Museum of Natural History in Washington DC. Reference. Emerson (1930, P).

**1884**. Order VII. – Coleoptera. Pp. 297–402 *in*: *The standard natural history. Edited by John Sterling Kingsley. Vol. II. Crustacea and insects. Illustrated by six hundred and sixty-six wood-cuts and twenty full-page plates.* S.E. Cassino and Company, Boston. vi + [1] + 555 + [1 (Special notice)] pp. + 20 pls. (4to) BHL

Dimmock’s contribution was issued in two parts: (pp. 297–336) 15 August 1884; (pp. 337–402) 27 September 1884. The dates of publication are listed on the last page, entitled “Special notice,” of the book.

The entire series consists of six volumes, 1883–1885, issued in parts. It was reissued in 1888, under the title “*The Riverside Natural History edited by John Sterling Kingsley*” in London by Kegan Paul, Trench & Co.

#### Disconzi, Francesco (Vicenza, Veneto, Italy: 8 September 1811 – 29 November 1875: Vicenza, Italy). Italian priest and naturalist; professor at the Seminario Vescovile di Vicenza; founding member of the *Società Entomologica Italiana*; his collection was originally deposited in the Seminario in Vicenza where it was destroyed during WWII. Reference. Rumor (1905: 524).

**1865**. *Entomologia Vicentina; ossia catalogo sistematico degl’insetti della provincia di Vicenza con osservazioni e descrizioni di moltissime specie degl’insetti utili e dei nocivi particolarmente all’agricoltura colla giunta di un metodo pratico sulla caccia degl’insetti e sul modo di apparecchiarli per le collezioni. Con disegni litografici.* G.B. Randi, Padova. [2] + 316 pp. + 18 pls. (8vo) [DP: 1865 (title page), 2 May 1866 (*Zool-Bot Gesell Wien*), July 1866 (*Quart J Sci* 11: 406), October 1866 (*Allg Bibl*)] GB, MDZ

This work consists of a list of insects from the province of Vicenza in northeastern Italy. It was published in three fascicles (Nissen 1969: 118). I have seen the cover of the first fascicle which reads “*Entomologia Vicentina. Fascicolo primo che contiene i Coleotteri, gli Ortotteri, i Neurotteri, gli Imenotteri e la prima famiglia dei Lepidotteri*” (pp. 1–144) and is dated 1865. The Coleoptera are on pages 27–97 in the first fascicle; many species are commented or described in footnotes but none appear to be new to science.

#### Distant, William Lucas (London, United Kingdom: 12 November 1845 – 4 February 1922: Wanstead [now part of Greater London], United Kingdom). British entomologist; worked early in his life in a London tannery; made two lengthy visits to the Transvaal; employed by the Trustees of the Natural History Museum at South Kensington, 1899–1920, to curate their collection of Hemiptera; his collection, mainly Hemiptera including over 2500 types, was acquired by the Natural History Museum of London. References. Anonymous (1922); Campion (1922).

**1892**. *A naturalist in the Transvaal. With coloured plates and original illustrations.* R.H. Porter, London. xvi + 277 pp. + 5 pls. (8vo) [DP: 1892 (title page; preface dated February 1892), 9 April 1892 (*Athenaeum*; *Spectator*), April 1892 (*English Cat Books*; *Nat Nov*), 2 May 1892 (*Author*), July 1892 (*Nat Sci*)] MCZ, BHL, GB

The appendix contains a section “Insecta” (pp. 185–262) written by the author, except for some parts (for example Gahan, 1892 [*q.v.*]). The Coleoptera are on pages 187–210 and on plate 1. The new species of Coleoptera described, except those in Gahan’s note, are: *Bolboceras batesii* (p. 191), *Amiantus undosus* (p. 199), *Paroeme gahani* (p. 202), *Tragocephala sulphurata* (p. 202), *Crossotus klugii* (p. 203) by W.L. Distant; *Lycus distanti* (p. 196) by J. Bourgeois; *Hedybius amoenus* (p. 197) by H. S. Gorham; and *Achaenops facialis* (p. 204) and *Menius distanti* (p. 205) by M. Jacoby.

#### Döbner, Eduard Philipp (Meiningen, Thuringia, Germany: 16 November 1810 – 29 December 1890: Aschaffenburg, Bavaria, Germany). German naturalist; professor of zoology and botany in Aschaffenburg (1844–1876). References. Baur (1891); Doebner (1902: 87); Christ (1983).

**1861**. *Handbuch der Zoologie mit besonderer Berücksichtigung derjenigen Thiere, welche in Bezug auf Forst- und Landwirthschaft, sowie hinsichtlich der Jagd vorzüglich wichtig sind. Zweiter Theil: Wirbellose Thiere. Mit in den Text eingedruckten Holzschnitten und mit 14 lithographirten Tafeln.* C. Krebs, Aschaffenburg. viii + [1] + 616 pp. + 14 pls. (8vo) [DP: 1862 (title page; *Vorrede* in the first *Theil* dated September 1861), 12 December 1861 (*Allg Bibl Deutsch*), 14 December 1861 (*Beil Illustr Ztg*), 16 December 1861 (*Allg Ztg*), December 1861 (*Allg Bibl*)] GB

The entire work consists of two volumes, issued simultaneously in 1861 despite the date on the title page. The Coleoptera are on pages 27–213 of the second volume.

#### Dohrn, Carl August (Stettin [currently Szczecin], Poland: 27 June 1806 – 4 May 1892: Stettin, Poland). Prussian entomologist of independent means, particularly interested in Coleoptera; travelled through Europe, North Africa and South America (1831–1837); served as president of the *Entomologischen Vereins* in Stettin (1843–1887); his specimens were placed in the collection of the Museum für Naturkunde in Stettin which was transferred after WWII to the Zoological Institute of the Polish Academy of Sciences in Warsaw. References. Dohrn (1893, P); Hess (1903); Oppenheimer (1993: 6–7).

**1855**. *Catalogus coleopterorum Europae. Herausgegeben vom entomologischen Vereine in Stettin. Fünfte Auflage.* F. Hessenland, Stettin. [2] + 98 + 12 pp. (8vo) [DP: 1855 (title page; *Vorwort* dated January 1855), February 1855 (*Ent Ztg* 16: 64)] CNC

The preface is signed by Carl August Dohrn to whom the work is usually credited.

**1856**. *Catalogus coleopterorum Europae. Herausgegeben vom entomologischen Vereine in Stettin. Sechste Auflage.* F. Hessenland, Stettin. [1] + 92 + 14 pp. (8vo) [DP: 1856 (title page; *Vorwort* dated August 1856), September–October 1856 (*Ent Ztg* 17: 320), December 1856 (*Lit Deutsch Natur Ztg*), July–December 1856 (*Bibliot Hist-Nat*), October–December 1856 (*Cat Muquardt*)] GB

The preface is signed C.A. Dohrn to whom the catalogue is usually credited.

**1858**. *Catalogus coleopterorum Europae. Herausgegeben vom entomologischen Vereine in Stettin. Siebente Auflage.* R. Grassmann, Stettin. [1] + xiii + [2 (Addenda et Emendanda)] + 104 pp. (8vo) [DP: 1858 (title page; *Vorwort* dated May 1858), September 1858 (*Ent Ztg* 19: 324)] SME, GB

The preface is signed C.A. Dohrn to whom the catalogue is usually credited.

#### Dokhtouroff, Vladimir Serghyeevich (1859 – 1890). Russian officer in the Imperial Guard; secretary of the Entomological Society of Russia; his collection of tiger beetles of the world was eventually scattered although a first selection was acquired by Walther Horn [*q.v.*]. Reference. Musgrave (1932: 70).

**1882**. *Species des cicindélides. Essai monographique sur la famille précédé de tables systématiques pour la détermination des genres. Première livraison. Manticorides et mégacéphalides avec 10 planches coloriées. I.* Trenké & Fusnot, St-Pétersbourg. v + 92 pp. + 1 pl. (8vo) [DP: 1882 (title page; preface dated 12 May 1882 [JD])] ANSP

The author intended to publish his *Species des cicindélides* in eight livraisons, each of 100–150 pages with nine or ten colored plates. Only the first livraison was issued and according to Junk (1935: 184) only four copies remain. Dokthouroff apparently burned the other copies because there were too many mistakes. Several new taxa are described in the work. Fleutiaux (1886) published an extensive review of the work.

#### Donndorff [Donndorf], Johann August (Quedlinburg, Saxony-Anhalt, Germany: 23 March 1754 – 22 November 1837: Quedlinburg, Germany). German naturalist, historian and polymath; government advocate; later, mayor of Quedlinburg and inspector of schools. References. Lommel (1877); Adler (2012: 52, P).

**1793**. *Handbuch der Thiergeschichte. Nach den besten Quellen und neuesten Beobachtungen. Zum gemeinnützigen Gebrauch.* Weidmann, Leipzig. xvi + 845 + [1 (Einige Verbesserungen)] pp. (8vo) [DP: 1793 (title page; *Vorrede* dated “Ostermesse, 1793”), 24 August 1793 (*Gött Anz*), December 1793 (*Observ Phys*)] GB

The Coleoptera are on pages 582–621.

**1799**. *Europäische Fauna oder Naturgeschichte der europäischen Thiere in angenehmen Geschichten und Erzählungen für allerley Leser vorzüglich für die Jugend. Angefangen von J.A.E. Goeze, fortgesetzt von Johann August Donndorft. Achter Band. Käfer.* Weidmann, Leipzig. xviii + 892 pp. (8vo) [DP: 1799 (title page), July 1799 (*Litt Ztg*), 17 August 1799 (*Gött Anz*), 14 December 1799 (*Allg Lit Ztg*)] GB

The entire series was published in nine volumes, 1791–1803. The Coleoptera are treated in volume 8.

#### Donovan, Edward (Cork, Ireland: 1768 – 1 February 1837: London, United Kingdom). Anglo-Irish artist and naturalist; wrote several popular books on natural history and botany for which he drew and colored the illustrations himself; apparently ruined by his publishers and died penniless; founded the London Museum and Institute of Natural History in 1807 where he displayed his collections and paintings; his collections were sold at auction in 1818. References. Watkins (1888); Lisney (1960: 257–277); Jackson (1985: 181–189); Gilbert (2004a: 552).

**1792–1813**. *The natural history of British insects; explaining them in their several states, with the periods of their transformations, their food, oeconomy, &c. together with the history of such minute insects as require investigation by the microscope. The whole illustrated by coloured figures, designed and executed from living specimens.* F. and C. Rivington [vols 1–10] / F.C. and J. Rivington [vols 11–16], London. (8vo) CMLE (mf), McG, BHL

*Vol. I.* 80 + [6 (Indices)] pp. + pls 1–36. [DP: 1792 (title page)].

*Vol. II.* 96 + [6] pp. + pls 37–72. [DP: 1793 (title page)].

*Vol. III.* 98 + [7 (Indices, Errata)] pp. + pls 73–108. [DP: 1794 (title page)].

*Vol. IV.* 96 + [5] pp. + pls 109–144. [DP: 1795 (title page)].

*Vol. V.* 110 + [7 (Indices, Errata)] pp. + pls 145–180. [DP: 1796 (title page)].

*Vol. VI.* 86 + [6] pp. + pls 181–216. [DP: 1797 (title page)].

*Vol. VII.* 96 + [7 (Indices)] pp. + pls 217–252. [DP: 1798 (title page)].

*Vol. VIII.* 88 + [5] pp. + pls 253–288. [DP: 1799 (title page)].

*Vol. IX.* 78 + [4 (Indices)] pp. + pls 289–324. [DP: 1800 (title page)].

*Vol. X.* 95 + [7] pp. + pls 325–360. [DP: 1801 (title page)].

*Vol. XI.* viii + 100 + [7 (Indices)] pp. + pls 361–396. [DP: 1806 (title page)]. The date of 1804 listed by Heppner (1982: 89) is incorrect.

*Vol. XII.* 102 + [5] pp. + pls 397–432. [DP: 1807 (title page), 1 September 1807 (*Athenaeum*), July–October 1807 (*Edinb Rev*), October 1807 (*Eclectic Rev*)].

*Vol. XIII.* 72 + [4 (Indexes)] pp. + pls 433–468. [DP: 1808 (title page), January 1809 (*Eclectic Rev*; *Crit Rev*; *Athenaeum*)].

*Vol. XIV.* 90 + [6] pp. + pls 469–504. [DP: 1810 (title page), May 1810 (*British Critic*), 1 June 1810 (*Monthly Mag*), July 1810 (*Eclectic Rev*), August 1810 (*Edinb Rev*)]. The date of April 1809 listed by Evenhuis (1997a: 206) from *The Edinburgh Review* could not be confirmed in this study.

*Vol. XV.* 83 + [6 (Indexes)] pp. + pls 505–540. [DP: 1811 (title page)]. The date of November 1811 listed by Evenhuis (1997a: 206) from *The Edinburgh Review* could not be confirmed.

*Vol. XVI.* 91 + [11] pp. + pls 541–576. [DP: 1813 (title page), 1 January 1814 (*Monthly Mag*; *Edinb Rev*)].

The entire work consists of 16 volumes, 1792–1813. Each volume was published in monthly parts (Evenhuis 1997a: 206) and the dates given here are for the completed volumes. A number of reprintings and editions are known (see Lisney 1960: 258–276).

**1798–1799**. *An epitome of the natural history of the insects of China: comprising figures and descriptions of upwards of one hundred new, singular, and beautiful species; together with some that are of importance in medicine, domestic economy, &c. The figures are accurately drawn, engraved, and coloured, from specimens of the insects; the descriptions are arranged according to the system of Linnæus; with references to the writings of Fabricius, and other systematic authors.* London. [96] pp. + [50] pls. (4to) [DP: 1798 (title page)] MCZ

The text and plates are unnumbered. This work was issued in parts. *The Monthly Magazine* recorded the first number on February 1798 and noticed that it will be completed in 17 monthly numbers. The work was noticed in the section “Books and pamphlets published in August 1799” by *The Edinburgh Magazine*. It was reviewed in the *British Critic* for the month of January 1800. Dates of publication are found on the plates. Eight plates deal with Coleoptera.

[1 April 1798]: *Cerambyx rubus*; *Cerambyx reticulator*; *Cerambyx farinosus*.

[1 May 1798]: *Buprestis vittata*; *Buprestis ocellata*.

[1 May 1798]: *Scarabaeus molossus*; *Scarabaeus seniculus*; *Scarabaeus bucephalus*.

[1 November 1798]: *Curculio chinensis*; *Curculio longipes*; ‒ *barbirostris*; ‒ *verrucosus*; ‒ *squamosus*; ‒ *pulverulentus*; ‒ *perlatus*.

[1 December 1798]: *Scarabaeus midas*; *Scarabaeus nasicornis*; ‒ *cinctus*; ‒ *sacer*; ‒ *leei*.

[1 January 1799]: *Mylabris cichorei*; *Sagra femorata*.

[1 March 1799]: *Cetonia chinensis*; *Melolontha viridis*.

[1 May 1799]: *Curculio pulverulentus*.

Of the 24 species of Coleoptera illustrated in this work, only one (*Curculio chinensis*) represents a new species, although it is a junior primary homonym.

Two subsequent editions were published in London: one on 28 May 1838 (title page), under the title “*Natural history of the insects of China: a new edition with additional observations &c., by J. O. Westwood. The figures drawn from specimens of the insects by E. Donovan*” [BHL], the other in 1842 under the title “*Natural history of the insects of China, containing upwards of two hundred and twenty figures and descriptions, by E. Donovan. A new edition brought down to the present state of the science, with systematic characters of each species, synonyms, indexes, and other additional matter, by J. O. Westwood*” [BHL, USNM]. A German translation by J.G. Gruber was published in Leipzig, in parts in 1802, under the title “*E. Donovan’s Natur-Geschichte der Chinesischen Insekten. Enthaltend gegen hundert neue, besondere und schöne Spezies nach der Natur gezeichnet, und genau Kolorirt, auf den Pflanzen, worauf sie gewöhnlich leben, nebst Beschreibung nach Linnés Ordnung, mit Beziehung auf Fabricius und andere*” (*n.v.*).

Donovan never set foot in China. He obtained most of the information from Sir George Stainton (Earl Macartney) of the Embassy to China, who travelled extensively in China and made collections and observations on natural history, which he conveyed to naturalists in England.

**1800–1804**. *An epitome of the natural history of the insects of India, and the islands in the Indian seas: comprising upwards of two hundred and fifty figures and descriptions of the most singular and beautiful species, selected chiefly from those recently discovered, and which have not appeared in the works of any preceding author. The figures are accurately drawn, engraved, and coloured, from specimens of the insects; the descriptions are arranged according to the system of Linnaeus; with references to the writings of Fabricius, and other systematic authors.* T. Bensley, London. 2 + [58] pp. + [58] pls. (4to) CNC, BHL

This work was issued in 14 parts, 1800–1804, with the pages and plates unnumbered. The publication dates are found on the plates. The Coleoptera are on six plates.

[1 February 1800]: *Curculio regalis*; *Curculio palmarum*.

[1 November 1800]: *Carabus 6 maculatus*; *Carabus 2 maculatus*.

[1 June 1802]: *Buprestis sternicornis*; *Buprestis chrysis*; – *aenea*; – *4 maculata*.

[1 February 1804]: *Scarabaeus atlas*.

[1 February 1804]: *Scarabaeus spinifer*; – *miliaris*; – *koenigii*; *Cetonia histrio*; *Cetonia caerulea*.

[1 June 1804]: *Pausus denticornis*; *Pausus thoracicus*; – *fichtelii*; – *pilicornis*.

Only the four species illustrated on the plate dated 1 June 1804 are new to science.

A second edition of this book was published in 1842, in London, under the title “*Natural history of the insects of India, containing upwards of two hundred and twenty figures and descriptions, by E. Donovan. A new edition, brought down to the present state of the science, with systematic characters of each species, synonyms, indexes, and other additional matter, by J. O. Westwood*” [CMLE]. A facsimile of the second edition was published in Delhi in 1985. The original edition was limited to 250 copies.

**1805**. *An epitome of the natural history of the insects of New Holland, New Zealand, New Guinea, Otaheite, and other islands in the Indian, Southern, and Pacific oceans: including the figures and descriptions of one hundred and fifty-three species of the more splendid, beautiful, and interesting insects, hitherto discovered in those countries, and which for the most part have not appeared in the works of any preceding author. The figures are correctly delineated from specimens of the insects; and with the descriptions are arranged according to the Linnaean system, with reference to the writings of Fabricius and other entomologists.* F.C. & J. Rivington, London. iv + [167] pp. + [41] pls. (4to) [DP: 1805 (title page; dedication dated 20 July 1805), August 1805 (*British Critic*)] CNC, AMNH, GB

The pages, plates and figures of this work are not numbered. The Coleoptera are on eight plates and described on the corresponding text. The plates are dated and the species illustrated are:

[1 January 1805]: *Cetonia australasiae*; – *punctata*; – *frontalis*; – *carinata*; – *dorsalis*; *Melolontha viridi-aenea*; *Lucanus aeneus* [Fab.]; *Lucanus parvus*.

[1 January 1805]: *Chrysomela 18-guttata* [Fab.]; – *brunnea* [Fab.]; – *cyanicornis* [Fab.]; – *cyanipes* [Fab.]; – *crassicornis* [Fab.]; – *nigricornis* [Fab.]; – *didymus* [Fab.]; *Erotylus* [*Cnodulon* in the text] *amethystinus* [Fab.]; – [*Cnodulon* in the text] *bicolor* [Fab.]; – [*Cnodulum* in the text] *smaragdulus* [Fab.].

[1 January 1805]: *Curculio spectabilis* [Fab.]; – *quadri-tuberculatus*; – *sex-spinosus*; *Brachycerus nigro-spinosus*; *Brentus lineatus*; *Lixus bidentatus*; *Rhynchaenus cylindrirostris* [Fab.].

[1 January 1805]^23^: *Lamia vermicularia*; *Lamia obliqua*; *Prionus fasciatus*; *Prionus bidentatus*; *Stenocrus* [*Stenocorus* in the text] *punctatus*; – *semipunctatus*; – *biguttatus*.

[1 January 1805]: *Buprestis variabilis*; – *cancellata*; – *crenata*; – *undulata*; – *splendida*.

[1 January 1805]: *Buprestis grandis*; – *macularia*; –*imperialis* [Fab.]; – *limbata*; – *suturalis*.

[2 February 1805]: *Cerambyx giraffa* [Schreibers]; *Cerambyx fichteli* [*fichtelii* in the text] [Schreibers]; *Prionus lepidopterus* [Schreibers]; *Clytus thoracicus*; – *sex-maculatus*; – *punctulatus*; *Saperda nigro-virens*; *Saperda collaris*.

[1 March 1805]: *Cerapterus macleaii*.

Some copies have an additional title page which reads “*General illustration of entomology: part I. An epitome of the insects of Asia, elucidated in one hundred and fifty plates; with occasional observations, and descriptions after the linnaean and fabrician manner. In three volumes.*” The first volume corresponds to “*An epitome of the natural history of the insects of China*” [1798–1799] and the second to “*An epitome of the natural history of the insects of India*” [1800–1804].

Percheron (1837: 87) mentioned that this work was issued by *cahiers*.

**1823–1827**. *Naturalist’s repository, or monthly miscellany of exotic natural history: consisting of elegantly coloured plates with appropriate scientific and general descriptions of the most curious, scarce, and beautiful productions of nature that have been recently discovered in various parts of the world; and more especially such novelties as from their extreme rarity remain entirely undescribed, or which have not been duly noticed by any preceding naturalists. The whole composed according to the latest improvements in the various departments of the science, and forming collectively a truly valuable compendium of the most important discoveries of quadrupeds, birds, fishes, insects, shells, marine productions, and every other interesting object of natural history, the produce of foreign climates.* W. Simpkin and R. Marshall, London. (8vo) AMNH, BHL

*Vol. II.* [234] pp. + pls xxxvii–lxxii. [DP: 1824 (title page), 1 July 1824 (*New Monthly Mag*), July 1824 (*Edinb Mag*)]. The Coleoptera are on plates lxii, dated 1 December 1823 (*Buprestis jucunda*, *Buprestis amoena*, *Buprestis leucosticta* and *Buprestis pulchella*), and lxx, dated 1 March 1824 (*Buprestis bicolor*).

*Vol. III.* iv + [102] pp. + pls lxxiii–cviii. [DP: 1825 (title page)]. The Coleoptera are on plates lxxxv, dated 1 June 1824 (*Buprestis macleayi* and *Buprestis cardinalis*), and xcv, dated 1 March 1823 (*Curculio croesus*).

The entire work consists of five volumes, 1823–1827, each consisting of 12 issues (numbers). The dates of publication are found on the plates and the date listed for volume 2 is for the completion of the volume. Coleoptera are depicted only on some plates of volumes 2 and 3.

The work was reissued in 1834 under the title “*The naturalist’s repository, or miscellany of exotic natural history, exhibiting rare and beautiful specimens of foreign birds, insects, shells, quadrupeds, fishes, and marine productions; more especially such new subjects as have not hitherto been figured, or correctly described; forming a compendium of the most interesting modern discoveries in zoology. In five volumes, with one hundred and eighty coloured plates*” [McG]. An abridged edition was published under the title “*The naturalist’s repository of exotic natural history: consisting of seventy-two elegantly coloured plates, with appropriate scientific and general descriptions, of the most curious, scarce, and beautiful quadrupeds, birds, fishes, insects, shells, marine productions, and other interesting objects of natural history, the produce of foreign climates. In two volumes*” by the author, and W. Simpkin and R. Marshall, London [MCZ]. There is no date on the title page but the plates are dated 1822–1824.

#### Drury, Dru (London, United Kingdom: 4 February 1725 – 15 January 1804: London, United Kingdom). British silversmith by profession and entomologist; spent a lot of energy and money on his collection of insects which included the specimens he described and named (in the index) in his *Illustrations of natural history* and also the type material of a number of species described by Fabricius; after his death, his collection was sold at auction by King and Lochee on 23–25 May 1805 and the whereabouts of most of his syntypes are unknown. References. Smith (1842: 17–71, P); Cockerell (1922); Weiss (1927); Hayek (1985, 2004); Harvey et al. (1996: 64–65).

**1770–1782**. *Illustrations of natural history. Wherein are exhibited upwards of two hundred and forty figures of exotic insects, according to their different genera; very few of which have hitherto been figured by any author, being engraved and coloured from nature, with the greatest accuracy, and under the author’s own inspection, on fifty copper-plates. With a particular description of each insect: interspersed with remarks and reflections on the nature and properties of many of them. To which is added a translation into French.* B. White, London. (4to) MCZ, BHL, GB

[*Vol. I*]. xxvii + [1] + 130 pp. + 50 pls. [DP: 1770 (title page), 23 April 1770 (Evenhuis 1997a: 211), May 1770 (*Univers Mag*), September 1770 (*Scots Mag*)].

*Vol. II.* vii + 90 + [4 (Indices of volumes I and II)] pp. + 50 pls. [DP: 1773 (title page), 14 May 1773 (Evenhuis 1997a: 211)]. In this volume the words “*two hundred and forty figures*” are changed to “*two hundred and twenty figures*” in the title. Plates 30–35 pertain to Coleoptera.

*Vol. III.* xxvi + 76 + [2 (index)] pp. + 50 pls. [DP: 1782 (title page), 23 July 1782 (Evenhuis 1997a: 212)]. In this volume the words “*two hundred and forty figures*” are changed to “*two hundred figures*” in the title.

As noted by Evenhuis (1997a: 212), the plates were sold as each one was ready. Therefore the dates indicated above are those in which the completed volumes were first sold or noticed.

The ICZN (1957a) ruled in Opinion 474 that the date of publication of the index, published with the second volume of Drury’s work in 1773, containing names formed in accordance with the principles of binominal nomenclature, is the official date of publication of the species described and figured but not so named in the first volume issued in 1770. Although published with the second volume, the index is often bound with the first volume.

#### Dufour, Léon Jean Marie (Saint-Sever, Landes, France: 11 April 1780 – 18 April 1865: Saint-Sever, France). French physician and naturalist; studied medicine in Paris (1799–1806); participated in the Peninsular War (1807–1814) as army doctor; after, returned to Saint-Sever to practice medicine; his collection was bequeathed to his friend Alexandre Laboulbène and is now at the Muséum National d’Histoire Naturelle in Paris. References. Dufour (1888); Léon-Dufour (1983); Duris and Diaz (1987, P); Boone (2003); Gouillard (2004: 26–28, P); Aguilar (2012, P).

**1843**. *Excursion entomologique dans les montagnes de la vallée d’Ossau.* Veronese, Pau. 118 pp. (8vo) [DP: 1843 (title page), 28 August 1843 (*Acad Sci Fr*), 15 November 1843 (*Soc Ent Fr*)] GB

This work is a descriptive catalogue of 768 species of Coleoptera from the Ossau Valley in the French Pyrenees. Many new species are described and none of them are recorded in Sherborn’s *Index Animalium*. The descriptions of all new species were reproduced by Siebold (1849).

The work was also issued in the *Bulletin de la Société des Sciences, Lettres et Arts de Pau* (Année 1843): 5–118.

#### Duftschmid, Caspar Erasmus (Gmunden, Upper Austria, Austria: 19 November 1767 – 17 December 1821: Linz, Upper Austria, Austria). Austrian physician and naturalist; his collection of beetles passed to his son, Johann Baptist Duftschmid, and eventually ended up at the Oberösterreichisches Landesmuseum in Linz. References. Dolezal (1959); Mitter (2003).

**1805–1825**. *Fauna Austriae, oder Beschreibung der österreichischen Insekten für angehende Freunde der Entomologie.* Akademie Buchhandlung, Linz und Leipzig. (8vo) MCZ, GB

*Erster Theil.* xxxvi + 37–311 + [4] pp. [DP: 1805 (title page; *Nachtrag* dated 28 December 1804), 30 September 1805 (*Neue Intell Lit Kunst*), 5 October 1805 (*Allg Lit Ztg*)].

*Zweyter Theil.* viii + 311 pp. [DP: 1812 (title page; *Hochwohlgeborner* dated 16 September 1811), July–December 1812 (*Verz Bücher Landkarten*)].

*Dritter Theil.* 289 pp. [DP: 1825 (title page), January–June 1825 (*Verz Bücher Landkarten*)].

The three volumes pertain to Coleoptera.

#### Duméril, André-Marie-Constant (Amiens, Somme, France: 1 January 1774 – 14 August 1860: Paris, France). French physician and zoologist; professor of anatomy and physiology at the Faculty of medicine in Paris; later, professor of herpetology and ichthyology at the Muséum National d’Histoire Naturelle in Paris; his collection was deposited at the Muséum where it was used for various exhibits in the galleries and has now disappeared. References. Milne-Edwards (1860); Flourens (1866); Adler (1989: 31–32, P); Damkaer (2002: 140–141, P); Jaussaud (2004: 198–200, P); Cambefort (2006: 165).

**1800**. [Tableaux de classification]. Nine folded tables *in*: *Leçons d’anatomie comparée de G. Cuvier, membre de l’Institut national, professeur au Collège de France et à l’École centrale du Panthéon, etc.; recueillies et publiées sous ses yeux par C. Duméril, chef des travaux anatomiques de l’École de Médecine de Paris. Tome I. Contenant les organes du mouvement.* Baudouin, Paris. xxxi + 521 + [1] pp. + 9 folded tables. (8vo) [DP: “An VIII” (title page; p. xxii dated 19 March 1800), 1 April 1800 (*Acad Sci Fr*), 19 April 1800 (Mathews 1920: 6), 22 March–20 April 1800 (*J Phys*), 21 April–20 May 1800 (*Soc Philom*), 30 May 1800 (*Décade*), 20 June–19 July 1800 (*Esprit J*)] CNC (mf), GB

The work includes nine folded tables of classification at the end of the first volume of the series; the “*Huitième tableau. Classification des insectes*” pertains to insects. These tables are the work of Duméril (Duméril 1822: 522). The entire series consists of five volumes (1800–1805). The first two tomes were written by Duméril from the presentations given by Cuvier who reviewed and corrected the text; volumes 3–5 were written by C.L. Duvernoy again from the presentations given by Cuvier. These volumes are usually credited to Cuvier. The five volumes have been placed on the *Official List of Works Approved as Available for Zoological Nomenclature* in Direction 32 (ICZN 1956a) with the endorsement that the Latin names in the systematic tables in volume 1 are to be treated as available names, in so far as they are provided with “indications” for the purposes of Article 25 through the citation of bibliographical references on page xix of the Introduction.

The five volumes were reissued in 1805 [GB]. A reedition of the reissue was published in Brussels in 1969 (*n.v.*). A second edition, corrected and enlarged, was issued in nine volumes in Paris, 1835–1846 [GB, MCZ]. A third edition, consisting of the first three volumes of the second edition (Smith 1993: 172), was issued in Brussels, 1836–1840 [GB].

An English translation of the first two volumes of the series was issued in London, 1802, under the title “*Lectures on comparative anatomy*” (Smith 1993: 172). The tables were apparently published separately as “*Synoptic tables of zoology, exhibiting a classification of animals, according to their internal structure*” (Smith 1993: 173). A German translation of the first two volumes appeared in Braunschweig, 1801–1802, under the title “*Vorlesungen über vergleichende Anatomie von G. Cüvier*” (Smith 1993: 173).

**1805**. *Zoologie analytique, ou méthode naturelle de classification des animaux, rendue plus facile a l’aide de tableaux synoptiques.* Allais, Paris. xxxii + 344 pp. (8vo) [DP: 1806 (title page; dedication dated 20 September 1805), 15 November 1805 (*J Typogr Bibl*), 23 October–21 November 1805 (*J Phys*), 20 January 1806 (*Acad Sci Fr*), January 1806 (*J Méd*)] McG, GB, BHL, ARC

The Coleoptera are on pages 191–236. A second printing of the book was issued in 1806 with an extra page (xxxiii) of errata (Bour 2010; Gregory 2010). The book was translated in German by L.F. von Froriep in 1806 [*q.v.*].

**1823**. *Considérations générales sur la classe des insectes. Ouvrage orné de soixante planches en taille-douce représentant plus de trois cent cinquante genres d’insectes.* F.G. Levrault, Paris. xii + 272 pp. + 60 pls. (8vo) [DP: 1823 (title page; *avant-propos* dated 3 March 1823), 14 April 1823 (*Acad Sci Fr*), 17 May 1823 (*Bibl Fr*)^24^, 5 June 1823 (*Rev Bibl-Brus*), 16 June 1823 (*Monit Univers*), August 1823 (*Not Geb Natur*)] CNC, GB, ARC, BHL, GAL

According to Percheron (1837: 94) the 60 plates in this work are those of the *Dictionnaire des sciences naturelles* [*q.v.*], edited by Georges Frédéric Cuvier, to which text was added.

**1860**. *Entomologie analytique. Histoire générale, classification naturelle et méthodique des insectes a l’aide de tableaux synoptiques. Tome premier. Première partie: contenant l’histoire générale des insectes et celle de l’ordre des Coléoptères.* Firmin Didot Frères, Fils et C^ie^, Paris. xxii + 664 pp. (4to) [DP: 1860 (title page), 11 January 1860 (*Soc Ent Fr*), 21 January 1860 (*Bibl Fr*), February 1860 (*Allg Bibl*)] BHL

The entire work was issued in two volumes, published simultaneously. The Coleoptera are on pages 223–664 of the first volume.

This work was also issued the same year as volume 31 of the *Mémoires de l’Académie des Sciences de l’Institut impérial de France* [DP: 1860 (title page), 11 August 1860 (*Bibl Fr*)].

#### Duncan, James (1804 – 30 November 1861). Scottish clergyman and naturalist; preached in the parish of Teviothead in Scotland. References. Elliot (1868: 322–324); Salmon (2000: 147–148).

**1835**. *The naturalist’s library. Conducted by Sir William Jardine, Bart. Entomology. Vol. II. Beetles.* W.H. Lizars, Edinburgh. vii + pp. 17–269 + 30 pls. (8vo) [DP: 1835 (title page), 16 May 1835 (*Lit Gaz*), 23 May 1835 (*Athenaeum*), 30 May 1835 (*Lancet*), May 1835 (Sheets-Pyenson 1981: 71)] GB

A second title page reads “*The natural history of beetles. Illustrated by thirty-two plates, numerous wood-cuts, with memoir and portrait of Ray.*” The book was reissued in 1843 and 1852 under the title “*The naturalist’s library. Edited by Sir William Jardine, Bart. Vol. XXXV. Entomology. Beetles*” [GB, BHL] by the same publisher, in 1846 under the title of the reissue except that “Vol. XXXV” is changed to “Vol. XXXIII” and the publisher is Henry G. Bohn, London [BHL] and in 1876 by Hardwicke and Bogue in London under the title “*Beetles, British and foreign. Containing a full description of the more important varieties*” [GB].

The work contains the descriptions of several new species of beetles, including *Cetonia discoidea* (p. 220), *Curculio brunneus* (p. 241), *Prionus corticinus* (p. 247), *Lamia tricincta* (p. 254) and *Alurnus marginatus* (p. 260).

The entire series “*The naturalist’s library*” was edited by William Jardine [1800–1874] and published in 40 volumes, 1833–1843, of which 13 are on mammals, 14 on birds, six on fishes and seven on entomology (see Iredale 1951). A German translation (incomplete) was published, 1836–1842, in 21 parts forming ten volumes under the title “*Naturgeschichtliches Cabinet des Thierreichs*” (*n.v.*) in Pesth (Evenhuis 1997a: 394).

#### Dunker [Duncker], Johann Heinrich August (Rathenow, Brandenburg, Germany: 14 January 1763 – 14 June 1843: Rathenow, Germany). German pastor and industrialist; in 1801, co-founded the Royal Privileged Optical Industrial Institute in Rathenow and was the first to produce eye-glasses industrially. Reference. Albrecht (1959).

**1795**. *Entomologisches Bilderbuch für junge Insektensammler. 1tes Heft, welches 41 Insekten enthält.* Johann Christian Hendel, Halle. 32 pp. + 2 pls. (8vo) [DP: 1795 (title page; dedication dated October 1795), 16 January 1796 (*Intell Allg Lit Ztg*)] ANSP

This work contains the description of several insect species, including 28 beetles; none of them are new to science. All are illustrated on the two plates.

#### Eberhard, Johann Peter (Altona [now part of Hamburg], Hamburg, Germany: 2 December 1727 – 17 December 1779: Halle an der Saale, Saxony-Anhalt, Germany). German physician and naturalist; professor of medicine, mathematics and physics at the University of Halle. References. Rochus von Liliencron (1877); Kraus (1888: 50).

**1768**. *Versuch eines neuen Entwurfs der Thiergeschichte. Nebst einem Anhang von einigen seltenen und noch wenig beschriebenen Thieren. Mit Kupfern.* Renger, Halle. [14] + 318 + [1 (Druckfehler)] pp. + 2 pls. (8vo) [DP: 1768 (title page; *Vorbericht* dated 16 April 1768), 12 August 1768 (*Jena Ztg*), 22 August 1768 (*Gött Anz*)] GB, BHL

The Coleoptera are on pages 192–204.

#### Eichhoff, Wilhelm Joseph (Prüm, Rhineland-Palatinate, Germany: 21 November 1823 – 5 December 1893: Strasbourg, Bas-Rhin, France). German forester and entomologist; his collection of scolytines and platypodines is at the Zoologisches Museum, Universität Hamburg while the other Coleoptera are at the Zoologisches Museum der Humboldt-Universität in Berlin and the Musée Zoologique, Université de Strasburg in France. Reference. Altum (1894).

**1880**. *Die europäischen Borkenkäfer. Für Forstleute, Baumzüchter und Entomologen. Mit 109 Original-Abbildungen in Holzschnitt.* Julius Springer, Berlin. vi + [2] + 315 pp. (8vo) [DP: 1881 (title page; *Vorwort* dated March 1880), 20 November 1880 (*Lit Centr Deutsch*), November 1880 (*Nat Nov*), 27 December 1880 (*Zool Anz*), December 1880 (*Zeit Forst Jagd*), July–December 1880 (*Bibliot Hist-Nat*; *Verz Bücher Landkarten*)] CNC, GB, BHL

Despite the title page is dated 1881, this work was issued in 1880. An abridged French translation was published in 1890 in *L’Abeille* 27: 1–151.

#### Eichwald, Karl Eduard Ivanovich von (Jelgava, Latvia: 4 July 1795 – 22 November 1876: Saint Petersburg, Russia). Russian physician, botanist and geologist; professor of zoology and midwifery in Kazan; professor of zoology and comparative anatomy in Vilnius; later, professor of zoology, mineralogy and medicine in Saint Petersburg (1838–1851). References. Embacher (1882); Anonymous (1886b: 632); Adler (2007: 59–60, P).

**1830**. *Zoologia specialis quam expositis animalibus tum vivis, tum fossilibus potissimum Rossiae in universum, et Poloniae in specie, in usum lectionum publicarum in Universitate Caesarea Vilnensi habendarum. Pars altera specialem Podozoorum expositionem continens. Cum icone tituli et duabus aliis lithographicis.* Josephi Zawadzki, Vilnae [= Vilnius]. 323 pp. + 5 pls. (8vo) [DP: 1830 (title page), 9 January 1830 (recto of title page)] GB, BHL

The entire work consists of three volumes, 1829–1831. The insects are in the second volume and the Coleoptera on pages 243–321.

#### Elera, Casto de (Mayorga de Campos, Castilla y León, Spain: 1 July 1852 – 29 August 1903: Manila, Philippines). Spanish clergyman, teacher and naturalist; professor of natural history and director of the museum at the University of Santo Tomás in Manila. References. Díaz Díaz (1988: 20–21); Aparicio and Majuelo (2015: 43).

**1895**. *Catálogo sistemático de toda la fauna de Filipinas conocida hasta el presente, y á la vez el de la coleccíon zoológica del Museo de PP. Dominicos del Colegio-Universidad de Sto. Tomás de Manila, escrito con motivo de la Exposición Regional Filipina. Volumen II. Articulados.* Colegio de Santo Tomás, Manila. [1] + 676 pp. (4to) [DP: 1895 (title page), 17 February 1896 (*Zool Anz*)] BHL, GB

The entire work was published in three volumes, 1895–1896. The first one deals with vertebrates and the third one with mollusks and radiata. The Coleoptera are on pages 1–189 of the second volume.

#### Emmons, Ebenezer (Middlefield, Massachusetts, USA: 16 May 1799 – 1 October 1863: Brunswick, North Carolina, USA). American geologist, naturalist and physician; practiced medicine and surgery in Berkshire County, Massachusetts early in his career; professor of mineralogy and geology at Rensselaer School in Troy, New York (1830–1839); worked for the New York State Geological Survey (1836–1850); state geologist for North Carolina (1851–1863); named the Adirondack Mountains (1838) and Taconic Mountains (1844). References. Marcou (1891, P); Anonymous (1896); Schneer (1970).

[**1855**]. *Agriculture of New-York: comprising an account of the classification, composition and distribution of the soils and rocks, and of the climate and agricultural productions of the state; together with descriptions of the more common and injurious species of insects. Volume V.* C. Van Benthuysen, Albany. viii + 272 pp. + pls A–C, 1–47. (4to) [DP: 1854 (title page; preface dated 25 July 1854), before 26 February 1855 (Evenhuis 1997a: 225), 15 March 1855 (*Norton Lit Gaz*), March 1855 (*Amer J Sci Arts*), 17 April 1855 (*Acad Nat Sci Phila*), April 1855 (*Monthly Lit Adv*)] MCZ, GB, BHL

Although dated 1854 on the title page, this book was apparently issued in 1855 (Barnes 1988: 47). The Coleoptera are on pages 31–138.

The entire “Agriculture of New-York” consists of four volumes (1–3 and 5), 1846–[1855].

#### Endrulat, Bernhard Ferdinand Julius (Berlin, Germany: 24 August 1828 – 17 February 1886: Poznań, Poland). German professor, editor, archivist, and naturalist. References. Schell (1904); Anonymous (2005a).

**1854**. [Endrulat, B. and Tessien, H.] *Zur fauna der Nieder-Elbe. Verzeichniss der bisher um Hamburg gefundenen Käfer. Mit Angabe der Fundorte und sonstigen Bemerkungen.* G.W. Niemeyer, Hamburg. vii + 46 +[1 (Druckfehler)] pp. (8vo) [DP: 1854 (title page; *Vorwort* dated “Frühjahr 1854”), 29 June 1854 (*Allg Bibl Deutsch*), April–June 1854 (*Cat Muquardt*), 31 July 1854 (*Intell Serapeum*), September 1854 (*Zeit Naturwiss*), July–December 1854 (*Bibliot Hist-Nat*)] CMLE, GB, MDZ

This work is a catalogue of the Coleoptera found around the city of Hamburg, Germany, with observations.

#### Erichson, Wilhelm Ferdinand (Stralsund, Mecklenburg-West Pomerania, Germany: 26 November 1809 – 18 November 1849: Berlin, Germany). German physician and entomologist; lecturer and later professor at Humboldt University (then known as the Friedrich-Wilhelm University) in Berlin; his collection is at the Zoologisches Museum der Humboldt-Universität. References. Klug (1850); Ratzeburg (1874: 165–167); Zimmerman (1993: 525, P); Smetana and Herman (2001: 60–61, P).

**1832**. *Genera dyticeorum. Dissertatio inauguralis, quam consensu et auctoritate gratiosi medicorum ordinis in Universitate Literaria Friderica Guilelma pro summis in medicina et chirurgia honoribus rite impetrandis die VII. m. Decembris a. MDCCCXXXII. H.l.q.s. Publice defendet auctor Guilelmus Ferdinandus Erichson Pomeranus. Opponentibus: O. Reich, med. et chir. dd., C. Hopffer, med. et chir. cand., A. Simmons, med. et chir. cand.* Nietack, Berolini [= Berlin]. [2] + 48 + [3 (Vita)] pp. (8vo) [DP: 7 December 1832 (title page)] GB

This thesis was also issued the same year by the same publisher under the title “*Genera dyticeorum. (Dissertatio inauguralis)*” [CMLE, GB].

**1837–1839**. *Die Käfer der Mark Brandenburg. Erster Band.* F. H. Morin, Berlin. viii + 740 pp. (8vo) CNC, GB, BHL

*Erste Abtheilung.* viii + 384 pp. [DP: 1837 (title page; *Vorrede* dated 20 July 1837), 15 September 1837 (*Allg Bibl Deutsch*), 20 September 1837 (*Lit Ztg*), 1 November 1837 (*Soc Ent Fr*), 7 November 1837 (*Frän Merkur*), 30 November 1837 (*Reper Deutsch Lit*), November 1837 (*Bull Litt Étr*), December 1837 (*Acad Imp Sci St-Pétersb*), July–December 1837 (*Verz Bücher Landkarten*)].

*Zweite Abtheilung.* Pp. 385–740. [DP: 1839 (title page), 12 April 1839 (*Allg Bibl Deutsch*), 17 April 1839 (*Lit Ztg*), April–June 1839 (*Foreign Quart Rev*), January–June 1839 (*Verz Bücher Landkarten*)].

**1839–1840**. *Genera et species staphylinorum insectorum coleopterorum familiae. Accedunt tabulae aeneae quinque.* F.H. Morin, Berolini [= Berlin]. viii + 954 pp. + 5 pls. (8vo) CNC, GB, BHL

*Erster Band*. 400 pp. [DP: 1 November 1839 (*Allg Bibl Deutsch*), 6 November 1839 (*Lit Ztg*), October–December 1839 (*Foreign Quart Rev*), November–December 1839 (*Bull Litt Étr*), July–December 1839 (*Verz Bücher Landkarten*)].

*Zweiter Band*. Pp. 401–954 + [1] + v–viii. [DP: 18 September 1840 (*Allg Bibl Deutsch*), 24 September 1840 (*Bibl Blätt*), 30 September 1840 (*Intell Serapeum*), September 1840 (*Bull Litt Étr*), 7 October 1840 (*Lit Ztg*), October–December 1840 (*Foreign Quart Rev*)].

The title page is dated 1840 and the preface (p. viii) signed “Berol. d. III. ante Cal. Aug. MDCCCXL” [= 29 July 1840]). No wrappers of this work were seen during this study.

**1840**. *Entomographien, Untersuchungen in dem Gebiete der Entomologie, mit besonderer Benutzung der Königl. Sammlung zu Berlin. Erstes Heft. Mit zwei Kupfertafeln.* F.H. Morin, Berlin. x + [1 (Inhalt)] + 180 pp. + 2 pls. (8vo) [DP: 1840 (title page; *Vorwort* dated 1 August 1840), 18 September 1840 (*Allg Bibl Deutsch*), 24 September 1840 (*Bibl Blätt*), 30 September 1840 (*Intell Serapeum*), September 1840 (*Ent Ver Stettin*; *Bull Litt Étr*), 7 October 1840 (*Lit Ztg*), October–December 1840 (*Foreign Quart Rev*), December 1840 (*Rev Zool*)] CMLE, GB, BHL

This book, of which only the first *Heft* was issued, contains four chapters, two of them dealing with Coleoptera: *II. Die Pachypoden, eine kleine Gruppe aus der Familie der Melolonthen* (pp. 29–43); *III. Die Malachien der Königl. Sammlung in Berlin* (pp. 44–134).

**1841**. Ueber die Insecten von Algier mit besonderer Berücksichtigung ihrer geographischen Verbreitung. Pp. 140–194 *in*: *Reisen in der Regentschaft Algier in den Jahren 1836, 1837 und 1838 von D. Moritz Wagner. Nebst einem naturhistorischen Anhang und einem Kupferatlas. Dritter Band.* Leopold Voss, Leipzig. xviii + [2 (Druckfehler)] + 296 pp. [+ 17 associated pls]. (8vo–text; Folio–plates) [DP: 1841 (title page; page xvi dated 10 September 1840), 6 February 1841 (Evenhuis 1997b: 795), 12 February 1841 (*Allg Bibl Deutsch*), 17 February 1841 (*Lit Ztg*), 20 February 1841 (*Intell Ztg Welt*), 13 April 1841 (*Allg Ztg*), April 1841 (*Rev Zool*), January–June 1841 (*Verz Bücher Landkarten*)] MCZ, BHL, GB

Another title page of this volume reads “*Bruchstücke zu einer Fauna der Berberei, mit besonderer Rücksicht auf die geographische Verbreitung der Thiere am becken des Mittelmeeres nacr den von Moritz Wagner in der Regentschaft Algier gesammelten Materialien von J.F. Brandt in St. Petersburg, M. Erdl in München, W.F. Erichson in Berlin, C.L. Koch in Regensburg, H. Nathusius in Hundisburg, E.A. Rossmaessler in Tharandt, H. Schlegel in Leyden, A. Wagner in München und R. Wagner in Göttingen.*”

The entire report of the voyage was published in three volumes and an atlas of 17 plates, published simultaneously in 1841. The insects are treated only in the last volume and depicted on plates 7–9.

**1845–1848**. *Naturgeschichte der Insecten Deutschlands. Erste Abtheilung. Coleoptera. Dritter Band.* Nicolai, Berlin. vi + 968 + [1 (Berichtigungen)] pp. (8vo) CNC, BHL, GB

The volume was published in six *Hefte*: **1**: (pp. 1–160) 1845 (wrapper; preliminaries [recto of wrapper] dated 28 May 1845), 15 October 1845 (*Intell Serapeum*), 21 November 1845 (*Leip Reper*), July–December 1845 (*Verz Bücher Landkarten*); **2**: (pp. 161–320) 1845 (wrapper), 15 October 1845 (*Intell Serapeum*), October 1845 (*Intell Allg Lit Ztg*), 21 November 1845 (*Leip Reper*), July–December 1845 (*Verz Bücher Landkarten*); **3**: (pp. 321–480) 1846 (wrapper), 13 August 1846 (*Allg Bibl Deutsch*), 4 September 1846 (*Leip Reper*), July–December 1846 (*Verz Bücher Landkarten*); **4**: (pp. 481–640) 1847 (wrapper), 6 October 1847 (*Lit Ztg*), 21 October 1847 (*Allg Bibl Deutsch*), October 1847 (*Intell Allg Lit Ztg*), 24 December 1847 (*Leip Reper*); **5**: (pp. 641–800) 1847 (wrapper), 20 January 1848 (*Allg Bibl Deutsch*; *Bibl Univers*), 31 January 1848 (*Intell Serapeum*), 11 February 1848 (*Leip Reper*); **6**: (pp. 801–968 + vi + [1]) 1848 (wrapper; *Vorrede* dated May 1848), 2 November 1848 (*Allg Bibl Deutsch*; *Lit Ztg*), 15 November 1848 (*Intell Serapeum*), 15 December 1848 (*Publ Circ*).

**1849**. Insecten. Pp. 553–617 *in*: *Reisen in Britisch-Guiana in den Jahren 1840–1844. Im Auftrag S^r.^ Mäjestat des Königs von Preussen ausgeführt von Richard Schomburgk. Nebst einer Fauna und Flora Guiana’s nach Vorlagen von Johannes Müller, Ehrenberg, Erichson, Klotzsch, Troschel, Cabanis und Andern. Mit Abbildungen und einer Karte von Britisch-Guiana aufgenommen von Sir Robert Schomburgk. Dritter Theil.* J.J. Weber, Leipzig. viii + [1] + pp. 533–1260 + [1 (Berichtigungen)]. (4to) [DP: 1848 (title page), 10 March 1849 (Evenhuis 1997b: 704), 13 March 1849 (Whitehead et al. 1988: 530), 22 March 1849 (*Lit Ztg*), 31 March 1849 (*Intell Serapeum*), 12 April 1849 (*Blätt Lit Unterhalt*), 15 May 1849 (*Publ Circ*), January–June 1849 (*Verz Bücher Landkarten*)] MANL, ARC, GB, BHL

Several recent authors, including Whitehead et al. (1988: 538), Evenhuis (1997b: 704) and Dickinson et al. (2011: 143), dated this book 1849 based on the recording dates found in journals; I follow them.

An additional title page of this work reads “*Versuch einer Fauna und Flora von Britisch-Guiana. Nach Vorlagen von Johannes Müller, Ehrenberg, Erichson, Klotzsch, Troschel, Cabanis und Andern. Systematisch bearbeitet von Richard Schomburgk.*” The entire expedition report consists of three volumes, 1847–1849. The Coleoptera are in the third volume on pages 555–579.

#### Escherich, Karl Leopold (Schwandorf, Bavaria, Germany: 28 September 1871 – 22 November 1951: Kreuth, Bavaria, Germany). German entomologist; professor of applied zoology at the University of Munich. References. Anonymous (1952, P); Saalas (1952, P); Sachtleben (1952).

**1897**. *Bestimmungs-Tabelle der europäischen Coleopteren. Meloidae. II. Theil: Zonitidae. XXXVI. Heft.* Brünn. 39 pp. (8vo) [DP: 1897 (title page), 23 June 1897 (*Soc Ent Fr*), June 1897 (*Nat Nov*)] SME

This work was also issued in *Verhandlungen des Naturforschenden Vereins in Brünn* 35 [1896]: 96–133 [DP: 1897 (title page), January 1898 (*Nat Nov*)].

#### Eschscholtz, Johann Friedrich Gustav von (Tartu, Estonia: 1 November 1793 – 19 May 1831: Tartu, Estonia). Russian physician and naturalist; one of the 37 men on board of the Russian circumnavigational expeditionary ship *Rurik* during 1815–1818 under the command of Otto von Kotzebue; upon his return, appointed professor of anatomy and a few years later, director of the zoological museum of the University of Tartu; in 1823–1826, participated in another expedition under the command of Kotzebue on the ship *Predpriyatie*; Kotzebue named one island in the Marshall Islands “Eschscholtz Atoll” which was renamed “Bikini Atoll” in 1946; his collection and types are at the Zoologisches Museum, Moscow State University. References. Essig (1931: 617–622, P); Lukina (1975); Sterling (1997); Adler (2007: 58–59, P).

**1822**. *Entomographien. Erste Lieferung. Mit zwei illuminirten Kupfertafeln.* G. Reimer, Berlin. 128 + iii pp. + 2 pls. (8vo) [DP: 1822 (title page; preliminaries dated 24 May 1822), July–December 1823 (*Verz Bücher Landkarten*)] MCZ, ARC, BHL, GB

This was the only part published. The text was also issued in 1823 in *Naturwissenschaftliche Abhandlungen aus Dorpat* 1: 57–186 [DP: July–December 1823 (*Verz Bücher Landkarten*)]. A French translation was published in 100 copies, 1835, by Lequien fils, Paris, under the title “*Oeuvres entomologiques de Eschscholtz. Tome premier. Entomographien. Berlin. 1822*” as part of the series *Bibliothèque entomologique, réimpression a petit nombre des ouvrages entomologiques devenus fort rares ou publiés dans les recueils académiques*.

**1829–1833**. *Zoologischer Atlas, enthaltend Abbildungen und Beschreibungen neuer Thierarten, während des Flottcapitains von Kotzebue zweiter Reise um die Welt, auf der Russisch-Kaiserlichen Kriegsschlupp* Predpriaetië *in den Jahren 1823–1826.* G. Reimer, Berlin. (Folio) McG, GB, BHL

*Erstes Heft.* iv + 17 pp. + pls 1–5. [DP: 1829 (title page; preliminaries dated May 1829), January–June 1830 (*Verz Bücher Landkarten*), 10 July 1830 (*Gött Anz*)].

*Zweites Heft.* 13 pp. + pls 6–10. [DP: 1829 (title page), July–December 1830 (*Verz Bücher Landkarten*)^25^].

*Drittes Heft.* 18 pp. + pls 11–15. [DP: 1829 (title page), July–December 1830 (*Verz Bücher Landkarten*)^25^].

*Viertes Heft.* 19 pp. + pls 16–20. [DP: 1831 (title page), April 1831 (*Bull Litt Étr*), January–June 1831 (*Verz Bücher Landkarten*), November 1831 (*J Pract Heil*), July–December 1831 (*Verz Derj Bücher*)].

*Fünftes Heft, herausgegeben von D. Martin Heinrich Rathke. Mit dem Bildnisse der Dr. Eschscholtz.* viii + 28 pp. + pls 21–25. [DP: 1833 (title page), 28 October 1833 (*Lit-Blatt*), 26 November 1833 (*Intell Ztg Welt*), July–December 1833 (*Verz Bücher Landkarten*)].

#### Esmarch, Heinrich Peter Christian (Ulsnis, Schleswig-Holstein, Germany: 21 February 1745 – 8 May 1830: Schleswig, Schleswig-Holstein, Germany). Rector of the Royal Cathedral School of Schleswig. Reference. Prahl (1890: 16).

**1787**. *Anfangsgründe der Naturgeschichte, welche zugleich zur Uebung in der lateinischen Sprache dienen können.* Korte, Flensburg und Leipiz. [4] + 751 pp. (8vo) [DP: 1787 (title page; preliminaries dated 20 September 1786)] GB

The Coleoptera are on pages 657–669.

#### Esper, Eugen Johann Christoph (Wunsiedel, Bavaria, Germany: 2 June 1742 – 27 July 1810: Erlangen, Bavaria, Germany). German zoologist with special interest in Lepidoptera; professor of zoology at the University of Erlangen; the journal *Esperiana, Buchreihe zur Entomologie*, created in 1990, commemorates his name and work; his collection is at the Zoologische Staatssammlung des Bayerischen Staates in Munich. References. Rosenhauer (1877); Eisinger (1919b); Willmann (2011: 154–155).

**1784**. *Naturgeschichte im Auszuge des Linnéischen-Systems mit Erklärung der Kunstwörter und dem Verzeichniss sämtlicher Geschlechter und Gattungen nebst Bemerkung der europäischen und innländischen Arten, wie auch sieben zur Kenntniss derselben dienenden Kupfertafeln zum Gebrauch academischer Vorlesungen.* Gabriel Nicolaus Raspe, Nürnberg. [23] + 740 + [4 (Druckfehler)] pp. + 7 pls. (8vo) [DP: 1784 (title page; dedication dated 16 April 1784), 26 November 1784 (*Nürnb Ztg*), 3 February 1785 (*Gött Anz*)] GB

The Coleoptera are on pages 174–195.

#### Étienne, Jean Auguste Célestin [Frère Ogérien] (Gresse-en-Vercors, Isère, France: 9 December 1825 – 15 December 1869: New York City, New York, USA). French brother, naturalist and geologist; director of the Christian School of Lons-le-Saunier in the Jura department; made a tour of inspection to the United States where he died. Reference. Girardot (1895).

**1863**. *Histoire naturelle du Jura et des départements voisins, ouvrage couronné par trois médailles d’or et une médaille de vermeil. Tome III. Zoologie vivante.*Victor Masson et Fils, Paris. xx + 570 pp. (8vo) [DP: 1863 (title page; dedication dated 24 March 1862), 16 May 1863 (*Bibl Fr*), January–June 1863 (*Bibliot Hist-Nat*), June 1863 (*Allg Bibl*)] GB

The Coleoptera are on pages 382–414.

#### Everts, Edouard Jacques Guillaume (The Hague, Netherlands: 12 May 1849 – 9 June 1932: The Hague, Netherlands). Dutch educator and coleopterist; secondary school teacher in The Hague (1874–1909); his collection of European Coleoptera is at the Instituut voor Taxonomische Zoologie, Universiteit van Amsterdam, while his collection of Dutch Coleoptera is at the National Natuurhistorisch Museum in Leiden. Reference. Oudemans (1933, P).

**1875**. *Lijst der in Nederland voorkomende schildvleugelige Insecten (Coleoptera).* Martinus Nijhoff, ’s Gravenhage. vii + 116 pp. (8vo) [DP: 1875 (title page; *Voorrede* dated April 1875), July 1875 (*Nederl Bibl*), July–December 1875 (*Bibliot Hist-Nat*)] GB

This is a list of Coleoptera found in Netherlands with localities. Two supplements were published in *Tijdschrift voor Entomologie* 20 [1876–77]: 168–185 and 24 [1880–81]: cxxix–clx.

**1879**. *Lijst der Nederlandsche Coleoptera, gerangschikt volgens Stein en Weise’s Catalogi Coleopterorum Europae, 2e editie, Berlijn 1877*. Den Haag [= The Hague]. 21 pp. (8vo) [DP: 1 October 1879 (title page)] GB

A second edition of 27 pages (*n.v.*) was published in 1888 (Derksen and Scheiding 1963: 695).

**1898–1903**. *Coleoptera Neerlandica. De schildvleugelige insecten van Nederland en het aangrenzend gebied.* Martinus Nijhoff, ’s Gravenhage. (8vo) CNC

*Eerste Deel. Met 62 houtsneêfiguren in den tekst.* viii + 677 + [1 (Verbeteringen)] pp. This volume was issued in two parts: **1**: (viii + 368 pp.) 1898 (title page; *voorrede* dated March 1898), 11 July 1898 (*Bibl Zool*), August 1898 (*Nat Nov*), 25 November 1898 (*Wien Ent Ztg* 17: 256); **2**: (pp. 369–677 + [1]) 1899 (wrapper), 27 March 1899 (*Bibl Zool*), March 1899 (*Nat Nov*), 10 June 1899 (*Wien Ent Ztg* 18: 196).

*Tweede Deel. Met 8 platen en 62 houtsneêfiguren in den tekst benevens een literatuur-overzicht.* iv + 796 + [2 (Verbeteringen)] pp. + 8 pls. This volume was issued in two parts: **1**: (400 pp. + 8 pls), 1901 (wrapper), 21 December 1901 (*Nederl Spectator*), 31 December 1901 (*Nederl Bibl*), January 1902 (*Nat Nov*); **2**: (pp. 401–796 + iv + [2]), 1903 (wrapper; *voorrede* dated March 1903), 4 April 1903 (*Nederl Spectator*), July 1903 (*Nat Nov*).

A third volume was published in 1922 and contains 668 pages.

#### Fabre, Jean-Henri Casimir (Saint-Léons, Aveyron, France: 22 December 1823 – 11 October 1915: Sérignan-du-Comtat, Vaucluse, France). Celebrated autodidact French entomologist, poet and educator; taught at primary school of Carpentras (Vaucluse) (1842–1848); professor of physics at the Imperial College d’Ajaccio in Corsica (1849–1852); professor of physics and chemistry at the Imperial Lyceum d’Avignon (1853–1871); retired at Orange (1871–1879) and Sérignan-du-Comtat (1879–1915) where he wrote textbooks and popular scientific works and studied insect behavior. References. Legros (1913); Delange (1981, P); Cambefort (2002, P); Delage (2005, P).

[**1872**]. *Faune avignonaise. 1^er^ fascicule. Insectes coléoptères observés aux environs d’Avignon.* Fr. Séguin ainé, Avignon. [3] + 162 pp. (8vo) [DP: 1870 (title page), 26 October 1872 (*Bibl Fr*), July–December 1872 (*Bibliot Hist-Nat*)] GB

Despite the date on the title page, this book seems to have been released only in 1872, apparently interrupted by the War of 1870 (Rowland-Brown 1915: 271). Only the first fascicle was issued. One new species of Coleoptera is described in this work, *Larinus leuzeae* (p. 109).

#### Fabricius, Johann Christian (Tønder, Sønderjylland, Denmark: 7 January 1745 – 3 March 1808: Kiel, Schleswig-Holstein, Germany). Danish entomologist and economist; at the age of 17, sent by his father to Uppsala and for the next two years studied under Linnaeus which profoundly influenced him; travelled across Europe for the next few years, attending lectures, studying collections, and collecting insects; returned to Copenhagen in 1769 and was appointed professor of economics at the University; accepted the professorship of natural history, economy and finance at Kiel in 1775; most specimens of Fabricius’ collection are now at the Zoologisk Museum, University of Copenhagen. References. Latreille (1808); Hope (1845); Ratzeburg (1874: 173–175); Papavero (1971: 25–31); Zimmerman (1993: 530, P); Smetana and Herman (2001: 61–62, P).

**1775**. *Systema entomologiae, sistens insectorvm classes, ordines, genera, species, adiectis synonymis, locis, descriptionibvs, observationibvs.* Kortii, Flensbvrgi et Lipsiae [= Flensburg & Leipzig]. [32] + 832 pp. (8vo) [DP: 1775 (title page; dedication dated 21 November 1774), 17 April 1775 (ICZN 1958: 4), 9 September 1775 (*Erlang Anmer Nachr*), 24 November 1775 (*Jena Ztg*)] CNC, GB

In Opinion 516, the ICZN determined that the date of publication of this work is “Easter Monday, 17th April 1775.” The ICZN decision is based on Fabricius’ own statement (see Hope 1845: ix) that the book appeared at the Leipzig Easter Book Fair in 1775. Evenhuis (2014a: 4) pointed out that the Fair was in fact presented on 7 May 1775.

[**1777**]. *Genera insectorvm eorvmqve characteres natvrales secvndvm nvmervm, figvram, sitvm et proportionem omnivm partivm oris adiecta mantissa speciervm nvper detectarvm.* Mich. Friedr. Bartsch, Chilonii [= Kiel]. [16] + 310 pp. (8vo) [DP: n.d. (title page; *prolegomena* dated 26 December 1776), October 1777 (*Allg Verz Bücher*)] CNC, ARC, GB, BHL

This book is sometimes dated 1776 but considering the date of the preliminaries, I agree with many bibliographers that it was probably published in 1777. Percheron (1837: 106) mentioned a second edition published in 1790 in Kiel but this could not be corroborated in this study (see also Hagen 1862: 220).

**1778**. *Philosophia entomologica sistens scientiae fvndamenta adiectis definitionibvs, exemplis, observationibvs, advmbrationibvs*. Carol. Ernest. Bohn, Hambvrgi et Kilonii [= Hamburg & Kiel]. [7] + [1 (Conspectvs)] + 178 pp. (8vo) [DP: 1778 (title page; *praefatio* dated 20 February 1778), August 1778 (*Allg Verz Bücher*)] CNC, GB, BHL

**1779**. *Reise nach Norwegen mit Bemerkungen aus der Naturhistorie und Oekonomie.* Carl Ernst Bohn, Hamburg. lxiv + 388 pp. (8vo) [DP: 1779 (title page; *Vorrede* dated 6 August 1779), 27 December 1779 (*Wöchentl Nachr*), 5 February 1780 (*Gött Anz*)] GB

A French translation was published in Paris, 1802, under the title “*Voyage en Norwège, avec des observations sur l’histoire naturelle et l’économie, traduit de l’Allemand de Jean-Chrétien Fabricius*” [GB]. A Dutch translation was issued in Amsterdam, 1781, under the title “*Reize naar Noorwegen, nevens aanmerkingen uit de natuurlijke historie en oeconomie*” (*n.v.*).

According to Evenhuis (1997a: 246), this work was also issued in 1780 in the second volume of *Bibliothek der neuesten Reisebeschreibungen* (*n.v.*).

**1781–1782**. *Species insectorvm exhibentes eorvm differentias specificas, synonyma avctorvm, loca natalia, metamorphosin adiectis observationibvs, descriptionibvs.* Carol. Ernest. Bohn, Hambvrgi et Kilonii [= Hamburg & Kiel]. (8vo) CNC, GB, BHL

*Tom. I.* viii + 552 pp. [DP: 1781 (title page; *lectori* dated 24 September 1780), March 1781 (*Allg Verz Bücher*), 17 November 1781 (*Gött Anz*)].

*Tom. II.* 494 pp. [DP: 1781 (title page)^26^, May 1782 (*Allg Verz Bücher*), 28 September 1782 (*Gött Anz*)]. This volume does not pertain to Coleoptera except that the “*Appendix specierum nuper detectarum*” (pp. 495–510), which contains the description of several new species, including 31 species of beetles, is often bound with it. Fabricius, in his autobiography (see Hope 1845: xi), mentioned that his *Systema insectorum* appeared in 1782. So likely the second volume was published in 1782 despite the title page bears the date 1781.

*Appendix specierum nuper detectarum*. Pp. 495–510. [DP: 28 September 1782 (*Gött Anz*)]. The *Göttingische gelehrte Anzeigen* noticed on 28 September 1782 the publication of the second tome with 510 pages, indicating that this appendix was included. However, the second tome is often cited as originally published with only 494 pages (e.g., Hagen 1862: 220; Evenhuis 1997a: 247) and at least one copy seen [GB] had only the first 494 pages. Possibly two different printings were done on different dates, one without the appendix, the other with it. I have not seen notification for the *Appendix synonymorum* (pp. 511–514 [numbered [111]–114]) and the *Index generum* (pp. 515–517) which are often also bound with the second tome.

**1787**. *Mantissa insectorvm sistens eorvm species nvper detectas adiectis characteribvs genericis, differentiis specificis, emendationibvs, observationibvs. Tom. I.* Christ. Gottl. Proft, Hafniae [= Copenhagen]. xx + 348 pp. (8vo) [DP: 1787 (title page; *lectori* dated 3 February 1787), June 1787 (*Minerva*), 14 September 1787 (*Allg Lit Ztg*), 23 February 1788 (*Gött Anz*)] CNC, GB, BHL

The entire work consists of two volumes issued in 1787 on different dates. The title of the second volume is different from that of the first and for that reason is listed separately (see next entry).

**1787**. *Mantissa insectorvm sistens species nvper detectas adiectis synonymis, observationibvs, descriptionibvs, emendationibvs. Tom. II.* Christ. Gottl. Proft, Hafniae [= Copenhagen]. 382 pp. (8vo) [DP: 1787 (title page), December 1787 (*Minerva*), 19 January 1788 (*Gött Anz*), April 1788 (*J Méd*)] CNC, GB, BHL

This volume does not pertain to beetles except for the *Appendix* (pp. 375–382) where 21 new species of beetles are described.

**1792–1794**. *Entomologia systematica emendata et aucta. Secundum classes, ordines, genera, species adjectis synonimis, locis, observationibus, descriptionibus.* Christ. Gottl. Proft, Hafniae [= Copenhagen]. (8vo) CNC, GB

*Tom. I.* [*Pars I*]. xx + 330 pp. [DP: 1792 (title page; p. x dated 30 March 1792), 22 December 1792 (*Gött Anz*), 15 May 1793 (*Allg Lit Ztg*)].

*Tom. I. Pars II*. 538 pp. [DP: n.d. (title page), in or before December 1792 (see remark below), 4 May 1793 (*Gött Anz*), 15 May 1793 (*Allg Lit Ztg*)]. As discussed by Löbl and Smetana (2011: 21), Hellwig (1792: 408) cited the correct page and number for the genus *Lyctus* in the second part of Fabricius’ book, clearly suggesting that he had Fabricius’ book in front of him. The cover of the issue for Hellwig’s paper in the journal *Neuestes Magazin für die Liebhaber der Entomologie* bears the date “Dezember 1792.” In addition, the cover of the journal (available on GB) has a text dated “Stralsund im December 1792” which mentioned that the second part of Fabricius’ *Entomologia systematica* is published. Therefore, it is assumed that Fabricius’ second part of the first volume of his *Entomologia systematica* was published in or before December 1792.

*Tom. IV.* [6] + 472 + [5 (Index generum)] pp. [DP: 1794 (title page), 26 January 1795 (*Gött Anz*)]. Several Coleoptera species are described in the “*Appendix specierum nuper detectarum*” (pp. 435–462).

The entire work was published in six parts forming four volumes, 1792–1794. The Coleoptera are in the first and fourth volumes.

**1796**. *Index alphabeticus in J.C. Fabricii Entomologiam Systematicam, emendatam et auctam, ordines, genera et species continens.* Proft et Storch, Hafniae [= Copenhagen]. 175 pp. (8vo) [DP: 1796 (title page), 5 May 1797 (*Allg Lit Ztg*), 20 May 1797 (*Gött Anz*)] CMLE, GB, BHL

This is an index to Fabricius’ *Entomologia Systematica* (1792–1794). A few replacement names, such as *Carabus actaeon* (p. 34), are introduced.

**1798**. *Supplementum entomologiae systematicae.* Proft et Storch, Hafniae [= Copenhagen]. [2] + 572 pp. (8vo) [DP^27^: 1798 (title page; preliminaries dated 10 February 1798), 14 July 1798 (*Intell Allg Lit Ztg*)] CNC, GB, BHL

**1799**. *Index alphabeticus in J.C. Fabricii Supplementum Entomologiae Systematicae, ordines, genera et species continens.* Proft et Storch, Hafniae [= Copenhagen]. 52 + [1] pp. (8vo) [DP: 1799 (title page), 12 December 1799 (*Gött Anz*)] CNC, GB, BHL

This is an index to Fabricius’ *Supplementum Entomologiae Systematicae* (1798). There seem to be no replacement names introduced.

**1801**. *Systema elevtheratorvm secvndvm ordines, genera, species: adiectis synonymis, locis, observationibvs, descriptionibvs.* Bibliopolii Academici Novi, Kiliae [= Kiel]. (8vo) CNC, GB

*Tomvs I*. xxiv + 506 pp. [DP: 1801 (title page; p. x dated 10 April 1801), 10 August 1802 (*Allg Lit Ztg*), 23 October–21 November 1802 (*J Lit Fr*)].

*Tomvs II*. 687 pp. [DP: 1801 (title page), 10 August 1802 (*Allg Lit Ztg*), 23 October–21 November 1802 (*J Lit Fr*)].

The “*Index genervm et speciervm*” in the second volume (pp. 611–687) was also issued separately in 1802 under the title “*Index genervm et speciervm Systematis Elevtheratorvm*” [GB]. Another edition of the index was issued in 1803, under the title “*Index alphabeticvs in I.C. Fabricii Systema Elevtheratorvm genera et species continens*,” by C.G. Fleckeisen in Helmstedt [CMLE].

A facsimile of Fabricius’ *Systema Eleutheratorum* was published in 1970 by Asher in Vaals.

#### Fabricius, Otto (Rudkøbing, Fyn, Denmark: 6 March 1744 – 20 May 1822: Copenhagen, Denmark). Danish clergyman, ethnographer and naturalist; at the age of 24 went to the west coast of Greenland to evangelize the Inuits and stayed for five and a half years, making observations on the habits of the Eskimos and the fauna; upon his return in 1773, appointed bishop in various places in Norway and Denmark, including Copenhagen from 1789 to the time of his death. References. Anonymous (1824a); Jensen (1923; 1932); Kapel (2005, P).

**1780**. *Favna Groenlandica, systematice sistens animalia Groenlandiae occidentalis hactenvs indagata, qvoad nomen specificvm, triviale, vernacvlvmqve; synonyma avctorvm plvrivm, descriptionem, locvm, victvm, generationem, mores, vsvm, captvramqve singvli, provt detegendi occasio fvit, maximaqve parte secvndvm proprias observationes.* Ioannis Gottlob Rothe, Hafniae et Lipsiae [= Copenhagen & Leipzig]. xvi + 452 pp. + 1 pl. (8vo) [DP: 1780 (title page; *ratio opvscvli* dated 12 May 1779), 13 August 1780 (Evenhuis 1997a: 251), 25 November 1780 (*Gött Anz*)] CNC, BHL, GB

#### Fairmaire, Léon (Paris, France: 20 June 1820 – 1 April 1906: Paris, France). French public servant and entomologist with a special interest in Coleoptera; most of his specimens are in the Muséum National d’Histoire Naturelle in Paris. References. Léveillé (1908, P); Smetana and Herman (2001: 63–64, P); Gouillard (2004: 49–50, P); Cambefort (2006: 168–169, P); Frank (2008: 1406–1407).

**1854–1856**. [Fairmaire, L. and Laboulbène, A.] *Faune entomologique française ou description des insectes qui se trouvent en France. Coléoptères. Tome premier.* Deyrolle, Paris. xxxv + 665 pp. (16mo) CNC, GB

This is the only volume of the series that was published. It was issued in three livraisons (the dates of publication are listed on page 660 in the book): **1**: (pp. 1–180) July 1854, 22 July 1854 (*Bibl Fr*); **2**: (pp. 181–370) February 1855; **3**: (pp. 371–665) June 1856, 2 August 1856 (*Bibl Fr*). The preface is dated 26 July 1854 (p. x).

**1860–1861**. [Fairmaire, L. and Germain, P.] *Coleoptera chilensia.* Félix Malteste et C^e^, Paris. (8vo) NCSU, YB (ph)

[*1*]. 8 pp. [DP: 15 June 1860 (p. 8), 7 July 1860 (*Bibl Fr*)].

*II*. 8 pp. [DP: 1 June 1861 (p. 8)].

The following taxa are described in the first part: Feronia (Platysma) convexipennis (p. 1); Feronia (Tapinopterus) sinuatipennis (p. 1); *Tropopsis unicolor* (p. 1); *Mordella rufo-axillaris* (p. 1); *Tetraonyx circumseptus* (p. 1); *Tetraonyx nitidipennis* (p. 2); *Anthicoxenus* (p. 2); *Anthicoxenus nigro-plagiatus* (p. 2); *Bolitophagus costulatus* (p. 2); *Adelium sulcatulum* (p. 2); *Anthicus lagenicollis* (p. 2); *Anthicus maculosus* (p. 3); *Anthicus semirufus* (p. 3); *Anthicus crux* (p. 3); *Anthicus holoxanthus* (p. 3); *Anthicus nigro-femoratus* (p. 3); *Anthicus planicollis* (p. 3); *Lagrioida* (p. 3); *Lagrioida rufula* (p. 4); *Lagrioida obscurella* (p. 4); *Meloe anthracinus* (p. 4); *Monotoma picipes* (p. 4); *Otideres denticulatus* (p. 4); *Heilipus loesicollis* (p. 4); *Heilipus tuberosus* (p. 4); *Brontes integricollis* (p. 5); *Callideriphus clathratus* (p. 5); *Cardiophorus humeralis* (p. 5); *Pyrophorus conicicollis* (p. 5); *Cryptohypnus nivalis* (p. 5); *Cryptohypnus andicola* (p. 5); *Campyloxenus* (p. 6); *Campyloxenus pyrothorax* (p. 6); *Homalocerus argus* (p. 6); *Homalocerus albidovarius* (p. 6); *Homalocerus balteatus* (p. 6); *Rhynchites violaceipennis* (p. 6); *Adioristus albostrigosus* (p. 7); *Arhenodes perforatus* (p. 7); *Adioristus crassirostris* (p. 7); *Adioristus praelongus* (p. 7); *Adioristus gonoderus* (p. 7); *Listroderes ? percostatus* (p. 8); *Stigmodera cyanicollis* (p. 8); *Chrysobothris bothrideres* (p. 8).

The following taxa are described in the second part: *Feronia orobia* (p. 1); *Lioschema* (p. 1); *Lioschema nigricolle* (p. 1); *Lioschema rubrovarium* (p. 1); *Cryptarcha subcaudata* (p. 1); *Dorcus pallido-cinctus* (p. 2); *Bolboceras nasutus* (p. 2); *Bolboceras binasutus* (p. 2); *Bolboceras distinguendus* (p. 2); *Lucidota bifenestrata* (p. 2); *Lucidota hoemorrhoa* (p. 2); *Lucidota ? subulipennis* (p. 3); *Silis crassicornis* (p. 3); *Thanasimus vittula* (p. 3); *Thanasimus semirufus* (p. 3); *Thanasimus parallelus* (p. 3); *Clerus semi-metallicus* (p. 3); *Corinthiscus* (p. 4); *Corinthiscus insignicorn is* (p. 4); *Xylopertha sulcicollis* (p. 4); *Exopioides spinicollis* (p. 4); *Physogaster globulus* (p. 4); *Physogaster oblongulus* (p. 4); *Euschatia punctato-striata* (p. 5); *Amomphopalpus* (p. 5); *Amomphopalpus quadriplagiatus* (p. 5); *Adelium aeneum* (p. 5); *Eucaliga* (p. 5); *C.* [probably an error for *E.*] *sanguinicollis* (p. 5); *Zonitis nigrotarsatus* (p. 6); *Cycloderus signaticollis* (p. 6); *Nacerdes janthina* (p. 6); *Ditylus cinerascens* (p. 6); *Nimales* (p. 6); *Nimales longicornis* (p. 6); *Mecopselaphus rubricollis* (p. 6); *Salpingus variegatus* (p. 7); *Salpingus anthracinus* (p. 7); *Homalocerus exquisitus* (p. 7); *Naupactus subvittatus* (p. 7); *Adioristus axillaris* (p. 7); *Megalometis xanthomelas* (p. 7); *Callideriphus clathratus* (p. 8).

**1863**. *Manuel entomologique. Genera des coléoptères d’Europe comprenant leur classification en familles naturelles, la description de tous les genres, des tableaux synoptiques destinés à faciliter l’étude, le catalogue de toutes les espèces, de nombreux dessins au trait de caractères par M. Jacquelin du Val (Camille) continué par M. L. Fairmaire et plus de quinze cents types représentant un ou plusieurs insectes de chaque genre dessinés et peints d’après nature avec le plus grand soin par M. Jules Migneaux. Tome troizième.* A. Deyrolle, Paris. Pp. 424–463 + [1]. (4to) [DP: 1863 (wrappers’ dates based on Sherborn’s notes at BMNH), 1 February 1864 (*Ent Soc Lond*)] CNC, GB, YB, BHL

A footnote on page 424 indicates that Fairmaire’s contribution starts from this page. Pages 424–463 form livraisons 118–120 of the entire work. The first 423 pages were authored by Jacquelin du Val [*q.v.*].

**1864–1868**. *Manuel entomologique. Genera des coléoptères d’Europe comprenant leur classification en famille naturelle, la description de tous les genres, des tableaux dichotomiques destinés à faciliter l’étude, le catalogue de toutes les espèces, de nombreux dessins au trait de caractères par Jacquelin du Val (Camille) et par M. L. Fairmaire et près de seize cents types représentant un ou plusieurs insectes de chaque genre dessinés et peints d’après nature avec le plus grand soin par M. Jules Migneaux et par M. Théophile Deyrolle. Tome quatrième.* Deyrolle fils, Paris. Pp. 97–295. (4to) CNC, YB, BHL

The first 95 pages, constituting livraisons 1–12 of the entire work, were published in 1854–1855 and authored by Jacquelin du Val [*q.v.*]. The remaining pages, by Fairmaire, constitute livraisons 121–144 and were issued as follows: **121–127**: (pp. 97–176) 1864 (Dallas 1865: 339, 419–420) with livraisons 121–123 recorded on 3 October 1864 (*Ent Soc Lond*), livraisons 124–125 on 1 May 1865 (*Soc Linn Norm*) and livraisons 126–127 on 5 June 1865 (*Ent Soc Lond*); **128–131**: (pp. 177–204) 1865 (Dallas 1866: 389) with livraison 128 recorded on 4 September 1865 (*Ent Soc Lond*), 129 on 6 November 1865 (*Ent Soc Lond*), 130 on 1 January 1866 (*Ent Soc Lond*), and 131 on 4 June 1866 (*Ent Soc Lond*); **132–136**: (pp. 205–?268) between January 1866 and 7 December 1868; **137–144**: (pp. ?269–295) 7 December 1868 (*Ent Soc Lond*). The title page is dated 1868.

**1870**. *Faune élémentaire des coléoptères de France contenant la description des genres et des espèces qui se rencontrent le plus fréquemment en France. 2^e^ édition.* Deyrolle Fils, Paris. [1] + 289 pp. + 10 pls. (18mo) [DP: 1870 (title page), 19 March 1870 (*Bibl Fr*), April 1870 (*Polybiblion*), May 1870 (*Allg Bibl*)] GB

The first edition was published by Fairmaire and Laboulbène in 1854–1856 [*q.v.*]. A fourth edition, “*revue et augmentée de tableaux synoptiques et de la description d’un grand nombre d’espèces*,” was issued in Paris [MCZ] and recorded on 15 May 1876 (*Pet Nouv Ent*). I have found no indication regarding a third edition.

**1882**. [Fairmaire, L., Lansberge, V. and Bourgeois, J.] *Mission G. Révoil aux pays Çomalis. Faune et flore. Coléoptères recueillis par M. G. Révoil chez les Çomalis. Descriptions.* Pp. iv + 104 pp. + 1 pl. *in*: *Georges Révoil. Faune et flore des pays Çomalis (Afrique orientale).* viii + 14 pp. + 2 pls + 14 pp. + 1 pl. + 25 pp. + 3 pls. + 12 pp. + 108 pp. + 6 pls + 39 pp. + 4 pls + 104 pp. + 1 pl. + 70 pp. + 6 pls + 78 pp. Challamel Ainé, Paris. (8vo) [DP: 1882 (title page), June 1882 (see *Soc Ent Fr* [*Annales* (6) 3: 22]), August 1882 (*Cat Lib Fr*; *Nat Nov*), 9 September 1882 (*Bibl Fr*), 20 October 1882 (*Soc Géog Fr*), October–November 1882 (*Polybiblion*)] CNC, BHL, GAL

This work contains ten parts, each separately paginated, issued separately in 1882. The wrapper for the contribution of Fairmaire et al. was not seen during this study but it is probably dated June 1882 as noticed in the *Bulletin bibliographique* (p. 22) of volume 3 (sixième série) of the *Annales de la Société Entomologique de France*. The new species of Scaraboidea in this work were previously published by van Lansberge in the *Bulletin ou Comptes Rendus de la Société entomologique de Belgique* 26 [1882]: xxi–xxxi [DP: 1 April 1882 (*Feuille Jeunes Nat*)].

**1882**. *Histoire naturelle de la France. 8^e^ partie. Coléoptères. Avec 27 planches.* Émile Deyrolle, Paris. [4] + 336 + [26] pp. + 27 pls. (8vo) [DP: n.d. (title page), 15 July 1882 (*Naturaliste*), August 1882 (*Nat Nov*), 16 September 1882 (*Bibl Fr*), 27 December 1882 (*Soc Ent Fr*)] ANXL, GAL

The book was reissued in 1890 (*n.v.*). A “Nouvelle édition entièrement refondue” by Louis-Marie Planet was published in 1913 in Paris [CNC].

**1888**. Coléoptères. Pp. Di.3–Di63 + pls 1–2 *in*: *Mission scientifique du Cap Horn. 1882–1883. Tome VI. Zoologie. Deuxième partie. Insectes, par L. Fairmaire, Signoret, J. et P. Mabille et J.-M.-F. Bigot. Arachnides, par E. Simon. Crustacés, par A. Milne-Edwards. Mollusques, par le Dr A.-T. de Rochebrune et J. Mabille.* Gautier-Villars et Fils, Paris. 63 pp. + 2 pls (Coléoptères) + 7 pp. (Hémiptères) + 9 pp. + 1 pl. (Névroptères) + 35 pp. + 3 pls (Lépidoptères) + 45 pp. + 4 pls (Diptères) + 42 pp. + 2 pls (Arachnides) + 76 pp. + 9 pls (Crustacés) + 143 pp. + 9 pls (Mollusques). (4to) [DP: 1888 (wrapper), 25 February 1888 (*Bibl Fr*), 28 March 1888 (*Soc Ent Fr*), March 1888 (*Nat Nov*), 10 April 1888 (*Soc Zool Fr*)] McG, GB

The second part of the sixth volume of the *Mission scientifique du Cap Horn* includes eight sections, each separately paginated. The title page of volume 6 (second part) bears the date of 1891, but the wrapper of the “Insectes” section (Coléoptères + Hémiptères + Névroptères + Lépidoptères + Diptères) has the date 1888. Woodward (1904: 603) and Derksen and Scheiding-Göllner (1968: 110) listed the publication date as 1887 but this date could not be confirmed by Evenhuis (1997a: 92) nor by myself. The date of “15 February 1888” listed for the publication of the “Insectes” section by Evenhuis (1997a: 92) and Low and Evenhuis (2012: 71, 73) and taken from the *Bibliographie de la France* is a lapsus for 25 February 1888.

The descriptions of the new Coleoptera species were first published in the *Annales de la Société Entomologique de France* (6) 5 [1885]: 33–70. A few changes were made in this fascicle: the name *Brachymys* Fairmaire, 1885 was changed to *Pachymys* and *Hornius* Fairmaire, 1885 to *Hornibius*.

#### Fallén, Carl Fredrik (Kristinehamn, Värmland, Sweden: 22 September 1764 – 26 August 1830: Esperöd, Kristianstad, Sweden). Swedish botanist and entomologist mostly interested in Diptera; professor of botany and natural history at the University of Lund and eventually curator of the natural history collections. References. Weibull and Tegnér (1868: 310–311); Hofberg (1876a: 290); Wijkmark (1914).

**1802**. *D.D. Observationes entomologicae, quas consensu ampliss. Fac. Philosoph. Lund. Publicae disquisitioni subjiciunt praeses Carl Fredric Fallén, et respondens Abraham Rönbeck, Scanus. In Lyceo Carolino die IX Junii MDCCCII. Part: I.* Berling, Lundae. 20 pp. (4to) [DP: 9 June 1802 (title page)] CNC

The entire work consists of three parts; the second and third ones were published in 1807 [*q.v.*].

**1807**. *D.D. Observationes entomologicae, quas consensu ampliss. Fac. Philosoph. Lund. Publicae disquisitioni subjiciunt praeses Carl Fredric Fallén, et respondens Daniel Danielsson, Smolandus. In Lyceo Carolino die XIII. Junii MDCCCVII. Part. II.* Berling, Lundae. Pp. 21–30. (4to) [DP: 13 June 1807 (title page)] CNC

**1807**. *D.D. Observationes entomologicae, quas consensu ampliss. Fac. Philosoph. Lund. Publicae disquisitioni subjiciunt praeses Carl Fredric Fallén, et respondens Petrus N. Block. Gothoburgensis. In Lyceo Carolino die X Dec. MDCCCVII. Part. III.* Berling, Lundae. Pp. 31–42. (4to) [DP: 10 December 1807 (title page)] CNC

**1807**. *Monographia cantharidum et malachiorum Sveciae, quam, consensu ampliss. Facult. Phil. Lund. publicae disquisitioni modeste subjiciunt auctor Carl Fred. Fallén, Philos. mag. et botanices demonstrator ord. et respondens John Elof Arrhenius Wermelandus. Die III Junii MDCCCVII.* Berling, Lundae. Pp. [2] + 5–16. (4to) [DP: 3 June 1807 (title page)] MCZ

**1807**. *Monographia cantharidum et malachiorum Sveciae, cujus continuationem consensu ampliss. Facult. Phil. Lund. Publicae disquisitioni modeste subjiciunt auctor Carl Fred. Fallén, Philos. mag. et botanices demonstrator ord. et respondens Carl M. Arrhenius, Wermelandus. Die III Junii MDCCCVII. H.p.m.s.* Berling, Lundae. Pp. [2] + 17–26. (4to) [DP: 3 June 1807 (title page)] MCZ

#### Fauconnet, Louis (Pontarlier, Doubs, France: 25 June 1837 – 15 August 1921: Autun, Saône-et-Loire, France). French pharmacist in Autun interested in Coleoptera; his collection was given in his lifetime to Maurice Pic [*q.v.*]. References. Constantin (1992: 35); Cambefort (2006: 170).

**1887**. *Catalogue raisonné des coléoptères de Saone-&-Loire.* G. Martet, Creusot. iii + 280 pp. (8vo) [DP: 1887 (title page; preliminaries dated 20 December 1886), September 1888 (*Nat Nov*)] GB

This is an annotated catalogue of the Coleoptera of the French department of Saône-et-Loire.

**1892**. *Faune analytique des coléoptères de France.* Bligny-Cottot, Autun. [2] + 519 + [9 (Index generum)] pp. (8vo) [DP: 1892 (title page; preliminaries dated 31 December 1891), 24 February 1892 (*Soc Ent Fr*), 13 March 1892 (*Soc Hist Nat Autun*), 9 April 1892 (*Bibl Fr*), April 1892 (*Nat Nov*)] CMLE, GAL, GB

Two supplements of four pages each were published, probably also in 1892, and are often inserted at the end of the book.

**1894**. *Genera des coléoptères de France.* Bligny-Cottot, Autun. [3] + 84 pp. (8vo) [DP: 1894 (title page), September 1894 (*Rev Ent* 13: 196), November 1894 (*Nat Nov*; *Soc Agr Fr*), 9 December 1894 (*Soc Hist Nat Autun*), January 1895 (*Zool Anz*), February 1895 (*Polybiblion*)] ANNX

#### Fauvel, Charles Adolphe Albert (Caen, Calvados, France: 27 October 1840 – 4 January 1921: Caen, France). French lawyer and entomologist mainly interested in the family Staphylinidae; founded the journal *Revue d’Entomologie*; his collection was neglected but eventually went to the Institut Royal des Sciences Naturelles de Belgique in Brussels. References. Smetana and Herman (2001: 65–66, P); Cambefort (2006: 170).

**1862–1878**. *Notices entomologiques.* F. Le Blanc-Hardel, Caen. (8vo) GB (part), CNC (part), ANSP (part)

*Première partie. Coléoptères de la Nouvelle-Calédonie, recueillis par M. E. Déplanche, chirurgien de la marine impériale, 1858–59–60.* 68 pp. + 3 pls. [DP: 1862 (title page), 13 September 1862 (*Bibl Fr*)]. Also issued in *Bulletin de la Société Linnéenne de Normandie* 7 [1861–62]: 120–185 [DP: 1862 (title page), 14 February 1863 (*Bibl Fr*)].

*Deuxième partie. I. Études sur les staphylinides de l’Amérique centrale principalement du Mexique. II. Description et figure d’une Aranéide inédite de la Nouvelle-Calédonie. Avec 1 planche lithographiée.* 66 pp. + 1 pl. [DP: 1864 (title page), 16 April 1864 (*Bibl Fr*), July–August 1864 (*Allg Bibl*)]. Also issued in *Bulletin de la Société Linnéenne de Normandie* 9 [1863–64]: 8–70 [DP: 1865 (title page), 6 May 1865 (*Bibl Fr*)].

*Troisième partie. I. Études sur les staphylinides de l’Amérique centrale principalement du Mexique. II. Notes synonymiques. III. Addenda et delenda au catalogue des Coléoptères de France de M. le Dr Grenier. IV. Énumération des insectes recueillis en Savoie et en Dauphiné (1861–1863) et descriptions d’espèces nouvelles.* 18 + 16 + 71 pp. [DP: 1865 (title page), 2 September 1865 (*Bibl Fr*)]. Also issued in *Bulletin de la Société Linnéenne de Normandie* 9 [1863–64]: 253–321, 348–361 [DP: 1865 (title page), 6 May 1865 (*Bibl Fr*)]; 10 [1864–65]: 9–26 [DP: 1866 (title page), 3 December 1866 (*Ent Soc Lond*)].

*Quatrième partie. I. Faune du Chili. Coléoptères staphylinides. II. Notes synonymiques. Avec une planche gravée.* 110 pp. + 1 pl. [DP: 1866 (title page), 23 June 1866 (*Bibl Fr*)]. Also issued in *Bulletin de la Société Linnéenne de Normandie* 10 [1864–65]: 246–353 [DP: 1866 (title page), 3 December 1866 (*Ent Soc Lond*)].

*Cinquième partie. I. Faune du Chili. Coléoptères staphylinides (Fin). II. Description des staphylinides de la Réunion. III. Catalogue des Coléoptères de la Nouvelle-Calédonie et dépendances, avec descriptions, etc. IV. Notes synonymiques. Avec une planche gravée.* 116 pp. + 1 pl. [DP: 1867 (title page)]. Also issued in *Bulletin de la Société Linnéenne de Normandie* (2e Série) 1 [1866]: 6–76, 172–214 [DP: 1868 (title page), 7 August 1869 (*Bibl Fr*)].

*Sixième partie. I. Les staphylinides de l’Afrique boréale. II. Révision du genre Cyrtothorax.* 86 pp. [DP: 1878 (title page), 1 March 1879 (*Bibl Fr*), March 1879 (*Nat Nov*), May 1879 (*Feuille Jeunes Nat*), 9 June 1879 (*Zool Anz*), January–June 1879 (*Bibliot Hist-Nat*)]. Also issued in *Bulletin de la Société Linnéenne de Normandie* (3e Série) 2 [1877–78]: 83–166 [DP: 1878 (title page), 10 May 1879 (*Bibl Fr*), May 1879 (*Nat Nov*)].

*Septième partie. Les staphylinides de l’Amérique du Nord.* 100 pp. [DP: 1878 (title page), August 1879 (*Nat Nov*)]. Also issued in *Bulletin de la Société Linnéenne de Normandie* (3e Série) 2 [1877–78]: 167–266 [DP: 1878 (title page), 10 May 1879 (*Bibl Fr*), May 1879 (*Nat Nov*)].

**1868–1869**. *Faune gallo-rhénane ou species des insectes qui habitent la France, la Belgique, la Hollande, le Luxembourg, la Prusse Rhénane, la Nassau et le Valais avec tableaux synoptiques et planches gravées. Coléoptères. Tome premier.* F. Le Blanc-Hardel, Caën. xxiv + 282 + 16 [supplément 1er] pp. + 4 pls. (8vo) CMLE, GB

This volume, the first of the serie *Faune gallo-rhénane*, covers the generalities and classification. It was issued in two livraisons: **1**: (pp. vi–xxiv, 1–146) 1868 (title page; preface dated 1 June 1868), 26 September 1868 (*Bibl Fr*), September 1868 (*Allg Bibl*), July–December 1868 (*Bibliot Hist-Nat*); **2**: (pp. 147–282, 1–16) 1868–1869 (title page), 1 December 1869 (*Pet Nouv Ent*), January 1870 (*Rev Mag Zool*).

This volume was also issued in the *Bulletin de la Société Linnéenne de Normandie* (2e Série) 2 [1867]: 175–379 + 4 pls [DP: 1868 (title page), 30 October 1869 (*Bibl Fr*)], 3 [1868]: 26–113 [DP: 1869 (title page)] under the title “*Faune gallo-rhénane ou description des insectes qui habitent la France, la Belgique, la Hollande, les provinces Rhénanes et le Valais. Avec tableaux synoptiques et planches gravées. Coléoptères.*”

The *Faune gallo-rhénane* consists of five volumes, 1868–1906. The first three volumes were authored by C.A.A. Fauvel, the fourth one by Jules Bourgeois, 1884–1893 [*q.v.*] and the fifth one by H. du Buysson, 1892–1900 [*q.v.*].

**1872–1875**. *Faune gallo-rhénane ou species des insectes qui habitent la France, la Belgique, la Hollande, le Luxembourg, la Prusse Rhénane, le Nassau et le Valais avec tableaux synoptiques et planches gravées. Coléoptères. Tome troisième.* F. Le Blanc-Hardel, Caën. 738 + 82 [suppléments] + xxxviii [catalogue] pp. + 4 pls. (8vo) CNC

This volume constitutes the third one of the series *Faune gallo-rhénane*. It was issued in four livraisons: **3**: (pp. 1–214, pls 1–2) May 1872 (Fauvel 1873: 47), 3 October 1872 (*Nature* 6: 456), 29 March 1873 (*Bibl Fr*), 15 April 1873 (*Academy*); **4**: (pp. 215–390, 1–24 [1e supplément aux staphylinides]) July 1873 (Fauvel 1874: 50), 18 April 1874 (*Academy*); **5**: (pp. 391–544, 25–46 [2e supplément aux staphylinides]) July 1874 (Fauvel 1875: 54), September 1874 (wrapper), 27 January 1875 (*Soc Ent Fr*), 15 March 1875 (*Acad Sci Fr*); **6**: (pp. 545–738, 47–82 [3e supplément aux staphylinides] + xxxviii [Catalogue systématique des staphylinides de la faune Gallo-Rhénane avec l’addition synonymique des espèces européennes, sibériennes, caucasiques et méditerranéennes et descriptions nouvelles]) December 1875 (wrapper), 14 August 1876 (*Acad Sci Fr*), 23 August 1876 (*Soc Ent Fr*).

This work was also issued in the *Bulletin de la Société Linnéenne de Normandie* (2e Série) 5 [1869–70]: 27–192 [DP: 1870 (title page), 1871 (cover), 17 August 1872 (*Bibl Fr*)], 6 [1870–72]: 8–136 [DP: 1 December 1873 (p. i)], 7 [1872–73]: 8–131 [DP: 1873 (title page and cover), 1874 (p. i of volume 6)], 8 [1873–74]: 167–340 [DP: 1874 (title page), 17 April 1875 (*Bibl Fr*)], 9 [1874–75]: 202–236 [DP: 1875 (title page), 2 December 1876 (*Bibl Fr*)]; 10 [1875–76]: 26–223, 232–267 [DP: 1876 (title page), 14 April 1877 (*Bibl Fr*)].

**1882–1890**. *Faune gallo-rhénane ou species des insectes qui habitent la France, la Belgique, la Hollande, le Luxembourg, la Prusse Rhénane, le Nassau et le Valais avec tableaux synoptiques et planches gravées. Coléoptères. Tome second.* F. Le Blanc-Hardel, Caen. 220 pp. (8vo) CMLE

This volume, the second of the *Faune gallo-rhénane*, covers the cicindélides and carabiques. It was issued, with separate pagination, in the *Revue d’Entomologie*. The dates of publication are: (pp. 1–4) January–February 1882; (pp. 5–12) 1 March 1882; (pp. 13–20) 15 March 1882; (pp. 21–28) 1 April 1882; (pp. 29–36) May 1882; (pp. 37–44) June 1882; (pp. 45–52) July 1882; (pp. 53–60) August 1882; (pp. 61–68) September 1882; (pp. 69–76) October 1882; (pp. 77–84) November 1882; (pp. 85–92) January 1883; (pp. 93–100) February 1883; (pp. 101–108) March 1883; (pp. 109–116) April 1883; (pp. 117–124) May 1883; (pp. 125–132) June 1883; (pp. 133–140) July 1883; (pp. 141–148) September 1883; (pp. 149–156) November 1883; (pp. 157–164) March 1884; (pp. 165–172) May 1885; (pp. 173–180) June 1885; (pp. 181–188) August 1885; (pp. 189–196) September 1885; (pp. 197–212) 1889; (pp. 213–220) 1890.

**1888–1891**. [Catalogue des coléoptères gallo-rhénans]. Caen. 48 pp. (8vo) CNC

This catalogue was included, with separate pagination, in the *Revue d’Entomologie* **7** [1888]: 1–16; **8** [1889]: 17–32; **10**(1): 33–40 [DP: January 1891 (title page)]; **10**(2): 41–48 [DP: February 1891 (title page)]. It covers only the Cicindelidae, Carabidae, Dytiscidae, Gyrinidae, Micropeplidae and part of the Staphylinidae. Apparently no title page was issued for this publication; the title reported here is that given in the content of parts 1 and 2 of volume 10 of the *Revue d’Entomologie*. Three new family-group names are proposed in this work.

#### Fée, Antoine Laurent Apollinaire (Ardentes, Indre, France: 7 November 1789 – 21 May 1874: Paris, France). French botanist and pharmacist; posted at the military hospital in Lille in 1825, then in Strasbourg in 1832 where he became pharmacist and was appointed director of the botanical gardens as well as professor of natural history at the Faculty of Medicine; stayed in Strasbourg until the Prussians took the city in 1871 and then moved to Paris. References. Knight (1872: 522–523); Limousin (1874); Chéreau (1877); Spieth (2007: 148).

**1830**. *Caroli Linnaei, Sueci, doctoris medicinae, systema naturae, sive regna tria naturae systematice proposita per classes, ordines, genera et species. Editio prima reedita.* F.G. Levrault, Parisiis. vi + 81 + [1(Errata)] pp. (8vo) [DP: 1830 (title page), 7 August 1830 (*Bibl Fr*), June–September 1830 (*Foreign Quart Rev*)] GB

This work is an adaptation of the first edition of Linnaeus’ *Systema Naturae* published in 1735. The Coleoptera are on pages 74–76. The genera are briefly described and some have binominal specific names associated with them. Two Coleoptera genera, *Notopeda* (p. 74) and *Buceros* (p. 75), were not used again as valid names by Linnaeus in his 1758 version of the *Systema Naturae*^28^ or subsequent publications nor by any other authors after 1758 until Fée made them available. One of the genera, *Notopeda*, is listed and attributed to Fée (1830) in Sherborn’s (1928: 4419) *Index Animalium* and Neave’s (1940: 355) *Nomenclator Zoologicus*.

#### Felsche, Carl (9 October 1839 – 11 April 1914: Leipzig, Saxony, Germany). German rentier and beetle collector; a large part of his collection went to the Staatliches Museum für Tierkunde in Dresden. Reference. Blumenthal (2004).

**1898**. *Verzeichniss der Lucaniden, welche bis jetzt beschrieben sind*. Ernst Heyne, Leipzig. iv + 88 [uneven pages without text] + [1] pp. (8vo) [DP: 1898 (title page; p. iv dated July 1898), 19 September 1898 (*Bibl Zool*), September 1898 (*Nat Nov*), 9 October 1898 (*Naturwiss Wochen* 13: 491), 12 October 1898 (*Soc Ent Fr*), October 1898 (*Ent Nachr* 24: 318), November 1898 (*Berl Ent Zeit* 43: 204)] AMNH

#### Ferrari, Johann [Giovanni] Angelo (Count) (1806 – 18 May 1876: Vienna, Austria). Austrian (Italian-born) entomologist who specialized on the study of Coleoptera, particularly Scolytinae; curator of the Coleoptera section at the Naturhistorisches Museum in Vienna; his scolytine types are in that museum but his personal collection went to the *Staatsgymnasium* in Nikolsburg (= Mikulov in Czech Republic). Reference. Katter (1876).

**1867**. *Die Forst- und Baumzuchtschädlichen Borkenkäfer (Tomicides Lac.) aus der Familie der Holzverderber (Scolytides Lac.), mit besonderer Berücksichtigung vorzüglich der europäischen Formen, und der Sammlung des k.k. zoologischen Kabinetes in Wien.* Carl Gerold’s Sohn, Wien. [2] + 95 + [1 (Index)] pp. (8vo) [DP: 1867 (title page; *Vorwort* dated January 1867), 22 August 1867 (*Allg Bibl Deutsch*), August 1867 (*Allg Bibl*), July–December 1867 (*Bibliot Hist-Nat*)] GB

Ferrari published supplements to his book in 1867 in *Coleopterologische Hefte* 2: 104–115 and in March 1869 in *Berliner Entomologische Zeitschrift* 12: 251–258.

#### Ferrer, Léon (*ca* 1834 – December 1903: Perpignan, Pyrénées-Orientales, France). Pharmacist at Perpignan; president of the Société Agricole, Scientifique et Littéraire des Pyrénées-Orientales for many years. Reference. Escarguel (1904, P).

**1859**. *Essai sur les insectes vésicants. Thèse présentée et soutenue a l’école supérieure de pharmacie de Paris le 27 août 1859 pour le grade de Pharmacien de première classe.* E. Thunot et C^e^, Paris. [3] + 64 pp. (4to) [DP: 27 August 1859 (title page), 3 September 1859 (*Bibl Fr*), 24 September 1859 (*Soc Imp Zool Acclim*), July–December 1859 (*Bibliot Hist-Nat*)] CMLE

#### Fiedler, Karl [Carl] Wilhelm (Malchin, Mecklenburg-Western Pomerania, Germany: 4 December 1758 – *ca* 1831). German apothecary; professor at the Institut of Forestry in Waldau near Cassel; later, professor at the “Anstalt für Bergwerksalumnen” in Cassel. References. Justi (1819: 148–153); Poggendorff (1863: 745).

**1790**. *Anleitung zur Kenntniss des Thierreichs nach den besten Schriftstellern.* C.F. Schwan und G.C. Götz, Mannheim. 256 + [4] pp. (8vo) [DP: 1790 (title page), 17 September 1791 (*Allg Lit Ztg*)] GB

The Coleoptera are on pages 170–184. This work was also issued the same year by the same publisher in the second volume of “*Allgemeines pharmazeutisches, chymisch-mineralogisches Wörterbuch oder alphabetische Anleitung zum Gebrauche für Apotheker, Chymisten und Mineralogen*” [GB].

#### Fischer, Jakob [Jacob] Benjamin (Riga, Latvia: 13 October 1731 – 25 July 1793: Riga, Latvia). Russian apothecary and naturalist in Riga; student of Linnaeus in Uppsala; later, worked as an accountant at an orphanage. References. Recke and Napiersky (1827: 568–569); Welding et al. (1970: 217–218).

**1778**. *Versuch einer Naturgeschichte von Livland. Mit Kupfern.* Johann Gottlob Immanuel Breitkopf, Leipzig. 16 + [8] + 374 + [16 (Register)] pp. + 2 pls. (8vo) [DP^29^: 1778 (title page; *Vorbericht* dated 9 April 1778), July 1778 (*Allg Verz Bücher*), 10 August 1778 (*Jena Ztg*)] GB

The Coleoptera are on pages 129–138. A second edition of this work was published in 1791 [*q.v.*].

Additions to this work were issued in 1784 in a book entitled “*J.J. Ferbers Anmerkungen zur physischen Erdbeschreibung von Kurland, nebst J.B. Fischers Zusätzen zu seinem Versuch einer Naturgeschichte von Liefland. Mit einem Kupfer*” [GB] published in Riga. The book is divided in two sections: the first one (pp. 1–196) are additions to Fischer’s “*Versuch einer Naturgeschichte von Livland*” and the second one (pp. 209–305) is Ferber’s “*Anmerkungen zur physischen Erdbeschreibung von Kurland.*” The Coleoptera in Fischer’s section are on pages 54–66.

**1791**. *Versuch einer Naturgeschichte von Livland. Zwote vermehrte und verbesserte Auflage. Mit Kupfern.* Friedrich Nicolovius, Königsberg. xxiv + 826 pp. + 4 pls. (8vo) [DP: 1791 (title page; p. [viii] dated 18 March 1791), 7 September 1791 (*Intell Allg Lit Ztg*), 15 October 1791 (*Gött Anz*)] GB

The Coleoptera are on pages 262–289. The first edition was issued in 1778 [*q.v.*].

#### Fischer, Joannes Baptista [Johann Baptist] (Munich, Germany: 1803 – 29 May 1832: Leiden, South Holland, Netherlands). German physician and naturalist; assistant to the botanist Carl Ludwig Blume (1796–1862) in Brussels. Reference. MacLean (1979: 30).

**1827**. *Tentamen conspectus cantharidiarum. Dissertatio inauguralis, quam pro summis in medicina et chirurgia honoribus legitime obtinendis eruditorum examini subjicit Joannes Baptista Fischer, Monacensis, Bavarus.* Mich. Lindauer, Monachii [= Munich]. [2] + 26 pp. (4to) [DP: 1827 (title page), January 1828 (*Isis*, Heft I: [3])] CMLE, GB

It seems that the thesis was defended on 22 March 1827 (Spengel 1828: 158). Whether the work was published at the time remains to be ascertained.

#### Fischer, Leopold Heinrich (Freiburg, Baden-Württemberg, Germany: 19 December 1817 – 2 February 1886: Freiburg, Germany). German mineralogist and zoologist; studied medicine in Freiburg and Vienna; practiced medicine in Freiburg; later, professor of mineralogy and geology at the University of Freiburg. References. Weech (1891: 129–133); Seifert (1961: 187–188).

**1843**. *Dissertatio inauguralis zoologica sistens enumerationem coleopterorum circa Friburgum Brisgoviae indigenarum annexis locis natalibus quam consensu et auctoritate illustris medicorum facultatis in alma et antiqua universitate Alberto-Ludoviciana Friburgensi pro doctoris medicinae gradu scripsit Leopoldus Henricus Fischer Friburgensis medicus et chirurgus licentiatus.* Fr. Wagner, Friburgi Brisgoviae [= Freiburg im Breisgau]. vi + 66 pp. (8vo) [DP: 1843 (title page), 22 February 1844 (*Allg Bibl Deutsch*), February 1844 (*Isis*, Heft II: [2]), 2 March 1844 (*Lit Ztg*), 22 March 1844 (*Leip Reper*), 13 November 1844 (*Soc Ent Fr*), 2 July 1845 (*Ent Ver Stettin*)] CMLE (mf)

This thesis consists of a list of Coleoptera from the surroundings of the city of Freiburg im Breisgau in Germany.

#### Fischer, Sigmund Caspar (Gondo [currently part of Zwischenbergen], Valais, Switzerland: 27 October 1793 – 16 February 1860: Hirtenberg, Lower Austria, Austria). Austrian (Swiss-born) physician and naturalist; professor of mineralogy, zoology and theoretical medicine at the Imperial medico-chirurgical Joseph Academy in Vienna. Reference. Callisen (1840: 56).

**1829**. *Handbuch der Zoologie oder Beschreibung der Thiere nach dem äussern und innern Baue, und ihren Verrichtungen.* J. G. Heubner, Wien. xxxiv + 599 pp. (8vo) [DP: 1829 (title page; *Vorrede* dated 1 August 1828), 18 April 1829 (*Bibl Deutsch*), 30 June 1829 (*Intell Ztg Welt*), January–June 1829 (*Verz Bücher Landkarten*)] GB, EUR

The Coleoptera are on pages 177–232.

#### Fischer von Waldheim, Johann Gotthelf (Waldheim, Saxony, Germany: 13 October 1771 – 18 October 1853: Moscow, Russia). German naturalist and paleontologist; studied comparative anatomy under Baron Cuvier in Paris; professor of natural history in Mainz, Germany; later, professor of natural history and director of the natural history museum at the Moscow Academy; founder of the *Société Impériale des Naturalistes de Moscou*. References. Büttner (1956); Weidner (1981: 127–129, P); Mearns and Mearns (1988: 151–153, P); Adler (2012: 59–60, P).

[**1808**]. *Museum Demidoff. Ou catalogue systématique et raisonné des curiosités de la nature et de l’art. Données à l’Université Impériale de Moscou. Par son Excellence Monsieur Paul de Demidoff. Tome troisième. Végétaux et animaux. Avec 6 planches.* Imprimerie de l’Université Impériale, Moscou. ix + 330 pp. + 6 pls. (4to) [DP: 1807 (title page; preface dated 30 December 1807 [JD])] ANSP

There is no author listed on the title page but the second page reads “*Museum-Demidoff. Mis en ordre systématique et décrit par G. Fischer. Tome troisième. Végètaux et animaux. Avec 6 planches.*” The Coleoptera are on pages 37–49, 53–73, 98–101 and the new species described are *Melolontha macrotarsa* (p. 41), *Machla serrata* (p. 41), *Curculio erythrophthalmus* (p. 42), *Coccoderus tuberculatus* (p. 42), *Curculio caesius* (p. 43), *Lamia cincta* (p. 44), *Erotylus oblongo-guttatus* (p. 44) and *Cassida cayennensis* (p. 49). Some of these species are dated 1809 (e.g., Sherborn in his *Index Animalium*) since Fischer von Waldheim redescribed them in the second volume of the *Mémoires de la Société Impériale des Naturalistes de Moscou*.

Despite the date on the title page, this book must have been published in 1808 considering the date of the preface. The entire series consists of three volumes, 1806–1808. Hagen (1862: 235) mentioned a second edition issued in 1817 (*n.v.*).

**1808**. *Tableaux synoptiques de zoognosie. Publiés à l’usage de ses élèves à l’Université Impériale de Moscou. Avec six planches.* Université Impériale, Moscou. [60] + 186 pp. + 6 pls. (4to) [DP: 1808 (title page; dedication dated 15 November 1808 [JD])] BHL

The half-title page reads “*Tabulae synopticae zoognosiae in usum auditorum editae. Cum tabulis aeneis*.” The first edition of this work was issued in 1805 under the title “*Tables synoptiques des animaux*” (*n.v.*); the third edition was issued in 1813 (see next entry).

Evenhuis (1997a: 264) dated this book 1809; however, the copy on BHL is clearly dated 1808 and the dedication 15 November 1808 in the Julian calendar or 28 November 1808 in the Gregorian calendar.

**1813**. *Zoognosia tabulis synopticis illustrata, in usum praelectionum Academiae Imperialis Medico-Chirugicae Mosquensis edita. Editio tertia, classium, ordinum, generum illustratione perpetua aucta. Volumen primum, tabulas synopticas generales et comparativas, nec non characterum quorundam explicationem iconographicam continens.* Nicolai Sergeidis Vsevolozsky, Mosquae [= Moscow]. xiii + [1] + 465 pp. (4to) [DP: 1813 (title page), p. [vi] dated 12 May 1813 [JD])] GB, BHL

The entire work was issued in three volumes, 1813–1814. The Coleoptera are on pages 232–268 of the first volume.

**1820–1828**. *Entomographia imperii Russici.* Augusti Semen, Mosquae [= Moscow]. (4to) CNC, GB

*Auctoritate societatis Caesareae Mosquensis naturae scrutatorum collecta et in lucem edita. Volumen I. Cum xxvi tabulis aeneis.* viii + 210 pp. + 25 pls. [DP: 1820–1822 (title page; preface dated 25 March 1821 [JD], dedication dated 18 May 1822 [JD]), June 1823 (*Allg Lit Ztg*)]. According to Sherborn (1922a: liii), the plates were issued in 1820 and the text after May 1822. However, the volume was issued by livraison as expressed by Fischer von Waldheim (1820: 6). A report about this volume by Latreille (1822), read at the Academy of sciences on 12 November 1821, indicated that 13 plates (six on Coleoptera, one on Orthoptera, two on Nevroptera and four on Lepidoptera) were published; furthermore it is evident from the context that at least the first 36 pages were issued at that time.

*Suae Caesareae Majestati Alexandro I dicata. Volumen II. Cum XL tabulis aeneis.* xx + 264 pp. + 40 pls. [DP: 1823–1824 (title page; preface dated 10 November 1824 [JD]). According to Sherborn (1922a: liii) the plates were issued in 1823 and the text after November 1824. However, this volume and the following one were very likely issued in livraisons as for the first volume.

*Auctoritate societatis Caesareae naturae scrutatorum Mosquensium, in lucem edita. Tomus III. Cicindeletas et Carabicorum partem continens, cum XVIII tabulis aeneis.* viii + [1] + 314 pp. + 18 pls. [DP: 1825–1828 (title page; approuved for publication 10 November 1828 [JD])]. Some of the plates were available as early as 1824. Indeed, in the second volume of the work, issued in 1824, Fischer von Waldheim stated (pp. 263–264) that plates I–IX of the third volume have been engraved; he also listed the species names for each plate. I have not been able to find any indication for the engraving dates of the remaining plates. The copy of this volume on GB has another title page, dated 1825, which reads “Entomographie de la Russie. Tome III.”

The entire series consists of five volumes, the fourth one, dealing with Orthoptera, was published in volume 8 of the *Nouveaux Mémoires de la Société Impériale des Naturalistes de Moscou*, 1846, and the fifth one, dealing with Lepidoptera and authored by Fischer von Waldheim and Eduard Eversmann, was published in 1851 by Semen, Moscow.

**1821**. *Genera insectorum systematice exposita et analysi iconographica instructa. Volumen primum. Genera Coleopterorum.* Augusti Semen, Mosquae [= Moscow]. xi + [1] + 104 pp. + 1 pl. (4to) [DP: 1821 (title page; preface dated 12 April 1821 [JD])] CNC, GB

Another title page, also dated 1821, reads “Genres des insectes publiés au nom de la Société Impériale des Naturalistes.” Sherborn (1922a: liii) mentioned that this work was published after September 1821. This is probably an error originating from the date on the recto of the title page which reads “15 Septembris. 1820” (not 1821).

This publication is part of the first volume of the *Entomographia imperii Russici* as indicated by Fischer von Waldheim in his preface of the *Entomographia* (p. [v]) and is usually bound with it.

**1821**. *Lettre adressée au nom de la Société impériale des Naturalistes de Moscou, à l’un de ses membres M. le docteur Chrétien-Henri Pander, par Gotthelf Fischer de Waldheim, directeur de la Société; contenant une notice sur un nouveau genre d’oiseau et sur plusieurs nouveaux insectes.* Auguste Semen, Moscou. 15 pp. (8vo) [DP: 1821 (title page; letter dated 17 December 1821[JD]), 13 June 1822 (*R Accad Sci Torino*)] GB

This booklet contains a letter addressed to Henri Pander by Fischer von Waldheim (pp. 3–4) and a section entitled “Catalogus Animalium in itinere inde ab Orenburg ad Bokaram usque collectorum” (pp. 5–15).

Several Coleoptera are described in this publication but many of them were already available from Fischer von Waldheim’s plates of the *Entomographia imperii Russici* published in 1820. The following genus and species are made available from this work: *Cicindela clypeata* (p. 9), *Harpalus ? tridentatus* (p. 10), *Clivina picta* (p. 10), *Scarites interruptus* (p. 10), *Aphodius clypeatus* (p. 11), *Melolontha porulosa* (p. 12), *Erodius ferrugineus* (p. 12), *Erodius pygmaeus* (p. 12), *Gnathosia* (p. 13), *Gnathosia glabra* (p. 14), *Brachyrhinus granulatus* (p. 14); *Brachyrhinus leucophyllus* (p. 14); *Curculio transparens* (p. 14); *Cionus trinotatus* (p. 14); *Cerambyx scapularis* (p. 15); *Coccinella buphthalmus* (p. 15).

According to Horn and Schenkling (1928b: 358), a German translation of this booklet was published in Berlin, 1823 (*n.v.*).

**1829**. *Museum historiae naturalis Universitatis Caesareae Mosquensis. Pars II. Insecta.* Typis Universitatis Caesareae, Mosquae [= Moscow]. 147 pp. (8vo) [DP: 1829 (title page; approuved for publication on 25 February 1829 [JD])] CNC (ph)

The second page is entitled “*Collectio insectorum Steveniana*.” This work was published anonymously and has been attributed to Christian von Steven by some bibliographers, including Hagen (1862: 197). However, there is a statement in Fischer von Waldheim (1829: 65) indicating that he was the author. This work is extremely rare. Numerous species- and genus-group taxa were first made avalaible in this work. The following species-group taxa are described: *Siagona dorsalis* (p. 8), *Scarites megacephalus* (p. 8), *Scarites thoracicus* (p. 8), *Scarites hecticus* (p. 8), *Cephalotes canaliculatus* (p. 9), *Cephalotes punctato-striatus* (p. 9), *Notiophilus aestuans* (p. 14), *Notiophilus quadripunctatus* (p. 14), *Amara compos* (p. 16), *Platynus microthorax* (p. 17), *Platynus elongatulus* (p. 17), *Agonum erythropus* (p. 17), *Agonum thoracicum* (p. 17), *Agonum ruficorne* (p. 17), *Calathus angustatus* (p. 18), *Argutor binotatus* (p. 18), *Argutor piceus* (p. 18), *Argutor bicolor* (p. 18), *Argutor picipes* (p. 18), *Poecilus erythropus* (p. 19), *Poecilus crassipes* (p. 19), *Poecilus crenatoistriatus* (p. 19), *Poecilus barbatus* (p. 19), *Steropus aeneus* (p. 20), *Ophonus restrictus* (p. 21), *Ophonus pygmaeus* (p. 21), *Ophonus impressus* (p. 21), *Ophonus clypeatus* (p. 21), *Harpalus interruptus* (p. 22), *Harpalus bicolor* (p. 22), *Harpalus frontalis* (p. 22), *Helaeus Platypterus* (p. 22), *Harpalus erythrocerus* (p. 23), *Harpalus brunnipes* (p. 23), *Stenolophus pygmaeus* (p. 23), *Dyticus torquatus* (p. 25), *Colymbetes lunulatus* (p. 26), *Colymbetes deplanatus* (p. 26), *Hydroporus nigrolineatus* (p. 27), *Hydroporus mixtus* (p. 27), *Staphylinus cucullatus* (p. 28), *Staphylinus platypus* (p. 29), *Buprestis costata* (p. 33), *Buprestis latifrons* (p. 33), *Buprestis granulata* (p. 34), *Buprestis confusa* (p. 34), *Buprestis bicolor* (p. 34), *Buprestis cestum* (p. 34), *Buprestis forficata* (p. 35), *Elater laevicollis* (p. 36), *Elater hastatus* (p. 37), *Lampyris bicincta* (p. 39), *Telephorus epomis* (p. 40), *Telephorus quadratus* (p. 40), *Telephorus axillaris* (p. 40), *Telephorus nigritarsis* (p. 41), *Clerus binotatus* (p. 44), *Silpha triangularis* (p. 46), *Rhipiphorus binotatus* (p. 77).

The series “*Museum historiae naturalis Universitatis Caesareae Mosquensis*” consists of three volumes authored by Fischer von Waldheim; the first volume (Animalia) was issued in 1822 and the third one (Mineralia. Petrefacta. Artefacta) in 1824.

**1842**. *Catalogus coleopterorum in Sibiria orientali a Cel. Gregorio Silide Karelin collectorum.* [Moscou]. 28 pp. (8vo) [DP: n.d. (title page), 11 August 1842 (*Königl Akad Wiss Berl*), 19 September 1842 (*Acad Sci Fr*), 18 October 1842 (*Acad Nat Sci Phila*), 21 October 1842 (*Amer Phil Soc*), 7 November 1842 (*Soc Géol Fr*), 10 January 1843 (*Ent Ver Stettin*)] NCSU, MDZ

This booklet contains two sections: *Coleoptera in Songoria rossica et Ajaguskensi districtu Omskensis gubernii lecta* (pp. 3–21) and *Coleoptera Sibirica sine indicatione loci numero collectoris conservato* (pp. 21–28). Several new species are described in both sections.

#### Flach, Karl (Aschassenburg, Bavaria, Germany: 13 September 1856 – 18 July 1920: Aschassenburg, Germany). German physician in Aschassenburg and coleopterist; his collection was scattered. Reference. Schwarzer (1920, P).

**1888**. *Bestimmungs-Tabellen der europäischen Coleopteren. XVII. Heft. Enthaltend die Familie der Phalacridae.* Ed. Reitter, Brünn. 27 + [1 (Index alphabeticus)] pp. + 1 pl. (8vo) [DP: 1888 (title page), April 1889 (*Nat Nov*)] SME

This work was also issued in *Verhandlungen des Naturforschenden Vereins in Brünn* 27 [1888]: 54–80 [DP: 1889 (title page), February 1890 (*Nat Nov*)]. A French translation was published in 1889 in *Revue d’Entomologie* 8: 13–32.

#### Fleischer, Anton [Antonín] (Rovečné, Czech Republic: 17 February 1850 – 22 October 1934: Brno, Czech Republic). Czech physician in Brno interested in Coleoptera; his collection of Palaearctic Coleoptera is at the Natural History Museum in Prague. Reference. Obenberger (1950, P).

**1899**. *Bestimmungs-Tabelle der europäischen Coleopteren. XXXIX. Heft. Enthaltend: Carabidae: Abtheilung: Scaritini.* Edm. Reitter, Paskau. 38 pp. (8vo) [DP: 1899 (title page), 25 October 1899 (*Soc Ent Fr*), October 1899 (*Nat Nov*), 24 November 1899 (*Bibl Zool*)] SME, GB

A Czech translation was published in *Vestnik Klubu Přirodovědeského v Protějove* 2 [1899]: 24–56.

#### Fleischer, Heinrich

**1896**. *Der Käferfreund. Praktische Anleitung zum Sammeln und Bestimmen der Käfer. Mit 12 Tafeln in feinstem Farbendruck.* Wilhelm Nitzschke, Stuttgart. [1] + 252 pp. + 12 pls. (8vo) [DP: n.d. (title page; *Vorwort* dated April 1896), July 1896 (*Nat Nov*), 19 October 1896 (*Bibl Zool*)] BHL

A second edition was published in 1905 in Esslingen and Munich by J. F. Schreiber [CMLE].

#### Fleming, John (1747 – 17 May 1829: London, United Kingdom). British surgeon of the Indian Medical Service, naturalist and politician; head of the Calcutta Botanic Garden. References. Britten (1916); Desmond (2004a).

**1810**. *A catalogue of Indian medicinal plants and drugs, with their names in the Hindustani and Sunscrit languages.* A.H. Hubbard, Calcutta. 72 + v pp. (8vo) [DP: 1810 (title page)] ARC

One new species of beetle, *Meloe trianthemae*, is described on page 59. A modified version of this work was issued the same year, under the same title, in *Asiatick Researches* 11: 153–196 (*n.v.*) where the description of the new species of *Meloe* is not included. The journal was reissued in 1812 in London [GB]. According to Jackson (1889: 279) the work was translated into Dutch and German (*n.v.*).

#### Fleming, John (near Bathgate, Scotland, United Kingdom: 10 January 1785 – 18 November 1857: Edinburgh, Scotland, United Kingdom). Scottish Presbyterian clergyman, zoologist and geologist; minister in various parishes in Scotland (1808–1834); awarded the chair of natural philosophy at the University and King’s College of Aberdeen in 1834; later, professor of natural history at Free Church College in Edinburgh. References. Bryson (1861); Moore (2004a); Adler (2007: 73–74, P).

**1821**. Insecta. Pp. 41–56 + pl. 85 *in*: *Supplement to the fourth, fifth, and sixth editions of the Encyclopaedia Britannica. With preliminary dissertations on the history of the sciences. Illustrated by engravings. Volume fifth.* Archibald Constable and Company, Edinburg. [4] + 257 + 586 + [2] pp. + pls 85–100. (4to) (*n.v.*).

Volume 5 of the series was published in two parts: **1**: (257 + 162 pp. + pls 85–92) July 1821; **2**: (pp. 163–586 + [2] + pls 93–100) May 1822 (Evenhuis 1997a: 226). The entire *Supplement to the fourth, fifth, and sixth editions of the Encyclopaedia Britannica* was published in six volumes with an addendum, 1815–1825. It was reissued, without the addendum, in 1824 [CAKL].

The *Encyclopaedia Britannica* is in its 15th edition (spanning 32 volumes and 32,640 pages); it was announced in 2012 that it will be the last printed edition.

**1822**. *The philosophy of zoology; or a general view of the structure, functions, and classification of animals. In two volumes. With engravings. Vol. II.* Archibald Constable & Co., Edinburgh [&] Hurst, Robinson & Co., London. 618 pp. (8vo) [DP: 1822 (title page), 22 June 1822 (*Lit Gaz*), June 1822 (*Blackw Mag*; Peddie and Waddington 1914: 208), July 1822 (*Not Geb Natur*), 1 August 1822 (*New Monthly Mag*)] McG, GB, ARC, BHL

The entire work was published in two volumes issued simultaneously. The Coleoptera are on pages 560–573 of the second volume. Several new family-group names are proposed. An Italian translation by G. Zendrini was published in Pavia, 1829, under the title “*Filosofia zoologica, ossia prospetto generale della struttura funzioni, e classificazione degli animali del dottore Giovanni Fleming*” [GB].

#### Fleutiaux, Edmond Jean-Baptiste (Argenteuil, Val-d’Oise, France: 22 October 1858 – 25 November 1951: Nogent-sur-Marne, Val-de-Marne, France). French coleopterist with a particular interest in click beetles; owned a wine and alcohol business; his collection is at the Muséum National d’Histoire Naturelle in Paris. Reference. Cambefort (2006: 172).

**1892**. *Catalogue systématique des Cicindelidae décrits depuis Linné.* H. Vaillant-Carmanne, Liège. 186 pp. (8vo) [DP: 1892 (title page), 11 January 1893 (*Soc Ent Fr*), June 1893 (*Nat Nov*)] CNC, GB, ARC

#### Formey, Johann Ludwig (Berlin, Germany: 7 February 1766 – 23 June 1823: Berlin, Germany). German physician; professor of therapy at the Collegium Medico-chirurgicum in Berlin; later, professor at the newly formed medico-surgical Academy in Berlin. References. Anonymous (1833a); Hirsch (1878).

**1796**. *Versuch einer medicinischen Topographie von Berlin.* Ernst Felisch, Berlin. xii + 382 pp. (8vo) [DP: 1796 (title page; dedication dated 22 April 1796), 29 June 1796 (*Intell Allg Lit Ztg*), 17 October 1796 (*Gött Anz*), 12 November 1796 (*Goth Ztg*)] GB

This work includes a list of Coleoptera from Berlin and surrounding areas (pp. 309–325) based on the collection of “Königlichen Leibchirurgus Herrn Collignon.” The same list is included in H. Wollheim’s “*Versuch einer medicinischen Topographie und Statistik von Berlin*” [GB] published in 1844 in Berlin (pp. 487–494).

#### Forsskål, Pehr (Helsinki, Finland: 11 January 1732 – 11 July 1763: Jerim, Yemen). Swedish (Finnish-born) naturalist and explorer; student of Linnaeus in Uppsala; studied languages and oriental philosophy at the University of Göttingen (1753–1756) before returning to Uppsala to study economics; died of the plague while on the Danish expedition to Egypt and Arabia (1761–1763); his manuscripts were published by Niebuhr, the sole survivor of the expedition; the insects from the expedition are in the Zoologisk Museum at the University of Copenhagen. References. Christensen (1918, P); Adler (2012: 28–29, P); Hansen et al. (2012: 35–36, P); Baack (2013).

**1775**. *Descriptiones animalium avium, amphibiorum, piscium, insectorum, vermium; quae in itinere orientali observavit Petrus Forskål. Post mortem auctoris edidit Carsten Niebuhr. Adjuncta est materia medica kahirina atque tabula maris rubri geographica*. Möller, Hauniae [= Copenhagen]. 19 + [3] + iii–xxxiv + [2] + 164 pp. (4to) [DP: 1775 (title page), 3 June 1775 (*Gött Anz*), 23 September 1775 (*Cat Hebd*), 25 September 1775 (*Jena Ztg*), 11 November 1775 (*Erlang Anmer Nachr*)] CNC, GB, BHL

This work was edited after Forsskål’s death by Carsten Niebuhr but the new taxa are attributed to Forsskål. The Coleoptera are on pages 77–80.

Forsskål published in 1776 “*Icones rerum naturalium, quas in itinere orientali depingi curavit Petrus Forskål. Post mortem auctoris ad regis mandatum aeri incisas edidit Carsten Niebuhr*” containing 15 pages of text and 43 plates. On plate 24, *Cicindela littorea* and *Tenebrio arundinaceus*, both described originally by Forsskål in 1775, are illustrated.

#### Forster, Johann Reinhold (Tczew, Poland: 1729 – 9 December 1798: Halle an der Saale, Saxony-Anhalt, Germany). German (Prussian-born) naturalist and traveler; minister in a parish in Prussia for 12 years; moved to Saint Petersburg and travelled over Russia with his son Georg collecting natural history objects; moved to London and became lecturer at the Dissenter’s Academy in Warrington, Lancashire; naturalist on the second voyage of James Cook with his son Georg (1772–1775); later, professor of natural history at Halle; most of his specimens are lost although some, including Coleoptera, have been discovered at the Linnaean Society in London. References. Hoare (1976, P); Day and Fitton (1977); Mearns and Mearns (1988: 155–159, P); Adler (2012: 35–37, P).

**1770**. *A catalogue of British insects.* William Eyres, Warrington. 16 pp. (8vo) [DP: 1770 (title page; preface dated 1 August 1770)] OLIL (microfilm)

**1771**. *A catalogue of the animals of North America. Containing an enumeration of the known quadrupeds, birds, reptiles, fish, insects, crustaceous and testaceous animals; many of which are new, and never described before. To which are added, short directions for collecting, preserving, and transporting, all kinds of natural history curiosities.* B. White, London. 43 pp. (8vo) [DP: 1771 (title page; dedication dated 24 April 1771), July 1771 (*Crit Rev*), August 1771 (*Scots Mag*), October 1771 (*Monthly Rev*)] GB, BHL

This booklet contains a simple list of species found in North America; several new species are named but not described. It was reissued in 1882 by The Willughby Society under the title “*Forster’s catalogue of the animals of North America, or Faunula Americana*.” The insect section (pp. 23–33) was reissued in Pennant (1787: 151–162).

**1771**. *Novae species insectorum. Centuria I.* T. Davies et B. White, Londini. viii + 100 pp. (8vo) [DP: 1771 (title page; preface dated 7 October 1771), November 1771 (*Crit Rev*; *Scots Mag*), December 1771 (*Monthly Rev*; *Gent Mag*)] CNC, GB

Only the first “Centuria” was published.

**1788**. *Enchiridion historiae naturali inserviens, quo termini et delineationes ad avium, piscium, insectorum et plantarum adumbrationes intelligendas et concinnandas, secundum methodum systematis Linnaeani continentur.* Hemmerde et Schwetschke, Halae [= Halle]. [16] + 224 pp. (8vo) [DP: 1788 (title page; preliminaries dated 15 February 1788), 27 March 1788 (*Gött Anz*; Mathews 1925: 42), 15 October 1788 (*Allg Lit Ztg*)] CNC, GB, BHL

The Coleoptera are on pages 144–149. A “*Editio altera emendatior*,” essentially a reset of the first edition (Sherborn 1902: xxiv), was issued in 1794 in Edinburgh (*n.v.*) and in 1797 in London [OLIL-online]. An emended French translation by J.B.F. Léveillé was published, “An VII” in Paris, under the title “*Manuel pour servir à l’histoire naturelle des oiseaux, des poissons, des insectes et des plantes; où sont expliqués les termes employés dans leurs descriptions, et suivant la méthode de Linné; traduit du latin de J. Reinhold Forster : augmenté d’un mémoire de Murray sur la conchyliologie, traduit de la même langue, et de plusieurs additions considérables extraites des ouvrages des Cit. Lacépède, Jussieu, Lamarck, Cuvier, etc.*” [GB]; it was noticed on 8 June 1799 (*J Typogr Bibl*).

**1795**. *Faunula Indica id est catalogus animalium Indiae orientalis quae hactenus naturae curiosis innotuerunt, concinnatus a Joanne Latham et Hugone Davies. Secundis curis editus, correctus et auctus.* Joannis Jacobi Gebaueri, Halae ad Salam [=Halle an der Saale]. [2] + 38 pp. (Folio) [DP: 1795 (title page; *praefatiuncula* dated 20 June 1795), 26 December 1795 (*Allg Lit Ztg*), December 1795 (*Gior Fis-Med*)] GB

This work consists of a list of the animals of East India. The Coleoptera are on pages 16–20.

#### Foudras, Antoine Casimir Marguerite Eugène (Lyon, Rhône, France: 19 November 1783 – 13 April 1859: Lyon, France). French lawyer in Lyon and naturalist; his collection was bequeathed to the Lycée Ampère in Lyon and later transferred to the Museum in Lyon. Reference. Mulsant (1860a, P).

**1860**. *Histoire naturelle des Coléoptères de France, par E. Mulsant. Altisides.* Magnin, Blanchard et C^ie^, Paris. 384 pp. (8vo) [DP: “1859–March 1860” (title page), 26 May 1860 (*Bibl Fr*), 30 May 1860 (*J Instr Publ*), June 1860 (*Allg Bibl*)] CNC, GB

This work is part of Mulsant’s grandiose project on the Coleoptera of France. It was also issued in the *Annales de la Société Linnéenne de Lyon* (Nouvelle Série) 6 [1859]: 137–384 [DP: “1859–January 1860” (title page), 16 June 1860 (*Bibl Fr*), 20 June 1860 (*J Instr Publ*)] and 7 [1860]: 17–128 [DP: 1860 – February 1861 (title page), 3 August 1861 (*Bibl Fr*)].

Page 1–23 is a “Notice sur Antoine-Casimir-Marguerite-Eugène Foudras” by Mulsant.

#### Fourcroy, Antoine[-]François de (Paris, France: 13 June 1755 – 16 December 1809: Paris, France). French chemist, physician, naturalist and counselor of state; professor of chemistry at the Ecole polytechnique and the Jardin du Roi (which became the Jardin des Plantes and later the Muséum National d’Histoire Naturelle) in Paris; supporter of the French revolution; director-general of public education in 1801; commander of the Legion of Honour. References. Palisot de Beauvois (1810); Rose (1848b: 426–427); Kersaint (1966, P); Jaussaud (2004: 227–228); Frank (2008 : 1524–1525).

**1785**. *Entomologia Parisiensis; sive catalogus insectorum quae in agro Parisiensi reperiuntur; secundum methodum Geoffraeanam in sectiones, genera & species distributus: cui addita sunt nomina trivalia & fere trecentae novae species. Pars prima.* Via et Aedibus Serpentineis, Parisiis. vii + [1] + 231 pp. (18 mo) [DP: 1785 (title page; certified by the *Académie des Sciences* on 13 July 1785 [recto of page vii]), before 1 August 1785 (Dawson 1958: 162), 12 August 1785 (*Gaz Fr*)] CNC, GB, BHL, GAL

This work was published in two parts, issued simultaneously, with continuous pagination. The Coleoptera are in the first part on pages 1–174 and 176. The book was reissued in 1805 by Méquignon in Paris (*n.v.*) as noted in the *Journal Général de la Littérature de France* 8: 98. All new taxa validated in this work are attributed to Geoffroy, as discussed by Ganglbauer and Heyden (1906: 301–302) and Cameron (1988).

Menke (1988) pointed out that this work is not consistently binomial and that under the *Code* [Article 11.4] the names are not available. There are about 1690 species described and named in both parts. Of these names only four are probably not binominal (Miguel A. Alonso-Zarazaga, personal communication): one in the first part (*Psylla chermes buxi*, p. 223) and three in the second part (*Phaloena pavonia major*, p. 257; *Phaloena pavonia media*, p. 257; *Phaloena pavonia minor*, p. 258). Sherborn (1902) retained one of these names, *Psylla chermes-buxi* (p. 200).

#### Fournel, Dominique Henri Louis (Génicourt, Meuse, France: 23 March 1813 – 25 August 1846: Metz, Moselle, France). French naturalist; professor of natural history at the Collège Royal in Metz. References. Malherbe (1847); Tribout de Morembert (1979).

**1840**. *Faune de la Moselle, ou manuel de zoologie, contenant la description des animaux libres ou domestiques observés dans le Département de la Moselle; ouvrage rédigé d’après la méthode de Cuvier. 2.^e^ partie. Animaux articulés. Tome premier. Annélides, crustacés, arachnides, insectes myriapodes et hexapodes, jusqu’aux lamellicornes exclusivement.* Verronnais, Metz. xix + [1] + 624 pp. (12mo) [DP: 1840 (title page), 15 August 1840 (*Echo Monde Sav*), August 1840 (*Mém Encyc*), 10 October 1840 (*Bibl Fr*), 22 October 1840 (*Bibl Blätt*), 23 October 1840 (*Allg Bibl Deutsch*), 28 October 1840 (*Lit Ztg*)] GB

The entire work consists of two volumes, 1836–1840. The Coleoptera are in the second volume (first volume of the second part) on pages 182–588. The second volume of the second part was planned but never published.

#### Fowler, William Weekes (Tavistock, Devon, United Kingdom: 10 January 1849 – 3 June 1923: Earley, Berkshire, United Kingdom). British clergyman and entomologist interested mainly in Coleoptera; headmaster of Lincoln Grammar School (1880–1900); rector of Rotherfield Peppard, near Henley, Oxfordshire (1901–1904); vicar of St. Peters, Earley; his collection is at the Nottingham Natural History Museum. Reference. Walker (1923).

**1887–1891**. *The Coleoptera of the British Islands. A descriptive account of the families, genera, and species indigenous to Great Britain and Ireland, with notes as to localities, habitats, etc.* L. Reeve & Co., London. (4to) CNC, BHL

*Vol. I. Adephaga–Hydrophilidae.* xxxii + 269 pp. + 38 pls (numbered A–B, 1–36). [DP: 1887 (title page; preface dated November 1886), 17 February 1887 (*Nature*), 26 March 1887 (*Athenaeum*), 11 June 1887 (*Academy*)].

*Vol. II. Staphylinidae.* [1] + 444 pp. + pls 37–70. [DP: 1888 (title page), 8 October 1888 (*Zool Anz*), October 1888 (*Nat Nov*)].

*Vol. III. Clavicornia (Leptinidae–Heteroceridae).* 399 pp. + pls 71–97. [DP: 1889 (title page), June 1889 (*Nat Nov*)].

*Vol. IV. Lamellicornia–Serricornia–Longicornia–Phytophaga.* 411 pp. + pls 99–142. [DP: n.d. (title page), July 1890 (*Nat Nov*), 10 October 1890 (*Bookseller*), 11 October 1890 (*Spectator*)].

*Vol. V. Heteromera–Rhynchophora–Abnormal Coleoptera.* xxviii + 490 pp. + pls 143–180. [DP: 1891 (title page; preface dated July 1891), 3 October 1891 (*Publ Circ*), 10 October 1891 (*Bookseller*), October 1891 (*Nat Nov*), 5 December 1891 (*Athenaeum*)].

This work was issued in 53 parts and the dates listed here for each volume are those in which the completed volumes were advertised. The collation for the 53 parts, as recorded in the *Zoological Record*, is as follows: **1–4**: (pp. 1–128 of vol. 1) 1886; **5–13**: (pp. 129–269 of vol. 1 + pp. 1–160 of vol. 2) 1887; **14–24**: (pp. 161–444 of vol. 2 + pp. 1–160 of vol. 3) 1888; **25–36**: (pp. 161–399 of vol. 3 and pp. 1–224 of vol. 4) 1889; **37–48**: (pp. 225–411 of vol. 4 + pp. 1–?352 of vol. 5) 1890; **49–53**: (pp. ?353–490 of vol. 5) 1891. The books were also issued in a smaller format without the colored plates. A supplement, forming volume 6 of the series and authored by W.W. Fowler and H. St. John Donisthorpe, was published in London in 1913.

#### Francillon, John^30^ (1743/44 – 23 June 1816). British jeweller in London and collector of natural history objects; acted as John Abbot [1751–*ca* 1840] agent, distributing his specimens and paintings to naturalists in England and Europe; his collections of insects were sold at auction in May and July 1817 (Bristish insects) and June 1818 (exotic insects). References. Cowan (1986); Leibee (2003).

**1795**. *Description of a rare* Scarabaeus, *from Potosi, in South America; with engraved representations of the same, coloured from nature.* C. Whittingham, London. [2] pp. + 1 pl. (4to) [DP: 1795 (title page), 17 February 1796 (*Allg Lit Ztg*)] CMLE, GB

The species described is *Scarabaeus macropus*.

#### Fricken, Heinrich Wilhelm Maria von (Ahaus, North Rhine-Westphalia, Germany: 27 November 1833 – 29 December 1890: Wiesbaden, Hesse, Germany). German educator; professor of natural history at the Städtische Gymnasium Laurentianum in Arnsberg, Germany. Reference. Rassmann (1881: 67).

**1869**. *Naturgeschichte der einheimischen Käfer nebst analytischen Tabellen zum Selbstbestimmen. Für Lehrer und Studirende und alle Freunde wissenschaftlicher Entomologie*. *Mit 63 in den Text gedruckten holzschnitten.* H.F. Grote, Arnsberg. viii + 222 + [1 (Berichtigungen)] pp. (8vo) [DP: 1869 (title page; *Vorwort* dated “Ostern 1869”),16 April 1869 (*Börsen Deutsch Buch*), 29 April 1869 (*Allg Bibl Deutsch*), January–June 1869 (*Bibliot Hist-Nat*)] GB

A “*Zweite, sehr vermehrte und verbesserte Auflage*” containing xvi + 344 pages was issued in 1872 by the same publisher [ULBM]; it was recorded on 3 August 1872 (*Ver Natur Zwickau*). A third (1880), fourth (1885) and fifth (1906) editions were also published.

#### Fritzen, Rudolf

**1865.** *Preussische Käfer. In systematischer Reihenfolge bearbeitet.* H. Brandenburg, Neustadt in Westpreussen [= Wejherowo]. [1] + 59 pp. (8vo) [DP: 1865 (title page; *Vorwort* dated January 1865), 9 February 1865 (*Allg Bibl Deutsch*), January–July 1865 (*Bibliot Hist-Nat*)] YB (ph, partial)

This work was to be published in five or six parts but only the first one was issued.

#### Frivaldszky, Janos [Ján, johann, joanne] (Rajec near Trenčín, Slovakia: 17 June 1822 – 29 March 1895: Budapest, Hungary). Hungarian entomologist particularly interested in cave dwelling arthropods, and later ornithologist; graduated in engineering but choose to become zoologist; curator at the Hungarian Natural History Museum in Budapest (1852–1895); his collection is at that museum. References. Horváth (1897, P); Kříž (2005, P); Hangay (2008).

**1897**. Coleoptera, Lepidoptera, Orthoptera et Neuroptera. Pp. 665–690 *in*: *Gróf Széchenyi Béla Keletázsiai utjának tudományos Eredménye. 1877–1880. Második Kötet. A gyűjtött anyag feldolgozása. 13 szövegközti ábratáblával és 21 lithographált táblával.* Kilián Frigyes Egyetemi Könyvárus Bizományában, Budapest. x + 877 + [4] pp. (4to) [DP: 1897 (title page; *előszó* dated 15 October 1896), 14 February 1897 (*Vas Ujs*)] NCSU

The scientific results of Count B. Széchenyi’s travels in eastern Asia consists of three volumes and an atlas (Folio), 1890–1897. One genus and several species of Coleoptera are listed as new taxa in Frivaldszky’s contribution but all of them were previously described in 1892 in *Természetrajzi Füzetek* 15: 114–125.

#### Fröhlich, Carl August Anton Tobias (Aschaffenburg, Bavaria, Germany: 31 July 1850 – 28 October 1928: Aschaffenburg, Germany). German practicing physician in Aschaffenburg. Reference. Marquart (2009: 246).

**1897**. *Beiträge zur Fauna von Aschaffenburg und Umgegend. III. Mittheilung des naturwissenschaftlichen Vereines daselbst. Die Käfer.* Gustav Fischer, Jena. vii + 158 pp. (8vo) [DP: 1897 (title page), August 1897 (*Nat Nov*), 12 September 1897 (*Naturwiss Wochen*), 11 October 1897 (*Zool Anz*), 30 October 1897 (*Naturwiss Rund*), 30 November 1897 (*Wien Ent Ztg* 16: 254), November 1897 (*Ent Nachr* 23: 327)] YB

This work is a catalogue of the Coleoptera of Aschaffenburg and its environs in Bavaria, Germany, with notes.

#### Froriep, Ludwig Friedrich von (Erfurt, Thuringia, Germany: 15 June 1779 – 28 July 1847: Weimar, Thuringia, Germany). German obstetrician, surgeon and entrepreneur; professor of obstetrics in Halle; professor of anatomy and surgery at the University of Tübingen; later, moved to Weimar and took over the Landes-Industrie-Comptoir owned by his father-in-law. References. Döring (1849); Dechambre (1880); Häfen (2007, P).

**1806**. *C. Dumeril’s Analytische Zoologie. Aus dem französischen, mit Zusätzen*. Landes-Industrie-Comptoirs, Weimar. vi + 343 + [1] pp. (8vo) [DP: 1806 (title page; *Vorrede* dated 18 September 1806), 15 October 1806 (*Intell Allg Lit Ztg*), November 1806 (*Monats-Bericht Landes*), July–December 1806 (*Verz Bücher Landkarten*)] GB

This book is a German translation of Duméril’s *Zoologie Analytique* published in 1805 [*q.v.*]. Froriep added material in the translation and provided the first inclusion of species to some of Duméril’s new genera.

#### Fuessly [Füssli, Füssly, Fuesslin], Johann [Hans] Kaspar [Caspar] (Zürich, Switzerland: 9 March 1743 – 4 May 1786: Winterthur, Switzerland). Swiss entomologist, artist and publisher in Zürich. References. Rose (1848b: 458); Evenhuis (1997a: 280, P).

**1775**. *Verzeichnis der ihm bekannten schweizerischen Inseckten mit einer ausgemahlten Kupfertafel: nebst der Ankündigung eines neuen Insecten Werks.* Heinrich Steiner und Compagnie, Zürich und Winterthur. xii + 62 pp. + 1 pl. (4to) [DP: 1775 (title page; *Vorrede* dated 24 February 1775), 18 August 1775 (*Jena Ztg*), 14 October 1775 (*Erlang Anmer Nachr*), 11 November 1775 (*Gött Anz*)] MCZ, GB, BHL

The Coleoptera are on pages 1–21. The ICZN (1958) determined in Opinion 516 that Fabricius’ (1775) *Systema entomologiae* has precedence over this publication.

**1794**. *Archives de l’histoire des insectes, publiées en Allemand par Jean Gaspar Fuessly. Traduites en François.* J. Ziegler, Winterthour. xii + 184 + [2] pp. + 50 pls^31^. (4to) [DP: 1794 (title page; preface dated 18 June 1793), 12 April 1794 (*Intell Allg Lit Ztg*)] CMNL, GB

This book contains the French translation of papers published in the journal *Archiv für Naturgeschichte* (1781–1786). The following ones pertain to Coleoptera: “Mémoire sur le genre *Cucujus*” by J.F.G. Herbst (p. 21–24); “Le Pausse *Cerocoma microcephala*. Fabr. Ent. Syst. Tom. I. part. 2. pag. 82. n°4.” by Andreas Dahl (p. 42); “Description de quatre capricornes rares. (*Cerambices*. Linn. et Fabr.) *Pl.* 32.” by J.R. Forster (p. 56–59); “Catalogue critique des insectes du Cabinet de M. Herbst.” by J.F.G. Herbst (p. 65–175). The book also contains a preface (p. v–xii), an alphabetic table of the insects mentioned in the book (p. 177–184), an “errata” sheet (p. [185]) and a notice to the binder (p. [186]).

The plates are numbered 1–18, 19a, 19b, 20–27, 28a, 28b, 29–36, 43–54. They were engraved by Johann Rudolph Schellenberg after Bertram, Herbst, Köchlin, Stolzma, Schrank and Schaffer. The translation was done by Joseph Philippe de Clairville (Jäggli 1958: 199).

According to Brunet (1845: 116), a printing of this volume was also issued in Zürich (*n.v.*).

**1795**. *Archives of entomology, containing the history, or ascertaining the characters and classes of insects not hitherto described, imperfectly known, or erroneously classified. Translated from the German of J.C. Fuessly; with notes, and the original plates, fifty-one in number, coloured. To which is added the French translation.* J. Johnson, London. iv + 49 + xii + 184 + [2] pp. + 51 pls. (4to) [DP: 1795 (title page; advertisement dated 15 April 1795), May 1795 (*Analyt Rev*)] OLIL-online, MCZ-online

This book contains English translation of the papers provided in the French translation (see previous entry) followed by a reissue of the French translation.

#### Fuss, Karl [Carl] Adolf (Sibiu, Romania: 29 October 1817 – 1 July 1874: Neudorf, Romania). Transylvanian pastor, educator and entomologist in Sibiu; his Coleoptera collection is at the Hungarian Natural History Museum in Budapest. References. Trausch (1868: 390); Anonymous (1876b); Teutsch (1878); Vlad-Antonie (2000, P).

**1857**. Die Käfer Siebenbürgens. Pp. 3–36 *in*: *Programm des Gymnasiums A.C. zu Hermannstadt und der mit demselben verbundenen Lehranstalten für das Schuljahr 185^6/7^. Veröffentlicht vom Direktor des Gymnasiums, Josef Schneider.* Diözesan, Hermannstadt. 67 pp. (4to) [DP: 1857 (title page), 8 November 1857 (*Ent Ver Stettin*), January–June 1858 (*Bibliot Hist-Nat*)] YB (ph)

This work consists of a systematic account of the Coleoptera of Transylvania, a region in northwest and central Romania. The genera are described and the species keyed. This section includes the Cicindelidae and part of the Carabidae.

**1858**. Die Käfer Siebenbürgens. Pp. 3–65 *in*: *Programm des Gymnasiums A.C. zu Hermannstadt und der mit demselben verbundenen Lehranstalten für das Schuljahr 185^7/8^. Veröffentlicht vom Direktor des Gymnasiums, Josef Schneider.* Diözesan, Hermannstadt. 92 pp. (4to) [DP: 1858 (title page), 4 August 1858 (*Zool-Bot Gesell Wien*)] YB (ph, partial)

This section includes the second and last part of the Carabidae.

#### Gahan, Charles Joseph (Roscrea, Ireland: 20 January 1862 – 21 January 1939: Aylsham, Norfolk, United Kingdom). British entomologist; worked at the British Museum (Natural History) from 1886 to 1926 first as an assistant in the Department of Zoology and later as curator in the Department of Entomology; did not seem to have a personal collection but the types of the species he described are at the Natural History Museum in London. Reference. Arrow (1939, P).

**1892**. Notes and descriptions. Pp. 206–210 *in*: *A naturalist in the Transvaal. By W.L. Distant. With coloured plates and original illustrations.* R.H. Porter, London. xvi + 277 pp. + 5 pls. (8vo) [DP: 1892 (title page; preface dated February 1892), 9 April 1892 (*Athenaeum*; *Spectator*), April 1892 (*English Cat Books*; *Nat Nov*), 2 May 1892 (*Author*), July 1892 (*Nat Sci*)] MCZ, BHL, GB

The new species described in Gahan’s contribution are: *Ootheca modesta* (p. 206), *Spilocephalus distanti* (p. 208), *Aenidea ? pretoriae* (p. 209) and *Ancylopus fuscipennis* (p. 210).

#### Galdo López de Neira, Manuel María José de (Madrid, Spain: 16 January 1825 – 19 July 1895: Madrid, Spain). Spanish politician and professor of natural history in Madrid. References. Rodríguez Guerrero (2009: 368–374, P); Gomis (2012, P); Anonymous (2015b, P).

**1856**. *Los tres reinos de la naturaleza. Museo pintoresco de historia natural. Descripcion completa de los animales, vegetales y minerales utiles y agradables; su forma, instinto, costumbres, virtudes ó aplicaciones á la agricultura, la medicina, y las artes en general, comprendiendo mayor numero de géneros que en todas las obras publicadas hasta et dia, con un tratado de geologia, ó teorfas actuales sobre la formacion y revoluciones del globo, y un bosquejo historico de los progresos de las ciencias naturales en general y en España: obra arreglada sobre los trabajos de los mas eminentes naturalistas de todos los paises, Buffon, Blanchard, Boitard, Brongniard, Cavanilles, los Cuvier, Daubenton, de Candolle, Humboldt, los Jussieu, Lacepede, Lagasca, Lamarck, Latreille, Lesson, Linneo, d’Orbigny, Rousseau, Saint Hilaire, Saint Pierre, Virey, Werner, etc. Con todos los descubrimientos posteriores hasta el dia, bajo la direccion de M. M. J. de Galdo. É ilustrada con una magnifica y numerosa coleccion de láminas en vista del natural, y los planos del Gabinete de Historia Natural y del Jardin Botánico de Madrid. Tomo VI. Zoologia.* Gaspar y Roig, Madrid. 708 + [1] pp. + 83 pls. (4to) [DP: 1856 (title page)] GB

The entire series was published in nine volumes, 1852–1858. The Coleoptera are on pages 85–372 of volume 6.

#### Galeazzi, Giacomo (? – 1869).

**1854**. *Coleoptera Europae dupleta quae pro mutua commutatione offerri possunt.* Milano. 19 pp. (8vo) [DP: 1854 (title page)] YB (ph) 

This is a list of duplicate European Coleoptera offered for mutual exchange. No new species are described.

#### Ganglbauer, Ludwig (Vienna, Austria: 1 October 1856 – 5 June 1912: Rekawinkel near Vienna, Austria). Austrian naturalist; worked at the Wiener Hofmuseum (which became later the Naturhistorisches Museum) and graduated to become director of the zoology department; the specimens he collected and his types are at the Naturhistorisches Museum in Vienna. References. Spaeth (1913a, P; 1913b, P); Dolezal (1964); Smetana and Herman (2001: 68–69, P); Klausnitzer (2003: 96–99, P).

**1891–1904**. *Die Käfer von Mitteleuropa. Die Käfer der österreichisch-ungarischen Monarchie, Deutschlands, der Schweiz, sowie des französischen und italienischen Alpengebietes.* Carl Gerold’s Sohn, Wien. (8vo) CNC, BHL 

*Erster Band. Familienreihe Caraboidea. Mit 55 Holzschnittfiguren im Text.* iii + 557 pp. [DP: 1892 (title page; *Vorwort* dated 14 November 1891; colophon dated 16 November 1891), 7 December 1891 (*Nied Gesell Natur Heil*), 24 December 1891 (*Allg Bibl Deutsch*), December 1891 (*Nat Nov*), 23 April 1892 (*Deutsch Litt*)]. 

*Zweiter Band. Familienreihe Staphylinoidea. 1. Theil: Staphylinidae, Pselaphidae. Mit 38 Holzschnittfiguren im Text.* vi + 880 + [1 (Corrigenda)] pp. [DP: 1895 (title page; *Vorwort* dated March 1895), April 1895 (*Nat Nov*), 4 May 1895 (*Deutsch Litt*), June 1895 (*Rev Ent* 14: 178; *Ent News* 6: 192; *Ent Monthly Mag* 31: 145), 6 July 1895 (*Lit Centr Deutsch*), 25 August 1895 (*Wien Ent Ztg* 14: 260)]. 

*Dritter Band. Familienreihe Staphylinoidea. II. Theil. Scydmaenidae, Silphidae, Clambidae, Leptinidae, Platypsyllidae, Corylophidae, Sphaeriidae, Trichopterygidae, Hydroscaphidae, Scaphidiidae, Histeridae. Familienreihe Clavicornia. Sphaeritidae, Ostomidae, Byturidae, Nitidulidae, Cucujidae, Erotylidae, Phalacridae, Thorictidae, Lathridiidae, Mycetophagidae, Colydiidae, Endomychidae, Coccinellidae. Mit 46 Holzschnitten im Text.* iii + 1046 pp. [DP: 1899 (title page; *Vorwort* dated June 1899)]. This *Band* was issued in two parts: (pp. 1–408) 13 January 1899 (*Zool-Bot Gesell Wien*), 31 January 1899 (*Wien Ent Ztg* 18: 40), January 1899 (*Nat Nov*), 22 February 1899 (*Soc Ent Fr*), 27 March 1899 (*Bibl Zool*), 15 April 1899 (*Biol Centr*); (pp. 409–1046 + iii) September 1899 (*Nat Nov*), 11 October 1899 (*Soc Ent Fr*), 15 November 1899 (*Wien Ent Ztg* 18: 288), 24 November 1899 (*Bibl Zool*), November 1899 (*Ent News*)]. Sharp (1899: 27) suggested that the first part may have been published at the end of 1898 but the wrapper is dated 1899. 

*Vierter Band, erste Hälfte. Dermestidae, Byrrhidae, Nosodendridae, Georyssidae, Dryopidae, Heteroceridae, Hydrophilidae. Mit 12 Holzschnitten im Text.* 286 pp. [DP: 1904 (title page), August 1904 (*Nat Nov*), 17 September 1904 (*Zool Anz*)]. 

**1892**. Käfer der Teleki-Expedition. Pp. 828–847 *in*: *Zum Rudolph-See und Stephanie-See. Die Forschungsreise des Grafen Samuel Teleki in Ost-Aequatorial-Afrika 1887–1888 geschildert von seinem Begleiter Ludwig Ritter von Höhnel. Mit 179 Original-Illustrationen und 2 grossen Karten in reichem Farbendruck.* Alfred Hölder, Wien. xviii + 877 pp. (8vo) GB 

 The book was issued in 30 parts as follows: **1–3**: (pp. 1–96) December 1891 (*Nat Nov*); **4–6**: (pp. 97–176) February 1892 (*Nat Nov*); **7–8**: (pp. 177–240) March 1892 (*Nat Nov*); **9–13**: (pp. 241–400) May 1892 (*Nat Nov*); **14–17**: (pp. 401–528) July 1892 (*Nat Nov*); **18–19**: (pp. 529–592) August 1892 (*Nat Nov*); **20–24**: (pp. 593–752) October 1892 (*Nat Nov*); **25–30**: (pp. 753–877) December 1892 (*Nat Nov*). The title page is dated 1892 and the *Vorwort* May 1892. 

 Ganglbauer’s contribution consists of a list of Coleoptera, with localities, collected during the expedition; the new species were described by Léon Fairmaire in the *Annales de la Société Entomologique de Belgique* 35 [1891]: cclxxix–cccvii. 

 An English translation was published in two volumes in London, 1894, under the title “*Discovery of Lakes Rudolf and Stefanie. A narrative of Count Samuel Teleki’s exploring & hunting expedition in eastern equatorial Africa in 1887 & 1888 by his companion Lieut. Ludwig von Höhnel*” [BHL]. Ganglbauer’s input is in the second volume on pages 329–345. 

**1894**. Coleoptera. Pp. 341–348 *in*: *Durch Massailand zur Nilquelle. Reisen und Forschungen der Massai-Expedition des deutschen Antisklaverei-Komite in den Jahren 1891–1893. Von Dr. Oscar Baumann. 386 Seiten Text mit 27 Vollbildern und 140 Text–illustrationen in Heliogravüre, Lichtdruck und Autotypie nach Photographien und Skizzen des Verfassers von Rud. Bacher und Ludwig Hans Fischer in Wien und einer Originalkarte in 1: 1,500,000 reducirt von Dr. Bruno Hassenstein.* Dietrich Reimer, Berlin. xiii + [1] + 385 + [1 (Druckfehler-Berichtigung)] pp. + 27 pls. (8vo) [DP: 1894 (title page), 24 May 1894 (*Nation* 58: 397), May 1894 (*Nat Nov*), 15 June 1894 (*Soc Géog Fr*), July 1894 (*Geog J* 4: 87)] ANSP, GB 

 Ganglbauer’s contribution consists of a list of Coleoptera collected during the Maasi Expedition (1891–1893), held under the direction of Oscar Baumann [1864–1899], with localities. No new species are described. The book was reprinted in 1968, New York, as part of the *Landmarks in Anthropology* series [McG]. 

#### Gaubil, Jean. French officer in the 17^th^ Light Infantry and coleopterist; Knight of the Legion of Honour; collected extensively in Algeria and Alsace. Reference. Constantin (1992: 39).

**1849**. *Catalogue synonymique des coléoptères d’Europe et d’Algérie*. Maison, Paris. 296 + [1 (Espèces nouvelles publiées par M. Léon Fairmaire, dans les Annales de la Société entomologique de France)] pp. (8vo) [DP: 1849 (title page; preface dated 15 July 1848), August 1849 (*Ent Ztg* 10: 256), November 1849 (*Rev Mag Zool*), 28 February 1850 (*Ent Ver Stettin*), 25 May 1850 (*Bibl Fr*)] GB, GAL, ARC

#### Gebauer, Christian August (Knobelsdorf [currently in Waldheim], Saxony, Germany: 28 August 1792 – 15 November 1852: Tübingen, Baden-Württemberg, Germany). German poet and naturalist; professor of philosophy at the University of Bonn (1818–1823); afterwards, lived in Mannheim, thence Stuttgart, Karlsruhe and finally Tübingen; often wrote under the pseudonym of Heinrich Rebau. References. Brümmer (1876: 225–226); Schott (1878).

[**1849**]. *Käfer-Büchlein oder Beschreibung der schönsten, nützlichsten und schädlichsten in- und ausländischen Käfer. Nebst einer kurzen Anweisung, Käfer zu fangen und sie, nebst ihren Eiern, Larven und Puppen für Sammlungen herzurichten. Für Knaben, die sich in ihren Freistunden gern angenehm und nützlich beschäftigen wollen, so wie für Landwirthe, Forstmänner und Gartenbesitzer. Herausgegeben zum Verfasser des Schmetterlingsbüchleins. Mit 5 Tafeln Abbildungen.* Fleischhauer & Spohn, Reutlingen. xvi + 61 pp. + 5 pls. (4to) [DP: n.d. (title page), July–December 1849 (*Verz Bücher Landkarten*)] SBB

Three subsequent “editions” of this work were issued, in ?1856 (*n.v.*), 1866 [BHL, GB, MDZ] and 1877 [SBB], all essentially reprints of the original edition with only a change in the title pages. The third and fourth printings have the name Heinrich Rebau as the author on the title pages.

#### Gebler, Friedrich [Fedor] August von (Zeulenroda, Thuringia, Germany: 15 December 1782 – 21 March 1850: Barnaul, Siberia, Russia). German physician and naturalist; medical inspector of the Altai mining district; his collection was acquired by Georges Mniszech and subsequently by René Oberthür [*q.v.*]. Reference. Mannerheim (1850).

**1830**. Bemerkungen über die Insekten Sibiriens, vorzüglich des Altai. Pp. 1–228 *in*: *Carl Friedrich von Ledebour’s Reise durch das Altai-Gebirge und die soongorische Kirgisen-Steppe. Auf Kosten der Kaiserlichen Universität Dorpat unternommen im Jahre 1826 in Begleitung der Herren D. Carl Anton Meyer und D. Alexander von Bunge. Zweiter Theil. Mit Kupfern und Karten.* G. Reimer, Berlin. 522 + 228 pp. (8vo) [DP: 1830 (title page), 4 May 1830 (*Intell Ztg Welt*), February–May 1830 (*Foreign Quart Rev*), 16 August 1830 (*Lit-Blatt*), October 1830 (*Bull Sci Nat Géol* 23: 24)] MCZ, GB

Ledebour’s *Reise durch das Altai-Gebirge* was published in two volumes, 1829–1830. Gebler’s contribution is in the second volume and separately paginated.

#### Géhin, Joseph Jean Baptiste (Remiremont, Vosges, France: 1 September 1816 – 2 December 1889: Remiremont, France). French pharmacist in Metz (Moselle) and amateur entomologist with a particular interest in the family Carabidae; his collection of *Carabus* and nearby groups was sold just before his death to René Oberthür [*q.v.*] and is now at the Muséum National d’Histoire Naturelle in Paris. References. Quépat (1887: 182); Constantin (1992: 39); Airale and Labrude (2004); Cambefort (2006: 176).

**1851–1852**. *Catalogue des coléoptères de la collection de J.-B. Géhin.* Dembour et Gangel [fasc. 1] / Gangel [fasc. 2], Metz. (8vo) MCZ

*1er fascicule. Famille des cicindeliens.* 22 + [1] pp. [DP: 1851 (title page; page 7 dated January 1851), 26 April 1851 (*Bibl Fr*), April 1851 (*Rev Mag Zool*), 22 May 1851 (*Ent Ver Stettin*), January–June 1851 (*Bibliot Hist-Nat*)].

*2e fascicule. Dytisciens.– Gyriniens.* [1] + 24 pp. [DP: 1852 (title page; page 6 dated August 1852), 7 November 1852 (*Ent Ver Stettin*)].

**1876**. *Catalogue des coléoptères carabiques de la tribu des carabides.* Nancy. [3] + 72 pp. (8vo) [DP: 1876 (title page; last page dated 10 July 1876), 1 August 1876 (*Pet Nouv Ent*)] CNC, ANXL

**1879**. *Nouvelles lettres pour servir à l’histoire des insectes de la tribu des carabides.* Sordoillet, Nancy. 24 pp. (8vo) [DP: November 1879 (title page), 15 January 1880 (*Naturaliste*), 13 March 1880 (*Bibl Fr*), March 1880 (*Nat Nov*), 3 May 1880 (*Zool Anz*), January–June 1880 (*Bibliot Hist-Nat*)] MCZ

**1885**. *Catalogue synonymique et systématique des coléoptères de la tribu des carabides. Avec des planches dessinées par Ch. Haury.* Remiremont [&] Prague. xxxviii + 104 pp. + 10 pls. (8vo) [DP: 1885 (title page; *introduction* dated 1 December 1885), 24 February 1886 (*Soc Ent Fr*), February 1886 (*Nat Nov*)] CNC

A lengthly criticism of this catalogue was published by Kraatz (1886).

#### Gemminger, Max (Munich, Bavaria, Germany: 22 January 1820 – 18 April 1887: Munich, Germany). German coleopterist; curator at the Zoological Museum [currently the Zoologische Staatssammlung München] in Munich (1849–1887); his collection is at the same museum. References. Will (1887); Scherer (1992: 62, P).

**1851**. *Systematische Uebersicht der Käfer um München. Ein Beitrag zu den Localfaunen Deutschlands. Mit einem Stahlstich.* Friedrich Mauke, Jena. x + 65 pp. + 1 pl. (4to) [DP: 1851 (title page; *Vorwort* dated April 1851), 4 September 1851 (*Ent Ver Stettin*; *Allg Bibl Deutsch*), 15 September 1851 (*Intell Serapeum*), July–December 1851 (*Bibliot Hist-Nat*), 5 January 1852 (*Ent Soc Lond*)] GB, MDZ, CMLE (mf)

This work is a simple checklist of the Coleoptera found around Munich.

**1868–1876**. [Gemminger, M. and Harold, E. von] *Catalogus coleopterorum hucusque descriptorum synonymicus et systematicus.* E.H. Gummi [vols 1–9] / G. Beck [vols 10–11] / Theodor Ackermann [vol. 12], Monachii [= Munich]. (8vo) CNC, BHL

*Tom. I. Cicindelidae – Carabidae.* xxxvi + 424 + [8 (Index Generum ad Tom. I.)] pp. [DP: 1868 (title page; preface dated June 1868), 20 August 1868 (*Allg Bibl Deutsch*), August 1868 (*Allg Bibl*), 2 November 1868 (*Ent Soc Lond*), 7 November 1868 (*Königl Akad Wiss Münc*), July–December 1868 (*Bibliot Hist-Nat*)].

*Tom. II. Dytiscidae, Gyrinidae, Hydrophilidae, Staphylinidae, Pselaphidae, Gnostidae, Paussidae, Scydmaenidae, Silphidae, Trichopterygidae, Scaphididae.* Pp. 425–752 + [6 (Index Generum ad Tom. II.)]. [DP: 1868 (title page), 7 November 1868 (*Königl Akad Wiss Münc*), 10 December 1868 (*Allg Bibl Deutsch*), December 1868 (*Allg Bibl*), July–December 1868 (*Bibliot Hist-Nat*), 4 January 1869 (*Ent Soc Lond*)].

*Tom. III. Histeridae, Phalacridae, Nitidulidae, Trogositidae, Colydidae, Rhysodidae, Cucujidae, Cryptophagidae, Derodontidae, Latrididae, Othnidae, Mycetophagidae, Thorictidae, Dermestidae, Byrrhidae, Georyssidae, Parnidae, Heteroceridae, Lucanidae.* Pp. 753–978 + [5 (Index Generum ad Tom. III)]. [DP: 1868 (title page), 25 February 1869 (*Allg Bibl Deutsch*), February 1869 (*Allg Bibl*), 5 April 1869 (*Ent Soc Lond*), 15 April 1869 (*Publ Circ*), January–June 1869 (*Bibliot Hist-Nat*)].

*Tom. IV. Scarabaeidae.* Pp. 979–1346 + [8 (Index Generum ad Tom. IV, Corrigenda)]. [DP: 1869 (title page), 27 May 1869 (*Allg Bibl Deutsch*), May 1869 (*Allg Bibl*), January–June 1869 (*Bibliot Hist-Nat*), 1 July 1869 (*Pet Nouv Ent*), 5 July 1869 (*Ent Soc Lond*), 15 July 1869 (*Publ Circ*)].

*Tom. V. Buprestidae, Trixagidae, Monommidae, Eucnemidae, Elateridae, Cebrionidae.* Pp. 1347–1608 + [6 (Index Generum ad Tom. V, Corrig. et addend.)]. [DP: 1869 (title page), 11 November 1869 (*Allg Bibl Deutsch*), 15 November 1869 (*Pet Nouv Ent*; *Ent Soc Lond*), 8 December 1869 (*Publ Circ*), December 1869 (*Allg Bibl*), July–December 1869 (*Verz Bücher Landkarten*; *Bibliot Hist-Nat*)].

*Tom. VI. Rhipidoceridae, Dascillidae, Malacodermidae, Cleridae, Lymexylonidae, Cupesidae, Ptinidae, Bostrychidae, Cioidae.* Pp. 1609–1800 + [5 (Index Generum ad Tom. VI.)]. [DP: 1869 (title page), December 1869 (*Allg Bibl*), 1 January 1870 (*Pet Nouv Ent*), 5 January 1870 (*Allg Bibl Deutsch*), 7 February 1870 (*Ent Soc Lond*)].

*Tom. VII. Tenebrionidae, Nilionidae, Pythidae, Melandryidae, Lagriidae, Pedilidae, Anthicidae, Pyrochroidae, Mordellidae, Rhipidophoridae, Cantharidae, Oedemeridae.* Pp. 1801–2179 + [10 (Addenda, Index Generum ad Tom. VI.I.)]. [DP: 1870 (title page), 15 May 1870 (*Pet Nouv Ent*), 19 May 1870 (*Allg Bibl Deutsch*), 27 May 1870 (*Abeille* 6: 367), 4 July 1870 (*Ent Soc Lond*), July 1870 (*Allg Bibl*)].

*Tom. VIII. Curculionidae.* Pp. 2181–2668 + [11 (Index Generum ad Tom. VIII; Addenda; Corrigenda)]. This tome was issued in two parts: (pp. 2181–2424) 4 March 1872 (*Ent Soc Lond*), April 1872 (*Allg Bibl*), January–June 1872 (*Bibliot Hist-Nat*); (pp. 2425–2668) 6 May 1872 (*Ent Soc Lond*). The title page is dated 1871.

*Tom IX. Scolytidae, Brenthidae, Anthotribidae, Cerambycidae.* Pp. 2669–2988 + [11 (Index Generum ad Tom. IX; Addenda; Corrigenda)]. This tome was issued in two parts: (pp. 2669–??) 2 December 1872 (*Ent Soc Lond*); (pp. ??–2988) 5 May 1873 (*Ent Soc Lond*). The title page is dated 1872. According to Derksen and Scheiding-Göllner (1965: 131), pages 2669–2702 of this volume was also issued, 1872 in Münich, under the title “*Scolytidae. Die Borkenkäfer. Besonders abgedruckt für die obersten Forst-Behörden aus dem IX. Bande des catalogus....*” (*n.v.*).

*Tom. X. Cerambycidae (Lamiini), Bruchidae.* Pp. 2989–3232 + [8 (Index Generum ad Tom. X; Corrigenda)]. [DP: 1873 (title page), 14 March 1874 (*Academy*), 8 April 1874 (*Soc Ent Fr*), 4 May 1874 (*Ent Soc Lond*), May 1874 (*Allg Bibl*), January–June 1874 (*Bibliot Hist-Nat*)].

*Tom. XI. Chrysomelidae (Pars I.).* Pp. 3233–3478 + [4 (Index Generum ad Tom. XI; Corrigenda)]. [DP: 1874 (title page), 26 September 1874 (*Academy*), October 1874 (*Allg Bibl*), 2 November 1874 (*Ent Soc Lond*), July–December 1874 (*Bibliot Hist-Nat*), 10 February 1875 (*Soc Ent Fr*)].

*Tom. XII. Chrysomelidae (Pars II.), Languridae, Erotylidae, Endomychidae, Coccinellidae, Corylophidae, Platypsyllidae. Accedit Index Generum universalis.* Pp. 3479–3822 + [2 (Conspectus Familiarum; Addenda; Corrigendum)] + lxxiii (Index Generum universalis). [DP: 1876 (title page), 18 November 1876 (*Academy*), 6 December 1876 (*Ent Soc Lond*), July–December 1876 (*Bibliot Hist-Nat*), 15 January 1877 (*Pet Nouv Ent*), 14 February 1877 (*Soc Ent Fr*), February 1877 (*Allg Bibl*)].

This catalogue, which inventory all beetles of the world, contains numerous unjustified emendations of genus-group names. The authors shared the various parts of the work between them, as indicated on page xxxiv, and new replacements and unjustified emendations have to be attributed to the proper author. The following gives authorship of all parts.

Gemminger: Gyrinidae (pp. 467–475); Trichopterygidae, Scaphididae (pp. 742–752); Eucnemidae (pp. 1462–1487); Cebrionidae (pp. 1605–1608); tome VI (pp. 1609–1800); tome VII (pp. 1801–2179); tome VIII (pp. 2181–2668); Cerambycidae (pp. 2751–3216).

Harold: Nitidulidae (pp. 802–838); Cucujidae, Cryptophagidae (pp. 868–891); Lathridiidae (pp. 892–905); Dermestidae, Byrrhidae (pp. 912–930); Lucanidae (pp. 941–978); tome IV (pp. 979–1346); Buprestidae (pp. 1347–1456); Elateridae (pp. 1487–1604); tome XI (pp. 3233–3478); Chrysomelidae [pars II] (pp. 3479–3676); Coccinellidae (pp. 3740–3818).

Gemminger and Harold: tome I (pp. 1–424); Dytiscidae (pp. 425–467); Hydrophilidae, Staphylinidae, Pselaphidae, Gnostidae, Paussidae, Scydmaenidae, Silphidae (pp. 475–741); Histeridae, Phalacridae (pp. 753–802); Trogositidae, Colydidae, Rhysodidae (pp. 838–868); Derodontidae (p. 891); Othnidae, Mycetophagidae, Thorictidae (pp. 905–912); Georyssidae, Parnidae, Heteroceridae (pp. 930–941); Trixagidae, Monommidae (pp. 1456–1461); Scolytidae (pp. 2669–2702); Brenthidae, Anthotribidae (pp. 2703–2750); Bruchidae (pp. 3217–3232); Languriidae, Erotylidae, Endomychidae (pp. 3676–3740); Corylophidae, Platypsyllidae (pp. 3818–3821).

#### Gené, Carlo Giuseppe (Turbigo, Lombardy, Italy: 9 December 1800 – 13 July 1847: Turin, Piedmont, Italy). Italian naturalist; assistant lecturer in natural history at the University of Pavia; later, professor of zoology and director of the zoological museum at the University of Turin; made four collecting trips to Sardinia between 1833 and 1838; his insect collection went to the Museo Civico di Storia Naturale in Milan via Carlo Agostino Bassi while his Sardinian Coleoptera are at the zoological museum of the University in Turin. References. Bassi (1848); Filippi (1848); Sismonda (1848); Lessona (1884: 39–56).

**1827**. *Sugli insetti piu nocivi alla agricoltura, agli animali domestici, ai prodotti della rurale economia, ecc. colla indicazione dei mezzi piu’ facili ed efficaci di allontanarli o di distruggerli.* Antonio Fortunato Stella e Figli, Milano. xii + 236 pp. + 3 pls. (8vo) [DP: 1827 (title page)] GB

This work forms volume 7 of the “*Biblioteca Agraria*” of Giuseppe Moretti. The Coleoptera are on pages 21–72. A second edition was published in 1835 [GB] where the Coleoptera are on pages 30–76. The book was also issued in Gené’s “*Dei preguidizi popolari intorno agli animali aggiuntevi le notizie sugli insetti nocivi all’agricoltura, agli animali domestici, ai prodotti della rurale economia colla indicazione dei mezzi più facili ed efficaci di allontanarli e di distruggerli*” (pp. 140–302) published in 1853 in Turin.

#### Geoffroy, Étienne Louis (Paris, France: 2 October 1725 – 12 April 1810: Chartreuse near Soissons, Aisne, France). French physician in Paris, chemist and entomologist; retired in Chartreuse where he became mayor of the village; his collection is at the Muséum National d’Histoire Naturelle in Paris. References. Ratzeburg (1874: 182); Chéreau (1881); Damkaer (2002: 54–55, P); Cambefort (2006: 177).

**1762**. *Histoire abrégée des insectes qui se trouvent aux environs de Paris; dans laquelle ces animaux sont rangés suivant un ordre méthodique. Tome premier.* Durand, Paris. xxviii + 523 pp. + pls 1–10. (4to) [DP: 1762 (title page), January 1763 (*J Oeco*), February 1863 (*Ann Typogr*), March 1763 (*Suite Clef*), April 1763 (*J Sav*), 4 May 1763 (*Affic Lyon*), 8 October 1763 (*Gött Anz*)] CMLE, GB

The entire work consists of two volumes, with 22 plates overall, issued simultaneously. The Coleoptera are in the first one (pp. 48–378). The work was published anonymously. It was reissued in 1764 under the title “*Histoire abrégée des insectes, dans laquelle ces animaux sont rangés suivant un ordre méthodique*” with Geoffroy’s name on the title page [CMLE]. A second edition was issued in 1799 [*q.v.*].

This work was suppressed for nomenclatural purposes by the ICZN (1954b) but eventually removed from the *Official Index of Rejected and Invalid Works in Zoological Nomenclature* (ICZN 1994). The Commission ruled that the following generic names of Coleoptera were made available from this work: *Altica*, *Anthrenus*, *Anthribus*, *Bostrichus*, *Cerocoma*, *Copris*, *Crioceris*, *Cryptocephalus*, *Diaperis*, *Galeruca*, *Gyrinus*, *Hydrophilus*, *Notoxus*, *Omalisus*, *Platycerus*, *Prionus*, *Ptilinus*, *Pyrochroa*, and *Stenocorus*. The generic names *Cistela* and *Rhinomacer* were suppressed for the purposes of the Principle of Priority but not for those of the Principle of Homonymy and *Byrrhus*, *Cucujus*, *Melolontha*, *Peltis*, and *Tritoma* were suppressed for the purposes of both the Principle of Priority and the Principle of Homonymy.

**1799**. *Histoire abrégée des insectes, dans laquelle ces animaux sont rangés suivant un ordre méthodique; nouvelle édition, revue, corrigée, & augmentée d’un supplément considérable. Tome Premier.* Calixte-Volland [&] Rémont, Paris. xxviii + 556 pp. + pls 1–10. (4to) [DP: “An VII” (title page), 19 July–17 August 1799 (*J Lit Fr*), 24 October 1799 (*Gött Anz*)] CNC, GAL, BHL

The entire work consists of two volumes, issued simultaneously. It came with colored or uncolored plates. The Coleoptera are in the first volume (pp. 48–378, 514–543). The book was reissued in 1800 by Delalain, Fils, under the same title [GB].

#### Georgi, Johann Gottlieb (Wachholzhagen near Treptow [currently Trzebiatów], Poland: 31 December 1729 – 8 November 1802: Saint Petersburg, Russia). German chemist, naturalist and geographer; moved to Saint Petersburg in 1769 and took part in the expeditions of Johann Peter Falck (1770) and of Peter Simon Pallas (1772) through Siberia; later, professor of chemistry in Saint Petersburg. References. Anonymous (1806: 10–12); Ratzel (1878).

**1801**. *Geographisch-physikalische und naturhistorische Beschreibung des russischen Reichs zur Uebersicht bisheriger Kenntnisse von demselben. Des dritten Theils siebenter Band. Bisher bekannt gewordene Thierarten.* Friedrich Nicolovius, Königsberg. Pp. 1679–2222. (8vo) [DP: 1801 (title page)] GB, GDZ

The entire work consists of three *Theile* forming seven *Bande*, 1797–1801, and one supplement, 1802 (see next entry). The Coleoptera are treated on pages 1989–2049 in the seventh *Band* of the third *Theil*.

**1802**. *Nachträge für dessen geographisch-physikalische und naturhistorische Beschreibung des russischen Reichs.* Friedrich Nicolovius, Königsberg. x + pp. 11–444. (8vo) [DP: 1802 (title page), 4 December 1802 (Sayre 1959: 12)] GB, MDZ

The Coleoptera are on pages 332–338. Many new species species are named but not described; a list of these species was provided by Hagen (1859: 102).

#### Gerbi, Ranieri (Chiesina Uzzanese near Pistoia, Tuscany, Italy: 16 July 1763 – 20 December 1839: Pisa, Tuscany, Italy). Italian scientist; professor of mathematics and physics at the University of Pisa. References. Pacinotti (1840); Majocchi (1841); Cantù (1844: 235–236); Boccardo (1880: 133–134).

**1794**. *Storia naturale di un nuovo insetto.* Gaetano Cambiagi, Firenze. 269 pp. + 1 pl. (8vo) [DP: 1794 (title page), 26 March 1795 (*Med-Chir Ztg*)] GB, MDZ

The species described is *Curculio antiodontalgicus* (p. 92); although listed as new the species was already made available by the same author the previous year (Gerbi 1793).

A “*seconda edizione, arricchita di aggiunte*” containing 112 pages and one plate was published in Venezia in 1795 [GB].

#### Germar, Ernst Friedrich (Glauchau, Saxony, Germany: 3 November 1786 – 8 July 1853: Halle an der Saale, Saxony-Anhalt, Germany). German mineralogist and entomologist (mainly Coleoptera and Hemiptera); professor of mineralogy, geography and paleontology at the University of Halle for about forty years and director of the mineralogy museum of the university; founded the journal *Magazin der Entomologie*; his collection was dispersed but most of his beetle specimens are at the Zoologisches Museum, Martin-Luther-Universität, in Halle (Curculionidae), the Zoologisches Museum der Humboldt-Universität in Berlin and the Deutsches Entomologisches Institut in Müncheberg. References. Schaum (1853; 1854); Ratzeburg (1874: 184–185); Weidner (1983: 281–282); Frank (2008: 1611–1612).

[**1814]–1847**. *Augusti Ahrensii Fauna Insectorum Europae.* Car. Aug. Kümmel, Halae [= Halle]. (12mo) CMLE, CNC (mf)

The entire work was published in 24 *fasciculi*, each containing chiefly 25 colored plates and one or two unnumbered pages of explanation for each plate. The first *fasciculus* was authored by A. Ahrens [*q.v.*] and the third one by Germar and F. Kaulfuss (see next entry). The remaining ones, including the second one^32^, are by Germar. Starting with fasciculus sextus the title is “*Fauna Insectorum Europae*.”

There are no dates on the title pages; those found during this study are: **fasc. II**: 1814 (Hagen 1862: 5; Rautenberg 1957: 613)^33^, April 1815 (*Allg Lit Ztg*), 1816 (Heyden 1906: 284); **fasc. IV**: 1821 (Heyden 1906: 284; Horn and Schenkling 1928b: 412), 30 May 1822 (Evenhuis 1997a: 294); **fasc. V**: January–June 1822 (*Verz Bücher Landkarten*), November 1822 (*Allg Lit Ztg*); **fasc. sextus [6**]: 1822 (Heyden 1906: 284; Horn and Schenkling 1928b: 412), January–June 1822 (*Verz Bücher Landkarten*); **fasc. septimus [7**]: 1822 (Heyden 1906: 284; Horn and Schenkling 1928b: 412), January–June 1822 (*Verz Bücher Landkarten*); **fasc. octavus [8**]: 1823 (Evenhuis 1997a: 294), 1824 (Heyden 1906: 284; Horn and Schenkling 1928b: 412), January–June 1824 (*Verz Bücher Landkarten*); **fasc. nonus [9**]: October 1824 (*Allg Lit Ztg*), July–December 1824 (*Verz Bücher Landkarten*); **fasc. decimus [10**]: October 1824 (*Allg Lit Ztg*), July–December 1824 (*Verz Bücher Landkarten*); **fasc. undecimus [11**]: January–June 1825 (*Verz Bücher Landkarten*), August 1825 (*Allg Lit Ztg*); **fasc. duodecimus [12**]: December 1825 (*Allg Lit Ztg*), 14 June 1826 (*Bibl Deutsch*), January–June 1826 (*Verz Bücher Landkarten*); **fasc. tertiusdecimus [13**]: 1827 (Heyden 1906: 284; Horn and Schenkling 1928b: 412; Evenhuis 1997a: 294), March 1828 (*Intell Jena Lit Ztg*), January–June 1828 (*Verz Bücher Landkarten*); **fasc. quartusdecimus [14**]: 1831 (Heyden 1906: 284; Horn and Schenkling 1928b: 412; Evenhuis 1997a: 294), January–April 1832 (*Foreign Quart Rev*), January–June 1832 (*Verz Bücher Landkarten*); **fasc. quintusdecimus [15**]: 1831 (Heyden 1906: 284; Horn and Schenkling 1928b: 412), 1832 (Evenhuis 1997a: 294), October–December 1832 (*Foreign Quart Rev*), July–December 1832 (*Verz Bücher Landkarten*); **fasc. sextusdecimus [16**]: 1834 (Heyden 1906: 284; Gaedike and Smetana 1978: 397; Fery 2007: 257), September 1834 (*Nouv Rev Germ*), July–December 1834 (*Verz Bücher Landkarten*); **fasc. decimus septimus [17**]: 1834 (Heyden 1906: 284; Gaedike and Smetana 1978: 397; Fery 2007: 257), September 1834 (*Nouv Rev Germ*), July–December 1834 (*Verz Bücher Landkarten*); **fasc. decimus octavus [18**]: 30 September 1836 (*Allg Bibl Deutsch*), September 1836 (*Intell Allg Lit Ztg*), 5 October 1836 (*Lit Ztg*), July–December 1836 (*Verz Bücher Landkarten*); **fasc. undevicesimus [19**]^34^: 1836 (Heyden 1906: 284; Horn and Schenkling 1928b: 412), 8 February 1837 (*Lit Ztg*), 10 February 1837 (*Allg Bibl Deutsch*), January–March 1837 (*Foreign Quart Rev*), April 1837 (*Bull Litt Étr*), May 1837 (Evenhuis 1997a: 294), January–June 1837 (*Verz Bücher Landkarten*), 23 August 1837 (*Allg Forst Jagd Ztg*); **fasc. vicesimus [20**]: 24 May 1838 (*Frän Merkur*), 20 June 1838 (*Lit Ztg*), 22 June 1838 (*Allg Bibl Deutsch*), January–June 1838 (*Verz Bücher Landkarten*), July–September 1838 (*Foreign Quart Rev*); **fasc. vicesimus primus [21**]: 10 April 1839 (*Lit Ztg*), 12 April 1839 (*Allg Bibl Deutsch*), January–June 1839 (*Verz Bücher Landkarten*); **fasc. vicesimus secundus [22**]: 4 June 1842 (*Pharm Centr* 13: 416), 1 July 1842 (*Neue Jena Lit Ztg*), 14 September 1842 (*Lit Ztg*), October–December 1842 (*Foreign Quart Rev*), July–December 1842 (*Verz Bücher Landkarten*); **fasc. vicesimus tertius [23**]: 1844 (Engelmann 1846: 483), 24 April 1845 (*Allg Bibl Deutsch*), 10 May 1845 (*Lit Ztg*), 13 May 1845 (*Ent Ver Stettin*), 16 May 1845 (*Leip Reper*), January–June 1845 (*Verz Bücher Landkarten*); **fasc. vicesimus quartus [24**]: 1 December 1847 (*Lit Ztg*), 23 December 1847 (*Allg Bibl Deutsch*), 14 January 1848 (*Leip Reper*), January 1848 (*Intell Allg Lit Ztg*).

Rautenberg (1957: 615–625) gave a list of all insects treated in the 24 fascicles.

[**1817**]. [Germar, E.F. and Kaulfuss, F.] *Augusti Ahrensii fauna insectorum Europae. Fasciculus III.* Car. Aug. Kümmel, Halae [= Halle]. 25 pls + [text]. (12mo) [DP: n.d. (title page), April 1817 (*Allg Lit Ztg*; *Leip Lit Ztg*), May 1817 (*Intell Jena Lit Ztg*)] CMLE

The date of 27 September 1816, taken from *Isis* (Oken’s), mentioned by Evenhuis (1997a: 294) for the publication of this fascicle could not be confirmed in this study. Hagen (1862: 273), Heyden (1906: 284) and Rautenberg (1957: 613) dated the book 1817.

**1817**. *Reise nach Dalmatien und in das Gebiet von Ragusa. Mit 9 illum. Kupfern und 2 Charten.* F.A. Brockhaus, Leipzig und Altenburg. xii + 323 pp. + 11 pls. (8vo) [DP: 1817 (title page; *Vorrede* dated 23 May 1817), August 1817 (*Intell Jena Lit Ztg*)] CMLE (mf), GB

Nonveiller (1984) gave an account of Germar’s travels to Dalmatia and a list of species included in this work.

**1823**. *Coleopterorum species novae aut minus cognitae, descriptionibus illustratae. Cum Tab. aen. II.* J.C. Hendelii et filii, Halae [= Halle]. xxiv + 624 pp. + 2 pls. (8vo) [DP: 1824 (title page; preface dated 10 September 1823), October 1823 (*Intell Jena Lit Ztg*; *Allg Lit Ztg*), November 1823 (*Isis*, Heft XI: [2]), July–December 1823 (*Verz Bücher Landkarten*)] CNC, GB, MDZ

The second page is entitled “*Insectorum species novae aut minus cognitae, descriptionibus illustratae. Volumen primum. Coleoptera. Cum Tab. aen. II.*” Despite that the title page is dated 1824, this book was published by October 1823. Ersch (1828: 1166) dated the book 1823.

This work contains the description of 891 species and several new genus-group taxa (Dejean 1824).

#### Gerstaecker, Carl Eduard Adolph (Berlin, Germany: 30 August 1828 – 20 June 1895: Greifswald, Mecklenburg-West Pomerania, Germany). German professor and entomologist; curator at the entomological department of the Museum für Naturkunde (Zoologisches Museum der Humboldt-Universität) and professor of zoology at the University in Berlin; later, professor of zoology at the University of Greifswald; parts of his collection are at the Ernst-Moritz-Arndt-Universität in Greifswald and the Zoologisches Museum der Humboldt-Universität in Berlin. References. Meldola (1895: lxxi–lxxii); Papavero (1973: 322–323); Uschmann (1964).

**1855**. *Rhipiphoridum coleopterorum familiae dispositio systematica. Dissertatio inauguralis. Zoologica quam consensu et auctoritate amplissimi philosophorum ordinis in alma litterarum universitate Friderica Guilelma pro summis in philosophia honoribus rite capessendis die IV. m. Julii a MDCCCLV. Hora XII. Publice defendet auctor A. Gerstaecker, med. Dr. Berolinensis.* Schlesinger, Berolini [= Berlin]. 36 pp. (4to) [DP: 4 July 1855 (title page)] GB

This work was also issued the same year by the publisher Friderici Nicolai under the title “*Rhipiphoridum coleopterorum familiae dispositio systematica. Accedit tabula aeri incisa.*” [MCZ, CNC, GB, BHL, MDZ] with the addition of a plate. It was noticed on 2 August 1855 (*Allg Bibl Deutsch*).

**1858**. *Entomographien. Abhandlungen im Bereich der Gliederthiere, mit besonderer Benutzung der koenigl. entomologischen Sammlung zu Berlin. Erster Band. Monographie der Familie Endomychidae.* Wilhelm Engelmann, Leipzig. xiv + 433 pp. + 3 pls. (8vo) [DP: 1858 (title page; *Vorwort* dated June 1858), 7 October 1858 (*Allg Bibl Deutsch*), 15 October 1858 (*Intell Serapeum*), October 1858 (*Allg Bibl*), 16 November 1858 (*Acad Nat Sci Phila*), July–December 1858 (*Bibliot Hist-Nat*)] MCZ, GB, BHL

The book has a second title page which reads “*Monographie der Endomychiden, einer Familie der Coleopteren. Mit drei Kupfertafeln.*”

**1862**. Fortsetzung. Pp. 268–348 + pls 15–20 *in*: *Naturwissenschaftliche Reise nach Mossambique auf Befehl seiner Majestät des Königs Friedrich Wilhelm IV in den Jahren 1842 bis 1848 ausgeführt von Wilhelm C.H. Peters. Zoologie. V. Insecten und Myriopoden. Bearbeitet in Verbindung mit Klug, Loew, Schaum, Hagen, Gerstaecker, Hopffer. Mit fünf und dreissig Kupfertafeln.* Georg Reimer, Berlin. xxi + 566 pp. [+ 35 associated pls]. (4to–text; Folio–plates) [DP: 1862 (title page; *Vorwort* dated December 1861), 31 July 1862 (*Allg Bibl Deutsch*; *Intell Serapeum*), July 1862 (Evenhuis 1997b: 612), August 1862 (*Allg Bibl*), 1 September 1862 (*Ent Soc Lond*), September 1862 (*Rev Germ*), July–December 1862 (*Bibliot Hist-Nat*; *Verz Bücher Landkarten*)] CNC, GB, BHL

The natural history report of Wilhelm Peters’ exploration in Mozambique was published in five volumes of text, 1852–1882. The Coleoptera are in the fifth volume on pages 145–348. Johann Christoph Friedrich Klug [*q.v.*] authored pages 145–267.

**1863**. Arthropoda. Pp. 1–421 *in*: *Handbuch der Zoologie. Zweiter Band. Arthropoden bearbeitet von A. Gerstaecker. Raderthiere, Würmer, Echinodermen, Coelenteraten und Protozoen bearbeitet von J. Victor Carus.* Wilhelm Engelmann, Leipzig. viii + 642 pp. (8vo) [DP: 1863 (title page; *Vorwort* dated February 1863), April–May 1863 (*Allg Bibl*), 16 July 1863 (*Publ Circ*), January–July 1863 (*Bibliot Hist-Nat*)] GB, MDZ, BHL

The entire work consists of two *Bande*. The Coleoptera are on pages 80–185 of the second *Band* issued in 1863. The first *Band*, authored by J. Victor Carus alone, was issued in two parts, 1868 and 1875.

**1873**. *Baron Carl Claus von der Decken’s Reisen in Ost-Afrika. Dritter Band: Wissenschaftliche Ergebnisse. Zweite Abtheilung: Gliederthiere (Insekten, Arachniden, Myriopoden und Isopoden). Mit 18 colorirten Kupfertafeln.* C.F. Winter, Leipzig und Heidelberg. xvi + 542 + [1 (Verbesserungen)] pp. + 18 pls. (4to) [DP: 1873 (title page; *Vorwort* dated 1 November 1872), January–June 1873 (*Bibliot Geo-Stat*), August 1873 (*Allg Bibl*)] MANL, GB, BHL

The book has a second title page which reads “*Baron Carl Claus von der Decken’s Reisen in Ost-Afrika in den Jahren 1859–1865. Herausgegeben im Auftrage der Mutter des Reisenden. Fürstin Adelheid von Pless. Wissenschaftlicher Theil. Dritter Band. Zweite Abtheilung*.” This book was also issued, with a different *Vorwort*, by the same publisher, 1873, under the title “*Die Gliederthiere-Fauna der Sansibar-Gebietes. Nach dem von Dr. O. Kersten während der v. d. Decken’schen Ost-Afrikanischen Expedition im Jahre 1862 und von C. Cooke auf der Insel Sansibar im Jahre 1864 gesammelten Material. Mit 18 colorirten Kupfertafeln*” [GB].

The entire trip report was issued in four *Bande* forming six volumes, 1869–1879. The Coleoptera are on pages 55–312 of the third *Band*.

#### Geyer, Karl [Carl] (Augsburg, Bavaria, Germany: 4 March 1802 – 1 June 1889: Augsburg, Germany). German artist (illustrater and engraver) and entomologist; assistant to lepidopterist Jacob Hübner; published posthumously supplements to some of Hübner’s works. Reference. Pfeuffer (2011).

**1823**. *Faunae insectorum Germanicae initia. Deutschlands Insecten. Heft 110*. Felsecker, Nürnberg. [24] pp. + 24 pls. (Oblong 16mo) [DP: 1823 (Sherborn 1923: 567), September 1823 (*J Lit Fr*), July–December 1823 (*Verz Bücher Landkarten*)] CNC

The entire series consists of 190 *Hefte*, the first 109 were authored by Panzer [*q.v.*] and *Hefte* 111–190 by G.A.W. Herrich-Schäffer [*q.v.*].

I have not seen the title page of this part. According to Hagen (1863: 27), this *Heft* was issued by the illustrator Geyer. Five new species of Coleoptera are described in this work, *Aphodius affinis*, *Aphodius obliteratus*, *Byrrhus signatus*, *Brentus lineatus* and *Osmia chrysomelina*, and none of them are attributed to Geyer in Sherborn’s *Index Animalium*.

#### Giebel, Christoph Gottfried Andreas (Quedlinburg, Saxony, Germany: 13 September 1820 – 14 November 1881: Halle an der Saale, Saxony-Anhalt, Germany). German naturalist and paleontologist; professor of zoology and director of the museum at the University of Halle; part of his collection is in that museum. References. Anonymous (1881, P); Fritsch (1882); Adler (2012: 115–116, P).

**1852**. *Deutschlands Petrefacten. Ein systematisches Verzeichniss aller in Deutschland und den angrenzenden Ländern vorkommenden Petrefacten nebst Angabe der Synonymen und Fundorte.* Ambrosius Abel, Leipzig. xiii + 706 pp. (8vo) GB

This work was issued in two parts: (pp. 1–320) 28 October 1852 (*Allg Bibl Deutsch*), 20 November 1852 (*Lit Centr Deutsch*), 30 November 1852 (*Intell Serapeum*); (pp. 321–706 + xiii) 6 January 1853 (*Allg Bibl Deutsch*), 15 January 1853 (*Säch Berg-Ztg*; *Intell Serapeum*), 10 February 1853 (*Monthly Lit Adv*). The work contains a list of fossil Coleoptera (pp. 647–656).

**1856**. *Fauna der Vorwelt mit steter Berücksichtigung der lebenden Thiere. Monographisch dargestellt. Zweiter Band. Gliederthiere. Erste Abtheilung: Insecten und Spinnen.* F.A. Brockhaus, Leipzig. xviii + 511 pp. (8vo) [DP: 1856 (title page; *Vorrede* dated February 1856), 25 September 1856 (*Allg Bibl Deutsch*), 29 September 1856 (*Naturwiss Ver Sach Thür*), July–September 1856 (*Cat Muquardt*), 25 October 1856 (*Lit Centr Deutsch*), October 1856 (*Allg Bibl*), 30 November 1856 (*Intell Serapeum*), July–December 1856 (*Bibliot Hist-Nat*)] GB

Another title page reads “*Die Insecten und Spinnen der Vorwelt mit steter Berücksichtigung der lebenden Insecten und Spinnen*.” The entire work was published in three volumes, 1847–1856. The Coleoptera are on pages 26–150 of the second volume.

#### Gillmeister, C.J.F. (Ludwigslust, Mecklenburg-West Pomerania, Germany: 1810 – December 1846). German veterinarian; his ptiliid collection is at the Zoologisches Museum, Universität Hamburg. Reference. Schrader and Hering (1863: 157–158).

**1845**. *Deutschlands Fauna in Abbildungen nach der Natur mit Beschreibungen von Jacob Sturm. V. Abtheilung. Die Insecten. Siebenzehntes Bändchen. Käfer. Mit 7 illuminirten und 2 schwarzen Kupfertafeln.* Nürnberg. xvi + 98 pp. + pls 320–328. (8vo) [DP: 1845 (title page; *Vorwort* dated August 1845), 6 November 1845 (*Ent Ver Stettin*), 19 December 1845 (*Leip Reper*)] GB

The half-title page reads “*Trichopterygia, Beschreibung und Abbildung der haarflügeligen Käfer von Dr. C.J.F. Gillmeister in Frankfurt a. M.*”

This volume is the 17th part of “*Deutschlands Fauna*,” a series started by Jacob Sturm [*q.v.*] and continued by his son, published between 1805 and 1857.

#### Giorna, Giuseppe (? – 1799). Italian naturalist and politician; joined the “Legione Lombarda” of the Cisalpine Republic, a sister republic of France in northern Italy from 1797–1802. Reference. Vassalli-Eandi (1811: cxxxv).

**1791**. *Calendario entomologico ossia osservazioni sulla stagioni proprie agl’ insetti nel clima piemontese, e particolarmente ne’ contorni di Torino.* Nella Stamperia Reale, Torino. 146 pp. (8vo) [DP: 1791 (title page)] GB

Many new species of insects are described in this book, including several Coleoptera. The work was reissued in 1874 in *Annali della R. Accademia d’Agricoltura di Torino* 16 [1873]: 87–161. Millin (1795 : 314–315) noted that this work was issued previously in journals, first in part (two semesters) in 1789 in *Giornale scientifico letterario* [*e delle arti di una societa filosofica di Torino*] (*n.v.*) and then in his totality in *Biblioteca Oltremontana* [*e Piemontese*] (*n.v.*).

This work is often credited to Michel Spirito Giorna [1741–1809] (e.g., Hagen 1862: 281; Horn and Schenkling 1928b: 423) but was written by his son Giuseppe (also called Giorna il figlio), as listed on the title page (see also Conci 1975: 924).

#### Girard, Maurice Jean Auguste (Givet, Ardennes, France: 13 September 1822 – 8 September 1886: Lion-sur-Mer, Calvados, France). French educator and entomologist; professor of physics, chemistry and natural history in Dijon and Paris. References. Poujade (1887); Renan (1887).

**1873**. *Les insectes. Traité élémentaire d’entomologie comprenant l’histoire des espèces utiles et de leurs produits, des espèces nuisibles et des moyens de les détruire, l’étude des métamorphoses et des moeurs, les procédés de chasse et de conservation. Avec planches coloriées. Introduction – Coléoptères.* J.-B. Baillière et Fils, Paris. viii + 840 pp. [+ 60 associated pls]. (8vo) [DP: 1873 (title page; *avertissement* dated December 1872), 12 March 1873 (*Soc Agr Fr*), 15 March 1873 (*Pet Nouv Ent*), 7 April 1873 (*Ent Soc Lond*), 4 April 1874 (*Bibl Fr*), May 1874 (*Polybiblion*)] CMLE, GB, BHL

The entire series consists of three volumes, 1873–1885. The second volume, 1879, pertains to “Orthoptères, Nevroptères, Hyménoptères porte-aiguillon;” the third one, 1885, to “Hymenoptères térébrants, Lépidoptères, Hémiptères, Diptères et ordres satellites.” The atlas came with colored or uncolored plates; the 60 plates associated with the first volume came from Guérin-Méneville’s *Iconographie du règne animal de G. Cuvier* as ascertained by Guérin-Méneville (1873: 412).

#### Girod-Chantrans [Girod de Chantrans], Justin (Besançon, Doubs, France: 26 September 1750 – 1 April 1841: Besançon, France). French serviceman, politician and naturalist, best known for his work on algae. References. Feller (1848: 130); Girod (1978).

**1810**. *Essai sur la géographie physique, le climat et l’histoire naturelle du département du Doubs, ouvrage approuvé par la classe des sciences physiques et mathématiques de l’Institut, et dans lequel on trouve une cryptogamie enrichie de la description d’un grand nombre d’espèces inédites. Tome premier.* Courcier, Paris. xxvi + 303 pp. (8vo) [DP: 1810 (title page), 8 January 1810 (*Acad Sci Fr*), 14 February 1810 (*Soc Encou Ind Nat*), 12 March 1810 (*J Typogr Bibl*)] GB

The entire work consists of two volumes, 1810. The Coleoptera are on pages 130–181 in the first volume.

#### Gistel [Gistl], Johannes von Nepomuk Franz Xavier (Munich, Bavaria, Germany: 11 August 1809 – 1873: Munich, Germany). German naturalist; professor of natural history and geography; librarian secretary and curator at the Museum of Natural History in Regensburg; well known for the countless emendations and new replacement names he proposed; wrote some of his papers under the name “G. Tilesius;” his collection was acquired by the Zoologische Staatssammlung München in 1877 but most of his specimens were lost through neglect or mislaid for lack of labelling and fire during WWII. References. Scherer (1992: 65–66); Pont (1995: 145); Adler (2012: 92–93, P).

**1829**. *Enumeratio coleopterorum agri Monacensis.* Monachii [= Munich]. vi + 7–38 pp. (8vo) [DP: 1829 (title page; *lectori* dated 16 March 1829), 25 August 1829 (*Beil Allg Ztg*), October 1829 (*Isis*, Heft 10: 1058)] MCZ, GB

This publication was also issued under the same title, 1831, in Augustae Vindelicorum (= Augsburg, Germany) [CMLE].

**1834**. *Die Insecten-Doubletten aus der Sammlung des Herrn Grafen Rudolph von Jenison Walworth zu Regensburg, welche sowohl im Kauf als im Tausche abgegeben werden. Nro. I. Käfer.* George Jaquet, München. 35 + [1] pp. (8vo) [DP: 1834 (title page)] CNC, GB

This is a list of duplicate beetles in the collection of Count Rudolph von Jenison Walworth of Regensburg available for purchase or exchange. Several new generic names are proposed and some of them are made available by the inclusion of one or more available species.

**1836**. *Ueber eine neue Familie, Sippe und Gattung aus der Ordnung der Käfer. Mit einer Abbildung.* Joseph A. Finsterlin, München. 8 pp. + 1 pl. (8vo) [DP: 1836 (title page), 7 October 1836 (*Allg Bibl Deutsch*), 12 October 1836 (*Lit Ztg*), July–December 1836 (*Verz Bücher Landkarten*)] GB

This booklet was reissued in 1837, under the title “*Mesoclastus paradoxus, eine neue Familie, Sippe und Gattung aus der Ordnung der Käfer*,” in *Faunus* (Neue Folge) 1: 54–59.

**1837–[1840**]. *Systema insectorum, secundum classes, ordines, genera, species, cum characteribus, synonymis, annotationibus, locis et iconibus. Tomus Imus. Coleoptera.* Fleischmann, Monachii [= Munich] [fasc. 1] / C.A. Jenni, filium, Bernae [fasc. 2]. (8vo) CMLE (mf), USNM

*Fasciculus 1mus. Mantichora–Dromica. Cum tabula aeneo incisa.* xvi + 64 pp. + 1 pl. [DP: 1837 (title page; *praefatio* dated 15 October 1836), March 1837 (*Isis*, Heft III: [2]), 13 April 1837 (*Lit Ztg*), 12 May 1837 (*Allg Bibl Deutsch*), June 1837 (*Bull Litt Étr*), January–June 1837 (*Verz Bücher Landkarten*), 5 August 1837 (*Acad R Sci Brux*), 20 December 1837 (*Soc Ent Fr*), 6 August 1838 (*Ent Soc Lond*)].

*Fasciculus IIdus. Cicindela–Cymindis.* Pp. 65–132. [DP: 1839 (title page), 15 September 1840 (*Intell Serapeum*), 16 September 1840 (*Lit Ztg*), 18 September 1840 (*Allg Bibl Deutsch*), September 1840 (*Ent Ver Stettin*)].

Despite the title page being dated 1839, the second fascicle was probably issued only in 1840. A title page, which reads “*Systema insectorum. Tomus I. Coleoptera.*” and dated 1837, was issued for the entire book.

**1848**. *Naturgeschichte des Thierreichs. Für höhere Schulen. Mit einem Atlas von 32 Tafeln (darstellend 617 illuminirte Figuren) und mehreren dem Texte eingedruckten Xylographien.* Hoffmann, Stuttgart. xvi + 216 + [4] pp. + 32 pls. (Folio) [DP: 1848 (title page; *Bevorwortung* dated “15 Ostermonat 1847”), June 1848 (*Isis*, Heft VI: [2]), 15 August 1848 (*Intell Serapeum*), 17 August 1848 (*Allg Bibl Deutsch*; *Lit Ztg*), 17 November 1848 (*Leip Reper*), 11 December 1848 (*Allg Ztg*)] CNC

A list of replacement names for genera is found in the introductory pages and a few additional ones in the text. A “*Zweite Auflage*” was published in 1851 by Scheitlin & Krais, in Stuttgart [BHL], which is merely a reprinting of the original edition with only a change in the title page (Sherborn 1922a: lix).

**1848**. *Neuestes und vollständigstes Handbuch der Naturgeschichte für Lehrer und Lernende, für Schule und Haus. Abtheilung I. Thierreich.* R. Hoffmann, Stuttgart. 640 pp. + 32 pls. (8vo) (*n.v.*).

This volume was issued in four *Lieferungen* as follows: **1**: (pp. 1–160 + 8 pls) 21 March 1848 (*Allg Anz*), 22 March 1848 (*Lit Ztg*), 30 March 1848 (*Allg Bibl Deutsch*); **2–3**: (pp. 161–480 + 16 pls) 20 July 1848 (*Allg Bibl Deutsch*), 29 July 1848 (*Deutsch Ztg*); **4**: (pp. 481–640 + 8 pls) July 1848 (*Isis*, Heft VII: [2]), 16 November 1848 (*Allg Bibl Deutsch*). The second *Abtheilung* (pp. 641–1037) was authored by Traugott Bromme and deals with plants and minerals (Evenhuis 1997a: 304). The entire work was reissued, 1850, under the title “*Handbuch der Naturgeschichte aller drei Reiche, für Lehrer und Lernende, für Schule und Haus. Von Dr. Joh. Gistel und Tr. Bromme. (Thierreich von Dr. Gistel, Pflanzen- und Steinreich von Tr. Bromme.) Mit 48 colorirten Tafeln und 42 Holzschnitten*” by the same publisher [McG, GB].

**1856**. *Die Mysterien der europäischen Insectenwelt. Ein geheimer Schlüssel für Sammler aller Insecten-Ordnungen und Stände, behufs des Fangs, des Aufenthalts-Orts, der Wohnung, Tag- und Jahreszeit u.s.w., oder autoptische Darstellung des Insectenstaats in seinem Zusammenhange zum Bestehen des Naturhaushaltes überhaupt und insbesondere in seinem Einflusse auf die phanerogamische und cryptogamische Pflanzenbevölkerung Europa’s. Zum ersten Male nach fünfundzwanzigjährigen eigenen Erfahrungen zusammengestellt und herausgegeben.* Tobias Dannheimer, Kempten. xii + 530 + [2 (Druckfehler)] pp. (12mo) [DP: 1856 (title page), 18 February 1856 (Evenhuis 1997a: 305), 3 April 1856 (*Allg Bibl Deutsch*), 21 April 1856 (*Ver Natur Presburg*), April 1856 (*Allg Bibl*), 31 May 1856 (*Intell Serapeum*), April–June 1856 (*Cat Muquardt*), January–June 1856 (*Bibliot Hist-Nat*), 1 July 1856 (*Acad Nat Sci Phila*)] MCZ, GB, BHL

**1856**. *Pleroma zu den Mysterien der europäischen Insektenwelt. Mit einem systematischen Verzeichniss der Schmetterlinge und Käfer Europa’s. Durch die neuesten Entdeckungen bis 1856 bereichert.* J. Schorner, Straubing. 250 pp. (8vo) [DP: 1856 (title page), 23 April 1857 (*Allg Bibl Deutsch*), January–June 1857 (*Bibliot Hist-Nat*), July 1857 (*Lit Deutsch Natur Ztg*)] MCZ, GB, MDZ

This work was reissued in Gistel’s first tome of *Vacuna oder die Geheimnisse aus der organischen und leblosen Welt* [*q.v.*], issued in 1857, on pages 207–453.

**1857**. *Vacuna oder die Geheimnisse aus der organischen und leblosen Welt. Ungedruckte Originalien-Sammlung von grösstentheils noch lebenden und verstorbenen Gelehrten aus dem Gebiete sämmtlicher Naturwissenschaften, der Medizin, Literaturgeschichte, des Forst- und Jagdwesens, der Oekonomie, Geschichte, Biographie, und der freien schönen Künste.* Schorner, Straubing. (4to) CMLE (mf), GB (vol. 1)

*Erster Band.* [1] + 453 pp. [DP: 1857 (title page), 20 May 1857 (*Chem Centr*), January–June 1857 (*Bibliot Hist-Nat*), October 1857 (Evenhuis 1997a: 305), 11 February 1858 (*Allg Bibl Deutsch*)].

*Zweiter Band.* 1031 pp. [DP: 1857 (title page), October 1857 (Evenhuis 1997a: 305), July–December 1857 (*Bibliot Hist-Nat*), 11 February 1858 (*Allg Bibl Deutsch*)].

Chapter 11 (pages 513–606) of this work was also published separately, the same year, under the title “*Achthundert und zwanzig neue oder unbeschriebene wirbellose Thiere*” [GB] by the same publisher (see Baker 1997b: 336, Fig. 4). Strand (1916) discussed this publication and listed in systematic order all insect species included, sometimes with comments.

#### Glaser, Ludwig (Grünberg, Hesse, Germany: 9 February 1818 – 20 January 1898: Mannheim, Baden-Württemberg, Germany). German educator and naturalist; taught at secondary schools in Biedenkopf, Friedberg, Worms and Bingen until he retired in 1879. Reference. Glaser (1898).

**1857**. *Naturgeschichte der Insecten mit besonderer Berücksichtigung der bei uns einheimischen für die gebildete Jugend höherer Lehranstalten, sowie überhaupt für Naturfreunde übersichtlich dargestellt und mit einer Insectenflora versehen.* Theodor Fischer, Cassel. [2] + iii + 321 pp. (8vo) [DP: 1857 (title page; *Vorwort* dated October 1857), 12 November 1857 (*Allg Bibl Deutsch*), July–December 1857 (*Bibliot Hist-Nat*)] GB

The Coleoptera are on pages 10–63. A second edition (*n.v.*), with the same number of pages, was published in 1863, not 1864 as it seems to be indicated on the title page, in Frankfurt a.M. by Völcker; it was recorded on 10 December 1863 (*Allg Bibl Deutsch*).

#### Gleditsch, Johann Gottlieb (Leipzig, Saxony, Germany: 5 February 1714 – 5 October 1786: Berlin, Germany). German botanist; professor of botany and silviculture at the Collegium Medico-Chirurgicum in Berlin and director of the local botanical garden. References. Anonymous (1789); Ratzeburg (1874: 187–190); Hess (1885: 106–108).

**1767**. *Vermischte physicalisch-botanisch-oeconomische Abhandlungen. Dritter Theil. Mit einem Kupfer und Register.* Joh. Jacob Curt, Halle. [24] + 397 + [11 (Hauptregister)] pp. + 1 pl. (8vo) [DP: 1767 (title page), 28 May 1768 (*Gött Anz*)] GB, EUR

The entire work consists of three volumes, 1765–1767. Chapter 5 (pp. 200–227) of the third volume is entitled “*Abhandlung von dem Begräbnisse des Maulwurfes und anderer kleinen Thiere*” and contains the descriptions of a few Coleoptera species, some of which are illustrated on the sole plate.

#### Gmelin, Johann Friedrich (Tübingen, Baden-Württemberg, Germany: 8 August 1748 – 1 November 1804: Göttingen, Lower Saxony, Germany). German chemist, naturalist and physician; made a three-year journey through Holland, England and Austria; professor of medicine at the University of Tübingen; later, professor of chemistry and medical sciences at the University of Göttingen (1773–1804). References. Anonymous (1841b); Ratzeburg (1874: 190–191); Wagenitz (1988: 65); Adler (2007: 32–33, P).

**1790**. *Caroli a Linné Systema Naturae per regna tria naturae, secundum classes, ordines, genera, species, cum characteribus, differentiis, synonymis, locis. Editio decima tertia, aucta, reformata. Tom. I. Pars IV.* Georg Emanuel Beer, Lipsiae [= Leipzig]. Pp. 1517–2224. (8vo) [DP: n.d. (title page), 21 May 1790 (Hopkinson 1908: 1036), 31 May 1790 (*Gött Anz*), 26 June 1790 (*Intell Allg Lit Ztg*), November 1790 (*J Méd*)] CNC, BHL

The entire work was published in ten parts forming three volumes, 1788–1793. The Coleoptera are in volume 1, part 4 (pp. 1526–2040). Only the first part of volume 1 has a title page. The other ones have only an undated half-title page which reads “*Caroli a Linné Systema Naturae.*” Other contemporary editions of the entire work were published in Lugduni [= Lyon] by J.B. Delamollière, 1788–1796 (see Kabat and Petit 1988), and Conimbricae (= Coimbra), 1793–1794 (see Evenhuis 1997a: 311).

#### Göbel, Ferdinand (Großenehrich, Thuringia, Germany: 8 April 1805 – 21 January 1876: Arnstadt, Thuringia, Germany). German high school teacher in Sondershausen; later, director of a secondary school in Arnstadt. References. Brauns and Theobald (1837: 557); Kössler (2008a).

**1854**. Grundlage zur Kenntniss der um Sondershausen vorkommenden Käfer. Ein Beitrag zur Naturgeschichte Nordthüringens. Pp. 3–25 *in*: *Zu der öffentlichen Prüfung des Fürstlich Schwarzburgischen Gymnasiums zu Sondershausen, welche den 3. und 4. April 1854 Statt finden wird, ladet ehrerbietigst ein der Director Dr. W. Kiefer.* F.A. Eupel, Sondershausen. 35 pp. (4to) [DP: 4 April 1854 (title page)] GB

This work contains a list of the Coleoptera from Sondershausen and surrounding areas in Thuringia, Germany.

#### Goeze, Johann August Ephraim (Aschersleben, Saxony-Anhalt, Germany: 28 May 1731 – 27 June 1793: Quedlinburg, Saxony-Anhalt, Germany). German zoologist, priest and deacon of Quedlinburg; studied theology at the University of Halle; worked mainly on aquatic invertebrates, particularly insects and worms. References. Schlichtegroll (1794: 182–226); Carus (1879); Müllerott (1964); Damkaer (2002: 99–100).

**1777–1778**. *Entomologische Beyträge zu des Ritter Linné zwölften Ausgabe des Natursystems.* Weidmann, Leipzig. (8vo) CNC, BHL

*Erster Theil.* xvi + 736 pp. [DP: 1777 (title page), 13 April 1777 (Evenhuis 1997a: 322), 4 August 1777 (*Jena Ztg*), August 1777 (*Allg Verz Bücher*), 17 November 1777 (*Gött Anz*), 9 December 1777 (*Erlang Anmer Nachr*)].

*Zweyter Theil.* lxxii + 352 pp. [DP: 1778 (title page), November 1778 (*Allg Verz Bücher*), 14 December 1778 (*Jena Ztg*)].

The entire work consists of three *Theile*, 1777–1780, the third one having been issued in four parts. The Coleoptera are treated in the first *Theil*. The second *Theil* deals with Hemiptera but the *Vorrede* (pp. iv–lxxi) contains additions to the first volume.

Sherborn (1902: xxvi) indicated that this work is not consistently binominal.

**1781–1783**. *Des Herrn Baron Karl Degeer Königlichen Hofmarschalls &c. &c. Abhandlungen zur Geschichte der Insekten aus dem Französischen übersetzt und mit Anmerkungen.* Gabriel Nikolaus Raspe, Nürnberg. (4to) GB

*Vierter und fünfter Band. Mit fünf und dreyssig Kupfertafeln.* [10] + 490 + [17 (Register)] pp. + 35 pls. [DP: 1781 (title page; *Vorerinnerung* dated “Ostermesse 1781”), 11 August 1781 (*Gött Anz*)].

*Siebender und letzter Band. Mit neun und vierzig Kupfertafeln.* [2] + 275 + [9 (Register)] pp. + 49 pls. [DP: 1783 (title page), 1 August 1783 (*Jena Ztg*), 8 November 1783 (*Gött Anz*)].

This is a German translation with comments of DeGeer’s “*Mémoires pour servir à l’histoire des insectes.*” It was published in seven *Bande*, 1778–1783. The Coleoptera are in the combined volumes 4 and 5 and in volume 7 (pp. 221–236).

The first two *Bande* of Goeze’s translation were originally issued in Leipzig, 1776–1777 [GB, first volume only] by Johann Carl Müller. No further parts were published in Leipzig.

#### Goldfuss, Georg August (Thurnau, Bavaria, Germany: 18 April 1782 – 2 October 1848: Bonn, North Rine-Westphalia, Germany). German zoologist and paleontologist; professor of zoology and mineralogy at the University of Bonn. References. Uschmann (1964); Langer (1969); Damkaer (2002: 200–201); Adler (2012: 69–70, P).

**1804**. *Envmeratio insectorvm elevtheratorvm Capitis Bonae Spei totivsqve Africae. Descriptione iconibvsqve nonnvllarvm speciervm novarvm illvstrata. Dissertatio inavgvralis qvam illvstris medicorvm ordinis avctoritate pro gradv doctoris medicinae et chirvrgiae proponit Georgivs Avgvstvs Goldfvss Thvrnaviensis. Die XVIII. Septemb. MDCCCIV.* Kvnstmann, Erlangae. viii + 9–44 pp. + 1 pl. (8vo) [DP: 18 September 1804 (title page)] CMLE, GB

The work was reissued in 1805 by Walker under the title “*Envmeratio insectorvm elevtheratorvm Capitis Bonae Spei totivsqve Africae descriptione iconibvsqve nonnvllarvm speciervm novarvm illvstrata. Cvm tabvla aenea*” [GB].

This thesis consists of a list of the Coleoptera from Cape of Good Hope and Africa (pp. 9–38) followed by the description of eight species (pp. 39–44) of which six are new to science.

**1820**. *Handbuch der Zoologie. Erste Abtheilung. Mit vier Tafeln.* Johann Leonhard Schrag, Nürnberg. xlvi + 696 pp. + 2 pls. (8vo) [DP: 1820 (title page), 18 October 1820 (*Allg Anz*), 15 November 1820 (*Regens Wochen*)] GB, MDZ

This work was published in two *Abtheilungen*, issued simultaneously. The Coleoptera are on pages 294–405 of the first *Abtheilung*. Goldfuss’ book forms the third *Theil* of Gotthilf Heinrich von Schubert’s *Handbuch der Naturgeschichte zum Gebrauch bei Vorlesungen* published in five *Theile*, 1813–1823.

**1826**. *Grundriss der Zoologie.* Johann Leonhard Schrag, Nürnberg. x + 734 + [2 (Druckfehler)] pp. (8vo) [DP: 1826 (title page; *Vorrede* dated 1 March 1826), 18 April 1826 (*Allg Ztg*), 6 May 1826 (*Intell Ztg Welt*), 31 May 1826 (*Bibl Deutsch*)] GB

Another title page reads “*Vollständiger Inbegriff der Pharmacie in ihren Grundlehren und praktischen Theilen. Ein Handbuch für Aerzte und Apotheker von J. Andreas Buchner. Vierten Theils, Dritter Band.*” The Coleoptera are on pages 195–248.

A second edition was issued in 1834 by the same publisher [GB]; the Coleoptera are on pages 281–317.

#### Gorham, Henry Stephen (Cookham, Berkshire, United Kingdom: 1839 – 22 March 1920: Great Malvern, Hereford and Worcester, United Kingdom). British clergyman and coleopterist; curate in several places in England; later, vicar of Shipley (West Sussex); his collection was dispersed but the Endomychidae and the types of Erotylidae and Languriidae are at the Natural History Museum in London while specimens of several other families ended up at the Muséum d’Histoire Naturelle in Paris via Maurice Pic [*q.v.*], Ernest Olivier and others. References. Anonymous (1920); Harvey et al. (1996: 88); Cambefort (2006: 179).

**1873**. *Endomycici recitati. A catalogue of the coleopterous group, Endomycici, with descriptions of new species, and notes.* Williams and Norgate, London and Edinburgh. [1] + 64 pp. + 1 pl. (8vo) [DP: 1873 (title page; preliminaries dated May 1873), 4 October 1873 (*Athenaeum*; *Spectator*), October 1873 (*English Cat Books*), 1 November 1873 (*Publ Circ*), 8 November 1873 (*Isis Natuur*), December 1873 (*Allg Bibl*), 2 February 1874 (*Ent Soc Lond*)] MCZ, GB

**1880–1886**. *Biologia Centrali-Americana. Insecta. Coleoptera. Vol. III. Part 2. Malacodermata.* Taylor and Francis, London. xii + 372 pp. + 13 pls. (4to) CNC

This work was published in 21 parts. The dates listed at the bottom of the first page of each sheet are: (pp. 1–24, pls 1–2) December 1880; (pp. 25–40, pl. 3) February 1881; (pp. 41–64, pl. 4) April 1881; (pp. 65–80, pl. 5) June 1881; (pp. 81–88) August 1881; (pp. 89–104) October 1881; (pp. 105–112, pl. 6) December 1881; (pp. 113–128) April 1882; (pp. 129–152, pl. 7) June 1882; (pl. 8) August 1882; (pp. 153–168, pl. 9) October 1882; (pp. 169–192) January 1883; (pp. 193–224) July 1883; (pl. 10) September 1883; (pp. 225–240, pl. 11) August 1884; (pp. 241–272) October 1884; (pp. 273–288, pl. 12) March 1885; (pp. 289–304) April 1885; (pp. 305–312) May 1885; (pp. 313–336) April 1886; (pp. 337–372, i–xii, pl. 13) May 1886. The collation for the plates is from Lyal (2011: 82).

**1887–1899**. *Biologia Centrali-Americana. Insecta. Coleoptera. Vol. VII. Erotylidae, Endomychidae, and Coccinellidae.* Taylor and Francis, London. xii + 276 pp. + 13 pls. (4to) CNC

This work was issued in 24 parts as follows (see Lyal 2011: 88): (pp. 1–32 + pl. 1) September 1887; (pp. 33–48 + pl. 2) December 1887; (pp. 49–72 + pl. 3) April 1888; (pp. 73–80 + pl. 4) June 1888; (pp. 81–88) August 1888; (pp. 89–112 + pl. 5) November 1888; (pl. 6) December 1888; (pp. 113–120) February 1889; (pp. 121–128) March 1889; (pp. 129–144 + pl. 7) March 1890; (pp. 145–152 + pl. 8) February 1891; (pp. 153–160) May 1891; (pp. 161–168 + pl. 9) March 1892; (pp. 169–176) May 1892; (pp. 177–192 + pl. 10) January 1894; (pp. 193–208) July 1894; (pl. 11) October 1894; (pp. 209–216) October 1895; (pp. 217–232) March 1897; (pp. 233–240 + pl. 12) May 1897; (pl. 13) August 1897; (pp. 241–248) January 1898; (pp. 249–256) December 1898; (pp. 257–276, i-xii) February 1899.

**1892**. Coleoptera – (continued). Pp. 44–58 *in*: *Supplementary appendix to travels amongst the Great Andes of the Equator by Edward Whymper with contributions by H.W. Bates. T.G. Bonney. G.A. Boulenger. Peter Cameron. F. Day. W.L. Distant. A.E. Eaton, F.D. Godman. H.S. Gorham. Martin Jacoby. E.J. Miers. A. Sidney Ollif. O. Salvin. David Sharp. T.R.R. Stebbing. Illustrated.* John Murray, London. xxii + [1 (Addenda)] + 147 pp. + 14 pls. (4to) [DP: 1891 (title page), 2 March 1892 (Sharp 1892: 61), 18 March 1892 (*Science*), 26 March 1892 (*Athenaeum*), 9 April 1892 (*Publ Circ*), April 1892 (*Nat Nov*), 16 June 1892 (*Nature*), 13 July 1892 (*Soc Ent Fr*)] CNC, GB

According to Sharp (1892: 61), the printing was done in 1891 but the work was not available for purchase until 2 March 1892.

**1893**. Descriptions of Coleoptera collected by Mr. John Whitehead on Kina Balu, Borneo. Pp. 296–299 *in*: *Exploration of Mount Kina Balu, North Borneo. By John Whitehead. With coloured plates and original illustrations.* Gurney and Jackson, London. x + [2] + 317 pp. + 32 pls. (Folio) [DP: 1893 (title page; introduction dated 10 May 1893), 22 July 1893 (*Athenaeum*), July 1893 (*English Cat Books*), August 1893 (*Nat Nov*)] MCZ, CNC, GB

The new species included in this work were published previously in the *Proceedings of the Zoological Society of London* 1892: 83–90 (+ pl. 4).

#### Górriz y Muñoz, Ricardo José (Cariñena, Aragon, Spain: 1850 – 1916: Saragossa, Aragon, Spain). Spanish pharmacist and naturalist; practiced in Paniza, Cariñena and Milagro, places where he collected plants and insects. References. Dusmet y Alonso (1919: 162); Andrés Arribas (2015: 38–39).

**1882**. *Ensayo para la monografía de los coleópteros melóidos indígenas con aplicacion á las ciencias médicas. (Con dos láminas litografiadas y coloreadas).* Julián Sanz y Navarro, Zaragoza. x + [1] + pp. 12–200 + [1] + 2 pls. (8vo) [DP: 1882 (title page)] BDH

Two new species are described in this work: *Meloe fissicornis* (p. 38) and *Mylabris lichtensteni* (p. 124).

#### Gory, Hippolyte Louis (Paris, France: 27/28 September 1800 – 26 April 1852: Paris, France). French captain of cavalry and coleopterist; founding member of the *Société Entomologique de France*; his collection was scattered after his death. Reference. Cambefort (2006: 179).

**1833**. *Monographie du genre Sisyphe.* Méguignon-Marvis Père et Fils, Paris. 15 pp. + 1 pl. (8vo) [DP: 1833 (title page), 24 August 1833 (*Bibl Fr*), 2 October 1833 (*Soc Ent Fr*)] MCZ, GB, MDZ

**1833–1836**. [Gory, H.L. and Percheron, A.] *Monographie des cétoines et genres voisins, formant, dans les familles naturelles de Latreille, la division des scarabées mélitophiles.* J.B. Baillière, Paris [&] Londres. 410 pp. + 77 pls. (8vo) CNC, BHL, GB, MDZ

This work was issued in 15 livraisons: **1**: (pp. 1–64 + 7 pls) 25 May 1833 (*Bibl Fr*); **2**: (pp. 65–106 + 5 pls) 24 March 1834 (*Acad Sci Fr*), 14 June 1834 (*Bibl Fr*); **3**: (pp. 107–122 + 5 pls) 19 May 1834 (*Acad Sci Fr*), 14 June 1834 (*Bibl Fr*); **4**: (pp. 123–??) before 20 October 1834; **5**: (pp. ??–??) 20 October 1834 (*Acad Sci Fr*); **6**: (pp. ??–??) 12 January 1835 (*Acad Sci Fr*); **7**: (pp. ??–??) 30 March 1835 (*Acad Sci Fr*); **8**: (pp. ??–234 + 5 pls) April–June 1835 (*Soc Ent Fr* [*Annales*, p. l]), 5 September 1835 (*Bibl Fr*), 21 September 1835 (*Acad Sci Fr*); **9**: (pp. 235–?? + 5 pls) July–September 1835 (*Soc Ent Fr* [*Annales*, p. lix]), 9 November 1835 (*Acad Sci Fr*); **10–11**: (pp. ??–298 + 10 pls) January– March 1836 (*Soc Ent Fr* [*Annales*, p. xxvi]), 18 April 1836 (*Acad Sci Fr*); **12** (pp. 299–?? + 5 pls), 9 May 1836 (*Acad Sci Fr*), April–June 1836 (*Soc Ent Fr* [*Annales*, p. xxxviii]); **13**: (pp. ??–?? + 5 pls) 23 May 1836 (*Acad Sci Fr*), April–June 1836 (*Soc Ent Fr* [*Annales*, p. xxxviii]); **14–15**: (pp. ??–410 + 10 pls) 14 November 1836 (*Acad Sci Fr*), 17 December 1836 (*Feuil J Lib*), October–December 1836 (*Soc Ent Fr* [*Annales*, p. cv]). The title page is dated 1833.

Sherborn (1922a: lx) gave the following collation and dates of publication: (pp. 1–64) May 1833; (pp. 65–202) 1834; (pp. 203–234) 1835; (pp. 235–406) 1836. The source of his dating is unknown. Mannerheim (1837: 127–137) gave an extended review of the book and noted that the first livraison is devoted “*aux généralités, au tableau synoptique des genres et aux planches qui y ont rapport, ainsi qu’au tableau des espèces*.”

**1836–1840**. [Gory, H. and Laporte, F.L.] *Histoire naturelle et iconographie des insectes coléoptères, publiée par monographies séparées; par H. Gory. Suite aux buprestides. Texte, tome II / Atlas, tome II.* P. Duménil, Paris. [454] pp. + 94 pls. (8vo) CNC, CMLE, GB

The entire work consists of four volumes of text and three volumes of plates, issued in livraisons combining text and plates. The first volume was authored by Laporte and Gory, 1835–1838 [*q.v.*], the second by Gory and Laporte, 1836–1840, and the fourth one by Gory alone, 1840–1841 (see next entry). The third volume is a reissue of the *Monographie du genre Clytus*, first published in 1836–1837 by Laporte and Gory [*q.v.*], dated 1841 where the author’s names are inverted. The content and dates of publication of this volume are discussed under the entry for Laporte and Gory, 1835–1838.

Although the title page indicated that Gory was the sole author of this volume, Gory (1841: 392) himself mentioned that by an unfortunate error the name of his friend was omitted on the title page [“par erreur qui m’est entièrement étrangère et contre laquelle j’ai déjà réclamé, le nom de mon collaborateur et ami se trouve accidentellement omis].”A corrected title page was eventually issued with Gory and Laporte as the authors.

[**1840]–1841**. *Histoire naturelle et iconographie des insectes coléoptères, supplément aux buprestides. Texte, tome IV / Atlas, tome IV.* P. Duménil, Paris. 356 + 7 (Tableau des espèces) + 2 (Liste des auteurs cités dans cet ouvrage) + [2] pp. + 60 pls. (8vo) CNC, GB

The entire series consists of four volumes of text and three volumes of plates (see previous entry). This volume, the fourth of the series, was issued in ten livraisons (corresponding to livraisons 43–52 of the entire series) containing text and plates. It was authored by Gory alone and is the only volume of the series continuously paginated. Dates found during this research are: **43–45**: (pp. 1–??) 7 April 1841 (*Soc Ent Fr*); **46–50**: (pp. ??–??) 1 September 1841 (*Soc Ent Fr*), 20 September 1841 (*Acad Sci Fr*); **51–52**: (pp. ??–356) November 1841 (*Rev Zool*). The title pages for the text and atlas are dated 1841 and the preliminaries August 1840 (p. 2). The Atlas contains 60 plates and some were issued with livraisons 40–43 (Guérin-Méneville 1840: 342) noticed on 16 November 1840 by the *Académie des Sciences de France*. Sherborn (1922a: lxxviii), without qualification, gave the dates of 1840 for pages 1–206 and 1841 for pages 207–356. Both Obenberger (1926: 1) and Nelson and Bellamy (1993: 298) gave the date 1841 for livraisons 43–52.

#### Gozis, Maurice Gilbert Perrot des (Montluçon, Allier, France: 12 November 1851 – 11 April 1909: Montluçon, France). French lawyer at Montluçon interested in entomology (Coleoptera and Orthoptera), history, genealogy and music; his collection was sold at auction. References. Clément (1909); Cambefort (2006: 156–157).

**1875**. *Catalogue des coléoptères de France et de la faune gallo-rhénane.* Montluçon. ii + 106 pp. (12mo) [DP: October 1875 (title page), 10 November 1875 (*Soc Ent Fr*), 10 October–13 November 1875 (*Soc Linn Nord Fr*), 1 December 1875 (*Feuille Jeunes Nat*)] CMLE

**1886**. *Recherche de l’espèce typique de quelques anciens genres. Rectifications synonymiques et notes diverses.* Herbin, Montluçon. 36 pp. (8vo) [DP: 1886 (title page), 10 March 1886 (*Soc Ent Fr*), July 1886 (*Nat Nov*), 18 September 1886 (*Bibl Fr*)] CNC, GB

#### Graells y de la Agüera, Mariano de la Paz (Tricio, La Rioja, Spain: 24 January 1809 – 14 February 1898: Madrid, Spain). Spanish naturalist; professor of zoology and comparative anatomy in Madrid; part of his collections were destroyed and the remaining is at the Museo Nacional de Ciencias Naturales in Madrid. References. Dusmet y Alonso (1919: 83–84); Agenjo (1943, P); Aragón Albillos (2005); Aguilar (2011, P); Galera (2011).

**1858**. *Memorias de la Comision del Mapa Geológico de España, año de 1855. Parte zoológica.* Imprenta Nacional, Madrid. 111 + [1 (Erratas)] pp. + 7 pls. (8vo) [DP: 1858 (title page; preface dated 30 December 1855), July–September 1858 (*Soc Ent Fr* [*Bulletin bibliographique*, p. cc])] CNC

This work includes two sections: “*Catalogo metódico de los insectos coleópteros de las dos primeras familias [Cicindelideos – Carabideos], observados en España hasta el dia por el vocal de la Seccion Zoológica D. Mariano de la Paz Graells*” (pp. 5–35) and “*Insectos nuevos de España, descubiertos y descritos por el Dr. D.M.P. Graells*” (pp. 36–111). These sections were also issued, the same year, in “*Memoria que comprende los trabajos verificados en el año de 1855 por las diferentes secciones de la Comision encargada de formar el mapa geológico de la provincia de Madrid y el general del reino. Presentada al Excmo. Sr. Ministro de Fomento por D. Guillermo Schulz*” [GB] on pages 43–73 and 74–149 + [1].

#### Gräfe, Heinrich Gotthilf Adam (Buttstädt, Thuringia, Germany: 3 March 1802 – 21 July 1868: Bremen, Bremen, Germany). German educator; rector of public schools in Jena and later in Cassel where he became a member of the school commission and the Kurhessian parliament; jailed in 1852 for his implication in the previous year riots and in the movement against the unpopular minister Hassenpglug who had dissolved the school commission; after his release moved to Geneva and eventually to Bremen where he was appointed director of the *Realschule in der Altstadt*. References. Lothholz (1879); Lindner (1884: 349–350).

**1836**. *Handbuch der Naturgeschichte der drei Reiche für Schule und Haus. In Verbindung mit J.F. Naumann. Erster Band. Thierreich.* Georg Reichardt, Eisleben und Leipzig. xx + 1081 + [2 (Druckfehler)] pp. (8vo) [DP: 1836 (title page), 8 July 1836 (*Allg Bibl Deutsch*)] GB, MDZ

This volume was first issued in 14 *Lieferungen* under the title “*Naturgeschichte der drei Reiche für Schule und Haus. In Verbindung mit J.F. Naumann*” as follows: **1–2**: (pp. 1–160) 16 August 1834 (*Bibl Deutsch*); **3**: (pp. 161–240) 25 December 1834 (*Bibl Deutsch*); **4–6**: (pp. 241–480) 24 January 1835 (*Bibl Deutsch*); **7–8**: (pp. 481–640) 26 November 1835 (*Bibl Deutsch*); **9**: (pp. 641–720) 27 November 1835–11 February 1836; **10**: (pp. 721–800) 12 February 1836 (*Allg Bibl Deutsch*); **11–12**: (pp. 801–960) 11 March 1836 (*Allg Bibl Deutsch*); **13–14**: (pp. xx + 961–1081) 8 July 1836 (*Allg Bibl Deutsch*).

The entire series was issued in three volumes, 1836–1838. The Coleoptera are in the first volume on pages 780–806. The first volume was also published simultaneously under the title “*Handbuch der Naturgeschichte der Thierreichs für Schule und Haus.*”

#### Gravenhorst, Johann Ludwig Christian (Brunswick, Lower Saxony, Germany: 14 November 1777 – 17 January 1857: Breslau [currently Wróclaw], Poland). German zoologist; professor at the University of Göttingen and later at the University of Breslau; director of the Breslau Zoologisches Museum; worked primarily on Ichneumonidae and Staphylinidae; his Coleoptera collection is at the Museum of Natural History, University of Wróclaw (formerly Breslau Zoologisches Museum). References. Letzner (1857); Adler (1989: 28, P); Smetana and Herman (2001: 70–71, P); Frank (2008: 1716); Jałoszyński and Wanat (2014).

**1802**. *Coleoptera microptera Brunsvicensia nec non exoticorum quotquot exstant in collectionibus entomologorum Brunsvicensium in genera familias et species distribuit.* Carolum Reichard, Brunsuigae [= Brunswick]. lxvi + 206 + [1 (Errata emendanda)] pp. (8vo) [DP: 1802 (title page; preliminaries dated March 1802), March–June 1802 (Pope and Marshall 1980: 120), 20 July–18 August 1802 (*Soc Philom*)] CNC, SME, GB, BHL

This book treats the species of Staphylinidae (*sensu stricto*) of the Duchy of Brunswick and some exotic ones found in the entomological collections in Brunswick. Many new species are described.

**1806**. *Monographia coleopterorvm micropterorvm.* Henric Dieterich, Gottingae. xvi + [1] + 236 + [12 (Indexes)] pp. + [1 folded table (Tabula affinitatum generum Coleopterorum micropterorum…)] (8vo) [DP: 1806 (title page), 20 December 1806 (*Gött Anz*), January–June 1807 (*Verz Bücher Landkarten*)] SME, GB, BHL, MDZ

This work constitutes a taxonomic revision of the staphylinid genera *Tachyporus* (pp. 1–21), *Tachinus* (pp. 22–34), *Staphylinus* (pp. 35–127), *Lathrobium* (pp. 128–135), *Paederus* (pp. 136–143), *Aleochara* (pp. 144–177), *Lomechusa* (pp. 178–182), *Oxytelus* (pp. 183–200), *Evaesthetus* (pp. 201–202), *Omalium* (pp. 203–219), *Anthophagus* (pp. 220–222), *Piestus* (pp. 223–224), *Stenus* (pp. 225–234) and *Oxyporus* (pp. 234–236). Many new species are described.

**1807**. *Vergleichende Uebersicht des Linneischen und einiger neuern zoologischen Systeme. Nebst dem eingeschalteten Verzeichnisse der zoologischen Sammlung des Verfassers und den Beschreibungen neuer Thierarten, die in derselben vorhanden sind.* Heinrich Dieterich, Göttingen. xx + 476 pp. (8vo) [DP: 1807 (title page), 19 March 1807 (*Gött Anz*), January–June 1807 (*Verz Bücher Landkarten*), 5 August 1807 (*Allg Lit Ztg*)] MCZ, GB

This is a catalogue of the animals in the author’s collection; many new species are described. The Coleoptera are on pages 61–242.

**1843**. *Vergleichende Zoologie*. Grass, Barth und Comp., Breslau. xx + 686 + [1 (Verbesserungen)] pp. (8vo) [DP: 1843 (title page; preliminaries [p. vi] dated 1 January 1843), 4 May 1843 (*Allg Bibl Deutsch*), 5 May 1843 (*Leip Reper*), January–June 1843 (*Verz Bücher Landkarten*), 4 July 1843 (*Lit Ztg*)] GB

A classification of the Coleoptera is included on pages 174–178.

#### Gredler, Vincenz Maria (Telfs, Tirol, Austria: 30 September 1823 – 4 May 1912: Bolzano, Trentino-Alto Adige, Italy). Italian (Austrian-born) Franciscan, entomologist and malacologist; worked primarily on the entomological fauna of Tirol; professor and director of the Franciscan Gymnasium in Bolzano; his collection of Coleoptera is located in that institution; the journal *Gredleriana*, published by the Museo di Scienze Naturali dell’Alto Adige, honors his memory. References. Dalla Torre (1912); Schröder (1912); Evenhuis (1997a: 325, P); Anonymous (2001, P).

**1863–1866**. *Die Käfer von Tirol nach ihrer horizontalen und vertikalen Verbreitung.* J. Eberle, Bozen. iv + [1] + 491 pp. (8vo) CNC, GB, MDZ

*I. Hälfte: Cicindelidae–Dascillidae. Mit mehren diagnosirten Novitäten.* iv + [2] + pp. 1–234. [DP: 1863 (title page; *Vorwort* dated 4 October 1862), May 1863 (Gredler 1873: 59)].

*II. Hälfte: Dascillidae–Schluss. Mit mehren diagnosirten Novitäten.* Pp. 235–491. [DP: 1866 (title page), 19 April 1866 (*Allg Bibl Deutsch*), May 1866 (*Allg Bibl*), January–June 1866 (*Bibliot Hist-Nat*), 31 July 1866 (*Intell Serapeum*)].

This work is a catalogue of the Coleoptera of Tirol in Austria with comments; several new species are described.

#### Grenier, Auguste Jean François (Les Andelys, Eure, France: 22 September 1814 – 13 July 1890: Bagnères, France). French physician and entomologist (1857–1869) mainly interested in Coleoptera of France; his collection, which included that of Charles Nicholas Aubé [*q.v.*] and in part that of Jules Linder, was bequeathed to Albert Léveillé and subsequently scattered. References. Bonvouloir (1891, P); Lhoste (1987: 73); Gouillard (2004: 45–46); Cambefort (2006: 182).

**1863**. *Catalogue des coléoptères de France par M. le D^r^ A. Grenier et matériaux pour servir a la faune des coléoptères français par MM. E. Allard, D^r^ Ch. Aubé, Ch. Brisout de Barneville, A. Chevrolat, L. Fairmaire, Al. Fauvel, D^r^ A. Grenier, D^r^ Kraatz, J. Linder, L. Reiche et Félicien de Saulcy.* A. Grenier, Paris. iv + 79 + 135 pp. (8vo) [DP: 21 July 1863 (title page), August 1863 (Grenier 1864: xiii), 7 November 1863 (*Bibl Fr*), July–December 1863 (*Bibliot Hist-Nat*)] CNC, GB, GAL

This work is divided in two parts. The first one is a checklist of the Coleoptera of France and the second one consists of the descriptions of 158 new species from France.

**1867**. *Matériaux pour servir à la faune des coléoptères de France recueillis et publiés par le D^r^. A. Grenier. 2^e^ cahier.* A. Grenier, Paris. iv + pp. 131–194 (8vo) [DP: July 1867 (title page; *avis* dated 3 July 1867), 10 August 1867 (*Bibl Fr*), 5 October 1867 (*Soc Ent Belg*)] MCZ, GB, GAL

This work includes: 1. Étude monographique sur le genre *Trechus* (espèces européennes) par L. Pandellé (pp. 131–161); 2. [Isolated descriptions of species by Ch. Brisout, Fél. de Saulcy, L. Pandellé] (pp. 161–167); 3. Synopsis des espèces françaises du genre *Proteinus* par L. Pandellé (pp. 168–169); 4. Synopsis des *Oxytelus* français du groupe du *depressus* par L. Pandellé (pp. 170–173); 5. [Isolated descriptions of species by Ch. Brisout and L. Pandellé] (pp. 173–182); 6. Synopsis des *Apion* français du groupe de l’*ulicis* par L. Pandellé (pp. 183–185); 7. [Isolated descriptions of species by Ch. Brisout] (pp. 185–194).

#### Griffini, Achille (Milan, Lombardy, Italy: 10 August 1870 – 24 June 1932: Brescia, Lombardy, Italy). Italian zoologist; assistant at the Museo di Zoologia of the University of Turin; later, professor in high school in several Italian cities; his collection was sold by lots. Reference. Conci (1975: 929–930).

**1894**. *Manuali Hoepli. Entomologia I.^o^ Coleotteri italiani. Con 215 incisioni.* Ulrico Hoepli, Milano. xv + 334 pp. (8vo) [DP: 1894 (title page), 24 June 1894 (*Gior Lib*), June 1894 (*Nat Nov*), 10 June–5 July 1894 (*Ist Ven Sci*), 15 July 1894 (*Bibl Ital-2*), 31 October 1894 (*Wien Ent Ztg* 13: 259), 31 December 1894 (*Monit Zool Ital*)] NAL

**1895**. *Il libro dei Coleotteri. Iconografia dei principali coleotteri italiani e dell più importanti specie Europee affini. Preceduta da notizie generali sugli insetti e principalmente sui coleotteri nonchè da indicazioni sulla raccolta, la preparazione, la coservazione e lo studio di questi. Opera illustrata da 50 ricche tavole in cromolitografia rappresentanti oltre 1300 specie, e da 179 incisioni intercalate nel testo.* Ulrico Hoepli, Milano. ix + [1] + 244 pp. + 50 [i–ii, 1–48] pls. (8vo) [DP: 1896 (title page), 15 October 1895 (*Bibl Ital-2*), November 1895 (*Nat Nov*)] NCSU, GDZ

#### Griffith, Edward (Stanwell, Surrey, United Kingdom: December 1790 – 8 January 1858: London, United Kingdom). British naturalist by avocation and a solicitor and functionary of the court of common pleas by profession; interested in the history of London’s parliamentary divisions; initiated an updated translation of the four-volume first edition of Cuvier’s classic work; corresponding member of the Philadelphia Academy of Sciences. References. Boulger (1890); Gruber (2004).

**1831–1832**. [Griffith, E. and Pidgeon, E.] *The class Insecta arranged by the Baron Cuvier, with supplementary additions to each order. And notices of new genera and species by George Gray, Esq.* Whittaker, Treacher, and Co., London. (4to or 8vo) CNC

*Volume the first.* 570 pp. + 53 pls. This volume was published in three parts (dates from Cowan 1969: 139): (pp. 1–192) June 1831; (pp. 193–384) September 1831; (pp. 385–570) December 1831. The title page is dated 1832. The second page is entitled “*The animal kingdom arranged in conformity with its organization, by the Baron Cuvier, with supplementary additions to each order, by Edward Griffith, and others. Volume the fourteenth.*”

*Volume the second.* viii + 796 pp. + 87 pls. This volume was published in four parts (dates from Cowan 1969: 139 unless otherwise noted): (pp. 1–192) March 1832; (pp. 193–384) June 1832; (pp. 385–576) September 1832; (pp. 577–796) December 1832 (Evenhuis 1997a: 328). The title page is dated 1832. The second page is entitled “*The animal kingdom arranged in conformity with its organization, by the Baron Cuvier, with supplementary additions to each order, by Edward Griffith, and others. Volume the fifteenth.*”

The entire series consists of 16 volumes, 1827–1835. These two volumes consist of a translation, with additions, of Latreille (1829) “*Le règne animal distribué d’après son organisation*” and form volumes 14 and 15 of “*The animal kingdom arranged in conformity with its organization*.” According to Freeman (1980: 100), two printings were done: the quarto copies have uncolored plates on India paper and the Demy octavo copies have colored plates. As indicated on the title page, the new genera and species are to be attributed to George Robert Gray [1808–1872]. The Coleoptera are on pages 170–570 of the first volume and pages 1–168 of the second volume.

**1835**. *A classified index and synopsis of the animal kingdom arranged in conformity with its organization, by the Baron Cuvier. With supplementary additions to each order, by Edward Griffith and others.* Whittaker and Co., London. cxix + 328 pp. (8vo) [DP: 1835 (title page), 7 March 1835 (*Lit Gaz*), 14 March 1835 (*Athenaeum*)] BHL, GB

The work is divided in three sections: Conspectus of the entire animal kingdom (pp. iii–cxix); Index to the animal kingdom (pp. 1–282); Alphabetical table of authors quoted in this work (pp. 283–328). The first section includes a list of all known genera in a classification schema with an “example of species.” The Coleoptera are on pages lxxii–xci.

This book forms the final (i.e., 16th) volume of “*The animal kingdom arranged in conformity with its organization*” 1827–1835. According to Smith (1993: 188) the book was also issued in 1844 (*n.v.*).

#### Grill, Claes Erik (Sala, Västmanland, Sweden: 18 February 1851 – 5 March 1919: Stockholm, Sweden). Swedish army colonel and entomologist, particularly interested in beetles; his collection was donated in 1918 to the College of Forestry in Stockholm. Reference. Trägårdh (1920, P).

**1895–1896**. *Förteckning öfver Skandinaviens, Danmarks och Finlands Coleoptera. Jämte deras synonymi och geografiska utbredning.* Entomologiska Föreningen, Stockholm. vi + [1] + 426 + [1] pp. (8vo) GB

This work was published in two parts: (pp. 1–184) May 1895 (*Nat Nov*), 25 September 1895 (*Wien Ent Ztg* 14: 275); (vi + pp. 185–426) December 1896 (*Nat Nov*), 28 February 1897 (*Wien Ent Ztg* 16: 80). The title page is dated 1896 and the preliminaries May 1896. A new replacement name, *Rhyncolus thomsoni* (p. 306), is proposed.

The work has another title page which reads “*Catalogus coleopterorum Scandinaviae, Daniae et Fenniae. Adjectis synonymis gravioribus, observationibus et indicata singulorum distributione geographia*.”

#### Grimmer, Karl Heinrich Benjamin. Coat of arms archivist in Graz (Austria) and coleopterist; his collection is at the Universalmuseum Joanneum (formely Landesmuseum Joanneum) in Graz. References. Gistel (1856: 190); Göth (1861: 60).

**1841**. *Steiermark’s Coleoptern mit Einhundert sechs neu beschriebenen Species.* C. Tanzer, Grätz. iv + 5–50 pp. (8vo) [DP: 1841 (title page; *Vorrede* dated “Herbstmonat 1840”), 26 May 1841 (*Lit Ztg*), May 1841 (*Bull Litt Étr*), 4 June 1841 (*Allg Bibl Deutsch*), January–June 1841 (*Verz Bücher Landkarten*), July–September 1841 (*Foreign Quart Rev*), December 1841 (*Rev Zool*)] CMLE (mf), GB, MDZ

This publication consists of a list of duplicate Coleoptera, presumably from the author’s collection, followed by the descriptions of 106 new species of beetles.

**1846**. *Grundlage zur Fauna Steyermark’s dargestellt durch das Coleoptern-Verzeichniss und des Doubletten-Vorraths, nebst Beobachtungen im Betreff der Varietaeten.* J.A. Kienreich, Gratz. [2] + 115 + [1] pp. (8vo) [DP: 1846 (title page), July 1846 (*Not Geb Natur*)] GB, EUR

This work contains a list of the Coleoptera in the author’s collection followed by a list of his duplicate species. There is no new species described.

#### Gronov [Gronovius], Lorenz [Laurens, Laurentius] Theodor [Theodorus] (Leiden, South Holland, Netherlands: 1 June 1730 – 8 August 1777: Leiden, Netherlands). Dutch naturalist and municipal officer in Leiden; son of the botanist Jan Frederik Gronovius [1686–1762]. References. Thomas (1870: 1088); Anonymous (2013a, P).

**1764**. *Zoophylacii Gronoviani. Fasciculus secundus exhibens enumerationem insectorum, quae in museo suo adservat, examini subjecit, systematice disposuit atque descripsit. Additis rarissimorum insectorum iconismis.* Sumptibus auctoris, Lugduni Batavorum [= Leiden]. [1] + pp. 141–236 + pls 14–17. (Folio) [DP: 1764 (title page), 19 December 1765 (*Gött Anz*)] GB

The entire work consists of three fascicles, sequentially paginated, issued in 1763, 1764 and 1781 (Wheeler 1956: 152). The arthropods make up the entire second fascicle. Many species are described but no specific names are provided, only generic ones. The entire work has been rejected for nomenclatural purposes and placed on the *Official Index of Rejected and Invalid Works in Zoological Nomenclature* (Opinion 261, ICZN 1954d). Meuschen published an index to this work in 1781, containing binominal names to all the animals described, and the index has also been rejected by the Commission in the same Opinion. The index, unnumbered, is usually included at the end of the third volume.

#### Guérin-Méneville [Guérin]^35^, Felix Édouard (Toulon, Var, France: 12 October 1799 – 26 January 1874: Paris, France). French entomologist interested first in systematics and later in economic entomology, particularly sericulture; founding member of the *Société Entomologique de France*; founded the journal *Magasin de Zoologie* which became later the *Revue et Magasin de Zoologie*; lecturer for some times at the Collège de France in Paris; his collection was scattered. References. Anonymous (1874d); Zimmerman (1993: 558); Smetana and Herman (2001: 72–73, P); Cambefort (2006: 183–185); Adler (2012: 86–87, P).

**1829–1837**. *Iconographie du règne animal de G. Cuvier, ou représentation d’après nature de l’une des espèces les plus remarquables et souvent non encore figurées, de chaque genre d’animaux. Avec un texte descriptif mis au courant de la science. Ouvrage pouvant servir d’atlas à tous les traités de zoologie.* J.B. Baillière, Paris. 450 pls. (8vo) CNC

This works contains 450 plates published in 45 livraisons, from 21 March 1829 to December 1837. The insects section contains 110 plates and the Coleoptera are on plates 3–51. Notices of publication for the Coleoptera plates are: **plates 3–5** [livr. 1]: 21 March 1829 (*Bibl Fr*), 23 March 1829 (*Acad Sci Fr*), January–March 1829 (*Foreign Quart Rev*); **6–7** [livr. 2]: 18 July 1829 (*Bibl Fr*); **8–10** [livr. 3]: 12 September 1829 (*Bibl Fr*), 21 September 1829 (*Acad Sci Fr*); **11–12** [livr. 4]: 16 November 1829 (*Acad Sci Fr*), 21 November 1829 (*Bibl Fr*); **13** [livr. 6]: 3 April 1830 (*Bibl Fr*), 3 May 1830 (*Acad Sci Fr*); **14** [livr. 5]: November–December 1829 (*Foreign Quart Rev*), 23 January 1830 (*Bibl Fr*), 15 February 1830 (*Acad Sci Fr*); **15–20** [livr. 37]: October–December 1834 (*Soc Ent Fr* [*Annales*, p. xcv]); **21–24** [livr. 9]: 25 September 1830 (*Bibl Fr*), 18 October 1830 (*Acad Sci Fr*); **24*bis*** [livr. 11]: 26 February 1831 (*Bibl Fr*); **25–28** [livr. 10]: 15 January 1831 (*Bibl Fr*), 31 January 1831 (*Acad Sci Fr*); **25*bis*** [livr. 11]: 26 February 1831 (*Bibl Fr*), 28 March 1831 (*Acad Sci Fr*); **28*bis*, 29–30** [livr. 12]: March 1831 (*Bull Sci Nat Géol*), 16 April 1831 (*Bibl Fr*), 13 June 1831 (*Acad Sci Fr*); **31** [livr. 28]: 23 March 1833 (*Bibl Fr*), 31 March 1833 (*Bibliologue*), January–March 1833 (*Soc Ent Fr* [*Annales*, p. xxxiii]), 1 July 1833 (*Acad Sci Fr*); **32–35** [livr. 35]: July–September 1834 (*Soc Ent Fr* [*Annales*, p. liii]); **36** [livr. 29]: April–June 1833 (*Soc Ent Fr* [*Annales*, p. xlii]); **37–38** [livr. 30]: April–June 1833 (*Soc Ent Fr* [*Annales*, p. xlii]), 6 July 1833 (*Bibl Fr*); **39, 39*bis*** [livr. 31]: July–September 1833 (*Soc Ent Fr* [*Annales*, p. lxii]); **40** [livr. 38]: 21 March 1835 (*Acad Sci Fr*), January–March 1835 (*Soc Ent Fr* [*Annales*, p. xxxv]), April 1835 (*Bull Bibl*); **41** [livr. 44]: 13 November 1837 (*Acad Sci Fr*); **42–44** [livr. 13]: March 1831 (*Bull Sci Nat Géol*), 21 May 1831 (*Bibl Fr*), 13 June 1831 (*Acad Sci Fr*); **45–46** [livr. 14]: March 1831 (*Bull Sci Nat Géol*), April–June 1831 (*Foreign Quart Rev*), 9 July 1831 (*Bibl Fr*); **47–49** [livr. 32]: July–September 1833 (*Soc Ent Fr* [*Annales*, p. lxii]); **49*bis*, 50** [livr. 36]: October–December 1834 (*Soc Ent Fr* [*Annales*, p. xcv]), 9 February 1835 (*Acad Sci Fr*); **51** [livr. 37]: October–December 1834 (*Soc Ent Fr* [*Annales*, p. xcv]). Cowan (1971b) commented on the collation and dates of publication of this work.

Each livraison had ten plates with black or color figures. The text was issued in 1844 [*q.v.*]. Several species were first made available through illustrations from the plates. The species depicted on the plates are listed in Appendix 12.

**1830–1831.** *Voyage autour du monde, exécuté par ordre du Roi, sur la corvette de Sa Majesté*, La Coquille, *pendant les années 1822, 1823, 1824 et 1825, sous le ministère et conformément aux instructions de S. E. M. Le Marquis de Clermont-Tonnerre, ministre de la marine; et publié sous les auspices de son Excellence Mgr. Le C^te^ De Chabrol, Ministre de la Marine et des Colonies, par M.L.I. Duperrey. Zoologie, par M. Lesson. Tome second. – 2^e^ partie.* Arthus Bertrand, Paris. 22 pls. (Folio) CAKL, BHL

The entire expedition report was divided into four sections: *Histoire du voyage*; *Botanique*; *Hydrographie*; and *Zoologie*. The *Zoologie* section was issued in two volumes of text and one Atlas of 157 plates, of which 22 (numbered 1–21 and 14*bis*) picture insects.

The plates were published before the text issued in 1838 [*q.v.*]. The Coleoptera are on plates *Insectes No 1–7*. The dates of publication of the plates are listed in Guérin-Méneville (1838: 271) as follows: **plate 1** [livr. 18]: 1 September 1830; **2** [livr. 19]: 25 November 1830; **3** [livr. 20]: 7 March 1831; **4** [livr. 23]: 25 July 1831; **5** [livr. 22]: 15 June 1831; **6** [livr. 20]: 7 March 1831; **7** [livr. 25]: 13 October 1831; **8** [livr. 26]: 15 November 1831; **9–10** [livr. 27]: 22 December 1831; **11** [livr. 24]: 5 September 1831; **12** [livr. 21]: 29 May 1831; **13–14** [livr. 17]: 26 May 1830; **14*bis*** [livr. 21]: 29 May 1831; **15–16** [livr. 18]: 1 September 1830; **17** [livr. 19] : 25 November 1830; **18** [livr. 22]: 15 June 1831; **19** [livr. 20]: 7 March 1831; **20** [livr. 23]: 25 July 1831; **21** [livr. 24]: 5 September 1831. Several Coleoptera taxa were made available from the plates. The species depicted on the plates are listed in Appendix 8 (see also Cretella 2010).

**1833**. Insectes. Pp. 441–512 + 5 pls *in*: *Voyage aux Indes-Orientales, par le nord de l’Europe, les provinces du Caucase, la Géorgie, l’Arménie et la Perse, suivi de détails topographiques, statistiques et autres sur le Pégou, les iles de Java, de Maurice et de Bourbon, sur le Cap-de-Bonne-Espérance et Sainte-Hélène, pendant les années 1825, 1826, 1827, 1828 et 1829, publié sous les auspices de LL. EE. MM. les Ministres de la Marine et de l’Intérieur; par M. Charles Bélanger. Dédié au Roi. Zoologie, par MM. Bélanger, Isidore Geoffroy-Saint-Hilaire, Lesson, Valenciennes, Deshayes et Guérin.* Arthus Bertrand, Paris. xxxix + 535 pp. [+ 40 associated pls]. (8vo–text; 4to–plates) [DP: 31 August 1833 (*Bibl Fr*)] CAKL, GB

The entire expedition report was published under the direction of the French botanist and explorer Charles Paulus Bélanger [1805–1881] and divided in three sections: *historique*, *zoologie*, and *botanique*. The *Zoologie* section consists of one volume of text and one volume of 40 plates, both with the date 1834 on the title page. The text and plates of the zoology volume were published in eight livraisons, 1831–1834 (Evenhuis 1997a: 79). The *insectes* section comprises the entire livraison 7 which was recorded on 31 August 1833 by the *Bibliographie de la France*^36^. The livraison consisted of five *feuilles* of text and five plates. It is assumed that the five plates are those dealing with insects although this cannot be confirmed at this time.

The Coleoptera are on pages 481–494 and several new species are described.

**1835–1838**. [Guérin-Méneville, F.E. and Percheron, A.R.] *Genera des insectes, ou exposition détaillée de tous les caractères propres a chacun des genres de cette classe d’animaux. 1^re^ série. Ordres et familles.* Méquignon-Marvis Père et Fils, Paris. 60 pls + text. (4to) CNC

This work was issued in six livraisons, each consisting of three or four unnumbered pages of text and a plate for each of ten insect genera (numbered 1–10) treated. Notices of publication of the livraisons are: **1**: 28 March 1835 (*Bibl Fr*), March 1835 (*Bull Bibl*), 1 April 1835 (*Soc Ent Fr*); **2**^37^: April–June (*Soc Ent Fr* [*Annales*, p. xlviii]), 5 August 1835 (*Soc Ent Fr*), August 1835 (*Bull Bibl*); **3**: 18 November 1835 (*Soc Ent Fr*); **4**: 20 April 1836 (*Soc Ent Fr*); **5**: 2 November 1836 (*Soc Ent Fr*); **6**: 1838 (Sherborn 1922a: lxiii). The title page is dated “1835–1838.”

A second version of this work was issued by J.B. Baillière with the title page reading “*Genera des insectes ou exposition détaillée de tous les caractères propres a chacun des genres de cette classe d’animaux. Ordres et familles. Avec 60 planches gravées et coloriées*” [GB]. The title page is also dated “1835–1838.”

The following Coleoptera genera were treated: *Therates* (no. 1 of livr. 1); *Evaniocera* (no. 2 of livr. 1); *Carabus* (no. 1 of livr. 2); *Cybister* (no. 2 of livr. 2); *Emus* (no. 1 of livr. 3); *Psiloptera* (no. 2 of livr. 3); *Oxysternus* (no. 3 of livr. 3); *Hydrous* (no. 4 of livr. 3); *Coprobius* (no. 5 of livr. 3); *Adesma* (no. 6 of livr. 3); *Helaeus* (no. 7 of livr. 3); *Cassida* (no. 1 of livr. 4); *Heilipus* (no. 2 of livr. 4); *Trogossita* (no. 3 of livr. 4); *Cucujus* (no. 4 of livr. 4); *Sternotomis* (no. 5 of livr. 4); *Pselaphacus* (no. 6 of livr. 4); *Coccinella* (no. 1 of livr. 5); *Endomychus* (no. 2 of livr. 5); *Calopus* (no. 1 of livr. 6); *Crioceris* (no. 2 of livr. 6); *Briaxis* (no. 3 of livr. 6). The first three genera were authored by Guérin-Méneville, all the other ones by Achille Rémi Percheron.

Mannerheim (1838) gave a lengthly review of the content of the first five livraisons and Evenhuis (2011a: 176–183) presented an analysis of the taxa and authorship depicted on each plate. The new taxa described in this work are: *Evaniocera* Guérin, 1835; *Emus nebulosus* Percheron, 1835; *Psiloptera tucumana* Percheron, 1835; *Hydrous senegalensis* Percheron, 1835; *Adesmia spinifera* Percheron, 1835; *Cassida nitidula* Percheron, 1836 [spelled *nilidula* in the text]; *Heilipus loricatus* Percheron, 1836; *Trogossita metallica* Percheron, 1836 [spelled *metellica* in the text]; *Sternotomis* Percheron, 1836; *Sternotomis aper* Percheron, 1836; *Pselaphacus* Percheron, 1836; *Pselaphacus nigropunctatus* Percheron, 1836; *Endomychus bivittatus* Percheron, 1836.

**1838**. *Voyage autour du monde, exécuté par ordre du Roi, sur la corvette de sa Majesté*, La Coquille, *pendant les années 1822, 1823, 1824 et 1825, sous le ministère et conformément aux instructions de S. E. M. Le Marquis de Clermont-Tonnerre, ministre de la marine; et publié sous les auspices de son Excellence Mgr le C^te^ De Chabrol, Ministre de la Marine et des Colonies, par M.L.I. Duperrey. Zoologie, par M. Lesson. Tome second. – 2^e^ partie.* Arthus Bertrand, Paris. xii + 9–319 pp. (4to) [DP: 1830 (title page; *avant-propos* dated 15 November 1838), 1838 (Lesson 1843: 48), 5 April 1839 (*Soc Géog Fr*)^38^] CAKL, BHL

The date on the title page pertains to the first year of publication of the plates (see Guérin-Méneville 1830–1831). The half-title page reads “*Première division. Crustacés, arachnides et insectes, par F.C. Guérin Méneville*.” The Coleoptera are on pages 57–152.

The expedition, which left Toulon in the Var department on 11 August 1822, was sponsored by the French government and had as its chief objective the collection of scientific data, and to report on the possibility of establishing a penal colony in Western Australia. The commander was Louis Isidore Duperrey [1786–1865].

**1839**. Insectes. Pp. 81–160 (2^e^ partie) + pls 33–46 *in*: *Voyage autour du monde par les mers de l’Inde et de Chine exécuté sur la corvette de l’État* La Favorite *pendant les années 1830, 1831 et 1832 sous le commandement de M. Laplace, capitaine de frégate: publié par ordre de M. le vice-amiral comte de Rigny ministre de la marine et des colonies. Tome V.* Arthus Bertrand, Paris. viii + 195 (1^re^ partie) + 200 (2^e^ partie) + 30 (Supplément) + 2 (table méthodique) pp. + 70 pls [numbered 1–60, Suppl. 1–10] (8vo) [DP: 1839 (title page; *avertissement* dated 12 December 1838)] USNM, BHL

The entire trip report was published in five volumes of text and two atlases, 1833–1839. The natural history section forms volume 5. It was published in six livraisons as indicated in the *Feuilleton du Journal de la Librairie* (6 April 1839) which recorded the fourth livraison on this date and mentioned that “*les livraisons 5 et 6, qui terminent cet ouvrage, paraîtront en avril*.” Collation and dates of publication for volume 5 are needed. Guérin-Méneville’s contribution (text and plates) was first issued in 1838 in *Magasin de Zoologie* 8 (Classe IX): pp. 1–80, pls 225–238.

**1843**. Animaux articulés. Pp. 33–98 (seconde partie) + pls 11–27 *in*: *Souvenirs d’un voyage dans l’Inde exécuté de 1834 à 1839 par M. Adolphe Delessert. Ouvrage enrichi de trente-cinq planches.* Fortin, Masson et C^ie^ [&] Langlois et Leclercq, Paris. iii + 134 + 107 (seconde partie) pp. + 35 pls. (8vo) [DP: 1843 (title page), 9 January 1843 (*Acad Sci Fr*), 14 January 1843 (*Bibl Fr*), 1 February 1843 (*Monit Lib*)] GB, BHL

The work relates to a five-year journey to India and southeast Asia by Adolphe Delessert [1809–1869]. It is divided in two parts; the first one deals with the history of the trip (134 pp.) and the second one with the natural history (107 pp.) . An account of Delessert’s travels was published by Eyriès (1843).

**1843–1849**. *Species et iconographie générique des animaux articulés ou représentation des genres, avec leur description et celle de toutes les espèces de cette grande division du règne animal, ouvrage formant une série de monographies complètes. Première partie: Insectes coléoptères.* Paris. (8vo) CNC, GB, GAL

This work was published in nine livraisons, issued as follows: **1**: 1 February 1843^39^, 1 March 1843 (*Soc Ent Fr*), 18 March 1843 (*Bibl Fr*), 1 April 1843 (*Monit Lib*; *Lit Ztg*), 14 April 1843 (*Leip Reper*), 15 April 1843 (*Intell Serapeum*); **2**: 5 April 1843 (*Soc Ent Fr*), April 1843 (*Rev Zool*); **3**^40^: 20 December 1843 (*Soc Ent Fr*); **4–9**: March–April 1849 (Sherborn 1922a: lxiii; Chandler 2000: 437). The pages are not numbered consecutively. The plates came colored or uncolored.

The content of each livraison is as follows: **livr. 1**: No 1 (Genus *Rhipicera*: 7 pp. + 1 pl.), No 2 (Genus *Sandalus*: 8 pp. + 1 pl.), No 3 (G. *Scirtes*: 6 pp. + 1 pl.) and No 4 (G. *Eucinetus*: 2 pp. + 1 pl.), all dated January 1843; **livr. 2**: No 5 (G. *Ptyocerus*: 5 pp. + 1 pl.), No 6 (G. *Selasia*: 3 pp. + 1 pl.), No 7 (G. *Chamaerhipis*: 2 pp. + 1 pl.) and No 8 (G. *Basodonta*: 2 pp. + 1 pl.), all dated February 1843; **livr. 3**: No 9 (G. *Elodes*: 16 pp. + 1 pl.), No 10 (G. *Bradytoma*: 2 pp. + 1 pl.), No 11 (G. *Octoglossa*: 2 pp. + 1 pl.), and No 12 (G. *Cladotoma*: 2 pp. + 1 pl.), all dated March 1843; **livr. 4**: No 13 (G. *Dascillus*: 8 pp. + 1 pl.), dated January 1849; No 14 (G. *Odontonyx*: 2 pp. + 1 pl.), dated January 1849; No 21 by M. de la Ferté-Sénectère (G. *Notoxus*: 36 pp. + 1 pl.), dated April 1847; No 22 by M. de la Ferté-Sénectère (G. *Mecynotarsus*: 5 pp. + 1 pl.), dated April 1847; **livr. 5** (all by M. de la Ferté-Sénectère): No 17 (G. *Eurygenius*: 3 pp. + 1 pl.), No 18 (G. *Stereopalpus*: 3 pp. + 1 pl.), No 19 (G. *Steropes*: 4 pp. + 1 pl.), No 20 (G. *Macrarthrius*: 10 pp. + 1 pl.), all dated November 1846; **livr. 6**: No 15 (G. *Anchytarsus*: 1 pp. + 1 pl.), dated January 1849; No 16 by M. Westwood (G. *Dodecatoma*: 1 pp. + 1 pl.)], dated April 1843; No 23 by M. de la Ferté-Sénectère (G. *Amblyderus*: 3 pp. + 1 pl.), dated April 1847; No 24 by M. de la Ferté-Sénectère (G. *Anthelephilus*: 5 pp. + 1 pl.), dated April 1847; **livr. 7**: No 25 by M. de la Ferté-Sénectère (G. *Formicomus*: 25 + [1 (Erratum)] pp. + 1 pl.), dated April 1847; No 26 by M. de la Ferté-Sénectère (G. *Tomoderus*: 8 pp. + 1 pl.), dated April 1847; No 27 by M. de la Ferté-Sénectère (G. *Anthicus* (première division): 45 pp. + 1 pl.), dated April 1847; No 33 (G. *Aploglossa*: 3 pp. + 1 pl.), dated February 1849; **livr. 8**: No 28 by M. de la Ferté-Sénectère (G. *Anthicus* (seconde division): pp. 47–83 + 1 pl.), dated April 1847; No 29 by M. de la Ferté-Sénectère (G. *Anthicus* (troisième division): pp. 85–132 + 1 pl.), dated May 1847; No 34 (G. *Therius*: 3 pp. + 1 pl.), dated February 1849; No 35 by M. Westwood (G. *Pachymesia*: 2 pp. + 1 pl.), dated February 1849; **livr. 9**: No 30 by M. de la Ferté-Sénectère (G. *Anthicus* (suite): pp. 133–181 + 1 pl.), dated May 1847; No 31 by M. de la Ferté-Sénectère (G. *Ochthenomus*: 9 pp. + 1 pl.), dated May 1847; No 32 by M. de la Ferté-Sénectère (G. *Agnathus*: 4 pp. + 1 pl.), dated May 1847; No 36 (G. *Cneoglossa*: 2 pp. + 1 pl.), dated February 1849; 8 pp. (Appendix aux monographies comprises depuis le No 17 jusqu’au No 32 inclusivement; Errata; Explication des planches).

**1844**. *Iconographie du règne animal de G. Cuvier, ou représentation d’après nature de l’une des espèces les plus remarquables et souvent non encore figurées, de chaque genre d’animaux. Avec un texte descriptif mis au courant de la science. Ouvrage pouvant servir d’atlas à tous les traités de zoologie. Insectes.* J.B. Baillière, Paris [&] Londres. 576 pp. (8vo) [DP^41^: 26 August 1844 (*Acad Sci Fr*; *Rev Zool*), 1 September 1844 (*Monit Lib*), 7 September 1844 (*Bibl Fr*), 30 September 1844 (*Intell Serapeum*)] CNC, GB, BHL

The text constitutes livraisons 46–50 of the work. The previous 45 livraisons contain plates only (see Guérin-Méneville 1829–1837). Many of the new taxa proposed in the text were made available earlier from the plates. The text contains 919 pages overall and each sections, i.e. mammals, birds, fishes, etc., are separately paginated. The title page of the insect section is dated 1829–1838.

[**1849**]. Insectes. Pp. 239–390 [+ 12 associated pls] *in*: *Voyage en Abyssinie exécuté pendant les années 1839, 1840, 1841, 1842, 1843 par une commission scientifique composée de MM. Théophile Lefebvre, Lieutenant de vaisseau, Chevalier de la Légion d’honneur, A. Petit et Quartin-Dillon, docteurs-médecins, naturalistes du Muséum, Vignaud, dessinateur. Publié par ordre du gouvernement sous les auspices de M. Le ministre de la marine. Quatrième partie. Histoire naturelle. – Zoologie par MM. O. des Murs, Florent Prévost, Guichenot et Guérin-Menneville. Tome sixième.* Arthus Bertrand, Paris. 398 pp. [+ 40 associated pls]. (8vo–text; Folio–plates) McG, CNC (mf), GB, BHL

This volume constitutes livraisons 9 and 10 of the expedition report. There is no date on the book but the insect section was recorded in the notice of entomological works for 1849 by Schaum (1850: 149–150). There is no indication as to the contribution of each of the authors listed on the title page. However Guérin-Méneville, being the only entomologist of the group of four, is assumed to be the author of the section “*Insectes*.” The plates do not have scientific names.

The entire expedition report consists of six volumes of text and three atlases of 202 plates (72 colored) issued in 46 livraisons (12 of text and 34 of plates), 1845–1851. It was divided into four parts: *Relation historique* (volumes 1 and 2 + one atlas of 59 plates); *Itinéraire; description et dictionnaire géographiques; physique et métérologie; statistique; ethnologie; linguistique; archéologie* (volume 3); *Botanique* (volumes 4 and 5 + one atlas of 103 plates); *Zoologie* (volume 6 + one atlas of 40 plates).

#### Gundlach, Johannes [Juan] Christopher [Cristóbal] (Marburg, Hesse, Germany: 17 July 1810 – 17 March 1896: Havanna, Cuba). Cuban (German-born) zoologist; moved to Cuba in 1839 where he spent almost all his time collecting; established in 1846 a museum for his collections at a farm called “El Refugio”; made three visits to Puerto Rico between 1873 and 1881; sold his collections in 1892 to the Instituto de Segunda Enseñanza de la Habana and was allowed a small salary as curator; never married and lived frugally. References. Ackermann (1896); Vilaró (1897); Ramsden (1915, P); Papavero (1971: 181–183); Adler (1989: 53, P); Frank (2008: 1754–1755).

[**1896**]. *Contribucion a la entomologia Cubana. Parte Quinta. Coleopteros.* Habana. 404 pp. (4to) [n.d. (title page)] ANSP

The sole copy I saw of this book had no publication date but the date of 1891 is generally attributed to it (e.g., Blackwelder 1957: 1106; Derksen and Scheiding-Göllner 1965: 236; Peck 2005: 7). However, as indicated on the wrappers of the issues of the *Anales de la Real Academia de Ciencias Medicas, fisicas y naturales de La Habana (Revista Cientifica)* for the years 1892 to 1896 (tomes 29–32), this work was originally published under the title “*Contribucíon al estudio de los Coléopteros de la isla de Cuba*” as separate page gatherings (“*pliego aparte*”) to the journal. Unfortunately, no information was found to fix the dates of publication of the various parts.

The entire work consists of three volumes, 1881–[1896]. Only the third one deals with Coleoptera.

#### Gutfleisch, Valentin (Fürth, Hesse, Germany: 27 February 1809 – 24 December 1855: Darmstadt, Hesse, Germany). German forester.

**1859**. [Gutfleisch, V. and Bose, F.C.] *Die Käfer Deutschlands. Nach des Verfassers Tode vervollständigt und herausgegeben von Dr. Fr. Chr. Bose.* Joh. Phil. Diehl, Darmstadt. xvi + 661 + [3] pp. (8vo) [DP: 1859 (title page; *Vorrede* dated February 1859), 21 April 1859 (*Allg Bibl Deutsch*), 31 May 1859 (*Intell Serapeum*), May 1859 (*Allg Bibl*), January–June 1859 (*Bibliot Hist-Nat*)] CMLE, GB, MDZ

#### Gyllenhal, Leonhard (Algustorp, Westgothland, Sweden: 3 December 1752 – 13 May 1840: Hoberg, Jämtland, Sweden). Swedish military officer and entomologist; retired from military service in 1799 after 27 years with the rank of major; built a private entomological museum in Hoberg which he called the “Fly house;” at the time of his death, had a collection of 400 boxes of prepared and identified insects, which he left to the Royal Swedish Society of Sciences in Uppsala (later deposited at the Uppsala University Zoological Museum); his main publication, *Insecta Suecica*, was awarded the gold medal of the Academy of Sciences. References. Anonymous (1842); Ratzeburg (1874: 216–217); Essig (1931: 642–643, P); Smetana and Herman (2001: 73–74, P).

**1808–1827**. *Insecta Svecica. Classis I. Coleoptera sive Eleuterata.* F.J. Leverentz, Scaris [= Skara] [vols 1–3] / Friedericum Fleischer, Lipsiae [= Leipzig] [vol. 4]. (8vo) CNC, GB, BHL

*Tomus I.* viii + [4 (Auctores citati)] + 572 pp. [DP: 1808 (title page; *praefatio* dated 27 April 1808)].

*Tomi I Pars II.* xix [Addenda] + [1 (Auctores citati)] + 660 pp. [DP: 1810 (title page)].

*Tomi I, Pars III.* [2 (Auctores citati)] + 730 + [2 (Errata corrigenda)] pp. [DP: 1813 (title page)].

*Tom. I. Pars IV. Cum appendice ad partes priores.* viii + [2 (Auctores citati)] + 761 + [1 (Emendanda)] pp. [DP: 1827 (title page; *praefatio* dated 31 January 1827), 6 May 1827^42^, January–June 1827 (*Verz Bücher Landkarten*), July–October 1827 (*Foreign Quart Rev*)].

Gaedike and Smetana (1978: 407) mentioned an “editio nova” of parts 1–3 (*n.v.*), with the same number of pages, published in Scaris and Hafniae [= Skara and Copenhagen] by the publisher G. Bonnier in 1820.

#### Hagenbach, Johann Jacob (Basel, Switzerland: 1801 – 1 September 1825: Basel, Switzerland). Swiss entomologist; curator at the Rijksmuseum in Leiden; died two days after his return to Basel from an expedition to Java. Reference. Anonymous (1827: 1511).

**1822**. *Symbola faunae insectorum Helvetiae exhibentia vel species novas vel nondum depictas. Fasciculus primus. Cum tabulis 15 color. ad viv. expressis.* J. Georgii Neukirch, Basileae [= Basel]. vi + 7–48 pp. + [15] pls. (8vo) [DP: 1822 (title page), January–June 1823 (*Verz Bücher Landkarten*), September 1823 (*Jena Lit Ztg*)] BHL, CNC (mf)

Only the first fascicle of this work was published. The new Coleoptera described are: *Leptura erythroptera* (p. 7; fig. 1 on pl. [1]), *Cassida angusticollis* (p. 8; fig. 2 on pl. [1]), *Saperda ustulata* (p. 11; fig. 4 on pl. [2]), and *Clytus koechlini* (p. 12; fig. 5 on pl. [3]).

**1825**. Mormolyce *novum coleopterorum genus.* Jac. Sturm, Norimbergae [= Nürnberg]. 8 pp. + 1 pl. (8vo) [DP: 1825 (title page), July 1825 (*Isis*, Heft VII: [2]), 14 April 1826 (*Alg Konst Letter*)] MCZ

The lengthly latin description of the genus and species were also issued, under the title “*Description du Mormolyce, nouveau genre d’insecte dans l’ordre des coléoptères*,” in the *Annales des Sciences Naturelles* 6 [1825]: 500–503 + pl. 21.

#### Hahn, Carl Wilhelm (Weingartsgreuth, Bavaria, Germany: 16 December 1786 – 7 October 1835: Nuremberg, Bavaria, Germany). German illustrator and zoologist known mostly for his work on spiders and Heteroptera; served in the army as quarter master (1813–1816). Reference. Sacher (1988: 146).

**1834**. *Gründliche Anweisung Krustenthiere, Vielfüsse, Asseln, Arachniden und Insecten aller Klassen zu sammeln, zu präpariren, aufzubewahren und zu versenden; nach mehr als zwanzigjähriger Erfahrung und eigener Ausübung für Sammler und Liebhaber.* E.H. Zeh, Nürnberg. vi + 154 pp. + 4 pls^43^. (8vo) [DP: 1834 (title page; *Vorwort* dated September 1834), November 1834 (*Jena Lit Ztg*), July–December 1834 (*Verz Bücher Landkarten*), 14 January 1835 (*Lit Ztg*)] CNC, GB, MDZ

The Coleoptera are on pages 56–99. A “*Zweite Ausgabe*” was issued by Lotzbeck in Nürnberg, 1854 [GB]; it is essentially identical to the 1834 printing except for the *Vorwort*.

#### Halbherr, Bernardino (Rovereto, Trentino-Alto Adige, Italy: 21 July 1844 – 31 March 1934: Rovereto, Italy). Italian entomologist; conservator at the Museo Civico di Rovereto; left his collection to the museum. References. Bonomi (1934, P); Heikertinger (1935).

**1885–1898**. *Elenco sistematico dei coleotteri finora raccolti nella Valle Lagarina*. Tipografia Roveretana, Rovereto. (8vo) CNC

*Fascicolo I. Cicindelidae–Carabidae.* 45 pp. [DP: 1885 (title page; preliminaries dated September 1885), 31 January 1886 (*Bibl Ital-2*), January 1886 (*Nat Nov*), 20 February 1886 (*Wien Ent Ztg* 5: 80), January–June 1886 (*Bibliot Hist-Nat*)].

*Fascicolo II. Haliplidae–Dyticidae–Gyrinidae–Hydrophilidae–Sphaeridiidae–Limnichidae–Dryopidae–Georyssidae–Heteroceridae.* 23 pp. [DP: 1887 (title page; second page dated February 1887), 20 June 1887 (*Wien Ent Ztg* 6: 176), July 1887 (*Nat Nov*), 30 September 1887 (*Bibl Ital-2*), October–December 1887 (*Bibliot Hist-Nat*)].

*Fascicolo III. Staphylinidae.* 53 pp. [DP: 1888 (title page; second page dated May 1888), 31 July 1888 (*Wien Ent Ztg* 7: 227), 15 August 1888 (*Bibl Ital-2*), October 1888 (*Nat Nov*)].

*Fascicolo IV. Pselaphidae inclusivo Histeridae.* 60 pp. [DP: 1890 (title page), 15 July 1890 (*Bibl Ital-2*), July 1890 (*Nat Nov*), September 1890 (*Polybiblion*)].

*Fascicolo V. Platyceridae–Scarabaeidae.* 35 pp. [DP: 1892 (title page; page 5 dated January 1892), 31 May 1892 (*Bibl Ital-2*), July 1892 (*Nat Nov*)].

*Fascicolo VI. Buprestidae–Eucnemidae–Elateridae–Dascillidae–Cantharidae.* 40 pp. [DP: 1894 (title page; first page dated November 1893), 31 October 1894 (*Wien Ent Ztg* 13: 260), 30 November 1894 (*Bibl Ital-2*), November 1894 (*Nat Nov*)].

*Fascicolo VII. Cleridae inclusivo Pythidae.* 40 pp. [DP: 1894 (title page; first page dated November 1894), 30 June 1895 (*Bibl Ital-2*), June 1895 (*Nat Nov*), November 1895 (*Monit Zool Ital*)].

*Fascicolo VIII. Curculionidae.* 64 pp. [DP: 1896 (title page; second page dated December 1895), 31 March 1896 (*Wien Ent Ztg* 15: 124), 15 July 1896 (*Bibl Ital-2*), July 1896 (*Nat Nov*), 19 October 1896 (*Bibl Zool*)].

*Fascicolo IX. Nemonychidae–Anthribidae–Mylabridae–Scolytidae–Cerambycidae.* 33 pp. [DP: 1896 (title page; second page dated November 1896), 28 February 1897 (*Wien Ent Ztg* 16: 80), 31 July 1897 (*Bibl Ital-2*), August 1897 (*Nat Nov*)].

*Fascicolo X. Chrysomelidae–Coccinellidae. Epilogo–Bibliografia–Indice alfabetico delle famiglie, dei generi e sottogeneri.* 79 pp. [DP: 1898 (title page; page 5 dated March 1898), 30 November 1898 (*Bibl Ital-2*), December 1898 (*Nat Nov*)].

Two supplements to this work were issued in 1908 and 1931 in Rovereto.

#### Haldeman, Samuel Steman (Locust Grove, Pennsylvania, USA: 12 August 1812 – 20 November 1880: near Harrisburg, Pennsylvania, USA). American scientist, naturalist, teacher, and linguist; professor of zoology at Franklin Institute in Philadelphia (1842–1843), of natural history at the University of Pennsylvania (1851–1855), of chemistry and geology at the University of Delaware (1855–1869), and of philology at the University of Pennsylvania (1869–1880); founding member of the Entomological Society of Pennsylvania; his types of Coleoptera were given to John L. LeConte and are now in the Museum of Comparative Zoology mixed with LeConte own specimens while his general collection was bought by Simon Snyder Rathvon and is now at the Franklin and Marshall College in Lancaster, Pennsylvania. References. Hart (1881); Lesly (1886); Mallis (1971: 33–36, P); Marché (2001).

**1852**. Appendix C. Insects. Pp. 366–378 + pls 9–10 *in*: *Exploration and survey of the valley of the Great Salt Lake of Utah, including a reconnoissance of a new route through the Rocky Mountains. By Howard Stansbury. Printed by order of the Senate of the United States.* Lippincott, Grambo & Co., Philadelphia. 487 pp. + 57 pls. (8vo) [DP: 1852 (title page; submission letter at the beginning dated 19 April 1952), 3 August 1852 (*Acad Nat Sci Phila*), September 1852 (*Amer J Sci Arts*)] GB, ARC, BHL

The title given is that of the official and first issue. The top of the title page reads “*Special session, March, 1851. Senate. Executive. No. 3.*” Two trade issues were also printed in 1852, shortly after the official issue, under the title “*An expedition to the valley of the Great Salt Lake of Utah: including a description of its geography, natural history, and minerals, and an analysis of its waters; with an authentic account of the Mormon settlement. Illustrated by numerous beautiful plates, from drawings taken on the spot. Also, a reconnoissance of a new route through the Rocky Mountains, and two large and accurate maps of that region*” by Lippincott, Grambo & Co. [GB], one of them with the addition of “Sampson Low Son & Co., London” representing the distributor of the work in England. All three issues are identical except for their title-pages (Dorr et al. 2003: 319). A second, revised edition was published in 1853 [GB] by Robert Amstrong, Washington, with the same title as the official issue except for “*Printed by order of House of Representatives of the United States*.” It contains 495 pages and 57 plates and according to Coville (1896: 138) there are changes in the text from the first printing in the appendices, some of which have nomenclatural incidences. A reissue by Lippincott, Grambo & Co. of the trade issue of 1852 was released in 1855 [GB]. The new title page differs solely in the change of the date from 1852 to 1855. A reprint of the official issue was released in 1988 by the Smithsonian Institution Press with an introduction by D.D. Fowler.

The new Coleoptera taxa described in this work are: *Panagaeus distinctus* (p. 373), *Carabus finitimus* (p. 373), *Cotalpa granicollis* (p. 374; pl. 9, fig. 11), *Euphoria cernii* (p. 374; pl. 9, fig. 10), *Hydrochus foveatus* (p. 375), *Philonthus comptus* (p. 375), *Eleodes cognata* (p. 376), Nyctobates (Iphthinus) intermedia (p. 376), *Horia stansburii* (p. 377), *Meloe parvus* (p. 377), *Henous* (p. 377) and *Henous techanus* (p. 377; pl. 9, figs 12–14).

#### Hamann, Otto (Leipzig, Saxony, Germany: 28 November 1857 – 3 May 1925: Berlin, Germany). German zoologist; professor of zoology and comparative anatomy in Göttingen; later, librarian in Berlin. References. Wagenitz (1988: 72); Anonymous (2006).

**1896**. *Europäische Höhlenfauna. Eine Darstellung der in den Höhlen Europas lebenden Tierwelt mit besonderer Berücksichtigung der Höhlenfauna Krains. Nach eigenen Untersuchungen. Mit 150 Abbildungen auf fünf lithographischen Tafeln.* Hermann Costenoble, Jena. xiii + 296 pp. + 5 pls. (8vo) [DP: 1896 (title page; *Vorwort* dated June 1896), 2 November 1896 (*Zool Centr*), 23 November 1896 (*Bibl Zool*), November 1896 (*Nat Nov*)] GB

The Coleoptera are on pages 53–141.

#### Hampe, Clemens (Luschwitz, Czech Republic: 1802 – 20 July 1884: Vienna, Austria). Austrian orthodox physician in Vienna and entomologist; practiced homeopathy; personal physician to the Prince of Liechtenstein; his collection of Coleoptera is at the Naturhistorisches Museum in Vienna. References. Reitter (1885, P); Schroers (2006: 51).

**1852**. Verzeichniss der von mir im Kaukasus, in Trans-Kaukasien, Armenien, Kurdistan und West-Persien gesammelten und von Dr. Hampe bestimmten Koleopteren. Pp. 302–315 *in*: *Reise nach Persien und dem Lande der Kurden. Von Moritz Wagner. Zweiter Band. Mit einem Anhang: Beiträge zur Völkerkunde und Naturgeschichte West-Asiens.* Arnold, Leipzig. iv + 315 + [1 (Druckfehlerverzeichniss)] pp. (8vo) [DP: 1852 (title page), 19 February 1852 (*Allg Bibl Deutsch*), 29 February 1852 (*Intell Serapeum*), January–June 1852 (*Bibliot Hist-Nat*)] GB, ARC

The expedition report consists of two volumes issued in 1852. The second volume includes an appendix on the history, natural history and ethnology of Armenia, Kurdistan and western Persia. A Swedish translation by Fr. T. Borg in two volumes was published in 1853 in Örebro under the title “*Resa till Persien och Kurdernas land*” (*n.v.*).

#### Harrer, Georg Albrecht (Regensburg, Bavaria, Germany: 12 February 1753 – 4 November 1822: Regensburg, Germany). Alderman in Regensburg. References. Fürnrohr (1838: 51); Pongratz (1963: 53).

**1784**. *Beschreibung derjenigen Insecten welche Herr D. Jacob Christoph Schäffer in CCLXXX ausgemahlten Kupfertafeln unter dem Titel Icones insectorum circa Ratisbonam indigenorum ehemals in drey Theilen herausgegeben hat. I. Theil Hartschaalige Insecten.* Keyser, Regensburg. [30] + 328 pp. (8vo) [DP: 1784 (title page; preliminaries dated 15 April 1784), 14 September 1784 (*Nürnb Ztg*), 20 September 1784 (*Gött Anz*)] NCSU, GB

This work provided specific names and descriptions for the unnamed insects illustrated on the plates of Schäffer’s “*Icones insectorvm circa Ratisbonam indigenorvm coloribvs natvram referentibvs expressae*,” 1766–1779 [*q.v.*].

The Coleoptera in the large sense are on pages 1–242, 244–246, 249–253. No more *Theil* was published.

**1791**. *Beschreibvngen zv des Herrn D. Iacob Christian Schaeffers natürlich avsgemahlten Abbildvngen regensbvrgischer Insecten. Erster Band.* Montag u. Weiß, Regensburg. [3] + xii + 144 pp. (4to) [DP: 1791 (title page; *Vorbericht* dated 12 April 1791), 31 October 1791 (*Gött Anz*)] GB, MDZ

This volume pertains to Coleoptera only. No other part was published.

#### Harris, Thaddeus William (Dorchester, Massachusetts, USA: 12 November 1795 – 16 January 1856: Cambridge, Massachusetts, USA). American physician, librarian, botanist and entomologist, mostly known for his work on economic entomology; graduated from Harvard University with a medical degree at the age of 25 but his physician practice lasted for only about a decade; accepted the position of librarian at Harvard University where he also taught natural science courses; his collection was bought by friends and presented to the Boston Society of Natural History in 1858 and was subsequently transferred to the Museum of Comparative Zoology in 1941. References. Harris (1882); Mallis (1971: 25–33, P); Elliott (1997; 2008, P).

**1833**. VIII.–Insects. Pp. 566–595 *in*: *Report on the geology, mineralogy, botany, and zoology of Massachusetts. Made and published by order of the government of that state: in four parts: Part I. Economical geology. Part II. Topographical geology. Part III. Scientific geology. Part IV. Catalogues of animals and plants. With a descriptive list of the specimens of rocks and minerals collected for the government. Illustrated by numerous wood cuts and an atlas of plates. By Edward Hitchcock.* J.S. and C. Adams, Amherst. xii + 692 pp. (8vo) [DP: 1833 (title page; p. [543] dated 1 October 1833), January 1834 (*Amer J Sci Arts*; *Amer Quart Obs* 2: 182), 8 March 1834 (*Lit J*), April 1834 (*North Amer Rev*)] GB, BHL

Harris’ contribution consists of a list of insects collected in Massachusetts. The Coleoptera are on pages 566–582. A few genus-group names are introduced for the first time, one of which, *Megalamera* (p. 581), is made available by the inclusion of one available species (*Chrysomela rhois* Forster) under it.

A second edition of this work was issued in 1835 (see next entry).

**1835**. VIII.–Insects. Pp. 553–602 *in*: *Report on the geology, mineralogy, botany, and zoology of Massachusetts. Made and published by order of the government of that state: in four parts: Part I. Economical geology. Part II. Topographical geology. Part III. Scientific geology. Part IV. Catalogues of animals and plants. With a descriptive list of the specimens of rocks and minerals collected for the government. Illustrated by numerous wood cuts and an atlas of plates. By Edward Hitchcock, A.M. Second edition, corrected and enlarged.* J.S. and C. Adams, Amherst. [1] + xii + 13–702 pp. (8vo) [DP: 1835 (title page), April 1836 (*North Amer Rev*)] BHL

The Coleoptera are on pages 553–575. A few new genus-group names of beetles are introduced for the first time, such as *Tapheicerus* (p. 558) and *Nothora* (p. 559), and made available by the inclusion of one or more available species under them.

Part IV of this work was also issued the same year, by the same publisher, under the title “*Catalogues of the animals and plants of Massachusetts. With a copious index*” [GB]. Harris’ contribution is on pages 33–82.

**1841**. *A report on the insects of Massachusetts, injurious to vegetation. Published agreeably to an order of the legislature, by the Commissioners on the Zoological and Botanical Survey of the state.* Folsom, Wells, and Thurston, Cambridge. viii + 459 pp. (8vo) [DP^44^: 1841 (title page; preliminaries dated 1 December 1841), January 1842 (*North Amer Rev*), 16 March 1842 (*Boston Soc Nat Hist*), 26 March 1842 (*Mag Lit Ausl*), January–March 1842 (*Amer J Sci Arts*), 9 May 1842 (*Nat Inst Sci*)] CMLE, GB, BHL

The work was reissued in 1842 by John Owen in Cambridge under the title “*A treatise on some of the insects of New England, which are injurious to vegetation*” [MCZ, GB]. A second edition was published, 1852 by White & Potter in Boston, under the title “*A treatise on some of the insects of New England which are injurious to vegetation. Second edition*” [CMLE, BHL, GB] and a third one, 1862 by William White in Boston, under the title “*A treatise on some of the insects injurious to vegetation. Third edition.*” [BHL]. Several printings of this edition are known (Evenhuis 1997a: 345).

#### Hart, Henry Chichester (Clontarf, Ireland: 29 July 1847 – 7 August 1908: Portsalon, Ireland). Irish explorer, ornithologist, botanist, etymologist, and Shakespearean scholar; naturalist to the HMS Discovery on the Polar Expedition under the command of Sir George Strong Nares (1875–1876); naturalist on the scientific expedition to Arabia and western Palestine sponsored by the Committee of the Palestine Exploration Fund (1883–1884). References. Barrington (1908); Higgins (1997).

**1891**. *Some account of the fauna and flora of Sinai, Petra, and Wâdy ’Arabah*. Published for the Committee of the Palestine Exploration Fund by Alexander P. Watt, London. x + 255 pp. + 10 pls. (4to) [DP: 1891 (title page), July 1891 (*Pal Expl Fund*)] ANXL, GB

The only new species of Coleoptera described in this book is *Galbella harti* O. Janson (p. 184, plate 10, figure 1). On page 176, the author mentioned that “in the following list [pp. 176–183], except where otherwise stated, the species have been determined by Mr. Oliver Janson.”

#### Hartig, Georg Ludwig (Gladenbach, Hesse, Germany: 2 September 1764 – 2 February 1837: Berlin, Germany). German forester; manager of forests at Hungen and afterwards at Dillenburg; chief inspector of forests in Stuttgart and later in Berlin; founded a school of forestry. References. Anonymous (1839: 761–762); Hartig et al. (1976, P).

**1834**. [Hartig, G. L. and Hartig, T.] *Forstliches und forstnaturwissenschaftliches Conversations-Lexicon. Ein Handbuch für Jeden, der sich für das Forstwesen und die dazu gehörigen Naturwissenschaften interessirt. Mit allerhöchsten Privilegien gegen den Nachdruck und den Verkauf desselben.* Nauck, Berlin. xiv + 1034 + [2 (Verbesserungen)] pp. (8vo) [DP: 1834 (title page; *Vorrede* dated 1 July 1834), July–December 1834 (*Verz Bücher Landkarten*), 4 March 1835 (*Lit Ztg*)] GB

Two new species of Coleoptera, *Bostrichus quadridens* (p. 109) and *Hylesinus minor* (p. 413), are described in this book. A “*zweite, revidirte Auflage*” with the same number of pages was issued in 1836 by J. G. Cotta in Stuttgart and Tübingen [GB, MDZ].

#### Hasselquist, Frederik (Tornevalla, Östergötland, Sweden: 14 January 1722 – 9 February 1752: Smyrna, Turkey). Swedish naturalist; pupil of Linnaeus; explored Egypt, Arabia and Palestine; his valuable journal, observations, and descriptions were published by Linnaeus. References. Båck (1758); Jourdan (1822); Swainson (1840: 211–213); Hansen et al. (2012: 31–33).

**1762**. *D. Friedrich Hasselquists Reise nach Palästina in den Jahren von 1749 bis 1752. Auf Behehl Ihro Majestät der Königinn von Schweden herausgegeben von Carl Linnäus. Aus dem Schwedischen.* Johann Christian Koppe, Rostock. [10] + 606 pp. (8vo) [DP: 1762 (title page), July 1762 (*J Étrang*; Evenhuis 1997a: 348), 15 August 1862 (*J Encycl*)] GB, ARC

The colophon on the last page reads “Leipzig, gedruckt den Joh. Gottl. Immanuel Breitkopf. 1761” and may be the reason why the date of 1761 is given to this publication in some bibliographies. This work contains descriptions of new species, including the beetles *Curculio cypri* (p. 449), *Chrysomela cichorii* (p. 449–450) and *Cerambyx smirnensis* (p. 450–451), attributed to Linnaeus since it is stated in the preface of the work that Linnaeus was responsible for the taxonomy and nomenclature. However, these names are not available for nomenclatural purposes because the work has been placed on the *Official Index of Rejected and Invalid Works in Zoological Nomenclature* (Direction 32, ICZN 1956a).

This work is a German translation by Th. H. Gadebusch of “*Frederic Hasselquists M.D. Iter Palaestinum, eller resa til Heliga Landet, förrättad ifrån år 1749 til 1752, med beskrifningar, rön, anmärkningar, öfver de märkvärdigaste naturalier, på Hennes Kongl. Maj:ts befallning, utgifven af Carl Linnaeus*” published in Holmiae [= Stockholm] in May 1757 [GB]. The original edition is also not available because it was published before the starting point of zoological nomenclature. It was placed on the *Official Index of Rejected and Invalid Works in Zoological Nomenclature* (Direction 32, ICZN 1956a).

An abridged English translation of Hasselquist’s work was published in London, 1766, under the title “*Voyages and travels in the Levant; in the years 1749, 50, 51, 52. Containing observations in natural history, physick, agriculture, and commerce: particularly on the Holy Land, and the natural history of the scriptures. Written originally in the Swedish language, by the late Frederick Hasselquist, M.D. Published, by Order of her present Majesty the Queen of Sweden by Charles Linnaeus*” [GB]. This edition has not been placed on the *Official Index of Rejected and Invalid Works in Zoological Nomenclature* and so the new species are available from this book. An abridged French translation was issued in Paris, 1769, under the title “Voyages dans le Levant, dans les années 1749, 50, 51 & 52. Contenant des observations sur l’histoire naturelle, la médecine, l’agriculture & le commerce, & particulierement sur l’histoire naturelle de la Terre Sainte. Par Frédéric Hasselquist. Publiés par ordre du Roi de Suéde, par Charles Linnaeus. Traduits de l’Allemand par M.***” [GB].

#### Hausmann, Johann Friedrich Ludwig (Hannover, Lower Saxony, Germany: 22 February 1782 – 26 December 1859: Göttingen, Lower Saxony, Germany). German mineralogist; professor of technology, mineralogy and geology at the University of Göttingen; secretary of the Royal Academy of Sciences of Göttingen for many years. References. Saalfeld (1820: 363–370); Meyer (1858: 463–464); Anonymous (1910c); Böhm (1997: 102–103).

**1799**. *Entomologische Bemerkungen. I. Heft.* Braunschweig. iv + 64 pp. (8vo) [DP: 1799 (title page), 6 July 1799 (*Intell Allg Lit Ztg*)] MCZ, MDZ, GB

This work was published anonymously. It was attributed to Johann Friedrich Ludwig Hausmann by Saalfeld (1820: 365), Carus and Engelmann (1861: 486), and Hagen (1862: 349). However, Evenhuis (1997a: 348–349) argued that the book was written by Friedrich Karl Hausmann [1767–1822], not J.F.L. Hausmann because he would have been only 16 years old at the time if he was the author.

#### Heer, Oswald (Niederuzwil, Switzerland: 31 August 1809 – 27 September 1883: Lausanne, Switzerland). Swiss theologian, paleontologist and entomologist; private docent of botany and entomology and later professor of botany at the University of Zürich; founded the Botanical Garden in Zürich; his collection is at the Eidgenossische Technische Hochschule-Zentrum in Zürich. References. Schröter (1883); Gray (1884); Jentzsch (1885); Taylor (1886); Zeiller (1887, P); Smetana and Herman (2001: 76–77, P).

**1836**. *Observationes entomologicae continentes metamorphoses coleopterorum nonnullorum adhuc incognitas. Cum Tab. aeneis VI.* Orellii, Fuesslini & Sociorum, Turici [= Zürich]; Mueller & Socios ac S. Suelpke, Amstelodami [= Amsterdam]; Black & Armstrong, Londini. [1] + 36 pp. + 6 pls. (8vo) [DP: 1836 (title page), 17 June 1836 (*Allg Bibl Deutsch*), 22 June 1836 (*Lit Ztg*), January–June 1836 (*Verz Bücher Landkarten*), November 1836 (*Bull Litt Étr*)] GB, MDZ

**1837–1841**. *Die Kaefer der Schweiz, mit besonderer Berücksichtigung ihrer geographischen Verbreitung. Als dritter Theil der auf Veranstaltung der allgemeinen schweizerischen Gesellschaft fur die gesammten Naturwissenschaften entworfenen Fauna Helvetica.* Petitpierre, Neuchatel. (4to) SME, GB

*Erster Theil. Erste Lieferung. Aus dem zweiten Bande der Neue Denkenschriften der allgemeinen schweizerischen Gesellschaft für die gesammten Naturwissenschaften besonders abgedruckt.* vi + 96 pp. [DP: 1837 (title page; *Vorwort* dated 10 December 1837), May 1838 (*Isis*, Heft V: [3])].

*Zweiter Theil. Erste Lieferung. Aus dem zweiten Bande der Neue Denkenschriften der allgemeinen schweizerischen Gesellschaft für die gesammten Naturwissenschaften besonders abgedruckt.* 55 pp. [DP: 1837 (title page)]. The title of this part is “*Die Kaefer der Schweiz, kritische Bemerkungen und Beschreibungen der neuen Arten. Als dritter Theil der auf Veranstaltung der allgemeinen schweizerischen Gesellschaft fur die gesammten Naturwissenschaften entworfenen Fauna Helvetica.* ”

*Erster Theil. Zweite Lieferung. Aus dem dritten Bande der Neue Denkenschriften der allgemeinen schweizerischen Gesellschaft für die gesammten Naturwissenschaften besonders abgedruckt.* 67 pp. [DP: 1839 (title page), 14 September 1840 (*Ent Ver Stettin*), 31 March 1841 (*Intell Serapeum*), January–June 1841 (*Verz Bücher Landkarten*)].

*Erster Theil. Dritte Lieferung. Aus dem fünften Bande der Neue Denkenschriften der allgemeinen schweizerischen Gesellschaft für die gesammten Naturwissenschaften besonders abgedruckt.* 79 pp. [DP: 1841 (title page), 6 September 1841 (*Ent Ver Stettin*), 31 December 1841 (*Allg Bibl Deutsch*; *Intell Serapeum*), January–June 1842 (*Verz Bücher Landkarten*)].

These works were published also under the same titles in *Neue Denkschriften der allg. schweizerischen Gesellschaft für die gesammten Naturwissenschaften* [*Nouveaux Mémoires de la Société Helvétique des Sciences Naturelles*], volumes 2 [DP: 1838 (title page)], 4 [DP: 1840 (title page), 2 July 1841 (*Allg Press Ztg*)], and 5 [DP: 1841 (title page), January–June 1842 (*Verz Bücher Landkarten*)].

**1838–1841**. *Fauna coleopterorum Helvetica. Pars I.* Orelii, Fuesslini et Sociorum, Turici [= Zürich]. xii + 652 pp. (12 mo) CNC, GB

*Fasciculus primus.* xii + pp. 1–144. [DP: 1838 (title page, preface dated 19 July 1838), 1 October 1838 (*Allg Ztg*), 12 October 1838 (*Allg Bibl Deutsch*), 24 October 1838 (*Lit Ztg*), October–December 1838 (*Foreign Quart Rev*), July–December 1838 (*Verz Bücher Landkarten*)].

*Fasciculus secundus.* Pp. 145–360. [DP: 1839 (title page), 17 April 1840 (*Allg Bibl Deutsch*), 22 April 1840 (*Lit Ztg*), 23 April 1840 (*Bibl Blätt*), 30 April 1840 (*Intell Serapeum*), April–June 1840 (*Foreign Quart Rev*)].

*Fasciculus tertius et ultimus.* Pp. 361–652. [DP: 1841 (title page), 15 September 1841 (*Lit Ztg*), 17 September 1841 (*Allg Bibl Deutsch*), 1 November 1841 (*Not Geb Pharm*), October–December 1841 (*Foreign Quart Rev*), July–December 1841 (*Verz Bücher Landkarten*)].

**1847**. *Die Insektenfauna der Tertiärgebilde von Oeningen und von Radoboj in Croatien. Erster Theil: Käfer.* Wilhelm Engelmann, Leipzig. 229 + [1 (Index der Gattungen)] pp. + 8 pls. (4to) [DP: 1847 (title page), 14 July 1847 (*Lit Ztg*), 22 July 1847 (*Allg Bibl Deutsch*), July 1847 (*Intell Allg Lit Ztg*), 24 September 1847 (*Leip Reper*), 27 November 1847 (*Gött Anz*)] GB, BHL

The entire work contains three parts, 1847–1853. Only the first one pertains to Coleoptera. The work was also published in volume 8 [DP: 1847 (title page)] of the *Neue Denkenschriften der allg. schweizerischen Gesellschaft für die gesammten Naturwissenschaften*.

**1852**. Die Lias-Insel des Aargau’s. Pp. 1–15 + 1 pl. *in*: *Zwei geologische Vorträge gehalten im März 1852 von Oswald Heer und A. Escher von der Linth*. *1. Ueber die Lias-Insel im Aargau, mit einer Tafel Lias-Insekten. 2. Ueber die Gegend von Zürich in der letzten Periode der Vorwelt, mit einer Blockkarte der Schweiz.* E. Kiesling, Zürich. 28 pp. + 2 pls. (4to) [DP: n.d. (title page), 15 May 1852 (*Naturhist Ver Rhein West*), 30 August 1852 (*Börsen Deutsch Buch*), 2 September 1852 (*Allg Bibl Deutsch*), 16 October 1852 (*Lit Centr Deutsch*)] GB

Heer’s section contains the descriptions of several new species of Liassic Coleoptera (pp. 11–15).

**1860**. Ueber die fossilen Calosomen. Pp. i–x + 1 pl. *in*: *Programm der eidgen. polytechnischen Schule für das Schuljahr 1860/61 beziehungsweise das erste Halbjahr (vom 15. Oct. 1860 bis 24. März 1861).* Orell, Füssli & Comp., Zürich. 15 + [1] + x pp. + 1 pl. (4to) [DP: 1860 (title page)] BHL

Heer’s section contains the descriptions of several new species of fossil *Calosoma* (Carabidae).

**1863–1865**. *Die Urwelt der Schweiz. Mit sieben landschaftlichen Bildern, elf Tafeln, einer geologischen Uebersichtskarte der Schweiz und zahlreichen in den Text eingedruckten Abbildungen.* Friedrich Schultheß, Zürich. xxix + 622 pp. + 11 pls. (8vo) MCZ, GB

This work was issued in 13 *Lieferungen*: **1**: (pp. 1–48 + pls 1–3) 10 December 1863 (*Allg Bibl Deutsch*), July–December 1863 (*Bibliot Geo-Stat*); **2**: (pp. 49–96 + pls 4–6) 24 March 1864 (*Allg Bibl Deutsch*); **3–4**: (pp. 97–192 + pl. 7) 30 March 1864 (Evenhuis 1997a: 352); **5–6**: (pp. 193–288 + pl. 8) 31 March–17 August 1864; **7–11**: (pp. 289–496 + pls 9–10) 18 August 1864 (Evenhuis 1997a: 352), 29 September 1864 (*Allg Bibl Deutsch*); **12–13**: (pp. 497–622, i–xxix + pl. 11) 23 February1865 (*Allg Bibl Deutsch*). The title page is dated 1865. The Coleoptera are on pages 86–91 and also discussed on pages 500–502; several genus-group and species-group taxa are made available through illustrations on plates 7–8.

A second edition, containing xix + 713 pages + 12 plates, was issued in 1879 and 1883 in Zürich under the title “*Die Urwelt der Schweiz. Mit acht landschaftlichen Bildern in Tondruck, zwölf fein gravirten Tafeln, einer geologischen Uebersichtskarte der Schweiz in Farbendruck und zahlreichen in den Text eingedruckten Abbildungen in Holzschnitt*. *Zweite Subscriptions-Ausgabe der zweiten, umgearbeiteten und vermehrten Auflage*” [GB]. An English translation by W.S. Dallas was published, 1876, in two volumes by Longmans, Green & Co., London, under the title “*The primaeval world of Switzerland. With 560 illustrations. By professor Heer. Edited by James Heywood*” [McG, GB]. A French translation was published in 1872 in Genève and Basel under the title “*Le monde primitif de la Suisse par Oswald Heer; traduit de l’Allemand par Isaac Demole*” [GB, GAL].

#### Hellwig, Johann Christian Ludwig (Garz on the island of Rügen, Germany: 8 November 1743 – 10 September 1831: Brunswick, Lower Saxony, Germany). German mathematician and entomologist; professor of philosophy at the University of Helmstadt; professor of mathematics and natural history at the Collegium Carolinum in Brunswick; his collection is at the Zoologisches Museum der Humboldt-Universität in Berlin. References. Poggendorff (1863: 1058); Spehr (1881); Koch (1969); Nohr (2008: 75–78, P).

**1795**. *Favna Etrvsca sistens insecta qvae in provinciis Florentina et Pisana praesertim collegit Petrvs Rossivs in regio Pisano Athenaeo Pvbl. Prof. et Soc. Ital. Tomvs primvs. Cvm XI Tab. Mantissae priore parte adiecta, itervm edita et annotatis perpetvis avcta a D. Ioh. Christ. Lvd. Hellwig.* C.G. Fleckeisen, Helmstadii. xxviii + 457 + [1(Corrigenda)] pp. + 11 pls. (8vo) [DP: 1795 (title page; *praefatio* dated April 1795), 25 July 1795 (*Intell Allg Lit Ztg*), 27 July 1795 (*Gött Anz*)] CMLE (mf), GB, BHL

The entire work consists of two volumes; the first one published in 1795 is almost entirely devoted to beetles. The second volume, published in 1807 and authored by Illiger, does not pertain to beetles. The work is essentially an expansion of Pietro Rossi’s first volume of *Fauna Etrusca*, 1790 [*q.v.*] and pages 5–103 of the first volume of *Mantissa insectorum*, 1792 [*q.v.*].

#### Henschel, Gustav (Zellhof, Upper Austria, Austria: 25 July 1835 – 17 March 1895: Gußwerk, Styria, Austria). Austrian forester; professor at the College of Agricultural Sciences in Vienna. References. Anonymous (1895a, P); Anonymous (1895b, P); Hess (1905).

**1895**. *Die schädlichen Forst- und Obstbaum-Insekten, ihre Lebensweise und Bekämpfung. Praktisches Handbuch für Forstwirthe und Gärtner. Dritte, neubearbeitete Auflage. Mit 197 Textabbildungen.* Paul Parey, Berlin. xii + 758 pp. (8vo) [DP: 1895 (title page), January 1895 (*Zool Anz*), February 1895 (*Nat Nov*), March 1895 (*Zeit Forst Jagd*)] ANNL, GB

The Coleoptera are on pages 33–228.

The first edition was published in 1861 in Wien by Wilhelm Braumüller under the title “*Leitfaden zur leichteren Bestimmung der schädlichen Forst-Insekten, mit Angabe ihrer Lebensweise, der gegen dieselben seither mit Erfolg angewendeten Vorbauungs- und Vertilgungsmittel, unter gleichzeitiger Berücksichtigung der den Obstbäumen schädlichen Arten. Für Forstleute, Oekonomen, Gärtner analytisch bearbeitet*” (*n.v.*); it contains xvi + 175 pages and was noticed on 5 December 1861 (*Allg Bibl Deutsch*). The second edition was issued in 1876 by the same publisher as for the first edition under the title “*Leitfaden zur Bestimmung der schädlichen Forst- und Obstbaum-Insekten nebst Angabe der Lebensweise, Vorbauung und Vertilgung. Für Forstleute, Ökonomen, Gärtner. Zweite vermehrte und verbesserte Auflage*” [GB]; it contains x + 269 pages and was noticed in March 1876 (*Centr Forst*).

#### Henshaw, Samuel (Boston, Massachusetts, USA: 29 January 1852 – 5 February 1941: Cambridge, Massachusetts, USA). American entomologist; technician (1876–1891) and then secretary and librarian (1892–1901) at Lowell Technological Institute; assistant to Hermann August Hagen at the Museum of Comparative Zoology in Cambridge, Massachusetts, and eventually director of the Museum (1911); editor of the journal *Psyche* for many years. References. Jackson (1941); Wade and Hyslop (1941).

**1885**. *List of the Coleoptera of America, north of Mexico*. American Entomological Society, Philadelphia. [2] + 161 pp. (8vo) [DP: 1885 (title page; preface dated 15 September 1885), 30 October 1885 (*Science* 6: 382), December 1885 (*Ent Amer* 1: 179)] MCZ, GB, BHL

**1895**. *Third supplement to the list of Coleoptera of America, north of Mexico.* American Entomological Society, Philadelphia. [1] + 62 pp. (8vo) [DP: 1895 (title page), June 1895 (*Ent News* 6: 192), August 1895 (*Nat Nov*)] MCZ, BHL

The first and second supplements were published in 1887 and 1889 in *Entomologica Americana* 2: 213–220 and 5: 127–138. An expanded version of the second supplement, entitled “*Second supplement to the list of Coleoptera of America, north of Mexico, together with bibliographical references to synopses, monographs, etc., published since the “Classification*”,” was published the same year by the Brooklyn Entomological Society.

#### Hentsch, Gustav Friedrich (? – 13 October 1813: Grimma, Saxony, Germany). German professor at the agricultural school in Meissen, Saxony. Reference. Ersch (1821: 124).

**1804**. *Epitome entomologiae systematicae secundum Fabricium continens genera et species insectorum Europaeorum.* Officinae publicae inservientis literaturae, Lipsiae [= Leipzig]. 4 + 218 + [2 (Index)] + vii pp. (4to) [DP: 1804 (title page), July–December 1804 (*Verz Bücher Landkarten*)] GB, GDZ

This is a list of insect names in Fabricius’ *Entomologia Systematica*, 1792–1794, with short descriptions. The book was announced as forthcoming at the St. Michael’s Fairs of 1804 in Leipzig, which was held on 30 September 1804 (Evenhuis 2014a: 4), in the 25 August 1804 issue of *Intelligenzblatt der Allgemeinen Literatur-Zeitung*.

#### Herbst, Johann Friedrich Wilhelm (Minden, North Rhine-Westphalia, Germany: 1 November 1743 – 5 November 1807: Berlin, Germany). German clergyman and naturalist; teacher in Berlin, then chaplain for the Prussian army, church preacher and finally member of the archdiocese in Berlin; his collection is at the Zoologisches Museum der Humboldt-Universität in Berlin. References. Anonymous (1807); Rose (1848c: 291); Evenhuis (1997a: 357, P); Damkaer (2002: 90–91).

**1784**. *Kurze Einleitung zur Kenntniss der Insekten, für Ungeübte und Anfänger.* Gottlieb August Lange, Berlin und Stralsund. (8vo) [DP: 1784 (title page; *Vorrede* dated 16 November 1784)] NCSU

[*Erster Band. 1tes Stüd.*]. [8] + pp. 5–96 + pls 229–240.

*2tes Stüd.* Pp. 99–150 + [2] + pls 241–252.

*3tes Stüd.* Pp. 155–204 + pls 253–264.

*4tes Stüd.* Pp. 207–278 + [12] + pls 265–276.

The entire work consists of three volumes, 1784–1786, each issued in four *Stukke* and including 48 plates. Only the first volume, published in 1784, pertains to Coleoptera (pp. 35–185). This volume was also published simultaneously as volume 6 of G.H. Borowski’s *Gemeinnüzzige Naturgeschichte des Thierreichs, darin die merkwürdigsten und nüzlichsten Thiere in systematischer Ordnung beschrieben, und die Geschlechter in Abbildungen nach der Natur vorgestellt werden*” [BHL, GB].

**1790–1806**. *Natursystem aller bekannten in- und ausländischen Insekten, als eine Fortsetzung der von Büffonschen Naturgeschichte. Der Käfer.* Joachim Pauli, Berlin. (8vo–text; Folio–plates) CNC

*Dritter Theil. Mit sechzehn illuminirten Kupfertafeln.* xiv + 325 pp. + pls 21–34, D–E. This volume was issued in two *Hefte*: **1**: (pp. 1–151, 6 pls) 19 September 1790 (*Allg Lit Ztg*); **2**: (pp. 152–325) 18 May 1791 (*Allg Lit Ztg*). The title page is dated 1790.

*Vierter Theil. Mit zwölf illuminirten Kupfertafeln.* viii + 197 pp. + pls 35–43 + F–H. This volume was issued in two *Hefte* (see Vorst 2008): **1**: (pp. 1–112 + pls 35–39, F) 1791 (wrapper), 23 March 1792 (*Allg Lit Ztg*); **2**: (pp. i–viii + 113–197 + pls 40–43, G–H) 27 September 1792 (*Gött Anz*). The title page of the book is dated 1792.

*Fünfter Theil. Mit 16 illuminirten Kupfertafeln.* xvi + 392 pp. + pls 44–59, J–N. [DP: 1793 (title page), 12 October 1793 (*Gött Anz*)].

*Sechster Theil. Mit 38 illuminirten Kupfertafeln.* xxiv + 520 pp. + pls 60–95, O–P. [DP: 1795 (title page), 5 August 1795 (*Intell Allg Lit Ztg*), 14 May 1796 (*Gött Anz*)].

*Siebenter Theil. Mit 26 illuminirten Kupfertafeln.* xi + 346 pp. + pls 96–116, Q–U. [DP: 1797 (title page), 7 October 1797 (*Intell Allg Lit Ztg*), 24 November 1797 (*Allg Lit Ztg*)].

*Achter Theil. Mit 24 illuminirten Kupfertafeln.* xvi + 420 pp. + pls 117–137, V–X. [DP^45^: 1799 (title page), 5 June 1800 (*Reichs-Anz*), 30 July 1800 (*Allg Lit Ztg*)].

*Neunter Theil. Mit 22 illum. Tafeln, Tab. 138–158 u. der Instr. Taf. Z.* xvi + 344 pp. + pls 138–158, Z. [DP: 1801 (title page), 5 December 1801 (*Intell Ztg Welt*), 20 February 1802 (*Intell Ztg Welt*), 30 July 1802 (*Allg Lit Ztg*)].

*Zehnter Theil. Mit 18 illuminirten Tafeln. Sub. 159 bis 177.* viii + 285 pp. + pls 159–177. [DP: 1806 (title page)].

The entire series was published in 21 volumes, 1783–1806, divided in two parts: *A. Käfer* issued in 10 volumes, 1785–1806, and *B. Schmetterlinge*, issued in 11 volumes, 1783–1804. The first two volumes of the series *Käfer* were authored by Jablonsky, 1785 [*q.v.*] and Jablonsky and Herbst, 1789 [*q.v.*] respectively.

#### Herrich-Schäffer [Schaeffer], Gottlieb August Wilhelm (Regensburg, Bavaria, Germany: 17 December 1799 – 14 April 1874: Regensburg, Germany). Bavarian physician, botanist and entomologist; practiced medicine in Regensburg from 1824 to 1856; chairman of the Regensburg Botanical Society (1861–1871); most of his original collection went to Otto Staudinger in Blasewitz and was scattered later. References. Anonymous (1874b); Hofmann (1874); Jahn (1969: 683–684); Frank (2008: 1793–1794).

**1829–1844**. *Deutschlands Insecten herausgegeben von Dr. G.W.F. Panzer. Fortgesetzt von Dr. G.A.W. Herrich-Schäffer.* Pustet, Regensburg. (Oblong 16mo) CNC

The entire series consists of 190 *Hefte*, each containing 24 small, loose, colored plates and one or two pages of text corresponding to each plate. The first 109 *Hefte*, 1792–1810, were authored by Panzer [*q.v.*], *Hefte* 110, issued in 1823, by Carl Geyer [*q.v.*], and *Hefte* 111–190, issued 1829–1844, by G.A.W. Herrich-Schäffer. Unless otherwise noted, the dates of publication of each *Heft* are from Sherborn (1923: 566–567) and Evenhuis (1997a: 362): **111**: March 1829, January–June 1829 (*Verz Bücher Landkarten*); **112**: July–December 1829 (*Verz Bücher Landkarten*); **113**: January–February 1830; **114**: 1 March 1830; **115–116**: January–June 1831 (*Verz Bücher Landkarten*); **117**: 3 July 1833; **118**: before October 1833; **119**: 1 October 1833; **120**: 1 December 1833; **121–124**: January–June 1834 (*Verz Bücher Landkarten*); **125–126**: 1834; **127–129**: 1834–1835; **130–131**: before May 1835; **132**: 1 May 1835; **133**: January–June 1835 (*Verz Bücher Landkarten*), September 1835 (*Nouv Rev Germ*); **134–136**: before 4 March 1836; **137–139**: 4 March 1836 (*Allg Bibl Deutsch*), 9 March 1836 (*Lit Ztg*), January–June 1836 (*Verz Bücher Landkarten*), 17 August 1836 (*Allg Forst Jagd Ztg*); **140**: before 1 October 1836; **141–143**: 1 October 1836; **144**: 1 January 1837; **145**: 1 February 1837; **146**: 1 March 1837; **147**: 1 April 1837; **148**: 1 May 1837; **149**: 1 June 1837; **150**: 1 July 1837; **151**: 1 August 1837; **152**: 1 September 1837; **153**: 1 January 1838; **154**: 1 February 1838; **155**: 1 March 1838, 9 May 1838 (*Lit Ztg*); **156**: 1 April 1838; **157**: 1 May 1838; **158**: 1 June 1838, 22 August 1838 (*Lit Ztg*); **159**: 1 July 1838; **160**: 1 August 1838; **161**: 1 September 1838; **162**: 1 October 1838; **163**: 1 November 1838; **164**: 1 December 1838, 2 January 1839 (*Lit Ztg*); **165**: 1 January 1839; **166**: before March 1839; **167**: 1 March 1839; **168**: April 1839; **169**: 1 May 1839; **170–173**: after May 1839; **174–175**: 25 September 1840 (*Allg Bibl Deutsch*), 1 October 1840 (*Bibl Blätt*); **176**: January 1841, 19 February 1841 (*Allg Bibl Deutsch*); **177**: 19 February 1841 (*Allg Bibl Deutsch*), February 1841; **178**: 25 September 1840 (*Allg Bibl Deutsch*)^46^, 1 October 1840 (*Bibl Blätt*), March 1841; **179**: 19 February 1841 (*Allg Bibl Deutsch*), April 1841; **180**: May 1841; **181**: June 1841; **182**: 16 July 1841 (*Allg Bibl Deutsch*), July 1841, 14 August 1841 (*Oest Med Wochen*); **183**: 1 December 1842 (*Ent Ztg* 4: 32); **184**: September 1844; **185–187**: before October 1844; **188**: October 1844; **189**: November 1844, 26 December 1844 (*Allg Bibl Deutsch*); **190**: 26 December 1844 (*Allg Bibl Deutsch*), December 1844, July–December 1844 (*Verz Bücher Landkarten*).

No title pages were seen during this study. The title listed here is that cited in *Allgemeine Bibliographie für Deutschland*.

Schenkling (1939: 216–217) provided a list of all beetles illustrated in *Hefte* 111–190.

**1840**. *Nomenclator entomologicus. Verzeichniss der europäischen Insecten; zur Erleichterung des Tauschverkehrs mit Preisen versehen. Zweites Heft. Coleoptera, Orthoptera, Dermatoptera und Hymenoptera. Mit 8 lithographirten Tafeln.* Friedrich Pustet, Regensburg. viii + 40 [Coleoptera] + 244 pp. + 8 pls. (8vo) [DP: 1840 (title page), 10 January 1840 (*Allg Bibl Deutsch*), 13 January 1840 (*Bibl Blätt*), 15 January 1840 (*Lit Ztg*)] CNC, GB, GAL

The first *Heft*, published in 1835, pertains to Lepidoptera and Hemiptera.

**1840**. II. Animalia articulata. Classis I. Insecta. Pp. 45–386 *in*: *Fauna Ratisbonensis, oder Uebersicht der in der Gegend um Regensburg einheimischen Thiere. Von K.L. Koch, Dr. A. Herrich-Schäffer, und F. Forster.* G.J. Manz, Regensburg. xvi + 478 pp. (8vo) [DP: 1840 (title page; *Einleitung* dated 3 August 1840), 6 November 1840 (*Allg Bibl Deutsch*), 12 November 1840 (*Bibl Blätt*), November–December 1840 (*Bull Litt Étr*)] GB

Herrich-Schäffer’s contribution consists of a list of the insects from the surroundings of Regensburg in Bavaria; several new species are described but none for Coleoptera (pp. 52–148).

A second title page of the book reads “*Naturhistorische Topographie von Regensburg. In Verbindung mit Forster, Herrich-Schäffer, Koch, v. Schmöger und v. Voith bearbeitet von Dr. A.E. Fürnrohr. Dritter Band, die Fauna von Regensburg enthaltend*.”

#### Heyden, Lucas Friedrich Julius Dominicus von (Frankfurt am Main, Hesse, Germany: 22 May 1838 – 13 September 1915: Frankfurt am Main, Germany). German military officer, entomologist, with a specialty on Coleoptera, and paleontologist; his collection of Palaearctic Coleoptera is at the Deutsches Entomologisches Institut in Müncheberg. References. Reitter (1915, P); Sattler (1915, P); Kobelt (1916, P); Weise (1916).

[**1870**]. *Entomologische Reise nach dem südlichen Spanien, der Sierra Guadarrama und Sierra Morena, Portugal und den Cantabrischen Gebirgen beschrieben von Lucas von Heyden, mit Beschreibungen der neuen Arten von L. v. Heyden und den Mitgliedern des Berliner entomol. Vereins: H. Allard (Paris), Ch. Brisout de Barneville (Saint-Germain-en-Laye), Desbrochers des Loges (Gannat), G. Dieck (Merseburg), v. Harold (München), v. Kiesenwetter (Bautzen), Kirsch (Dresden), Kraatz (Berlin), Löw (Guben), F. de Saulcy (Metz), Scriba (Wimpfen), Seidlitz (Dorpat) und einem Anhange: v. Heyden: Revision der europäischen Hymenoplia-Arten, Allard: Révision des curculionides Byrsopsides.* Dr. G. Kraatz [&] Nicolai, Berlin; Friedrich Fleischer, Leipzig; L. Buquet, Paris. 218 pp. + 2 pls. (8vo) [DP: 1870 (title page), 10 June 1871 (*Wien Kirch*), January–June 1871 (*Bibliot Hist-Nat*)] CNC, GB

This work was issued as a *Beiheft* (supplement) to volume 14 [1870] of the *Berliner Entomologische Zeitschrift*. It is divided into four parts: the first one (pp. 1–56) is an account by Heyden of his voyage through Spain and Portugal; the second one (pp. 57 [75 in error]–176) contains the descriptions of several new genera and 141 new species by the author and several members of the Berlin Entomological Society; the third one (pp. 177–183) is a revision of the European species of *Hymenoplia*; the fourth one contains a revision of the curculionid genera *Rhytirhinus* and *Gronops* (pp. 185–206) and a revision of the genus *Sphenophorus* (pp. 207–210) by Ernest Allard. The section by Allard was first issued in 1869 [*q.v.*].

Evenhuis (1997a: 364) mentioned, without qualification, that this work was not published until 1871. However, Rye (1871: 233–234 under Allard and 239–240 under Heyden) recorded the entire publication in his review of the entomological literature for the year 1870 while Brauer (1871: 180) noticed pages 1–176 in his review for 1870.

**1880–1882**. *Catalog der Coleopteren von Sibirien mit Einschluss derjenigen der Turanischen Länder, Turkestans und der chinesischen Grenzgebiete. Mit specieller Angabe der einzelnen Fundorte in Sibirien und genauer Citirung der darauf bezüglichen einzelnen Arbeiten nach eigenem Vergleich, sowie mit besonderer Rücksicht auf die geographische Verbreitung der einzelnen Arten über die Grenzländer, namentlich Europa und Deutschland. Herausgegeben von der Deutschen Entomologischen Gesellschaft als besonderes Heft der Deutschen Entomologischen Zeitschrift.* A.W. Schade, Berlin. xxiv + 224 pp. (8vo) CNC, GB

This catalogue of the beetles of Siberia, including also those from Turan, Turkistan and the area around the Chinese border, was issued in three parts as supplements to the *Deutsche Entomologische Zeitschrift*: **1**: (pp. 1–96) in or before October 1880 (date of publication of page iv of volume 24 of the *Deutsche Entomologische Zeitschrift* where these pages are noted); **2**: (pp. i–xxiv, 97–112) in or before October 1881 (date of publication of page 6 of volume 25 of the *Deutsche Entomologische Zeitschrift* where this part is noted); **3**: (pp. 113–224) after 15 February 1882 (date on page 221), March 1882 (*Nat Nov*), April 1882 (*Humboldt* 1: 234). The title page is dated 1880–1881.

**1883**. [Heyden, L. von, Reitter, E. and Weise, J.] *Catalogus coleopterorum Europae et Caucasi. Editio tertia.* Edw. Janson, Londini; Nicolai, Berolini [= Berlin]; Luc. Buquet, Parisiis. [2] + 228 pp. (8vo) [DP: 1883 (title page), July 1883 (*Nat Nov*), 25 October 1883 (*Naturwiss Ver Sach Thür*), 24 November 1883 (*Deutsch Litt*), 15 December 1883 (*Athenaeum–Prague*), July–December 1883 (*Bibliot Hist-Nat*),] CMLE, GB

According to the preface (see p. [2]), Lucas von Heyden wrote the text from Cicindelidae to Gyrinidae (pp. 1–32) and Tenebrionidae to Pythidae (pp. 125–146), Eduard Eppelsheim that on Staphylinidae (pp. 37–64), Edmund Reitter that from Pselaphidae to Cisidae (pp. 64–125), Ludwig Ganglbauer that on Cerambycidae (pp. 183–191), and Julius Weise that on Curculionoidea (pp. 146–182), Chrysomelidae (pp. 191–206) and Coccinellidae (pp. 206–209). There is no indication as to who wrote the section Hydrophilidae to Heteroceridae (pp. 32–37).

**1891**. [Heyden, L. von, Reitter E. and Weise, J.] *Catalogus coleopterorum Europae, Caucasi et Armeniae rossicae. Auctoribus Dr. L. v. Heyden, E. Reitter & J. Weise cum aliis sociis coleopterologicis. Edidit Edmund Reitter.* R. Friedländer & Sohn, Berlin; Edmund Reitter, Mödling; Caen. viii + 420 pp. (8vo) [DP: 1891 (title page), 13 May 1891 (*Soc Ent Fr*), 14 May 1891 (*Allg Bibl Deutsch*), May 1891 (*Nat Nov*)] CNC, GB

According to the preface (see p. iv), Ludwig Ganglbauer wrote the text on Carabidae (pp. 1–58), Lucas von Heyden that on Dytiscidae to Heteroceridae (pp. 58–77) and that on Cebrionidae to Oedemeridae (pp. 210–270), Eduard Eppelsheim that on Staphylinidae (pp. 77–122), Edmund Reitter that on Pselaphidae to Elateridae (pp. 123–210) and that on Cerambycidae (pp. 337–355) and Julius Weise that on Curculionoidea (pp. 270–337) and that to Chrysomelidae and Coccinellidae (pp. 355–392). Louis Bedel and Albert Fauvel also contributed to the redaction of the work but there is no indication as to what they did precisely.

The copies seen have two columns per page but some, containing vi + 812 pages, were printed with one column per page (Alonso-Zarazaga and Lyall 1999: 230). A second edition of this work was issued in 1906 in Berlin.

**1893–1898**. *Catalog der Coleopteren von Sibirien, mit Einschluss derjenigen des östlichen Caspi-Gebietes, von Turcmenien, Turkestan, Nord-Thibet und des Amur-Gebietes. Mit specieller Angabe der einzelnen Fundorte und genauer Citirung der darauf bezüglichen Literatur. Herausgegeben von der Deutschen Entomologischen Gesellschaft.* A.W. Schade, Berlin. (8vo) CNC

*Nachtrag I.* 217 pp. [DP: 1893–1895 (Heyden 1896: 3)]. Although the title page is dated 1893, this work was issued in parts by the *Deutschen Entomologischen Gesellschaft*. I have not found dates of publication except that the *Société Entomologique de France* received pages 97–144 on 27 March 1895, 145–176 on 13 November 1895 and 177–217 on 10 June 1896. The whole publication was noted in February 1896 (*Nat Nov*).

*Nachtrag II.* 84 pp. [DP: 1896 (title page; preface dated 31 October 1895)]. This work was probably also issued in parts, 1896–1898. The *Zoological Record* recorded pages 1–32 in 1896 and pages 49–84 in 1898 and the *Société Entomologique de France* received pages 1–32 on 24 February 1897 and 33–48 on 13 October 1897.

*Nachtrag III.* 24 pp. [DP: 1898 (title page; last page dated end of August 1898)].

This publication consists of a catalogue of the beetles of Siberia, including also those of the eastern region of the Caspian Sea, from Turkmenistan, Turkistan, north Tibet and the Amur region.

#### Heyne, Alexander (Leipzig, Saxony, Germany: 1 July 1869 – 23 December 1927: Berlin, Germany). German bookseller, natural history dealer and entomologist; had no personal collections. Reference. Korschefsky (1929).

**1893–1908**. [Heyne, A. and Taschenberg, O.] *Die exotischen Käfer in Wort und Bild. Begonnen von Alexander Heyne. Fortgeführt und vollendet von Dr. Otto Taschenberg.* G. Reusche, Leipzig. [2] + vii + 262 + [i–l (Register)] pp. + 40 [numbered 1–39 and 21*bis*] pls. (4to) USNM, BHL, GB

This work was issued in 27 *Lieferungen*, as follows (collation and dates of publication from Blackwelder 1949 unless otherwise noted): **1**: (ii [*Vorwort*] + pp. 1–6, pls 1, 16) 1893 (cover; *Vorwort* dated December 1893), December 1893 (*Nat Nov*); **2**: (pp. 7–10, pls 2, 17) 1894, May 1894 (*Nat Nov*); **3–4**: (pp. 11–26, pls 3–4, 6–7) 1895, January 1895 (*Zool Anz*), February 1895 (*Nat Nov*); **5**: (pp. 27–34, pls 5, 9) 1896, July 1896 (*Nat Nov*), 10 August 1896 (*Bibl Zool*); **6**: (pp. 35–42, pls 8, 10) 1896, August 1896 (*Nat Nov*); **7–8**: (pp. 43–58, pls 11–14) 1897, September 1897 (*Nat Nov*), October 1897 (*Allg Bibl*); **9**: (pp. 59–66, pls 15, 19) 1900, January 1901 (*Nat Nov*); **10**: (pp. 67–74, pls 18, 20) 1901, April 1901 (*Nat Nov*); **11–12**: (pp. 75–90, pls 22–23, 33, 35) 1902, 26 September 1902 (*Zool Anz*), October 1902 (*Nat Nov*); **13–14**: (pp. 91–106, pls 21, 21*bis*, 24–25) 1903, October 1903 (*Nat Nov*); **15–16**: (pp. 107–122, pls 26–29) 1904, April 1904 (*Nat Nov*); **17–18**: (pp. 123–138, pls 30–31) 1904, October 1904 (*Nat Nov*); **19–20**: (pp. 139–170, pls 32, 34) 1905, April 1905 (*Nat Nov*); **21–22**: (pp. 171–194, pls 36–37) 1906, April 1906 (*Nat Nov*), June 1906 (*Ent Litt*); **23–24**: (pp. 195–218, pls 38–39) 1907, August 1907 (*Nat Nov*); **25–26**: (pp. 219–262) 1907, August 1907 (*Nat Nov*), October 1907 (*Ent Litt*); **27**: (vii + pp. i–l [Index]) 1907, February 1908 (*Nat Nov*), April 1908 (*Ent Litt*). The title page is dated 1908. The publisher of the first ten *Lieferungen* was Ernst Heyne (Friedländer 1908: 63) and G. Reusche for the following ones. However, the cover of the book reads “*Die exotischen Käfer in Wort und Bild von A. Heyne u. Prof. Taschenberg*” and the publisher and place of publication is “J.F. Schreiber, Esslingen & München.”

The first 12 *Lieferungen* were authored by Alexander Heyne (Blackwelder 1957: 1117); based on the title page, the remaining ones were apparently authored by Otto Taschenberg [1854–1922] alone although Blackwelder (1957: 1117) credited them to Heyne and Taschenberg.

**1894**. *Systematisches und alphabetisches Verzeichnis der bis 1892 beschriebenen exotischen Cicindelidae. Nach Fleutiaux, Catalogue systématique des Cicindelidae décrits depuis Linné.* Ernst Heyne, Leipzig. 36 + [2] pp. (8vo) [DP: 1894 (title page), 15 June 1894 (*Soc Ent*; *Ent Zeit* 8: 51), June 1894 (*Nat Nov*), 15 July 1894 (*Naturwiss Wochen*)] MCZ, GB

#### Hill, John (Peterborough, Cambridgeshire, United Kingdom: 1714 – 21 November 1775: London, United Kingdom). British naturalist, physician and apothecary. References. Rose (1848: 321–322); Rousseau (2012, P).

**1773**. *A general natural history: or, new and accurate descriptions of the animals, vegetables, and minerals of the various parts of the world; with their virtues, and uses, in medicine and mechanics: illustrated by a general review of the knowledge of the Ancients; and the improvements and discoveries of later ages in these studies. With a great number of figures, elegantly engraved. Enlarged with additional plates.* [*Vol. III. The history of animals*]. London. iv + 586 + [4 (Index)] pp. + 29 pls. (Folio) [DP: 1773 (title page)] GB

The entire work consists of three volumes, issued in 1773. The Coleoptera are on pages 38–58 of the third volume. The species are described but not named. The genera are also described and have scientific names, one of them, *Hemisphaeria* (p. 42) has not been used subsequently. However, since the author did not applied the Principle of Binominal Nomenclature in this work, the name is unavailable (ICZN 1999: Article 11.4).

The first edition of this work was published in 1748–1752 [GB], also in three volumes.

#### Hoeven, Jan van der (Rotterdam, South Holland, Netherlands: 9 February 1801 – 10 March 1868: Leiden, South Holland, Netherlands). Dutch zoologist; professor of zoology and mineralogy at the University of Leiden (1826–1860). References. Harting (1868); Salverda (1868); Groshans (1870).

**1828**. *Handboek der dierkunde, of grondbeginsels der natuurlijke geschiedenis van het dierenrijk. Eersten deels, tweede stuk. Gekorvene, spinachtige en schaaldieren.* J. Allart, Rotterdam. vi + vii + [1] + pp. 173–446 + [1]. (8vo) [DP: 1828 (title page; *Voorrede* dated 23 November 1828), 15 December 1828 (back cover)] GB

The entire work was issued in four parts forming two volumes, 1827–1833, plus an atlas. The Coleoptera are on pages 314–354 of the second part of the first volume. A second, improved and expanded edition in two volumes was published in Amsterdam in 1849 (vol. 1) and 1855 (vol. 2) [MDZ]. A German translation of the second edition was issued under the title “*Handbuch der Zoologie. Nach der zweiten höllandischen Ausgabe*” [GB, BHL] in Leipzig, 1850 (vol. 1) and 1856 (vol. 2). An English translation of the second edition was published under the title “*Handbook of zoology. Translated from the second Dutch edition by the Rev. William Clark*” [CAKL, GB] in Cambridge in 1856 (vol. 1) and 1858 (vol. 2).

Additions and corrections to the first volume of the second edition were published by Rudolph Leuckart in Leipzig, 1856, under the title “*Nachträge und Berichtigungen zu dem ersten Bande von J. van der Hoeven’s Handbuch der Zoologie. Eine systematisch geordnete Übersicht der hauptsächlichsten neueren Leistungen über die Zoologie der wirbellosen Thiere*” [GB].

#### Hoffmann, Johann Jacob Julius (Meissenheim, Baden-Württemberg, Germany: 27 December 1767 – 12 February 1814: Meissenheim, Germany). German physician in Meissenheim.

**1803**. [Hoffmann, J.J.J., Koch, J.D.W., Müller, P.W.J. and Linz, J.M.] *Entomologische Hefte enthaltend Beiträge zur weitern Kenntniss und Aufklärung der Insektengeschichte. Eine Vorarbeit zu einer künftigen Faune des Departements vom Donnersberge und den angrenzenden Gegenden der Departemente von der Saar, und von Rhein und Mosel. Ausgearbeitet von einigen Freuden der Naturgeschichte.* Friedrich Esslinger, Frankfurt am Main. (8vo) MCZ, GB, MDZ

*Erstes Heft. Mit einer Kupfertafel von Hrn. Sturm.* xvi + 119 pp. + 1 pl. [DP: 1803 (title page; *Vorrede* dated February 1802 and signed J.J. Hoffmann, J.D.W. Koch, P.W.J. Müller, J.M. Linz), 20 April 1804 (*Neue Leip Lit*)]. This *Heft* includes: *Monographie der von den Verfassern in dem Departemente vom Donnersberge, und den angrenzenden Gegenden der Departemente von der Saar, und von Rhein und Mosel einheimisch beobachteten Stutzkäfer. (Hister.)* (pp. 1–119). The author’s name(s) is not listed.

*Zweites Heft. Mit zwei Kupfertafeln von Hrn. Sturm.* 130 + [4] pp. + 2 pls. [DP: 1803 (title page)]. This *Heft* includes: I. *Monographie der von den Verfassern in dem Departemente vom Donnersberge, und den angrenzenden Gegenden der Departemente von der Saar, und von Rhein und Mosel einheimisch beobachteten Flohkäfer. (Haltica.)* (pp. 5–90); II. *Monographie der von den Verfassern in dem Departemente vom Donnersberge, und den angrenzenden Gegenden der Departemente von der Saar, und von Rhein und Mosel einheimisch bemerkten Dorcatomen* (pp. 91–105); III. *Vermischte Bemerkungen* (pp. 107–119); *Nachtrag* (pp. 120–130 about *Hister*). The author’s name(s) is not listed.

#### Hoffmeister, Philipp (Eiterhagen, Hesse, Germany: 17 April 1804 – 5 March 1874: Marburg, Hessen, Germany). German preacher, artist and naturalist. References. Gerland (1863: 51–65); Berkemann (2013, P).

**1838**. *Das zweckmässige Fangen, Tödten und Aufbewahren der Käfer. Eine kurze Anweisung für Anfänger und Liebhaber der Entomologie. Mit 1 Kupfertafel.* C.A. Eyraud, Neuhaldensleben. 116 pp. + 6 pls. (8vo) [DP: 1838 (title page), 24 August 1838 (*Allg Bibl Deutsch*), 5 September 1838 (*Lit Ztg*), July–December 1838 (*Verz Bücher Landkarten*)] CMLE, GB, ARC

This book was published under the pseudonym “Fel[ix] Heur.”

#### Hofmann, Craft Ernst. In an undated announcement of books^47^ this author is described as a practical entomologist who has travelled for 25 years, with short breaks, to almost all countries of Europe.

**1834**. *Verzeichniss aller in Europa vorkommenden Geschlechter der Insecten nach Latreille’s System.* München. ii + 21 + [1 (Druckfehler)] pp. (8vo) [DP: 1834 (title page; *Vorerinnerung* dated July 1834)] CNC (mf)

This work is a checklist of the genera of European insects. The Coleoptera are on pages 1–10.

A “*Neue Ausgabe*” was issued in 1841 by Beck in Nördlingen and noticed on 26 May 1841 (*Lit Ztg*) [GB, MDZ]. There are no differences between the two “editions” regarding the Coleoptera.

#### Hofmann, Ernst (Frankfurt am Main, Hesse, Germany: 5 May 1837 – 29 January 1892: Regensburg, Bavaria, Germany). German zoologist; curator at the state museum of natural history in Stuttgart. References. Hofmann (1892); Steudel (1893).

**1883**. *Der Käfersammler. 20 kolorierte Tafeln mit 502 Abbildungen und begleitendem Text.* Hoffmann, Stuttgart. viii + 135 + [1] pp. + 20 pls. (8vo) [DP: n.d. (title page; *Vorwort* dated “Frühjahr 1883”), May 1883 (*Nat Nov*), January–June 1883 (*Bibliot Hist-Nat*; *Verz Bücher Landkarten*), 20 July 1883 (*Science*), July 1883 (*Allg Bibl*; *Humboldt* 2: 281), 6 August 1883 (*Zool Anz*)] SBB

Seven editions of this work were issued, the last one in 1910.

#### Hope, Frederic William (London, United Kingdom: 3 January 1797 – 15 April 1862: London, United Kingdom). British naturalist; travelled in France, Germany, Netherlands and Switzerland in the 1830s; later, professor of zoology at Oxford; founding member of the Entomological Society of London in 1833; his collections and library were donated to Oxford University with the stipulation that a curator was appointed and so the position of Hope Professor of zoology was created. References. Westwood (1862); Pettigrew (1863: 157–162); Zimmerman (1993: 574–576, P); Foote (2004a); Frank (2008: 1846–1847).

**1834**. [Descriptions of beetles]. Pp. 54–55 + pl. 5 *in*: *A history of Egyptian mummies, and an account of the worship and embalming of the sacred animals by the Egyptians; with remarks on the funeral ceremonies of different nations. And observations on the mummies of the Canary Islands, of the ancient Peruvians, Burman priests, &c. By Thomas Joseph Pettigrew.* Longman, Rees, Orme, Brown, Green, and Longman, London. xxi + 264 + [1 (Errata et corrigenda)] pp. + 13 pls. (4to) [1834 (title page; dedication dated 20 March 1834), 19 April 1834 (*Athenaeum*), 26 April 1834 (*Lit Gaz*), April 1834 (Peddie and Waddington 1914: 444), 15 May 1834 (*R Soc Lond*)] GB

A few new beetle species are described in a footnote in this work by Hope: *Necrobia mumiarum* (p. 54), *Dermestes pollinctus* (p. 54), *Dermestes roei* (p. 55) and *Dermestes elongatus* (p. 55).

**1836**. *Buprestidae.* London. 13 pp. [DP: 1836 (Dunning 1867: cix)] (*n.v.*).

This booklet was privately and anonymously issued by Hope and distributed by him in 1836. The Commission ruled that this work was not published within the meaning of the *Code* (ICZN 1950b). In some 19th Century publications, the booklet was referred to as “*Synopsis of Australian Buprestidae*” (see Anonymous 1868b).

**1837**. *The coleopterist’s manual, containing the lamellicorn insects of Linneus and Fabricius.* Henry G. Bohn, London. xiii + [1 (Errata)] + 121 + [1 (Description of the plates)] pp. + 4 pls. (8vo) [DP: 1837 (title page), 20 January 1838 (*Lit Gaz*; *Athenaeum*), 27 January 1838 (*Spectator*), 31 January 1838 (*Lit Ztg*), 5 February 1838 (*Ent Soc Lond*), March 1838 (*Bull Litt Étr*)] CNC, GB

**1838**. *The coleopterist’s manual, part the second, containing the predaceous land and water beetles of Linneus and Fabricius.* Henry G. Bohn, London. xvi + 168 + [1 (Description of the plates)] pp. + 4 pls. (8vo) [DP: 1838 (title page), 19 January 1839 (*Lit Gaz*), February 1839 (*Gent Mag*), 4 March 1839 (*Ent Soc Lond*), 6 March 1839 (*Soc Ent Fr*)] CNC, GB

[**1841**]. *The coleopterist’s manual, part the third, containing various families, genera, and species, of beetles, recorded by Linneus and Fabricius. Also, descriptions of newly discovered and unpublished insects.* J.C. Bridgewater and Bowdery & Kerby, London. 191 + [2 (Description of the plates, Errata)] pp. + 3 pls. (8vo) [DP^48^: 1840 (title page), April 1841 (Low 1853: 171), 10 May 1841 (*Monthly Lit Adv*)] CNC

**1845**. *A catalogue of the lucanoid Coleoptera, in the collection of the Rev. F.W. Hope, M.A., F.R.S., &c. President of the Entomological Society of London. Together with descriptions of the new species therein contained.* J.C. Bridgewater, London. 31 pp. (8vo) [DP: 1845 (title page), September 1846 (*Rev Zool*), 9 November 1846 (*Ashmolean Soc*)] MCZ, NAL

The author(s) of this booklet is not mentioned anywhere but all new taxa are credited to Hope. Hagen (1862: 381; 1863: 275) and Horn and Schenkling (1928b: 574; 1929: 1319) listed this reference under both Hope and Westwood.

#### Hoppe, David Heinrich (Bruchhausen-Vilsen, Lower Saxony, Germany: 15 December 1760 – 1 August 1846: Regensburg, Bavaria, Germany). German apothecary, physician, botanist and entomologist; professor of natural history at the Lyceum in Regensburg; founded in 1790 the *Regensburgische Botanische Gesellschaft*, the first botanical organization in Bavaria, and presently the world’s oldest existing botanical society. References. Callisen (1832: 120–121); Anonymous (1845b, P); Fürnrohr (1849, P); Frahm and Eggers (2001: 208).

**1795**. *Envmeratio insectorvm elytratorvm circa Erlangam indigenarvm observationibvs iconibvsqve illvstrata. Dissertatio inavgvralis qvam svmmi nvminis avspicio ex decreto gratiosae facvltatis medicae pro svmmis in medicina honoribvs legitime obtinendis pvblico ervditorvm examini svbmittit David Henricvs Hoppe. Die April. CI*Ɔ*I*Ɔ*CCLXXXXV.* Typis Hilpertianis, Erlangae. 70 pp. + 1 pl. [dated 1793]. (8vo) [DP: April 1795 (title page)] GB

This work consists of a list of the Coleoptera in the vicinity of Erlangen in Bavaria (pp. 9–23) followed by observations on and descriptions of some species, including several new ones (pp. 24–70).

This dissertation was also published the same year under the title “*Envmeratio insectorvm elytratorvm circa Erlangam indigenarvm secvndvm systema Fabricianvm observationibvs iconibvsqve illvstrata*” by Bibliopolio Palmiano, Erlangae [CMLE].

**1796**. *Entomologisches Taschenbuch für die Anfänger und Liebhaber dieser Wissenschaft auf das Jahr 1796.* Montag- und Weiss, Regensburg. [14] + 240 pp. (8vo) [DP: n.d. (title page; *Vorbericht* dated 15 December 1795), 25 March 1796 (*Allg Lit Ztg*)] MCZ, GB, BHL, MDZ

This book is divided into 17 sections, all but three authored by Hoppe. The other three sections were written by a professor in Regensburg named Duval.

**1797**. *Entomologisches Taschenbuch für die Anfänger und Liebhaber dieser Wissenschaft auf das Jahr 1797*. Montag und Weiss, Regensburg. [2] + 252 pp. (8vo) [DP: n.d. (title page), 23 August 1797 (*Allg Lit Ztg*)] CMLE, MDZ

The book is divided into 14 sections, authored besides Hoppe by Duval, Schranck, Brahm, Andersch and Dallinger. Two sections, those of Brahm and Andersch, are taxonomically more important as they included new beetle taxa; they are recorded in this work under their respective author names.

#### Horn, George Henry (Philadelphia, Pennsylvania, USA: 7 April 1840 – 24 November 1897: Beesley’s Point, New Jersey, USA). American physician by vocation and coleopterist by devotion, longtime close friend of John Lawrence LeConte [*q.v.*]; served as surgeon in the infantry with the California Volunteers (1862–1866) which allowed him to collect over a large part of California as well as in Arizona and Nevada; visited Europe in 1874, 1882, and 1888; never married; his collection was bequeathed to the American Entomological Society and placed at the Academy of Natural Sciences in Philadelphia before being transferred to the Museum of Comparative Zoology in 1974. References. Calvert (1898); Essig (1931: 654–658, P); Mallis (1971: 248–252, P); Zimmerman (1993: 578–580, P); Smetana and Herman (2001: 80–81, P).

**1890**. Fam. Throscidae [pp. 193– 209]; Fam. Eucnemidae [pp. 210–257 + pl. 10] *in*: *Biologia Centrali-Americana. Insecta. Coleoptera. Vol. III. Part 1. Serricornia.* Taylor and Francis, London. xv + [1 (Errata et corrigenda)] + 690 pp. + 27 pls. (4to) [DP: November 1890 (bottom of the first page of each sheet)] CNC

This volume was issued in 32 parts between February 1882 and August 1897 (Lyal 2011: 82).

#### Horn, Hermann Wilhelm Walther (Berlin, Germany: 19 October 1871 – 10 July 1939: Berlin, Germany). German physician, bibliographer and coleopterist, specialist of tiger beetles (Cicindelinae); travelled to several countries in southern Europe and northern Africa in 1896, to Ceylan and India in 1899 and to the Western Hemisphere in 1902; appointed in 1904 director of Kraatz’s entomological museum which became later the Deutsche Entomologische Institut; his cicindelid collection is at that museum in Müncheberg. References. Heikertinger (1939); Korschefsky (1939); Frank (2008: 1855–1856).

**1891**. [Horn, H.W.W. and Roeschke, H.] *Monographie der paläarktischen Cicindelen. Analytisch bearbeitet mit besonderer Berücksichtigung der Variations-fähigkeit und geographischen Verbreitung. Mit 6 Tafeln.* Berlin. ix + 199 pp. + 6 pls. (8vo) [DP: 1891 (title page), end of May 1891 (Kraatz 1891: 9), August 1891 (*Nat Nov*)] CNC, GB, SME

This work was issued as supplement to *Deutsche Entomologische Zeitschrift* for the year 1891 and forms part 23 of the *Bestimmungs-Tabellen der europäischen Coleopteren* edited by Edmund Reitter.

**1898–1901**. *Revision der Cicindeliden mit besonderer Berücksichtigung der Variationsfähigkeit und geographischen Verbreitung.* A.W. Schade, Berlin. 64 pp. (8vo) BHL

This unfinished work was issued in parts as supplements to *Deutsche Entomologische Zeitschrift* as follows: (pp. 1–32) April 1898 (bottom of p. 3)^49^, 13 July 1898 (*Soc Ent Fr*), August 1898 (*Nat Nov*); (pp. 33–64) July 1901 (bottom of pp. 33, 49), 12 October 1901 (*Bibl Zool*).

#### Host, Nicolaus Thomas (Fiume [currently Rijeka], Croatia: 6 December 1761 – 13 January 1834: Schönbrunn [now part of Vienna], Austria). Austrian physician, botanist and entomologist; personal physician of the Austrian Emperor Francis I; director of the botanical garden at the Belvedere palaces in Vienna. References. Neilreich (1855: 35–36); Reichardt (1881); Goidanich (1975: 461–491, P).

[**1790**]. Entomologica. Pp. 291–302 + pl. 23 *in*: *Nicolai Josephi Jacquin collectanea ad botanicam, chemiam, et historiam naturalem, spectantia, cum figuris. Vol. III.* Wappler, Vindobonae [= Vienna]. 306 pp. + 23 pls. (4to) [DP: 1789 (title page), 18 April 1790 (Stafleu 1963: 63), 16 August 1790 (*Gött Anz*), 5 December 1790 (*Allg Lit Ztg*)] CNC, GB, GAL

The entire series of Nikolaus-Joseph Jacquin [1727–1817] consists of five volumes, 1787–1797. The Coleoptera described in Host’s contribution in the third volume are: *Scarabaeus sacer* (p. 291), *Curculio mutabilis* (p. 293), *Curculio cardiniger* (p. 295), *Curculio corruptor* (p. 296), *Elater mordelloides* (p. 298), and *Carabus pilosus* (p. 299).

Although dated 1789 on the title page, this book may have been published only the following year.

#### Hotz [Hotze], Johannes (Richterswil, Switzerland: 27 June 1734 – 4 July 1801: Frankfurt am Main, Hesse, Germany). Swiss physician; studied in Leipzig and Tübingen; practiced in his native town; moved to Frankfurt a.M. in 1796; personal friend to Johann Kaspar Lavater, Johann Wolfgang von Goethe and Johann Heinrich Pestalozzi. References. Stettbacher (1942); Wild (2001).

**1758**. *De Balneis infantvm dissertatio adnexa Bvprestis descriptione cvm tabvla aenea. Qvam rectore Vniversitatis magnificentissimo serenissimo principe ac domino Domino Io. Carolo Lvdovico comite palatino Rheni, vtrivsqve Bavariae dvce rel. rel. Praeside viro excellentissimo atqve experientissimo Georgio Friderico Sigwart praeceptore svo pie devenerando pro Doctoris medicinae gradv pvblice ventilabit avctor Ioannes Hotz, Tigvro-Helvetvs die XXVIII. Ian. MDCCLVIII.* Erhard, Tvbingae. 48 + [8] pp.+ 1 pl. (4to) [DP: 28 January 1858 (title page; p. [viii] dated 25 January 1758)] GB, MDZ

Authorship of the thesis is not well established. Some bibliographers credit it to Georg Friedrich Sigwart [1711–1795], others to Hotz. Two buprestid species are described and illustrated in a section entitled “Descriptio Bvprestis tabvla aenea illvstrata” (pp. 40–48). The thesis is not binominal.

#### Houlbert, Constant Vincent (Voutré, Mayenne, France: 18 July 1857 – 22 December 1947: Rennes, Ille-et-Vilaine, France). French naturalist; professor at the School of Medicine and Pharmacy in Rennes; later, conservator at the Museum of Natural History in Rennes. References. Des Abbayes (1948); Jolivet (1948, P).

**1892**. *Petite faune analytique des coléoptères français les plus communs contenant la description de 200 espèces d’insectes environ et un grand nombre de tableaux dichotomiques.* Paul Dupont, Paris. 78 pp. (16mo) [DP: 1892 (title page), 1 July 1892 (*Soc Sci Méd Ouest*; *Feuille Jeunes Nat*), 23 July 1892 (*Bibl Fr*), August 1892 (*Nat Nov*), September 1892 (*Polybiblion*)] ARC, YB (ph)

#### Houttuyn, Martin [Maarten, Martinus] (Hoorn, North Holland, Netherlands: 26 March 1720 – 27 April 1798: Amsterdam, Netherlands). Dutch physician and naturalist; practiced first in Hoorn and from 1753 in Amsterdam; his collection of zoological specimens was sold at auction in 1787. References. Evenhuis (1997a: 372); Damkaer (2002: 60–61); Boeseman and de Ligny (2004); Adler (2012: 30–31).

**1766**. *Natuurlyke historie of uitvoerige beschryving der dieren, planten en mineraalen, volgens het samenstel van der Heer Linnaeus. Met naauwkeurige afbeeldingen. Eerste deels.* De Erven van F. Houttuyn, Amsterdam. (8vo) CAKL, HATH

*Negende Stuk. De insekten.* vi + [8] + 640 pp. + pls 71–76. [DP: 1766 (title page; *voor-berigt* dated 21 July 1766), 9 March 1767 (*Gött Anz*)].

*Tiende Stuk. Vervolg der insekten.* [8] + 528 pp. + pls 77–83. [DP: 1766 (title page)].

Houttuyn’s entire series consists of 37 volumes comprising 21,500 pages of text and 296 copper plates (Merrill 1938: 303) issued 1761–1785. It was divided into three parts: the first one (*Eerste deel*) deals with zoology, the second one (*Tweede deel*) with botany and the third one (*Derde deel*) with minerals. The first *deel* was issued in 18 parts [*stukke*], 1761–1772 with the index published in 1773. The Coleoptera are in *stukke* 9 (pp. 108–112, 134–640) and 10 (pp. 1–104) issued in 1766.

**1787**. *Animalium Musaei Houttuyniani Index*. *Catalogus van eene uitmuntende verzameling van allerley soort van dieren en dierlyke zaaken, tot opheldering der natuurlyke historie. In meer dan dertig jaaren vergaderd en, volgens het samenstel van den wydberoemden Linnaeus, in orde geschikt. Benevens een appendix van een kostbaare party goud- en zilver-ertsen en andere mineralen en zeldzaame naturaliën. Welk alles verkogt zal worden, op woensdag den 14 maart 1787, en volgende dagen, ten huize van A. Dankmeyjer en Zoon, in ‘t Oude Zyds Heeren Logement te Amsterdam. Door de Makelaars J. Posthumus, Nic. Blinkvliet, Pieter Posthumus, Will. Jan Lobe, Pieter Bel en Joh. Zac. Rycke. By wien de catalogus voor 8 St. is te bekomen. Alles zal op maandag en dinsdag kunnen bezien worden.* J. Van der Burgh, Amsterdam. viii + 178 pp. (8vo) [DP: 14 March 1787 (title page)] GB

This is a catalog of specimens in Houttuyn’s collection to be sold at auction. The Coleoptera are on pages 57–81. At least one new beetle species is described, *Carabus trigonus* (p. 79). In Opinion 380, the ICZN (1956b) ruled that this catalog is suppressed for the purposes of nomenclature.

A copy of this catalogue is included in Boeseman and de Ligny (2004: Appendix B).

#### Hummel, Arvid-David (Gothenburg, Göteborg and Bohus, Sweden: 30 April 1778 – 20 October 1836: Ekenäs, Uusimaa, Finland). Russian (Swedish-born) government official for the censorship in Saint Petersburg and entomologist; started his career as notary in Gothenburg (1796–1801); moved to Stockholm and in 1807 to Saint Petersburg; spent his last years in Tammisaari (= Ekenäs), Finland. References. Carlander (1889: 501–503); Tommila (1963, P).

**1822–1829**. *Essais entomologiques.* Imprimerie de la Chancellerie privée du Ministère de l’Intérieur, St. Petersbourg. (8vo) CMLE

*N^o^ II. Sur les insectes de Saint-Pétersbourg, pendant l’été 1822. Lettre à la Société Impériale des Naturalistes de Moscou.* [2] + 30 pp. [DP: 1822 (title page; printing permit dated 3 October 1822 [JD]), November 1822 (Sahlberg 1875: 89)].

[*N^o^ III*]. *Observations sur les insectes de 1823. Monographia pelophilarum. Novae species. Avec une gravure.* [2] + 48 pp. + 1 pl. [DP: 1823 (title page; printing permit dated 25 October 1823 [JD])]. This part contains five sections: I. Corrections et additions aux Essais entomologiques, No. I. et II. (pp. 1–5); II. La teigne des meubles (pp. 6–14); III. Insectes de 1823 (pp. 15–33); IV. Monographia pelophilarum a C.G. Lib. Bar. de Mannerheim (pp. 34–42); V. Novae species (pp. 43–48).

*N^o^. IV. Insectes de 1824. Novae species.* [2] + 71 + [1] pp. [DP: 1825 (title page; printing permit dated 16 February 1825 [JD]), 1 May 1825 (Tommila 1963: 161)]. This part contains five sections: I. Corrections et additions aux Essais entomologiques, No. II et III (pp. 1–5); II. Insectes de 1824 (pp. 6–18); III. Novae coleopterorum species imperii Rossici incolae, a Carolo Gustavo Comite de Mannerheim descriptae (pp. 19–41); IV. Coleopterorum Sibiriae species novae, a Dom. Doct. Freder. Gebler descriptae (pp. 42–57); V. Novae species variorum (pp. 58–71).

*N^o^. VI. Insectes de 1826. Novae species. Catalogus insectorum quae itinere Petropoli in Chersonesum Tauricum et Iberiam anno 1825 collegit Benedictus Jäger. Avec une gravure.* [2] + 48 + [1] pp. [DP: 1827 (title page; printing permit dated 2 February 1827 [JD])]. This part contains the following sections: I. Insectes de 1826 (pp. 1–20); II. Supplementa quaedam in genus Carabum systemati entomologico a C.G. Comite de Mannerheim oblata (pp. 21–26); III. Catalogus insectorum quae itinere Petropoli in Chersonesum Tauricum et Iberiam anno 1825 collegit Benedictus Jäger (pp. 27–48).

*N^o^ VII. Tome second. N^o^ I.* 38 pp. [DP: 1829 (title page; printing permit dated 15 January 1829 [JD]), 13 September 1829 [JD] (*Soc Imp Nat Moscou*)].

The entire work consists of seven parts, 1821–1829. Parts 1 and 5 have no beetle content. While many articles were written by Hummel, some were authored by other entomologists. A title page was issued for the first six parts which reads besides the title “*Tome premier. N^o^ I.–VI.*” and is dated 1829.

#### Illiger, Johann Carl Wilhelm (Brunswick, Lower Saxony, Germany: 19 November 1775 – 10 May 1813: Berlin, Germany). German naturalist; professor of zoology and director of the Zoological Museum in Berlin; founded the journal *Magazin für Insektenkunde* which was published from 1802 to 1807. References. Fischer (1839); Hess (1881b); Wagenitz (1988: 88).

**1798**. *Verzeichniss der Käfer Preussens. Entworfen von Johann Gottlieb Kugelann Apotheker in Osterode. Ausgearbeitet von Johann Karl Wilhelm Illiger. Mit einer Vorrede des Professors und Pagenhofmeisters Hellwig in Braunschweig, und dem angehängten Versuche einer natürlichen Ordnungs- und Gattungs-Folge der Insekten.* Johann Jacob Gebauer, Halle. xlii + [1] + 510 + [1 (Zusätze)] pp. (8vo) [DP: 1798 (title page; *Vorrede* dated September 1798), 19 September 1798 (*Intell Allg Lit Ztg*; Alonso-Zarazaga and Krell 2011: 67), 17 November 1798 (*Braunsch Mag*)] CNC, GB

This work treats the Coleoptera of Prussia. It was started by Kugelann and finished by Illiger. A preview of this book was published by Johann Christian Ludwig Hellwig about eight months prior to its publication and the generic names *Aphodius* and *Oryctes* were made available in the preview (Alonso-Zarazaga and Krell 2011: 67). Corrections and additions to this work were published by Illiger (1801a).

**1800–1802**. *Olivier’s Entomologie oder Naturgeschichte der Insekten mit ihren Gattungs- und ArtMerkmalen, ihrer Beschreibung und Synonymie. Käfer. Uebersetzt und mit Zusätzen und Anmerkungen durchgängig begleitet von Karl Illiger.* Karl Reichard, Braunschweig. (4to) MCZ, MDZ, GB

*Erster Theil. Mit Kupfern.* xvi + 309 + [1] pp. + 2 pls. [DP: 1800 (title page; *Vorrede* dated 15 April 1800), 3 February 1801 (*Allg Lit Ztg*)]. This volume treats the genera *Lucanus* (pp. 36–79), *Lethrus* (pp. 80–85) and *Scarabaeus* (pp. 85–309).

*Zweiter Theil. Mit Kupfern.* iv + 266 + [1] pp. + pls 3–5. [DP: 1802 (title page; *Vorbericht* dated 1 August 1801)]. This volume treats the genera *Trox* (pp. 1–15), *Melolontha* (pp. 16–109), *Cetonia* (pp. 110–210), *Hexodon* (pp. 211–215) and *Hister* (pp. 216–245).

This work is a translation of the first volume of Olivier’s *Entomologie, ou histoire naturelle des insectes* [*q.v.*] with additional notes. According to Hagen (1862: 399), a new printing was issued in 1822 with only a change in the title (*n.v.*). Supplementary illustrations for this work were published by Sturm, 1802–1803 [*q.v.*].

#### Imhoff, Ludwig (Basel, Switzerland: 22 October 1801 – 13 September 1868: Basel, Switzerland). Swiss physician and zoologist; his first (and most complete) collection, including types, was bought by Louis Agassiz for the Museum of Comparative Zoology in Cambridge, Massachusett, while two subsequent collections including among others exotic beetles and the majority of the Curculionidae described by him went to the Naturhistorisches Museum in Basel. References. Bischoff-Ehinger (1869); Müller (1869); Rütimeyer (1869).

**1856**. *Versuch einer Einführung in das Studium der Koleoptern. In zwei Theilen und einem, 25 Tafeln lithographirter Abbildungen nebst Text enthaltenden, Anhange. Auf Kosten des Verfassers.* Schweighauser, Basel. xxxi + [2] + 114 + [2] + 272 + 25 pls. (8vo) [DP: 1856 (title page), 25 December 1856 (*Allg Bibl Deutsch*), October–December 1856 (*Cat Muquardt*), 31 January 1857 (*Intell Serapeum*), February 1857 (*Allg Bibl*), 11 April 1857 (*Lit Centr Deutsch*), January–June 1857 (*Bibliot Hist-Nat*)] MCZ, GB, MDZ

This book is divided in two sections. The first one is an introduction to the study of Coleoptera. The second one consists of descriptions of the various groups of beetles (mainly families and tribes) with a discussion of the genera belonging to each of them.

#### Jablonsky [Jablonski], Carl [Karl] Gustav (1756 – 25 May 1787: Berlin, Germany). German naturalist and illustrator; private secretary to the Queen of Prussia. References. Cates (1868: 508); Hess (1881a).

**1785**. *Natursystem aller bekannten in- und ausländischen Insekten, als eine Fortsetzung der von Büffonschen Naturgeschichte. Nach dem System des Ritters Carl von Linne bearbeitet. Der Käfer erster Theil. Mit sechs illuminirten Kupfertafeln.* Joachim Pauli, Berlin. xxiv + 310 pp. + pls 1–6. (8vo) [DP: 1785 (title page), 10 June 1785 (*Jena Ztg*), 4 August 1785 (*Neue Leip Ztg*), 12 November 1785 (*Gött Anz*), 8 December 1785 (*Allg Lit Ztg*)] CNC, GB

The series was continued by Jablonsky and Herbst (see next entry) and by Herbst, 1790–1806 [*q.v.*].

**1787–1789**. [Jablonsky, C.G. and Herbst, J.F.W.] *Natursystem aller bekannten in- und ausländischen Insekten, als eine Fortsetzung der von Büffonschen Naturgeschichte. Nach dem System des Ritters von Linné und Fabricius zu bearbeiten angefangen. Der Käfer zwenter Theil. Mit siebzehn illuminirten Kupfertafeln.* Joachim Pauli, Berlin. xvi + lxiv + 330 + [6 (Register)] pp. + pls 7–20, A–C. (8vo) CNC, GB

This part was issued in two *Hefte*: **1**: (lxiv + pp. 1–128 + pls 7–12, A–C) 21 November 1787 (*Allg Lit Ztg*); **2**: (xvi + pp. 129–330 + [6] + pls 13–20) 11 April 1789 (*Leip Intell-Blatt*), 29 August 1789 (*Allg Lit Ztg*). The title page is dated 1789 and the *Vorrede* 1 March 1789.

#### Jacoby, Martin (Altona [currently part of Hamburg], Hamburg, Germany: 12 April 1842 – 24 December 1907: London, United Kingdom). German musician who played in the orchestra of the Royal Italian Opera in London and later became a violin tutor; his interests turned to entomology and he developed a particular interest in Coleoptera and especially phytophagous beetles; his first chrysomelid collection is at the Museum of Comparative Zoology in Cambridge, Massachusetts, while his second is at the Natural History Museum in London (first selection) and at the Museum of Comparative Zoology. References. Anonymous (1908, P); Waterhouse (1908: xcvii–xcviii).

**1880–1892**. *Biologia Centrali-Americana. Insecta. Coleoptera. Vol. VI. Part 1. Phytophaga (part).* Taylor and Francis, London. xix + [1(Errata et corrigenda)] + 625 pp. (4to) CNC

This work was published in several parts (Lyal 2011: 86–87)^50^ as follows: (pp. 1–24 + pl. 1) June 1880; (pp. 25–48 + pl. 2) October 1880; (pp. 49–72 + pl. 3) December 1880; (pp. 73–88 + pl. 4) February 1881; (pp. 89–104 + pl. 5) June 1881; (pp. 105–120 + pl. 6) August 1881; (pp. 121–128) October 1881; (pp. 129–144 + pl. 7) December 1881; (pp. 145–168 + pl. 8) February 1882; (pp. 169–184 + pl. 9) June 1882; (pp. 185–208 + pl. 10) August 1882; (pp. 209–224 + pl. 11) October 1882; (pp. 225–256 + pls 12–14) January 1883; (pp. 257–264 + pl. 15) March 1883; (pp. 265–280 + pl. 16) February 1884; (pp. 281–304) April 1884; (pp. 305–320 + pl. 17) June 1884; (pp. 321–336 + pls 18–19) October 1884; (pp. 337–352 + pl. 20) January 1885; (pp. 353–368 + pl. 21) May 1885; (pp. 369–392 + pl. 22) August 1885; (pp. 393–408) November 1885; (pp. 409–432 + pl. 23) January 1886; (pp. 433–440 + pl. 24) February 1886; (pp. 441–464 + pl. 25) June 1886; (pp. 465–472 + pl. 26) July 1886; (pp. 473–480) October 1886; (pp. 481–488) November 1886; (pp. 489–496 + pl. 27) December 1886; (pp. 497–504 + pl. 28) January 1887; (pp. 505–528 + pl. 29) June 1887; (pp. 529–544 + pl. 30) August 1887; (pp. 545–560 + pl. 31) October 1887; (pp. 561–568 + pl. 32) November 1887; (pp. 569–584) December 1887; (pp. 585–600) January 1888; (pp. 601–625 + pl. 33) February 1888; (pp. i–xix + [1] + cancels of pp. 1,2,19,20,37,38,73,74) April 1892. This volume includes treatment of the following families: Sagridae (pp. 1–2), Crioceridae (pp. 2–19), Megalopodidae (pp. 19–26), Clythridae (pp. 26–37), Cryptocephalidae (pp. 38–73), Chlamydidae (pp. 73–90), Lamprosomidae (pp. 90–105), Eumolpidae (pp. 105–187), Chrysomelidae (pp. 188–262), and Galerucidae (pp. 263–625).

Pages 1, 2, 19, 20, 37, 38, 73, 74 were reissued in April 1892 to replace those originally published. The minor differences between the two sets of pages are noted by Lyal (2011: 85).

**1888–1892**. *Biologia Centrali-Americana. Insecta. Coleoptera. Vol. VI. Part 1, Supplement. Phytophaga (part).* Taylor and Francis, London. ii + 374 pp. + 43 pls. (4to) CNC

This work was published in several parts (see Lyal 2011: 87): (pp. 1–24 + pls 34, 35) June 1888; (pp. 25–40) August 1888; (pp. 41–56) October 1888; (pp. 57–72) November 1888; (pp. 73–80) December 1888; (pp. 81–88 + pl. 36) February 1889; (pp. 89–104) April 1889; (pp. 105–120 + pl. 37) May 1889; (pp. 121–128) July 1889; (pp. 129–136 + pl. 38) August 1889; (pp. 137–152) October 1889; (pp. 153–168 + pl. 39) November 1889; (pp. 169–176) February 1890; (pp. 177–184) May 1890; (pp. 185–200) June 1890; (pp. 201–208) August 1890; (pp. 209–216) October 1890; (pp. 217–224) November 1890; (pp. 225–232 + pl. 40) December 1890; (pp. 233–256) February 1891; (pp. 257–264) May 1891; (pp. 265–272) July 1891; (pl. 41) September 1891; (pp. 273–280) November 1891; (pp. 281–312) December 1891; (pl. 42) February 1892; (pp. 313–344) March 1892; (pp. 345–374, [i] + pl. 43) April 1892. The title page is dated 1888–1892, which applies only to the text pages, not to the plates.

**1892**. Coleoptera – (continued). Pp. 82–88 *in*: *Supplementary appendix to travels amongst the Great Andes of the Equator by Edward Whymper with contributions by H.W. Bates. T.G. Bonney. G.A. Boulenger. Peter Cameron. F. Day. W.L. Distant. A.E. Eaton, F.D. Godman. H.S. Gorham. Martin Jacoby. E.J. Miers. A. Sidney Ollif. O. Salvin. David Sharp. T.R.R. Stebbing. Illustrated.* John Murray, London. xxii + [1 (Addenda)] + 147 pp. + 14 pls. (4to) [DP: 1891 (title page), 2 March 1892 (Sharp 1892: 61), 18 March 1892 (*Science*), 26 March 1892 (*Athenaeum*), 9 April 1892 (*Publ Circ*), April 1892 (*Nat Nov*), 16 June 1892 (*Nature*), 13 July 1892 (*Soc Ent Fr*)] CNC, GB

According to Sharp (1892: 61), the printing was done in 1891 but the work was not available for purchase until 2 March 1892.

#### Jacquelin du Val [Jacquelin-Duval], Pierre Nicolas Camille (Prades, Pyrénées-Orientales, France: 28 July 1828 – 5 July 1862: Paris, France). French coleopterist; went to Paris in 1849 to study medicine but soon turned to entomology; his collection was given to the Muséum National d’Histoire Naturelle in Paris. References. Migneaux (1862); Gouillard (2004: 46); Cambefort (2006: 193–195, P); Frank (2008: 2049).

**1854–1861**. *Manuel entomologique. Généra des coléoptères d’Europe comprenant leur classification en familles naturelles, la description de tous les genres, des tableaux synoptiques destinés à faciliter l’étude, le catalogue de toutes les espèces, de nombreux dessins au trait de charactères et plus de treize cents types représentant un ou plusieurs insectes de chaque genre dessinés et peints d’après nature avec le plus grand soin par M. Jules Migneaux.* A. Deyrolle, Paris. (8vo) CNC, BHL, YB

*Tome premier.* cclxxvi + 140 pp. + pls i–xv, 1–43. [DP: 1857 (title page)]. This volume was issued in 30 livraisons (corresponding to livraisons 14–44 of the entire work): **14–26**: (pp. 1–100) 1855 (Sherborn’s notes^51^); **27–30** (pp. 101–140), 1856 (Sherborn’s notes); **31–44**: (i–cclxxvi) 1856 (Sherborn’s notes).

*Tome deuxième.* 285 + [2] pp. + pls 1–67. [DP: 1857–1859 (title page)]. This volume was issued in 28 livraisons (livraisons 45–73 of the entire work). According to Sherborn’s notes and Tottenham (1949: 360), the dates on the wrappers are as follows: **livr. 45–47** (pp. 1–32) 1856; **48–57** (pp. 33–128) 1857^52^; **58–67** (pp. 129–232) 1858; **68–73** (pp. 233–288) 1859. Livraisons 48–49 (pp. 33–48) were noticed on 5 September 1857 (*Bibl Fr*) and livraisons 59–60 (pp. 137–160) on 11 September 1858 (*Bibl Fr*). On the other hand, Reinwald (1859: 103; 1860: 109) noted that livraisons 50–60 (pp. 49–160) were published in 1858 and livraisons 61–73 (pp. 161–288) in 1859. Gerstaecker (1858: 255) mentioned that livraisons 45–54 (pp. 1–96 + 28 pls) were issued in 1857. Based on the evidences gathered during this study, the following year dates should be retained in my opinion: livr. 45–54 (pp. 1–96) 1857; livr. 55–67 (pp. 97–232) 1858; livr. 68–73 (pp. 233–288) 1859.

*Tome troisième.* 463 + [1] pp. + pls 1–100. [DP: 1859–1863 (title page)]. The third volume of the series was issued in 46 livraisons (livraisons 74–120 of the entire work), the first 43 (74–117) were authored by Jacquelin du Val, the remaining three (118–120) by L. Fairmaire [*q.v.*]. According to Sherborn’s notes, the date on the wrappers are as follows: **livr. 74–87**: (pp. 1–136) 1859; **88–102**: (pp. 137–272) 1860; **103–110**: (pp. 273–352) 1861; **111–117**: (pp. 353–423) 1863. On the other hand, livraisons 74–76 were noticed as published in the year 1859 (Reinwald 1860: 109), livraisons 77–91 in the year 1860 (Reinwald 1861:110), livraisons 92–106 in the year 1861 (Reinwald 1862: 118), livraisons 107–111 in the year 1862 (Reinwald 1863: 117), and livraisons 112–118 in the year 1863 (Reinwald 1864: 117). Gerstaecker (1862: 328) mentioned that livraisons 103–111 (including plates 66–85) were issued in 1861.

*Tome quatrième.* Pp. 1–95 + pls 1–30. [DP: 1854 (title page)]. The first 95 pages of this tome were authored by Jacquelin du Val, the remaining ones by L. Fairmaire, 1864–1868 [*q.v.*]. These 95 pages forms the first 12 livraisons of the entire work: **1–4**: (pp. 1–32) 1854 (Sherborn’s notes), 11 January 1854 (*Soc Ent Fr* [p. v, Jacquelin du Val and Migneaux mentioned that the first four livraisons are published]); **5–6**: (pp. 33–48) 1854 (Sherborn’s notes), 27 September 1854 (*Soc Ent Fr* [p. lxiv, Deyrolle announced that six livraisons have been published and that the next one will be issued only on 1 January 1855]); **7–12**: (pp. 49–95) 1855 (Sherborn’s notes).

*Catalogue*. 284 pp. [with only pp. 1–200 and 237–284 numbered]. The catalogue was issued along with the livraisons of the *Manuel entomologique* with separate pagination. The following pages only can be dated with confidence: (pp. [201–236]) February 1855 (footnote on p. 42); (pp. 1–32) November 1855 (footnote on p. 42); (pp. 33–42) January 1856 (footnote on p. 42); (pp. 43–52) 1 May 1856 (p. 47, 52); (pp. 53–84) 1857 (wrapper of livraison 54, *fide* Sherborn’s notes). The title page bears the date 1868. The following new taxa are described in the catalogue: *Bembidium flavoposticatum* Jacquelin du Val, 1855 (p. 31), *Hydroporus delarouzei* Jacquelin du Val, 1856 (p. 34), *Stenus novator* Jacquelin du Val, 1858^53^ (p. 74), *Saprinus cribellaticollis* Jacquelin du Val, 1858 (p. 99), *Ammoecius pyrenaeus* Jacquelin du Val, 1859 (p. 130), *Tribolium confusum* Jacquelin du Val, 1862^54^ (p. 181), *Chitona cretica* Fairmaire, 1863 (p. 199), *Cataphorticus* Jacquelin du Val, 1855 (p. [217]), *Larinus confinis* Jacquelin du Val, 1855 (p. [224]), *Bagous formicetorum* Jacquelin du Val, 1855 (p. [233]), *Timarcha scabripennis* Fairmaire, ?1867 (p. 261), *Timarcha chalcosoma* Fairmaire, ?1867 (p. 261), *Timarcha olivieri* Fairmaire, ?1867 (p. 261), *Timarcha parnassia* Fairmaire, ?1867 (p. 261). At least two *nomina nova*, *Feronia crenatipennis* Jacquelin du Val, 1855 (p. 16) and *Apion denominandum* Jacquelin du Val, 1855 (p. [206]), are proposed.

The plates were issued along with the livraisons of the *Manuel entomologique*. They are either attached to the respective tome or bounded separately, with separate title pages reading besides the title “*Tome premier Atlas*; *Tome deuxième Atlas*; *Tome troisième Atlas*; *Tome quatrième Atlas*” and dated 1868. Only the following plates could be dated with confidence at this time based on Sherborn’s notes: plates 1–18 (vol. 4) 1854; 19–30 (vol. 4) 1855; 31–34 (vol. 1) 1855; 35–41 (vol. 1) 1856; 1–16 (vol. 2) 1856; 17–33 (vol. 2) 1857. Scientific names are provided on the plates.

**1857**. Coleopteros. Pp. 1–136 *in*: *Historia fisica politica y natural de la Isla de Cuba por D. Ramon de la Sagra. Segunda parte. Historia natural. Tomo VII. Crustaceos aragnides é insectos.* Arthus Bertrand, Paris. xxxii + 371 pp. [+ 20 associated pls]. (4to-text; Folio-plates) [DP: 1856 (title page; *advertencia* dated 20 September 1857; p. [350] dated October 1857)] BHL

The entire series was published in Spanish, in 4to, in 12 volumes between 1837 and 1857, and in French (see next entry). The Spanish version was issued in 190 fascicles (Hidalgo 1868: 258) and each fascicle usually consisted of 32 to 40 pages of text and four plates (Aguayo 1946: 158). Collation and dates of publication for each fascicle are desired.

Considering the date for the *advertencia* and that on page [350] it looks like the date of 1856 on the title page of tome 7 is an error for 1857 as mentioned by Woodward (1913: 1781). However Aguayo (1946: 174) argued that most likely the first fascicles of tome 7 of the Spanish version came out, as for the French version, in 1856 and that the volume was finished in 1857. Evenhuis (1997b: 679) treated the Spanish and French versions as published simultaneously, until further evidence is found.

Volume 8 is the *Atlas de Zoologia* and its title page is dated 1855. The Coleoptera are on plates “Articulata (Insecta)” 6–11. Scientific names are provided for the insects illustrated on each plate. Until the dates of publication and collation are established for the fascicles, it is not possible to determine if the plates were really issued before the text.

Many new species are described and illustrated in this work and, based on the evidence available at this time, I believe it is preferable to date them from 1857 as done by others (e.g., Low et al. 2013: 115).

**1857**. Ordre des Coléoptères, Lin. Pp. 1–328 *in*: *Histoire physique, politique et naturelle de l’Ile de Cuba par Ramon de la Sagra. Seconde partie: histoire naturelle. Tome septième.* Arthus Bertrand, Paris. lxxxvii + 868 pp. [+ 20 associated pls]. (8vo-text; Folio-plates) [DP: 1857 (title page; page 842 dated 10 October 1857), 24 October 1857 (*Bibl Fr*), 13 November 1857 (*Presse*), 22 December 1857 (*Acad Nat Sci Phila*)^55^] MCZ

This edition was issued in 80 fascicles forming 11 volumes in 8vo and 9 atlases in folio (Lorenz 1869: 163).

Blackwelder (1957: 1139) stated, without qualification, that pages 1–136 of Jacquelin du Val’s section were published in 1856 and pages 137–328 in 1857. However, this could not be confirmed in this study. In fact it seems that pages 1–136 referred to by Blackwelder concern the Spanish version.

According to Evenhuis (1997b: 679), livraisons 78–80 form volume 7 of the series.

**1859–1860**. *Glanures entomologiques ou recueil de notes monographiques, descriptions, critiques, remarques et synonymies diverses.* E. Donnaud, Paris. (18mo) CNC, BHL

*Cahier 1.* 60 pp. + 1 pl. [DP: 25 October 1859 (title page; preface dated 10 October 1859), 12 November 1859 (*Bibl Fr*)].

*Cahier 2.* Pp. 61–164. [DP: 18 May 1860 (title page), 16 June 1860 (*Bibl Fr*), July 1860 (*Allg Bibl*)].

#### Janson, Edward Wesley (London, United Kingdom: 12 March 1822 – 14 September 1891: London, United Kingdom). British natural history agent, publisher, bookseller and entomologist (particularly interested in Elateridae) in London; curator of the collections of the Entomological Society of London (1850–1863) and then librarian until 1874; founded two journals, *Journal of Entomology* (1862–1866) and *Cistula Entomologica* (1869–1885); his collection was sold by his firm and the Elateridae, which contained the collections of several authors including Candèze, were acquired by F.D. Godman who presented them to the British Museum (Natural History) in 1903. References. Ratzeburg (1874: 270); Anonymous (1891a); Gilbert (2005: 43, P); Cambefort (2006: 196).

**1863**. *British beetles. Transferred from Curtis’s British entomology. With descriptions.* Bell and Daldy, London. vi + 58 pp. + 29 pls. (4to) [DP: 1863 (title page), 18 April 1863 (*Athenaeum*), 30 April 1863 (*Bookseller*), 15 May 1863 (*Publ Circ*), June 1863 (*Allg Bibl*), July–December 1863 (*Bibliot Hist-Nat*)] McG, BHL

This work consists of descriptions of all 259 species of beetles illustrated and discussed in Curtis’ British Entomology, 1824–1839 [*q.v.*] following by 29 plates, each with eight or nine illustrations of beetles extracted from the plates of the same work. The plates are identical to Curtis, 1858 [*q.v.*]; they came colored or not.

#### Janson, Oliver Erichson (London, United Kingdom: 1850 – 25 November 1926: Highgate, Middlesex, United Kingdom). British natural history dealer, publisher, and entomologist who specialized in Coleoptera (particularly Cetoniinae); collected in Iceland in 1906 and in Ireland during World War I; son of Edward Wesley Janson [*q.v.*]; his cetoniid collection of the world was acquired by Frans Titus Valck Lucassen and is now at the Nationaal Natuurhistorisch Museum in Leiden; his collection of British beetles was acquired by the Museum of Zoology, University of Cambridge. References. Anonymous (1927, P); Cambefort (2006: 196).

**1885**. On a new species of Coleoptera of the family Cetoniidae, from E. Timor. P. 496 *in*: *A naturalist’s wanderings in the eastern Archipelago, a narrative of travel and exploration from 1878 to 1883, by Henry O. Forbes, F.R.G.S. With numerous illustrations, from the author’s sketches, and descriptions by Mr. John B. Gibbs.* Sampson Low, Marston, Searle & Rivington, London. xix + 536 pp. (8vo) [DP: 1885 (title page; preface dated 30 January 1885), 9 May 1885 (*Spectator*; *Athenaeum*), 4 June 1885 (*Bookseller*)] GB

The species described is *Clinteria forbesi*.

The book was also issued the same year in New York by Harper & Brothers [GB, BHL]. It was republished by Oxford University Press in 1989 with the addition of an introduction by the Earl of Cranbrook.

A two-volume German translation of this work was published in 1886 by Costenoble in Jena under the title “*Wanderungen eines Naturforschers im Malayischen Archipel von 1878 bis 1883. Aus dem Englischen von Reinhold Teuscher*” [SBB].

**1890**. Cetoniidae. P. 54 *in*: *Scientific results of the second Yarkand mission; based upon the collections and notes of the late Ferdinand Stoliczka, Ph.D. Coleoptera. Published by order of the government of India.* Office of Superintendent of Government printing, Calcutta. 79 pp. + 2 pls. (4to) [DP: 1890 (title page; colophon dated 13 November 1890), 4 March 1891 (*Asia Soc Beng*), 10 June 1891 (*Vetens Akad*), 21 June 1891 (*Accad Lincei*), 6 August 1891 (*Nature*), 19 September 1891 [JD] (*Soc Imp Nat Moscou*)] CAKL, GB

#### Jekel, Henri (Paris, France: 21 September 1816 – 4 August 1891: Paris, France). French natural history dealer and coleopterist (mainly interested in Scarabaeidae and Curculionidae); sold the remaining of his holdings to Achille Deyrolle [*q.v.*] and Henri Donckier de Donceel in 1879 who scattered them. References. Constantin (1992: 46); Cambefort (2006: 203).

**1849**. *C.J. Schoenherr, genera et species curculionidum. Catalogus.* H. Jekel, Parisiis. 279 pp. (12mo) [DP: 1849 (title page), 21 April 1849 (*Bibl Fr*), 3 May 1849 (*Lit Ztg*), 7 May 1849 (*Ent Soc Lond*), 27 June 1849 (*Soc Ent Fr*)] USNM, GB, BHL

This work is a straitforward catalogue based on Schönherr’s *Genera et species curculionidum*, 1833–1845 [*q.v.*].

**1853**. *Spécimen Fabricia Entomologica*. *Recueil d’observations nouvelles sur les insectes. Monographies, révisions de groupes et de genres. Classification, synonymies et rectifications. Descriptions de genres nouveaux et d’espèces nouvelles, &.* H. Jekel, Paris. 3 + 8 + 8 pp. (8vo) [DP: 1853 (title page; *préambule* dated April 1853)] MCZ

Besides the *préambule* (pp. 1–3), this work contains two sections on the genus *Lordops* Schönherr, one (pp. I.ii.1–8) dated February 1853, the second (I.iv.1–8) April 1853; these dates apparently refer to the months when the text was written.

[**1854]–1859**. *Fabricia Entomologica. Recueil d’observations nouvelles sur les insectes; monographies, révisions de groupes et de genres, classifications, synonymies et rectifications, descriptions de genres nouveaux et d’espèces nouvelles, &. Première partie.* H. Jekel, Paris. [3] + 249 pp. (8vo) CMLE, AMNH, BHL

This work was published in three livraisons: **1**: (pp. II.1–16; III.1–16; IV.1–16; V.1–16; VI.1–16)^56^ 1853 (title page, *préambule* dated April 1853), 26 August 1854 (*Bibl Fr*), 4 September 1854 (*Ent Soc Lond*), July–December 1854 (*Bibliot Hist-Nat*); **2**: (pp. 97–184*ter*) 1854 (title page), 13 June 1857 (*Bibl Fr*), 6 July 1857 (*Ent Soc Lond*), August 1857 (*Allg Bibl*), July–December 1857 (*Bibliot Hist-Nat*); **3**: (pp. 185–249) 1859 (title page), 28 May 1859 (*Bibl Fr*), 6 June 1859 (*Ent Soc Lond*), July 1859 (*Allg Bibl*), July–December 1859 (*Bibliot Hist-Nat*).

Despite the title page is dated 1853, the first livraison was likely not published until 1854. Similarly the second livraison was probably not issued until 1857.

**1855–1860**. *Insecta Saundersiana: or characters of undescribed insects in the collection of William Wilson Saunders, Esq., F.R.S., F.L.S., &c. Coleoptera. Curculionides.* John van Voorst, London. (8vo) CNC, GB

*Part I.* [1] + 153 + [1] pp. + 2 pls. [DP: 1855 (title page; preface dated 30 January 1855), 5 March 1855 (*Ent Soc Lond*)].

[*Part II*]. [1] + pp. 155–244 + [3] + 1 pl. [DP: 1860 (title page; preface dated March 1860), 7 May 1860 (*Ent Soc Lond*)].

**1873–1879**. *Coleoptera Jekeliana adjecta eleutheratorum bibliotheca. Enumération systématique & synonymique des coléoptères européens & exotiques composant la collection de Henri Jekel. Observations critiques. Descriptions d’espèces nouvelles. Reproduction et traduction de genres et espèces publiés dans des ouvrages rares français et étrangers de manière à former insensiblement la bibliothèque du coléoptériste.* Paris. 224 pp. (8vo) CMLE (livraisons 1, 2), GAL (livraisons 1, 2), GB (livraisons 1, 2)

This work, published by lithography of the original manuscript, was issued in three livraisons: **1**: (pp. 1–96) 1873 (title page), 23 July 1873 (*Soc Ent Fr*), 1 August 1873 (*Pet Nouv Ent*); **2**: (pp. 101–196) 1875 (title page; last page dated July 1875), 22 September 1875 (*Soc Ent Fr*), 1 October 1875 (*Pet Nouv Ent*), 3 November 1875 (*Ent Soc Lond*), 12 February 1876 [JD] (*Soc Imp Nat Moscou*); **3**: (pp. 201–224) 1879 (Derksen and Scheiding-Göllner 1965: 422) (*n.v.*).

This work includes: *Plan de l’ouvrage* (pp. 1–7) [last page dated March 1873]; *Catalogue* (pp. 9–18) [last page dated October 1872]; *Fam. Staphylinides* (pp. 19–50) [with pages 19–48 dated November 1872, pp. 49–50 dated December 1872]; *Catalogue des coléoptères recueillis en Syrie par Théodore Kotschy, énumérés ou décrits par L. Redtenbacher, avec quelques descriptions par Klug (Voyage de Joseph Russeger en Europe, Asie & Afrique de 1835 à 1841, Vol. I, p. 973–990, 1843, 8o avec 2 pl. col.)* (pp. 51–96) [with pp. 51–72 dated January 1873, pp. 73–96 dated February 1873]; *Curculionides* (pp. 101–196) [with pp. 101–124 dated April 1875, pp. 125–156 dated May 1875, pp. 157–188 dated June 1875, pp. 189–196 dated July 1875]. The only reference I have found to a third livraison is that of Derksen and Scheiding-Göllner (1965: 422) and no library seems to own a copy which raises the question whether or not it was actually published.

#### Joly, Nicolas (Toul, Meurthe-et-Moselle, France: 11 July 1812 – 17 October 1885: Toulouse, Haute-Garonne, France). French naturalist and anthropologist; professor of zoology, anatomy and comparative physiology at the Faculty of Sciences and the Medical School in Toulouse. References. Tissandier (1885); Clos (1886).

**1840**. *Histoire d’un petit crustacé (Artemia salina, Leach.) auquel on a faussement attribué la coloration en rouge des marais salans méditerranéens; suivie de recherches sur la cause réelle de cette coloration.* Boehm et Comp., Montpellier. 72 pp. + 3 pls. (4to) [DP: 1840 (title page), 9 March 1840 (*Acad Sci Fr*)] MCZ, GB

This work includes the description of a new species of beetle, *Hydroporus salinus* Joly, in a footnote on page 42.

#### Jordan, Heinrich-Ernst Karl (Almstedt, Lower Saxony, Germany: 7 December 1861 – 12 January 1959: Tring, Hertfordshire, United Kingdom). British (German-born) entomologist who wrote mainly on beetles, Lepidoptera, and fleas; curator and later director of the Walter Rothschild’s private museum in Tring; president of the International Commission on Zoological Nomenclature for 19 years; editor of the journal *Novitates Zoologicae* from 1894 to 1939; the Karl Jordan medal is awarded by the Lepidopterist Society at irregular intervals since 1973. References. Riley (1960); Zimmerman (1993: 589, P); Salmon (2000: 197–199, P); Johnson (2012).

**1897**. Carambycidae [sic!]. Pp. 452–454 *in*: *Through unknown African countries. The first expedition from Somaliland to Lake Lamu. By A. Donaldson Smith. Illustrated.* Edward Arnold, London [&] New York. xvi + 471 pp. (8vo) [DP: 1897 (title page), 13 March 1897 (*Athenaeum*; *Academy*; *Publ Circ*), March 1897 (*English Cat Books*; *Nat Nov*), 8 April 1897 (*Bookseller*)] CAKL, GB, BHL

This section is part of Appendix I (pp. 447–454) under the title “*Coleoptera. By Dr. Karl Jordan*” which includes the Scarabaeidae (pp. 448–452) by J.W. Shipp [*q.v.*] and the Cerambycidae by Jordan. The taxa described in Jordan’s contribution are: *Philagathes bipartitus* Jord. (p. 452); *Demagogus donaldsoni* Jord. (p. 452); *Rhaphidopsis guttata* Jord. (p. 453); *Cubilia* Jord., gen. nov. (Niphoniinarum) (p. 453); *Cubilia smithi* Jord. (p. 454).

The book was reissued the same year under the same title except that “*Lake Lamu*” is changed to “*Lake Rudolf*.” The book was reprinted by Greenwood Press, New York, in 1969.

**1898**. Appendix III. Some new Coleoptera discovered by Captain H.C. Webster. Pp. 380–381 *in*: *Through New Guinea and the cannibal countries. By H. Cayley-Webster. With illustrations and map.* T. Fisher Unwin, London. xvii + 387 pp. (8vo) [DP: 1898 (title page; preface dated 11 August 1898), 3 December 1898 (*Academy*), 10 December 1898 (*Athenaeum*; *Publ Circ*), December 1898 (*English Cat Books*)] GB

Three species of Cerambycidae are described in Jordan’s contribution. They were previously described in volume 5 of *Novitates Zoologicae* (pp. 419–420) issued in August 1898.

#### Jordana y Morera, Ramón (Cervera, Catalonia, Spain: 18 February 1839 – 1900: Madrid, Spain). Spanish forest engineer and naturalist; worked in the Philippines from 1873 to 1885. References. Casals Costa (1989: 382); Pinar (1999: 429).

**1885**. *Bosquejo geográfico é histórico-natural del Archipiélago Filipino.* Moreno y Rojas, Madrid. xiv + [1] + 461 pp. + 12 pls. (4to) [DP: 1885 (title page), November 1885 (*Nat Nov*)] GB

The Coleoptera are treated on pages 229–248 and a list of beetles of the Philippines is included.

#### Judeich, Johann Friedrich (Dresden, Saxony, Germany: 27 January 1828 – 28 March 1894: Tharandt, Saxony, Germany). German forester; director of the Academy of Forestry in Tharandt (1866–1894). References. Anonymous (1894a, P); Hess (1905).

**1889**. [Judeich, J.F. and Nitsche, H.] *Lehrbuch der mitteleuropäischen Forstinsektenkunde mit einem Anhange: Die forstschädlichen Wirbelthiere. Als achte Auflage von D^r^. J.T.C. Ratzeburg. Die Waldverderber und ihre Feinde. II. Abtheilung. Specieller Theil, I. Hälfte: Geradflügler, Netzflügler und Käfer. Mit 3 colorirten Tafeln, 77 Textfiguren und 3 illustrirten Bestimmungstafeln*. Eduard Hölzel, Wien. [5] + pp. 265–623 + 3 pls. (8vo) [DP: 1889 (title page), February 1889 (*Nat Nov*), March 1889 (*Centr Forst*), 22 June 1889 (*Lit Centr Deutsch*), June 1889 (*Naturwiss Rund*), 2 September 1889 (*Zool Anz*)] HATH

The entire work was issued in four parts, 1885–1895. The Coleoptera are in the second part on pages 281–616. The work was reissued in two volumes in 1895 [GB, BHL] by Paul Parey, Berlin.

#### Jung, Hugo Moritz Christian (Arnstadt, Thuringia, Germany: 29 January 1844 – 3 February 1919: Arnstadt, Germany). German businessman, educator and coleopterist; professor at the *Fürstlichen Realschule* of Arnstadt; his collections of Coleoptera and Lepidoptera are at the Arnstädter Schlossmuseum in Arnstadt. Reference. Thalmann (1929).

**1895–1896**. *Verzeichnis der in der Umgebung Arnstadts vorkommenden Käfer. Beilage zum Osterprogramm der Fürstlichen Realschule zu Arnstadt.* Bussjaeger, Arnstadt. v + 104 pp. (8vo) ULBD

[*Erster Theil*]. v + pp. 1–48. [DP: 1895 (title page), 15 July 1895 (*Bibl Monat*), 10 August 1895 (*Lit Centr Deutsch*), August 1895 (*Nat Nov*)].

*Zweiter Theil.* Pp. 49–104. [DP: 1896 (title page), 15 June 1896 (*Bibl Monat*), 12 September 1896 (*Lit Centr Deutsch*), October 1896 (*Nat Nov*)].

This work consists of an annotated catalogue of the Coleoptera in the vicinity of Arnstadt in Thuringia.

#### Jurine, Louis (Geneva, Switzerland: 6 February 1751 – 17 September 1819: Geneva, Switzerland). Swiss physician and naturalist; practiced medicine in Geneva and taught courses in anatomy and surgery at the *Académie Nationale de Médecine*; his collections are at the Natural History Museum of Geneva. References. Thillaye (1822); Thiele (1852); Marston (1999); Sigrist (1999, P); Damkaer (2002: 175–186).

**1803**. Dénomination des insectes rares que Mr. Jurine a trouvés dans la vallée de Chamonix et sur les montagnes qui l’environnent. Pp. 100–125 *in*: *Description des cols, ou passages des Alpes, par M.r Bourrit. Seconde partie.* G.J. Manget, Genève. iv + 213 pp. (8vo) [DP: “1803.–An XI” (title page), 17 November 1803 (*J Typogr Bibl*), February 1804 (*Esprit J*), 24 March 1804 (*Neue Intell Lit Kunst*), 16 June 1804 (*Gött Anz*)] GB, GAL

Jurine’s section is essentially a list of insects of the Chamonix Valley in eastern France and neighboring mountains with short annotations for some of them. A number of species of beetles are indicated as new (“nouvelle espèce”) and two of them are briefly described, *Silpha bicolor* (p. 101) and *Cryptocephalus trifasciatus* (p. 112); both names are junior primary homonyms.

Jurine’s contribution was translated in German and issued in Salis and Steinmüller (1807: 64–81). In the *Index Animalium*, *Silpha bicolor* is not compiled and *Cryptocephalus trifasciatus* is credited to “Salis, Alpina, II. 1807, 72” (Sherborn 1931: 6598).

A second edition of Bourrit’s work was issued in Geneva in 1828 under the title “*Guide du voyageur aux glaciers de Chamouni suivi de la description des Alpes. Seconde édition*” [GB]. It is essentially a reissued of the first edition with a change in the title page.

#### Kaeseberg, Karl. Professor at Wuppertal in Germany.

**1895**. Ueber die auf der Expedition des Grafen von Götzen 1893/94 gesammelten Coleoptera. Pp. 396–401 + 1 pl. *in*: *Durch Afrika von Ost nach West. Resultate und Begebenheiten einer Reise von der Deutsch-Ostafrikanischen Küste bis zur Kongomündung in den Jahren 1893/94 von G.A. Graf von Götzen. Mit zahlreichen Original-Illustrationen von W. Kuhnert und Sütterlin nach den Photographien, und 2 grossen Karten von Richard Kiepert nach den Original-Aufnahmen des Verfassers.* Dietrich Reimer, Berlin. xii + 417 pp. + 30 pls. (8vo) [DP: 1895 (title page; *Vorwort* dated 2 September 1895), December 1895 (*Nat Nov*), 15 February 1896 (*Lit Centr Deutsch*)] AMNH, GB, ARC

Two new species of beetles are described by Kaeseberg: *Aspidomorpha götzeni* (p. 400) and *Monolepta kerstingi* (p. 401).

A second edition of this work was issued in 1899 with xiv + 426 pages + 32 plates [BOT]. Kaeseberg’s section is on pages 404–409 and there are no changes from the first edition.

#### Karsch, Anton (Münster, North Rhine-Westphalia, Germany: 19 June 1822 – 15 March 1892: Münster, Germany). German physician and naturalist; practicing physician in Münster; professor of natural history at the Royal Academy in Münster. References. Ascherson (1892); Landois (1892, P); Wunschmann (1906).

**1862–1863**. *Die Insektenwelt. Ein Taschenbuch zu entomologischen Exkursionen für Lehrer und Lernende.* E.C. Brunn, Münster. lxx + 490 pp. (16mo) YB

This work was issued in two parts: (pp. i–xxiv + 1–224) 25 September 1862 (*Allg Bibl Deutsch*), 28 September 1862 (*Regens Morgen*); (pp. xxv–lxx + 225–490) January–June 1863 (*Bibliot Hist-Nat*). The title page is dated 1863 and the *Vorrede* 11 August 1862. The Coleoptera are on pages vii–xxiii and 9–114. A second edition was published in 1882–1883 (see next entry).

**1882–1883**. *Die Insektenwelt. Ein Taschenbuch zu entomologischen Exkursionen für Lehrer und Lernende. Zweite vermehrte und verbesserte, mit 389 Abbildungen in Holzschnitt bereicherte Auflage.* Otto Lenz, Leipzig. cxliv + 702 pp. (8vo) GB, ARC, BHL

This work was published in seven *Lieferungen* as follows: **1**: (pp. 1–96) April 1882 (*Nat Nov*); **2**: (pp. 97–192) July 1882 (*Nat Nov*); **3**: (pp. 193–288) October 1882 (*Nat Nov*); **4–5**: (pp. 289–576) March 1883 (*Nat Nov*); **6**: (pp. 577–671) July 1883 (*Nat Nov*); **7**: (pp. 673–702 + iii–cxxxvi) September 1883 (*Nat Nov*). The title page is dated 1883 and the *Vorwort* (p. iv) 11 August 1883. The Coleoptera are on pages xiii–xli and 7–182.

#### Karsch [Karsch-Haack], Ferdinand Anton Franz (Münster, North Rhine-Westphalia, Germany: 2 September 1853 – 20 December 1936: Berlin, Germany). German entomologist and anthropologist; curator at the Zoologisches Museum der Humboldt-Universität in Berlin (1878–1921) and lecturer in agricultural entomology at the Landwirtschaftlichen Hochschulke in Berlin; openly gay, he published numerous works on sexuality and particularly homosexuality. References. Anonymous (1937); Damm (2001).

**1881**. Gliederthiere der Expedition nach Kufra. Pp. 370–385 *in*: *Kufra. Reise von Tripolis nach der Oase Kufra. Ausgeführt im Auftrage der Afrikanischen Gesellschaft in Deutschland von Gerhard Rohlfs. Nebst Beiträgen von P. Ascherson, J. Hann, F. Karsch, W. Peters, A. Stecker. Mit 11 Abbildungen und 3 Karten.* F.A. Brockhaus, Leipzig. viii + 559 + [1 (Berichtigungen)] pp. + 21 tables (Meterologische Beobachtungen) + 7 pls. (8vo) [DP: 1881 (title page; *Vorwort* dated August 1881), October 1881 (*Nat Nov*; *Gesell Erdk Berl*), 10 November 1881 (*Blätt Lit Unterhalt*), 15 November 1881 (*Oest Monat Orient*), November 1881 (*Allg Bibl*), July–December 1881 (*Bibliot Hist-Nat*)] GB, BHL

This book relates the travels of Friedrich Gerhard Rohlfs [1831–1896] from Tripoli to the oasis of Kufra in central Libyan Desert. Several new species of Coleoptera are mentioned and described in Karsch’s contribution. However, all of them were previously described by Karsch in *Berliner Entomologische Zeitschrift* 25 [1881]: 41–50 [DP: April 1881 (title page)].

#### Katter, Friedrich Carl Albert (Gross-Bünzow, Mecklenburg-West Pomerania, Germany: 11 December 1842 – 22 April 1913: Lugano, Switzerland). German educator and entomologist; high school teacher at the *Pädagogium* in Putbus, on the island of Rügen, Germany (1873–1907); his collection was scattered. Reference. Kössler (2008b).

**1885**. *Monographie der europäischen Arten der Gattung Meloë mit besonderer Berücksichtigung der Biologie dieser Insekten. II. Theil: Beschreibung der Arten. Beilage zu dem Jahresbericht des Königlichen Pädagogiums zu Putbus über das Schuljahr 1884–85.* Otto Dornblüth, Bernburg. Pp. 35–61. (8vo) [DP: 1885 (title page), June 1885 (*Humboldt* 4: 338), July 1885 (*Nat Nov*), September 1885 (*Allg Bibl*), 1 October 1885 (*Wien Ent Ztg* 4: 255), 23 November 1885 (*Zool Anz*), 28 November 1885 (*Lit Centr Deutsch*), July–December 1885 (*Bibliot Hist-Nat*)] GB, ARC

One new species is described in this section, *Meloe carnicus* (p. 41). The first part, issued in 1883, deals with the biology of *Meloe*.

#### Kaup, Johann Jakob (Darmstadt, Hesse, Germany: 20 April 1803 – 4 July 1873: Darmstadt, Germany). German zoologist and paleontologist; assistant and later director of the natural history museum located in the palace of Grand Duke Ludewig I in Darmstadt. References. Anonymous (1873); Heldmann (1977); Adler (2007: 54, P).

**1866**. *Einige Cerambyciden der grossherzoglichen Sammlung zu Darmstadt. Beschrieben von J.J. Kaup. Abgebildet von Fr. Kerz.* Eduard Zernin, Darmstadt & Leipzig. [7] pp. + 3 pls. (4to) [DP: 1866 (title page; *Vorwort* dated March 1866)] YB (ph)

This publication was reproduced in 1895 [YB (ph)]. The new Cerambycidae taxa described are *Westwoodia* (p. [3]), *Westwoodia duivenbodei* (p. [3]), *Batocera wieneckei* (p. [4]), *Batocera rosenbergii* (p. [5]), *Batocera whitei* (p. [5]), Batocera (Apriona) punctatissima (p. [6]), Batocera (Apriona) flavescens (p. [6]), *Batocera deyrollei* (p. [6]), Batocera (Apriona) humeralis (p. [7]).

**1871**. *Monographie der Passaliden.* Nicolai, Berlin. 125 pp. + 5 pls. (8vo) [DP: 1871 (title page), January 1871 (*Berl Ent Zeit* 15: iii [footnote])] MCZ, GB

This work was issued as a supplement, with separate pagination, to the *Berliner Entomologische Zeitschrift*, forming *Heft* 4 of volume 15. It contains two sections, the *Monographie der Passaliden* itself (pp. 1–116) and the *Erster Nachtrag zur Monographie der Passaliden* (pp. 117–120).

A cover, dated 1871, was printed for the reprint [GB] which reads “*Monographie der Passaliden. Mit fünf lithographirten Tafeln nach Zeichnungen von Theodor Compton in Winscombe, Mitglied des Berliner Entomologischen Vereins.*”

#### Kelch, Johann August (Dąbie, Poland: 16 March 1797 – 26 August 1859: Racibórz, Poland). Prussian educator and naturalist; high school teacher in Racibórz for 40 years; part of his Coleoptera collection is at the Gymnasium in Racibórz while another part went to the Deutches Entomologisches Institut in Müncheberg via Karl Letzner. References. Anonymous (1860); Schmidt (1861: 571).

**1846**. Grundlage zur Kenntniss der Käfer Oberschlesiens, insonders der Umgegend von Ratibor. Pp. 1–54 *in*: *Zu der öffentlichen Prüfung aller Classen des Königlichen Gymnasiums zu Ratibor den 4. und 7. April, und dem mit Entlassung der Abiturienten verbundenen Redeactus den 20. April laden ergebenst ein Director und Lehrer-Collegium*. Bögner, Ratibor. [2] + 67 pp. (4to) [DP: 1846 (title page; last page dated 20 March 1846), 7 May 1846 (*Ent Ver Stettin*)] MCZ, GB

This publication consists of a list, with brief annotations, of the Coleoptera of Upper Silesia, particularly of the vicinity of Racibórz. A supplement was issued in 1852 by Kelch in the same school program (*n.v.*).

#### Kerremans, Charles (Brussels, Belgium: 18 December 1847 – 10 October 1915: Brussels, Belgium). Belgian military officer and coleopterist interested in the family Buprestidae; captain of infantry; assigned to the Belgian military department of cartography; his first collection of Buprestidae was purchased by the British Museum (Natural History), London, in 1903 while his second collection (including Madagascar but excluding the remaining African material) went to the Muséum National d’Histoire Naturelle, Paris, in 1923; the African material is at the Musée royal de l’Afrique centrale in Tervuren, Belgium. Reference. Rabaud (1915).

**1880**. *Catalogue des coléoptères de Belgique et des régions voisines.* A.-N. Lebègue et C^ie^, Bruxelles. vii + 67 pp. (8vo) [DP: 1880 (title page), November 1880 (*Bibl Belg*), 4 December 1880 (*Soc Ent Belg*), 5 December 1880 (*Soc R Bot Belg*), 8 December 1880 (*Soc Ent Fr*), 15 January 1881 (*Naturaliste*), January 1881 (*Nat Nov*)] YB (ph)

**1884**. *Énumération des buprestides décrits postérieurement au catalogue de MM. Gemminger & de Harold, 1870–1883.* P. Weissenbruch, Bruxelles. 43 pp. (8vo) [DP: 1884 (title page; preliminaries dated 1 April 1884), August 1884 (*Ent Nachr* 10: 265), September 1884 (*Nat Nov*)] GB

This work was also issued in *Annales de la Société Entomologique de Belgique* 29: 119–157 [DP: 1885 (title page), 13 May 1885 (*Soc Ent Fr*), May–June 1885 (*Soc R Belg Géog*), August 1885 (*Nederl Ent Ver*)].

#### Kiesenwetter, Ernst August Hellmuth von (Dresden, Saxony, Germany: 5 November 1820 – 18 March 1880: Dresden, Germany). German lawyer and entomologist; private councilor to the King of Saxony; his collection of Coleoptera was bought by Clément Müller of Dresden and is now at the Zoologische Staatssammlung des Bayerischen Staates in Munich. References. Kirsch (1880); Kraatz (1880, P); Smetana and Herman (2001: 85–86, P); Klausnitzer (2003: 104, P).

**1849**. [Kiesenwetter, E.A.H. von and Schaum, H.R.] *Catalogus coleopterorum Europae.* Bautzen. 82 + [7] pp. (8vo) [DP: 1849 (title page), 18 April 1849 (*Ent Ztg* 10: 96), 9 May 1850 (*Allg Bibl Deutsch*), 15 May 1850 (*Intell Serapeum*)] MCZ, GB, BHL

No author is listed on this publication but Kiesenwetter (1849: 98) mentioned that himself and Schaum, with the kind participation of Dohrn, Märkel and Suffrian, produced the catalogue.

**1856–1863**. *Naturgeschichte der Insecten Deutschlands begonnen von Dr. W.F. Erichson, fortgesetzt von Prof. Dr. H. Schaum, Dr. G. Kraatz und H.v. Kiesenwetter. Erste Abtheilung. Coleoptera. Vierter Band.* Nicolai, Berlin. vi + 745 + [1 (Berichtigungen)] pp. (8vo) CNC, BHL

This volume was issued in four *Hefte*: **1**: (pp. 1–172) 1857 (wrapper), 18 December 1856 (*Allg Bibl Deutsch*), July–December 1856 (*Bibliot Hist-Nat*), January 1857 (*Allg Bibl*), March 1857 (*Lit Deutsch Natur Ztg*); **2**: (pp. 173–384) May 1858 (back cover of wrapper), 24 June 1858 (*Allg Bibl Deutsch*), July 1858 (*Allg Bibl*); **3**: (pp. 385–568) 1860 (wrapper), 20 December 1860 (*Allg Bibl Deutsch*), January 1861 (*Allg Bibl*); **4**: (pp. 569–745 + [1] + vi) 1863 (wrapper; *Vorrede* dated *Sommer* 1863), 19 November 1863 (*Allg Bibl Deutsch*), November–December 1863 (*Allg Bibl*), July–December 1863 (*Bibliot Hist-Nat*).

**1877**. *Naturgeschichte der Insecten Deutschlands begonnen von Dr. W.F. Erichson, fortgesetzt von Prof. Dr. H. Schaum, Dr. G. Kraatz, H.v. Kiesenwetter, Julius Weise, Edm. Reitter und Dr. G. Seidlitz. Erste Abtheilung. Coleoptera. Fünfter Band. Erste Hälfte.* Nicolai, Berlin. 200 pp. (8vo) [DP: 1877 (wrapper), 22 September 1877 (*Academy*), 29 September 1877 (*Lit Centr Deutsch*), July–December 1877 (*Bibliot Hist-Nat*)] CNC, BHL

This volume was issued in five *Lieferungen*. The first one was authored by Kiesenwetter and the last four by Seidlitz, 1893–1898 [*q.v.*].

#### Kirby, William (Witnesham, Suffolk, United Kingdom: 19 September 1759 – 4 July 1850: Barham near Ipswich, Suffolk, United Kingdom). British clergyman and naturalist; rector of Barham for 68 years; founding member of the Entomological Society of London; his collection was given to the Entomological Society of London in 1835 and subsequently transferred to the Natural History Museum. References. Freeman (1852, P); Zimmerman (1993: 599, P); Smetana and Herman (2001: 86, P); Moore (2004b).

**1802**. *Monographia apum Angliae; or, an attempt to divide into their natural genera and families, such species of the Linnean genus Apis as have been discovered in England; with descriptions and observations. To which are prefixed some introductory remarks upon the class Hymenoptera, and a synoptical table of the nomenclature of the external parts of these insects. With plates. Vol. II.* J. Raw, Ipswich. 388 pp. + pls 15–18. (8vo) [DP: 1802 (title page), 1 June 1802 (*Monthly Mag*), June 1802 (*Monthly Epit*; Peddie and Waddington 1914: 322), September 1802 (*Anti-Jac Rev Mag*)] GB, BHL

This work consists of two volumes, dealing with bees, issued simultaneously. On page 168 of the second volume, the author described a meloid beetle larva under the new scientific name *Pediculus melittae*.

**1815–1826**. [Kirby, W. and Spence, W.] *An introduction to entomology: or elements of the natural history of insects: with plates.* Longman, Hurst, Rees, Orme, and Brown [vols 1–2] / Longman, Rees, Orme, Brown, and Green [vols 3–4], London. (8vo) CNC

*Vol. I.* xxiii + [1] + 512 + [2] pp. + pls 1–3. [DP: 1815 (title page), 1 April–10 July 1815 (*British Rev* 6: 246), July 1815 (Sherborn 1922a: lxxiii; *Lond Med Phys J*), 1 August 1815 (*Monthly Mag*), August 1815 (*Eclectic Rev*; *Lit Panor*; *Christ Obs*), July–October 1815 (*Edinb Rev*)].

*Vol. II.* [2] + 529 + [1] pp. + pls 4–5. [DP: 1817 (title page), March 1817 (*Edinb Rev*), 1 April 1817 (*Monthly Mag*), June 1817 (*British Critic*), 10 July 1817 (*Monthly Lit Adv*), 1 August 1817 (*New Monthly Mag*)].

*Vol. III.* v + [3] + 732 pp. + pls 6–20. [DP: 1826 (title page), 7 January 1826 (*Lit Gaz*), January 1826 (Sherborn 1922a: lxxiii), February 1826 (*Monthly Mag*; *Ann Phil*; *Not Geb Natur*), March 1826 (*Monthly Rev*)].

*Vol. IV.* iv + 634 pp. + pls 21–30. [DP: 1826 (title page), 7 January 1826 (*Lit Gaz*), January 1826 (Peddie and Waddington 1914: 322), February 1826 (*Monthly Mag*; *Ann Phil*; *Not Geb Natur*), March 1826 (*Monthly Rev*)].

Eight editions of this work were published: second, 1816–1818 (dealing only with first two volumes); third, 1817–1822 (dealing with the first two volumes only); fourth, 1822–1826; fifth, 1828; sixth, 1843 (dealing with the first two volumes only); seventh, 1856 (dealing with the first two volumes issued in one volume); and eight, 1865 (dealing with the first two volumes issued in one volume). A German translation was published under the title “*Einleitung in die Entomologie oder Elemente der Naturgeschichte der Insecten*” [GB] in four volumes, 1823–1833, in Stuttgart and later Tübingen. A Dutch translation of volumes 1 and 2 of the fourth edition was published in 1829–1830 under the title “*Inleiding tot de entomologie*” (*n.v.*). According to Evenhuis (1997a: 417), a Russian translation was published, 1863, in Moscow (*n.v.*).

**1837**. *Fauna Boreali-Americana; or the zoology of the northern parts of British America: containing descriptions of the objects of natural history collected on the late Northern Land Expeditions, under command of captain Sir John Franklin, R.N. by John Richardson, assisted by William Swainson, and the Reverend William Kirby. Illustrated by several coloured engravings. Published under the authority of the Right Honourable the Secretary of State for colonial affairs.* Josiah Fletcher, Norwich. xxxix + 329 + [1] pp. + 8 pls. (4to) [DP: 1837 (title page), 14 October 1837 (*Athenaeum*), 23 October 1837 (Evenhuis 1997b: 646), 28 October 1837 (*Lit Gaz*), 6 November 1837 (*Ent Soc Lond*), 10 November 1837 (*Allg Bibl Deutsch*), November 1837 (*Bull Litt Étr*), 6 December 1837 (*Soc Ent Fr*)] CNC, GB

The half title-page reads “*Fauna Boreali-Americana. Part the fourth and last. The insects. by the Rev. William Kirby.*”

The entire *Fauna Boreali-Americana* was published in four volumes, 1829–1837, and authored by John Richardson [1786–1865], William Swainson and Williams Kirby. Kirby was the sole author of the fourth volume. According to Graesse (1863: 20), this volume was also issued, 1837, by E. Bohn in London, under the title “*Entomologia Boreali-Americana or a natural history of the insects of North America, most specially the provinces under the Dominion of Great-Britain, containing descriptions of the objects collected in the late northern expeditions under the command of Capt. Sir J. Franklin; complemented by an enumeration of all those taken in the arctic regions by Capt. Parry, Sir J. Ross and Back, as well as those described by O. Fabricius in his Fauna Grönlandica*” (*n.v.*). A facsimile of the book was issued in 1978 by Arno Press, New York, under the title “*Fauna Boreali-Americana or the zoology of the northern parts of British America. Part four. Insects. John Richardson, William Swainson and William Kirby*” [CMLE].

The descriptions of the insects were reissued, along with occasional remarks of Charles James Stewart Bethune, under the title “*Insects of the northern parts of British America. Compiled by Rev. C. J. S. Bethune from Kirby’s Fauna Boreali-Americana: Insecta*” in *The Canadian Entomologist* volumes 2–8, 1870–1876.

#### Kirby, William Forsell (Leicester, Leicestershire, United Kingdom: 14 January 1844 – 20 November 1912: London, United Kingdom). British entomologist, mostly interested in Lepidoptera; assistant naturalist at the Museum of the Royal Dublin Society (1867–1879); curator at the British Museum (Natural History) in London (1879–1909). References. Kirby (1912, P); Morice (1913: clxvi–clxviii); Harvey et al. (1996: 120–121); Bragg (2007b, P).

**1885**. *Elementary text-book of entomology. With 87 plates containing over 650 figures.* W. Swan Sonnenschein and Co., London. viii + 240 + [1] pp. + 87 pls. (8vo) [DP: 1885 (title page), 9 May 1885 (*Athenaeum*; *Spectator*), 4 June 1885 (*Bookseller*), June 1885 (*Nat Nov*; *Allg Bibl*), 18 July 1885 (*Academy*)] GB, BHL

The Coleoptera are on pages 15–80. A second edition was issued in 1892 [BHL].

#### Kliemstein, Josef (Linz, Upper Austria, Austria: 1791 – 1861: Attersee, Upper Austria, Austria). Austrian physician in Gmunden. Reference. Oberhammer (1983: 21).

**1817.** *Dissertatio inauguralis enumerans genera Coleopterorum in Duftschmid M. Dr. Fauna Austriae adhuc descripta quam annuentibus illustrissimo ac magnifico domino praeside et directore ac clarissimis D.D. professoribus pro Doctoris Medici laurea obtinenda in antiquissima et celeberrima Universitate Vindobonensi publicae disquisitioni submittit Josephus Kliemstein, Austriacus Lincensis. In Theses adnexas disputabitur in Universitatis Palatio. Die Mensis Anni 1817.* Feichtinger, Lincii. viii + 9–30 pp. (4to) [DP: 1817 (title page)] GB

The digitized copy I have seen has “Julii” handwritten between “Mensis” and “Anni” on the title page. The thesis was reissued under the same title in 1818 [GB]. It is essentially a list of the genera in the first two volumes of Duftschmid’s *Fauna Austriae* with short latin descriptions.

#### Klika, Josef (Nesvačily [currently part of Rožmitál pod Třemšínem], Czech Republic: 23 December 1833 – 9 October 1873: Kutná Hora, Czech Republic). Bohemian educator and naturalist; taught in the cities of Loket (1856–1857), Pardubice (1858–1872) and Kutná Hora (1872–1873), all currently in Czech Republic. Reference. Anonymous (1874c).

**1873**. *Brouci. Soustavný popis brouků* *ve střední Evropě, zvláště v Čechách žijících, se zvláštním vzhledem ku škodě, kterou brouci a larvy jejich činí, a s vytknutím prostředkův, jimiž lze škodu tuto zameziti aneb aspoň umírniti. Ku potřebě učitelůvi i žákův, polních hospodářův, zahradnikův, lesníkův, sběratelů broukův a přátel přírodních věd vůbec. Připojen návod ku zřizeni sbírky broukův. S 8 rytinami a 8 tabulkami, obsahujícími malované obrázky broukův.* I.L. Kober, Praze [= Prague]. xxiv + 448 pp. + 8 pls^57^. (8vo) [DP: 1873 (title page; preliminaries dated March 1873)] SME

A second title page reads “*Přírodopis názorný pro školu I dům. Čásť druhá. Brouci.*” This work treats the beetles of Central Europe, particularly those found in Bohemia. The first part of the series was published in 1870 and pertains to Lepidoptera.

#### Kliment, Josef (Chlumec nad Cidlinou, Czech Republic: 6 April 1859 – 11 March 1927: Nĕmecký Brod [currently Havlíčkův Brod], Czech Republic). Bohemian school teacher and coleopterist in Havlíčkův Brod. References. Koleška (1984: 179–180); Anonymous (2015f).

**1894–1899**. *Čeští brouci. Dílo o broucích Čech, Moravy a Slezska. (Přírodopis brouků střední Evropy.) Obsahuje 69 řádů, 251 čeleď, 1002 rody, 5181 druh (odrůdu a odchylku). Se 2481 vyobrazeními brouků na 46 barvotiskových tabelích, 4 litografovanými tabulkami ku části všeobecné. Obrazy maloval Vladimír Zoufal, profesor v Prost*ĕ*jov*ĕ. *Ku spisu jsou přiloženy 44 listy štítků českolatinských jmen popsaných jedinců.* Františke Riedl, Nĕmeckém Brodĕ [= Havlíčkův Brod]. xvi + 811 pp. + 50 [A-D, 1–46] pls. (8vo) SME

This work, treating the beetles of Bohemia, Moravia and Silesia, was published in 47 issues (sešit) as follows: **1**: (pp. 1–16 + pls 1–2) 1894 (Český Katalog Bibliografický, za rok 1894 [1895]: 53), 1 December 1894–20 February 1895 (*Čas Mus Král České* 69: 167), February 1895 (*Zool Anz*), March 1895 (*Nat Nov*); **2–4**: June 1895 (*Českos Akad Věd*); **5–9**: October 1895 (*Českos Akad Věd*); **10–11**: December 1895 (*Českos Akad Věd*); **12–15**: April 1896 (*Českos Akad Věd*); **16**: June 1896 (*Českos Akad Věd*); **17–20**: October 1896 (*Českos Akad Věd*); **21–22**: 1 October–31 December 1896 (*Čas Mus Král České* 70: 598), January 1897 (*Českos Akad Věd*); **23**: January 1897 (*Českos Akad Věd*); **24–26**: April 1897 (*Českos Akad Věd*); **27–30**: 1 June–30 September 1897 (*Čas Mus Král České* 71: 487), November 1897 (*Českos Akad Věd*); **31–32**: November 1897 (*Českos Akad Věd*); **33–34**: 1 October–31 December 1897 (*Čas Mus Král České* 72: 103), January 1898 (*Českos Akad Věd*); **35–38**: May 1898 (*Českos Akad Věd*); **39–42**: May–December 1898; **43–44**: December 1898 (*Českos Akad Věd*); **45–47**: April 1899 (*Českos Akad Věd*). The title page is dated 1899 and the preliminaries January 1899. The content of each part is desired.

#### Klug, Johann Christoph Friedrich (Berlin, Germany: 5 May 1775 – 3 February 1856: Berlin, Germany). German physician and entomologist; professor of medicine and entomology at the University in Berlin and curator of the insect collections at the Zoological Museum of the University; his collection is at the Zoologisches Museum der Humboldt-Universität in Berlin. References. Gerstaecker (1856); Anonymous (1857: liv); Hess (1882a); Evenhuis (1997a: 419, P).

**1824**. *Entomologische Monographieen. Mit 10 illuminirten Kupfertafeln.* G. Reimer, Berlin. xiv + 242 pp. + 10 pls. (8vo) [DP: 1824 (title page; *Vorrede* dated September 1824), January–June 1825 (*Verz Bücher Landkarten*), October 1825 (*Allg Lit Ztg*)] CNC, BHL, GB

This work consists of monographies of the Coleoptera genera *Ctenostoma* (pp. 1–8), *Agra* (pp. 9–42), *Megalopus* (pp. 43–84), *Chlamys* (pp. 85–160), *Mastigus* (pp. 161–168) and of some Hymenoptera genera (pp. 169–232). Pages 233–239 consist of a supplement to the monography of the genus *Chlamys* owing to the publication of Kollar’s *Monographia chlamydum* [*q.v.*] in 1824.

**1829**. *Preis-Verzeichniss vorräthiger Insectendoubletten des Königl. zoologischen Museums der Universität.* Berlin. 18 pp. (8vo) [DP: 1829 (title page)] MCZ

This is a catalogue of the insect duplicates in the zoological museum of the Berlin University (currently the Humboldt University of Berlin) with prices. Many new Coleoptera species are briefly described and a few genus-group names are made available for the first time. A facsimile was printed in 1929 [CNC, HATH].

**1829–1845**. *Symbolae physicae, seu icones et descriptiones insectorum, quae ex itinere per Africam borealem et Asiam occidentalem Friderici Guilelmi Hemprich et Christiani Godofredi Ehrenberg studio novae aut illustratae redierunt. Precensuit Dr. Fr. Klug regis iussu et impensis eddit Dr. C.G. Ehrenberg.* Mittler, Berolini [= Berlin]. (Folio) McG

*Decas primas.* [33] pp. + pls 1–10. [DP: 1829 (cover), February–May 1830 (*Foreign Quart Rev*), January–June 1830 (*Verz Bücher Landkarten*)].

*Decas secunda.* [23] pp. + pls 11–20. [DP: 1830 (cover), January–June 1831 (*Verz Bücher Landkarten*), 6 August 1831 (*Bibl Deutsch*), July–December 1831 (*Verz Derj Bücher*)].

*Decas tertia.* [40] pp. + pls 21–30. [DP: 1832 (cover), 1 October 1832 (*Soc Ent Fr*), July–December 1832 (*Verz Bücher Landkarten*)].

*Decas quarta.* [40] pp. + pls 31–40. [DP: 1834 (cover), December 1834 (*Nouv Rev Germ*), July–December 1834 (*Verz Bücher Landkarten*), 28 January 1835 (*Lit Ztg*)].

*Decas quinta.* [41] pp. + pls 41–50. [DP: 1845 (cover), March 1845 (*Intell Allg Lit Ztg*), 5 June 1845 (*Allg Bibl Deutsch*), 14 June 1845 (*Lit Ztg*), April–June 1845 (*Foreign Quart Rev*), January–June 1845 (*Verz Bücher Landkarten*), 15 August 1845 (*Leip Reper*)].

Each part consists of unnumbered pages of text and ten plates. These decads form the third part of the zoology section of Ehrenberg’s “*Symbolae physicae*” (Bauer 2000: 11). Klug alone wrote the five decads relating to insects while Ehrenberg wrote the text for the other three zoological sections (Mammalia, Aves and Animalia evertebrata). A list of all insects described by Klug in the *Symbolae Physicae* was published by Baker (1997a: 181–193).

The specimens described by Klug originated from the natural history expedition of the German Friedrich Wilhelm Hemprich [1796–1825] and Christian Gottfried Ehrenberg [1795–1876] to Egypt and the Middle East during the period 1820–1825.

**1833**. *Bericht über eine auf Madagascar veranstaltete Sammlung von Insecten aus der Ordnung Coleoptera. Eine in der Königl. Akademie der Wissenschaften am 29. März 1832 gelesene Abhandlung. Mit fünf illuminirten Tafeln.* Königlichen Akademie der Wissenschaften, Berlin. 135 pp + 5 pls. (4to) [DP: 1833 (title page; page 132 dated 13 November 1833), 7 May 1834 (*Soc Ent Fr*), July 1834 (*Intell Jena Lit Ztg*), 4 August 1834 (*Ent Soc Lond*), July–December 1834 (*Verz Bücher Landkarten*)] MCZ, GB

This work contains the descriptions of many Coleoptera, including several new ones, brought back by Jules Prosper Goudot from his stay on the east coast of Madagascar and left at the museum of the University of Berlin.

Klug’s contribution was also issued in *Abhandlungen der Königlichen Akademie der Wissenschaften zu Berlin* (Jahre 1832): 91–223 + 5 pls [DP: 1834 (title page), July 1834 (*Intell Jena Lit Ztg*)].

**1834**. *Jahrbücher der Insectenkunde, mit besonderer Rücksicht auf die Sammlung im Königl. Museum zu Berlin. Erster Band. Mit 2 illuminirten Kupfertafeln.* Theod. Chr. Friedr. Enslin, Berlin. viii + 296 pp. + 2 pls. (8vo) [DP: 1834 (title page; *Vorrede* dated May 1834), 6 September 1834 (*Bibl Deutsch*), September 1834 (*Intell Allg Lit Ztg*), 19 November 1834 (*Soc Ent Fr*), August–November 1834 (*Foreign Quart Rev*), 20 December 1834 (*Gött Anz*), 31 December 1834 (*Lit Ztg*), July–December 1834 (*Verz Bücher Landkarten*)] MCZ, BHL, GB

Only the first part (*Band*) of this work was published. Four sections of this publication pertain to Coleoptera: I. Uebersicht der Cicindeletae der Sammlung (pp. 1–47); II. Uebersicht der Carabici der Sammlung (pp. 48–82); III. Uebersicht der Histeroides der Sammlung (pp. 83–208)^58^; IV. Die Arten der Gattung *Megalopus* (pp. 208–223). Several new species are described in each section.

**1835**. Insekten. Pp. 27–52 + pls XV–XVI *in*: *Reise um die Erde durch Nord-Asien und die beiden Oceane, in den Jahren 1828, 1829 und 1830 ausgeführt von Adolph Erman. Naturhistorischer Atlas.* G. Reimer, Berlin. vi + 64 pp. + 17 pls. (Folio) [DP: 1835 (title page; *Vorwort* dated 11 August 1835), 30 December 1835 (*Lit Ztg*), January–February 1836 (*Acad Imp Sci St-Pétersb*), January–June 1836 (*Verz Bücher Landkarten*)] CNC, GB

The second page is entitled “*Verzeichniss von Thieren und Pflanzen, welche auf einer Reise um die Erde gesammelt wurden von Adolph Erman. Mit XVII. Tafeln.*”

The account of Georg Adolf Erman’s round-the-world voyage was published in five volumes and one atlas, 1833–1848.

[**1842**]. [Klug, J.C.F. and Erichson, W.F.] [*Verzeichniss der verkäuflichen Doubletten des Berliner Museum*]. Berlin. 15 pp. (8vo) [DP: preliminaries dated 10 August 1842 (p. 2)] CNC (mf)

As far as I know, no title page was issued for this work. It consists of a list of mammals, birds, insects and molluscs duplicates in the zoological museum of the Berlin University with prices. The introductory matters are signed by Klug and the new species are signed “Kl[ug]” or “Er[ichson]”. The work is divided into the following sections: I. Säugethiere und Vögel (pp. 3–4); II. Insecten (pp. 4–13); III. Conchylien (13–14); Anhang (pp. 14–15).

The following new beetle species from Senegal are described in this pamphlet, all credited to Erichson: *Brachinus elegantulus* (p. 4), *Scarites troglodytes* (p. 4), *Hypolithus attenuatus* (p. 5), *Stenolophus micans* (p. 5), *Sphaeridium senegalense* (p. 6), *Hister calidus* (p. 6), *Aphodius russata* (p. 7), *Aphodius discolor* (p. 7), *Oxyomus granosus* (p. 7), *Philax senegalensis* (p. 8), *Allecula sanguinicollis* (p. 8), *Allecula spadicea* (p. 9), *Mylabris haemorrhoa* (p. 9), *Mylabris maculosa* (p. 9), *Cryptocephalus oblitus* (p. 10), *Raphidopalpa vinula* (p. 10), *Callichroma opulenta* (p. 10). The descriptions were reproduced by Dohr (1859).

**1850**. *Verzeichniss verkäuflicher Doubletten der entomologischen Sammlung der Königlichen Universität zu Berlin.* Berlin. 20 pp. (8vo) [DP: n.d. (title page; preliminaries dated 28 January 1850), 19 October 1850 [JD] (*Soc Imp Nat Moscou*)] YB (ph)

Several new species are mentioned in this list but none are described.

**1862**. Coleoptera. Käfer. Pp. 145–267 + pls 8–15 *in*: *Naturwissenschaftliche Reise nach Mossambique auf Befehl seiner Majestät des Königs Friedrich Wilhelm IV in den Jahren 1842 bis 1848 ausgeführt von Wilhelm C.H. Peters. Zoologie. V. Insecten und Myriopoden. Bearbeitet in Verbindung mit Klug, Loew, Schaum, Hagen, Gerstaecker, Hopffer. Mit fünf und dreissig Kupfertafeln.* Georg Reimer, Berlin. xxi + 566 pp. [+ 35 associated pls]. (4to–text; Folio–plates) [DP: 1862 (title page; *Vorwort* dated December 1861), 31 July 1862 (*Allg Bibl Deutsch*; *Intell Serapeum*), July 1862 (Evenhuis 1997b: 612), August 1862 (*Allg Bibl*), 1 September 1862 (*Ent Soc Lond*), September 1862 (*Rev Germ*), July–December 1862 (*Bibliot Hist-Nat*; *Verz Bücher Landkarten*)] CNC, GB, BHL

The natural history report of Wilhelm Peters’ natural history exploration in Mozambique was published in nine volumes, 1852–1882. The Coleoptera are in the fifth volume of the zoology section, on pages 145–348. Carl Eduard Adolph Gerstaecker [*q.v.*] authored pages 268–348.

#### Kneifl [Kneifel], Reginald (Nieder-Lindewiese [currently Lipová-lázně], Czech Republic: 11 January 1761 – 7 December 1826: Vienna, Austria). Austrian naturalist; professor of zoology and mineralogy and librarian at the private boarding school Theresianum in Vienna (1805–1824). Reference. Wurzbach (1864: 143).

**1811**. *Das Thierreich. Ein Handbuch für die Hörer der Philosophie.* Geistinger, Wien und Triest. vi + [2] + 360 pp. (8vo) [DP: 1811 (title page)] GB

The Coleoptera are on pages 214–235. A second “*Auflage*” was issued also in Vienna in 1819 unde the title “*Das Thierreich mit systematischer Darstellung der für das Schulbuch in dem k.k. Gymnasium gelieferten Abbildungen auch als Leitfaden bey Vorlesungen brauchbar*.” The two editions are essentially identical.

#### Knoch, August Wilhelm (Brunswick, Lower Saxony, Germany: 8 June 1742 – 2 June 1818: Brunswick, Germany). German naturalist; professor of physics and mineralogy at the Collegium Carolinum in Brunswick; his Coleoptera collection is at the Zoologisches Museum der Humboldt-Universität in Berlin. References. Zincken (1818); Poggendorff (1863: 1281); Hess (1882b).

**1781–1783**. *Beiträge zur Insektengeschichte*. Schwickert, Leipzig. (8vo) GDZ, BHL

*I. Stück.* 10 + 98 + [3] pp. + 6 pls. [DP: 1781 (title page; preliminaries dated 8 February 1781)]. This part treats several species of Lepidoptera and the beetles *Curculio albinus* (pp. 81–87) and *Curculio nebulosus* (pp. 87–93).

*II. Stück.* viii + 102 + [2] pp. + 7 pls. [DP: 1782 (title page; preliminaries dated “1sten Ostermond 1782”), 26 April 1783 (*Gött Anz*)]. This part treats several species of Lepidoptera and the beetle *Scarabaeus hemipterus* (pp. 95–98).

*III. Stück.* [2] + 138 + [2] pp. + 6 pls. [DP: 1783 (title page)]. This part treats several species of Lepidoptera.

A fourth *Stück* was planning (Anonymous 1785: 230) but not published.

**1801**. *Neue Beyträge zur Insectenkunde. Mit Abbildungen. Erster Theil.* Schwickert, Leipzig. xii + 208 pp. + 9 pls. (8vo) [DP: 1801 (title page), 17 August 1802 (*Allg Lit Ztg*), 23 October–21 November 1802 (*J Lit Fr*)] CNC, BHL, GB

Only the first part (*Theil*) was issued. It contains the descriptions of a number of new Coleoptera genera and several new species.

#### Kolbe, Hermann Julius (Halle, North Rhine-Westphalia, Germany: 2 June 1855 – 26 November 1939: Berlin, Germany). German entomologist; curator at the Zoologisches Museum der Humboldt-Universität in Berlin (1890–1921); his personal collection was scattered but his Coleoptera types are in the Zoologisches Museum in Berlin. Reference. Sachtleben (1940).

**1890**. Die Käfer des Kilimandscharo-Gebietes. Pp. 336–338 *in*: *Ostafrikanische Gletscherfahrten. Forschungsreisen im Kilimandscharo-Gebiet. Von Dr. Hans Meyer. Mit 3 Karten, 20 Tafeln in Heliogravüre und Lichtdruck und 19 Textbildern.* Duncker & Humblot, Leipzig. xiv + 376 pp. + 21 pls. (8vo) [DP: 1890 (title page; *Vorwort* dated “Herbst 1890”), 6 December 1890 (*Gesell Erdk Berl*), December 1890 (*Nat Nov*), 19 January 1891 (*Ausland*), 14 February 1891 (*Deutsch Litt*)] GB, ARC

Several new species are named in the catalogue of the species collected by Hans Meyer in the Kilimanjaro region but none are described.

An English translation was published in 1891 by Longmans, Green, and Co., London, under the title “*Across East African glaciers. An account of the first ascent of Kilimanjaro*” [GB].

**1897**. *Coleopteren. Die Käfer Deutsch-Ost-Afrikas. Mit 4 Taflen, gezeichnet von Nic. Prillwitz.* Dietrich Reimer, Berlin. 367 + [1] pp. + 4 pls. (8vo) [DP: 1897 (title page)] BHL

This publication was issued in three parts in 1897 as follows: (pp. 1–160) April 1897 (*Nat Nov*), 14 June 1897 (*Bibl Zool*); (pp. 161–320) June 1897 (*Nat Nov*), 16 August 1897 (*Bibl Zool*); (pp. 321–368) December 1897 (*Nat Nov*), 24 January 1898 (*Bibl Zool*). Kolbe’s contribution was also issued in 1898 in “*Deutsch-Ost-Afrika. Wissenschaftliche Forschungsresultate über Land und Leute unseres ostafrikanischen Schutzgebietes und der angrenzenden Länder. Band IV. Die Thierwelt Ost-Afrikas und der Nachbargebiete. Herausgegeben unter Redaktion von Professor Dr. K. Möbius. Wirbellose Thiere*” (*n.v.*).

**1898**. Coleoptera gesammelt von Herrn Premier-Lieutenant Werther in der Massai-Steppe. Pp. 305–310 *in*: *Die mittleren Hochländer des nördlichen Deutsch-Ost-Afrika. Wissenschaftliche Ergebnisse der Irangi-Expedition 1896–1897 nebst kurzer Reisebeschreibung. Im Auftrage der Irangi-Gesellschaft herausgegeben von dem Führer der Expedition C. Waldemar Werther. Unter Mitwirkung der Herren: Dr. Bruno Hassenstein, Professor Dr. F. Karsch, H.J. Kolbe, Professor Dr. F. von Luschan, P. Matschie, Professor Dr. A. Reichenow, A. Seidel, L. von Tippelskirch, Dr. G. Tornier, Dr. E. Wagner und G. Witt. Mit 5 Vollbildern und 126 Text Illustrationen in Photogravüre, Lichtdruck, Lithographie und Autotypie sowie 2 Original-Karten von Dr. B. Hassenstein und Prem.-Lieut. Werther.* Hermann Paetel, Berlin. 8 + 493 + [1] pp. (8vo) [DP: 1898 (title page; *Vorwort* dated January 1898), July 1898 (*Nat Nov*), August 1898 (*Allg Bibl*), July–August 1898 (*Luz Orient List*)] GB, ARC, SDL

Besides six species already known, one new species, *Macropoda neumanni* (pp. 307–308), is also described from Waldemar Werther’s expedition to northern Tanzania.

#### Kolenati, Friedrich Anton (Prague, Czech Republic: 12 April 1812 – 17 July 1864: Ovčárna pod Pradědem, Czech Republic). Bohemian naturalist; assistant professor of botany at the University of Prague; assistant in zoology at the Academy of Sciences in Saint Petersburg (1842–1846); professor of zoology, botany, mineralogy, and paleontology at the Polytechnic Institute in Brno; the bulk of his collection is at the Polytechnic Institute in Brno but some of his Coleoptera (including types) are at the Natural History Museum in Prague. References. Poggendorff (1863: 1301–1302); Wurzbach (1864: 316–319); Flasar (1997); Smetana and Herman (2001: 89–90, P).

**1845–1846**. *Meletemata entomologica.* Imperialis Academiae Scientarium, Petropoli [= Saint Petersburg]. (8vo) CMLE, SME, BHL, GB

*Fasc. I. Accedunt tabulae II coloratae.* [2] + 88 pp. + 2 pls. [DP: 1845 (title page; *prooemium* dated June 1845), August 1845 (bottom of first page of each sheet), 15 November 1845 [JD] (*Soc Imp Nat Moscou*), 22 January 1846 (*Allg Bibl Deutsch*), 30 January 1846 (*Leip Reper*), 15 February 1846 (*Intell Serapeum*), January–April 1846 (*Foreign Quart Rev*)]. Another title page reads “*Insecta Caucasi cum distributione geographica. Coleopterorum pentamera carnivora. Accedunt tabulae II coloratae.*”

*Fasc. III. Accedunt tabulae III.* [3] + 44 pp. + pls 12–14. [DP: 1846 (title page; *praefamen* dated 1 December 1845), January 1846 (bottom of first page of each sheet), 4 June 1846 (*Ent Ver Stettin*), 19 September 1846 [JD] (*Soc Imp Nat Moscou*), 14 October 1846 (*Soc Ent Fr*), 17 December 1846 (*Allg Bibl Deutsch*), 31 December 1846 (*Intell Serapeum*), July–December 1846 (*Verz Bücher Landkarten*)]. Another title page reads “*Brachelytra Caucasi cum distributione geographica adnexis pselaphinis, scydmaenis, notoxidibus et xylophagis. Accedunt tabulae III coloratae.*”

*Fasc. V. Accedunt tabulae III.* [4] + iii + 169 + [1] pp. + pls 17–19. [DP: 1846 (title page; page iii dated May 1846), June 1846 (bottom of first page of each sheet), 19 September 1846 [JD] (*Soc Imp Nat Moscou*), 9 October 1846 (*Ent Ver Stettin*), 14 October 1846 (*Soc Ent Fr*), 8 July 1847 (*Allg Bibl Deutsch*), 15 July 1847 (*Intell Serapeum*), 6 August 1847 (*Leip Reper*)]. Another title page reads “*Insecta Caucasi. Coleoptera, Dermaptera, Lepidoptera, Neuroptera, Mutillidae, Aphaniptera, Anoplura. Accedit tabula: XVII. XVIII. XIX.*”

The work consists of five fascicles, the second and fourth do not treat Coleoptera. It was continued in volumes 29, 31 and 32 of the *Bulletin de la Société Impériale des Naturalistes de Moscou*.

#### Kollar, Vincenz (Krzanowice, Poland: 15 January 1797 – 30 May 1860: Vienna, Austria). Austrian entomologist; curator at the Naturhistorisches Museum in Vienna. References. Schiner (1860); Ratzeburg (1874: 290–291); Hess (1882c); Evenhuis (1997a: 424, P).

**1824**. *Monographia chlamydum. Cum tabulis aeneis coloratis duabus.* J.G. Heubner, Viennae. [2] + 49 pp. + 2 pls. (Folio) [DP: 1824 (title page; *praefatio* dated December 1823), June 1824 (*Isis*, Heft VI: [2]), February 1825 (*Allg Lit Ztg*)] BHL, GB

This work consists of a revision of the genus *Chlamys* Knoch (currently *Chlamisus* Rafinesque) in the family Chrysomelidae.

**1844**. [Kollar, V. and Redtenbacher, L.] Aufzählung und Beschreibung der von Freiherrn Carl v. Hügel auf seiner Reise durch Kaschmir und das Himaleyagebirge gesammelten Insecten. Mit 28 Steindruck-Tafeln. Pp. 395–564 + 28 pls *in*: *Kaschmir und das Reich der Siek, von Carl Freiherrn von Hügel. In vier Bänden. Vierter Band. Zweite Abtheilung.* Hallberger, Stuttgart. Pp. 247–581 + [6] + 28 pls. (8vo) [DP: 1844 (title page; page 402 dated March 1844)] McG, GB, MDZ

The entire report of the trip was published in four volumes, 1840–1848, the fourth one in two parts (*Abtheilungen*) continuously paginated, the first one (pp. 1–246) issued in 1842 (title page). The second *Abtheilung* was issued in two sections, with pages 247–581 published in 1844 (title page) and pages 587–865 in 1848 (title page). The Coleoptera are on pages 497–564 and plates 23–28. Hagen (1862: 430) dated Kollar and Redtenbacher’s contribution 1842, Horn and Schenkling (1928b: 656) 1848.

Authorship of the new species in Kollar and Redtenbacher’s contribution should be credited to both authors since there is nothing in the contents of Hügel’s work that would indicate that one or the other is responsible for the content of each order. However, the descriptions of eight new species of beetles, *Sternocera dasypleuros* (p. 504), *Agrilus caschmirensis* (p. 505), *Lacon brachychaetus* (p. 506), *Ludius caschmirensis* (p. 507), *Cardiophorus vicinus* (p. 507), *Cardiophorus consentaneus* (p. 508), *Onitis castaneus* (p. 517), *Onitis himaleyicus* (p. 518), have the name “Kollar” listed at the end. These species should be credited to Kollar alone.

**1849**. [Kollar, V. and Redtenbacher, L.] *Ueber den Charakter der Insecten-Fauna von Süd-Persien.* Wien. 12 pp. [DP: 1849 (title page)] GB

This work was also issued in *Denkschriften der kaiserlichen Akademie der Wissenschaften* (*mathematisch-naturwissenschaftliche Classe*) 1: 42–53 [DP: 1850 (title page)]. Volume 1 of this journal was published in two *Abtheilungen*. The contribution by Kollar and Redtenbacher was issued in the first *Abtheilung* and this section was published in 1849 (Stefan 1876: 208) and the second one in 1850. The date of 1850 is usually attributed to this work (e.g., Löbl and Smetana 2003: 744; 2010: 842; 2013: 628).

This work treats the Coleoptera (pp. 4–9) and the Lepidoptera (pp. 10–12). Several new species are described; all those on beetles are credited to Redtenbacher and those on Lepidoptera to Kollar.

#### Kotschy, Karl Georg Theodor (Ustroń, Poland: 15 April 1813 – 11 June 1866: Vienna, Austria). Austrian explorer and botanist; collected extensively in the Middle East and northern Africa; director of the Imperial Cabinet of Curiosities in Vienna (now the Naturhistorisches Museum Wien). References. Reichardt (1882); Rechinger (1960); Knoll (1969); Riedl-Dorn (2001).

**1864**. Uebersicht der von Cypern bisher gekannten Thiere. Pp. 570–590 *in*: *Die Insel Cypern: ihrer physischen und organischen Natur nach mit Rücksicht auf ihre frühere Geschichte geschildert von Dr. F. Unger und Dr. Th. Kotschy. Mit einer topographisch-geognostischen Karte, 42 Holzschnitten und einer Radirung.* Wilhelm Braumüller, Wien. xii + 598 pp. (8vo) [DP: 1865 (title page; preliminaries dated 16 October 1863), 15 November 1864 (*Intell Serapeum*), 8 December 1864 (*Allg Bibl Deutsch*)] GB

The work includes a list of the animals of Cyprus. The Coleoptera are on pages 574–590; several new species are mentioned, some named, but none are described. Despite the title page is dated 1865, this book was published in 1864.

Franz Unger and Theodor Kotschy wrote different sections of the book as indicated in the *Inhalt* (p. xi).

#### Kraatz, Gustav (Berlin, Germany: 13 March 1831 – 2 November 1911: Berlin, Germany). German entomologist interested mainly in Coleoptera; professor at the University of Berlin; founding member of the *Berliner Entomologische Gesellschaft*; long-time editor of the *Deutsche Entomologische Zeitschrift*; his collection is at the Deutsches Entomologisches Institut in Müncheberg. References. Horn and Zang (1906); Jordan (1910); Smetana and Herman (2001: 90–92, P); Wessel (2007, P).

**1856–1857**. *Naturgeschichte der Insecten Deutschlands begonnen von Dr. W. F. Erichson, fortgesetzt von Prof. Dr. H. Schaum, Dr. G. Kraatz und H. v. Kiesenwetter. Erste Abtheilung. Coleoptera. Zweiter Band.* Nicolai, Berlin. viii + 1080 pp. (8vo) MCZ, BHL

This work was published in six *Lieferungen*: **1–2**: (pp. 1–376) 1856 (wrapper), 20 June 1856 (Zerche 1987a: 92–93), 17 July 1856 (*Allg Bibl Deutsch*), August 1856 (*Allg Bibl*); **3–4**: (pp. 377–768) 1857 (wrapper), May 1857 (Kraatz 1859: vii), 10 September 1857 (*Allg Bibl Deutsch*), 30 November 1857 (*Intell Serapeum*), July–December 1857 (*Bibliot Hist-Nat*); **5–6**: (pp. 769–1080, i–vii) 1858 (wrapper), 8 November 1857 (*Ent Ver Stettin*), 10 December 1857 (*Allg Bibl Deutsch*), July–December 1857 (*Bibliot Hist-Nat*), 28 February 1858 (*Intell Serapeum*), February 1858 (*Allg Bibl*). The title page is dated 1858 and the *Vorrede* November 1857.

**1865**. *Revision der Tenebrioniden der alten Welt aus Lacordaire’s Gruppen der Erodiides, Tentyriides, Akisides, Piméliides, und der europäischen Zophosis-Arten.* Nicolai, Berlin. vi + 393 pp. (8vo) [DP: 1865 (title page), 16 March 1865 (*Allg Bibl Deutsch*), 31 March 1865 (*Bookseller*), May 1865 (*Allg Bibl*), January–July 1865 (*Bibliot Hist-Nat*)] CNC, GB

**1869**. *Verzeichniss der Käfer Deutschlands. Herausgegeben von dem Entomologischen Verein in Berlin. Als Beiheft zum Jahrgange 1869 der Berliner entomologischen Zeitschrift für die Mitglieder des Vereins.* Nicolai, Berlin. vi + 84 pp. (4to) [DP: 1869 (title page; *Vorwort* dated January 1869), April 1869 (*Allg Bibl*), 6 May 1869 (*Allg Bibl Deutsch*), 7 June 1869 (*Ent Soc Lond*)] CMLE, GB

This publication consists of a list of the Coleoptera of Germany.

**1873**. *Die Käfer Europas. Nach der Natur beschrieben von Dr. G. Kraatz im Anschluss an die Käfer Europa’s von Dr. H.C. Küster. Neunundzwanzigstes Heft. Mit Beiträgen von H. v. Kiesenwetter.* Bauer & Raspe, Nürnberg. iv + [104] pp. (12mo) [DP: 1873 (title page; *Inhalt* dated March 1873), March 1873 (Rye 1875: 227), 1 May 1873 (*Academy*), May 1873 (*Land Centr Deutsch*), September 1873 (*Allg Bibl*)] CNC

The pages are unnumbered. The first 28 *Hefte* of the series were authored by Küster, 1844–1854 [*q.v.*], the following ones by Schilsky, 1894–1912 [*q.v.*].

#### Krackowizer [Krakowizer], Josef Christian Serafin (Spital am Pyhrn, Upper Austria, Austria: 14 December 1814 – 23 February 1900: Steyr, Upper Austria, Austria). Austrian physician and botanist; practiced first in Stierning, later in Steyr. References. Rosenthal (1900); Anonymous (2015c, P).

**1842**. *Dissertatio inauguralis sistens enumerationem systematicam curculionidum Archiducatus Austriae, praecipuorum synonymorum penu auctam, quam consensu et auctoritate illustrissimi ac magnifici domini praesidis et directoris, perillustris ac spectabilis domini decani, nec non clarissimorum ac celeberrimorum D.D. professorum pro doctoris medicinae laurea. Summisque in arte medica privilegiis et honoribus capessendis in antiquissima et celeberrima scientiarum Universitate Vindobonensi publico eruditorum judicio submittit Josephus Krackowizer, oriundus ex Spital am Pyhrn in Austria superiore. In theses adnexas disputabitur in Universitatis aedibus die mensis Februarii 1842.* Caroli Ueberreuter, Vindobonae [= Vienna]. 82 pp. (8vo) [DP: February 1842 (title page), 6 September 1842 (*Ent Ver Stettin*)] CNC (mf)

This thesis consists of a list, with synonyms and references, of Austrian curculionides (*sensu lato*); no new taxa are made available in it.

#### Krassow, Carl Reinhold Adolf von (Stralsund, Mecklenburg-West Pomerania, Germany: 15 April 1812 – 13 February 1892: Pansevitz on the island Rügen, Mecklenburg-West Pomerania, Germany). Prussian civil servant and politician. Reference. Petrich (1906).

**1838**. [Krassow, C.R.A. von and Leyde, E.] *Lehrbuch der Zoologie für Gymnasien und höhere Bürgerschulen. Zweite Ausgabe.* Ernst Siegfried Mittler, Berlin, Posen und Bromberg. xii + 337 pp. (8vo) [DP: 1838 (title page), 22 June 1838 (*Allg Bibl Deutsch*), January–June 1838 (*Verz Bücher Landkarten*), 4 July 1838 (*Lit Ztg*)] GB

The book was also issued under the title “*Lehrbuch der Naturgeschichte für Gymnasien und höhere Bürgerschulen. Erster Theil. Zweite Ausgabe.*” The Coleoptera are on pages 184–198. The first edition (*n.v.*) was published in 1835 and noticed on 31 May 1835 (*Allg Bibl Deutsch*).

#### Kriechbaumer, Josef [Joseph] (Tegermsee, Bavaria, Germany: 21 March 1819 – 2 May 1902: Munich, Bavaria, Germany). German entomologist specialist of Hymenoptera and particularly Ichneumonidae; cantonal school teacher at Chur, Switzerland, for nine years; later, curator at the Zoological State Museum in Munich. References. Anonymous (1902); Konow (1902); Taschenberg (1902).

**1844**. *Uebersicht der cerambyciden Münchens. Dissertatio inauguralis.* München. iv + 22 pp. (8vo) [DP: 1844 (title page), 6 March 1846 (*Ent Ver Stettin*)] CMLE (mf), GB

This thesis presents an overview of the Cerambycidae of Munich. One new species is described, *Callidium angustum* (p. 8).

#### Kuhn, Kaspar [Caspar] (Rohrbach, Bavaria, Germany: 8 November 1819 – 10 February 1906: Ottobeuren, Bavaria, Germany). German theologian; joined the Benedictine Monastery St. Stephan in Augsburg in 1848 and after ordination in 1853 taught at a Latin school; moved to Ottobeuren in 1873 where he founded a theater club and in 1880 a museum of antiquities and natural history where he remained curator until his death. Reference. Moeller and Jahn (2005: 814).

**1858**. *Die Käfer des südbayerischen Flachlandes, analytisch beschrieben. Mit einer lithographirten Tafel.* K. Kollmann, Augsburg. viii + 400 pp. + 1 pl. (16mo) [DP: 1858 (title page; *Vorwort* dated 3 March 1858), 10 June 1858 (*Allg Bibl Deutsch*), 22 June 1858 (*Bayer Landbote*), July 1858 (*Allg Bibl*), 23 October 1858 (*Lit Centr Deutsch*), July–December 1858 (*Bibliot Hist-Nat*)] CNC

This work treats the Coleoptera of south Bavarian lowlands.

#### Künckel d’Herculais, Jules Philippe Alexandre (Paris, France: 10 February 1843 – 19 December 1918: Conflans-sur-l’Oise, Yvelines, France). French entomologist; assistant in entomology at the Muséum National d’Histoire Naturelle in Paris for nearly half a century; professor at the *Institut national d’Agronomie* in Paris; sent to Algeria and Argentina to study grasshopper invasions. References. Birabén (1900); Jaussaud and Brygoo (2004: 312–313); Cambefort (2006: 205).

**1887–[1891**]. *Histoire physique, naturelle et politique de Madagascar publiée par Alfred Grandidier. Volume XXII. Histoire naturelle des coléoptères. Tome II. – Atlas.* Imprimerie Nationale, Paris. 54 pls. (4to) CMLE, BHL

The first volume was authored by Charles Alluaud, 1902 [*q.v.*]. The two volumes form volumes 21 and 22 of the “*Histoire physique, naturelle et politique de Madagascar*.” Overall, the altas consists of 54 plates, issued in two parts: **1**: (pls 1–25) 1887 (title page), June 1888 (*Nat Nov*), 23 July 1888 (*Zool Anz*), 25 July 1888 (*Soc Ent Fr*); **2**: (pls 26–54) 1890 (Derksen and Scheiding-Göllner 1965: 191), September 1891 (*Nat Nov*), 14 October 1891 (*Soc Ent Fr*).

Scientific names are provided on the plates for all beetles illustrated and some species were first made available from the plates. A list of the species illustrated is provided in Appendix 10.

#### Küster, Heinrich Carl (Erlangen, Bavaria, Germany: 14 February 1807 – 17 April 1876: Bamberg, Bavaria, Germany). German entomologist and malacologist; professor of natural history and technology at the gymnasium in Erlangen; later, administrator of the telegraph station in Bamberg; his collections, which were in poor condition, were scattered. References. Meyer (1876); Bolling (1968).

**1840**. *Systematisches Verzeichniss der in der Umgegend Erlangens beobachteten Thiere. Erstes Heft. Wirbelthiere, Mollusken und Käfer enthaltend.* Erlangen. iv + 46 pp. (8vo) [DP: 1840 (title page), 28 October 1840 (*Lit Ztg*), 30 October 1840 (*Allg Bibl Deutsch*), 5 November 1840 (*Bibl Blätt*)] CNC (mf)

This is a catalogue of the vertebrates, molluscs and beetles observed in the neighborhood of Erlangen in Bavaria.

**1844–1854**. *Die Käfer Europa’s. Nach der Natur beschrieben. Mit Beiträgen mehrerer Entomologen.* Bauer & Raspe, Nürnberg. (16mo) MCZ, CNC

*Erstes Heft. Mit 2 Tafeln Abbildungen.* [208] pp. + 2 pls. [DP: 1844 (title page; *Vorwort* dated November 1844), 15 February 1845 (*Med Corr-Blatt*), 6 March 1845 (*Allg Bibl Deutsch*), 12 March 1845 (*Lit Ztg*), 13 May 1845 (*Ent Ver Stettin*), 16 May 1845 (*Leip Reper*), January–June 1845 (*Verz Bücher Landkarten*)].

*Zweites Heft. Mit 2 Tafeln Abbildungen.* [204] pp. + 2 pls. [DP: 1845 (title page; p. [4] dated June 1845), 28 August 1845 (*Allg Bibl Deutsch*), 10 September 1845 (*Lit Ztg*), 19 September 1845 (*Leip Reper*), 11 December 1845 (*Ent Ver Stettin*), July–December 1845 (*Verz Bücher Landkarten*)].

*Drittes Heft. Mit 2 Tafeln Abbildungen.* [202] pp. + 2 pls. [DP: 1845 (title page; p. [2] dated September 1845), 11 December 1845 (*Ent Ver Stettin*), 26 February 1846 (*Allg Bibl Deutsch*), April 1846 (*Allg Forst Jagd Ztg*), 8 May 1846 (*Leip Reper*)].

*Viertes Heft. Mit 3 Tafeln Abbildungen.* [204] pp. + 3 pls. [DP: 1846 (title page; *Vorwort* dated February 1846), 9 October 1846 (*Ent Ver Stettin*), 5 November 1846 (*Allg Bibl Deutsch*), 30 November 1846 (*Intell Serapeum*), 18 December 1846 (*Leip Reper*), July–December 1846 (*Verz Bücher Landkarten*)].

*Fünftes Heft. Mit 3 Tafeln Abbildungen.* [204] pp. + 3 pls. [DP: 1846 (title page; *Anzeige* dated 30 June 1846), 9 October 1846 (*Ent Ver Stettin*), 5 November 1846 (*Allg Bibl Deutsch*), 30 November 1846 (*Intell Serapeum*), 18 December 1846 (*Leip Reper*), July–December 1846 (*Verz Bücher Landkarten*)].

*Sechstes Heft. Mit 2 Tafeln Abbildungen.* [202] pp. + 2 pls. [DP: 1846 (title page; *Inhalt* dated July 1846), 14 January 1847 (*Ent Ver Stettin*), 11 March 1847 (*Allg Bibl Deutsch*), 16 April 1847 (*Leip Reper*), January–June 1847 (*Verz Bücher Landkarten*)].

*Siebentes Heft. Mit 2 Tafeln Abbildungen.* [202] pp. + 2 pls. [DP: 1846 (title page; *Inhalt* dated September 1846), 4 March 1847 (*Ent Ver Stettin*), 11 March 1847 (*Allg Bibl Deutsch*), 16 April 1847 (*Leip Reper*), January–June 1847 (*Verz Bücher Landkarten*)].

*Achtes Heft. Mit 2 Tafeln Abbildungen.* [202] pp. + 2 pls. [DP: 1847 (title page; *Inhalt* dated March 1847), 1 July 1847 (*Allg Bibl Deutsch*), 10 July 1847 (*Ent Ver Stettin*), 6 August 1847 (*Leip Reper*)].

*Neuntes Heft. Mit 3 Tafeln Abbildungen.* [202] pp. + 3 pls. [DP: 1847 (title page; *Inhalt* dated April 1847), 23 September 1847 (*Allg Bibl Deutsch*), 12 November 1847 (*Leip Reper*)].

*Zehntes Heft. Mit 3 Tafeln Abbildungen.* [202] pp. + 3 pls. [DP: 1847 (title page; *Inhalt* dated May 1847), 7 November 1847 (*Ent Ver Stettin*), 27 April 1848 (*Allg Bibl Deutsch*)]. Includes *Systematisches Verzeichniss der in der ersten Serie (Heft I–X) der Käfer Europa’s von Prof. Apetz, v. Kiesenwetter und Dr. H.C. Küster beschriebenen Arten* (28 pp. numbered, without publication date).

*Elftes Heft. Mit 2 Tafeln Abbildungen.* [202] pp. + 2 pls. [DP: 1847 (title page; *Inhalt* dated July 1847), 27 April 1848 (*Allg Bibl Deutsch*), 23 June 1848 (*Leip Reper*)].

*Zwölftes Heft. Mit 2 Tafeln Abbildungen.* [202] pp. + 2 pls. [DP: 1847 (title page; *Inhalt* dated November 1847), 27 April 1848 (*Allg Bibl Deutsch*), 22 June 1848 (*Ent Ver Stettin*), 23 June 1848 (*Leip Reper*)]. Hagen (1862: 439) listed this *Heft* as published in 1848.

*Dreizehntes Heft. Mit 2 Tafeln Abbildungen.* [204] pp. + 3 pls. [DP: 1848 (title page; *Inhalt* dated February 1848; *Einladung* dated 1 September 1848), 30 November 1848 (*Allg Bibl Deutsch*), December 1848 (*Ent Ver Stettin*)].

*Vierzehntes Heft. Mit 2 Tafeln Abbildungen.* [202] pp. + 2 pls. [DP: 1848 (title page; *Inhalt* dated May 1848), 30 November 1848 (*Allg Bibl Deutsch*), December 1848 (*Ent Ver Stettin*)].

*Fünfzehntes Heft. Mit 3 Tafeln Abbildungen.* [202] pp. + 3 pls. [DP: 1848 (title page; *Inhalt* dated September 1848), 26 February 1849 (*Ent Ver Stettin*), 15 March 1849 (*Intell Serapeum*)].

*Sechzehntes Heft. Mit 3 Tafeln Abbildungen.* [202] pp. + 3 pls. [DP: 1849 (title page; *Inhalt* dated January 1849), 15 July 1849 (*Intell Serapeum*), July–December 1849 (*Verz Bücher Landkarten*), 13 August 1850 (*Ent Ver Stettin*)].

*Siebzehntes Heft. Mit 2 Tafeln Abbildungen.* [202] pp. + 2 pls. [DP: 1849 (title page; *Inhalt* dated March 1849), 15 October 1849 (*Intell Serapeum*), July–December 1849 (*Verz Bücher Landkarten*)].

*Achtzehntes Heft. Mit 2 Tafeln Abbildungen.* [204] pp. + 2 pls. [DP: 1849 (title page; *Inhalt* dated August 1849)].

*Neunzehntes Heft. Mit 2 Tafeln Abbildungen.* [214] pp. + 2 pls. [DP: 1849 (title page; *Inhalt* dated October 1849), 24 January 1850 (*Allg Bibl Deutsch*), 13 February 1850 (*Bibl Lit Anz*), 6 June 1850 (*Ent Ver Stettin*)].

*Zwanzigstes Heft. Mit 2 Tafeln Abbildungen.* 32 + [202] + 2 pls. [DP: 1850 (title page; *Inhalt* dated February 1850), 13 August 1850 (*Ent Ver Stettin*), 22 August 1850 (*Allg Bibl Deutsch*), 15 September 1850 (*Intell Serapeum*)]. This volume has two other title pages. One reads “*Die Käfer Europa’s. Nach der Natur beschrieben. Zweite Serie. Heft XI–XX. Mit Beiträgen von Prof. Apetz, Professor Erichson und H. v. Kiesenwetter*” and is dated “1847–50.” The second one reads “*Systematisches Verzeichniss der in zweiten Serie (Heft XI–XX) der Käfer Europa’s von Prof. Apetz, Prof. Erichson, von Kiesenwetter und Dr. H.C. Küster beschriebenen Arten.*”

*Einundzwanzigstes Heft. Mit 2 Tafeln Abbildungen.* [214] pp. + 2 pls. [DP: 1850 (title page; *Vorwort* dated September 1850), 21 November 1850 (*Allg Bibl Deutsch*), January 1851 (*Ent Ver Stettin*)].

*Zweiundzwanzigstes Heft. Mit 3 Tafeln Abbildungen.* [204] pp. + 3 pls. [DP: 1851 (title page; *Inhalt* dated November 1851), 11 December 1851 (*Allg Bibl Deutsch*)].

*Dreiundzwanzigstes Heft. Mit 3 Tafeln Abbildungen.* [2] + 4 + [200] pp. + 3 pls. [DP: 1851 (title page; *Inhalt* dated June 1851), 22 April 1852 (*Allg Bibl Deutsch*), April–June 1852 (*Cat Muquardt*), January–June 1852 (*Bibliot Hist-Nat*)].

*Vierundzwanzigstes Heft. Mit 2 Tafeln Abbildungen.* [78] + 4 + [132] pp. + 2 pls. [DP: 1852 (title page; *Vorwort* dated May 1852), 28 October 1852 (*Allg Bibl Deutsch*), October–December 1852 (*Cat Muquardt*), July–December 1852 (*Bibliot Hist-Nat*)].

*Fünfundzwanzigstes Heft. Mit 2 Tafeln Abbildungen.* [202] pp. + 2 pls. [DP: 1852 (title page; *Inhalt* dated October 1852), 13 January 1853 (*Allg Bibl Deutsch*), January–March 1853(*Cat Muquardt*), 23 June 1853 (*Ent Ver Stettin*), January–June 1853 (*Bibliot Hist-Nat*)].

*Sechsundzwanzigstes Heft. Mit 2 Tafeln Abbildungen.* [202] pp. + 2 pls. [DP: 1853 (title page; *Inhalt* dated August 1853), 27 October 1853 (*Allg Bibl Deutsch*), 30 November 1853 (*Intell Serapeum*), October–December 1853 (*Cat Muquardt*), July–December 1853 (*Bibliot Hist-Nat*)].

*Siebeundzwanzigstes Heft. Mit 2 Tafeln Abbildungen.* [206] pp. + 2 pls. [DP: 1853 (title page; *Inhalt* dated September 1853), 5 January 1854 (*Allg Bibl Deutsch*), 15 February 1854 (*Intell Serapeum*), January–March 1854 (*Cat Muquardt*), January–June 1854 (*Bibliot Hist-Nat*)]. Hagen (1862: 439) listed this *Heft* as published in 1854.

*Achtundzwanzigstes Heft. Mit 2 Tafeln Abbildungen.* [202] pp. + 2 pls. [DP: 1854 (title page; *Inhalt* dated February 1854), 4 January 1855 (*Allg Bibl Deutsch*), January–March 1855 (*Cat Muquardt*), January–June 1855 (*Bibliot Hist-Nat*; *Verz Bücher Landkarten*)]. Hagen (1862: 439) listed this *Heft* as published in 1855.

The pages are not numbered. The entire series consists of 48 *Hefte*, 1844–1912. The first 28 were authored by Küster, the 29th by G. Kraatz, 1873 [*q.v.*], and the remaining ones by J. Schilsky, 1894–1912 [*q.v.*].

#### Kuthy, Dezsö [Desiderius] (Szarvas, Békés, Hungary: 7 June 1844 – 10 September 1917: Budapest, Hungary). Hungarian entomologist; educated as a lawyer but because of serious lung problem turn to natural science; curator of the Coleoptera and later Orthoptera collections at the Hungarian Natural History Museum (1895–1914). Reference. Csiki (1918, P).

[**1898**]. *Coleoptera. A Magyar birodalom állatvilága. A Magyar birodalomból eddig ismert állatok rendszeres lajstroma. Magyarorszáh ezeréves fennállásának emlékére kiadta a K.M. Természettudományi Társulat. / Fauna regni Hungariae. Animalium Hungariae hucusque cognitorum enumeratio systematica. In memoriam regni Hungariae mille abhinc annis constituti edidit Regia Societas Scientiarum Naturalium Hungarica.* Budapest. 213 pp. (4to) [DP: 1896 (title page; preliminaries dated 1 March 1897), January 1899 (*Nat Nov*)] (*n.v.* except title page)

This work is usually cited as published in 1896 or 1897; the title page is dated 1896 but the preliminaries 1 March 1897. However, some clues suggest that the book was issued only in 1898. The *Naturae Novitates* recorded the work in January 1899 listing it as “Budapest 1896 (1898)” which seems to indicate that the title page is dated 1896 but that the work was published in 1898. Aigner-Abafi (1897), in the October 1897 issue of *Rovartani Lapok*, discussed the parts of the series *Fauna regni Hungariae* that were published and did not mentioned Kuthy’s contribution. Kuthy’s part was mentioned by the same author only the following year (Aigner-Abafi 1898: 252). Finally Kuthy’s section was recorded by Seidlitz (1900: 126) in his review of the entomological literature for the year 1898.

This work was reissued in 1900 as part of *Arthropoda* volume of the *Fauna Regni Hungariae* [GB].

#### Kuwert, August Ferdinand (Nida, Lithuania: 15 October 1828 – 14 August 1894: Wernsdorf near Königsberg [currently Kaliningrad], Russia). German entomologist of independent means; his main collection of Coleoptera was acquired by René Oberthür [*q.v.*] and is now at the Muséum National d’Histoire Naturelle in Paris. References. Anonymous (1894b, P); Barthe (1895: 108–109).

**1890**. *Bestimmungs-Tabellen der europäischen Coleopteren. XIX. Heft. Enthaltend die Familie der Hydrophilidae. I. Abtheilung: Hydrophilini.* Ed. Reitter, Brünn. 121 pp. (8vo) [DP: 1890 (title page), June 1890 (*Nat Nov*)] SME

This work was also issued in *Verhandlungen des Naturforschenden Vereins in Brünn* 28 [1889]: 3–121 [DP: 1890 (title page), January 1891 (*Nat Nov*)].

**1890**. *Bestimmungs-Tabellen der europäischen Coleopteren. XX. Heft. Enthaltend die Familie der Hydrophilidae. II. Abtheilung: Sphaeridiini und Helophorini.* Ed. Reitter, Brünn. 172 pp. + 4 pls. (8vo) [DP: 1890 (title page), 31 December 1890 (*Wien Ent Ztg* 9: 319), December 1890 (*Nat Nov*)] SME

This work was also issued in *Verhandlungen des Naturforschenden Vereins in Brünn* 28 [1889]: 159–328 [DP: 1890 (title page), January 1891 (*Nat Nov*)].

#### Labram, Jonas David (Basel, Switzerland: 3 February 1785 – 3 April 1852: Basel, Switzerland). Swiss naturalist illustrator in Basel. Reference. Burckhardt (1907).

**1835–[1849**]. [Labram, D. and Imhoff, L.] *Insekten der Schweiz, die vorzüglichsten Gattungen je durch eine Art bildlich dargestellt von J.D. Labram. Nach Anleitung und mit Text von Dr. Ludwig Imhoff.* C.F. Spittler [later, J.G. Bahnmaier], Basel. 457 pls + [463] pp. (8vo) MCZ

*Erstes Bändchen. 1tes bis 20tes Heft.* 82 pp. + 80 pls. [DP: 1836 (title page; first page dated June 1836)]. *Heft* 1 was recorded on 4 April 1835 (*Bibl Deutsch*), *Hefte* 7–8 on 6 June 1835 (*Bibl Deutsch*) and *Heft* 20 on 2 September 1836 (*Allg Bibl Deutsch*), 7 September 1836 (*Lit Ztg*), 26 October 1836 (*Amts Reg Frankfurt*) and 19 November 1836 (*Schweizer-Bote*).

*Zweites Bändchen. 21tes bis 40tes Heft.* 81 pp. + 80 pls. [DP: 1838 (title page)]. *Hefte* 21–22 were noticed on 3 March 1837 (*Allg Bibl Deutsch*).

*Drittes Bändchen. 41ftes bis 60ftes Heft.* J.G. Bahnmaier. 81 pp. + 80 pls. [DP: 1842 (title page)]. *Hefte* 41–44 were noticed on 13 December 1839 (*Amts Reg Postdam*), 29 December 1839 (*Amts Reg Koblenz*), 10 January 1840 (*Allg Bibl Deutsch*) and *Hefte* 45–52 on 28 May 1841 (*Amts Reg Bromberg*), 5 June 1841 (*Amts Reg Liegnitz*) and 18 June 1841 (*Amts Reg Minden*).

*Viertes Bändchen. 61ftes bis 80ftes Heft.* 81 pp. + 80 pls. [DP^59^: n.d. (title page), 1845 (Hagen 1862: 443; Horn and Schenkling 1928b: 677)].

*Fünftes Bändchen. 81ftes bis 100ftes Heft.* 81 pp. + 80 pls. [DP: n.d. (title page)]. *Hefte* 81–90 were noticed on 15 June 1848 (*Publ Circ*) and 23 June 1848 (*Leip Reper*) and *Hefte* 91–100 on 15 February 1849 (*Intell Serapeum*)]. Horn and Schenkling (1928b: 677) mentioned that this part was published in 1845 but it seems to be a pure guess.

[*Sechstes Bändchen*]. 57 pp. + 56 pls. [DP: unknown]. This section was unfinished and issued without title page.

This work was issued in 114 *Hefte*, each consisting of a four unnumbered, uncolored or colored plates and four pages of text. At least the first 44 *Hefte* were issued under the title “*Sammlung von Abbildungen schweizerischer Insecten*.” Imhoff wrote the text and Labram provided the illustrations.

Based on information gathered at this time, the following dates should be retained for the different *Hefte*: **1–8**: 1835; **9–20**: 1836; **21–22**: 1837; **23–40**: 1838; **41–44**: 1839; **45–52**: 1841; **53–60**: 1842; **61–80**: 1845; **81–90**: 1848; **91–100**: 1849; **101–114**: 1852.

**1838–1851**. [Labram, D. and Imhoff, L.] *Singulorum generum curculionidum unam alteramve speciem additis iconibus. Die Gattungen der Rüsselkäfer erläutert durch bildliche Darstellung einzelner Arten.* Schweighauser, Basel. 152 pls + text. (12mo) CMLE, AMNH, MCZ

This work was issued in 19 *Hefte*, each containing eight colored plates and eight leafs of descriptions in Latin and German. The pages and plates are not numbered. The date of publication of the *Hefte* are: **1**: 1838 (wrapper), 9 July 1838 (*Allg Ztg*), 14 July 1838 (*Feuil J Lib*), 20 July 1838 (*Allg Bibl Deutsch*), 1 August 1838 (*Lit Ztg*), July–December 1838 (*Verz Bücher Landkarten*); **2**: 1838 (Sherborn 1922a: lxxvi), 18 January 1839 (*Allg Bibl Deutsch*), 30 January 1839 (*Lit Ztg*), 8 March 1839 (*Amts Reg Postdam*), 22 March 1839 (*Amts Reg Minden*), January–March 1839 (*Foreign Quart Rev*), January–June 1839 (*Verz Bücher Landkarten*); **3**: 1839 (Sherborn 1922a: lxxvi), 3 April 1839 (*Lit Ztg*), 24 May 1839 (*Amts Reg Postdam*), January–June 1839 (*Verz Bücher Landkarten*); **4**: 1839 (Sherborn 1922a: lxxvi), 30 August 1839 (*Amts Reg Postdam*), July–December 1839 (*Verz Bücher Landkarten*); **5**: 1839 (Sherborn 1922a: lxxvi), 10 August 1840 (*Ent Ver Stettin*), 18 September 1840 (*Amts Reg Postdam*), 25 September 1840 (*Amts Reg Minden*), 30 October 1840 (*Allg Bibl Deutsch*); **6**: 1840 (Sherborn 1922a: lxxvi), 30 October 1840 (*Allg Bibl Deutsch*), 5 November 1840 (*Bibl Blätt*); **7**: 1840 (Sherborn 1922a: lxxvi), January–June 1841 (*Verz Bücher Landkarten*); **8**: 1841 (Sherborn 1922a: lxxvi), 18 May 1841 (*Börsen Deutsch Buch*), January–June 1841 (*Verz Bücher Landkarten*), 10 September 1841 (*Allg Bibl Deutsch*); **9**: 1841 (Sherborn 1922a: lxxvi), 13 December 1841 (*Ent Ver Stettin*), January–June 1842 (*Verz Bücher Landkarten*); **10**: 1842 (Sherborn 1922a: lxxvi), July–December 1842 (*Verz Bücher Landkarten*); **11**: 1843 (Sherborn 1922a: lxxvi), 28 November 1844 (*Allg Bibl Deutsch*), July–December 1844 (*Verz Bücher Landkarten*), 10 January 1845 (*Leip Reper*); **12**: 1845 (wrapper), 15 May 1845 (*Allg Bibl Deutsch*), 4 June 1845 (*Lit Ztg*), January–June 1845 (*Verz Bücher Landkarten*); **13**: 1845 (wrapper), January–June 1846 (*Verz Bücher Landkarten*); **14**: 4 February 1847 (*Ent Ver Stettin*), 10 February 1847 (*Lit Ztg*), 18 February 1847 (*Allg Bibl Deutsch*), 19 March 1847 (*Leip Reper*), January–June 1847 (*Verz Bücher Landkarten*), 1848 (Sherborn 1922a: lxxvi); **15**: 1848 (wrapper), 30 March 1848 (*Allg Bibl Deutsch*), 22 June 1848 (*Ent Ver Stettin*), 23 June 1848 (*Leip Reper*); **16**: 1849 (Sherborn 1922a: lxxvi), 7 June 1849 (*Ent Ver Stettin*); **17**: 1849 (Sherborn 1922a: lxxvi), 21 February 1850 (*Allg Bibl Deutsch*), 28 February 1850 (*Intell Serapeum*), 6 June 1850 (*Ent Ver Stettin*); **18**: 1849 (Sherborn 1922a: lxxvi), 19 December 1850 (*Allg Bibl Deutsch*), January–June 1851 (*Bibliot Hist-Nat*; *Verz Bücher Landkarten*); **19**: 1851 (wrapper), 1852 (Lacordaire 1863: 17; Sherborn 1922a: lxxvi). A title page was issued in 1842 which reads beside the title “*Pars I. Fascic. 1–10* / *Erster Theil. Heft 1–10*” [CNC].

Based on evidence gathered during this study, the following years should be retained: Hefte 1–2 [1838], 3–4 [1839], 5–7 [1840], 8–9 [1841], 10 [1842], 11 [1843], 12–13 [1845], 14 [1847], 15 [1848], 16–17 [1849], 18 [1850], 19 [1851].

The content of each *Heft* is as follows: **Heft 1**: [ii] + *Belopherus militaris*; Arrhenodes (Nemorhinus) duplicatus; *Arrhenodes dispar*; Arrhenodes (Hormocerus) coronatus; *Platymerus germari*; *Antliarhinus zamiae*; *Eutrachelus temmincki*. **Heft 2**: Arrhenodes (Trachelizus) pygmaeus; *Ceocephalus reticulatus*; *Ischnomerus erythroderes*; Brentus (Nemocephalus) laevis; *Brentus vulneratus*; *Brenthus anchorago*; *Brentus lineicollis*; Brenthus (Ischyromerus) madagascariensis. **Heft 3**: *Claeoderes radulirostris*; *Ulocerus tetraurus*; *Episus aculeatus*; *Cylas* Latr., *Oxyrhynchus* Schonh., *Bruchus* Schönherr, *Spermophagus* Steven, *Urodon* Schönh. **Heft 4**: *Stenocerus* Schönh.; *Nemotrichus* Dejean; *Meconemus*; *Acorynus* Schh.; *Mecocerus* Schönh.; *Xenocerus* Germar; *Ptychoderes* Schönh.; *Phloeotragus* Schönh. **Heft 5**: *Gymnognathus* Schönh.; *Araeocerus* Schönh.; *Phaenithon* Schönh.; *Eugonus* Schh.; *Brachytarsus* Schh.; *Tophoderus* Dejean; *Phaenithon* Schönh.; *Xylinades* Latreille. **Heft 6**: *Deuterocrates*; *Tropideres* Schönh.; *Cratoparis* Dejean, Schönh.; *Lagopezus* Dejean, Schönh.; *Systaltocerus*; *Eucorynus* Schönh. **Heft 7**: *Anthribus* Schh.; *Polycorynus*; *Chirotenon*; *Decataphanes*; *Anacerastes*. **Heft 8**: *Uterosomus* Chevrolat; *Platyrrhinus* Clairville; *Trachylizus adustus* Schh.; *Ceocephalus riisii*; *Taphroderes striolatus*; *Arrhenodes corniger* Varietas; *Analotes* Schh.; *Discotenes*. **Heft 9**: *Camarotus* Germ. Schh.; *Pterocolus* Schh.; *Cybebus* Schh.; *Attelabus* L. Schh.; *Apoderus* Ol. Schh. **Heft 10**: *Camarotus* Germ. Schh.; *Pterocolus* Schh.; *Cybebus* Schh.; *Attelabus* L. Schh.; *Apoderus* Ol. Schh.; *Rhynchites* Herbst. Schh.; *Cephalobarus macrocephalus* Schönh.; *Blaberus* Schh.; *Mecocerus* Schönh.; *Platymerus germari*; Fam. Anthribides; Index partis primae (Fascic. 1-10.). **Heft 11**: *Tanaos* Schonh.; *Eugnamptus* Schonh.; *Rhinomacer* F. Schh.; *Deodycorhynchus*; *Belus* Schh.; *Homalocerus* Schh.; *Ithycerus* Dalm. Schh.; *Eurhynchus* Schh. **Heft 12**: *Brachycerus* F. **Heft 13**: *Microcerus* Gyll.; *Rhigus* Dalm.; *Cydianerus* Schh.; *Polyteles* Schh.; *Entimus* Grmr.; *Phaedropus* Schh.; *Mesoptilius*. **Heft 14**: *Somatodes* Schh.; *Cherrus* Dalm.; *Catasarcus* Schh.; *Ophryastes* Germ.; *Pachyrhynchus* Germ.; *Apocyrtus* Erichs.; *Deracanthus* Schh. **Heft 15**: *Hipporhinus* Billb.; *Polyphrades* Schh.; *Prosayleus* Schh.; *Dermatodes* Schh.; *Platycopes* Dalm.; *Blosyrus* Schh. **Heft 16**: *Stigmatrachelus* Schh.; *Hypomeces* Schh.; *Anaemerus* Schh.; *Polyclaeis* Schh.; *Tanymecus* Grmr.; *Protenomus* Schh.; *Hadromerus* Schh.; *Eudiagogus* Schh. **Heft 17**: *Platyomus* Schh.; *Platyaspistes* Schh.; *Eustales* Schh.; *Cratopus* Dalm.; *Macropterus* Boheman. **Heft 18**: *Holonychus* Schh.; *Proscephaladeres* Schh.; *Rhyssocarpus* Schh.; *Atmetonychus* Schh.; *Megalostylus* Schh.; *Cyphus* Schh. **Heft 19**: *Sitones* Schh.; *Exophthalmus* Schh.; *Diaprepes* Schh.; *Tetrabothynus* Schh.; *Prepodes* Schh. (pro parte.); *Catamonus* Schh.; *Lachnopus* Schh. (pro parte.); *Chlorophanus* Dalm.

**1848–1852**. [Labram, D. and Imhoff, L.] *Die schweizerischen Käfergattungen in Abbildungen nach der Natur von J.D. Labram. Nach Anleitung und mit Text von Dr. Ludwig Imhoff.* Bahnmaier, Basel. 136 pls + text. (8vo) CMLE (mf), CNC (mf), SDL

This work was issued in 34 *Hefte*, each consisting of four color plates and four leafs of text. The plates and the text are unnumbered. A title page was issued for the combined first 20 *Hefte* which reads, in addition to the title, “*Erstes Bändchen. 1stes bis 20stes Heft.*” I have seen wrappers for three *Hefte* and no dates of publication are listed. The entire work was issued 1848–1852 (Carus and Engelmann 1861: 705). I have seen notices for the following *Hefte*^60^: **1–2**: 30 November 1848 (*Lit Ztg*); **15–18**: July–December 1849 (*Verz Bücher Landkarten*); **19–20**: 7 February 1850 (*Allg Bibl Deutsch*), 30 April 1850 (*Bibl Lit Anz*); **21–22**: 23 May 1850 (*Allg Bibl Deutsch*); **23–24**: 26 September 1850 (*Allg Bibl Deutsch*); **25–26**: 19 December 1850 (*Allg Bibl Deutsch*); **27–28**: 27 February 1851 (*Allg Bibl Deutsch*); **29–30**: 3 June 1851 (*Börsen Deutsch Buch*), 5 June 1851 (*Allg Bibl Deutsch*), January–June 1851 (*Bibliot Hist-Nat*); **31–32**: 27 November 1851 (*Allg Bibl Deutsch*), July–December 1851 (*Bibliot Hist-Nat*); **33–34**: 25 March 1852 (*Allg Bibl Deutsch*), January–March 1852 (*Cat Muquardt*), January–June 1852 (*Bibliot Hist-Nat*).

The content of this work is identical to the fifth and sixth *Bande* of Labram and Imhoff’s *Insekten der Schweiz* [*q.v.*] (Geiger 1946: 33).

#### Lacordaire, Jean Théodore (Recey-sur-Ource, Côte-d’Or, France: 1 February 1801 – 18 July 1870: Liège, Belgium). Belgian (French-born) entomologist; made four collecting expeditions to South America (1824–1832); professor of zoology and comparative anatomy at the University of Liège (1835–1870); his collection was sold successively by families as soon as they were treated in his *Genera des Coléoptères*. References. Candèze (1872, P); Morren (1873, P); Zimmerman (1993: 615–617, P); Smetana and Herman (2001: 93, P); Cambefort (2006: 210–211, P).

**1842**. *Révision de la famille des cicindélides de l’ordre des coléoptères.* Félix Oudart, Liége. 40 pp. (8vo) [DP: July 1842 (title page), August 1842 (*Rev Zool*), 5 October 1842 (*Soc Ent Fr*), 5 November 1842 (*Acad R Sci Belg*), 10 January 1843 (*Ent Ver Stettin*)] MCZ, GB

The first page is entitled “*Révision de la famille des cicindélides (Cicindelidae) de l’ordre des coléoptères, accompagnée de la création de quelques genres nouveaux*.” The work was also issued, under the title of the first page, in *Mémoires de la Société Royale des Sciences de Liège* 1(1): 85–120 [DP: January 1843 (title page)].

**1842**. *Monographie des erotyliens, famille de l’ordre des coléoptères.* Roret, Paris. xiv + 543 pp. (8vo) [DP: September 1842 (title page), 3 October 1842 (*Acad Sci Fr*), 5 October 1842 (*Soc Ent Fr*), 22 October 1842 (*Bibl Fr*), 2 November 1842 (*Lit Ztg*), 5 November 1842 (*Acad R Sci Belg*), 12 November 1842 (*Feuil J Lib*), 15 November 1842 (*Monit Lib*), 11 December 1842 (*J Débats*), 15 December 1842 (*Presse*)] CNC, GB, BHL

This work has been recorded by Hagen (1862: 444) under the title “*Iconographie de la famille des érotyliens*.” However, this title is incorrect as noted by Schmidt-Göbel (1876: 152).

**1845–1848**. *Monographie des coléoptères subpentamères de la famille des phytophages.* C. Muquardt, Bruxelles et Leipzig. (8vo) MCZ, GB

*Tome premier.* liv + 740 pp. [DP: 1845 (title page)]. This volume was issued in two parts: **1**: (pp. i–liv + 1–320) 1 May 1845 (recto of title page), 3 June 1845 (*Ent Ver Stettin*), 25 June 1845 (*Soc Ent Fr*), 17 October 1845 (*Leip Reper*); **2**: (pp. 321–740) 15 November 1845 (recto of title page), 24 December 1845 (*Soc Ent Fr*).

*Tome second.* vi + 890 pp. [DP: May 1848 (title page), 14 June 1848 (*Soc Ent Fr*), 7 September 1848 (*Lit Ztg*)].

These volumes were also issued as volumes 3 [DP: May 1845 (title page)] and 5 [DP: 1848 (title page), 14 October 1848 [JD] (*Soc Imp Nat Moscou*)] of the *Mémoires de la Société Royale des Sciences de Liège*.

**1854–1872**. *Histoire naturelle des insectes. Genera des Coléoptères ou exposé méthodique et critique de tous les genres proposés jusqu’ici dans cet ordre d’insectes.* Roret, Paris. (8vo) CNC, BHL

*Tome premier contenant les familles des cicindélètes, carabiques, dytiscides, gyrinides et palpicornes.* xx + 486 pp. [DP: 1854 (title page), 8 February 1854 (*Soc Ent Fr*), 13 February 1854 (*Acad Sci Fr*), 18 March 1854 (*Feuil J Lib*), 8 April 1854 (*Bibl Fr*), 23 May 1854 (*Acad Nat Sci Phila*), January–June 1854 (*Bibliot Hist-Nat*)].

*Tome deuxième contenant les familles des paussides, staphyliniens, psélaphiens, scydménides, silphales, sphériens, trichoptérygiens, scaphidiles, histériens, phalacrides, nitidulaires, trogositaires, colydiens, rhysodides, cucujipes, cryptophagides, lathridiens, mycétaphagides, thorictides, dermestins, byrrhiens, géoryssins, parnides, hétérocérides.* 548 pp. [DP: 1854 (title page), 2 October 1854 (*Acad Sci Fr*), 4 November 1854 (*Acad R Sci Belg*), 8 February 1855 (*Ent Ver Stettin*), 5 March 1855 (*Ent Soc Lond*)].

*Tome troisième contenant les familles des pectinicornes et lamellicornes.* 594 pp. [DP: 1856 (title page), 29 October 1855 (*Acad Sci Fr*), 1 December 1855 (*Acad R Sci Belg*), 12 January 1856 (*Courrier Lib*), 29 March 1856 (*Bibl Fr*), March 1856 (*Allg Bibl*)].

*Tome quatrième contenant les familles des buprestides, throscides, eucnémides, élatérides, cébrionides, cérophytides, rhipicérides, dascyllides, malacodermes, clérides, lyméxylones, cupésides, ptiniores, bostrichides et cissides.* 579 pp. [DP: 1857 (title page), 25 May 1857 (*Acad Sci Fr*), 23 June 1857 (*Acad Nat Sci Phila*), 27 June 1857 (*Bibl Fr*), 4 July 1857 (*Acad R Sci Belg*), September 1857 (*Allg Bibl*), July–December 1857 (*Bibliot Hist-Nat*)].

*Tome cinquième première partie. Contenant les familles des Ténébrionides, Cistélides, Nilionides, Pythides, Mélandryides, Lagriides, Pédilides, Anthicides, Pyrochroïdes, Mordellides, Rhipiphorides, Stylopides, Meloïdes et Oedémérides.* 400 pp. [DP: 1859 (title page), 27 June 1859 (*Acad Sci Fr*), 2 July 1859 (*Acad R Sci Belg*), 16 July 1859 (*Bibl Fr*), September 1859 (*Allg Bibl*)].

*Tome cinquième seconde partie. Contenant les familles des Ténébrionides, Cistélides, Nilionides, Pythides, Mélandryides, Lagriides, Pédilides, Anthicides, Pyrochroïdes, Mordellides, Rhipiphorides, Stylopides, Meloïdes et Oedémérides.* Pp. 401–750. [DP: 1859 (title page), 27 June 1859 (*Acad Sci Fr*), 2 July 1859 (*Acad R Sci Belg*), 16 July 1859 (*Bibl Fr*), September 1859 (*Allg Bibl*)].

*Tome sixième contenant la famille des curculionides.* 637 pp. [DP: 1863 (title page), 1 August 1863 (*Acad R Sci Belg*), 10 August 1863 (*Acad Sci Fr*), 22 August 1863 (*Bibl Fr*), 7 September 1863 (*Ent Soc Lond*), September–October 1863 (*Allg Bibl*), July–December 1863 (*Bibliot Hist-Nat*)].

*Tome septième contenant les familles des curculionides (suite), scolytides, brenthides, anthribides et bruchides.* 620 pp. [DP: 1866 (title page), 12 December 1865 (*Bookseller*), 5 January 1866 (*Acad R Sci Belg*), 20 January 1866 (*Bibl Fr*), January–June 1866 (*Bibliot Hist-Nat*), 5 November 1866 (*Ent Soc Lond*)].

*Tome huitième contenant les familles des tricténotomides et des longicornes.* 552 pp. [DP: 1869 (title page), 28 November 1868 (*Bibl Fr*), November 1868 (Dallas 1869: 194), 16 December 1868 (*Acad R Sci Belg*), July–December 1868 (*Bibliot Hist-Nat*), 1 February 1869 (*Ent Soc Lond*), 8 February 1869 (*Amer Ent Soc*)].

*Tome neuvième première partie. Famille des longicornes (suite).* 409 pp. [DP: 1869 (title page), 4 November 1869 (*Acad R Sci Belg*), 13 November 1869 (*Bibl Fr*), December 1869 (*Polybiblion*), July–December 1869 (*Bibliot Hist-Nat*)].

*Tome neuvième deuxième partie. Famille des longicornes (fin).* Pp. 411–930. [DP: 1872 (title page), 1 April 1872 (*Pet Nouv Ent*), 25 May 1872 (*Bibl Fr*), 5 June 1872 (*Asia Soc Beng*), 29 June 1872 (*Lit Centr Deutsch*), January–June 1872 (*Bibliot Hist-Nat*), August 1872 (*Polybiblion*)].

*Atlas.* 47 + 1 (Errata) pp. + 134 pls. [DP: n.d. (title page)]. The plates were issued by livraisons but no publication dates were found for them during this study. Scientific names are provided for the illustrations on each plate.

Volumes 10, 11, and 12 of the series were authored by Chapuis, 1874–1876 [*q.v.*]. The series is part of Roret’s “*Collections des suites à Buffon*.” Volumes 3, 7 and 8 were issued in the year preceding that indicated on the title page.

#### LaFerté-Sénectère, François [Faustin] Thibault de la Carte (Marquis de) (Paris, France: 18 June 1807 – 18 April 1886: Tours, Indre-et-Loire, France). French coleopterist; built one of the largest private beetle collection of his time which was put on sale in 1859. Reference. Cambefort (2006: 211–212).

**1849**. *Monographie des Anthicus et genres voisins, coléoptères hétéromères de la tribu des trachélides.* Sapia, Paris. xxii + 340 pp. + 16 pls. (8vo) [DP: 1848 (title page), 5 May 1849 (*Bibl Fr*), 17 May 1849 (*Lit Ztg*), 11 July 1849 (*Soc Ent Fr*)] CNC, GB, BHL

This publication is almost an integral reproduction of sections published by LaFerté-Sénectère in livraisons 4–9 of Guérin-Méneville’s *Species et iconographie générique des animaux articulés*, 1843–1849 [*q.v.*]. The “préliminaires” (pp. ix–xxii), most of the “supplément” (pp. 297–305), and the “tabula analytica” (pp. 307–321) are new. As noted by Schaum (1849: 164) and Chandler (2000: 434), this work although dated 1848 on the title page was issued in 1849.

Chandler (2000: 437) determined that Laferté-Sénectère’s treatment of the Anthicidae in livraisons 4–9 of the *Species et iconographie générique des animaux articulés* [*q.v.*] should take precedence for priority of names over this publication.

#### Laicharting [Laicharding], Johann Nepomuk von (Innsbruck, Tirol, Austria: 4 February 1754 – 7 May 1797: Innsbruck, Austria). Austrian botanist and entomologist; professor of natural history at the University of Innsbruck; his collection is at the Tiroler Landesmuseum, also known as the Ferdinandeum, in Innsbruck. References. Dipauli (1834); Wurzbach (1865: 1–5); Thaler (2003).

**1781–1784**. *Verzeichniss und Beschreibung der Tyroler-Insecten. I. Theil Käferartige Insecten.* Johann Caspar Füessly, Zürich. (8vo) NCSU, GB

*I. Band.* [2] + xii + 248 pp. [DP: 1781 (title page; *Vorrede* dated 14 October 1780), July 1781 (*Allg Verz Bücher*)].

*II. Band.* xiv + 176 pp. [DP: 1784 (title page), 31 August 1784 (*Nürnb Ztg*)].

An extended review of the first *Band* was published by Leske (1782).

#### Lamarck, Jean Baptiste Pierre Antoine de Monet de (Bazantin-le-Petit, Somme, France: 1 August 1744 – 18 December 1829: Paris, France). French naturalist; soldier (1761–1768); botanist to the King, in charge of the herbarium at the *Jardin du roi*; later, took the chair of insects, worms and microscopic animals at the Muséum National d’Histoire Naturelle in Paris. References. Packard (1901); Landrieu (1909, P); Delange (1984); Jaussaud (2004: 323–326).

**1801**. *Systême des animaux sans vertèbres, ou tableau général des classes, des ordres et des genres de ces animaux; présentant leurs caractères essentiels et leur distribution, d’après la considération de leurs rapports naturels et de leur organisation, et suivant l’arrangement établi dans les galeries du Muséum d’Hist. Naturelle, parmi leurs dépouilles conservées; précédé du discours d’ouverture du Cours de Zoologie, donné dans le Muséum National d’Histoire Naturelle l’an 8 de la République.* Deterville, Paris. viii + 432 pp. (8vo) [DP: “An IX–1801” (title page), 25 January 1801 (*J Typogr Bibl*), 20 March 1801 (*Nouv Litt*), 22 March–20 April 1801 (*Soc Philom*), 20 April 1801 (*Décade*)] GB, GAL

The Coleoptera are on pages 203–242. Another issue published the same year [GB] has an additional page numbered “402 bis.” Another printing was issued by Maillard, Déterville and Moulardier (*n.v.*) in 1802 (Bather 1908: 476).

This work has been placed on the *Official List of Works Approved as Available for Zoological Nomenclature* in Direction 32 (ICZN 1956a) with the endorsement that nothing in the work is to be treated as constituting the designation or selection of type species for the genera discussed therein.

**1812**. *Extrait du cours de zoologie du Muséum d’Histoire naturelle, sur les animaux sans vertèbres; présentant la distribution et la classification de ces animaux, les caractères des principales divisions, et une simple liste des genres; a l’usage de ceux qui suivent ce cours.* D’Hautel [&] Gabon, Paris. 127 pp. (8vo) [DP: October 1812 (title page), 6 November 1812 (*Bibl Fr*), 9 November 1812 (*Acad Sci Fr*), 19 December 1812 (*Merc Fr*)] GB, BHL

The classification of the Coleoptera is on pages 71–80.

**1817**. *Histoire naturelle des animaux sans vertèbres, présentant les caractères généraux et particuliers de ces animaux, leur distribution, leurs classes, leurs familles, leurs genres, et la citation des principales espèces qui s’y rapportent; précédée d’une introduction offrant la détermination des caractères essentiels de l’animal, sa distinction du végétal et des autres corps naturels, enfin, l’exposition des principes fondamentaux de la zoologie. Tome quatrième.* Deterville [&] Verdière, Paris. 603 pp. (8vo) [DP: March 1817 (title page), 24 March 1817 (*Acad Sci Fr*), 5 April 1817 (*Bibl Fr*)] McG, GB, GAL

The entire work consists of seven volumes, 1815–1822. The Coleoptera are on pages 266–603 of volume 4. A second, in 11 volumes, and third editions, in three volumes, were published by Gérard Paul Deshayes and Henri Milne-Edwards [*q.v.*]. An Italian resumé was issued by F. Baldassini in 1834 [*q.v.*].

#### Lameere, Auguste Alfred Lucien (Ixelles, Belgium: 12 June 1864 – 6 May 1942: Brussels, Belgium). Belgian naturalist mainly interested in Coleoptera and particularly the Cerambycidae; professor at the Université libre de Bruxelles; his collection of cerambycides, which included almost all the types of the species he had described then, was given in 1901 to the *Institut Royal des Sciences Naturelles de Belgique* in Brussels. References. Selys-Longchamps (1954, P); Pauly (2001: 53–54, P).

**1900**. *Manuel de la faune de Belgique. Tome II. Insectes inférieurs avec 721 figures. Corrodants, dermaptères, orthoptères, plécoptères, agnathes, odonates, thysanoptères, hémiptères, planipennes, panorpates, trichoptères, coléoptères.* H. Lamertin, Bruxelles. 857 + [1] pp. (12mo) [DP: 1900 (title page), 30 September 1900 (*Bibl Belg*), 13 October 1900 (*Acad R Sci Belg*), November 1900 (*Réper Bibl Lib Fr*), December 1900 (*Ent News* 11: 634), January 1901 (*Nat Nov*)] GB

The entire series *Manuel de la faune de Belgique* by Lameere consists of three volumes, 1895–1907. The Coleoptera are on pages 258–830 of the second volume.

#### Laporte, François Louis Nompar de Caumont (Comte de Castelnau) (London, United Kingdom: 24 December 1802 – 4 February 1880: Melbourne, Australia). French naturalist; led scientific expeditions to North America (1837–1841) and South America (1843–1847); served as French consul in several cities in the world including Bahia, Cape Town, and Melbourne (1863–1877); his first collection was given to the National Institution of the Promotion of Science in Washington DC (precursor to the Smithsonian) in 1842 but was probably destroyed by fire in January 1865, while part of his later collection was left to the Melbourne Museum in Australia. References. Whitley (1965, P); Papavero (1971: 149–159); Gilbert (2005: 34); Cambefort (2006: 140); Evenhuis (2012b, P).

**1834–[1835**]. *Etudes entomologiques, ou description d’insectes nouveaux: et observations sur leur synonymie.* Méquignon-Marvis Père et Fils, Paris. 159 pp. + 4 pls. (8vo) CNC

This work was issued in two livraisons: **1**: (pp. 1–94 + pls 1–2) 1834 (wrapper), 9 August 1834 (*Bibl Fr*), 1 September 1834 (*Acad Sci Fr*), 5 November 1834 (*Soc Ent Fr*), 3 December 1834 (*Lit Ztg*); **2**: (pp. 95–159 + pls 3–4) 1834 (wrapper)^61^, January–March 1835 (*Soc Ent Fr* [*Annales*, p. xxxiv], 3 June 1835 (*Soc Ent Fr*), 26 October 1835 (*Acad Sci Fr*). Hagen (1862: 450) dated this work 1834.

Since a fire destroyed all unsold copies of the livraisons in December 1835, the work was reprinted with a new title page dated 1835 and reading “*Études entomologiques, ou description d’insectes nouveaux, et observations sur la synonymie. Première partie. Carnassiers*” [GB, SBB].

This series was continued in 1835 and 1836 in volumes 3 and 4 of the journal *Revue Entomologique*.

**1835–1838**. [Laporte, F.L.N. and Gory, H.] *Histoire naturelle et iconographie des insectes coléoptères, publiée par monographies séparées. Tome I. Monographie des Psilocera, Eurydera, Nycteis, Eunostus, buprestides (chrysochroïtes et agrilites).* P. Duménil, Paris. (8vo) CNC, GB

The entire series was issued in 52 livraisons. Each livraison came with five plates and one or two *feuilles* of text forming four volumes of text and three volumes of plates. The first three volumes of text and the corresponding plates, livraisons 1–42, were authored by Laporte and Gory (volumes 1 and 3) and Gory and Laporte (volume 2). Volume 4, livraisons 43–52, was authored by Gory alone, 1840–1841 [*q.v.*].

The publication dates of the first 42 livraisons, as far as known, are as follows: **1**: April–June 1835 (*Soc Ent Fr* [*Annales*, p. l]), 2 September 1835 (*Soc Ent Fr*), 28 September 1835 (*Acad Sci Fr*); **2**: April–June 1835 (*Soc Ent Fr* [*Annales*, p. l]), 2 September 1835 (*Soc Ent Fr*), 5 October 1835 (*Acad Sci Fr*); **3**: July–September 1835 (*Soc Ent Fr* [*Annales*, p. lix]), 12 October 1835 (*Acad Sci Fr*), 4 November 1835 (*Soc Ent Fr*); **4**: 30 November 1835 (*Acad Sci Fr*), 2 December 1835 (*Soc Ent Fr*); **5**: 15 February 1836 (*Acad Sci Fr*), 17 February 1836 (*Soc Ent Fr*); **6**: 30 May 1836 (*Acad Sci Fr*), 6 July 1836 (*Soc Ent Fr*); **7**: April–June 1836 (*Soc Ent Fr* [*Annales*, p. xxxviii]), 4 July 1836 (*Acad Sci Fr*; *Soc Ent Fr*); **8**: July–September 1836 (*Soc Ent Fr* [*Annales*, p. liv]), 24 October 1836 (*Acad Sci Fr*); **9**: 24 October 1836 (*Acad Sci Fr*); **10**: 21 December 1836 (*Soc Ent Fr*), 24 December 1836 (*Bibl Fr*), 20 February 1837 (*Acad Sci Fr*); **11**: 15 February 1837 (*Soc Ent Fr*), 20 February 1837 (*Acad Sci Fr*); **12**: 17 April 1837 (*Acad Sci Fr*), 19 April 1837 (*Soc Ent Fr*); **13**: 17 July 1837 (*Acad Sci Fr*), 2 August 1837 (*Soc Ent Fr*); **14–15**: 18 September 1837 (*Acad Sci Fr*), 1 November 1837 (*Soc Ent Fr*); **16**: 1 November 1837 (*Soc Ent Fr*); **17**: 17 January 1838 (*Soc Ent Fr*); **18**: 21 March 1838 (*Soc Ent Fr*), 2 April 1838 (*Acad Sci Fr*); **19–21**: 6 August 1838 (*Acad Sci Fr*); **22**: 24 September 1838 (*Acad Sci Fr*); **23–24**: 5 November 1838 (*Acad Sci Fr*); **25–26**: 4 March 1839 (*Acad Sci Fr*), 6 March 1839 (*Soc Ent Fr*); **27–28**: 20 March 1839 (*Soc Ent Fr*), 25 March 1839 (*Acad Sci Fr*); **29–30**: 24 June 1839 (*Acad Sci Fr*); **31–33**: 30 September 1839 (*Acad Sci Fr*), 2 October 1839 (*Soc Ent Fr*); **34**: 2 October 1839 (*Soc Ent Fr*), 23 December 1839 (*Acad Sci Fr*); **35**: 18 December 1839 (*Soc Ent Fr*), 23 December 1839 (*Acad Sci Fr*); **36**: 5 February 1840 (*Soc Ent Fr*), 10 February 1840 (*Acad Sci Fr*); **37**: 18 March 1840 (*Soc Ent Fr*), 27 April 1840 (*Acad Sci Fr*); **38**: 27 April 1840 (*Acad Sci Fr*); **39–42**:

Although the dates of publication of the livraisons are relatively well known, very little information is available about the content of each livraison. In the introduction of the first volume, the authors reported that livraison 3 (published in 1835) deals with the carabid genera *Psilocera*, *Eurydera*, *Nycteis*, *Eunostus*, livraisons 8–11 (published in 1836) with the genus *Clytus*, and the remaining ones (livr. 1–2, 4–7, 12–42) with the buprestides. I also found on the Internet that the first livraison contains the genera *Sternocera* (12 pp.) and *Julodis* (20 pp.). Table 1 gives a list of the taxa treated in Laporte & Gory (1835–38, tome 1) and Gory & Laporte (1839–40, tome 2) along with the number of species treated, number of pages and plates, and dates of publication reported by Nelson and Bellamy (1993: 298)^62^ and those found in this research.

**Table 1.** List of taxa treated in Laporte & Gory (1835-38) and Gory & Laporte (1839-40).

Taxa	Volume	No of species	No of pages	No of plates	Nelson & Bellamy dates	Dates found in this research
*Sternocera*	1	12	12	3	1835, 1840	1835
*Julodis*	1	43	31	9	1835, 1840	1835
*Acmaeodera*	1	53	31	9	1835, 1840	?1835
*Chrysochroa*	1	28	26	7	1835, 1840	?1835
*Chrysodema*	1	33	26	6	1835, 1840	1837*
*Ptosima*	1	4	5	1	1835, 1840	1838†
*Nascio*	1	1	3	1	1837	1838†
*Acherusia*	1	1	2	1	1837	1838†
*Asthraeus*	1	1	2	1	1837	1838†
*Bulis*	1	2	3	1	1837	1838†
*Bubastes*	1	1	2	1	1836	1838†
Acantha	1	2	3	1	1837	1838†
*Apatura*	1	15	10	2	1837	1838†
*Aurigena*	2	4	5	1	1836	1838†
*Capnodis*	2	10	10	2	1836	1838†
*Coeculus*	2	5	5	1	1839, 1840	1838†
*Buprestis*	2	221	160	39	1836 (pp. 1–64) 1837 (65–160)	1837 (pp. 1–48)* 1838 (49–160)†
*Stigmodera*	2	87	72	16	1838	1837*
*Polycesta*	2	7	6	1	1838, 1840	1837*
*Colobogaster*	2	17	17	3	1837	?1836
*Chrysobothris*	2	81	59	10	1837	?1836
*Belionota*	2	6	7	1	1838	1837*
*Castalia*	2	3	4	1	1839	1838†
*Paecilonata*	2	6	5	1	1839	1838†
*Zemina*	2	9	6	2	1839	1838†
*Stenogaster*	2	3	3	1	1839	1838†
*Eurybia*	2	1	2	1	1838	1838†
*Agrilus*	2	94	70	15	1837	1839‡
*Pseudagrilus*	2	1	2	1	1839	1839‡
*Amorphosoma*	2	16	13	3	1839	1839‡
*Eumerus*	2	5	5	1	1839	1839‡
*Coraebus*	2	28	18	4	1839	1839‡
*Anthaxia*	2	49	37	8	1839	1839‡
*Evagora*	2	10	7	2	1839	1839‡
*Sphenoptera*	2	62	40	10	1839	1839‡
*Cratomerus*	2	1	2	1	1839	1839‡
*Sponsor*	2	1	2	1	1839	1839‡
*Cisseis*	2	6	5	1	1839	1839‡
*Ethon*	2	8	6	1	1839	1840!
*Brachys*	2	13	9	2	1840	1840!
*Trachys*	2	13	11	2	1840	1840!
*Aphanisticus*	2	6	5	1	1840	1840!

* Based on Erichson’s (1838: 220–221) review of the entomological literature for the year 1837.

† Based on Erichson’s (1839: 324–328) review of the entomological literature for the year 1838.

‡ Based on Erichson’s (1840: 240–242) review of the entomological literature for the year 1839.

! Based on Erichson’s (1841: 162) review of the entomological literature for the year 1840.

[**1836–1837**]. [Laporte, F.L.N. and Gory, H.] *Monographie du genre Clytus.* J.B. Baillières, Paris. 124 pp. + 20 pls. (8vo) (*n.v.*).

This work was issued as livraisons 8–11 of Laporte and Gory’s “*Histoire naturelle et iconographie des insectes Coléoptères, publiée par monographies séparées*” as listed on page 6 of the first volume of the series. These livraisons were noticed as follows: **8**: July–September 1836 (*Soc Ent Fr* [*Annales*, p. liv]), 24 October 1836 (*Acad Sci Fr*); **9**: 24 October 1836 (*Acad Sci Fr*); **10**: 21 December 1836 (*Soc Ent Fr*), 24 December 1836 (*Bibl Fr*), 20 February 1837 (*Acad Sci Fr*); **11**: 15 February 1837 (*Soc Ent Fr*), 20 February 1837 (*Acad Sci Fr*). The content of each livraison is desired.

This work is dated 1835 by Agassiz (1850: 200) and Hagen (1862: 450) but Sherborn (1922a: lxxviii) believed it was issued in 1836. A copy (titled “travail manuscrit”) was presented to the *Académie des Sciences* on 14 December 1835 (Anonymous 1835: 472) for which Duméril (1836) read a report at the academy meeting of 4 January 1836 for approval. The work was probably printed in 1835 but accessible to the public only during 1836.

The monography was reissued under the title “*Histoire naturelle et iconographie des insectes coléoptères, par H. Gory. Monographie des Clytus. Texte et planches, tome III*” by P. Duménil, Paris, in 1841 [CNC, GB]. Due to an error by the printer, the name of Laporte was left out on the title page and so a second title page was issued later with the difference that “*par H. Gory*” was changed to “*par H. Gory et F.-L. de La Porte, Comte de Castelnau*.” In the first printing, the author order was Laporte and Gory.

**1840**. *Histoire naturelle des insectes coléoptères; avec une introduction renfermant l’anatomie et la physiologie des animaux articulés, par M. Brullé; ouvrage accompagné de 155 planches gravées sur acier représentant plus de 800 sujets.* P. Duménil, Paris. (8vo) CNC, GB

*Tome premier.* cxxv + 324 pp. + 19 pls. [DP: 1840 (title page), 26 December 1840 (*Acad Sci Fr*), December 1840 (Sherborn 1922: lxxvii)].

*Tome deuxième.* 563 + [1 (Tableau du placement des planches)] pp. + 38 pls. [DP: 1840 (title page), 26 December 1840 (*Acad Sci Fr*), December 1840 (Sherborn 1922: lxxvii)].

The half-title page reads “*Histoire naturelle des animaux articulés, annelides, crustacés, arachnides, myriapodes et insectes.*” Engelmann (1846: 527) indicated that these two volumes were originally issued in livraisons, 1835–1840. I found no evidence to support this^63^. The plates of this work are uncolored. These two volumes were reprinted in 1850 in Paris under the title “*Histoire naturelle des insectes Coléoptères; avec une introduction renfermant l’anatomie et la physiologie des animaux articulés, par M. Brullé; ouvrage accompagné de 81* [*46* for tome 2] *planches gravées sur acier et coloriées au pinceau avec le plus grand soin, représentant plus de 500 sujets*” [BHL] with continuously numbered and colored plates.

The *Histoire naturelle des animaux articulés, annelides, crustacés, arachnides, myriapodes et insectes* was issued in four volumes, the first two dealing with Coleoptera, the third one by Emile Blanchard with Orthoptera, Neuroptera, Hemiptera, Hymenoptera, Lepidoptera and Diptera, and the fourth one by Pierre Hippolyte Lucas with arachnids and myriapods.

#### Latreille, Pierre André (Brives-la-Gaillarde, Corrèze, France: 29 November 1762 – 6 February 1833: Paris, France). French naturalist; illegitimate son of general Jean Baptiste Joseph Sahuguet d’Amarzit, baron of Espagnac; moved to Paris at the age of 16 to pursue his studies and eventually obtained his master from the University of Paris; turned to priesthood and was ordained in 1786; jailed in 1793 first at Brives and later at Bordeaux as a non-juror priest and sentenced to be deported to Guyana but released at the last minute when the prisoners boarded the ship; later, professor at the Muséum National d’Histoire Naturelle in Paris; his first collection was sold to Pierre François Marie Dejean [*q.v.*] in 1826 while his second collection was acquired by Thomas Norris of Manchester and sold at auction in London in 1873. References. Peyerimhoff (1932: 6–7, 66–68); Dupuis (1974); Adler (1989: 19, P); Smetana and Herman (2001: 94–96, P); Jaussaud (2004: 333–334); Cambefort (2006: 213–215); Piguet (2007).

[**1797**]. *Précis des caractères génériques des insectes, disposés dans un ordre naturel.* Prévôt, Paris [&] F. Bourdeaux, Brive. xiii + [1] + 201 + [7] pp. (8vo) [DP: “An 5” (title page), 30 January 1797 (*Acad Sci Fr*)^64^, 21 December 1796–18 February 1797 (*Soc Philom*), 29 July 1797 (*Intell Allg Lit Ztg*), July–August 1797 (*Esprit J*)] GB, BHL

This work is usually dated 1796 (Latreille 1817: 270; Duméril 1822: 519; Hagen 1862: 452; Horn and Schenkling 1928b: 690). Evenhuis (1997b: 437) presented arguments as to why the book should be dated 1797.

A facsimile edition was published by A. Hermann, Paris, in 1907 [CNC, YB].

**1802**. *Histoire naturelle des fourmis, et recueil de mémoires et d’observations sur les abeilles, les araignées, les faucheurs, et autres insectes. Avec figures.* Barrois, Paris. xvi + 445 pp. + 12 pls. (8vo) [DP: “An X–1802” (title page), April 1802 (Latreille 1802: 369), 30 May 1802 (*Décade*), 21 May–19 June 1802 (*J Lit Fr*), 13 July 1802 (*J Typogr Bibl*), July 1802 (*Esprit J*), 23 October–21 November 1802 (*Mag Encycl*), 11 December 1802 (*Intell Allg Lit Ztg*)] MCZ, GB, BHL, GAL

One section (pp. 396–401), entitled “*Mémoire sur un nouveau genre d’insectes, précédé de quelques observations sur les genres qui l’avoisinent*,” contains the description of the new beetle genus *Elmis*.

**1802–1805**. *Histoire naturelle, générale et particulière, des crustacés et des insectes. Ouvrage faisant suite aux oeuvres de Leclerc de Buffon, et partie du cours complet d’histoire naturelle rédigé par C.S. Sonnini, membre de plusieurs sociétés savantes.* F. Dufart, Paris. (8vo) CNC, BHL, GB

*Familles naturelles des genres. Tome troisième.* xii + pp. 13–467 + [1 (Errata)] pp. [DP: “An X” (title page), 6 November 1802 (*J Typogr Bibl*), 22 November–21 December 1802 (*J Phys*)]. Griffin (1938: 157) had shown that the date on the title page is incorrect and that the book was issued in an XI of the Revolutionary Calendar (23 September 1802–23 September 1803); he also pointed out that the date of May–September 1802 given by Richards (1935: 174) was inexact. Copies of this volume are known with the title reading “*Histoire naturelle, générale et particulière, des crustacés et des insectes. Ouvrage faisant suite à l’histoire naturelle générale et particulière, composée par Leclerc de Buffon, et rédigée par C.S. Sonnini, membre de plusieurs sociétés savantes.*”

*Tome huitième.* 411 pp. + pls 67–73. [DP: “An XII” (title page), February–March 1804 (Dupuis 1986: 208)].

*Tome neuvième.* 416 pp. + pls 74–80. [DP: “An XII” (title page), 19 August–17 September 1804 (*J Phys*; Dupuis 1986: 209)].

*Tome dixième.* 445 pp. + pls 81–90. [DP: “An XII” (title page), 19 August–17 September 1804 (*J Phys*; Dupuis 1986: 209)].

*Tome onzième.* 422 pp. + pls 91–93. [DP: “An XII” (title page), 19 August–17 September 1804 (*J Phys*; Dupuis 1986: 209)].

*Tome douzième.* 424 pp. + pls 94–97. [DP: “An XII” (title page), 19 August–17 September 1804 (*J Phys*; Dupuis 1986: 209)].

The entire work consists of 14 volumes, 1802–1805. The Coleoptera are in volumes 3 (pp. 74–239), 8–11, and 12 (pp. 1–80). According to Dupuis (1986: 210), some parts dealing with Coleoptera were authored by Anselme Gaetano Desmarest [1784–1838].

Separate printings of the third volume are known. Apparently the first printing had some typographic errors which were corrected in the subsequent printings (Evenhuis 1997b: 439).

**1804**. Tableau méthodique des insectes. Pp. 129–200 *in*: *Nouveau dictionnaire d’histoire naturelle, appliquée aux arts, principalement à l’agriculture et à l’économie rurale et domestique: par une société de naturalistes et d’agriculteurs: avec des figures tirées des trois règnes de la nature. Tome XXIV.* Déterville, Paris. 84 (Addition d’articles connus pendant l’impression de ce dictionnaire) + 85 (Avis de l’éditeur + Explication et développemens des caractères + Table des noms latins) + 238 (Tableaux méthodiques d’histoire naturelle) + 18 (Table alphabétique des figures) + 34 (Liste alphabétique des souscripteurs) pp. (8vo) [DP: “An XII” (title page), 7 March 1804 (*J Typogr Bibl*), 21 February–21 March 1804 (*J Lit Fr*), 20 April 1804 (*J Paris*)] CISTI, GB

The Coleoptera are on pages 138–161 in Latreille’s contribution.

**1805–1809**. *Genera crustaceorum et insectorum secundum ordinem naturalem in familias disposita, iconibus exemplisque plurimis explicata.* Amand Koenig, Parisiis et Argentorati. (8vo) CNC, BHL, GB

*Tomus primus.* xviii + 302 + [1 (Emendanda)] pp. + 16 pls. [DP: 1806 (title page; *proemium* dated August 1805), 17 September 1805 (*J Typogr Bibl*), 28 September 1805 (*Intell Allg Lit Ztg*), November 1805 (*Mag Encycl*)].

*Tomus secundus.* 280 pp. [DP: 1807 (title page), 1806 (*J Lit Fr* 9 (11): 321), 8 February 1807 (*J Typogr Bibl*), 15 April 1807 (*Télé Litt*), 27 June 1807 (*Intell Allg Lit Ztg*)].

*Tomus tertius.* 258 + [1] pp. [DP: 1807 (title page), 15 April 1807 (*Télé Litt*), 4 May 1807 (*J Typogr Bibl*), 27 June 1807 (*Intell Allg Lit Ztg*), 10 August 1807 (*Acad Sci Fr*)].

*Tomus quartus et ultimus*. 399 pp. [DP: 1809 (title page), 25 February 1809 (*Télé Litt*), 20 March 1809 (*J Typogr Bibl*), 10 April 1809 (*Acad Sci Fr*), January–June 1809 (*Verz Bücher Landkarten*)]. Although this volume does not deal with Coleoptera, the “*Addenda et castiganda*” (pp. 367–388) contains information relevant to beetles.

The work was published in four volumes and came with colored or uncolored figures. The first and second volumes of the series were issued prior to the year indicated on their title pages.

**1809–1817**. Insectes de l’Amérique équinoxiale, recueillis pendant le voyage de MM. de Humboldt et Bonpland. Pp. 197–283, 344–397 + pls 15–18, 22–25 [vol. 1], pp. 9–138 + pls 31–43 [vol. 2] *in*: *Recueil d’observations de zoologie et d’anatomie comparée; faites dans l’océan Atlantique, dans l’intérieur du nouveau continent et dans la mer du sud, pendant les années 1799, 1800, 1801, 1802 et 1803, par Al. de Humboldt et A. Bonpland.* Levrault, Schoell et Comp.^ie^, Paris. (4to) MCZ, AMNH

*Premier volume.* viii + 412 pp. + 30 pls. This volume was published in six livraisons: **1**: (pp. i–viii + 1–46 + [2] + pls 1–7) 1805 (title page)^65^; **2**: (pp. 49–104) July 1807 (*Mag Encycl*); **3**: (pp. 105–196) May 1807 (Evenhuis 1997a: 381), July 1807 (*Mag Encycl*); **4**: (pp. 197–293) 1809 (Sherborn 1899b: 428); **5–6**: (pp. 294–412) 1809 (Sherborn 1899b: 428), 9 November 1810 (*Allg Lit Ztg*). The title page is dated “An XIII–1805” and the preface (p. viii) February 1805. The plates have no nomenclatural significance since there are no caption names. Two issues were published simultaneously for the first volume (see Sherborn 1899b: 428). The collation given here is for the first issue. A short review of the six livraisons of the second issue was published in April 1810 (Anonymous 1810). A second edition of this volume [GB, GAL] forming the seventh livraison and containing viii + 368 pages and 30 plates was issued in 1812 (Sherborn 1899b: 428; MacGillavry 1931: 175) despite that the title page is dated 1811^66^; Latreille’s contribution is on pages 127–252. This edition is essentially identical to the first one except that some errors were corrected; none of them affect scientific names for Coleoptera.

*Second volume.* Smith et Gide. 352 pp. + pls 31–57. This volume was issued in seven livraisons: **8**: (pp. 1–64 + pls 31–34) 2 January 1813 (*Bibl Fr*); **9**: (pp. 65–96 + pls 35–38) 24 September 1813 (*Bibl Fr*); **10**: (pp. 97–144 + pls 39–44) 13 December 1817 (*Bibl Fr*); **11–12**: (pp. 145–224 + pls 45–48, 48*bis*, 49, 51) 1821 (wrapper), 15 September 1821 (*Bibl Fr*), 17 September 1821 (*Acad Sci Fr*); **13**: (pp. 225–256 + pls 50, 52–54) 1827 (wrapper), 17 January 1827 (*Bibl Fr*), 26 March 1827 (*Acad Sci Fr*); **14**: (pp. 257–352 + pls 55–57) 15 December 1832 (*Bibl Fr*). The title page is dated 1833. I have seen wrappers for livraisons 11–13 only.

The expedition report of the “*Voyage aux régions équinoxiales du nouveau continent, fait en 1799, 1800, 1801, 1802, 1803 et 1804, par Al. de Humboldt et A. Bonpland*” was published in 23 volumes, 1805–1837, divided into six parts, each with their own title. A facsimile of the two volumes on zoology was published at 200 copies in 1972 by Theatrvm Orbis Terrarvm, Amsterdam [&] Da Capo Press, New York under the title “*Recueil d’observations de zoologie et d’anatomie comparée, Paris 1799–1803 (par Al. de Humboldt et A. Bonpland)*.”

A list of all new species described in this work is included in MacGillavry (1931: 176–180). No names are listed on the plates and so they are not nomenclaturally significant.

**1810**. *Considérations générales sur l’ordre naturel des animaux composant les classes des crustacés, des arachnides, et des insectes; avec un tableau méthodique de leurs genres, disposés en familles.* F. Schoell, Paris. 444 pp. (8vo) [DP: 1810 (title page), 23 May 1810 (Evenhuis 1997b: 440), 24 May 1810 (*Télé Litt*), 30 May 1810 (*Intell Jena Lit Ztg*), 9 June 1810 (*Allg Lit Ztg*)] CNC, GB, BHL

This work includes a “*Table des genres avec l’indication de l’espèce qui leur sert de type*” (pp. 421–444) where type species of many beetle genera are listed for the first time. This work has been placed on the *Official List of Works Approved as Available for Zoological Nomenclature* in Direction 4 (ICZN 1954a: 635).

**1816**. *Le règne animal distribué d’après son organisation, pour servir de base à l’histoire naturelle des animaux et d’introduction à l’anatomie comparée. Par M. le Ch^er^. Cuvier. Avec figures, dessinées d’après nature. Tome III, contenant les crustacés, les arachnides et les insectes.* Déterville, Paris. xxix + 653 pp. (8vo) [DP: 1817 (title page), 2 December 1816 (*Acad Sci Fr*; Roux 1976: 31), 7 December 1816 (*Bibl Fr*)] CNC, BHL, GB

The entire work consists of four volumes, issued simultaneously in 1816 (Dickinson et al. 2011: 85). The third volume was authored by Latreille and the Coleoptera are on pages 170–365. It was translated in German by Schinz [*q.v.*] in 1823.

**1818**. *Tableau encyclopédique et méthodique des trois règnes de la nature. Vingt-quatrième partie. Crustacés, arachnides et insectes.* Agasse, Paris. 38 + [1 (Fautes essentielles à corriger)] pp. + pls 269–397. (4to) [DP: 1818 (title page), 8 August 1818 (*Bibl Fr*)] CNC, GB, BHL

This publication constitutes livraison 86 of the “*Encyclopédie méthodique*” published by Charles-Joseph Panckoucke and includes plates 269–397 with an explanatory text (pp. 1–38) where scientific names are provided for each illustration. The Coleoptera are on plates 356–372. The first 268 plates, of which plates 135–246 pertain to Coleoptera, were issued 1790–1797 under the editor Pierre-Joseph Bonnaterre (Evenhuis 2003: 37). Only vernacular names were provided on the plates. However, an explanatory text for these plates with scientific names was published in 1828 (*n.v.*) with livraison 100 of the series (Evenhuis 2003: 37); the text (142 pp.) was reissued along with the plates in 1830 under the title “*Tableau encyclopédique et méthodique des trois règnes de la nature. Insectes, papillons, crustacés et arachnides*” [GAL] with Latreille as the author. Remarks about Panckoucke’s *Encyclopédie* are included under Olivier, 1790–1811 [*q.v.*].

**1822–1826**. [Latreille, P.A. and Dejean, P.F.M.A.] *Histoire naturelle et iconographie des insectes coléoptères d’Europe.* Crevot, Paris. 198 pp. + 15 pls. (8vo) CNC, GB

This work was issued in three livraisons: **1**: (pp. 1–90 + pls i–v) 1822 (wrapper), 20 July 1822 (*Bibl Fr*), 5 August 1822 (*Acad Sci Fr*), August 1822 (*Not Geb Natur*), July–August 1822 (*Rev Bibl-Brus*); **2**: (pp. 91–134 + pls vi–x) 1824 (wrapper), 15 November 1824 (*Acad Sci Fr*), 20 November 1824 (*Bibl Fr*), 15 December 1824 (*Rev Bibl-Brus*), December 1824 (*Not Geb Natur*); **3**: (pp. 135–198 + pls xi–xv) 1824 (wrapper)^67^, 13 February 1826 (*Acad Sci Fr*), 15 February 1826 (*Bibl Fr*), January–February 1826 (*Rev Bibl-Brus*).

As stated on page 38, Latreille was responsible for the generalities and systematics, while Dejean was responsible for the descriptions of the species. Two new genera were described in this work, *Tricondyla* and *Calleida*, and they should be credited to Latreille. Sixteen new species were described and are credited to Dejean. In addition, six new species were made available by illustrations on the plates: *Therates caerulea* (pl. 1, fig. 2), *Therates spinipennis* (pl. 1, fig. 3), *Collyris major* (pl. 2, fig. 4), *Graphipterus minutus* (pl. 6, fig. 4), *Galerita unicolor* (pl. 6, fig. 6), *Cordistes maculatus* (pl. 7, fig. 5). These species should be credited to Latreille and Dejean (see Bousquet 2004: 41). This work came with colored or uncolored plates.

**1825**. *Familles naturelles du règne animal, exposées succinctement et dans un ordre analytique, avec l’indication de leurs genres.* J.-B. Baillière [&] Baudouin Frères, Paris. 570 pp. (8vo) [DP: 1825 (title page; preface dated 20 October 1824), 16 May 1825 (*Acad Sci Fr*), 21 May 1825 (*Bibl Fr*), 15 June 1825 (*Rev Bibl-Brus*), June 1825 (*J Pharm*; *Not Geb Natur*)] CNC, GB

All supraspecific names in this work are in French. Berthold [*q.v.*] published in 1827 a German translation of Latreille’s work and provided Latinization for the vernacular names. A German translation of parts of this work was issued by Thon in his *Entomologisches Archiv* 1(1) [1827]: 10–12; 1(2) [1827]: 21–24; 1(3) [1828]: 47–79; 1(4) [1828]: 93–112. The translation includes the Coleoptera section (pp. 48–71).

**1825–1828**. [Latreille, P.A., Lepeletier, A.L.M., Audinet-Serville, J.G. and Guérin-Méneville, F.E.] *Encyclopédie méthodique, ou par ordre de matières; par une société de gens de lettres, de savans et d’artistes; précédée d’un vocabulaire universel, servant de table pour tout l’ouvrage, ornée des portraits de Mm. Diderot & d’Alembert, premiers éditeurs de l’Encyclopédie. Histoire naturelle. Entomologie, ou histoire naturelle des crustacés, des arachnides et des insectes. Tome dixième.* Mme Veuve Agasse, Paris. 832 + [1] pp. (4to) CNC, GB

This volume was published in two livraisons: **1**: (pp. 1–344 [livraison 96 of the entire *Encyclopédie*]) 1 October 1825 (*Bibl Fr*), 30 October 1825 (*Rev Bibl-Brus*); **2**: (pp. 345–832 + [1 (Errata)] [livraison 100]) 13 December 1828 (*Bibl Fr*). Remarks about the *Encyclopédie Méthodique* are included under Olivier, 1790–1811 [*q.v.*].

**1827**. Description des insectes figurés sur la pl. LVIII du tom. II, recueillis par M. Cailliaud, et décrits par M. Latreille. Pp. 271–292 *in*: *Voyage à Méroé, au Fleuve Blanc, au-delà de Fâzoql dans le midi du royaume de Sennâr, à Syouah et dans cinq autres oasis; fait dans les années 1819, 1820, 1821 et 1822, par M. Frédéric Cailliaud, de Nantes. Accompagné de cartes géographiques, de planches représentant les monumens de ces contrées, avec des détails relatifs à l’état moderne et à l’histoire naturelle. Dédié au Roi. Tome quatrième.* Debure, Paris. 416 pp. (8vo) [DP: 1827 (title page), 15 September 1827 (*Bibl Fr*), 20 October 1827 (*Rev Bibl-Brus*), July–October 1827 (*Foreign Quart Rev*)] McG, GB, GAL

The account of the *voyage* was published in four volumes, 1823–1827, and one atlas. It was reprinted by Cregg International Publishers, 1972, and by LTR-Verlag, 1982. The new Coleoptera described in Latreille’s contribution are: *Scarites heros* (p. 274), *Elater notodonta* (p. 275), *Buprestis cailliaudi* (p. 277), *Ateuchus aegyptiorum* (p. 279), *Gymnopleurus bicolor* (p. 281), *Melolontha pallidula* (p. 284), *Cantharis aethiops* (p. 286) and *Mylabris trigrinipennis* (p. 286). The title page of the atlas is dated 1823 but there are no names associated with figure 58, just numbers.

This voyage was the last of the French explorer Frédéric Cailliaud [1787–1869]. He went to the deserts of Egypt, Ethiopia, and Nubia (Sudan), where he discovered several important remains of ancient Egypt and the Roman Empire. His descriptions and views are important today, because many monuments were destroyed shortly after he had seen and drawn them.

**1829**. *Le règne animal distribué d’après son organisation, pour servir de base à l’histoire naturelle des animaux et d’introduction à l’anatomie comparée. Par M. le Baron Cuvier. Avec figures, dessinées d’après nature. Nouvelle édition, revue et augmentée.* Déterville, Paris. (8vo) CNC, BHL

*Tome IV. Crustacés, arachnides et partie des insectes.* xxvii + 584 pp. [DP: 1829 (title page), 11 April 1829 (*Bibl Fr*), 27 April 1829 (*Acad Sci Fr*), 15 May 1829 (*Rev Bibl-Brus*), June 1829 (*Not Geb Natur*)].

*Tome V. Suite et fin des insectes.* xxiv + 556 pp. [DP: 1829 (title page), 11 April 1829 (*Bibl Fr*), 27 April 1829 (*Acad Sci Fr*), 15 May 1829 (*Rev Bibl-Brus*), June 1829 (*Not Geb Natur*)].

These two volumes were also issued in 1829 under the title “*Les crustacés, les arachnides et les insectes, distribués en familles naturelles, ouvrage formant les tomes 4 et 5 de celui de M. le Baron Cuvier sur le règne animal (deuxième édition)*” by the same publisher. The entire series consists of five volumes, 1829–1830. The first three were authored by Georges Cuvier [*q.v.*]. The Coleoptera are on pages 352–581 of volume 4 and 1–167 of volume 5.

An English translation of Latreille’s two volumes was published in New York, 1831, as volumes 3 and 4 of “*The animal kingdom arranged in conformity with its organization, by the Baron Cuvier. The crustacea, arachnides and insecta by P.A. Latreille. Translated from the French, with notes and additions, by H. M’Murtrie. In four volumes, with plates*” [GB, BHL]. The Coleoptera are on pages 264–571 of volume 3. Abridged versions of this translation were issued in 1832, and reprinted in 1833 in New York [GB, BHL] and in 1834 in London (*n.v.*).

Another English translation, essentially similar to that published in 1831 (Evenhuis 1997a: 175), of Latreille’s two volumes was issued by G. Henderson, London, in volumes 3 and 4 of “*The animal kingdom, arranged according to its organization, serving as a foundation for the natural history of animals, and an introduction to comparative anatomy. By Baron Cuvier. With figures designed after nature: the Crustacea, arachnides, & Insecta, by M. Latreille. Translated from the latest French edition. With additional notes, and illustrated by nearly 500*^68^ *additional plates. In four volumes*” [GB]. The entire series was published in 50 parts between 1 July 1833 and 1 April 1835 for the text; the plates cannot be dated at this time but most likely several plates were issued with each part (Evenhuis 1997a: 175). The Coleoptera are on pages 361–472 of volume 3 and 1–144 of volume 4 and on plates 6–61 of the Insecta plates. Most of the plates, at least all those related to beetles except plates 8, 9^69^ and 32, were also issued in Guérin-Méneville’s “*Iconographie du règne animal de G. Cuvier*.” The Coleoptera plates in Guérin-Méneville were published 1829–1837 [*q.v.*] and a number of new taxa were made available by the illustrations. Whether some of the plates for Latreille’s translation were issued earlier than in Guérin-Méneville remains to be documented.

Additional English translations of Latreille’s work were published by Griffith and Pidgeon in 1831–1832 [*q.v.*] and Carpenter and Westwood in 1849 [*q.v.*].

A German translation, with additions, of Latreille’s two volumes was published, 1836–1839, in Leipzig, in volume 5 of the 6-volume series “*Das Thierreichs, geordnet nach seiner Organisation. Als Grundlage der Naturgeschichte der Thiere und Einleitung in die vergleichende Anatomie. Vom Baron von Cuvier. Nach der zweiten, vermehrten Ausgabe übersetzt und durch Zusätze erweitert von F.S. Voigt*” [GB]. The Coleoptera are in the “*Fünfter Band, die eigentlichen Insekten enthaltend*” on pages 1–346, issued in 1839.

#### Leach, William Elford (Plymouth, Devon, United Kingdom: 2 February 1791 – 25 August 1836: Palazzo San Sebastiano near Tortona, Piedmont, Italy). British physician and naturalist; assistant librarian and later curator at the British Museum (Natural History) from 1813 to 1822; afterwards moved with his elder sister to the continent where they stayed at various villas in southern France and northern Italy; his collection was acquired in 1826 by the British Museum (Natural History). References. Knight (1867: 824–825); Mearns and Mearns (1988: 223–226); Damkaer (2002: 147–155); Gilbert (2004b).

**1814–1817**. *The zoological miscellany; being descriptions of new, or interesting animals. Illustrated with coloured figures, drawn from nature, by R.P. Nodder.* E. Nodder & Son, London. (8vo) CNC, BHL

*Vol. I.* 137 pp. + pls 1–60. [DP: 1814 (title page)].

*Vol. II.* 154 pp. + pls 61–120. [DP: 1815 (title page), December 1815 (Evenhuis 1997b: 443)].

*Vol. III.* v + [1 (Errata et corrigenda)] + 151 pp. + pls 121–150. [DP: 1817 (title page), September 1817 (*Ladies Monthly Mus* 6: 50)]. The exact title of this volume is “*The zoological miscellany; being descriptions of new or interesting animals. Illustrated with coloured figures, engraved from original drawings, by R.P. Nodder.*”

This work was issued in parts, starting almost certainly in January 1814 according to Sherborn (1922a: cxxxi). The Royal Society of London were presented by W.E. Leach with no 1 of “*The Zoological Miscellany*” on 3 February 1814, no 2 on 3 March 1814, no 4 on 5 May 1814, no 5 on 9 June 1814, nos 6–10 on 10 November 1814, no 11 on 8 December 1814, no 12 on 12 January 1815, no 13 on 2 February 1815, no 14 on 2 March 1815, no 15 on 6 April 1815, no 16 on 4 May 1815, no 17 on 1 June 1815, nos 18–22 on 9 November 1815, no 23 on 7 December 1815 and no 24 on 11 January 1816 (*R Soc Lond* [*Philosophical Transactions*] 1814: 605–609; 1815: 448–454; 1816: 362–363). These numbers refer to the first two volumes, suggesting that each had 12 issues. I have seen an advertisement, appended to the second volume, stating that “The Third Volume of the Zoological Miscellany will be published on the 1st of January, 1817;” this indicates that the third volume was not issued by parts but published at once on this date. Mathews (1920: 13) also noted that “Vol. III. appeared as one item, January 1, 1817.” Collation of each part for the first two volumes is desired. Mathews (1920: 13) noted that each part contained “five plates and about one signature of text.”

**1815**. Entomology. Pp. 57–172 *in*: *The Edinburgh Encyclopaedia; or dictionary of arts, sciences, and miscellaneous literature. Conducted by David Brewster. With the assistance of gentlemen eminent in science and literature.* [*Vol. IX, part I.*]*^70^* William Blackwood, Edinburgh. 384 pp. (4to) [DP: April 1815 (Sherborn 1937b: 112)] ARC

The entire *Edinburgh Encyclopaedia* was published in 18 volumes, 1808–1830. For dates of publication of each volume, see Sherborn (1937b: 112) and Evenhuis (1997a: 123–124). An American edition, 1812–1831, was published in 18 volumes, each in two parts making 36 volumes in all (see Stone 1900: 686), in Philadelphia by J. and E. Parker [later E. Parker and J. Delaplaine] under the title “*The American edition of the new Edinburgh Encyclopaedia conducted by David Brewster, assisted by upwards of one hundred gentlemen in Europe, most eminent in science and literature, and now improved for the people of the United States, in the civil, religious, and natural history of their country, in American biography and in the great discoveries in mechanics and the arts*.” In this edition, the entry *Entomology* is on pages 646–758 of volume 8, issued in 1816. This American edition was reprinted in 1832 under the title “*The Edinburgh Encyclopaedia, conducted by David Brewster. With the assistance of gentlemen eminent in science and literature. The first American edition, corrected and improved by the addition of numerous articles relative to the institutions of the American continent, its geography, biography, civil and national history, and to various discoveries in science and the arts. In eighteen volumes.*”

**1819**. Appendix. No. IV [Notice of Reptiles, Insects, &c]. Pp. 493–496 *in*: *Mission from Cape Coast Castle to Ashantee, with a statistical account of that kingdom, and geographical notices of other parts of the interior of Africa. By T. Edward Bowdich, Esq. Conductor.* John Murray, London. viii + 512 pp. (4to) [DP: 1819 (title page), 27 March 1819 (*Lit Gaz*), 25 April 1819 (*Gal Weekly Repert*), April 1819 (Peddie and Waddington 1914: 69), May 1819 (*Blackw Mag*; *British Rev*; *Gent Mag*; *Phil Mag*)] LAC, GB

Four Coleoptera genera are made available for the first time in this appendix by description and/or inclusion of an available species: *Tefflus* (p. 494) [generic description and inclusion of *Carabus megerlei* Fab.]; *Odontomerus* (p. 494) [inclusion of *Buprestis serratus* Fab.]; *Phyllotoma* (p. 495) [inclusion of *Melolontha reflexa* Fab.] and *Petrognatha* (p. 495) [generic description and inclusion of *Lamia gigas* Fab.].

The book was reissued with a new introduction and notes in London, 1966, by Cass (*n.v.*). A “New edition, with introductory preface by his daughter, Mrs. Hale” [GB] of Bowdich’s work was issued by Griffith & Farran in London, 1873; some parts were left out including the appendix.

#### LeConte, John Eatton (near Shrewsbury, New Jersey, USA: 22 February 1784 – 21 November 1860: Philadelphia, Pennsylvania, USA). American naturalist and army officer; served in the United States Army Corps of Topographical Engineers (1818–1831), first as captain and later as major; father of John Lawrence LeConte [*q.v.*]; his Coleoptera collection was likely given to his son. References. Barnhart (1917); Brodhead (1997); Adler (2007: 52, P).

**1849**. Coleopterous insects. Pp. 25–36 [Appendix] *in*: *Statistics of the state of Georgia: including an account of its natural, civil, and ecclesiastical history; together with a particular description of each county, notices of the manners and customs of its aboriginal tribes, and a correct map of the state*. *By George White.* W.Thorne Williams, Savannah. 624 + 77 (Appendix) pp. (8vo) [DP: 1849 (title page)] USNM, GB, BHL, ARC

A number of new genera, such as *Albux*, *Argestes*, and *Anisofilia*, are proposed and made available by the inclusion of at least one available species. The section is signed by “John Le Conte.” Hardy et al. (1986: 472) presented evidence suggesting that the author was Major John Eatton LeConte, and not his son, John Lawrence LeConte.

A reprint was issued in 1972 in Spartanburg (South Carolina).

#### LeConte, John Lawrence (New York, New York, USA: 13 May 1825 – 15 November 1883: Philadelphia, Pennsylvania, USA). American entomologist of independent means who became the most outstanding North American coleopterist of the 19th Century; received a medical degree in 1846 but never owned a private practice; visited the south shore of Lake Superior in 1844 with his cousin Joseph LeConte; travelled to the Rocky Mountains the next year; explored the north shore of Lake Superior in 1848 in company of several scientists including Louis Agassiz; went to California via Panama in 1849; volunteered as a surgeon to the Army Medical Corps during the Civil War and soon raised to the rank of lieutenant colonel; served as geologist on a railroad survey through Kansas and New Mexico during the summer of 1867; appointed assistant director of the US Mint in Philadelphia in 1878, a position held until his death; his collection was deposited at the Museum of Comparative Zoology, Cambridge, Massachusetts. References. Horn (1884); Sallé (1884); Scudder (1884); Mallis (1971: 242–248); Zimmerman (1993: 642–643, P); Stephens (1999); Smetana and Herman (2001: 97–98, P).

**1850**. General remarks upon the Coleoptera of Lake Superior. Pp. 201–242* + pl. 8 *in*: *Lake Superior: its physical character, vegetation, and animals, compared with those of other and similar regions. By Louis Agassiz. With a narrative of the tour, by J. Elliot Cabot. And contributions by other scientific gentlemen. Elegantly illustrated.* Gould, Kendall and Lincoln, Boston. x + [2] + 428 pp. + 8 pls. (8vo) [DP: 1850 (title page; preface dated March 1850), 9 March 1850 (*Lit World-NY*), May 1850 (*Amer J Sci Arts*), 31 May 1850 (*Intell Serapeum*), 15 July 1850 (*Bibl Lit Anz*), July 1850 (*Christ Exam*)] CNC

The book was reissued by Arno, New York, in 1970 as part of the “*American environmental studies*” series, and by R.E. Krieger Pub. Co., Huntington (N.Y.), in 1974.

**1857**. *No. 1. Report upon insects collected on the survey.* 72 pp. + 2 pls. (4to) [DP: June 1857] CNC, YB

The copies I have seen and the one reproduced by Hardy et al. (1995: 65–142) have no cover and the publisher and place and date of publication are not listed. However, the paper came with a printed slip reading “Entomological report on route adjacent to 47^th^ parallel; from Vol. 9 of the Reports of Explorations and Survey for a Railroad route from the Mississippi River to the Pacific Ocean: printed in June, 1857.” This publication is a preprint for the official version published in 1860 by Thomas H. Ford in “*Reports of explorations and surveys, to ascertain the most practicable and economical route for a railroad from the Mississippi River to the Pacific Ocean. Made under the direction of the Secretary of War, in 1853–5, according to acts of Congress of March 3, 1853, May 31, 1854, and August 5, 1854. Volume XII. Book II.*” Two editions were published the same year, one for the Senate [GB] and one for the House of Representatives [GB]. LeConte’s contribution was also issued in 1859 by Baillière Brothers, New York, in “*The natural history of Washington Territory, with much relating to Minnesota, Nebraska, Kansas, Oregon, and California, between the thirty-sixth and forty-ninth parallels of latitude, being those parts of the final reports on the survey of the Northern Pacific railroad route, containing the climate and physical geography, with full catalogues and descriptions of the plants and animals collected from 1853 to 1857. By J.G. Cooper, M.D., and Dr. G. Suckley, U.S.A., naturalists to the expedition. This edition contains a new preface, giving a sketch of the explorations, a classified table of contents, and the latest additions by the authors. With fifty-five new plates of scenery, botany, and zoology, and an isothermal chart of the route.*” [GB] and in 1860 by the same publisher in “*The natural history of Washington Territory and Oregon, with much relating to Minnesota, Nebraska, Kansas, Utah, and California, between the thirty-sixth and forty-ninth parallels of latitude, being those parts of the final reports on the survey of the Northern Pacific railroad route, relating to the natural history of the regions explored, with full catalogues and descriptions of the plants and animals collected from 1853 to 1860 by Geo. Suckley, M.D., and J.G. Cooper, M.D., naturalists to the late N. P. Railroad exploration. With the co-operation of Messrs. Baird, Girard, Stimpson, Geo. Gibbs, Kennicott, Torrey, Gray, Cassin, and Lawrence. Insects by Dr. John LeConte. Mollusca by Wm. Cooper, Esq. This edition contains a new preface, giving a sketch of the explorations, a classified table of contents, and the latest additions and corrections by the authors. With sixty-six plates of scenery, botany, and zoology.*” [GB]. These two editions contains an appendix (pp. 72a–d), missing in the Senate and House of Representatives editions as well as in the 1857 version, containing a list of species added to the fauna since the publication of the original report in 1857 as well as a new version of tables 1 and 2.

**1859**. *The complete writings of Thomas Say on the entomology of North America. Edited by John L. Le Conte, M.D. with a memoir of the author, by George Ord. In two volumes.* Baillière Brothers, New York. (8vo) CNC, GB

*Vol. I.* xxiv + 412 pp. + 54 pls. [DP: 1859 (title page; preface dated 1 May 1859), 12 November 1859 (*Amer Publ Circ*), 13 December 1859 (*Acad Nat Sci Phila*), 1 January 1860 (*Ohio Cult*)].

*Vol. II.* iv + 814 pp. [DP: 1859 (title page), 12 November 1859 (*Amer Publ Circ*), 13 December 1859 (*Acad Nat Sci Phila*), 1 January 1860 (*Ohio Cult*)].

This is essentially a collection of Thomas Say entomological publications, missing a few articles published in New Harmony, Indiana. Notes have been added by LeConte throughout the text and some of them are nomenclaturally significant. These volumes were reprinted in 1883 by J.E. Cassino, Boston, in 1891 by A.E. Foote, Philadelphia, and in 1978 by Arno Press, New York. Another printing, published in 1869, by J.W. Bouton, New York, has the title “*American entomology. A description of the insects of North America, by Thomas Say, with illustrations drawn and colored after nature. Edited by John L. Le Conte, M.D. with a memoir of the author, by George Ord.*” [CNC].

**1859**. *The Coleoptera of Kansas and eastern New Mexico.* Smithsonian Contributions to Knowledge [no 126]. The Smithsonian Institution, Washington City. vi + 58 pp. + 2 pls. (4to) [DP: December 1859 (title page)] CNC, GB

**1861–1873**. *Classification of the Coleoptera of North America. Prepared for the Smithsonian Institution.* Smithsonian Miscellaneous Collections [no 136] [Part I], [no] 265 [Part II]. The Smithsonian Institution, Washington [DC] (8vo) CNC

*Part I.* xxiv + pp. 1–214 + 209–286. This volume was issued in two sections: **1**: (pp. xxiv + 1–214) May 1861 (title page), 12 August 1861 (*Ent Soc Phila*); **2**: (pp. 209–286) March 1862 (last page). Pages 209–214 of the first section form the index. The second section came with a revised version of the first section. Subsequent printings were issued with minor changes in the text.

*Part II.* Pp. 279–348. [DP: May–June 1873 (title page)]. The first page of each sheet is dated May 1873.

**1863**. *List of the Coleoptera of North America. Prepared for the Smithsonian Institution. Part I.* Smithsonian Miscellaneous Collections [no] 140. The Smithsonian Institution, Washington [DC]. [3] + 56 pp. (8vo) [DP: March 1863 (title page; advertisement at the beginning of the work dated 2 March 1863)] CNC, GB

Page 50 is entitled “Additions” and includes the description of two carabid species (*Cychrus guyotii* and *Dyschirius obesus*); pages 51–56 form the “Index.” This work was reissued, with minor changes, and continued in 1866 [*q.v.*].

**1863–1866**. *New species of North American Coleoptera. Prepared for the Smithsonian Institution.* Smithsonian Miscellaneous Collections [no] 167 [Part I], 264 [Part II]. The Smithsonian Institution, Washington [DC] (8vo) CNC

*Part I.* [1] + pp. 1–92 + 87–177. This volume was issued in four sections: **1**: (pp. [1] + 1–92) March 1863 (title page); **2**: (pp. 87–128) December 1865 (bottom of pp. 87, 97 and 113); **3**: (pp. 129–160) March 1866 (bottom of pp. 129 and 145); **4**: (pp. 161–177) April 1866 (bottom of p. 161). Pages 87–92 of the first section and 169–177 of the fourth section are indexes.

*Part II.* Pp. 169–240. This volume was issued in two sections: **1**: (pp. 169–184) May 1873 (bottom of p. 169); **2**: (pp. 185–240) June 1873 (bottom of pp. 185, 201, 217, and 233). The title page is dated May–June 1873. This part has no index.

**1866**. *List of the Coleoptera of North America. Prepared for the Smithsonian Institution. Part I.* Smithsonian Miscellaneous Collections [no] 140. The Smithsonian Institution, Washington [DC] [3] + 78 pp. (8vo) [DP: April 1866 (title page), 4 June 1866 (*Ent Soc Lond*)] CNC

Pages 1–49 were reprinted with minor changes from the 1863 edition [*q.v.*]. Part II was never published.

**1876**. Appendix H 10. New species of Coleoptera, collected by the expeditions for geographical surveys west of one hundredth meridian, in charge of Lieut. Geo. M. Wheeler, United States engineers. Pp. 516–520 *in*: *Annual Report of the Chief of Engineers to the Secretary of War for the year 1876. In three parts. Part III.* Government Printing Office, Washington [DC]. 755 + [6 (Index)] pp. (4to) [DP: 1876 (title page; page 126 dated 9 October 1876), 21 March 1877 (*J Frank Inst* 103: 219)] GB

This book contains part of the appendices to the Report of the Chief of Engineers. They were also issued the same year under the title “*Report of the Secretary of War; being part of the message and documents communicated to the two Houses of Congress at the beginning of the second session of the forty-fourth Congress. Volume II. Part III.*” [GB].

The reprint of LeConte’s contribution has an extra page (errata) and its title reads “*Report upon new species of Coleoptera collected by the expeditions for geographical surveys west of the 100th meridian, Lieut. Geo. M. Wheeler, Corps of Engineers, U.S. Army, in charge; being extract from Appendix JJ of the Annual Report of the Chief of Engineers for 1876.*”

**1883**. [LeConte, J.L. and Horn, G.H.] *Classification of the Coleoptera of North America. Prepared for the Smithsonian Institution.* Smithsonian Miscellaneous Collection [no] 507. The Smithsonian Institution, Washington [DC]. xxxviii + 567 pp. (8vo) [DP: 1883 (title page; advertisement dated February 1883), March 1883 (Scudder 1884: xiv), 13 April 1883 (*Amer Ent Soc*), 9 May 1883 (*Soc Ent Fr*), May 1883 (*Brook Ent Soc* [*Bulletin* 6: 11]), January 1884 (*Nat Nov*)] CNC, GB, BHL

#### Leder, Hans (Javornik, Czech Republic: 4 February 1843 – 19 May 1921: Katharein near Opava, Czech Republic). Austrian (Silesian-born) explorer, naturalist and ethnologist; explored Algeria (1867–1872), Caucasus several times (1875–1877, 1882–1888) and Mongolia (1891); his Coleoptera collection was scattered. References. Hetschko (1922); Manndorff (1985).

**1886**. Die Coleopteren des Talysch-Gebietes. Nach den neuesten Materialien bearbeitet von E. Reitter, Dr. Eppelsheim, A. Chevrolat, L. Ganglbauer und Dr. G. Kraatz, zusammengestellt von Hans Leder. Pp. 89–235 *in*: *Die Fauna und Flora des südwestlichen Caspi-Gebietes. Wissenschaftliche Beiträge zu den Reisen an der persisch -russischen Grenze. Von Dr. Gustav Radde. Unter mitwirkung von Dr. O. Böttger, E. Reitter, Dr. Eppelsheim, A. Chevrolat, L. Ganglbauer, Dr. G. Kraatz, Hans Leder, Hugo Christoph und Dr. G. von Horvath. Mit 3 Tafeln.* F.A. Brockhaus, Leipzig. viii + [1] + 425 pp. + 3 pls. (8vo) [DP: 1886 (title page; *Vorwort* dated January 1886), 29 May 1886 (*Lit Centr Deutsch*), June 1886 (*Nat Nov*; *Allg Bibl*), January–June 1886 (*Bibliot Hist-Nat*), 7 July 1886 (*Zool-Bot Gesell Wien*)] MCZ, GB

The section on Coleoptera comprises a list of many hundred species prepared by Hans Leder, followed by descriptions of new taxa by Reitter, Eppelsheim, Chevrolat, Ganglbauer, and Kraatz. However, most genera and species indicated as new were previously published in various journals.

#### Lefèvre, Édouard (Chartres, Eure-et-Loir, France: 1839 – 17 June 1894: Paris, France). French civil servant and entomologist who specialized in Coleoptera; worked at the minister of civil engineering. References. Fairmaire (1895, P); Lhoste (1987: 78–79); Gouillard (2004: 49).

**1885**. *Exploration scientifique de la Tunisie. Liste des coléoptères recueillis en Tunisie en 1883 par M. E. Letourneaux, membre de la Mission de l’exploration scientifique de la Tunisie, dressée par M. Ed. Lefèvre, avec le concours de MM. L. Fairmaire, de Marseul et D^r^ Sénac.* Imprimerie Nationale, Paris. 16 pp. (8vo) [DP: 1885 (title page), 12 September 1885 (*Bibl Fr*), 13 October 1885 (*Soc Zool Fr*), October 1885 (*Nat Nov*), 5 November 1885 (*Soc Amis Sci Nat Rouen*), 6 November 1885 (*Soc Géog Fr*), 14 November 1885 (*Soc Linn Nord Fr*), July–December 1885 (*Bibliot Hist-Nat*)] GB, BHL, MCZ

Several species are described in this opuscule but all were made available previously in journals.

#### Lehmann, Johann Christian (Schöningen, Lower Saxony, Germany: 3 January 1768 – ?). German apothecary; owned a pharmacy in Bad Lauterberg im Harz. References. Wagenitz (1988: 108); Heerde (2006: 381–382).

**1796**. *Dissertatio inavgvralis medica exhibens catalogvm insectorvm Coleopterorvm medicatorvm. Qvam consentiente illvstri medicorvm ordine in Academia Georgia Avgvsta pro svmmis in arte salvtari honoribvs capessendis die XIV. Ivl. MDCCXCVI, pvblico examini exponit Ioannes Christianvs Lehmann Schoeningensi - Brvnsvicensis.* Barmeier, Goettingae. 32 pp. (4to) [DP: 14 July 1796 (title page)] GDZ

#### Leimbach, Anton Ludwig Gotthelf (Treysa [currently part of Schwalmstadt], Hesse, Germany: 4 January 1848 – 11 June 1902: Arnstadt, Thuringia, Germany). German educator and botanist; professor at secondary schools in Elberfeld, Krefeld, Wattenscheid, Sondershausen and Arnstadt; his insect collection is at the Deutsches Entomologisches Institut in Müncheberg. References. Reineck (1902); Vollmann (1902).

**1886**. Über die Cerambyciden des Harzes. Pp. 1–16 *in*: *Zu der öffentlichen Prüfung des Fürstlich Schwarzburgischen Gymnasiums zu Sondershausen, welche am 12. April 1886 stattfinden wird, ladet ehrerbietigst ein der Direktor Dr. W. Kiefer, Geh. Schulrat, Inhaber des Fürstl. Schwarzb. Ehrenkreuzes II. Klasse.* Sondershausen. 26 pp. (4to) [DP: 12 April 1886 (title page)] ULBD

This work treats the Cerambycidae of the Harz, a mountain range in northern Germany extending across part of Lower Saxony, Saxony-Anhalt and Thuringia. It was also issued separately the same year in Sondershausen under the title “*Die Cerambyciden des Harzes. Ein kleiner Beitrag zur geographischen Verbreitung des Käfer*” (*n.v.*); it was noticed on May 1886 (*Nat Nov*). No new taxa are proposed in this work.

#### Lentz, Friedrich Leonhard (Königsberg [currently Kaliningrad], Russia]: 23 January 1813 – 10 September 1887: Königsberg, Russia). Prussian naturalist, classical scholar and high school teacher in Königsberg; his collection of Prussian Coleoptera is at the Zoologisches Museum in Königsberg. References. Netolitzky and Vogel (1917: 56); Anonymous (2013b).

**1853**. *Preussische Käfer für die sammelnde Jugend. Mit 22 Abbildungen.* Gräfe und Unzer, Königsberg. 50 + [1] pp. + 3 pls. (8vo) [DP: 1853 (title page), 5 May 1853 (*Allg Bibl Deutsch*), January–June 1853 (*Bibliot Hist-Nat*; *Verz Bücher Landkarten*), September 1853 (*Ent Ztg* 14: 325)] Online

This work consits of descriptions of 267 species of Coleoptera found in Prussia.

**1857**. *Neues Verzeichniss der preussischen Käfer.* E.J. Dalkowski, Königsberg. 170 pp. (8vo) [DP: 1857 (title page)] MCZ, GB

Part of this work was also issued in *Der neue Preussischen Provinzial-Blätter* 11: 45–64, 124–138, 248–273 [DP: 1857 (title page)]; 12: 27–43, 109–126, 165–174 [DP: 1857 (title page)]. A supplement to this work was published in 1861 in *Schriften der Königlichen Physikalisch-Ökonomischen Gesellschaft zu Königsberg* 1 [1860]: 139–146.

This work is a catalogue of the Prussian Coleoptera; annotations are provided for some of the species.

**1879**. *Catalog der preussischen Käfer neu bearbeitet.* W. Koch, Königsberg. ii + 64 pp. (4to) [DP: 1879 (title page), 3 October 1879 (*Phys-Ökon Gesell König*), October 1879 (*Nat Nov*), 12 January 1880 (*Zool Anz*)] GB, BHL

This publication forms no. 4 of *Beiträge zur Naturkunde Preussens herausgegeben von der Physikalisch-Ökonomischen Gesellschaft zu Königsberg*.

#### Lenz, Harald Othmar (Schnepfenthal near Waltershausen, Thuringia, Germany: 27 February 1799 – 13 January 1870: Schnepfenthal, Germany). German naturalist and classical philologist; from 1824, professor at a reformatory in Schnepfenthal. References. Ratzeburg (1874: 305–307); Burbach (1884).

**1836**. *Gemeinnützige Naturgeschichte. Dritter Band: Amphibien. Fische. Weichthiere. Kerbthiere. Pflanzenthiere. Mit sechs Tafeln Abbildungen.* Becker, Gotha. viii + 530 pp. + 6 pls. (8vo) [DP: 1836 (title page; *Vorrede* dated June 1836), 19 August 1836 (*Allg Bibl Deutsch*), 24 August 1836 (*Lit Ztg*), July–December 1836 (*Verz Bücher Landkarten*)] GB

The entire work was published in four *Bande*, 1835–1839. The Coleoptera are on pages 181–276 of the third volume. Three subsequent editions were published, 1842–1846, 1851–1856, 1859–1868.

#### Lenz, Johann Georg (Schleusingen, Thuringia, Germany: 2 April 1748 – 28 February 1832: Jena, Thuringia, Germany). German mineralogist; professor of mineralogy at the University of Jena. References. Schwabe (1834); Günther (1858: 217–218); Gümbel (1883).

[**1783**]. *Anfangsgründe der Thiergeschichte zum Gebrauch akademischer Vorlesungen. Mit Kupfern.* Joh. Rudolph Cröcker, Jena. [2] + 479 + [8] + 7 pls. (8vo) [DP: 1783 (title page), 7 April 1783 (*Jena Ztg*)] GB, SBB

Meusel (1797: 410) stated that this work was actually published in 1782. The Coleoptera are on pages 320–361. The first part of the work ([5] + 93 + [2] pp. + 2 pls) was also issued in 1782 under the title “*Anfangsgründe der Naturgeschichte*” by the same publisher and recorded on 15 July 1782 (*Jena Ztg*).

#### Lepekhin [Lepechin, Lepyokhin, Lepjochin], Ivan [Johann] Ivanovich (Saint Petersburg, Russia: 21 September 1740 – 18 April 1802: Saint Petersburg, Russia). Russian naturalist and explorer; led an expedition organized by the Academy of Saint Petersburg to investigate the Volga region, the Urals and northern European Russia (1768–1772); in 1773 led an nine-month expedition to Byelorussia; director of the botanical garden in Saint Petersburg (1773–1802); permanent secretary of the Russian Academy (1783–1802). References. Anonymous (1806: 8–10); Lukina (1965); Anonymous (2015e, P).

**1774–1783**. *Tagebuch der Reise durch verschiedene Provinzen des russischen Reiches in den Jahren 1768 und 1769. Aus dem russischen übersetzt von M. Christian Heinrich Hase.* Richter, Altenburg. (4to) MCZ, GB

*Erster Theil.* [9] + 331 + [1] pp. + 23 pls. [DP: 1774 (title page; *Vorbericht* dated 9 August 1774), December 1774 (*Teutsch Merkur*), 23 January 1775 (*Jena Ztg*), 25 February 1775 (*Gött Anz*)]. The dates of 3 April 1774 and April 1774 found by Evenhuis (1997b: 451) are unlikely considering the date of the preliminaries. Beckmann (1774: 538) and Hagen (1862: 470) reported that this *Theil* has 16 plates; this is due to the fact that some plates are combined on a single leaf.

*Zweyter Theil*. [2] + 211 pp. + 11 pls. [DP: 1775 (title page), 30 April 1775 (Evenhuis 1997b: 451), 16 October 1775 (*Jena Ztg*), 19 December 1775 (*Erlang Anmer Nachr*)]. In this volume the first part of the title is changed to “*Tagebuch der Reise durch verschiedene Provinzen des russischen Reiches im Jahr 1770.*”

*Dritter Theil*. 234 pp. + 17 pls. [DP: 1783 (title page), 29 May 1783 (*Gött Anz*)]. In this volume the first part of the title is changed to “*Tagebuch der Reise durch verschiedene Provinzen des russischen Reiches im Jahr 1771.*” Hagen (1862: 470) noted that this *Theil* has nine plates but Beckmann (1784: 40) reported 17 plates which is the number of plates in the copies I have seen. Stuck (1784: 174) and Müller (1784: 582) reported the publication date of this volume as 1782.

This is a German translation of a work by Lepekhin published in Russian in three volumes, 1770, 1772 and 1780 (*n.v.*). Several species of Coleoptera are described and illustrated, but not named, in the first and second volumes.

#### Leske, Nathanael Gotfried (Bad Muskau, Saxony, Germany: 22 October 1757 – 25 November 1786: Marburg, Hesse, Germany). German naturalist and geologist; professor of natural history at the University of Leipzig; later, professor of natural history at the University of Marburg; his collection is at the Natural History Museum in Dublin. References. Löper (1787); Karsten (1789); Schrader and Hering (1863: 249); Andert and Prescher (1977); Otto et al. (2002).

**1779**. *Anfangsgründe der Naturgeschichte. Erster Teil. Algemeine Natur- und Tiergeschichte mit 10 Kupfertafeln.* Siegfried Lebrecht Crusius, Leipzig. xxxviii + 560 + [41] pp. + 10 pls. (8vo) [DP: 1779 (title page; *Vorrede* dated 22 October 1779), 12 February 1780 (*Zug Gött Anz*), 18 September 1780 (*Jena Ztg*)] GB, GDZ

Only the first part was published. The Coleoptera are on pages 415–435. A second edition, with xliv + 681 + [2] pages and 12 plates, was published in Leipzig, 1784 [CAKL, GB]. Another edition, containing [2] + 524 + [13] pages and six plates, was issued in 1788 in Vienna under the title “*Anfangsgründe der Naturgeschichte des Thierreiches. Abgekürzte, zum Leitfaden für Vorlesungen an der Universität zu Wien bestimmte Auflage*” [GB]. An Italian translation of the second edition with additional material was issued in Milan, 1785, in two volumes under the title “*Elementi di storia naturale di N.G. Leske professore di storia naturale a Lipsia, e membro di molte societa scientifiche, ed economiche. Tradotti dal tedesco, aumentati, e migliorati da Ermenegildo Pini*” [ANSP, GB]. A Russian translation was published in St. Petersbourg in 1791 under the title “*Nachalniia osnovaniia estestvennoi istorii: soderzhashchiia tsarstva zhivotnykh, proizrastienii, i izkopaemykh po sistematicheskomu Zhivotnykh raspolozheniiu G. Leske, na Niemetskom iazykie pisannomu; izdano Akademikom Nikolaem Ozereskovskim*” (*n.v.*).

**1785**. *Reise durch Sachsen in Rüksicht der Naturgeschichte und Ökonomie unternommen und beschrieben.* J.G. Müller, Leipzig. xxiv + 548 + [1] pp. + 39 pls. (4to) MCZ, GB

This work was issued in two *Hefte*: (pp. 1–262 + 20 pls) 8 July 1785 (*Jena Ztg*); (pp. 263–548 + xxiv + 19 pls) 6 December 1785 (*Allg Lit Ztg*), 27 January 1786 (*Jena Ztg*). The title page is dated 1785 and page [viii] 4 October 1785.

The following new species of Coleoptera, all collected at Königsbrük in Upper Lusatia, are described in this work: *Elater pilosus* (p. 11), *Silpha rufa* (p. 14), *Chrysomela rufitarsis* (p. 15), *Carabus lepidus* (p. 17) and *Scarabaeus recticornis* (p. 45).

#### Lesson, René Primevère (Rochefort, Charente-Maritime, France: 20 March 1794 – 28 April 1849: Rochefort, France). French surgeon and naturalist; served in the French Navy during the Napoleonic wars; pharmacist and botanist on Duperrey’s round-the-word voyage on *La Coquille* (1826–1829); chief pharmacist of the Navy at Rochefort. References. Lesson (1846: 53–66); Delaunay (1954); Huard and Wong (1965: 199–207); Adler (2007: 57, P).

**1830–1832**. *Centurie zoologique, ou choix d’animaux rares, nouveaux ou imparfaitement connus; enrichi de planches inédites, dessinées d’après nature par M. Prêtre, gravées et coloriées avec le plus grand soin.* F.G. Levrault, Paris. x + pp. 11–244 + 80 pls. (8vo) GB, BHL

This work was published in 16 livraisons. Sherborn (1922a: lxxx) gave the following dates of publication: **1–3**: (pp. 1–48 + pls 1–10) by November 1830; **4–12**: (pp. 49–192 + pls 11–64) by March 1831; **13–16**: (pp. 193–244 + pls 65–80) May 1832. Dickinson et al. (2011: 118) indicated that evidence from the *Bibliographie de la France* yields livraisons 1–2 in 1830, 3–8 in 1831 and 9–16 in 1832^71^. The title page is dated 1830, the dedication January 1830 and the post-scriptum (p. 229) February 1831. Lesson (1839: 104) commented that his genus *Megalonyx* (Aves), proposed in his *Centurie zoologique* on page 200, has the inscription of January 1831 because it was published in that month. Lesson’s statement is probably incorrect.

A single insect is illustrated and described, *Cetonia Dumerilii* Lesson on plate 13 (pp. 54–55).

**1832–1835**. *Illustrations de zoologie, ou recueil de figures d’animaux peintes d’après nature. Ouvrage orné de planches dessinées et gravées par les meilleurs artistes, et servant de complément aux traités généraux ou spéciaux publiés sur l’histoire naturelle et a les tenir au courant des nouvelles découvertes et des progrès de la science.* Arthus Bertrand, Paris. 60 pls + text. (8vo) CAKL, GB, BHL

This work was issued in 20 livraisons consisting each of three plates and corresponding unnumbered text. The following notices of publication of the livraisons were found (see also Mathews 1911: 12): **1**: 14 July 1832 (*Bibl Fr*), 3 December 1832 (*Acad Sci Fr*); **2**: 1 September 1832 (*Bibl Fr*), 28 January 1833 (*Acad Sci Fr*); **3**: July–September 1832 (*Foreign Quart Rev*), 13 October 1832 (*Bibl Fr*), 11 March 1833 (*Acad Sci Fr*); **4**: 3 November 1832 (*Bibl Fr*), 20 May 1833 (*Acad Sci Fr*); **5**: 1 December 1832 (*Bibl Fr*); **6**: 20 February 1833 (*Bibliologue*), 23 February 1833 (*Bibl Fr*), 16 September 1833 (*Acad Sci Fr*); **7**: 13 April 1833 (*Bibl Fr*); **8**: 10 August 1833 (*Bibl Fr*), 3 March 1834 (*Acad Sci Fr*); **9**: 24 August 1833 (*Bibl Fr*), 21 July 1834 (*Acad Sci Fr*); **10**: 19 October 1833 (*Bibl Fr*), 11 August 1834 (*Acad Sci Fr*); **11**: 21 December 1833 (*Bibl Fr*), 15 September 1834 (*Acad Sci Fr*); **12**: 22 March 1834 (*Bibl Fr*); **13**: 17 May 1834 (*Bibl Fr*), 6 October 1834 (*Acad Sci Fr*); **14**: 2 August 1834 (*Bibl Fr*), 20 October 1834 (*Acad Sci Fr*); **15**: 24 November 1834 (*Acad Sci Fr*), 17 January 1835 (*Bibl Fr*); **16**: 1835; **17**: 1835; **18**: 21 December 1835 (*Acad Sci Fr*); **19**: 1835; **20**: 21 December 1835 (*Acad Sci Fr*). There is no date on the title page and the *préface* is dated November 1831. Hagen (1862: 472) dated this work 1831–1835.

Only two beetles are illustrated, *Tetropthalma chiloensis* Lesson on plate 24 [+ 4 pp.] issued in 1833 (livraison 8) and *Sagra buquetii* Lesson on plate 30 [+ 3 pp.] issued also in 1833 (livraison 10).

As seen by the entries in the *Bibliographie de la France*, the title of this work on the covers of the livraisons was “*Illustrations de zoologie, ou choix de figures, peintes d’après nature, des espèces inédites et rares d’animaux récemment découverts, et accompagnées d’un texte descriptif général et particulier.*”

#### Leunis, Johannes (Mahlerten, Lower Saxony, Germany: 2 June 1802 – 30 April 1873: Hildesheim, Lower Saxony, Germany). German clergyman and naturalist; professor of natural history at the Collegium Josephinum in Hildesheim (1824–1873) and from 1844 also vicar of the city. References. Keese (1873); Wunschmann (1883); Mägdefrau (1985).

**1844**. *Synopsis der drei Naturreiche. Ein Handbuch für höhere Lehranstalten und für Alle, welche sich wissenschaftlich mit Naturgeschichte beschäftigen wollen. Mit vorzüglicher Berücksichtigung der nützlichen und schädlichen Naturkörper Deutschlands, so wie der zweckmässigsten Erleichterungsmittel zum Selbstbestimmen. Erster Theil. Zoologie.* Hahn, Hannover. xxxi + [1] + 476 + [1] pp. (8vo) [DP: 1844 (title page; *Vorrede* dated 15 October 1843), 4 April 1844 (*Allg Bibl Deutsch*), 17 April 1844 (*Lit Ztg*), 23 April 1844 (*Ent Ver Stettin*), April 1844 (*Intell Allg Lit Ztg*), 3 May 1844 (*Leip Reper*), 5 May 1844 (*Neue Würz Ztg*), 1 June 1844 (*Allg Schul Ztg*), January–June 1844 (*Verz Bücher Landkarten*)] MCZ

The Coleoptera are on pages 188–237. A second edition was published in 1860 [*q.v.*].

**1860**. *Synopsis der drei Naturreiche. Ein Handbuch für höhere Lehranstalten und für Alle, welche sich wissenschaftlich mit Naturgeschichte beschäftigen und sich zugleich auf die zweckmä*β*igste Weise das Selbstbestimmen der Naturkörper erleichtern wollen. Mit vorzüglicher Berücksichtigung der nützlichen und schädlichen Naturkörper Deutschlands, so wie der wichtigsten vorweltlichen Thiere und Pflanzen. Zweite, gänzlich umgearbeitete, mit 2000 Holzschnitten und mit der etymologischen Erklärung sämmtlicher Namen vermehrte Auflage. Erster Theil. Zoologie. Mit nahe an 1000 Abbildungen auf 702 Holzstöcken.* Hahn, Hannover. lxvi + 1014 pp. (8vo) [DP: 1860 (title page), 14 April 1860 (*Gött Anz*), June 1860 (*Neuer Anz Bibl*), January–June 1860 (*Bibliot Hist-Nat*)] GB

The entire series was published in three volumes, 1860–1878. The Coleoptera are in the first volume (pp. 433–518). The alternative title reads “*Synopsis der Naturgeschichte des Thierreichs ... Zweite, gänzlich umgearbeitete, mit Holzschnitten (nahe an 1000 Abbildungen auf 702 Holzstöcken) und mit der etymologischen Erklärung sämmtlicher Namen vermehrte Auflage.*” The alternative title page has Leunis as sole author while the first title page has Leunis and Friedrich Adolph Roemer [1809–1869]. A third edition, authored by Hubert Ludwig [1852–1913], was published in Hannover in two volumes, 1883–1886, under the title “*Dr. Johannes Leunis Synopsis der drei Naturreiche. Ein Handbuch für höhere Lehranstalten und für Alle … Erster Theil. Zoologie. Dritte, gänzlich umgearbeitete, mit vielen hundert Holzschnitten vermehrte Auflage*” [GB]; in this edition the Coleoptera are on pages 56–206 of the second volume. The first edition of the work was published in 1844 [*q.v.*].

#### Levrat, Jean Nicolas Berthélemy Gustave (Lyon, Rhône, France: 16 January 1823 – 28 August 1859: Lyon, France). French businessman (velvet factory) and amateur coleopterist; the whereabouts of his collection are unknown except for the cerambycids which went to the Muséum National d’Histoire Naturelle in Paris via Maurice Pic. References. Mulsant (1860b, P); Lhoste (1987: 76–77).

**1859**. *Études entomologiques. Premier cahier.* J. Nigon, Lyon. 99 + [2] pp. (8vo) [DP: 1859 (title page), 14 July 1859 (*Soc Stat Mars*)] ARC, MANL

This work is divided into 15 sections: 1. De l’utilité de la science entomologique (pp. 1–8); 2. Souvenirs du Mont Pilat (pp. 9–18); 3. Description d’une espèce nouvelle du genre *Pimelia* (pp. 19–20); 4. Strophes (pp. 21–24); 5. Description de trois coléoptères nouveaux (pp. 25–28); 6. Description d’une espèce nouvelle du genre *Poecilus* (pp. 29–31); 7. Description de quelques coléoptères nouveaux des environs de Tunis (pp. 33–36); 8. Description d’un longicorne nouveau du genre *Purpuricenus* (pp. 37–38); 9. Description d’un buprestide nouveau (pp. 39–40); 10. Description d’une espèce nouvelle du genre *Pimelia* (pp. 41–42); 11. Description d’un carabique nouveau (pp. 43, 46); 12. Note pour servir a l’histoire du *Dryops femorata* F. Scht. (pp. 47, 54–55); 13. Causes de détérioration chez les coléoptères (pp. 57–60); 14. Emploi de l’éther comme moyen de dissolution de l’oléine transsudante chez les coléoptères (pp. 61–64); 15. Énumération des insectes coléoptères du Mont-Pilat (pp. 65–99 + [1]). Sections 3, 5, 6, 8, 9 and 13 were first published in various volumes of the *Annales de la Société Linnéenne de Lyon* between 1847 and 1858.

Only the first *cahier* of this work was published.

#### Lewis, George (London, United Kingdom: 5 August 1839 – 5 September 1926: Folkestone, Kent, United Kingdom). British entomologist; worked for a firm engaged in the tea trade; made prolonged stays in China and Japan; his collection is at the Natural History Museum in London. Reference. Edwards (1927).

**1879**. *A catalogue of Coleoptera from the Japanese Archipelago.* Taylor and Francis, London. 31 pp. (8vo) [DP: 1879 (title page), October 1879 (*Nat Nov*), November 1879 (*Ent Monthly Mag* 16: 137), 12 January 1880 (*Zool Anz*)] MCZ, GB

**1888**. Fam. Histeridae. Pp. 182–244 + pls. 4–8 *in*: *Biologia Centrali-Americana. Insecta. Coleoptera. Vol. II. Part 1.* Taylor and Francis, London. xii + 717 pp. + 19 pls. (4to) CNC

This work was published in four parts in 1888. The dates of publication are indicated at the bottom of the first page of each sheet: (pp. 182–208) June 1888; (pp. 209–232 + pl. 5) October 1888; (pp. 233–240 + pl. 6) November 1888; (pp. 241–244) December 1888. The collation for the plates is from Lyal (2011: 81).

#### Lichtenstein, Anton August Heinrich (Helmstedt, Lower Saxony, Germany: 25 August 1753 – 17 February 1816: Helmstedt, Germany). German clergyman and naturalist; assistant director and later director of the Academic school of the Johanneum in Hamburg (1777–1799); general superintendent and professor of theology at the University of Helmstedt (1799–1810). References. Schröder et al. (1866: 471–476); M’Clintock and Strong (1887: 668); Volkmann (1996).

**1796**. *Catalogus musei zoologici ditissimi Hamburgi, d III Februar 1796. Auctionis lege distrahendi. Sectio tertia continens Insecta. Verzeichniss von höchstseltenen, aus allen Welttheilen mit vieler Mühe und Kosten zusammen gebrachten, auch aus unterschiedlichen Cabinettern, Sammlungen und Auctionen ausgehobenen Naturalien welche von einem Liebhaber, als Mitglied der batavischen und verschiedener anderer naturforschenden Gesellschaften gesammelt worden. Dritter Abschnitt, bestehend in wohlerhaltenen, mehrentheils ausländischen und höchstseltenen Insecten, die Theils einzeln, Theils mehrere zusammen in Schachteln festgesteckt sind, und welche am Mittewochen, den 3^ten^ Februar 1796 und den folgenden Tagen auf dem Eimbeckschen Hause öffentlich verkauft werden sollen durch dem Mackler Peter Heinrich Packischefsky.* G.F. Schniebes, Hamburg. xii + 222 + [2] pp. (8vo) [DP: 3 February 1796 (*cover*)] (*n.v.*).

This is the third section of a catalogue of specimens sold at auction. A reprint of this catalogue, under the title “*Catalogus musei zoologici ditissimi Hamburgi, d. 16 Majus 1797*,” was issued one year later with the “sold” items struck out (Sherborn 1899a: 272). In Opinion 1820, the Commission (ICZN 1995) suppressed this publication and its reprint of 1797 for nomenclatural purposes but nevertheless retained 19 specific taxa proposed in the 1796 catalogue as available, including the following eight beetles: *Brachycerus caperans*, *Brachycerus junix*, *Brentus gnatho*, *Cassida caedemadens*, *Cassida ephippium*, *Cassida haematites*, *Cicindela nitida*, and *Sagra purpurea*.

This catalogue is extremely rare and only three copies are currently known (Burger and Thompson 1981: 356).

#### Linck, Johann Wilhelm (Leipzig, Saxony, Germany: 25 December 1760 – 25 December 1805: Leipzig, Germany). German practicing physician in Leipzig. References. Adelung and Rotermund (1810: 1848); Poggendorff (1863: 1464).

**1787**. *Dispvtatio inavgvralis de coccionellae natvra, viribvs et vsv. Qvam gratiosi ordinis medicorvm avctoritate pro gradv doctoris pvblicae disqvisitioni svbmittit Ioannes Gvilielmvs Linck Ph.D. et A.L.M. medicin. Bacc. Lipsiensis. Die VI. Febr. A.D. MDCCLXXXVII.* Sommer, Lipsiae [= Leipzig]. [5] + 31 pp. + 1 pl. (4to) [DP: 6 February 1787 (title page)] CAKL, DFG

The work includes the descriptions of 21 *Coccinella* species in footnotes on pages 8–11, all of them illustrated on the sole plate. The descriptions are linked to a Latin sentence which reads “Habemus igitur coccionellam *lividam* a), *trilineatam* b), *variabilem* c), *oblongam* d), *gemellam* e), *conglomeratam* e), *reppensem* f), *campestrem* g), *fasciatam* h), *tigrinam* i), *punctis duodecim* k), *quatuordecim* l), *sedecim* m), *novem decim* n), *viginti* o), *viginti quatuor* p), *et innumeris* q), …decem et innumeris r), quatuordecim s), quindecim t), viginti v) ornatas guttis.” These descriptors seem to refer to scientific names of species already known, i.e. *livida*, *trilineata*, *variabilis*, *oblonga*, *gemella*, *conglomerata*, *reppensis*, *campestris*, *fasciata*, *tigrina*, *duodecimpunctata*, *quatuordecimpunctata*, *sedecimpunctata*, *novemdecimpunctata*, *vigintipunctata*, *vigintiquatuorpunctata*, *quatuordecimguttata*, *quindecimguttata*, and *vigintiguttata*. Sherborn recorded three of Linck’s names in his *Index Animalium* (online version): *gemella*, *innumeris punctata*, and *oblonga*. According to Miguel A. Alonso-Zarazaga (personal communication) the descriptors are not scientific names and the pamphlet is doubtfully binominal.

#### Linell, Martin Larsson (Grönby, Kristianstad, Sweden: 24 June 1849 – 3 May 1897: Washington DC, USA). American (Swedish-born) entomologist; moved to America in 1879 and first found work in a chemical laboratory in Brooklyn, New York; later, curator in entomology at the U.S. National Museum (1888–1897). References. Chittenden (1897); Schwarz (1899).

**1899**. [Linell, M.L. and Schwarz, E.A.] Order Coleoptera. Pp. 328–335 *in*: *The fur seals and fur-seal islands of the North Pacific Ocean. By David Starr Jordan, commissioner in charge of fur-seal investigations of 1896-97. With the following official associates: Leonard Stejneger and Frederic A. Lucas, of the U.S. National Museum. Jefferson F. Moser in command of the U.S. Fish Commission steamer Albatross. Charles H. Townsend, of the U.S. Fish Commission. George A. Clark, secretary and stenographer. Joseph Murray, special agent. With special papers by other contributors. Part 4.* Governement Printing Office, Washington [DC]. 384 pp. + 86 pls. (8vo) [DP: 1898 (titled page), July 1899 (Allen 1899: 886), August 1899 (*Pop Sci Monthly*)] ANXL, GB

An additional title page reads “*Part IV. The Asiatic fur-seal islands and fur-seal industry. By Leonhard Stejneger, of the United States National Museum*.”

The entire work consists of four volumes, 1899. According to Allen (1899: 886), although all parts are dated 1898 on their title pages, parts 1, 2 and 4 were issued in July 1899 and part 3 in November 1899.

#### Linnaeus, Carl [Carl von Linné]^72^ (Raashult, Smaaland, Sweden: 23 May 1707 – 10 January 1778: Hammarby near Uppsala, Sweden). Swedish physician and naturalist; professor of medicine and botany at the University of Uppsala; established the binomial system of nomenclature. References. Stoever (1794, P); Fée (1832); Greenwood (1886: 1–33, P); Bolton (1889: 49–64, P); Miall (1912: 310–319); Adler (1989: 11–12, P).

**1758**. *Systema naturae per regna tria naturae, secundum classes, ordines, genera, species, cum characteribus, differentiis, synonymis, locis. Tomus I. Editio decima, reformata.* Laurentii Salvii, Holmiae [= Stockholm]. [iv] + 823 + [1, Emendanda, Addenda] pp. (8vo) [DP: 1758 (title page), 1 January 1758 (ICZN 1999: Article 3), 8 February 1758 (Sherborn 1899c: vi)] CNC, GB

The tenth edition of the *Systema Naturae* was issued in two volumes, 1758–1759. The second volume deals with plants; the third volume dealing with minerals was not published. The first volume was reprinted in Leipzig, 1894, by the Societatis Zoologicae Germanicae (*n.v.*). A facsimile of this edition was published by the British Museum (Natural History) in 1956. Three more editions of this work were published including the twelfth edition, authored by Linnaeus, 1766–1768 [*q.v.*], and the thirteenth by Gmelin, 1788–1792 [*q.v.*]. According to Woodward (1907: 7), the eleventh edition was issued in Lipsiae [= Leipzig] in 1762 and contains no additions. Sherborn (1899c) published an index to generic and specific names in this and 12th editions.

The first edition of this work was issued in 1735 in Leiden.

**1759**. *Animalium specierum in classes, ordines, genera, species methodica dispositio, additis characteribus, differentiis atque synonymis, accommodata ad Systema Naturae & in formam enchyridii redacta, secundum decimam Holmensem editionem.* Theodorum Haak, Lugduni Batavorum [= Leiden]. [2] + 253 + [3] pp. (8vo) [DP: 1759 (title page)] GB, BHL

The Coleoptera are on pages 109–136. Specific names are not given in this work; instead, numbers corresponding to the species numbers listed in the tenth edition of the *Systema Naturae* are provided.

[**1760**]. *Fauna Svecica sistens animalia Sveciae regni: mammalia, aves, amphibia, pisces, insecta, vermes. Distributa per classes & ordines, genera & species, cum differentiis specierum, synonymis auctorum, nominibus incolarum, locis natalium, descriptionibus insectorum. Editio altera, auctior.* Laurentii Salvii, Stockholmiae. [48] + 579 pp. + 2 pls. (8vo) [DP: 1761 (title page; dedication dated 28 July 1761), 14 November 1760 (inscription on Matthew Maty’s copy at the BMNH, see Soulsby 1933: 91)] CNC, GB

According to Evenhuis (1997b: 480), the title page and prefatory material (first 48, unnumbered pages) were probably added in 1761 to unbound copies available by the end of 1760. The first edition of this work was published in 1746 [GB].

**1762**. *I.N.J. Dissertatio medica, de Melöe-vesicatorio, quam cons. nobil. et exper. facult. med. in illustri athen. Upsaliensi, praeside viro nobilissimo et experientissimo, D:no Doct. Carolo Linnae. Publico examini modeste submittit Canutus Aug. Lenaeus, Jemtlandus. In aud. Car. maj. ad diem XXII. Dec. anni MDCCLXII. H.a.m.s.* Upsaliae [= Uppsala]. 15 pp. (8vo) [DP: 22 December 1762 (cover)] GB

This dissertation was also published in 1763 in “*Amoenitates academicae; seu dissertationes variae physicae, medicae, botanicae, antehac seorsim editae, nunc collectae et auctae cum tabulis aeneis. Volumen sextum*” (pp. 132–147) and also in volume 6 (pp. 132–147) of the Schreber’s edition of the “*Amoenitates academicae*” published in 1789 [GB].

The “*Amoenitates academicae*” [BHL] is a collection of 150 dissertations published in seven volumes, 1749–1769. The dissertations were written by Linnaeus and defended by his pupils. Several printings of this work were issued. A subsequent edition in ten volumes, 1785–1790, by J.C.D. Schreber in Erlangen [GB], contains the original dissertations (volumes 1–7) and 36 additional dissertations (volumes 8 and 9). The tenth volume contains orations by Linnaeus, two biographies of Linnaeus, and four dissertations of Carl von Linné, the younger (Soulsby 1933: 100). An account of the 186 dissertations is given by Millin de Grandmaison (1789a: 315–385; 1789b: 1–106).

**1763**. *D.D. Centuria insectorum rariorum quam consent. experimentiss. fac. med. In Regia Academia Upsaliensi, praeside nobilissimo atque celeberrimo D:o Doct. Carolo von Linné, equite aurat. de Stella Polari. Publico examini submittit Boas Johansson, Calmariensis. In audit Carol. maj. d. XXIII. Junii. anni MDCCLXIII. H.a.m.s.* Upsaliae [= Uppsala]. [3] + 32 pp. (8vo) [DP: 23 June 1763 (title page)] GB

This dissertation was also published the same year in “*Amoenitates academicae; seu dissertationes variae physicae, medicae, botanicae, antehac seorsim editae, nunc collectae et auctae cum tabulis aeneis. Volumen sextum*” (pp. 384–415) and also in 1789 in volume 6 (pp. 384–415) of Schreber’s edition of the “*Amoenitates academicae*” [GB].

**1764**. *Museum S:ae R:ae M:tis Ludovicae Ulricae Reginae Svecorum, Gothorum, Vandalorumque &c. &c. &c. In quo animalia rariora, exotica, imprimis insecta & conchilia describuntur & determinantur. Prodromi instar editum*. Laur. Salvii, Holmiae [= Stockholm]. [6] + 720 + [2 (Index)] pp. (8vo) [DP: 1764 (title page; *praefatio* dated 30 July 1764), 22 August 1765 (*Gött Anz*)] CAKL, GB, GDZ, BHL, GAL

The Coleoptera are on pages 2–104. This work is based on the collections formed by Louisa Ulrika of Prussia, Queen of Sweden between 1751 and 1771 by her marriage to King Adolf Frederick.

**1764**. *Reisen durch Oeland und Gothland, welche auf Befehl der hochlöblichen-Reichsstände des Königreichs Schweden im Jahr 1741. angestellt worden. Aus dem schwedischen übersetzt. Mit Kupfern.* Johann Jacob Curt, Halle. [29] + 364 + [22] pp. + 2 pls. (8vo) [DP: 1764 (title page), 11 June 1764 (*Neue Leip Ztg*)] GB, SDL

This book is a German translation of Linnaeus “*Öländska och Gothländska resa på riksens högloflige ständers befallning förrättad åhr 1741. Med anmärkningar uti oeconomien, natural-historien, antiquiteter &c. med åtskillige Figurer*” published in 1745 at Stockholm and Uppsala [GDZ].

Sherborn, in his *Index Animalium*, listed several species of insects being described for the first time in this translation, including one beetle, *Carabus niger* (p. 109). However, it seems that Linnaeus simply described the color of the *Carabus* and did not propose a specific name. This is supported by the fact that the species is listed in the index as “*Carabus niger*, elytris viridibus &c [p. 3 of the “zweytes Register”]” while the other species are listed with the generic and specific names only. In addition, *Carabus niger* is not included in Linnaeus’ 1758 *Systema Naturae* while all other beetle species described in the original edition of 1745 were also described in 1758.

**1767–1768**. *Systema naturae per regna tria naturae, secundum classes, ordines, genera, species, cum characteribus, differentiis, synonymis, locis. Editio duodecima, reformata.* Laurentii Salvii, Holmiae [= Stockholm]. (8vo) CNC, GB, BHL

*Tom. I. Pars II.* Pp. 533–1327 + [37 (Prayer, Index, Errata, Addenda, Synonymy)] pp. [DP: 1767 (title page), 14 June 1767 (Evenhuis 1997b: 482)].

*Tomus III.* 236 + [20 (Indexes, Errata)] pp. [DP: 1768 (title page), December 1768 (Stafleu and Cowan 1981: 107)]. The title for this volume is “*Systema naturae per regna tria naturae, secundum classes, ordines, genera, species, cum characteribus & differentiis.*”

The 12th edition of the *Systema Naturae* consists of three volumes, 1766–1768, with the first one published in two parts continuously paginated. The Coleoptera are in the first volume, second part (pp. 541–686), and in the “*Appendix Animalium*” (pp. 223–228) of the third volume. This edition was also issued in 1767–1770 by Joannis Thomae nob. de Trattnern in Vindobonae [= Vienna] as “*Editio decima tertia, ad editionem duodecimam reformatam Holmiensem*” [GB]. According to Evenhuis (1997b: 482) it is essentially a reprint of the 12th edition except that the errata section at the end of volume 3 is lacking.

**1767**. *D.D. Fundamenta entomologiae, quae venia nobiliss. fac. med. in illustr. ad salam swion. Athenaeo. Praeside. Illustri atque nobillissimo viro D:no Doct. Carolo von Linné. Publice defendere conabitur stipend. regius. Andreas Johann. Bladh. Ostrobotniensis. In audit. Carol. maj. die Junii MDCCLXVII.* Joh. Edman, Upsaliae [= Uppsala]. 34 pp. (4to) [DP: 14 June 1767 (title page of the 1769 issue)] CNC, GB

This work was also issued in 1789 in volume 7 (pp. 129–159) of Schreber’s edition of the “*Amoenitates academicae*.” For information regarding the “*Amoenitates academicae*” see under Linnaeus, 1763.

**1771**. *D.D. Pandora et flora Rybyensis, quam, dissertatione academica, consens. nobiliss. & exper. fac. med. Upsal. Praeside, viro nobilissimo, D.D. Carolo à Linné. Publicae ventilationi offert Daniel Henr. Söderberg, Junecopia-Smölandus. In auditor. Carol. maj. die XXVI. Junii, MDCCLXXI, horis anse meridiem solisis.* Joh. Edman, Upsaliae [= Uppsala]. [DP: 26 June 1771 (cover)] GB, BHL

This thesis gives a list of the insects and plants of Ryby, a village in the province of Södermanland, Sweden. The Coleoptera are on pages 7–10; of the 165 species of beetles listed, four are described in footnotes but all of them were described previously by Linné. This work was also published in 1785 in volume 8 (pp. 75–106) of Schreber’s edition of the “*Amoenitates academicae*“ [GB].

**1771**. *Mantissa plantarum altera generum editionis VI. & specierum editionis II.* Laurentii Salvii, Holmiae [= Stockholm]. [4] + pp. 143–587 + [1 (Addenda)]. (8vo) [DP: 1771 (title page; *praefatio* dated 1 September 1771), 17 October 1771 (Evenhuis 1997b: 482), 9 March 1772 (*Jena Ztg*), 21 December 1772 (*Gött Anz*)] CNC, GB

Although the book is mainly about plants, it includes a “*Regni animalis appendix*” (pp. 521–552) which contains descriptions of new animals, including 16 Coleoptera (pp. 529–533). The entire work was published in two parts, the first one (pp. 1–142) issued in 1767. A facsimile edition was published in 1961 by Cramer and Swann as tomus VII of their *Historiae naturalis classica*.

**1775**. *Dissertatio entomologica, bigas insectorum sistens, quam divinis auspiciis, conf. illustr. facult. med. Upsal. praeside, D:no Doct. Carolo à Linné. Publicae eruditorum disquisitioni modeste subjicit stipendiarius rhyzelianus Andreas Dahl, Westrogothus. In aud. Carol. major. d. XVIII Dec. MDCCLXXV. H.a.m.s.* Edmann, Upsaliae [= Uppsala]. iii + 7 pp. + 1 pl. (8vo) [DP: 18 December 1775 (title page)] USNM

This work was also issued in 1785 in volume 8 (pp. 303–309 + pl. 6) of Schreber’s edition of the “*Amoenitates Academicae*” [GB].

#### Łomnicki, Aloys [Alojzy] Maryan [Marjan, Maryjan] (Baworowie near Ternopil, Ukraine: 1 September 1845 – 25 September 1915: Lviv, Ukraine). Galician naturalist, paleontologist and geologist; professor at gymnasiums in Lviv and Ternopil (1869–1904); curator at the Dzieduszycki Museum in Lviv (1905–1915). References. Czyżewski (1988: 73–79, P); Maligranda (2008: 62).

**1884**. *Catalogus coleopterorum Haliciae.* L. Zontaki, Leopoli [= Lviv]. [2] + 43 pp. (8vo) [DP: 1884 (title page; preliminaries dated November 1883), February 1884 (*Nat Nov*; *Humboldt* 3: 156), March 1884 (*Allg Bibl*), January–June 1884 (*Bibliot Hist-Nat*), 10 July 1884 (*Wien Ent Ztg* 3: 191), 29 September 1884 (*Zool Anz* 7: 509)] Online

This is a catalogue of the Coleoptera of Galicia (= Halicz), an historic region of east central Europe, former Austrian crown land, today in Ukraine and Poland.

#### López Seoane, Víctor (El Ferrol, Galicia, Spain: 28 September 1832 – 11 June 1900: La Coruña, Galicia, Spain). Spanish physician, lawyer and naturalist; following his marriage in 1870 became manager of the numerous lands in Galicia owned by his wife. Reference. Dosil Mancilla (2007: 35–48, P).

[**1877**]. *Notas para la fáuna Gallega.* El Eco Ferrolano, Ferrol. 16 pp. (8vo) [DP: 1877 (title page; p. 5 dated 3 August 1877)] CSIC

The cover is dated 1878 but the title page 1877. One new species of Coleoptera, *Otiorhynchus cantabricus* (p. 11), is described.

#### Løvendal, Emil Adolf (Randers, Århus, Denmark: 14 July 1839 – 6 July 1901: Copenhagen, Denmark). Danish engraver and coleopterist; curator of the Westermann’s collection at the Zoological Museum in Copenhagen. References. Hansen (1902, P); Engelhart (1918: 20–21, P).

**1898**. *De Danske barkbiller (Scolytidae et Platypodidae Danicae) og deres betydning for skov- og havebruget. Med 89 i texten indtrykte afbildninger og 5 kobbertavler. Udgivet paa Carlsbergfondets bekostning.* Schubot, Kjøbenhavn [= Copenhagen]. xii + 212 pp. + 5 pls. (4to) [DP: 1898 (title page; *Forord* dated March 1898), June 1898 (*Nat Nov*; *Nat Sci*), 10 September 1898 (*Wien Ent Ztg* 17: 240)] CNC, GB, ARC

#### Lucas, Pierre Hippolyte (Paris, France: 18 January 1814 – 5 July 1899: Chêne-Bougeries, Swizerland). French invertebrate zoologist; entered the Muséum National d’Histoire Naturelle in Paris at the age of 13 as preparator and retired from the Muséum in 1892 as assistant naturalist; took part to a scientific mission in Algeria (1839–1842) and visited the country again in 1850 and 1872. References. Lesne (1901, P); Gouillard (2004: 63–64, P); Jaussaud (2004: 360–362); Cambefort (2006: 222–223).

**1846–1849**. *Exploration scientifique de l’Algérie pendant les années 1840, 1841, 1842 publiée par ordre du Gouvernement et avec le concours d’une Commission Académique. Sciences Physiques. Zoologie. II. Histoire naturelle des animaux articulés. Deuxième partie. Insectes.* A. Bertrand, Paris. 590 pp. [+ 47 associated pls]. (4to–text; Folio–plates) McG, MCZ, BHL

The entire *Exploration scientifique de l’Algérie* was published in 25 volumes, 1844–1867. The “*Zoologie*” section consists of five volumes, 1845–1867. The “*Histoire naturelle des animaux articulés*” subsection, authored by Lucas, was published in 33 livraisons, 1845–1849, bound in three volumes with an atlas containing 122 plates issued along with the livraisons. The Coleoptera form the entire volume 2 and are depicted on 47 plates. The text and plates relating to Coleoptera were issued in the following livraisons: **1–3**: (pp. 1–40 + pls 1–10, 14, 24, 26, 27) 28 March–15 August 1846; **4**: (pp. 41–80 + pls 15, 21, 22, 28, 34) 28 March–15 August 1846; **5**: (pp. 81–120 + pls 11, 20, 23, 31) 15 August 1846, 25 September 1846 (*Leip Reper*); **6**: (pp. 121–160 + pls 13, 19, 25, 33, 36) 15 August 1846, 25 September 1846 (*Leip Reper*); **7**: (pls 12, 16, 18) 15 August–November 1846; **8**: (pls 30, 35, 38, 46, 47) 15 August–November 1846; **9**: (pl. 40) 15 August–November 1846; **12**: (pls 42, 43) before 15 May 1847; **13**: (pl. 17) before 15 May 1847; **14**: (pp. 161–200 + pls 29, 37, 41, 45) before 15 May 1847; **15**: (pp. 201–240) before 15 May 1847; **16**: (pp. 241–280 + pl. 44) before 15 May 1847; **17**: (pp. 281–320) before 15 May 1847; **18**: (pp. 321–360) before 20 November 1847; **Supplément 2**: (pp. 361–536) 20 November 1847; **19**: (pl. 32) November 1847; **22**: (pls 39) 31 December 1848; **30**: (pp. 537–590) before 13 June 1849. The dates and collation are from Evenhuis (2012a) unless otherwise noted. The plates include scientific names for the species illustrated and some plates were issued before the text. Appendix 9 lists all the species depicted on the Coleoptera plates.

Based on the information available, I believe the following years should be accepted for the text: (pp. 1–160) 1846; (pp. 161–536) 1847; (pp. 537–590) 1849.

**1857–1859**. *Animaux nouveaux ou rares recueillis pendant l’expédition dans les parties centrales de l’Amérique du Sud, de Rio de Janeiro a Lima, et de Lima au Para; exécutée par ordre du gouvernement Français pendant les années 1843 a 1847, sous la direction du Comte Francis de Castelnau. Ouvrage qui a obtenu une médaille hors ligne de la Société de Géographie. Entomologie.* P. Bertrand, Paris. 204 pp. + 20 pls. (4to) MCZ, GB, BHL, EUR

This work is part of the “*Expédition dans les parties centrales de l’Amérique du Sud, de Rio de Janeiro a Lima, et de Lima au Para*,” under the direction of François Laporte, published in 17 sections forming 15 volumes, 1850–1859, divided into seven parts. The *Zoologie* section (seventh part) was published in 30 livraisons, 1855–1859, presented in seven segment headings, each separately paginated and with their own title and author and bound in three volumes. The *entomologie* segment is part of the third volume which, along with the Myriapodes and scorpions by P. Gervais and Mollusques by H. Huppé, forms livraisons 21–30 of the *Zoologie* section (Sherborn and Woodward 1901b: 164). These livraisons were noticed as follows: **21**: 2 May 1857 (*Bibl Fr*); **22–25**: 30 November 1857 (*Acad Sci Fr*); **26–28**: 7 March 1859 (*Acad Sci Fr*); **29–30**: 10 October 1859 (*Acad Sci Fr*). The title pages of the third volume and the entomology part are dated 1857. The Coleoptera are on pages 24–196.

Dating of Lucas’ contribution has been problematic since the collation has not been established. Sherborn and Woodward (1901b: 164) mentioned that livraisons 21–24 pertain to molluscs and that the entomology section was published in 1859. Gerstaecker (1859: 329) noted that the entomology portion could not have been published in 1857, the date on the title page, since it contains quotations of works published at the end of 1857. Some of the digitized copies seen (BHL, EUR) contain two unnumbered pages entitled “Table et classification des matières contenues dans le troisième volume” giving the collation for the entire third volume. Lucas’ contribution was issued in the following livraisons: **22**: title page; **23**: pls 1–2 [Crustacés] + pls 1–2 [Lépidoptères]; **25**: signatures 1–4 (pp. 1–32) + pl. 1 [Arachnides] + pls 1, 2–3, 7 [Coléoptères]; **26**: signatures 5–9 (pp. 33–72) + pls 4–6 [Coléoptères]; **27**: signatures 10–14 (pp. 73–112) + pls 8–9, 13–14 [Coléoptères]; **28**: signatures 15–20 (pp. 113–160) + pls 1a, 10–12 [Coléoptères]; **29**: signatures 21–26 (pp. 161–204).

#### Ludwig, Christian Friedrich (Leipzig, Saxony, Germany: 19 May 1757 – 8 July 1823: Leipzig, Germany). German physician and naturalist; professor at the University of Leipzig (1782–1823). References. Anonymous (1824b); Schmidt (1824).

**1799**. *Erste Aufzählung der bis jezt in Sachsen entdeckten Insekten. Im Namen der Linneischen Societät.* Christian Gottlieb Rabenhorst, Leipzig. viii + 66 pp. (8vo) [DP: 1799 (title page; preliminaries dated January 1799), 3 May 1799 (*Allg Lit Anz*)] GB

This is a first list of the insects discovered in Saxony; the Coleoptera are on pages 3–32.

#### Macleay, William Sharp (London, United Kingdom: 21 July 1792 – 26 January 1865: Sydney, Australia). British civil servant and zoologist; attaché to the British embassy in Paris for about ten years; appointed British commissioner of arbitration in Havana, Cuba in 1825 where he eventually became commissary judge and judge; retired in 1836 at the age of 44, he moved to Australia three years later; his collection of insects was bequeathed to his cousin William John Macleay who transferred it to the Macleay Museum, University of Sydney. References. King (1865: xliii–xlvi); MacMillan (1967); Swainston (1985); Zimmerman (1993: 663, P); Clark (2004a).

**1819–1821**. *Horae entomologicae: or essays on the annulose animals. Vol. I.* S. Bagster, London. xxx + [2] + 524 pp. + 3 pls. (8vo) CMLE, GB

*Part I. Containing general observations on the geography, manners, and natural affinities of the insects which compose the genus Scarabaeus of Linnaeus; to which are added a few incidental remarks on the genera Lucanus and Hister of the same author. With an appendix and plates.* xxx + [2] + 160 pp. + 3 pls. [DP: 1819 (title page), 1 November 1819 (*Monthly Mag*; *New Monthly Mag*), November 1819 (*Blackw Mag*)].

*Part II. Containing an attempt to ascertain the rank and situation which the celebrated Egyptian insect, Scarabaeus sacer, holds among organized beings.* [1] + pp. 161–524. [DP^73^: 1821 (title page)].

The work was published in two parts and is very rare because most of the stock was destroyed by fire (Junk 1935: 218). An abridged French version was published in Paris by Lequien Fils, 1833 (see comment for next entry).

**1825**. *Number I. of Annulosa Javanica, or an attempt to illustrate the natural affinities and analogies of the insects collected in Java by Thomas Horsfield, M.D.F.L. & G.S. and deposited by him in the museum of the honourable East-India Company.* Kingsbury, Parbury, and Allen, London. xii + 50 pp. + 1 pl. (4to) [DP: 1825 (title page), 1 June 1825 (*New Monthly Mag*), 30 June 1825 (*Phil Mag*), July 1825 (*Orient Herald*; Evenhuis 1997b: 507), September 1825 (*Not Geb Natur*)] CNC, GB, BHL

A French translation of 163 pages and five plates was published at 100 copies by Lequien Fils, Paris, in 1833, as part of *Bibliothèque entomologique*, under the title “*Annulosa Javanica, ou description des insectes de Java, par M. W. S. MacLeay, précédés d’un extrait des Horae entomologicae du même auteur*” along with an appendix of 96 pages containing an abridged French version of Macleay’s *Horae entomologicae*.

**1826**. Annulosa. Catalogue of insects, collected by Captain King, R.N. Pp. 438–469 + pl. B *in*: *Narrative of a survey of the intertropical and western coasts of Australia. Performed between the years 1818 and 1822 by Captain Phillip P. King. With an appendix, containing various subjects relating to hydrography and natural history. In two volumes, illustrated by plates, charts, and wood-cuts. Vol. II.* John Murray, London. vii + [1 (List of plates)] + 637 pp. + 7 pls. (8vo) [DP: 1826 (title page), 15 April 1826 (Sherborn 1922a: lxxiii; Common and Moulds 1973), May 1826 (*Dublin Phil J*)] (*n.v.*).

The entire work consists of two volumes issued simultaneously. A second printing was issued in January 1827 [CAKL, GB]. The first printing, 1826, is extremely rare. Zimmerman (1993: 664–667) discussed in detail the 1826 print and argued that “the restricted 1826 issue of King’s “Narrative” was not freely available when issued in 1826. Hence, for the purposes of priority the 1827 date of issue should be accepted.” A facsimile was printed in Adelaide, 1969 [OLIL].

**1838**. *Illustrations of the Annulosa of South Africa; being a portion of the objects of natural history chiefly collected during an expedition into the interior of South Africa, under the direction of Dr. Andrew Smith, in the years 1834, 1835, and 1836; fitted out by “The Cape of Good Hope association for exploring central Africa.” Published under the authority of the Lords Commissioners of her Majesty’s treasury.* Smith, Elder & Co., London. 75 pp. + 4 pls. (4to) [DP: 1838 (title page), 8 September 1838 (*Lit Gaz*; Low and Evenhuis 2014: 484), 24 October 1838 (*Lit Ztg*)] BHL

This publication forms volume 5 and last of “*Illustrations of the zoology of South Africa: consisting chiefly of figures and descriptions of the objects of natural history collected during an expedition into the interior of South Africa, in the years 1834, 1835, and 1836; fitted out by “The Cape of Good Hope association for exploring Central Africa”: together with a summary of African zoology, and an inquiry into the geographical ranges of species in that quarter of the globe by Andrew Smith*” published in 28 parts, 1838–1849 (see Waterhouse 1880; Low and Evenhuis 2014). The insect section was issued as part 3.

This work contains three sections, two dealing with Coleoptera: “*On the Cetoniidae of South Africa*” (pp. 3–52) and “*On a new species of Cerapterus*” (pp. 72–75).

#### Maehler, Franz Joseph. Physician in Heidelberg, Germany.

**1850**. *Enumeratio coleopterorum circa Heidelbergam indigenarum adjectis synonymis locisque natalibus.* Ernest Mohr, Heidelbergae. viii + 116 pp. (8vo) [DP: 1850 (title page; preliminaries dated September 1850), 10 November 1850 (*Ent Ver Stettin*), 31 January 1851 (*Börsen Deutsch Buch*), 27 March 1851 (*Allg Bibl Deutsch*), 15 April 1851 (*Intell Serapeum*), January–June 1851 (*Bibliot Hist-Nat*)] GB, MDZ

This work contains a list of the Coleoptera species, with short habitat information for many of them, from the neighborhood of Heidelberg in southwestern Germany.

#### Maillard, J. French abbot, canon and director of the seminary in Séez.

**1850**. *Le petit entomologiste collecteur au nord de Paris. Ou description des insectes qui se trouvent dans un rayon de 120 kilomètres au nord de Paris. I^re^ Partie. Coléoptères.* Victor Masson, Paris. 96 pp. (12mo) [DP: 1850 (title page; p. 6 dated 25 March 1850), 18 May 1850 (*Bibl Fr*)] GAL

#### Mäklin, Friedrich [Fredrik] Wilhelm (Joutseno parish, southeastern Finland: 26 May 1821 – 8 January 1883: Helsinki, Finland). Finnish naturalist; professor of zoology at the University of Helsinki; his collection is at the Finnish Museum of Natural History, University of Helsinki. References. Reuter (1883); Sandahl (1883); Smetana and Herman (2001: 105–106, P).

**1847**. *Ad cognitonem specierum Fennicarum generis Mycetopori symbolae, quas venia amplissimi ordinis philosphorum universitatis in Fennia Alexandrinae praeside Johanne Magno a Tengström, publicae subjicit disquisitioni Fredricus Guilielmus Mäklin, Wiburgensis. In Auditorio Philosophico die XIII Febr. MDCCCXLVII h.a.m.s*. Frenckelliana, Helsingforsiae. 16 pp. (8vo) [DP: 13 February 1847 (title page), 14 April 1847 [JD] (*Soc Imp Nat Moscou*), 14 July 1847 (*Soc Ent Fr*), 28 July 1847 (*Ent Ver Stettin*)] SME, GB

This work is a review of the staphylinid genus *Mycetoporus* of the Finnish territory; several new species are described.

**1853**. *Bidrag till kännedom om insekternas geografiska utbredning i norden med hufvudsakligt afseende på Skandinaviens och Finlands fauna. Akademisk afhandling, hvilken med den Vidtberömda Fysisk-Mathematiska Fakultetens vid Kejserliga Alexanders Universitetet i Finland tillstånd och under inseende af Alexander v. Nordmann, för licentiat-grad till offentlig granskning framställes af Fredrik Wilhelm Mäklin, filos. kandidat, E.O. Amanuens vid Kejserl. Alex. Univ. zoolog. museum. I hist. filol. Lärosalen den 21 Maj 1853. p.v.t.f.m.* J.C. Frenckell & Son, Helsingfors. 42 pp. (8vo) [DP: 21 May 1853 (title page)] SME, GB

This thesis consists of a discussion of northern insects with special consideration for the fauna of Scandinavia and Finland; most of the text pertains to Coleoptera. A German translation by Osten-Sacken was published in *Stettiner Entomologische Zeitung* 18 [1857]: 171–192.

**1855**. *Bidrag till kännedom om såkallade vikarierande former bland Coleoptera i norden. Akademisk afhandling, hvilken med den Vidtberömda Fysisk-Mathematiska Fakultetens vid Kejserliga Alexanders Universitetet i Finland tillstånd till offentlig granskning framställes af Fredrik Wilhelm Mäklin, fys. mathem. licentiat, E.O. Amanuens vid Univ. zoolog. museum. I Hist.-filolog. Lärosalen d. 12 Maj 1855 p.v.t.f.m.* J.C. Frenckell & Son, Helsingfors. 54 pp. (8vo) [DP: 12 May 1855 (title page)] CNC, GB

This thesis consists of an account of the beetles occurring in the north. A German translation by Osten-Sacken was published in *Stettiner Entomologische Zeitung* 18 [1857]: 321–348.

**1862**. *Die Arten der Gattung Acropteron Perty, monographisch dargestellt.* Finnischen Litteratur Gesellschaft, Helsingfors. 23 pp. [DP: 1862 (title page), 3 November 1862 (*Ent Ztg* 24: 32)] GB

This booklet consists of a review of the tenebrionid genus *Acropteron*; several new species are described. This work was also issued in *Acta Societatis Scientiarum Fennicae* 7: 103–127 [DP: 1863 (title page)].

#### Malinowsky, von. Army Captain and Major de Place in Magdeburg where he died; his collection of insects was purchased by the University of Berlin, currently the Humboldt University.

**1816**. *Elementarbuch der Insektenkunde vorzüglich der Käfer. Nebst einer Anweisung die Insekten zu erkennen, zu bestimmen, zu finden, aufzuspiessen, zu sammlen, zu stellen, aufzubewahren und zu versenden. Ein Geschenk für kleine Insektensammler.* Gottfried Basse, Quedlinburg. xi + 228 pp. (8vo) [DP: 1816 (title page; *Vorrede* dated November 1815), January–June 1816 (*Verz Bücher Landkarten*), July 1816 (*Allg Lit Ztg*), May 1817 (*Jena Lit Ztg*)] SBB

#### Mannerheim, Carl Gustav von (near Lemo, Turku ja Pori, Finland: 10 August 1797 – 9 October 1854: Stockholm, Sweden). Swedish (Finnish-born) public servant and entomologist; governor of Viipuri province and chief judge of the Court of Appeals in Viborg [currently Vyborg in Russia] (1839–1854); received the Grand Cross of the Order of Saint Stanislaus and became a Knight of the Order of Saint Vladimir; his collection is at the Finnish Museum of Natural History, University of Helsinki. References. Essig (1931: 698–700, P); Saalas (1954, P); Smetana and Herman (2001: 107–108, P).

**1823**. *Eucnemis, insectorum genus monographice tractatum iconibusque illustratum.* Officina Directorii Institutionis Publicae, Petropoli [= Saint Petersburg]. 36 pp. + 2 pls. (8vo) [DP: 1823 (title page; censur dated 16 March 1823 [JD])] CNC, GB

**1830**. *Précis d’un nouvel arrangement de la famille des brachélytres de l’ordre des insectes coléoptères.* St. Petersbourg. 87 pp. (4to) [DP: 1830 (title page), February 1831 (*Bull Sci Nat Géol* 24: 211)] CNC

This work was reissued in 1831 in *Mémoires présentés à l’Académie Impériale des Sciences de St. Pétersbourg* 1: 415–50l [DP: October 1831 (verso of title page)]. Although the title page of the separate reads “*Extrait du Tome I. des Mémoires présentés à l’Acad. Imp. des sciences de St. Pétersbourg par divers savans*” it is evident that it was issued prior to the journal version.

An extended account of this publication was published by Audinet-Serville in *Bulletin des Sciences Naturelles et de Géologie* 24 [1831]: 211–232.

#### Marschall, August Friedrich (Count) (Vienna, Austria: 10 December 1804 – 11 October 1887: Obermeidling [now part of Vienna], Austria). Austrian geologist and zoologist; court chamberlain and archivist of the Geological Survey of Austria in Vienna. References. Rogenhofer (1887); Pelzeln (1889, P).

**1873**. *Nomenclator zoologicus continens nomina systematica generum animalium tam viventium quam fossilium, secundum ordinem alphabeticum disposita sub auspiciis et sumptibus C.R. Societatis Zoologico-Botanicae.* M. Salzer, Vindobonae [= Vienna]. iv + 482 pp. (8vo) [DP: 1873 (title page; *praefatio* dated 20 October 1873), 5 November 1873 (*Zool-Bot Gesell Wien*), 15 January 1874 (*Kaiser Akad Wiss*), 20 January 1874 (*Kaiser Geol Reichs*)] GB, BHL

The Coleoptera are on pages 164–254.

#### Marseul, Sylvain Augustin de (Fougerolles-du-Plessis, Mayenne, France: 21 January 1812 – 16 April 1890: Paris, France). French priest and coleopterist; after teaching in Paris and Mans, founded an institution of secondary education in Laval (Mayenne) in 1842 where he remained director until 1848; about a year later, became study director at the *Institution Sainte-Marie* in Paris for three years; in 1854, made an eight-month stay in North America during which he met John L. LeConte in Philadelphia; upon his return to Paris, devoted all his time to the study of Coleoptera and particularly the family Histeridae; founded in 1864 the journal *L’Abeille*, devoted principally to the study of European beetles; his collection and library was bequeathed to the Muséum National d’Histoire Naturelle in Paris and his journal *L’Abeille* to the *Société Entomologique de France* which maintained its publication after Marseul’s death. References. Perraudière (1891); Gouillard (2004: 45, P); Cambefort (2006: 230–232, P).

**1857**. *Catalogue des coléoptères d’Europe.* Paris. xvi + 200 pp. (16mo) [DP: 1857 (title page; preliminaries dated 1 March 1857), 26 September 1857 (*Bibl Fr*), November 1857 (*Allg Bibl*), July–December 1857 (*Bibliot Hist-Nat*)] GB, GAL

**1863**. *Catalogue des coléoptères d’Europe et du bassin de la Méditerranée en Afrique & en Asie. 1863. Deuxième édition.* Ach. Deyrolle, Paris. [1] + 300 pp. (12mo) [DP: 1863 (title page; p. [1] dated 15 June 1863), November 1863^74^ (Marseul 1864: xxv), December 1863 (Schaum 1864: i), 4 April 1864 (*Ent Soc Lond*)] CMLE, GB

The first edition was published in 1857 (see previous entry).

**1872**. *Monographie des mylabrides.* J. Desoer, Liége. 302 pp. + 6 pls. (8vo) [DP: 1872 (title page)] GB

This work was also issued in *Mémoires de la Société Royale des Sciences de Liège* (2) 3: 363–662 [DP: 1873 (title page)].

#### Marsham, Thomas (London, United Kingdom: *ca* 1748 – 26 November 1819: London, United Kingdom). British entomologist, with a particular interest in Coleoptera; employed in the Exchequer Loan Office and later as secretary to the West India Dock Company; co-founded the Linnean Society of London for which he was secretary and treasurer for many years; his collection was sold at auction in September 1819 and a first selection was acquired by James Francis Stephens and is now at the Natural History Museum in London. References. Walker (1997); Smetana and Herman (2001: 108, P); Foote (2004b).

**1802**. *Entomologia Britannica, sistens insecta Britanniae indigena, secundum methodum Linnaeanam disposita. Tomus I. Coleoptera.* Wilks et Taylor, Londini. xxxi + 547 + [1 (Errata et corrigenda)] pp. (8vo) [DP: 1802 (title page; preface dated June 1802), August 1802 (*Univers Mag*), September 1802 (Peddie and Waddington 1914: 370)] CNC, GB, BHL

This work was also issued in two volumes under the title “*Coleoptera Britannica, sistens insecta Coleoptera Britanniae indigena, secundum methodum Linnaeanam disposita. In two volumes*” [NAL, GB]. This edition, which is very rare, contains 30 colored plates that are missing in the other edition.

Only the first volume of the planned series was published. It brought Marsham such a loss that his personal finances never recovered (Allen 2010).

#### Martin, Matthew (County of Somerset, United Kingdom: 1748 – 20 November 1838: London, United Kingdom). British merchant, naturalist and philanthropist; tradesman in Exeter; investigated and wrote report on London mendicity. References. Anonymous (1893); Lisney (1960: 222–223, P).

**1787**. *Observations on marine vermes, insects, &c. With notes and quotations from different authors. Fasciculus II.* Exeter. Pp. 13–26 + 1 pl. (4to) [DP: 1787 (cover)] GB

The entire work consists of two fascicles, with the first one published in 1786. Some species of *Cassida* are briefly described (pp. 18–22) but none are new to science.

#### Martorell y Peña, Manuel (1828 – 23 March 1890: Barcelona, Catalonia, Spain). Spanish naturalist, nephew of Miguel Cuní y Martorell [*q.v.*]; first director of the Museum Martorell (currently Museu de Ciències Naturals) in Barcelona (1880–1890). Reference. Gómez-Alba (1990: 12).

**1879**. *Catálogos sinonímicos de los insectos encontrados en Cataluña, aumentados con los recientemente hallados por el autor, en los diversos órdenes de los Coleópteros, Hemípteros, Hymenópteros, Ortópteros, Lepídópteros, Dípteros y Nevrópteros.* Sucesores de N. Ramirez y C., Barcelona. 200 + [1 (Erratas)] pp. (8vo) [DP: 1879 (title page), 7 January 1880 (*Soc Espa Hist Nat*)] GB, BDH

This work consists of a list of insects from Catalonia with synonyms. The Coleoptera are on pages 5–67.

#### Martyn, Thomas (Coventry, Warwickshire, United Kingdom: 1760 – 1816). British naturalist illustrator, entomologist, conchologist and pamphleteer; founded the *Academy for Illustrating and Painting Natural History*; the splendor of his plates for his *Universal Conchologist* (1784–1787) brought him rewards in the form of gold medals from Pope Pius VI, the German Emperor Ferdinand, and the king of Naples; not to be confused with the botanist Thomas Martyn [1735–1825]. References. Boulger (1893); Weiss (1926); McConnell (2004).

**1792**. *The English entomologist: exhibiting all the coleopterous insects found in England; including upwards of 500 different species, the figures of which have never before been given to the public. The whole accurately drawn & painted after nature. Arranged and named according to the Linnean system.* London. 33 + [4 (General table)] pp. + 44 pls [42 numbered hand-colored and 2 unnumbered entitled “*Aurea numismata*”]. (Folio) [DP: 1792 (title page), February 1793 (*Gior Fis-Med*)] HOU, GB

The Coleoptera species are identified by names but the new species, although identified and illustrated, are not named. Two of the three copies I have seen had three unnumbered pages each forming a dedication to Charles IV, King of Spain and the Indies, and dated 21 March 1793. Weiss (1938: 322) mentioned that the paper and plates in some instances carry 1801 watermarks and that the title page was probably engraved in 1792 but the book was actually published later, no doubt between 1793 and 1801. The fact that the book was recorded in the February 1793 issue of a journal published in Pavia, Italy, clearly suggests that the book was first issued in 1792. The dedication was added later to unsold copies or possibly to copies from a subsequent printing.

The book was also issued in French, dated 1792 on the title page, under the title “*Entomologist anglois: ouvrage où l’on a rassemblé tous les insectes coleopteres qui se trouvent en Angleterre et qui forment plus de 500 espèces différentes d’insectes, dont les figures n’ont jamais été données au public; le tout exactement dessiné et peint d’après nature, arrangé selon le système de Linnaeus, et suivant les memes denominations*” [CMLE, GB]. The English version was reissued in May 1836 (Peddie and Waddington 1914: 371) under the title “*The English entomologist: exhibiting all the kind of Coleopterous insects found in England, by descriptions expressed in French and English, and by 500 figures contained in 42 coloured plates*” (*n.v.*) as advertised on the cover of *The Entomological Magazine* for July 1836 (see *Mag Nat Hist* July 1836: 447).

#### Masters, George (Maidstone, Kent, United Kingdom: July 1837 – 23 June 1912: Sydney, Australia). Australian (British-born) gardener, entomologist and energetic collector of natural history specimens; emigrated from England to Australia in 1856; assistant curator at the Australian Museum in Sydney (1864–1874); curator of the Macleay Museum (1874–1912), a position he held after the Museum was moved to the University of Sydney; his private collection of Coleoptera is at the Macleay Museum in Sydney. References. Whitley (1971, P); Marks (1983, P); Zimmerman (1993: 684, P).

**1871-1874**. *Catalogue of the described Coleoptera of Australia*. F. White, Sydney. [1] + 312 pp. (8vo) AMNH

*Part I. Cicindelidae, Carabidae, Dytiscidae, Gyrinidae, Hydrophilidae, Staphylinidae.* Pp. [1] + 1–64 [DP: 1871 (title page; preface dated 29 November 1871)] .

*Part II. Pselaphidae, Paussidae, Scydmaenidae, Silphidae, Scaphididae, Histeridae, Phalacridae, Nitidulidae, Trogositidae, Colydidae, Cucujidae, Cryptophagidae, Latrididae, Mycetophagidae, Dermestidae, Byrrhidae, Georyssidae, Parnidae, Heteroceridae, Lucanidae, Scarabaeidae, Buprestidae.* Pp. 65–128. [DP: 1872 (title page), March 1873 (*Allg Bibl*), January–June 1873 (*Bibliot Hist-Nat*)].

*Part III. Buprestidae, Trixagidae, Eucnemidae, Elateridae, Cebrionidae, Rhipidoceridae, Dascillidae, Malacodermidae, Cleridae, Limexylonidae, Ptinidae, Bostrychidae, Tenebrionidae.* Pp. 129–192. [DP: 1872 (title page), March 1873 (*Allg Bibl*), January–June 1873 (*Bibliot Hist-Nat*)].

*Part IV. Tenebrionidae, Cistelidae, Pythidae, Melandryidae, Lagriidae, Pedilidae, Anthicidae, Pyrochroidae, Mordellidae, Rhipidophoridae, Cantharidae, Oedemeridae, Curculionidae.* Pp. 193–246. [DP: 1872 (title page), 14 May 1873 (*R Soc Victoria*)].

*Part V. Curculionidae, Scolytidae, Brenthidae, Anthotribidae, Cerambycidae, Bruchidae.* Pp. 247–312. [DP: 1874 (title page)].

#### Matthews, Andrew (London, United Kingdom: 18 June 1815 – 14 September 1897: Gumley, Leicestershire, United Kingdom). British clergyman and naturalist with special interest in ornithology and Coleoptera; rector of Gumley for 44 years; his trichopterygids, amphizoids, scaphidiids, silphids and corylophids of the world are at the Natural History Museum in London. Reference. Fowler (1897).

**1872**. *Trichopterygia illustrata et descripta. A monograph of the Trichopterygia. With thirty-one plates.* E.W. Janson, London. xiii + [1] + 188 + [1 (Errata)] pp. + 31 (numbered 1–30, 7A) pls. (4to) [DP: January 1872 (title page), 4 March 1872 (*Ent Soc Lond*), April 1872 (*Ent Monthly Mag* 8: 277)] CNC, GB

**1876**. *An essay on the genus Hydroscapha.* E.W. Janson, London. 20 pp. + 1 pl. (8vo) [DP: 1876 (title page)] GB

**1883**. [Matthews, A. and Fowler, W.W.] *Catalogue of British Coleoptera.* West, Newman & Co., London. 47 pp. (8vo) [DP: 1883 (title page; preface dated December 1882), February 1883 (*Nat Nov*), April 1883 (*Entomologist* 16: 95; *Ent Monthly Mag* 19: 263), June 1883 (*Can Ent* 15: 115), 23 August 1883 (*Zool Anz*)] MANL

**1887–1888**. Fam. Silphidae [pp. 72–101]; Fam. Corylophidae [pp. 102–125]; Fam. Trichopterygidae [pp. 126–156]; Fam. Sphaeriidae [pp. 156–158]; Fam. Scaphidiidae [pp. 158–181]. Pp. 72–181 + pls 3–4 *in*: *Biologia Centrali-Americana. Insecta. Coleoptera. Vol. II. Part 1.* Taylor and Francis, London. xii + 717 pp. + 19 pls. (4to) CNC

This work was published in several parts. The dates at the bottom of the first page of each sheet are as follows: (p. 72) October 1887; (pp. 73–88) November 1887; (pp. 89–96) December 1887; (pp. 97–136 + pl. 3) January 1888; (pp. 137–168 + pl. 4) February 1888; (pp. 169–176) April 1888; (pp. 177–181) June 1888. The collation for the plates is from Lyal (2011: 81).

**1899**. *A monograph of the coleopterous families Corylophidae and Sphaeriidae. Edited by Philip B. Mason. With nine plates.* O.E. Janson & Son, London. [4] + 220 + [1 (Explanation of plates)] pp. + 9 pls. (4to) [DP: 1899 (title page; editor’s preface dated November 1899), December 1899 (*Nat Nov*), 10 March 1900 (*Wien Ent Ztg* 19: 87)] CNC, GB

**1900**. *Trichopterygia illustrata et descripta. A monograph of the Trichopterygia. Supplement. Edited by Philip B. Mason. With seven plates.* O.E. Janson & Son, London. [2] + 112 + [1 (General explanation of plates ix–xv)] pp. + 7 pls. (4to) [DP: 1900 (title page; editor’s preface dated July 1900), September 1900 (*Nat Nov*), October 1900 (*Ent Monthly Mag* 36: 246)] CNC, GB

#### Megerle von Mühlfeld, Johann Karl [Carl] (Vienna, Austria: 1765 – 12 September 1842: Vienna, Austria). Austrian naturalist particularly interested in conchology and entomology; first custodian of the natural history and mineral collections at the Imperial Court Museum of Natural History in Vienna; established an auction hall in the Civic Hospital in Vienna; his first collection, donated in 1808 to the Imperial Court Museum, was destroyed by fire in 1848; a second collection ended up at the Naturhistorisches Museum in Vienna after his death. References. Bergmann (1863: 58–59); Schenkling (1935); Evenhuis (1997b: 525–526).

**1803**. *Appendix ad catalogum insectorum, quae mense Novembris MDCCCII. Viennae Austriae auctionis lege vendita fuere.* Wien. [18] pp. [DP: 2 April 1803 (Evenhuis 1997b: 527)] (*n.v.*).

Megerle von Mühlfeld published 11 auction catalogues, 1801–1805, which consist of lists of specimens for sale along, in some cases, with structural character states. The ICZN (1993) suppressed these catalogues for the purposes of nomenclature but conserved the names *Saperda alboguttata* Megerle, 1803 [p. 10] (Coleoptera: Cerambycidae) and *Hippobosca variegata* Megerle, 1803 [p. 17] (Diptera: Hippoboscidae). The catalogue cited here is the one containing the descriptions of both species.

**1812**. *Bemerkungen, Berichtigungen und Zusätze zu Illiger’s Zusätzen, Berichtigungen und Bemerkungen zu Fabricii Systema Eleutheratorum.* Linz. 44 pp. (8vo) [DP: 1812 (title page), August 1812 (*Ann Öst Lit*), July–December 1812 (*Verz Bücher Landkarten*)] CMLE (mf), GB

This work consists of corrections and additions to Illiger’s comments on Fabricius’ *Systema Eleutheratorum* published in *Magazin für Insektenkunde* 1 [1802]: 306–425; 3 [1804]: 146–180; 4 [1805]: 69–174; 5 [1806]: 221–246; 6 [1807]: 296–317. An extended review of Megerle von Mühlfeld’s book, comprising a reprint of most of the text with numerous comments, was published in 1815 in *Magazin der Entomologie* 1(2): 135–179.

#### Melliss, John Charles (Saint Helena: 23 January 1835 – 23 August 1910: London, United Kingdom). British engineer and naturalist; served as an officer in the Royal Engineers of the British Army; appointed government surveyor in St. Helena (1860–1871); afterwards returned to London and eventually formed the engineering firm J.C. Melliss and Co. Reference. Anonymous (1911).

**1875**. *St. Helena: a physical, historical, and topographical description of the island, including its geology, fauna, flora, and meteorology. The botanical plates from original drawings by Mrs. J.C. Melliss.* L. Reeve & Co., London. xiv + 426 pp. + 56 pls. (8vo) [DP: 1875 (title page; preface dated January 1875), 6 March 1875 (*Athenaeum*), 20 March 1875 (*Academy*), 2 April 1875 (*Publ Circ*), 29 April 1875 (*Nature*), 1 May 1875 (*Publ Weekly*), 8 May 1875 (*Sat Rev*)] GB

The Coleoptera are on pages 131–163.

#### Melsheimer, Frederick Ernst (Bethlehem, Pennsylvania, USA: 28 April 1784 – 10 March 1873: Davidsburg, York County, Pennsylvania, USA). American country physician in Davidsburg, Pennsylvania, interested in astronomy and Coleoptera; son of Frederick Valentine Melsheimer [*q.v.*]; first and only president of the first entomological society in America, the Entomological Society of Pennsylvania, formed in 1842; his collection was sold to Louis Agassiz in 1864 and his now at the Museum of Comparative Zoology in Cambridge, Massachusetts, where it has been incorporated into the general collection; however, the type specimens of Coleoptera were transferred by J.L. LeConte [*q.v.*] into his collection. References. Hagen (1884); Osborn (1937: 13–24); Mallis (1971: 9–13).

**1853**. *Catalogue of the described Coleoptera of the United States. Revised by S.S. Haldeman and J.L. LeConte.* Smithsonian Institution, Washington DC. xvi + 174 pp. (8vo) [DP: July 1853 (title page), 15 August 1853 (*Norton Lit Gaz*)] CNC, BHL, GB

#### Melsheimer, Frederick Valentine (Negenborn, Lower Saxony, Germany: 25 September 1749 – 30 June 1814: Hanover, Pennsylvania, USA). German clergyman and naturalist; at the age of 26 appointed chaplain to the Brunswick Dragoons, an auxiliary regiment, and sailed to Quebec; taken prisoner at the battle of Bennington by the American troops in August 1777; paroled after an imprisonment of 14 months in Massachusetts and sent to New York and later to Pennsylvania where he soon resigned as chaplain and became a pastor of Lutheran churches; married with 11 children of which two, Frederick Ernst [*q.v.*] and Johann Friedrich, became interested in entomology; his collection was given to his son, Frederick Ernst and eventually purchased by Harvard University. References. Hagen (1884); Osborn (1937: 13–24); Mallis (1971: 9–13, P); Onofrio (1999: 145–147).

**1806**. *Catalogue of insects of Pennsylvania. Part first.* Hanover. vi + 60 pp. (8vo) [DP: 1806 (title page; introduction dated August 1806)] BHL

This is the first separate publication on insects published in America. It enumerates 111 genera and 1363 species of Coleoptera (Meisel 1929: 367), but does not contain any available new taxa. Comments about this work were published by Schwarz (1895).

#### Ménétriés, Édouard (Paris, France: 2 October 1802 – 10 April 1861: Saint Petersburg, Russia). French naturalist; worked at the Jardins des Plantes under Cuvier and Latreille; made an expedition to Brazil (1821–1825); moved to Saint Petersburg in 1826; visited Caucasus (1829–1830) and continued to the Caspian coast up to the Persian boundary; curator of the entomological collections of the Zoological Museum of the Academy of Sciences (1832–1861); to meet ends, taught at a college of girls; most specimens from his collections are in the Zoological Institute in Saint Petersburg and the Zoological Museum, Moscow State University, but some types may have ended up in other collections mainly because Motschulsky used the collections as his own, taking a lot of material to his home, retained the most interesting specimens, used duplicates for exchange, and returned to the museum only the remaining. References. Anonymous (1863a, P); Mearns and Mearns (1988: 255–259, P); Adler (2007: 60–61, P).

**1832**. *Catalogue raisonné des objets de zoologie recueillis dans un voyage au Caucase et jusqu’aux frontières actuelles de la Perse entrepris par ordre de S.M. l’Empereur.* Académie Impériale des Sciences, St.-Pétersbourg. xxxii + [2] + 271 + iv + [1 (Rectifications et fautes typographiques)] pp. + 6 pls. (4to) [DP: 1832 (title page), September 1832 (verso of title page), 30 November 1832 [JD] (*Soc Imp Nat Moscou*)] CNC, SME, BHL, GB

The Coleoptera are on pages 90–240. Many new species are described by the author and by Carl Johann Schönherr for the Curculionites.

**1851**. Insecten. Pp. 43–76 + pl. 3 *in*: *Reise in den äussersten Norden und Osten Sibiriens während der Jahre 1843 und 1844 mit allerhöchster Genehmigung auf Veranstaltung der Kaiserlichen Akademie der Wissenschaften zu St. Petersburg ausgeführt und in Verbindung mit vielen Gelehrten herausgegeben von Dr. A. Th. v. Middendorff. Zweiter Band. Theil 1.* Eggers & Comp., St. Petersburg. v + 516 pp. + 32 pls. (4to) [DP: 1851 (title page), 30 September 1851 [JD] (recto of second title page), 31 December 1851 (*Intell Serapeum*), 1 May 1852 (*Lit Centr Deutsch*), January–June 1852 (*Bibliot Hist-Nat*)^75^] AMNH, BHL, GB

The half-title page reads “*Dr. A. Th. v. Middendorff’s Reise in den äussersten Norden und Osten Sibiriens. Band II. Zoologie. Theil 1. Wirbellose Thiere: Annulaten. Echinodermen. Insecten. Krebse. Mollusken. Parasiten. Bearbeitet von F. Brandt, W.F. Erichson, Seb. Fischer, E. Grube, E. Ménétriés, A. Th. v. Middendorff. (Mit 32 lithographirten Tafeln).*” The entire report of Alexander Theodor von Middendorff’s voyage in the extreme northern and eastern Siberia was issued in *Lieferungen*, 1848–1875, forming four volumes, each issued in two parts.

#### Meuschen, Friedrich Christian (Hanau, Hesse, Germany: 15 September 1719 – 20 February 1811: Berlin, Germany). German diplomat, naturalist interested mainly in conchology, and natural history dealer; secretary to the German legation in Copenhagen; later, liaison secretary in The Hague. References. Evenhuis (1997b: 540–541); Holthuis (1998).

**1778**. *Musevm Gronovianvm sive index rervm natvralivm tam mammalivm amphibiorvm piscivm insectorvm conchyliorvm zoophytorvm plantarvm et mineralivm exqvisitissimorvm qvam arte factarvm nonnvllarvm. Inter qvae eminet herbarivs siccvs plantarvm a tovrnefortio claitonio Linnaeo aliisqve botanicis collectarvm. Qvae omnia mvlta cvra et magnis svmptibus sibi comparavit vir amplissimvs & celeberrimvs Lavr. Theod. Gronovivs J.V.D. Quae publice subhasta dictrahentur in aedibus defuncti. Ad diem Mercurii 7. Octobris & seqq. 1778. Diebus I. & 2. Octobris Museum patebit.* Th. Haak & Socios, Lugdvni Batavorvm [= Leiden]. vi + 251 + [1 (Emendanda)] pp. (8vo) [DP: 7 October 1778 (title page)] GB

This work is a catalogue of the zoological, botanical and mineral collections formed by Lorenz Theodor Gronov and put on sale by auction in October 1778. Descriptions of new species are included but the names are not available because the work has been rejected for nomenclatural purposes and placed on the *Official Index of Rejected and Invalid Works in Zoological Nomenclature* (Opinion 260, ICZN 1954c).

**1787**. *Musevm Geversianvm sive index rervm natvralivm continens instrvctissimam copiam pretiosissimorvm omnis generis ex tribvs regnis natvrae objectorvm qvam dvm in vivis erat magna diligentia mvltaqvae cvra comparavit vir amplissimvs Abrahamvs Gevers olim consiliarivs primvsquae vrbis Rotterodamensis consvl praefectvs sylvarvm Hollandiae & Westfrisiae societatis Indiae orientalis director academiae caesareae natvrae cvriosorvm socivs etc. etc. Publice distrahendam Rotterodami in aedibus nobilissimi defuncti diebus 12. Septembris & seqq. 1787.* P. & J. Holsteyn, Rotterodami [= Rotterdam]. [3] + 659 pp. (8vo) [DP: 12 September 1787 (title page), 22 September 1787 (*Gött Anz*), November 1787 (*Esprit J*)] GB

This is an auction catalogue of natural history objects in the collection of Abraham Gevers of Rotterdam. Many Coleoptera species are mentioned, some briefly described. Based on Sherborn’s *Index Animalium* (online version), no new species of beetles are included.

#### Meyer, Ernst Julius Jacob (13 March 1810 – ?). Practicing physician in Berlin and later in Dresden, Germany.

**1840**. *Versuch einer medicinischen Topographie und Statistik der Haupt- und Residenz-Stadt Dresden. Nebst einem Grundrisse von Dresden und drei Tafeln mit graphischen Darstellungen.* B.G.H. Schmidt, Stolberg am Harz und Leipzig. xx + 350 pp. + 3 pls. (4to) [DP: 1840 (title page; p. [vi] dated 11 July 1840), 14 October 1840 (*Lit Ztg*), 15 October 1840 (*Intell Serapeum*), 16 October 1840 (*Allg Bibl Deutsch*), 22 October 1840 (*Bibl Blätt*)] GB, MDZ

Chapter 5 (pp. 73–99) consists of a list of animals found in the surroundings of Dresden. The Coleoptera are on pages 82–94.

#### Meyer, Friedrich Albrecht Anton (Hamburg, Hamburg, Germany: 29 June 1768 – 29 November 1795: Göttingen, Lower Saxony, Germany). German physician and naturalist; lecturer and curator of the museum at the University of Göttingen. References. Hess (1885a); Willenberg (2008: 223); Adler (2012: 49).

**1791**. *Carol. Pet. Thunberg. Characteres generum insectorum, variis cum adnotationibus denuo edidit Frid. Alb. Ant. Meyer.* Ioh. Chr. Dieterich, Gottingae. 48 pp. (8vo) [DP: 1791 (title page; *praefamen* dated 18 January 1791), 15 June 1791 (*Intell Allg Lit Ztg*), 26 October 1791 (*Allg Lit Ztg*)] GB

This is a revised edition of Thunberg’s “*Periculum entomologicum, quo characteres generum insectorum*” which was issued in 1789 [*q.v.*].

**1792**. *Gemeinnützliche Naturgeschichte der giftigen Insekten. Erster Theil, der die Panzerflügel, Pergamentflügel, Staub- und Aderflügel enthält.* Heinrich August Rottmann, Berlin. xii + 187 pp. (8vo) [DP: 1792 (title page; *Vorrede* dated 29 July 1792), 3 December 1792 (*Gött Anz*), 21 February 1793 (*Allg Lit Ztg*)] GDZ, GB

The Coleoptera are on pages 9–93; one new species is described (*Meloe bimaculatus*, p. 49).

**1793**. *Tentamen monographiae generis Meloes.* Vanderhoek et Ruprecht, Gottingae. 32 pp. (8vo) [DP: 1793 (title page; preliminaries dated 5 September 1793), 2 November 1793 (*Gött Anz*)] GB

This work is a revision of the genus *Meloe*; a few new species are described.

#### Meyer, Paul (Hamburg, Hamburg, Germany: 15 May 1876 – 5 April 1951: Vienna, Austria). German businessman and coleopterist; worked in several cities in southern Europe before settling in Vienna. Reference. Scheerpeltz (1952).

**1896**. *Bestimmungs-Tabellen der europäischen Coleopteren: Curculionidae. 4. Theil: (Die palaearctischen Cryptorrhynchiden.) XXXV. Heft.* Edmund Reitter, Paskau. 56 pp. (8vo) [DP: 1896 (title page), 28 October 1896 (*Soc Ent Fr*), October 1896 (*Nat Nov*), 23 November 1896 (*Bibl Zool*)] SME

#### Michow, Heinrich Paul Gotthilf (Stargard [currently Stargard Szczeciński], Poland: 8 December 1839 – 30 May 1916: Hamburg, Germany). German professor and school director in Hamburg; later, assistant at the Institute of Geography at the University of Hamburg. Reference. Anonymous (1916, P).

**1873**. *Inaugural–Dissertation. Die Begrenzung der deutschen Necrophoren-Arten. (Coleopterorum genus Necrophorus Fabr.).* Hossfeld & Oetling, Jena. 29 pp. (8vo) [DP: 1873 (title page)] GB, MDZ

#### Millet de la Turtaudière, Pierre-Aimé (Angers, Maine-et-Loire, France: 30 April 1783 – 18 June 1873: Angers, France). French naturalist of independent means. References. Caumont (1867); Boreau (1874).

[**1870**]. *Faune des invertébrés de Maine-et-Loire comprenant les 2^e^, 3^e^ et 4^e^ embranchements du règne animal ou seconde partie de la faune de Maine-et-Loire. Tome premier.* E. Barassé, Angers. xxi + [1] + 370 pp. (8vo) [DP: 1870 (title page), 21 October 1871 (*Bibl Fr*), July–December 1871 (*Bibliot Hist-Nat*)] GB

The entire work consists of two volumes, 1870–1872. The Coleoptera are on pages 77–300, 349–361 of the first volume. This volume may have been published only in 1871 considering the recording dates found.

#### Milne-Edwards, Henri (Bruges, Belgium: 23 October 1800 – 29 July 1885: Paris, France). French zoologist; professor of hygiene and natural history at *École Centrale des Arts et Manufactures* near Paris; later, held the chair of entomology and eventually the chair of zoology at the Muséum National d’Histoire Naturelle in Paris; also professor of zoology, anatomy and physiology at the Université de Paris; founding member of the *Société Entomologique de France*. References. Anonymous (1885a); Quatrefages (1885); Berthelot (1891); Anthony (1974); Tort (1996); Jaussaud (2004: 382–384).

**1828**. *Résumé d’entomologie, ou d’histoire naturelle des animaux articulés, par MM. V. Audouin et H. Milne Edwards. Tome second. Histoire naturelle des insectes, contenant l’esquisse de l’organisation, des caractères, des moeurs, et de la description de ces animaux, précédée d’une introduction historique, et suivie d’une biographie, d’une bibliographie et d’un vocabulaire; complétée par une iconographie de 48 planches.* Bureaux de l’Encyclopédie Portative, Paris. viii + 260 + [1 (Errata)] pp. (32mo) [DP: 1828 (title page), 6 December 1828 (*Bibl Fr*)] McG, GAL, GB

This publication forms *Livraison* 30 of the “*Encyclopédie portative, ou résumé universel des sciences, des lettres et des arts, en une collection de traités séparés; par une société de savans et de gens de lettres, sous les auspices de MM. Audouin, de Barante, de Blainville, Bory de Saint Vincent, Champollion, Cordier, Cuvier, Depping, C. Dupin, Edwards, Eyriès, de Férussac, de Gérando, Jomard, de Jussieu, Laya, Letronne, Quatremère de Quincy, Thénard et autres savans illustres; et sous la direction de M.C. Bailly de Merlieux*” published in 51 volumes, 1825–1830. The first tome of the *Résumé d’entomologie*, published in 1829, was authored by Jean-Victor Audouin and deals with annelids, crustaceans, and arachnids. The second tome was authored by Milne-Edwards.

The Coleoptera are on pages 86–134. All beetle names are in French.

**1828**. *Iconographie des insectes, ou collection de figures représentant les insectes qui peuvent servir de types pour chaque famille, avec des détails anatomiques, dessinées sur pierre; accompagnée d’une explication des planches par M. H. Milne Edwards, et faisant le complément du Résumé d’entomologie par MM. Audouin et Milne Edwards.* Bureaux de l’Encyclopédie Portative, Paris. 32 pp. + 48 pls. (32mo) [DP: 1828 (title page), 6 December 1828 (*Bibl Fr*), 31 January 1829 (*Rev Bibl-Brus*)] CMLE

This publication forms the *Livraison* 30bis of the “*Encyclopédie portative, ou résumé universel des sciences, des lettres et des arts, en une collection de traités séparés*…”

The *Iconographie* and the *Résumé d’entomologie* (see previous entry) were also issued in 1829 in one octavo volume (8vo) under the title “*Précis d’entomologie ou d’histoire naturelle des animaux articulés, par MM. V. Audouin et H. Milne Edwards. Deuxième division. Histoire naturelle des insectes, contenant l’esquisse de l’organisation, des caractères, des mœurs, et de la description de ces animaux, précédée d’une introduction historique, et suivie d’une biographie, d’une bibliographie et d’un vocabulaire par M.H. Milne Edwards. Ouvrage complété par une iconographie des insectes, ou collection de figures représentant les insectes qui peuvent servir de types, avec des détails anatomiques; dessinées sur pierres, accompagnée d’une explication des planches*” [GB].

**1850–1851**. [Milne-Edwards, H., Blanchard, E. and Lucas, H.] *Muséum d’histoire naturelle de Paris. Catalogue de la collection entomologique. Classe des Insectes. Ordre des Coléoptères. Tome 1.* Gide et Baudry, Paris. iv + 240 pp. (8vo) CNC, GB

The volume was published in two livraisons: **1**: (pp. iv + 1–128) 19 October 1850 (*Bibl Fr*), 9 December 1850 (*Acad Sci Fr*; *Presse*); **2**: (pp. 129–240) 23 July 1851 (*Soc Ent Fr*), 2 August 1851 (*Bibl Fr*), 5 November 1851 (*J Instr Publ*). The title page is dated 1850 and the preface 25 April 1850. The half-title page reads “*Catalogue de la Collection Entomologique du Muséum d’Histoire Naturelle de Paris.*” This volume deals with Scarabaeidae (*sensu lato*). The content of the introduction, written by Milne-Edwards, clearly suggests that Blanchard should be regarded as the sole author of all new taxa proposed in this volume.

#### Mingazzini, Pio (Rome, Italy: 4 May 1864 – 25 May 1905: Florence, Tuscany, Italy). Italian zoologist; professor of zoology at the University of Catania (1897–1901) and after at the University of Messina; accepted in 1903 the chair of invertebrate zoology at the University of Florence. References. Stefanelli (1905); Conci and Poggi (1996 : 290).

**1887**. *La concimazione del terreno vegetale per opera di alcuni Lamellicorni con osservazioni sulle loro abitudini. Tre tavole in fototipia.* Stabilimento Tipograpico Italiano, Roma. 38 + [2] pp. + 3 pls. (8vo) [DP: 1887 (title page)] GB

#### Molina, Juan Ignazio (near Villa Alegre, Maule, Chile: 24 June 1740 – 12 September 1829: Imola, Bologna, Italia). Chilean Jesuit, historian and naturalist; moved to Italy in 1768 when the Jesuits were expelled from Chile; professor of natural history at the University of Bologna. References. Briones (1968, P); Tamayo (2009, P); Charrier and Hervé (2011, P).

**1782**. *Saggio sulla storia naturale del Chili.* S. Tommaso d’Aquino, Bologna. 367 + [1 (Errori)] pp. (8vo) [DP: 1782 (title page)] McG, GB

A second edition of this book was published in1810 (see next entry). A French translation was published under the title “*Essai sur l’histoire naturelle du Chili, par M. l’Abbé Molina; traduit de l’Italien, & enrichi de notes, par M. Gruvel, D.M.*” in Paris, 1789 [McG]. An English translation was published in Middletown (Connecticut), 1808, under the title “*The geographical, natural and civil history of Chili. By Abbe Don. J. Ignatius Molina. Illustrated by a half-sheet map of the country. With notes from the Spanish and French versions, and an appendix, containing copious extracts from the Araucana of Don Alonzo de Ercilla. Translated from the original Italian, by an American gentleman. In two volumes*” [McG, GB]. This edition was reprinted by AMS Press, New York, in 1973. Another English translation was published in 1809, in London, by Longman, Hurst, Rees, and Orme, under the title “*The geographical, natural, and civil history of Chili. Translated from the original Italian of the Abbe Don J. Ignatius Molina. To which are added, notes from the Spanish and French versions, and two appendixes, by the English editor; the first, an account of the archipelago of Chiloe, from the descripcion historial of P.F. Pedro Gonzalez de Agueros; the second, an account of the native tribes who inhabit the southern extremity of South America, extracted chiefly from Falkner’s description of Patagonia. In two volumes*” [CAKL, GB]. A German translation by J.D. Brandis was published in Leipzig, 1786, under the title “*Versuch einer Naturgeschichte von Chili*” [MCZ, MDZ].

Two new species of Coleoptera are described in this book, *Lucanus pilmus* (pp. 209, 347) and *Chrysomela maulica* (pp. 209, 348).

**1810**. *Saggio sulla storia naturale del Chili. Seconda edizione accresciuta e arricchita di una nuova carta geografica e del ritratto dell’autore*. Masi e Comp., Bologna. [3] + v + 306 + [2] pp. (4to) [DP: 1810 (title page)] McG, GB

One new species of beetle is described in this edition, *Scarabaeus citricola* (p. 181).

#### Morley, Claude (Blackheath [currently in Greater London], United Kingdom: 22 June 1874 – 13 November 1951: Woodbridge, Suffolk, United Kingdom). British antiquary and entomologist who specialised in Hymenoptera and Diptera; his collection of beetles was presented to the Bury St. Edmunds Museum about 1905. Reference. Blair (1952).

**1899**. *The Coleoptera of Suffolk*. James H. Keys, Plymouth. xiv + 113 pp. (8vo) [DP: 1899 (title page; introduction dated 31 December 1898), June 1899 (*Nat Nov*; *Ent Monthly Mag* 35: 147; *Entomologist* 32: 165), 1 July 1899 (*Ent Record* 11: 196), 15 July 1899 (*Zool Anz*), August 1899 (*English Cat Books*)] ANNX

This is a catalogue of the species with localities and short habitat notes. A supplement of iv + 12 pages was issued in 1915 by the same publisher (*n.v.*).

#### Morris, Francis Orpen (Cove near Cork, Ireland: 25 March 1810 – 10 February 1893: Newburnholme, East Yorkshire, United Kingdom). Irish clergyman, ornithologist and entomologist; served as curate at various parishes in England; later, rector at Newburnholme. References. Woodward (1894); Morris (1897, P); Kofoid (1938).

**1865–1867**. *A catalogue* [*of*] *British insects, in all the orders.* Longmans, Green, Reader, and Dyer, London. 125 pp. (8vo) [DP: 1865 (title page; preface dated 1 March 1865)] GB

The publication dates, as discussed by Smith (1973), that should be retained for this work are: (pp. 1–8) 26 August 1865; (pp. 9–16) 3 November 1865; (pp. 17–24) 6 January 1866; (pp. 25–32) 12 February 1866; (pp. 33–40) 14 June 1866; (pp. 41–48) 24 December 1866; (pp. 49–56) 11 March 1867; (pp. 57–125) April 1867. The catalogue was noticed “at length published” on 1 June 1867 (*Science-Gossip*). The Coleoptera are on pages 1–31.

#### Motschulsky, Victor de (Saint Petersburg, Russia: 11 April 1810 – 5 June 1871: Simferopol, Crimea). Russian military officer and entomologist; made several collecting trips in Europe and Asia as well as a 10-month trip to the United States and Panama in 1853–1854; his collection was bequeathed to the *Société Impériale des Naturalistes de Moscou* where it was stored in poor condition before being acquired in 1911 by the Zoological Museum, Moscow State University. References. Horn (1927a; 1927b); Essig (1931: 712–715, P); Zimmerman (1993: 698, P); Smetana and Herman (2001: 110–112, P).

**1850**. *Die Kaefer Russlands.* W. Gautier, Moscau. iv + xi + 91 [last page numbered 72] pp. (8vo) [DP: 1850 (title page; approved for publication on 5 May 1850 [JD]), 13 August 1850 (*Ent Ver Stettin*), 23 October 1850 (*Soc Ent Fr*)] CNC

The half-title page reads “*Die Kaefer Russlands. I. Insecta Carabica*.”

**1853**. *Hydrocanthares de la Russie, catalogisés par Victor de Motschulsky*. Société de Litérature Finnoise, Helsingfors. 15 pp. (8vo) [DP: 1853 (title page)] MCZ

**1853–1862**. *Etudes entomologiques.* Société de Litérature Finnoise, Helsingfors [*années* 1–9] / Ferdinand Thomass, Dresde [*années* 10–11]. (8vo) CNC

[*Première année*]. 80 pp. [DP: 1853 (title page), 29 October 1853 [JD] (*Soc Imp Nat Moscou*)].

*Seconde année.* 56 pp. [DP: 1854 (title page), March 1854 (*Rev Mag Zool*), 18 November 1854 [JD] (*Soc Imp Nat Moscou*)].

*Troisième année.* 69 pp. [DP: 1854 (title page), March 1854 (*Rev Mag Zool*), 18 November 1854 [JD] (*Soc Imp Nat Moscou*)].

*Quatrième année.* 84 pp. + 1 pl. [DP: 1855 (title page), 3 January 1856 (*Zool-Bot Gesell Wien*), 28 December 1855 [JD = 9 January 1856] (*Soc Imp Nat Moscou*)].

*Cinquième année.* 88 pp. + 1 pl. [DP: 1856 (title page), 4 February 1857 (*Zool-Bot Gesell Wien*), 24 January 1857 [JD = 5 February 1857] (*Soc Imp Nat Moscou*)].

*Sixième année.* 112 pp. + 1 pl. [DP: 1857 (title page), 14 November 1857 [JD] (*Soc Imp Nat Moscou*)^76^, 5 May 1858 (*Naturfors Ver Riga*), 1 June 1858 (*Acad Nat Sci Phila*)].

*Septième année.* 192 pp. + 1 pl. [DP: 1858 (title page), 18 December 1858 [JD] (*Soc Imp Nat Moscou*)].

*Huitième année.* 187 pp. + 1 pl. [DP: 1859 (title page), 21 April 1860 [JD] (*Soc Imp Nat Moscou*)].

*Neuvième année.* 41 pp. [DP: 1860 (title page), 12 October 1861 [JD] (*Soc Imp Nat Moscou*)].

*Dixième année.* 24 pp. [DP: 1861 (title page), April 1862 (*Nederl Ent Ver*), January–June 1862 (*Bibliot Hist-Nat*), 11 October 1862 [JD] (*Soc Imp Nat Moscou*)].

*Onzième année.* 55 pp. [DP: 1862 (title page), 13 December 1862 [JD] (*Soc Imp Nat Moscou*)].

Although Motschulsky wrote most articles, some were authored by others.

**1860**. *Reisen und Forschungen im Amur-Lande in den Jahren 1854–1856 im Auftrage der Kaiserl. Akademie der Wissenschaften zu St. Petersburg ausgeführt und in Verbindung mit mehreren Gelehrten herausgegeben von Dr. Leopold v. Schrenck. Band II. Zweite Lieferung. Coleopteren. Mit 6 colorirten Tafeln und einer Karte.* Eggers und Comp., H. Schmitzdorff und Jacques Issakof, St-Pétersburg. Pp. 77–257 + [1 (Errata)] + pls 6–11. (4to) [DP^77^: December 1860 (recto of cover), 25 January 1861 [JD] (*Acad Imp Sci St-Pétersb*), 16 February 1861 [JD] (*Soc Imp Nat Moscou*), July 1861 (*Allg Bibl*)] MCZ

The account of Leopold von Schrenck’s trip to the Amur region was published in 12 *Lieferungen* forming four volumes, 1858–1900. Motschulsky’s contribution constitutes the second *Lieferung* of the second *Band*. It has a separate title page which reads “*Coléoptères de la Sibérie orientale et en particulier des rives de l’Amour*” and the first text page (p. 79) is entitled “*Coléoptères rapportés de la Sibérie orientale et notamment des pays situées sur les bords du fleuve Amour par MM. Schrenck, Maack, Ditmar, Voznessenski etc. déterminés et décrits par V. de Motschulsky*.” An extended recapitulation of Motschulsky’s contribution appeared in *L’Abeille* 16 [1878]: 51–168.

The second *Band* was issued in three *Lieferungen*; the first one deals with Lepidoptera and the third one with Molluscs. Its title page is dated 1859–1867 and reads “*Reisen und Forschungen im Amur-Lande in den Jahren 1854–1856 im Auftrage der Kaiserl. Akademie der Wissenschaften zu St. Petersburg ausgeführt und in Verbindung mit mehreren Gelehrten herausgegeben von Dr. Leopold v. Schrenck. Zweiter Band. Zoologie: Lepidopteren, Coleopteren, Mollusken. Mit 28 colorirten Tafeln und 3 Karten.*”

#### Müller, Otto Friedrich [Frederik] (Copenhagen, Denmark: 2 March 1730 – 26 December 1784: Copenhagen, Denmark). Danish naturalist; tutor of a young son of a noble family (1753–1771) with whom he travelled through Germany, Switzerland, Italy, France, and Holland between 1765 and 1767; married a wealthy Norwegian widow in 1773 which enable him to devote himself to science; his insect collection was destroyed in 1801 when the English bombarded Copenhagen. References. Jourdan (1824); Christensen (1924: 135–149, P); Spärck (1932); Anker (1950: 8–14, P); Smetana and Herman (2001: 113, P); Damkaer (2002: 73–87).

**1764**. *Favna insectorvm Fridrichsdalina, sive methodica descriptio insectorvm agri Fridrichsdalensis, cvm characteribvs genericis et specificis, nominibvs trivialibvs, locis natalibvs, iconibvs allegatis, novisqve plvribvs speciebvs additis.* IO. Frid. Gleditschii, Hafniae et Lipsiae [= Copenhagen & Leipzig]. xxiv + 96 pp. (8vo) [DP: 1764 (title page; preliminaries dated January 1764), 26 March 1764 (*Gött Anz*)] CNC, GB, GDZ

This work treats the insects found in the surroundings of the estate of Frederiksdal near Copenhagen. Müller did not cite authors for most species listed in his book. However the majority of the species were made available previously and only those preceded by an asterisk represent new species.

Additions to this work were issued on pages 232–237 in Müller’s “*Flora Fridrichsdalina sive methodica descriptio plantarum in agro Fridrichsdalensi simulque per regnum Daniae crescentium cum characteribus genericis & specificis; nominibus trivialibus, vernaculis, pharmaceuticis; locis natalibus specialissimis; iconibus optimis allegatis, ac speciebus pluribus in Dania nuper detectis*” [GB] published in Argentorati [= Strasbourg], 1767.

**1776**. *Zoologiae Danicae prodromus, seu animalium Daniae et Norvegiae indigenarum characteres, nomina, et synonyma imprimis popularium.* Hallageriis, Havniae. xxxii + 282 pp. (8vo) [DP: 1776 (title page; preface dated 31 March 1776), about 20 May 1776 (Anker 1950: 28), July 1776 (*Allg Verz Bücher*)] McG, GB

As for the previous work, only names preceded by an asterisk in this volume represent new species.

#### Müller, Philipp Ludwig Statius (Esens, Lower Saxony, Germany: 25 April 1725 – 5 January 1776: Erlangen, Bavaria, Germany). German pastor and zoologist; Lutheran clergyman at Amersfoort and Leeuwarden (Netherlands); later, professor of philosophy and librarian at the University of Erlangen. References. Anonymous (1776); Breyer (1776); Hess (1885b); Adler (2012: 39–40).

**1774**. *Des Ritters Carl von Linné Königlich schwedischen Leibarztes &c. &c. vollständiges Natursystem nach der zwölften lateinischen Ausgabe und nach Anleitung des holländischen Houttuynischen Werks mit einer ausführlichen Erklärung. Fünfter Theil. Von den Insecten. Erster Band. Nebst zwey und zwanzig Kupfertafeln. Mit Churfürstlicher Sächsischer Freyheit.* Gabriel Nicolaus Raspe, Nürnberg. [12] + 758 pp. + 22 pls. (8vo) [DP: 1774 (title page; *Vorbericht* dated 28 September 1774), 21 November 1774 (*Jena Ztg*)] GB, GDZ

This is an emended German translation of Linnaeus’ 12th edition of his *Systema Naturae* [*q.v.*]. The entire work was issued in six sections (*Theile*) forming nine volumes (*Bande*), 1773–1776, including a “Supplement und Register” (see next entry). The insects are treated in the fifth section, published in two volumes (1774–1775); the Coleoptera are on pages 45–398 of the first volume.

**1776**. *Des Ritters Carl von Linné Königlich schwedischen Leibarztes &c. &c. vollständiges Natursystems Supplements- und Register-Band über alle sechs Theile oder Classen des Thierreichs. Mit einer ausführlichen Erklärung. Nebst drey Kupfertafeln. Mit Churfürstl. Sächsischer Freyheit.* Gabriel Nicolaus Raspe, Nürnberg. [13] + 384 + [40] pp. + 3 pls. + 536 pp. (8vo) [DP: 1776 (title page; *Vorbericht* dated 4 January 1776), 21 April 1776 (Evenhuis 1997b: 558), 30 September 1776 (*Jena Ztg*), 8 October 1776 (*Gött Anz*)] GB, GDZ

This volume is a supplement and index to the first eight volumes of Müller’s work. The Coleoptera are on pages 213–262.

#### Mulsant, Martial Étienne (Mornand near Villefranche, Rhône, France: 2 March 1797 – 4 November 1880: Lyon, Rhône, France). French ornithologist and entomologist (Coleoptera and Hemiptera); professor of natural history at the Lyceum and librarian in Lyon; chevalier of the Legion of Honour; his impressive *Histoire naturelle des coléoptères de France* brought him to became a corresponding member of the *Académie des Sciences*; Mulsant was probably more popular in Germany, where he was known as *Pater entomologicus*, than in France; his collection passed on to his son, abbot Victor Mulsant, who left it in his monastery of Saint-Chamond where it was neglected before being eventually transferred to the Muséum National d’Histoire Naturelle in Paris. References. Félissis-Rollin (1881); Locard (1882); Smetana and Herman (2001: 114, P); Gouillard (2004: 38–39); Cambefort (2006: 238–239).

**1830–[1831**]. *Lettres à Julie sur l’entomologie, suivies d’une description méthodique de la plus grande partie des insectes de France, ornées de planches, dessinées et gravées par MM. Lanvain et Duménil.* Louis Babeuf, Lyon [&] Treuttel et Wurtz, Paris. (8vo) GB

*Tome I.* x + 392 + [1(Errata; Addenda)] pp. [DP: 1830 (title page), 5 June 1830 (*Bibl Fr*), 7 June 1830 (*Acad Sci Fr*), 10 June 1830 (*Gaz Litt*), 15 August 1830 (*Rev Bibl-Brus*)].

*Tome II.* 402 + [1 (Errata)] pp. + 8 pls. [DP: 1830 (title page), 9 July 1831 (*Bibl Fr*), 27 February 1832 (*Acad Sci Fr*)].

This work consists of two volumes. Despite the date on the title page, the second volume was probably issued only in 1831.

**1839–1863**. *Histoire naturelle des coléoptères de France.* L. Maison [*Longicornes*– *Latipennes*] / Magnin, Blanchard et C^ie^. [*Vésicants*– *Longicornes*], Paris. (8vo) CNC, CMLE, MCZ

*1^re^ Livraison. –Longicornes.* xi + [1] + 304 pp. + 3 pls. [DP: 1839 (title page), 19 October 1839 (*Bibl Fr*), 1 November 1839 (*Allg Bibl Deutsch*), 6 November 1839 (*Lit Ztg*), 29 January 1840 (*J Débats*), February 1840 (*Rev Zool*)]. This work was reissued in 1840 by Maison, Paris [&] Ch. Savy Jeune, Lyon under the same title [GB] and in 1842 by Maison under the title “*Histoire naturelle des coléoptères de France. Longicornes*” [GB].

*Lamellicornes.* viii + 623 + [8 (Rectifications et additions a la monographie des longicornes)] pp. + 3 pls. [DP: 1842 (title page), 18 July 1842 (*Acad Sci Fr*), 6 August 1842 (*Bibl Fr*), 13 August 1842 (*Feuil J Lib*), 17 August 1842 (*Lit Ztg*), 19 August 1842 (*J Débats*), 1 September 1842 (*Monit Lib*), 10 September 1842 (*Monthly Lit Adv*), October–December 1842 (*Foreign Quart Rev*), 31 December 1842 (*Intell Serapeum*)].

*Palpicornes.* vii + 196 pp. + 1 pl. + [1 (Errata et addenda)] + [2 (Lamellicornes.–supplément)] + [2 (longicornes.– supplément)] + [2 (rectifications aux longicornes)] pp. [DP: 1844 (title page), 1 May 1844 (*Monit Lib*), 18 May 1844 (*Bibl Fr*), 21 June 1844 (*Leip Reper*), 24 June 1844 (*Acad Sci Fr*), 3 August 1844 (*J Débats*)].

*Sulcicolles. – Sécuripalpes.* xxiv + [2] + 25 + [2] + 278 + [4 (addenda et errata)] pp. + 1 pl. [DP: 1846 (title page; preface dated 12 November 1845), 26 September 1846 (*Bibl Fr*), 2 October 1846 (*J Débats*), 3 October 1846 (*Feuil J Lib*), 30 October 1846 (*Leip Reper*), October 1846 (*Nouv Rev Encycl*), 6 November 1846 (*Ent Ver Stettin*), July–December 1846 (*Verz Bücher Landkarten*)].

*Latigènes.* [2] + x + 396 + [3 (longicornes. – supplément)] pp. [DP: 1854 (title page; dedication dated 2 October 1854), 16 July 1855 (*Acad Sci Fr*), 21 July 1855 (*Feuil J Lib*), 31 August 1855 (*Intell Serapeum*)].

*Pectinipèdes.* [4] + 96 + [3 (latigènes. – supplément)] + [4 (longicornes, – supplément)] + [2 (palpicornes. – supplément)] + [2 (lamellicornes. – supplément)] pp. [DP: 1856 (title page; dedication dated 15 November 1855), 4 October 1856 (*Feuil J Lib*), 20 December 1856 (*Bibl Fr*; *Courrier Lib*), 17 February 1857 (*Acad Nat Sci Phila*)].

*Barbipalpes*. [4] + 115 pp. + 1 pl. [DP: 1856 (title page; dedication dated 12 September 1856), 5 September 1857 (*Bibl Fr*), November 1857 (*Allg Bibl*)]. Also issued in *Annales de la Société Linnéenne de Lyon* (Nouvelle Série) **3** [1856]: 193–304 [DP: 1856 (title page), 5 September 1857 (*Bibl Fr*), November 1857 (*Allg Bibl*)].

*Longipèdes.* [4] + 171 + [1 (Explication de la planche II)] pp. + 1 pl. [DP: 1856 (title page; dedication dated 10 October 1856), 5 September 1857 (*Bibl Fr*), November 1857 (*Allg Bibl*)]. Also issued in *Annales de la Société Linnéenne de Lyon* (Nouvelle Série) **3** [1856]: 305–471 [DP: 1856 (title page), 5 September 1857 (*Bibl Fr*)].

*Latipennes.* [4] + 44 + [1] + [3 (latigènes. – supplément. – ulomiens)] pp. [DP: 1856 (title page; dedication dated 4 November 1856), 5 September 1857 (*Bibl Fr*), November 1857 (*Allg Bibl*)]. Also published in the *Annales de la Société Linnéenne de Lyon* (Nouvelle Série) **3** [1856]: 473–516 [DP: 1856 (title page), 5 September 1857 (*Bibl Fr*)].

*Vésicants.* [2] + 201 pp. + 1 pl. + [6 (longipèdes. – supplément. – mordelliens)] + [3 (barbipalpes. – supplément. – orchésiens)] pp. [DP: 1857 (title page; dedication dated 10 November 1857), 24 July 1858 (*Feuil Comm*), 28 August 1858 (*Bibl Fr*), 2 September 1858 (*Rev Instr Publ*), 20 November 1858 (*Courrier Lib*), July–December 1858 (*Bibliot Hist-Nat*)]. Also issued in *Annales de la Société Linnéenne de Lyon* (Nouvelle Série) **4** [1857]: 209–416 [DP: 1857 (title page), 28 August 1858 (*Bibl Fr*)].

*Angustipennes*. [4] + 172 + [3 (vesicants. – supplément. – sitarates)] pp. [DP: 1858 (title page; dedication dated 16 December 1858), 17 September 1859 (*Bibl Fr*), September 1859 (Bonvouloir 1859: lxxiii), December 1859 (*Allg Bibl*), July–December 1859 (*Bibliot Hist-Nat*)]. Also issued in the *Annales de la Société Linnéenne de Lyon* (Nouvelle Série) **5** [1858]: 65–242 [DP: 1858 (title page), 24 September 1859 (*Bibl Fr*)].

*Rostrifères.* [4] + 56 pp. [DP: 1859 (title page; dedication dated 4 October 1859), 11 February 1860 (*Bibl Fr*), 14 March 1860 (*J Instr Publ*), March 1860 (*Allg Bibl*)]. Also issued in the *Annales de la Société Linnéenne de Lyon* (Nouvelle Série) **6** [1859]: 49–106 [DP: 1859. – January 1860 (title page), 16 June 1860 (*Bibl Fr*), 20 June 1860 (*J Instr Publ*)].

*Mollipennes.* [4] + 440 pp. + 3 pls. [DP: 1862 (title page; dedication dated 16 May 1862), 13 June 1863 (*Bibl Fr*), July–December 1863 (*Bibliot Hist-Nat*)]. Also issued in the *Annales de la Société Linnéenne de Lyon* (Nouvelle Série) **9** [1862]: 57–496, 596–598 [DP: 1862. – February 1863 (title page), 15 August 1863 (*Bibl Fr*)].

*Longicornes.* [3] + 590 pp. [DP: 1862–63 (title page), 2 January 1864 (*Bibl Fr*), January 1864 (*Allg Bibl*)]. Without qualification, Löbl and Smetana (2010: 803) reported that pages 1–480 were published in 1862 and pages 481–590 in 1863. Also issued in the *Annales des Sciences Physiques et Naturelles d’Agriculture et d’Industrie de Lyon* (Troisième Série) **6** [1862]: 307–466 [DP: 1862 (title page), 9 May 1863 (*Bibl Fr*)], **7** [1863]: 97–320 [DP: 1863 (title page), 4 February 1865 (*Bibl Fr*)], **8** [1864]: 1–208 [DP: 1864 (title page)].

**1851**. *Species des coléoptères trimères sécuripalpes.* Charles Savy Jeune, Lyon. xi + 1104 pp. (8vo) CNC, GB

*Première partie.* vi + 554 pp. + vii–xi. [DP: 1851 (title page; preface dated 29 December 1848), 17 May 1851 (*Feuil J Lib*)].

*Deuxième partie*. Pp. 555–1104. [DP: 1851 (title page), 17 May 1851 (*Feuil J Lib*)].

This work was also issued in the *Annales des Sciences Physiques et Naturelles d’Agriculture et d’Industrie de Lyon* (Deuxième Série) **2** [1850]: xv + pp. 1–1104. [DP: 1850 (title page), 19 December 1850 (*Soc Stat Mars*), 23 December 1850 (*Acad Sci Fr*), 27 January 1851 (*Ent Soc Lond*), 6 February 1851 (*Ent Ver Stettin*)].

A “supplément à la monographie des coléoptères trimères sécuripalpes” was published in 1853 in the third “cahier” of the *Opuscules entomologiques* [*q.v.*].

**1852–1876**. *Opuscules entomologiques.* L. Maison, Paris [cahiers 1–2, 4–7] / F. Dumoulin, Lyon [cahier 3] / Magnin, Blanchard & C^ie^, Paris [cahiers 8–10] / Magnin et Blanchard, Paris [cahiers 11–13] / Deyrolle Fils, Paris [cahiers 14–16]. (8vo) CNC, GAL

*Premier cahier.* [3] + 190 pp. [DP: 1852 (title page; preface dated 18 February 1852), 22 July 1852 (*J Débats*), 24 July 1852 (*Bibl Fr*), 31 July 1852 (*Neuer Anz Bibl*), 7 November 1852 (*Ent Ver Stettin*), July–December 1852 (*Bibliot Hist-Nat*)].

*Deuxième cahier.* viii + 194 pp. + 2 pls. [DP: 1853 (title page; preface dated 2 May 1853), 2 October 1854 (*Ent Soc Lond*)].

*Troisième cahier.* [1] + 205 pp. [DP: 1853 (title page), 2 October 1854 (*Ent Soc Lond*)]. The entire cahier is the “*Supplément a la monographie des Coléoptères trimères sécuripapes*” by Mulsant. The paper was also published in the *Annales de la Société Linnéenne de Lyon* (Nouvelle Série) 1 [1852–53]: 129–333 [DP: 1853 (title page), 2 October 1854 (*Ent Soc Lond*), October 1854 (*Bull Soc Sav*)].

*Quatrième cahier.* [2] + 242 pp. + 4 pls. [DP: 1853 (title page; dedication dated 1 August 1853), 2 October 1854 (*Ent Soc Lond*)]. The entire cahier is the “*Essai d’une division des derniers mélasomes*” by Mulsant and Rey. The paper was also published in the *Mémoires de l’Académie des Sciences, Belles-Lettres et Arts de Lyon* (Nouvelle Série) 2 [1852]: 226–329 [DP: 1852 (title page; after 18 July 1853)], 3 [1853]: 20–158 [DP: 1853 (title page; after 6 December 1853)].

*Cinquième cahier.* 255 pp. [DP: 1854 (title page; dedication dated 25 October 1854), 21 July 1855 (*Feuil J Lib*)]. The entire cahier is the “*Essai d’une division des derniers mélasomes*” by Mulsant and Rey. The paper was also published in the *Mémoires de l’Académie des Sciences, Belles-Lettres et Arts de Lyon* (Nouvelle Série) 4 [1854]: 153–332 [DP: 1854 (title page)] and *Annales de la Société Linnéenne de Lyon* (Nouvelle Série) 2 [1854–55]: 77–142 [DP: 1855 (title page), 2 August 1856 (*Bibl Fr*)].

*Sixième cahier.* [3] + 208 pp. [DP: 1855 (title page; dedication dated 16 December 1855), 4 October 1856 (*Feuil J Lib*)].

*Septième cahier.* viii + 192 pp. [DP: 1856 (title page), 30 May 1857 (*Bibl Fr*), July 1857 (*Allg Bibl*)].

*Huitième cahier.* [3] + 147 pp. + 3 pls. [DP: 1858 (title page; dedication dated 27 July 1858), January 1859 (*Rev Mag Zool*)].

*Neuvième cahier.* [3] + 196 pp. [DP: 1859 (title page; dedication dated 10 May 1859), 19 November 1859 (*Bibl Fr*)].

*Dixième cahier.* [3] + 160 pp. [DP: 1859 (title page; preface dated 20 June 1859), 25 February 1860 (*Bibl Fr*), February 1860 (*Allg Bibl*)].

*Onzième cahier.* [3] + 183 + [1 (Table des espèces décrites)] pp. [DP: 1859–1860 (title page; dedication dated 10 May 1860), 15 December 1860 (*Bibl Fr*)].

*Douzième cahier.* [3] + 195 + [1 (Table alphabétique des espèces décrites)] pp. [DP: 1861 (title page; dedication dated 16 October 1861), April 1862 (*Allg Bibl*), 10 May 1862 (*Bibl Fr*), 15 June 1862 (*Intell Serapeum*)].

*Treizième cahier.* [3] + 190 pp. [DP: 1863 (title page; dedication dated 15 October 1863), 9 March 1864 (*Soc Ent Fr*)].

*Quatorzième cahier.* 241 + [2 (Table alphabétique, Notices, Errata)] pp. + 3 pls. [DP: July 1870 (title page), 1 August 1871 (*Pet Nouv Ent*), September 1871 (*Entomologist* 5: 378)].

*Quinzième cahier.* [2] + 212 pp. [DP: 1873 (title page; dedication dated 12 May 1873), 1 October 1873 (*Pet Nouv Ent*), 25 October 1873 (*Bibl Fr*), July–December 1873 (*Bibliot Hist-Nat*)].

*Seizième cahier.* [2] + 212 pp. [DP: 1875 (title page; dedication dated 8 December 1875), 1 May 1876 (*Pet Nouv Ent*), 6 May 1876 (*Bibl Fr*), 1 June 1876 (*Feuille Jeunes Nat*), September 1876 (*Polybiblion*)].

Each *cahier* contains one to several papers, all with Mulsant as sole author or co-author. All papers were also published in Lyon journals.

**1862**. *Souvenirs d’un voyage en Allemagne*. Magnin, Blanchard et C^ie^, Paris. [4] + 144 pp. (8vo) [DP: 1862 (title page; dedication dated 8 December 1861), 22 November 1862 (*Bibl Fr*)] GB

This work was also issued in the *Annales de la Société Linnéenne de Lyon* (Nouvelle Série) 8: 289–432 [DP: 1861–February 1862 (title page), 11 October 1862 (*Bibl Fr*)].

One new beetle species, *Callidium aeneipennis*, is described and attributed to “Kirchbaumer” in a footnote on page 107.

**1863–1880**. [Mulsant, E. and Rey, C.] *Histoire naturelle des Coléoptères de France.* Magnin, Blanchard et C^ie^ [*Angusticolles* & *Diversipalpes*] / F. Savy [*Térédiles*– *Vésiculifères*] / Deyrolle [*Floricoles*– *Brévipennes. Phléochariens*], Paris. (8vo) CNC, CMLE, GAL

*Angusticolles*. [4] + 134 pp. + 2 pls. [DP: 1863–1864 (title page; dedication dated 8 December 1863), 24 September 1864 (*Bibl Fr*), 12 December 1864 (*Ent Soc Phila*), December 1864 (*Allg Bibl*)]. Also published in the *Annales de la Société Linnéenne de Lyon* (Nouvelle Série) 10 [1863]: 247–380 [DP: 1863 – February 1864^78^ (title page), 17 September 1864 (*Bibl Fr*)].

*Diversipalpes.* [2] + 23 + [1] pp. [DP: 1863–1864 (title page; dedication dated 10 December 1863), 12 December 1864 (*Ent Soc Phila*), 24 September 1864 (*Bibl Fr*)]. Also published in the *Annales de la Société Linnéenne de Lyon* (Nouvelle Série) 10 [1863]: 381–404 [DP: 1863 – February 1864^55^ (title page), 17 September 1864 (*Bibl Fr*)].

*Térédiles.* [5] + 391 + [3 (Table alphabétique des térédiles de France)] pp. + 10 pls. [DP: 1864 (title page; dedication dated 24 July 1864), 18 March 1865 (*Bibl Fr*), May 1865 (*Allg Bibl*)]. Also published in the *Annales de la Société Linnéenne de Lyon* (Nouvelle Série) 11 [1864]: 289–420 [DP: 1864 – February 1864 [error for 1865] (title page), 13 May 1865 (*Bibl Fr*)] and 12 [1865]: 1–284 + 10 pls [DP: 6 January 1866 (title page), 28 April 1866 (*Bibl Fr*)].

*Fossipèdes – Brévicolles.* [4] + 124 pp. + 5 pls. [DP: 1865 (title page; dedication dated 10 June 1865), 27 January 1866 (*Feuil J Lib*), 24 February 1866 (*Bibl Fr*), 1 March 1866 (*Rev Instr Publ*), March–April 1866 (*Allg Bibl*), January–June 1866 (*Bibliot Hist-Nat*)]. Also published in the *Annales des Sciences Physiques et Naturelles d’Agriculture et d’Industrie de Lyon* (Troisième Série) 9 [1865]: 338–468 + 5 pls [DP: 1865 (title page), 25 August 1866 (*Bibl Fr*)].

*Colligères.* [4] + 187 pp. + 3 pls. [DP: February 1866 (title page; dedication dated 2 December 1865), 7 April 1866 (*Feuil J Lib*), 28 April 1866 (*Bibl Fr*), May 1866 (*Allg Bibl*), January–June 1866 (*Bibliot Hist-Nat*)]. Also published, under the title “*Colligères et Simplicitarses*” in the *Annales de la Société Linnéenne de Lyon* (Nouvelle Série) 13 [1866]: 89–282 + 3 pls [DP: 30 June 1866 (title page), 13 October 1866 (*Bibl Fr*)].

*Scuticolles.* [4] + 186 pp. + 2 pls. [DP: 1867 (title page; dedication dated 4 February 1867), 14 September 1867 (*Feuil J Lib*)]. Also published in the *Annales de la Société Linnéenne de Lyon* (Nouvelle Série) 15 [1867]: 1–188 + 3 pls. [DP: 15 January 1868 (title page), 17 October 1868 (*Bibl Fr*), October–December 1868 (*Naturwiss Gesell Isis*)].

*Vésiculifères.* [2] + 2 + 308 + 4 (Table alphabétique and Errata des fossipèdes et brévicolles; numbered 125–128) pp. + 7 pls. [DP: 1867 (title page, dedication dated 14 November 1866), 14 September 1867 (*Feuil J Lib*), 28 September 1867 (*Bibl Fr*), September 1867 (*Allg Bibl*)]. Also published in the *Annales des Sciences Physiques et Naturelles d’Agriculture et d’Industrie de Lyon* (Troisième Série) 11 [1867]: 625–943 + 7 pls [DP: 1867 (title page), 17 July 1869 (*Bibl Fr*)].

*Floricoles.* [4] + 315 + [3 (Table alphabétique de la tribu des Floricoles) pp. + 19 pls [DP: March 1868 (title page; dedication dated 10 October 1867)]. Also published in the *Annales de la Société Linnéenne de Lyon* (Nouvelle Série) 15 [1867]: 237–402 [DP: 15 January 1868 (title page), 17 October 1868 (*Bibl Fr*), October–December 1868 (*Naturwiss Gesell Isis*)] and 16 [1868]: 83–231 + 19 pls [DP: 28 December 1868 (title page), 16 April 1870 (*Bibl Fr*)].

*Gibbicolles.* 8 + 224 + [2 (Table alphabétique)] pp. + 14 pls. [DP: November 1868 (title page; dedication dated 8 September 1868), 1 May 1869 (*Bibl Fr*), 22 May 1869 (*Lit Centr Deutsch*), January–June 1869 (*Bibliot Hist-Nat*)]. Also published in the *Annales de la Société Impériale d’agriculture d’Histoire naturelle et Arts utiles de Lyon* (Quatrième Série) 1 [1868]: 179–420 + 14 pls [DP: 1869 (title page), 15 July 1871 (*Bibl Fr*)].

*Piluliformes.* [4] + 175 + [1 (Piluliformes paraissant jusqu’a ce jour étrangers a la France et décrits dans ce volume)] + [2 (Explication des planches)] pp. + 2 pls. [DP: 1869 (title page; dedication dated 25 April 1869), 20 November 1869 (*Bibl Fr*), 15 December 1869 (*Pet Nouv Ent*), July–December 1869 (*Bibliot Hist-Nat*), January 1870 (*Polybiblion*)]. Also published in the *Annales de la Société Linnéenne de Lyon* (Nouvelle Série) 17 [1869]: 201–378 + [2] + 2 pls [DP: 28 December 1869 (title page), 16 April 1870 (*Bibl Fr*)].

*Lamellicornes – Pectinicornes.* [2] + 735 (Lamellicornes) + [1 (Genres contenus dans ce volume)] + 42 (Tribu des pectinicornes) + [1 (Tableau des pectinicornes de France)] pp. + 3 pls. [DP: September 1871 (title page; dedication dated 8 September 1871), 1 August 1872 (*Pet Nouv Ent*), 12 October 1872 (*Bibl Fr*), July–December 1872 (*Bibliot Hist-Nat*), December 1872 (*Polybiblion*)]. Also published in the *Annales de la Société d’Agriculture Histoire Naturelle et Arts utiles de Lyon* (Quatrième Série) 2 [1869]: 241–650 [DP: 1870 (title page), 3 November 1871 (*Soc Agr Hist Nat Arts Lyon*), 27 January 1872 (*Bibl Fr*)] and 3 [1870]: 155–480 + 2 pls [Lamellicornes], 481–523 + 1 pl. [Pecticornes] [DP: 1871 (title page), 12 April 1873 (*Bibl Fr*), 6 September 1873 (*Soc Ent Belg*)]. In the journal issue, Mulsant is listed as sole author of this work in the *Table des matières*.

*Improsternés – Uncifères – Diversicornes spinipèdes.* [2] + 17 + [1 (Tableau des Improsternés de France)] + 57 + 39 + [1 (Tableau des Diversicornes de France)] + 57 + [1 (Tableau des Spinipèdes)] pp. + 2 pls. [DP: December 1872 (title page; dedication dated 8 December 1872), 3 May 1873 (*Soc Ent Belg*), 15 May 1873 (*Pet Nouv Ent*), 16 August 1873 (*Bibl Fr*), 6 September 1873 (*Soc Ent Belg*), October 1873 (*Polybiblion*), July–December 1873 (*Bibliot Hist-Nat*)]. Also published in the *Annales de la Société d’Agriculture Histoire Naturelle et Arts utiles de Lyon* (Quatrième Série) 4 [1871]: 61–78 [Improsternés], 79–136 [Uncifères], 137–176 [Diversicornes], 177–234 [Spinipèdes] [DP: 1872 (title page), 11 April 1874 (*Soc Ent Belg*), 18 April 1874 (*Bibl Fr*)]. In the journal issue, Mulsant is listed as sole author in the *Table des matières*.

*Brévipennes. (Aléochariens).* [4] + 321 + [5] + [3 (Table alphabétique des coléoptères brévipennes) + [1 (Erratum)] pp. + 5 pls. [DP: 1871 (title page; dedication dated 25 August 1871), 6 September 1873 (*Soc Ent Belg*)]. Also published in the *Annales de la Société Linnéenne de Lyon* (Nouvelle Série) 19 [1872]: 91–411 + [11] + 5 pls [DP: 31 December 1872 (title page), 9 July 1873 (*Soc Ent Fr*), 19 July 1873 (*Bibl Fr*), 6 September 1873 (*Soc Ent Belg*)].

*Brévipennes (Aléochariens).* [4] + 155 pp. + 2 pls. [DP: 1873 (title page; dedication dated 4 July 1873), before 25 December 1873 (mentioned in a letter to Brisout de Barneville dated “25 décembre 1873” published by Fauvel (1874: 70–80)), 10 January 1874 (*Bibl Fr*), 28 January 1874 (*Soc Ent Fr*), 2 February 1874 (*Acad Sci Fr*), February 1874 (*Polybiblion*), January–June 1874 (*Bibliot Hist-Nat*)]. Also published in the *Mémoires de l’Académie des Sciences, Belles-Lettres & Arts de Lyon, Classe des Sciences* 20 [1873–74]: 23–175 + 2 pls. [DP: 11 July 1874 (*Bibl Fr*), July–December 1874 (*Bibliot Hist-Nat*)].

*Brévipennes Aléochariens – suite –.* [2] + 695 pp. + 5 pls. [DP: December 1873 (title page; dedication dated 18 December 1873), 11 April 1874 (*Acad Sci Fr*), 1 May 1874 (*Pet Nouv Ent*), 2 May 1874 (*Bibl Fr*), 23 May 1874 (*Lit Centr Deutsch*), June 1874 (*Polybiblion*), January–June 1874 (*Bibliot Hist-Nat*)]. Also published in the *Annales de la Société d’Agriculture, Histoire Naturelles et Arts utiles de Lyon* (Quatrième Série) 6 [1873]: 33–738 + 5 pls [DP: 1874 (title page), 19 February 1875 (*Soc Agr Hist Nat Arts Lyon*), 17 April 1875 (*Bibl Fr*), January–June 1875 (*Bibliot Hist-Nat*), 8 September 1875 (*Soc Ent Fr*)]. In the journal issue, Mulsant is listed as the sole author.

*Brévipennes Aléochariens (suite). – Aléocharaires*. [2] + 565 pp. + 5 pls. [DP: December 1874 (title page; dedication dated 8 December 1874), 1 February 1875 (*Pet Nouv Ent*), 14 April 1875 (*Soc Ent Fr*), 17 April 1875 (*Bibl Fr*), 17 May 1875 (*Acad Sci Fr*), 29 May 1875 (*Lit Centr Deutsch*), January–June 1875 (*Bibliot Hist-Nat*), July 1875 (*Polybiblion*)]. Also published in the *Annales de la Société Linnéenne de Lyon* (Nouvelle Série) 20 [1873]: 285–447 [DP: 1874 (title page), 9 September 1874 (*Soc Ent Fr*), 17 October 1874 (*Bibl Fr*), 5 December 1874 (*Soc Ent Belg*)] and 21 [1874]: 1–403 + [5] + 5 pls [DP: 1875 (title page), 14 April 1875 (*Soc Ent Fr*), 24 April 1875 (*Bibl Fr*), 17 May 1975 (*Acad Sci Fr*)].

*Brévipennes. Aléochariens (suite). – Myrmédoniaires (2e partie).* [2] + 470 pp. + 4 pls. [DP: November 1875 (title page; dedication dated 1 October 1875), November 1875 (Desmarest et al. 1876: xx), 22 January 1876 (*Bibl Fr*), 1 February 1876 (*Pet Nouv Ent*), 9 February 1876 (*Soc Ent Fr*), 1 March 1876 (*Feuille Jeunes Nat*), 4 March 1876 (*Lit Centr Deutsch*), June 1876 (*Polybiblion*)]. Also published in the *Annales de la Société d’Agriculture, Histoire Naturelles et Arts utiles de Lyon* (Quatrième Série) 7 [1874]: 27–503 + 9 pls [DP: 1875 (title page), 15 July 1876 (*Bibl Fr*), 11 October 1876 (*Soc Ent Fr*)].

*Brévipennes – Suite.* [4] + 712 pp. + 6 pls. [DP: May 1877 (title page; dedication dated 17 May 1877), 11 August 1877 (*Bibl Fr*), 15 August 1877 (*Pet Nouv Ent*), 1 October 1877 (*Feuille Jeunes Nat*), October 1877 (*Polybiblion*), July–December 1877 (*Bibliot Hist-Nat*)]. Also published in the *Annales de la Société d’Agriculture, Histoire Naturelles et Arts utiles de Lyon* (Quatrième Série) 8 [1875]: 145–856 + 6 pls [DP: 1876 (title page), 21 July 1877 (*Bibl Fr*), July–December 1877 (*Bibliot Hist-Nat*), 12 June 1878 (*Soc Ent Fr*)].

*Brévipennes Xantholiniens.* [2] + 128 + [3 (Explication des planches)] pp. + 3 pls. [DP: June 1877 (title page; dedication dated 10 June 1877), 15 August 1877 (*Pet Nouv Ent*), 25 August 1877 (*Bibl Fr*), 1 October 1877 (*Feuille Jeunes Nat*), July–December 1877 (*Bibliot Hist-Nat*)]. Also issued in *Mémoires de l’Académie des Sciences, Belles-Lettres & Arts de Lyon, Classe des Sciences* 22: 217–344 + 3 pls [DP: 1876–1877 (title page), 27 October 1877 (*Bibl Fr*), July–December 1877 (*Bibliot Hist-Nat*)].

*Brévipennes. Pédériens. – Évesthétiens.* [2] + 338 + [1 (Tableau méthodique de la famille des Évesthétiens)] + [1 (Table alphabetique des espèces décrites (Évesthétiens)] pp. + 6 pls. [DP: 1878 (title page; dedication dated 10 May 1878), 15 November 1878 (*Pet Nouv Ent*), 23 November 1878 (*Bibl Fr*), July–December 1878 (*Bibliot Hist-Nat*), 11 January 1879 (*Lit Centr Deutsch*), 27 January 1879 (*Zool Anz*), January 1879 (*Nat Nov*), 12 February 1879 (*Soc Ent Fr*), February 1879 (*Polybiblion*)]. Also issued in the *Annales de la Société Linnéenne de Lyon* (Nouvelle Série) 24 [1877]: 1–338 + [9] + 6 pls [DP: October 1878 (title page), 13 November 1878 (*Soc Ent Fr*), 8 February 1879 (*Bibl Fr*), February 1879 (*Nat Nov*)].

*Brévipennes. Oxyporiens. – Oxytéliens.* [2] + 408 pp. + 7 pls. [DP: 1879 (title page; dedication dated 10 January 1879), 1 August 1879 (*Naturaliste*), August 1879 (*Nat Nov*)]. Also published in the *Annales de la Société d’agriculture, Histoire naturelle et Arts utiles de Lyon* (Quatrième Série) 10 [1877]: 443–863 + 7 pls [DP: 1878 (title page), 19 July 1879 (*Bibl Fr*), August 1879 (*Nat Nov*), 24 March 1880 (*Soc Ent Fr*)].

*Brévipennes. Phléochariens. – Trigonuriens. – Protéiniens – Phléobiens.* 74 pp. + 2 pls. [DP: 1879 (title page), December 1879 (*Nat Nov*), 24 January 1880 (*Bibl Fr*), 3 May 1880 (*Zool Anz*), January–June 1880 (*Bibliot Hist-Nat*), 28 July 1880 (*Soc Ent Fr*)]. Also issued in the *Annales de la Société Linnéenne de Lyon* (Nouvelle Série) 25 [1878]: 191–264 + [3] + 2 pls [DP: October 1879 (title page)^79^, 7 February 1880 (*Bibl Fr*), February 1880 (*Nat Nov*), 23 June 1880 (*Soc Ent Fr*), January–June 1880 (*Bibliot Hist-Nat*)].

This series was continued by Rey alone [*q.v.*].

**1866**. *Monographie des coccinellides. Ire partie coccinelliens.* F. Savy [&] Deyrolle, Paris. 292 + [2] pp. (8vo) [DP: 1866 (title page), 24 March 1866 (*Bibl Fr*), March–April 1866 (*Allg Bibl*), January–June 1866 (*Bibliot Hist-Nat*)] CMLE, GB, GAL

This work was also issued in *Mémoires de l’Académie des Sciences, Belles-Lettres & Arts de Lyon, Classe des Sciences* 15: 1–112 [DP: 1865–66 (title page), 13 July 1867 (*Bibl Fr*)], 16: 1–112 [DP: 1866–67 (title page), 25 July 1868 (*Bibl Fr*)] and 17: 1–66 [DP: 1869–70 (title page), 16 April 1870 (*Bibl Fr*)].

#### Munck af Rosenschöld, Eberhard (Lund, Sweden: 11 July 1811 – 1869: Paraguay). Swedish physician and naturalist; participated to the expedition of Göran Adolph Oxehufvud to South America in 1840; later, settled in Paraguay where he practiced medicine and studied natural history; executed in 1869 on order of president Francisco Solano López. References. Hofberg (1876b: 34); Anonymous (1877); Mörner (1955).

**1835**. *Prodromus faunae coleopterorum Lundensis, quem, venia ampl. facult. philos. Lundensis, praeside Sv. Nilsson. Pro gradu philos. doctoris publicae disquisitioni subjicit auctor Eb. Munck af Rosenschold, Lundensis. In Acad. Carol. die XVIII Junii MDCCCXXXV. Part 1.* C.Fr. Berling, Lundae. 20 pp. (8vo) [DP: 18 June 1835 (title page)] YB (ph, partial)

This thesis is a preliminary work on the Coleoptera of Lund, Sweden. According to Sherborn (1922a: xcvi) there are no new species described in it. Both Hagen (1863: 14) and Sherborn (1922a: xcvi) recorded this work under Sven Nilsson [1787–1883].

#### Murray, Andrew Dickson (Edinburgh, Scotland, United Kingdom: 19 February 1812 – 10 January 1878: London, United Kingdom). British judicial officer and naturalist; writer to the signet in Edinburgh; later, assistant secretary to the Royal Horticultural Society in London; travelled to Utah and California in 1873; the whereabouts of his collection are unknown. References. Anonymous (1878c); Boulger (1894).

**1853**. *Catalogue of the Coleoptera of Scotland by Andrew Murray of Conland, W.S. Aided by the Rev. William Little, Messieurs James Hardy, Robt. Hislop, John T. Syme, Dr W.H. Lowe, and other Scottish entomologists.* William Blackwood and Sons, Edinburgh and London. viii + 145 + [1 (Errata)] pp. (8vo) [DP: 1853 (title page; preface dated April 1853), 1 August 1853 (*Ent Soc Lond*), 1 October 1853 (*Publ Circ*)] CNC (mf), BHL, GB

[**1856**]. Order 1.–Coleoptera. Pp. 421–446 *in*: *The natural history of Dee Side and Braemar. By the late William MacGillivray. Edited by Edwin Lankester.* London. xx + 507 pp. + 5 pls. (8vo) [DP: 1855 (title page; editor’s preface dated 10 August 1855), 9 February 1856 (*Lancet*), 23 February 1856 (*Athenaeum*), 1 March 1856 (*Med Times Gaz*), March 1856 (*Gent Mag*)] GB

Murray’s contribution consists of an annotated list of beetles from the region of Aberdeenshire county in east Scotland. Despite the date on the title page, this book seems to have been distributed only in 1856.

#### Nicolai, Ernst August (Arnstadt, Thuringia, Germany: 2 December 1800 – 2 October 1874: Arnstadt, Germany). German physician and naturalist in Arnstadt; his collection of insects is lost. Reference. Reinhold (2015: 72).

**1822**. *Dissertatio inauguralis medica sistens coleopterorum species agri Halensis quam consensu illustrissimi medicorum ordinis in celeberrima Academia Fridericiana Halensi et Vitebergensi consociata pro summis in medicina et chirurgia honoribus rite obtinendis. Die X. mensis septembris MDCCCXXII. Publice defendet auctor Ernestus Augustus Nicolai Arnstadio-Thuringus.* Frid. Aug. Grunert, Halae [= Halle]. 44 + [4] pp. (8vo) [DP: 10 September 1822 (title page)] CAKL, SBB

This thesis is an annotated catalogue of the Coleoptera found in fields of the German city of Halle; several new species are described.

#### Nodier, Charles (Besançon, Doubs, France: 29 April 1780 – 29 January 1844: Paris, France). French bibliophile, bibliographer, librarian, well-known storyteller and entomologist up to his 40s; member of the *Académie Française*; honorary member of the *Société Entomologique de France*; his beetle collection was dispersed. References. Mennessier-Nodier (1867); Magnin (1911); Oliver (1964, P); Cambefort (2006: 243).

**1821**. *Promenade de Dieppe aux montagnes d’Écosse.* J.N. Barba, Paris. 334 + [1] pp. + 3 pls. (12mo) [DP: 1821 (title page), 17 November 1821 (*Bibl Fr*), 5 December 1821 (*Album*)] HOU, GB, GAL

The new species *Carabus hookeri* is described in a footnote on pages 224 and 225.

#### Nördlinger, Hermann von (Stuttgart, Baden-Württemberg, Germany: 13 August 1818 – 19 January 1897: Ludwigsburg, Baden-Württemberg, Germany). German forester, botanist and entomologist; professor at the Grand-Jouan agricultural school at Nozay (France); later, professor of silviculture in Hohenheim and Tübingen (Germany). References. Ratzeburg (1874: 379–380); Graner (1897); Rösler (1999).

**1855**. *Die kleinen Feinde der Landwirthschaft oder Abhandlung der in Feld, Garten und Haus schädlichen oder lästigen Kerfe, sonstigen Gliederthierchen, Würmer und Schnecken, mit besonderer Berücksichtigung ihrer natürlichen Feinde und der gegen sie anwendbaren Schutzmittel. Mit Holzschnitten von Allgaier & Siegle nach Zeichnungen von Schnorr & Federer.* J.G. Cotta, Stuttgart und Augsburg. xxiv + 636 pp. (8vo) [DP: 1855 (title page; *Einleitung* dated January 1855), April 1855 (*Lit Deutsch Natur Ztg*), 10 May 1855 (*Allg Bibl Deutsch*), January–June 1855 (*Verz Bücher Landkarten*), 16 July 1855 (*Norton Lit Gaz*)] CMLE, GB

The Coleoptera are on pages 61–207. A second edition was published in 1869 [*q.v.*].

**1856**. *Nachträge zu Ratzeburg’s Forstinsekten. Ein Programm bei Gelegenheit der Jahresprüfung an der Königl. land- und forstwirthschaftlichen Akademie zu Hohenheim im August 1856.* Julius Weise, Stuttgart. iv + 83 pp. + 1 pl. (8vo) [DP: August 1856 (title page; *Einleitung* dated July 1856), 6 November 1856 (*Allg Bibl Deutsch*), December 1856 (*Allg Bibl*), July–December 1856 (*Bibliot Hist-Nat*), October–December 1856 (*Cat Muquardt*)] CMLE, GB

A second edition was issued in 1880 [*q.v.*].

**1869**. *Die kleinen Feinde der Landwirthschaft oder Abhandlung der in Feld, Garten und Haus schädlichen oder lästigen Schnecken, Würmer, Gliederthierchen, insbesondere Kerfe, mit Berücksichtigung ihrer natürlichen Feinde und der gegen sie anwendbaren Schutzmittel. Mit Holzschnitten von Allgaier & Siegle, nach Zeichnungen von Schnorr und Federer. Zweite Auflage.* J.G. Cotta, Stuttgart. xxiv + 760 pp. (8vo) [DP: 1869 (title page; *Vorwort* dated August 1869), 11 November 1869 (*Allg Bibl Deutsch*), July–December 1869 (*Bibliot Hist-Nat*; *Verz Bücher Landkarten*)] GB

The Coleoptera are on pages 76–264. The first edition was published in 1855 [*q.v.*].

**1880**. *Lebensweise von Forstkerfen oder Nachträge zu Ratzeburg’s Forstinsekten. Zweite vermehrte Auflage.* J.G. Cotta, Stuttgart. v + 73 pp. (4to) [DP: 1880 (title page; *Einleitung* dated December 1879), June 1880 (*Nat Nov*), January–June 1880 (*Bibliot Hist-Nat*), 10 July 1880 (*Lit Centr Deutsch*), July 1880 (*Zeit Forst Jagd*), September 1880 (*Allg Forst Jagd Zeit*)] BHL

The Coleoptera are on pages 1–44. The first edition was published in 1856 [*q.v.*].

**1884**. *Lehrbuch des Forstschutzes. Abhandlungen der Beschädigungen des Waldes durch Menschen, Thiere und die Elemente unbelebter Natur, sowie der dagegen zu ergreifenden Massregeln. Mit 222 in den Text gedruckten Holzschnitten.* Paul Parey, Berlin. xxiv + 520 pp. (8vo) [DP: 1884 (title page; *Einleitung* dated November 1883), 26 April 1884 (*Lit Centr Deutsch*), April 1884 (*Nat Nov*), May 1884 (*Allg Bibl*), January–June 1884 (*Bibliot Hist-Nat*)] GB

The Coleoptera are on pages 128–209.

#### Nordmann, Alexander von (Ruotensalmi [currently Kotka], Kymi, Finland: 24 May 1803 – 25 June 1866: Helsinki, Finland). Finnish naturalist and artist; professor of zoology and botany at the Richelieu Lyceum in Odessa; later, professor of natural history at the University of Helsinki. References. Eichwald (1870); Mearns and Mearns (1988: 284–287, P); Damkaer (2002: 253–271, P); Adler (2012: 109–110, P).

**1837**. *Symbolae ad monographiam Staphylinorum*. Academiae Caesareae Scientiarum, Petropoli [= Saint Petersburg]. 167 pp. + 2 pls. (4to) [DP: 1837 (title page), 9 February 1838 (*Allg Bibl Deutsch*)] GB, MDZ

This publication was also issued the same year in livraison 1–2 of the *Mémoires présentés à l’Académie Impériale des Sciences de Saint-Pétersbourg par divers savants, et lus dans ses Assemblées* 4 [1845]: 1–167 (+ 2 pls) [DP: October 1837 (recto of title page of the livraison), 16 November 1837 [JD] (*Acad Imp Sci St-Pétersb*)].

The work consists of a review of the species of *Staphylinidae* (*sensu stricto*); many new taxa (genera, species) are described.

#### Nowicki, Maksymilian Siła (Jablunkov, Czech Republic: 9 October 1826 – 30 October 1890: Kraków, Poland). Polish zoologist and pioneer conservationist; professor of zoology at the University of Kraków (1863–1890); his collection of Polish insects is at the Museum Dzieduszycki in Lviv, Ukraine. References. Anonymous (1887: 191–195); Wierzejski (1891, P); Fedorowicz and Kawecki (1962, P).

**1872**. *Beschreibung einer neuen Käferart nebst Ausweis der Literatur über die Käferfauna Galiziens.* Krakau. 7 pp. (8vo) [DP: 1872 (title page), 12 March 1873 (*Naturfors Ver Brünn*), March 1873 (*Zool Gart*)] MCZ

The new species described is Clythra (Labidostomis) kluczyckii (p. 3).

**1873**. *Beiträge zur Insektenfauna Galiziens.* Jagellonische Universitäts-Buchdruckerei, Krakau. 52 pp. (8vo) [DP: 1873 (title page)] YB (ph)

This work comprises two sections: *Beschreibung neuer Dipteren* (pp. 3–6) and *Verzeichniss galizischer Käfer* (pp. 7–52); the last section constitutes a catalogue of the Coleoptera of Galicia, an historic area and former Austrian crown land in east central Europe.

**1873**. *Beschreibung neuer Käferarten.* Jagellonische Universitäts-Buchdruckerei, Krakau. 6 pp. (8vo) [DP: 1873 (title page), 20 September 1873 [JD] (*Soc Imp Nat Moscou*)] YB (ph)

The new species described are: *Feronia ehrhartii* (p. 3), *Evaniocera striolata* (p. 5), and *Mylabris obsoleta* (p. 6).

#### Oberthür, René (Rennes, Ille-et-Vilaine, France: 14 April 1852 – 27 April 1944: Rennes, France). French coleopterist of independent means; involved in the very successful printing business founded by his father; built one of the largest privately owned collection of beetles ever assembled which was sold to the Muséum National d’Histoire Naturelle in 1952 for the sum of 32 million francs. References. Cambefort (2004: P); Cambefort (2006: 244–250).

**1883–1884**. *Coleopterorum novitates. Recueil spécialement consacré à l’étude des coléoptères. Tome Ier.* René Oberthür, Rennes. 80 pp. + 2 pls. (8vo) YB

This work was issued in four parts as follows: **1**: (pp. 1–16) 15 June 1883 (p. 16); **2**: (pp. 17–48) 1 August 1883 (p. 46), 28 September 1883 (*Science*); **3**: (pp. 49–64) August 1883 (p. 64); **4**: (pp. 65–80) January 1884 (p. 66). The following articles, all authored by R. Oberthür unless otherwise noted, are included: *Scaphidiides nouveaux* (pp. 5–16); *Description de carabiques nouveaux* by Chaudoir (pp. 17–39); *Nouvelles espèces de Monommides* (pp. 40–46); *Trois Nebria nouvelles* (pp. 47–48); *Description d’un Coptolabrus nouveau* (p. 50); *Liste des carabiques récoltés à Saint-Laurent-du-Maroni, en 1878 et 1879 par M. le Dr Charles Nodier médecin de la marine et description des espèces nouvelles* (pp. 51–54); *Description d’un genre nouveau et de deux espèces nouvelles de géotrupides* (p. 55–58); *Trois espèces nouvelles du genre Helota* (pp. 59–61); *Note synonymique sur le genre Lemodes, Boh. et description de deux espèces nouvelles* (pp. 62–64); *Note sur le genre Chalaenus, Westw.* by Leon Fairmaire (pp. 65–66); *Description d’espèces nouvelles d’hétéromères de Madagascar* by L. Fairmaire (pp. 67–80). The last paper is unfinished and the only publication I know that recorded some of the new species is Champion (1895) (see Froussart 1961: 56). The following new taxa are described in Fairmaire’s paper: *Himatismus emarginifrons (p. 67), *Uloma curvipes* (p. 68), *Pycna (p. 68), *Pycna aphodina (p. 69), *Dolichoderus pentaphyllus* (p. 69), *Dolichoderus furcillatus* (p. 69), *Nycteropus splendidulus (p. 70), *Nycteropus caeruleipes (p. 70), *Camaria trapezicollis (p. 71), *Amarsenes viridistriatus (p. 71), *Amarsenes humbloti* (p. 72), *Amarsenes chalcophanus (p. 72), *Porphyrhyba alternecostata* (p. 73), *Camariodes cuprinus* (p. 74), *Chalcocyclus* (p. 74), *Chalcocyclus speculifer* (p. 74), *Nesogena subparallela* (p. 75), *Nesogena rugulicollis* (p. 75), *Nesogena purpuriventris* (p. 76), *Nesogena humerosa (p. 76), *Nesogena fuscoaenea (p. 77), *Nesogena obscuripes (p. 77), *Nesogena fastidiosa (p. 77), *Hyperchalca humbloti (p. 78), *Cistela alternata* (p. 78), *Cistela vittula* (p. 79), *Allecula tenuestriata* (p. 79), *Allecula fuscata* (p. 80), *Allecula lignicolor* (p. 80), *Allecula gentilis* (p. 80). Those preceded by an asterisk were redescribed in Fairmaire (1894).

#### Oechsner, Georg (Ibind, Bavaria, Germany: 15 October 1797 – 29 March 1863: Aschaffenburg, Bavaria, Germany). German educator and naturalist; professor of natural history in Aschaffenburg. References. Kittel (1863); Buttler and Klein (2000: 74–75).

**1854**. *Die Käfer der Umgegend Aschaffenburgs. Ein Beitrag zu den Lokalfaunen Bayerns. Systematisch zusammengestellt. Programm der königlichen Landwirthschafts- und Gewerbs-Schule zu Aschaffenburg zur Feier ihrer öffentlichen Schlussprüfung und Preisevertheilung im Schuljahre 1853 in 1854.* Wailandt, Aschaffenburg. iv + 48 pp. (4to) [DP: n.d. (title page), 3 September 1854 (*Neue Münch Ztg*), 8 September 1854 (*Gemein Wochen*)] GB, MDZ

This work consists of a checklist of the Coleoptera from the surroundings of Aschaffenburg, in Bavaria.

#### Oken, Lorenz (Bohlsbach near Offenburg, Baden-Württemberg, Germany: 1 August 1779 – 11 August 1851: Zürich, Switzerland). German naturalist and philosopher; professor of medicine at the University of Jena (1807–1819); professor at the University of Munich (1827–1832); appointed professor of natural history at the University of Zürich in 1833; established the journal *Isis* which lasted for 30 years (1817–1847); the whereabouts of his collection of beetles are unknown. References. Owen (1858); Ecker (1883, P); Lang (1887); Büttner (1999); Damkaer (2002: 159–163, P); Adler (2007: 40–41, P).

**1815**. *Lehrbuch der Naturgeschichte. Dritter Theil. Zoologie. Mit vierzig Kupfertafeln. Erste Abtheilung. Fleischlose Thiere.* August Schmid und Comp., Jena. xxviii + 850 + xviii + 40 pls. (8vo) [DP: 1815 (title page)] GB

The entire work consists of three volumes, 1813–1826. The Coleoptera are on pages 763–826 of the first part (*Abtheilung*) of the third volume. The work was also published the same year by the author in Jena & C.H. Reclam in Leipzig [GB]. In Opinion 417, the ICZN (1956c) ruled that volume 3 of Oken’s *Lehrbuch der Naturgeschichte* published in 1815–1816 is to be rejected for nomenclatural purposes since the author did not apply the principles of binominal nomenclature.

**1836**. *Allgemeine Naturgeschichte für alle Stände*. *Fünften Bandes dritte Abtheilung, oder Thierreich, zweiten Bandes letzte Abtheilung.* Hoffmann, Stuttgart. Pp. 1051–1845 + [1]. (8vo) [DP: 1836 (title page)] GB

The entire series was issued in *Lieferungen* and consists of seven *Bande* forming 13 volumes, 1833–1841. The Coleoptera are on pages 1629–1817 in the third part (*Abtheilung*) of the fifth volume (*Band*). Volume 5 consists in part of *Lieferung* 24 (pp. 1585–1680), recorded on 4 March 1836 (*Allg Bibl Deutsch*), and *Lieferungen* 26–27 (pp. 1681–1845), recorded on 17 June 1836 (*Allg Bibl Deutsch*). An atlas of 164 plates was also issued (Horn and Schenkling 1928c: 898). Collation and dates of publication for the entire series are desired.

**1843**. *Lehrbuch der Naturphilosophie. Dritte, neu bearbeitete Auflage.* Friedrich Schulthess, Zürich. xii + 523 pp. (8vo) [DP: 1843 (title page), August–October 1843 (*Neue Helvetia* 1: 561), 9 November 1843 (*Allg Bibl Deutsch*), 30 November 1843 (*Blätt Lit Unterhalt*), 2 December 1843 (*Lit Ztg*), 22 December 1843 (*Leip Reper*), July–December 1843 (*Verz Bücher Landkarten*)] MDZ

A classification of the Coleoptera is presented on pages 483–484; several new family-group names are proposed. The first edition of Oken’s work was issued in three volumes in 1809–1811 [GB], the second in 1831 [GB].

An English translation of this work was issued in 1847 by the Ray Society in London under the title “*Elements of physiophilosophy by Lorenz Oken, M.D. from the German by Alfred Tulk*” [GB].

#### Oliveira, Manoel [Manuel] Paulino d’ [de] (14 November 1837 – 25 August 1899). Portuguese naturalist; professor of zoology and director of the zoological museum at the University of Coimbra; his collection was acquired by the University of Coimbra. References. Silva and Aranha (1893: 283); Nobre (1901).

**1876**. *Mélanges entomologiques sur les insectes du Portugal.* Imprimerie de l’Université, Coimbre. 59 pp. (8vo) [DP: 1876 (title page), 10 January 1877 (*Soc Ent Fr*), 1 March 1877 (*Pet Nouv Ent*)] GB

This work was also published, almost integrally, in the journal *O Instituto* (Segunda Serie) 22 [1876]: 110–117, 171–177, 230–235, 293–301; 23 [1876]: 23–29, 164–171, 222–226, 268–271. One new taxon, *Carabus antiquus* v[ar]. *vieirae* (p. 173), described in the journal version is not mentioned in the separate issue.

[**1883–1896**]. *Catalogue des insectes du Portugal. Coleoptères.* Imprensa da Universidade, Coimbra. 393 pp. (8vo) [DP: n.d. (title page)] ANXL

This catalogue is often dated 1893 following Derksen and Scheiding-Göllner (1968: 251) but it was issued in parts. The first one, signatures 1–14 (pp. 1–112), was noticed in 1883^80^ (Reitter 1883: 206). I have not seen further notification until it was recorded in *Naturae Novitates* on February 1896. However, the content of the catalogue was first published in the journals *Revista da Sociedade de Instrucção do Porto* **2** [1882]: 37–44, 94–101, 147–155, 232–240, 307–316, 366–374, 416–423, 468–475, 495–502, 593–601, 669–676; **3** [1883]: 12–20, 65–72, 129–136, 164–173, 233–241, 281–288, 336–344, 406–414, 476–483, 525–532, 556–563; **4** [1884]: 39–48, 89–96, 136–143, 185–192, 233–240, 280–288, 424–432, 498–504, 529–536 and *O Instituto. Revista Scientifica e Litteraria* (Segunda Serie) **35** [1887–1888]: 366–374, 469–476, 533–541, 656–664; **36** [1887–1888]: 80–88, 203–211, 351–358, 489–494, 686–692, 752–758; **37** [1889–1890]: 433–440, 583–589, 830–839; **38** [1890–1891]: 280–286, 358–364, 522–531, 577–587, 663–667, 918–927; **41** [1893–1894]: 405–409, 448–455, 679–685. Diniz (1966: 9–10) provided the dates of publication for the different sections of the journal version.

The catalogue includes a number of new species, indicated as “mihi” or “sp.n.,” such as *Cymindis heydeni* (p. 29), *Platynus mattosi* (p. 38) and *Homalota skalitzkyi* (p. 90).

#### Olivier, Guillaume-Antoine (Arcs near Toulon, Var, France: 19 January 1756 – 1 October 1814: Lyon, Rhône, France). French physician and naturalist; practiced medicine in Montpellier until 1792; participated in a scientific and diplomatic mission to the Ottoman Empire (1793–1798) in company of Jean Guillaume Bruguière; later, professor of zoology at the veterinary school of Alfort, near Paris; his collection was sold a few years after his death but a substantial part was subsequently acquired by his grand-son Ernest Olivier; ultimately the collection was deposited in 1995 at the Muséum National d’Histoire Naturelle in Paris. References. Walckenaer (1822); Ratzeburg (1874: 389–390); Papavero (1971: 187–188); Smetana and Herman (2001: 118–119, P); Damkaer (2002 : 104–109, P); Cambefort (2006: 250–251); Adler (2012: 55–56, P).

**1789–1808**. *Entomologie, ou histoire naturelle des insectes, avec leurs caractères génériques et spécifiques, leur description, leur synonymie, et leur figure enluminée. Coléoptères.* Baudouin [vols 1–2] / Lanneau [vols 3–4] / Desray [vols 5–8], Paris. (4to) CNC, BHL, GB

*Tome premier.* xx + [433 (eight genera treated, each separately paginated)] + xix + [2 (Errata du tome I)] pp. [DP: 1789 (title page)]. The genera treated are: No. 1. Lucane. *Lucanus* (26 pp.); No. 2. Léthrus. *Lethrus* (4 pp.); No. 3. Scarabé. *Scarabaeus* (190 pp.); No. 4. Trox. *Trox* (14 pp.); No. 5. Hanneton. *Melolontha* (84 pp.); No. 6. Cétoine. *Cetonia* (92 pp.); No 7. Hexodon. *Hexodon* (4 pp.); No. 8. Escarbot. *Hister* (19 pp.).

*Tome second.* [4] + [458 (28 genera treated, each separately paginated)] + xix + [3 (Errata du tome II)] pp. [DP: 1790 (title page)]. The genera treated are: No. 9. Dermeste. *Dermestes* (16 pp.); No. 10. Nicrophore. *Nicrophorus* (8 pp.); No. 11. Bouclier. *Silpha* (22 pp.); No. 12. Nitidule. *Nitidula* (22 pp.); No. 13. Byrrhe. *Byrrhus* (10 pp.); No. 14. Anthrène. *Anthrenus* (10 pp.); No. 15. Sphéridie. *Sphaeridium* (12 pp.); No. 16. Vrillette. *Anobium* (12 pp.); No. 17. Ptine. *Ptinus* (10 pp.); No. 17 *bis.* Ptilin. *Ptilinus* (4 pp.); No. 18. Ips. *Ips* (16 pp.); No. 19. Trogossite. *Trogossita* (8 pp.); No. 20. Scaphidie. *Scaphidium* (6 pp.); No. 21. Mélyre. *Melyris* (12 pp.); No. 22. Tille. *Tillus* (4 pp.); No. 23.; Drile. *Drilus* (4 pp.); No. 24. Omalise. *Omalisus* (4 pp.); No. 25. Lymexylon. *Lymexylon* (6 pp.); No. 26. Téléphore. *Telephorus* (18 pp.); No. 27. Malachie. *Malachius* (14 pp.); No. 28. Lampyre. *Lampyris* (28 pp.); No. 29. Lycus. *Lycus* (12 pp.); No. 30. Mélasis. *Melasis* (4 pp.); No. 30. *Bis.* Cébrion. *Cebrio* (6 pp.); No. 31. Taupin. *Elater* (54 pp.); No. 32. Bupreste. *Buprestis* (96 pp.); No. 33. Cicindele. *Cicindela* (32 pp.); No. 34. Elaphre. *Elaphrus* (8 pp.)

*Tome troisième.* [1] + [520 (35 genera treated, each separately paginated)] + xxviii pp. [DP: 1795 (title page)]. The genera treated are: No. 35. Carabe. *Carabus* (116 pp.); No. 36. Scarite. *Scarites* (16 pp.); No. 37. Manticore. *Manticora* (4 pp.); No. 38. Élophore, *Elophorus* (8 pp.); No. 39. Hydrophile, *Hydrophilus* (16 pp.); No. 40. Dytique, *Dytiscus* (39 pp.); No. 41. Gyrin, *Gyrinus* (14 pp.); No. 41. (*Bis.*) Dryops, *Dryops* (4 pp.); No. 42. Staphylin, *Staphylinus* (38 pp.); No. 43. Oxypore, *Oxyporus* (4 pp.); No. 44. Pédere, *Paederus* (7 pp.); No. 44. (*bis*). Cossyphe, *Cossyphus* (4 pp.); No. 45. Meloé, *Meloe* (8 pp.); No. 46. Cantharide, *Cantharis* (20 pp.); No. 47. Mylabre, *Mylabris* (15 pp.); No. 48. Cérocome, *Cerocoma* (6 pp.); No. 49. Lagrie, *Lagria* (6 pp.); No. 50. Oedemere, *Aedemera* (16 pp.); No. 51. Notoxe, *Notoxus* (6 pp.); No. 52. Apale, *Apalus* (6 pp.); No. 53. Pyrochre, *Pyrochroa* (6 pp.); No. 53. *bis.* Horie, *Horia* (5 pp.); No. 54. Cistele, *Cistela* (13 pp.); No. 55. Diapère, *Diaperis* (8 pp.); No. 56. Opatre, *Opatrum* (12 pp.); No. 57. Tenebrion, *Tenebrio* (19 pp.); No. 57. *bis.* Serropalpe, *Serropalpus* (6 pp.); No. 58. Hélops, *Helops* (18 pp.); No. 59. Pimelie, *Pimelia* (30 pp.); No. 60. Blaps. *Blaps* (10 pp.); No. 61. Sepidie. *Sepidium* (9 pp.); No. 62. Scaure. *Scaurus* (6 pp.); No. 63. Erodie. *Erodius* (7 pp.); No. 64. Mordelle. *Mordella* (10 pp.); No. 65. Ripiphore. *Ripiphorus* (8 pp.).

*Tome quatrième.* [1] + [490 (18 genera treated, each separately paginated)] + xxi + [2 (Errata du Tome IV)] pp. [DP: 1795 (title page) – 1800]. The genera treated are: No. 66. Prione. *Prionus* (41 pp.); No. 67. Capricorne. *Cerambix* (132 pp.); No. 68. Saperde. *Saperda* (41 pp.); No. 69. Stencore. *Stenocorus* (30 pp.); No. 70. Callidie. *Callidium* (72 pp.); No. 71. Spondyle. *Spondylis* (4 pp.); No. 72. Calope. *Calopus* (4 pp.); No. 73. Lepture. *Leptura* (34 pp.); No. 74. Nécydale. *Necydalis* (10 pp.); No. 74 *bis.* Cucuje. *Cucujus* (10 pp.); No. 75. Donacie. *Donacia* (12 pp.); No. 75 *bis.* Lupère. *Luperus* (4 pp.); No. 76. Clairon. *Clerus* (18 pp.); No. 76 *bis.* Nécrobie. *Necrobia* (6 pp.); No. 77. Bostriche. *Bostrichus* (18 pp.); No. 78. Scolyte. *Scolytus* (14 pp.); No. 79. Bruche. *Bruchus* (24 pp.); No. 80. Macrocéphale. *Macrocephalus* (16 pp.). This volume is usually dated 1795, the date on the title page. However, due to a diplomatic and scientific mission of Olivier to the Ottoman Empire, livraison 23, which comprised about 3/4 of the volume (?starting at page 81 of Capricorne), was published in 1800. It was recorded on the Fructidor an VIII (= 18 August–22 September 1800) issue of the *Journal général de la Littérature de France*.

*Tome cinquième.* [1] + 612 pp. [DP: 1807 (title page)]. The genera treated are: No. 81. Attelabe. *Attelabus* (pp. 1–40); No. 82. Brachycère. *Brachycerus* (pp. 41–66); No. 83. Charanson, *Curculio* (pp. 67–428); No. 84. Brente, *Brentus* (pp. 429–444); No. 84*bis.* Cylas. *Cylas* (pp. 445–447); No. 85. Myctère, *Mycterus* (pp. 448–451); No. 86. Rhinosime, *Rhinosimus* (pp. 452–456); No. 87. Rhinomacer, *Rhinomacer* (pp. 457–460); No. 88. Langurie, *Languria* (pp. 461–464); No. 89. Érotyle, *Erotylus* (pp. 465–486); No. 89*bis.* Triplax, *Triplax* (pp. 487–493); No. 90. Sagre, *Sagra* (pp. 494–501); No. 91. Chrysomèle, *Chrysomela* (pp. 502–580); Suite du No. 91. Doryphore, *Doryphora* (pp. 581–590); No. 91*bis.* Hélode. *Helodes* (pp. 591–595); No. 92. Paropside, *Paropsis* (pp. 596–605); No. 92*bis.* Adorie, *Adorium* (pp. 606–612). Two “genera” treated, Attelabe (no. 81) and Charanson (no. 83), are subdivided into several genera.

*Tome sixième.* [1] + pp. 613–1104. [DP: 1808 (title page)]. The genera treated in this volume are: No. 93. Galeruque, *Galeruca* (pp. 613–666); No. 93*bis.* Altise, *Altica* (pp. 667–724); No. 94. Criocère, *Crioceris* (pp. 725–749); No. 94*bis.* Orsodacne, *Orsodacna* (pp. 750–755); No. 95. Hispe, *Hispa* (pp. 756–779); No. 95*bis.* Ctenode, *Ctenodes* (pp. 779–781). No. 96. Gribouri, *Cryptocephalus* (pp. 782–839); Suite du No. 96. Clytre, *Clytra* (pp. 840–872); Suite du No. 96. Chlamyde, *Chlamys* (pp. 872–877); Suite du No. 96. Colaspe, *Colaspis* (pp. 877–894); Suite du No. 96. Eumolpe, *Eumolpus* (pp. 894–916); No. 96*bis.* Mégalope, *Megalopus* (pp. 917–921); No. 97. Casside, *Cassida* (pp. 922–984); No. 98. Coccinelle, *Coccinella* (pp. 985–1061); No. 99. Eumorphe, *Eumorphus* (pp. 1062–1068); No. 100. Endomyque, *Endomychus* (pp. 1069–1074). Pages 1075–1100 are the *Explication des planches* for tome V and VI, pages 1101–1104 are “Avis au relieur, pour collationner et réunir en volumes les planches et le texte de l’Entomologie de M. Olivier.”

*Tome septième. Planches. – Genres 1 à 65.* [DP: 1808 (title page)]. The first 63 plates (No. I. Pl. Ire to No. 8. Pl. III) are associated with tome 1, the next 63 (No. 9. Pl. I. to No. 34. Pl. Ire) with tome 2, the next 65 (No. 35. Pl. Ire to No. 65. Pl. I) with tome 3. Despite that the title page is dated 1808, these plates were issued from 1789 to 1795.

*Tome huitième. Planches. – Genres 66 à 100.* [DP: 1808 (title page)]. The first 72 plates (No. 66. Pl. I to No. 80. Pl. II) are associated with tome 4, the next 59 (No. 81. Pl. I to No. 92 and 92.*bis* Pl. I) with tome 5 and the last 41 (No. 93. Pl Ire to No. 99 and 100. Pl. I.) with tome 6. Despite that the title page is dated 1808, these plates were issued from 1795 to 1808.

Tome 5 was supposed to include all pages of tomes 5 and 6 but because of the size it was divided in two volumes but the signature at the bottom of the pages of tome 6 bears “Coléoptères. Tome V”. On page 1101, the publisher reported that the entire work consists of 3,162 pages and 363 plates issued in 30 livraisons. Although the plates were issued at the same time as the text, he suggested to bind them separately in two volumes, which form *tomes septième* and *huitième*. Collation and dates of publication of each livraison are desired. Notifications for the following livraisons^81^ were seen: **1** (genera *Lucanus*, *Lethrus*, *Scarabaeus*): 15 May 1790 (*J Encycl*); **2** (*Trox*, *Melolontha*): May 1790 (*Observ Phys*); **3** (*Cetonia*, *Hexodon*, *Hister*): October 1790 (*Observ Phys*), 15 December 1790 (*J Encycl*); **4** (*Dermestes* to *Anobium*): October 1790 (*Observ Phys*); **5** (*Ptinus* to *Telephorus*): 20 May 1791 (*J Encycl*); **12**: April 1791 (*Observ Phys*); **13–14**: June 1791 (*Observ Phys*); **15–16**: April 1792 (*Observ Phys*); **17**: June 1792 (*Observ Phys*); **19**: February 1793 (*Observ Phys*); **23**: 18 August–22 September 1800 (*J Lit Fr*); **25**: (12 pls + text of volume 5) 15 October 1807 (*Télé Litt*), 16 November 1807 (*J Typogr Bibl*), 23 November 1807 (*Acad Sci Fr*); **26**: (12 pls + text of volume 5) 11 January 1808 (*Acad Sci Fr*), 15 January 1808 (*Télé Litt*), 25 January 1808 (*J Typogr Bibl*); **27**: (12 pls + text of volume 5/6) 13 June 1808 (*Acad Sci Fr*), 25 June 1808 (*Télé Litt*), 27 June 1808 (*J Typogr Bibl*); **28**: (12 pls + text of volume 5/6) 25 July 1808 (*Acad Sci Fr*), 25 October 1808 (*Télé Litt*), 21 November 1808 (*J Typogr Bibl*); **29–30**: 21 November 1808 (*Acad Sci Fr*).

**1790–1811**. *Encyclopédie méthodique, ou par ordre de matières; par une société de gens de lettres, de savans et d’artistes; précédée d’un vocabulaire universel, servant de table pour tout l’ouvrage, ornée des portraits de Mm. Diderot & d’Alembert, premiers éditeurs de l’Encyclopédie. Histoire naturelle. Insectes.* Panckoucke [vols 5–7] / Agasse [vol. 8], Paris. (4to) CNC, GB

*Tome cinquième.* 793 pp. [DP: (pp. 1–368 [livraison 41]) 17 November 1790 (*Merc Fr*); (pp. 369–793 [livraison 44]) 3 June 1791 (Evenhuis 1997a: 227–228)]. The title page is dated 1790.

*Tome sixième.* 704 pp. [DP: (pp. 1–368 [livraison 47]) 21 November 1791 (*Merc Fr*); (pp. 369–704 [livraison 51]) 1 October 1792 (*Merc Fr*)]. The title page is dated 1791.

*Tome septième.* 827 pp. [DP: (pp. 1–368 [livraison 54]) 13 May 1793 (Evenhuis 1997a: 227–228); (pp. 369–827 [livraison 61]) 9 February 1797 (*Merc Fr*)]. The title page is dated 1792.

*Tome huitième.* 722 pp. [DP: (pp. 1–360 [livraison 75]) 30 April 1811 (*Bibl Fr*), 25 May 1811 (*Merc Fr* [p. 365]); (pp. 361–722 [livraison 77])^82^ 3 July 1812 (*Bibl Fr*)]. The title page is dated 1811.

On page 45 of tome 8, a footnote stated that from the letter L (p. 443 of tome 7) to page 45 (of tome 8), the redaction of the *Encyclopedie* was confided to other persons. A number of entries, such as Libellule (p. 554) and Mouche (p. 827), are signed M [Manuel] or B.E. Manuel. None of the entries for Coleoptera are signed except for Lagrie (p. 443) which is signed “(Fab.).” According to Olivier (1874), Manuel simply copied the entries for Coleoptera from Olivier’s *Entomologie*, volumes 1–4, or from Olivier’s manuscripts. From pages 468 on of tome 8, a number of entries are signed “(Lat.)” and were written by Pierre André Latreille. The only Coleoptera entry authored by Latreille is Pambore (pp. 674–678).

The entire *Encyclopédie Méthodique*, one of the major scientific publication achievements of all time (Evenhuis 2003: 1), was published in 102 livraisons, 1782–1832. The *Histoire naturelle* section consisted of 14 volumes of text, comprising 13,671 pages, and 15 volumes of plates, comprising 1,971 plates (Evenhuis 2003: 14). The *insectes* section covers volumes 4–10. Separates of some of the volumes were also published simultaneously under the title “*Dictionnaire encyclopédique de l’histoire naturelle*.”

#### Olliff, Arthur Sidney (Millbrook, Hampshire, United Kingdom: 21 October 1865 – 29 December 1895: Sidney, Australia). Australian (British-born) entomologist; curator and private secretary to Lord Walsingham (1883–1885); moved to Australia and became assistant entomologist at the Australian Museum in Sydney (1885–1890); later, appointed government entomologist at the Agricultural Department of New South Wales (1890–1895); part of his collection is at the Australian Museum in Sydney and at the British Museum (private collection of non-Australian Coleoptera) in London. References. Guthrie (1896); Zimmerman (1993: 713); Smetana and Herman (2001: 119–120, P); Daniels (2004).

**1892**. Coleoptera – (Continued). Pp. 58–81 *in*: *Supplementary appendix to travels amongst the Great Andes of the Equator by Edward Whymper with contributions by H.W. Bates. T.G. Bonney. G.A. Boulenger. Peter Cameron. F. Day. W.L. Distant. A.E. Eaton, F.D. Godman. H.S. Gorham. Martin Jacoby. E.J. Miers. A. Sidney Ollif. O. Salvin. David Sharp. T.R.R. Stebbing. Illustrated.* John Murray, London. xxii + [1 (Addenda)] + 147 pp. + 14 pls. (4to) [DP: 1891 (title page), 2 March 1892 (Sharp 1892: 61), 18 March 1892 (*Science*), 26 March 1892 (*Athenaeum*), 9 April 1892 (*Publ Circ*), April 1892 (*Nat Nov*), 16 June 1892 (*Nature*), 13 July 1892 (*Soc Ent Fr*)] CNC, GB

According to Sharp (1892: 61), the printing was done in 1891 but the work was not available for purchase until 2 March 1892.

#### Ormay, Sándor (Dolný Kubin, Slovakia: 2 April 1855 – 27 March 1938: Budapest, Hungary). Hungarian educator and coleopterist; professor at a gymnasium in Sibiu, currently in Romania. Reference. Kaszab (1939).

**1888**. *Adatok Erdély bogárfaunájához. Supplementa faunae coleopterorum in Transsilvania.* Adolf Reissenberger, Nagy-Szeben [= Sibiu]. [2] + 54 pp. (8vo) [DP: 1888 (title page), 13 July 1888 (*Sieben Ver*), 12 October 1888 (*Wien Ent Ztg* 7: 276), November 1888 (*Term Közl*)] MCZ

The text is written in Hungarian and Latin. Four new taxa are described: *Pterostichus etelkae* (p. 13), *Alexia reitteri* (p. 27), *Meleus elekesi* (p. 40), and *Pidonia lurida* v. *gangelbaueri* (p. 45).

**1890**. *Ujabb adatok Erdély bogárfaunájához. Recentiora supplementa faunae coleopterorum in Transsilvania.* Rudnyánszky A. Könyvnyomdája, Budapest. 65 pp. + 1 pl. (8vo) [DP: 1890 (title page; p. 9 dated June 1890), 15 October–15 November 1890 (*Leopoldina* 26: 194), 27 November 1890 (*R Soc Lond*), December 1890 (*Term Közl*)] MCZ

The text is written in Hungarian and Latin.

#### Ormerod, Eleanor Anne (Sedbury Park, Gloucestershire, United Kingdom: 11 May 1828 – 19 July 1901: St. Albans, Hertfordshire, United Kingdom). British economic entomologist of independent means. References. Wallace (1904, P); Le-May Sheffield (2000); Clark (2004b, P).

**1889**. *Notes and descriptions of a few injurious farm & fruit insects of South Africa. With descriptions and identifications of the insects by Oliver E. Janson, F.E.S.* Simpkin, Marshall & Co., London. viii + 116 pp. (8vo) [DP: 1889 (title page; preface dated May 1889), 4 July 1889 (*Cultiv Count Gent*), July 1889 (*Nat Nov*; *Entomologist* 22: 192), 7 August 1889 (*Bookseller*), 22 August 1889 (*Nature*), 30 September 1889 (*Torch Col Book Circ*)] GB

The Coleoptera are on pages 1–37. One new beetle species is described, *Iphidea capensis* Baly (p. 34); the species is attributable to Joseph Sugar Baly [*q.v.*] who provided a description inserted in the book.

#### Osculati, Gaetano (San Giorgio al Lambro near Biassono, Lombardy, Italy: 25 October 1808 – 14 March 1894: Milan, Lombardy, Italy). Italian naturalist and explorer of independent means; travelled to Middle East (1830–31, 1841–42) and to America (1834–36, 1846–48). References. Calidoni (1924, P); Papavero (1973: 343–347); Conci and Poggi (1996: 298, P).

**1844**. *Note d’un viaggio nella Persia e nelle Indie orientali negli anni 1841, 1842. Edizione fuori di commercio.* Luca Corbetta, Monza. 72 pp. + 1 pl. + [1 (errata)] pp. (8vo) [DP: 1844 (title page; dedication dated 20 July 1844), 19 September 1844 (Anonymous 1844: 102), 19 October 1844 [JD] (*Soc Imp Nat Moscou*), 12 April 1845 (*Lit Ztg*)] CMLE (mf), GB

Page 57 is entitled “*Coleopterorum enumeratio quae ad Persiam et Indias orientales itinere a Cajetano Osculati collecta novarum specierum descriptionibus adjectis*” and dated August 1844. Page 72 is entitled “*Coleopterorum species novae quae in ista enumeratione commemoratae sunt disgnosibus atque observationibus illustrate*” and contains the descriptions of the following eight species: *Carabus osculati* Villa; *Carabus orientalis* Osculati; *Sphodrus armeniacus* Osculati; *Lithophilus osculati* Marietti; *Anisoplia mariettii* Osculati; *Adesmia villae* Osculati; *Adesmia de vecchii* Osculati; *Adimonia orientalis* Villa.

The cover of this work is entitled “*Coleopteri raccolti nella Persia, Indostan ed Egitto e note del viaggio*.”

**1850**. *Esplorazione delle regioni equatoriali lungo il Napo ed il fiume delle Amazzoni: frammento di un viaggio fatto nelle due Americhe negli anni 1846–1847–1848. Corredata di 2 carte topografiche e di 20 vedute e costumi ritratti dal vero dallo stesso autore.* Bernardoni, Milano. 320 pp. + 11 pls. (8vo) [DP: 1850 (title page; *prefazione* dated 1 June 1850), 18 October 1850 (*Soc Géog Fr*)] AMNH, GB

One new genus, *Boeoscelis*, and one new species, *Boeoscelis osculati*, are described from the river Napo on page 202 by Spinola. Several new species of Coleoptera and Hymenoptera are mentioned in the tables on page 201 but none are described. The text relating to the tables and the descriptions of the new genus and species were reissued by Spinola (1864).

A second edition of this work, containing 344 pages and 13 plates, was issued in 1854, also in Milan [GB, MDZ]. A two-volume annotated edition, edited by Gerolamo Bottoni, was published in Milan, 1929 (*n.v.*).

#### Oudemans, Johannes Theodorus (Amsterdam, Netherlands: 22 November 1862 – 20 February 1934: Amsterdam, Netherlands). Dutch entomologist and dendrologist; lecturer in zoology at the University of Amsterdam; later, estate manager. References. Weber (1932, P); Meijere (1934, P).

**1896–1900**. *De nederlandsche insecten. Met 38 steendrukplaten en 427 figuren in den tekst.* Martinus Nijhoff, ’s Gravenhage. xv + 836 pp. + 38 pls. (8vo) GAL

This work was issued in 15 parts. The following table gives the dates of publication provided by MacGillavry (1939), taken from the original wrappers, and those found in *Naturae Novitates*.

As discussed by MacGillavry (1939), it appears that the publication dates on some of the wrappers are incorrect. The Coleoptera are on pages 597–712.

**Table:** 

Part	Content	MacGillavry	*Naturae Novitates*
1	(pp. 1–48, pls 1, 11, 12)	September 1896	October 1896
2	(pp. 49–96, pls 2–4)	1896	January 1897
3	(pp. 97–144, pls 5, 6, 13)	1896	February 1897
4	(pp. 145–192, pls 14–16)	1896 (recte 1897?)	August 1897
5	(pp. 193–240, pls 7–9)	1896 (recte 1897?)	August 1897
6	(pp. 241–288, pls 10, 17, 18)	1897	December 1897
7	(pp. 289–336, pls 19, 33, 35)	1897	May 1898
8	(pp. 337–384, pls 36–38)	1897 (recte 1898?)	November 1898
9	(pp. 385–432, pls 20–22)	1897 (corrected to 1898)	November 1898
10	(pp. 433–480, pls 23, 24, 34)	1897 (recte 1898?)	May 1899
11	(pp. 481–528, pls 25–27)	1899	May 1899
12	(pp. 529–576, pls 28–30)	1899	October 1899
13	(pp. 577–640, pls 31–32)	1899	October 1899
14	(pp. 641–736)	1899	September 1900
15	(pp. 737–836, i–xv, new pls 15 & 16)	1899 (recte 1900?)	September 1900

#### Paget, Charles John (Great Yarmouth, Norfolk, United Kingdom: 24 January 1811 – 12 April 1844: Great Yarmouth, United Kingdom). British naturalist; brother of Sir James Paget [1814–1899], surgeon and pathologist, best known for his studies of the progressive bone disorder known as Paget’s Disease. Reference. Palmer (1895: 74–76).

**1834**. [Paget, C.J. and Paget, J.] *Sketch of the natural history of Yarmouth and its neighbourhood containing catalogues of the species of animals, birds, reptiles, fish, insects, and plants, at present known.* F. Skill, Yarmouth. xxxii + 88 pp. (8vo) [DP: 1834 (title page; introduction dated 11 October 1834), October 1834 (Peddie and Waddington 1914: 429)] GB

The work includes a list of animals of Great Yarmouth in England with short annotations; the Coleoptera are on pages 19–31.

#### Palisot de Beauvois, Ambroise-Marie-François-Joseph (Arras, Pas-de-Calais, France: 27 July 1752 – 21 January 1820: Paris, France). French naturalist and traveler; studied law and worked as lawyer at the parliament in Paris for some times; explored the kingdoms of Oware and Benin in western Africa, Haiti and United States of America (1786–1798); Knight of the Legion of Honour; a large part of his collections were lost but some specimens were bought by Pierre August Dejean [*q.v.*]. References. Silvestre (1820); Thiébaut-de-Berneaud (1821, P); Depping (1822); Merrill (1936, P); Adler (2007: 37, P).

**1805–1821**. *Insectes recueillis en Afrique et en Amérique, dans les royaumes d’Oware et de Benin, à Saint-Domingue et dans les États-Unis, pendant les années 1786–1797.* Lavrenet, Schoell et C^ie^, Paris. xvi + 276 pp. + 90 pls. (Folio) CNC

This work was published in 15 livraisons. It includes 90 plates of which 29 are on Coleoptera and separately numbered. Notices of publication of the livraisons were found as follows: **1**: (pp. i–xvi + 1–24, pls 1, 7 of the Coleoptera) 14 October 1805 (*Acad Sci Fr*), December 1805 (Evenhuis 1997b: 588), 1 January 1806 (*Rev Phil*); **2**: (pp. 25–40, pls 3, 5) December 1805 (Evenhuis 1997b: 588), 6 January 1806 (*Acad Sci Fr*); **3**: (pp. 41–56, pls 1b, 11) May 1806 (Evenhuis 1997b: 588), 15 September 1806 (*Acad Sci Fr*); **4**: (pp. 57–72, pl. 4) 15 June 1807 (*Acad Sci Fr*); **5**: (pp. 73–88, pls 2, 8) September 1807 (Evenhuis 1997b: 588); **6**: (pp. 89–100, pls 2b, 3b) 3 July 1809 (*Acad Sci Fr*); **7**: (pp. 101–120, pls 3c, 15) 26 August 1811 (*Acad sci Fr*); **8**: (pp. 121–136, pls 30, 32) 14 December 1812^83^ (*Acad sci Fr*); **9**: (pp. 137–156, pls 1c, 30b) 26 August 1817 (*Acad Sci Fr*); **10**: (pp. 157–172, pls 6, 31) 27 October 1817 (*Acad Sci Fr*); **11**: (pp. 173–192, pls 4b, 6b)^84^ 9 November 1818 (*Acad Sci Fr*); **12**: (pp. 193–208, pls 1d, 3d) 1818 (Menke 1963: 702); **13**: (pp. 209–224, pls 1e, 9, 34) 1819 (Menke 1963: 702), 17 January 1820 (*Acad Sci Fr*); **14**: (pp. 225–240, pl. 35) 1820 (Menke 1963: 702); **15**: (pp. 241–276, pls 36, 37) 1821 (Menke 1963: 702). The last livraison was brought out by J.G. Audinet-Serville. A list of the new Coleoptera taxa proposed in this work is included in Appendix 4.

A list of the Coleoptera described in this work, with their respective genera and synonymy, was published by Chevrolat (1853).

#### Pallas, Peter Simon (Berlin, Germany: 22 September 1741 – 8 September 1811: Berlin, Germany). German naturalist and explorer; after studying medicine, settled in The Hague; joined the Academy of sciences in Saint Petersburg in 1767; made a six-year expedition across Russia to beyond Lake Baikal (1768–1774) and an expedition to the Crimea (1793–1794); later, moved to Crimea (1795–1810) and finally back to Berlin (1810–1811); part of his Coleoptera collection is at the Zoologisches Museum der Humboldt-Universität in Berlin. References. Cuvier (1819: 197–156); Smith (1843: 17–76, P); Mearns and Mearns (1988: 288–297, P); Wendland (1992); Adler (2007: 26–28, P); Vermeulen (2013, P).

**1771–1776**. *Reise durch verschiedene Provinzen des russischen Reichs.* Kayserlichen Academie der Wissenschaften, St. Petersburg. (4to) McG, GAL

*Erster Theil.* [10] + 504 pp. + pls 1–8a, 8b, 9–10a, 10b, 11 + D–I, K–O. [DP: 1771 (title page; *Vorrede* dated 28 April 1770), 3 February 1772 (*Jena Ztg*), 2 May 1872 (*Erlang Anmer Nachr*), 27 June 1772 (*Gött Anz*)]. The following species of Coleoptera are described: *Scarabaeus polyceros* (p. 461), *Scarabaeus cephalotes* (p. 461), *Scarabaeus bidens* (p. 461), *Scarabaeus humerosus* (p. 462), *Scarabaeus oxypterus* (p. 462), *Scarabaeus albellus* (p. 462), *Scarabaeus vertumnus* (p. 462), *Chrysomela adonidis* (p. 463), *Chrysomela asiatica* (p. 463), *Curculio nomas* (p. 463), *Curculio candidatus* (p. 463), *Curculio pictus* (p. 463), *Curculio piceus* (p. 464), *Curculio crucifer* (p. 464), *Curculio inderiensis* (p. 464), *Buprestis variolaris* (p. 464), *Buprestis tatarica* (p. 464), *Carabus marginatus* (p. 464), *Carabus pictus* (p. 465), *Cicindela lacteola* (p. 465), *Cicindela atrata* (p. 465), *Cerambyx carinatus* (p. 465), *Attelabus polymorphus* (p. 465), *Attelabus bimaculatus* (p. 466), *Meloe erythrocephalus* (p. 466).

*Zweyter Theil. Erstes Buch vom Jahr 1770.* [4] + 368 pp. + pls 1–8, 11. [DP: 1773 (title page; *Vorrede* dated 19 April 1772)].

*Zweyter Theil. Zweytes Buch vom Jahr 1771.* Pp. 371–744 + pls 9–10, 12–14 + A–H, J–T, T*, U, W–Z. [DP: 1773 (title page), 18 March 1775 (*Erlang Anmer Nachr*), 1 July 1775 (*Gött Anz*)]. The following species of Coleoptera are described: *Scarabaeus albus* (p. 718), *Scarabaeus spireae* (p. 719), *Tenebrio leucogrammus* (p. 719), *Tenebrio buprestoides* (p. 719), *Buprestis aurata* (p. 719), *Buprestis picta* (p. 719); *Meloe fenestrata* (p. 720), *Meloe quadrimaculata* (p. 720), *Meloe necydalea* (p. 720), *Meloe sibirica* (p. 720), *Meloe* an *algira* ? Lin., *Meloe trifascis* (p. 721), *Meloe ocellata* (p. 721), *Meloe festiva* (p. 721), *Meloe lutea* (p. 722), *Meloe atrata* (p. 722), *Meloe vralensis* (p. 722), *Curculio ireos* (p. 722), *Attelabus senex* (p. 723), *Cerambyx hieroglyphicus* (p. 723), *Cerambyx perforatus* (p. 723), *Cerambyx glicyrrhizae* (p. 723), *Cerambyx halodendri* (p. 724), *Cerambyx floralis* (p. 724), *Leptura violacea* (p. 724), *Cicindela coerulea* (p. 724), *Cicindela gracilis* (p. 724), *Chrysomela longimana* (p. 725), *Chrysomela atraphaxidis* (p. 725), *Chrysomela asclepiadea* (p. 725), *Chrysomela absinthii* (p. 725), *Coccinella axyridis* (p. 726), *Coccinella ocellata* (p. 726), *Coccinella cimicifugae* (p. 726), *Staphylinus tataricus* (p. 726).

*Dritten Theils vom Jahr 1772 und 1773.* [20] + 454 pp. + pls 1–7. [DP: 1776 (title page; *Vorrede* dated 10 February 1776), 21 April 1776 (Evenhuis 1997b: 594)].

*Dritten Theils. Zweytes Buch.* Pp. 457–760 + [32 (Indexes, Errata)] + pls A–G, G*, H–I, K–Z, Aa–Gg, Gg*, Hh–Ii, Kk–Nn. [DP: 1776 (title page), 5 August 1776 (*Wöchentl Nachr* 4: 260)]. The following species of Coleoptera are described: *Scarabaeus ammon* (p. 707), *Tenebrio echinatus* (p. 707), *Carabus bucida* (p. 707), *Buprestis cariosa* (p. 708).

Sherborn (1902: xli) mentioned a second edition of the first volume published in Saint Petersburg, 1801 (*n.v.*). An abridged edition was published in three volumes and an atlas, 1776–1778, by Johann Georg Fleischer in Frankfurt and Leipzig under the title “*Reise durch verschiedene provinzen des russischen Reichs in einem ausführlichen Auszuge*” [GB]. An amended French translation was published in five volumes (4to) and one atlas (Folio), 1788–1793, in Paris by Lagrange under the title “*Voyages de M.P.S. Pallas, en differentes provinces de l’empire de Russie, et dans l’Asie septentrionale; traduits de l’Allemand, par M. Gauthier de la Peyronie* [GB]; a second French edition was published also in Paris in eight volumes^85^ and one atlas, in “An II” of the French Republican Calendar, under the title “*Voyages du Professeur Pallas, dans plusieurs provinces de l’empire de Russie et dans l’Asie septentrionale; traduits de l’allemand par le C. Gauthier de La Peyronie. Nouvelle édition, revue et enrichie de notes par les CC. Lamarck et Langlès*” [GB]. A Russian translation was published in three volumes, 1773–1788, in St. Petersburg, under the title “*Putešestvija po raznym’ provincijam Rossijskoj imperii*” (*n.v.*); a second edition in two volumes was issued in 1809–1820 (Wendland 1992: 942). An emended Italian translation was published in five volumes in 1816 in Milano under the title “*Viaggi del Signor Pallas in diverse province dell’Imperio Russo sino ai confini della China compendiati dal Cav. Compagnoni*” [GB]. Vermeulen (2013: 64) mentioned an English translation issued in 1788–1789 but this could not be confirmed in this study. A facsimile of the original edition was issued in Graz, in 1967.

**1772**. *Spicilegia zoologica quibus novae imprimis et obscurae animalium species iconibus, descriptionibus atque commentariis illustrantur. Fasciculus nonus.* Gottl. August. Lange, Berolini [= Berlin]. 86 pp. + 5 pls. (4to) [DP: 1772 (title page), 5 October 1772 (*Jena Ztg*), 26 November 1772 (*Hall Ztg*), 12 December 1772 (*Erlang Anmer Nachr*)] MCZ, ARC, GB

The entire work consists of 14 fascicles, 1767–1780. In fascicle 9, the following Coleoptera are described: *Cassida perforata* (p. 3–4), *Leptura plumipes* (pp. 4–5), *Ligniperda terebrans* (pp. 6–7), and *Ligniperda cornuta* (p. 8). The first 10 fascicles are usually bound together with the title page reading “*Spicilegia zoologica. Tome I. Continens quadrupedium, avium, amphibiorum, piscium, insectorum, molluscorum aliorumque marinorum fasciculos decem*” and dated 1774.

A German translation of the first 11 fascicles was published, 1769–1779, in Berlin and Stralsund, under the title “*Naturgeschichte merkwürdiger Thiere, in welcher vornehmlich neue und unbekannte Thierarten durch Kupferstiche, Beschreibungen und Erklärungen erläutert werden.*” [GB]. A Dutch translation of the first six fascicles was issued in Utrecht, 1767–1770, under the title “*Dierkundig mengelwerk, in het welke de nieuwe of nog duistere zoorten van dieren door naauwkeurige afbeeldingen, beschryvingen en verhandelingen opgehelderd worden*.” [GB]. According to Zaunick (1925), the six fascicles of the Dutch edition were reissued in Amsterdam, 1779, with a new title page.

**1781–[1806**]. *Icones insectorvm praesertim Rossiae Sibiriaeqve pecvliarivm qvae collegit et descriptionibvs illvstravit.* Wolfgang Walther, Erlangae. (4to) CMLE (mf), BHL, GB (parts 1–2)

This work was published in parts as follows: ([6] + pp. 1–56, pls A–C) 1781 (title page; *praemonenda* dated August 1779), before 29 November 1781 (Evenhuis 1997b: 595); (pp. 57–96, pls D–F) 1782 (Sherborn 1891: 236), June 1782 (*Allg Verz Bücher*); (pp. 97–104, pls G–H) 1798 (Sherborn 1891: 236); (pls I and K) ?1806 (Hagen 1863: 26). Hagen (1858: 136) mentioned that plates I and K were published but Sherborn (1891: 236) had doubt (see Evenhuis 1997b: 595–596).

#### Palliardi, Anton Alois (Prague, Czech Republic: 19 November 1799 – 23 November 1873: Františkovy Lázně, Czech Republic). Bohemian physician and naturalist in Františkovy Lázně. Reference. Anonymous (1874a).

**1825**. *Dissertatio inauguralis physiographica sistens descriptiones decadum duarum Carabicorum novorum et minus cognitorum. Quam consensu et auctoritate Illustrissimi ac Magnifici Domini Praesidis et Directoris, Perillustris ac Spectabilis Domini Decani nec non Clarissimorum Dominorum Professorum pro doctoris medici laurea rite obtinenda in antiquissima ac celeberrima Universitate Vindobonensi publicae disquisitioni submittit Antonius Aloysius Palliardi Bohemus Pragensis*. *In theses adnexas disputabitur in Universitate palatio die mensis Junii MDCCCXXV.* Ferdinandi Ullrich, Vindobonae [= Vienna]. [2] + x + 44 pp. (8vo) [DP: June 1825 (title page)] GB

The thesis was also issued the same year with the addition of four plates by the publisher J.G. Heubner in Wien under the title “*Beschreibung zweyer Decaden neuer und wenig bekannter Carabicinen. Mit vier Kupfertafeln.*” [CMLE]. The pamphlet was noticed on 20 August 1825 (*Intell Ztg Welt*), 16 November 1825 (*Allg Anz*), and November 1825 (*Allg Lit Ztg*).

#### Panzer, Georg Wolfgang Franz (Etzelwang, Bavaria, Germany: 31 May 1755 – 28 June 1829: Hersbruck, Bavaria, Germany). German physician, botanist, and entomologist; practiced medicine at Hersbruck, near Nuremberg. References. Anonymous (1831); Spiess (1891: 196–199); Eisinger (1919a, P); Weidner (1983: 308–309, P).

**1785–1788**. *Drury’s Abbildungen und Beschreibungen exotischer Insekten, mit sein illuminirten Kupfertafeln. Aus dem Englischen übersezt und mit vollständiger Synonymie und erläuternden Bemerkungen.* Adam Wolfgang Winterschmidt, Nürnberg. 203 + [5] pp. + 50 pls. (4to) NCSU, GB

This work is an emended German translation of Drury’s “*Illustrations of natural history*” [*q.v.*]. It was published in four *Hefte*: **1**: (pp. 1–48 + pls 1–6) 1785 (title page), 22 August 1785 (*Jena Ztg*), 15 December 1785 (*Allg Lit Ztg*), 29 December 1785 (Evenhuis 1997b: 599); **2**: (pp. 49–104 + pls 7–28) 1786^86^ (Evenhuis 1997b: 599); **3**: (pp. 105–152 + pls 29–40) 1787 (Evenhuis 1997b: 599), 30 November 1789 (*Allg Lit Ztg*); **4**: (pp. 153–203 + pls 41–50) 1788 (Evenhuis 1997b: 599), 30 November 1789 (*Allg Lit Ztg*).

**1785–1802**. *Johann Euseb Voets Beschreibungen und Abbildungen hartschaaligter Insecten. Coleoptera Linn. Aus dem Original getreu übersetzt mit der in selbigem fehlenden Synonymie und beständigen Commentar versehen von Dr. Georg Wolfgang Franz Panzer.* Valentin Bischoff, Nürnberg [vols 1–2] / Johann Jacob Palm, Erlangen [vols 3–5]. (4to) BHL

*Erster Theil. Mit drey und zwanzig Kupfertafeln.* xii + 103 pp. + pls 1–22. [DP: 1785 (title page; *Vorbericht* dated 31 May 1785), 26 August 1785 (*Jena Ztg*), 13 December 1785 (*Allg Lit Ztg*)]. A second printing, with the same title except that it ends “*Erster Theil. Enthaltend Tab. I–22. nebst Tittelkupfer*”, was issued in 1793 by J.J. Palm in Erlangen [CMLE, GB].

*Zweeter Theil. Mit sieben und zwanzig Kupfertafeln.* [4] + 134 pp. + pls 23–48. [DP: 1791 (title page; *Vorbericht* dated 14 January 1791), 7 February 1792 (*Allg Lit Ztg*). A second printing, with the same title except that it ends “*Zweyter Theil. Enthaltend Tab. 23–48. nebst Tittelkupfer*”, was issued in 1793 by J.J. Palm in Erlangen [CMLE, BHL].

*Dritter Theil. Mit fünf und zwanzig Kupfertafeln.* [4] + 68 pp. + pls 1–24. [DP: 1794 (title page; *Vorbericht* dated 27 December 1793), 18 January 1795 (*Intell Allg Lit Ztg*)]. From this volume, “Wolfgang” on the title page is spelled “Wolffgang.”

*Vierter Theil. Mit fünf und zwanzig Kupfertafeln.* [14] + 120 [last one numbered 112] pp. + pls 25–48. [DP: 1798 (title page; *Vorbericht* dated 10 November 1797)].

*Fünfter Theil. Mit zwölf Kupfertafeln.* 114 + [2] pp. + pls 1–12. [DP: 1802 (title page; last page dated 16 August 1802), 30 December 1803 (*Allg Lit Ztg*)]. Other title pages of this *Theil* read “*Beyträge zur Geschichte der Insekten. Mit zwölf Kupfertafeln*”, “*Ioannis Evsebii Voetii Icones et descriptiones Coleopterorum. Tomvs qvintvs. Cvm tabvlis XII aeneis*” and “*Symbolae entomologicae. Cvm tabvlis XII aeneis*.”

According to Horn and Schenkling (1928c: 912), *Theile* 1 and 2 of this work were first issued in 1782 and 1783. In fact Panzer published in Nürnberg a work in parts under the title “*Voets Käferwerk, übersetzt und mit einigen Anmerkungen begleitet von Georg Wolfgang Franz Panzer*” (*n.v.*, but see Beckmann 1783: 335–336) for which the following notifications only were found during this study. **Vol. 1**: (signatures A–B + pls 1–3) 17 May 1782 (*Jena Ztg*), 23 November 1782 (*Gött Anz*); (signatures C–D + pls 4–6) 21 January 1783 (*Nürnb Ztg*), 24 February 1783 (*Gött Anz*); (signatures E–H + pls 7–12) 1784 (Beckmann 1784: 405), 23 July 1784 (*Jena Ztg*); (signatures I–M + pls 13–22) 15 August 1785 (*Gött Anz*); **Vol. 2**: (signatures A–H + pls 23–37) 29 October 1790 (*Allg Lit Ztg*).

As indicated by Sherborn (1902: xli), the first three parts of this work consist of a translation of “Voet’s ed.I” (i.e., work reissued in 1806 under the title *Catalogus systematicus coleopterorum*… [*q.v.*]) and the last two parts form a new work by Panzer. As for the original text, the translation is not consistently binominal but the new parts are consistent with the Principle of Binominal Nomenclature; several new species are described in the fourth *Theil* and one (*Geotrupes iphiclus*, p. 85) in the fifth *Theil*.

**1792–1810**. *Faunae insectorum Germanicae initia. Deutschlands Insecten.* Felsecker, Nürnberg. (Oblong 16mo) CNC, BHL

The entire series consists of 190 *Hefte*, each containing 24 small, loose, colored plates and one or two pages of text for each plate. The first 109 *Hefte*, 1792–1810, were authored by Panzer, *Heft* 110, issued in 1823, by Carl Geyer [*q.v.*], and *Hefte* 111–190, issued 1829–1844, by G.A.W. Herrich-Schäffer [*q.v.*]. The dates of publication of each *Heft*, as supplied by Sherborn (1923: 566–567) and Evenhuis (1997b: 600) unless otherwise noted, are as follows: **Heft 1**: 1 September 1792; **2**: October 1792; **3**: November 1792; **4**: December 1792; **5**: January 1793; **6**: February 1793; **7**: March 1793; **8**: April 1793; **9**: May 1793; **10**: June 1793; **11**: July 1793; **12**: August 1793; **13**: September 1793; **14**: October 1793; **15**: November 1793; **16**: December 1793; **17**: January 1794; **18**: February 1794; **19**: March 1794; **20**: April 1794; **21**: 3 May 1794 (*Intell Allg Lit Ztg*), May 1794; **22**: June 1794; **23**: July 1794; **24**: 21 August 1794; **25**: 16 April 1795; **26**: 21 September 1795; **27–28**: after 21 September 1795; **29–35**: before 29 March 1796; **36**: 29 March 1796, 16 April 1796 (*Intell Allg Lit Ztg*); **37–38**: after March 1796; **39–40**: before 22 September 1796; **41**: 22 September 1796 (Hagen 1863: 203); **42**: before 9 October 1797; **43–49**: 9 October 1797; **50–59**: before 1 May 1798; **60**: 1 May 1798; **61–70**: before June 1799; **71**: June 1799; **72–73**: after June 1799; **74–79**: June 1800; **80–83**: after June 1800; **84**: 17 June 1801; **85**: 26 December 1801; **86–90**: March 1802 (*Monatl Anz*); **91–94**: 17 May 1803; **95–96**: before October 1804; **97–99**: 1805; **100**: January–June 1806 (*Verz Bücher Landkarten*); **101–104**: 1806; **105–107**: January–June 1808 (*Verz Bücher Landkarten*); **108**: 1809, July–December 1809 (*Verz Bücher Landkarten*), 2 April 1810 (*Intell Jena Lit Ztg*); **109**: 1810, January–June 1810 (*Verz Bücher Landkarten*), 1813 (Hagen 1863: 27).

No title pages for the individual *Hefte* were seen during this study except for some posted online. For example, the title page of the first *Heft* reads “*Faunae insectorum Germanicae initia. Deutschlands Insecten herausgegeben von Dr. G.W.F. Panzer. Ites Heft. Nürnberg in der Felseckerschen Buchhandlung.*” There was no date on the title pages available.

As discussed by Saunders (1888) there are two editions of this work and some of the names differ between the two and new names have been issued in the second edition. Saunders mentioned that he could trace the second edition up to *Heft* 72 but that changes in the text and illustrations were made only up to *Heft* 37.

Panzer issued an index after each 12 *Hefte*, 1793–1809 [*q.v.*]. He also supplied in 1813 [*q.v.*] an index to the Coleoptera only. Finally, an index to the entire 109 *Hefte* of both editions was published by Saunders (1888).

**1793–1809**. *Favnae insectorvm Germanicae initia oder Devtschlands Insecten.* Felsecker, Nürnberg. (Oblong 16mo) CNC

*Erster Iahrgang. I–XII. Heft.* xvi pp. [DP: 1793 (title page; *Vorbericht* dated 21 August 1793)].

*Zweiter Iahrgang. XIII–XXIV. Heft.* xvi pp. [DP: 1794 (title page; *Vorbericht* dated 21 August 1794)].

*Dritter Iahrgang. XXV–XXXVI. Heft.* xiv pp. [DP: 1796 (title page; *Vorbericht* dated 29 March 1796)].

*Vierter Iahrgang. XXXVII–XLVIII. Heft.* xiv pp. [DP: 1797 (title page)].

*Fünfter Iahrgang. XLIX–LX. Heft.* xiv pp. [DP: 1798 (title page; *Vorbericht* dated 1 May 1798)].

*Sechster Iahrgang. LXI–LXXII. Heft.* xiii + [1 (errata)] pp. [DP: 1799 (title page)].

*Siebenter Iahrgang. LXXIII–LXXXIV. Heft.* xiii pp. [DP: 1801 (title page; *Vorbericht* dated 3 September 1801)].

*Achter Iahrgang. LXXXIV* [sic!]–*XCVI. Heft.* xv pp. [DP: 1805 (title page)].

*Neunter Iahrgang. XCVII–CVIII. Heft.* 13 pp. [DP: 1809 (title page)].

This is a systematic index, following Fabricius’ method, of the species included in the first 108 *Hefte* of Panzer’s “*Favnae insectorvm Germanicae*.” An index to the second edition of Panzer’s work was issued for the first 24 *Hefte*, divided into two *Iahrgang*; the date of publication of the first year is 1796 and for the second one 1799 (see Saunders 1888: [2]).

**1794**. *Faunae insectorum Americes borealis prodromus. Cum tab. aeneis.* Felsecker, Norimbergae [= Nürnberg]. 8 pp. + 1 pl. (4to) [DP: 1794 (title page)] MCZ, UFM

This small work contains the descriptions of several species of the genus *Scarabaeus*. The single plate depicts three species of *Scarabaeus* which are described and named in the text and three tenebrionids that are not named nor described.

**1795**. *Deutschlands Insectenfaune oder entomologisches Taschenbuch für das Iahr 1795*. Felsecker, Nürnberg. [32] + 370 + [2] pp. + 12 pls. (8vo) [DP: n.d. (title page; *Vorbericht* dated 19 December 1794), 14 February 1795 (*Intell Allg Lit Ztg*), 1 June 1795 (*Ober Lit*), 11 June 1795 (*Gött Anz*), 21 September 1795 (*Allg Lit Ztg*)] CMLE, GB, ARC

There is an additional title page which reads “*Entomologia Germanica exhibens insecta per Germaniam indigena secvndvm classes, ordines, genera, species adiectis synonymis, locis, observationibvs. I. Elevterata. Cvm tabvlis aeneis.*”

**1804**. *Systematische Nomenclatur über weiland Herrn Dr. Jacob Christian Schäffers natürlich ausgemahlte Abbildungen regensburgischer Insekten*. Johann Jakob Palm, Erlangen. xvi + 260 pp. (4to) [DP: 1804 (title page; *lectori* dated February 1804), 20 November 1804 (*Ober Lit*)] GB, ARC, MDZ

Another title page reads “*D. Jacobi Christiani Schaefferi Iconvm insectorvm circa Ratisbonam indigenorvm envmeratio systematica opera et stvdio*.”

This work provides specific names for the unnamed insects illustrated on each of the 280 plates of Schäffer’s “*Icones insectorvm circa Ratisbonam indigenorvm coloribvs natvram referentibvs expressae*,” 1766–1769 [*q.v.*]. Many new species are described, including the beetle *Necydalis flavimana* (p. 105).

**1805**. *Kritische Revision der Insektenfaune Deutschlands nach dem system. I – XCVI. Heft. I. Baendchen.* Felssecker, Nürnberg. [12] + 144 pp. (8vo) [DP: 1805 (title page; *Vorbericht* dated 11 April 1805), January–June 1805 (*Verz Bücher Landkarten*)] CNC, GB, BHL

The entire work consists of two volumes, 1805–1806. Only the first one deals with Coleoptera.

**1813**. *Index entomologicvs sistens omnes insectorum species in G.W.F. Panzeri Favna Insectorvm Germanica descriptas atque delineatas secundum methodum Fabricianam: adiectis emendationibus, observationibus. Pars I. Eleutherata.* Felsecker, Norimbergae [= Nürnberg]. viii + 216 pp. (8vo) [DP: 1813 (title page; *lectori* dated 3 December 1812), January–June 1813 (*Verz Bücher Landkarten*)] CNC, GB, GDZ, BHL

This is essentially an index to all beetles published in the first 109 *Hefte* of Panzer’s “*Favnae insectorvm Germanicae*.”

#### Parry, Frederick John Sidney (London, United Kingdom: 28 October 1810 – 1 February 1885: Bushey Heath, Hertfordshire, United Kingdom). British coleopterist with a special interest in Lucanidae; served in the army 1831–1835 and retired with the grade of Major; his collection and library were auctioned on 16 May 1885 in London but a large part is at the Natural History Museum in London. Reference. Anonymous (1885c).

**1875**. *Catalogus coleopterorum lucanoidum. Editio tertia.* E.W. Janson, London. 29 pp. (8vo) [DP: 1875 (title page), 12 February 1876 (*Ent Soc Lond*), 12 February 1876 [JD] (*Soc Imp Nat Moscou*), 26 April 1876 (*Soc Ent Fr*), 5 May 1876 (*Ent Monats* 1: 74), 15 May 1876 (*Pet Nouv Ent*)] Online

The first and second editions of the catalogue were published in 1864 and 1870 in *The Transactions of the Entomological Society of London*.

#### Pascoe, Francis Polkinghorne (Penzance, Cornwall, United Kingdom: 1 September 1813 – 20 June 1893: Brighton, East Sussex, United Kingdom). British physician and entomologist; surgeon in the Navy, serving on the Australian, West Indian and Mediterranean stations; his collection of over 42000 specimens, including many types, was sold to the British Museum (Natural History) in 1893. References. McLachlan (1893b); Zimmerman (1993: 715–718, P); Woodward (2004a); Gilbert (2005: 48).

**1868**. *A list of the Australian longicorns, chiefly described and arranged by Francis P. Pascoe, Esq., with additional localities and corrections by George Masters, Esq.* F. White, Sydney. [1] + 27 pp. (8vo) [DP: 1868 (title page), 31 October 1868 (*Trüb Amer Orient Lit Rec*), December 1868 (*Allg Bibl*)] GB

**1877**. *Zoological classification: a handy book of reference, with tables of the subkingdoms, classes, orders, &c. of the animal kingdom, their characters, and lists of the families and principal genera.* John van Voorst, London. iv + [2] + 204 pp. (12mo) [DP: 1877 (title page), 17 March 1877 (*Athenaeum*), 31 March 1877 (*Academy*; *Spectator*), 2 May 1877 (*Bookseller*), 31 May 1877 (*Nature*)] GB

The Coleoptera are on pages 97–110. A “second edition. With additions and a glossary” was issued in London in 1880 [BHL].

**1882**. *The student’s list of British Coleoptera, with synoptic tables of the families and genera.* Taylor and Francis, London. viii + 120 pp. (8vo) [DP: 1882 (title page), May 1882 (*Nat Nov*), 5 June 1882 (*Bookseller*), June 1882 (*English Cat Books*; *Entomologist* 15: 142), January–June 1882 (*Bibliot Hist-Nat*), July 1882 (*Allg Bibl*; *Ent Monthly Mag* 19: 46), 7 August 1882 (*Zool Anz*)] GB, ARC

**1888**. *A list of the described longicornia of Australia and Tasmania.* Taylor and Francis, London. vi + 48 pp. (12mo) [DP: 1888 (title page), 9 January 1889 (*Soc Ent Fr*), 27 February 1889 (*Linn Soc NS Wales*)] NCSU

#### Paykull, Gustaf [Gustavo de] (Stockholm, Sweden: 23 July 1757 – 28 January 1826: Stockholm, Sweden). Swedish court officer, poet, ornithologist and entomologist; his collection of insects is at the Naturhistoriska Riksmuseet in Stockholm. References. Blumm (1844); Heurlin et al. (1906: 257, P); Smetana and Herman (2001: 122–123, P).

**1789**. *Monographia staphylinorum Sveciae.* Johann. Edman, Upsaliae [= Uppsala]. [8] + 81 + [1 (Emendanda)] pp. (8vo) [DP: 1789 (title page; *lectori* dated 20 May 1789), 27 May 1790 (*Allg Lit Ztg*)] CNC, GB

**1790**. *Monographia caraborum Sueciae.* Johann. Edman, Upsaliae [= Uppsala]. [5] + 138 pp. (8vo) [DP: 1790 (title page; *lectori* dated 4 July 1790), 3 September 1791 (*Goth Ztg*)] CNC, GB

Some copies have a five-page section entitled “Appendix to Staph.” bound with the book.

**1792**. *Monographia curculionum Sueciae.* Joh. Edman, Upsaliae [= Uppsala]. [6] + 151 + [1 (Emendanda)] pp. (8vo) [DP: 1792 (title page; *lectori* dated 19 December 1791), October 1792 (*Esprit J*), December 1792 (*Observ Phys*)] CMLE, GDZ, GB

This work was published before Fabricius’ tome 1 of his *Entomologia systematica* since Fabricius mentioned Paykull’s species with exact page.

**1798–1800**. *Fauna Svecica. Insecta.* Joh. F. Edman, Upsaliae [= Uppsala]. (8vo) CNC, GB

*Tomus I.* [viii] + 358 + [2 (Errata)] pp. [DP: n.d. (title page; *proëmium* dated 29 January 1798), 20 June 1798 (*Allg Lit Ztg*), August 1798 (*Analyt Rev*), 22 October–20 November 1798 (*Soc Philom*), November 1798 (Sherborn 1902: xlii)].

*Tomus II.* 234 + [2 (Errata)] pp. [DP: n.d. (title page), 20 March 1799 (*Allg Lit Ztg*)].

*Tomus III.* 459 pp. [DP: 1800 (title page), 17 April 1801 (*Allg Lit Ztg*), July 1801 (*Esprit J*)].

**1811**. *Monographia histeroidum.* Stenhammer et Palmblad, Upsiliae [= Uppsala]. [4] + 112 + [2 (Index specierum)] pp. + 13 pls. (8vo) [DP: 1811 (title page; *proaemium* dated 19 January 1811)] CNC, GB

#### Pensa, Antonio. Italian physician in Milan.

**1832**. *De insectis venenatis agri Ticinensis. Dissertatio inauguralis quam annuentibus magnifico domino rectore illustrissimo facultatis directore, spectabili d. decano ac clarissimis D.D. professoribus auspice J. Maria Zendrini hist. nat. p. o. una cum adnexis thesibus defendendis publicae disquisitioni offerebat in C. R. Archigymnasio Ticinensi mense maii MDCCCXXXII lauream medicam assecuturus Antonius Pensa Mediolanensis.* Bizzoni, Ticini Regii [= Pavia]. 31 pp. (8vo) [DP: May 1832 (title page)] GB

This thesis is essentially an enumeration of families and species of venomous insects in the surroundings of Pavia, accompanied by brief descriptions and comments. Nine species of Coleoptera are mentioned: seven Carabidae, one Meloidae, and one Cerambycidae.

#### Peragallo, Alexandre Barthélémy Hyppolite (Montmorillon, Vienne, France: 3 April 1822 – 30 July 1904: Plougasnou, Finistère, France). Director of indirect taxation in Nice.

**1879**. *Les insectes coléoptères du département des Alpes-Maritimes avec indication de l’habitat, des époques d’apparition et des mœurs de ces insectes, des plantes sur lesquelles ils vivent, des dommages qu’ils causent a l’agriculture et des services qu’ils lui rendent.* Malvano-Mignon, Nice. 239 pp. (8vo) [DP: 1879 (title page), 23 August 1879 (*Bibl Fr*), August 1879 (*Nat Nov*), October 1879 (*Feuille Jeunes Nat*), 17 November 1879 (*Zool Anz*), 15 January 1880 (*Naturaliste*)] GAL, GB

#### Percheron, Achille Rémy (Paris, France: 25 January 1797 – 3 June 1869: Paris, France). French naturalist and bibliographer, member of a wealthy Norman family; studied law but soon turned to natural history; gave his collection to the Lycée Turgot in Paris. References. Vapereau (1870: 1420); Dantès (1875: 776).

**1834**. *Monographie des passales et des genres qui en ont été séparés; accompagnée de planches dessinées par l’auteur, ou toutes les espèces ont été figurées.* J.Albert Mercklein, Paris. iii + 107 + [1] pp. + 7 pls. (8vo) [DP: 1835 (title page), October–December 1834 (*Soc Ent Fr* [*Annales*, p. xcv]), 3 January 1835 (*Feuil J Lib*), 10 January 1835 (*Bibl Fr*), 12 January 1835 (*Acad Sci Fr*), February 1835 (*Bull Bibl*)] CMLE, GB, BHL

Most authors date this book 1835 but it was issued late 1834. Agassiz (1854: 92) dated the book 1834.

#### Pérez Arcas, Laureano (Requena, Valencia, Spain: 4 July 1824 – 24 September 1894: Requena, Spain). Spanish zoologist; professor of zoology at the University of Madrid; founder of the *Sociedad Española de Historia Natural*; his collection is at the Museo Nacional de Ciencias Naturales in Madrid. References. Martínez y Sáez (1895, P); Yeves Ochando (1997, P); Vives Noguera (2011: 52–53).

**1869**. *Revista crítica de las especies españolas del género Percus (Bon.).* M. Rivadeneyra, Madrid. 30 pp. (8vo) [DP: 1869 (title page)] MCZ, GB

This work was also published in *Boletin–Revista de la Universidad de Madrid* 2 (Seccion 1) [1870]: 193–220 [DP: 25 November 1869 (p. 193)].

#### Perkins, Robert Cyril Layton (Badminton, Gloucestershire, United Kingdom: 15 November 1866 – 29 September 1955: Bovey Tracy, Devon, United Kingdom). British naturalist; selected by a joint committee of the Royal Society and the British Association for the Advancement of Science to investigate the land-fauna of the Hawaiian Islands (1892–1902); later, worked for the Division of Entomology of the Hawaiian Sugar Planters’ Association. References. Scott and Benson (1955, P); Fullaway (1956); Scott (1956); Harvey et al. (1996: 159); Evenhuis (2007: 27–48, P).

**1900**. II. Coleoptera Rhynchophora, Proterhinidae, Heteromera and Cioidae. Pp. 117–270 + pls 7–10 *in*: *Fauna Hawaiiensis or the zoology of the Sandwich (Hawaiian) isles: being results of the explorations instituted by the joint committee appointed by the Royal Society of London for promoting natural knowledge and the British Association for the advancement of science and carried on with the assistance of those bodies and of the Trustees of the Bernice Pauahi Bishop Museum at Honolulu. Edited by David Sharp. Volume II. Part III. Coleoptera. I. by D. Sharp and R.C.L. Perkins. Pages 91–270; plates VI, VII, VIII, IX, X, uncoloured.* University Press, Cambridge. Pp. 91–270 + 5 pls. (4to) [DP: 8 February 1900 (title page), March 1900 (*Nat Nov*)] CNC

Remarks about the *Fauna Hawaiiensis* are included under Sharp, 1900.

#### Perroud, Benoît-Philibert (Lyon, Rhône, France: 12 February 1796 – 10 February 1878: Lyon, France). French solicitor in Lyon (up to his 50s) and entomologist; maintained relations with captains and clergymen, including Father Xavier Montrouzier, missionary in New Caledonia; travelled to England and Germany, with Étienne Mulsant [*q.v.*], and to Algeria; built a large beetle collection through purchases, gifts, and his own collecting particularly in Provence and Languedoc; his collection passed to his son who sold it to Maurice Pic [*q.v.*] in 1932. References. Mulsant (1878, P); Cambefort (2006: 260, P).

**1853–1855**. *Mélanges entomologiques.* L. Maison [parts 2–3] / F. Savy [part 4], Paris. (8vo) CMLE (mf), NCSU, CNC (mf)

*Deuxième partie.* 142 + [1] pp. [DP: 1853 (title page), 21 July 1854 (*Soc Agr Hist Nat Arts Lyon*), 2 October 1854 (*Ent Soc Lond*)]. This publication was also issued in the *Annales de la Société Linnéenne de Lyon* (Nouvelle Série) 1 [1852–53]: 389–528 [DP: 1853 (title page), 2 October 1854 (*Ent Soc Lond*), 5 October 1854 (*Acad R Sci Belg*)].

*Troisième partie.* 100 pp. [DP: 1855 (title page), 20 June 1856 (*Soc Agr Hist Nat Arts Lyon*), 5 July 1856 (*Bibl Fr*), 19 July 1856 (*Athenaeum Fr*), 1 December 1856 (*Ent Soc Lond*), July–December 1856 (*Bibliot Hist-Nat*)]. This publication was also issued in the *Annales de la Société Linnéenne de Lyon* (Nouvelle Série) 2 [1854–55]: 321–420 [DP: 1855 (title page), 2 August 1856 (*Bibl Fr*), 1 December 1856 (*Ent Soc Lond*)].

*Quatrième partie*. 212 pp. [DP: 1864 (title page), 31 December 1864 (*Bibl Fr*), 5 January 1865 (*Rev Instr Publ*), 10 January 1865 (*Acad Imp Sci Lyon*)]. The cover has Perroud as sole author but the first page, which is entitled “*Essai sur la faune entomologique de Kanala (Nouvelle-Calédonie) et description de quelques espèces nouvelles ou peu connues*,” has Perroud and Xavier Montrousier [1821–1897] as authors. This part was also issued in the *Annales de la Société Linnéenne de Lyon* (Nouvelle Série) 11: 46–257 + 1 pl. [DP: 1864–February 1865 (title page)^87^, 13 May 1865 (*Bibl Fr*)].

The first part entitled “*Description de quelques Coléoptères nouveaux ou peu connus, (tribu des carabiques, famille des truncatipennes, section des anthiaires). Premier fascicule*” was published in the *Annales de la Société Linnéenne de Lyon* (Années 1845–46): 25–64 [DP: 1847 (title page), 27 March 1847 (*Bibl Fr*)].

#### Perty, Joseph Anton Maximilian (Ornbau, Bavaria, Germany: 17 September 1804 – 8 August 1884: Bern, Switzerland). German naturalist; private docent in Munich (1831–1833); professor of zoology and later dean at the University of Bern (1833–1876); the Brazilian specimens collected from the Spix and Martius expedition are in the Zoologische Staatssammlung des Bayerischen Staates in Munich. References. Hess (1887); Kurmann (1959); Papavero (1971: 110–111); Kirschner (2001).

**1830–1833**. *Delectus animalium articulatorum, quae in itinere per Brasiliam annis MDCCCXVII–MDCCCXX jussu et auspiciis Maximiliani Josephi I. Bavariae regis augistissimi peracto collegerunt Dr. J.B. de Spix et Dr. C.F.Ph. de Martius. Digessit, descripsit, pingenda curavit Dr. Maximilianus Perty, praefatus est et edidit Dr. C.F.Ph. de Martius.* Monachii [= Munich]. 224 pp. + 40 pls. (4to) MCZ, GB

This work was published in three fascicles: **1**: (pp. 1–60 + pls 1–12) 1830 (title page), January–June 1831 (*Verz Bücher Landkarten*), July–September 1831 (*Foreign Quart Rev*), July–December 1831 (*Verz Derj Bücher*); **2**: (pp. 61–124 + pls 13–24) 1832 (Sherborn 1922a: ci), May–July 1832 (*Foreign Quart Rev*), November 1832 (*Isis*, Heft XI: 1212); **3**: (pp. 125–224 + pls 25–40) December 1833 (*Isis*, Heft XII: 1164), March 1834 (*Bull Litt Étr*), May–July 1834 (*Foreign Quart Rev*). The colophon is dated December 1833. The half-title page reads “*Delectus animalium articulatorum in Brasilia collectorum*.” The entire work, along with the “*De insectorum in America meridionali*” [*q.v.*], was reissued in 1834.

The book consists of descriptions of Brasilian arthropods; many new genera and species are described. The Coleoptera are in the first and second fascicles (pp. 1–116 + pls 1–22).

**1831**. *Observationes nonnullae in Coleoptera Indiae orientalis. Dissertatio philosophico- entomologica, quam unacum praemissis thesibus auctoritate et consensu illustris philosophorum ordinis in Academia Ludovico-Maximilianea facultatem legendi rite adepturus die XXV^ta^ Jan. MDCCCXXXI. Loco solito publice defendet Maximilianus Perty. Cum tabula I.* Mich. Lindauer, Monachii [= Munich]. xxxxiv pp. + 1 pl. (4to) [DP: 25 January 1831 (title page), February 1831 (*Isis*, Heft II: [2]), 16 May 1831 (*Bibl Deutsch*), January–June 1831 (*Verz Bücher Landkarten*), July–September 1831 (*Foreign Quart Rev*)] MCZ, GB, MDZ

This thesis pertains to the Coleoptera of east India which is taken in a very wide sense as many species are cited and some described from China, Cochin China, Sumatra, Java and Amboina. The work contains two main sections: the “*Conspectus coleopterorum Indiae Orientalis*” (pp. ix–xxvii) which is the catalogue of the species and the “*Specierum quarundam novarum, conspectu praecedente indicatarum, descriptiones*” (pp. xxxii– xxxxii) containing the descriptions of new genera and species.

**1833**. *De insectorum in America meridionali habitantium vitae genere, moribus ac distributione geographica observationes nonnullae.* Monachii [= Munich]. 46 pp. (Folio) [DP: 1833 (title page), December 1833 (*Isis*, Heft XII: 1172)] GB, MDZ

This publication consists of a discussion of the insects of South America by orders and for Coleoptera by major groups. The Coleoptera are on pages 4–20 and some new species are briefly described (e.g., *Pterotarsus griseus* and *Calirhipis latreilli*, p. 7); these species are not included in Sherborn’s *Index Animalium*.

This work was reissued in 1834 along with “*Delectus animalium articulatorum*” [*q.v.*] and noticed on 12 April 1834 (*Bayer Ann*).

**1839–1840**. *Allgemeine Naturgeschichte, als philosophische und humanitätswissenschaft für Naturforscher, Philosophen und das höher gebildete Publikum. III. Band.* C. Fischer, Bern. xii + pp. 467–1119 + xxxiv (Sachverzeichniss). (8vo) BHL, GB

This volume was issued in two parts: **1** [*Lieferungen* 4–5 of the series]: (pp. 241–720) 27 November 1839 (*Lit Ztg*), 29 November 1839 (*Allg Bibl Deutsch*), July–December 1839 (*Verz Bücher Landkarten*); **2** [*Lieferungen* 6–7]: (pp. 721–1119 + xii + xxxiv) 17 November 1840 (*Allg Ztg*), 19 November 1840 (*Schweizer-Bote*), 25 November 1840 (*Lit Ztg*), 27 November 1840 (*Allg Bibl Deutsch*). The title page is dated 1841 and the *Vorrede* September 1840.

The entire series consists of four volumes (*Bande*), 1837–1846, with the second and third ones continuously paginated. The Coleoptera are on pages 917–939 of the third *Band* and a few new family-group names of Coleoptera are proposed. The first three volumes of the series were reissued in 1843 by the same publisher.

#### Petagna, Vincenz (Naples, Campania, Italy: 17 January 1734 – 6 October 1810: Naples, Italy). Italian physician, botanist and entomologist; professor of botany at the University of Naples; director of the Monte Oliveto Botanical Garden in Naples; not known to have owned a personal collection of insects. References. Olivier-Poli (1825: 43–44); Stellati (1834); Minieri-Riccio (1844: 267); Castiglione (2010: 529–530).

**1786**. *Specimen insectorum ulterioris Calabriae*. Petri Perger, Neapoli. vii + 46 pp. + 1 pl. (4to) [DP: 1786 (title page), 19 November 1786 (*Acad R Sci Turin* 1790: ciii)] GDZ, GB

The original edition is extremely rare (Evenhuis 1997b: 610). A German edition, essentially identical to the Naple edition, was published in 1787 by Varrentrapp et Wenner, in Francfurt and Mainz [CMLE, GB, BHL]. An “*editio nova cvm XXXVIII iconibus ad naturam coloratis*,” identical to the Naple edition, was issued in Lipsiae [= Leipzig] by Ioannem Sommer in 1808 [CMLE (mf), GB] and again in 1820 [NAL].

This work treats the insects of Calabria. The Coleoptera are on pages 1–28 and the following new species are described: *Scarabaeus candidae* (p. 3), *Silpha 2-maculata* (p. 7), *Curculio triangularis* (p. 14), *Rhinomacer cacruleus* (p. 14), *Rhagium nigrum* (p. 17), *Buprestis bruttia* (p. 22), *Buprestis stephanelli* (p. 23) and *Mylabris melanura* (p. 27).

**1792**. *Institutiones entomologicae*. Cajetani Raymundi, Neapoli. (8vo) MCZ, BHL

*Tomus I.* xii + 439 pp. [DP: 1792 (title page)].

*Tomus II.* Pp. 441–718 + [10] + 10 pls. [DP: 1792 (title page)].

The taxonomic section (pp. 129–716) of this work treats the known genera of insects and the European species. The Coleoptera are on pages 130–301 of the first volume and in the addenda of the second volume (pp. 712–718); a few new species are proposed.

#### Petiver, James (Hillmorton near Rugby, Warwickshire, England: *ca* 1663 – April 1718: London, United Kingdom). British apothecary, collector, and promoter of natural history; his collections, books and manuscripts were purchased by Sir Hans Sloane for £4000 and subsequently passed to the British Museum (Natural History) in London. References. Stearns (1953); Lisney (1960: 42–64); Salmon (2000: 103–105); Allen (2004).

**1767**. *Opera historiam naturalem spectantia: containing several thousand figures of birds, beasts, fish, reptiles, insects, shells, corals, and fossils; also of trees, shrubs, herbs, fruits, fungus’s, mosses, sea-weeds, &c. from all parts, adapted to Ray’s History of plants, on above three hundred copper-plates, with English and Latin names. The shells have English, Latin, and native names. N.B. Above one hundred of these plates were never published before. To which are now added seventeen curious tracts, most of them so scarce as not to be purchased, which completes all he ever wrote upon natural history. The two volumes containing above ten thousand articles, engraved in the most accurate manner, from originals, the gifts of the most eminent persons in all nations. The additions corrected by the late Mr. James Empson, of the British Museum, &c*. *Volume I.* John Millan, London. [2] + 12 + 4 + 4 + 12 + 10 + 30 pp. + 178 pls [numbered 1–156, 1–22], ii pp. + 2 pls. (Folio) [DP: 1767 (title page)] MCZ, BHL.

The entire work was published in two volumes, issued simultaneously; the second volume pertains to botany. The content of this publication was apparently issued by Petiver earlier in various essays, most of them inserted in his “*Musei Petiveriani centuria rariora naturae continens*” (*n.v.*), 1696–1703, and “*Gazophylacii naturae & artis*” (*n.v.*), 1702–1711 (see Johnston 2004: 5). The 180 plates were also issued, 1764, in Petiver’s “*Opera, historiam naturalem spectantia; or, Gazophylacium. Containing several 1000 figures of birds, beasts, reptiles, insects, fish, beetles, moths, flies, shells, corals, fossils, minerals, stones, fungusses, mosses, herbs, plants, &c. from all nations, on 156 copper-plates, with Latin and English names. The shells, &c. have English, Latin, and native names. Vol. 1. N.B. About 100 of these plates were never published before.*” [MCZ]. Although the title is similar to the 1767 issue, the text is in part different. For example, the 30-page section, which includes “De Scarabaeis & Bombyliis Anglicanis: or, an account of English beetles and bees” (pp. 11–30), is missing in the 1764 issue. The work is not binominal and so no new taxa are available from it.

#### Petri, Karl (Sibiu, Romania: 17 December 1852 – 22 November 1932: Sibiu, Romania). Romanian educator and entomologist; teacher and later director of the Bürgerschule in Sighișoara, central Romania (1877–1916); his Coleoptera collection was donated to the Natural History Museum of Sibiu in 1930. Reference. Kurir (1979).

**1885**. *Ergebnisse entomologischer Excursionen im Gebiete Schässburgs.* Pp. 5–?34 *in*: *Programm des evang. Gymnasiums A.B. in Schässburg und der damit verbundenen Lehranstalten. Zum Schlusse des Schuljahres 1884/85 veröffentlicht vom Direktor Daniel Höhr.* Friedr. J. Horeth, Schässburg. 68 pp. (4to) [DP: 1885 (title page), 1 July–30 September 1885 (*Kaiser Geol Reichs* 1885: 311), 15 October 1885 (*Ver Sieb Land* 8: 120)] YB (ph, partial)

Several new Coleoptera species-group taxa are described in this work (see Reitter 1887: 247) from the material collected in the region of Schässburg (= Sighişoara) in central Romania.

#### Pfaff, Christoph Heinrich (Stuttgart, Baden-Württemberg, Germany: 2 March 1773 – 23 April 1852: Kiel, Schleswig-Holstein, Germany). German physician, chemist and physicist; professor of medicine and chemistry at the University of Kiel; member of the Prussian Academy of Sciences. References. Karsten (1887); Kragh and Bak (2000).

**1824**. *System der Materia medica nach chemischen Principien mit Rücksicht auf die sinnlichen Merkmale und die Heilverhältnisse der Arzneymittel. Für Ärzte, Chemiker und Apotheker. Siebenter oder zweyter Supplementband. Enthaltend Nachträge zu den sechs ersten Bänden, nebst vollständigem Sach- und Namenregister über alle sieben Bände.* Fr. Chr. Wilh. Vogel, Leipzig. xiv + 424 pp. (8vo) [DP: 1824 (title page; *Vorrede* dated 16 April 1824), August 1824 (*Intell Jena Lit Ztg*), November–December 1824 (*Arch Med Erf* 1824: 538)] MDZ, GB

Another title page reads “*Die neuesten Entdeckungen in der Chemie der Materia medica; systematisch dargestellt nebst eigenthümlichen Versuchen. Für Ärzte, Chemiker und Apotheker. Zweyter Band.*” One new species of beetle is described, *Lytta coerulea* (p. 209).

The entire work consists of seven volumes, 1808–1824.

#### Philippi, Rudolph Amandus [Rodolfo Amando] (Berlin, Germany: 14 September 1808 – 23 July 1904: Santiago, Chile). German zoologist and botanist; high school teacher in Cassel, Germany (1835–1850); moved to Santiago in 1851; professor of botany and zoology at the University of Santiago and director of the Natural History Museum; his collection is at that museum. References. Barros-Arana (1904); Gotschlich (1904); Losch (1905); Papavero (1973: 275–281); Zirnstein (2001); Smith-Ramírez (2004, P).

**1860**. *Reise durch die Wueste Atacama auf Befehl der Chilenischen Regierung im Sommer 1853–54 unternommen und beschrieben. Nebst einer Karte und XXVII Tafeln.* Eduard Anton, Halle. ix + [1] + 192 + 62 pp. + 27 pls. (4to) [DP: 1860 (title page; *Vorrede* dated 29 August 1858), 8 November 1860 (*Allg Bibl Deutsch*), 15 November 1860 (*Intell Serapeum*), 31 December 1860 (*Publ Circ*)] McG, GB

A list of Coleoptera collected during Philippi’s journey to the Atacama Desert is given on pages 171–173. A few species are described but all, except Opatrum ? brevicolle Ph. Germain (p. 172), were first made available by Philibert Germain [1827–1913] in *Anales de la Universidad de Chile* 1854: 325–336; 1855: 386–407.

The book was also published in Spanish the same year under the title “*Viage al desierto de Atacama hecho de orden del gobierno de Chile en el verano 1853–54*” [GB, BNDC]. The list of Coleoptera is on pages 153–155.

#### Phillips, John (Marden, Wiltshire, United Kingdom: 25 December 1800 – 24 April 1874: Oxford, Oxfordshire, United Kingdom). British geologist; professor of geology at the University of Dublin and later at the University of Oxford. References. Anonymous (1874e); Bonney (1896); Morrell (2005, P).

**1871**. *Geology of Oxford and The Valley of The Thames.* Clarendon Press, Oxford. xxiv + 523 pp. + 17 pls. (8vo) [DP: 1871 (title page), 18 November 1871 (*Athenaeum*), 1 December 1871 (*Academy*), 14 December 1871 (*Nature*)] GB, BHL

Two new fossil beetle genera, *Buprestidium* (p. 174) and *Curculionidium* (p. 174), are made available through illustrations. No species are associated with these genera.

#### Pic, Maurice (Marcigny near Digoin, Saône-et-Loire, France: 23 March 1866 – 29 December 1957: Les Guerreaux, Saône-et-Loire, France). Fanatic French entomologist of independent means who, in the course of his life, described more than 18,500 new species and almost 6,000 new varieties and aberrations; assembled one of the largest beetle collection of his time, mainly through purchases, which unfortunately was left in discord for some time and is now at the Muséum National d’Histoire Naturelle in Paris. References. Villiers (1958, P); Cambefort (2006: 263–269, P).

**1890**. *Variétés, 1. Article.* L. Jacquet, Lyon. 3 pp. [DP: 1890 (Pic 1898: 191), 10 December 1890 (*Soc Ent Fr*)] (*n.v.*).

Based on Pic’s (1898: 210–224) index to his new species and varieties, the following taxa were described in this publication: *Callimus abdominalis* v[ariety] *nigricolle* (p. 3), *Strangalia distigma* v. *tenietensis* (p. 3) and *Xylotrechus arvicola* v. *obliquefasciatus* (p. 3).

**1891–1917**. *Matériaux pour servir à l’étude des longicornes.* L. Jacquet, Lyon [cahiers 1–2]; Jacquet Frères, Lyon [cahier 3]; Bussière, Saint-Amand (Cher) [cahiers 4–10]. (8vo) CNC, CMLE, BHL (except vol. 1)

*1er cahier.* v + 67 pp. [DP: June 1891 (title page; *avant-propos* dated 1 June 1891), 14 October 1891 (*Soc Ent Fr*)]. A second edition of this *cahier*, containing 53 pages, was published in June 1913 [CNC, BHL].

*2me cahier.* v + 59 + [1 (Errata)] pp. [DP: January 1898 (title page; *avant-propos* dated 20 November 1897), 9 April 1898 (*Bibl Fr*), 30 May 1898 (*Bibl Zool*), 14 December 1898 (*Soc Ent Fr*)].

*3me cahier.* iv + 17 pp. [DP: February 1900 (title page; *avant-propos* dated 10 December 1899), 11 April 1900 (*Soc Ent Fr*)].

*3me cahier, 2me partie. Catalogue bibliographique et synonymique des longicornes d’Europe et des régions avoisinantes.* 120 + [1] pp. The pages were published as follows with the dates of publication listed at the bottom of some pages (the *avant-propos* is dated December 1899): (pp. 1–42) 1900; (pp. 43–66) December 1900; (pp. 67–74) November 1901; (pp. 75–78) July 1902; (pp. 79–82) June 1903; (pp. 83–86) May 1904; (pp. 87–90) July 1906; (pp. 91–94) October 1908; (pp. 95–98) March 1910; (pp. 99–102) April 1911; (pp. 103–106) September 1912; (pp. 107–110) February 1914; (pp. 111–114) February 1915; (pp. 115–118) February 1916; (pp. 119–120 + [1]) February 1917. The pages were annexed with the regular *cahier*.

*3me cahier, 3me partie.* 33 pp. [DP: November 1901 (title page; p. 3 dated September 1901), 11 January 1902 (*Bibl Fr*)].

*4me cahier, 1re partie.* 37 pp. [DP: July 1902 (title page; *avant-propos* dated 20 June 1902), 14 January 1903 (*Soc Ent Fr*)].

*4me cahier, 2me partie.* 33 pp. [DP: June 1903 (title page; *avant-propos* dated 10 May 1903)].

*5me cahier, 1re partie.* 22 + [1] pp. [DP: May 1904 (title page; *avant-propos* dated 20 March 1904)].

*5me cahier, 2me partie.* 38 + [1] pp. [DP: April 1905 (title page; *avant-propos* dated 2 March 1905)].

*6me cahier, 1re partie.* 24 + [1] pp. [DP: July 1906 (title page; *avant-propos* dated 20 June 1906), October 1906 (*Nat Nov*), November 1906 (*Ent Litt*)].

*6me cahier, 2e partie.* 28 + [1] pp. [DP: March 1907 (title page; *avant-propos* dated 20 February 1907), 8 May 1907 (*Soc Ent Fr*), May 1907 (*Nat Nov*), July 1907 (*Ent Litt*)].

*7me cahier, 1re partie.* 24 + [1] pp. [DP: October 1908 (title page; *avant-propos* dated 10 August 1908), December 1908 (*Nat Nov*)].

*7me cahier, 2e partie.* 24 + [1] pp. [DP: March 1910 (title page; *avant-propos* dated 20 November 1909), June 1910 (*Ent Litt*)].

*8me cahier, 1re partie.* 24 + [1] pp. [DP: April 1911 (title page; *avant-propos* dated 10 March 1911)].

*8me cahier, 2e partie.* 24 + [1] pp. [DP: 25 September 1912 (title page; *avant-propos* dated 28 June 1912)].

*9me cahier, 1e partie.* 24 + [1] pp. [DP: 10 February 1914 (title page; p. 2 dated 8 January 1914)].

*9me cahier, 2e partie.* 24 + [1] pp. [DP: 20 February 1915 (title page; *avant-propos* dated 12 January 1915)].

*10me cahier, 1re partie.* 20 + [1] pp. [DP: 10 February 1916 (title page; *avant-propos* dated 4 January 1916), 10 May 1916 (*Soc Ent Fr*)].

*10me cahier, 2e partie.* 20 + [1] pp. [DP: 8 February 1917 (title page; *avant-propos* dated 10 January 1917), 13 June 1917 (*Soc Ent Fr*)].

**1891**. *Descriptions de longicornes de Syrie.* L. Jacquet, Lyon. 2 pp. [DP: 1891 (Pic 1898: 191), 19 December 1891 (*Soc Ent Fr*)] (*n.v.*).

Based on Pic’s (1898: 210–224) index to his new species and varieties, the following taxa were described in this publication: *Agapanthia brevis* (p. 1), *Strangalia syriaca* (p. 1), *Conizonia delagrangei* (p. 2), *Coptosia minuta* (p. 2), *Leptura scutellata* F.? v[ariety] *inscutellata* (p. 2) and *Phytoecia grandis* (p. 2).

**1897**. *Variétés, 2eme article.* L. Jacquet, Lyon. 3 pp. [DP: 1897 (Pic 1898: 191)] (*n.v.*).

Based on Pic’s (1898: 210–224) index to his new species and varieties, the following taxa were described in this publication: *Osphya bipunctata* v[ariety] *obscuripennis* (p. 2), *Osphya aeneipennis* v. *immaculata* (p. 3); *Osphya aeneipennis* v. *maculicollis* (p. 3), *Crioceris macilenta* v. *corsica* (p. 3); *Crioceris macilenta* v. *hipponensis* (p. 3); *Crioceris macilenta* v. *jacqueti* (p. 3); *Crioceris macilenta* v. *lineata* (p. 3); *Crioceris macilenta* v. *tournieri* (p. 3); *Lema rugicollis* v. *obscurior* (p. 3).

**1900**. *Bestimmungs-Tabelle der europäischen Coleopteren. XL. Heft. Enthaltend: Hylophilidae (früher Euglenini und Xylophilini). Bearbeitet von Maurice Pic unter Mithülfe der Frau Therese Pic in Digoin.* Edm. Reitter, Paskau. 20 + [1 (Index)] pp. (8vo) [DP: 1900 (title page; *Vorwort* dated November 1899), February 1900 (*Nat Nov*), 16 March 1900 (*Bibl Zool*), 11 April 1900 (*Soc Ent Fr*)] SME, GB

#### Pictet [de la Rive], François Jules (Geneva, Switzerland: 27 September 1809 – 15 March 1872: Geneva, Switzerland). Swiss zoologist and palaeontologist; professor of zoology and comparative anatomy at the Academy of Geneva. References. Soret (1872, P); Peters et al. (1980); Gilbert (2005: 48); Hollier and Hollier (2014, P).

**1854**. *Traité de paléontologie ou histoire naturelle des animaux fossiles considérés dans leurs rapports zoologiques et géologiques. Seconde édition, revue, corrigée, considérablement augmentée, accompagnée d’un atlas de 110 planches grand in-4^o^. Tome deuxième.* J.-B. Baillière, Paris. 727 pp. (8vo) [DP: 1854 (title page), 29 April 1854 (*Bibl Fr*)] GB, BHL

The entire work consists of four volumes, 1853–1857, and one atlas of 110 plates [BHL] issued in livraisons. The Coleoptera are on pages 318–360 of the second volume. The first edition was published in four volumes, 1844–1846, under the title “*Traité élémentaire de paléontologie*…” [GB].

#### Piller, Mathias (Gratz, Styria, Austria: 25 April 1733 – 10 November 1788: Buda [currently part of Budapest], Hungary). Austrian Jesuit and naturalist; professor of zoology, botany and mineralogy at the University of Tyrnau (= Trnava); later, professor of natural history at the Royal University of Buda, Hungary. References. De Backer and De Backer (1861: 445); Wurzbach (1870: 293); Paušek-Baždar (1995).

**1783**. [Piller, M. and Mitterpacher, L.] *Iter per Poseganam Sclavoniae provinciam mensibus Junio, et Julio anno MDCCLXXXII.* Regiae Universitatis, Budae. 147 pp. + 16 pls. (4to) [DP: 1783 (title page), May 1784 (*J Polit*), 11 September 1784 (*Gött Anz*), October 1784 (*New Rev*)] CNC, BHL

This is an account of Piller and Ludovico Mitterpacher’s collecting trip to Požega in Slavonia, Croatia. They give for each station visited a list of plants and animals observed and described the species they considered new. A German translation of excerpts of this work with annotations was issued by Mader (1788: 92–187). The descriptions of the new species of insects were extracted and most commented by Borckhausen (1790). Marseul (1892: 291–300) reproduced all the original descriptions of new Coleoptera in this work. Rautenberg (1954: 101–104) gave a list of the insects illustrated on plates 4–9.

#### Pini, Ermenegildo [Carlo] (Milan, Lombardy, Italy: 17 June 1739 – 3 January 1825: Milan, Italy). Italian priest; professor of mathematics and natural history at the San Alessandro College in Milan as well as curator of the natural history museum. References. Rovida (1832); Anonymous (1863b).

**1808**. *Elementi di storia naturale degli animali. Ad uso de’ licei del regno d’Italia.* Stamperia Reale, Milano. xii + 417 pp. + 12 pls. (8vo) [DP: 1808 (title page), September 1808 (*Gior Bibl Univers*)] GB

The Coleoptera are on pages 263–280.

#### Pirazzoli, Odoardo (Imola, Emilia-Romagna, Italy: 6 April 1815 – 30 March 1884: Imola, Italy). Italian engineer, army officer and coleopterist; captain of the National Guard of Imola; his beetle collection is at the Museo Civico “Giuseppe Scarabelli” in Imola. Reference. Conci (1975: 988).

**1855**. *Coleopteri Italici. Genus novum Leptomastax.* Typographia Galeati, Forocornelii [= Imola]. 5 pp. + 1 pl. (8vo) [DP: 1855 (title page), 6 February 1856 (*Zool-Bot Gesell Wien*)] CNC

#### Pittier, Henri François (Bex, Switzerland: 13 August 1857 – 27 January 1950: Caracas, Venezuela). Swiss geographer, botanist and ethnologist; moved to Costa Rica in 1887 where he studied the fauna and flora of the country and founded the Physical Geographic Institute; relocated to the United States in 1901 to work at the Department of Agriculture, section botany; stayed in Venezuela in 1913 and returned for good in 1917; the Henri Pittier National Park in Venezuela is named after him. References. Häsler and Baumann (2000); Seitz (2007).

**1895**. [Pittier, H.F. and Biolley, P.] *Invertebrados de Costa Rica. I. Coleópteros. (Especies hasta hoy coleccionadas y determinadas).* Tip. Nacional, San José de Costa Rica A.C. 40 + [2] pp. (8vo) [DP: 1895 (title page)] MCZ, GB

The entire work consists of three parts, 1895–1897; only the first one pertains to beetles.

#### Poda von Neuhaus, Nicolaus (Vienna, Austria: 3 October 1723 – 29 April 1798: Vienna, Austria). Austrian Jesuit and naturalist; professor of physics at the University of Graz (1758–1766); professor of mathematics and mechanics in Schemnitz [= Banská Štiavnica, Slovakia] (1766–1771); the whereabouts of his collection of insects are unknown. References. Ernesti (1806: 109–110); Conci (1975: 989); Speta (2004).

**1761**. *Insecta musei Graecensis, quae in ordines, genera et species juxta Systema Naturae Caroli Linnaei digessit Nicolaus Poda, e Societate Jesu, Philosophiae Doctor, et matheseos professor. Honoribus reverendissimorum, illustrissimorum, perillustrium, reverendorum, praenobilium, nobilium, ac eruditorum D.D. cum in alma ac celeberrima Universitate Graecensi prima ac suprema Philosophiae Laurea insignirentur, oblata anno 1761. Die 3. Septembris.* Haeredum Widmanstadii, Graecii [= Graz]. [6] + 127 + [12] pp. + 2 pls. (8vo) [DP: 3 September 1761 (title page)] GB, MDZ

A separate was published the same year under the title “*Insecta musei Graecensis, quae in ordines, genera et species juxta Systema Naturae Caroli Linnaei digessit Nicolaus Poda, e Societate Jesu, philosophiae doctor, et matheseos professor. Prostant apud Joannem Baptistam Dietrich, bibliopolam*” by the same publisher [GDZ]. A facsimile of the separate was published by W. Junk in Berlin, 1915 [CNC].

Poda von Neuhaus’ book is considered the first purely entomological work to adhere to the binominal nomenclature.

#### Poey [Poey y Aloy], Felipe (Havana, Cuba: 26 May 1799 – 28 January 1891: Havana, Cuba). Cuban entomologist and ichthyologist; studied law in Madrid; left for Cuba in 1823; travelled to France (1825–1833); founding member of the *Société Entomologique de France*; director of the Museum of Natural History in Havana; professor of zoology and comparative anatomy at the University of Havana; his collection of beetles went to the Academy of Natural Sciences in Philadelphia via T.B. Wilson. References. Jordan (1884, P); Quiroga y Rodriguez (1891, P); Mestre (1915, P).

**1851–1854**. *Memorias sobre la historia natural de la Isla de Cuba, acompañadas de sumarios latinos y extractos en frances. Tomo 1.* Barcina, Habana. 463 pp. + 34 pls. (4to) BHL, GB

The entire work consists of two volumes issued in eight parts, 1851–1861. The Coleoptera are treated in chapter 25, entitled “*Conspectus familiarum coleopterorum*,” in the first volume (pp. 302–337). This volume was published in five parts as noted on page 449 of the work (see also Norman 1938: 135): **1**: (pp. 1–40 + pls 1–8) November 1851; **2**: (pp. 41–120 + pls 9–14) April 1852; **3**: (pp. 121–200 + pls 15–22) October 1852; **4**: (pp. 201–280 + pls 23–30) May 1853; **5**: (pp. 281–453 + pls 31–34) June 1854. The title page is dated 1851. A facsimile of the entire work was published by Antiquariaat Junk in Lochem (Netherlands), 1975 [CNC].

#### Poiret, Jean Louis Marie (Saint-Quentin, Aisne, France: 11 June 1755 – 7 April 1834: Paris, France). French clergyman, botanist and explorer; sent by Louis XVI to La Calle [El Kala] in Algeria in 1785–86 to study the flora; defrocked during the French Revolution and became professor of natural history in Soissons (Aisne); the electronic journal *Poiretia*, created in 2009 to survey, describe and map animals and plants living in North Africa, honors his memory. Reference. Baudemant (1887).

**1789**. *Voyage en Barbarie, ou lettres écrites de l’ancienne Numidie pendant les années 1785 & 1786, sur la religion, les coutumes & les moeurs des Maures & des Arabes-Bédouins; avec un essai sur l’histoire naturelle de ce pays. Première partie.* J.B.F. Née de la Rochelle, Paris. xxiv + 363 + [1] pp. (8vo) [DP: 1789 (title page), 6 March 1789 (*Gaz Fr*), June 1789 (*J Sav*), 1 September 1789 (*J Hist Lit*), 28 September 1789 (*Gött Anz*), October 1789 (*An Lit*), December 1789 (*Esprit J*)] HOU, GB, BHL

The entire work was published in two volumes issued simultaneously but only the first one deals with insects (pp. 291–354). The only new species of Coleoptera described is *Scarabaeus marginatus* (p. 291). A German translation was published in two volumes in Strasburg, 1789, under the title “*Reise in die Barbarey oder Briefe aus Alt-Numidien geschrieben in den Jahren 1785 und 1786 über die Religion, Sitten und Gebräuche der Mauren und Bedouin-Araber. Nebst einem Versuche über die Naturgeschichte dieses Landes. Mit Kupfern*” [GB]. An English translation was published in London, 1791, under the title “*Travels through Barbary, in a series of letters, written from the ancient Numidia, in the years 1785 and 1786, and containing an account of the customs and manners of the Moors, and Bedouin arabs. Translated from the French of the Abbe Poiret*” [OLIL-online]. An abridged Swedish version, translated and edited by Samuel Ödmann, was published in Stockholm, 1792, under the title “*Herr Poirets bref om Numidien. I sammandrag*” (*n.v.*).

#### Pontoppidan, Erik Ludvigsen (Aarhuus, Denmark: 24 August 1698 – 20 December 1764: Copenhagen, Denmark). Danish prelate, historian, and topologist; professor of theology at the University of Copenhagen; bishop of Bergen (1748–1764); vice chancellor at the University of Copenhagen (1755–1764); published mainly translations and material supplied by other authors. References. Nielsen (1911); Evenhuis (1997b: 622, P); Damkaer (2002: 37–38, P); Thomas (2009: 333).

**1763**. *Den danske atlas eller Konge-Riget Dannemark, med dets naturlige egenskaber, elementer, indbyggere, vaerter, dyr og andre affødninger, dets gamle tildragelser og naervaerende omstaendingheder i alle provintzer, staeder, kirker, slotte og herre-gaarde. Forestillet ved en udførlig lands-beskrivelse, saa og oplyst med dertil forfaerdigede land-kort over enhver provintz, samt ziret med staedernes prospecter, grund-ridser, og andre merkvaerdige kaaber-stykker. Efter Høy-Kongelig allernaadigst Befalning. Tomus I.* A.H. Godiche, Kiøbenhavn [= Copenhagen]. [6] + xl + [4] + 723 + [1] pp. + 30 pls. (4to) [DP: 1763 (title page; preliminaries dated 31 March 1763)] CAKL, GDZ

Pontoppidan’s work was published in three volumes, 1763–1767, and continued by Hans de Hofman and Jacob Langebek in four further volumes, 1768–1781 (see Henningsen 2010). The Coleoptera are in the first volume on pages 664–678. A German translation of pages 361–700 was issued in Copenhagen and Hamburg, 1765, under the title “*Erich Pontoppidans, Kurzgefasste Nachrichten, die Naturhistorie in Dännemark betreffend. Aus dem Dänischen übersetzt. Mit Kupfern*” [MCZ, CNC, BHL, GB]. It seems there is no additional material included in this translation. Another German translation was published in two volumes, 1766–1767, in Copenhagen under the title “*Erich Pontoppidans, Dänischer Atlas, oder Beschreibung des Königreichs Dännemark, nach seiner politischen und physikalischen Beschaffenheit. Mit Landkarten und andern Kupferstichen versehen. Aus dem dänischen übersetzt, und mit Anmerkungen begleitet von Johann Adolph Scheiben*” [CAKL, GB].

#### Pouchet, Félix-Archimède (Rouen, Seine-Maritime, France: 26 August 1800 – 6 December 1872: Rouen, France). French naturalist and leading proponent of spontaneous generation; professor at the Rouen Museum of natural history and the Rouen Jardin des Plantes; later, professor at the School of Medicine at Rouen. References. Anonymous (1868a: 660–661); Knight (1872: 1005); Coquidé (2015).

**1841**. *Zoologie classique, ou histoire naturelle du règne animal. Second edition, considérablement augmentée. Tome second, contenant les animaux invertébrés. Ouvrage accompagné d’un atlas, de 44 planches et de 5 grands tableaux, le tout gravé sur acier.* Librarie encyclopédique de Roret, Paris. 656 pp. (8vo) [DP: 1841 (title page; *préface* in the first volume dated 1 February 1841), 6 March 1841 (*Bibl Fr*), 24 March 1841 (*Lit Ztg*), March 1841 (*Rev Zool*), 30 April 1841 (*Intell Serapeum*), 24 May 1841 (*Acad Sci Fr*)] GB

The entire work consists of two volumes issued simultaneously and one atlas (*n.v.*). The Coleoptera are on pages 31–99 of the second volume. The first edition was published in one volume in Paris, 1832, under the title “*Traité élémentaire de zoologie, ou histoire naturelle du règne animal*” [GAL] and noticed on 30 June 1832 (*Bibl Fr*); the Coleoptera are on pages 317–350 in this edition.

#### Prada, Teodoro (Rosate, Lombardy, Italy: 29 June 1815 – 4 June 1892: Pavia, Lombardy, Italy). Italian zoologist; professor of mineralogy and zoology at the University of Pavia; his collections are at the Museo Civico di Storia Naturale of Pavia which was merged recently to the museum of the University of Pavia. References. Conci and Poggi (1996: 318, P); Guaschi and Violani (2014, P).

**1857**. *Curculioniti dell’agro Pavese*. Fratelli Fusi, Pavia. 67 pp. (8vo) [DP: 1857 (title page), 4 November 1857 (*Zool-Bot Gesell Wien*), January–June 1858 (*Bibliot Hist-Nat*)] YB (ph)

The work contains the descriptions of eight species, all of them provided by Virginio Betta. Five of them were not described previously: *Smicronyx aericollis* (p. 51), *Smicronyx minusculus* (p. 51), *Gymnetron areolatus* (p. 61), *Gymnetron flavipes* (p. 62), and *Mecinus sericatus* (p. 62). These species should be credited to Betta.

Villa (1860) provided a review of this work.

#### Precht, Karl Heinrich (Riga, Latvia: 1 April 1777 – 30 June 1819: Riga, Latvia). Baltic pastor and naturalist; deacon in Riga. References. Anonymous (1820a); Recke and Napiersky (1831: 443–444).

**1818**. *Verzeichniss der bis jetzt, vornehmlich in der Umgegend von Riga, und im Rigischen Kreise bekannt gewordenen und systematisch bestimmten käferartigen Insecten. (Coleoptera Linnaei, Eleutherata Fabricii.) Bei Gelegenheit eines merkwürdigen Amts-Jubelfestes dem Druck übergeben.* J.C.D. Müller, Riga. 39 pp. (4to) [DP: 1818 (title page; recto of title page dated 30 January 1818), 2 July 1818 (*Rigasche Stadt-blätter*), February 1819 (*Allg Lit Ztg*)] YB (ph)

This work, essentially a checklist of the beetles in the vicinity of Riga, was published anonymously. A number of new species are named but not described.

#### Preller, Carl Heinrich (Lübeck, Schleswig-Holstein, Germany: 20 February 1830 – 2 July 1890: Hamburg, Hamburg, Germany). German teacher and entomologist; director of an institute for boys near Eutin (1854–1856), then at Preetz (1856–1860) in northern Germany; private teacher in Hamburg; his collection of Coleoptera was deposited at the Zoologische Museum der Universität Hamburg where it was destroyed during WWII. Reference. Schröder and Klose (1873: 120–121).

**1861**. *Beiträge zu einem natürlichen System der Coleopteren.* Friedrich Frommann, Jena. 46 pp. (8vo) [DP: 1861 (title page), 14 March 1861 (*Allg Bibl Deutsch*), April 1861 (*Allg Bibl*), January–June 1861 (*Bibliot Hist-Nat*)] GB

**1862**. *Die Käfer von Hamburg und Umgegend. Ein Beitrag zur nordalbingischen Insektenfauna.* Otto Meissner, Hamburg. xii + 158 pp. + 1 pl. (8vo) [DP: 1862 (title page; *Vorwort* dated December 1861), 13 March 1862 (*Allg Bibl Deutsch*), March 1862 (*Allg Bibl*), 14 June 1862 (*Lit Centr Deutsch*), June 1862 (*Wien Ent Monats*), January–June 1862 (*Bibliot Hist-Nat*)] CMLE, GB, BHL, MDZ

This work consists of a list, with short annotations, of the beetles of Hamburg and surrounding areas. Several new species-group taxa are described.

A second edition, issued in 1867 by the same publisher, contains xii + 227 + [1] pages (*n.v.*); it was recorded on 2 October 1867 (*Börsen Deutsch Buch*).

#### Preudhomme de Borre, Charles François Paul Alfred (Jemeppe sur Meuse [currently part of Seraing], Belgium: 14 April 1833 – 27 February 1905: Grand-Saconnex near Geneva, Switzerland). Belgian entomologist; alderman of the commune of Jemeppe (1861–1866); curator at the Musée Royal d’Histoire Naturelle in Brussels (1869–1889); later, moved to Switzerland; bequeathed his large collection to the Muséum d’Histoire Naturelle de Genève. References. Anonymous (1905, P); Lameere (1906, P); Sarasin (1906, P).

**1882–1891**. *Matériaux pour la faune entomologique de la province de Limbourg. Coléoptères.* M. Collée, Tongres [*centuries* 1–2]; W. Klock, Hasselt [*centuries* 3–4]. (8vo) MCZ

*Première centurie.* 32 pp. [DP: 1882 (title page), 29 October 1882 (*Acad R Sci Belg*), 1 November 1882 (*Feuille Jeunes Nat*), 22 November 1882 (*Soc Ent Fr*), 27 November 1882 (*Zool Anz*), December 1882 (*Bibl Belg*; *Nat Nov*)].

*Deuxième centurie.* 46 pp. [DP: 1882 (title page), 1 February 1883 (*Acad R Sci Belg*), 2 April 1883 (*Zool Anz*), April 1883 (*Bibl Belg*), June 1883 (*Nat Nov*), January–June 1883 (*Bibliot Hist-Nat*)].

*Troisième centurie.* 50 pp. [DP: 1890 (title page), March 1990 (*Bibl Belg*), 10 May 1890 (*Soc R Belg Géog*), 5 June 1890 (*Acad R Sci Belg*), 7 June 1890 (*Soc R Malac Belg*), August 1890 (*Nat Nov*), 29 September 1890 (*Zool Anz*), 22 October 1890 (*Soc Ent Fr*), 28 October 1890 (*Soc Zool Fr*)]. The title on the wrappers for this and next *centuries* is “*Matériaux pour la faune entomologique du Limbourg*. *Coléoptères*.”

*Quatrième centurie.* 57 pp. [DP: 1891 (title page), 4 June 1891 (*Acad R Sci Belg*), 6 June 1891 (*Soc R Malac Belg*), June 1891 (*Bibl Belg*; *Nat Nov*), 14 October 1891 (*Soc Ent Fr*)].

Preudhomme de Borre published several coleopterological accounts of Belgian provinces; this is the only one that was not also published in journals.

#### Preyssler [Preysler], Johann [Jan] Daniel Eduard (Prague, Czech Republic: 1768 – 23 April 1839: Prague, Czech Republic). Bohemian naturalist; inspector and surveyor of mines in Prague; his Coleoptera collection is at the Natural History Museum in Prague. References. Eiselt (1836: 64–65); Evenhuis (1997b: 626).

**1790**. *Verzeichniss böhmischer Insekten. Erstes Hundert mit zwei Kupfertafeln.* Schoenfeld-Meissner, Prag. 108 pp. + 2 pls. (4to) [DP: 1790 (title page), 20 September 1790 (*Gött Anz*)] SME, GB

Preyssler’s work presents the descriptions of one hundred Bohemian insects. A few of these species are new to science as well as one new beetle genus (*Claviger*, p. 68).

**1791**. Beschreibungen und Abbildungen derjenigen Inseckten, welche in Sammlungen nicht aufzubewahren sind, dann aller, die noch ganz neu, und solcher, von denen wir noch keine oder doch sehr schlechte Abbildung besitzen. Erste Sammlung. Pp. 55–152 *in*: *Sammlung physikalischer Aufsätze, besonders die Böhmische Naturgeschichte betreffend, von einer Gesellschaft Böhmischer Naturforscher; herausgegeben von Dr. Johann Mayer*. *Mit Kupfern.* Walther, Dresden. [2] + 270 + [2] pp. + 7 pls (shared with volume 2). (8vo) [DP: 1791 (title page), July 1791 (*Berg J*), 19 November 1791 (*Gött Anz*)] MCZ, BHL, GB

The new Coleoptera described are *Cantharis leucogastra* (p. 58) and *Silpha denticulata* (p. 65).

**1792**. Beschreibungen und Abbildungen derjenigen Inseckten, welche in Sammlungen nicht aufzubewahren sind, dann aller, die noch ganz neu, und solcher, von denen wir noch keine oder doch sehr schlechte Abbildungen besitzen. (Fortsetung von I. B. Seite 55.) Vierte Sammlung. Pp. 1–46 *in*: *Sammlung physikalischer Aufsätze, besonders die Böhmische Naturgeschichte betreffend, von einer Gesellschaft Böhmischer Naturforscher; herausgegeben von Dr. Johann Mayer*. *Zwenter Band. Mit Kupfern.* Walther, Dresden. [2] + 361 pp. + 7 pls (shared with volume 1). (8vo) [DP: 1792 (title page), July 1792 (*Berg J*), 18 October 1792 (*Gött Anz*)] MCZ, BHL

The new Coleoptera species described are *Hister sesquicornis* (p. 3) and *Elater quadripustulatus* (p. 8).

**1793**. [Preyssler, J.D.E., Lindacker, J.C. and Hoser, J.K.] Beobachtungen über Geganstände der Natur auf einer Reise durch den Böhmer Wald im Sommer 1791. Pp. 135–378 *in*: *Sammlung physikalischer Aufsätze, besonders die Böhmische Naturgeschichte betreffend, von einer Gesellschaft Böhmischer Naturforscher; herausgegeben von Dr. Johann Mayer. Dritter Band, mit Kupfern.* Walther, Dresden. [2] + 408 pp. + 3 pls. (8vo) [DP: 1793 (title page), April 1793 (*Berg J*)] GB

Several Coleoptera species are described by Preyssler including new ones like *Leptura dispar* (p. 179) and *Carabus truncatus* (p. 373).

#### Procházka, Johann. Headmaster of a public school in Mistek, Moravia; later, headmaster of a school for girls in Witkowitz (currently Ostrava), Moravia.

**1894**. *Bestimmungs-Tabelle der europäischen Coleopteren: Cantharidae. II. Theil: Genus Danacaea. XXX. Heft. (Mit einer Tafel.).* Brünn. 30 + [1] pp. + 1 pl. (8vo) [DP: 1894 (title page), 22 May 1895 (*Soc Ent Fr*), May 1895 (*Nat Nov*)] SME

The title on page [7] reads “*Revision der Coleopteren-Gattung Danacaea Laporte aus der paläarctischen Fauna.*”

This work was also issued in *Verhandlungen des Naturforschenden Vereins in Brünn* 33 [1894]: 7–35 [DP: 1895 (title page), January 1896 (*Nat Nov*)].

#### Provancher, Léon (Courtnoyer [currently in Bécancour], Quebec, Canada: 10 March 1820 – 23 March 1892: Cap-Rouge [currently part of Quebec City], Quebec, Canada). French Canadian priest, editor, horticulturist, botanist, mycologist, entomologist, conchologist, and writer, best known for his work on Hymenoptera; ordained at the age of 24 and for the next 25 years worked in various parishes in Quebec; retired from his sacerdotal duties in 1869, the year he founded *The Naturaliste Canadien*, the first French scientific journal in North America, and devoted the remaining of his life mostly to his entomological activities; travelled to Europe, Egypt and the Middle East in 1882 and again in 1884 where he had the opportunity to meet a number of entomologists; nicknamed by some the “Canadian Buffon” or the “Canadian Linné;” his collection was deposited at the Université Laval, Quebec. References. Huard (1926); Essig (1931: 734–735, P); Desmeules (2004, P).

**1877**. *Petite faune entomologique du Canada precedée d’un traite elementaire d’entomologie. Volume I – Les Coleopteres.* C. Darveau, Québec. viii + 785 + [1 (Errata)] pp. (8vo) [DP: 1877 (title page; preface dated January 1874), January 1877 (*Natur Can* 9: 305)] CNC, YB, GB

The first part of the systematic treatment (pp. 127–271), though slightly modified, was originally published in *Le Naturaliste Canadien*, volumes 4 [1872], 5 [1873], and 6 [1874]. Three supplements were published in *Le Naturaliste Canadien* 9 [1877]: 305–319, 321–338; 10 [1878]: 365–385; and 11 [1879]: 301–329.

The entire series was published in three volumes, 1877–1886; the second volume pertains to Orthoptera, Neuroptera and Hymenoptera, and the third one to Hemiptera.

#### Putzeys, Jules Antoine Adolphe Henri (Liège, Belgium: 1 May 1809 – 2 January 1882: Brussels, Belgium). Belgian civil servant, horticulturist and entomologist mainly interested in carabids (Coleoptera); head clerck, director and finally secretary-general at the Ministry of Justice (1840–1880); after his death, his European and exotic carabids were given by his sons to the *Société Entomologique de Belgique* who deposited them at the Musée Royal de Bruxelles. References. Preudhomme de Borre (1882); Dewalque (1905).

**1845**. *Prémices entomologiques.* H. Dessain, Liège. 64 + [1] pp. + 1 pl. (8vo) [DP: May 1845 (title page), 2 July 1845 (*Ent Ver Stettin*), 27 August 1845 (*Soc Ent Fr*), 21 March 1846 [JD] (*Soc Imp Nat Moscou*)] MCZ, GB

The work contains two sections: 1. Note monographique sur le genre *Pasimachus* et sur un nouveau genre voisin (pp. 1–12); II. Descriptions de 62 espèces nouvelles de Coléoptères appartenant aux familles des Cicindélides et des Carabiques, avec l’indication des caractères de cinq nouveaux genres (pp. 13–64).

This work was also issued in the second part of the *Mémoires de la Société royale des Sciences de Liège* 2: 353–417 [DP: 1846 (title page)].

**1846**. *Broscosoma, carabidum genus novum, descr. atque fig. illustr.* D. Raes, Bruxellis. 7 pp. +1 pl. (8vo) [DP: October 1846 (title page)^88^, September 1846 (*Rev Zool*), 14 January 1847 (*Ent Ver Stettin*), 13 March 1847 [JD] (*Soc Imp Nat Moscou*), 5 May 1847 (*Ent Soc Lond*), 11 August 1847 (*Soc Ent Fr*)] MCZ

**1846**. *Monographie des Clivina et genres voisins.* H. Dessain, Liège. 145 pp. (8vo) [DP: January 1846 (title page), 6 March 1846 (*Ent Ver Stettin*), 11 March 1846 (*Soc Ent Fr*), September 1846 (*Rev Zool*)] MCZ, GB

This work was also issued in the second part of the *Mémoires de la Société Royale des Sciences de Liège* 2 (2): 521–663 [DP: 1846 (title page), 9 February 1846 (*Acad R Sci Belg*)] under the title “*Monographie des Clivina et genres voisins, précédée d’un tableau synoptique des genres de la tribu des scaritides.*”

**1861**. *Postscriptum ad clivinidarum monographiam atque de quibusdam aliis. Mense Novembris 1861.* H. Dessain, Leodii [= Liège]. 78 pp. + 2 pls. (8vo) [DP: 1862 (title page), November 1861 (Putzeys 1866: 1)] AMNH

Despite the title page reads “*Mense Novembris 1861*” the date listed at the bottom of the page is 1862. This work was also published in *Mémoires de la Société Royale des Sciences de Liège* 18: 1–78 [DP: 1863 (title page)].

#### Quensel, Conrad (Leyda, Skåne, Sweden: 10 December 1767 – 22 August 1806: Stockholm, Sweden). Swedish naturalist; worked at the Botanical garden of the University of Uppsala; intendant of the cabinet of natural history of the Royal Swedish Academy of Sciences in Stockholm; later, professor of chemistry and natural sciences at the military school in Stockholm. References. Catteau-Calleville (1823); Anonymous (1845a).

**1790**. *D.D. Dissertatio historico-naturalis ignotas insectorum species continens, cujus partem primam, Consent. Ampliss. Facult. Philisoph. publico examini modeste subjiciunt praeses Conrad. Quensel, philosophiae magister, et respondens Carolus P. Lundgård, Scani, d. XXI Jun. a. MDCCXC.* Berling, Lundae. 20 pp. (4to) [DP: 21 June 1790 (title page)] GDZ

The following 13 new species of Coleoptera are described in this thesis: *Dermestes collaris* (p. 8), *Dermestes marginellus* (p. 9), *Dermestes xanthocephalus* (p. 10), *Bruchus rufipes* (p. 11), *Bruchus serraticornis* (p. 12), *Silpha quadrimaculata* (p. 12), *Chrysomela binotata* (p. 14), *Curculio filirostris* (p. 15), *Curculio hypoleucus* (p. 16), *Curculio virescens* (p. 16), *Elater quercinus* (p. 17), *Carabus arboreus* (p. 18), and *Tenebrio planus* (p. 19).

#### Quoy, Jean René Constantin (Maillé near Maillezais, Vendée, France: 10 November 1790 – 4 July 1869: Rochefort, Charente-Maritime, France). French naturalist and physician; zoologist on the ship *L’Uranie* for the round-the-world trip under the command of Louis de Freycinet (1817–1820); naturalist on the ship *L’Astrolabe* under the command of Dumont-d’Urville (1826–1829); professor of anatomy and medicine at the Rochefort Naval School (1824–1835); posted at naval hospitals in Toulon (1835–1837) and Brest (1838–1848); inspector general of the navy health service (1848–1858); commander of the Legion of Honour. References. Richemond (1870); Noël (1960); Fardet (1995).

**1824–1826**. [Quoy, J.R.C. and Gaimard, J.P.] *Voyage autour du monde, entrepris par ordre du Roi, sous le ministère et conformément aux instructions de s. exc. M. le vicomte du Bouchage, secrétaire d’état au département de la marine, exécuté sur les corvettes de S. M. l*’Uranie *et la* Physicienne, *pendant les années 1817, 1818, 1819 et 1820; publié sous les auspices de S. E. M. le comte Corbière, secrétaire d’état de l’intérieur, pour la partie historique et les sciences naturelles, et de S. E. M. le marquis de Clermont-Tonnerre, secrétaire d’état de la marine et des colonies, pour la partie nautique; par M. Louis de Freycinet. Zoologie.* Pillet aîné, Paris. [4] + 712 pp. [+ 96 associated pls]. (4to–text; Folio–plates) MCZ, BHL

The *Zoologie* section of this round-the-world travels was published in 16 livraisons forming two volumes (pp. 1–376 and 377–712) of text (usually bound together) and one atlas of 96 plates of which 80 are colored. The dates of publication of the livraisons as recorded by the *Bibliographie de la France* and the *Académie des Sciences* are: **1**: (pp. 1–40 + 6 pls) 26 June 1824 (*Bibl Fr*); **2**: (pp. 41–88 + 6 pls) 26 July 1824 (*Acad Sci Fr*), 31 July 1824 (*Bibl Fr*); **3**: (pp. 89–128 + 6 pls) 28 August 1824 (*Bibl Fr*); **4**: (pp. 129–176 + 6 pls) 18 September 1824 (*Bibl Fr*), 4 October 1824 (*Acad Sci Fr*); **5**: (pp. 177–224 + 6 pls) 9 October 1824 (*Bibl Fr*); **6**: (pp. 225–272 + 6 pls) 20 November 1824 (*Bibl Fr*); **7**: (pp. 273–312 + 6 pls) 18 December 1824 (*Bibl Fr*), 17 January 1825 (*Acad Sci Fr*); **8**: (pp. 313–360 + 6 pls) 29 January 1825 (*Bibl Fr*), 7 March 1825 (*Acad Sci Fr*); **9**: (pp. 361–408 + 6 pls) 26 March 1825 (*Bibl Fr*), 4 April 1825 (*Acad Sci Fr*); **10**: (pp. 409–456 + 6 pls) 7 May 1825 (*Bibl Fr*); **11**: (457–496 + 6 pls) 18 June 1825 (*Bibl Fr*), 27 June 1825 (*Acad Sci Fr*); **12**: (pp. 497–536 + 6 pls) 27 June 1825 (*Acad Sci Fr*), 6 August 1825 (*Bibl Fr*); **13**: (pp. 537–576 + 6 pls) 1 October 1825 (*Bibl Fr*); **14**: (pp. 577–616 + 6 pls) 17 December 1825 (*Bibl Fr*); **15**: (pp. 617–664 + 6 pls) 26 April 1826 (*Bibl Fr*), 15 May 1826 (*Acad Sci Fr*); **16**: (pp. 665–712 + 6 pls) 14 June 1826 (*Bibl Fr*), 31 July 1826 (*Acad Sci Fr*). The title pages of both volumes are dated 1824 and the preface June 1824. The only species of Coleoptera described are *Cetonia bifasciata* (p. 548), *Curculio lemniscatus* (p. 549), and *Rhynchoenus doryphorus* (p. 550). They are illustrated on plate 82.

#### Rafinesque [Rafinesque-Schmaltz], Constantine Samuel (Constantinople [now Istanbul], Turkey: 22 October 1783 – 18 September 1840: Philadelphia, Pennsylvania, USA). Eccentric and controversial naturalist who published on almost every conceivable subjects although his main contributions were to botany; spent his youth chiefly in France and Italy and at the age of 18 sailed to Philadelphia where he lived for three years; moved to Sicily where he stayed for 14 years; in July 1815 sailed again for the United States where he landed nearly four months later without property, books, or collections as the vessel he boarded wrecked off at Long Island Sound; professor of botany at Transylvania University in Lexington, Kentucky (1819–1826); eventually drifted to Philadelphia where he died in great poverty. References. Jordan (1886); Call (1895, P); Richmond (1909: 37); Adler (1989: 25–26, P); Damkaer (2002: 157–159, P); Warren (2004, P).

**1814**. *Principes fondamentaux de somiologie ou les loix de la nomenclature et de la classification de l’empire organique ou des animaux et des végétaux contenant les règles essentielles de l’art de leur imposer des noms immuables et de les classer méthodiquement.* Franc. Abate, Palerme. 50 + [2] pp. (8vo) [DP: 1814 (title page)] GB, MDZ

This work includes a number of unjustified emendations of generic names; among the Coleoptera, *Apionus* (p. 29) was proposed in replacement of *Apion* Herbst.

**1815**. *Analyse de la nature ou tableau de l’univers et des corps organisés.* Palerme. 224 pp. (8vo) [DP: 1815 (title page), April–15 July 1815 (Thompson et al. 1999: 499), April–21 July 1815 (Stafleu and Cowan 1983: 557), April–July 1815 (Richmond 1909: 38), July 1815 (Ward et al. 1996: 306)] GAL, GB

Evenhuis (1997b: 630) explained the fate of this book, one of the rarest of natural history books. It was reprinted by Friedlander & Sohn, Berlin, in 1900 (*n.v.*). An English translation by Arthur J. Cain of this and other works of Rafinesque was published in 1990 in *Tryonia* 20: 1–243.

The work contains several unjustified emendations (or unnecessary replacement names) of Coleoptera genera: *Hesione* (p. 109) for *Lebia* [as *Lesbia*] Latr.; *Anthiana* (p. 109) for *Anthia* Fabr.; *Agrana* (p. 109) for *Agra* Fabr.; *Stenomus* (p. 110) for *Stenus* Latr.; *Dolopes* (p. 110) for *Proteinus* Latr.; *Cupesius* (p. 110) for *Cupes* Fabr.; *Alycus* (p. 110) for *Lycus* Fabr.; *Zygitis* (p. 110) for *Zygia* Fabr.; *Gryneus* (p. 111) for *Tilius* Ol.; *Cleronus* (p. 111) for *Clerus* Ol.; *Burecus* (p. 111) for *Sandalus* Latr.; *Hopliatus* (p. 111) for *Hoplia* Ill.; *Erutela* (p. 112) for *Rutela* Latr.; *Hydrolus* (p. 112) for *Hydrophilus* Geof.; *Stereolus* (p. 112) for *Throscus* Latr.; *Histeranus* (p. 112) for *Hister* L.; *Elminus* (p. 112) for *Elmis* Latr.; *Peltinus* (p. 112) for *Silpha* L.; *Cephacerus* (p. 113) for *Erodius* Fabr.; *Pimidia* (p. 113) for *Pimelia* Fabr.; *Stenopsis* (p. 113) for *Akis* Fabr.; *Espidum* (p. 113) for *Sepidium* Fabr.; *Scauris* (p. 113) for *Scaurus* Fabr.; *Trestonia* (p. 113) for *Toxicum* Latr.; *Lygophilus* (p. 114) for *Epitragus* Latr.; *Soradeus* (p. 114) for *Heleus* Latr.; *Oenasium* (p. 114) for *Oenas* Latr.; *Apalomus* (p. 114) for *Apalus* Fabr.; *Hypulus* (p. 114) for *Helops* Fabr.; *Pytholus* (p. 114) for *Pytho* Latr.; *Horianella* (p. 114) for *Horia* Fabr.; *Rhinomacus* (p. 115) for *Rhinomacer* Fabr.; *Cylanus* (p. 115) for *Cylas* Latr.; *Apionus* (p. 115) for *Apion* Herbst; *Rhinostomus* (p. 115) for *Rhina* Latr.; *Oxyrinus* (p. 115) for *Oxystoma* Dum.; *Langura* (p. 116) for *Languria* Latr.; *Psoanus* (p. 116) for *Psoa* Herbst; *Cryptophagus* (p. 116) for *Cis* Latr.; *Chlamisus* (p. 116) for *Chlamys* Latr.; *Clythraria* (p. 116) for *Clythra* Fabr.; *Daunus* (p. 117) for *Spondylis* Fabr.; *Clytanus* (p. 117) for *Clytus* Fabr.; *Latmius* (p. 117) for *Rhagium* Fabr.; *Sagrania* (p. 117) for *Sagra* Fab.; *Eurymus* (p. 117) for *Lycoperdina* Latr.

#### Rambur, Pierre-Jules (Ingrandes-de-Touraine, Indre-et-Loire, France: 21 July 1801 – 10 August 1870: Geneva, Switzerland). French physician and entomologist interested mainly in Lepidoptera and Neuroptera but also in Coleoptera; investigated Corsica and Andalusia; founding member of the *Société Entomologique de France*; his collection passed to Paul Mabille, his nephew, and eventually was acquired by René Oberthür [*q.v.*]. References. Graslin (1872); Lhoste (1987: 120–121); Cambefort (2006: 276).

**1837–1838**. *Faune entomologique de l’Andalousie. Deux forts volumes in-8, accompagnés de 50 planches dessinées et gravées par d’habiles artistes, tirées en colour et terminées au pinceau avec le plus grand soin.* [*Volume I*]. Arthus Bertrand, Paris. 144 pp. + 4 [numbered 1, 2, 19, 20] pls. (8vo) MCZ, GB, MDZ

According to the prospectus dated October 1837, the entire work was to be completed in ten livraisons. However only five livraisons (Higgins 1958: 311), bound in two volumes, were published. The Coleoptera are in the first volume, issued in two livraisons: **1**: (pp. 1–80 + pls 1–2) December 1837 (back of cover), 30 December 1837 (*Feuil J Lib*)^89^, 27 January 1838 (*Bibl Fr*), 31 January 1838 (*Lit Ztg*), 9 February 1838 (*Allg Bibl Deutsch*); **2**: (pp. 81–144 + pl. 19) July 1838 (Higgins 1958: 313). According to Higgins (1958: 313), the publishing date of March 1838 for the second livraison given by Mabille (1872: 309) is in error. Plate 20, containing illustrations of various species of *Asida*, was issued in livraison 3, issued in December 1838 (Higgins 1958: 314). Higgins (1958: 313) gives a list of the new names proposed in each livraison along with the species illustrated on the plates.

#### Ratzeburg, Julius Theodor Christian (Berlin, Germany: 16 February 1801 – 24 October 1871: Berlin, Germany). German forester and naturalist; private lecturer at the University of Berlin; professor of natural history at the Forestry School in Eberswalde; founded the botanic garden of forestry at Eberswalde where he worked until his retirement in 1869; his collection was donated to the Deutsches Entomologisches Institut, now in Müncheberg, but most specimens were destroyed during World War II. References. Danckelmann (1872, P); Ratzeburg (1874: 421–429); Judeich and Nitsche (1885: 1–6, P); Schwerdtfeger (1972).

**1837**. *Die Forst-Insecten oder Abbildung und Beschreibung der in den Wäldern Preussens und der Nachbarstaaten als schädlich oder nützlich bekannt gewordenen Insecten; in systematischer Folge und mit besonderer Rücksicht auf die Vertilgung der Schädlichen. Erster Theil. Die Käfer. Mit 22 theils in Kupfer gestochenen theils lithographirten Tafeln und vielen Holzschnitten.* Nicolai, Berlin. x + [4] + 202 pp. + 22 pls. (4to) [DP: 1837 (title page; *Vorrede* dated April 1837), 3 November 1837 (*Allg Bibl Deutsch*), 8 November 1837 (*Lit Ztg*), 20 December 1837 [JD] (*Soc Imp Nat Moscou*), July–December 1837 (*Verz Bücher Landkarten*), 2 February 1838 [JD] (*Acad Imp Sci St-Pétersb*), 6 April 1838 (*Allg Forst Jagd Ztg*), 30 April 1838 (*Acad Sci Fr*)] CNC, GB

The entire work includes three volumes, 1837–1844. The Coleoptera are in the first volume. A second edition of the volume on Coleoptera was issued in 1839 (next entry).

**1839**. *Die Forst-Insecten oder Abbildung und Beschreibung der in den Wäldern Preussens und der Nachbarstaaten als schädlich oder nützlich bekannt gewordenen Insecten; in systematischer Folge und mit besonderer Rücksicht auf die Vertilgung der Schädlichen. Erster Theil. Die Käfer. Mit 22 theils in Kupfer gestochenen theils lithographirten Tafeln und vielen Holzschnitten. Zweite mit Zusätzen und Berichtigungen vermehrte Auflage.* Nicolai, Berlin. xvi + 247 + [4] pp. + 22 pls. (4to) [DP: 1839 (title page; *Vorwort* dated July 1839), 23 October 1839 (*Lit Ztg*), 25 October 1839 (*Allg Bibl Deutsch*), July–December 1839 (*Verz Bücher Landkarten*)] GB, BHL

A “*Erster Nachtrag zu Ratzeburg’s Forst-Insecten Band I. (Käfer) oder Veränderungen der zweiten Ausgabe aus der zweiten Ausgabe desselben Werkes besonders abgedruckt. Mit mehreren Holzschnitten*” consisting of iv + [4] + 55 + [1] pages, was published in Berlin in 1839 [GB] and noticed on 29 November 1839 (*Allg Bibl Deutsch*).

#### Raulin, Victor (Paris, France: 8 August 1819 – 10 February 1905: Montfaucon-d’Argonne, Meuse, France). French paleobotanist and geologist; professor of geology, mineralogy and botany at the Faculty of Science of Bordeaux. References. Micé (1905); Zimmermann (1905: 178–179); Legée (1979).

**1869**. *Description physique et naturelle de l’ile de Crète. Itinéraire, histoire, population, agriculture, industrie, commerce, géographie physique et mathématique, physique du sol, géologie, botanique, zoologie. Publiée sous les auspices de M. le Ministre de l’Instruction Publique. Tome second.* Arthus Bertrand, Paris. viii + pp. 463–1078. (8vo) [DP: 1869 (title page), 23 April 1870 (*Bibl Fr*), 25 April 1870 (*R Geog Soc*), 30 April 1870 (*Feuil J Lib*), June 1870 (*Allg Bibl*)] GB

The entire series consists of two volumes and one atlas. The zoology section in the second volume includes a list of Coleoptera known from the island of Crete (pp. 1005–1013). Localities and sometimes brief habitat data are listed for the species collected by Raulin during his travels to Crete in 1845. All the specimens were determined by Pierre Hyppolyte Lucas who also described the new species from Raulin’s trip in 1853 and 1854 in *Revue et Magasin de Zoologie Pure et Appliquée*. The entire section on zoology was also issued in volume 24 (5th and 6th livraisons) of the *Actes de la Société Linnéenne de Bordeaux* [DP: 25 February 1870 (p. 770)]; the Coleoptera are on pages 673–681.

#### Razumovsky [Razoumowsky, Razumovskij], Grigory [Gregor] Kirillovich von (Count) (Saint Petersburg, Russia: 10 November 1759 – 3 June 1837: Český Rudolec, Czech Republic). Russian political philosopher, naturalist, mineralogist, and geologist; studied in Leiden; lived in Switzerland from 1782 to 1793. References. Minina (2005); Weidmann (2011, P).

**1789**. *Histoire naturelle du Jorat et de ses environs; et celle des trois lacs de Neufchatel, Morat et Bienne; précédées d’un essai sur le climat, les productions, le commerce, les animaux de la partie du pays de Vaud ou de la Suisse Romande, qui entre dans le plan de cet ouvrage. Tome premier.* Jean Mourer, Lausanne. xvi + 322 pp. + 3 pls. (8vo) [DP: 1789 (title page), 7 May 1789 (*R Soc Lond*)] CAKL, GB

The entire work consists of two volumes with their title pages dated 1789. The beetles are treated on pages 134–173 and 289–293 of the first volume.

The second volume of this work was received by the *Accademia delle Scienze di Torino* on 4 January 1789 (*Acad R Sci Turin* 1790: cxiv) suggesting that the first volume, and possibly both, may have been published in 1788.

#### Redtenbacher, Ludwig (Kirchdorf an der Krems, Upper Austria, Austria: 10 July 1814 – 8 February 1876: Vienna, Austria). Austrian coleopterist; studied medicine at the University of Vienna; professor of zoology at the University of Prague; worked at the insect collection of the Imperial Natural History Collection (the future Natural History Museum) in Vienna as research assistant, adjunct curator, curator, chairman, and director; his collection of Austrian Coleoptera is at the Naturhistorisches Museum in Vienna. References. Wurzbach (1873: 121); Westwood (1877: xliii–xliv); Klausnitzer (2003: 92–96, P).

**1843**. *Tentamen dispositionis generum et specierum coleoptrorum pseudotrimerorum Archiducatus Austriae. Dissertatio inauguralis.* Caroli Ueberreuter, Vindobonae [= Vienna]. 32 pp. (8vo) [DP: 1843 (title page)] GB

This thesis treats the lycoperdinines (currently in the family Endomychidae) and coccinellides of the archduchy of Austria, roughly corresponding to the current states of Lower Austria and Upper Austria; three new genera and several new species are described.

Some bibliographers, including Hagen (1863: 66), date the thesis 1844. However, the copy I have seen has the date 1843 on the title page and Erichson (1844: 293) discussed the work in his review of the entomological literature for 1843. This dissertation was issued again in 1844 in *Zeitschrift für die Entomologie* 5: 113–132.

**1843**. Zoologie. Entomologie. Pp. 971–990 *in*: *Reise in Griechenland, Unteregypten, im nördlichen Syrien und südöstlichen Kleinasien, mit besonderer Rücksicht auf die naturwissenschaftlichen Verhältnisse der betreffenden Länder, unternommen in dem Jahre 1836, von Joseph Russegger. Zweiter Theil. Mit 2 Karten vom Taurus, 1 Blatt mit Gebirgs-Durchschnitten, 20 botanischen und 15 zoologischen Tafeln.* E. Schweizerbart, Stuttgart. Pp. 471–1102. (8vo) [DP: 1843 (title page)] MCZ, MDZ, GB

The entire report of Joseph Russegger’s expedition was published in seven volumes of text, issued in parts (*Abtheilungen*), and one atlas, 1841–1849. The second *Theil* was issued as *Abtheilungen* 3 and 4 (pp. 471–870) noticed on 18 November 1842 (*Allg Bibl Deutsch*) and *Abtheilung* 5 (pp. 871–1102) noticed on 18 May 1843 (*Allg Bibl Deutsch*). Pages 973–978 of Redtenbacher’s contribution are entitled “*Bemerkungen über die in Syrien von Theodor Kotschy gesammelten Käfer*” and page 978 is dated 1 October 1842; pages 979–990 is the “*Coleopterorum Syriae genera et species novae*.”

The first two *Theile* are consecutively paginated and bound as *Erster Band* under the title page “*Reisen in Europa, Asien und Africa, mit besonderer Rücksicht auf die naturwissenschaftlichen Verhältnisse der betreffenden Länder, unternommen in den Jahren 1835 bis 1841, von Joseph Russegger. Mit einem Atlas, enthaltend: geographische und geognostische Karten, Gebirgs-Profile, Landschaften, Abbildungen aus dem Gebiete der Flora und Fauna. Ersten Band. Reise in Griechenland, Unteregypten, im nördlichen Syrien und südostlichen Kleiasten*.” The title page of the first *Band* is also dated 1843 but the content was issued 1841–1843 and noticed in *Allgemeine Bibliographie für Deutschland* from 17 December 1841 (*Abtheilung* 1) to 18 May 1843 (*Abtheilung* 5).

The title page for the atlas is dated 1848. Two plates pertain to Coleoptera and the species depicted are: **Tab. A**: 1. *Cymindis serie-punctata*; 2. *Cymindis adusta*; 3. *Scarites punctatostriatus*; 4. *Morio olympicus*; 5. *Procerus syriacus*; 6. *Carabus paphius*; 7. *Pristonychus crenatus*; 8. *Pristonychus quadricollis*; 9. *Feronia punctata*; 10. *Julodis intricata*; 11. *Julodis sulcata*; 12. *Chalcophora quadrioculata*; 13. *Malachius ephippiger*; 14. *Dasytes vulpinus*; 15. *Telopes dispar*. **Tab. B**: 16. *Onthophagus centromaculatus*; 17. *Onthophagus aleppensis*; 18. *Aphodius suturalis*; 19. *Amphicoma syriaca*; 20. *Amphicoma cupripennis*; 21. *Mylabris coeruleo-maculata*; 22. *Mylabris sexnotata*; 23. *Bruchus signatus*; 24. *Phytonomus pictus*; 25. *Tychius alboguttatus*; 26. *Mononychus syriacus*; 27. *Phytoecia humeralis*; 28. *Malacosoma thoracica*; 29. *Clythra aleppensis*; 30. *Clythra unifasciata*; 31. *Clythra lineola*.

Redtenbacher’s contribution was also issued the same year, by the same publisher, in “*Abbildungen und Beschreibungen neuer und seltener Thiere und Pflanzen in Syrien und im westlichen Taurus gesammelt von Th. Kotschy. Herausgegeben von den DD. Fenzl, Heckel & Redtenbacher*” [GB].

**1845**. *Die Gattungen der deutschen Kaefer-Fauna nach der analytischen Methode bearbeitet, nebst einem kurz gefassten Leitfaden, zum Studium dieses Zweiges der Entomologie. (Mit zwei Kupfertafeln).* Carl Ueberreuter, Wien. [12] + 177 + [1 (Verbesserungen)] pp. + 2 pls. (8vo) [DP: 1845 (title page; preliminaries dated July 1845), September 1845 (*Ent Ztg* 6: 295), 2 October 1845 (*Allg Bibl Deutsch*), 8 October 1845 (*Lit Ztg*), 15 October 1845 (*Intell Serapeum*), 31 October 1845 (*Leip Reper*), October–December 1845 (*Foreign Quart Rev*), July–December 1845 (*Verz Bücher Landkarten*)] CNC, GB

**1847–1849**. *Fauna Austriaca. Die Käfer. Nach der analytischen Methode.* Carl Gerold, Wien. xxvii + 883 pp. + 2 pls. (8vo) CNC, GB

This book was published in five *Hefte*: **1**: (pp. 1–160) 9 July 1847 (*Freun Naturwiss*), 14 July 1847 (*Lit Ztg*), 15 July 1847 (*Allg Bibl Deutsch*), 6 August 1847 (*Leip Reper*), 8 October 1847 (*Ent Ver Stettin*); **2**: (pp. 161–320) 30 July 1847 (*Freun Naturwiss*), 15 September 1847 (*Lit Ztg*), 23 September 1847 (*Allg Bibl Deutsch*), 8 October 1847 (*Ent Ver Stettin*), 12 November 1847 (*Leip Reper*); **3**: (pp. 321–480) 15 October 1847 (*Freun Naturwiss*), 9 December 1847 (*Allg Bibl Deutsch*), 11 February 1848 (*Leip Reper*), 4 March 1848 (*Ent Ver Stettin*); **4**: (pp. 481–640) 9 February 1848 (*Lit Ztg*), 24 February 1848 (*Bibl Univers*), 30 March 1848 (*Allg Bibl Deutsch*), April 1848 (*Ent Ztg* 9: 128), 22 June 1848 (*Ent Ver Stettin*), 23 June 1848 (*Leip Reper*); **5**: (pp. 641–883, i–xxvii) 31 May 1849 (*Intell Serapeum*), 7 June 1849 (*Lit Ztg*), July 1849 (*Ent Ztg* 10: 223). The *Vorrede* in the last *Heft* is dated March 1849.

The section “*Systematisches Verzeichniss aller in diesem Werke enthaltenen Arten*” (pp. 831–874) was also issued separately in 1849 under the title “*Systematisches Verzeichniss der deutschen Käfer als Tauschkatalog eingerichtet*” [GB].

**1856–1858**. *Fauna Austriaca. Die Käfer. Nach der analytischen Methode. Zweite, gänzlich umgearbeitete, mit mehreren Hunderten von Arten und mit der Charakteristik sämmtlicher europäischen Käfergattungen vermehrte Auflage. Mit zwei Kupfertafeln.* Carl Gerold’s Sohn, Wien. cxxxvi + 1017 pp. + 2 pls. (8vo) CNC, GB, BHL

This second edition was published in nine *Hefte*: **1**: (pp. 1–128) 24 September 1856 (Zerche 1987b: 137), 30 October 1856 (*Allg Bibl Deutsch*), November 1856 (*Lit Deutsch Natur Ztg*), 3 December 1856 (*Zool-Bot Gesell Wien*), July–December 1856 (*Bibliot Hist-Nat*), October–December 1856 (*Cat Muquardt*), February 1857 (*Allg Bibl*); **2**: (pp. 129–256) 25 December 1856 (*Allg Bibl Deutsch*), October–December 1856 (*Cat Muquardt*), 7 January 1857 (*Zool-Bot Gesell Wien*), 31 January 1857 (*Intell Serapeum*); **3–4**: (pp. 257–512) 3 June 1857 (*Zool-Bot Gesell Wien*), 25 June 1857 (*Allg Bibl Deutsch*), January–June 1857 (*Bibliot Hist-Nat*), 15 August 1857 (*Intell Serapeum*); **5–6**: (pp. 513–768) 1857 (wrapper), 4 November 1857 (*Zool-Bot Gesell Wien*), 8 November 1857 (*Ent Ver Stettin*), 26 November 1857 (*Allg Bibl Deutsch*), July–December 1857 (*Bibliot Hist-Nat*), 5 January 1858 (*Acad Nat Sci Phila*), 15 February 1858 (*Intell Serapeum*); **7–8**: (pp. 769–1008) 5 May 1858 (*Zool-Bot Gesell Wien*), 3 June 1858 (*Allg Bibl Deutsch*), June 1858 (*Allg Bibl*), 3 August 1858 (*Acad Nat Sci Phila*); **9**: (pp. i–cxxxvi + 1009–1017 + 2 pls) 26 August 1858 (*Allg Bibl Deutsch*), 31 August 1858 (*Intell Serapeum*), 21 September 1858 (*Acad Nat Sci Phila*), 6 October 1858 (*Zool-Bot Gesell Wien*), October 1858 (*Allg Bibl*). The title page is dated 1858 and the *Vorrede* May 1858.

[**1868**]. *Reise der Österreichischen Fregatte* Novara *um die Erde in den Jahren 1857, 1858, 1859 unter den befehlen des Commodore B. von Wüllerstorf-Urbair. Zoologischer Theil. Zweiter Band. Coleopteren. Mit Fünf Tafeln.* Karl Gerold’s Sohn, Wien. iv + 249 pp. + 5 pls. (4to) [DP: 1867 (title page), 22 May 1868 (*Kaiser Akad Wiss*; Higgins 1963a: 158), 13 August 1868 (*Allg Bibl Deutsch*), August 1868 (*Allg Bibl*), 2 September 1868 (*Asia Soc Beng*), July–December 1868 (*Bibliot Hist-Nat*), December 1868 (*Leopoldina* 6: 100)] CNC, GB

Despite the title page is dated 1867, this publication was apparently not issued before 1868.

The entire report of the expedition was divided in eight sections (see Spitzka 1877), 1861–1875. The zoology section consists of two *Bande* and 228 plates, issued 1864–1875. The second *Band* is treated in three *Abtheilungen* and the first *Abtheilung* consists of two separate divisions, A and B. The Coleoptera are in division A along with the Hymenoptera by Henri de Saussure, Formicidae by Gustav L. Mayr and Neuroptera by Friedrich Brauer. A title page, dated 1868, was issued for division A; the title is identical to the one listed here except for the end which reads “*Zoologischer Theil. Zweiter Band. I. Abtheilung A.*” [GB]. The work came with black and white or colored figures.

**1871–1874**. *Fauna Austriaca. Die Käfer, nach der analytischen Methode. Dritte, gänzlich umgearbeitete und bedeutend vermehrte Auflage.* Carl Gerold’s Sohn, Wien. (8vo) CNC, GB

*Erster Band. Mit zwei Kupfertafeln.* 564 pp. This volume was issued in five parts in 1871 and 1872 (Rye 1874: 236). I have seen notices for only the first two parts, the first one (pp. 1–112) on 15 June 1871 (*Allg Bibl Deutsch*) and 15 August 1871 (*Amer Publ Circ*) and the second one (pp. 113–224) on 19 October 1871 (*Allg Bibl Deutsch*) and December 1871 (*Allg Bibl*). However, the entire volume was published by 15 September 1872 as noted in *Petites Nouvelles Entomologiques*. The title page is dated 1874 as for the second *Band*.

*Zweiter Band.* cliii + 571 + [1 (Druckfehler)] pp. + 2 pls. This volume was issued in parts as follows: (pp. 1–?224) 1 June 1873 (*Pet Nouv Ent*); (pp. ?225–?448) 15 August 1873 (*Academy*); (pp. ?449–571 + i–cliii + 2 pls) 1 January 1874 (*Pet Nouv Ent*), 4 February 1874 (*Zool-Bot Gesell Wien*). The title page is dated 1874 and the *Vorrede* [p. xii] September 1873.

A French translation of the identification keys to the families and genera, adapted for the Belgian fauna, was published under the title “*Tables dichotomiques pour servir a la détermination des familles et des genres de coléoptères d’Europe d’après L. Redtenbacher*” by M. Weissenbruch, in Brussels, 1878 (*n.v.*) and reissued in 1884, under the title “*Tables dichotomiques pour servir a la détermination des familles & des genres des coléoptères de Belgique d’après L. Redtenbacher*” [GAL] by Gustave Mayolez in Brussels.

#### Redtenbacher, Wilhelm (Kirchdorf an der Krems, Upper Austria, Austria: 29 May 1817 – 8 October 1870: Vienna, Austria). Austrian physician in Vienna; brother of Ludwig Redtenbacher [*q.v.*]. Reference. Schlager (1870).

**1842**. *Dissertatio inauguralis entomologica sistens quaedam genera et species coleopterorum Archiducatus Austriae nondum descriptorum, quam consensu et auctoritate illustrissimi ac magnifici domini praesidis et directoris, perillustris ac spectabilis Domini Decani, nec non clarissimorum ac celeberrimorum D.D. Professorum pro doctoris medicinae laurea summisque in medicina honoribus et privilegiis rite et legitime consequendis in antiquissima ac celeberrima Universitate Vindobonensi publicae disquisitioni submittit Guilielmus Redtenbacher, Kirchdorfensis ex Austria superiori. Disputabitur publice in theses adnexas in Universitatis aedibus die mensis Januarii 1842.* Caroli Ueberreuter, Vindobonae [= Vienna]. 31 pp. (8vo) [DP: January 1842 (title page)] GB

This thesis consists of descriptions of 26 new species of Coleoptera from the archduchy of Austria; two new genera are also described. It was also issued the same year by the same publisher under the title “*Quaedam genera et species coleopterorum Archiducatus Austriae nondum descriptorum. Dissertatio inauguralis*” [CNC, GB, BHL].

#### Reich, Gottfried Christian (Wunsiedel, Bavaria, Germany: 19 July 1769 – 5 January 1848: Berlin, Germany). German physician and naturalist; professor of medicine at the University of Erlangen; later, professor at the University of Berlin. Reference. Pagel (1888).

**1797**. *Mantissae insectorvm iconibvs illvstratae species novas avt nondvm depictas exhibentis. Fascicvlvs I.* Felsecker, Noribergae [= Nürnberg]. 16 pp. + 1 pl. (8vo) [DP: 1797 (title page; *lectori* dated August 1797)] GB, GDZ

This work consists of the descriptions of 12 new species of *Curculio*. Only the first fascicle was published. Hagen (1863: 66) mentioned, based on Sturm, another printing issued the same year in Erlangen but it probably does not exist.

#### Reiche, Louis Jérome (Gerinchem, South Holland, Netherlands: 20 December 1799 – 16 May 1890: Neuilly-sur-Seine, Hauts-de-Seine, France). French (Dutch-born) merchant and manufacturer in Paris and entomologist; founding member of the *Société Entomologique de France*; sold his collection by lots and his large library after the War of 1870 because of financial difficulties. References. Brisout de Barneville (1891, P); Gouillard (2004: 44, P).

**1850**. Entomologie. Pp. 257–471 *in*: *Voyage en Abyssinie dans les provinces du Tigré, du Samen et de l’Ahmara. Dédié à S. A. R. Monseigneur le Duc de Nemours par MM. Ferret et Galinier. Tome troisième. Publié par ordre du gouvernement.* Paulin, Paris. 536 pp. (8vo) GB, BHL, GAL

The expedition report was published in three volumes of text and one atlas of 50 plates. The third volume, containing the entomology section, was issued in three parts (Sherborn and Woodward 1901b: 161) as follows: (pp. 1–84) 1847; (pp. 85–224) 1848; (pp. 225–536) 1850^90^. The title page is dated 1847 but the cover 1848.

An atlas [BHL] in Folio containing nine maps and 17 plates on botany and 33 on zoology was issued. Plates 16–33 pertain to insects (pls 16–26 to Coleoptera) and scientific names are listed on them; they are assumed to have been published along with the text in 1850 despite that the title page of the Atlas is dated 1847–1848.

**1854**. *Catalogue des espèces d’insectes coléoptères recueillies par M. F. de Saulcy pendant son voyage en Orient.* Gide et J. Baudry, Paris. iv + 19 pp. (4to) [DP: 1854 (title page), 11 November 1854 (*Bibl Fr*), December 1854 (*Bibl Cathol*)] BOT, GB

Reiche’s booklet is part of “*Voyage autour de la Mer Morte et dans les terres bibliques exécuté de Décembre 1850 à Avril 1851 par F. de Saulcy*.” The expedition report was published by sections, each separately paginated and issued at different times.

**1872**. [Reiche, L. and Lallemant, C.] *Catalogue des coléoptères de l’Algérie et contrées voisines avec description d’espèces nouvelles par M.L. Reiche avec la collaboration principale de M. Lallemant et l’aide de plusieurs entomologistes.* F. Le Blanc-Hardel, Caen. 44 pp. [DP: 1872 (title page), July 1872 (*Abeille* 8: cxxxiii), 15 August 1872 (*Pet Nouv Ent*)] SME

This catalogue was also published in the *Mémoires de la Société Linnéenne de Normandie* **15** [1865–1869] (4): 1–44 [DP: 1869 (title page), 17 August 1872 (*Bibl Fr*)]. Despite that the title page is dated 1869, this journal volume was not issued before 1872.

An earlier version of this catalogue was issued in parts, under the title “Catalogue des Coléoptères de l’Algérie,” in the *Bulletin de la Société de Climatologie Algérienne* 5: 25–42, 139–158 [DP: 1868 (title page)]. The author listed on page 25 is [Charles] Lallemant but in a footnote, link to Brachinidae, on page 37 Lallemant stated “M.L. Reiche… qui, depuis longtemps, s’occupait de rédiger un catalogue des Coléoptères décrits de l’Algérie, vient de réunir son travail au mien, de sorte qu’à partir de ce moment, notre collaboration est commune” [M.L. Reiche who, for a long time, was occupied in writing a catalogue of the Coleoptera described from Algeria, has combined his work to mine, so that from this time our collaboration is shared]. On page 139 the authors listed are Lallemant and Reiche. The new species described, *Brachinus lethierryi* (page 39), is credited to Reiche alone in the text.

#### Reichenbach, Anton Benedict (Leipzig, Saxony, Germany: 7 July 1807 – 11 November 1880: Leipzig, Germany). German educator and naturalist in Leipzig; brother of Heinrich Gottlieb Ludwig Reichenbach [*q.v.*]. Reference. Hagen (1863: 69).

**1857**. *Der Käferfreund. Anleitung die Käfer zu sammeln und zu bestimmen, nebst Aufzählung und Beschreibung der bekanntesten europäischen, vorzüglich deutschen Arten, mit Andeutung ihres Nutzens oder Schadens und der Mittel, die schädlichen zu vertilgen. Ein Handbuch für Freunde der Käferkunde, so wie für Landwirthe und Forstleute insbesondere. Mit 204 Abbildungen auf 12 naturgetreu colorirten Tafeln.* Theodor Thomas, Leipzig. xv + [1] + 244 pp. + 12 pls. (8vo) [DP: n.d. (title page; *Vorrede* dated “Ostern 1857”), 13 August 1857 (*Allg Bibl Deutsch*), 25 October 1857 (*Leip Ztg*), July–December 1857 (*Bibliot Hist-Nat*)] GB, BHL

#### Reichenbach, Heinrich Gottlieb Ludwig (Leipzig, Saxony, Germany: 8 January 1793 – 17 March 1879: Dresden, Saxony, Germany). German botanist and ornithologist; director of the Dresden natural history museum and professor at the Surgical-Medical Academy in Dresden (1820–1862); director of the Dresden botanical gardens. References. Friedrich (1879); Hess (1888).

**1816**. *Monographia pselaphorvm. Dissertatio entomologica. Amplissimi philosophorvm ordinis auctoritate illvstris ictorvm ordinis concessv in avditorio ivridico D.I. IVNII CI*Ɔ*I*Ɔ*CCCXVI h.c. pvplice defendenda ab auctore Henrico Theophilo Lvdovico Reichenbach Lipsiensi. Adivncto socio Gvstavo Kvnze Lipsiensi. Cum Tabl. II. Aeneis.* Ioach. Bernh. Hirschfeld, Lipsiae [= Leipzig]. 77 + [3] pp. + 2 pls. (8vo) [DP: 1 June 1816 (title page)] ANSP, ARC

This thesis was also published the same year under the title “*Monographia pselaphorvm. Cum Tab. II. aeneis XXIII. specierum icones exhibentibus*” by Leopold Voss in Lipsiae [CNC, GB] and recorded on October 1816 (*Allg Lit Ztg*).

#### Reitter, Edmund (Müglitz [currently Mohelnice], Czech Republic: 22 October 1845 – 15 March 1920: Paskov, Czech Republic). Austrian coleopterist and insect dealer; moved to Vienna in 1879, to Mödling (near Vienna) in 1881 and to Paskov in 1891; published extensively and described over 1000 genera and 6400 species-group taxa; a large part of his collection, consisting of 250000 specimens including 4500 types, is at the Hungarian Natural History Museum in Budapest. References. Heikertinger (1920, P); Smetana and Herman (2001: 126–128, P); Klausnitzer (2003: 99–105, P); Cambefort (2006: 279–280).

**1882–1885**. *Naturgeschichte der Insecten Deutschlands begonnen von Dr. W.F. Erichson fortgesetzt von Prof. Dr. H. Schaum, Dr. G. Kraatz, H.v. Kiesenwetter, Jul. Weise und Edm. Reitter. Erste Abtheilung. Coleoptera. Dritter Band. Zweite Abtheilung.* Nicolai, Berlin. vi + 362 pp. (8vo) CNC, BHL

This volume was published in two *Lieferungen*: **1**: (pp. i–vi + 1–198) 1882 (wrapper), 19 August 1882 (*Academy*), August 1882 (*Nat Nov*), 26 October 1882 (*Naturwiss Ver Sach Thür*), 23 December 1882 (*Lit Centr Deutsch*), July–December 1882 (Griffin 1939a: 218), 27 January 1883 (*Deutsch Litt*); **2**: (pp. 199–362) 1885 (wrapper), March 1885 (*Nat Nov*), January–June 1885 (*Bibliot Hist-Nat*), 27 July 1885 (*Zool Anz*).

**1884**. *Bestimmungs-Tabellen der europäischen Coleopteren. XI. Bruchidae (Ptinidae auct.).* Brünn. 29 pp. (8vo) [DP: 1884 (title page), October 1884 (*Nat Nov*)] SME

This work was also issued in *Verhandlungen des Naturforschenden Vereins in Brünn* 22 [1883]: 295–323 [DP: 1884 (title page), February 1885 (*Nat Nov*)].

**1884**. *Bestimmungs-Tabellen der europäischen Coleopteren. XII. Necrophaga. (Platypsyllidae, Leptinidae, Silphidae, Anisotomidae und Clambidae).* Brünn. 122 pp. (8vo) [DP: 1885 (title page), end of 1884 (Reitter 1886: 219), February 1885 (*Nat Nov*)] SME

This work was also issued in *Verhandlungen des Naturforschenden Vereins in Brünn* 23 [1884]: 3–122 [DP: 1885 (title page), April 1886 (*Nat Nov*)].

A French translation was published in 1890 under the title “*Tableaux analytiques pour déterminer les coléoptères d’Europe. I Nécrophages Platypsillides, Leptinides, Silphides, Anisotomides, Clambides*” [BHL] as a supplement to *Revue Scientifique du Bourbonnais et du Centre de la France*.

**1885**. *Bestimmungs-Tabellen der europäischen Coleopteren. I. Heft. Enthaltend die Familien: Cucujidae, Telmatophilidae, Tritomidae, Mycetaeidae, Endomychidae, Lyctidae und Sphindidae. II. Auflage.* Edmund Reitter, Mödling. 45 pp. (8vo) [DP: 1885 (title page)] SME, GB

The first edition was published in 1880 in *Verhandlungen der k.k. zoologischen-botanischen Gesellschaft in Wien* 29 [1879]: 71–100.

**1886**. *Bestimmungs-Tabellen der europäischen Coleopteren. III. Heft. Enthaltend die Familien: Scaphidiidae, Lathridiidae und Dermestidae. II. Auflage.* Edmund Reitter, Mödling. 75 pp. (8vo) [DP: 1886 (title page), March 1887 (*Nat Nov*), 1887 (Derksen and Scheiding-Göllner 1968: 384)] SME

**1887**. *Bestimmungs-Tabellen der europäischen Coleopteren. XVI. Heft. Enthaltend die Familien: Erotylidae und Cryptophagidae.* Ed. Reitter, Brünn. 55 + [1 (Index generum)] pp. (8vo) [DP: 1887 (title page), September 1887 (*Nat Nov*)] SME

This work was also issued in *Verhandlungen des Naturforschenden Vereins in Brünn* 26 [1887]: 3–56 [DP: 1888 (title page), December 1888 (*Nat Nov*)]. A French translation was published in 1891 in *Coléoptériste* 1: 163–202.

**1892**. *Bestimmungs-Tabelle der Lucaniden und coprophagen Lamellicornen*. *XXIV. Heft.* Brünn. 230 pp. (8vo) [DP: 1892 (title page; preliminaries [p. 3] dated September 1892), June 1893 (*Nat Nov*)] SME

The title on page [3] reads “*Bestimmungs-Tabellen der lucaniden und coprophagen lamellicornen des palaearctischen Faunengebietes.*”

This work was also issued in *Verhandlungen des Naturforschenden Vereins in Brünn* 30 [1891]: 141–262 [DP: 1892 (title page), March 1893 (*Nat Nov*)] and 31 [1892]: 3–109 [DP: 1893 (title page), March 1894 (*Nat Nov*)]. A French translation was published, with separate pagination, in volumes 17–22 [1909–1915] of the journal *Miscellanea Entomologica*.

**1893**. *Bestimmungs-Tabelle der unechten Pimeliden aus der palaearctischen Fauna. XXV. Heft.* Brünn. 51 pp. (8vo) [DP: 1893 (title page), January 1894 (*Nat Nov*)] SME

This work was also issued in *Verhandlungen des Naturforschenden Vereins in Brünn* 31 [1892]: 201–250 [DP: 1893 (title page), March 1894 (*Nat Nov*)].

**1894**. *Bestimmungs-Tabelle der europäischen Coleopteren: Nitidulidae. I. Theil: Genus Epuraea Er. XXVII. Heft.* Brünn. 20 + [1] pp. (8vo) [DP: 1894 (title page), July 1894 (*Nat Nov*)] SME

The title on page [3] reads “*Analytische Uebersicht der europäischen Arten der Coleopteren-Gattung Epuraea Er.*”

This work was also issued in *Verhandlungen des Naturforschenden Vereins in Brünn* 32 [1893]: 18–36 [DP: 1894 (title page), January 1895 (*Nat Nov*)].

**1894**. *Bestimmungs-Tabelle der Coleopteren-Familie der Cleriden. XXVIII. Heft.* Brünn. 55 + [1] pp. (8vo) [DP: 1894 (title page), July 1894 (*Nat Nov*), 15 August 1894 (*Soc Ent*)] SME

This work was also issued in *Verhandlungen des Naturforschenden Vereins in Brünn* 32 [1893]: 37–89 [DP: 1894 (title page), January 1895 (*Nat Nov*)].

**1894**. *Bestimmungs-Tabelle der europäischen Coleopteren: Cantharidae. 1. Theil: Drilini. XXIX. Heft.* Paskau. 8 pp. (8vo) [DP: 1894 (title page), July 1894 (*Nat Nov*)] SME

**1895**. *Bestimmungs-Tabelle der Borkenkäfer (Scolytidae) aus Europa und den angrenzenden Ländern. XXXI. Heft.* Brünn. 64 pp. (8vo) [DP: 1895 (title page), 22 May 1895 (*Soc Ent Fr*), May 1895 (*Nat Nov*)] SME

This work was also issued in *Verhandlungen des Naturforschenden Vereins in Brünn* 33 [1894]: 36–97 [DP: 1895 (title page), January 1896 (*Nat Nov*)]. A second edition was published in 1913 as a supplement to volume 32 of *Wiener Entomologische Zeitung*.

**1895**. *Bestimmungs-Tabellen der europäischen Coleopteren Meloidae. 1. Theil: Meloini. XXXII. Heft.* Paskau. 13 pp. (8vo) [DP: 1895 (title page), 22 May 1895 (*Soc Ent Fr*), May 1895 (*Nat Nov*)] SME

An Italian translation by Vittorio Ronchetti was published in *Rivista Italiana di Scienze Naturali* 19 [1899]: 101–107, 133–135.

**1895**. *Bestimmungs-Tabellen der europäischen Coleopteren: Curculionidae. 3. Theil: (Stierlin’s 20. Gruppe Coryssomerini und 28. Baridiini.) XXXIII. Heft.* Paskau. 31 pp. (8vo) [DP: 1895 (title page), September 1895 (*Nat Nov*)] SME

**1896**. *Bestimmungs-Tabelle der europäischen Coleopteren: XXXIV. Heft. Enthaltend: Carabidae. I. Abtheilung: Carabini, gleichzeitig mit einer systematischen Darstellung sämmtlicher Subgenera der Gattung Carabus L.* Brünn. 165 pp. (8vo) [DP: 1896 (title page), 24 June 1896 (*Soc Ent Fr*), July 1896 (*Nat Nov*), 15 September 1896 (*Soc Ent*)] SME

This work was also issued in *Verhandlungen des Naturforschenden Vereins in Brünn* 34 [1895]: 36–198 [DP: 1896 (title page), February 1897 (*Nat Nov*)].

**1897**. *Anhang zur Bestimmungs-Tabelle der Carabidae. I. Abtheilung: Carabini. Enthaltend einen Index der Arten und Druckfehlerberichtigungen. XXXIV. Heft. II. Theil.* Edm. Reitter, Paskau. 13 + [1] pp. (8vo) [DP: 1897 (title page), June 1897 (*Nat Nov*)] SME

**1897**. Uebersicht der Arten der Coleopteren-Gattung *Asclera* Schm. Pp. 37–39 *in*: *Fest-Schrift zur Feier des fünfzigjährigen Bestehens des Vereins für schlesische Insektenkunde in Breslau. 1847–1897.* Maruschke & Berendt, Breslau. [1] + 141 + [1] pp. + 4 pls. (4to) [DP: 1897 (title page), 13 May 1896–13 May 1897 (*Soc Fauna Flora Fenn*), 15 April–15 May 1897 (*Leopoldina* 33: 75), May 1897 (*Nat Nov*), 14 June 1897 (*Bibl Zool*)] GB

**1897**. Ueber die Arten der Coleopteren-Gattung: *Anemia* Cast., aus der palaearctischen Fauna. Pp. 40–43 *in*: *Fest-Schrift zur Feier des fünfzigjährigen Bestehens des Vereins für schlesische Insektenkunde in Breslau. 1847–1897.* Maruschke & Berendt, Breslau. [1] + 141 + [1] pp. + 4 pls. (4to) [DP: 1897 (title page), 13 May 1896–13 May 1897 (*Soc Fauna Flora Fenn*), 15 April–15 May 1897 (*Leopoldina* 33: 75), May 1897 (*Nat Nov*), 14 June 1897 (*Bibl Zool*)] GB

**1898.** *Bestimmungs-Tabelle der europäischen Curculionidae. V. Theil: (Stierlin’s 29. und 30. Gruppe). Cossonini und Calandrini. XXXVII. Heft.* Paskau. 20 pp. (8vo) [DP: 1898 (title page), 24 May 1899 (*Soc Ent Fr*), May 1899 (*Nat Nov*), 15 July 1899 (*Bibl Zool*)] SME

This work was also issued in *Verhandlungen des Naturforschenden Vereins in Brünn* 37 [1898]: 3–20 [DP: 1899 (title page), December 1899 (*Nat Nov*), 19 January 1900 (*Bibl Zool*)].

**1898**. *Bestimmungs-Tabelle der Melolonthidae aus der europäischen Fauna und den angrenzenden Ländern. II. Theil: enthaltend die Gruppen der Dynastini, Euchirini, Pachypodini, Cetonini, Valgini und Trichiini. XXXVIII. Heft.* Brünn. 93 pp. (8vo) [DP: 1898 (title page), 24 May 1899 (*Soc Ent Fr*), May 1899 (*Nat Nov*), 15 July 1899 (*Bibl Zool*)] SME

This work was also issued in *Verhandlungen des Naturforschenden Vereins in Brünn* 37 [1898]: 21–111 [DP: 1899 (title page), December 1899 (*Nat Nov*), 19 January 1900 (*Bibl Zool*)]. A French translation was published, with separate pagination, in 1908–1909 in volume 16 of *Miscellanea Entomologica*.

**1900**. *Bestimmungs-Tabelle der europäischen Coleopteren. XLI. Heft. Enthaltend Carabidae. Abtheilung: Harpalini und Licinini.* Brünn. Pp. 33–155. (8vo) [DP: 1900 (title page), October 1900 (*Nat Nov*), 17 November 1900 (*Zool Anz*), 28 November 1900 (*Soc Ent Fr*)] SME

This work was also issued in *Verhandlungen des Naturforschenden Vereins in Brünn* 38 [1899]: 33–155 [DP: 1900 (title page), April 1901 (*Nat Nov*)]. A French translation was published, with separate pagination, in 1905–1906 in volumes 5 and 6 of *Miscellanea Entomologica*.

**1900**. *Bestimmungs-Tabelle der Tenebrioniden-Abtheilungen: Tentyrini und Adelostomini, aus Europa und den angrenzenden Gebieten. XLII. Heft. (42.).* Paskau. Pp. 82–197. (8vo) [DP: 1900 (title page), October 1901 (*Nat Nov*), 22 November 1901 (*Soc Ent Fr*)] SME

This work was also issued in *Verhandlungen des Naturforschenden Vereins in Brünn* 39 [1900]: 82–197 [DP: 1901 (title page), April 1902 (*Nat Nov*)].

#### Rendu, Louis-Victor (Maisons-Alfort, Val-de-Marne, France: 3 May 1809 – 11 June 1877: Paris, France). French lawyer and agronomist; chief inspector of agriculture. References. Tochon (1877a; 1877b); Albrier (1878).

**1838**. *Zoologie descriptive, ou histoire naturelle des animaux appliquée a l’agriculture. Tome deuxième. Animaux vertébrés.–Articulés.–Rayonnés.* J. Angé, Paris. 475 pp. (12mo) [DP: 1838 (title page), 12 May 1838 (*Bibl Fr*), 20 August 1838 (*Acad Sci Fr*)] GB

The Coleoptera are on pages 102–172.

#### Retzius, Anders Jahan [Johann] (Kristianstad, Sweden: 3 October 1742 – 6 October 1821: Stockholm, Sweden). Swedish chemist, botanist and entomologist; professor of natural history, economics and chemistry at the University of Lund. References. Anonymous (1822); Hoefer (1863: 50).

**1783**. *Caroli Lib. Bar. De Geer regiae avlae maresch. r. ord. Wasiaci commend. crvcig. r. ord. de Stella Bor. eqvit. avrat. r. Acad. Scient. Svec. Membr. et Parisinae correspond. Genera et species insectorvm e generosissimi avctoris scriptis extraxit, digessit, latine qvoad partem reddidit, et terminologiam insectorvm Linneanam addidit Anders Iahan Retzivs.* Siegfried Lebrecht Crvsivm, Lipsiae [= Leipzig]. [4] + vi + 7–220 + 32 pp. (8vo) [DP: 1783 (title page), 8 November 1783 (*Gött Anz*)] CNC, GB, BHL

The Coleoptera are treated on pages 16–22 (genera) and 101–173 (species). Some scientific names included in this work are first latinizations of DeGeer’s vernacular names published in his “*Mémoires pour servir à l’histoire des insectes*” 1774–1778 [*q.v.*].

This work has been placed on the *Official List of Works Approved as Available for Zoological Nomenclature* in Opinion 2333 (ICZN 2014).

#### Rey, Claudius (Lyon, Rhône, France: 8 September 1817 – 31 January 1895: Lyon, France). French entomologist mainly interested in Coleoptera and Hemiptera; financially independent until 1847; subsequently worked in a vineyard in southern France before returning to Lyon; his collection was bequeathed to the Muséum d’Histoire Naturelle in Lyon. References. Belon (1895, P); Guillebeau (1895, P); Smetana and Herman (2001: 129, P); Gouillard (2004: 48–49).

**1880–1884**. *Histoire naturelle des Coléoptères de France.* J.-B. Baillière et Fils, Paris. (8vo) USNM, CMLE, NCSU

*Brévipennes (Omaliens.–Pholidiens)* ^91^. 430 pp. + 6 pls. [DP: 1880 (title page), January 1882 (*Nat Nov*)]. This work was also published in the *Annales de la Société Linnéenne de Lyon* (Série Nouvelle) 27 [1880]: 1–430 [DP: 1880 (title page), 24 August 1881 (*Soc Ent Fr*), October 1881 (*Nat Nov*)].

*Brévipennes. Habrocériens, Tachyporiens, Trichophyens.* 295 pp. + 4 pls. [DP: 1883 (title page), 26 April 1884 (*Bibl Fr*), May 1884 (*Nat Nov*), January–June 1884 (*Bibliot Hist-Nat*), June 1884 (*Bull Mens*)]. This work was also published in the *Annales de la Société Linnéenne de Lyon* (Série Nouvelle) 28 [1881]: 135–308 [DP: 1882 (title page), 1 July 1882 (*Bibl Fr*), July 1882 (*Nat Nov*), 23 August 1882 (*Soc Ent Fr*)] and 29 [1882]: 13–123 [DP: 1883 (title page), 23 June 1883 (*Bibl Fr*), 11 July 1883 (*Soc Ent Fr*), July 1883 (*Nat Nov*)].

*Brévipennes. Micropéplides – Sténides.* 263 pp. + 3 pls. [DP: 1884 (title page), July 1884 (*Bull Mens*), 20 September 1884 (*Bibl Fr*), October 1884 (*Nat Nov*), November 1884 (*Polybiblion*), July–December 1884 (*Bibliot Hist-Nat*)]. This work was also published in the *Annales de la Société Linnéenne de Lyon* (Série Nouvelle) 30 [1883]: 153–415 + [3] + 3 pls [DP: 1884 (title page), 2 August 1884 (*Bibl Fr*), 25 August 1884 (Puthz 1973: 24), August 1884 (*Nat Nov*)].

[**1886**]. *Histoire naturelle des coléoptères de France. Palpicornes. Deuxième édition.* Ed. André, Beaune (Côte-d’Or). 374 + [1 (Errata)] pp. + 2 pls. (8vo) [DP: 1885 (title page), 20 November 1886 (*Bibl Fr*), 22 December 1886 (*Soc Ent Fr*), October–December 1886 (*Bibliot Hist-Nat*), December 1886 (*Nat Nov*)] NCSU, GAL

The content of this work was also published in the *Annales de la Société Linnéenne de Lyon* (Série Nouvelle) 31 [1884]: 213–396 [DP: 1885 (title page), 31 October 1885 (*Bibl Fr*), December 1885 (*Nat Nov*)] and 32 [1885]: 1–190 [DP: 1886 (title page), 27 November 1886 (*Bibl Fr*), December 1886 (*Nat Nov*)].

#### Richter, C.F.W. (? – 1849: Breslau [currently Wrocław], Poland). Teacher and later court district secretary in Brzeg, Poland.

**1820**. *Supplementa faunae insectorum Europae. Fasciculus I. Tab. I.–XII.* R.F. Schoene, Vratislaviae [= Wrocław]. [3] + 12 pp. + 12 pls. (12mo) [DP: 1820 (title page; *prooemium* dated July 1820)] UFM

Only this fascicle was published. The new Coleoptera described are: *Curculio equestris* (p. 2), *Curculio perlatus* (p. 3), *Curculio alpinus* (p. 4), *Chrysomela caerulea* (p. 5), *Chrysomela lichenis* (p. 6), *Chrysomela decora* (p. 7), *Haltica horticola* (p. 8), *Lyctus aeneus* (p. 9), *Prionus sudeticus* (p. 10), *Saperda dahlii* (p. 11), *Cryptocephalus quadriguttatus* (p. 12).

#### Risso, Joseph Antoine (Nice, Alpes-Maritimes, France: 8 March 1777 – 25 August 1845: Nice, France). French apothecary and naturalist; professor of physical and natural sciences at the Lyceum of Nice; professor of medical chemistry at the Nice Medical and Pharmacy Preparatory School. References. Monod and Hureau (1977, P); Julien (1980); Damkaer (2002: 163–167); Fredj and Meinardi (2007, P).

[**1827**]. *Histoire naturelle des principales productions de l’Europe méridionale et particulièrement de celles des environs de Nice et des Alpes Maritimes. Tome cinquième.* F.-G. Levrault, Paris. viii + 403 pp. + 10 pls. (8vo) [DP: 1826 (title page), 22 September 1827 (*Bibl Fr*), 20 October 1827 (*Rev Bibl-Brus*), October 1827 (*Not Geb Natur*)] GB, GAL

The entire series consists of five volumes, all dated 1826 on their title pages. Considering that the *Bibliographie de la France* recorded volumes 3 and 5 in September 1827, these volumes were likely issued that year. Volumes 1 and 4 were recorded in the same journal on 8 November 1826. The work came with black and white or colored plates.

The fifth volume of the series includes a list of the insects of the Maritime Alps (pp. 187–257). The Coleoptera are on pages 188–209 where the following new species are described: *Gyrinus splendens* “L[each] et R[isso]” (p. 191), *Meloe marginatus* “(N.)” (p. 201), *Timarcha affinis* “Leach, Risso” (p. 206) and *Galeruca desmaresti* “L[each] et R[isso]” (p. 207). Evenhuis (1997b: 649) mentioned that the insect section was done by W.E. Leach. I found no indication to this effect in the book. There are about 30 new species of insects described in the volume, all but a few are credited to Leach and Risso (usually indicated by “L. R.”); the others are simply noted “(N[obis]).” In my opinion, all new species but those denoted by (N.) should be credited to Leach and Risso and the others to Risso alone. Sherborn in his *Index Animalium* credited all new species in this book to Risso alone.

#### Ritsema, Coenraad [Conrad] (Haarlem, North Holland, Netherlands: 13 April 1846 – 9 January 1929: Wageningen, Gelderland, Netherlands). Dutch entomologist; curator at the Rijksmuseum van Natuurlike in Leiden (1873–1916). References. Anonymous (1912, P); Holthuis (1995: 60–61, P).

[**1882–1886**]. *Coleoptera door verschillende specialiteiten bewerkt en tot een geheel bijeengebracht.* E.J. Brill, Leiden. 210 pp. + 3 pls. (*n.v.*).

This work was issued in three parts: **1**: (pp. 1–72 + pl. i) September 1882 (*Nat Nov*); **2**: (pp. 73–180) January 1885 (*Nat Nov*); **3**: (pp. 181–210) 1886 (title page, *cf.* Woodward 1913: 1707). It was reissued by the same publisher in 1887 as part of “*Midden-Sumatra. Reizen en onderzoekingen der Sumatra-expeditie, uitgerust door het aardrijkskundig genootschap, 1877–1879, beschreven door de leden der expeditie, onder toezicht van Prof. P.J. Veth. Vierde deel. Natuurlijke historie. Eerste gedeelte. Fauna. Eerste helft*” [GB, SBB].

The Coleoptera families were treated by several authors: *Fam. Cicindelidae* (pp. 1–3) and *Fam. Carabidae* (pp. 3–8) by Jules Putzeys; *Fam. Dytiscidae* (pp. 8–11) and *Fam. Gyrinidae* (pp. 11–13) by Maurice Régimbart; *Fam. Staphylinidae* (pp. 15–17) by Albert Fauvel; *Fam. Scaphididae* (p. 17), *Fam. Histeridae* (pp. 17–18), *Fam. Nitidulidae* (pp. 18–19), *Fam. Trogositidae* (pp. 19–20), *Fam. Colydidae* (p. 20), *Fam. Dermestidae* (p. 20), *Fam. Byrridae* (pp. 20–21), *Fam. Parnidae* (p. 21–22) and *Fam. Apatidae* (pp. 79–80) by Edmund Reitter; *Fam. Lucanidae (Lucanini)* (pp. 22–23) by Frederick John Sidney Parry; [*Fam Lucanidae*] *(Passalini)* (pp. 23–24), [*Fam. Scarabaeidae*] *(Cetonini)* (pp. 44–50), *Fam. Buprestidae* (pp. 50–53), *Fam. Rhipidoceridae* (p. 59), *Fam. Dascillidae* (pp. 59–60), *Fam. Lymexylonidae* (p. 79), *Fam. Monommidae* (p. 102), *Fam. Pedilidae* (p. 107), *Fam. Mordellidae* (p. 109), *Fam. Rhipidophoridae* (p. 109), *Fam. Cantharidae* (pp. 109–110) and *Fam. Oedemeridae* (p. 110) by Conrad Ritsema; *Fam. Scarabaeidae (Coprini)* (pp. 24–29) by Edgar von Harold; [*Fam. Scarabaeidae*] *(Melolonthini)* (pp. 29–37) and [*Fam. Scarabaeidae*] *(Rutelini)* (pp. 37–43) by David Sharp; [*Fam. Scarabaeidae*] *(Dynastini)* (pp. 43–44) by David Sharp and Conrad Ritsema; *Fam. Eucnemidae* (pp. 53–54) by Henri de Bonvouloir; *Fam. Elateridae* (pp. 54–59) by Ernest Candèze; *Fam. Malacodermidae* (pp. 60–77), *Fam. Cleridae* (pp. 77–79), [*Fam. Chrysomelidae*] *(Cassidinae)* (pp. 181–183), *Fam. Erotylidae* (pp. 183–187), *Fam. Endomychidae* (pp. 187–189) and *Fam. Coccinellidae* (pp. 189–194) by Henry Stephan Gorham; *Fam. Tenebrionidae* (pp. 80–100), *Fam. Cistelidae* (pp. 100–101), *Fam. Nilionidae* (p. 102) and *Fam. Lagriidae* (pp. 102–106) by Léon Fairmaire; *Fam. Anthicidae* (pp. 107–109) by Sylvain de Marseul; *Fam. Curculionidae* (pp. 110–123), *Fam. Brenthidae* (pp. 124–125) and *Fam. Anthribidae* (pp. 126–129) by Willem Roelofs; *Fam. Scolytidae* (pp. 123–) by Wilhelm Eichhoff; *Fam. Cerambycidae* (pp. 129–140) by Francis Pascoe and Conrad Ritsema; *Fam. Chrysomelidae* [except Hispinae et Cassidinae] (pp. 141–180) by Martin Jacoby; and [*Fam. Chrysomelidae*] *(Hispinae)* (pp. 180–181) by Alfred Preudhomme de Borre.

All new species collecting during the trip were published prior to this publication in the journal *Notes from the Leyden Museum*.

#### Römer [Roemer], Johann Jacob (Zürich, Switzerland: 8 January 1763 – 15 January 1819: Zürich, Switzerland). Swiss physician, botanist and entomologist; practiced in Zürich and taught at the Institute of Medical Surgery; later, director of the botanical garden at the University of Zürich. References. Schinz (1819); Wunschmann (1889); Anonymous (2005c).

**1789**. *Genera insectorum Linnaei et Fabricii iconibus illustrata.* Henric. Steiner et Socios, Vitoduri Helvetorum [= Winterthur]. viii + 86 + [3 (Index tabularum)] pp. + 37 pls. (4to) [DP: 1789 (title page), 20 May 1791 (*Allg Lit Ztg*)] BHL, GB

The Coleoptera are on pages 1–12 and plates 1–7. The first 32 plates were first issued in Sulzer’s *Abgekurzte Geschichte der Insecten* [*q.v.*] published in 1776.

#### Rosenhauer, Wilhelm Gottlob (Wunsiedel, Bavaria, Germany: 11 September 1813 – 13 June 1881: Erlangen, Bavaria, Germany). German physician and zoologist; conservator of the zoology and mineralogy collections at the University of Erlangen; later, professor at the same university; his Andalusian Coleoptera went to the Muséum National d’Histoire Naturelle in Paris via René Oberthür [*q.v.*]. References. Katter (1881); Cambefort (2006: 282).

**1842**. *Die Lauf- und Schwimmkäfer Erlangens, mit besonderer Berücksichtigung ihres Vorkommens und ihres Verhältnisse zu denen einiger anderer Staaten Europa’s. Inaugural – Abhandlung, vorgelegt der medicinischen Facultät zu Erlangen.* J.J. Barfus, Erlangen. viii + 38 pp. (4to) [DP: 1842 (title page; *Vorwort* dated 19 July 1842), 7 January 1843 (*Lit Ztg*), 10 January 1843 (*Ent Ver Stettin*), 12 January 1843 (*Allg Bibl Deutsch*), January–June 1843 (*Verz Bücher Landkarten*)] GB

This work is an annotated catalogue of the Carabidae and Dytiscidae of Erlangen in Bavaria. Two new species, *Harpalus truncatus* (p. 12) and *Amara planiuscula* (p. 21), are described.

**1846**. *Broscosoma und Laricobius, zwei neue Käfergattungen, entdeckt, beschrieben und in Stahl abgebildet.* Theodor Blaesing, Erlangen. 8 pp. + 1 pl. (8vo) [DP: 1846 (title page), 5 May 1847 (*Lit Ztg*), 31 May 1847 (*Intell Serapeum*), 11 June 1847 (*Leip Reper*), January–June 1847 (*Verz Bücher Landkarten*), 4 March 1848 (*Ent Ver Stettin*)] MCZ, GB, MDZ

This booklet was reissued in Rosenhauer’s “*Beiträge zur Insekten-Fauna Europas*” on pages 1–8 (see next entry).

**1847**. *Beiträge zur Insekten-Fauna Europas. Erstes Bändchen; enthält die Beschreibung von sechzig neuen Käfern aus Bayern, Tyrol, Ungarn, etc., so wie die Käfer Tyrols, nach dem Ergebnisse von vier Reisen. Mit einer Tafel Abbildungen.* Theodor Blaesing, Erlangen. x + 159 + [1] pp. + 1 pl. (8vo) [DP: 1847 (title page; *Vorwort* dated 2 November 1847), 9 February 1848 (*Lit Ztg*), 10 February 1848 (*Allg Bibl Deutsch*), 15 February 1848 (*Intell Serapeum*), 24 February 1848 (*Bibl Univers*), February 1848 (*Intell Allg Lit Ztg*), 4 March 1848 (*Ent Ver Stettin*), 23 June 1848 (*Leip Reper*), 12 July 1848 (*Allg Ztg*)] CNC, GB

This work contains two sections: “*Beschreibung von sechzig neuen Käferarten aus Bayern, Tyrol, Ungarn u.s.w.*” (pp. 1–66) where 60 new European species of Coleoptera are described, though a few were already available, and “*Die Käfer Tyrol’s, nach dem Ergebnisse von vier Reisen mit besonderer Berücksichtigung ihres Vorkommens und ihrer Verbreitung zusammengestellt*” (pp. 67–159) which consists of an annotated catalogue of the Coleoptera of Tirol; two new species, *Allecula aterrima* (p. 122) and *Cistela laevis* (p. 123), are described in the second section.

**1856**. *Die Thiere Andalusiens nach dem Resultate einer Reise zusammengestellt, nebst den Beschreibungen von 249 neuen oder bis jetzt noch unbeschriebenen Gattungen und Arten. Mit drei Kupfertafeln.* Theodor Blaesing, Erlangen. viii + 429 pp. + 3 pls. (8vo) [DP: 1856 (title page; *Vorwort* dated October 1856), November 1856 (Evenhuis 1997b: 668), 18 December 1856 (*Allg Bibl Deutsch*), July–December 1856 (*Bibliot Hist-Nat*), October–December 1856 (*Cat Muquardt*), January 1857 (*Allg Bibl*)] SME, GB

This work consists of an annotated catalogue of the animals collected during a journey to Andalusia. According to the title, 249 new genus- and species-group taxa are described. The Coleoptera are on pages 17–360 and plate 3.

#### Rossi, Pietro [Peter] (Florence, Tuscany, Italy: 23 January 1738 – 21 December 1804: Pisa, Tuscany, Italy). Italian physician and entomologist; professor of logic at the University of Pisa (1763–1801), afterward professor of entomology (1801–1804); first university professor of entomology in the world; the whereabouts of his collections are unknown. References. Baccetti (1962); Conci (1975: 1005–1006).

**1790**. *Fauna Etrusca sistens insecta quae in provinciis Florentina et Pisana praesertim collegit.* Thomae Masi & Sociorum, Liburni [= Livorno]. (4to) CNC, GB

*Tomus primus.* xxii + [1] + 272 pp. + 10 pls (shared with volume 2). [DP: 1790 (title page; *lectori* dated September 1789), 5 February 1791 (*Gött Anz* 1791: 224), February 1791 (*Observ Phys*), 17 March 1791 (*Gött Anz*)].

*Tomus secundus.* 348 pp. [DP: 1790 (title page), 5 February 1791 (*Gött Anz* 1791: 224), February 1791 (*Observ Phys*), 17 March 1791 (*Gött Anz*)].

The second volume does not pertain to Coleoptera but the “*Notanda, et addenda*” section (pp. 341–346) includes information about beetles.

A German edition of this work and the *Mantissa insectorum* (next entry) with additions was published by Hellwig in 1795 [*q.v.*] and Illiger in 1807.

**1792–1794**. *Mantissa insectorum exibens species nuper in Etruria collectas a Petro Rossio adiectis faunae Etruscae illustrationibus, ac emendationibus.* Polloni [vol. 1] / Prosperi [vol. 2], Pisis [= Pisa]. (4to) AMNH, GB

[*Tom. I.*] 148 pp. [DP: 1792 (title page; *lectori* dated March 1792)].

*Tom. II.* 154 pp. + 8 pls. [DP: 1794 (title page)].

This work contains descriptions of insects recently collected by the author in Etruria, an ancient country in central Italy. The Coleoptera are on pages 5–101 of the first volume and in the “*Appendix*” (pp. 79–126) of the second volume where 70 additional species of beetles are described (pp. 79–104) including several new to science.

#### Rossmässler, Emil Adolf (Leipzig, Saxony, Germany: 3 March 1806 – 8 April 1867: Leipzig, Germany). German naturalist, politician and science popularizer; professor of zoology and botany at the forestry college in Tharandt (1830–1849); deputy of Pirna in the Frankfurt National Assembly (1848–1849); dismissed on assumption of high treason, he was acquitted and then moved to Leipzig. References. Schmidt (1867); Ratzeburg (1874: 441–446); Wunschmann (1889).

**1832**. *Systematische Uebersicht des Thierreichs, ein Leitfaden für die Vorlesungen über Zoologie bei der Königl. Akademie für Forst- und Landwirthe zu Tharand. Nebst einem Atlas mit erklärenden Textblättern.* Arnold, Dresden und Leipzig. iv + 164 + [1] pp. (8vo) [DP: 1833 (title page; *Vorwort* dated September 1832), 6 November 1832 (*Bibl Deutsch*), November 1832 (*Monatl Bericht Bücher*), 15 December 1832 (*Allg Ztg*), July–December 1832 (*Verz Bücher Landkarten*)] GB

The Coleoptera are on pages 66–72. Despite the date on the title page, this work was published in 1832. A second edition with same number of pages was issued in 1835 [GB] under a slightly different title. An Atlas of 12 plates was published in 1835 under the title “*Gallerie der Thierwelt, ein Atlas zur Uebersicht des Theirreichs*” (*n.v.*).

#### Roth von Schreckenstein, Friedrich Josef Anton (Eichstätt, Bavaria, Germany: 12 October 1753 – 1808: Donaueschingen, Baden-Württemberg, Germany). German noble (baron), historian and naturalist in Immendingen, Germany; his ambition was to improve agriculture for the benefit of the common good. References. Gradmann (1802: 523–525); Anonymous (1812); Anonymous (2013c).

**1801**. *Verzeichniss der Kaefer, welche um den Ursprung der Donau und des Nekars, dann um den untern Theil des Bodensees vorkommen.* J.G. Cotta, Tübingen. 67 pp. (8vo) [DP: 1801 (title page)] GB, MDZ

This is an annotated catalogue of the Coleoptera found in the area near the source of the Danube and the Neckar to the lower part of Lake Constance. It was published anonymously.

#### Rothschild, Jules (Hofgeismar, Hesse, Germany: 3 June 1838 – 8 August 1900: Paris, France). French (German-born) publisher and naturalist in Paris; became French citizen in 1867; Knight of the Legion of Honor. References. Anonymous (1900a); Anonymous (2015d).

**1875**. *Musée entomologique illustré. Histoire naturelle iconographique des insectes publiée par une réunion d’entomologistes français et étrangers sous la direction de J. Rothschild. Tome premier. Les Coléoptères.* J. Rothschild, Paris. 384 pp. + 48 pls. (4to) [DP: 1876 (title page), 18 December 1875 (*Rev Sci*), 8 January 1876 (*Feuil J Lib*)] BHL

This book includes a second title page which reads “*Les Coléoptères. Organisation–moeurs–chasse–collections–classification. Iconographie et histoire naturelle des coléoptères d’Europe avec 48 planches en couleur et 335 vignettes*.”

The entire series *Musée entomologique illustré* consists of three volumes, 1875–1878.

#### Runde, Wilhelm Hermann (Halle an der Saale, Saxony-Anhalt, Germany: 28 November 1810 – 4 December 1867: Dölau [currently part of Halle an der Saale], Germany). German practicing physician first in Altlandsberg near Berlin, later in Klepzig and Dölau near Merseburg and finally in Brachwitz near Halle. Reference. Lönnecker (2008: 238).

**1835**. *Brachelytrorum species agri Halensis. Dissertatio inauguralis medica quam auctoritate atque consensu gratiosi medicorum ordinis Halensis ut summos in medicina et chirurgia honores rite adipiscatur Die XXVIII. mensis Octobris a. MDCCCXXXV. Una cum thesibus publice defendet auctor Guilielmus Hermannus Runde Halensis. Opponeentibus: F. Kerstein. F. Succow.* Ploetz, Halae [= Halle]. [6] + 32 + [1 (Curriculum vitae)] pp. (8vo) [DP: 28 October 1835 (title page), 25 November 1835 (*Lit Ztg*), July–December 1835 (*Verz Bücher Landkarten*)] MCZ, GB

This thesis pertains to the Staphylinidae of Halle. Several new species are described.

#### Rye, Edward Caldwell (London, United Kingdom: 10 April 1832 – 7 February 1885: Stockwell [currently in Greater London], United Kingdom). British legal clerk, librarian and coleopterist; solicitor’s clerk and later barristers’ clerk; librarian at the Royal Geographical Society (1875–1885); editor for the *Zoological Record* for several years; his collection was acquired by Philip Brookes Mason and is now housed at the Bolton Museum. References. Anonymous (1885b); Carrington (1885).

**1866**. *British beetles: an introduction to the study of our indigenous Coleoptera.* Lovell Reeve & Co., London. xv + 280 pp. + 16 pls. (8vo) [DP: 1866 (title page; preface dated February 1866), 24 February 1866 (*Athenaeum*), 1 March 1866 (*Publ Circ*), 24 March 1866 (*Lit Centr Deutsch*), 31 March 1866 (*Bookseller*), March–April 1866 (*Allg Bibl*), January–June 1866 (*Bibliot Hist-Nat*)] MANL, GB, BHL

A “second edition, revised and in part re-written by the Rev. Canon Fowler” was issued by the same publisher in 1890 [MANL] and recorded on 3 May 1890 (*Bookseller*).

#### Sahlberg, Carl Reinhold (Eura, Turku ja Pori, Finland: 22 January 1779 – 18 October 1860: Uusikartano near Pöytyä, Turku ja Pori, Finland). Finnish naturalist; professor of natural history and economy at the University of Turku; later, professor of zoology and botany at the University of Helsinki; founded the *Societas pro Fauna et Flora Fennica*; his collection is at the Finnish Museum of Natural History, University of Helsinki. References. Sanmark (1861); Törnroth (1861); Renvall (1869: 174–175); Smetana and Herman (2001: 132–133, P).

**1817–1839**. *Dissertatio entomologica. Insecta Fennica enumerans.* [51 theses]. Frenckel, Aboae. (8vo)

*Cujus particulam primam cons. ampl. facult. philos. Aboënsis publicae censurae subjicit mag. Carolus Reginaldus Sahlberg, respondente Carolo Johanne Boij stip. publ. Satac. In auditorio juridico die 15 Dec. 1817. H.a.m.s.* viii + 8 pp. [DP: 15 December 1817 (cover)] DORIA

*Cujus partem secundam consensu ampl. facult. philos. Aboënsis praeside Carolo Reginaldo Sahlberg. Publico examini subjicit pro gradu philosophico Fredericus Gabriel Sanmark, stip. publ. Satacundensis. In auditorio medico die 15 Maji 1819. H.a.m.s.* Pp. 9–24. [DP: 15 May 1819 (cover)] DORIA

*Cujus partem tertiam consensu ampl. facult. philos. Aboënsis praeside Carolo Reginaldo Sahlberg. Publico examini subjicit pro gradu philosophico Andreas Wilhelmus Mennander, stip. publ. Aboënsis. In auditorio medico die 19 Maji 1819. H.a.m.s.* Pp. 25–40. [DP: 19 May 1819 (cover)] DORIA

*Cujus particulam quartam consensu ampl. facult. philos. Aboënsis praeside Carolo Reginaldo Sahlberg. Publicae censurae submittit pro gradu philosophico Johannes Hirn, Wiburgensis. In auditorio theol. die 19 Junii 1819. H.a.m.c.* Pp. 41–56. [DP: 19 June 1819 (cover)] DORIA

*Cujus particulam quintam cons. ampl. facul. philos. Aboënsis praeside Carolo Reginaldo Sahlberg. Publicae censurae submittit pro gradu philosophico Andreas Josephus Europaeus, stip. publ. Wiburgensis. In auditorio philosophico die 13 Dec. 1820. H.a.m.s.* Pp. 57–72. [DP: 13 December 1820 (cover)] DORIA

*Cujus particulam sextam cons. ampl. facul. philos. Aboënsis praeside Carolo Reginaldo Sahlberg. Publico examini subjicit pro laurea Gustavus Magnus Nordström v. d. m. Wiburgensis. In auditorio philosophico die 20 Dec. 1820. H.a.m.s.* Pp. 73–88. [DP: 20 December 1820 (cover)] DORIA

*Cujus particulam septimam cons. ampl. facult. philos. Aboënsis praeside Carolo Reginaldo Sahlberg. Publicae disquisitioni submittit pro gradu philosophico Adolphus Wilhelmus Wegelius Aboënsis. In auditorio philosophico die 18 Dec. 1822. H.a.m.s.* Pp. 89–104. [DP: 18 December 1822 (cover)] DORIA

*Cujus particulam octavam cons. ampl. facult. philos. Aboënsis praeside Carolo Reginaldo Sahlberg. Publico examini subjicit pro laurea Wilhelmus Fridericus Brummer Aboënsis. In auditorio philosophico die 18 Dec. 1822. H.p.m.s.* Pp. 105–120. [DP: 18 December 1822 (cover)] DORIA

*Cujus particulam nonam, cons. ampl. facult. philos. Aboënsis praeside Carolo Reginaldo Sahlberg. Publicae disquisitioni submittit pro gradu philosophico Johannes Nyman, stip. bilmark. Ostrobotthniensis. In auditorio juridico die 10 Maji 1823. H.a.m.s.* Pp. 121–136. [DP: 10 May 1823 (cover)] MDZ, DORIA

*Cujus particulam decimam, cons. ampl. facult. philos. Aboënsis praeside Carolo Reginaldo Sahlberg. Publicae submittit censurae pro laurea Ewaldus Erlandus Rosenback, stip. publ. Satacundensis. In auditorio juridico die 10 Maji 1823. H.p.m.s.* Pp. 137–152. [DP: 10 May 1823 (cover)] MDZ, DORIA

*Cujus particulam undecimam, cons. ampl. facult. philos. Aboënsis, praeside Carolo Reginaldo Sahlberg. Publicae disquisitioni submittit pro gradu philosophico Jacobus Johannes Lindeqvist: stip. publ. Wiburgensis. In auditorio philosophico die XVI 1824. H.a.m.s.* Pp. 153–168. [DP: 10 May 1824 (Hagen 1863: 102; Horn and Schenkling 1928c: 1034)] MDZ, DORIA

*Cujus particulam duodecimam, cons. ampl. facult. philos. Aboënsis, praeside Carolo Reginaldo Sahlberg. Publicae disquisitioni submittit pro laurea Gabriel Geitlin, linguae Russicae ad universitatem magister, Borealis. In auditorio juridico die XX Dec. 1826. H.a.m.s.* Pp. 169–184. [DP: 20 December 1826 (cover)] DORIA

*Cujus particulam tredecimam, cons. ampl. facult. philos. Aboënsis, praeside Carolo Reginaldo Sahlberg. Publicae censurae submittit pro gradu philosophico Franciscus Johannes Rabbe, stipend. brem. Satacundensis. In auditorio philos. die XIX Maji 1827. H.a.m.s.* Pp. 185–200. [DP: 19 May 1827 (cover)] DORIA

*Cujus particulam decimam quartam, cons. ampl. facult. philos. Aboënsis, praeside Carolo Reginaldo Sahlberg. Publicae censurae submittit pro laurea Johannes Ascholin, Satacundensis. In auditorio philos. die XIX Maji 1827. H.p.m.s.* Pp. 201–216. [DP: 19 May 1827 (cover)] DORIA

*Cujus particulam decimam quintam, cons. ampl. facult. philos. Aboënsis, praeside Carolo Reginaldo Sahlberg. Publicae censurae offert pro laurea Petrus Philippus Lindforss, stip. publ. Nyland. In auditorio medico die XXVI Maji 1827. H.a.m.c.* Pp. 217–232. [DP: 26 May 1827 (cover)] DORIA

*Cujus particulam decimam sextam, cons. ampl. facult. philos. Aboënsis, praeside Carolo Reginaldo Sahlberg. Publicae censurae offert pro summis in philosophia honoribus Gustavus Johannes Mechelin, stipend. publ. Wiburgensis. In auditorio medico die XXVI Maji 1827. H.p.m.s.* Pp. 233–248. [DP: 26 May 1827 (cover)] DORIA

*Cujus particulam decimam septimam, cons. ampl. facult. philos. Aboënsis, praeside Carolo Reginaldo Sahlberg. Publicae proponit censurae, pro summis in philosophia honoribus, Arndtius Gerhardus Lindforss; ad scholam Helsingforssensem vicarius collega, Nyland. In auditorio philos. die XXIII Junii 1827. H.a.m.s.* Pp. 249–264. [DP: 23 June 1827 (cover)] DORIA

*Cujus particulam decimam octavam, cons. ampl. facult. philos. Aboënsis, praeside Carolo Reginaldo Sahlberg. Pro laurea publicae subjicit censurae, Johannes Ludovicus Runeberg, stip. publ. Ostrobottniensis. In auditorio philos. die XXIII Junii 1827. H.p.m.s.* Pp. 265–270. [DP: 23 June 1827 (cover)] DORIA

*Cujus particulae decimae octavae continuationem, cons. ampl. facult. philos. Aboënsis, praeside Carolo Reginaldo Sahlberg. Pro laurea publicae proponit disquisitioni Alexander von Nordtman, stip. publ. nob. Wiburg. In auditorio philos. die VI Julii 1827. H.a.m.s.* Pp. 271–280. [DP: 6 July 1827 (cover)] DORIA

*Cujus particulam undevigesimam cons. ampl. facult. philos. ad Imper. Univers. Alexandr. in Fennia, praeside Carolo Reginaldo Sahlberg. Publicae submittit censurae Andreas Magnus Höglund, stipend. publ. Nyl. In auditorio jurid. die 24 Febr. 1830. H.a.m.s.* Pp. 281–296. [DP: 24 February 1830 (cover)] MDZ

*Cujus particulam vigesimam cons. ampl. facult. philos. ad Imper. Univers. Alexandr. in Fennia, praeside Carolo Reginaldo Sahlberg. Publico submittit examini Gustavus Asp, Satacundensis. In auditorio jurid. die 31 Martii 1830. H.a.m.s.* Pp. 297–312. [DP: 31 March 1830 (cover)] MDZ

*Cujus particulam vigesimam primam cons. ampl. facult. philos. ad Imper. Univers. Alexandr. in Fennia, praeside Carolo Reginaldo Sahlberg. Publico submittit examini Johannes Ernestus Adhem. Wirzén, stipend. publicus, Satacundensis. In auditorio jurid. die 31 Martii 1830. H.p.m.s.* Pp. 313–328. [DP: 31 March 1830 (cover)] MDZ

*Cujus particulam vigesimam secundam cons. ampl. facult. philos. ad Imper. Univers. Alexandr. in Fennia, praeside Carolo Reginaldo Sahlberg. Publico examini offert Carolus Ephraim Lilius, stipend. publicus, Satacundensis. In auditorio theol. die 27 Novembris 1830. H.p.m.s.* Pp. 329–344. [DP: 27 November 1830 (cover)] MDZ

*Cujus particulam vigesimam tertiam cons. ampl. facult. philos. ad Imper. Univers. Alexandr. in Fennia, praeside Carolo Reginaldo Sahlberg. Publico examini offert. Johannes Philippus Palmén, stipend. publicus, Satacundensis. In auditorio jurid. die 9 Februarii 1831. H.a.m.s.* Pp. 345–360. [DP: 9 February 1831 (cover)] MDZ

*Cujus particulam vigesimam quartam, cons. ampl. facult. philos. ad Imper. Univers. Alexandr. in Fennia, praeside Carolo Reginaldo Sahlberg. Publico examini offert Evertus Julius Bonsdorff, stipend. publicus, Wiburgensis. In auditorio jurid. die 28 Maji 1831. H.a.m.s.* Pp. 361–376. [DP: 28 May 1831 (cover)] MDZ

*Cujus particulam vigesimam quintam, cons. ampl. facult. philos. ad Imper. Univers. Alexandr. in Fennia, praeside Carolo Reginaldo Sahlberg. Publicae submittit censurae Gabriel Reginaldus Hartman, stipend. publ. Nylandus. In auditorio jurid. die 28 Maj 1831. H.p.m.s.* Pp. 377–392. [DP: 28 May 1831 (cover)] MDZ

*Cujus particulam vigesimam sextam, cons. ampl. facult. philos. ad Imper. Univers. Alexandr. in Fennia, praeside Carolo Reginaldo Sahlberg. Publico examini offert Gustavus Samuel Crusell, stipend. publicus, Borealis. In auditorio jurid. die 15 Junii 1831. H.a.m.s.* Pp. 393–408. [DP: 15 June 1831 (cover)] MDZ

*Cujus particulam vigesimam septimam, cons. ampl. facult. philos. ad Imper. Univers. Alexandr. in Fennia, praeside Carolo Reginaldo Sahlberg. Publicae submittit censurae Uno Cygnaeus, stip. Ekestubb. Wiburgensis. In auditorio jurid. die 16 Junii 1832. H.a.m.c.* Pp. 409–424. [DP: 16 June 1832 (cover)] MDZ

*Cujus particulam vigesimam octavam, cons. ampl. facult. philos. ad Imper. Univers. Alexandr. in Fennia, praeside Carolo Reginaldo Sahlberg. Publico examini offert Nicolaus Constantinus Lignell, stip. De la Myhl. Borealis. In auditorio jurid. die 16 Junii 1832. H.p.m.c.* Pp. 425–440. [DP: 16 June 1832 (cover)] MDZ

*Cujus particulam vigesimam nonam, cons. ampl. facult. philos. ad Imper. Univers. Alexandr. in Fennia, praeside Carolo Reginaldo Sahlberg. Publicae offert censurae Augustus Edvardus Granfelt. In audit. philos. die 9 Nov. 1833. H.a.m.s.* Pp. 441–456. [DP: 9 November 1833 (cover)] MDZ

*Cujus particulam trigesimam, cons. ampl. facult. philos. ad Imper. Univers. Alexandr. in Fennia, praeside Carolo Reginaldo Sahlberg. Publicae offert censurae Johannes Ernestus Sourander, stip. publ. Satacundensis. In audit. philos. die 9 Nov. 1833. H.p.m.s.* Pp. 457–472. [DP: 9 November 1833 (cover)] MDZ

*Cujus particulam trigesimam primam, cons. ampl. facult. philos. ad Imper. Univers. Alexandr. in Fennia, praeside Carolo Reginaldo Sahlberg. Publicae submittit censurae Carolus Fredricus Haartman, Aboënsis. In audit. philos. die 7 Dec. 1833. H.a.m.c.* Pp. 473–488. [DP: 7 December 1833 (cover)] MDZ

*Cujus particulam trigesimam secundam, cons. ampl. facult. philos. ad Imper. Univers. Alexandr. in Fennia, praeside Carolo Reginaldo Sahlberg. Publicae submittit censurae Gustavus Johannes Ahlquist ... In audit. philos. die 7 Maji 1834 ...* Pp. 489–504. [DP: 7 May 1834] (*n.v.*).

*Cujus particulam trigesimam tertiam, cons. ampl. facult. philos. ad Imper. Univers. Alexandr. in Fennia, praeside Carolo Reginaldo Sahlberg. Publicae examini offert Nicolaus Johannes Wilhem Idman ... In auditorio philos. die 7 Maji 1834 ...* Pp. 505–519 [DP: 7 May 1834] (*n.v.*).

*Cujus particulam primam partis secundae, cons. ampl. facult. philos. ad Imper. Univers. Alexandr. in Fennia, publicae subjicit disquisitioni Carolo Reginaldo Sahlberg, respondente Josepho Grönberg, stip. Segerer. Satacundensi. In audit. philos. die 10 Maji 1834. H.p.m.c.* Pp. 1–16. [DP: 10 May 1834 (cover)] MDZ

*Cujus particulam secundam partis secundae, cons. ampl. facult. philos. ad Imper. Univers. Alexandr. in Fennia, publicae subjicit disquisitioni Carolus Reginaldus Sahlberg, respondente Augusto Magno Lilio, stip. publ. Satacundensi. In audit. philos. die 10 Dec. 1834. H.a.m.s.* Pp. 17–32. [DP: 10 December 1834 (cover)] MDZ

*Cujus particulam tertiam partis secundae, cons. ampl. facult. philos. ad Imper. Univers. Alexandr. in Fennia, publicae subjicit disquisitioni Carolus Reginaldus Sahlberg, respondente Jacobo Fredrico Blank, Ostrobottniensi. In audit. philos. die 6 Maji 1835. H.a.m.s.* Pp. 33–48. [DP: 6 May 1835 (cover)] MDZ

*Cujus particulam quartam partis secundae, cons. ampl. facult. philos. ad Imper. Univers. Alexandr. in Fennia, publicae submittit censurae Carolus Reginaldus Sahlberg, respondente Carolo Helenio, stip. publ. Boreali. In auditorio philos. die 6 Maji 1835. H.p.m.c.* Pp. 49–64. [DP: 6 May 1835 (cover)] MDZ

*Cujus particulam quintam partis secundae, cons. ampl. facult. philos. ad Imper. Univers. Alexandr. in Fennia, publicae subjicit disquisitioni Carolus Reginaldus Sahlberg, respondente Jacobo Gustavo Appelberg, Ostrobottniensi. In audit. philos. die 16 Maji 1835. H.a.m.s.* Pp. 65–80. [DP: 16 May 1835 (cover)] MDZ

*Cujus particulam sextam partis secundae, cons. ampl. facult. philos. ad Imper. Univers. Alexandr. in Fennia, publicae submittit censurae Carolus Reginaldus Sahlberg, respondente Carolo Wilhelmo Törnegren, Satacundensi. In auditorio philos. die 16 Maji 1835. H.p.m.c.* Pp. 81–96. [DP: 16 May 1835 (cover)] MDZ

*Cujus particulam septimam partis secundae, cons. ampl. facult. philos. ad Imper. Univers. Alexandr. in Fennia, publicae submittit censurae Carolus Reginaldus Sahlberg, respondente Carolo Wilhelmo Spåre, Nobili, stip. Ekestubb. Satakundensi. In auditorio philos. die 14 Nov. 1835. H.a.m.c.* Pp. 97–112. [DP: 14 November 1835 (cover)] MDZ

*Cujus particulam octavam partis secundae, cons. ampl. facult. philos. ad Imper. Univers. Alexandr. in Fennia, publicae subjicit disquisitioni Carolus Reginaldus Sahlberg, respondente Carolo Reginaldo Bonsdorff, Viburgensi. In audit. Philos. die 14 Nov. 1835. H.p.m.s.* Pp. 113–128. [DP: 14 November 1835 (cover)] MDZ

*Cujus particulam nonam partis secundae, cons. ampl. facult. philos. ad Imper. Univers. Alexandr. in Fennia, publicae submittit censurae Carolus Reginaldus Sahlberg, respondente Johanne Justo Staudinger, Ostrobottniensi. In audit. philos. die 7 Maji 1836. H.a.m.s.* Pp. 129–144. [DP: 7 May 1836 (cover)] MDZ

*Cujus particulam decimam partis secundae, cons. ampl. facult. philos. ad Imper. Univers. Alexandr. in Fennia, publico offert examini Carolus Reginaldus Sahlberg, respondente Alfredo Wacklin, Ostrobottniensi. In audit. philos. die 7 Maji 1836. H.p.m.s.* Pp. 145–160. [DP: 7 May 1836 (cover)] MDZ

*Cujus particulam undecimam partis secundae, cons. ampl. facult. philos. ad Imper. Univers. Alexandr. in Fennia, publicae submittit censurae Carolus Reginaldus Sahlberg, respondente Axelio Fredrico Granfelt, stip. Ekestubb. Satacundensi. In auditorio philos. die 3 Junii 1837. H.a.m.c.* Pp. 161–176. [DP: 3 June 1837 (cover)] MDZ

*Cujus particulam duodecimam partis secundae, cons. ampl. facult. philos. ad Imper. Univers. Alexandr. in Fennia, publicae submittit censurae Carolus Reginaldus Sahlberg, respondente Carolo Gustavo Idman, Satacundensi. In auditorio philos. die 9 Dec. 1837. H.a.m.c.* Pp. 177–192. [DP: 9 December 1837 (cover)] MDZ

*Cujus particulam decimam tertiam partis secundae, cons. ampl. facult. philos. ad Imper. Univers. Alexandr. in Fennia, publicae submittit censurae Carolus Reginaldus Sahlberg, respondente Fredrico Ferdinando Idman Satacundensi. In auditorio philos. die 16 Maji 1838. H.a.m.c.* Pp. 193–208. [DP: 16 May 1838 (cover)] MDZ

*Cujus particulam decimam quartam partis secundae, cons. ampl. facult. philos. ad Imper. Univers. Alexandr. in Fennia, publico offert examini Carolus Reginaldus Sahlberg, respondente Zacharia Topelio, stip. Bilmark. Borea-Ostrobottniensi. In auditorio philos. die 16 Maji 1838. H.p.m.s.* Pp. 209–224. [DP: 16 May 1838 (cover)] MDZ

*Cujus particulam decimam quintam partis secundae, cons. ampl. facult. philos. ad Imper. Univers. Alexandr. in Fennia, publicae offert censurae Carolus Reginaldus Sahlberg, respondente Carolo Lundahl, Satacundensi. In auditorio philos. die 17 Novembr. 1838. H.a.m.c.* Pp. 225–240. [DP: 17 November 1838 (cover)] MDZ

*Cujus particulam decimam sextam partis secundae, cons. ampl. facult. philos. ad Imper. Univers. Alexandr. in Fennia, publicae submittit censurae Carolus Reginaldus Sahlberg, respondente Johanne Jacobo Rahm, Austro-Ostrobottniensi, stip. publ. In auditorio jurid. die 17 Novembr. 1838. H.p.m.s.* Pp. 241–256. [DP: 17 November 1838 (cover)] MDZ

*Cujus particulam decimam septimam partis secundae, cons. ampl. facult. philos. ad Imper. Univers. Alexandr. in Fennia, publicae submittit censurae Carolus Reginaldus Sahlberg, respondente Joachimo Wilhelmo Pipping, Boreali. In auditorio philos. die 10 Aprilis 1839. H.a.m.s.* Pp. 257–272. [DP: 10 April 1839 (cover)] MDZ

*Cujus particulam decimam octavam partis secundae, cons. ampl. facult. philos. ad Imper. Univers. Alexandr. in Fennia, publicae submittit censurae Carolus Reginaldus Sahlberg, respondente Carolo Bernhardo Sjöberg, Austro-Ostrobottniensi. In auditorio philos. die 13 Aprilis 1839. H.a.m.s.* Pp. 273–288. [DP: 13 April 1839 (cover)] MDZ

These theses were written by Carl Reinhold Sahlberg and defended by his students. The first 33 dissertations were reissued without the dissertation titles, 1834, in Helsinki under the title “*Insecta Fennica, dissertationibus academicis, a. 1817–1834 editis, enumerata a Carolo Reginaldo Sahlberg. Pars I:a.*” [CNC] with viii + 519 pages.

**1823**. *Periculum entomographicum, species insectorum nondum descriptas proponens, quod venia facult. philos. Aboënsis ampliss. praeside Carolo Reginaldo Sahlberg.* [5 theses]. Frenckel, Aboae. (8vo) DORIA

*Publicae censurae submittit pro laurea Adolphus Wilhelmus Dammert, stipend. publicus, Aboënsis. In auditorio philos. die XIII Junii 1823. H.a.m.s. P. I.* Pp. 1–16. [DP: 13 June 1823 (cover)].

*Publicae censurae submittit pro laurea Wilhelmus Forssman. Stipend. publicus, Nylandus. In auditorio philos. die XIII Junii 1823. H.p.m.s. P. II*. Pp. 17–32. [DP: 13 June 1823 (cover)].

*Publicae censurae submittit pro laurea Carolus Grönlund Satacundensis. In auditorio philos. die XIV Junii 1823. H.a.m.s. P. III.* Pp. 33–48. [DP: 14 June 1823 (cover)].

*Publicae censurae submittit pro laurea Johannes Fridericus Homén, stipend. publicus Satacundensis. In auditorio philos. die XIV Junii 1823. H.p.m.s. P. IV.* Pp. 49–64. [DP: 14 June 1823 (cover)].

*Publicae censurae submittit pro laurea Carolus Tengström, Ostrobottn. In auditorio philos. die XVI Junii 1823. H.a.m.c. P. V.* Pp. 65–82 + 4 pls. [DP: 16 June 1823 (cover)].

These theses were also issued, without the dissertation titles, in 1823 under the title “*Periculi entomographici, species insectorum nondum descriptas proposituri fasciculus. Cum tabuli IV aeneis*” [CNC, GB, BHL] with 82 + [1 (Corrigenda)] pages and four plates. The text was reissued in 1829 in volume 2 (pages 12–31) of *Entomologisches Archiv* [CNC]; the illustrations on the four color plates were combined in one black and white plate inserted on page 81.

#### Sahlberg, Johan [John] Reinhold (Helsinki, Finland: 6 June 1845 – 8 May 1920: Helsinki, Finland). Finnish entomologist, specialist of Coleoptera and Homoptera; son of Reinhold Ferdinand Sahlberg [*q.v.*]; professor of entomology at the University of Helsinki; made expeditions to Finland, Karelia, Siberia, the Mediterranean region, and Central Asia; his types and one specimen of each species of his collection are at the Finnish Museum of Natural History, University of Helsinki. References. Saalas (1920, P; 1960, P); Smetana and Herman (2001: 133–134, P).

**1885**. *Bidrag till Tschuktsch-halföns Insektfauna. Coleoptera och Hemiptera, insamlade under* Vega-*expeditionen vid halföns norra och östra kust, 1878–1879.* F. & G. Beijers, Stockholm. Pp. 1–42. (8vo) [DP: n.d. (title page), 1 May 1885 (*Wien Ent Ztg* 4: 126), 13 May 1885 (*Soc Ent Fr*)] McG, MCZ

This paper was also issued, 1887, in “Vega-*expeditionens Vetenskapliga Iakttagelser bearbetade af deltagare i resan och andra forskare. Utgifna af A.E. Nordenskiöld. Fjerde Bandet. (Med 47 Taflor.)*” [GB, GAL]; the volume contains [4] + 582 pages + 47 plates and was noticed in November 1887 (*Nat Nov*). The entire report of the *Vega* expedition was published in five volumes, 1882–1887.

**1885**. *Coleoptera och Hemiptera, insamlade af* Vega-*expeditionens medlemmar å Berings sunds amerikanska kust uti omgifningarna af Port Clarence, vid Grantley Harbour och sjön Iman-Ruk den 23–26 juli 1879.* F. & G. Beijers, Stockholm. Pp. 43–57. (8vo) [DP: n.d. (title page), 1 May 1885 (*Wien Ent Ztg* 4: 126), 13 May 1885 (*Soc Ent Fr*)] McG

This paper was also issued, 1887, in “Vega-*expeditionens Vetenskapliga Iakttagelser bearbetade af deltagare i resan och andra forskare. Utgifna af A.E. Nordenskiöld. Fjerde Bandet. (Med 47 Taflor.)*” [GB, GAL].

**1885**. *Coleoptera och Hemiptera, insamlade af* Vega-*expeditionens medlemmar på Bering-ön den 15–18 augusti 1879.* F. & G. Beijers, Stockholm. Pp. 59–71. (8vo) [DP: n.d. (title page), 1 May 1885 (*Wien Ent Ztg* 4: 126), 13 May 1885 (*Soc Ent Fr*)] McG, MCZ

This paper was also issued, 1887, in “Vega-*expeditionens Vetenskapliga Iakttagelser bearbetade af deltagare i resan och andra forskare. Utgifna af A.E. Nordenskiöld. Fjerde Bandet. (Med 47 Taflor.)*” [GB, GAL].

#### Sahlberg, Reinhold Ferdinand (Turku, Turku ja Pori, Finland: 23 December 1811 – 18 March 1874: Yläne [currently part of Pöytyä], Turku ja Pori, Finland). Finnish physician and naturalist; son of Carl Reinhold Sahlberg [*q.v.*] and father of Johan Reinhold Sahlberg [*q.v.*]; participated to a round-the-world expedition (1839–1843); adjunct professor of zoology and botany at the University of Helsinki (1845–1852); travelled to Brazil (1849–1851); his Coleoptera collection is at the Zoological Museum, University of Turku and (particularly specimens from South America and Sitka) at the Finnish Museum of Natural History, University of Helsinki. References. Renvall (1869: 175–176); Smetana and Herman (2001: 134–135, P).

**1834**. *Dissertatio academica novas coleopterorum Fennicorum species sistens, quam cons. ampl. facult. philos. ad Imper. Univers. Alexandr. in Fennia, praeside Carolo Reginaldo Sahlberg, publicae submittit censurae auctor Reginaldus Ferdinandus Sahlberg Amanuensis ad Museum Universitatis Ordinarius, Satacundensis, in audit. philos. die 10 Maji 1834. H.a.m.c.* Frenckel, Helsingforsiae. 12 pp. (8vo) [DP: 10 May 1834 (title page), 15 September 1834 [JD] (*Soc Imp Nat Moscou*)] CMLE, GB

This dissertation, written by Reinhold Ferdinand Sahlberg and defended by one of his student, was also published, under the title “*Novae coleopterorum Fennicorum species*,” in *Bulletin de la Société Impériale des Naturalistes de Moscou* 7 [1834]: 267–280.

**1844**. *In faunam insectorum Rossicam symbola, novas ad Ochotsk lectas carabicorum species continens, quam, venia amplissimae facultatis philosophicae ad Universitatem Imperialem Alexandream in Fennia, P. P. Reginaldus Ferdinandus Sahlberg. Respondente Josepho Benjamino von Pfaler, Stipendiar. Publ. Satacund. In Auditorio Philos. die VII Decembris MDCCCXLIV. H.a.m.s.* Frenckel, Helsingforsiae. 69 pp. (8vo) [DP: 7 December 1844 (title page)] ANSP, GB, MDZ

This thesis contains the descriptions of 25 new species of Carabidae from Russia.

#### Saint-Amans, Jean Florimond Boudon de (Agen, Lot-et-Garonne, France: 24 June 1748 – 28 October 1831: Agen, France). French naturalist and antiquarian; served in the French expeditionary force in the French Antilles (1768–1773); later, professor of natural history at the college of Lot-et-Garonne department in Agen. References. Jouannet (1832); Ducourneau (1844: 317–320, P); Philbert (1847); Hoefer (1863 : 1022–1023); Chambers (2000: 388).

[**1800**]. *Philosophie entomologique, ouvrage qui renferme les généralités nécessaires pour s’initier dans l’étude des insectes, et des aperçus sur les rapports naturels de ces petits animaux avec les autres êtres organisés; suivi de l’exposition des méthodes de Géoffroi, et de celle de Linné combinée avec le système de Fabricius : pour servir d’introduction à la connoissance des insectes, en procurant le moyen de les classer et de les rapporter à leurs genres, dont on donne les caractères essentiels et la synonimie.* R.^d^ Noubel [&] Dugour, Agen [&] Paris. vii + [1] + 152 + [1 (Errata)] pp. (8vo) [DP: “An VII” (title page), 1799 (Hagen 1863: 103), 5 May 1800 (*Nouv Litt*), 25 May 1800 (*J Typogr Bibl*), 30 May 1800 (*Monit Univers*), 21 May–19 June 1800 (*J Lit Fr*)] GB, GAL

The Coleoptera are on pages 109–121. The book was also issued with an extra page, numbered “108(bis);” in addition the publishers are Noubel and Villier and there is no date on the title page [GB].

#### Salis-Marschlins, Carl Ulysses von (Marschlins Castle [currently in Igis, Canton of Graubünden], Switzerland: 28 September 1760 – 16 January 1818: Marschlins Castle, Switzerland). Swiss naturalist interested in botany, entomology, and conchology. Reference. Salis-Marschlins (1923).

**1793**. *Reisen in verschiedne Provinzen des Königreichs Neapel. Erster Band. Mit Kupfern.* Ziegler und Söhne, Zürich und Leipzig. 442 [numbered 12–77, 94–96, 81, 98–111, 96–442] + [4 (Druckfehler)] pp. + 10 pls. (8vo) [DP: 1793 (title page), 27 June 1793 (*Donns-Blatt*), 30 July 1793 (*J Encycl*), 16 November 1793 (*Handlungs-Ztg*)] GDZ

Only the first volume of Salis-Marschlins’ travels account was published. One new species of beetle, *Silpha choeradica* (p. 106), is described.

The book was translated in English under the title “*Travels through various provinces of the Kingdom of Naples in 1789. Translated from the German, by Anthony Aufrere, Esq. Illustrated with engravings*” and published in London, 1795 [GB].

#### Salvañá y Comas, Joaquín [Joaquim] Marià [Mariano] (Mataró, Catalonia, Spain: 21 January 1828 – 26 February 1902: Barcelona, Catalonia, Spain). Spanish naturalist particularly interested in molluscs; professor of applied chemistry at the Ateneu Mataroní in Mataró.

**1870**. *Apuntes para la geografía y fauna entomológicas de Mataró.* Gregorio Juste, Madrid. 44 pp. (8vo) [DP: 1870 (title page), 11 September 1870 (*Siglo Médico* 17: 586), 2 November 1870 (*J Pharm* (4) 12: 365)] EUR, GB, BDH

This work includes a list of the Coleoptera of Mataró in Spain (pp. 12–23). Two new species of beetles are described: *Meloe iluronensis* (pp. 37–38) and *Meloe ineditus* (p. 38).

#### Samouelle, George (*ca* 1790 – 13 February 1846: London, United Kingdom). British entomologist interested mainly in Lepidoptera; clerk in the book business establishment of Longman & Co. before assuming the position of curator of insects at the British Museum from 1821 to 1841; dismissed for excessive drinking, addressing his superiors with foul language, and removing registration numbers from labeled insects in the collection; established in 1826 with Edward Newman and two others a small, cozy and exclusive dining club (the Entomological Club) which survives to this day; had no personal collection. References. Harvey et al. (1996: 180); Evenhuis (1997b: 679); Salmon (2000: 137–138); Damkaer (2002: 167–168).

**1819**. *The entomologist’s useful compendium; or an introduction to the knowledge of British insects, comprising the best means of obtaining and preserving them, and a description of the apparatus generally used; together with the genera of Linné, and the modern method of arranging the classes Crustacea, Myriapoda, spiders, mites and insects, from their affinities and structure, according to the views of Dr. Leach. Also an explanation of the terms used in entomology; a calendar of the times of appearance and usual situations of near 3,000 species of British insects; with instructions for collecting and fitting up objects for the microscope. Illustrated with twelve plates.* Thomas Boys, London. 496 pp. + 12 pls. (8vo) [DP: 1819 (title page; dedication dated March 1819), May 1819 (*British Critic*), June 1819 (Peddie and Waddington 1914: 513; *Blackw Mag*), 1 July 1819 (*Monthly Mag*), 11 September 1819 (*Lit Gaz*), October 1819 (*Phil Mag*)] CNC, GB

The work was reissued in March 1824 [CMLE] by Longman, Hurst, Rees, Orme, Brown, and Green, London, under the same title except that the word “*near*” before “*3,000 species*” has been deleted. A second edition with “considerable alterations and additions, with fourteen plates” was announced in several British magazines in 1836 to be published in “about fourteen monthly parts.” As far as I know only the first two parts were published, in 1836 (*n.v.*).

**1819**. *A nomenclature of British entomology, or a catalogue of above 4,000 species of the classes Crustacea, Myriapoda, spiders, mites and insects, alphabetically arranged, and intended as labels for cabinets of British insects, etc. from the Entomologist’s useful compendium.* Thomas Boys, London. [1] + 44 pp. (8vo) [DP: 1819 (title page), 1 July 1819 (*Monthly Mag*), July 1819 (*Blackw Mag*)] CMLE (mf), GB

This work is essentially an index to “*The entomologist’s useful compendium*” [*q.v.*].

**1832–1834**. *The entomological cabinet; being a natural history of British insects.* London. (12mo) MCZ

[*Vol. I.*] xii + [133] pp. + [72] pls. This volume was published in 12 parts, each consisting of six unnumbered plates and 9–19 unnumbered pages of explanatory text. The dates of publication are taken from the plates (see Evenhuis 1997b: 681): **1**: ([10] pp. + [6] pls) 2 January 1832; **2**: ([14] pp. + [6] pls) 1 February 1832; **3**: ([10] pp. + [6] pls) 1 March 1832; **4**: ([10] pp. + [6] pls) 2 April 1832; **5**: ([19] pp. + [6] pls) 1 May 1832; **6**: ([10] pp. + [6] pls) 1 June 1832; **7**: ([10] pp. + [6] pls) 2 July 1832; **8**: ([9] pp. + [6] pls) 1 August 1832; **9**: ([10] pp. + [6] pls) 1 September 1832; **10**: ([10] pp. + [6] pls) 1 October 1832; **11**: ([10] pp. + [6] pls) 1 November 1832; **12**: ([11] pp. + [6] pls) 1 December 1832. The title page is dated 1833, the dedication 24 March 1832.

*Vol. II. Families of insects.* xii + [144] pp. + [84] pls. This volume was published in 14 parts, each consisting of six unnumbered plates and 7–15 unnumbered pages of explanatory text. The dates of publication are taken from the plates (see Evenhuis 1997b: 681): **13**: ([8] pp. + [6] pls) 1 January 1833; **14**: ([7] pp. + [6] pls) 1 February 1833; **15**: ([9] pp. + [6] pls) 1 March 1833; **16**: ([11] pp. + [6] pls) 2 April 1833; **17**: ([8] pp. + [6] pls) 1 May 1833; **18**: ([8] pp. + [6] pls) 1 June 1833; **19**: ([10] pp. + [6] pls) 1 July 1833; **20**: ([9] pp. + [6] pls) 1 August 1833; **21**: ([10] pp. + [6] pls) 1 October 1833; **22**: ([9] pp. + [6] pls) 1 November 1833; **23**: ([13] pp. + [6] pls) 1 December 1833; **24**: ([15] pp. + [6] pls) 1 February 1834; **25**: ([15] pp. + [6] pls) 1 April 1834; **26**: ([12] pp. + [6] pls) 1 July 1834. The title page is dated 1834 and the *address* 6 February 1834.

There are 71 plates that pertain to Coleoptera; one new species is described, *Berosus apicalis* (pl. 5–1).

A one-volume second edition was published in 1841 by G. Henderson, London [BHL]. It is essentially a reissued of the first edition except for the preliminaries and in the erasure of the dates from the plates of the first five parts (Townsend 1940: 1134).

#### Saunders, Edward (Wandsworth [currently part of Greater London], United Kingdom: 22 March 1848 – 6 February 1910: Bognor Regis, West Sussex, United Kingdom). British entomologist, interested first in Coleoptera, then in Hemiptera and finally in aculeate Hymenoptera; worked at Lloyd’s, a London based insurance and reinsurance market; his British Coleoptera went to the Hunterian Museum, University of Glasgow, via Thomas George Bishop and his Buprestidae are at the Natural History Museum in London. References. Anonymous (1910b, P); Poulton (1920, P); Foote (2004c).

**1869**. *Insecta Saundersiana: or characters of undescribed species in the collection of William Wilson Saunders, Esq., F.R.S., F.L.S., &c. Vol. III. Buprestidae. Part I.* John van Voorst, London. [2] + 27 pp. + 2 pls. (8vo) [DP: 1869 (title page; preface dated 14 June 1869)] CNC

**1870**. *Catalogue of the species contained in the genus* Buprestis *of Linnaeus, previous to its subdivision by Eschscholtz in 1829, referring each to its present genus.* John van Voorst, London. v + [1] + 7–37 pp. (8vo) [DP: November 1870 (title page; preface dated 13 October 1870)] CMLE, GB

**1871**. *Catalogus buprestidarum synonymicus et systematicus.* E.W. Janson, London. xxiii + [1] + 171 pp. (8vo) [DP: September 1871 (title page; preface dated 11 September 1871), 1 November 1871 (*Pet Nouv Ent*), 6 November 1871 (*Ent Soc Lond*)] CMLE, GB

#### Say, Thomas (Philadelphia, Pennsylvania, USA: 27 June 1787 – 10 October 1834: New Harmony, Indiana, USA). American naturalist; founding member of the Academy of Natural Sciences in Philadelphia; explored coastal Georgia and eastern Florida (1817–1818); appointed as zoologist on Major Long’s expeditions to the Rocky Mountains (1819–1820) and to St. Peters River, Lake Winnipeg, and Lake of the Woods (1823); curator at the Academy of Natural Sciences of Philadelphia (1812–1826); later, settled in New Harmony, Indiana (1826–1834); most of his collection has been destroyed but 770 of his specimens are conserved in the Museum of Comparative Zoology at Cambridge. References. Weiss and Ziegler (1931, P); Mallis (1971: 16–25, P); Adler (1989: 26–27, P); Stroud (1992, P); Mawdsley (1993); Heppner (2009, P).

**1817**. *American entomology, or descriptions of the insects of North America. Illustrated by coloured figures from drawings executed from nature.* Michell & Ames, Philadelphia. x + [26] pp. + 6 pls. (8vo) [DP: 1817 (title page), September 1817 (*Analec Mag*), December 1817 (*Amer Monthly Mag*)] MCZ, BHL, GB

The Coleoptera illustrated and described are: *Geotrupes tityus* (pl. 2), *Nemognatha immaculata* (pl. 3), *Notoxus monodon* and *Notoxus bicolor* (pl. 4), *Cicindela formosa* and *Cicindela decemnotata* (pl. 6). The booklet was reissued in 1958 by Lost Cause Press, Louisville.

This booklet is extremely rare since it was printed more as a prospectus and only in a small quantity (Heppner 2009: 85). The text and plates were included in Say (1824) who mentioned (p. vii) that the 1817 issue was “never properly published.”

**1824**. Appendix. Part I. – Natural History. 1. Zoology. Pp. 253–378 + pls 14–15 *in*: *Narrative of an expedition to the source of St. Peter’s River, Lake Winnepeek, Lake of the Woods, &c. &c. performed in the year 1823, by order of the Hon. J.C. Calhoun, Secretary of War, under the command of Stephen H. Long, Major U.S.T.E. Compiled from the notes of Major Long, Messrs. Say, Keating, and Colhoun, by William H. Keating, A.M. &c. In two volumes. Vol. II.* H.C. Carey & I. Lea, Philadelphia. vi + 459 pp. + pls 6–15. (8vo) [DP: 1824 (title page; preface in first volume dated 29 November 1824), 1 February 1825 (*US Lit Gaz*), April 1825 (*North Amer Rev*), 8 July 1825 (*New Eng Farmer*)] BOT, GB, BHL

The expedition report was published in two volumes. Say’s contribution is in the second volume. A British edition of this work was published in April 1825 by Whittaker, London, and contains vi + 248 + 154 (Appendix) pages and three plates [MCZ, GB], with the same title except that one “*&c.*” and the word “*Major*” between “*Long*” and “*U.S.T.E.*” are left out. Say’s contribution is on pages 3–123 of the Appendix. A reedition, without parts I–III of the Appendix, was published in 1959 by Ross & Haines, Minneapolis, under the same title except for the fact that one “*&c.*” is left out and the end is “*Keating, & Colhoun. In one volume.*”

**1824–1828**. *American entomology, or descriptions of the insects of North America. Illustrated by coloured figures from original drawings executed from nature.* Samuel Augustus Mitchell, Philadelphia. (8vo) McG, BHL

[*Vol. I*]. viii + [105] pp. + pls 1–18. [DP: 1824 (title page), November 1824 (*Can Mag*), 14 December 1824 (*Acad Nat Sci Phila*), January 1825 (*North Amer Rev*)].

[*Vol. II*]. [121] pp. + pls 19–36. [DP: 1825 (title page), 25 March 1826 (*Lit Gaz*)]. The date of July 1825 listed by Evenhuis (1994: 543; 1997b: 688) and taken from *The North American Review* refers to the first volume.

[*Vol. III*]. [136] pp. + pls 37–54. [DP: 1828 (title page), August 1828 (Stroud 1992: 214)].

The three volumes were published without volume numbers and pagination. The text was reprinted in LeConte 1859 [*q.v.*]. In 1836, Lequien fils announced its intention to publish a French translation of this work in 15 or 16 livraisons, as part of his series *Bibliothèque entomologique*, under the title “*Oeuvres entomologiques de Th. Say, contenant l’Entomolgique américaine, les mémoires insérés dans le Journal de l’Académie des sciences naturelles de Philadelphie, dans les Transactions de la Société philosophique d’Amérique, dans le Journal de Boston, etc., etc. Recueillies et traduites par M.A. Gory*.” Only four livraisons were published, all in 1837, and none included part of this work.

**1830–1834**. *Descriptions of new species of North American insects, and observations on some of the species already described.* New Harmony [Indiana]. 81 pp. (8vo) HOU

This booklet was issued in six parts, as follows: **1**: (pp. [1]–41) 1830 (Harris 1869: 222), with pages 18–41 printed on 20 August 1830 (bottom of p. 19); **2**: (pp. 42–49) 1831 (Harris 1869: 222), 11 August 1831 (received by Harris, see Scudder 1899: 414); **3**: (pp. 50–57), 1832 (Harris 1869: 222), 24 September 1832 (received by Harris, see Scudder 1899: 414); **4**: (pp. 58–65) 1833 (Harris 1869: 223), 21 July 1833 (received by Harris, see Scudder 1899: 414); **5**: (pp. 66–73 [incorrectly numbered 46–53]) 1834 (Harris 1869: 223), 21 July 1834 (received by Harris, see Scudder 1899: 414); **6**: (pp. 73½–80) 1 August 1834 (printed date on the paper, see Scudder 1899: 413). The first 17 pages, which are not numbered, are reprints of Say’s short papers published in the third volume of *The Disseminator of useful knowledge* (pp. [1–4] in double columns) and the first volume of *The Disseminator* (pp. [5–17] in single column). This publication was reissued, with minor editorial changes, in the *Transactions of the American Philosophical Society* (new series) 4 [1834]: 409–470 and 6 [1839]: 155–190. The only complete copy I know is in Houghton Library at Harvard University.

**1831**. *Descriptions of new species of North American insects, found in Louisiana by Joseph Barabino.* School Press, New Harmony [Indiana]. 17 [numbered 3–19] pp. (8vo) [DP: March 1831 (title page)] HOU, YB (ph)

The new species of Coleoptera described are *Cupes cinerea* (p. 6), *Hydrophilus castus* (p. 7), *Trox alternatus* (p. 7), *Tenebrio rufinasus* (p. 8), *Oedemera apicialis* (p. 9), *Acanthocinus quadrigibbus* (p. 9), *Altica mellicollis* (p. 10) and *Scymnus terminatus* (p. 11). The four other beetles described in the booklet were first described in previous papers of Say.

**1831–1832**. *Descriptions of new species of curculionites of North America, with observations on some of the species already known.* School Press, New Harmony [Indiana]. 30 pp. (8vo) YB (ph)

The second page of the booklet bears the title “*Descriptions of North American curculionides & an arrangement of some of our known species agreeably to the method of Schoenherr*.” This pamphlet consists of four signatures, each of eight printed pages (Prena 2015: 402). The first signature (pp. 1–8) was probably issued in July 1831 as indicated on the title page and the remaining ones (pp. 9–30) likely in 1832 (Prena 2015: 403). Harris received the second and third signatures on 5 May 1832 and the fourth signature (pp. 25–30) on 26 December 1832 (Scudder 1899: 400). Pages 25–30 has a separate title reading “Supplement.”

**1832**. *New species of North American insects, found by Joseph Barabino, chiefly in Louisiana.* School Press, New Harmony [Indiana]. 16 pp. (8vo) [DP: January 1832 (title page)] YB (ph)

The new species of Coleoptera described are *Buprestis thureura* (p. 3), *Dermestes nubilus* (p. 3), *Ateuchus humectus* (p. 4), *Trox aequalis* (p. 5), *Lamia crypta* (p. 5), *Altica exapta* (p. 6) and *Altica ocreata* (p. 7).

#### Schäffer [Schaeffer], Jacob Christian Gottlieb von (Querfurt, Saxony-Anhalt, Germany: 30 May 1718 – 5 January 1790: Regensburg, Bavaria, Germany). German clergyman, naturalist, private teacher, and paper maker in Regensburg. References. Meusel (1812: 71–79); Jourdan (1825); Fürnrohr (1838: 28–54); Wunschmann (1890); Fürnrohr (1908, P).

**1764**. *Opvscvla entomologica qvae jam institvta habet avspiciis avgvstissimi potentis simiqve Daniae et Norvegiae Regis Friderici V. proxime edenda indicit eorvmqve specimina qvaedam exhibet. / Nachricht und Proben von der unter huldreichster Förderung Sr. Königl. Maj. zu Dännemark Norwegen Friederich des V^ten^ nächstens zu liefernden Herausgabe gewisser unternommener Insectenwerke.* Johann Leopold Montag, Regensburg. 15 + [6 (Specimina. Proben.)] pp. + 3 pls. (4to) [DP: 1764 (title page; dedication dated September 1764), 1 October 1764^92^, 24 January 1765 (*Neue Ztg*)] MCZ, GB

The text is in Latin and German. The six unnumbered pages appear to be a sample of his 1766 publication [*q.v.*]. In the sample, part of one of his forthcoming table is included where the generic name “*Thelophorus*” is described. This genus is currently credited to Schäffer’s *Elementa entomologica* (1766) under the spelling *Telephorus*.

**1764–1779**. *Abhandlungen von Insecten.* Johann Leopold Montag, Regensburg. (4to) GB

*Erster Band. Nebst XVI. Kupfertafeln mit ausgemahlten Abbildungen.* xl + 402 pp. + 16 pls [DP: 1764 (title page)]. The section “*III. Verschiedene Zwiefalter und Käfer mit Hörnern*” (pp. 113–152 + 3 pls) pertains to Coleoptera; it was first issued in 1758 under the same title. Engelmann (1846: 506) listed a second “Aufl[age]” of this section issued in 1763 (*n.v.*).

*Zweyter Band. Nebst XVIII. Kupfertafeln mit ausgemahlten Abbildungen.* 344 pp. + 18 pls [DP: 1764 (title page)]. The section “*VII. Der weichschaalige Cronen- und Käulenkäfer*” (pp. 289–312 + 1pl.) pertains to Coleoptera.

*Dritter und lezter Band. Nebst XIV. Kupfertafeln mit ausgemahlten Abbildungen.* [4] + 158 pp. + 14 pls [DP: 1779 (title page; *Vorrede* dated 2 January 1779)]. The following sections pertain to Coleoptera: *III. Der flügellose Blattkäfer* (pp. 51–64 + 2 pls); *IV. Der Blasenblattkäfer* (pp. 65–80 + 1 pl.); *VII. Der Geiferkäfer* (pp. 99–108 + 1 pl.); *IX. Der Kropfkrautsrüsselkäfer* (pp. 119–124 + 1 pl.).

**1766**. *Elementa entomologica. CXXXV. tabvlae aere excvsae floridisqve coloribvs distinctae. / Einleitung in die Insectenkenntnis. CXXXV. ausgemahlte Kupfertafeln.* Weiss, Ratisbonae [= Regensburg]. Pl. 135 + [6] + [12] pp. + pls 1–12 + [1] pp. + pl. 13 + [16] + [119] pp. + pls 14–132 + [3] pp. + pls 133–134 + [3] pp. (4to) [DP: 1766 (title page; *praefatio* dated 1 January 1766), 14 April 1766 (*Neue Hall Ztg*), 20 April 1766^93^, July 1766 (*Gaz Litt Eur-Paris*), 5 September 1766 (*Jena Ztg*)] CNC, GB

The work contains the following sections: *Sectio I. De insectorvm strvctvra et facie externa. Erster Abschnitt. Von dem aeusserlichen baue und der Gestalt der Insecten* ([12] pp. + pls 1–12); *Sectio II. De classibvs insectorvm. Zweyter Abschnitt. Von den Classen der Insecten* ([1] p. + pl. 13); *Sectio III. De generibvs insectorvm. Dritter Abschnitt. Von den Geschlechtern der Insecten* ([16 (Tabvla genervm characteristica)] + [119] + pls 14–132); *Sectio IV. De instrvmentis et ratione insecta commode captandi tractandi et adservandi. Vierter Abschnitt. Von der Werkzeugen der Behandlung und Sammlung der Insecten* ([3] pp. + pls 133–134); *Index. Register* ([3] pp.). The half-title page at the beginning is followed by pl. 135.

An appendix to this work was published in 1777 [*q.v.*]. The book was reissued, also in Regensburg, in 1777 with the appendix under the title “*Elementa entomologica cvm appendice. CXL tabvlae aeri incisae et nigrae. Einleitvng in die Insectenkenntnis mit einem Nachtrage. CXL schwarze Kvpfertafeln*” [GB]. A “second edition” was published in 1778 under the title “*Elementa entomologica cvm appendice. Einleitvng in die Insectenkenntnis mit einem Nachtrage. CXL tabvlae aeri excvsae floridisqve coloribvs distinctae. Editio secvnda*” [SDL] and a “third edition” in 1780 under the title “*Elementa entomologica cvm appendice. Einleitung in die Insectenkenntnis mit einem Nachtrage. CXL tabvlae aeri incisae floridisqve coloribvs distinctae. Editio tertia*” [GB]. Fürnrohr (1838: 44) mentioned another “edition” in 1787 (*n.v.*).

**1766–[1776**]. *Icones insectorvm circa Ratisbonam indigenorvm coloribvs natvram referentibvs expressae. / Natürlich ausgemahlte Abbildungen Regensburgischer Insecten.* Heinrich Gottfried Zunkel [vols 1–2] / Weiss [vol. 3], Regensburg. (4to) GDZ

*Volvm. I. Pars I. Ersten Bandes erster Theil.* [6] + [50] pp. + pls 1–50. [DP: n.d. (title page; *praefatio* dated August 1766), 24 November 1766 (*Jena Ztg*), June 1767 (*Gaz Litt Eur-Amsterdam*)].

*Volvm. I. Pars II. Erster Band. Zweyter Theil.* [6] + [50] + [12 (Index)] pp. + pls 51–100. [DP: n.d. (title page), late 1766 or early 1767 (Kerzhner 2005: 74), 13 March 1767 (*Jena Ztg*)].

*Volvm. II. Pars I. Zweyten Bandes erster Theil.* [4] + [50] pp. + pls 101–150. [DP: n.d. (title page)].

*Volvm. II. Pars II. Zweyter Band. Zweyter Theil.* [4] + [50] + [6 (Index)] pp. + pls 151–200. [DP: n.d. (title page), 1769 (Prange 1784: 195; Hagen 1863: 114)].

*Volvm. III. et vltimvm. Dritter und letzter Band.* [4] + [80] + [6 (Index)] pp. + pls 201–280. [DP: n.d. (title page)]. Evenhuis (1997b: 692) noted that this volume was listed as being shown at the 1776 Leipzig Easter Book Fair, which was held on 28 April 1776 (Evenhuis 2014a: 4).

The dates of publication of the five parts of this work are problematic. Kolenati (1845: 10) mentioned that the first volume was published in 1766 and the other two in 1769. Agassiz (1854: 255) gave the date 1766–1769 and Brunet (1865: 332) and Schönherr (1806: xix) dated the three volumes 1766 and 1769 respectively. The three volumes were recorded as published 1766–1775 (Anonymous 1777a: 199). Lacordaire (1838: 648), Engelmann (1846: 506), Hagen (1863: 114), Horn and Schenkling (1928c: 1053) and Evenhuis (1997b: 692) mentioned that the work was published 1766–1779.

A second printing of this work was issued by Breitfeld, in Ratisbonae [=Regensburg], in 1779 [GB]. Another printing of the three volumes was published in the same city, 1791 [BHL]. The text is written in both Latin and German.

Schäffer provided only generic names to the insects illustrated; specific names were supplied by Harrer 1784 [*q.v.*] and Panzer 1804 [*q.v.*].

**1777**. *Elementorvm entomologicorvm appendix qvinqve insectorvm nova genera exhibens. / Fvnf neve Insectengeschlechter zvr Einleitvng in die Insectenkenntnis. Tabvlae V. vivis coloribvs expressae*. Ratisbonae [= Regensburg]. [5] pp. + pls 136–140. [DP: 1777 (title page), 20 April 1777^94^, August 1777 (*Allg Verz Bücher*)] SDL

This publication is an appendix to *Elementa entomologica* issued in 1766 [*q.v.*].

#### Schaufuss, Ludwig Wilhelm (Greiz, Thuringia, Germany: 24 August 1833 – 16 July 1890: Meissen, Saxony, Germany). German zoologist and natural history dealer in Dresden; provided material to private collectors and institutions from around the world; built at his expenses a museum in Dresden which he named “Museum Ludwig Salvator;” editor of the journal “*Numquam Otiosus*” [1870–1890] for which he wrote most articles; his personal collection and those of the museum were passed on to his son, Camillo Schaufuss, who eventually scattered them; nevertheless, many of Schaufuss specimens are at the Deutsches Entomologisches Institut in Müncheberg and the Zoologisches Museum der Humboldt-Universität in Berlin. References. Hinrichsen (1887: 547–548); Anonymous (1891b, P); Anonymous (2013d, P).

**1869**. *Beitrag zur Kenntniss der Coleopteren-Fauna der Balearen*. Prag. 31 pp. (8vo) [DP: 1869 (title page; preface dated January 1869), 7 April 1869 (*Zool-Bot Gesell Wien*), 3 May 1869 (*Ent Soc Lond*)] CNC

According to Rye (1870: 185) this booklet, published anonymously by the Archduke Luis Salvador of Austria [Ludwig Salvator von Österreich-Toskana of his real name], is virtually the work of Schaufuss, who determined the species and described those that were new (16 in number). The 332 species enumerated result from a stay during 1867 in the Balearic isles by the Archduke, and from a visit of a few days to Majorca and Minorca by Schaufuss and Samuel Brannan in 1866.

**1877**. *Pselaphiden Siam’s.* Ferdinand Thomass, Dresden. 25 pp. (4to) [DP: June 1877 (title page), 15 July 1877 (*Pet Nouv Ent*)] CNC

This pamphlet contains descriptions of new genera and species of pselaphids from Thailand in the author’s collection.

**1880**. *Der Société entomologique de Belgique zu Brüssel zur Feier ihres fünfundzwanzigsten Stiftungstages die herzlichsten Festgrüsse aus dem Museum Ludwig Salvator in Oberblasewitz-Dresden. (Anhang: Schaufuss, Sechzig neue Pselaphiden.)* F. Thomass, Dresden-Oberblasewitz. 35 pp. (8vo) [DP: 16 October 1880 (title page)] Online

This work, in which the author describes 60 new species of pselaphids, was also issued the same date in *Nunquam Otiosus* 3 [1879]: 481–511 [DP: 16 October 1880 (wrapper)].

#### Schaum, Hermann Rudolph (Glauchau, Saxony, Germany: 29 April 1819 – 15 September 1865: Bonn, North Rhine-Westphalia, Germany). German physician and entomologist interested particularly in Coleoptera; practiced medicine in Stettin; later, professor of medicine at the University of Berlin; travelled to North America (1847–1849); his collection, which included part of his uncle Ernst Friedrich Germar’s collection [*q.v.*], was sold to different parties. References. Kiesenwetter (1866); Ratzeburg (1874: 456–461).

**1841**. *Symbolae ad monographiam Scydmaenorum, insectorum generis. Dissertatio inauguralis medica quam consensu et auctoritate gratiosi medicorum ordinis Halensis ut summos in medicina et chirurgia honores rite adipiscatur d. XXX m. Novembr. a. MDCCCXLI una cum thesibus publice defendet auctor Herm. Rud. Schaum Glauchaviensis. Opponentibus: H.W. Cordts, Dr. Med., S. v.d. Porten, D.D.* Fr. Schimmelpfennig, Halis [= Halle]. [4] + 31 + [2] pp. (8vo) [DP: 30 November 1841 (title page)] CNC (mf), MDZ

This work, which consists of a revision of the world species of *Scydmaenus*, was also published the same year in *Analecta entomologica* (see next entry).

**1841**. *Analecta entomologica. (Dissertatio inauguralis.) Cum tabula aenea.* Voss, Halis Saxonum [= Halle an der Saale]. 49 pp. + 1 pl. (8vo) [DP: 1841 (title page), 15 December 1841 (*Lit Ztg*), 17 December 1841 (*Allg Bibl Deutsch*), July–December 1841 (*Verz Bücher Landkarten*)] MCZ, GB

This volume contains four sections: *Symbolae ad monographiam Scydmaenorum* (pp. 1–31); *Quaedam de characteribus Cremastochilorum* (pp. 32–33); *Notae criticae ad familiam Cetoniarum* (pp. 34–39); *Decas novarum Cetonidarum* (pp. 40–49).

**1848**. *Verzeichniss der Lamellicornia melitophila*. Ploetzian, Stettin. iv + 5–74 pp. (8vo) [DP: 1848 (title page), April 1848 (*Ent Ztg* 9: 128), 16 September 1848 [JD] (*Soc Imp Nat Moscou*), 5 April 1849 (*Ent Ver Stettin*)] CMLE (mf), CNC (mf)

This is a catalogue of the world cetoniines (family Scarabaeidae).

**1852**. *Catalogus coleopterorum Europae. Herausgegeben vom entomologischen Verein in Stettin. Vierte Auflage.* Trowitzsch und Sohn, Berlin. v + 96 + 12 pp. (8vo) [DP: 1852 (title page; preliminaries dated 1 August 1852), November 1852 (*Ent Ztg* 13: 408), July–December 1852 (*Bibliot Hist-Nat*), 7 February 1853 (*Ent Soc Lond*)] CMLE

The preliminaries are signed by Hermann Rudolph Schaum to whom this catalogue is usually credited.

**1856–1860**. *Naturgeschichte der Insecten Deutschlands begonnen von Dr. W.F. Erichson, fortgesetzt von Prof. Dr. H. Schaum, Dr. G. Kraatz und H. v. Kiesenwetter. Erste Abtheilung. Coleoptera. Erster Band. Erste Hälfte.* Nicolai, Berlin. vi + 791 pp. (8vo) CNC

This volume was published in four *Hefte*. The dates of publication of the first three are indicated in the *Vorrede* of the work. **1**: (pp. 1–190) April 1856, 17 July 1856 (*Allg Bibl Deutsch*); **2**: (pp. 191–352) March 1857, 10 September 1857 (*Allg Bibl Deutsch*), 30 November 1857 (*Intell Serapeum*); **3**: (pp. 353–552) May 1858, 24 June 1858 (*Allg Bibl Deutsch*), July 1858 (*Allg Bibl*); **4**: (pp. 553–791 + i–vi) 7 May 1860 (*Ent Soc Lond*), 19 July 1860 (*Allg Bibl Deutsch*), August 1860 (*Allg Bibl*), 4 September 1860 (*Acad Nat Sci Phila*), July–December 1860 (Griffin 1939a: 218). The title page is dated 1860 and the preface February 1860.

The second *Hälft* of this work was authored by Schaum and Kiesenwetter, 1868 [*q.v.*].

**1859**. *Catalogus coleopterorum Europae. In Verbindung mit Dr. G. Kraatz und H.v. Kiesenwetter.* Nicolai, Berlin. iv +121 pp. (8vo) [DP: 1859 (title page; *Vorwort* dated November 1858), February 1859 (*Wien Ent Monats* 3: 64), 13 April 1859 (*Soc Ent Fr*), April 1859 (*Allg Bibl*)] SME, GB

**1862**. *Catalogus coleopterorum Europae. Editio secunda aucta et emendata.* Friderici Nicolai, Berolini [= Berlin]. 130 pp. (8vo) [DP: 1862 (title page), 7 April 1862 (*Ent Soc Lond*), 17 April 1862 (*Allg Bibl Deutsch*), 30 April 1862 (*Intell Serapeum*)] SME, GB

**1867**. [Schaum, H.R. and Kiesenwetter, E.A.H.] *Naturgeschichte der Insecten Deutschlands begonnen von Dr. W.F. Erichson fortgesetzt von H. Schaum, G. Kraatz und H. v. Kiesenwetter. Erste Abtheilung. Coleoptera. Erster Band. Zweite Hälfte.* Nicolai, Berlin. [1] + 144 pp. (8vo) [DP: 1868 (title page), 19 December 1867 (*Allg Bibl Deutsch*), July–December 1867 (*Bibliot Hist-Nat*), 30 June 1868 (*Intell Serapeum*), 25 July 1868 (*Lit Centr Deutsch*)] CNC, GB

The first *Hälft* of this work was authored by Schaum alone, 1856–1860 [*q.v.*].

#### Schellenberg, Johann Rudolf [Rudolph] (Basel, Switzerland: 4 January 1740 – 6 August 1806: Winterthur, Switzerland). Swiss natural history illustrator, engraver and poet. References. Brunn (1890); Weidner (1983: 310–311); Thanner et al. (1987, P); Bürger (1997: 114–118).

**1802**. *Entomologische Beyträge. Erstes Heft, mit 10 illuminirten Kupfertafeln.* Steiner, Winterthur. 24 pp. + 10 pls. (4to) [DP: 1802 (title page), 21 October 1803 (*Allg Lit Ztg*)] GDZ

Three species of Coleoptera are described and illustrated in this booklet: *Lymexylon dermnestoides* [sic!] Fabricius (plate I); *Lymexylon proboscideum* (plate II); and *Lymexylon biguttatum* (plate III). Only the last one represents a new species.

#### Schenkling, Carl [Karl] Gotthilf (Döblitz [currently part of Löbejün-Wettin], Saxony-Anhalt, Germany: 18 February 1835 – 20 September 1911: Laucha an der Unstrut, Saxony-Anhalt, Germany). German coleopterist and professor in Laucha. Reference. John (1911, P).

**1884–[1886**]. *Die deutsche Käferwelt. Allgemeine Naturgeschichte der Käfer Deutschlands sowie ein praktischer Wegweiser, die deutschen Käfer leicht und sicher bestimmen zu lernen. Mit 1 schwarzen und 23 Farbendruck-Tafeln.* Oskar Leiner, Leipzig. xxxviii + 434 pp. + 24 pls. (8vo) YB

This book was issued in 11 *Lieferungen* as follows: **1**: (pp. 1–48 + 3 pls) 1–15 November 1884 (*Humboldt* 4: 43), November 1884 (*Zool Gart*), 19 December 1884 (*Phys Gesell Berl*), July–December 1884 (*Bibliot Hist-Nat*), 15 January 1885 (*Wien Ent Ztg* 4: 31); **2–3**: (pp. 49–144) September 1885 (*Nat Nov*); **4–5**: (pp. 145–240) September 1885 (*Nat Nov*; *Humboldt* 4: 456); **6–7**: (pp. 241–304) October 1885 (*Humboldt* 4: 492), December 1885 (*Nat Nov*); **8–9**: (pp. 305–?400) December 1885 (*Humboldt* 5: 74), January 1886 (*Nat Nov*); **10–11**: (pp. ?401–434 + i–xxxviii) March 1886 (*Nat Nov*), 5 June 1886 (*Lit Centr Deutsch*), January–June 1886 (*Bibliot Hist-Nat*), 14 August 1886 (*Naturwiss Rund*). There is no date on the title page and the *Vorwort* is dated 1885.

#### Scheuereck [Scheureck], Friedrich [Fridrich] August. Artist and engraver in Leipzig.

**1796**. *Unterhaltungen in der Naturgeschichte aller Arten Insekten zum nützlichen Gebrauch für die Jugend aus verschiedenen Schriften berühmter Naturforscher zusammengetragen, mehrentheils aber aus eignen Beobachtungen und Erfahrungen verfertiger. Erster Band. Von den Käfern. Mit Kupfern.* Johann Heinrich Kaven, Altona und Leipzig. [4] + 815 + [1] pp. + 22 pls. (8vo) [DP: 1796 (title page)] BHL

This volume, published under the initials “F.A.S.,” treats the Coleoptera (pp. 47–62, 72–574) and “Hemiptera” (pp. 63–71, 575–771). A second *Band*, pertaining to Lepidoptera, was issued in 1799.

#### Schilling, Peter Samuel (Juliusburg, Schleswig-Holstein, Germany: 10 April 1773 – 15 December 1852: Breslau [currently Wrocław], Poland). German educator and naturalist; professor of natural history at the Maria Magdalenen Gymnasium in Breslau from 1798 to 1843. References. Schönborn (1853: 23–24); Anonymous (1854: 60–61); Meister (1893: 45).

**1839**. *Ausführliche Naturgeschichte des Thier-, Pflanzen- und Mineralreichs. Ein Handbuch für alle Stände, systematisch bearbeitet. Dritter Band. Thier-Reich: Fische und wirbellose Thiere. Mit 78 Tafeln Abbildungen.* Heinrich Richter, Breslau [= Wrocław]. viii + 636 + [1] pp. + 78 pls. (8vo) [DP: 1839 (title page)] GB

An additional title page reads “*Ausführliche Naturgeschichte der Fische und der wirbellosen Thiere in systematischer Ordnung mit vielen Abbildungen.*” The entire series was issued in four volumes, 1837–1841. The Coleoptera are on pages 286–379 of the third volume. A second edition, apparently simply a reissued of the first edition, was published in 1843 (*n.v.*) and noticed on 28 January 1843 (*Lit Ztg*).

#### Schilsky, Friedrich Julius (Groß-Neuendorf, Branderburg, Germany: 9 February 1848 – 17 August 1912: Berlin, Germany). German entomologist, specialist on Coleoptera; public school teacher in Berlin; his collection is at the Zoologisches Museum der Humboldt-Universität in Berlin. References. Horn (1912, P); Pape (1912).

**1888**. *Systematisches Verzeichnis der Käfer Deutschlands mit besonderer Berücksichtigung ihrer geographischen Verbreitung. Zugleich ein Käfer-Verzeichnis der Mark Brandenburg.* Nicolai (R. Stricker), Berlin. vi + [2] + 159 pp. (8vo) [DP: 1888 (title page; *Vorbemerkung* dated April 1888), May 1888 (*Humboldt* 7: 287), June 1888 (*Nat Nov*), 31 July 1888 (*Wien Ent Ztg* 7: 227), August 1888 (*Ent Ztg* 49: 218)] CMLE, GB

Additions and corrections to this work were published in *Deutsche Entomologische Zeitschrift* 1888: 321–328; 1889: 337–344, 357–365.

**1894–1912**. *Die Käfer Europa’s. Nach der Natur beschrieben von Dr. H.C. Küster und Dr. G. Kraatz. Fortgesetzt von J. Schilsky.* Bauer & Raspe, Nürnberg. (8vo) CNC

*Dreissigstes Heft.* viii + [258] pp. [DP: 1894 (title page; *Vorwort* dated March 1894), April 1894 (*Nat Nov*)].

*Einunddreissigstes Heft.* viii + [331] pp. [DP: 1895 (title page; *Vorwort* dated February 1895), March 1895 (*Nat Nov*; *Zool Anz*)].

*Zweiunddreissigstes Heft. Mit 1 Kupfertafel und 1 Textfigur.* viii + [330] pp. + 1 pl. [DP: 1896 (title page; *Vorwort* dated February 1896), April 1896 (*Nat Nov*), 15 May 1896 (*Wien Ent Ztg* 15: 190), 10 June 1896 (*Soc Ent Fr*), 13 June 1896 (*Lit Centr Deutsch*)].

*Dreiunddreissigstes Heft.* iv + [375] pp. [DP: 1897 (title page; *Inhalt* dated March 1897), March 1897 (*Nat Nov*), 3 May 1897 (*Bibl Zool*), July 1897 (*Deutsch Ent Zeit* 1897: 207)].

*Vierunddreissigstes Heft.* viii + [382] pp. [DP: 1897 (title page; *Vorwort* dated December 1897), December 1897 (*Deutsch Ent Zeit* 1897: 416), 24 January 1898 (*Bibl Zool*), January 1898 (*Nat Nov*), 28 February 1898 (*Wien Ent Ztg* 17: 72)].

*Fünfunddreissigstes Heft.* viii + [355] pp. [DP: 1898 (title page; *Vorwort* dated December 1898), December 1898 (*Nat Nov*), 22 February 1899 (*Soc Ent Fr*), 27 March 1899 (*Bibl Zool*)].

*Sechsunddreissigstes Heft.* iv + [354] pp. [DP: 1899 (title page; *Inhalt* dated December 1899), 1900 (wrapper), 19 January 1900 (*Bibl Zool*), January 1900 (*Nat Nov*), 10 March 1900 (*Wien Ent Ztg* 19: 87)].

*Siebenunddreissigstes Heft.* iv + [288] pp. [DP: 1900 (title page; *Inhalt* dated December 1900), 21 December 1900 (Sharp 1901: 67), 1901 (wrapper), January 1901 (*Nat Nov*), 1 February 1901 (*Bibl Zool*)].

*Achtunddreissigstes Heft.* vi + [362] pp. [DP: 1901 (title page; *Inhalt* dated December 1901), end of 1901 (Sharp 1902: 65), 1902 (wrapper), January 1902 (*Nat Nov*), 26 February 1902 (*Soc Ent Fr*), 7 March 1902 (*Bibl Zool*)].

*Neununddreissigstes Heft.* iv + [356] pp. [DP: 1902 (title page; *Inhalt* dated December 1902), December 1902 (*Nat Nov*), 14 February 1903 (*Bibl Zool*)].

*Vierzigstes Heft.* viii + [468] pp. [DP: 1903 (title page; *Inhalt* dated December 1903), 15 February 1904 (*Soc Ent*), 20 April 1904 (*Wien Ent Ztg* 23: 80)].

*Einundvierzigstes Heft.* iv + [476] pp. [DP: 1905 (title page; *Inhalt* dated March 1905), May 1905 (*Nat Nov*), 1 June 1905 (*Wien Ent Ztg* 24: 207)].

*Zweiundvierzigstes Heft.* vi + [374] pp. [DP: 1906 (title page; *Vorwort* dated February 1906), March 1906 (*Nat Nov*), April 1906 (*Ent Litt*)].

*Dreiundvierzigstes Heft.* viii + cxix + [107] pp. [DP: 1906 (title page; *Vorwort* dated April 1906), May 1906 (*Nat Nov*), July 1906 (*Ent Litt*)].

*Vierundvierzigstes Heft.* iv + [426] pp. [DP: 1907 (title page; *Inhalt* dated June 1907), September 1907 (*Nat Nov*), November 1907 (*Ent Litt*)].

*Fünfundvierzigstes Heft.* iv + [412] pp. [DP: 1908 (title page; *Inhalt* dated March 1908), April 1908 (*Nat Nov*), May 1908 (*Ent Litt*)].

*Sechsundvierzigstes Heft.* viii + [465] pp. [DP: 1910 (title page; *Vorwort* dated March 1910), March 1910 (*Nat Nov*)].

*Siebenundvierzigstes Heft.* iv + [432] pp. [DP: 1911 (title page; *Inhalt* dated March 1911), April 1911 (*Nat Nov*), May 1911 (*Ent Litt*)].

*Achtundvierzigstes Heft*. *Mit Beiträgen von W. Hubenthal*. v + [406] pp. [DP: 1912 (title page; *Vorwort* dated May 1912), July 1912 (*Nat Nov*), August 1912 (*Ent Litt*)].

The first 28 *Hefte* of the series were authored by Küster, 1844–1854 [*q.v.*] and the 29th by Kraatz, 1873 [*q.v.*].

#### Schinz, Heinrich Rudolf (Zürich, Switzerland: 30 March 1777 – 8 March 1861: Zürich, Switzerland). Swiss physician and naturalist; professor of natural history at the University of Zürich. References. Hunziker (1890); Mearns and Mearns (1988: 464); Watkins and Boelens (2015: 140).

**1823**. *Das Thierreich eingetheilt nach dem Bau der Thiere als Grundlage ihrer Naturgeschichte und der vergleichenden Anatomie von dem Herrn Ritter von Cuvier Staatsrath von Frankreich beständiger Secretar der Academie der Wissenschaften u.f.w. Aus dem Französischen frey übersetzt und mit vielen Zusätzen versehen. Dritter Band. Krebse, Spinnen, Insekten.* J.G. Cotta, Stuttgart und Tübingen. xviii + [1] + 932 pp. (8vo) [DP: 1823 (title page; *Vorbericht* dated August 1823), 24 March 1824 (*Allg Ztg*), January–June 1824 (*Verz Bücher Landkarten*)] GB

This volume is a German translation with additions of Cuvier’s third volume of “*Le règne animal distribué d’après son organisation*” authored by Latreille and issued in 1816 [*q.v.*]. The German translation of the four volumes of Cuvier’s work was published in four volumes, 1821–1825.

The Coleoptera are on pages 224–535.

#### Schiødte, Jörgen Matthias Christian (Copenhagen, Denmark: 20 April 1815 – 22 April 1884: Copenhagen, Denmark). Danish entomologist; professor at the National Agricultural school in Copenhagen (1840–1883); director of the entomology section at the Natural History Museum of Copenhagen from 1842; also professor at the University of Copenhagen. References. Hansen (1884); Bourgeois (1886); Henriksen (1926: 226–240, P); Smetana and Herman (2001: 140, P).

**1840–1841**. *Genera og species af Danmarks Eleutherata at tjene som fauna for denne orden og som indledning til dens anatomie og historie. Förste bind. Med fem of tyve kobbertavler.* H.C.Klein, Kjöbenhavn [= Copenhagen]. xii + 612 + [1] + xxii pp. + 25 pls. (8vo) McG, GB

This book was published in two parts. The first one (pp. 1–360) was issued in 1840 (Erichson 1841: 155–157) and the second part (pp. 361–612 + i–xii) in 1841 (Erichson 1842: 199). The title page is dated 1841 and the preface November 1841.

**1849**. *Specimen faunae subterraneae. Bidrag til den underjordiske fauna. Med fire Kobbertavler*. Bianco Luno, Kjöbenhavn [=Copenhagen]. 39 pp. + 4 pls. (4to) [DP: 1849 (title page), 15 July 1850 (*Bibl Lit Anz*)] GB

This work was also issued in *Det Kongelige Danske Videnskabernes Selskabs Skrifter* (5) 2: 1–39 + 4 pls [DP: 1851 (title page)]. Hagen (1863: 126) dated the journal version 1849 but this is the date of the “*förste bind*” of volume 5; the “*andet bind*” was issued in 1851. An English translation was published, 1851, in *The Transactions of the Entomological Society of London* (new series) 1: 134–157.

#### Schlechtendal, Dietrich Herrman Reinhard von (Halle an der Saale, Saxony-Anhalt, Germany: 28 October 1834 – 5 July 1916: Halle an der Saale, Germany). German entomologist who worked mainly on Cynipidae; assistant at the Geologisch-Mineralogischen Institut of the University of Halle for many years. References. Haupt (1916, P); Taschenberg (1918a, P).

**1879**. [Schlechtendal, D.H.R. and Wünsche, O.] *Die Insecten. Eine Anleitung zur Kenntniss derselben. Mit 15 Tafeln.* B.G. Teubner, Leipzig. xii + 267 pp. + 7 pls. (8vo) [DP: 1879 (title page; *Vorwort* dated May 1879), 26 July 1879 (*Lit Centr Deutsch*), July 1879 (*Nat Nov*), 2 August 1879 (*Jenaer Lit*), 8 September 1879 (*Zool Anz*)] GB

The entire work was issued in three continuously paginated *Abtheilungen*, all issued in 1879. The first *Abtheilung* pertains to Coleoptera and Hymenoptera. The Coleoptera are on pages 1–166.

#### Schlosser [Schlosser-Klekovski], Joseph Calasanz [Josip Krasoslav] (Heinrichswald [currently Jindřichov], Czech Republic: 25 January 1808 – 27 April 1882: Zagreb, Croatia). Moravian physician and naturalist; practiced medicine in Križevci, Croatia. References. Kanitz (1863: 129–130); Wurzbach (1875: 142–143); Vukotinović (1883).

**1877–1879**. *Fauna kornjašah trojedne kraljevine. Na sviet izdala Jugoslavenska Akademija Znanosti i Umjetnosti.* U Knjižarnici lavoslava Hartmana na prodaju, Zagrebu. lviii + 995 pp. + 1 pl. (8vo) NCSU

This work was issued in three parts by the Academy of Agram: **1**: (pp. i–lviii + 1–342) 1877 (title page; *predgovor* dated 25 December 1877); **2**: (pp. 343–726) 7 November 1878 (*Kaiser Akad Wiss*); **3**: (pp. 727–995) August 1879 (*Nat Nov*). According to Kirby (1881: 16), Schlosser’s work appears to be an adaptation or translation of Redtenbacher’s *Fauna Austriaca*. A few new species are described, including *Notiophilus melanophthalmus* (p. 12).

#### Schluga, Joannes Baptista. Slovenian naturalist and physician in Vienna.

**1766**. *Primae lineae cognitionis insectorum cum figuris aeneis.* Joannis Pauli Kraus, Vienna. 47 + [4] pp. + 2 pls. (8vo) [DP: 1766 (title page), 20 October 1766 (*Neue Hall Ztg*), 6 June 1767 (*Gött Anz*)] GB

The book was reissued in 1767 [GB].

#### Schmidt, Robert Carl Ernst (Sorau [currently Żary], Poland]: 9 May 1813 – ?).

**1841**. *Silpharum monographiae particula prima. Dissertatio quam amplissimi ordinis philosophorum auctoritate in Academia Vratislaviensi pro summis in philosophia honoribus rite obtinendis die XXXI. Martii a. MDCCCXLI h.l.q.c. Publice defendet auctor Robertus Schmidt. Soraviensis.* Novis Fritzianis, Vratislaviae [= Wrocław]. [2] + 36 pp. (8vo) [DP: 31 March 1841 (title page), 3 January 1842 (*Ent Ver Stettin*)] YB (ph)

This thesis contains a review of the species of *Silpha*; one new species is described (*Silpha alpina*, p. 31).

#### Schmidt, Wilhelm Ludwig Ewald (Nattwerder near Postdam, Brandenburg, Germany: 4 May 1805 – 5 June 1843: Stettin [currently Szczecin], Poland). Prussian physician and naturalist, interested as an entomologist mainly in Coleoptera; practicing physician and teacher at the Royal Gymnasium in Stettin. Reference. Dieckhoff (1843).

[**1839**]. *Verzeichniss europäischer Käfer.* Stettin. 58 pp. (8vo) [DP: n.d. (title page)] BYU

The author is not listed on the title page but W.L.E. Schmidt actually wrote the catalogue (Anonymous 1841a: 128; Hagen 1863: 351). There is no date on the title page; Hagen (1863: 351), Sherborn (1922a: cxix) and Horn and Schenkling (1928a: 177) listed it as published in 1839.

**1844**. *Catalogus coleopterorum Europae. Zusammengestellt auf Veranlassung des Entomologischen Vereins zu Stettin.* Stettin. 76 + [6] pp. (8vo) [DP: 1844 (title page), September 1844 (*Acad Imp Sci St-Pétersb*)] MCZ

This catalogue is usually credited to Wilhelm Ludwig Ewald Schmidt (e.g., Engelmann 1846: 508).

#### Schmidt-Göbel, Hermann Maximilian (Prague, Czech Republic: 1809 – 17 August 1882: Klosterneuburg near Vienna, Austria). Bohemian physician and naturalist; professor in Prague and later professor of zoology at the University of Lemberg in Ukraine; his Burmese Coleoptera are at the Natural History Museum in Prague. References. Anonymous (1882); Türckheim (1883).

**1836**. *Dissertatio inauguralis zoologica de Pselaphis faunae Pragensis cum anatomia Clavigeri, quam consensu et auctoritate perillustris, celeberrimi ac magnifici domini praesidis ac directoris, illustris, celeberrimi ac spectabilis domini decani nec non celeberrimorum D.D. professorum, pro doctoratus medicinae laurea summisque in medicina honoribus ac privilegiis obtinendis in alma ac antiquissima reg. carolo-ferdinandea universitate Pragena publicae eruditorum disquisitioni submittit Herrmannus Max. Schmidt, Bohemus Pragensis. In theses adnexas disputabitur in magna Aula Carolina, hora 12. matutina diei 2. Augusti, anno MDCCCXXXVI.* Theophili Haase, Pragae [= Prague]. [4] + 50 pp. + 2 pls. (8vo) [DP: 2 August 1836 (title page), 7 June 1837 (*Lit Ztg*), January–June 1837 (*Verz Bücher Landkarten*), 7 July 1837 (*Allg Bibl Deutsch*)] SME, MDZ, GB

This thesis consists of a review of the Pselaphinae of Prague; five new species are described.

**1838**. *Beytrag zu einer Monographie der Pselaphen, enthaltend neue Species aus Asien. Erste Lieferung. Mit zwei Tafeln.* Gottlieb Haase Söhne, Prag. 16 pp. + 2 pls. (8vo) [DP: 1838 (title page)] CNC

Only the first *Lieferung* was published.

**1846**. *Med. Dr. Joh. Wilh. Helfer’s hinterlassene Sammlungen aus Vorder- und Hinter-Indien. Nach seinem Tode im Auftrage des böhm. National-Museums unter Mitwirkung Mehrerer. I. Lieferung mit 3 Kupfertafeln.* Gottlieb Haase Söhne, Prag. viii + 94 pp. + 3 pls. (4to) [DP: June 1846 (title page), 4 February 1847 (*Ent Ver Stettin*), 12 May 1847 (*Lit Ztg*)] SME, GB

This is the only part published. The second page is entitled “*Faunula coleopterorum Birmaniae, adjectis nonnullis Bengaliae indigenis*” which was probably the projected title for the entire work. There is no caption on the three plates and some of the species figured are not treated in the text. However, these species are listed by names on the back of the wrapper as follows: *Libresthis* m. *truncata* m. (pl. 2, fig. 4), *Holconotus* m. *ferrugineus* m. (pl. 2, fig. 6), *Batoscelis* Dej. *polita* m. (pl. 2, fig. 8), *Agreuter* m. *melas* m. (pl. 3, fig. 2), *Eupalamus* m. *clivinoides* m. (pl. 3, fig. 4), *Indalma* m. *elegans* m. (pl. 3, fig. 5), *Phreoryctes* m. *pusillus* m. (pl. 3, fig. 6), *Loxoncus* m. *elevatus* m. (pl. 3, fig. 9). These new species, along with the new genera, are made available from the illustrations.

#### Schmiedlein, Gottfried Benedikt (Leipzig, Saxony, Germany: 1739 – 21 February 1808: Leipzig, Germany). German physician, naturalist and meteorologist in Leipzig. References. Baur (1816: 426); Richter (1863: 94).

**1784**. *Taschenbuch für Insectenfreunde, oder Grundriß eines encyclopädischen Insectencabinets, besonders der innländischen, nach dem Linneischen System, mit deutschen und lateinischen Namen, und Anführung der Werke, worinnen sie am besten abgebildet sind: angehenden Sammlern zum Nutzen entworfen.* Schwickert, Leipzig. xii + pp. 13–134. (8vo) [DP: 1784 (title page; *Vorbericht* dated “Oster-Messe 1784”), 17 July 1784 (*Leip Intell-Blatt*)] GB, MDZ

This book was published anonymously. The Coleoptera are on pages 13–30.

**1786**. *Einleitung in die nähere Kenntnis der Insectenlehre nach dem Linnéischen System, zum Gebrauch angehender Sammler. Nebst zwo Kupfertafeln.* Adam Friedrich Böhme, Leipzig. [10] + 494 pp. + 2 pls. (8vo) [DP: 1786 (title page; *Vorbericht* dated “Ostermesse 1786”), 22 February 1787 (*Neue Leip Ztg*)] GB

The Coleoptera are on pages 198–252.

#### Schneider, David Heinrich [Hinrich] (Stralsund, Mecklenburg-West Pomerania, Germany: 13 October 1755 – 26 November 1826: Stralsund, Germany). German lawyer and entomologist; member of the city council of Stralsund (1795–1808); founded the journal *Neuestes Magazin für Liebhaber der Entomologie*, published 1791–1794; what remained of his collection was sold at auction in 1828. Reference. Grewolls (1995: 389).

**1785**. *Nomenclator entomologicus oder systematisches Nahmen-Verzeichniss der bis jezt bekannt gewordenen Insekten herausgegeben von dem Verfasser der systematischen Beschreibung der europäischen Schmetterlinge.* Christian Lorenz Struck, Stralsund. [2] + 67 + [1] pp. (8vo) [DP: 1785 (title page; *Nachricht* dated “Oster-Messe. 1785”), 25 July 1785 (*Gött Anz*)] GB, MDZ

The work was published anonymously. The Coleoptera are on pages 1–25.

**1799**. *Verzeichniss einer Parthei Insekten, welche am 6ten März 1800 zu Stralsund in öffentlicher Auction einzeln verkauft werden sollen.* Stralsund. 23 pp. (8vo) [DP: 1 September 1799 (Hagen 1863: 135)] (*n.v.*).

This is a catalogue of a collection of insects to be sold individually at auction in Stralsund on 6 March 1800. Based on Sherborn’s *Index Animalium* (online version), the following new species of beetles were described in this catalogue: *Passalus assimilis* (p. 3), *Elater trinotatus* (p. 7), *Buprestis echinata* (p. 8), *Cryptocephalus multiguttatus* (p. 9) and *Hispa dorsalis* (p. 11). However, these names are not available since the work was placed on the *Official Index of Rejected and Invalid Works in Zoological Nomenclature* in Opinion 1820 (ICZN 1995).

#### Schneider, Oscar (Löbau, Saxony, Germany: 18 April 1841 – 8 September 1903: Dresden, Saxony, Germany). German naturalist; professor at the Freimaurer-Institut and later at Anne’s secondary school in Dresden; travelled to Egypt, Palestine, Austria, Tyrol, northern Italy and Corsica; part of his European Coleoptera are in the Staatliches Museum für Tierkunde in Dresden and the Museum für Naturkunde Görlitz, the remaining was scattered. References. Anonymous (1903a, P); Heller (1903, P).

**1878**. [Schneider, O. and Leder, H.] *Beiträge zur Kenntniss der kaukasischen Käferfauna.* Buckart, Brünn. 359 + [1 (Berichtigungen)] pp. + 6 pls. (8vo) [DP: 1878 (title page; preliminaries dated November 1877), 25 January 1879 (*Gesell Erdk Berl*), 15 February 1879 [JD] (*Soc Imp Nat Moscou*), February 1879 (*Nat Nov*)] GB

This work was also issued in *Verhandlungen des Naturforschenden Vereines in Brünn* 16 [1877]: 3–258 [DP: 1878 (title page), 15 April–15 May 1879 (*Leopoldina* 15: 120), July 1879 (*Nat Nov*)] and 17 [1878]: 3–104 [DP: 1879 (title page), November 1879 (*Nat Nov*)].

Several new species are described in this publication and credited to Jules Putzeys, Eduard Eppelsheim, Félicien de Saulcy, Edmund Reitter, Julius Weise, Ernst von Kiesenwetter, Lucas von Heyden, Ernest Allard, Theodor Kirsch, Carlo Emery, Wilhelm Stierlin and Henri Tournier. It also includes a contribution by Julius Weise entitled “Beitrag zur Kenntniss der Gattung *Laena* Latreille” [*q.v.*] on pages 227–241.

#### Schoch, Gustav (Zürich, Switzerland: 11 September 1833 – 27 February 1899: Zürich, Switzerland). Swiss physician and entomologist; practiced medicine in Fehraltorf, Meilen and Zürich (1861–1879); natural history teacher at a gymnasium in Zürich from 1879 to shortly before his death; part of his cetonid collection is at the Eidgenössische Technische Hochschule in Zürich. Reference. Ris (1899).

**1878**. *Practische Anleitung zum Bestimmen der Käfer Deutschlands und der Schweiz, nach der analytischen Methode. Mit 150 Abbildungen auf 10 Tafeln.* Julius Hoffmann, Stuttgart. 183 pp. + 10 pls. (8vo) [DP: 1878 (title page), May 1878 (*Zool Gart* 19: 160), 1 June 1878 (*Lit Centr Deutsch*), 15 June 1878 (*Ent Nachr* 4: 165), January–June 1878 (*Bibliot Hist-Nat*; *Verz Bücher Landkarten*), June 1878 (*Allg Bibl*), July 1878 (*Wien Obst Gart* 3: 296), 23 September 1878 (*Zool Anz*)] (*n.v.* except title page).

**1895**. *Die Genera und Species meiner Cetonidensammlung.* Zürcher & Furrer [vol. 1] / E. Zwingil [vol. 2], Zürich. (4to) MCZ

*I. Teil: Trib. Goliathidae, Gymnetidae, Madagassae, Schizorrhinidae.* iii + 63 + [1] pp. [DP: n.d. (title page), 1895 (Derksen and Scheiding-Göllner 1968: 62), 1 April–15 June 1895 (*Naturfors Gesell Zürich*), August 1895 (*Nat Nov*)].

*II. Teil: Tribus Cetoniadae, Diplognathidae und Cremastochilidae.* Pp. 86–147 + [1]. [DP: n.d. (title page), 1895 (Derksen and Scheiding-Göllner 1968: 62), November 1895 (*Nat Nov*), 25 December 1895 (*Wien Ent Ztg* 14: 302)].

**1895**. *Nachtrag zu den Gattungen und Arten meiner Cetoniden-Sammlung I. Teil.* E. Zwingil, Zürich. Pp. 68–82. (4to) [DP: August 1895 (title page; *Nat Nov*)] MCZ, GB

**1896**. *Lamellicornia melitophila. Catalogus systematicus cetonidarum et trichiidarum ad huc cognitarum.* Zürich. 95 pp. (8vo) [DP: 1896 (title page), July 1896 (*Nat Nov*), 10 August 1896 (*Zool Anz*), August 1896 (*Ent Nachr* 22: 256), 31 October 1896 (*Wien Ent Ztg* 15: 284)] MCZ, GB

#### Schönherr, Carl Johann (Stockholm, Sweden: 10 June 1772 – 28 March 1848: Sparresäter near Skara, Sweden). Swedish entomologist; sole manager of the silk manufactory established by his father (1791–1811); then, retired to his estate in Sparresäter; his collection was bequeathed to the Naturhistoriska Riksmuseet in Stockholm. References. Carlson (1849); Dohrn (1849: 193–199); Mannerheim (1849); Zimmerman (1993: 743–753, P); Meeningen (1998).

**1806–1817**. *Synonymia insectorum, oder: Versuch einer Synonymie aller bisher bekannten Insecten; nach Fabricii Systema Eleutheratorum geordnet. Mit Berichtigungen und Anmerkungen, wie auch Beschreibungen neuer Arten und illuminirten Kupfern. Erster Band. Eleutherata oder Käfer.* Heinr. A. Nordström, Stockholm [vol. 1] / Carl Friedr. Marquard, Stockholm [vol. 2] / Lewerentz, Skara [vol. 3]. (8vo) CNC, GB

*Erster Theil. Lethrus – Scolytes.* xxii + 293 pp. + 3 pls. [DP: 1806 (title page; *Einleitung* dated March 1806), 20 March 1807 (*Allg Lit Ztg*), January–June 1807 (*Verz Bücher Landkarten*)].

*Zweiter Theil. Spercheus – Cryptocephalus.* ix + [1] + 424 pp. + 1 pl. [DP: 1808 (title page; *Vorerinnerung* dated August 1808)]. In the title of this volume, “*&c.*” has been added between “*Eleutheratorum*” and “*geordnet*.”

*Dritter Theil. Hispa – Molorchus.* xi + 506 pp. [DP: 1817 (title page; *Vorerinnerung* dated March 1817)]. The title of this volume is “*Synonymia insectorum, oder Versuch einer Synonymie aller bisher bekannten Insecten; nach Fabricii Systema Eleutheratorum etc. geordnet. Mit Berichtigungen und Anmerkungen, wie auch mit Beschreibungen neuer Arten und mit illuminirten Kupfern.*” Hagen (1863: 136) mentioned another printing for this volume by Bruzelius, Uppsala, the same year (*n.v.*) with the title reversed.

**1817**. *Appendix ad C.J. Schönherr Synonymiam Insectorum Tom. I. Part. 3. sistens descriptiones novarum specierum.* Lewerentz, Scaris [= Skara]. 266 pp. + 2 pls [numbered 5 and 6]. (8vo) [DP: 1817 (title page)] CNC, BHL

The half-title page reads “*Appendix. Descriptiones novarum specierum insectorum*.” The work contains the descriptions of many new species of beetles (pp. 3–198) and an alphabetic index to the species included in the third part of his *Synonymia insectorum* issued in 1817 (pp. 199–262).

**1826**. *Curculionidum dispositio methodica cum generum characteribus, descriptionibus atque observationibus variis seu prodromus ad Synonymiae Insectorum. Partem IV.* Fridericum Fleischer, Lipsiae [= Leipzig]. x + [1] + 338 pp. (8vo) [DP: 1826 (title page, *lectori* dated 7 October 1825), January–June 1826 (*Verz Bücher Landkarten*), 27 December 1826 (*Bibl Deutsch*)] CNC, GB

**1833–1845**. *Genera et species curculionidum, cum synonymia hujus familiae; species novae aut hactenus minus cognitae, descriptionibus A Dom. Leonardo Gyllenhal, C. H. Boheman, et entomologis aliis illustratae.* Roret, Parisiis. (8vo) CNC, GB

*Tomus primus. Pars prima.* xv + 381 pp. [DP: 1833 (title page, *lectori* dated 1 May 1833), June 1833 (Zimmerman 1993: 756), 6 July 1833 (*Bibl Fr*), 8 August 1833 (*Soc Ent Fr*)]. This volume has another title page which reads “*Synonymia insectorum, oder Versuch einer Synonymie aller von mir bisher bekannten Insecten. Mit Berichtigungen und Anmerkungen, wie auch mit Beschreibungen neuer Arten. Erster Band. Eleutherata oder Kaefer. Vierter Theil. Fam. Curculionides.*”

*Tomus primus. Pars secunda.* Pp. 385–681. [DP: 1833 (title page), 28 September 1833 (*Bibl Fr*), 2 October 1833 (*Soc Ent Fr*)].

*Tomus secundus. Pars prima.* 326 + [1] pp. [DP: 1834 (title page), April 1834 (*Isis*, Heft IV: [2]), 7 May 1834 (*Soc Ent Fr*), 14 June 1834 (*Bibl Deutsch*), January–June 1834 (*Verz Bücher Landkarten*), July 1834 (*Ent Mag*), 13 August 1834 (*Lit Ztg*)]. This volume has another title page which reads “*Synonymia insectorum, oder Versuch einer Synonymie aller von mir bisher bekannten Insecten. Mit Berichtigungen und Anmerkungen, wie auch mit Beschreibungen neuer Arten. Zweyter Band. Eleutherata oder Kaefer. Vierter Theil zweite Abtheilung. Fam. Curculionides.*”

*Tomus secundus. Pars secunda.* Pp. 329–669 + [3 (Corrigenda ad genera et species Curculionidum. Tomus primus)]. [DP: 1834 (title page), 13 September 1834 (*Bibl Fr*), 1 October 1834 (*Soc Ent Fr*), 1 November 1834 (*Bibl Deutsch*), 3 November 1834 (*Ent Soc Lond*), July–December 1834 (*Verz Bücher Landkarten*)].

*Tomus tertius. Pars prima.* [3] + 505 pp. [DP: 1836 (title page), 2 December 1835 (*Soc Ent Fr*), 5 December 1835 (*Bibl Fr*), 23 December 1835 (*Lit Ztg*), 4 January 1836 (*Ent Soc Lond*)].

*Tomus tertius. Pars secunda.* Pp. 507–858 + [3 (Corrigenda ad genera et species Curculionidum. Tomus secundus)]. [DP: 1836 (title page), 16 March 1836 (*Soc Ent Fr*), 19 March 1836 (*Bibl Fr*), 30 March 1836 (*Lit Ztg*)].

*Tomus quartus. Pars prima.* [3] + 600 pp. [DP: 1837 (title page; *lectori* dated 20 September 1836), 11 November 1837 (*Bibl Fr*), 15 November 1837 (*Soc Ent Fr*), 22 November 1837 (*Lit Ztg*), 4 December 1837 (*Ent Soc Lond*)].

*Tomus quartus. Pars secunda.* Pp. 601–1121 + [3 (Corrigenda ad genera et species Curculionidum. Tomus tertius)]. [DP: 1838 (title page), 6 January 1838 (*Bibl Fr*), 17 January 1838 (*Lit Ztg*), 5 February 1838 (*Ent Soc Lond*), February 1838 (*Isis*, Heft II: [2])]. This part was probably issued in 1837 as it was included in Erichson’s review of the entomological literature for 1837 (see Erichson 1838: 231).

*Tomus quintus. Pars prima. Supplementum continens.* viii + 456 pp. [DP: 1839 (title page; *lectori* dated 1 February 1838), 15 June 1839 (*Bibl Fr*), 28 June 1839 (*Allg Bibl Deutsch*), 3 July 1839 (*Lit Ztg*), 15 July 1839 (*Rev Bibl-Par*), 7 August 1839 (*Soc Ent Fr*)]. For tomes 5 and 6 (pars 1), the title is: *Genera et species curculionidum, cum synonymia hujus familiae; species novae aut hactenus minus cognitae, descriptionibus Dom. L. Gyllenhal, C. H. Boheman, O.J. Fahraeus et entomologis aliis illustratae*.

*Tomus quintus. Pars secunda. Supplementum continens.* viii + pp. 465–970 + [4 (Corrigenda ad genera et species Curculionidum. Tomus quartus)]. [DP: 1840 (title page; *lectori* dated 15 March 1839), 18 March 1840 (*Soc Ent Fr*), 21 March 1840 (*Bibl Fr*), 8 April 1840 (*Lit Ztg*), 30 June 1840 (*Intell Serapeum*)].

*Tomus sextus. Pars prima. Supplementum continens.* [2] + 474 pp. [DP: 1840 (title page), 5 August 1840 (*Soc Ent Fr*), 8 August 1840 (*Bibl Fr*), 26 August 1840 (*Lit Ztg*), 15 September 1840 (*Intell Serapeum*), 8 December 1840 (*Ent Ver Stettin*)].

*Tomus sextus. Pars secunda. Supplementum continens.* [2] + 495 pp. [DP: 1842 (title page; *lectori* dated 12 September 1840), 7 September 1842 (*Soc Ent Fr*), 24 September 1842 (*Bibl Fr*), 4 October 1842 (*Ent Ver Stettin*), 5 October 1842 (*Lit Ztg*), 15 October 1842 (*Monit Lib*), 17 December 1842 [JD] (*Soc Imp Nat Moscou*)]. For this tome and the followings, the title is: *Genera et species curculionidum, cum synonymia hujus familiae; species novae aut hactenus minus cognitae, descriptionibus A Dom. L. Gyllenhal, C. H. Boheman, O.J. Fahraeus et entomologis aliis illustratae*.

*Tomus septimus. Pars prima. Supplementum continens.* [2] + 479 pp. [DP: 1843 (title page; *lectori* dated 1841), 7 December 1842 (*Soc Ent Fr*), 5 January 1843 (*Allg Bibl Deutsch*), 14 January 1843 (*Bibl Fr*), 17 February 1843 (*Leip Reper*)].

*Tomus septimus. Pars secunda. Supplementum continens.* [3] + 461 pp. [DP: 1843 (title page), 15 November 1843 (*Soc Ent Fr*), 2 December 1843 (*Bibl Fr*), 10 December 1843 (*Monit Lib*), 13 December 1843 (*Lit Ztg*), 21 December 1843 (*Allg Bibl Deutsch*), 22 December 1843 (*Leip Reper*)].

*Tomus octavus. Pars prima. Supplementum continens.* [1] + 442 pp. [DP: 1844 (title page), 17 April 1844 (*Soc Ent Fr*), 29 June 1844 (*Bibl Fr*), June 1844 (*Isis*, Hefte V–VI: [2]), January–June 1844 (*Verz Bücher Landkarten*), 2 July 1844 (*Ent Ver Stettin*), 4 July 1844 (*Allg Bibl Deutsch*), 10 July 1844 (*Monit Lib*; *Lit Ztg*), 15 July 1844 (*Intell Serapeum*), 19 October 1844 [JD] (*Soc Imp Nat Moscou*)].

*Tomus octavus. Pars secunda. Supplementum continens.* [4] + 504 pp. [DP: 1845 (title page; *lectori* dated 12 April 1844), 12 March 1845 (*Soc Ent Fr*), 30 April 1845 (*Lit Ztg*; *Intell Serapeum*), 13 May 1845 (*Ent Ver Stettin*), 16 May 1845 (*Leip Reper*), 17 May 1845 (*Bibl Fr*), May 1845 (*Isis*, Heft V: [2]), 20 September 1845 [JD] (*Soc Imp Nat Moscou*)].

The half-title page reads “*Synonymia insectorum. Genera et species Curculionidum*.”

A catalogue of this work was published in Paris by Jekel, 1849 [*q.v.*]. Volumes 3 (part 1), 7 (part 1) and very likely also 4 (part 2) were issued in the year prior to that listed on the title pages.

**1847**. *Mantissa secunda familiae Curculionidum seu descriptiones novorum quorundam generum Curculionidum.* Norstedt et Filii, Holmiae [= Stockholm]. 86 pp. (8vo) [DP: 1847 (title page), 8 December 1847 (*Lit Ztg*), 13 January 1848 (*Ent Ver Stettin*), 14 January 1848 (*Leip Reper*), 29 February 1848 (*Blätt Lit Unterhalt*; *Neue Jena Lit Ztg*), 22 March 1848 (*Soc Ent Fr*)] BHL, GB

This work was also published, under the same title, in *Kongl. Vetenskaps-Akademiens Handlingar, för år 1846*: 51–136 [DP: 1848 (title page)].

#### Schrank, Franz von Paula (Varnbach near Scharding, Austria: 21 August 1747 – 23 December 1835: Munich, Bavaria, Germany). German naturalist, ex Jesuit, and priest; professor of mathematics and physics at the lyceum in Amberg (Bavaria) before teaching botany and rural economy at the University of Ingolstadt in Bavaria and later at Landshut; director of the botanical garden in Munich. References. Döring (1837); Wunschmann (1891a); Stein (1912); Zimmermann (1981); Damkaer (2002: 98–99); Adler (2012: 45–46, P).

**1776**. *Beyträge zur Naturgeschichte. Mit sieben von dem Verfasser selbst gezeichneten, und in Kupfer gestochenen Tabellen.* Gebrüdern Veith, Augsburg. [6] + 137 + [3] pp. + 7 pls. (8vo) [DP: 1776 (title page), 21 April 1776 (Evenhuis 1997b: 706), September 1776 (*Allg Verz Bücher*), 16 December 1776 (*Jena Ztg*)] CNC, GB

Another printing was done in Leipzig, the same year, by Caspar Fritsch [BHL]. The Coleoptera are on pages 61–72; several new species are described.

**1781**. *Envmeratio insectorvm Avstriae indigenorum. Cum figuris.* Vidvam Eberhardi Klettel et Franck, Avgvstae Vindelicorvm [= Augsburg]. [22] + 548 + [2 (Errata)] pp. + 4 pls. (8vo) [DP: 1781 (title page), June 1781 (*Allg Verz Bücher*), 5 April 1782 (*Jena Ztg*)] CNC, GB, BHL

The Coleoptera are on pages [7–12] and 1–238.

**1785**. [Schrank, F.P. and Moll, K.E.R. von] *Naturhistorische Briefe über Oestreich, Salzburg, Passau und Berchtesgaden. Erster Band.* Joh. Jos. Mayer, Salzburg. [6] + 332 + [3] pp. (8vo) [DP: 1785 (title page; *Vorbericht* dated “1 Herbstmon. 1784”), 27 September 1785 (*Neue Leip Ztg*)] CAKL, GB, BHL

This volume is divided in 21 *Briefe* and the Coleoptera are treated in *Briefe* 9 and 10 (pp. 153–194). The entire work was published in two volumes, both issued in 1785.

**1786**. *Baiersche Reise. Mit Kupfern.* Johann Baptist Strobl, München. [12] + 276 + [1 (Errata)] pp. + 2 pls. (8vo) [DP: 1786 (title page; *Vorrede* dated 5 April 1786), 3 June 1786 (*Salzb Intell*), 18 January 1787 (*Gött Anz*)] GB, GDZ

Three new species of beetles are described in this work, *Carabus bacarozzo* (p. 22), *Cistela fusca* (p. 63) and *Mordella octopunctata* (p. 82).

**1795**. *Naturhistorische und ökonomische Briefe über das Donaumoor. Nebst einer Kupfertafel.* Schwan und Götz, Mannheim. [4] + 211 + [4 (Inhalt)] pp. (4to) [DP: 1795 (title page; *Vorrede* dated 5 August 1794), 7 January 1795 (*Intell Allg Lit Ztg*), 2 April 1796 (*Gött Anz*)] GDZ, GB

The work is divided in 12 *Briefe* and the Coleoptera are in *Brief* 8 on pages 136–137. Three new species of beetles are described, *Bostrichus anomalus* (p. 136), *Coccinella 13 punctata* (p. 137) and *Carabus unicolor* (p. 137).

**1796**. *Sammlung naturhistorischer und physikalischer Aufsäze. Mit Kupfern.* Raspe, Nürnberg. xvi + 456 pp. + 7 pls. (8vo) [DP: 1796 (title page; *Vorrede* dated 5 March 1796), 11 June 1796 (*Allg Lit Ztg*), 17 September 1796 (*Gött Anz*)] NCSU, GB, BHL, GDZ

The book is divided into ten sections, all but four (sections 4, 5, 9 and 10) authored by Schrank. In section 2 (pp. 97–226), entitled “*Naturhistorische Beobachtungen um Pöttmes, Neuburg und Weihering*,” the following species of Coleoptera are described: *Curculio carpini* (p. 117), *Curculio betuli* (p. 117), *Curculio prismatifer* (p. 118), *Staphylinus rhyssostomus* (p. 119), *Dermestes graphicus* (p. 160), *Staphylinus turfosus* (p. 164) and *Chrysomela sylphoides* (p. 211).

**1798**. *Favna Boica. Durchgedachte Geschichte der in Baiern einheimischen und zahmen Thiere. Erste Band.* Stein, Nürnberg. (8vo) MCZ, GB, BHL

*Erste Abtheilung.* xii + 292 pp. [DP: 1798 (title page; *Vorrede* dated 20 July 1797), 26 May 1798 (*Gött Anz*)].

*Zweyte Abtheilung.* Pp. 295–720. [DP: 1798 (title page)].

The entire work consists of three volumes (*Bande*), issued in six parts (*Abtheilungen)*, 1798–1804, on the fauna of Bavaria. The Coleoptera are in the first volume on pages 354–720.

**1802**. *Landshutische Nebenstunden zur Erweiterung der Naturgeschichte. Erstes Heft. Mit zwey Kupfertafeln.* Anton Weber, Landshut. viii + 120 pp. + 2 pls. (8vo) [DP: 1802 (title page; *Vorrede* dated 8 June 1801)] GB

This work comprises additions (pp. 99–120) to the author’s *Fauna Boica* [*q.v.*]; the Coleoptera are on pages 106–120. The entire work consists of two parts, 1802–1803.

#### Schreber, Johann Christian Daniel von (Weissensee, Thuringia, Germany: 17 January 1739 – 10 December 1810: Erlangen, Bavaria, Germany). German physician and naturalist; studied medicine, natural sciences, and theology in Halle and Uppsala under Carl von Linné; practicing physician in Bützow (Mecklenburg); later, appointed professor of medicine and botany at Erlangen; eventually also hold the position of director of the botanical garden in Erlangen. References. Fikenscher (1806: 85–96), Wunschmann (1891a); Evenhuis (1997b: 708, P); Adler (2012: 44–45, P); Stubbe and Stubbe (2014, P).

**1759**. *Novae species insectorvm. Cvm figvris aeneis coloribvs pictis.* Schneideriana, Halae Magdebvrgicae [= Halle]. 16 pp. + 1 pl. (8vo) [DP: 1759 (title page)^95^] MCZ, GB

A facsimile of this work was published by Griffin (1939b).

#### Schröter, Johann Samuel (Rastenberg, Thuringia, Germany: 25 February 1735 – 24 March 1808: Buttstädt, Thuringia, Germany). German theologian and naturalist particularly interested in conchology, mineralogy and paleontology; rector of the Stadtschule in Dornburg (1758–1763); pastor in Thangelstedt and Kettewitz, then deacon in Weimar, and finally (1775–1808) superintendent and head minister in Buttstädt. References. Hess (1891a); Friess (1982, P); Köhler et al. (2013, P).

**1776**. *Abhandlungen über verschiedene Gegenstände der Naturgeschichte. Erster Theil. Mit ausgemahlten Kupfern.* J. Just. Gebauer und J. Jac. Gebauer, Halle. [26] + 488 pp. + 3 pls. (8vo) [DP: 1776 (title page; p. [6] dated 28 February 1776), 31 May 1776 (*Jena Ztg*), 25 September 1776 (*Goth Ztg*), 20 September 1777 (*Gött Anz*)] MCZ, GB

The entire work consists of two volumes, 1776–1777. The entomology section is in the first volume. On page 333, Schröter described a *Cerambyx rubus* which he compared to *Cerambyx rubus* Linnaeus; Schröter’s species is currently considered a junior synonym of *Batocera rufomaculata* (DeGeer). Schröter’s name is listed as available in Sherborn (1902: 845).

#### Schubert, Gotthilf Heinrich von (Hohenstein-Ernstthal, Saxony, Germany: 26 April 1780 – 30 June 1860: Oberhaching, Bavaria, Germany). German physician, naturalist and philosopher; professor at the University of Erlangen and later at Munich. References. Wagner (1861); Hess (1891b); Fromm (2007); Petermann (2008, P).

**1826**. *Allgemeine Naturgeschichte oder Andeutungen zur Geschichte und Physiognomik der Natur.* J.J. Palm und Ernst Enke, Erlangen. xvi + 1296 pp. (8vo) [DP: 1826 (title page), 4 June 1826 (*Allg Ztg*), January–June 1826 (*Verz Bücher Landkarten*)] GB

The Coleoptera are on pages 773–824. A second edition of this work was published in three volumes, 1835–1837, under the title “*Die Geschichte der Natur. Als zweite, gänzlich umgearbeitete Auflage der allgemeinen Naturgeschichte*” [GB]; the Coleoptera are in the third volume on pages 278–299.

#### Schulz, Johann Heinrich (Słupsk, Poland: 24 October 1799 – 29 September 1869: Berlin, Germany). German educator and naturalist; schoolmaster in Berlin. References. Koner (1846: 325); Schalow (1919: 525–531).

**1836**. *Lehrbuch der Zoologie. Zum Gebrauche für Lehrer an höheren Schulanstalten für das männliche und weibliche Geschlecht, sowie zur Selbstbelehrung.* Wilhelm Logier, Berlin. vi + 601 + [1] pp. (8vo) [DP: 1836 (title page; *Vorrede* dated March 1836), 10 June 1836 (*Allg Bibl Deutsch*), January–June 1836 (*Verz Bücher Landkarten*), 21 September 1836 (*Lit Ztg*)] GB

The Coleoptera are on pages 454–483.

#### Schwaab, Wilhelm (Homberg, Hesse, Germany: 4 July 1810 – 15 September 1879: Cassel, Hesse, Germany). German educator; professor of natural sciences in Cassel. References. Weber (1846: 431–432); Gross (1861: 8).

**1851**. *Geographische Naturkunde von Kurhessen*. Theodor Fischer, Cassel. 136 + [1] pp. (8vo) [DP: 1851 (title page), 3 July 1851 (*Allg Bibl Deutsch*), July–December 1851 (*Bibliot Hist-Nat*)] GB, MDZ

The book includes a catalogue of the insects of Kurhessen, at the time a federal state in Germany. The Coleoptera are on pages 79–98.

#### Schwägrichen, Christian Friedrich (Leipzig, Saxony, Germany: 16 September 1775 – 2 May 1853: Leipzig, Germany). German botanist; professor of botany and natural history (1801–1852) at the University of Leipzig. References. Wunschmann (1891b); Frahm and Egger (2001: 481–482).

**1804**. *Topographiae natvralis Lipsiensis specimen III. Qvo ad avdiendam orationem qva mvnvs professoris historiae natvralis extraordinarii die XIV. novembris MDCCCIV. H.l.q.c. Avspicabitvr hvmanissime invitat Fridericvs Schwaegrichen.* Solbrig, Lipsiae [= Leipzig]. 16 pp. (4to) [DP: 14 November 1804 (title page)] GB

This work is an annotated catalogue of the Coleoptera of Leipzig.

**1804**. Appendix. Insecta quaedam nova, detecta et descripta. Pp. 359–362 *in*: *Reise auf den Glockner. Von J.A. Schultes, M.D. II. Theil. Mit zwey Kupfern und einer Karte.* J.V. Degen, Wien. 366 pp. + 2 pls. (8vo) [DP: 1804 (title page), July 1804 (*Monatl Corresp*)] GB

The cover has the title “*Reise auf den Glockner. An Kärnthens, Salzburgs und Tyrols Grenze.*”

One new species of beetle, *Malachius abbreviatus*, is described (p. 359). The appendix was reissued in Salis and Steinmüller (1807: 83–85).

#### Schwarz, Christian (Nuremberg, Bavaria, Germany: 2 January 1760 – 6 July 1835: Nuremberg, Germany). German executive public servant and entomologist. References. Priem (1875: 483); Mummenhoff (1891); Grieb (2007: 1407).

**1793**. *Nomenclator über die in den Röselschen Insekten-Belustigungen und Kleemännschen Beyträgen zur Insekten-Geschichte abgebildeten und beschriebenen Insekten und Würmer mit möglichst vollständiger Synonymie. Erste Abtheilung. Käfer.* Raspe, Nürnberg. 100 pp. (4to) [DP: 1793 (title page; *Vorerinnerung* dated February 1793)] MDZ, GB

The entire series was published in seven *Abtheilungen*, 1793–1830. Only the first one pertains to Coleoptera.

#### Scopoli, Johann [Giovanni] Anton [Antonio] (Cavalese, Trentino-Alto Adige, Italy: 13 June 1723 – 8 May 1788: Pavia, Lombardy, Italy). Italian physician, botanist and entomologist; practiced medicine in Vienna and Carniola; professor of mineralogy and metallurgy in Schemnitz, Hungary (1767–1776); professor of chemistry and botany at the University of Pavia (1776–1788); the whereabouts of his insect collection are unknown. References. Perini (1852: 505–508); Voss (1882); Damkaer (2002: 55–58, P); Speta (2004, P).

**1763**. *Entomologia Carniolica exhibens insecta Carnioliae indigena et distributa in ordines, genera, species, varietates. Methodo Linnaeana.* Ioannis Thomae Trattner, Vindobonae [= Vienna]. [30] + 418 (numbered 1–416, 419–420) + [1 (Notanda, Errata)] pp. (8vo) [DP: 1763 (title page), 23 June 1763 (Evenhuis 1994: 544; 1997b: 719), 13 October 1764 (*Gött Anz*)] CNC, GB, GDZ

The original work was published without plates. A reprint, issued in 1764 (*n.v.*), contains an additional unnumbered page “*Monitum auctoris*” mentioning the forthcoming publication of 43 plates (Evenhuis 1997b: 719). These plates were published separately. According to Higgins (1963b: 168), a photolithographed reprint, limited to 50 copies only, was published by Friedländer & Sohn, Berlin, in 1880. A facsimile was issued in 1972 by the Akademische Druck- u. Verlaganstalt in Graz (Austria), with the 43 plates and a biography of Scopoli by O. Guglia (pp. iii–xxxiii), under the title “*Entomologia Carniolica einführung Otto Guglia*” [McG]. Moll (1789) gave a complete list of the insects depicted on the plates.

**1769**. *Annvs II. Historico-natvralis. I. Iter Goriziense. II. Iter Tyrolense. III. De Cucurbita pepone observationes. IV. Lichenis islandici vires medicae.* Christ. Gottlob Hilscher, Lipsiae [= Leipzig]. 118 pp. (8vo) [DP: 1769 (title page), 10 September 1770 (*Gött Anz*)] GB

The entire work consists of five volumes, 1768–1772 (see next entry). One new beetle species is described in this book, *Scarabaeus buprestoides* (p. 8), which was not listed by Sherborn in his *Index Animalium*.

A German translation of this volume was published in 1781 under the title “*Bemerkungen aus der Naturgeschichte; zweites Jahr, enthaltend: I. Die Reise nach Görz. II. Die Reise nach Tyrol. III. Bemerkungen über den Pfebenkürbis. IV. Die Arzneikräften der isländischen Flechte. Aus dem Lateinischen übersetzt von Karl, Freyherrn von Meidinger*” in Vienna [GB].

**1772**. *Annvs V. Historico-natvralis. I. Emendationes et additamenta ad ann. I. II. III. IV. II. Tentamen mineralogicum I. De minera argenti alba. III. Tentamen mineralogicum II. De sulphure. IV. Tentamen mineralogicum III. De pseudogalena, auripigmento, aliisque. V. Observationes zoologicae.* Christ. Gottlob Hilscher, Lipsiae [= Leipzig]. 128 pp. (8vo) [DP^96^: 1772 (title page), 7 July 1772 (*Erlang Anmer Nachr*), 21 November 1772 (*Gött Anz*)] MCZ

The entire work consists of five volumes, 1768–1772 (see previous entry). The Coleoptera are on pages 75–109 of the last volume.

**1777**. *Introdvctio ad historiam natvralem sistens genera lapidvm, platarvm, et animalivm hactenvs detecta, caracteribvs essentialibvs donata, in tribvs divisa, svbinde ad leges natvrae.* Wolfgangvm Gerle, Pragae [= Prague]. [6] + 506 + [34] pp. (8vo) [DP: 1777 (title page), 13 April 1777 (Evenhuis 1997b: 720), 10 May 1777 (Rickett and Stafleu 1961: 141)] BHL, GB

The Coleoptera are on pages 438–444. This work has been placed on the *Official List of Works Approved as Available for Zoological Nomenclature* in Opinion 329 (ICZN 1955).

**1786**. *Deliciae florae et faunae Insubricae seu novae, aut minus cognitae species plantarum et animalium quas in Insubria Austriaca tam spontaneas, quam exoticas vidit, descripsit, et aeri incidi curavit. Pars I.* S. Salvatoris, Ticini [= Pavia]. ix + 85 + [1 (Errata)] pp. + 25 pls. (Folio) [DP: 1786 (title page), 26 June 1786 (Evenhuis 1997b: 721), August 1786 (*New Rev*), 21 October 1786 (*Gött Anz*)] BOT, GB

The entire work was published in three fascicles, 1786–1788. It consists of descriptions of animals and plants from the Duchy of Milan. Six new species of *Scarabaeus*, *Scarabaeus speciosissimus* (p. 48), *Scarabaeus cerealis* (p. 49), *Scarites silenus* (p. 50), *Scarabaeus fuliginosus* (p. 51), *Scarabaeus sinuatus* (p. 52), and *Scarabaeus fuscus* (p. 53), are described in the first fascicle and illustrated on plate 21.

#### Scriba, Ludwig Gottlieb (Nieder-Beerbach [currently Mühltal], Hesse, Germany: 3 June 1736 – 31 May 1804: Darmstadt, Hesse, Germany). German clergyman, theologian, and entomologist; pastor in Hessen-Darmstadt. References. Ernesti (1809: 52–53); Winter (1891); Weidner (1983: 312–314).

**1790–1793**. *Beiträge zu der Insekten-Geschichte.* Varrentrapp und Wenner, Frankfurt. (4to) MCZ, GB

*Erstes Heft. Mit 6. ausgemahlten Kupfertafeln.* [5] + 68 pp. + 6 pls. [DP: 1790 (title page; *Vorrede* dated 29 March 1790), 18 August 1790 (*Intell Allg Lit Ztg*), 31 August 1790 (*Frank Anz*), 7 November 1790 (*Allg Lit Ztg*), 28 March 1791 (*Gött Anz*)].

*Zweites Heft. Mit sechs ausgemahlten Kupfertafeln.* [1] + pp. 69–194 + pls 7–12. [DP: 1791 (title page), 2 July 1791 (*Gött Anz*)].

*Dritttes Heft. Mit sechs ausgemahlten Kupfertafeln.* [1] + pp. 195–280 + pls 13–18. [DP: 1793 (title page)].

#### Scudder, Samuel Hubbard (Boston, Massachusetts, USA: 13 April 1837 – 17 May 1911: Cambridge, Massachusetts, USA). American paleontologist and entomologist; secretary, librarian, curator, vice president and president of the Boston Society of Natural History (1862–1887); assistant librarian at Harvard University (1879–1882); paleontologist for the U.S. Geological Survey (1886–1892); described over 2000 fossil insects which are at the Museum of Comparative Zoology in Cambridge, Massachusetts. References. Kingsley (1911, P); Mayor (1924, P); Essig (1931: 758–762, P); Mallis (1971: 185–191, P).

**1882–1884**. *Nomenclator zoologicus. An alphabetical list of all generic names that have been employed by naturalists for recent and fossil animals from the earliest times to the close of the year 1879. In two parts: I. Supplemental list. II. Universal index.* Bulletin of the United States National Museum. No. 19. Smithsonian Institution, Washington DC. xix + [2] + 376 + 340 pp. (8vo) [DP: 1882–1884 (wrapper; title page dated 1882; advertisement dated 26 April 1882)] CMLE, GB

This work was published in two parts. The first one, entitled “*I.– Supplemental list of genera in zoology. List of all generic names employed in zoology and paleontology to the close of the year 1879, chiefly supplemental to those catalogued by Agassiz and Marschall, or indexed in the Zoological Record*” was recorded in August 1882 (*Amer J Sci Arts*; *Nat Nov*). The second part, “*Universal Index*,” was noticed on July 1884 (*Nat Nov*).

**1893**. *Tertiary Rhynchophorus Coleoptera of the United States*. Monographs of the United States Geological Survey volume XXI. Government Printing Office, Washington [DC]. xi + 206 pp. + 12 pls. (4to) [DP: 1893 (title page), November 1894 (*Nat Nov*)] GB

**1900**. *Adephagous and clavicorn Coleoptera from the Tertiary deposits at Florissant, Colorado with descriptions of a few other forms and a systematic list of the non-rhynchophorous Tertiary Coleoptera of North America.* Monographs of the United States Geological Survey volume XL. Government Printing Office, Washington [DC]. 148 pp. + 11 pls. (4to) [DP: 1900 (title page), 24 May 1901 (*Bibl Zool*), 9 August 1901 (*Bookseller*), September 1901 (*Nat Nov*)] BHL, GB

#### Seidlitz, Georg Carl Maria von (Tschornaja Rjetschka near Saint Petersburg, Russia: 19 June 1840 – 15 July 1917: Irschenhausen near Ebenhausen, Bavaria, Germany). German entomologist interested particularly in Coleoptera; associate professor of zoology at the University of Tartu; later, assistant prosector at the University of Königsberg; his personal collection of Coleoptera is at the Zoologische Staatssammlung München. References. Bickhardt (1917, P); Hedicke (1918).

**1865**. *Monographie der Curculioniden-Gattung Peritelus Germ. Mit einer Kupfertafel.* A.W. Schade, Berlin. ii + 82 + [2] pp. + 1 pl. (8vo) [DP: 1865 (title page; preliminaries dated 30 August 1865), 8 November 1865 (*Zool-Bot Gesell Wien*)] GB

This work was also issued in *Berliner Entomologische Zeitschrift* 9 [1865]: 271–355 + pl. 4 [DP: January 1866 (p. vi)]. A separate issue was also published on 14 March 1866 (recto of title page) under the title “*Monographie der Curculioniden-Gattung Peritelus Germ. Eine mit Genehmigung der Hochverordneten physiko-mathematischen Facultät der Kaiserlichen Universität zu Dorpat zur Erlangung der Würde eines Magisters der Zoologie verfasste und zur öffentlichen Vertheidigung bestimmte Abhandlung. Mit einer Kupfertafel*” by C. Mattiesen in Dorpat [GB].

**1868**. *Die Otiorhynchiden s.str. nach den morphologischen Verwandtschaftsverhältnissen ihres Hautscelet’s vergleichend dargestellt. Herausgegeben von dem Entomologischen Vereine in Berlin.* Fr. Fleischer, Berlin. iv + 153 pp. (8vo) [DP: 1868 (title page), 27 August 1868 (*Allg Bibl Deutsch*), August 1868 (*Allg Bibl*), July–September 1868 (*Naturwiss Gesell Isis*), 17 October 1868 [JD] (*Soc Imp Nat Moscou*), July–December 1868 (*Bibliot Hist-Nat*), 5 April 1869 (*Ent Soc Lond*)] CMLE, GB

The title on the wrapper reads “*Die Otiorhynchiden im engeren Sinne eine Gruppe aus der Familie der Rüsselkäfer nach den morphologischen Verwandtschaftsverhältnissen ihres Hautscelet’s vergleichend dargestellt. Herausgegeben von dem Entomologischen Vereine in Berlin*.”

**1872–1875**. *Fauna Baltica. Die Käfer (Coleoptera) der Ostseeprovinzen Russlands.* H. Laakmann, Dorpat. xlii + 142 [Gattungen] + 560 [Arten, Register] pp. + 1 pl. (8vo) CNC

This work was published in four *Lieferungen* (dates of publication, except for the last one, are listed in the *Vorwort*, issued in *Lieferung* 4): **1**: (pp. 1–24 [Gattungen] + 1–128 [Arten]) January 1872; **2**: (pp. i–xx + 25–48 [Gattungen] + 129–208 [Arten]) June 1872; **3**: (pp. 49–80 [Gattungen] + 209–340 [Arten]) January 1874; **4**: (pp. [2 (Vorwort)] + xxi–xlii + 81–142 [Gattungen] + 341–560 [Arten, Register]) 1875 (title page; *Vorwort* dated August 1875; approved 11 October 1875), 26 April 1876 (*Soc Ent Fr*), April 1876 (*Allg Bibl*), May 1876 (*Polybiblion*). The entire volume was reissued in 1875 as volume 5 of *Archiv für die Naturkunde Liv-, Ehst- und Kurlands. Zweite Series. Biologische Naturkunde* [DP: January–June 1876 (*Bibliot Hist-Nat*)].

**1887**. *Bestimmungs-Tabelle der Dytiscidae und Gyrinidae des europäischen Faunengebietes.* Brünn. 136 pp. (8vo) [DP: 1887 (title page), July 1887 (*Nat Nov*)] SME, GB

This work was also issued in *Verhandlungen des Naturforschenden Vereins in Brünn* 25 [1886]: 3–136 [DP: 1887 (title page), December 1887 (*Nat Nov*)]. A French translation was published in *Miscellanea Entomologica* 8 [1900]: 32–41, 53–65, 97–103, 128–137; 9 [1901]: 6–12, 161–179; 10 [1902]: 43–57, 161–167; 11 [1903]: 16–28, 40–45, 81–88, 113–124, 172–175; 12 [1904]: 3–6, 42–55.

**1887–1891**. *Fauna Baltica. Die Kaefer (Coleoptera) der deutschen Ostseeprovinzen Russlands. Zweite neu bearbeitete Auflage. Mit 1 Tafel.* Hartung, Königsberg. lvi + 192 [Gattungen] + 818 [Arten, Nachträge, Register] pp. + 1 pl. (8vo) CNC

This work was published in six *Lieferungen* (dates of publication listed in the *Vorwort*, issued in *Lieferung* 6): **1**: (pp. i–xl + 1–16 [Gattungen] + 1–96 [Arten]) August 1887, 24 September 1887 (*Lit Centr Deutsch*); **2**: (pp. 17–48 [Gattungen] + 97–224 [Arten]) Spring 1888, 26 May 1888 (*Lit Centr Deutsch*), June 1888 (*Nat Nov*); **3**: (pp. xli–xlviii + 49–80 [Gattungen] + 225–336 [Arten]) Fall 1888, October 1888 (*Nat Nov*); **4**: (pp. 81–128 [Gattungen] + 337–512 [Arten]) Spring 1889, March 1889 (*Nat Nov*), 13 April 1889 (*Lit Centr Deutsch*); **5**: (pp. 129–160 [Gattungen] + 513–608 [Arten]) Spring 1890, 23 April 1890 (*Soc Ent Fr*), April 1890 (*Nat Nov*); **6**: (pp. [6 (Vorwort zu der zweiten Auflage der Fauna Baltica und zu der Fauna Transsylvanica)] + xlix–lvi + 161–192 [Gattungen] + 609–818 [Arten, Nachträge, Register]) February 1891, March 1891 (*Nat Nov*), 2 April 1891 (*Allg Bibl Deutsch*). The title page is dated 1891 and the *Vorwort* January 1891.

**1888–1891**. *Fauna Transsylvanica. Die Kaefer (Coleoptera) Siebenbürgens*. *Mit 1 Tafel.* Hartung, Königsberg. lvi + 192 [Gattungen] + 914 [Arten, Nachträge, Register] pp. + 1 pl. (8vo) CNC

This work was published in six *Lieferungen* (dates of publication listed in the *Vorwort* of the *Fauna Baltica*, 1887–1891 [*q.v.*]): **1–2**: (pp. i–xl + 1–48 [Gattungen] + 1–240 [Arten]) Spring 1888, 26 May 1888 (*Lit Centr Deutsch*), May 1888 (*Humboldt* 7: 287), June 1888 (*Nat Nov*); **3–4**: (pp. xli–xlviii + 49–128 [Gattungen] + 241–544 [Arten]) Spring 1889, March 1889 (*Nat Nov*), 13 April 1889 (*Lit Centr Deutsch*), August 1889 (*Allg Bibl*); **5–6**: (pp. xlix–lvi + 129–192 [Gattungen] + 545–914 [Arten, Nachträge, Register]) February 1891, March 1891 (*Nat Nov*), 2 April 1891 (*Allg Bibl Deutsch*). The title page is dated 1891 and the *Vorwort* January 1891.

**1893–1898**. *Naturgeschichte der Insecten Deutschlands begonnen von Dr. W.F. Erichson, fortgesetzt von Prof. Dr. H. Schaum, Dr. G. Kraatz, H.v. Kiesenwetter, Julius Weise, Edm. Reitter und Dr. G. Seidlitz. Erste Abtheilung. Coleoptera. Fünfter Band. Erste Hälfte.* Nicolai, Berlin. xxviii + pp. 201–877. (8vo) CNC, BHL

This volume was issued in five *Lieferungen*, the first one authored by Kiesenwetter, 1877 [*q.v.*]. The dates of publication of the last four *Lieferungen* are listed in the *Vorbemerkung* (p. xxviii) of the volume as follows: **2**: (pp. 201–400) March 1893; **3**: (pp. 401–608) May 1894; **4**: (pp. 609–800) September 1896^97^; **5**: (pp. v–xxviii, 801–877) September 1898.

**1896–1920**. *Naturgeschichte der Insecten Deutschlands begonnen von Dr. W. F. Erichson fortgesetzt von Prof. Dr. H. Schaum, Dr. G. Kraatz, H. v. Kiesenwtter, Julius Weise, Edm. Reitter und Dr. G. Seidlitz. Erste Abtheilung. Coleoptera. Fünfter Band. Zweite Hälfte.* Nicolai, Berlin [*Lieferungen* 1–3] / K.F. Koehlers, Leipzig [*Lieferung* 4]. 1206 pp. (8vo) NCSU, USNM, BHL

This work was issued in four *Lieferungen*: **1**: (pp. 1–304) 1896 (wrapper; preliminaries [recto of wrapper] dated November 1896), November 1896 (*Nat Nov*); **2**: (pp. 305–680) 1898 (wrapper; preliminaries [recto of wrapper] dated May 1898), 22 June 1898 (*Soc Ent Fr*), June 1898 (*Nat Nov*), 25 November 1898 (*Wien Ent Ztg* 17: 255); **3**: (pp. 681–968) 1899 (wrapper; preliminaries [recto of wrapper] dated July 1899), August 1899 (*Nat Nov*), 16 September 1899 (*Bibl Zool*), 25 October 1899 (*Soc Ent Fr*); **4**: (pp. 969–1206) 1920 (title page; p. 1206 dated 12 November 1919), November–December 1920 (*Nat Nov*). The register (pp. 1181–1206) was written by R. Stringe.

#### Sénac, Hippolyte (Paris, France: 12 March 1830 – 23 October 1892: Ussel, Allier, France). French physician and entomologist, particularly interested in Coleoptera; practiced medicine in Vichy; his collection was dispersed after his death but his tenebrionids, which included those of Reiche, were given to the *Société Entomologique de France* who deposited them at the Muséum National d’Histoire Naturelle in Paris. References. Léveillé (1894, P); Cambefort (2006: 291–292).

**1884–1887**. *Essai monographique sur le genre* Pimelia *(Fabricius).* Jacques Lechevalier, Paris. (8vo) AMNH, GAL

*Première partie. Espèces a tarses postérieurs et intermédiaires comprimés (1re division de Solier).* xix + 106 pp. [DP: 1884 (title page), 14 January 1885 (*Soc Ent Fr*), March 1885 (*Nat Nov*), 25 July 1885 (*Bibl Fr*), July–December 1885 (*Bibliot Hist-Nat*)].

*Deuxième et dernière partie. Espèces a tarses postérieurs et intermédiaires non comprimés (2e division de Solier).* xv + 160 pp. [DP: 1887 (title page), 14 December 1887 (*Soc Ent Fr*), 17 December 1887 (*Bibl Fr*), October–December 1887 (*Bibliot Hist-Nat*), January 1888 (*Nat Nov*), 19 March 1888 (*Zool Anz*)].

#### Sériziat, Charles Victor Émile (Saint-Omer, Pas de Calais, France: 29 November 1835 – 12 January 1910: Mirecourt, Vosges, France). French army medical officer and amateur entomologist. References. Cosson (1887: xcviii–xcix); Constantin (1992: 83).

**1880**. *Histoire des coléoptères de France précédée d’une introduction a l’étude de l’entomologie par M. Ch. Naudin. A l’usage des collèges, des institutions de jeunes gens des écoles normales primaires, etc. Ouvrage adopté par le Conseil de l’Université.* Firmin-Didot et C^ie^, Paris. v + 375 pp. (12mo) [DP: 1880 (title page), March 1880 (*Nat Nov*), 14 April 1880 (*Soc Ent Fr*), 24 April 1880 (*Bibl Fr*), April 1880 (*Polybiblion*), May 1880 (*Allg Bibl*), 1 June 1880 (*Naturaliste*), January–June 1880 (*Bibliot Hist-Nat*)] GB

A second edition, essentially identical to the first, was published in 1883 [GB].

#### Serres, Pierre Toussaint Marcel de (Montpellier, Hérault, France: 3 November 1780 – 22 July 1862: Montpellier, France). French geologist and naturalist; professor of mineralogy and geology at Montpellier University from 1809. References. Rumelin (1854: 128–131); Rouville (1863).

**1822**. *Essai pour servir a l’histoire des animaux du Midi de la France.* Gabon, Paris [&] Montpellier. 95 + [4] pp. (4to) [DP: 1822 (title page), 23 November 1822 (*Bibl Fr*), November 1822 (*Rev Bibl-Brus*)] GB

This work includes a list of all the animals in the South of France. The Coleoptera are on pages 66–70; a number of new species are named but not described.

**1824**. Règne animal. Des diverses espèces d’animaux que l’on observe dans le département de l’Hérault, et particulièrement des espèces qui peuvent servir à caractériser la bande isotherme de 15 à 20^o^, dans laquelle se trouve compris ce département. Pp. 89–179 *in*: *Statistique du département de l’Hérault; par M.^r^ Hippolyte Creuzé de Lesser.* Montpellier. [4] + 606 pp. (4to) [DP: 1824 (title page), 18 September 1824 (*Bibl Fr*), October 1824 (*J Sav*)] GB

De Serres’ section includes a list of the Coleoptera of the Hérault department (pp. 150–154).

**1829**. *Géognosie des terrains tertiaires, ou tableau des principaux animaux invertébrés des terrains marins tertiaires, du midi de la France.* Pomathio-Durville, Montpellier [&] Paris. xcii + 276 + [1] pp. + 6 pls. (8vo) [DP: 1829 (title page), 4 July 1829 (*Bibl Fr*), October 1829 (*J Sav*), July–October 1829 (*Foreign Quart Rev*)] GB

One new species of beetle, *Brachyurus undatus* (p. 272), is made available by the inclusion of a drawing (pl. V, fig. 8).

#### Sharp, David (Towcester, Northamptonshire, United Kingdom: 15 October 1840 – 27 August 1922: Brockenhurst, Hampshire, United Kingdom). British physician and entomologist particularly interested in Coleoptera; practicing physician in Dumfriesshire (1867–1883); curator at the University Museum in Cambridge (1890–1909); editor of the entomology section of the *Zoological Record* for several years; his collections are in the Natural History Museum in London. References. Calvert (1922); Walker (1922, P); Zimmerman (1993: 763–766, P); Smetana and Herman (2001: 142–143, P); Clark (2004c); Cambefort (2006: 292–293).

**1871**. *Catalogue of British Coleoptera.* E.W. Janson, London. 37 pp. (4to) [DP: 1 July 1871 (title page), 3 July 1871 (*Ent Soc Lond*), 15 July 1871 (*Pet Nouv Ent*), 14 October 1871 (*Soc Ent Belg*)] AMNH

A second edition of this work was published in 1883 and recorded on February 1883 (*Nat Nov*).

**1882–1887**. *Biologia Centrali-Americana. Insecta. Coleoptera. Vol. I. Part 2.* Taylor and Francis, London. xv + [1(Errata)] + 824 pp. + 19 pls. (4to) CNC

This work was published in several parts, 1882–1887. The dates indicated at the bottom of the first page of each sheet are as follows: (pp. 1–48 + pl. 1) April 1882; (pp. 49–80) October 1882; (pp. 81–144 + pls 2-4) December 1882; (pp. 145–192) May 1883; (pp. 193–240 + pls 5, 6) July 1883; (pp. 241–288) September 1883; (pp. 289–296) November 1883; (pp. 297–312 + pl. 7) December 1883; (pp. 313–344) February 1884; (pp. 345–376 + pls 8, 9) April 1884; (pp. 377–392) June 1884; (pp. 393–416 + pl. 10) January 1885; (pp. 417–448 + pl. 11) March 1885; (pp. 449–456) April 1885; (pp. 457–472 + pl. 12) May 1885; (pp. 473–504 + pl. 13) July 1885; (pp. 505–512) August 1885; (pp. 513–536) October 1885; (pp. 537–552) June 1886; (pp. 553–576 + pl. 14) July 1886; (pp. 577–608) August 1886; (pp. 609–632 + pl. 15) October 1886; (pp. 633–648 + pl. 16) November 1886; (pp. 649–672) December 1886; (pp. 673–704 + pl. 17) January 1887; (pp. 705–736 + pl. 18) February 1887; (pp. 737–744 + pl. 19) March 1887; (pp. 745–768) September 1887; (pp. 769–800) October 1887; (pp. 801–824 + i–xv + [1]) November 1887. The collation for the plates is from Lyal (2011: 80). This volume covers the families Haliplidae (pp. 1–2), Dytiscidae (pp. 3–48), Gyrinidae (pp. 48–52), Hydrophilidae (pp. 53–116), Heteroceridae (pp. 116–119), Parnidae (pp. 119–140), Georissidae (p. 141), Cyathoceridae (p. 141–144), and Staphylinidae (pp. 145–744); there is also a supplement (pp. 748–802).

**1885**. Tribe Bruchides. Pp. 437–504 + pl. 26 *in*: *Biologia Centrali-Americana. Insecta. Coleoptera. Vol. V. Longicornia by Henry Walter Bates, F.R.S. Bruchides by David Sharp, M.B.* Taylor and Francis, London. xii + 525 pp. + 26 pls. (4to) CNC

The dates listed at the bottom of the first page of each sheet for Sharp’s contribution are: (pp. 437–440) October 1885; (pp. 441–464 + pl. 26) November 1885; (pp. 465–504) December 1885.

**1887**. Fam. Pselaphidae [pp. 1–46]; Fam. Scydmaenidae [pp. 46–71]. Pp. 1–71 + pls 1–2 *in*: *Biologia Centrali-Americana. Insecta. Coleoptera. Vol. II. Part 1.* Taylor and Francis, London. xii + 717 pp. + 19 pls. (4to) CNC

This work was published in five parts during 1887. The dates listed at the bottom of the first page of each sheet are as follows: (pp. 1–16 + pl. 1) February 1887; (pp. 17–40) March 1887; (pp. 41–56 + pl. 2) April 1887; (pp. 57–64) May 1887; (pp. 65–71) October 1887. The collation for the plates is from Lyal (2011: 81).

**1888–1905**. Fam. Phalacridae [pp. 244–264]; Fam. Nitidulidae [pp. 265–388]; Fam. Trogositidae [pp. 388–437]; Fam. Synteliidae [pp. 438–440]; Fam. Adimeridae [441–443]; Fam. Colydiidae [443–498]; Fam. Rhysodidae [pp. 498–499]; Fam. Cucujidae [pp. 499–563]; Fam. Monotomidae [pp. 563–579]; Fam. Cryptophagidae [pp. 579–626]; Fam. Lathridiidae [pp. 626–638]; Fam. Mycetophagidae [638–642]; Fam. Dermestidae [642–669]; Fam. Byrrhidae [670–690]; The Central-American Rhipidandri [pp. 690–692]. Pp. 244–692 + pls 7–19 *in*: *Biologia Centrali-Americana. Insecta. Coleoptera. Vol. II. Part 1.* Taylor and Francis, London. xii + 717 pp. + 19 pls. (4to) CNC

This work was published in several parts, 1888–1905. The dates indicated at the bottom of the first page of each sheet are as follows: (pp. 244–256) December 1888; (pp. 257–264) February 1889; (pl. 7) March 1889; (pp. 265–280 + pl. 8) August 1889; (pp. 281–304 + pl. 9) October 1889; (pp. 305–312) February 1890; (pp. 313–336) March 1890; (pl. 10) June 1890; (pp. 337–368) February 1891; (pp. 369–384) April 1891; (pl. 11) July 1891; (pp. 385–432) September 1891; (pp. 433–440 + pl. 12) October 1891; (pl. 13) April 1892; (pp. 441–464 + pl. 14) October 1894; (pp. 465–488) November 1894; (pp. 489–496) January 1895; (pl. 15) December 1896; (pp. 497–520 + pl. 16) June 1899; (pp. 521–552) August 1899; (pp. 553–560 + pl. 17) September 1899; (pp. 561–584) February 1900; (pp. 585–608 + pl. 18) March 1900; (pp. 609–624) April 1900; (pp. 625–632) January 1902; (pp. 633–672 + pl. 19) March 1902; (pp. 673–688) May 1902; (pp. 689–692) March 1905. The collation for the plates is from Lyal (2011: 81).

**1889–1911**. Fam. Curculionidae. Subfam. Attelabinae [pp. 1–43]; Subfam. Pterocolinae [pp. 43–45]; Subfam. Allocoryninae [pp. 45–46]; Subfam. Apionina [pp. 47–86]; Subfam. Thecesterninae [pp. 86–87]; Subfam. Otiorhynchina [p. 87]; Series Otiorhynchinae apterae [pp. 87–178]. Pp. 1–178 + pls 1–7 *in*: *Biologia Centrali-Americana. Insecta. Coleoptera. Vol. IV. Part 3. Rhynchophora. Curculionidae. Attelabinae, Pterocolinae, Allocoryninae, Apioninae, Thecesterninae, Otiorhynchinae by David Sharp and G.C. Champion.* Taylor and Francis, London. vi + 354 pp. + 15 pls. (4to) CNC

This work was published in several parts, 1888–1905. The dates listed at the bottom of the first page of each sheet are: (pp. 1–24 + pl. 1) April 1889; (pp. 25–40) May 1889; (pl. 2) October 1880; (pp. 41–56) November 1890; (pp. 57–80 + pl. 3) December 1890; (pp. 81–88) February 1891; (pp. 89–136 + pls 4, 5) October 1891; (pp. 137–168) November 1891; (pl. 6) December 1891; (pp. 169–178 + pl. 7) May 1911. The collation for the plates is from Lyal (2011: 83). The remainder of the volume, authored by George Charles Champion and containing the Otiorhynchinae alatae and the supplement to the Thecesterninae and Otiorhynchinae, was issued from May to December 1911.

**1890**. Haliplidae, Dytiscidae, Gyrinidae, Hydroptilidae, Staphylinidae, and Scarabaeidae (except Cetoniini). Pp. 37–53 *in*: *Scientific results of the second Yarkand mission; based upon the collections and notes of the late Ferdinand Stoliczka, Ph.D. Coleoptera. Published by order of the government of India.* Office of Superintendent of Government Printing, Calcutta. 79 pp. + 2 pls. (4to) [DP: 1890 (title page; colophon dated 13 November 1890), 4 March 1891 (*Asia Soc Beng*), 10 June 1891 (*Vetens Akad*), 21 June 1891 (*Accad Lincei*), 6 August 1891 (*Nature*), 19 September 1891 [JD] (*Soc Imp Nat Moscou*)] CAKL, GB

**1892**. Coleoptera – (continued). Pp. 40–44 *in*: *Supplementary appendix to travels amongst the Great Andes of the Equator by Edward Whymper with contributions by H.W. Bates. T.G. Bonney. G.A. Boulenger. Peter Cameron. F. Day. W.L. Distant. A.E. Eaton, F.D. Godman. H.S. Gorham. Martin Jacoby. E.J. Miers. A. Sidney Ollif. O. Salvin. David Sharp. T.R.R. Stebbing. Illustrated.* John Murray, London. xxii + [1 (Addenda)] + 147 pp. + 14 pls. (4to) [DP: 1891 (title page), 2 March 1892 (Sharp 1892: 61), 18 March 1892 (*Science*), 26 March 1892 (*Athenaeum*), 9 April 1892 (*Publ Circ*), April 1892 (*Nat Nov*), 16 June 1892 (*Nature*), 13 July 1892 (*Soc Ent Fr*)] CNC, GB

According to Sharp (1892: 61), the printing was done in 1891 but the work was not available for purchase until 2 March 1892.

**1893**. [Sharp, D. and Fowler, W.W.] *Catalogue of British Coleoptera.* L. Reeve & Co., London. 46 pp. (8vo) [DP: 1893 (title page), July 1893 (*Entomologist* 26: 232)] McG

**1895**. Fam. Brenthidae. Pp. 1–80 + pls 1–3 *in*: *Biologia Centrali-Americana. Insecta. Coleoptera. Vol. IV. Part 6. By David Sharp; W.F.H. Blandford; and Karl Jordan.* Taylor and Francis, London. [4] + 396 pp. + 14 pls. (4to) CNC

This work was published in three parts in 1895 (see Lyal 2011: 85): (pp. 1–32 + pl. 1) May 1895; (pp. 33–48 + pl. 2) July 1895; (pp. 49–80 + pl. 3) August 1895.

**1899**. *Insects. Part II. Hymenoptera continued (Tubulifera and Aculeata), Coleoptera, Strepsiptera, Lepidoptera, Diptera, Aphaniptera, Thysanoptera, Hemiptera, Anoplura.* Macmillan & Co., London. xii + 626 pp. (8vo) [DP: 1899 (title page), 15 July 1899 (*Athenaeum*), August 1899 (*Nat Nov*), 15 September 1899 (*Ent Record* 11: 249), September 1899 (*Entomologist* 32: 226; *Ent Monthly Mag* 35: 214), 16 November 1899 (*Nature*)] BHL

This work forms volume 6 of the ten-volume series “*The Cambridge natural history*” edited by S.F. Harmer and A.E. Shipley, 1895–1909. Volume 6 was reprinted in 1901 [BHL] and the entire series in 1959 and 1968. Evenhuis (1997a: 341) mentioned that a Russian translation of the insect portion in volumes 5 and 6 was published in Saint Petersburg in 1910 (*n.v.*).

**1899**. On the insects from New Britain. Pp. 381–394 + pl. 35 *in*: *Zoological results based on material from New Britain, New Guinea, Loyalty Islands and elsewhere, collected during the years 1895, 1896 and 1897, by Arthur Willey. Part IV. (May, 1900).* University Press, Cambridge. viii + pp. 357–530 + [2] + pls 35–53. (4to) [DP: May 1900 (title page), September 1899 (Sharp 1901: 69)] URIL, BHL

The “Zoological results” were published in six parts, 1898–1902. Volume 4 is dated May 1900 on the title page but separate copies (*n.v.*) of Sharp’s contribution were distributed in September 1899 (Sharp 1901: 69).

**1900**. Coleoptera. I. Coleoptera phytophaga. Pp. 91–116 + pl. 6 *in*: *Fauna Hawaiiensis or the zoology of the Sandwich (Hawaiian) isles: being results of the explorations instituted by the joint committee appointed by the Royal Society of London for promoting natural knowledge and the British Association for the advancement of science and carried on with the assistance of those bodies and of the Trustees of the Bernice Pauahi Bishop Museum at Honolulu. Edited by David Sharp. Volume II. Part III. Coleoptera. I. by D. Sharp and R.C.L. Perkins. Pages 91–270; plates VI, VII, VIII, IX, X, uncoloured.* University Press, Cambridge. Pp. 91–270 + 5 pls. (4to) [DP: 8 February 1900 (title page), March 1900 (*Nat Nov*)] CNC

The *Fauna Hawaiiensis* was issued in 18 parts forming three volumes, 1899–1913. Four parts deal with Coleoptera. The second part, authored by Sharp, was issued in volume 3 part 3 on 9 April 1903, the third one, authored by Sharp and Hugh Scott, was published in volume 3 part 5 on 18 December 1908, and the last one, authored by R.C.L. Perkins, Hugh Scott, and Sharp, was issued in volume 3 part 6 on 17 December 1910.

#### Shaw, George (Bierton, Buckinghamshire, United Kingdom: 10 December 1751 – 22 July 1813: London, United Kingdom). British physician, deacon and naturalist; co-founder of the Linnaean Society in 1788; assistant lecturer in botany at Oxford University; later, librarian and curator at the British Museum (Natural History). References. Anonymous (1813); Chalmers (1816: 416–423); Rose (1848d: 19–20); Adler (1989: 17, P); Woodward (2004b).

**1789–1813**. *Vivarium naturae or the naturalist’s miscellany.* London. (8vo) CAKL, GDZ (partial), HATH (partial)

[Vol. 1; Fasc. I–XII]. Pls 1–37 + text [DP: (pls 1–15) August 1789–December 1789; (pls 16–37) January 1790–July 1790].

[Vol. 2; Fasc. XIII–XXIV]. Pls 38–74 + text [DP: (pls 38–52) August 1790–December 1790; (pls 53–74) January 1791–July 1791].

[Vol. 3; Fasc. XXV–XXXVI]. Pls 75–110 + text [DP: (pls 75–89) August 1791–December 1791; (pls 90–110) January 1792–July 1792].

[Vol. 4; Fasc. XXXVII–XLVIII]. Pls 111–146 + text [DP: (pls 111–125) August 1792–December 1792; (pls 126–146) January 1793–July 1793].

[Vol. 5; Fasc. XLIX–LX]. Pls 147–182 + text [DP: (pls 147–161) August 1793–December 1793; (pls 162–182) January 1794–July 1794].

[Vol. 6; Fasc. LXI–LXXII]. Pls 183–218 + text [DP: (pls 183–197) August 1794–December 1794; (pls 198–218) January 1795–July 1795].

[Vol. 7; Fasc. LXXIII–LXXXIV]. Pls 219–254 + text [DP: (pls 219–230) August 1795–December 1795; (pls 231–254) January 1796–July 1796].

[Vol. 8; Fasc. LXXXV–XCVI]. Pls 255–300 + text [DP: (pls 255–268) August 1796–December 1796; (pls 269–300) January 1797–July 1797].

[Vol. 9; Fasc. XCVII–CVIII]. Pls 301–348 + text [DP: (pls 301–316) August 1797–December 1797; (pls 317–348) January 1798–July 1798].

[Vol. 10; Fasc. CIX–CXX]. Pls 349–396 + text [DP: (pls 349–364) August 1798–December 1798; (pls 365–396) January 1799–July 1799].

[Vol. 11; Fasc. CXXI–CXXXII]. Pls 397–444 + text [DP: (pls 397–412) August 1799–December 1799; (pls 413–444) January 1800–July 1800].

[Vol. 12; Fasc. CXXXIII–CXLIV]. Pls 445–492 + text [DP: (pls 445–460) August 1800–December 1800; (pls 461–492) January 1801–July 1801].

[Vol. 13; Fasc. CXLV–CLVI]. Pls 493–540 + text [DP: (pls 493–508) August 1801–December 1801; (pls 509–540) January 1802–July 1802].

[Vol. 14; Fasc. CLVII–CLXVIII]. Pls 541–588 + text [DP: (pls 541–556) August 1802–December 1802; (pls 557–588) January 1803–July 1803].

[Vol. 15; Fasc. CLXIX–CLXXX]. Pls 589–636 + text [DP: (pls 589–604) August 1803–December 1803; (pls 605–636) January 1804–July 1804].

[Vol. 16; Fasc. CLXXXI–CXCII]. Pls 637–684 + text [DP: (pls 637–652) August 1804–December 1804; (pls 653–684) January 1805–July 1805].

[Vol. 17; Fasc. CXCIII–CCIV]. Pls 685–732 + text [DP: (pls 685–700) August 1805–December 1805; (pls 701–732) January 1806–July 1806].

[Vol. 18; Fasc. CCV–CCXVI]. Pls 733–780 + text [DP: (pls 733–748) August 1806–December 1806; (pls 749–780) January 1807–July 1807].

[Vol. 19; Fasc. CCXVII–CCXXVIII]. Pls 781–828 + text [DP: (pls 781–796) August 1807–December 1807; (pls 797–728) January 1808–July 1808].

[Vol. 20; Fasc. CCXXIX–CCXL]. Pls 829–876 + text [DP: (pls 829–844) August 1808–December 1808; (pls 845–876) January 1809–July 1809].

[Vol. 21; Fasc. CCXLI–CCLII]. Pls 877–924 + text [DP: (pls 877–892) August 1809–December 1809; (pls 893–924) January 1810–July 1810].

[Vol. 22; Fasc. CCLIII–CCLXIV]. Pls 925–972 + text [DP: (pls 925–940) August 1810–December 1810; (pls 941–972) January 1811–July 1811].

[Vol. 23; Fasc. CCLXV–CCLXXVI]. Pls 973–1020 + text [DP: (pls 973–988) August 1811–December 1811; (pls 989–1020) January 1812–July 1812].

[Vol. 24; Fasc. CCLXXVII–CCLXXXVII]. Pls 1021–1064 + text [DP: (pls 1021–1036) August 1812–December 1812; (pls 1037–1064) January 1813–July 1813].

This work was issued by monthly fascicles from August 1789 to July 1813. A total of 287 fascicles were published and bound in 24 volumes. There are 1068 plates, of which 1064 were actually published; the last four were prepared but the text not written owing to the death of Shaw (Sherborn 1895: 376).

A number of fascicles are available on GDZ and each has a title page which reads (for example no 25): *Number XXV. (To be continued Monthly, Price 1s. 6d.) of the Naturalist’s miscellany: containing accurate and elegant coloured figures of the most curious and beautiful productions of nature, with descriptions in Latin and English in the Linnaean manner. To which are added descriptions more at large, and calculated for general information. By George Shaw, M.D. F.R.S. The figures by Fred. P. Nodder, botanic painter to Her Majesty*.

Except for the first one, the volumes seen have no title pages, but have dedicatory pages in Latin and English where the volume number is indicated. The first volume has a page reading “*Vivarium naturae or the naturalist’s miscellany. Vol. I. Dedicated by permission to Her Majesty. By G. Shaw M.D. F.R.S. the figures by F.P. Nodder, botanic painter to Her Majesty. London. Printed for Nodder & C^o^. 15 Brewer Str. Golden Sq. 1790*.” followed by a page reading “*Vivarium naturae, sive rerum naturalium, variae et vividae icones, ad ipsam naturam. Depictae et descriptae*” and another one reading “*The naturalist’s miscellany: or, colored figures of natural objects; drawn and described immediately from nature.*” The collation and date of publication of each fascicle are available in Dickinson et al. (2006: 340–343).

Based on Sherborn’s *Index Animalium* (online version), three new species of beetles are described and illustrated in this work: *Ptinus fatidicus* (on pl. 104 in fascicle 34, published in May 1792), *Scarabaeus cyaneus* (on pl. 316 in fascicle 100, issued in December 1797) and *Scarabaeus loxanus* (on pl. 911 in fascicle 249, published in May 1810). A number of additional beetles are illustrated but these were previously described.

**1806**. *General zoology or systematic natural history. With plates from the first authorities and most select specimens engraved by M^r^. Heath & M^rs^. Griffith. Vol. VI.* G. Kearsley, London. (8vo) BHL

*Part I. Insecta.* xii + [2] + 239 pp. + 79 pls. [DP: 1806 (title page), 1 February 1806 (*Monthly Mag*)].

*Part II. Insecta.* viii + pp. [9]–10 + 241–509 + pls 80–137. [DP: 1806 (title page), 1 February 1806 (*Monthly Mag*)].

The entire series was published in 28 parts forming 14 volumes. Shaw authored the first eight volumes and James Francis Stephens the remaining six volumes. The insect section is in volume 6 and the Coleoptera are in the first part (pp. 19–109, pls 1–39).

#### Shipp, John William (*ca* 1872 – 15 February 1898: Oxford, Oxfordshire, United Kingdom). British entomologist; worked at the Zoological Department of the Oxford University Museum and later at Walter Rotschchild’s Museum in Tring. Reference. Anonymous (1898).

**1897**. Scarabaeidae. Pp. 448–452 *in*: *Through unknown African countries. The first expedition from Somaliland to Lake Lamu. By A. Donaldson Smith. Illustrated.* Edward Arnold, London [&] New York. xvi + 471 pp. (8vo) [DP: 1897 (title page), 13 March 1897 (*Athenaeum*; *Academy*; *Publ Circ*), March 1897 (*English Cat Books*; *Nat Nov*), 8 April 1897 (*Bookseller*)] CAKL, GB, BHL

This section is part of Appendix I (pp. 447–454) under the title “*Coleoptera. By Dr. Karl Jordan*” which includes the Scarabaeidae (pp. 448–452) by Shipp and the Cerambycidae by K. Jordan [*q.v.*]. The taxa described are: *Heliocopris donaldsoni* Shipp (p. 448); *Heliocopris coriaceus* Shipp (p. 449); *Copris convexiusculus* Shipp (p. 450); *Onthophagus smithi* Shipp (p. 450); *Aphodius smithi* Shipp (p. 451). The book was reprinted by Greenwood Press, New York, in 1969.

#### Shuckard, William Edward (Brighton, East Sussex, United Kingdom: 1802 – 10 November 1868: Kennington [currently in Greater London], United Kingdom). British bookseller and entomologist, mostly interested in Hymenoptera; librarian of the Royal Society of London (1835–1843); editor of the journal *Lloyd’s List* (1844–1861); his collection was sold at auction in London. References. Newman (1868); Woodward (1897: 166); Foote (2004d).

**1839**. *Elements of British entomology, containing a general introduction to the science, a systematic description of all the genera, and a list of all the species of British insects, with a history of their transformation, habits, economy, and distribution, with outline figures of the families and their larvae and pupae, an explanation of the technical terms, and full directions for collecting. Part I. Illustrated with fifty wood-cuts.* Hippolyte Baillière, London. 240 pp. (8vo) [DP: 1839 (title page), May 1839 (Low 1853: 328), 3 June 1839 (*Ent Soc Lond*), 8 June 1839 (*Lit Gaz*), 15 June 1839 (*Athenaeum*), 19 June 1839 (*Lit Ztg*), January–June 1839 (*Verz Bücher Landkarten*), July 1839 (*Gent Mag*)] MCZ, GB

Only the first part was published.

**1839–1840**. *The British Coleoptera delineated, consisting of figures of all the genera of British beetles, drawn in outline by W. Spry, M.E.S. Edited by W.E. Shuckard, Libr^n^. R.S.* W. Crofts, London. vii + 83 pp. + 94 pls. (8vo) CMLE (mf), BHL, GB

This work was issued in parts. The first eight parts were recorded on these dates: **1–2**: 1 June 1839 (*Athenaeum*; *Lit Gaz*), 3 June 1839 (*Ent Soc Lond*), 28 June 1839 (*Allg Bibl Deutsch*), July 1839 (*Gent Mag*); **3**: 1 July 1839 (*Ent Soc Lond*); **4**: 5 August 1839 (*Ent Soc Lond*); **5–6**: 7 October 1839 (*Ent Soc Lond*); **7**: 4 November 1839 (*Ent Soc Lond*); **8**: 2 December 1839 (*Ent Soc Lond*). The title page is dated 1840 and the preface July 1840. Considering the date of the preface, the book was certainly issued in more than eight parts. It was recorded on 15 August 1840 (*Athenaeum*). Collation for each part is desired. The book was reissued in 1861 by Henry G. Bohn, London, under the same title [CMLE, GB, BHL].

Authorship of this work is contentious; some sources cite both Shuckard and William Spry as authors, others only Shuckard.

#### Siebke, Johan Heinrich Spalckhawer (Tøyen near Christiania, Norway: 23 February 1816 – 10 May 1875: Christiana [currently Oslo], Norway). Norwegian entomologist; curator of entomology at the Zoological Museum of the University of Christiana (1849–1875); also taught natural history in various schools in Christiania for 27 years; his collection of Norwegian insects is at the Zoological Museum, University of Oslo. References. Schneider (1876); Natvig (1943: 22–25, P); Evenhuis (1997b: 735, P).

**1875**. *Enumeratio insectorum Norvegicorum fasciculus II. Catalogum coleopterorum continens. Univ.-program for første semester 1875.* A.W. Brøgger, Christiania. Pp. 62–334. (8vo) [DP: 1875 (title page)] CNC, BHL, GB

The entire series consists of five fascicles, 1874–1880, the last one authored by Jacob Sparre-Schneider. The first two are continuously paginated. A supplement to the first two fascicles was issued by Wilhelm Maribo Schøyen in *Forhandlinger i Videnskabs-Selskabet i Christiania* (Aar 1879) (3): 1–76.

#### Siegel, Moritz. Civil servant for the department of buildings (*Baudepartement*) in Laibacher, Carniola (currently Ljubljana in Slovenia).

**1866**. *Versuch einer Käfer-Fauna Krains.* Ign. v. Kleinmayr & F. Bamberg, Laibach. iv + 120 pp. (8vo) [DP: 1866 (title page), 7 March 1866 (*Börsen Deutsch Buch*), 2 April 1866 (*Allg Lit Ztg Kath*)] GB, MDZ

This publication is an annotated catalogue of the Coleoptera of Carniola, a region of southern Europe. One new family-group name of beetles, Colaphidae (p. 102), is proposed.

The work was also issued in *Mittheilungen des Musealvereins für Krain* 1: 89–209 [DP: after 1 June 1866 (date of the *Vorwort*), 24 December 1866 (*Allg Lit Ztg Kath*)].

#### Simonkai [Simkovics], Lajos [Ludwig Philipp] (Nyíregyháza, Hungary: 9 January 1851 – 2 January 1910: Budapest, Hungary). Hungarian high school teacher and botanist, born Ludwig Philipp Simkovics; taught in today Oradea, Pancevo (1880–1881), Arad (1881–1891), and Budapest (1891–1908). References. Tuzson (1910, P); Somlyay (2011, P).

**1893**. *Aradvármegye és Arad szabad királyi város természetrajzi leirása. Harmadik rész. Aradmegye és aradváros állatvilága. (Fauna comitatus et urbis Arad.).* Monographia-bizottság, Arad. vi + 134 pp. (4to) [DP: 1893 (title page)] GB

This work consists of an annotated catalogue of the fauna of Arad county in western Romania. The Coleoptera are on pages 83–107.

#### Sloane, Thomas Gibson (Melbourne, Victoria, Australia: 20 April 1858 – 20 October 1932: Young, New South Wales, Australia). Australian entomologist, specialist on the family Carabidae; make his living as a merino sheep breeder and manager of a family company property known as Moorilla Station at Young; his collection, which included part of the van der Poll’s collection, was mostly destroyed by museum pests after his death but the remnants were subsequently deposited at the Australian National Insect Collection in Canberra. References. Carter (1932, P); Zimmerman (1993: 775, P).

**1896**. Carabidae. Pp. 380–384 *in*: *Report on the work of the Horn scientific expedition to central Australia. Part II. – Zoology. Edited by Baldwin Spencer.* Dulau and Co., London [&] Melville, Mullen and Slade, Melbourne. iv + 431 pp. + 29 pls. (4to) [DP: February 1896 (title page), June 1896 (*Nat Nov*), 16 July 1896 (*Nature*)] McG

This expedition, fitted by William Austin Horn, was set in 1894 to investigate part of Australia between Oodnadatta and Macdonnell Ranges (Musgrave 1932: 155). The entire report was published in four parts. The remaining of the Coleoptera of the expedition were treated by Blackburn, 1896 [*q.v.*].

#### Smith, Frederick (London, United Kingdom: 30 December 1805 – 16 February 1879: London, United Kingdom). British engraver and entomologist, particularly interested in Hymenoptera; curator of the collections and library of the Entomological Society of London (1841–1850); subsequently, curator at the British Museum (Natural History) in London; not known to own a collection of Coleoptera. References. Anonymous (1879); Dunning (1879, P); Gilbert (2005: 53–54, P).

**1851**. *List of the coleopterous insects in the collection of the British Museum. Part I.– Cucujidae, &c.* Printed by Order of the Trustees, London. [1] + 25 pp. (8vo) [DP: 1851 (title page; preface dated 30 November 1851), 13 December 1851 (Sherborn 1934: 310)] CNC, BHL, GB

This work was Part V of the *Nomenclature of coleopterous insects in the collection of the British Museum*.

**1852**. *Nomenclature of coleopterous insects in the collection of the British Museum. Part VI. Passalidae.* Printed by Order of the Trustees, London. [1] + 23 pp. + 1 pl. (8vo) [DP: 1852 (title page; preface dated 1 September 1852), 9 October 1852 (Sherborn 1934: 310)] CNC, GB

#### Snellen van Vollenhoven, Samuel Constant (Rotterdam, Netherlands: 18 October 1816 – 22 March 1880: The Hague, Netherlands). Dutch illustrator and entomologist, financially more or less independent; curator at the Nationaal Natuurhistorisch Museum in Leiden (1854–1873); his collection was purchased by the Museum. References. May (1880); van der Wulp (1880, P); Krikken et al. (1981, P).

**1848**. *Bijdrage tot de fauna van Nederland. Naamlijst van schildvleugelige insecten.* R.F. van Arum, Haarlem. v + [1] + pp. 7–50. (4to) [DP: 1848 (title page; *Voorberigt* dated July 1848)] GB

This work consists of an annotated list of beetles of Netherlands.

**1854**. Naamlijst van Nederlandsche Schildvleugelige Insekten. Pp. 1–70 *in*: *Bouwstoffen voor eene Fauna van Nederland, onder medewerking van onderscheidene geleerden en beoefenaars der Dierkunde, bijeenverzameld door J.A. Herklots. Tweede Deel. 1ste Stuk.* E.J. Brill, Leiden. 88 pp. (8vo) [DP: November 1854 (title page)] NCSU, GB

The second volume of this work was published in three *Stuke*, the second one (pp. 89–206) in June 1856 and the third one (pp. 207–316, i–iv) in January 1858. Four new species of beetles are described on page 70: *Staphylinus pygmaeus*, *Stenus roscidus*, *Anaspis assimilis* and *Anaspis testacea*.

**1869**. [Snellen van Vollenhoven, S.C. and Sélys Longchamps, M.E. de] *Recherches sur la faune de Madagascar et de ses dépendances, d’après les découvertes de François P.L. Pollen et D.C. van Dam. Ouvrage dédié à S. M. Guillaume III, Roi des Pays-Bas. 5^me^ partie. 1^re^ livraison. Insectes.* J.K. Steenhoff, Leyde. 25 pp. + 2 pls. (4to) [DP: 1869 (title page), May 1869 (*Nederl Bibl*), 7 June 1869 (*Ent Soc Lond*), July 1869 (*Allg Bibl*)] BHL, GB

This livraison contains four sections: 1. “Liste des insectes rapportés de l’île de la Réunion, des îles Comores et de Madagascar par MM. Pollen et Van Dam” (pp. 1–6); 2. “Description des espèces nouvelles de Coléoptères, Orthoptères, Lépidoptères et Hémiptères, mentionées dans la liste précédente” (pp. 7–14) where all new species are attributed to “Voll. ”; 3. “Odonates recueillis a Madagascar, aux iles Mascareignes et Comores, déterminés et décrits par M. le B^on.^ De Sélys Longchamps” (pp. 15–20); 4. “Énumeration des odonates de Madagascar et des iles Comores et Mascareignes, par M. le B^on.^ De Selys Longchamps” (p. 21–24); finally page 25 entitled “Remarques et résumé” is dated Liége, 5 June 1867. The new species of Coleoptera described are: *Dineutes bidens*, Voll. (p. 7), *Pleophylla unicolor*, Voll. (p. 8), Anomala (Rhinoplia) bivittata, Voll. (p. 8), *Parachilia pollenii*, Voll. (p. 9), *Psiloptera mayottensis*, Voll. (p. 9), Bostrichus (Xylopertha) iracundus, Voll. (p. 10), Phymasterna ? humeralis, Voll. (p. 10).

The entire work was published in livraisons grouped in five parts: 1. *Relation de voyage*; 2. *Mammifères et oiseaux*; 3. *Reptiles*; 4. *Poissons*; 5. *Insectes, crustacés, mollusques*. The last part was issued in three livraisons, 1869–1877, which are usually bound in one volume with the title page reading “*Recherches sur la faune de Madagascar et de ses dépendances, d’après les découvertes de François P.L. Pollen et D.C. van Dam. 5^me^ partie*” and dated 1877.

#### Solier, Antoine Joseph Jean (Marseille, Bouches-du-Rhône, France: 8 February 1792 – 27 November 1851: Marseille, France). French military and entomologist; captain of engineers in the French army (1813–1838); collected in France, Algeria and the Mediterranean region; his collection was scattered after his death by Achille Deyrolle. References. Mulsant (1852, P); Smetana and Herman (2001: 145, P); Cambefort (2006: 294).

**1843**. *Essai sur les collaptérides de la tribu des Molurites.* Imprimerie Royale, Turin. 129 pp. + 4 pls. (4to) [DP: 1843 (recto of title page)] (*n.v.* except title page and recto of title page).

This work was also published in *Memorie della Reale Accademia delle Scienze di Torino* (Serie II) 6: 213–339 + pls 1–4 [DP: 1844 (title page)]. Hagen (1863: 174) dated the reprint 1842 but this could be a lapsus for 1843.

[**1849**]. Orden III. Coleopteros. Pp. 105–380, 414–508 *in*: *Historia fisica y politica de Chile segun documentos adquiridos en esta republica durante doce años de residencia en ella y publicada bajo los auspicios del supremo gobierno por Claudio Gay. Zoologia. Tomo cuarto.* Paris [&] Museo de Historia Natural de Santiago, Santiago. 511 pp. (8vo) [DP: 1849 (title page)] CNC, BHL, GB

Pages 381–413 of volume 4 were authored by Spinola, 1849 [*q.v.*]. Information about the “*Historia fisica y politica de Chile*” of Claude Gay is included under Blanchard [1851].

[**1851**]. Insectos. Coleopteros. Pp. 5–285 *in*: *Historia fisica y politica de Chile segun documentos adquiridos en esta republica durante doce años de residencia en ella y publicada bajo los auspicios del supremo gobierno por Claudio Gay. Zoologia. Tomo quinto.* Paris [&] Museo de Historia Natural de Santiago, Santiago. 564 + [3 (numbered 561–563)] pp. (8vo) [DP: 1851 (title page)] CNC, GB

The remaining pages of the volume were authored by Emile Blanchard, 1851 [*q.v.*].

#### Sowerby, James (London, United Kingdom: 21 March 1757 – 25 October 1822: London, United Kingdom). British naturalist and illustrator; primarily noted for his botanical and mineralogical illustrations. References. Simpkins (1974); Damkaer (2002: 141–143, P); Cleevely (2004, P).

**1804–1806**. *The British miscellany: or coloured figures of new, rare, or little known animal subjects; many not before ascertained to be inhabitants of the British Isles; and chiefly in the possession of the author.* London. vi + 131 pp. + 60 pls. (4to) CAKL, GB

This work was issued in twelve parts, 1804–1806. The title page is dated “MDCCCIV–VI” and the dedication 1 February 1806. The book was also issued by R. Taylor & Co. in 1806 under the same title except that “Vol. I” was added [GB, BHL]; it also includes an alphabetical index at the end (pp. 133–136). A second volume was intended but not completed. No title page for the second volume was issued and only 31 pages and 16 plates (pls 61–76) were printed [GB, MDZ]. Collation and dates of publication for the whole work are desired.

In a letter addressed to John Freeman and dated 6 March 1806, William Kirby mentioned “the Entomological part of it [Sowerby’s British Miscellany] is now done by me” (see Freeman 1852: 275).

#### Speiser, Ferenc [Ferencz, Franz] (Apatin, Serbia: 17 November 1854 – 7 January 1933: Kalocsa, Bács-Kiskun, Hungary). Hungarian Jesuit and coleopterist; teacher and later director at the gymnasium in Kalocsa where his collection was deposited. References. Hegedűs (1933); Miklós (1942: 17–18).

**1893**. *Kalocsa környékének bogárfaunája. Coleoptera regionis Colocensis*. Antonius Malatin, Coloczae [= Kalocsa]. 60 pp. (8vo) [DP: 1893 (title page), 30 November 1893 (*Wien Ent Ztg* 12: 309)] SME

This work is a catalogue of the Coleoptera of the region of Kalocsa in southern Hungary.

#### Spinola, Maximilien [Massimiliano] (marquis) (Pézenas, Hérault, France: 10 July 1780 – 12 November 1857: Tassarolo, Piedmont, Italy). Italian entomologist of independent means; his contributions were mainly in the orders Hymenoptera, Hemiptera and Coleoptera; his collections are in the Museo Regionale di Science Naturali in Turin. References. Gestro (1915, P); Generani and Scaramozzino (2000 : 231–232, P); Aguilar (2013, P).

[**1845**]. *Essai monographique sur les clérites insectes coléoptères.* Frères Ponthenier, Gênes. (8vo) CNC, GB, GAL

*Tome premier.* ix + [1] + 386 pp. [DP: 1844 (title page), April 1845 (*Bibl Ital-1*), 14 June 1845 (*Lit Ztg*), June 1845 (*Bibliot Genève*), 25 July 1845 (*Ist Ven Sci*)].

*Tome second.* 216 + [4 (Fautes d’impression et autres)] pp. + 47 pls. [DP: 1844 (title page), April 1845 (*Bibl Ital-1*), 14 June 1845 (*Lit Ztg*), June 1845 (*Bibliot Genève*), 25 July 1845 (*Ist Ven Sci*)].

This work is cited as published in 1845 by Engelmann (1846: 553) and Brunet (1865: 336). According to Brunet (1865: 336), the volumes, except for about ten copies, were destroyed by the author because he was criticized to sell his book too much [“L’édition, sauf pour une dizaine d’exemplaires, a été détruite par l’auteur à qui on reprochait de vendre son livre trop cher”]. Considering the number of libraries that hold a copy of the work as seen in WorldCat, Brunet’s statement is incorrect.

[**1849**]. IX. Cleroideos. Pp. 381–414 *in: Historia fisica y politica de Chile, segun documentos adaquiridos en esta republica durante doce años de residencia en ella y publicada bajo los auspicios del supremo gobierno por Claudio Gay. Zoologia. Tomo cuarto.* Paris [&] Museo de Historia Natural de Santiago, Chile. 511 pp. (8vo) [DP: 1849 (title page)] CNC, BHL, GB

The remaining of the Coleoptera pages of volume 4 were authored by Solier, 1849 [*q.v.*]. Information about the “*Historia fisica y politica de Chile*” of Claude Gay is included under Blanchard [1851].

#### Stark, John (Blythsmuir, Peeblesshire, Scotland, United Kingdom: 14 October 1779 – 24 December 1849: Edinburgh, Scotland, United Kingdom). Scottish naturalist and printer in Edinburgh. Reference. Anonymous (1850).

**1828**. *Elements of natural history, adapted to the present state of the science, containing the generic characters of nearly the whole animal kingdom, and descriptions of the principal species. With illustrative engravings. Vol. II. Invertebrata, &c.* Adam Black and John Stark, Edinburgh and Longman, Rees, Orme, Brown & Green, London. 515 + [1] pp. + pls 5–8. (8vo) [DP: 1828 (title page; advertisement in first volume dated 14 October 1828), 15 November 1828 (*Lit Gaz*), November 1828 (Peddie and Waddington 1914: 559), December 1828 (*Monthly Rev*)] BHL, GB

The entire work was published in two volumes, issued simultaneously. The Coleoptera are on pages 249–312 of the second volume. Another printing was issued the same year by William Blackwood, Edinburgh, and T. Cadell, London [GB (vol. 1 only)].

#### Steffahny, Gustav Emil. Polish physician in Puck.

**1842**. *Tentamen monographiae Byrrhorum, coleopterorum generis. Dissertatio inauguralis zoologico-medica quam consensu et auctoritate gratiosi medicorum ordinis in alma literarum Universitate Friderica Guilhelma ut summi in medicina et chirurgia honores rite sibi concedantur die XVIII. m. Octobris a. MDCCCXLII. H.l.q.s. Publice defendet auctor Gustavus Aemilius Steffahny Borussus Occidentalis. Opponentibus: Julio Riese, Dr. Med. Pract. Herrmanno Berendt, Med. DD. Hans Behr, Med. Cand.* Berolini [= Berlin]. [5] + 42 pp. (8vo) [DP: 18 October 1842 (title page)] CNC (mf)

This dissertation was reissued the following year, under the title “*Tentamen monographiae generis Byrrhi*” in *Zeitschrift für die Entomologie* 4: 1–42 [DP: 1843 (title page), 5 May 1843 (*Leip Reper*)].

#### Stein, Christian Gottfried Daniel (Leipzig, Saxony, Germany: 14 October 1771 – 14 June 1830: Berlin, Germany). German geographer; professor at a gymnasium in a Berlin monastery. References. Pölitz (1830); Zeune (1830); Ratzel (1893).

**1820**. *Handbuch der Naturgeschichte für die gebildeten Stände, Gymnasien und Schulen besonders in Hinsicht auf Geographie. I ^r^. Band. Zweite verbesserte und vermehrte Auflage. Mit 131 Abbildungen auf 15 Kupfertafeln*. J.C. Hinrich, Leipzig. [2] + 350 pp. (8vo) [DP: 1820 (title page; *Vorrede* in the second *Band* dated 1 November 1819), 2 April 1820 (*Beil Allg Ztg*), 29 April 1820 (*Beil Aarau Ztg*), 29 June 1820 (*Bremer Ztg*), January–June 1820 (*Verz Bücher Landkarten*), 23 December 1820 (*Gött Anz*)] GB

The entire work was published in two volumes, issued simultaneously. The Coleoptera are on pages 232–247 in the first volume. The first edition was issued in two volumes under the same title in 1812 (*n.v.*) and noticed on 15 August 1812 (*Beil Ztg Frankfurt*). A third edition, also under the same title and in two volumes, was published in 1829 (*n.v.*) and noticed on 14 February 1829 (*Bibl Deutsch*).

#### Stein, Johann Philip Emil Friedrich (Berlin, Germany: 16 May 1814 – 2 April 1882: Berlin, Germany). German entomologist; curator at the Zoologisches Museum der Humboldt-Universität in Berlin from 1852 to 1882; his collection is deposited in that museum. References. Dohrn (1882); Göllner-Scheiding (2007, P).

**1868**. *Catalogus coleopterorum Europae*. Friderici Nicolai, Berolini [= Berlin]. iv + 149 pp. (8vo) [DP: 1868 (title page), September 1868 (*Allg Bibl*), 1 October 1868 (*Allg Bibl Deutsch*), July–December 1868 (*Bibliot Hist-Nat*)] MCZ, GB, ARC

**1877**. [Stein, J.P.E.F. and Weise, J.] *Catalogi Coleopterorum Europae. Editio secunda.* Edw. Janson, Londini [= London] [&] Nicolai, Berolini [= Berlin] [&] Luc. Buquet, Parisiis. [2] + 212 pp. (8vo) [DP: 1877 (title page), 29 September 1877 (*Lit Centr Deutsch*), 1 October 1877 (*Ent Nachr* 3: 153), July–December 1877 (*Bibliot Hist-Nat*), December 1877 (*Polybiblion*)] USNM, GB

#### Steiner, Johann Friedrich Rudolph [Rudolf] (Brunswick, Lower Saxony, Germany: 1 June 1742 – 1 March 1804: Weimar, Thuringia, Germany). German court and chief architect in Weimar. References. Gräbner (1836: 225); Anonymous (2015g).

**1788**. *Entwurf der Insectenwissenschaft oder was von der Kentnis, Erzeugung, Verwandlung und Samlung der Insecten zu wissen nöthig ist. Nebst einer Classen-Ordnung der Conchylien und ihrer Behandlung.* Christian Gottlob Hilscher, Leipzig. 126 pp. (8vo) [DP: 1788 (title page), 4 October 1788 (*Allg Lit Ztg*)] GB

The Coleoptera are on pages 35–41. The book was published under the initials “I.F. St. b.m.” Hagen (1863: 199), following Ochsenheimer (1805: 8), attributed the authorship to “J.F. Stopp.” I was unable to find anything regarding someone named “J.F. Stopp.” One online source credited the work, though presumably, to Johann Friedrich Rudolph Steiner (Anonymous 2015g). The letters “b.m.” possibly mean “Bau Meister” (= architect, builder) and would support the idea that Steiner is the author. Furthermore, Steiner (1785) published a small book on scolytine beetles a few years earlier, with his full name on the title page, indicating that he was interested in entomology.

#### Stephens, James Francis (London, United Kingdom: 16 September 1792 – 22 December 1852: London, United Kingdom). British entomologist; clerk in the Admiralty at Somerset House in London; retired in 1845 to work unpaid at the British Museum (Natural History); his collection was purchased by the British Museum. References. Gray (1853); Neave and Griffin (1933: 129–130); Smetana and Herman (2001: 148, P); Foote (2004e); Gilbert (2005: 55–56, P).

**1827–1835**. *Illustrations of British entomology; or, a synopsis of indigenous insects: containing their generic and specific distinctions; with an account of their metamorphoses, times of appearance, localities, food, and economy, as far as practicable. Embellished with coloured figures of the rarer and more interesting species. Mandibulata.* Baldwin & Cradock, London. (8vo) CNC, GB

*Vol. I*. iv + 186 + [2] pp. + pls 1–9. This volume was issued in 15 parts. The dates are listed at the bottom of the first page of each sheet as follows: **1**: (pp. i–iv + 1–16) 1 May 1827; **2**: (pp. 17–28) 1 June 1827; **3**: (pp. 29–36) 1 July 1827; **4**: (pp. 37–44) 1 August 1827; **5**: (pp. 45–52) 1 September 1827; **6**: (pp. 53–60) 1 October 1827; **7**: (pp. 61–68) 1 November 1827; **8**: (pp. 69–76) 1 December 1827; **9**: (pp. 77–84) 1 January 1828; **10**: (pp. 85–92) 1 February 1828; **11**: (pp. 93–100) 1 March 1828; **12**: (pp. 101–108) 1 April 1828; **13**: (pp. 109–124) 1 May 1828; **14**: (pp. 125–140) 1 June 1828; **15**: (pp. 141–186 + [2 (List of plates + Errata)] 30 June 1828.

*Vol. II.* 200 pp. + pls 10–15. This volume was issued in eight parts. The dates listed at the bottom of the first page of each sheet are: **16**: (pp. 1–32) 1 July 1828; **17**: (pp. 33–64) 1 August 1828; **18**: (pp. 65–96) 1 September 1828; **19**: (pp. 97–112) 1 October 1828; **20**: (pp. 113–128) 1 January 1829; **21**: (129–160) 1 February 1829; **22**: (pp. 161–176) 1 March 1829; **23**: (pp. 177–200) 15 June 1829.

*Vol. III.* 374 + [6] pp. + pls 16–19. This volume was issued in ten parts. The dates listed at the bottom of the first page of each sheet are: **24**: (pp. 1–6) 30 April 1830; **25**: (pp. 7–54) 31 May 1830; **26**: (pp. 55–86) 30 June 1830; **27**: (pp. 87–102) 31 July 1830; **28**: (pp. 103–150) 15 August 1830; **29**: (pp. 151–166) 31 August 1830; **30**: (pp. 167–182) 30 September 1830; **31**: (pp. 183–230) 31 October 1830; **32**: (pp. 231–246) 30 November 1830; **33**: (pp. 247–374 + [6 (Index, List of plates, Errata)] 31 December 1830.

*Vol. IV.* 413 + [1] pp. + pls 20–23. This volume was issued in 12 parts. The dates listed at the bottom of the first page of each sheet are: **34**: (pp. 1–14) 31 January 1831; **35**: (pp. 15–30) 28 February 1831; **36**: (pp. 31–78) 31 March 1831; **37**: (pp. 79–94) 30 April 1831; **38**: (pp. 95–110) 31 July 1831; **39**: (pp. 111–126) 31 August 1831; **40**: (pp. 127–142) 30 September 1831; **41**: (pp. 143–222) 31 October 1831; **42**: (pp. 223–270) 30 November 1831; **43**: (pp. 271–286) 1 December 1831; **44**: (pp. 287–366) 31 December 1831; **45**: (pp. 367–423 + [1 (List of plates, Errata)] 31 January 1832.

*Vol. V*. 447 + [1] pp. + pls 24–27. This volume was issued in 13 parts. The dates listed at the bottom of the first page of each sheet are: **46**: (pp. 1–16) 31 January 1832; **47**: (pp. 17–64) 29 February 1832; **48**: (pp. 65–96) 31 March 1832; **49**: (pp. 97–160) 30 April 1832; **50**: (pp. 161–192) 31 May 1832; **51**: (pp. 193–224) 30 June 1832; **52**: (pp. 225–240) 31 July 1832; **53**: (pp. 241–288) 30 November 1833; **54**: (pp. 289–304) 31 December 1833; **55**: (pp. 305–368) 31 January 1834; **56**: (pp. 369–384) 28 February 1835; **57**: (pp. 385–432) 31 March 1835; **58**: (pp. 433–447 + [1 (List of plates)] 30 April 1835.

The entire series was published in two sections, Mandibulata (seven volumes) and Haustellata (four volumes), 1828–1837. The Coleoptera are in the first five volumes of the section Mandibulata. A supplement was issued in 1846 but contains no text on Coleoptera.

**1829**. *The nomenclature of British insects; being a compendious list of such species as are contained in the Systematic Catalogue of British Insects, and forming a guide to their classification, &c. &c.* Baldwin and Cradock, London. [2] + 68 pp. (8vo) [DP: 1829 (title page), 1 June 1829 (Blackwelder 1949: 92; see also Herman 2001: 4051), 3 June 1829 (*Athenaeum*), 6 June 1829 (*Lit Gaz*), June 1829 (Peddie and Waddington 1914: 561), 1 July 1829 (*New Monthly Mag*), August 1829 (*Blackw Mag*), November 1829 (*Monthly Rev*)] CNC, GB

This work was also issued printed on one side for the purpose of labelling cabinets. A second edition was published in 1833 [*q.v.*].

**1829**. *A systematic catalogue of British insects: being an attempt to arrange all the hitherto discovered indigenous insects in accordance with their natural affinities. Containing also the references to every English writer on entomology, and to the principal foreign authors. With all the published British genera to the present time.* Baldwin & Cradock, London. xxxiv + [1] + 416 [Part I. Insecta Mandibulata] + 388 [Part II. Insecta Haustellata] pp. (8vo) [DP: 1829 (title page), 15 July 1829 (Blackwelder 1949: 92; see also Herman 2001: 4051), 18 July 1829 (*Lit Gaz*), 22 July 1829 (*Athenaeum*), August 1829 (Sherborn 1922a: cxix), September 1829 (*Blackw Mag*), November 1829 (*Monthly Rev*)] CNC, GB, BHL

This work contains two sections separately paginated.

**1833**. *The nomenclature of British insects; together with their synonymes: being a compendious list of such species as are contained in the Systematic Catalogue of British Insects, and of those discovered subsequently to its publication; forming a guide to their classification, &c.&c. Second edition.* Baldwin and Cradock, London. iv + 135 columns. (8vo) [DP: 1833 (title page), 21 July 1833 (*Mag Nat Hist* 6: 436–437)] ANSP

This work was left unfinished; a second part was “to be published in the autumn [of 1833]” (*Ent Mag* 1: 419–420) but never appeared.

**1839**. *A manual of British Coleoptera, or beetles; containing a brief description of all the species of beetles hitherto ascertained to inhabit Great Britain and Ireland; together with a notice of their chief localities, times and places of appearances, etc.* Longman, Orme, Brown, Green, and Longmans, London. xii + 443 pp. (8vo) [DP: 1839 (title page; introduction dated 15 October 1839), 9 November 1839 (*Athenaeum*), 16 November 1839 (*Lit Gaz*), November 1839 (Low 1953: 345), 2 December 1839 (*Ent Soc Lond*), December 1839 (*Gent Mag*)] CNC, GB

#### Stewart, Charles

**1802.** *Elements of natural history; being an introduction to the Systema Naturae of Linnaeus; comprising the characters of the whole genera, and most remarkable species; particularly of all those that are natives of Britain, with the principal circumstances of their history and manners. Likewise an alphabetical arrangement, with definitions, of technical terms: in two volumes; with twelve explanatory copper-plates. Vol. II. containing the fifth and sixth classes, viz. V. Insects. and VI. Vermes.* T. Cadell Jun. and W. Davies, London. iv + 491 + [1] pp. + pls 7–12. (8vo) [DP: 1802 (title page), 1 April 1802 (*Monthly Mag*), July 1802 (*Anti-Jac Rev Mag*), November 1802 (*British Critic*), March 1803 (*Monthly Rev*)] GB

The work was published anonymously in two volumes, 1801–1802. However, a second edition published in August 1817, Edinburgh, under the title “*Elements of the natural history of the animal kingdom: comprising the characters of the whole genera, and of the most remarkable species, particularly those that are natives of Britain; with the principal circumstances of their history and manners*” has the author’s name listed [McG, GB].

The Coleoptera are on pages 21–98 and 304–305 in the second volume.

#### Stierlin, Wilhelm Gustav (Schaffhausen, Switzerland: 2 November 1821 – 28 March 1907: Schaffhausen, Switzerland). Swiss physician and entomologist particularly interested in Coleoptera; practiced medicine in the Canton of Schaffhausen; his collection was purchased by Otto Leonhard of Dresden. References. Daniel (1908: 394–397, P); Stierlin (1908, P).

**1861**. *Revision der europäischen Otiorhynchus-Arten.* Nicolai, Berlin. 344 pp. (8vo) [DP: 1861 (title page; *Einleitung* dated 11 September 1860), 23 June 1861 (*Lit Centr Deutsch*)] CNC, GB

This publication was issued as a supplement to volume 5 of the *Berliner Entomologische Zeitschrift*.

**1886–1900**. *Fauna coleopterorum Helvetica. Die Käfer-Fauna der Schweiz nach der analytischen Methode.* Bolli & Böcherer, Schaffhausen. (8vo) CNC

*I. Theil.* xii + 667 pp. [DP: 1900 (title page), December 1900 (*Nat Nov*), 1 February 1901 (*Bibl Zool*)].

*II. Theil.* xii + 662 pp. [DP: 1898 (title page)]. The second *Theil* was first issued in several parts as supplements to volumes 7–10 of *Mittheilungen der Schweizerischen Entomologischen Gesellschaft* under the title “*Coleoptera Helvetiae*.” The dates of publication of the parts, as listed by Derksen and Scheiding-Göllner (1965: 20) unless otherwise noted, are as follows: **1**: (pp. 1–64) 1886; **2**: (pp. 65–112) 1888–1889; **3**: (pp. 113–192)^98^ 1890; **4**: (pp. 193–224) 1891, September 1891 (*Nat Nov*); **5**: (pp. 225–256) 1891, January 1892 (*Nat Nov*); **6**: (pp. 257–288) 1892, August 1892 (*Nat Nov*); **7**: (pp. 289–320) 1893, April 1893 (*Nat Nov*); **8**: (pp. 321–352) 1893, September 1893 (*Nat Nov*); **9**: (pp. 353–448)^99^ 1894; **10**: (pp. 449–480) 1895, December 1895 (*Nat Nov*); **11**: (pp. 481–512) 1895, January 1896 (*Nat Nov*); **12**: (pp. 513–544) 1896, August 1896 (*Nat Nov*); **13**: (pp. 545–576) 1896, December 1896 (*Nat Nov*); **14**: (pp. 577–624) 1897, February 1898 (*Nat Nov*), 7 March 1898 (*Bibl Zool*); **15**: (pp. 625–662, i–xii) 1898, 18 April 1898 (*Bibl Zool*), April 1898 (*Nat Nov*); **16**: (pp. i–xii) 1898, 19 September 1898 (*Bibl Zool*).

This work was part of the series *Fauna Insectorum Helvetiae*.

#### Sturm, Jacob (Nuremberg, Bavaria, Germany: 21 March 1771 – 28 November 1848: Nuremberg, Germany). German naturalist, particularly interested in botany and beetles, insect dealer, illustrator and engraver in Nuremberg; received in 1846 a Ph.D. *honoris causa* from the University of Breslau; co-founded the *Naturhistorische Gesellschaft Nürnberg*. References. Hilpert (1849, P); Ratzeburg (1874: 475–476); Wunschmann (1894); Eisinger (1919c, P); Weidner (1983: 326–328, P); Adler (2012: 53–54, P).

**1796**. *Verzeichniss meiner Insecten-Sammlung. Gesammelt und herausgegeben von Jacob Sturm. Mit vier Kupfertafeln.* Nürnberg. 64 pp. + 4 pls. (12mo) [DP: 1796 (title page; *Vorbericht* dated July 1796), 22 September 1796 (see Hagen 1863: 203^100^), 12 October 1796 (*Intell Allg Lit Ztg*)] USNM, BHL

**1800**. *Verzeichnis meiner Insecten-Sammlung, oder entomologisches Handbuch für Liebhaber und Sammler. Erstes Heft. Mit vier ausgemalten Kupfertafeln.* Nürnberg. xvi + 112 + [4] pp. + 4 pls. (8vo) [DP: 1800 (title page; *Vorrede* dated February 1800), 21 May 1800 (*Intell Allg Lit Ztg*)] BHL, GB

**1801–1803**. *Abbildungen zu Karl Illiger’s Uebersetzung von Olivier’s Entomologie oder Naturgeschichte der Insecten mit inhren Gattungs- und Artmerkmalen ihrer Beschreibung und Synonymie. Käfer.* Nürnberg. (4to) NAL, BHL, GB

*Erster Theil. Mit vier und funfzig illuminirten Kupfertafeln.* iv + 136 pp.^101^ + 54 pls. [DP: 1802 (title page; *Vorrede* dated March 1801), 28 January 1803 (*Allg Lit Ztg*)].

*Zweiter Theil. Mit zwei und vierzig illuminirten Kupfertafeln.* 132 pp. + pls 55–96. [DP: 1803 (title page)].

This work was published in 16 *Hefte* (Hagen 1863: 204), the first nine forming the first *Theil* and the other seven the second *Theil*. I have seen wrappers for the first nine *Hefte*, all dated 1801 on their title pages, with the *Vorrede* in the first *Heft* dated March 1801. The collation is as follows: **1**: (iv + 4 pp.); **2**: (pp. 5–20); **3**: (pp. 21–28); **4**: (pp. 29–36); **5**: (pp. 37–44); **6**: (pp. 49–56); **7**: (pp. 57–72); **8**: (pp. 73–96); **9**: (pp. 97–136). The first *Heft* was recorded on 17 June 1801 (*Reichs-Anz*) and the first six *Hefte* on 30 June 1802 (*Intell Allg Lit Ztg*).

There is no author name on the title pages but the *Vorrede* is signed by Jacob Sturm. The work consists of supplementary illustrations for the German translation of Olivier’s *Entomologie, ou histoire naturelle des insectes* [*q.v.*] issued by Illiger, 1800–1802 [*q.v.*]; descriptions of all the species illustrated are provided in Latin and German.

**1805–1847**. *Deutschlands Fauna in Abbildungen nach der Natur mit Beschreibungen. V. Abtheilung. Die Insecten.* Nürnberg. (8vo) CNC, GB

*Erstes Bändchen. Käfer. Mit 20 illuminirten Kupfertafeln.* 10 + [1] + xxxxiiii + 268 pp. + 20 pls. [DP: 1805 (title page; *Vorrede* dated 16 February 1805), 20 February 1806 (*Allg Lit Ztg*)].

*Zweites Bändchen. Käfer. Mit 32 illuminirten Kupfertafeln.* [4] + 279 pp. + pls 21–52. [DP: 1807 (title page; *Vorbericht* dated March 1807)].

*Drittes Bändchen. Käfer. Mit 24, von dem Verfasser nach der Natur gezeichneten, und in Kupfer gestochenen, illuminirten Kupfertafeln.* 192 pp. + pls 53–76. [DP: 1815 (title page; *Vorbericht* dated January 1815)].

*Viertes Bändchen. Käfer. Mit 24, von dem Verfasser nach der Natur gezeichnet, und in Kupfer gestochenen, illuminirten Kupfertafeln.* 179 pp + pls 77–104. [DP: 1818 (title page)].

*Fünftes Bändchen. Käfer. Mit 33 illuminirten Kupfertafeln.* 220 pp. + pls 105–137. [DP: 1824 (title page), October 1824 (*Isis*, Heft X: [2]), July–December 1824 (*Verz Bücher Landkarten*)].

*Sechstes Bändchen. Käfer. Mit 27 illuminirten Kupfertafeln.* 188 pp. + pls 138–163. [DP: 1825 (title page), January 1826 (*Isis*, Heft I: [2]), July–December 1826 (*Verz Bücher Landkarten*)].

*Siebentes Bändchen. Käfer. Mit 21 illuminirten Kupfertafeln.* [2] + 186 pp. + pls 164–184. [DP: 1827 (title page), July–December 1827 (*Verz Bücher Landkarten*)].

*Achtes Bändchen. Käfer. Mit 18 illuminirten Kupfertafeln.* vi + 170 pp. + pls 185–202. [DP: 1834 (title page; *Vorerinnerung* dated December 1833), 26 February 1834 (*Lit Ztg*), March 1834 (*Isis*, Heft III: [2]), January–June 1834 (*Verz Bücher Landkarten*)].

*Neuntes Bändchen. Käfer. Mit 14 illuminirten Kupfertafeln.* xii + 120 pp. + pls 203–216. [DP: 1835 (title page; *Vorerinnerung* dated October 1835), September 1835 (*Isis*, Heft IX: [2]), October–December 1835 (*Foreign Quart Rev*), July–December 1835 (*Verz Bücher Landkarten*)].

*Zehntes Bändchen. Käfer. Mit 12 illuminirten Kupfertafeln.* 108 pp. + pls 216–227. [DP: 1836 (title page), 5 August 1836 (*Allg Bibl Deutsch*), 10 August 1836 (*Lit Ztg*), October 1836 (*Bull Litt Étr*), July–December 1836 (*Verz Bücher Landkarten*), 18 January 1837 (*Allg Forst Jagd Ztg*)].

*Elftes Bändchen. Käfer. Mit 16 illuminirten Kupfertafeln.* 148 pp. + pls 227–243. [DP: 1837 (title page), 7 June 1837 (*Lit Ztg*), June 1837 (*Bull Litt Étr*), January–June 1837 (*Verz Bücher Landkarten*), September 1837 (*Isis*, Heft IX: [2]), 22 December 1837 (*Allg Bibl Deutsch*)].

*Zwölftes Bändchen. Käfer. Mit 15 illuminirten Kupfertafeln.* 88 pp. + pls 244–258. [DP: 1837 (title page), 6 December 1837 (*Lit Ztg*), 22 December 1837 (*Allg Bibl Deutsch*), July–December 1837 (*Verz Bücher Landkarten*)].

*Dreizehntes Bändchen. Käfer. Mit 13 illuminirten Kupfertafeln.* 128 pp. + pls 259–271. [DP: 1838 (title page), May 1838 (*Isis*, Heft V: [3]), 25 July 1838 (*Lit Ztg*), July–December 1838 (*Verz Bücher Landkarten*)].

*Vierzehntes Bändchen. Käfer. Mit 16 illuminirten Kupfertafeln.* 123 + [1] pp. + pls 272–287. [DP: 1839 (title page), 8 May 1839 (*Lit Ztg*), January–June 1839 (*Verz Bücher Landkarten*), 20 September 1839 (*Allg Bibl Deutsch*)].

*Fünfzehntes Bändchen. Käfer. Mit 16 illuminirten Kupfertafeln.* xii + 140 pp. + pls 288–303. [DP: 1844 (title page; *Vorbericht* dated December 1843), 29 March 1844 (*Leip Reper*), 9 May 1844 (*Allg Bibl Deutsch*), 18 May 1844 (*Lit Ztg*), January–June 1844 (*Verz Bücher Landkarten*)]. Pages 131–137 and plate 303 were also issued separately at the same time under the title “*Anophthalmus. Blindlaufkäfer. Neue Gattung aus der Familie der Caraben. Mit einer colorirten Tafel.*” (Hagen 1863: 205).

*Sechzehntes Bändchen. Käfer. Mit 16 illuminirten Kupfertafeln.* 114 pp. + pls 304–319. [DP: 1845 (title page), 16 January 1845 (*Allg Bibl Deutsch*), 25 January 1845 (*Lit Ztg*), 21 February 1845 (*Leip Reper*), May 1845 (*Isis*, Heft V: [2]), January–June 1845 (*Verz Bücher Landkarten*), 11 December 1845 (*Ent Ver Stettin*)].

*Achtzehntes Bändchen. Käfer. Mit 16 illuminirten Kupfertafeln.* vi + 90 pp. + pls 329–344. [DP: 1846 (title page; *Vorbericht* dated October 1846), 7 January 1847 (*Allg Bibl Deutsch*), January–June 1847 (*Verz Bücher Landkarten*)].

*Neunzehntes Bändchen. Käfer. Mit 16 illuminirten Kupfertafeln.* vi + 120 pp. + pls 345–360. [DP: 1847 (title page; *Vorbericht* dated November 1847), 13 January 1848 (*Allg Bibl Deutsch*), 28 January 1848 (*Leip Reper*), January 1848 (*Intell Allg Lit Ztg*), 4 March 1848 (*Ent Ver Stettin*), April 1848 (*Isis*, Heft IV: [2]), January–June 1848 (*Verz Bücher Landkarten*)]. Pages 112–116 were also issued separately at the same time under the title “*Beschreibung einer neuen Art von Anophthalmus. Blindlaufkäfer.*” (Hagen 1863: 205).

Volume 17 of the series was authored by C.J. F. Gillmeister, 1845 [*q.v.*], volumes 20–23 by J.H.C.F. Sturm, 1849–1857 [*q.v.*], son of J. Sturm.

The half-title page reads “*Deutschlands Insecten.*”

**1826**. *Catalog meiner Insecten-Sammlung. Erster Theil. Käfer. Mit 4 ausgemalten Kupfertafeln.* Nürnberg. viii + 207 + 16 + [2 (Avertissement)] pp. + 4 pls. (8vo) [DP: 1826 (title page), July–December 1826 (*Verz Bücher Landkarten*), January 1827 (*Isis*, Heft I: [2]), June 1827 (*Allg Lit Ztg*)] CNC, GB

The work includes the following sections: *Systematische Folge der Gattungen in meiner Sammlung* (pp. 1–52); *Beschreibungen und Abbildungen einiger neuen Arten aus meiner Sammlung, zur Erläuterung des Systems* (pp. 53–84); *Catalog oder alphabetisches Verzeichnifs der Käfer in meiner Sammlung* (pp. 85–207); *Verzeichnifs der bei mir vorräthigen Insecten* (pp. 1–14); *Verzeichnifs der in meinem Verlage erschienenen naturhistorischen Werke* (pp. 15–16); *Avertissement* (2 unnumbered pages). Many new Coleoptera species are described in the second section.

**1843**. *Catalog der Kaefer-Sammlung von Jacob Sturm. Mit 6 ausgemalten Kupfertafeln.* Nürnberg. xii + 386 pp. + 6 pls. (8vo) [DP: 1843 (title page; *Vorbericht* dated January 1843), 27 July 1843 (*Allg Bibl Deutsch*), 26 August 1843 (*Lit Ztg*), August 1843 (*Ent Ver Stettin*), 1 September 1843 (*Leip Reper*), July–December 1843 (*Verz Bücher Landkarten*)] CNC, GB

This work includes the following sections: *Catalog der Kaefer-Sammlung von Jacob Sturm* (pp. 1–321); *Beschreibungen und Abbildungen neuer so wie einiger noch wenig bekannten Arten aus meiner Sammlung* (pp. 323–360); *Alphabetisches Register* (pp. 361–386).

#### Sturm, Johann Heinrich Christian Friedrich (Nuremberg, Bavaria, Germany: 6 February 1805 – 24 January 1862: Nuremberg, Germany). German engraver and naturalist in Nuremberg; son of Jacob Sturm [*q.v.*]. References. Hauck (1862); Mearns and Mearns (1988: 356–358, P).

**1849–1856**. *Dr. Jacob Sturm’s Deutschlands Fauna in Abbildungen nach der Natur mit Beschreibungen. V. Abtheilung. Die Insecten.* Nürnberg. (8vo) CNC, GB

*Zwanzigstes Bändchen. Käfer. Mit 16 illuminirten Kupfertafeln.* vi + 103 pp. + pls 361–376. [DP: 1849 (title page; *Vorwort* dated October 1849), November 1849 (*Ent Ztg* 10: 352), 20 December 1849 (*Ent Ver Stettin*), July–December 1849 (*Verz Bücher Landkarten*), 13 February 1850 (*Bibl Lit Anz*)]. Pages 91–100 and plate 376 were also issued separately at the same time under the title “*Leptodirus. Gattung aus der Familie der Scydmaenides beschrieben und abgebildet. Mit 1 colorirten Tafel.*” [GB].

*Einundzwanzigstes Bändchen. Käfer. Mit 16 illuminirten Kupfertafeln.* vi + 116 pp. + pls 377–392. [DP: 1851 (title page; *Vorwort* dated July 1851), 13 November 1851 (*Allg Bibl Deutsch*), December 1851 (*Ent Ver Stettin*), July–December 1851 (*Bibliot Hist-Nat*), 7 January 1852 (*Zool-Bot Gesell Wien*)]. Pages 109–113 were also issued separately at the same time under the title “*Abbildung und Beschreibung einer dritten deutschen Art von Anophthalmus. Blindlaufkäfer.*” (Hagen 1863: 205).

*Zweiundzwanzigstes Bändchen. Käfer. Mit 16 illuminirten Kupfertafeln.* vi + 97 pp. + pls 393–408. [DP: 1853 (title page; *Vorwort* dated May 1853), 1 September 1853 (*Allg Bibl Deutsch*), 8 September 1853 (*Ent Ver Stettin*), July–September 1853 (*Cat Muquardt*), 15 October 1853 (*Intell Serapeum*), July–December 1853 (*Bibliot Hist-Nat*)]. Pages 90–95 and plate 408 were also issued separately at the same time under the title “*Abbildung und Beschreibung einer vierten und fünften Art von Anophthalmus. Blindlaufkäfer.*” (Hagen 1863: 205).

*Dreiundzwanzigstes Bändchen. Käfer. Mit 16 illuminirten Kupfertafeln.* 123 pp. + pls 409–424. [DP: 1857 (title page), 25 December 1856 (*Allg Bibl Deutsch*), October–December 1856 (*Cat Muquardt*), 31 January 1857 (*Intell Serapeum*), 4 February 1857 (*Zool-Bot Gesell Wien*), February 1857 (*Allg Bibl*), 6 April 1857 (*Ent Soc Lond*), January–June 1857 (*Bibliot Hist-Nat*)].

These constitute parts 20–23 of the series started by his father, Jacob Sturm [*q.v.*]. The half-title page reads “*Dr. Jacob Sturm’s Deutschlands Insecten.*”

#### Suckow [Succow], Friedrich Wilhelm Ludwig (Heidelberg, Baden-Württemberg, Germany: 1770 – 21 June 1838: Mannheim, Baden-Württemberg, Germany). German physician and naturalist; professor of natural history and curator of the natural history museum in Mannheim. Reference. Pagel (1894).

**1819**. *Naturgeschichte der Insekten. Ersten Bandes erster Theil. Mit drey Kupfertafeln.* Engelmann, Heidelberg. x + 262 pp. + 3 pls. (8vo) [DP: 1819 (title page; *Vorrede* dated March 1819), July–September 1819 (*Allg Bericht Bücher*), November 1819 (*Isis*, Heft XI: 1750] CNC (mf)

This work pertains to the Scarabaeidae (*sensu lato*) where the following genera are treated: *Lethrus*, *Geotrupes*, *Scarabaeus*, *Onitis*, *Copris*, *Ateuchus*, *Aphodius*, *Hexodon*. Only the first *Theil* was published.

#### Sulzer, Johann Heinrich (Winterthur, Switzerland: 18 September 1735 – 14 August 1814: Winterthur, Switzerland). Swiss physician and entomologist in Winterthur. Reference. Hegner (1816).

**1761**. *Die Kennzeichen der Insekten, nach Anleitung des Königl. Schwed. Ritters und Leibarzts Karl Linnaeus, durch XXIV. Kupfertafeln erläutert und mit derselben natürlichen Geschichte begleitet von J.H. Sulzer. Mit einer Vorrede des Herrn Johannes Gessners.* Heidegger und Comp., Zürich. xxviii + 203 + 67 + [1] pp. + 24 pls. (4to) [DP: 1761 (title page; *Vorrede* dated 26 August 1761 [p. xvi])] MCZ, GB, BHL

Specific names in the first section (203 pp.) of the text are vernacular but Latinized in the “*Erklärungs-Tafeln zum Natursistem der Insekten*” section (67 pp.).

[**1775**]–**1776**. *Abgekürzte Geschichte der Insecten. Nach dem Linaeischen System.* H. Steiner u. Comp., Winterthur. (4to) MCZ, BHL, GB

*Erster Theil.* xxviii + 274 pp. [DP: 1776 (title page; dedication dated 2 March 1776), 21 April 1776 (Evenhuis 1997b: 755), September 1776 (*Allg Verz Bücher*), 18 October 1776 (*Jena Ztg*)].

*Zweeter Theil. Welcher XXXII. ausgemahlte Kupfertafeln enthält.* [1] + 71 + [1] pp. + 32 pls. [DP: 1776 (title page), 21 April 1776 (Evenhuis 1997b: 755), September 1776 (*Allg Verz Bücher*), 18 October 1776 (*Jena Ztg*)].

This work consists of two parts issued simultaneously. It is sometimes recorded under the title “*Abgekürzte Geschichte schweizerischer und ausländischer Insecten*” (e.g., Jäggli 1958: 199; Huber 2013: 403); I have not been able to confirm if a second title page was issued.

The plates for this book were probably available already in 1775 since they are consistently mentioned, sometimes with the species named, by Fuessli (1775); there are no names on the plates themselves.

#### Swainson, William (London, United Kingdom: 8 October 1789 – 6 December 1855: Lower Hutt, New Zealand). British naturalist, traveler and illustrator; worked for the Army Commissariat in Sicily (1807–1815); explored Brazil where he collected extensively (1816–1818); upon his return, lived in England, earning a living as an artist and author, before moving with his family to New Zealand in 1841; his collections were sold at auction in London in 1823 and 1840. References. Anonymous (1857: xlix–liii); Carvalho (1918); Galloway (1978); Mearns and Mearns (1988: 359–364, P); Natusch (2004, P); Adler (2012: 107–108, P).

**1840**. [Swainson, W. and Shuckard, W.E.] *The cabinet cyclopaedia. Conducted by the Rev. Dionysius Lardner assisted by eminent literary and scientific men. Natural history. On the history and natural arrangement of insects.* Longman, Orme, Brown, Green, & Longmans, and John Taylor, London. [3] + 406 pp. (12mo) [DP: 1840 (title page; Advertisement dated November 1840), 19 December 1840 (*Athenaeum*; *Lit Gaz*), December 1840 (Sherborn 1922a: cxxi), 27 January 1841 (*Lit Ztg*)] GB

This book forms volume 129 of Lardner’s series *The cabinet cyclopaedia*. The first unnumbered page reads “Those paragraphs in this volume with the initials W.E.Sh. are written by Mr. Shuckard, and where several of these follow each other they are affixed to the last only; but the system of classification is exclusively Mr. Swainson’s.” The Coleoptera are on pages 190–334.

#### Tarnier, Frédéric (? – 1890). French naturalist interested mainly in Coleoptera, Lepidoptera and shells; built up a collection of Curculionidae, rich in species from Morocco, which was acquired by Jules Desbrochers des Loges [*q.v.*]. Reference. Constantin (1992: 85).

**1860**. Insectes coléoptères. Pp. 87–96 *in*: *Iles Açores. Notice sur l’histoire naturelle des Açores suivie d’une description des mollusques terrestres de cet archipel avec cinq planches gravées et coloriées; par Arthur Morelet.* J.-B. Baillière et Fils, Paris. [1] + 214 + [2] + [5] pp. + 5 pls. (8vo) [DP: 1860 (title page), 11 August 1860 (*Bibl Fr*), 3 September 1860 (*Ent Soc Lond*), 15 September 1860 (*Publ Circ*), September 1860 (*Allg Bibl*)] MCZ, BHL

#### Taschenberg, Ernst Ludwig (Naumburg, Saxony-Anhalt, Germany: 10 January 1818 – 19 January 1898: Halle an der Saale, Saxony-Anhalt, Germany). German entomologist; professor of entomology at the University of Halle (Saale). Reference. Taschenberg (1918b).

**1878–1879**. *Praktische Insekten-Kunde oder Naturgeschichte aller derjenigen Insekten, mit welchen wir in Deutschland nach den bisherigen Erfahrungen in nähere Berührung kommen können nebst Angabe der Bekämpfungsmittel gegen die schädlichen unter ihnen. Mit vielen Holzschnitten.* M. Heinsius, Bremen. (8vo) GB

*Erster Theil*. vi + 233 pp. [DP: 1879 (title page), 19 December 1878 (*Isis Lieb*), December 1878 (*Allg Bibl*; *Zool Gart*), July–December 1878 (*Verz Bücher Landkarten*), 13 January 1879 (*Zool Anz*), January 1879 (*Nat Nov*)]. Another title page reads “*Einführung in die Insekten-Kunde. Mit 46 Holzschnitten*.”

*Zweiter Theil.* viii + 401 pp. [DP: 1879 (title page), September 1879 (*Allg Bibl*; *Nat Nov*), 1 November 1879 (*Lit Centr Deutsch*), 17 November 1879 (*Zool Anz*), 18 December 1879 (*Isis Lieb*), July–December 1879 (*Bibliot Hist-Nat*), January 1880 (*Zool Gart*)]. Another title page reads “*Die Käfer und Hautflügler. Mit 98 Holzschnitten*.”

The entire series consists of five volumes, 1878–1880. The Coleoptera are in the first (pp. 37–66) and second volumes (pp. 1–311). Despite the title page of the first *Theil* is dated 1879, the volume was issued in 1878.

**1881**. *La creacion. Historia natural. Division de la obra: Zoología ó reino animal traducida y arreglada de la última edicion alemana de la obra del célebre Dr. A.E. Brehm. Antropologia, botanica, mineralogia, geologia y paleontologia escritas por eruditos autores españoles con presencia de los mas completos y recientes datos de estas diferentes ramas de la ciencia. Tomo VI. Insectos por el Doctor E.L. Taschenberg.* Montaner y Simón, Barcelona. xiv + 392 pp. (4to) [DP: 1881 (title page)] GB

This volume is an expanded translation of Taschenberg’s “*Die Insekten, Tausendfüssler und Spinnen*” issued in 1877 and forming volume 9 of “*Brehms Thierleben*” series of ten volumes, 1876–1879. The German edition is not structured in a systematic way and so is not included in this compilation. The Coleoptera are on pages 1–94 in the Italian version which is systematically structured; several parts were added in the translation. This volume is structurally very similar to volume 6 of Vilanova y Piera’s “*La creacion*” [*q.v.*] issued by the same publisher; some parts in fact are identical.

#### Thienemann, Friedrich August Ludwig (Gleina, Thuringia, Germany: 22/25 December 1793 – 24 June 1858: Trachenberge [currently part of Dresden], Saxony, Germany). German physician and zoologist, best known for his work in ornithology; professor of zoology at the University of Leipzig; later, curator of the natural history collections in Dresden (currently the Staatliches Museum für Tierkunde) and from 1839 librarian at the Royal Public Library. References. Koenig-Warthausen (1859); Jentsch (1894).

**1828**. *Lehrbuch der Zoologie.* August Rücker, Berlin. xx + 686 pp. (8vo) [DP: 1828 (title page; *Vorwort* dated September 1828), 6 December 1828 (*Bibl Deutsch*), July–December 1828 (*Verz Bücher Landkarten*), 13 January 1829 (*Intell Jena Lit Ztg*), January–March 1829 (*Foreign Quart Rev*)] GB

Another title page reads “*Encyclopädie der speciellen Naturgeschichte von D. C.F. Naumann, D. H.G.L. Reichenbach und D. F.A.L. Thienemann. III. Band: Zoologie.*” The Coleoptera are on pages 267–334.

#### Thiersch, Ernst Ludwig (Kirchscheidungen [currently part of Laucha an der Unstrut], Saxony-Anhalt, Germany: 9 July 1786 – 10 August 1869: Dresden, Saxony, Germany). German forester; from 1818, chief forester in Eibenstock, Saxony. References. Ratzeburg (1874: 483–484); Hess (1885: 369–371).

**1829**. *Die Forstkäfer, oder vollständige Naturgeschichte der vorzüglichsten, den Gebirgsforsten schädlichen Insekten, hauptsächlich der Borkenkäfer mit Angabe der Mittel zu ihrer Vertilgung. Nebst zwei Kupfertafeln.* J.G. Cotta, Stuttgart und Tübingen. ix + 37 pp. + 2 pls. (8vo) [DP: 1830 (title page; *Vorwort* dated January 1829), 17 October 1829 (*Allg Forst Jagd Ztg*), January–June 1830 (*Verz Bücher Landk*)] GB

This work treats 11 species of insects, including eight species of beetles. One of them is listed as “*Bostrichus abietiperda*, mihi” (p. 19). However from the context it is evident that the author refers to the species described under the same name by Bechstein in 1818 [*q.v.*]. Despite the date on the title page, this work was issued in 1829.

#### Thomson, Carl Gustaf (Mellan-Grefvie, Sweden: 13 October 1824 – 20 September 1899: Lund, Sweden). Swedish entomologist; adjunct professor and curator of the entomological collections at the Zoological Museum, University of Lund; his collection of Coleoptera is at that museum. References. Bengtsson (1900, P); Smetana and Herman (2001: 150–151, P).

**1857**. *Skandinaviens Coleoptera, synoptiskt bearbetade. Häftet I. Carabici.* Berlingska Boktryckeriet, Lund. [2] + 64 pp. (8vo) [DP: 1857 (title page; preliminaries dated December 1856), March 1858 (*Allg Bibl*)] CNC (ph)

**1859–1868**. *Skandinaviens Coleoptera, synoptiskt bearbetade.* Berling [vols 1–3] / Lundberg [vols 4–10], Lund. (8vo) CNC, SME

*Tom. I.* [3] + 290 pp. [DP: 1859 (title page; *förord* dated April 1859), 14 December 1859 (*Vetens Akad*)].

*Tom. II.* 304 pp. [DP: 1860 (title page), 21 February 1861 (*Kaiser Akad Wiss*), April 1861 (*Allg Bibl*)].

*Tom. III.* 278 pp. [DP: 1861 (title page), 8 May 1861 (*Vetens Akad*), September 1861 (*Ent Ztg* 22: 299)].

*Tom. IV.* 269 pp. [DP: 1862 (title page), 12 November 1862 (*Vetens Akad*)].

*Tom. V.* 340 pp. [DP: 1863 (title page), 10 June 1863 (*Vetens Akad*), September–October 1863 (*Allg Bibl*)].

*Tom. VI.* 385 pp. [DP: 1864 (title page), 5 September 1864 (*Ent Soc Lond*), 10 October 1864 (*Ent Soc Phila*), 12 October 1864 (*Vetens Akad*), January 1865 (*Allg Bibl*)].

*Tom. VII.* 394 pp. [DP: 1865 (title page), 11 October 1865 (*Vetens Akad*), 8 February 1866 (*Kaiser Akad Wiss*)]. This tome was issued in two parts: (pp. 1–??) 7 August 1865 (*Ent Soc Lond*); (pp. ??–394) 1 January 1866 (*Ent Soc Lond*).

*Tom. VIII.* 409 pp. + lxxv (Systematisk förteckning öfver de i Thomsons Skandinaviens Coleoptera Tom. I–VIII beskrifna arter). [DP: 1866 (title page), 10 October 1866 (*Vetens Akad*)].

*Tom. IX.* 407 pp. [DP: 1867 (title page), 1 June 1868 (*Ent Soc Lond*)].

*Tom. X.* 420 pp. [DP: 1868 (title page)].

The first volume includes a *Conspectus familiarum et generum Coleopterorum Scandinaviae* (pp. 1–161) in which the author lists the families, subfamilies, tribes and genera (along with their type species) of Coleoptera for the area covered. Several of the type species listed are the first valid ones published.

**1862**. *Skandinaviens insecter, en handbok i entomologi, till elementar- läroverkens tjenst. (Med öfver 100 i texten tryckte figurer).* F. Berling, Lund. [2] + xx + 392 pp. + 1 pl. (8vo) [DP: 1862 (title page; *förord* dated May 1862), January 1863 (*Allg Bibl*)] GB

The Coleoptera are on pages 1–98. A second, expanded edition was issued in 1885 [*q.v.*].

**1869–1897**. *Opuscula entomologica.* Lundberg, Lund [vol. 1] / Håkan Ohlssons, Lund [vols 2–3] / Berling, Lund [vols 4–5] / Fr. Berling, Lund [vols 6–7] / Sandberg & Jönssons, Trelleborg [vol. 8] / Haquinus Ohlsson, Lundae [vols 9–13] / E. Malmström, Lundae [vols 14–21] / Haqu Olai, Lundae [vol. 22]. (8vo) CNC

*Fasciculus primus.* Pp. 3–82. [DP: 1869 (title page)]. No section deals with Coleoptera.

*Fasciculus secundus.* Pp. 83–304. [DP: 1870 (title page), February 1871 (*Ent Monthly Mag* 7: 204)]. One section deals with Coleoptera: *IX. Några för Sveriges Fauna nya Coleoptera* (pp. 124–140).

*Fasciculus tertius.* Pp. 305–358 + 2 pls [DP: 1870 (title page)]. The following sections pertain to Coleoptera: *XVIII. Bidrag till Sveriges Insect-fauna. (1) a) Coleoptera* (pp. 322–339); *XIX. Nägra ord om insect-kroppens sammansättning, särskilt med hänsyn till Coleoptera, samt förklaring af plancherna* (pp. 341–356).

*Fasciculus quartus.* Pp. 361–452. [DP: 1871 (title page), 4 November 1872 (*Ent Soc Lond*), 1 June 1873 (*Pet Nouv Ent*)]. The following section pertains to Coleoptera: *XX. Bidrag till Sveriges Insect-fauna. (2) a) Coleoptera* (pp. 361–394).

*Fasciculus quintus.* Pp. 455–530 + [2 (Explicatio Tabulae)] pp. + 1 pl. [DP: 1873 (title page)]. The following section pertains to Coleoptera: *XXII. Några för Sveriges fauna nya Coleoptera* (pp. 527–530).

*Fasciculus sextus.* Pp. 535–612. [DP: 1874 (title page)]. The following section pertains to Coleoptera: *XXIII. Bidrag till Skandinaviens Coleoptera* (pp. 535–553).

*Fasciculus septimus.* Pp. 615–729 + [2 (Explicatio tabulae)] pp. + 1 pl. [DP: 1875 (title page), November 1875 (*Allg Bibl*), July–December 1875 (*Bibliot Hist-Nat*), 3 May 1876 (*Ent Soc Lond*)]. This fascicle has only one chapter: *XXVI. Några anmärkningar öfver arterna af slägtet Carabus*.

*Fasciculus octavus.* Pp. 732–841. [DP: 1877 (title page), 23 March 1878 (*Svensk Bokh*), March 1878 (*Nederl Ent Ver*), January–June 1878 (*Bibliot Hist-Nat*)]. No section deals with Coleoptera.

*Fasciculus nonus.* Pp. 843–936. [DP: 1883 (title page), 9 January 1884 (*Soc Ent Fr*)]. No section deals with Coleoptera.

*Fasciculus decimus.* Pp. 939–1040. [DP: 1884 (title page), 12 November 1884 (*Soc Ent Fr*)]. The following section pertains to Coleoptera: *XXXIV. Bidrag till Sveriges insectfauna. a) Coleoptera* (pp. 1029–1036).

*Fasciculus undecimus.* Pp. 1043–1182. [DP: 1887 (title page), 25 January 1888 (*Soc Ent Fr*), February 1888 (*Ent Nachr* 14: 63), March 1888 (*Nat Nov*)]. No section pertains to Coleoptera.

*Fasciculus duodecimus.* Pp. 1185–1318. [DP: 1888 (title page), 13 June 1888 (*Soc Ent Fr*), July 1888 (*Nat Nov*), 17 September 1888 (*Zool Anz*), October 1888 (*Ent Nachr* 14: 302)]. The following section pertains to Coleoptera: *XXXVII. Bidrag till Sveriges insectfauna. a) Coleoptera* (pp. 1202–1208).

*Fasciculus tredecimus.* Pp. 1321–1438. [DP: 1889 (title page), April 1889 (*Nat Nov*), 12 June 1889 (*Soc Ent Fr*), June 1889 (*Ent Nachr* 15: 196), 2 September 1889 (*Zool Anz*)]. The following section pertains to Coleoptera: *XXXXI. Bidrag till Sveriges insectfauna. a) Coleoptera* (p. 1401).

*Fasciculus XIVmus.* Pp. 1441–1534. [DP: 1890 (title page), 25 June 1890 (*Soc Ent Fr*), June 1890 (*Nat Nov*), September 1890 (*Humboldt* 9: 442)]. The following section pertains to Coleoptera: *XLIV. Bidrag till Sveriges insectfauna. a) Coleoptera* (pp. 1526–1527).

*Fasciculus XVmus.* Pp. 1537–1656. [DP: 1891 (title page), September 1891 (*Nat Nov*), 14 October 1891 (*Soc Ent Fr*)]. The following section pertains to Coleoptera: *XLVI. Bidrag till Sveriges insectfauna. a) Coleoptera* (p. 1601).

*Fasciculus XVImus.* Pp. 1659–1772 + [1]. [DP: 1892 (title page), 22 June 1892 (*Soc Ent Fr*), July 1892 (*Nat Nov*; *Ent Nachr* 18: 224)]. The following section pertains to Coleoptera: *XLVI. Bidrag till Sveriges insectfauna. a) Coleoptera* (p. [1773]).

*Fasciculus XVIImus.* Pp. 1777–1886. [DP: 1892 (title page), 8 February 1893 (*Soc Ent Fr*), February 1893 (*Nat Nov*), April 1893 (*Ent Nachr* 19: 128)]. The following section pertains to Coleoptera: *XLVIII. Bidrag till Sveriges insectfauna. a) Coleoptera* (p. 1862).

*Fasciculus XVIIImus.* Pp. 1889–1967. [DP: 1893 (title page), 14 June 1893 (*Soc Ent Fr*), August 1893 (*Nat Nov*)]. No section pertains to Coleoptera.

*Fasciculus XIXmus.* Pp. 1971–2137. [DP: 1894 (title page), 23 May 1894 (*Soc Ent Fr*), July 1894 (*Nat Nov*)]. No section pertains to Coleoptera.

*Fasciculus XXmus.* Pp. 2141–2339. [DP: 1895 (title page), May 1895 (*Nat Nov*), July 1895 (*Ent Nachr* 21: 221)]. No section pertains to Coleoptera.

*Fasciculus XXImus.* Pp. 2343–2404. [DP: 1896 (title page), 24 June 1896 (*Soc Ent Fr*), June 1896 (*Nat Nov*)]. The following section pertains to Coleoptera: *LIV. Bidrag till Sveriges insectfauna. a) Coleoptera* (pp. 2389–2390).

*Fasciculus XXIImus.* Pp. 2407–2452. [DP: 1897 (title page), July 1897 (*Nat Nov*), 11 October 1897 (*Bibl Zool*), 10 November 1897 (*Soc Ent Fr*)]. The following section pertains to Coleoptera: *LVII. Bidrag till Sveriges insectfauna. a) Coleoptera* (p. 2451).

**1885**. *Skandinaviens insecter, en handbok i entomologi, till allmänna läroverkens tjenst. Andra omarbetade upplagan. Första häftet: Coleoptera. (Med 5 planscher.).* Malmström & Komp, Lund. xx + 186 pp. + 5 pls. (8vo) [DP: 1885 (title page; *förord* dated May 1885), 27 June 1885 (*Svensk Bokh*), 11 November 1885 (*Soc Ent Fr*), July–December 1885 (*Bibliot Hist-Nat*)] YB

#### Thomson, James Livingston (New York, New York, USA: 15 March 1828 – December 1897). Eccentric French-American entomologist of independent means; educated in France where he spent a large part of his life; his primary activity consisted in the development of his beetle collection, particularly the showy groups such as Cerambycidae, Buprestidae, and cetoniids, and his entomological publications; married to Delia Stewart Parnell in 1859, sister of the influent Irish politician Charles Stewart Parnell, for whom he manifested an extreme jealousy in a way that made him publicly ridiculous; after the death of his wife, married a second time in 1885; his collection was scattered but a large part was sold to René Oberthür and is now in the Muséum National d’Histoire Naturelle in Paris. References. Hayek (1989); Cambefort (2006: 299–301, P).

**1857–1858**. *Monographie des cicindélides ou exposé méthodique et critique des tribus, genres et espèces de cette famille. Tome premier.* J.B. Baillière, Paris. xvii + 66 pp. + 10 pls. (4to) CMLE, GB

This work was issued in three livraisons: **1**: (xvii + 18 pp. + pls 1–3) 1857 (wrapper), 25 February 1857 (*Soc Ent Fr*), 7 March 1857 (*Bibl Fr*), May 1857 (*Allg Bibl*), January–June 1857 (*Bibliot Hist-Nat*); **2**: (pp. 19–42 + pls 4–6) 1857 (wrapper); **3**: (pp. 43–66 + pls 7–10) 1859 (wrapper), 21 December 1858 (*Acad Nat Sci Phila*). The work came with colored or uncolored plates.

**1857–1858**. *Archives entomologiques ou recueil contenant des illustrations d’insectes nouveaux ou rares*. Paris. (4to) CNC, BHL

*Tome premier.* 514 + [1 (Errata)] pp. + 22 (including frontispiece) pls. This volume was issued in 12 livraisons. Unless otherwise stated, the dates of publication are from Hayek (1989: 90–91)^102^: **1**: (pp. 5–24 + 3 pls) 25 March 1857, 28 March 1857 (*Bibl Fr*; *Courrier Lib*); **2**: (pp. 25–48 + 2 pls) 24 June 1857; **3**: (pp. 49–80 + 3 pls) 24 June 1857; **4**: (pp. 81–104 + 1 pl.) August 1857; **5**: (pp. 105–152) August 1857; **6**: (pp. 153–200 + 1 pl.) August 1857; **7**: (pp. 201–248 + 2 pls) *ca* 10 November 1857 (Guérin-Méneville 1857: 571); **8**: (pp. 249–280) *ca* 10 November 1857 (Guérin-Méneville 1857: 571); **9**: (pp. 281–344) 25 December 1857; **10**: (pp. 345–424) 1 June 1858 (*Acad Nat Sci Phila*), 7 June 1858; **11**: (pp. 425–464) April–June 1858 (*Soc Ent Fr* [*Bulletin bibliographique*, p. cxx]), 3 August 1858 (*Acad Nat Sci Phila*); **12**: (pp. [465]–514 + [1]) April–June 1858 (*Soc Ent Fr* [*Bulletin bibliographique*, p. cxx]), 21 December 1858 (*Acad Nat Sci Phila*). The title page is dated 1857, the preface 15 March 1857. The complete tome was recorded on 26 June 1858 (*Bibl Fr*).

*Tome deuxième.* 469 + [1 (Table des noms d’auteurs)] + [1 (Errata)] pp. + 15 (including frontispiece) pls. This volume was issued in seven livraisons as follows (Hayek 1989: 90–91): **13–14**: (pp. [9]–80) June 1858; **15**: (pp. 81–256) June 1858; **16–17**: (pp. 257–336) November 1858; **18–19**: (pp. 337–[472]) 1858, probably even early 1859. The title page is dated 1858. The third page is entitled “*Voyage au Gabon. Histoire naturelle des insectes et des arachnides recueillis pendant un voyage fait au Gabon en 1856 et en 1857 par M. Henry C. Deyrolle sous les auspices de MM. le Comte de Mniszech et James Thomson précédée de l’histoire du voyage par M. James Thomson – Arachnides, par M. H. Lucas.*”

The first volume contains papers by nine authors, but most of them are by Thomson himself. His papers are mainly descriptions of new genera and species, but there is also an essay on Aristotle’s entomology and a long reply to Guérin-Méneville’s accusation of plagiarism (published in *Revue et Magazin de Zoologie* (2) 9: 565–581) by Thomson. The second volume is devoted to an account of an expedition to Gabon in 1856 and 1857, undertaken by Deyrolle under the auspices of Count Mniszech and Thomson, and descriptions of the insects and other invertebrates collected there. The Coleoptera are on pages 29–256 and many new taxa are described by Thomson (pp. 29–239); the remaining pages (240–256) constitute a “Supplément aux Coléoptères” written by Louis Alexandre August Chevrolat where one new genus and several new species are described.

The two volumes were published with black and white or colour plates. According to Hayek (1989: 89) the second volume was also issued as a limited edition of 30 copies under the title “*Voyage au Gabon*” (*n.v.*).

**1859**. *Arcana naturae ou recueil d’histoire naturelle.* J.B. Baillière et fils, Paris. 132 pp. + 13 pls. (Folio) CNC

This work was issued in three livraisons: **1**: (pp. 1–64 + 4 pls) 5 November 1859 (*Bibl Fr*), December 1859 (*Allg Bibl*); **2**: (pp. 65–120) end of 1859, probably early 1860 (Hayek 1989: 93); **3**: (pp. 121–132) end of 1859, probably early 1860 (Hayek 1989: 93). The title page is dated 1859 and the *avertissement* 1 January 1859. The work came with colored or uncolored plates. Based on the entry in the *Bibliographie de la France*, it appears that the title on the first livraison was “*Arcana naturae, ou archives d’histoire naturelle; recueil scientifique de l’empire français, destiné à faciliter aux savants de tous les pays le moyen de publier leurs travaux ou observations sur diverses branches des sciences qui se rapportent à l’étude de la nature.*”

The volume includes the following papers: *Essai synoptique sur la sous-tribu des scarabaeitae vrais* (pp. 3–22 + pl. 1) by J. Thomson; *Mémoire sur les amibes a corps nu ou amibes proprement dites* (pp. 23–34 + pls 2–3) by H. Nicolet; [Description de *Mitracephala humboldtii* Thomson] (p. 34) by J. Thomson; *Description d’une espèce nouvelle d’oiseau* (pp. 35–36 + pl. 4) par Jules Verreaux; *Monographie du genre Psalidognathus de la division des Prionitae (Cerambycidae)* (pp. 37–44) par J. Thomson; *Notice monographique sur un genre nouveau de coléoptères de la famille des cérambycides (longicornes)* (pp. 45–49 + pl. 5) by Lucien Buquet; *Description d’un genre nouveau établi aux dépens de plusieurs espèces de Rhopalophora de Dejean* (pp. 50–54 + pl. 5) by A. Chevrolat; *Description de deux nouvelles espèces du genre Cycnoderus (Serville)* (pp. 55–56) by A. Chevrolat; *Essai monographique sur le genre Rhopalophora* (pp. 55–64) by A. Chevrolat; *Monographie du genre Batocera de la famille des Cerambycidae* (pp. 65–84 + pls 6–8) by J. Thomson; *Notice historique sur le genre Cicindela suivie de la description de sept espèces nouvelles de Cicindelidae* (pp. 85–92) by J. Thomson; *Description de deux espèces nouvelles de Carabidae* (pp. 93–94 + pl. 5) by J. Thomson; *Observations sur plusieurs genres de Cerambycidae* (pp. 95–96) by J. Thomson; *Revue du genre Taeniotes de la famille des Cerambycidae* (pp. 96–99) by J. Thomson; *Description d’un genre nouveau de coléoptères de la famille des cérambycides (longicornes)* (pp. 99–100) by Lucien Buquet; *Monographie du genre Spheniscus de la famille des Tenebrionidae* (pp. 101–113 + pls 10–11) by J. Thomson; *Insectes de la région du Nil Blanc* (pp. 114–120 + pl. 9) by J. Thomson; *Descriptions of some genera and species of Coleoptera from the vicinity of the southern boundary of the United States of America* (pp. 121–128 + pls 12–13) by John L. LeConte; *Description de deux curculionides* (p. 129) by J. Thomson.

**1860–1861**. *Essai d’une classification de la famille des cérambycides et matériaux pour servir à une monographie de cette famille.* Paris. xvi + 404 pp. + 3 pls. (8vo) CNC, GB

This volume was issued in four livaisons: **1**: (pp. xvi + 1–128) 1860 (title page; dedication dated 1 May 1860); **2–4**: (pp. 129–396) 1861 (colophon on page 396; *Soc Ent Fr* [*Bulletin bibliographique*, p. lxiii]), 28 January 1862 (*Ent Soc Lond*). In a paper published in August 1861, Bates (1861: 150) cited the correct page for Thomson’s treatment of the genus *Aegomorphus* on page 336 indicating that Thomson’s *classification*, at least up to that page, was published; probably livraisons 2–4 were issued by then.

Pages [397]–404 form the “Table méthodique” and “errata” and were missing in the two copies of the book I have seen although a photocopy of the pages was included in the CNC copy. These pages may have been published separately; the colophon at the end of page 404 is “1861.”

**1860–1868**. *Musée scientifique ou recueil d’histoire naturelle.* Paris. 96 pp. + 9 pls. (4to) MCZ, GB (pp. 5–88 only)

This book was published in three livraisons (Hayek 1989: 93–94) with the last three pages (pp. 93–96) published in 1868 (colophon on page 96). The dates of publication of the livraisons are: **1**: (pp. 5–40) 1860 (title page), 13 October 1860 (*Bibl Fr*); **2**: (pp. 41–72) 1860 (*Soc Ent Fr* [*Bulletin bibliographique*, p. cxxxii]), after 1 September 1860 (date on page 67); **3**: (pp. 73–92) 1861 (*Soc Ent Fr* [*Bulletin bibliographique*, p. lxiii]), after 1 July 1861 (date on p. 92).

The volume contains the following papers: *Monographie de la famille des nilionides* (pp. 5–14); *Agaocephalitarum synopsis* (pp. 14–19); *Lycanthropa novum genus* (p. 20); *Calophthalmus novum genus* (p. 20–21); *Evaniosomitarum enumeratio* (pp. 21–23); *Coléoptères nouveaux du Soudan par M. L. Reiche* (pp. 23–25); *Famille des trictenotomides* (p. 25–30); *Cétonides* (p. 30–38); *Supplément a l’essai synoptique sur la sous-tribu des scarabaeitae vrais publié dans l’Arcana naturae, 1859, p. 1* (pp. 39–41); *Revue du genre Therates de la famille des cicindélides* (pp. 41–45); *Supplément a la monographie de la famille des nilionides publiée dans cet ouvrage, page 1* (pp. 45–46); *Matériaux pour servir a une monographie nouvelle de la famille des clérides* (pp. 46–67); *Catalogue des paussides de la collection de M. James Thomson* (pp. 67 [dated 1 September 1860]–72); *Monographie de la famille des parandrides* (pp. 73–87); *Avis aux entomologistes* (p. 88 [dated 15 June 1861]); *Réponse a M. A. Chevrolat* (p. 89 [dated 1 July 1861]–92); *Monographie du genre* Mormolyce (N.B. – le tirage à part de ce travail a paru le 10 aout 1862) (pp. 93–95); *Note rectificative et corrections* (p. 95–96); *Table des matières* (p. 96). All the papers are authored by Thomson except for one by Reiche. The work came with colored or uncolored plates.

**1862**. *Monographie du genre* Mormolyce. Paris. 3 pp. (8vo) [DP: 10 August 1862 (Thomson 1862: xxxiv)] (*n.v.*).

Deyrolle (1862: xxxiv) mentioned that this booklet bears the date of 10 June 1862 which he believed was incorrect; in the same meeting Thomson (1862: xxxiv) indicated that a typographic error occurred and that the correct date of publication was 10 August 1862. This work was reprinted in 1868 in *Musée scientifique ou recueil d’histoire naturelle* (pp. 93–95). Two new species are described, *Mormolyce blattoides* and *Mormolyce castelnaudii*; the last one was described under the same name earlier in 1862 by Deyrolle in the *Annales de la Société Entomologique de France* (4e Série) 2: 314.

**1864–1865**. *Systema cerambycidarum ou exposé de tous les genres compris dans la famille des cérambycides et familles limitrophes.* H. Dessain, Liége. 578 pp. (8vo) CNC, GB

This work was issued in four livraisons: **1–2**: (pp. 1–272) 1864 (Dallas 1865: 336; Reinwald 1865: 211), September–October 1864 (*Bibl Belg*), January 1865 (*Allg Bibl*); **3**: (pp. 273–352) 1864 (Dallas 1865: 336), March–April 1865 (*Allg Bibl*), April–May 1865 (*Bibl Belg*); **4**: (pp. 353–538 + [2]) 1865 (Dallas 1866: 292). Page 541 is entitled “*Diagnoses d’espèces nouvelles qui seront décrites dans l’appendix du Systema cerambycidarum*” and dated April 1865. This section (pp. 541–578) was likely published separately from the main text. Although often listed in bibliographies as issued in volume 19 of the *Mémoires de la Société Royale des Sciences de Liège*, the section was not included in the *Mémoires*. The title page of the book is dated 1864 and the dedication 1 January 1864.

The work was also published (except pages 541–578) in *Mémoires de la Société Royale des Sciences de Liège* 19: 1–538 [DP: 1866 (title page), October–November 1866 (*Bibl Belg*)].

**1867–1868**. *Physis: recueil d’histoire naturelle.* Société Entomologique de France, Paris. (8vo) MCZ, BHL

*Tome premier.* 170 + [2] pp. [DP: 1867 (title page)]. This volume was published in three parts: **1**: (pp. 5–84) 1 August 1867 (p. 5), 12 October 1867 (*Bibl Fr*); **2**: (pp. 85–132) 1 November 1867 (p. 85); **3**: (pp. 133–170 + [2]) 1 December 1867 (p. 133). The volume includes: *I. D’une classification nouvelle de la famille des cérambycides (insectes coléoptères)* (pp;. 5–10); *II. Révision de la sous-tribu des dorcadionites (famille des cérambycides, insectes coléoptères)* (pp. 10–84); *III. Description d’un coléoptère* [*Chelonarium le contei*] (p. 84); *IV. Révision du groupe des mallodonites (insectes coléoptères, prionites, cérambycides)* (pp. 85–106); *V. Révision des parandrides (insectes coléoptères)* (pp. 106–118); *VI. Supplément a la révision de la sous-tribu des dorcadionites. (p. 10)* (pp. 118–128); *VII. Description de deux coléoptères* [*Hyporhagus bonvouloirii* and *Enotes lacordairei*] (pp. 129–130); *VIII. Spheniscorum monographiae appendix* (pp. 130–132); *IX. Ibidionitarum species novae* (pp. 133–163); *X. Description d’un coléoptère* [*Purpuricenus deyrollei*] (p. 164); Table alphabétique des coupes génériques et spécifiques comprises dans ce volume (pp. 165–170); Table méthodique des articles compris dans ce volume (p. [171]); Errata (p. [172]).

*Tome deuxième.* 208 pp. [DP: 1868 (title page)]. This volume was published in three parts: **4**: (pp. 5–40) 15 May 1868 (p. 5); **5**: (pp. 41–100) 1 August 1868 (p. 41); **6**: (pp. 101–208) 15 October 1868 (p. 101), 27 March 1869 (*Bibl Fr*). It includes: *XI. Exposé du bain des anciens ou bain a base de calorique sec considéré avec l’adjonction de l’hydropathie, des exercises du corps et de l’hygiène, comme un moyen de régénération pour l’homme* (pp. 5–40); *XII. Révision du groupe des oncidérites (lamites, cérambycides, coléoptères)* (pp. 41–92); *XIII. Révision du groupe des aereneites (lamites, cérambycides, coléoptères)* (pp. 92–98); *XIV. Toeniotitarum gen. speciesque nova* (pp. 99–100); *XV. Matériaux pour servir a une révision des desmiphorites (lamites, cérambycides, coléoptères)* (pp. 101–146); *XVI. Matériaux pour servir a une révision des lamites (cérambycides, col.)* (pp. 146–200); *XVII. Note rectificative* (p. 201); Table alphabétique des coupes génériques et spécifiques comprises dans ce volume (pp. 202–208); Table méthodique des articles compris dans ce volume (p. 208); Errata (p. 208).

The third volume, 1868–1874, is devoted to a phylosophical essay entitled “*Questions de science absolue*” and has no entomological content.

**1878**. *Typi buprestidarum musaei Thomsoniani.* Émile Deyrolle, Paris. 103 + 4 [Oeuvres de M.J. Thomson] pp. (8vo) [DP: 1878 (title page; preface dated 1 January 1878), 15 February 1878 (*Pet Nouv Ent*), 13 March 1878 (*Soc Ent Fr*), 6 April 1878 (*Bibl Fr*), 11 May 1878 (*Lit Centr Deutsch*), January–June 1878 (*Bibliot Hist-Nat*), 30 September 1878 (*Zool Anz*)] CNC, GB

The title on the cover reads “*Typi buprestidarum musaei Thomsoniani. Tiré à cent exemplaires seulement.*”

**1878**. *Typi cetonidarum suivis de typi monommidarum et de typi nilionidarum musaei Thomsoniani.* Émile Deyrolle, Paris. 44 pp. (8vo) [DP: 1878 (title page; *préface* dated 1 March 1878), 1 June 1878 (*Pet Nouv Ent*), 12 June 1878 (*Soc Ent Fr*), 29 June 1878 (*Bibl Fr*), January–June 1878 (*Bibliot Hist-Nat*), 30 September 1878 (*Zool Anz*)] NAL (ph)

**1879**. *Typi buprestidarum musaei Thomsoniani. Appendix I^a^.* Émile Deyrolle, Paris. 87 pp. (8vo) [DP: 1879 (title page; table méthodique on p. 83 dated 1 May 1879), 11 June 1879 (*Soc Ent Fr*), 15 June 1879 (*Naturaliste*), 9 August 1879 (*Bibl Fr*), August 1879 (*Nat Nov*), 1 November 1879 (*Lit Centr Deutsch*), 17 November 1879 (*Zool Anz*), December 1879 (*Polybiblion*)] CNC, GB

#### Thon, Theodor C. G. (Eisenach, Thuringia, Germany: 14 May 1792 – 17 November 1838: Jena, Thuringia, Germany). German naturalist, mineralogist and stenographer; lecturer and later professor at the University of Jena; established the journal *Entomologisches Archiv* of which two volumes were issued, 1827–1830. Reference. Mitzschke (1908).

**1826**. *Abbildungen auslaendischer Insecten. 1. Abtheilung, Kaefer. Taf. / Icones insectorum exoticorum. Sectio I. Coleoptera. Tab.* Croeker, Jena. [4] + 4 pp. + 1 pl. (4to) [DP: 1826 (title page; p. [2] dated August 1826), May 1827 (*Isis*, Heft V: [2])] YB (ph)

Eiselt (1836: 183) mentioned that this work was published 1827–1828 and contains six plates. Hagen (1863: 219) listed six plates issued 1826–1828 although he had seen but a single plate with four pages of text. I have not been able to confirm that Thon’s work includes six plates. I have seen a single plate and found no review or record but for the first plate. The text is written in German and Latin.

Five Brazilian species of the genus *Cassida* are described in the text and illustrated on the plate: *Cassida gibbosa* Fabricius (p. 1), *Cassida assimilis* Sturm (p. 1), *Cassida decemguttata* Sturm (p. 2), *Cassida platynota* Germar (p. 3) and *Cassida oblonga* Sturm (p. 4). Except for *Cassida gibbosa* Fabricius and *Cassida platynota* Germar, the other three species are described for the first time. Authorship for these three species should be attributed to Thon since there is no indication in the work that Sturm was responsible for both the name and for satisfying the criteria of availability (see ICZN 1999, Article 50.1.1). These three species are not included in Sherborn’s *Index Animalium*.

**1838**. [Thon, T. and Reichenbach, A.B.] *Die Insekten, Krebs- und Spinnenthiere, mit besonderer Berücksichtigung der in Deutschland lebenden, dargestellt in getreuen Abbildungen und mit ausführlicher Beschreibung. Mit 131 Tafeln, mehr als 3000 Abbildungen enthaltend*. Eduard Eisenach, Leipzig. 482 + [9] pp. + 131 pls. (8vo) [DP: 1838 (title page), 17 August 1838 (*Allg Bibl Deutsch*), August 1838 (*Intell Jena Lit Ztg*)] CNC (mf), GB

This work was also issued in 26 *Hefte*, 1835–1838, under the title “*Die Naturgeschichte in getreuen Abbildungen und mit ausführlicher Beschreibung derselben*. *Insekten.*” I have seen notices for the following *Hefte*: **1–2**: (pp. 1–32 + 12 pls) 1835 (Anonymous 1836: 127); **3–4**: (pp. 33–64 + 12 pls) 1835 (Anonymous 1836: 127), 27 January 1836 (*Lit Ztg*), 29 January 1836 (*Allg Bibl Deutsch*); **5–6**: (pp. 65–96 + 12 pls) 13 May 1836 (*Allg Bibl Deutsch*); **7–8**: (pp. 97–128 + 12 pls) 24 June 1836 (*Allg Bibl Deutsch*); **11–12**: (pp. ?177–224) 16 November 1836 (*Lit Ztg*); **13–14**: (pp. 225–256 + 11 pls) 4 January 1837 (*Lit Ztg*), 6 January 1837 (*Allg Bibl Deutsch*); **17–18**: 5 July 1837 (*Lit Ztg*); **23–24**: 17 January 1838 (*Lit Ztg*); **25–26**: 8 August 1838 (*Lit Ztg*).

The Coleoptera are on pages 265–422.

#### Thunberg, Carl Peter (Jönköping, Sweden: 11 November 1743 – 8 August 1828: Uppsala, Sweden). Swedish naturalist and explorer, sometimes called “the father of South African botany” or the “Japanese Linnaeus;” pupil of Linnaeus in Uppsala; left Sweden for Paris in 1770 to continue his studies in medicine and natural history; visited the Dutch colonies and Japan to collect specimens for Dutch botanical gardens (1771–1778); professor of medicine and botany at the University of Uppsala; his collections are at the Universitets Zoologiska Institut in Uppsala. References. Anonymous (1829b); Mohnike (1831: 1–56); Hoefer (1866a: 315–317); Muller and Rookmaaker (1992); Adler (2007: 30–31, P); Hansen et al. (2012: 47–49, P).

**1781–1791**. *Dissertatio entomologica novas insectorum species, sistens.* Johann Edman, Upsaliae [= Uppsala]. (4to) MCZ, BHL

*Cujus partem primam, cons. exper. facult. med. Upsal., publice ventilandam exhibent praeses Carol. P. Thunberg, et respondens Samuel Nicol. Casström, stipendiarius Regius. Uplandus. In audit. gust. maj. D. XV Dec. anno MDCCLXXXI. Horis solitis.* 28 pp. + 1 pl. [DP: 15 December 1781 (cover)]. Hagen (1863: 220) mentioned that this part was also issued in 1783 in *Acta Medicorum Suecicorum* 1: 261–288.

*Cujus partem secundam, cons. exper. facult. med. Upsal., publice ventilandam exhibent praeses Carol. P. Thunberg, et respondens Johannes M. Ekelund, Fjerdhundrensis. In audit. gust. maj. D. Maji anno MDCCLXXXIII. Horis solitis.* [1] + pp. 29–52 + pl. 2 [DP: 23 April 1783 (title page of the 1801 issue; Horn and Schenkling 1929: 1226)].

*Cujus partem tertiam, cons. exper. facult. med. Upsal., publice ventilandam exhibent praeses Carol. P. Thunberg, et respondens David Lundahl, Smolandus. In audit. gust. maj. D. XXV. Maji anno MDCCLXXXIV. Horis solitis.* Pp. 53–68 + [1 (Corrigenda)] + pl. 3 [DP: 25 May 1784 (cover)].

*Cujus partem qvartam, cons. exper. facult. med. Upsal., publice ventilandam exhibent praeses Carol. P. Thunberg, et respondens Carolus P. Engström. Stipendiarius Regius Uplandus. In audit. gust. maj. D. XXVI Maji anno MDCCLXXXIV. Horis solitis.* Pp. 69–84 + pl. 4 [DP: 26 May 1784 (cover)].

*Cujus partem quintam, cons. exper. facult. med. Upsal. praeside Carol. P. Thunberg. Publico examini subjicit Johannes Olai Noraeus, Uplandus. In audit. gust. maj. D. XIII. Jun. MDCCLXXXIX. H.a.m.s.* [1] + pp. 85–106 + pl. 5 [DP: 13 June 1789 (cover)].

*Cujus partem sextam consensu exp. fac. med. Upsal. praeside Car. Petr. Thunberg. Publico examini subjicit Andreas Johannes Lagus, Fenno. In audit. hort. bot. D. I Junii MDCCXCI. H.a.m.s.* [3] + pp. 107–130 + pl. 6 [DP: 28 May 1791 (title page of the 1801 issue)].

All these dissertations, except the second one, pertain to Coleoptera. The dissertations were also issued in 1801 in Göttingen under the title “*Dissertationes academicae Vpsaliae habitae svb praesidio Carol. Petr. Thvnberg. Volvmen tertivm. Cvm tab. XII. aeneis.*” [GB].

**1784–1795**. *D.D. Dissertatio entomologica sistens insecta Svecica.* Joh. Fred. Edman, Upsaliae [= Uppsala]. (4to) CNC, BHL

*Quorum partem quintam. Cons. exp. facult. med. Ups. praeside Carol. Pet. Thunberg. Publico examini subjicit Isaacus Haij, Stip. Kåhreanus Westrogoth. In audit. botanico die 10 Maji 1794. H.a.m.s.* [2] + pp. 63–72. [DP: 10 May 1794 (cover)].

*Quorum partem sextam venia exp. ord. med. Ups. praeside Carol. Pet. Thunberg. Publico examini offert Samuel Kinmanson, Stip. Hedmann. Ostro-Goth. In audit. botanico die 3 Dec. 1794. H.a.m.s.* [2] + pp. 73–81. [DP: 3 December 1794 (cover)].

*Quorum partem octavam, venia exp. ord. med. Upsal. praeside Carol. Pet. Thunberg. Publico examini subjicit Jonas Kullberg, Stip. Victor. Vestro-Goth. In audit. botanico die 15 Dec. 1794. H.a.m.s.* [2] + pp. 99–104. [DP: 13 December 1794 (title page of the 1801 issue)].

*Quorum partem nonam, venia exp. ord. med. Upsal. praeside Carol. Pet. Thunberg. Publico examini subjicit Steno Edvardus Westman, Stockholmiensis. In audit. botanico die 20 Maji 1795. H.a.m.c.* [2] + pp. 105–113. [DP: 20 May 1795 (cover)].

This work consists of nine academic dissertations written by Thunberg and defended by his pupils. Only those related to Coleoptera are listed. The dissertations were also issued in 1801 in Göttingen under the title “*Dissertationes academicae Vpsaliae habitae svb praesidio Carol. Petr. Thvnberg. Volvmen tertivm. Cvm tab. XII. aeneis.*” [GB].

**1787–1798**. *D.D. Museum naturalium Academiae Upsaliensis.* Johan. Edman [vols 3, 4, 23; Appendices 1–4, 6] / Excubebant Regiae Academiae Typographi [vols 29, 33] / Zeipel et Palmblad [Appendix 24], Upsaliae [= Uppsala]. (4to) MCZ

*Cujus partem tertiam. Consensu exp. fac. med. Upsal. praeside Carol. Pet. Thunberg. Publico examini proponit, And. Gustav. Ekeberg. Stipendiarius Sparrfeldianus, Uplandus. In audit. gust. maj. d. XXI. Junii. Anni MDCCLXXXVII. H.a.m.s.* [1] + pp. 33–42. [DP: 21 June 1787 (cover)].

*Cujus partem qvartam. Consensu exp. fac. med. Upsal. praeside Carol. Pet. Thunberg. Publico examini subjicit, Petrus a Bjerkén, Stip. Leijell. Angermannus. In audit. gust. maj. d. XIX Dec. MDCCLXXXVII. H.a.m.s.* [1] + pp. 43–58 + 1 pl. [DP: 19 December 1787 (cover)].

*Cujus partem XXIII. Venia exp. fac. medicae Upsal. praeside Carol. Pet. Thunberg. Publicae defert censurae Jacobus Wilhelmus Rudolphi Nycopia Sudermannus. In audit. botanico die XXIII Maji MDCCCIV. H.a.m.s.* 11 pp. [DP: 23 May 1804 (cover)].

*Cujus partem XXIX. Nonnullis adjectis aphorismis. Venia exp. fac. med. Upsal. praeside C. P. Thunberg. P. P. Zacharias Sjöström. Gestricio-Helsingus. In auditor. botanico d. XXVI Maji MDCCCXIX. H.a.m.s.* [2] + 8 pp. [DP: 26 May 1819 (cover)].

*Cujus partem XXXIII, ultimam venia exp. fac. med. Upsal. praeside C. P. Thunberg. P. P. Canutus Ludovicus Altahr Vermel. In audit. botanico d. XXIII Maji MDCCCXXI. H.p.m.s.* [2] + 7 pp. [DP: 23 May 1821 (cover)].

*Appendix I. Quam consensu exp. fac. med. Upsal. praeside Carol. Pet. Thunberg. Publico examini proponit Jonas Lundelius, Smolandus. In audit. gust. maj. d. 9 Febr. 1791. Horis ante merid. solitis.* [1] + pp. 111–121. [DP: 9 February 1791 (cover)].

*Appendix II. Quam consensu exp. fac. med. Upsal. praeside Carol. Pet. Thunberg. Publico examini proponit Hans Yman, Smolandus. In audit. gust. maj. d. April 1791. Horis ante merid. solitis.* [1] + pp. 123–129. [DP: April 1791 (cover)].

*Appendix III. Quam venia exp. ord. med. Ups. praeside Carol. Pet. Thunberg. Publico examini subjicit Petrus J. Aspelin, Smolandus. In audit. botanico die 18 Dec. 1794. H.a.m.s.* pp. 131–143. [DP: 18 December 1794 (cover)].

*Cujus Appendic. IV. Venia exp. ord. med. Upsal. praeside Carol. Pet. Thunberg. Proponit examini subjicit Petrus Sundberg, Stip. Strandb. Ostrogothus. In audit. botan. d. XXIII. Nov. MDCCXCVI. H.a.m.c.* pp. 145–150. [DP: 23 November 1796 (cover)].

*Cujus Appendix. VI. Venia exp. ord. med. Upsal. praeside Carol. Pet. Thunberg. Proponit Johannes Ericus Forsström, Dalekarlus. In audit. botan. d. XXI Junii MDCCXCVIII. H.a.m.s.* [3] + 111–117. [DP: 21 June 1798 (cover)].

*Cujus Append. XXIV. Venia exp. fac. med. Upsal. praeside Carol. P. Thunberg. Proponit Olavus Sjöstrand Smolandus. In audit. botanico d. VIII Jun. MDCCCXVIII. H.a.m.s.* [2] + 30–36. [DP: 8 June 1818 (cover)].

This work is a catalogue of plants and animals in the collection of the Museum in Uppsala serving as dissertations to Thunberg’s students. It was divided in two series: the Museum itself appearing in 33 parts, published 1787–1821, and its Appendix issued in 26 parts, published 1791–1819, with two further unnumbered parts added in 1827 (Hagen 1857a: 202–204; Rookmaaker 1989: 151–152). Only dissertations with Coleoptera contents are listed.

**1789**. *Periculum entomologicum, quo characteres generum insectorum, consensu exper. fac. med. Upsal. praeside Carol. Pet. Thunberg. Publicae censurae proponit Samuel Törner, Norcopia-Gothus. In audit. gustav. maj. D. X Jun. MDCCLXXXIX. Horis consvetis.* Joh. Edman, Upsaliae [= Uppsala]. 16 pp. (8vo) [DP: 10 June 1789 (cover)] BHL

The dissertation was also issued in Göttingen, 1801, in “*Dissertationes academicae Vpsaliae habitae svb praesidio Carol. Petr. Thunberg. Volvmen tertivm. Cvm tab. XII. aeneis*” on pages 249–263 [GB].

A Swedish translation was published in 1793 in Uppsala under the title “*Characteres generum insectorum. Kånneteken på insect-slågterne. Under Herr Prof. och Rid. Doct. Carl Pet. Thunbergs presidio utgifne 1788, af Samuel To̊rner*” [SDL].

**1821**. *D.D. Opatum insecti genus. Quod venia exp. fac. med. Upsal. praeside C.P. Thunberg. P.P. Axelius Loffman Vermel. In audit. botanico d. IX Jun. MDCCCXXI. h.p.m.s.* Upsaliae [= Uppsala]. [4] + pp. 27–34. (4to) [DP: 9 June 1821 (cover)] GB, CNC (mf)

The genus dealt with is *Opatrum* of the family Tenebrionidae. The generic name is mispelled in the title.

#### Tigny, F. Martin Grostête T. de (Orléans, Loiret, France: 3 September 1736 – 1 May 1799). French naturalist; served for many years in one of the companies of the “maison du roi;” later, “trésorier de France.” Reference. Walckenaer (1830: 157–158).

**1801**. *Histoire naturelle des insectes, composée d’après Réaumur, Geoffroy, Degeer, Roesel, Linnée, Fabricius, et les meilleurs ouvrages qui ont paru sur cette partie; rédigée suivant la méthode d’Olivier; avec des notes, plusieurs observations nouvelles, et des figures dessinées d’après nature.* Deterville, Paris. (18mo) [DP: “An X” (title page), 23 September–22 October 1801 (*J Lit Fr*), 26 December 1801 (*Gött Anz*), January 1802 (*Esprit J*)] BHL

*Tome V.* 336 pp. + 7 pls.

*Tome VI.* 331 pp. + 7 pls.

*Tome VII.* 320 pp. + 10 pls.

*Tome VIII.* 319 pp. + 9 pls.

*Tome IX.* 303 pp. + 7 pls.

The entire work was published in ten volumes, issued simultaneously in 1801. These volumes are often dated from 1802 (Hagen 1863: 223; Horn and Schenkling 1929: 1231) but they were published by the end of 1801. The Coleoptera are in volumes 5 (pp. 120–336), 6, 7, 8 and 9 (pp. 1–58). A second edition (*n.v.*), differing from the first merely in new title pages (Sherborn 1922a: cxxiii), was issued also in ten volumes in 1813. A “*troisième édition, revue, augmentée et mise au niveau des connaissances actuelles par M. F.E. Guérin*” [BHL] was issued in 1828 and recorded on 3 May 1828 (*Bibl Fr*). In this edition, the Coleoptera are in volumes 2 (pp. 121–363), 3, 4 and 5. The third edition was reissued in 1830 [GB].

An Italian translation by D.A. Farini was issued by fascicles under the title “*Storia naturale degli insetti ricavata dalle opere migliori intorno a’ medesimi, e da quelle specialmente di Linneo, Fabricius, Réaumur, Geoffroy, Dègeer e Reasel, redatta giusta il metodo di Olivier, con note, molte nuove osservazioni, e con disegni tratti dal naturale*,” in three volumes, 1832–1835 (*n.v.*).

According to Walckenaer (1830: 158), this work was written by de Tigny’s wife but was published under her husband name apparently because the name of a woman could have a negative impact on the sale of a scientific book [“sans doute, que le nom d’une femme pouvait nuire au débit d’un livre scientifique”].

#### Tournier, Henri (26 September 1834 – 27 August 1904). Wealthy Swiss entomologist interested in Palaearctic Coleoptera and Hymenoptera; merchant in Peney-le-Jorat (now Jorat-Menthue) in Switzerland; his rich collection of Coleoptera was sold to Maurice Pic [*q.v.*]. Reference. Cambefort (2006: 302).

**1868**. *Description des dascillides du Bassin du Léman.* Georg, Bâle et Genève [&] F. Savy, Paris. 95 + [1] pp. + 4 pls. (8vo) [DP: 1868 (title page), 2 November 1868 (*Ent Soc Lond*), July–December 1868 (*Bibliot Hist-Nat*)] CMLE, GB, MDZ

This work was published by the *Association Zoologique du Léman*.

#### Townson, Robert (Richmond, Surrey, United Kingdom: 4 April 1762 – 27 June 1827: Varroville, New South Wales, Australia). British scholar, scientist, and settler; travelled extensively in Europe, collaborating with professors at the Universities he visited; landed in Sydney, Australia, in 1807 with the approval of the British government but soon got confronted with the hot-tempered governor William Bligh, former commander of H.M.S. *Bounty*, over his grant of land. References. Goodin (1967); Torrens (1999, P; 2004).

**1797**. *Travels in Hungary, with a short account of Vienna in the year 1793. Illustrated with a map and sixteen other copper-plates.* G.G. and J. Robinson, London. xviii + [1] + 506 pp. + 16 pls. (4to) [DP: 1797 (title page), March 1797 (*Monthly Mag*), May 1797 (*Crit Rev*), July 1797 (*European Mag*), August 1797 (*British Critic*)] HOU, GB

Pages 455–476 and plates 11 and 12 form the “*Appendix. Entomologia*” which deal only with Coleoptera. A French translation was published in Paris in three volumes, an 7 of the French Revolution [= 22 September 1798–22 September 1799], under the title “*Voyage en Hongrie: précédé d’une description de la ville de Vienne et des jardins impériaux de Schoenbrun*” [GB].

Merkl (1998) gave a brief account of the new species of Coleoptera described in Townson’s book.

#### Tristán, José Fidel (San José, Costa Rica: 6 September 1874 – 23 January 1932: San José, Costa Rica). Costa Rican educator, entomologist and archaeologist; professor and director at the Colegio Superior de Señoritas (1908–1921) and at the Liceo de Costa Rica (1921–1930); director of the Museo Nacional de Costa Rica (1930–1932). References. Calvert (1932, P); Garro (2016, P).

**1897**. *Insectos de Costa Rica: pequeña colección arreglada.* Tipografia Nacional, San José. 21 pp. (8vo) [DP: 1897 (title page; colophon dated 22 December 1896)] BDH

This is an annotated list of insects from Costa Rica based on a small collection sorted out by the author; 108 species of Coleoptera are included on pages 3–13.

#### Trost, Patriz (Berching, Bavaria, Germany: 27 October 1763 – ?). Canon in Eichstätt, Bavaria.

**1801**. *Kleiner Beytrag zur Entomologie in einem Verzeichnisse der Eichstettischen bekannten und neuentdekten Insekten mit Anmerkungen für Kenner und Liebhaber. Erster Heft.* Johann Jakob Palm, Erlangen. viii + 71 pp. (8vo) [DP: 1801 (title page; *Vorrede* dated 1 January 1801), 26 August 1801 (*Allg Lit Ztg*), 14 November 1801 (*Gött Anz*)] MCZ, GB, GDZ

No other parts of this work were published. The Coleoptera are on pages 1–43. Several new species are described.

#### Truqui, Eugen [Eugene, Eugenio] (? – April 1860: Rio de Janeiro, Brazil). Sardinian vice-consul at Cyprus and consul-general in Rio de Janeiro; his insect collections from Brazil, Syria and Cyprus are at the Museo Regionale di Scienze Naturali in Turin.

**1847**. *Amphicoma et Eulasia insectorum coleopterorum genera. Monographice disserta.* Sodales, Taurini [= Turin]. 48 pp. + 3 pls. (4to) [DP: 1847 (title page; preliminaries dated “14 ante cal. Novembris” 1847), 27 September 1848 (*Soc Ent Fr*)] AMNH

The content of this booklet was also issued in 1848 in *Studi Entomologici* 1: 1–48.

#### Turra, Antonio (Vicenza, Veneto, Italy: 25 March 1730 – 6 September 1796: Vicenza, Italy). Italian botanist and practicing physician in Vicenza. References. Baseggio (1845); Stafleu and Cowan (1986: 551).

**1780**. *Florae Italicae prodromus.* Ex Officina Turraeana, Vicentiae. 68 + 16 pp. (8vo) [DP: 1780 (title page; preliminaries in the second part dated 10 October 1780)] MDZ

There is no title page for the second section of 16 pages. The first two pages of the section form the introduction; the next page has the title “Insecta Vicetina.” The section consists of a list of insects from Vicenza in northeast Italy; the Coleoptera are on pages 3–7.

#### Turton, William (Olveston, Gloucestershire, United Kingdom: 21 May 1762 – 28 December 1835: Bideford, Devon, United Kingdom). British physician and naturalist, particularly interested in conchology; practiced medicine in Swansea, Dublin, Torquay and Bideford. References. Stafleu and Cowan (1986: 553); Damkaer (2002: 143–144); Woodward (2004c).

[**1800**]. *A general system of nature, through the three grand kingdoms of animals, vegetables and minerals: systematically divided into their several classes, orders, genera, species, and varieties, with their habitations, manners, economy, structure and peculiarities. Translated from Gmelin’s last edition of the celebrated Systema Naturae, by Sir Charles Linné: amended and enlarged by the improvements and discoveries of later naturalists and societies, with appropriate copper-plates. Vol. II.* Lackington, Allen, and Co., London. 717 + [2 (Errata)] pp. + 1 pl. (8vo) [DP: 1800 (title page)] GB

The entire work was published in four volumes. Jenyns (1835: xxxi) noted that all four volumes were issued in 1800 but Evenhuis (1997b: 780) noted that the colophon of the third volume (*n.v.*) is dated 1801 which leaves doubt that the series was indeed published in 1800. Copies of all four volumes with the date 1802 on their title pages are known [BHL] and *The Critical Review* published a notice of all four volumes in July 1802. The Coleoptera are on pages 12–523 of the second volume.

A second edition was published in London, 1806, in seven volumes under the title “*A general system of nature, through the three grand kingdoms of animals, vegetables, and minerals, systematically divided into their several classes, orders, genera, species, and varieties, with their habitations, manners, economy, structure, and peculiarities. By Sir Charles Linné: translated from Gmelin, Fabricius, Willdenow, &c. Together with various modern arrangements and corrections, derived from the Transactions of the Linnean and other societies, as well as from the classical works of Shaw, Thornton, Abbot, Donovan, Sowerby, Latham, Dillwyn, Lewin, Martyn, Andrews, Lambert, &c.&c. with a life of Linné, appropriate copper-plates, and a dictionary explanatory of the terms which occur in the several departments of natural history. In seven volumes*” [McG, GB]. The first four volumes are essentially reprints of the original edition (Evenhuis 1997b: 782); the additional three volumes treat the vegetable and mineral kingdoms.

#### Ulke, Henry (Ząbkowice Sląskie, Poland: 25 January 1821 – 18 February 1910: Washington DC, USA). American (Prussian-born) portrait painter by profession, musician, and naturalist; immigrated to the United States and settled first in New York, where he hold a number of jobs, and then in Washington DC, in 1857, where he opened a photographic studio; commissioned to paint cabinet officers, including presidents, and got the surname of “painter of presidents;” collected extensively for nearly 30 years, mainly in eastern United State and eastern Canada; sold his collection to the Carnegie Museum in Pittsburgh. References. Mallis (1971: 258–260); Spilman (1984: 4–6, P).

**1875**. Chapter XI. Report upon the collections of Coleoptera made in portions of Nevada, Utah, California, Colorado, New Mexico, and Arizona, during the years 1871, 1872, 1873, and 1874. Pp. 809–827 + pl. 41 *in*: *Report upon geographical and geological explorations and surveys west of the one hundredth meridian, in charge of First Lieut. Geo. M. Wheeler, corps of engineers, U.S. Army, under the direction of Brig. Gen. A.A. Humphreys, chief of engineers, U.S. Army. Published by authority of Hon. Wm. W. Belknap, Secretary of war, in accordance with acts of Congress of June 23, 1874, and February 15, 1875. In six volumes, accompanied by one topographical and one geological atlas. Vol. V.– Zoology.* Engineer Department, United States Army, Washington [DC]. 1019 + [1] pp. + 45 pls. (4to) [DP: 1875 (title page)] CNC

#### Van den Branden, Constant

**1884**. *Catalogue des coléoptères carnassiers aquatiques (Haliplidae, Amphizoïdae, Pelobiidae & Dytiscidae).* P. Weissenbruch, Bruxelles. 118 pp. (8vo) [DP: 1884 (title page; preliminaries dated 2 June 1883), June 1884 (*Ent Nachr* 10: 187), July 1884 (*Nat Nov*)] GB

This work was also issued in *Annales de la Société Entomologique de Belgique* 29: 5–118 [DP: 1885 (title page), 13 May 1885 (*Soc Ent Fr*), May–June 1885 (*Soc R Belg Géog*), August 1885 (*Nederl Ent Ver*)].

#### Van der Stegen de Putte, Joseph François Philippe (Baron) (Brussels, Belgium: 17 September 1754 – 6 May 1799: Brussels, Belgium). Naturalist and deputy burgomaster in Brussels; professor of natural sciences at the École centrale du département de la Dyle in Brussels; founded the first botanical garden in Brussels. Reference. Morren (1849: 162).

**1792**. *Le guide du naturaliste dans les trois regnes de la nature, ou méthode analytique, par laquelle on peut découvrir le nom générique de l’animal, du végétal, ou du minéral, que l’on se propose de connoître.* Lemaire, Bruxelles. 515 + [1] pp. (8vo) [DP: 1792 (title page), 1 November 1792 (*J Hist Lit*)] GB

This work includes a key to the genera of Coleoptera (pp. 96–117).

[**1798–1799**]. *Cours d’histoire naturelle, contenant une distribution méthodique, facile, et en grande partie nouvelle, des trois règnes de la nature, en ordres et genres, avec leurs caractères distinctifs; ainsi que la détermination caractéristique, historique, économique, médicinale, etc., des espèces animales et végétales, indigènes au département de la Dyle (ci-devant Brabant), et des espèces étrangères les plus intéressantes ou les plus connues; enfin celle de toutes les productions du règne minéral; avec l’explication des termes techniques, et celle du système sexuel de Linné. Partie générique. Tome II.* Emmanuel Flon, Bruxelles. 416 pp. (8vo) [DP: “An VII” [= 22 September 1798–22 September 1799] (title page)] GB

The entire work was to be published in nine or ten volumes but only the first two were issued, in An VI (tome I) and VII (tome 2) of the French Revolution, due to the death of the author. They were issued by *cahiers* of four *feuilles* (Anonymous 1799a: 242). Date of publication and collation of each *cahier* are desired. The Coleoptera are on pages 331–360 of the second volume.

#### Vilanova y Piera, Juan (Valencia, Valencia, Spain: 5 May 1821 – 7 June 1893: Madrid, Spain). Spanish geologist, paleontologist and prehistorian; professor of natural history at the University of Oviedo; later, professor at the University of Madrid, teaching first natural history and later geology and paleontology. References. Aleixandre (1893); Gozalo Gutiérrez (1993, P).

**1875**. *La creacion. Historia natural escrita por una sociedad de naturalistas y publicada bajo la direccion del Doctor D. Juan Vilanova y Piera. Tomo VI. Articulados.* Montaner y Simón, Barcelona. xii + 656 pp. + 12 pls. (4to) [DP: 1875 (title page)] GB

The entire series consists of eight volumes, 1872–1876. The Coleoptera are in volume 6 on pages 1–88.

#### Villa, Antonio (Milan, Lombardy, Italy: 24 August 1806 – 26 June 1885: Milan, Italy). Italian government employee and naturalist; formed with his brother Giovanni Battista a small museum, particularly strong in shells, insects, minerals and fossils, which was destroyed. References. Cantù (1844: 164–165); Stoppani (1885); Conci (1975: 1043–1044).

**1833**. [Villa, A. and Villa, G.B.] *Coleoptera Europae dupleta in collectione Villa quae pro mutua commutatione offerri possunt.* Mediolani [= Milan]. 36 pp. (8vo) [DP: 1833 (title page), 2 July 1834 (*Soc Ent Fr*)] CMLE

The work consists of a checklist of European beetles from the Villa collection available for exchange (pp. 1–32), followed by the description of 44 new species in a section (pp. 32–36) entitled “*Coleopterorum species novae in catalogo dupletorum extantes. Diagnosibus, adumbrationibus atque observationibus illustratae.*”

**1835**. [Villa, A. and Villa, G.B.] *Supplementum Coleopterorum Europae dupletorum catalogo collectionis Villa idest species aliae, quae nunc pro mutua commutatione itidem offerri possunt; nec non emendationes aliquarum specierum in catalogo anni 1833 extantium.* Mediolani [= Milan]. Pp. 37–50. (8vo) [DP: 1835 (title page), 20 April 1836 (*Soc Ent Fr*), 2 May 1836 (*Ent Soc Lond*)] CMLE

The work consists of a supplement to the checklist of European beetles from the Villa collection available for exchange (pp. 37–47), followed by the description of 23 new species in a section (pp. 47–50) entitled “*Coleopterorum species novae in supplemento salutatae. Diagnosibus atque observationibus illustratae.*”

[**1839**]. [Villa, A. and Villa, G.B.] *Alterum supplementum Coleopterorum Europae sive additio ad Catalogum et Supplementum I dupletorum collectionis Villa continens species alias, nunc pro mutua commutatione adhuc offerrendas; nec non aliquarum specierum emendationes et synonima quae in Catalogo anni 1833 et Supplementum anni 1835 oblatae fuerunt.* Mediolani [= Milan]. Pp. 51–66. (8vo) [DP: 1838 (title page; *lectori* [p. 66] dated 30 December 1838), May 1839 (*Rev Zool*)] CMLE

Considering the date of the *lectori* it is doubtful that the booklet was issued in 1838 as listed on the title page. Sherborn (1922a: cxxvi) gave the date as “1838 (1839).”

**1844**. [Villa, A. and Villa, G.B.] Insetti della Lombardia. Pp. 406–478 *in*: *Notizie naturali e civili su la Lombardia. Vol. I.* Giuseppe Bernardoni, Milano. cxii + 491 + [1] pp. + 4 folded pls. (8vo) [DP: 1844 (title page)] GB

The Villas’ brothers contribution includes a “*Catalogo degli insetti Coleopteri della Lombardia*” (pp. 416–478); the reprint of the catalogue was offered on 11 December 1844 (*Soc Ent Fr*).

#### Villers, Charles Joseph de (Rennes, Ille-et-Vilaine, France: 24 July 1724 – 3 January 1810: Lyon, Rhône, France). French naturalist; professor of physics in Lyon; his collections were sold to a Mr. Tissier in Lyon and their whereabouts are unknown. Reference. Mulsant (1840).

**1789**. *Caroli Linnaei entomologia, Faunae Suecicae descriptionibus aucta; DD. Scopoli, Geoffroy, de Geer, Fabricii, Schrank, &c. speciebus vel in systemate non enumeratis, vel nuperrime detectis, vel speciebus Galliae australis locupletata, generum specierumque rariorum iconibus ornata. Curante & augente Carolo de Villers.* Piestre et Delamolliere, Lugduni [=Lyon]. (8vo–text; Folio–plates) CNC, BHL, GB

*Tomus primus.* [4] + xvi + 765 + [1 (*lectori benevolo*)] pp. + 3 pls. [DP: 1789 (title page), 22 January 1790 (*Allg Lit Ztg*), 13 March 1790 (*Gött Anz*)]. This volume includes the Coleoptera (pp. 10–427) and Hemiptera (pp. 428–567). It also has two other sections: “*Analytica comparataque D.D. Linnaei, De Geerii, Geoffroyi, Fabricii entomologiarum synopsis*” (pp. 568–627) and “*Nomenclator entomologus, insectorum systematis naturae genera, species Linn. antiquas recentioresque nuperrime detectas, necnon praecipuorum entomologorum synonymiam, ordine alphabetico indicans*” (pp. 629–765).

*Tomus quartus.* 556 + ccxiii pp. + 1 pl. [DP: 1789 (title page), 22 January 1790 (*Allg Lit Ztg*), 13 March 1790 (*Gött Anz*)]. This volume includes the Insecta aptera (pp. 1–198), and the following sections: “*Insectorum illustrationes*” (pp. 199–556, the Coleoptera being on pp. 199–373); “*Analytica comparataque D.D. Linnaei, De Geerii, Geoffroyi, Fabricii entomologiarum synopsis*” (pp. ii–xx); “*Tomi III Nomenclator entomologus, insectorum systematis naturae genera, species Linn. antiquas recentioresque nuperrime detectas, necnon praecipuorum entomologorum synonymiam, ordine alphabetico indicans*” (pp. xxi–cli); “*Tomi IV Nomenclator entomologus, insectorum systematis naturae genera, species Linn. antiquas recentioresque nuperrime detectas, necnon praecipuorum entomologorum synonymiam, ordine alphabetico indicans*” (pp. cliii–clxxxv); and “*Joh. Christ. Fabricii philosophiae entomologicae epitome*” (pp. clxxxvii–ccxiii).

The entire series consists of four volumes issued simultaneously in 1789. The Coleoptera are in the first and last volumes only.

#### Voet, Johann Eusebius (Dordrecht, South Holland, Netherlands: 24 January 1706 – 28 September 1788: The Hague, Netherlands). Dutch civil servant educated as a physician, illustrator and entomologist. Reference. Eijnatten (2003: 434).

**1806**. *Catalogus systematicus coleopterorum. Catalogue systématique des coléoptères. Systematische naamlijst van dat geslacht van insecten dat men torren noemt.* Bakhuysen, Haag [= The Hague]. (4to) CNC, BHL

*Tomus I.* [2] + 104 [the last 8 pages incorrectly numered 67–74] (Latin text) + 114 (French text) + 111 (Dutch text) pp. + 55 pls. [DP: 1806 (title page of some copies)].

*Tomus II.* 82 (Latin text) + 86 (French text) + 87 (Dutch text) + 20 (Index tabularum. Tom. I. and II) pp. + 50 pls. [DP: 1806 (title page)].

This work is written in Latin, French and Dutch. It was started by J.E. Voet and completed by Bakhuysen in 1806. According to Hagen (1857b: 407), Bakhuysen was responsible in the first volume for signatures M and N (pp. 89–104) of the three texts, plates 49–55, the final title page, and the dedication and introduction (first two unnumbered pages). In the second volume, he was responsible for signatures D to L (pp. 25–82 [Latin], 25–86 [French], 25–87 [Dutch]), plates 25–50, the title page, and the index. The section by Voet was issued much earlier, in 1766 according to Leske (1779: 412) and Engelmann (1846: 556)^103^, under the title “*Catalogue raisonné ou systématique du genre des insectes, qu’on appelle Coleoptrées*” (*n.v.*, but see Beckmann 1776: 104–108; Fuessly 1778; Prange 1784: 209). It was noticed also (Anonymous 1767: 418) under the title “*Catalogue raisonné & systématique des insectes, compris sous le nom général de Coléoptères, avec 8 estampes de couleur naturelle, & la description en latin, françois & hollandois.*”

Alonso-Zarazaga and Lyal (1999: 8), following Sherborn (1902: liv), argued that the work is not consistently binominal and rejected all names proposed in it. I agree with them.

#### Vogt, Carl Christoph (Giessen, Hesse, Germany: 5 July 1817 – 5 May 1895: Geneva, Switzerland). German zoologist, geologist and physiologist; professor of zoology at the University of Giessen; elected to the Frankfurt Parliament (1848–1849); later, moved to Switzerland and became professor of geology and zoology at the University of Geneva; proponent of polygenist evolution. References. Krause (1896); Taschenberg (1920); Judel (2004, P).

**1850–1851**. *Zoologische Briefe. Naturgeschichte der lebenden und untergegangenen Thiere, für Lehrer, höhere Schulen und Gebildete aller Stände. Mit vielen Abbildungen. Erster Band.* Literarische Anstalt (J. Rütten), Frankfurt a.M. 719 pp. (8vo) GB

The entire work consists of two volumes, 1850–1852. The Coleoptera are on pages 642–677 of the first *Band* which was issued in seven *Lieferungen* as follows: **1–2**: (pp. 1–192) 12 December 1850 (*Allg Bibl Deutsch*); **3–4**: (pp. 193–384) 27 March 1851 (*Allg Bibl Deutsch*); **5–6**: (pp. 385–576) 29 May 1851 (*Allg Bibl Deutsch*); **7**: (pp. 577–719) 14 August 1851 (*Allg Bibl Deutsch*). The title page is dated 1851 and the “*Einleitung*” 1 September 1850.

#### Voigt [Voight], Friedrich Siegmund [Sigismund] (Gotha, Thuringia, Germany: 1 October 1781 – 10 December 1850: Jena, Thuringia, Germany). German physician and naturalist; professor of medicine and botany and director of the botanical garden in Jena (1807–1850). References. Wunschmann (1896a); Robin (2006).

**1838**. *Lehrbuch der Zoologie. Vierter Band. Spezielle Zoologie. Crustazeen; Arachniden; Hymenopteren; Käfer. Mit lithographirten Tafeln.* Schweizerbart, Stuttgart. 459 + [1 (Verbesserungen)] pp. (8vo) GB, BHL

This volume was issued in two parts: **1**: (pp. 1–240) January–June 1838 (*Verz Bücher Landkarten*); **2**: (pp. 241–459) 21 December 1838 (*Allg Forst Jagd Ztg*), 28 December 1838 (*Allg Bibl Deutsch*), July–December 1838 (*Verz Bücher Landkarten*). The title page is dated 1838. The Coleoptera are on pages 319–454. The entire series consists of six volumes, 1834–1840, and one atlas of 22 plates published in 1842. A second edition was issued in 1844–1845 [EUR]; there seems to be no change regarding the fourth volume.

Voigt’s volume was also published under the title “*Naturgeschichte der drei Reiche. Zur allgemeinen Belehrung bearbeitet von G.W. Bischoff, J.R. Blum, H.G. Bronn, K.C. v. Leonhard, F.S. Leuckart und F.S. Voigt. Mit Abbildungen. Zehnter Band. Spezielle Zoologie. Crustazeen; Archniden; Hymenopteren; Käfer*” and formed livraisons 36–37 and 39–40 of the series.

#### Wahnschaffe, Carl Wilhelm Maximilian [Max] (Elbingerode, Saxony-Anhalt, Germany: 10 May 1823 – 6 October 1884: Weferlingen [now part of Oebisfelde-Weferlingen], Saxony-Anhalt, Germany). German forester and entomologist; his collection of Coleoptera is at the Kulturhistorisches Museum Magdeburg. References. Kraatz (1884); Bandoly (2002).

**1883**. *Verzeichniss der im Gebiete des Aller-Vereins zwischen Helmstedt und Magdeburg aufgefundenen Käfer.* C.A. Eyraud, Neuhaldensleben. [2] + 456 pp. (8vo) [DP: 1883 (title page; *Vorwort* dated 10 August 1883), September 1883 (*Nat Nov*), 25 October 1883 (*Naturwiss Ver Sach Thür*), October 1883 (*Zeit Math Naturwiss Unter* 14: 629), November 1883 (*Humboldt*), July–December 1883 (*Bibliot Hist-Nat*)] GB

The main part of this work consists of an annotated catalogue of the Coleoptera found between Helmstedt and Magdeburg in Germany (pp. 27–428).

#### Walckenaer, Charles Athanase (Paris, France: 25 December 1771 – 27 April 1852: Paris, France). French civil servant, biographer, geographer and naturalist; prefect of Niève and later of Aisne; conservator for the Department of Maps at the National Library of France; permanent secretary of the *Académie des Inscriptions et Belles Lettres* in Paris (1840–1852); made baron in 1823; founding member of the *Société Entomologique de France*. References. Hoefer (1866b: 495–499); Fierro (1983: 13); Evenhuis (2012a: 61).

**1802**. *Faune parisienne, insectes. Ou histoire abrégée des insectes des environs de Paris, classés d’après le système de Fabricius; précédée d’un discours sur les insectes en général, pour servir d’introduction à l’étude de l’entomologie; accompagnée de sept planches gravées. Tome premier.* Dentu, Paris. vii + cxxx + 303 pp. + 7 pls. (8vo) [DP: “An XI.–1802” (title page), 19 August–22 September 1802 (*J Lit Fr*), 30 September 1802 (*J Typogr Bibl*), 9 October 1802 (*Merc Fr*), 18 November 1802 (*Observ Spec*), 23 October–21 November 1802 (*Soc Philom*), October–December 1802 (Richards 1935: 174)] CNC, GB, GAL

The entire work was published in two volumes, issued simultaneously. Only the first one deals with Coleoptera (pp. 1–276).

#### Walker, Francis (Arno’s Grove, Southgate, United Kingdom: 31 July 1809 – 5 October 1874: Wanstead, Essex, United Kingdom). British entomologist; worked on contract for the British Museum (1835–1873); well known for describing more than 23,000 new taxa in several orders in the series of British Museum Catalogues. References. Newman (1874b); Papavero (1973: 268–271); Graham (1979, P); Harvey et al. (1996: 209); Evenhuis (2011b, P).

**1859**. List of Ceylon insects. Pp. 269–293 *in*: *Ceylon: an account of the island physical, historical, and topographical with notices of its natural history, antiquities and productions by Sir James Emerson Tennent, K.C.S., LL.D. &c. Illustrated by maps, plans, and drawings. Volume I.* Longman, Green, Longman, and Roberts, London. xxxv + [1] + 619 pp. (8vo) [DP: 1859 (title page; introduction dated 13 July 1859), 15 October 1859 (*Athenaeum*; *Lit Gaz*; *Publ Circ*), 26 October 1859 (*Bookseller*), October 1859 (*Edinb Rev*)] GB, GAL

The entire work was published in two volumes issued simultaneously. Five editions, variously revised, were published within eight months, 1859–1860. Walker’s section was also published in 1861 on pages 442–463 of “*Sketches of the natural history of Ceylon with narratives and anecdotes illustrative of the habits and instincts of the mammalia, birds, reptiles, fishes, insects, &c. including a monograph of the elephant and a description of the modes of capturing and training it with engravings from original drawings by Sir J. Emerson Tennent, K.C.S., LL.D. &c.*” [GB, GAL].

**1866**. List of Coleoptera. Pp. 309–334 *in*: *The naturalist in Vancouver Island and British Columbia. By John Keast Lord, F.Z.S. In two volumes – Vol. II.* Richard Bentley, London. vii + [2] + 375 pp. + 3 pls. (8vo) [DP: 1866 (title page), 16 June 1866 (*Athenaeum*), 30 June 1866 (*Bookseller*), June 1866 (*English Cat Books*), 2 July 1866 (*Publ Circ*)] CNC, GB

Pages 289–375 form the “*Appendix. A list of mammals, birds, insects, reptiles, fishes, shells, annelides, and diatomaceae, collected by myself in British Columbia and Vancouver Island, with notes on their habits. New species, together with those possessing any novel interest, are described in Vol. I. or II.*” in which John Keast Lord acknowledged “Mr. Walker, for naming and describing the Coleoptera” (p. 290).

The entire work consists of two volumes issued simultaneously in 1866. The Coleoptera are in the appendix of the second volume.

**1871**. *List of Coleoptera collected by J.K. Lord, Esq. in Egypt, Arabia and near the African shore of the Red Sea. With characters of the undescribed species*. E.W. Janson, London. 19 pp. (8vo) [DP: 1871 (title page), 1 July 1871 (*Soc Ent Belg*), 15 August 1871 (*Pet Nouv Ent*)] McG, GB

Two new genera and 50 new species are described in this work.

#### Waltl, Joseph (Wasserburg, Bavaria, Germany: 28 July 1805 – 4 March 1888: Passau, Bavaria, Germany). German physician, naturalist and insect dealer; professor of natural history, chemistry and technology in Passau; his collection of Coleoptera is at the Naturhistorisches Museum in Vienna. References. Evenhuis (1997b: 802); Beolens et al. (2013: 228).

**1835**. *Reise durch Tyrol, Oberitalien und Piemont nach dem südlichen Spanien*. *Nebst einem Anhange zoologischen Inhalts.* Pustet, Passau. [8] + 247 + 120 pp. (12mo) [DP: 1835 (title page), February 1835 (*Isis*, Heft II: [2]), January–June 1835 (*Verz Bücher Landkarten*), June 1835 (*Isis*, Heft VI: 485), July 1835 (*Nouv Rev Germ*), 9 September 1835 (*Lit Ztg*)] (*n.v.*).

The book is divided in two *Theile*, each separately paginated. The first one has the same title as the book and the second one is entitled “*Ueber die Tiere Andalusiens.*” The Coleoptera are treated in the second *Theil* (pp. 36–85); several new species and two new genera of beetles are described (pp. 51–85).

A second printing (“*Zweite Auflage*”) was released in 1839 [GB, MDZ] which, according to Evenhuis (1997b: 803), is essentially a reprint of the first edition.

An extract of this work regarding the insects was translated in French by G. Silbermann and published under the title “*Des insectes d’Andalousie*” in *Revue Entomologique* 4 [1836]: 137–164 and another French extract about the Coleoptera was issued in *L’Abeille* 6 [1869]: 1–33.

#### Walton, John (Knaresborough, North Yorkshire, United Kingdom: 23 July 1784 – 3 January 1862: Knaresborough, North Yorkshire, United Kingdom). British sugar refiner and entomologist particularly interested in Curculionidae; his collection was sold at auction in 1863 and several lots were bought by the British Museum (Natural History) and the Oxford University Museum. References. Smith (1862: 125–127); Gilbert (2005: 59).

**1856**. *List of British Curculionidae, with synonyma.* Printed by Order of the Trustees, London. [2] + 46 pp. (12mo) [DP: 1856 (title page; preface dated 10 September 1856), 11 October 1856 (Sherborn 1934: 310), 13 October 1856 (Janson 1857: 84), 8 November 1856 (*Substitute*)] McG, GB

#### Wasmann, Erich (Meran [currently Merano], Trentino-Alto Adige, Italy: 29 May 1859 – 27 February 1931: Valkenburg, Limburg, Netherlands). Austrian Jesuit and naturalist, mainly interested in myrmecophiles and termitophiles; the journal *The Wasmann Journal of Biology* was established by the Jesuits in his honor; his myrmecophilous and termitophilous Coleoptera are at the Natuurhistorich Museum in Maastricht, Netherlands. References. Heikertinger (1931, P); Smetana and Herman (2001: 154–155, P); Barantzke (1999).

**1884**. *Der Trichterwickler. Eine naturwissensschaftliche Studie über den Thierinstinkt. Mit einem Anhange über die neueste Biologie und Systematik der Rhynchitesarten und ihrer Verwandten (Attelabiden, Rhynchitiden und Nemonygiden) (Mit Holzschnitten und Tafeln).* Aschendorff, Münster. vii + 266 pp. + 3 pls. (8vo) [DP: 1884 (title page), 27 September 1884 (*Lit Centr Deutsch*), September 1884 (*Humboldt* 3: 427), October 1884 (*Allg Bibl*; *Nat Nov*), 5 November 1884 (*Wiener Ent Ztg* 3: 287), July–December 1884 (*Verz Bücher Landkarten*; *Bibliot Hist-Nat*)] BHL

The work includes an appendix (pp. 169–258) on the biology and systematics of the species of *Rhynchites* and their relatives where identification keys are provided for the genera and species of Attelabides, Rhynchitides and Nemonychides of Europe, Mediterranean Basin and Siberia (pp. 237– 256).

**1894**. *Kritisches Verzeichniss der myrmekophilen und termitophilen Arthropoden. Mit Angabe der Lebensweise und mit Beschreibung neuer Arten.* Felix L. Dames, Berlin. xiii + [1 (Druckfehler)] + [1 (Erklärung der Zeichen und Abkürzungen] + 231 pp. (8vo) [DP: 1894 (title page), November 1894 (*Nat Nov*; *Zool Anz*), 15 December 1894 (*Academy*), December 1894 (*Acad Rev*)] CNC, GB

This work includes a catalogue of the myrmecophilous and termitophilous arthropods (pp. 59–202) and a section where several new genera, species and varieties of beetles and one Isopoda are described (pp. 205–221).

#### Waterhouse, Charles Owen (London, United Kingdom: 19 June 1843 – 4 February 1917: London, United Kingdom). British entomologist, interested mainly in Coleoptera; curator in entomology at the British Museum (1866–1910); son of George Robert Waterhouse [*q.v.*]; his collections of British Coleoptera are at the National Museums of Scotland in Edinburgh while his collections of exotic Coleoptera are at the Natural History Museum in London. References. Champion (1917, P); Distant (1917); Zimmerman (1993: 807, P).

**1879**. *Illustrations of typical specimens of Coleoptera in the collection of the British Museum. Part I.–Lycidae.* Printed by Order of The Trustees, London. [1] + x + 83 pp. + 18 pls. (8vo) [DP: 1879 (title page; preface dated 22 November 1879), 10 January 1880 (Sherborn 1934: 310), January 1880 (*Nat Nov*), 22 March 1880 (*Zool Anz*), January–June 1880 (*Bibliot Hist-Nat*)] CNC, BHL

**1882–1897**. Fam. Buprestidae. Pp. 1–193, 663–666 + pls 1–9 *in*: *Biologia Centrali-Americana. Insecta. Coleoptera. Vol. III. Part 1. Serricornia.* Taylor and Francis, London. xv + [1 (Errata et corrigenda)] + 690 pp. + 27 pls. (4to) CNC

This work was published in parts as follows (see Lyal 2011: 82): (pp. 1–16 + pl. 1) February 1882; (pp. 17–32 + pl. 2) August 1882; (pp. 33–48 + pl. 3) October 1887; (pp. 49–64 + pl. 4) February 1889; (pp. 65–80) March 1889; (pp. 81–104) April 1889; (pp. 105–112 + pl. 5) May 1889; (pp. 113–120 + pl. 6) July 1889; (pp. 121–136) August 1889; (pp. 137–144) October 1889; (pp. 145–168 + pls 7, 8) November 1889; (pp. 169–193) December 1889; (pl. 9) February 1890; (pp. 663–666 [Appendix]) August 1897.

**1884**. Coleoptera. P. 576 *in*: *Report on the zoological collections made in the Indo-Pacific Ocean during the voyage of H.M.S.* ʻAlertʼ *1881–2.* Printed by Order of the Trustees, London. xxv + 684 pp. + 54 pls. (8vo) [DP: 1884 (title page; preface dated 20 June 1884), July 1884 (Livingstone 1928: 71), September 1884 (*Nat Nov*), October 1884 (*Ann Mag Nat Hist* (5) 14: 291), 29 December 1884 (*Zool Anz*)] GB, BHL

One new species of Coleoptera is described, *Cratopus adspersus*.

**1885**. Description of a new longicorn coleopteron; On the coleopterous insects collected by Mr. H.O. Forbes in the Timor-laut Islands. Pp. 276; 370–375 *in*: *A naturalist’s wanderings in the eastern Archipelago, a narrative of travel and exploration from 1878 to 1883, by Henry O. Forbes, F.R.G.S. With numerous illustrations, from the author’s sketches, and descriptions by Mr. John B. Gibbs.* Sampson Low, Marston, Searle & Rivington, London. xix + 536 pp. (8vo) [DP: 1885 (title page; preface dated 30 January 1885), 9 May 1885 (*Athenaeum*; *Spectator*), 4 June 1885 (*Bookseller*)] GB

The species described on page 276 is *Megacriodes forbesii* and it was first published in the *Annals and Magazine of Natural History* in May 1881 (pp. 408–409). All new species in the second section (pp. 370–375) were first published in the *Proceedings of the Zoological Society of London* in April 1884 (pp. 213–219). The book was also issued the same year in New York by Harper & Brothers [GB, BHL]. It was republished by Oxford University Press in 1989 with the addition of an introduction by the Earl of Cranbrook (viii).

A two-volume German translation of this work was published in 1886 by Costenoble in Jena under the title “*Wanderungen eines Naturforschers im Malayischen Archipel von 1878 bis 1883. Aus dem Englischen von Reinhold Teuscher*” [SBB].

**1885**. On the insects collected on Kilima-njaro by Mr. H.H. Johnston. Pp. 372–377 *in*: *The Kilima-njaro Expedition. A record of scientific exploration in eastern equatorial Africa. And a general description of the natural history, languages, and commerce of the Kilima-njaro district. By H.H. Johnston, F.Z.S., F.R.G.S. With six maps and over eighty illustrations by the author.* Kegan Paul, Trench, and Co., London. xv + 572 pp. (8vo) [DP: 1886 (title page; preface dated November 1885), 12 December 1885 (*Athenaeum*; *Spectator*), December 1885 (*English Cat Books*), 2 January 1886 (*Academy*), 6 January 1886 (*Bookseller*)] GB, ARC

Six new species of Coleoptera are described in Waterhouse’s contribution.

**1900**. [Waterhouse, C.O., Gahan, C.J. and Arrow, G.J.] Order 4.–Coleoptera. Pp. 89–127 + pls 10–11 *in*: *A monograph of Christmas Island (Indian Ocean): physical features and geology by Charles W. Andrews. With descriptions of the fauna and flora by numerous contributors. Illustrated by twenty-two plates, a map, and numerous illustrations in the text.* Printed by order of the Trustees, London. xiii + [1] + 337 pp. + 22 [numbered 1–2, 2*bis*, 3–21] pls. (8vo) [DP: 1900 (title page; introductory note dated 15 February 1900), April 1900 (*Nat Nov*; *Zoologist*), 28 June 1900 (*Nature*)] ANXL, BHL, GB

#### Waterhouse, George Robert (London, United Kingdom: 6 March 1810 – 21 January 1888: London, United Kingdom). British naturalist; curator at the museum of the Zoological Society of London; later, assistant curator of mineralogy and geology at the British Museum and eventually curator upon the death of Charles Konig; invited to join Charles Darwin on the voyage of the Beagle but declined; his types are in the Natural History Museum and his British beetle collection was passed on to his sons. References. Waterhouse (1889); Woodward (1899); Desmond (2004b); Gilbert (2005: 59).

**1858–1861**. *Catalogue of British Coleoptera.* Taylor and Francis, London. iv + 117 pp. (8vo) McG, GB

This catalogue was published in six parts: **1**: (pp. 1–32) 3 April 1858 (*Ent Weekly Intel*), 5 April 1858 (*Ent Soc Lond*); **2**: (pp. 33–48) 1858 (Gerstaecker 1859: 360), 7 March 1859 (*Ent Soc Lond*); **3**: (pp. 49–64) 2 January 1860 (*Ent Soc Lond*); **4**: (pp. 65–80) 6 August 1860 (*Ent Soc Lond*); **5**: (pp. 81–96) 7 January 1861 (*Ent Soc Lond*); **6**: (pp. 97–117 + i–iv) 28 January 1862 (*Ent Soc Lond*). The title page is dated 1858 and the introduction 12 April 1861. Likely the last part was published in 1861. Horn and Schenkling (1929: 1298) mentioned that this catalogue was published in 1858 and that a new edition was issued in 1861. This could not be corroborated during this study. It seems obvious from the records of the Entomological Society of London that a sole edition was issued between 1858 and 1861.

#### Weber, Friedrich (Kiel, Schleswig-Holstein, Germany: 3 August 1781^104^ – 21 March 1823: Kiel, Germany). German physician, botanist and entomologist; pupil of Johann Christian Fabricius [*q.v.*]; professor of medicine and botany and supervisor of the hospital at the University of Kiel; his types are apparently in the Zoologisk Museum at the University of Copenhagen via Fabricius collection. References. Wunschmann (1896b); Evenhuis (1997b: 807, P).

**1795**. *Nomenclator entomologicus secundum Entomologiam Systematicam ill. Fabricii adjectis speciebus recens detectis et varietatibus.* Carolum Ernestum Bohn, Chilonii et Hamburgi [= Kiel & Hamburg]. viii + 171 + [1] pp. (8vo) [DP: 1795 (title page), 14 October 1796 (*Allg Lit Ztg*)] GB

This work consists of a list of insect species in Fabricius’ *Entomologia Systematica* published in 1792–1794 [*q.v.*]. It has been placed on the *Official List of Works Approved as Available for Zoological Nomenclature* in Direction 32 (ICZN 1956a).

**1801**. *Observationes entomologicae, continentes novorvm qvae condidit genervm characteres, et nvper detectarvm speciervm descriptiones.* Impensis Bibliopolii Academici Novi, Kiliae [= Kiel]. xii + 116 + [1 (Errata)] pp. (8vo) [DP: 1801 (title page; *lectori* dated August 1800), before March 1801 (Evenhuis 1997b: 809), 29 August 1801 (*Intell Allg Lit Ztg*)] CNC, GB

The book is divided in two chapters: *I. Genervm, partim ab avtore conditorvm, partim ab eo emendatorvm characteres* (pp. 1–32); *II. Descriptiones nvper detectorvm speciervm* (pp. 33–116).

Illiger (1801b) reviewed Weber’s *Observationes entomologicae* in a paper published in volume 1 of the *Magazin für Insektenkunde*. The *Vorerinnerung* (pp. iii–vi) of the journal is dated March 1801 and this is the basis for Evenhuis’ (1997b: 809) date of publication.

**1804**. [Weber, F. and Mohr, D.M.H.] *Naturhistorische Reise durch einen Theil Schwedens. Mit drei Kupfertafeln.* Heinrich Dieterich, Göttingen. xii + 13–207 + [1 (*Verbesserungen*)] pp. + 3 pls. (8vo) [DP: 1804 (title page; *Vorerinnerung* dated September 1803), 15 April 1804 (Sayre 1959: 23), 15 September 1804 (*Intell Allg Lit Ztg*), 22 April 1805 (*Neue Leip Lit*)] MCZ, GB

A few new species of insects, including the beetle *Elophorus minutissimus* (p. 64), are described in this work.

#### Weigel, Johann Adam Valentin (Sommerhausen near Würzburg, Bavaria, Germany: 29 September 1740 – 24 June 1806: Ober-Haselbach near Landeshut [currently Kamienna Góra], Poland). Lutheran preacher and naturalist; pastor in Haselbach, Dittersbach and Bolkenhain (= Bolków, Poland). References. Weis (1842: 265); Letzner (1858: 13–15).

**1805**. *Geographische, naturhistorische und technologische Beschreibung des souverainen Herzogthums Schlesien. Zehnter Theil. Verzeichnis der bisher entdeckten, in Schlesien lebenden Thiere*. Himburg, Berlin. viii + 358 pp. (8vo) [DP^105^: 1806 (title page; *Vorrede* dated 1 August 1805), 10 August 1805 (*Intell Ztg Welt*), 14 August 1805 (*Intell Allg Lit Ztg*)] GB, MDZ

The entire work was published in ten *Theile*, 1800–1805. A list of insects from Silesia is included in the tenth *Theil* (pp. 45–330) and the Coleoptera are on pages 45–165.

This work is sometimes recorded (e.g., Hagen 1863: 263; Horn and Schenkling 1929: 1302) under the title “*Faunae Silesiacae prodromus. Verzeichniss der Thiere, die in Schlesien bisher entdeckt und bestimmt sind*” by the same publisher. Likely, the tenth *Theil* was also issued separately with another title page. Despite the date of 1806 on its title page, this work was published in 1805.

#### Weise, Julius (Sommerfeld [currently Lubsko], Poland: 6 June 1844 – 25 February 1925: Herichsdorf [currently Malinnik], Poland). Prussian coleopterist, specialist of the beetle families Chrysomelidae and Coccinellidae; worked as a teacher in various schools in Postdam and Berlin until 1912 when he retired; his collection of Chrysomelidae and Coccinellidae is at the Zoologisches Museum der Humboldt-Universität in Berlin while the rest of his collection was scattered. Reference. Heikertinger (1924, P).

**1878**. Beitrag zur Kenntniss der Gattung *Laena* Latreille. Pp. 227–241 *in*: *Beiträge zur Kenntniss der kaukasischen Käferfauna von Dr. Oscar Schneider und Hans Leder.* W. Burkart, Brünn. 359 + [1 (Berichtigungen)] pp. + 6 pls. (8vo) [DP: 1878 (title page; preliminaries dated November 1877), 4 January 1879 (*Gesell Erdk Berl*), 15 February 1879 [JD] (*Soc Imp Nat Moscou*), February 1879 (*Nat Nov*)] GB

Weise’s contribution was also issued in *Verhandlungen des Naturforschenden Vereines in Brünn* 16 [1877]: 227–241 [DP: 1878 (title page), 15 April–15 May 1879 (*Leopoldina* 15: 120), July 1879 (*Nat Nov*)].

**1881–1893**. *Naturgeschichte der Insecten Deutschlands begonnen von Dr. W.F. Erichson, fortgesetzt von Prof. Dr. H. Schaum, Dr. G. Kraatz, H. von Kiesenwetter, J. Weise und E. Reitter. Erste Abtheilung. Coleoptera. Sechster Band.* Nicolai, Berlin. vi + 1161 pp. + 1 pl. (8vo) CNC, BHL

This work was published in six *Lieferungen* (dates of publication, except for the last *Lieferung*, are listed in the *Vorrede*): **1**: (pp. 1–192) 1 October 1881; **2**: (pp. 193–368) 1 December 1882; **3**: (pp. 369–568) 15 October 1884; **4**: (pp. 569–768) 15 June 1886; **5**: (pp. 769–960) 10 May 1888; **6**: (pp. i–xiv + 961–1161) 1893 (title page; *Vorrede* dated “*Lichtmess* 1893” [= 2 February 1893]), March 1893 (*Nat Nov*; Griffin 1939a: 218).

**1885**. *Bestimmungs-Tabellen der europäischen Coleopteren. II. Heft. Coccinellidae. II. Auflage. Mit Berücksichtigung der Arten aus dem nördlichen Asien.* Edmund Reitter, Mödling. 83 pp. (8vo) [DP: 1885 (title page), May 1885 (*Nat Nov*)] SME

A French edition was issued in 1892 in *L’Abeille* 28: 1–96. The first edition of this work was published in 1879 in *Zeitschrift für Entomologie* (Neue Folge) 7: 88–156.

#### Well, Johann Jacob [Jakob] von (Prague, Czech Republic: 1 March 1725 – 4 April 1787: Vienna, Austria). Austrian apothecary, physician and naturalist; professor of natural history at the University of Vienna. References. Hecker (1839: 580); Wurzbach (1886: 225).

**1781**. Additamenta quaedam ad entomologiam. Pp. 380–388 + pl. 23 *in*: *Nicolai Josephi Jacquin miscellanea austriaca ad botanicum, chemiam, et historiam naturalem spectantia, cum figuris. Vol. II.* Kraus, Vindobonae [= Vienna]. 423 + [1] pp. + 23 pls. (4to) [DP^106^: 1781 (title page), 17 June 1782 (*Gött Anz*)] CNC, BHL, GB

The Coleoptera species described in this work are: *Scotias psylloides* (pp. 383), *Buprestis fastuosa* (p. 385), and *Dityscus anastomozans* (p. 386).

#### Wencker, Joseph Antoine (Haguenau, Bas-Rhin, France: 29 March 1824 – 20 February 1873: Viterne, Meurthe-et-Moselle, France). French draftsman at the *Chemins de fer de l’Est* and amateur coleopterist; his collection was sold at auction by Émile Deyrolle in Paris. References. Constantin (1992: 90); Cambefort (2006: 312).

**1866**. [Wencker, J. and Silbermann, G.] *Catalogue des coléoptères de l’Alsace et des Vosges. Suivi de descriptions de plusieurs espèces nouvelles par Ch. Brisout de Barneville et Wencker.* G. Silbermann, Strasbourg. vi + 142 pp. (4to) [DP: 1866 (title page; page vi dated April 1866), January–July 1866 (*Bibl Alsacien*), 15 September 1866 (*Bibl Fr*), 20 September 1866 (*Rev Instr Publ*), August–September 1866 (*Allg Bibl*), 8 December 1866 (*Publ Circ*)] CMLE, GB

A supplement to the catalogue was published in 1891 by Albert Claudon in *Mittheilungen der Naturhistorischen Gesellschaft in Colmar* (Neue Folge) 1 [1889–1890]: 99–130.

#### Wessel, August Wilhelm (Osnabrück, Lower Saxony, Germany: 14 June 1817 – ?). German naturalist and high school teacher at Aurich in Lower Saxony. Reference. Stafleu and Cowan (1988: 200).

**1875**. Beitrag zur Käferfauna Ostfrieslands. Pp. 3–24 *in*: *Programm des Königlichen Gymnasiums zu Aurich. Ostern 1875.* Dunkmann, Aurich. 32 + [1] pp. (4to) [DP: “Ostern 1875” (title page)] ULBD

This work is essentially a checklist of the Coleoptera of East Frisia, a coastal region in the German state of Lower Saxony. An improved version of this work was published under the same title in April 1877 in *Abhandlungen des naturwissenschaftlichen Vereins zu Bremen* 5: 367–394.

#### Westhoff, Friedrich (Münster, North Rhine-Westphalia, Germany: 8 September 1857 – 12 November 1896: Münster, Germany). German entomologist, local historian and writer; assistant at the Museum of the Academy of Münster and lecturer of zoology. References. Reeker (1897, P); Berger (1996).

**1881–1882**. *Die Käfer Westfalens.* Max Cohen & Sohn, Bonn. (8vo) GB

*I. Abtheilung.* xxviii + 140 pp. [DP: 1881 (title page; preliminaries dated 15 January 1881), 22 September 1881 (*Nature*), 6 October 1881 (*Kaiser Akad Wiss*), 11 October 1881 (*Soc Fauna Flora Fenn*), 18 October 1881 (*Gesell Naturfors Freun Berl*), 11 November 1881 (*Soc Bot Fr*), December 1881 (*Nat Nov*), 18 February 1882 [JD] (*Soc Imp Nat Moscou*)].

*II. Abtheilung.* Pp. 141–323. [DP: 1882 (title page), February 1882 (*Nat Nov*)].

This work, which treats the Coleoptera of Westphalia in Germany, was issued as a supplement to volume 38 of *Verhandlungen des naturhistorischen Vereins der preussischen Rheinlande und Westfalens*.

#### Westwood, John Obadiah (Sheffield, South Yorkshire, United Kingdom: 22 December 1805 – 2 January 1893: Oxford, Oxfordshire, United Kingdom). British entomologist and paleontologist; professor of entomology at the University of Oxford; founding member of the Entomological Society of London; his collections were purchased by the Reverend Hope who gave them to the University of Oxford. References. McLachlan (1893a, P); Wandolleck (1894, P); Zimmerman (1993: 810, P); Salmon (2000: 148–150, P); Foote (2004f).

**1835–1840**. Descriptions of insects figured in plates 9 and 10. Pp. liii–lv + pls 9–10 *in*: *Illustrations of the botany and other branches of the natural history of the Himalayan mountains, and of the flora of Cashmere. By J. Forbes Royle.* Wm. H. Allen and Co., London. (4to) McG, GB

*Vol. I.* viii + [8] + lxxviii + [2] + 472 pp.

*Vol. II.–Plates.* [4] pp. + 97 pls.

These volumes were issued in 11 livraisons, 1833–1840, bound as one volume of text and one volume of plates (dates of publication of the text are listed on page [2] of the first volume; see also Sprague 1933, Stearn 1943: 485, and Stafleu and Cowan 1983: 963): **1**: (pp. 1–40, pls 4, 11–18, 22) September 1833; **2**: (pp. v–xii, 41–72, pls 1, 19–21, 23–28) March 1834, 1 March 1834 (*Athenaeum*); **3**: (pp. xiii–xx, 73–104, pls 2, 5, 29, 31–35, 37, 38) June 1834, 28 June 1834 (*Athenaeum*); **4**: (pp. 105–136, pls 30, 39, 40, 42, 44–46, 64, 76 [as 75], 78) September 1834; **5**: (pp. 137–176, pls 3, 41, 48–51, 57, 62–64) January 1835; **6**: (pp. 177–216, pls 7, 36, 43, 55, 56, 58, 60, 61, 75 [as 75a], frontispiece to vol. 1) April 1835, 27 April 1835 (*Geol Soc Lond*); **7**: (pp. 217–248, pls 8, 9, 47, 52, 59, 64, 67–69, 71, 77) August 1835, 22 August 1835 (*Athenaeum*), 24 August 1835 (Stafleu and Cowan 1983: 963); **8**: (pp. 249–288, pls 53, 54, 66, 70, 72, 73, 79, 80, 87, 88) December 1835; **9**: (pp. 289–336, pls 10, 81–86, 90, 100) May 1836, 10 May 1836 (*Monthly Lit Adv*); **10**: (pp. 337–384, pls 89, 91–96, 99, frontispiece to vol. 2) February 1839, 23 February 1839 (*Athenaeum*); **11**: (pp. 385–472, xxi–lxxviii + [2], title pages, pls 6, 97) 1840, 20 April 1840 (*Geol Soc Lond*), 25 April 1840 (*Spectator*; *Athenaeum*), 12 May 1840 (*Monthly Lit Adv*), 22 May 1840 (*Allg Bibl Deutsch*), May 1840 (*Monthly Rev*). The title page of the first volume is dated 1839 and the dedication 28 November 1839.

The genera and species of Coleoptera described in the text (pp. liv–lv) and illustrated on plate 9 are: *Cetonia roylii* Hope (p. liv), *Geotrupes orientalis* (p. liv), *Onthophagus phanoeoides* (p. lv), *Lucanus lunifer* (p. lv), *Lamia wallichii* (p. lv), *Porus*, Hope (p. lv), *Porus ochraceus* (p. lv), *Aphodius irregularis*, Hope (p. lv), *Anisotelus*, Hope (p. lv), *Anisotelus bimaculatus* (p. lv), *Elater cyanopterus*, Hope (p. lv), *Ripiphorus apicalis* (p. lv). The insect names are listed on the plates. Since plate 9 was issued earlier (August 1835) than the text (1840), all genera and species are made available by the illustrations and dated from 1835.

The book also includes a section by F.W. Hope entitled “*On the entomology of the Himalayas and of India*” (pp. xxxvii–lii) which includes “*Analysis of the entomology of the Himalayas and of India*” (pp. xliii–lii) where many groups of beetles are discussed briefly: Cicindelidae (p. xliii), Dryptidae (p. xliii), Lebiadae (p. xliii), Brachinidae (p. xliii), Scaitidae [sic] (p. xliii), Harpalidae (p. xliii), Pogonidae (p. xliv), Calathidae (p. xliv), Feroniadae (p. xliv), Sphodridae (p. xliv), Anchomenidae (p. xliv), Callistidae (p. xliv), Dicoelidae (p. xliv), Proceridae (p. xliv), Nebriadae (p. xliv), Bembidiidae and Trechidae (p. xlv), Dyticidae (p. xlv), Gyrinidae (p. xlv), Hydrophilidae (p. xlv), Necrophaga (p. xlv), Erotylidae (p. xlv), Dermestidae (p. xlv), Byrrhidae (p. xlv), Histeridae (p. xlv), Lucanidae (p. xlvi), Lamellicornes (p. xlvi), Geotrupidae (p. xlvi), *Scarabaeus* (p. xlvi), Melolonthidae (p. xlvi), Mimelae and *Euchlora* (p. xlvii), *Popillia* (p. xlvii), Trichiidae (p. xlvii), Cetoniadae (p. xlvii), Buprestidae (p. xlvii), Elateridae (p. xlvii), Lampyridae (p. xlvii), Malacodermata (p. xlvii), Cleridae (p. xlviii), Curculionidae (p. xlviii), Prionidae (p. xlviii), Lamiadae (p. xlviii), Sagridae (p. xlviii), Gallerucidae (p. xlviii), Chrysomelidae (p. xlviii), Eumolpidae (p. xlviii), Cassididae (p. xlix), Coccinellidae (p. xlix), Hispidae (p. xlix), Tenebrionidae (p. xlix), Pimeliariae (p. xlix), Helopidae (p. xlix), Mordellidae (p. xlix), Cantharidae (p. xlix), Staphylinidae (p. xlix).

**1837**. *Illustrations of exotic entomology, containing upwards of six hundred and fifty figures and descriptions of foreign insects, interspersed with remarks and reflections on their nature and properties. By Dru Drury. A new edition, brought down to the present state of the science, with the systematic characters of each species, synonyms, indexes, and other additional matter.* Henry G. Bohn, London. (4to) CNC, BHL

*Vol. I.* xxvi + 123 pp. + 50 pls. [DP: 1837 (title page), 26 August 1837 (*Lit Gaz*), August 1837 (Sherborn 1922a: xlvi and 1932: cxxxvii), 2 September 1837 (*Athenaeum*), 8 May–8 September 1837 (*Analyst* 7: 167), 11 September 1837 (*Monthly Lit Adv*)]. The Coleoptera taxa treated are on pages 59–90.

*Vol. II.* vi + 100 pp. + 50 pls. [DP: 1837 (title page), 26 August 1837 (*Lit Gaz*), August 1837 (Sherborn 1922a: xlvi and 1932: cxxxvii), 2 September 1837 (*Athenaeum*), 8 May–8 September 1837 (*Analyst* 7: 167), 11 September 1837 (*Monthly Lit Adv*)]. The Coleoptera taxa treated are on pages 57–69.

*Vol. III.* vi + 93 + [1 (Errata)] pp. + 50 pls. [DP: 1837 (title page), 26 August 1837 (*Lit Gaz*), August 1837 (Sherborn 1922a: xlvi and 1932: cxxxvii), 2 September 1837 (*Athenaeum*), 8 May–8 September 1837 (*Analyst* 7: 167), 11 September 1837 (*Monthly Lit Adv*)]. The Coleoptera taxa treated are on pages 54–55, 60–61, 68–77.

The entire work was issued in three volumes published simultaneously. Horn and Schenkling (1929: 1313) and Evenhuis (1997b: 813) dated the second volume 1840 and the third one 1842 but by August 1837 all three volumes were published.

This is a new edition of Drury’s *Illustrations of natural history*, 1770–1782 [*q.v.*].

**1838–1840**. *An introduction to the modern classification of insects; founded on the natural habits and corresponding organisation of the different families. In two volumes.* Longman, Orme, Brown, Green, and Longmans, London. (8vo) CNC, GB, BHL

*Vol. I.* xii + [1 (Explanation of plate)] + 462 pp. + 1 pl. This volume was issued in eight parts, 1838, as follows: **1**: (pp. 1–48) May 1838 (wrapper), 1 May 1838 (*Publ Circ*), 7 May 1838 (*Ent Soc Lond*); **2**: (pp. 49–112) June 1838 (wrapper), 1 June 1838 (*Publ Circ*), 4 June 1838 (Griffin 1932: 83); **3**: (pp. 113–160) July 1838 (wrapper); **4**: (pp. 161–224) August 1838 (wrapper); **5**: (pp. 225–288) September 1838 (wrapper); **6**: (pp. 289–352) October 1838 (wrapper), 1 October 1838 (*Ent Soc Lond*); **7**: (pp. 353–400) November 1838 (wrapper), 5 November 1838 (*Ent Soc Lond*); **8**: (pp. 401–462) December 1838 (wrapper), 3 December 1838 (*Ent Soc Lond*). The title page is dated 1839.

*Vol. II.* xi + 587 + 158 pp. This volume contains the suite to “*An introduction to the modern classification of insects*” (xi + 587 pp.), issued in eight parts in 1839 and 1840, and an appendix entitled “*Synopsis of the genera of British insects*” (158 pp.), issued along with six parts of the *Introduction*, 1838–1840. The dates of publication of the *Introduction* are as follows: **9**: (pp. 1–128) January 1839 (wrapper); **10**: (pp. 129–192) February 1839 (wrapper), 4 February 1839 (*Ent Soc Lond*); **11**: (pp. 193–224) March 1839 (wrapper), 4 March 1839 (*Ent Soc Lond*); **12**: (pp. 225–256) April 1839 (wrapper); **13**: (pp. 257–288) June 1839 (wrapper), 3 June 1839 (*Ent Soc Lond*); **14**: (pp. 289–352) November 1839 (wrapper); **15**: (pp. 353–400) January 1840 (wrapper), 6 January 1840 (Griffin 1932: 84); **16**: (pp. 401–587) June 1840 (wrapper), 1 June 1840 (Griffin 1932: 84), 6 June 1840 (*Athenaeum*). The dates of publication of the *Synopsis* are as follows (see ICZN 1957c): **1**: (pp. 1–16) May 1838; **3**: (pp. 17–32) July 1838; **7**: (pp. 33–48) November 1838; **13**: (pp. 49–80) June 1839; **15**: (pp. 81–96) January 1840; **16**: (pp. 97–154) June 1840, 6 June 1840 (*Athenaeum*). The title page is dated 1840. The *Synopsis* is taxonomically and nomenclaturally important since it includes type species designations for all genera treated.

These two volumes have been placed on the *Official List of Works Approved as Available for Zoological Nomenclature* in Direction 32 (ICZN 1956a) with the endorsement that in the separately paged “Synopsis” attached to volume 2 the species specified against the names of the genera enumerated are to be treated as having been there definitely selected to be the type species of those genera.

**1840**. The articulated animals. Pp. 387–637 *in*: *Cuvier’s animal kingdom, arranged according to its organization; forming the basis for a natural history of animals, and an introduction to comparative anatomy. Mammalia, birds, and reptiles, by Edward Blyth. The fishes and radiata, by Robert Mudie. The molluscous animals, by George Johnston, M.D. The articulated animals, by J.O. Westwood, F.L.S. Illustrated by three hundred engravings on wood.* Wm. S. Orr and Co., London. vii + [1] + 670 pp. (4to) [DP: 1840 (title page; preface dated June 1840), 5 September 1840 (*Athenaeum*), 26 September 1840 (*Lit Gaz*), September 1840 (Low 1853: 86), 10 October 1840 (*Monthly Lit Adv*)] GB

The Coleoptera are on pages 491–556. Type species are designated for some genus-group taxa.

**1841–1845**. *Arcana entomologica; or illustrations of new, rare, and interesting insects. In two volumes.* William Smith, London. (8vo) CNC, GB

*Vol. I.* iv + 192 pp. + 48 pls. This volume was published in 12 parts. The publication dates are as follows (Baker 1996: 442): **1**: (pp. 1–16, pls 1–4) 1 May 1841; **2**: (pp. 17–32 + pls 5–8) 1841 (probably 1 July), September 1841 (*Rev Zool*); **3**: (pp. 33–48 + pls 9–12) 1841 (probably 1 September); **4**: (pp. 49–64 + pls 13–16) 1841 (probably 1 November); **5**: (pp. 65–80 + pls 17–20) 1 January 1842; **6**: (pp. 81–96 + pls 21–24) 1 March 1842; **7**: (pp. 97–112 + pls 25–28) 1 May 1842; **8**: (pp. 113–128 + pls 29–32) 1 July 1842; **9**: (pp. 129–144 + pls 33–36) 1 September 1842; **10**: (pp. 145–160 + pls 37–40) 1 November 1842; **11**: (pp. 161–176 + pls 41–44) 1 January 1843; **12**: (pp. 177–192 + pls 45–48) 1 March 1843. The title page is dated 1845. The volume contains separately titled articles explaining the plates and eight others [numbered [I], II, III, IV, V, VII, IX, X] treating various aspects of entomology [*Entomological intelligence, notices of new works, &c.*]. The Coleoptera are treated on the following plates: **1** (pp. 1–6 [*Descriptions of some Asiatic cornuted species of Cetoniidae*]); **10**: (pp. 35–40 [*The coleopterous genus Hypocephalus illustrated*]); **12**: (pp. 43–44 [*Descriptions of some new genera of Australian heteromerous beetles in the collection of the Rev. F.W. Hope*]); **15**: (pp. 57–58 [*Descriptions of some new longicorn beetles from the Indian Archipelago*]); **19**: (pp. 69–72 [*Descriptions of some Cetoniidae from tropical Africa*]); **21–23**: (pp. 81–90 [*On the scaritideous beetles of New Holland*]); **28**: (pp. 103–104 [*Descriptions of some new species of Cetoniidae, from Australia, Asia, and the Asiatic islands*]); **29–30**: (pp. 113–122 [*On the goliathideous Cetoniidae of Asia*]); **32**: (pp. 125–128 [*Illustrations of some species of Cetoniidae from Madagascar*]); **33–36**: (pp. 129–141 [*On the goliathideous Cetoniidae of Asia. Part II*]); **42–44**: (pp. 163–176 [*On the goliathideous Cetoniidae of Africa. Part I*]); **45–46**: (pp. 177–188 [*On the goliathideous Cetoniidae of Africa. Part II*]).

*Vol. II.* [2] + 191 + [1 (Errata et corrigenda)] pp. + 48 pls [numbered 49–68, 68–95]. This volume was published in 12 parts. The publication dates are as follows (Baker 1996: 442): **13**: (pp. 1–16 + pls 49–52) 1 May 1843; **14**: (pp. 17–32 + pls 53–56) 1 July 1843; **15**: (pp. 33–48 + pls 57–60) 1 September 1843; **16**: (pp. 49–64 + pls 61–64) 1 November 1843; **17**: (pp. 65–80 + pls 65–68) 1 January 1844; **18**: (pp. 81–96 + pls 68 [number duplicated]–71) 1 March 1844; **19**: (pp. 97–112 + pls 72–75) 1 May 1844; **20**: (pp. 113–128 + pls 76–79) 1 July 1844; **21**: (pp. 129–144 + pls 80–83) 1 September 1844; **22**: (pp. 145–160 + pls 84–87) 1 November 1844; **23**: (pp. 161–176 + pls 88–94) 1 February 1845; **24**: (pp. 177–192 + pl. 95) 1 June 1845. The title page is dated 1845. The volume contains separately titled articles explaining the plates and five others [numbered XIV, XV, XVI, XVIII, XIX] treating various aspects of entomology [*Entomological intelligence, notices of new works, &c.*]. The Coleoptera are treated on the following plates: **49–50**: (pp. 1–12 [*Monograph of the coleopterous family Paussidae. Part I*]); **56**: (pp. 25–28 [*On the longicorn Coleoptera of New Zealand*]); **58**: (pp. 37–40 [*Monograph of the coleopterous family Paussidae. Part II*]); **64**: (pp. 57–58 [*Descriptions of some African longicorn beetles*]); **67**: (pp. 71–72 [*Illustrations of two new goliath beetles*]); **68**: (pp. 73–80 [*Monograph of the coleopterous family Paussidae. (Part III)*]); **69**: (pp. 83–86 [*Illustrations of some species of longicorn beetles from tropical western Africa*]); **73**: (pp. 99–100 [*On two species of Inca from tropical America*]); **78**: (pp. 125–126 [*Illustrations of some species of longicorn beetles from tropical western Africa*]); **81**: (pp. 131–134 [*Descriptions of two species of goliath beetles, from tropical western Africa*]); **84–86**: (pp. 147–158 [*Illustrations of some African species of longicorn beetles*]); **87**: (pp. 159–160 [*Illustrations of four species of the genus Chiroscelis*]); **88–95**: (pp. 161–190 [*Monograph of the coleopterous family Paussidae. Part IV*]); **96**: (pp. 191–192 [*Illustrations of two species of goliath beetles from Africa*]).

This work was first issued in parts (subscribers’ issue) and later sold by the publisher as two complete volumes (publisher’s issue). The title pages of the publisher’s issue differ in that the words “*In two volumes*” were omitted and the dates listed are “1st May, 1841–1st March, 1843” (volume 1) and “1st May, 1843–1st June, 1845” (volume 2) (see Baker 1996).

**1847–1848**. *The cabinet of Oriental entomology; being a selection of some of the rarer and more beautiful species of insects, natives of India and the adjacent islands, the greater portion of which are now for the first time described and figured.* William Smith, London. 2 (Systematic arrangement) + 2 (Notice) + 88 pp. + 42 pls. (4to) CMLE, BHL

Each plate of this work has his own date, from 1 January 1847 to 31 December 1847. Sherborn (1922a: cxxviii) and Evenhuis (1997b: 815) dated the species illustrated from the dates of the plates. However, there are no scientific names listed on the plates and the new species must date from the text. As seen on the back cover of the *Journal of the Asiatic Society of Bengal* for September 1847, “No. I of the Cabinet of Oriental entomology” was published on January 1, 1847 and “each number will contain three beautifully-colored plates.” The text is not mentioned. The title page of the book is dated 1848 and Low (1853: 390) mentioned that it was issued in June 1848; it was recorded on 15 June 1848 (*Publ Circ*) and noted in the section for new publication from May 9th to June 9th, 1848 (*Monthly Lit Adv*). The “Notice” in the book itself is dated 1 January 1847.

**1874**. *Thesaurus entomologicus oxoniensis; or, illustrations of new, rare, and interesting insects, for the most part contained in the collections presented to the University of Oxford by the Rev. F.W. Hope, M.A., D.C.L., F.R.S., &c. With forty plates from drawings by the author.* Clarendon Press, Oxford. xxiv + 205 pp. + 40 pls. (4to) [DP: 1874 (title page; advertisement dated October 1873), 12 December 1874 (*Athenaeum*; *Spectator*), 18 December 1874 (*Publ Circ*; *Lit World-Lond*)] CNC, BHL

The work includes an “*Orbituary notice of the Rev. Fred. Wm. Hope*” by T.J. Pettigrew (pp. xvii–xxiv). The book was published in four parts, received by the Entomological Society of London on the following dates: **1** (pp. 1–?? + 10 pls) 2 February 1874; **2**: (pp. ??–?? + 10 pls)^107^ 1 February 1875; **3–4**: (pp. ??–205 + 20 pls) 7 December 1874. Collation for each part is desired.

**1881**. IV. Entomology. Pp. 331–365 + pls E–H *in*: *Matabele land and the Victoria Falls: a naturalist’s wanderings in the interior of South Africa from the letters & journals of the late Frank Oates, F.R.G.S. Edited by C.G. Oates, B.A.* C. Kegan Paul & Co., London. xliii + 383 pp. + 18 pls. (8vo) [DP: 1881 (title page; preface dated May 1881), 30 July 1881 (*Academy*), 13 August 1881 (*Athenaeum*; *Spectator*), 1 September 1881 (*Publ Circ*), September 1881 (*Nat Nov*), 12 November 1881 (*Sat Rev*), July–December 1881 (*Bibliot Hist-Nat*)] MCZ, GB

The following new beetle taxa are described: Dromica (Myrmecoptera) oatesii (p. 359), *Derosphaerius* (p. 362), and *Derosphaerius anthracinus* (p. 362). A second edition of this work was published in 1889 (see next entry).

**1889**. IV. Entomology. Pp. 338–389 + pls 5–9 *in*: *Matabele land and the Victoria Falls: a naturalist’s wanderings in the interior of South Africa from the letters & journals of the late Frank Oates, F.R.G.S. Edited by C.G. Oates, B.A. Second edition*. Kegan Paul, Trench & Co., London. xlix + 433 pp. + 22 pls. (8vo) [DP: 1889 (title page; preface dated July 1889), 2 November 1889 (*Athenaeum*), 16 November 1889 (*Spectator*), 30 November 1889 (*Academy*), 14 December 1889 (*Bookseller*), December 1889 (*Nat Nov*)] BHL

The following new beetle taxa are proposed in this edition: *Anthia hottentotta* (p. 368), *Toxicum matabelicum* Olliff (p. 376), *Oatesius* Westwood (p. 376, as a replacement name for *Derosphaerius*), *Tapinolachnus oatesii* Olliff (p. 378), *Tapinolachnus aquilus* Olliff (p. 379), and *Eunidia batesii* Olliff (p. 381).

#### White, Adam (Edinburgh, Scotland, United Kingdom: 29 April 1817 – 4 January 1879: Glasgow, Scotland, United Kingdom). British entomologist; curator of the entomological collections at the British Museum (1835–1863); forced to retire after a nervous breakdown; his collection is at the Natural History Museum in London. References. McLachlan (1879); Woodward (1900a); Evenhuis (1997b: 817, P); Clark and Presswell (2001); Datta (2004).

**1841**. Notes on some insects from King George’s Sound, collected and presented to the British Museum by Captain George Grey. Pp. 450–482 *in*: *Journals of two expeditions of discovery in north-west and western Australia, during the years 1837, 38, and 39, under the authority of Her Majesty’s Government. Describing many newly discovered, important, and fertile districts, with observations on the moral and physical condition of the aboriginal inhabitants, &c. &c. By George Grey, Esq. In two volumes. Vol. II.* T. and W. Boone, London. vii + 482 pp. (8vo) [DP: 1841 (title page), 13 November 1841 (*Athenaeum*; *Lit Gaz*), November 1841 (Low 1853: 144), 4 December 1841 (*Spectator*), 10 December 1841 (*Monthly Lit Adv*), 16 February 1842 (*Soc Ent Fr*)] GB

A facsimile of this work was issued in 1964 in Adelaide.

**1843**. [White, A. and Doubleday, E.] List of the annulose animals hitherto recorded as found in New Zealand, with the descriptions of some new species. Pp. 265–295 *in*: *Travels in New Zealand; with contributions to the geography, geology, botany, and natural history of that country. By Ernest Dieffenbach, M.D. In two volumes. –Vol. II.* John Murray, London. iv + 396 pp. + 1 pl. (8vo) [DP: 1843 (title page), 21 January 1843 (*Athenaeum*; *Lit Gaz*), 28 January 1843 (*Spectator*), January 1843 (Low 1853: 95), 25 February 1843 (*Lit Ztg*), March 1843 (*Gent Mag*), 15 April 1843 (*Intell Serapeum*)] BOT, BHL, GB

The entire work consists of two volumes issued simultaneously. It was reprinted by Capper Press, in Christchurch, New Zealand, in 1974.

**1846**. Insects. Pp. 1–24 + pls 1–6 *in*: *The zoology of the voyage of H.M.S.* Erebus *&* Terror, *under the command of Captain Sir James Clark Ross, R.N., F.R.S., during the years 1839 to 1843. By authority of the Lords Commissioners of the Admiralty. Edited by John Richardson and John Edward Gray. Vol. II. Reptiles, fishes, Crustacea, insects, Mollusca.* E.W. Janson, London. [1] + 17 (reptiles) + viii + 139 (fishes) + 5 (Crustacea) + 51 (insects) + 7 (Mollusca) pp. + 98 pls. (4to) MCZ, BHL

This volume contains five separately paginated sections dealing with various zoological groups, published 1844–1875. The insect section contains 51 pages and 10 plates. The first 24 pages were published in April 1846 and the remaining ones in June 1874 (Evenhuis 2015b: Table 3). Adam White is the author of the first part, A. White and A.G. Butler authored the second part. The Coleoptera are on pages 1–23.

**1846**. Descriptions of new or unfigured species of Coleoptera from Australia. Pp. 505–512 + 2 pls *in*: *Discoveries in Australia; with an account of the coasts and rivers explored and surveyed during the voyage of H.M.S.* Beagle, *in the years 1837–38–39–40–41–42–43. By command of the Lords Commissioners of the admiralty. Also a narrative of Captain Owen Stanley’s visits to the islands in the Arafura Sea. By J. Lort Stokes. Vol. I.* T. and W. Boone, London. xii + [2] + 521 pp. + 15 pls. (8vo) [DP: 1846 (title page), 25 April 1846 (*Athenaeum*), April 1846 (Low 1853: 347), 2 May 1846 (*Spectator*), 11 May 1846 (*Monthly Lit Adv*), 15 May 1846 (*Intell Serapeum*), 1 June 1846 (*Monthly List Muquardt*), 6 June 1846 (*Law Times*), April–July 1846 (*Foreign Quart Rev*), July 1846 (*Edinb Rev*)] WID, GB

This work was published in two volumes, issued simultaneously.

**1846**. Appendix. List of Annulosa (principally insects,) found on the journey of Henry H. Methuen, Esq. Drawn up by Adam White, M.E.S., &c. Pp. 307–318 + 2 pls *in*: *Life in the wilderness; or wanderings in South Africa by Henry H. Methuen.* Richard Bentley, London. xii + [1 (Illustrations)] + 318 pp. + 2 pls. (8vo) [DP: 1846 (title page; preface dated July 1846), 25 July 1846 (*Athenaeum*), 1 August 1846 (*Spectator*; *Publ Circ*; *Monthly List Muquardt*), 10 August 1846 (*Monthly Lit Adv*)] GB

The following new beetle species are made available for the first time through a description and/or illustration: *Anthia tatumana* (p. 312), Scarabaeus (Pachylomerus) pearsoni (p. 312; Pl. 1, fig. 2), *Machla sappho* (p. 314; Pl. 2, fig. 2) and *Mecocorynus advena* (p. 315; Pl. 1, fig. 3).

A second edition of this book was issued in October 1848 [GB]. The appendix is identical to the first edition but on pages 353–363.

**1847–1849**. *Nomenclature of coleopterous insects in the collection of the British Museum.* Printed by Order of the Trustees, London. (8vo) CNC

*Part I. Cetoniadae.* [1] + 56 pp. [DP: 1847 (title page; preface dated June 1847), 3 July 1847 (Sherborn 1926a: 272), 26 April 1848 (*Soc Ent Fr*), 1 May 1848 (*Publ Circ*)].

*Part II. – Hydrocanthari.* [2] + 59 pp. [DP: 1847 (title page; preface dated November 1847), 18 December 1847 (Sherborn 1926a: 272), 26 April 1848 (*Soc Ent Fr*), 1 May 1848 (*Publ Circ*), 6 June 1848 (*Acad Nat Sci Phila*)].

*Part III. – Buprestidae.* [1] + 52 pp. [DP: 1848 (title page; preface dated 4 June 1848), 8 July 1848 (Sherborn 1926a: 272)].

*Part IV. Cleridae.* [1] + 68 pp. [DP: 1849 (title page; preface dated June 1849), 21 July 1849 (Sherborn 1926a: 272)].

**1848**. Descriptions of two species of beetles brought from Borneo by Hugh Low, Esq. Pp. 413–416 + 1 pl. *in*: *Sarawak; its inhabitants and productions: being notes during a residence in that country with his excellency Mr. Brooke. By Hugh Low.* Richard Bentley, London. xxiv + 416 pp. + 6 pls. (8vo) [DP: 1848 (title page; preface dated 22 December 1847), 1 January 1848 (*Athenaeum*; *Publ Circ*), 10 January 1848 (*Monthly Lit Adv*), January 1848 (Low 1853: 218), 3 February 1848 (*Bibl Univers*)] GB, BHL

The two species described and illustrated, *Sarothrocera lowii* White and *Cladognathus tarandus* (Thunberg), were already available.

**1851**. List of insects taken by Sir John Richardson and John Rae, Esq., in arctic North America, drawn up by Adam White. Pp. 357–363 *in*: *Arctic searching expedition: a journal of a boat-voyage through Rupert’s land and the arctic sea, in search of the discovery ships under command of Sir John Franklin. With an appendix on the physical geography of North America. By Sir John Richardson, C.B., F.R.S. In two volumes. Vol. II. Published by authority.* Longman, Brown, Green, and Longmans, London. vii + 426 pp. (8vo) [DP: 1851 (title page), 15 November 1851 (*Athenaeum*; *Publ Circ*; *Lit Gaz*), November 1851 (Low 1853: 44)] ARC, GB

This work was reissued in one volume, 1852, by Harper & Brothers, New York [GB]; Adam White’s contribution is on pages 473–478.

**1851**. Descriptions of some apparently new species of annulosa, (collected by Mr. Macgillivray during the voyage of H.M.S. *Rattlesnake*). Pp. 387–395 + pl. 4 *in*: *Narrative of the voyage of H.M.S.* Rattlesnake, *commanded by the late Captain Owen Stanley, R.N., F.R.S. &c. during the years 1846–1850. Including discoveries and surveys in New Guinea, the Louisiade Archipelago, etc. To which is added the account of Mr. E. B. Kennedy’s expedition for the exploration of the Cape York Peninsula. By John Macgillivray, naturalist to the expedition. Published under the sanction of the Lords Commissioners of the Admiralty. In two volumes. Vol. II.* T. & W. Boone, London. iv + [2] + 395 pp. + 7 pls. (8vo) [DP: 1852 (title page), 6 December 1851 (*Athenaeum*; *Lit Gaz*), 12 December 1851 (*Monthly Lit Adv*), 15 December 1851 (*Publ Circ*), December 1851 (Low 1853: 33), 1 January 1852 (*Monthly List Muquardt*)] MCZ, GB

The expedition report was published in two volumes both issued in December 1851, despite the title pages being dated 1852. The only Coleoptera species described is *Pachyrhynchus stanleyanus* (p. 388). A facsimile of the work was published in Adelaide in 1967.

**1853–1855**. *Catalogue of coleopterous insects in the collection of the British Museum.* Printed by Order of the Trustees, London. (8vo) CNC

*Part VII. Longicornia I.* iv + 174 + [1 (Explanation of plates)] pp. + 4 pls. [DP: 1853 (title page; preface dated 10 February 1853), 9 April 1853 (Sherborn 1934: 310), 10 August 1853 (*Soc Ent Fr*)].

*Part VIII. Longicornia II.* [1] + Pp. 175–412 + pls 5–10. [DP: 1855 (title page; preface dated November 1855), 8 December 1855 (Sherborn 1934: 310)].

#### Wiedemann, Christian Rudolph Wilhelm (Brunswick, Lower Saxony, Germany: 7 December 1770 – 31 December 1840: Kiel, Schleswig-Holstein, Germany). German physician, historian and naturalist; professor of anatomy at Brunswick’s Collegium Carolinum; later, professor of medicine at the University of Kiel; his Coleoptera are at the Zoologisches Museum, Universität Hamburg in Germany. References. Nitzsch (1841); Pont (1995); Evenhuis and Pont (2013).

**1824**. *Munus rectoris in Academia Christiana Albertina aditurus analecta entomologica ex Museo Regio Havniensi maxime congesta profert iconibusque illustrat.* E Regio Typographeo Scholarum, Kiliae [= Kiel]. 60 pp. + 1 pl. (4to) [DP: 1824 (title page), 19 June 1824 (*Intell Ztg Welt*), June 1824 (*Allg Lit Ztg*), July 1824 (*Intell Jena Lit Ztg*)] CNC, BHL, GB

One new species of Coleoptera is described in this work, *Harpalus rajah* (pp. 7–8).

#### Wiegmann, Arend Friedrich August (Brunswick, Lower Saxony, Germany: 2 June 1802 – 15 January 1841: Berlin, Germany). German zoologist; professor at Humboldt University in Berlin; founded and edited the journal *Archiv für Naturgeschichte*. References. Anonymous (1841c); Adler (1989: 28–29, P); Evenhuis (1997b: 823, P).

**1832**. [Wiegmann, A.F.A. and Ruthe, J.F.] *Handbuch der Zoologie.* C.G. Lüderitz, Berlin. vi + 621 + [1 (Druckfehler)] pp. (8vo) [DP: 1832 (title page; *Vorrede* dated 31 May 1832), 14 August 1832 (*Intell Ztg Welt*), July–December 1832 (*Verz Bücher Landkarten*), October–December 1832 (*Foreign Quart Rev*)] GB

The Coleoptera are on pages 277–346. A second edition was issued in 1842, and recorded on 26 October 1842 (*Lit Ztg*), despite the title page is dated 1843 [GB]. A third (1848), fourth (1853), fifth (1859) and sixth (1864) editions, all authored by Franz Herman Troschel [1810–1882] and Johann Friedrich Ruthe [1788–1859], were published subsequently.

#### Wilbrand, Johann Bernhard (Clarholz, North Rhine-Westphalia, Germany: 8 March 1779 – 6 May 1846: Giessen, Hesse, Germany). German physician and naturalist; professor of comparative anatomy, physiology and natural history at the University of Giessen and director of the botanical garden in the same city. References. Wilbrand (1831); Hess (1898); Murken (1970, P); Maass (1994, P).

**1829**. *Handbuch der Naturgeschichte des Thierreichs. Nach der verbesserten Linneschen Methode. Nebst einer Tabelle: Uebersicht des Thierreichs.* Georg Friedrich Heyer, Giessen. vii + [1] + 612 pp. (8vo) [DP: 1829 (title page; *Vorrede* dated February 1829), January–June 1829 (*Verz Bücher Landkarten*), 22 August 1829 (*Bibl Deutsch*), August 1829 (*Intell Jena Lit Ztg*)] GB

The Coleoptera are on pages 454–483.

#### Wilhelm, Gottlieb Tobias (Augsburg, Bavaria, Germany: 16 October 1758 – 12 December 1811: Augsburg, Germany). German protestant pastor and naturalist in Augsburg. References. Anonymous (1862a); Pfeuffer (2003: 106–113, P); Anonymous (2014, P).

**1796**. *Unterhaltungen aus der Naturgeschichte. Der Insecten. Erster Theil.* Mart. Engelbrecht, Augsburg. xlviii + 376 pp. + 47 pls. (8vo) [DP: 1796 (title page; *Vorerinnerung* dated July 1796)] GB

The entire series was published in 25 volumes, 1792–1821 (Nissen 1969: 433). The section on insects was issued in three volumes, 1796–1798, and the Coleoptera are on pages 1–236 and plates 1–29 of the first volume. A new edition in 27 volumes (*n.v.*) was issued by Schlosser, also in Augsburg, 1817–1824 (Engelmann 1846: 134).

A French translation in two volumes (probably the first two volumes of the original) was published in Bâle under the title “*Récréations tirées de l’histoire naturelle, traduites de l’allemand, de M. Wilhelm, ministre de la parole de Dieu, à Augsbourg, par le traducteur du Socrate rustique. Classe des insectes*” (*n.v.*), 1799–1800 (Engelmann 1846: 557).

#### Wilken, Carl (Bolzum, Lower Saxony, Germany: 22 October 1825 – 1 June 1882). German professor at the Gymnasium Andreanum in Hildesheim.

**1867**. *Käfer-Fauna Hildesheims. Separatabdruck aus dem Schul-Programm des Gymnasium Andreanum zu Hildesheim vom Jahre 1867.* Gebr. Gerstenberg, Hildesheim. xi + 164 pp. (8vo) [DP: 1867 (title page), 1 June 1867 (*Lit Centr Deutsch*), 18 June 1867 (*Naturwiss Ver Bremen*), July–September 1867 (*Ent Ztg* 28: 319)] GB

This work is an annotated catalogue of the Coleoptera of Hildesheim in Lower Saxony; one new species, *Luperus dispar* (p. 148), is described, though probably unintentionally.

#### Wilmsen, Friedrich Philipp (Magdeburg, Saxony-Anhalt, Germany: 23 February 1770 – 4 May 1831: Berlin, Germany). German protestant pastor in Berlin. References. Anonymous (1833b); Anonymous (1834: 960–962); Sydow (1898: 309–311).

**1821**. *Handbuch der Naturgeschichte für die Jugend und ihre Lehrer. Zweiter Band: Amphibien, Fische und Insekten.* Carl Friedrich Amelang, Berlin. x + 978 pp. (8vo) [DP: 1821 (title page; *Vorrede* dated October 1820), January–June 1821 (*Verz Bücher Landkarten*), September 1821 (*Leip Lit Ztg*), 20 November 1821 (*Intell Ztg Welt*), November 1821 (*Allg Lit Ztg*)] GB

The series consists of three volumes issued in 1821. The Coleoptera are on pages 507–583 of the second volume. A “*zweite verbesserte und vermehrte Auflage*” was published in 1831 by the same publisher [SBB, GB]; in this edition the Coleoptera are on pages 447–527.

#### Wilson, James (Paisley, Scotland, United Kingdom: November 1795 – 18 May 1856: Woodville near Edinburgh, Scotland, United Kingdom). Scottish zoologist of independent means; his collection of insects was purchased by the British Museum (Natural History) in 1868. References. Anonymous (1856b); Hamilton (1859, P); Woodward (1900b); Foote (2004g).

**1834**. [Wilson, J. and Duncan, J.] *Entomologia Edinensis, or a description and history of the insects found in the neighbourhood of Edinburgh. Coleoptera.* William Blackwood, Edinburgh and T. Cadell, London. vi + [2] + 351 pp. + 2 pls. (8vo) [DP: 1834 (title page), 3 February 1834 (*R Soc Edinb*), 8 February 1834 (*Athenaeum*; *Spectator*), 15 February 1834 (*Lit Gaz*), February 1834 (Peddie and Waddington 1914: 642), 1 March 1834 (*Mag Nat Hist* 7: 188), 10 March 1834 (*Monthly Lit Adv*), 19 March 1834 (*Lit Ztg*)] MCZ, GB

**1835**. *A treatise on insects, general and systematic; being the article “Entomology,” from the seventh edition of the Encyclopaedia Britannica. With five hundred and forty figures.* Adam and Charles Black, Edinburgh. 327 pp. + 20 [numbered 220–239] pls. (4to) [DP: 1835 (title page; “Note by the author” dated July 1835), 14 November 1835 (*Athenaeum*), November 1835 (Peddie and Waddington 1914: 642), 26 December 1835 (*Lit Gaz*)] GB

This book consists of the articles “Entomology” (pp. 59–301), “Ant” (pp. 303–308) and “Bee” (pp. 309–327) from the seventh edition of the *Encyclopaedia Britannica* (*n.v.*) preceded by a section entitled “Notices regarding the principal authors in entomology, and their works” (pp. 5–57).

Wilson presented a copy of the book to the Royal Society of Edinburgh on 1 December 1834 (see *Transactions of the Royal Society of Edinburgh* 13: 596) which is surprising considering the July 1835 date in the “Note by the author” (first page, unnumbered) and the recording dates found in journals. Evenhuis (1997b: 828) mentioned that this was most likely a preview copy.

#### Wolf, Johann (Nuremberg, Bavaria, Germany: 26 May 1765 – 12 February 1824: Nuremberg, Germany). German educator and ornithologist in Nuremberg. References. Jordan (1824); Schmidt (1826); Frahm and Egger (2001: 571); Adler (2012: 54–55, P).

**1816–1822**. *Abbildungen und Beschreibungen merkwürdiger naturgeschichtlicher Gegenstände. Mit 36 illuminirten Kupfern.* Conrad Tyroff, Nürnberg. (4to) ANSP, BHL, GB (vol. 1)

[*I. Band*]. 168 + [4] pp. + 36 pls. [DP: 1818 (title page; *Vorrede* dated 28 June 1815)].

*II. Band.* 156 + [4] pp. + 36 pls. [DP: 1822 (title page)].

According to Hagen (1863: 293) this work was issued in 27 *Hefte*. I have seen wrappers for the first 12 *Hefte* dated as follows: **1**: (1–16 + pls 1–3) 1816; **2**: (pp. 17–32 + pls 4–6) 1816; **3**: (pp. 33–44 + pls 7–9) 1816; **4**: (pp. 45–56 + pls 10–12) 1816; **5**: (pp. 57–68 + pls 13–15) 1816; **6**: (pp. 69–84 + pls 16–18) 1816; **7**: (pp. 85–100 + pls 19–21) 1817; **8**: (pp. 101–112 + pls 22–24) 1817; **9**: (pp. 113–128 + pls 25–27) 1817; **10**: (pp. 129–140 + pls 28–30) 1817; **11**: (pp. 141–156 + pls 31–33) 1817; **12**: (pp. 157–168 + pls 34–36) 1818. Collation and dates of publication of each *Heft* for the second volume are desired. The *Hefte* are bound in two volumes and a title page has been issued for each; the dates on the title pages are for the completed volumes. Three species of Coleoptera are described and illustrated in the second volume, including one new species: *Copris lanciger* Fabricius (pp. 79–81), *Cassida punctatissima* Mihi (pp. 81–82) and *Cetonia clavata* Olivier (pp. 83–84). Sherborn (1929: 5253) dated the new species *Cassida punctatissima* in 1819.

#### Wollaston, Thomas Vernon (Scotter, Lincolnshire, United Kingdom: 9 March 1822 – 4 January 1878: Teignmouth, Devon, United Kingdom). British entomologist; his fragile health forced him to spent winters in milder climate and so investigated several North Atlantic islands such as Madeira, Canaries, and Cape Verde; the vast majority of his beetles are deposited at the Natural History Museum in London and the Oxford University Museum. References. Anonymous (1878b); Salmon (2000: 161–162, P); Smetana and Herman (2001: 156–157, P); Foote (2004h); Machado Carillo (2006, P).

**1854**. *Insecta Maderensia; being an account of the insects of the islands of the Madeiran group.* John van Voorst, London. xliii + 634 pp. + 13 pls. (4to) [DP: 1854 (title page; preface dated 14 July 1854), 3 September 1854 (letter of Wollaston at CNC^108^, dated September 4, in which Wollaston mentioned that his *Insecta Maderensia* is “out yesterday”), 4 September 1854 (*Ent Soc Lond*), 9 September 1854 (*Athenaeum*), 4 November 1854 (*Lit Gaz*), July–December 1854 (*Bibliot Hist-Nat*)] CNC, GB

**1857**. *Catalogue of the coleopterous insects of Madeira in the collection of the British Museum.* The Trustees of the British Museum of Natural History, London. xvi + 234 pp. (8vo) [DP: 1857 (title page; preface dated 15 August 1857), 7 September 1857 (*Ent Soc Lond*), 10 October 1857 (Sherborn 1934: 310)] CNC, GB

**1864**. *Catalogue of the coleopterous insects of the Canaries in the collection of the British Museum.* The Trustees of the British Museum of Natural History, London. xiii + 648 pp. (8vo) [DP: 1864 (title page; preface dated 30 January 1864), 25 June 1864 (Sherborn 1934: 310), 4 July 1864 (*Ent Soc Lond*), 5 September 1864 (*Acad Sci Fr*)] CNC, GB

**1865**. *Coleoptera Atlantidum, being an enumeration of the coleopterous insects of the Madeiras, Salvages, and Canaries.* John van Voorst, London. xlvii + 526 + 140 [Appendix, Indexes] pp. (8vo) [DP: 1865 (title page; preface dated 25 October 1865), 30 December 1865 (*Bookseller*; *Lond Rev*), December 1865 (*English Cat Books*), 1 January 1866 (*Ent Soc Lond*), January 1866 (*Allg Bibl*), January–June 1866 (*Bibliot Hist-Nat*)] CNC, GB, BHL

[**1868**]. *Coleoptera Hesperidum, being an enumeration of the coleopterous insects of the Cape Verde Archipelago.* John van Voorst, London. xxxix + 285 pp. (8vo) [DP: 1867 (title page; preface dated 7 December 1867), 1 February 1868 (*Spectator*), 3 February 1868 (*Ent Soc Lond*), 8 February 1868 (*Athenaeum*), 2 March 1868 (*Bookseller*), 16 March 1868 (*Publ Circ*), March 1868 (*Allg Bibl*), January–June 1868 (*Bibliot Hist-Nat*)] MCZ, GB, BHL

Despite the date on the title page, this book was probably published only in 1868 considering the recording entries (publications of the week) in *The Spectator* and *The Athenaeum*.

**1877**. *Coleoptera Sanctae-Helenae.* John van Voorst, London. xxv + 256 pp. + 1 pl. (8vo) [DP: 1877 (title page), 15 December 1877 (*Athenaeum*), 22 December 1877 (*Academy*; *Spectator*), 28 December 1877 (*Lit World-Lond*), December 1877 (*English Cat Books*), 4 January 1878 (*Bookseller*), 16 January 1878 (*Ent Soc Lond*)] CNC, GB, BHL

#### Wood, John George (London, United Kingdom: 21 July 1827 – 3 March 1889: Coventry, West Midlands, United Kingdom). British naturalist, lecturer and writer; chaplain at St. Bartholomew’s Hospital in London (1856–1862); precentor to the Canterbury Diocesan Choral Union (1868–1876). References. Wood (1890, P); Upton (1910); Woodward (2004d, P).

**1871**. *Common British beetles. With illustrations by E. Smith and T.W. Wood.* George Routledge & Sons, London. [1] + 175 + [1] pp. + 12 pls. (12mo) [DP: n.d. (title page), 22 April 1871 (*Athenaeum*; *Spectator*), 1 June 1871 (*Publ Circ*)] MANL

Wood (1890: 83) mentioned that this book was issued in 1870 but the recording entries (publications of the week) in *The Athenaeum* and *The Spectator* suggest that it was published only in 1871. According to Derksen and Scheiding-Göllner (1972: 455), a new edition of this work was issued in 1873 and another one in 1880 (*n.v.*).

**1871**. *Insects at home. Being a popular account of British insects, their structure, habits, and transformations. With upwards of 700 figures by E.A. Smith and J.B. Zwecker, engraved by G. Pearson.* Longmans, Green, and Co., London. xx + 670 pp. + 20 pls. (8vo) [DP: 1872 (title page; preface dated October 1871), 11 November 1871 (*Athenaeum*; *Sat Rev*; *Spectator*), 18 November 1871 (*Punch*), 1 December 1871 (*Publ Circ*; *Lit World-Lond*), 2 December 1871 (*Bookseller*)] CNC, ARC

The Coleoptera are on pages 14–222. The book was also issued in 1872 by Charles Scribner and Company, New York, under the same title except that the word “*British*” is left out [GB, BHL]. It was issued again under the title “*Insects at home: being a popular account of all those insects which are useful or destructive, and minutely describing their structure, habits, and transformations. With upwards of seven hundred figures, by E.A. Smith and J.B. Zwecker; engraved by G. Pearson*” in 1873 [GB] by William Rutter in Philadelphia and in 1883 [GB] and 1884 [BHL] by John B. Alden, New York. A “new edition” with the same number of pages was published by the original publisher in 1892 [GB].

**1874**. *Insects abroad. Being a popular account of foreign insects, their structure, habits, and transformations. Illustrated with six hundred figures, by E.A. Smith and J.B. Zwecker, engraved by G. Pearson.* Longmans, Green, and Co., London. xii + 780 pp. + 20 pls. (8vo) [DP: 1874 (title page; preface dated 9 May 1874), 31 October 1874 (*Athenaeum*; *Sat Rev*; *Spectator*), October 1874 (*English Cat Books*), 2 November 1874 (*Publ Circ*)] BHL, GB

The Coleoptera are on pages 1–274. A “new edition” was issued by the same publisher in 1883 [BHL] and again in 1892 [BHL]; they are essentially identical to the first edition.

#### Wood, William (Kendal, Cumbria, United Kingdom: 22 February 1774 – 26 May 1857: Ruislip, Greater London, United Kingdom). British surgeon, malacologist and entomologist; practiced first in Wingham, near Canterbury, and later in London up to 1814; after, entered into business as a bookseller and publisher, dealing chiefly with natural history works. References. Woodward (1900); Coan and Petit (2011: 1–5).

**1821**. *Illustrations of the Linnaean genera of insects. Vol. I.* W. Wood, London. xvi + xvi + 118 pp. + 42 pls. (12mo) [DP: 1821 (title page), October 1821 (*Quart Rev*; Peddie and Waddington1914: 646), November 1821 (*Christ Obs*), 9 December 1821 (*Gal Lit Gaz*)] GB

The work conists of two volumes, issued simultaneously in 1821. The Coleoptera on pages 1–78 and plates 1–29 in the first volume.

#### Wulfen, Franz Xaver Freiherr von (Belgrade, Serbia: 5 November 1728 – 16 March 1805: Klagenfurt, Carinthia, Austria). Austrian Jesuit priest, botanist, mineralogist and alpinist; school teacher (chiefly of mathematics and physics) in several cities in Europe; discovered the lead molybdate mineral wulfenite. References. Wurzbach (1889: 265–269); Rompel (1913); Klemun (1989, P).

**1786**. *Descriptiones qvorvmdam Capensivm insectorvm.* Wolfgang Walther, Erlangae. xl pp. + 2 pls. (4to) [DP: 1786 (title page; preliminaries dated 14 February 1784), 4 December 1786 (*Allg Lit Ztg*)] MCZ, GB

This work includes the description of several new species of Coleoptera from the Cape Colony, South Africa.

#### Wünsche, Otto (Bautzen, Saxony, Germany: 19 March 1839 – 6 January 1905: Zwickau, Saxony, Germany). German botanist; professor at a public school in Zittau; later, professor at the gymnasium in Zwickau. References. Schorler (1906); Frahm and Egger (2001: 572).

**1895**. *Die verbreitetsten Käfer Deutschlands. Ein Übungsbuch für den naturwissenschaftlichen Unterricht. Mit 2 Tafeln.* B.G. Teubner, Leipzig. xvi + 212 pp. + 2 pls. (8vo) [DP: 1895 (title page; *Vorwort* dated 24 May 1895), 17 August 1895 (*Lit Centr Deutsch*; *Deutsch Litt*), September 1895 (*Nat Nov*), 12 October 1895 (*Naturwiss Rund*), 20 October 1895 (*Deutsch Forst-Ztg*), October 1895 (*Zeit Forst Jagd*), 10 November 1895 (*Naturwiss Wochen*), 25 December 1895 (*Wien Ent Ztg* 14: 303)] YB

#### Wüstnei, Wilhelm Heinrich Carl [Karl] Franz (Schwerin, Mecklenburg-West Pomerania, Germany: 9 May 1839 – 27 April 1907: Sønderborg, Denmark). German educator; professor in Sønderborg (1874–1904). Reference. Wullenweber (1922).

**1886–1887**. *Verzeichnis der in näheren Umgebung Sonderburgs bisher aufgefundenen Käfer. Beilage zum Programm des Königlichen Realprogymnasiums zu Sonderburg.* Sonderburg. (8vo) ULBD

*(Erste Hälfte.)*. 30 pp. [DP: 1886 (title page; preliminaries dated end of February 1886), 8 May 1886 (*Lit Centr Deutsch*), 9 October 1886 (*Deutsch Litt*)].

*(Zweite Hälfte.)*. Pp. 35–56. [DP: 1887 (title page; last page dated end of February 1887), 23 April 1887 (*Lit Centr Deutsch*), July–September 1887 (*Bibliot Hist-Nat*)].

This is a list, with short annotations for some species, of the beetles in the vicinity of Sønderborg, Denmark, which was under German rule 1864–1920.

#### Yeats, Thomas Pattinson (? – 17 August 1782: Liverpool, Merseyside, United Kingdom). British naturalist; felt into the sea from a wharf and drowned; his collections were sold at auction in May 1783. References. Mendes de Costa (1812: 515); Gorton (1833).

**1773**. *Institutions of entomology: being a translation of Linnaeus’s ordines et genera insectorum; or, systematic arrangement of insects. Collated with the different systems of Geoffroy, Schaeffer and Scopoli; together with observations of the translator.* R. Horsfield, London. viii + 272 pp. (8vo) [DP: 1773 (title page), July 1773 (*Univers Mag*), November 1773 (*Monthly Rev*; *Scots Mag*), December 1773 (*Crit Rev*)] BHL

This work consists mainly of morphological descriptions of genera with notes. The Coleoptera are on pages 26–95.

#### Zenker, Jonathan Carl [Karl] (Sundremda, Thuringia, Germany: 1 March 1799 – 6 November 1837: Jena, Thuringia, Germany). German physician and naturalist; professor of botany and natural history at the University of Jena. References. Zenker (1839); Hess (1900); Frahm and Eggers (2001: 576).

**1828**. *Das thierische Leben und sein Formen. Ein zoologische Handbuch zum Gebrauche academischer Vorträge und zum Selbststudium.* Cröker, Jena. xxiv + 720 + [10] pp. (8vo) [DP: 1828 (title page; *Vorerinnerung* dated 4 July 1828), September 1828 (*Intell Jena Lit Ztg*), July–December 1828 (*Verz Bücher Landkarten*)] GB

The Coleoptera are on pages 470–491.

**1836**. *Naturgeschichte schädlicher Thiere. Versuch einer naturhistorischen Darstellung der für Oekonomie, Gärtnerey und Forstwirthschaft wichtigsten schädlichen Thiere Deutschlands, nebst den zweckmässigsten Mitteln zu ihrer Vertilgung oder Vertreibung. Mit sechszehn illuminirten Kupfertafeln in Quart, und einem vollständigen Realregister. Ein integrirender Theil der allgemeinen Encyklopädie der gesammten Land- und Hauswirthschaft der Deutschen.* Baumgärtner, Leipzig. xxviii + [1] + 406 pp. (8vo) [DP: 1836 (title page), 30 September 1836 (*Allg Bibl Deutsch*), 12 November 1836 (*Allg Ztg*)] GB, MDZ

The Coleoptera are on pages 307–351. An atlas (4to) with 16 plates was also issued in 1836.

#### Zetterstedt, Johann Wilhelm (Mjölby, Östergötland, Sweden: 20 May 1785 – 23 December 1874: Lund, Sweden). Swedish naturalist; explorer of northern Sweden; professor of botany and economy at the University of Lund; the whereabouts of his Coleoptera are unknown. References. Anonymous (1856a: 77–81); Dohrn (1875); Evenhuis (1997b: 836, P); Smetana and Herman (2001: 157–158, P).

**1828**. *Fauna insectorum Lapponica. Pars I.* Schulz, Hammone [= Hamburg]. xx + [1] + 563 pp. (8vo) [DP: 1828 (title page; *praefatio* dated 2 January 1826), 4 April 1829 (*Bibl Deutsch*), April–June 1829 (*Foreign Quart Rev*), January–June 1829 (*Verz Bücher Landkarten*)] CNC, GB

This publication concerned the Coleoptera (pp. 1–440), Orthoptera and Hemiptera. It was reissued in 1844 under the title “*Coleoptera, Orthoptera et Hemiptera: faunae insectorum Lapponicae*” [GB].

**1837–1840**. *Insecta Lapponica.* Leopoldi Voss, Lipsiae [= Leipzig]. vi + [2] + 10–1139 + [1] columns. (4to) CNC, BHL

This work was published in six *Hefte* as follows: **1**: (columns 1–192) August 1837 (*Isis*, Heft VIII: [2]), 6 October 1837 (*Allg Bibl Deutsch*), 11 October 1837 (*Lit Ztg*), July–December 1837 (*Verz Bücher Landkarten*); **2**: (columns 193–384) 22 December 1837 (*Allg Bibl Deutsch*), 3 January 1838 (*Lit Ztg*); **3**: (columns 385–576) March 1838 (*Isis*, Heft III: [3]), 6 April 1838 (*Allg Bibl Deutsch*), 18 April 1838 (*Lit Ztg*), January–June 1838 (*Verz Bücher Landkarten*); **4**: (columns 577–768) 17 August 1838 (*Allg Bibl Deutsch*), 29 August 1838 (*Lit Ztg*), July–December 1838 (*Verz Bücher Landkarten*); **5**: (columns 769–960) 1 November 1839 (*Allg Bibl Deutsch*), July–December 1839 (*Verz Bücher Landkarten*); **6**: (columns 961–1139 + [1]) 7 February 1840 (*Allg Bibl Deutsch*), 12 February 1840 (*Lit Ztg*), 29 February 1840 (*Intell Serapeum*). The title page is dated 1840 and the *praefamen* 3 March 1837. The vast majority of pages are in double columns, each numbered; the other ones, even in single column, are double numbered. The Coleoptera are in columns 10–240. The collation and dates of publication noted by Sherborn (1922: cxxxi) and Evenhuis (1997b: 839) could not be confirmed in this study.

#### Zimmermann, Karl Christoph [Christian] Andreas (Quedlinburg, Saxony-Anhalt, Germany: 6 September 1800 – December 1867). German musician (composer), naturalist, and teacher; left Hamburg in August 1832 for Philadelphia and soon moved to South Carolina (on foot) where he taught music and drawings; his collection was bought by Dr. Lewis (Philadelphia) and thence by R. Crotch who sold it to the Museum of Comparative Zoology in Cambridge, Massachusetts. References. Hagen (1889); Weidner (1983: 282).

**1831**. *Monographie der Carabiden. Erstes Stück.* Berlin [&] Halle. viii + 76 pp. (8vo) [DP: 1831 (title page; *Vorwort* dated June 1831), October–December 1831 (*Foreign Quart Rev*), July–December 1831 (*Verz Bücher Landkarten*), April–June 1832 (*Soc Ent Fr* [*Annales*, p. 244])] MCZ, GB

**1831**. *Monographia amaroidum*. 48 + 48 pp. (8vo) [DP: 1831 (Hagen 1889: 71)] MCZ

[*Part I*]. 48 pp. This part includes a general description of the family Amaroides (pp. 1–35); key to a general division of the Coleoptera (pp. 36–39); de corporis partibus externis (pp. 40–43); general description of Amaroides (pp. 44–48).

[*Part II*]. 48 pp. This part includes the characters of the Amaroides (pp. 1–11); key to genera (p. 11–12); treatment of *Leirus*, Megerle including lengthy description of the genus and of all the species (pp. 12–30); treatment of *Leioscelis*, Mihi including description of the genus and species except that the last one is incomplete and cut short (pp. 31–48 with pp. 33–48 incorrectly numbered 49–60).

According to Hagen (1889: 71–72), this work, written in Latin, exists only in proof sheets. As such, it does not constitute a published work within the meaning of the ICZN (1999: Article 9).

#### Zinke, Georg Gottfried (1771 – 1813/1849^109^). German physician and naturalist in Kahla, near Jena; proved the infectious nature of rabies.

**1798**. *Naturgeschichte der schädlichen Nadelholz-Insecten nebst Anweisung zu ihrer Vertilgung. Ein nützliches Lesebuch für Naturforscher, Forstmänner und Oekonomen. Mit ausgemahlten Kupfern.* Industries-Comptoirs, Weimar. 126 pp. + 5 pls. (8vo) [DP: 1798 (title page; *Vorbericht* dated 1 December 1797), 29 April 1798^110^, 25 June 1798 (*Reichs-Anz*)] GB, SDL

The Coleoptera are on pages 15–50. Hagen (1863: 305) mentioned that the work includes two plates; both digitized copies seen had five plates. The work was also issued the same year in the first *Band* of Johann Jacob von Lincker’s “*Der besorgte Forstmann. Eine Zeitschrift über Verderbniss der Wälder durch Thiere, und vorzüglich Insecten überhaupt, besonders aber durch die jetzt in Teutschland herrschenden Kiefer- Fichten- Tannen- und Birkenraupen*” on pages 25–82 [DP: 28 February 1798 (*Intell Lit Ztg*)] and 137–204 [DP: 7 April 1798 (*Intell Lit Ztg*)].

#### Zschach, Johann Jacob [Jakob] (Leipzig, Saxony, Germany: 1737 – 8 June 1809: Leipzig, Germany). Curator of the Museum Leskeanum.

**1788**. *Museum N. G. Leskeanum. Pars entomologica ad systema entomologiae Cl. Fabricii ordinata. Cum tab. aen. pictis.* J.G. Müller, Lipsiae [= Leipzig]. [2] + 136 pp. + 3 pls. (8vo) [DP: 1788 (title page), 14 July 1788 (*Gött Anz*), 15 August 1788 (*Alg Konst Letter*), October 1788 (*Esprit J*), 10 November 1788 (*Allg Lit Ztg*)] ANSP, GB

This is a catalogue of the collection of natural history objects formed by Nathaniel Gottfried Leske. Most of the new species (identified by an asterisk) in this work are described but not named; those described in Coleoptera are seven species of *Donacia* (pp. 27–28). Unnamed new species were named by Gmelin 1790 [*q.v.*]. This work also appeared in “*Mvsevm Leskeanvm. Regnvm Animale. Qvod ordine systematico disposvit atqve descripsit D.L. Gvstavvs Karsten. Vol. I. Cum IX. iconibus pictis*” [GB] published in 1789 (see Vane-Wright 1976: 20).

### Dictionaries

***Allgemeine Encyclopädie*** *der Wissenschaften und Künste in alphabetischer Folge von genannten Schriftstellern bearbeitet und herausgegeben von J.S. Ersch und J.G. Gruber.* [167 volumes]. 1818–1889. Johann Friedrich Gleditsch [later F.A. Brockhaus], Leipzig. (4to) GB

*Erster Theil mit Kupfern und Charten. A – Aëtius.* xv + 482 pp. + 9 pls. [DP^111^: 1818 (title page; *Vorbericht* dated August 1818)]. This volume was published in two parts: (pp. 1–264) 14 August 1818 (*Leip Lit Ztg*); (pp. 265–482) October 1818 (*Allg Lit Ztg*), 24 December 1818 (*Leip Lit Ztg*)].

*Zweiter Theil mit Kupfern und Charten. Äga – Aldus.* lii + [18] + 430 pp. + 3 pls. [DP: 1819 (title page), 24 July 1819 (*Leip Lit Ztg*), July 1819 (*Allg Lit Ztg*)].

*Dritter Theil mit Kupfern und Charten. Ale – Anax.* 484 pp. + 5 pls. [DP: 1819 (title page), January 1820 (*Allg Lit Ztg*), January–June 1820 (*Verz Bücher Landkarten*)].

*Vierter Theil mit Kupfern und Charten. Anaxagoras – Appel.* 475 pp. + 3 pls. [DP: 1820 (title page), April 1820 (*Allg Lit Ztg*), 20 May 1820 (*Leip Lit Ztg*), January–June 1820 (*Verz Bücher Landkarten*)].

*Fünfter Theil mit Kupfern und Charten. Appellation – Arzilla.* 469 pp. + 3 pls. [DP: 1820 (title page), November 1820 (*Allg Lit Ztg*), July–December 1820 (*Verz Bücher Landkarten*)].

*Sechster Theil mit Kupfern und Charten. Arzneikunde – Azzolini.* 528 pp. + 3 pls. [DP: 1821 (title page), April 1821 (Evenhuis 1997a: 234), May 1821 (*Allg Lit Ztg*), July–December 1821 (*Verz Bücher Landkarten*)].

*Siebenter Theil mit Kupfern und Charten. B – Barzelletten.* 472 pp. + 7 pls. [DP: 1821 (title page), 5 December 1821 (Evenhuis 1997a: 234), July–December 1821 (*Verz Bücher Landkarten*), January 1822 (*Allg Lit Ztg*)].

*Achter Theil mit Kupfern und Charten. Bas – Bendorf.* 478 + [1] pp. + 1 pl. [DP: 1822 (title page), 13 April 1822 (*Neue Spey Ztg*), January–June 1822 (*Verz Bücher Landkarten*)].

*Neunter Theil mit Kupfern und Charten. Bene – Bibeh.* 416 pp. + 6 pls. [DP: 1822 (title page), December 1822 (*Allg Lit Ztg*), January–June 1823 (*Verz Bücher Landkarten*)].

*Zehnter Theil mit Kupfern und Charten. Bibel – Blei.* 419 pp. + 6 pls. [DP: 1823 (title page), June 1823 (*Allg Lit Ztg*), January–June 1823 (*Verz Bücher Landkarten*)].

*Eilfter Theil mit Kupfern und Charten. Bleiberg – Bonzen.* 420 pp. [DP: 1823 (title page), December 1823 (*Allg Lit Ztg*), January–June 1824 (*Verz Bücher Landkarten*)].

*Zwölfter Theil mit Kupfern und Charten. Boochanpoor – Brezow.* 458 + [2] pp. + 1 pl. [DP: 1824 (title page), January–June 1824 (*Verz Bücher Landkarten*)].

*Dreizehnter Theil mit Kupfern und Charten. Briänsk – Bukuresd*. [4] + 421 pp. + 4 pls. [DP: 1824 (title page), February 1825 (*Allg Lit Ztg*), January–June 1825 (*Verz Bücher Landkarten*)].

*Vierzehnter Theil mit Kupfern und Charten. Bulacan – Calza.* 240 + [1] + 183 pp. + 3 pls. [DP: 1825 (title page), January–June 1825 (*Verz Bücher Landkarten*), September 1825 (Evenhuis 1997a: 234)].

*Funfzehnter Theil mit Kupfern und Charten. Camaldulenser – Cazouls les Beziers.* 421 pp. + 1 pl. [DP: 1826 (title page), January–June 1826 (*Verz Bücher Landkarten*), September 1826 (*Allg Lit Ztg*)].

*Sechszehnter Theil mit Kupfern und Charten. Cea – Chiny.* [1] + 386 pp. + 2 pls. [DP: 1827 (title page; p. [1] dated March 1827), April 1827 (*Allg Ztg*), July–December 1827 (*Verz Bücher Landkarten*)].

*Siebzehnter Theil mit Kupfern und Charten. Chiococca – Claytonia.* [2] + 424 pp. + 3 pls. [DP: 1828 (title page; *Vorrede* dated 30 March 1828), January–June 1828 (*Verz Bücher Landkarten*)].

*Achtzehnter Theil mit Kupfern und Charten. Clearfield – Comum.* [2] + 402 + [4] pp. + 3 pls. [DP: 1828 (title page; *Vorrede* dated November 1828), January–June 1829 (*Verz Bücher Landkarten*)].

*Neunzehnter Theil mit Kupfern und Charten. Conami – Corythus.* 399 pp. + 5 pls. [DP: 1829 (title page), July–December 1829 (*Verz Bücher Landkarten*)].

*Zwanzigster Theil mit Kupfern und Charten. Cos – Czvittinger.* 458 pp. + 3 pls. [DP: 1829 (title page), January–June 1830 (*Verz Bücher Landkarten*)].

*Ein und zwanzigster Theil mit Kupfern und Charten. Nachträge: Caberea – Cryptostoma.* [2] + 473 pp. + 6 pls. [DP: 1830 (title page; *Vorwort* dated August 1830), January–June 1831 (*Verz Bücher Landkarten*)].

*Erste Section. A – G. Herausgegeben von J.G. Gruber. Zweiundzwanzigster Theil. Nachträge: Carlowitz – Cyrillus und D – Dani.* 157 + 244 pp. + 11 pls. [DP: 1832 (title page), 28 July 1832 (*Bibl Deutsch*), August 1832 (Evenhuis 1997a: 235), July–December 1832 (*Verz Bücher Landkarten*)].

*Erste Section. A – G. Herausgegeben von J.G. Gruber. Dreiundzwanzigster Theil. Daniel – Demeter.* 467 pp. + 7 pls. [DP: 1832 (title page), October–December 1832 (Evenhuis 1997a: 235), 9 February 1833 (*Bibl Deutsch*), January–March 1833 (*Foreign Quart Rev*)].

*Erste Section. A – G. Herausgegeben von J.G. Gruber. Vierundzwanzigster Theil. Demetria – Didymus.* 554 pp. [DP: 1833 (title page), 23 December 1833 (*Bibl Deutsch*), January–June 1834 (*Verz Bücher Landkarten*)].

*Erste Section. A – G. Herausgegeben von J.G. Gruber. Fünfundzwanzigster Theil. Die – Dipyr.* 480 pp. [DP: 1834 (title page), December 1834 (Evenhuis 1997a: 235), July–December 1834 (*Verz Bücher Landkarten*)].

*Erste Section. A – G. Herausgegeben von J.G. Gruber. Sechsundzwanzigster Theil. Dir – Dominium Mundi.* 508 pp. [DP: 1835 (title page), 1 October 1835 (*Bibl Deutsch*), July–December 1835 (*Verz Bücher Landkarten*)].

*Erste Section. A – G. Herausgegeben von J.G. Gruber. Siebenundzwanzigster Theil. Dominus – Drury.* 507 pp. + 2 pls. [DP: 1836 (title page), 27 May 1836 (*Allg Bibl Deutsch*), May 1836 (*Bull Litt Étr*), January–June 1836 (*Verz Bücher Landkarten*)].

*Erste Section. A – G. Herausgegeben von J.G. Gruber. Achtundzwanzigster Theil. Drus – Dziewonna.* 492 pp. + 1 pl. [DP: 1836 (title page), 13 January 1837 (*Allg Bibl Deutsch*), 25 January 1837 (*Lit Ztg*), January–June 1837 (*Verz Bücher Landkarten*)].

*Erste Section. A – G. Herausgegeben von J.G. Gruber. Neunundzwanzigster Theil. Nachträge: Dacia – Dziura-Wiatrzina und E – Ebergassing.* 370 + 98 pp. + 3 pls. [DP: 1837 (title page), 15 December 1837 (*Allg Bibl Deutsch*), 23 December 1837 (*Lit Ztg*)].

*Erste Section. A – G. Herausgegeben von J.G. Gruber. Dreißigster Theil. Eberhard – Ecklonia.* 504 pp. [DP: 1838 (title page), 28 September 1838 (*Allg Bibl Deutsch*), July–September 1838 (Evenhuis 1997a: 235)].

*Erste Section. A – G. Herausgegeben von J.G. Gruber. Einunddreißigster Theil. Eckmühl – Ehstland.* 479 pp. [DP: 1838 (title page), October–December 1838 (Evenhuis 1997a: 235), 18 January 1839 (*Allg Bibl Deutsch*)].

*Erste Section. A – G. Herausgegeben von J.G. Gruber. Zweiunddreißigster Theil. Ei – Eisen.* 490 pp. + 3 pls. [DP: 1839 (title page), 27 September 1839 (*Allg Bibl Deutsch*), July–September 1839 (Evenhuis 1997a: 235)].

*Erste Section. A – G. Herausgegeben von J.G. Gruber. Dreiunddreißigster Theil. Eisenach – Elzheimer.* 485 pp. + 4 pls. [DP: 1840 (title page), 10 July 1840 (*Allg Bibl Deutsch*), October–December 1840 (Evenhuis 1997a: 235)].

*Erste Section. A – G. Herausgegeben von J.G. Gruber. Vierunddreißigster Theil. Em – Enstasis.* 485 pp. [DP: 1840 (title page), 4 December 1840 (*Allg Bibl Deutsch*), October–December 1840 (Evenhuis 1997a: 235)].

*Erste Section. A – G. Herausgegeben von J.G. Gruber. Fünfunddreißigster Theil. Ent – Epilogus.* 479 pp. + 3 pls. [DP: 1841 (title page), 29 October 1841 (*Allg Bibl Deutsch*), October–December 1841 (Evenhuis 1997a: 235), July–December 1841 (*Verz Bücher Landkarten*)].

*Erste Section. A – G. Herausgegeben von J.G. Gruber. Sechsunddreißigster Theil. Epimachus – Ergyne.* 481 + [2] pp. + 3 pls. [DP: 1842 (title page), 29 July 1842 (*Allg Bibl Deutsch*), July–September 1842 (Evenhuis 1997a: 235)].

*Erste Section. A – G. Herausgegeben von J.G. Gruber. Siebenunddreißigster Theil. Erhaben – Erz- und Erbtruchsesse.* 502 pp. [DP: 1842 (title page), 23 December 1842 (*Allg Bibl Deutsch*), October–December 1842 (Evenhuis 1997a: 235)].

*Erste Section. A – G. Herausgegeben von J.G. Gruber. Achtunddreißigster Theil. Es – Euganei.* 470 pp. [DP: 1843 (title page), 7 December 1843 (*Allg Bibl Deutsch*), 15 December 1843 (*Intell Serapeum*), 26 January 1844 (*Leip Reper*)].

*Erste Section. A – G. Herausgegeben von J.G. Gruber. Neununddreißigster Theil. Eugen – Ezzelino.* 492 pp. [DP: 1843 (title page), 7 December 1843 (*Allg Bibl Deutsch*), 15 December 1843 (*Intell Serapeum*), October–December 1843 (Evenhuis 1997a: 235), 26 January 1844 (*Leip Reper*)].

*Erste Section. A – G. Herausgegeben von J.G. Gruber. Vierzigster Theil. Nachträge: Eccard – Exeter und F – Fabricius.* 453 + 80 pp. + 3 pls. [DP: 1844 (title page), October–December 1844 (Evenhuis 1997a: 235), 9 January 1845 (*Allg Bibl Deutsch*), 15 January 1845 (*Intell Serapeum*), 25 January 1845 (*Lit Ztg*)].

*Erste Section. A – G. Herausgegeben von J.G. Gruber. Einundvierzigster Theil. Fabrik – Farvel.* 469 pp. [DP: 1845 (title page), 28 August 1845 (*Allg Bibl Deutsch*), 15 September 1845 (*Intell Serapeum*), 17 September 1845 (*Lit Ztg*), 19 September 1845 (*Leip Reper*)].

*Erste Section. A – G. Herausgegeben von J.G. Gruber. Zweiundvierzigster Theil. Fas – Ferchard.* 464 pp. + 1 pl. [DP: 1845 (title page), 18 December 1845 (*Allg Bibl Deutsch*), 31 December 1845 (*Lit Ztg*), October–December 1845 (Evenhuis 1997a: 235), 31 January 1846 (*Blätt Lit Unterhalt*)].

*Erste Section. A – G. Herausgegeben von F.G. Gruber. Dreiundvierzigster Theil. Ferdinand I. – Fichtentinctur.* 483 pp. + 3 pls. [DP: 1846 (title page), 20 August 1846 (*Allg Bibl Deutsch*), August 1846 (*Intell Allg Lit Ztg*), 18 September 1846 (*Leip Reper*), July–September 1846 (Evenhuis 1997a: 235)].

*Erste Section. A – G. Herausgegeben von F.G. Gruber. Vierundvierzigster Theil. Ficinus – Fizes.* 470 pp. [DP: 1846 (title page), 17 December 1846 (*Allg Bibl Deutsch*), 15 January 1847 (*Leip Reper*)].

*Erste Section. A – G. Herausgegeben von F.G. Gruber. Fünfundvierzigster Theil. Flaach – Flustra.* 480 pp. [DP: 1847 (title page), 15 December 1847 (*Intell Serapeum*), 16 December 1847 (*Allg Bibl Deutsch*), October–December 1847 (Evenhuis 1997a: 235), 28 January 1848 (*Leip Reper*)] .

*Erste Section. A – G. Herausgegeben von F.G. Gruber. Sechsundvierzigster Theil. Fluth und Ebbe – Fortunius.* 491 pp. [DP: 1847 (title page), 15 December 1847 (*Intell Serapeum*), 16 December 1847 (*Allg Bibl Deutsch*), 28 January 1848 (*Leip Reper*)].

*Erste Section. A – G. Herausgegeben von F.G. Gruber. Siebenundvierzigster Theil. Foruli – Franz Regis (der Heilige).* 474 pp. [DP: 1848 (title page), 28 December 1848 (*Allg Bibl Deutsch*), 4 January 1849 (*Lit Ztg*), 31 January 1849 (*Intell Serapeum*)].

*Erste Section. A – G. Herausgegeben von F.G. Gruber. Achtundvierzigster Theil. Franz I. – Freiburg.* 480 pp. [DP: 1848 (title page), 28 December 1848 (*Allg Bibl Deutsch*), 4 January 1849 (*Lit Ztg*), 31 January 1849 (*Intell Serapeum*)].

*Erste Section. A – G. Herausgegeben von F.G. Gruber. Neunundvierzigster Theil. Freidhoff – Friedrich (Bischöfe).* 459 pp. [DP: 1849 (title page), October–December 1849 (Evenhuis 1997a: 235), 15 January 1850 (*Intell Serapeum*), 28 February 1850 (*Bibl Lit Anz*)].

*Erste Section. A – G. Herausgegeben J.G. Gruber. Funfzigster Theil. Friedrich (Fürsten) – Fuker.* 469 pp. [DP: 1849 (title page), October–December 1849 (Evenhuis 1997a: 235), 15 January 1850 (*Intell Serapeum*)].

*Erste Section. A – G. Herausgegeben J.G. Gruber. Einundfunfzigster Theil. Fulcher – Fyzabad. Nachträge: Farel – Fuss.* 550 pp. [DP: 1850 (title page), 26 December 1850 (*Allg Bibl Deutsch*), October–December 1850 (Evenhuis 1997a: 235), 15 January 1851 (*Intell Serapeum*)].

*Erste Section. A – G. Herausgegeben von J.G. Gruber. Zweiundfunfzigster Theil. G – Gallatin.* 436 pp. [DP: 1851 (title page), October–December 1851 (Evenhuis 1997a: 235), 8 January 1852 (*Allg Bibl Deutsch*), 31 January 1852 (*Intell Serapeum*), January–March 1852 (*Cat Muquardt*)].

*Erste Section. A – G. Herausgegeben von J.G. Gruber. Dreiundfunfzigster Theil. Galle – Garet.* 471 pp. [DP: 1851 (title page), October–December 1851 (Evenhuis 1997a: 235), 8 January 1852 (*Allg Bibl Deutsch*), 31 January 1852 (*Intell Serapeum*), January–March 1852 (*Cat Muquardt*)].

*Erste Section. A – G. Herausgegeben von J.G. Gruber. Vierundfunfzigster Theil. Gargano – Gauhe.* 469 pp. [DP: 1852 (title page), 13 January 1853 (*Allg Bibl Deutsch*)].

*Erste Section. A – G. Herausgegeben von M.H.E. Meier. Fünfundfunfzigster Theil. Gaukes – Gefreiter.* 472 pp. [DP: 1852 (title page), December 1852 (Evenhuis 1997a: 235), 13 January 1853 (*Allg Bibl Deutsch*)].

*Erste Section. A – G. Herausgegeben von M.H.E. Meier. Sechsundfunfzigster Theil. Gefühl – Gellenau.* 461 pp. [DP: 1853 (title page), 29 December 1853 (*Allg Bibl Deutsch*), October–December 1853 (*Cat Muquardt*)].

*Erste Section. A – G. Herausgegeben von M.H.E. Meier. Siebenundfunfzigster Theil. Gellert – Genezareth.* 437 pp. [DP: 1853 (title page), 29 December 1853 (*Allg Bibl Deutsch*), October–December 1853 (*Cat Muquardt*)].

*Erste Section. A – G. Herausgegeben von M.H.E. Meier. Achtundfunfzigster Theil. Genf – Genzano.* 495 pp. [DP: 1854 (title page), April–June 1855 (*Cat Muquardt*)].

*Erste Section. A – G. Herausgegeben von M.H.E. Meier. Neunundfunfzigster Theil. Geoaris – Georg III. (König von England).* 478 pp. + 3 pls. [DP: 1854 (title page), December 1854 (Evenhuis 1997a: 235), April–June 1855 (*Cat Muquardt*)].

*Erste Section. A – G. Herausgegeben von M.H.E. Meier. Sechzigster Theil. Georg IV. (König von England) – Gerhard.* 477 pp. [DP: 1855 (title page), 8 November 1855 (*Allg Bibl Deutsch*), December 1855 (Evenhuis 1997a: 235), October–December 1855 (*Cat Muquardt*)].

*Erste Section. A – G. Herausgegeben von M.H.E. Meier. Einundsechzigster Theil. Gerhardinger – Gersdorffsburg.* 477 pp. [DP: 1855 (title page), 20 December 1855 (*Allg Bibl Deutsch*), December 1855 (Evenhuis 1997a: 235), October–December 1855 (*Cat Muquardt*)].

*Erste Section. A – G. Herausgegeben von Hermann Brockhaus. Zweiundsechzigster Theil. Gersen – Geschlecht.* 462 pp. [DP: 1856 (title page), 18 December 1856 (*Allg Bibl Deutsch*), October–December 1856 (*Cat Muquardt*)].

*Erste Section. A – G. Herausgegeben von Hermann Brockhaus. Dreiundsechzigster Theil. Geschlechtsapparat – Gesen.* 477 pp. [DP: 1856 (title page), 18 December 1856 (*Allg Bibl Deutsch*), October–December 1856 (*Cat Muquardt*)].

*Erste Section. A – G. Herausgegeben von Hermann Brockhaus. Vierundsechzigster Theil. Gesenius – Getränk.* 469 pp. [DP: 1857 (title page), November 1857 (Evenhuis 1997a: 235), 3 December 1857 (*Allg Bibl Deutsch*), February 1858 (*Allg Bibl*)].

*Erste Section. A – G. Herausgegeben von Hermann Brockhaus. Fünfundsechzigster Theil. Getreide – Gewerke.* 488 pp. [DP: 1857 (title page), November 1857 (Evenhuis 1997a: 235), 3 December 1857 (*Allg Bibl Deutsch*), February 1858 (*Allg Bibl*)].

*Erste Section. A – G. Herausgegeben von Hermann Brockhaus. Sechsundsechzigster Theil. Gewicht – Gidom.* 461 pp. [DP: 1857 (title page), December 1857 (Evenhuis 1997a: 236), 14 January 1858 (*Allg Bibl Deutsch*), February 1858 (*Allg Bibl*), 15 April 1858 (*Intell Serapeum*)].

*Erste Section. A – G. Herausgegeben von Hermann Brockhaus. Siebenundsechzigster Theil. Gie – Girieux.* 451 pp. [DP: 1858 (title page), 2 December 1858 (*Allg Bibl Deutsch*), 31 December 1858 (*Intell Serapeum*)].

*Erste Section. A – G. Herausgegeben von Hermann Brockhaus. Achtundsechzigster Theil. Giro – Glarus.* 499 pp. + 1 pl. [DP: 1859 (title page), 7 July 1859 (*Allg Bibl Deutsch*), 31 August 1859 (*Intell Serapeum*)].

*Erste Section. A – G. Herausgegeben von Hermann Brockhaus. Neunundsechzigster Theil. Glas – Gliers.* 468 pp. [DP: 1859 (title page), 1 December 1859 (*Allg Bibl Deutsch*)].

*Erste Section. A – G. Herausgegeben von Hermann Brockhaus. Siebzigster Theil. Glimes – Gnandstein.* 471 pp. [DP: 1860 (title page), 5 July 1860 (*Allg Bibl Deutsch*), August 1860 (*Allg Bibl*)].

*Erste Section. A – G. Herausgegeben von Hermann Brockhaus. Einundsiebzigster Theil. Gnaphalieen – God Save the King*! 454 pp. [DP: 1860 (title page), 6 December 1860 (*Allg Bibl Deutsch*), January 1861 (*Allg Bibl*)].

*Erste Section. A – G. Herausgegeben von Hermann Brockhaus. Zweiundsiebzigster Theil. Godunow – Götz.* 455 pp. [DP: 1861 (title page), 11 July 1861 (*Allg Bibl Deutsch*), August 1861 (*Allg Bibl*)].

*Erste Section. A – G. Herausgegeben von Hermann Brockhaus. Dreiundsiebzigster Theil. Götze – Gondouin.* 476 pp. [DP: 1861 (title page), 5 December 1861 (*Allg Bibl Deutsch*), 31 December 1861 (*Intell Serapeum*), December 1861 (*Allg Bibl*)].

*Erste Section. A – G. Herausgegeben von Hermann Brockhaus. Vierundsiebzigster Theil. Gondrai – Gorzubitai.* 460 pp. [DP: 1862 (title page), 7 August 1862 (*Allg Bibl Deutsch*)].

*Erste Section. A – G. Herausgegeben von Hermann Brockhaus. Fünfundsiebzigster Theil. Nebst einer lithographirten Tafel. Gosa – Gott.* 479 pp. + 1 pl. [DP: 1862 (title page), 8 January 1863 (*Allg Bibl Deutsch*), 15 January 1863 (*Intell Serapeum*)].

*Erste Section. A – G. Herausgegeben von Hermann Brockhaus. Sechsundsiebzigster Theil. Gottähnlichkeit – Graaf.* 444 pp. [DP: 1863 (title page), 17 December 1863 (*Allg Bibl Deutsch*), January 1864 (*Allg Bibl*)].

*Erste Section. A – G. Herausgegeben von Hermann Brockhaus. Siebenundsiebzigster Theil. Graagaas – Gradisca.* 467 pp. [DP: 1864 (title page), 15 December 1864 (*Intell Serapeum*), 29 December 1864 (*Allg Bibl Deutsch*)].

*Erste Section. A – G. Herausgegeben von Hermann Brockhaus. Achtundsiebzigster Theil. Gradiscaner Krieg – Grammatico.* 467 pp. [DP: 1864 (title page), 15 December 1864 (*Intell Serapeum*), 29 December 1864 (*Allg Bibl Deutsch*)].

*Erste Section. A – G. Herausgegeben von Hermann Brockhaus. Neunundsiebzigster Theil. Grammatik – Granson.* 470 pp. [DP: 1865 (title page), 21 December 1865 (*Allg Bibl Deutsch*), 31 March 1866 (*Intell Serapeum*)].

*Erste Section. A – G. Herausgegeben von Hermann Brockhaus. Achtzigster Theil. Griechenland. (Geographie und Geschichte Alt-Griechenlands.).* 444 pp. [DP: 1862 (title page), 7 August 1862 (*Allg Bibl Deutsch*)].

*Erste Section. A – G. Herausgegeben von Hermann Brockhaus. Einundachtzigster Theil. Griechenland. A. Alt-Griechenland. (Griechische Sprache und Dialekte. – Griechische Musik, Rhythmik und Metrik. – Griechische Metrologie* [sic]. – *Griechische Literatur.* 455 pp. [DP: 1863 (title page), 17 December 1863 (*Allg Bibl Deutsch*)].

*Erste Section. A – G. Herausgegeben von Hermann Brockhaus. Zweiundachtzigster Theil. Griechenland. A. Alt-Griechenland. (Religion oder Mythologie, Theologie und Gottesverehrung der Griechen. – Griechische Kunst.).* 508 pp. [DP: 1864 (title page), 15 December 1864 (*Intell Serapeum*), 29 December 1864 (*Allg Bibl Deutsch*)].

*Erste Section. A – G. Herausgegeben von Hermann Brockhaus. Dreiundachtzigster Theil. Nebst einer Kupfertafel. Griechenland. A. Alt-Griechenland. (Griechische Staatsalterthümer. – Griechische Privatalterthümer. – Griechisches Theater.) B. Griechenland im Mittelalter und in der Neuzeit. (Geographie.).* 444 pp. + 1 pl. [DP: 1866 (title page), December 1866 (Evenhuis 1997a: 236), 3 January 1867 (*Allg Bibl Deutsch*), 30 April 1867 (*Intell Serapeum*)].

*Erste Section. A – G. Herausgegeben von Hermann Brockhaus. Vierundachtzigster Theil. Griechenland. B. Griechenland im Mittelalter und in der Neuzeit. (Griechische Kirche. – Christlich-Griechische Kunst. I. und II. Abschnitt.).* 474 pp. [DP: 1866 (title page), December 1866 (Evenhuis 1997a: 236), 3 January 1867 (*Allg Bibl Deutsch*), 3 April 1867 (*Gött Anz*), 30 April 1867 (*Intell Serapeum*)].

*Erste Section. A – G. Herausgegeben von Hermann Brockhaus. Fünfundachtzigster Theil. Griechenland. B. Griechenland im Mittelalter und in der Neuzeit. (Christlich–Griechische Kunst. III. und IV. Abschnitt. – Geschichte Griechenlands vom Beginn des Mittelalters bis auf unsere Zeit. I. und II. Periode.).* 465 pp. [DP: 1867 (title page), 7 November 1867 (*Allg Bibl Deutsch*), November 1867 (*Allg Bibl*), December 1867 (Evenhuis 1997a: 236)].

*Erste Section. A – G. Herausgegeben von Hermann Brockhaus. Sechsundachtzigster Theil. Griechenland. B. Griechenland im Mittelalter und in der Neuzeit. (Geschichte Griechenlands vom Beginn des Mittelalters bis auf unsere Zeit. II. und III. Periode. – Griechisch-römisches Recht im Mittelalter und in der Neuzeit. I. und II. Periode.).* 471 pp. [DP: 1868 (title page), March 1868 (*Allg Bibl*), 2 April 1868 (*Allg Bibl Deutsch*), 31 October 1868 (*Intell Serapeum*)].

*Erste Section. A – G. Herausgegeben von Hermann Brockhaus. Siebenundachtzigster Theil. Griechenland. B. Griechenland im Mittelalter und in der Neuzeit. (Griechisch-römisches Recht im Mittelalter und in der Neuzeit. III. Periode. – Geschichte Griechenlands im neunzehnten Jahrhundert. – Geschichte der byzantinischen und neugriechischen Literatur.) Systematisches Inhaltsverzeichniß der Griechenland behandelnden Theile 80–87 dieses Werks.* 401 pp. [DP: 1869 (title page), January 1870 (*Allg Bibl*)].

*Erste Section. A – G. Herausgegeben von Hermann Brockhaus. Achtundachtzigster Theil. Grant – Greding.* 469 pp. [DP: 1868 (title page), 31 December 1868 (*Allg Bibl Deutsch*), December 1868 (*Allg Bibl*)].

*Erste Section. A – G. Herausgegeben von Hermann Brockhaus. Neunundachtzigster Theil. Green – Gregorius (IV. Heilige, Kirchenväter und Gelehrte).* 478 pp. [DP: 1869 (title page), December 1869 (Evenhuis 1997a: 236), January 1870 (*Allg Bibl*)].

*Erste Section. A – G. Herausgegeben von Hermann Brockhaus. Neunzigster Theil. Gregorius (IV. Heilige, Kirchenväter und Gelehrte) – Grezin.* 456 pp. [DP: 1871 (title page), January 1871 (Evenhuis 1997a: 236), March 1871 (*Allg Bibl*)].

*Erste Section. A – G. Herausgegeben von Hermann Brockhaus. Einundneunzigster Theil. Grias – Grizio.* 439 pp. [DP: 1871 (title page), December 1871 (Evenhuis 1997a: 236), April 1872 (*Allg Bibl*), January–June 1872 (*Bibliot Philo*)].

*Erste Section. A – G. Herausgegeben von Hermann Brockhaus. Zweiundneunzigster Theil. Gröbel – Grossbritannien (Geschichte, Abschnitt I–V).* 480 pp. [DP: 1872 (title page), December 1872 (Evenhuis 1997a: 236), July–December 1872 (*Bibliot Philo*), February 1873 (*Allg Bibl*)].

*Erste Section. A – G. Herausgegeben von Hermann Brockhaus. Dreiundneunzigster Theil. Grossbritannien (Geschichte, Abschnitt VI und VII, Schluss, und Statistik).* 505 pp. [DP: 1874 (title page), February 1875 (*Allg Bibl*)].

*Erste Section. A – G. Herausgegeben von Hermann Brockhaus. Vierundneunzigster Theil. Grossburgk – Grumus.* 445 pp. [DP: 1875 (title page), September 1875 (*Allg Bibl*)].

*Erste Section. A – G. Herausgegeben von Hermann Brockhaus. Fünfundneunzigster Theil. Grün – Guano.* 450 pp. [DP: 1875 (title page), July–December 1875 (Evenhuis 1997a: 236), February 1876 (*Allg Bibl*)].

*Erste Section. A – G. Herausgegeben von Hermann Brockhaus. Sechsundneunzigster Theil. Guanta – Gulaping.* 418 pp. [DP: 1877 (title page), March 1878 (*Allg Bibl*)].

*Erste Section. A – G. Herausgegeben von Hermann Brockhaus. Siebenundneunzigster Theil. Gulapíngslög – Gussonea.* 427 pp. [DP: 1878 (title page), March 1878 (*Allg Bibl*), January–June 1878 (*Bibliot Philo*)].

*Erste Section. A – G. Herausgegeben von Hermann Brockhaus. Achtundneunzigster Theil. Guss-stahl – Gymnastik.* 405 pp. [DP: 1880 (title page), November 1880 (*Nat Nov*)].

*Erste Section. A – G. Herausgegeben von Hermann Brockhaus. Neunundneunzigster Theil. Gymnesiae – Gyzels, und Nachträge: Gara – Gwalior. Nebst Register über die I. Section, die Buchstaben A–G umfassend.* 196 + 205 pp. [DP: 1882 (title page), September 1882 (*Allg Bibl*), 3 March 1883 (*Lit Centr Deutsch*)].

*Zweite Section. H – N. Herausgegeben von G. Hassel und W. Müller. Erster Theil mit Kupfern und Charten. H – Hamburgh.* 382 + [2] pp. + 3 pls. [DP: 1827 (title page), August 1827 (*Allg Lit Ztg*), July–December 1827 (*Verz Bücher Landkarten*)].

*Zweite Section. H – N. Herausgegeben von G. Hassel und W. Müller. Zweiter Theil mit Kupfern und Charten. Hamcken – Harrespur.* vii + 416 pp. [DP: 1828 (title page; *Vorbericht* dated March 1828), January–June 1828 (*Verz Bücher Landkarten*)].

*Zweite Section. H – N. Herausgegeben von G. Hassel und A.G. Hoffmann. Dritter Theil mit Kupfern und Charten. Harrich – Hebung.* 409 + [2] pp. + 4 pls. [DP: 1828 (title page), July–December 1828 (*Verz Bücher Landkarten*)].

*Zweite Section. H – N. Herausgegeben von G. Hassel und A.G. Hoffmann. Vierter Theil mit Kupfern und Charten. Hecabona – Heinrich (fürstliche Personen.).* 396 pp. + 4 pls. [DP: 1828 (title page), January–June 1829 (*Verz Bücher Landkarten*)].

*Zweite Section. H – N. Herausgegeben von G. Hassel und A.G. Hoffmann. Fünfter Theil mit Kupfern und Charten. Heinrich (Minnesänger) – Hequaesi.* x + 410 + [2] pp. + 3 pls. [DP: 1829 (title page; *Vorwort* dated May 1829), July–December 1829 (*Verz Bücher Landkarten*)].

*Zweite Section. H – N. Herausgegeben von G. Hassel und A.G. Hoffmann. Sechster Theil mit Kupfern und Charten. Heräa – Herpes.* xii + 430 + [1] pp. + 3 pls. [DP: 1829 (title page; *Vorrede* dated 20 December 1829), January–June 1830 (*Verz Bücher Landkarten*)].

*Zweite Section. H – N. Herausgegeben von G. Hassel und A.G. Hoffmann. Siebenter Theil mit Kupfern und Charten. Herpestes – Hibiscus.* 418 + [2 (*Erklärung*)] pp. + 2 pls. [DP: 1830 (title page; *Erklärung* dated end of October 1830), January–June 1831 (*Verz Bücher Landkarten*)].

*Zweite Section. H – N. Herausgegeben von A.G. Hoffmann. Achter Theil mit Kupfern und Charten. Hibo – Hirudines.* 506 + [6] pp. + 4 pls. [DP: 1831 (title page), January–June 1832 (*Verz Bücher Landkarten*)].

*Zweite Section. H – N. Herausgegeben von A.G. Hoffmann. Neunter Theil. Hirudo – Höklyn.* 447 pp. + 4 pls. [DP: 1832 (title page), October–December 1832 (Evenhuis 1997a: 237), 9 February 1833 (*Bibl Deutsch*)].

*Zweite Section. H – N. Herausgegeben von A.G. Hoffmann. Zehnter Theil. Holacanthus – Hormuz.* 501 + [4] pp. + 1 pl. [DP: 1833 (title page), 23 December 1833 (*Bibl Deutsch*), January–June 1834 (*Verz Bücher Landkarten*)].

*Zweite Section. H – N. Herausgegeben von A.G. Hoffmann. Elfter Theil. Horn – Hultschin.* 496 pp. + 8 pls. [DP: 1834 (title page), December 1834 (Evenhuis 1997a: 237), July–December 1834 (*Verz Bücher Landkarten*)].

*Zweite Section. H – N. Herausgegeben von A.G. Hoffmann. Zwölfter Theil. Hum – Hypexodon.* 479 pp. + 6 pls. [DP: 1835 (title page), June–August 1835 (Evenhuis 1997a: 237), 1 October 1835 (*Bibl Deutsch*), July–December 1835 (*Verz Bücher Landkarten*)].

*Zweite Section. H – N. Herausgegeben von A.G. Hoffmann. Dreizehnter Theil. Hypha – Hyzne. Nachträge: Haagen – Hystrix. I – Jacobi*. 259 + 226 pp. [DP: 1836 (title page), 13 January 1837 (*Allg Bibl Deutsch*), January–June 1837 (*Verz Bücher Landkarten*)].

*Zweite Section. H – N. Herausgegeben von A.G. Hoffmann. Vierzehnter Theil. Jacobia – Iba.* 494 pp. [DP: 1837 (title page), 15 December 1837 (*Allg Bibl Deutsch*), 23 December 1837 (*Lit Ztg*)].

*Zweite Section. H – N. Herausgegeben von A.G. Hoffmann. Funfzehnter Theil. Ibaba – Jesztreb.* 478 pp. + 3 pls. [DP: 1838 (title page), October–December 1838 (Evenhuis 1997a: 237), 18 January 1839 (*Allg Bibl Deutsch*)].

*Zweite Section. H – N. Herausgegeben von A.G. Hoffmann. Sechszehnter Theil. Jeta – Indictment.* 448 pp. [DP: 1839 (title page), 27 September 1839 (*Allg Bibl Deutsch*), July–September 1839 (Evenhuis 1997a: 237)].

*Zweite Section. H – N. Herausgegeben von A.G. Hoffmann. Siebzehnter Theil. Indien – Indo-China.* 464 pp. [DP: 1840 (title page), 10 July 1840 (*Allg Bibl Deutsch*), October–December 1840 (Evenhuis 1997a: 237)].

*Zweite Section. H – N. Herausgegeben von A.G. Hoffmann. Achtzehnter Theil. Indogermanischer Sprachstamm – Insectenstich.* 542 pp. [DP: 1840 (title page), October–December 1840 (Evenhuis 1997a: 237), 15 January 1841 (*Allg Bibl Deutsch*), 31 January 1841 (*Intell Serapeum*)].

*Zweite Section. H – N. Herausgegeben von A.G. Hoffmann. Neunzehnter Theil. Insel – Inuus.* 481 pp. [DP: 1841 (title page), 29 October 1841 (*Allg Bibl Deutsch*), October–December 1841 (Evenhuis 1997a: 237), July–December 1841 (*Verz Bücher Landkarten*)].

*Zweite Section. H – N. Herausgegeben A.G. Hoffmann. Zwanzigster Theil. Invaginatio – Johann (Herzoge von Burgund).* 488 pp. [DP: 1842 (title page), 29 July 1842 (*Allg Bibl Deutsch*), July–September 1842 (Evenhuis 1997a: 237)].

*Zweite Section. H – N. Herausgegeben A.G. Hoffmann. Einundzwanzigster Theil. Johann (Infant von Castilien) – Johann-Boniten.* 524 pp. [DP: 1842 (title page), 23 December 1842 (*Allg Bibl Deutsch*), October–December 1842 (Evenhuis 1997a: 237)].

*Zweite Section. H – N. Herausgegeben A.G. Hoffmann. Zweiundzwanzigster Theil. Johanne – Ionisches Portal.* 490 pp. [DP: 1843 (title page), 7 December 1843 (*Allg Bibl Deutsch*), 15 December 1843 (*Intell Serapeum*), October–December 1843 (Evenhuis 1997a: 237), 26 January 1844 (*Leip Reper*)].

*Zweite Section. H – N. Herausgegeben A.G. Hoffmann. Dreiundzwanzigster Theil. Ionium Mare – Irkutzk.* 445 pp. [DP: 1844 (title page), October–December 1844 (Evenhuis 1997a: 237), 9 January 1845 (*Allg Bibl Deutsch*), 15 January 1845 (*Intell Serapeum*), 25 January 1845 (*Lit Ztg*)].

*Zweite Section. H – N. Herausgegeben A.G. Hoffmann. Vierundzwanzigster Theil. Irland – Ismuc.* 473 pp. [DP: 1845 (title page), 18 December 1845 (*Allg Bibl Deutsch*), 31 December 1845 (*Lit Ztg*), October–December 1845 (Evenhuis 1997a: 237)].

*Zweite Section. H – N. Herausgegeben A.G. Hoffmann. Fünfundzwanzigster Theil. Isnagar – Italien.* 513 pp. + 1 pl. [DP: 1846 (title page), April 1846 (Evenhuis 1997a: 237), 17 December 1846 (*Allg Bibl Deutsch*), 15 January 1847 (*Leip Reper*)].

*Zweite Section. H – N. Herausgegeben A.G. Hoffmann. Sechsundzwanzigster Theil. Italiener – Jüdeln.* 432 pp. [DP: 1847 (title page), 15 December 1847 (*Intell Serapeum*), 16 December 1847 (*Allg Bibl Deutsch*), 28 January 1848 (*Leip Reper*)].

*Zweite Section. H – N. Herausgegeben A.G. Hoffmann. Siebenundzwanzigster Theil. Juden – Jüdische Literatur.* 471 pp. [DP: 1850 (title page), 26 December 1850 (*Allg Bibl Deutsch*), October–December 1850 (Evenhuis 1997a: 235), 15 January 1851 (*Intell Serapeum*)].

*Zweite Section. H – N. Herausgegeben von A.G. Hoffmann. Achtundzwanzigster Theil. Jüdische Münzen – Jungermannia.* 478 + [2] pp. [DP: 1851 (title page), October–December 1851 (Evenhuis 1997a: 237), 8 January 1852 (*Allg Bibl Deutsch*), 31 January 1852 (*Intell Serapeum*), January–March 1852 (*Cat Muquardt*)].

*Zweite Section. H – N. Herausgegeben von A.G. Hoffmann. Neunundzwanzigster Theil. Junges Europa – Jury-Tabocas.* 452 pp. [DP: 1852 (title page), December 1852 (Evenhuis 1997a: 237), 13 January 1853 (*Allg Bibl Deutsch*)].

*Zweite Section. H – N. Herausgegeben von A.G. Hoffmann. Dreißigster Theil. Jus – Izzo. Nachträge: Jabaltsa – Integralfunctionen.* 483 pp. [DP: 1853 (title page), December 1853 (Evenhuis 1997a: 237), 26 January 1854 (*Allg Bibl Deutsch*), January–March 1854 (*Cat Muquardt*)].

*Zweite Section. H – N. Herausgegeben von A.G. Hoffmann. Einunddreißigster Theil. Nachträge zu I: Integralrechnung – Junius (Adrian).* 449 pp. [DP: 1855 (title page), 8 November 1855 (*Allg Bibl Deutsch*), December 1855 (Evenhuis 1997a: 237), October–December 1855 (*Cat Muquardt*)].

*Zweite Section. H – N. Herausgegeben von August Leskien. Zweiunddreißigster Theil. K – Karabulaken.* 395 pp. [DP: 1882 (title page), 3 March 1883 (*Lit Centr Deutsch*)].

*Zweite Section. H – N. Herausgegeben von August Leskien. Dreiunddreißigster Theil. Karachitaier – Karl V. von Lothringen.* 399 pp. [DP: 1883 (title page), 1 September 1883 (*Lit Centr Deutsch*), September 1883 (*Allg Bibl*; *Polybiblion*)].

*Zweite Section. H – N. Herausgegeben von August Leskien. Vierunddreißigster Theil. Karl (Herzog von Guise) – Kauffahrer.* 398 pp. [DP: 1883 (title page), December 1883 (Evenhuis 1997a: 237), January 1884 (*Nat Nov*), 9 February 1884 (*Lit Centr Deutsch*)].

*Zweite Section. H – N. Herausgegeben von August Leskien. Fünfunddreißigster Theil. Kaufmann – Khôrasân.* 399 pp. [DP: 1884 (title page)].

*Zweite Section. H – N. Herausgegeben von August Leskien. Sechsunddreißigster Theil. Khorsabad – Klein (Julius Leopold).* 391 pp. [DP: 1884 (title page), July–December 1884 (*Bibliot Philo*)].

*Zweite Section. H – N. Herausgegeben von August Leskien. Siebenunddreißigster Theil. Kleinasien – Kochen.* 392 pp. [DP: 1885 (title page), May 1885 (*Nat Nov*), 20 June 1885 (*Lit Centr Deutsch*), June 1885 (*Allg Bibl*)].

*Zweite Section. H – N. Herausgegeben von August Leskien. Achtunddreißigster Theil. Kocher – Köppen (Friedrich).* 392 pp. [DP: 1885 (title page), July–December 1885 (*Bibliot Philo*), 2 January 1886 (*Lit Centr Deutsch*), January 1886 (*Allg Bibl*), February 1886 (*Nat Nov*)].

*Zweite Section. H – N. Herausgegeben von August Leskien. Neununddreißigster Theil. Köppen (Peter V.) – Kriegk.* 388 pp. [DP: 1886 (title page), November 1886 (*Allg Bibl*; *Nat Nov*)].

*Zweite Section. H – N. Herausgegeben von August Leskien. Vierzigster Theil. Kreigsakademie – Kurzsichtigkeit.* 392 pp. [DP: 1887 (title page)].

*Zweite Section. H – N. Herausgegeben von August Leskien. Einundvierzigster Theil. Kusnezk – Landsmannschaften.* 384 pp. [DP: 1887 (title page), December 1887 (*Nat Nov*)].

*Zweite Section. H – N. Herausgegeben von August Leskien. Zweiundvierzigster Theil. Landstände – Lehrte.* 392 pp. [DP: 1888 (title page), August 1888 (*Nat Nov*)].

*Zweite Section. H – N. Herausgegeben von August Leskien. Dreiundvierzigster Theil. Leibeigenschaft – Ligatur.* 392 pp. [DP: 1889 (title page), 20 July 1889 (*Lit Centr Deutsch*), July 1889 (*Nat Nov*), August 1889 (*Allg Bibl*)].

*Dritte Section. O – Z. Herausgegeben von M.H.E. Meier und L.F. Kämtz. Erster Theil mit Kupfern und Charten. O – Odyssee.* 424 pp. + 3 pls. [DP: 1830 (title page), January–June 1831 (*Verz Bücher Landkarten*)].

*Dritte Section. O – Z. Herausgegeben von M.H.E. Meier und L.F. Kämtz. Zweiter Theil. Odysseis – Olba.* 432 pp. + 3 pls. [DP: 1832 (title page), 9 April 1832 (*Bibl Deutsch*), January–June 1832 (*Verz Bücher Landkarten*)].

*Dritte Section. O – Z. Herausgegeben von M.H.E. Meier und L.F. Kämtz. Dritter Theil. Olbasa – Onocrotalus.* 490 pp. [DP: 1832 (title page), August 1832 (Evenhuis 1997a: 238), 9 February 1833 (*Bibl Deutsch*)].

*Dritte Section. O – Z. Herausgegeben von M.H.E. Meier und L.F. Kämtz. Vierter Theil. Onod – Ordinaten.* 516 + [4] + 2 pls. [DP: 1833 (title page), 23 December 1833 (*Bibl Deutsch*), January–June 1834 (*Verz Bücher Landkarten*)].

*Dritte Section. O – Z. Herausgegeben von M.H.E. Meier und L.F. Kämtz. Fünfter Theil. Ordination – Oroz.* 514 pp. + 3 pls. [DP: 1834 (title page), December 1834 (Evenhuis 1997a: 238), July–December 1834 (*Verz Bücher Landkarten*)].

*Dritte Section. O – Z. Herausgegeben von M.H.E. Meier und L.F. Kämtz. Sechster Theil. Orphaniten – Osteologie.* 479 pp. + 3 pls. [DP: 1835 (title page), June–August 1835 (Evenhuis 1997a: 238), 1 October 1835 (*Bibl Deutsch*), July–December 1835 (*Verz Bücher Landkarten*)].

*Dritte Section. O – Z. Herausgegeben von M.H.E. Meier und L.F. Kämtz. Siebenter Theil. Osteolamacia – Otzenhausen.* 499 pp. [DP: 1836 (title page), 27 May 1836 (*Allg Bibl Deutsch*), January–June 1836 (*Verz Bücher Landkarten*)].

*Dritte Section. O – Z. Herausgegeben von M.H.E. Meier und L.F. Kämtz. Achter Theil. Ouabash – Ozzy. Nachträge: Obajj – Ozodicera. P – Pachnamunis.* 440 + 59 pp. [DP: 1836 (title page), 13 January 1837 (*Allg Bibl Deutsch*), 25 January 1837 (*Lit Ztg*), January–June 1837 (*Verz Bücher Landkarten*)].

*Dritte Section. O – Z. Herausgegeben von M.H.E. Meier und L.F. Kämtz. Neunter Theil. Pacholenus – Palermo-Seide.* 489 pp. + 3 pls. [DP: 1837 (title page), 15 December 1837 (*Allg Bibl Deutsch*), 23 December 1837 (*Lit Ztg*)].

*Dritte Section. O – Z. Herausgegeben von M.H.E. Meier und L.F. Kämtz. Zehnter Theil. Pales – Panus.* 500 pp. [DP: 1838 (title page), 28 September 1838 (*Allg Bibl Deutsch*), July–September 1838 (Evenhuis 1997a: 238)].

*Dritte Section. O – Z. Herausgegeben von M.H.E. Meier und L.F. Kämtz. Elfter Theil. Panvinius – Parczenzew.* 482 pp. + 3 pls. [DP: 1838 (title page), October–December 1838 (Evenhuis 1997a: 238), 18 January 1839 (*Allg Bibl Deutsch*), 31 January 1839 (*Blätt Lit Unterhalt*)].

*Dritte Section. O – Z. Herausgegeben von M.H.E. Meier und L.F. Kämtz. Zwölfter Theil. Pardaillan – Pascalia.* 490 pp. [DP: 1839 (title page), 27 September 1839 (*Allg Bibl Deutsch*), July–September 1839 (Evenhuis 1997a: 238)].

*Dritte Section. O – Z. Herausgegeben von M.H.E. Meier und L.F. Kämtz. Dreizehnter Theil. Pasch – Paukenperlen.* 460 pp. [DP: 1840 (title page), 10 July 1840 (*Allg Bibl Deutsch*), October–December 1840 (Evenhuis 1997a: 238)].

*Dritte Section. O – Z. Herausgegeben von M.H.E. Meier und L.F. Kämtz. Vierzehnter Theil. Paul – Pehuenches.* 486 pp. [DP: 1840 (title page), 4 December 1840 (*Allg Bibl Deutsch*), October–December 1840 (Evenhuis 1997a: 238)].

*Dritte Section. O – Z. Herausgegeben von M.H.E. Meier und L.F. Kämtz. Funfzehnter Theil. Peiden – Pendulinus*. 497 pp. + 2 pls. [DP: 1841 (title page), 29 October 1841 (*Allg Bibl Deutsch*), October–December 1841 (Evenhuis 1997a: 238), July–December 1841 (*Verz Bücher Landkarten*)].

*Dritte Section. O – Z. Herausgegeben von M.H.E. Meier und L.F. Kämtz. Sechszehnter Theil. Peneda – Perigymna.* 484 pp. + 1 pl. [DP: 1842 (title page), 29 July 1842 (*Allg Bibl Deutsch*), July–September 1842 (Evenhuis 1997a: 238)].

*Dritte Section. O – Z. Herausgegeben von M.H.E. Meier. Siebzehnter Theil. Perikles – Perse-Rasch.* 501 pp. + 1 pl. [DP: 1842 (title page), 23 December 1842 (*Allg Bibl Deutsch*), October–December 1842 (Evenhuis 1997a: 238)].

*Dritte Section. O – Z. Herausgegeben von M.H.E. Meier. Achtzehnter Theil. Perses – Peter Ludwig (Herzog von Parma).* 491 pp. [DP: 1843 (title page), 7 December 1843 (*Allg Bibl Deutsch*), 15 December 1843 (*Intell Serapeum*), October–December 1843 (Evenhuis 1997a: 238), 26 January 1844 (*Leip Reper*)].

*Dritte Section. O – Z. Herausgegeben von M.H.E. Meier. Neunzehnter Theil. Peter (Graf von Gravina) – Peutelkofel.* 475 pp. + 1 pl. [DP: 1844 (title page), October–December 1844 (Evenhuis 1997a: 238), 9 January 1845 (*Allg Bibl Deutsch*), 15 January 1845 (*Intell Serapeum*), 25 January 1845 (*Lit Ztg*)].

*Dritte Section. O – Z. Herausgegeben von M.H.E. Meier. Zwanzigster Theil. Peutinger – Pfitzer.* 472 pp. + 2 pls. [DP: 1845 (title page), 28 August 1845 (*Allg Bibl Deutsch*), 15 September 1845 (*Intell Serapeum*), 17 September 1845 (*Lit Ztg*), 19 September 1845 (*Leip Reper*), October 1845 (Evenhuis 1997a: 238)].

*Dritte Section. O – Z. Herausgegeben von M.H.E. Meier. Einundzwanzigster Theil. Pflanzeisen – Phantasma.* 479 pp. + 1 pl. [DP: 1846 (title page), 20 August 1846 (*Allg Bibl Deutsch*), August 1846 (*Intell Allg Lit Ztg*), 18 September 1846 (*Leip Reper*), July–September 1846 (Evenhuis 1997a: 238)].

*Dritte Section. O – Z. Herausgegeben von M.H.E. Meier. Zweiundzwanzigster Theil. Phantast – Philipp (Könige).* 465 pp. [DP: 1846 (title page), 17 December 1846 (*Allg Bibl Deutsch*), 15 January 1847 (*Leip Reper*)].

*Dritte Section. O – Z. Herausgegeben von M.H.E. Meier. Dreiundzwanzigster Theil. Philipp (Weltliche Kurfürsten) – Philosophiana*. 474 pp. [DP: 1847 (title page), 15 December 1847 (*Intell Serapeum*), 16 December 1847 (*Allg Bibl Deutsch*), 28 January 1848 (*Leip Reper*)].

*Dritte Section. O – Z. Herausgegeben von M.H.E. Meier. Vierundzwanzigster Theil. Philosophie – Phokylides.* 485 pp. [DP: 1848 (title page), 28 December 1848 (*Allg Bibl Deutsch*), 4 January 1849 (*Lit Ztg*), 31 January 1849 (*Intell Serapeum*)].

*Dritte Section. O – Z. Herausgegeben von M.H.E. Meier. Fünfundzwanzigster Theil. Phol – Phyxios.* 457 pp. [DP: 1850 (title page), 26 December 1850 (*Allg Bibl Deutsch*), October–December 1850 (Evenhuis 1997a: 238), 15 January 1851 (*Intell Serapeum*)].

This encyclopedia was issued in 167 volumes, divided in three sections, originally under the direction of Johann Samuel Ersch [1766–1828] and Johann Gottfried Gruber [1774–1851]. It includes numerous genus-group name entries on beetles and some of them are quite detailed. These were signed by various authors including Burmeister, J. Victor Carus, Döbereiner, Germar, Giebel, and D. Thon. Whether there are new taxonomic acts, as in some Diptera entries (Evenhuis 1997a: 238), remains to be investigated.

***The British cyclopaedia of natural history***: *combining a scientific classification of animals, plants, and minerals; with a popular view of their habits, economy, and structure. By authors eminent in their particular department. Arranged and edited by Charles F. Partington. Complete in three volumes.* 1834–1837. Orr & Smith, London. (4to) CNC

*First volume* [ABA–CET]. xvi + 796 pp. [DP: (pp. 1–544) 1 April–1 December 1834; (pp. 545–796) 1 January–1 April 1835]. The title page is dated 1835.

*Second volume* [CET–JAC]. 888 pp. [DP: (pp. 1–512) 1 May–1 December 1835; (pp. 513–888) 1 January–1 June 1836]. The title page is dated 1836. The exact title of this and following volume is “*The British cyclopaedia of natural history: combining a scientific classification of animals, plants, and minerals; with a popular view of their habits, economy, and structure. The various articles are written expressly for this work by authors eminent in their particular department. The whole arranged and edited by Charles F. Partington. Complete in three volumes*.”

*Third volume* [JAC–ZYG]. viii + 844 pp. [DP: (pp. 1–448) 1 July–1 December 1836; (pp. 449–844 + i–viii) 1 January–June 1837 (preface dated June 1837)]. The title page is dated 1837.

For precise dates of publication of each montly parts, see Evenhuis (1997b: 604–605). Although no evidence can be found in the *Cyclopaedia* as to the author of the entomological entries, Westwood (1841: 14) indicated that he was the author. In some cases, type species designations for genera are given. The work was issued either with colored or uncolored plates. The three volumes were reissued as volumes 6–8 of the ten-volume “*The British Cyclopaedia of the arts, sciences, history, geography, literature, natural history, and biography; copiously illustrated by engravings on wood and steel by eminent artists edited by Charles F. Partington*” by Wm. S. Orr and Co., London [GB]. Woodward (1913: 1526) mentioned that volumes 1 and 2 were reissued with new title pages dated 1842 (*n.v.*).

***The Cyclopaedia***; *or, universal dictionary of arts, sciences, and literature. By Abraham Rees with the assistance of eminent professional gentleman. Illustrated with numerous engravings, by the most distinguished artists.* [45 volumes, 39 of text and 6 of plates]. 1802–1820. Longman, Hurst, Rees, Orme, & Brown, London. (4to) BHL

**Table:** 

Volume	Part	Contents	Signatures	DP
I	1	A–Agoge	B–Z, Aa–Zz, 3A–3F	2 January 1802
	2	Agogliastro–Amaranthoides	3G–3Z, 4A–4Z, 5A–5L	4 May 1802
II	1	Amaranthus–Antimony	B–Z, Aa–Zz, 3A–?3F	18 October 1802
	2	Antimony–Arteriotomy	?3G–3Z, 4A–4Z, 5A–5L	7 April 1803
III	1	Artery–Babel-Mandeb	B–Z, Aa–Zz, 3A–3G	22 September 1803
	2	Babel-Mandeb–Battersea	3H–3Z, 4A–4Z, 5A–5O	17 March 1804
IV	1	Battery Point–Biörnstahl	B–Z, Aa–Zz, 3A–3C	17 August 1804
	2	Biot–Book-Binding	3D–3Z, 4A–4Z, 5A–5H	13 April 1805
V	1	Book-keeping–Brunia	B–Z, Aa–Zz, 3A–3F	1 June 1805
	2	Brunia–Calvart	3G–3Z, 4A–4Z, 5A–5K	26 December 1805
VI	1	Calvary–Cape of Good Hope	B–Z, Aa–Zz, 3A–3E	18 February 1806
	2	Cape of Good Hope–Castra	3F–3Z, 4A–4Z, 5A–5L	17 June 1806
VII	1	Castrametation–Chalk	B–Z, Aa–Zz, 3A–3E	1 October 1806
	2	Chalk–Chronology	3F–3Z, 4A–4Z, 5A–5M	9 February 1807
VIII	1	Chronometer–Clavaria	B–Z, Aa–Zz, 3A–3D	18 May 1807
	2	Clavaria–Colliseum	3E–3Z, 4A–4Z, 5A–5G	10 August 1807
IX	1	Collision–Congregation	B–Z, Aa–Zz, 3A–3E	27 November 1807
	2	Congregation–Corne	3F–3Z, 4A–4Z, 5A–5H	8 March 1808
X	1	Cornea–Croisade	B–Z, Aa–Zz, 3A–3E	2 May 1808
	2	Croisadde–Czyrcassy	3F–3Z, 4A–4Z, 5A–5C	2 July 1808
XI	1	D–Deluge	B–Z, Aa–Zz, 3A–?3C	23 September 1808
	2	Deluge–Dissimilitude	?3D–3Z, 4A–4Z, 5A–5E	3 December 1808
XII	1	Dissimulation–Dynamics	B–Z, Aa–Zz, 3A–?3C	14 February 1809
	2	Dynamics–Eloanx	?3D–3Z, 4A–4Z, 5A–5E	22 May 1809
XIII	1	Elocution–Equation	B–Z, Aa–Zz, 3A–3C	18 August 1809
	2	Equation–Extremum	3D–3Z, 4A–4Z, 5A–5H	25 November 1809
XIV	1	Extrinsic–Fibro-cartilage	B–Z, Aa–Zz, 3A–3B	3 February 1810
	2	Fibro-cartilage–Food	3C–3Z, 4A–4Z, 5A–5C	13 April 1810
XV	1	Food–Froberger	B–Z, Aa–Zz, 3A–3C	27 June 1810
	2	Froberger–Generation	3D–3Z, 4A–4Z, 5A–5F	8 October 1810
XVI	1	Generation–Gniewe	B–Z, Aa–Zz, 3A–3C	29 November 1810
	2	Gnoien–Gretna	3D–3Z, 4A–4Z, 5A–5E	25 January 1811
XVII	1	Gretry–Hatfield Regis	B–Z, Aa–Zz, 3A–3C	8 March 1811
	2	Hatfield Regis–Hibe	3D–3Z, 4A–4Z, 5A–5F	22 April 1811
XVIII	1	Hibuscus–Huysum	B–Z, Aa–Zz, 3A–3C	3 June 1811
	2	Huzanka–Increment	3D–3Z, 4A–4Z, 5A–5D	20 August 1811
XIX	1	Increments–Josephus	B–Z, Aa–Zz, 3A–3C	14 September 1811
	2	Josephus–Kilmes	3D–3Z, 4A–4Z, 5A–5E	16 December 1811
XX	1	Kiln–Lauremberg	B–Z, Aa–Zz, 3A–3C	27 January 1812
	2	Lauremberg–Lights-Horse	3D–3Z, 4A–4Z, 5A–5E	19 March 1812
XXI	1	Light-House–Longitude	B–Z, Aa–Zz, 3A–?3C	12 May 1812
	2	Longitude–Machinery	?3D–3Z, 4A–4Z, 5A–5D	27 July 1812
XXII	1	Machinery–Manganese	B–Z, Aa–Zz, 3A–3D	27 August 1812
	2	Manganese–Mattheson	3E–3Z, 4A–4Z, 5A–5G	4 November 1812
XXIII	1	Matthew–Metals	B–Z, Aa–Zz, 3A–3C	11 December 1812
	2	Metals–Monsoon	3E–3Z, 4A–4Z, 5A–5F	9 February 1813
XXIV	1	Monster–Muscle	B–Z, Aa–Zz, 3A–?3D	30 March 1813
	2	Muscle–Newton	?3E–3Z, 4A–4Z, 5A–5E	26 April 1813
XXV	1	Newtonian–Oleinae	B–Z, Aa–Zz, 3A–3C	15 July 1813
	2	Oleinae–Ozunicze	3D–3Z, 4A–4Z, 5A–5E	15 September 1813
XXVI	1	P–Passiflora	B–Z, Aa–Zz, 3A–3C	27 November 1813
	2	Passiflora–Perturbation	3D–3Z, 4A–4Z, 5A–5E	18 January 1814
XXVII	1	Pertussis–Picus	B–Z, Aa–Zz, 3A–3C	22 March 1814
	2	Picus–Poetics	3D–3Z, 4A–4Z, 5A–5E	7 May 1814
XXVIII	1	Poetry–Preaching	B–Z, Aa–Zz, 3A–3C	14 July 1814
	2	Preaching–Punjgoor	3D–3Z, 4A–4Z, 5A–5F	16 September 1814
XXIX	1	Punishment–Ramists	B–Z; Aa–Zz, 3A–3C	14 December 1814
	2	Ramists–Repton	3D–3Z, 4A–4Z, 5A–5F	26 January 1815
XXX	1	Republic–Rock	B–Z, Aa–Zz, 3A–?3C	21 March 1815
	2	Rock–Rzemien	?3D–3Z, 4A–4Z, 5A–5H	1 June 1815
XXXI	1	S–Sarabanda	B–Z, Aa–Zz, 3A–3B	11 July 1815
	2	Sarabanda–Scotium	3C–3Z, 4A–4Z, 5A–5D	21 September 1815
XXXII	1	Scotland–Shammy	B–Z, Aa–Zz, 3A–3C	22 December 1815
	2	Shammy–Sindy	3D–3Z, 4A–4Z, 5A–5F	28 February 1816
XXXIII	1	Sine–Sound	B–Z, Aa–Zz, 3A–?3C	17 May 1816
	2	Sound–Starboard	?3D–3Z, 4A–4Z, 5A–5G	27 July 1816
XXXIV	1	Starch–Stuart	B–Z, Aa–Zz, 3A–3C	26 October 1816
	2	Stuart–Szydlow	3D–3Z, 4A–4Z, 5A–5B	11 December 1816
XXXV	1	T–Testudo	B–Z, Aa–Zz, 3A–3C	19 March 1817
	2	Testudo–Toleration	3D–3Z, 4A–4Z, 5A–5F	1 May 1817
XXXVI	1	Tolerium–Tumours	B–Z, Aa–Zz, 3A–?3C	13 August 1817
	2	Tumours–Vermelho	3D–3Z, 4A–4Z, 5A–5G	24 October 1817
XXXVII	1	Vermes–Union	B–Z, Aa–Az, 3A–?3C	20 December 1817
	2	Union–Wateeoo	?3D–3Z, 4A–4Z, 5A–5C	23 March 1818
XXXVIII	1	Water–Whitby	B–Z, Aa–Zz, 3A–3C	29 May 1818
	2	Whitby–Wzetin	3D–3Z, 4A–4Z, 5A–5I	30 July 1818
XXXIX	1	X–Zytomiers; Aam–Baldwin	B–Z, Aa–Zz	30 December 1818
	2	Baldwin–Zollikofer	3A–3Z, 4A–4Q	27 October 1819
	3	Preface; Catalogue & analysis of plates; Index of plates; Title pages	4R–4Z, 5A–5E	29 July 1820

This encyclopaedia was published under the editorship of Abraham Rees [1743–1825]. The entries are not signed but those on entomology were written by Edward Donovan as indicated in the preface (p. v). The pages, except for the preface, are unnumbered. The work was issued in 85 parts, 79 of text and six of plates (Pestana 1979: 353), each bound. Following instructions by the publisher, the parts were to be taken apart and rebound in volumes, each but the last of the text volume consisting of two parts. Title pages for the 39 volumes were issued, each bearing the date of 1819.

Dates of publication were not provided with the original parts. Those given in the table are extracted from an article in the *Philosophical Magazine* (Anonymous 1820b). The source for the dating is not indicated but are possibly from the publisher.

In addition to the 39 volumes of text, six volumes of plates were published. These latter six volumes contain plates issued with the text parts plus the plates of last six parts (Pestana 1979: 353). The title pages of the plate volumes are dated 1820. The natural history plates are in plate volume 5; 16 plates pertain to beetles and scientific names are provided on the plates. The dates of publication of these plates are desired.

An American edition (*n.v.*) of Rees’s encyclopaedia was published in Philadelphia in 47 volumes, 1810–1824 (Anonymous 1872: 181).

***Deutsche Encyclopädie*** *oder allgemeines Real-Wörterbuch aller Künste und Wissenschaften von einer Gesellschaft Gelehrten.* [23 volumes + atlas]. 1778–1807. Varrentrapp Sohn und Wenner, Frankfurt am Main. (4to) MDZ, GB

*Erster Band. A–Ar.* [6] + 851 + [1] pp. [DP: 1778 (title page; *Vorrede* dated 25 April 1778), 10 September 1778 (*Gött Anz*), September 1778 (*Esprit J*)].

*Zweyter Band. As–Bar.* [8] + 880 pp. [DP: 1779 (title page; *Vorrede* dated “Oster-Messe 1779”), 2 September 1779 (*Gött Anz*)].

*Dritter Band. Bar–Blass.* [1] + 949 pp. [DP: 1780 (title page; *Vorrede* dated 21 March 1780), 1 July 1780 (*Gött Anz*)].

*Vierter Band. Blat–Cam.* 889 pp. [DP: 1780 (title page), 5 February 1781 (*Gött Anz*)].

*Fünfter Band. Can–Cn.* [2] + 744 pp. [DP: 1781 (title page; *Vorrede* dated “Herbstmesse 1781”), 14 February 1782 (*Gött Anz*)].

*Sechster Band. Coa–Dec.* [1] + 848 pp. [DP: 1782 (title page), 2 August 1782 (*Jena Ztg*)].

*Siebenter Band. Ded–Eh.* [2] + 1054 pp. [DP: 1783 (title page; *Vorrede* dated 5 March 1783), 13 June 1783 (*Jena Ztg*), 5 July 1783 (*Gött Anz*)].

*Achter Band. Ei–Erz.* 924 pp. [DP: 1783 (title page), 10 April 1784 (*Gött Anz*)].

*Neunter Band. Es–Fey.* [1] + 940 pp. [DP: 1784 (title page; *Vorrede* dated 21 August 1784), 8 November 1784 (*Jena Ztg*)].

*Zehnter Band. Fi–Gai.* 780 pp. [DP: 1785 (title page), 17 June 1785 (*Jena Ztg*)].

*Eilfter band. Gal–Ger.* [1] + 908 pp. [DP: 1786 (title page; *Vorbericht* dated 21 March 1786), 8 July 1786 (*Gött Anz*), 17 November 1786 (*Jena Ztg*)].

*Zwölfter Band. Ger–Gol.* 850 pp. [DP: 1787 (title page)].

*Dreyzehender Band. Gom–Hae.* 740 pp. [DP: 1788 (title page), 29 June 1789 (*Ober Lit*)].

*Vierzehender Band. Hae–Heid.* 810 pp. [DP: 1789 (title page)].

*Fünfzehender Band. Heil–Holz.* [2] + 1036 pp. [DP: 1790 (title page)].

*Sechszehender Band. Hom–Jaz.* 797 pp. [DP: 1791 (title page)].

*Siebenzehender Band. Jba–Jnz.* 811 + [1] pp. [DP: 1793 (title page)].

*Achtzehender Band. Jo–Kal.* [1] + 802 pp. [DP: 1794 (title page; *Vorbericht* dated 8 October 1794), 7 February 1795 (*Intell Allg Lit Ztg*)].

*Neunzehender Band. Kam–Kep.* [1] + 757 pp. [DP: 1796 (title page; *Vorbericht* dated 8 September 1796)].

*Zwanzigster Band. Ker–Kirchenväter.* 775 pp. [DP: 1799 (title page)].

*Ein und zwanzigster Band. Kirchen–Kny*. 750 pp. [DP: 1801 (title page), 23 January 1802 (*Ober Lit*)].

*Zwey und zwanzigster Band*. *Ko–Kraz.* 812 pp. [DP: 1802 (title page)].

*Drey und zwanzigster Band*. *Kre–Ky.* 817 pp. [DP: 1804 (title page), 19 January 1805 (*Ober Lit*)].

*Erster Kupfer Band.* [1] + 75 pls. [DP: 1807 (title page; *Vorbericht* dated 2 November 1807), 27 January 1808 (*Neue Leip Lit*)].

The *Deutsche Encyclopädie* was left unfinished after 23 volumes of text and one volume of plates, published between 1778 and 1807. It was first published under the direction of Ludwig Julius Friedrich Höpfner, Christoph Ludwig Nebel, Johann Ludwig Friederich Diez, Andreas Böhm, Erich Christian Klevesahl and Henrich Martin Gottfried Köster, all professors in Giessen. Many entries pertain to Coleoptera, some extremely detailed; that for “*Käfer*” span 68 pages. The entries are signed with numbers and the authors are identified in the second unnumbered page at the beginning of volume 15. The entry for “*Käfer*” was written by Ludwig Gottlieb Scriba [*q.v.*].

***Dictionnaire classique d’histoire naturelle***, *par Messieurs Audouin, Isid. Bordon, Ad. Brongniart, De Candolle, Dandebard de Férussac, A. Desmoulins, Drapiez, Edwards, Flourens, Geoffroy de Saint-Hilaire, A. De Jussieu, Kunth, G. de Lafosse, Lamouroux, Latreille, Lucas fils, Presle-Duplessis, C. Prévost, A. Richard, Thiébaut de Berneaud, et Bory de Saint-Vincent. Ouvrage dirigé par ce dernier collaborateur, et dans lequel on a ajouté, pour le porter au niveau de la science, un grand nombre de mots qui n’avaient pu faire partie de la plupart des dictionnaires antérieurs.* [17 volumes including an atlas]. 1822–1831. Ray et Gravier [&] Baudouin Frères, Paris. (8vo) McG, GB, GAL

*Tome premier* [A–ARZ]. xvi + 604 pp. [DP: May 1822 (title page), 27 May 1822 (*Acad Sci Fr*), 1 June 1822 (*Bibl Fr*), 11 June 1822 (*J Paris*), June 1822 (*Rev Bibl-Brus*), September 1822 (*Not Geb Natur*)].

*Tome second* [ASA–CAC]. 621 pp. [DP: December 1822 (title page)^112^, 30 December 1822 (*Acad Sci Fr*), 31 December 1822 (*Rev Bibl-Brus*), 4 January 1823 (*Bibl Fr*)].

*Tome troisième. CAD–CHI.* 592 pp. [DP: July 1823 (title page), 6 September 1823 (*Bibl Fr*), 18 September 1823 (*J Paris*), 20 September 1823 (*Rev Bibl-Brus*)].

*Tome quatrième. CHI–COZ.* 628 pp. [DP: December 1823 (title page)^113^, 27 December 1823 (*Bibl Fr*), 20 January 1824 (*Rev Bibl-Brus*)].

*Tome cinquième. CRA–D.* 653 + [1] pp. [DP: April 1824 (title page), 15 May 1824 (*Bibl Fr*), 25 June 1824 (*Rev Bibl-Brus*)].

*Tome sixième. E–FOUQ.* 593 + [1 (Errata)] pp. [DP: September 1824 (title page)^114^, 9 October 1824 (*Bibl Fr*), 10 November 1824 (*Rev Bibl-Brus*)].

*Tome septième. FOUR–G.* 626 + [1] pp. [DP: February 1825 (title page), 5 March 1825 (*Bibl Fr*), 10 May 1825 (*Rev Bibl-Brus*)].

*Tome huitième. H–INV.* viii + 609 + [2] pp. [DP: September 1825 (title page), 5 September 1825 (*Acad Sci Fr*), 10 September 1825 (*Bibl Fr*), 30 September 1825 (*Rev Bibl-Brus*)].

*Tome neuvième. IO–MACIS.* 596 pp. [DP: February 1826 (title page), 25 February 1826 (*Bibl Fr*), January–February 1826 (*Rev Bibl-Brus*)].

*Tome dixième. MACL–MN.* 642 + [1] pp. [DP: June 1826 (title page)^115^, 5 July 1826 (*Bibl Fr*), 31 July 1826 (*Acad Sci Fr*), July 1826 (*Rev Bibl-Brus*)].

*Tome onzième. MO–NSO.* 615 + [1 (Errata)] pp. [DP: January 1827 (title page), 10 February 1827 (*Bibl Fr*), 19 February 1827 (*Acad Sci Fr*), 3 March 1827 (*Rev Bibl-Brus*)].

*Tome douzième. NUA–PAM.* 634 + [1] pp. [DP: August 1827 (title page), 18 August 1827 (*Bibl Fr*), 11 September 1827 (*Rev Bibl-Brus*)].

*Tome treizième. PAN–PIV.* 648 pp. [DP: January 1828 (title page), 1 March 1828 (*Bibl Fr*), 15 April 1828 (*Rev Bibl-Brus*)].

*Tome quatorzième. PLA–ROY.* 710 pp. [DP: September 1828 (title page), 11 October 1828 (*Bibl Fr*), 25 December 1828 (*Rev Bibl-Brus*), September–December 1828 (*Foreign Quart Rev*)].

*Tome quinzième. RUA–S.* 754 pp. [DP: May 1829 (title page), 27 June 1829 (*Bibl Fr*), 14 August 1829 (*Rev Bibl-Brus*)].

*Tome seizième. T–Z.* Rey et Gravier [&] Amable Gobin et Cie. 4 + 748 pp. [DP: October 1830 (title page), 30 October 1830 (*Bibl Fr*), 30 November 1830 (*Rev Bibl-Brus*)].

*Tome dix-septième et dernier. Atlas et illustration des planches.* vii + 141 pp. + 140 pls. [DP: 1831 (title page), 29 October 1831 (*Bibl Fr*)]. The plates were issued by livraisons of ten plates or less each as follows: **livr. 1**: May 1822 (wrapper); **2**: December 1822 (wrapper); **3**: July 1823 (wrapper); **4**: December 1823 (wrapper); **5**: April 1824 (wrapper); **6**: September 1824 (wrapper); **7**: February 1825 (wrapper); **8**: September 1825 (wrapper); **9**: February 1826 (wrapper); **10**: June 1826 (wrapper); **11**: January 1827 (wrapper); **12**: August 1827 (wrapper); **13**: January 1828 (wrapper); **14**: September 1828 (wrapper); **15**: May 1829 (wrapper); **16**: October 1830 (wrapper).

Jean Baptiste Geneviève Marcellin Baron de Bory de Saint-Vincent [1778–1846] was the editor of this dictionary. The authors responsible for the Coleoptera entries are Audoin (“AUD.”) and Guérin-Méneville (“G.”). The atlas contains 140 plates but only one (pl. 5 of the seventh livraison) depicts beetles. Eight species are figured: “*Buprestis amaena* Kirby” (Fig. 1); “*Buprestis guerini* Dejean” (Fig. 2); “*Lamia formosa* Olivier” (Fig. 3); “*Brentus latirostris* Dejean” (Fig. 4); “*Saperda IX guttata* Dejean” (Fig. 5); “*Monochamus lateralis* Dejean” (Fig. 6); “*Cassida zebra* Dejean” (Fig. 7); “*Galleruca albicornis* Wiedemann” (Fig. 8). Some of these species were first made available from the illustrations. Authorship should be attributed to the author of the respective generic entry in the dictionary: *Buprestis* (Audinet-Serville); *Lamia* (Guérin-Méneville); *Brentus* (Audinet-Serville); *Saperda* (Guérin-Méneville); *Monochamus* (Guérin-Méneville); *Cassida* (Audinet-Serville); *Galeruca* (Guérin-Méneville).

An Italian translation in 17 volumes was published in Venezia, 1831–1843, under the title “*Dizionario classico di storia naturale….*” [GB].

***Dictionnaire classique des sciences naturelles***, *présentant la définition, l’analyse et l’histoire de tous les êtres qui composent les trois règnes, leur application générale aux arts, à l’agriculture, à la médecine, à l’économie domestique, etc.; résumant tous les faits présentés par les dictionnaires d’histoire naturelle; augmenté des nombreuses découvertes acquises depuis la publication de ces ouvrages. Par M. Drapiez.* [10 volumes + atlas]. 1837–1845. Meline, Cans et compagnie, Bruxelles. (4to) CAKL, GB, BHL

*Tome premier* [A–B]. 640 pp. [DP: 1837 (title page)].

*Tome second* [C–COL]. 570 pp. [DP: 1837 (title page)].

*Tome troisième* [COM–D]. 606 pp. [DP: 1838 (title page)].

*Tome quatrième* [E–F]. 515 pp. [DP: 1838 (title page), January 1839 (*Bibl Belg*).

*Tome cinquième* [G–H]. 599 pp. [DP: 1839 (title page)].

*Tome sixième* [I–L]. 567 pp. [DP: 1839 (title page)].

*Tome septième* [M–N]. 732 pp. [DP: 1841 (title page), September 1841 (*Bibl Belg*)].

*Tome huitième* [O–PIV]. 712 pp. [DP: 1842 (title page)].

*Tome neuvième* [PL–SL]. 704 pp. [DP: 1844 (title page)].

*Tome dixième* [SM–Z]. 747 pp. [DP: 1845 (title page), 11 August 1845 (*Monthly Lit Adv*)].

*Planches.* 13 pp. + 200 pls. [DP: 1845 (title page)].

Pierre Auguste Joseph Drapiez [1778–1856] was the editor of this dictionary. Several authors, including Audouin, Duméril and Latreille, contributed to the entomological entries but none of them are signed. Therefore, authorship is to be attributed to Drapiez as editor. Type species designations are given for many genera. The dating of the plates, issued in 48 livraisons and bound in a separate volume, is desired. The Coleoptera are on plates 110–121. Except for plate 111, all names on the plates are in vernacular form; the scientific names are listed in the explanation sheets of the plates (pp. 1–13).

This dictionary was reissued in 1853 by the same publisher under the title “*Les trois règnes de la nature. Dictionnaire classique des sciences naturelles, présentant la définition, l’analyse et l’histoire de tous les êtres qui composent les trois règnes, leur application générale aux arts, à l’agriculture, à la médecine, à l’économie domestique, etc.; résumant les travaux de Buffon, Daubenton, Lacépède, Cuvier, de Jussieu, etc., etc. Augmenté des nombreuses découvertes acquises depuis la publication de ces ouvrages. Par M. Drapiez*” [BHL].

***Dictionnaire pittoresque d’histoire naturelle*** *et des phénomènes de la nature, contenant l’histoire des animaux, des végétaux, des minéraux, des météores, des principaux phénomènes physiques et des curiosités naturelles, avec des détails sur l’emploi des productions des trois règnes dans les usages de la vie, les arts et métiers et les manufactures. Rédigé par une société de naturalistes, sous la direction de M. F.-E. Guérin.* [9 volumes of text + 3-volume atlas]. 1833–1840. Paris. (4to) GB, BHL

*Tome premier* [AAL–CAR]. viii + 640 pp. [DP: 1833–1834 (title page)]. Livraisons 1–14 (pp. 1–112) were issued in 1833 and livraisons 15–80 (pp. 113–640) in 1834 (Evenhuis 1997a: 334).

*Tome deuxième* [CAR–EDO]. 640 pp. [DP: 1835 (title page)].

*Tome troisième* [EDR–HOL]. 640 pp. [DP: 1835 (title page)].

*Tome quatrième* [HOL–MAM]. 640 pp. [DP: 1836 (title page)].

*Tome cinquième* [MAM–NEM]. 640 pp. [DP: 1837 (title page)].

*Tome sixième* [NEM–PAL]. 640 pp. [DP: 1838 (title page)].

*Tome septième* [PAL–PIE]. 640 pp. [DP: 1838 (title page)].

*Tome huitième* [PIE–SCO]. 640 pp. [DP: 1839 (title page)].

*Tome neuvième* [SCO–ZYG]. 663 + [1 (Principales fautes a corriger)] + 12 [Table de l’Atlas] pp. [DP: 1839 (title page)].

*Atlas*. 720 pls. [DP: 1840 (title page)].

This dictionary, under the direction of Felix Édouard Guérin-Méneville, was issued in 721 livraisons (see Tome 9, bottom of p. 641; incorrectly indicated as 741^e^) between September 1833 and February 1840 (Natural History Museum, online catalogue record). Dates of publication are known for a number of livraisons (see Evenhuis 1997a: 334–335). The entries on Coleoptera are signed by Guérin-Méneville [Guér.], Achille Percheron [A.P.] and Hippolyte Lucas [H.L.].

An Italian translation, largely based on this dictionary, was issued in six volumes, 1839–1844, in Milan by Ercole Marenesi under the title “*Dizionario pittoresco della storia naturale e delle manifatture ad uso della gioventu compilato sulle opere di F.E. Guèrin e degli autori piu’ recenti.*” (*n.v.*).

***Dictionnaire raisonné et abrégé d’histoire naturelle***, *par d’anciens professeurs; ouvrage consacré aux progrès des sciences, de l’agriculture et des arts.* [2 volumes]. Fournier Frêres, Paris. (8vo) [DP: 1807 (title page), 24 December 1806 (*J Typogr Bibl*), December 1806 (*Mag Encycl*)] (*n.v.* except for title page of first volume).

*Tome premier.* iv + 571 + [1] pp.

*Tome second.* 551 + [1] pp.

Although not specified in the publication, Nicolas Marie Thérèse Jolyclerc [1746–1817] was the editor of this dictionary (Barbier 1809: 53). The entries for the insects were written by Jean Baptiste de Lamarck. The type species of at least one genus (*Scarabaeus*; see Krell et al. 2012) is mentioned in the text. Despite the date on the title page, this dictionary was issued by December 1806.

The dictionary was reissued in 1822 by Samson fils in Paris under the title “*Nouveau dictionnaire raisonné et abrégé d’histoire naturelle, contenant une description claire et précise de tous les produits de la nature, animaux, végétaux, minéraux, etc., leurs diverses espèces, leurs mœurs particulières, leurs propriétés, leur conformation, les lieux où on les trouve, etc., etc. : d’après MM. de Lacepède, pour les quadrupèdes et les poissons ; de Buffon, pour les oiseaux ; Linné, de Jussieu et Wildenow, pour la botanique ; de Lamarck et Latreille, pour les insectes ; Hauy, pour la minéralogie et la cristallographie, etc. Enfin, pour les observations et les recherches dans toutes les parties de cette science, d’après les ouvrages les plus célèbres, et notamment ceux de Descartes, Newton, Delalande, Delaplace, Daubanton, Fabricius, Chaptal, de Fourcroy, Lavoisier, Sage, Werner, Lamétherie et autres : dans lequel se trouve, en outre, une synonymie beaucoup plus complèteque dans aucuns des dictionnaires qui ont paru jusqu’à ce jour, classée, ainsi que l’explication des termes et expressions techniques d’histoire naturelle, à leur ordre alphabétique. Par une société de professeurs. Ouvrage consacré aux progrès des sciences, de l’agriculture et des arts*” [GB]. The two volumes contain the same number of pages except that the extra page at the end of each volume is missing suggesting that this edition is simply a reissue of the first one with the typographical errors corrected.

***Dictionnaire des sciences naturelles***, *dans lequel on traite méthodiquement des différents êtres de la nature, considérés, soit en eux-mêmes, d’après l’état actuel de nos connoissances, soit relativement à l’utilité qu’en peuvent retirer la médecine, l’agriculture, le commerce et les arts; suivi d’une biographie des plus célèbres naturalistes: ouvrage destiné aux médecins, aux agriculteurs, aux manufacturiers, aux artistes, aux commerçans, et à tous ceux qui ont intérêt à connoître les productions de la nature, leurs caractères génériques et spécifiques, leur lieu natal, leurs propriétés et leurs usages. Par plusieurs professeurs du Muséum national d’Histoire naturelle et des autres principales Écoles de Paris.* [6 volumes + atlas]. 1804–1806. Levrault, Schoell et C.^ie^, Paris. (8vo) GB

*Tome premier* [A-ALZ]. lxii + 560 pp. [DP: “An XII (1804)” (title page), 19 August–22 September 1804 (*J Lit Fr*), 12 October 1804 (Sayre 1959: 70), 15 October 1804 (*J Typogr Bibl*), 22 October 1804 (*Décade*)].

*Tome second* [AMA-ARGE]. [1 (Liste alphabétique des noms des Auteurs)] + 515 pp. [DP: “An XII (1804)” (title page), 19 August–22 September 1804 (*J Lit Fr*), 12 October 1804 (Sayre 1959: 70), 15 October 1804 (*J Typogr Bibl*), 22 October 1804 (*Décade*)].

*Tome troisième* [ARGI-BAM]. [1] + 492 pp. [DP: “An XII (1804)” (title page), 30 January 1805 (*J Typogr Bibl*; Sayre 1959: 70), 16 October 1805 (*Neue Leip Lit*)].

*Tome quatrième* [BAN-BLU]. [1] + 483 pp. [DP: “An XIII (1805)” (title page), 1 January 1806 (*Monit Univers*), 8 January 1806 (*J Typogr Bibl*; Sayre 1959: 70)].

*Tome cinquième* [BOA-BYT]. [1] + 480 pp. [DP: “An XIV (1806)”, 1 January 1806 (*Monit Univers*), 8 January 1806 (*J Typogr Bibl*; Sayre 1959: 70)].

*Tome sixième* [CAA-CAL]. [DP: 1806 (Mathews 1925: 34), after 8 January 1806 (Evenhuis 1997a: 181)] (*n.v.*)^116^.

*Atlas du Dictionnaire des Sciences naturelles*. 35 pp. + 42 pls. (4to). The atlas was published in two livraisons: **1**: (15 pp. + 21 pls) “An XII.–1804” (title page); **2**: (20 pp. + 21 pls) “An XIII–1805” (title page). The plates are not numbered and all names cited are in vernacular form.

The dictionnaire was abandoned after volume 6. Many genus-group name entries are included and some of these names are given type species designations (Evenhuis 1997a: 181). Georges Frédéric Cuvier [1773–1838] was the editor. Various authors were responsible for the entries; André-Marie-Constant Duméril was responsible for the “*Histoire des insectes*.”

***Dictionnaire des sciences naturelles***, *dans lequel on traite méthodiquement des différents êtres de la nature, considérés soit en eux-mêmes, d’après l’état actuel de nos connoissances, soit relativement à l’utilité qu’en peuvent retirer la médecine, l’agriculture, le commerce et les arts. Suivi d’une biographie des plus célèbres naturalistes. Ouvrage destiné aux médecins, aux agriculteurs, aux commerçans, aux artistes, aux manufacturiers, et à tous ceux qui ont intérêt à connoitre les productions de la nature, leurs caractères génériques et spécifiques, leur lieu natal, leurs propriétés et leurs usages. Par plusieurs professeurs du Jardin du roi, et des principales écoles de Paris*. [61 volumes]. 1816–1845. F.G. Levrault, Strasbourg [&] Le Normant, Paris. (8vo) McG, BHL, GB

*Tome premier* [A-ALZ]. [1] + lxii + 560 + 138 (Supplément) pp. [DP: 1816 (title page), 12 October 1816 (*Bibl Fr*), October 1816 (Cassini 1834: 145)].

*Tome second* [AMA-ARGE]. [1] + 515 + 123 (Supplément) pp. [DP: 1816 (title page), 12 October 1816 (*Bibl Fr*), October 1816 (Cassini 1834: 145)].

*Tome troisième* [ARGI-BAM]. [1] + 492 + 174 (Supplément) pp. [DP: 1816 (title page), December 1816 (Cassini 1834: 145), 11 January 1817 (*Bibl Fr*)].

*Tome quatrième* [BAN-BLU]. [1] + 483 + 124 (Supplément) pp. [DP: 1816 (title page), December 1816 (Cassini 1834: 146), 11 January 1817 (*Bibl Fr*)].

*Tome cinquième* [BOA-BYT]. [1] + 480 + 260 (Supplément) pp. [DP: 1817 (title page), 8 March 1817 (*Bibl Fr*), 19 March 1817 (*Constit*), March 1817 (Cassini 1834: 146)].

*Tome sixième* [CAA-CAQ]. [1] + 531 + 108 (Supplément) pp. [DP: 1817 (title page), 24 May 1817 (*Bibl Fr*), May 1817 (Cassini 1834: 146)].

*Tome septième* [CAR-CER]. [1] + 534 pp. [DP: 1817 (title page), 24 May 1817 (*Bibl Fr*), May 1817 (Cassini 1834: 146)].

*Tome huitième* [CER-CHI]. [1] + 603 pp. [DP: 1817 (title page), 23 August 1817 (*Bibl Fr*), August 1817 (Cassini 1834: 146)].

*Tome neuvième* [CHL-COF]. [1] + 560 pp. [DP: 1817 (title page), 6 December 1817 (*Bibl Fr*)^117^, December 1817 (Cassini 1834: 146)].

*Tome dixième* [COG-COR]. [1] + 596 pp. [DP: 1818 (title page), 23 May 1818 (*Bibl Fr*), May 1818 (Cassini 1834: 147)].

*Tome onzième* [COS-CRIS]. [1] + 615 pp. [DP: 1818 (title page), December 1818 (Cassini 1834: 147), 9 January 1819 (*Bibl Fr*)].

*Tome douzième* [CRIT-DAZ]. [1] + 564 pp. [DP: 1818 (title page), December 1818 (Cassini 1834: 147), 9 January 1819 (*Bibl Fr*)].

*Tome treizième* [DEA-DZW]. [1] + 578 pp. [DP: 1819 (title page), 24 July 1819 (*Bibl Fr*), July 1819 (Cassini 1834: 147)].

*Tome quatorzième* [EA-EOU]. [1] + 558 pp. [DP: 1819 (title page), 14 August 1819 (*Bibl Fr*), August 1819 (Cassini 1834: 148)].

*Tome quinzième* [EPA-EUO]. [1] + 543 pp. [DP: 1819 (title page), 6 November 1819 (*Bibl Fr*), November 1819 (Cassini 1834: 148)].

*Tome seizième* [EUP-FIK]. [1] + 567 pp. [DP: 1820 (title page), 8 April 1820 (*Bibl Fr*), April 1820 (Cassini 1834: 148)].

*Tome dix-septième* [FIL-FYS]. [1] + 546 pp. [DP: 1820 (title page), 22 July 1820 (*Bibl Fr*), July 1820 (Cassini 1834: 148)].

*Tome dix-huitième* [GA-GJU]. [1] + 594 pp. [DP: 1820 (title page), 6 April 1821 (*Bibl Fr*), April 1821 (Cassini 1834: 149)].

*Tome dix-neuvième* [GLA-GRZ]. [1] + 540 pp. [DP: 1821 (title page), 26 January 1821 (*Bibl Fr*), January 1821 (Cassini 1834: 149)].

*Tome vingtième* [GUA-HEO]. [1] + 572 pp. [DP: 1821 (title page), 29 June 1821 (*Bibl Fr*), June 1821 (Cassini 1834: 149)].

*Tome vingt-unième* [HEP-HUIS]. [1] + 540 pp. [DP: 1821 (title page), 29 September 1821 (*Bibl Fr*), September 1821 (Cassini 1834: 150)].

*Tome vingt-deuxième* [HUIT-IDYE]. [1] + 570 pp. [DP: 1821 (title page), 29 December 1821 (*Bibl Fr*), December 1821 (Cassini 1834: 150)].

*Tome vingt-troisième* [IEA-IRY]. [1] + 645 pp. [DP: 1822 (title page), November 1822 (Cassini 1834: 150), 1 December 1822 (*Rev Bibl-Brus*), 14 December 1822 (*Bibl Fr*)].

*Tome vingt-quatrième* [ISA-KYV]. [1] + 533 pp. [DP: 1822 (title page), 10 August 1822 (*Bibl Fr*), August 1822 (Cassini 1834: 151), July–August 1822 (*Rev Bibl-Brus*)]. This volume was issued before tome 23.

*Tome vingt-cinquième* [LAA-LEO]. [1] + 483 pp. [DP: 1822 (title page), 23 November 1822 (*Bibl Fr*), November 1822 (Cassini 1834: 151; *Rev Bibl-Brus*), December 1822 (*Not Geb Natur*)].

*Tome vingt-sixième* [LEP-LIN]. [1] + 555 pp. [DP^117^: 1823 (title page), May 1823 (Cassini 1834: 152), 16 June 1823 (*Monit Univers*), 19 June 1823 (*Bibl Fr*), 30 June 1823 (*Rev Bibl-Brus*)].

*Tome vingt-septième* [LIO-MAC]. [1] + 551 pp. [DP: 1823 (title page), June 1823 (Cassini 1834: 152), 26 July 1823 (*Bibl Fr*), 15 August 1823 (*Rev Bibl-Brus*)].

*Tome vingt-huitième* [MAD-MANA]. [1] + 492 pp. + 5 tables [DP^118^: 1823 (title page), 27 September 1823 (*Bibl Fr*), September 1823 (Cassini 1834: 153), 10 November 1823 (*Rev Bibl-Brus*)].

*Tome vingt-neuvième* [MANB-MELI]. [1] + 544 pp. [DP: 1823 (title page), 27 December 1823 (*Bibl Fr*), December 1823 (Cassini 1834: 153), 20 January 1824 (*Rev Bibl-Brus*)].

*Tome trentième* [MELL-MEZ]. [1] + 483 pp. [DP: 1824 (title page), 29 May 1824 (*Bibl Fr*), May 1824 (Cassini 1834: 153), 10 September 1824 (*Rev Bibl-Brus*)].

*Tome trente-unième* [MI-MOLLUG]. [1] + 576 pp. [DP: 1824 (title page), 14 August 1824 (*Bibl Fr*), August 1824 (Cassini 1834: 153), 10 September 1824 (*Rev Bibl-Brus*)].

*Tome trente-deuxième* [MOLLUS-MORF]. [1] + 567 pp. [DP: 1824 (title page), 13 November 1824 (*Bibl Fr*), November 1824 (Cassini 1834: 153), 15 December 1824 (*Rev Bibl-Brus*)].

*Tome trente-troisième* [MORG-MYC]. [1] + 588 pp. [DP: 1824 (title page), December 1824 (Cassini 1834: 153), 22 January 1825 (*Bibl Fr*), 20 February 1825 (*Rev Bibl-Brus*)].

*Tome trente-quatrième* [MYD-NIK]. [1] + 560 pp. [DP: 1825 (title page), April 1825 (Cassini 1834: 154), 18 June 1825 (*Bibl Fr*), 25 July 1825 (*Rev Bibl-Brus*)].

*Tome trente-cinquième* [NIL-OJO]. [1] + 534 pp. [DP: 1825 (title page), 8 October 1825 (*Bibl Fr*), 30 October 1825 (*Rev Bibl-Brus*), October 1825 (Cassini 1834: 154)].

*Tome trente-sixième* [OKA-OSC]. [1] + 560 pp. [DP: 1825 (title page), 8 October 1825 (*Bibl Fr*), 30 October 1825 (*Rev Bibl-Brus*), October 1825 (Cassini 1834: 155)].

*Tome trente-septième* [OSE-PARM]. [1] + 559 pp. [DP: 1825 (title page), December 1825 (Cassini 1834: 155), 31 May 1826 (*Bibl Fr*), June 1826 (*Rev Bibl-Brus*)].

*Tome trente-huitième* [PARN-PERRON]. [1] + 528 pp. [DP: 1825 (title page), December 1825 (Cassini 1834: 155), 29 April 1826 (*Bibl Fr*), June 1826 (*Rev Bibl-Brus*)].

*Tome trente-neuvième* [PERROQ-PHOQ]. [1] + 559 pp. [DP: 1826 (title page), 29 April 1826 (*Bibl Fr*), April 1826 (Cassini 1834: 155), June 1826 (*Rev Bibl-Brus*)].

*Tome quarantième* [PHOR-PIM]. [1] + 492 pp. [DP: 1826 (title page), 24 June 1826 (*Bibl Fr*), June 1826 (Cassini 1834: 156; *Rev Bibl-Brus*)].

*Tome quarante-unième* [PIN-PLO]. [1] + 558 pp. [DP: 1826 (title page), June 1826 (Cassini 1834: 156), 23 September 1826 (*Bibl Fr*), October 1826 (*Rev Bibl-Brus*)].

*Tome quarante-deuxième* [PLU-PORC]. [1] + 536 pp. [DP: 1826 (title page), August 1826 (Cassini 1834: 156), 23 September 1826 (*Bibl Fr*), October 1826 (*Rev Bibl-Brus*)].

*Tome quarante-troisième* [PORCE-PSY]. [1] + 544 pp. [DP: 1826 (title page), 30 September 1826 (*Bibl Fr*), September 1826 (Cassini 1834: 156), October 1826 (*Rev Bibl-Brus*)].

*Tome quarante-quatrième* [PTA-RAZ]. [1] + 526 pp. [DP: 1826 (title page), December 1826 (Cassini 1834: 157), 20 January 1827 (*Bibl Fr*), 3 March 1827 (*Rev Bibl-Brus*)].

*Tome quarante-cinquième* [RE-ROCHER]. [1] + 548 pp. [DP: 1827 (title page), 21 February 1827 (*Bibl Fr*), February 1827 (Cassini 1834: 157), 17 March 1827 (*Rev Bibl-Brus*)].

*Tome quarante-sixième* [ROCHES-SAF]. [1] + 546 pp. [DP: 1827 (title page), April 1827 (Cassini 1834: 157), 19 May 1827 (*Bibl Fr*), 16 June 1827 (*Rev Bibl-Brus*)].

*Tome quarante-septième* [SAG-SAY]. [1] + 562 pp. [DP: 1827 (title page), May 1827 (Cassini 1834: 157), 20 June 1827 (*Bibl Fr*), 12 July 1827 (*Rev Bibl-Brus*), August 1827 (*J Sav*)].

*Tome quarante-huitième* [SCA-SERQ]. [1] + 572 pp. [DP: 1827 (title page), June 1827 (Cassini 1834: 158), 21 July 1827 (*Bibl Fr*), 18 August 1827 (*Rev Bibl-Brus*)].

*Tome quarante-neuvième* [SERR-SOUG]. [1] + 539 pp. [DP: 1827 (title page), September 1827 (Cassini 1834: 158), 13 October 1827 (*Bibl Fr*), 5 November 1827 (*Rev Bibl-Brus*)].

*Tome cinquantième* [SOUI-STE]. [1] + 554 pp. [DP: 1827 (title page), 24 November 1827 (*Bibl Fr*), November 1827 (Cassini 1834: 158), December 1827 (*J Sav*), 2 January 1828 (*Rev Bibl-Brus*)].

*Tome cinquante-unième* [STI-SYST.^E^-L]. [1] + 534 pp. [DP: 1827 (title page), December 1827 (Cassini 1834: 159), 12 January 1828 (*Bibl Fr*), 28 February 1828 (*Rev Bibl-Brus*)].

*Tome cinquante-deuxième* [SYST.^E^-M-TEL]. [1] + 570 pp. [DP: 1828 (title page), March 1828 (Cassini 1834: 160), 12 April 1828 (*Bibl Fr*), 6 June 1828 (*Rev Bibl-Brus*)].

*Tome cinquante-troisième* [TEM-THÉOR.^E^-F]. [1] + 508 pp. [DP: 1828 (title page), May 1828 (Cassini 1834: 160), 7 June 1828 (*Bibl Fr*), 3 August 1828 (*Rev Bibl-Brus*)].

*Tome cinquante-quatrième* [TH-TORTR]. [1] + 557 pp. [DP^119^: 1829 (title page), 25 April 1829 (*Bibl Fr*), April 1829 (Cassini 1834: 160), 31 May 1829 (*Rev Bibl-Brus*)].

*Tome cinquante-cinquième* [TORTUE-TS]. [1] + 566 pp. [DP: 1828 (title page), 30 August 1828 (*Bibl Fr*), August 1828 (Cassini 1834: 160), 11 November 1828 (*Rev Bibl-Brus*)].

*Tome cinquante-sixième* [TUA-VAZ]. [1] + 546 pp. [DP: 1828 (title page), September 1828 (Cassini 1834: 161), 11 October 1828 (*Bibl Fr*), 25 December 1828 (*Rev Bibl-Brus*), September–December 1828 (*Foreign Quart Rev*)].

*Tome cinquante-septième* [VEA-VERS]. [1] + 628 pp. [DP: 1828 (title page), December 1828 (Cassini 1834: 161), 10 January 1829 (*Bibl Fr*), 28 February 1829 (*Rev Bibl-Brus*)].

*Tome cinquante-huitième* [VERT-VY]. [1] + 518 pp. [DP: 1829 (title page), February 1829 (Cassini 1834: 161), 14 March 1829 (*Bibl Fr*), 30 April 1829 (*Rev Bibl-Brus*)].

*Tome cinquante-neuvième* [WAA-ZOOP]. [1] + 520 pp. [DP: 1829 (title page), June 1829 (Cassini 1834: 161), April–June 1829 (*Foreign Quart Rev*)].

*Tome soixantième* [ZOOPH-ZYT]. [1] + 631 pp. [DP: 1830 (title page), June 1830 (Cassini 1834: 162), 10 July 1830 (*Bibl Fr*), 15 September 1830 (*Rev Bibl-Brus*)].

*Tome soixante-unième*. vi + 232 pp. [DP: 1845 (title page), 27 September 1845 (*Feuil J Lib*), 15 August 1846 (*Bibl Fr*)]. This volume includes a preface and a supplement (pp. 1–48) and the biographies (pp. 49–232).

*Supplément. Tome I* [A-AYE]. F.G. Bertrand. viii + 527 pp. [DP: 1840 (title page)]. This volume was issued in two livraisons: **1**: (pp. 1–272) 24 October 1840 (*Bibl Fr*), 7 November 1840 (*Feuil J Lib*), 30 November 1840 (*Intell Serapeum*); **2**: (pp. 273–527) 22 May 1841 (*Feuil J Lib*).

The first five volumes are reprint of the unfinished *Dictionnaire des sciences naturelles*, 1804–1806 (see previous entry), to which supplements were added to bring them up to date. Georges Frédéric Cuvier was the editor. All entomological entries were written by André-Marie-Constant Duméril (initials C.D.). The work also included 1220 plates, published in octavo and in folio (Stafleu and Cowan 1976: 586), either colored or uncolored. The Coleoptera plates are in the following atlas-volume “*Dictionnaire des sciences naturelles. Planches, 2.^e^ partie: règne organisé. Zoologie. Insectes, par M. André-Marie-C. Duméril* [*et*] *crustacés, par M. Anselme-Gaétan Desmarest*” [BHL] published by F.G. Levrault, and containing 27 pages and 60 plates. The title page is dated 1816–1830. All names listed on the 22 Coleoptera plates are in vernacular form and therefore have no nomenclature incidence.

An Italian translation of this dictionary was published in Firenze in 22 volumes, 1830–1851, under the title “*Dizionario delle scienze naturali nel quale si tratta metodicamente dei differenti esseri della natura, considerati o in loro stessi, secondo lo stato attuale delle nostre cognizioni, o relativamente all’utilità che ne può risultare per la medicina, l’agricoltura, il commercio, e le arti. Accompagnato da une biografia de’pui’ celebri naturalisti. Opera utile ai medici, agli agricoltori, ai mercanti, agli artisti, ai manifattori, e a tutti coloro, che desiderano conoscere le produzioni della natura, i loro caratteri generici e specifici, il loro luogo natale, le loro proprieta, ed usi. Redatta da vari professori del Giardino del Re, e delle principali scuole di Parigi. Prima traduzione dal francese con aggiunte e correzioni*” [GB].

***Dictionnaire universel d’histoire naturelle*** *résumant et complétant tous les faits présentés par les encyclopédies, les anciens dictionnaires scientifiques, les oeuvres complètes de Buffon, et les meilleurs traités spéciaux sur les diverses branches des sciences naturelles; donnant la description des êtres et des divers phénomènes de la nature, l’étymologie et la définition des noms scientifiques, les principales applications des corps organiques et inorganiques, à l’agriculture, à la médecine, aux arts industriels, etc.; dirigé par M. Charles d’Orbigny, et enrichi d’un magnifique atlas de planches gravées sur acier.* [13 volumes + 3-volume atlas]. 1839–1849. MM. Renard, Martinet et Cie., Paris. (4to) CNC, GB

*Tome premier* [AAL–APH]. viii + 232 (Discours prélimiaire) + 649 pp. [DP: 1841 (title page)]. This volume was published in 12 livraisons (1–12) as follows: **1**: (pp. 1–48) 17 June 1839 (*Soc Géol Fr*); **2**: (pp. 49–?80) August 1839; **3**: (pp. ?81–?136) 16 December 1839 (*Soc Géol Fr*); **4–7**: (pp. ?137–400) January–July 1840; **8–11**: (pp. 401–560) August–November 1840; **12**: (pp. 561–649) September 1841.

*Tome deuxième* [APH–BYZ]. 795 + [1 (Errata du deuxième tome)] pp. [DP: 1842 (title page)]. This volume was issued in 12 livraisons (13–24) as follows: **13–17**: (pp. 1–?320) 1 February–November 1841; **18–24**: (pp. ?321–795) 24 January–30 June 1842.

*Tome troisième* [CAA–CLA]. 744 pp. [DP: 1843 (title page)]. This volume was published in 12 livraisons (25–36) as follows: **25**: (pp. 1–64) 22 August 1842; **26**: (pp. 65–128) 1 September 1842; **27**: (pp. 129–176) 10 October 1842; **28**: (pp. 177–240) 7 November 1842; **29**: (pp. 241–?296) November 1842; **30–35**: (pp. ?297–688) 2 January–29 May 1843; **36**: (pp. 689–744) 1 July 1843.

*Tome quatrième* [CLA–DIC]. 752 pp. [DP: 1844 (title page)]. This volume was issued in 12 livraisons (37–48) as follows: **37**: (pp. 1–64) 31 July 1843; **38**: (pp. 65–128) 4 September 1843; **39**: (pp. 129–?192) 2 October 1843; **40–41**: (pp. ?193–304) November 1843; **42**: (pp. 305–?368) 26 December 1843; **43–47**: (pp. ?369–704) 19 February–29 April 1844; **48**: (pp. 705–752) 20 May 1844, 1 June 1844 (*Bibl Fr*), 10 June 1844 (*Monit Lib*).

*Tome cinquième* [DIC–GAL]. 768 pp. [DP: 1844 (title page)]. This volume was published in 12 livraisons (49–60) as follows: **49**: (pp. 1–64) 24 June 1844; **50**: (pp. 65–128) 1 July 1844; **51**: (pp. 129–176) 12 August 1844; **52**: (pp. 177–240) 26 August 1844; **53**: (pp. 241–296) 23 September 1844; **54–56**: (pp. 297–?488) 14 October–16 December 1844; **57–59**: (pp. ?489–728) 6 January–17 February 1845; **60**: (pp. 729–768) 22 March 1845.

*Tome sixième* [GAL–HYS]. 792 pp. [DP: 1845 (title page)]. This volume was issued in 12 livraisons (61–72) as follows: **61–72**: (pp. 1–792) 28 April–22 December 1845.

*Tome septième* [IAC–MAR]. 806 pp. [DP: 1846 (title page)]. This volume was published in 12 livraisons (73–84) as follows: **73–74**: (pp. 1–?128) 29 December 1845; **75–84**: (pp. ?129–806) January–31 August 1846.

*Tome huitième* [MAR–OID]. 766 pp. [DP: 1846 (title page)]. This volume was issued in 12 livraisons (85–96) as follows: **85–86**: (pp. 1–640) 21 September–14 December 1846; **93–94**: (pp. 641–704) 18 January 1847; **95–96**: (pp. 705–766) 6 February 1847.

*Tome neuvième* [OIE–PHO]. 776 pp. [DP: 1847 (title page)]. This volume was published in 12 livraisons (97–108) as follows: **97–106**: (pp. 1–648) February–July 1847; **107–108**: (pp. 649–776) 17 July 1847.

*Tome dixième* [PHO–REP]. 760 pp. [DP: 1847 (title page)]. This volume was issued in 12 livraisons (109–120) as follows: **109–120**: (pp. 1–760) 9 August–27 December 1847.

*Tome onzième* [REP–STE]. 816 pp. [DP: 1848 (title page)]. This volume was published in 12 livraisons (121–132) as follows: **121–122**: (pp. 1–144) 10 January 1848; **123–124**: (pp. 145–272) 28 February 1848; **125–126**: (pp. 273–416) March–August 1848; **127–132**: (pp. 417–816) 9 September 1848.

*Tome douxième* [STE–VAN]. 816 pp. [DP: 1848 (title page)]. This volume was issued in 12 livraisons (133–144) as follows: **133–134**: (pp. 1–128) 9 September 1848; **135**: (pp. 129–192) 10 September–31 December 1848; **136**: (pp. 193–224) 2 January 1849; **137–143**: (pp. 225–760) January–July 1849; **144**: (pp. 761–816) 7 July 1849.

*Tome treizième* [VAN–ZYZ]. 384 pp. [DP: 1849 (title page)]. This volume was published in 6 livraisons (145–150) as follows: **145–147**: (pp. 1–192) 7 July 1849; **148–149**: (pp. 193–??) 10 September 1849; **150**; (pp. ??–384) 5 November 1849.

The dates of publication and collation^120^ are extracted from Evenhuis (1997b: 575–576) unless otherwise noted for the first volume. Charles Victor Dessalines d’Orbigny [1806–1876] was the editor of this dictionary and Chevrolat [C.] and Duponchel [D.] were responsible for the Coleoptera entries. The three atlases came with colored or uncolored plates. The second atlas, with the title page reading “*Dictionnaire universel d’histoire naturelle dirigé par M. Charles d’Orbigny. Atlas. Zoologie. Reptiles, poissons et insectes. Tome deuxième*” and dated 1849, contains 17 plates depicting beetles. Some of the species illustrated were first made available from these illustrations. This seems to be the case with *Agra latreillei* (pl. 1, fig. 4) and *Brachinus succinctus* (pl. 1, fig. 6).

A second edition was published in 14 volumes of text and three Atlases [GB (vols 2–4, 6, 8, 10, 13–14, Atlas 1–2)] from 1867 to 1869 under the title “*Dictionnaire universel d’histoire naturelle servant de complément aux oeuvres de Buffon, de G. Cuvier, aux encyclopédies, aux anciens dictionnaires scientifiques et résumant les traités spéciaux sur les diverses branches des sciences naturelle, etc. Deuxième édition revue, considérablement augmentée et enrichie d’un magnifique Atlas de plus de 300 planches gravées et coloriées.*” An abridged edition, with M. De Wegmann as co-editor, was published, 1842–1844, in two volumes issued in 80 livraisons (Evenhuis 1990: 219). Copies with different dates of publication on the title page of each volume are know as shown by Sherborn and Palmer (1899: 351).

***Encyclopédie catholique***, *répertoire universel et raisonné des sciences, des lettres, des arts et des métiers, formant une bibliothèque universelle, publiée par la Société de l’Encyclopédie Catholique, sous la direction M. l’Abbé Glaire, de M. le V^te^ Walsh, et d’un comité d’orthodoxie.* [18 volumes]. 1839–1848, 1857–1859. Parent Desbarres, Paris. (4to) GB (vols 1–18), GAL (vols 1–7, 9–17, supplements 1–3)

*Tome premier. A.–Alex.* cxiii + [2] + 675 pp. [DP: 1839 (title page)].

*Tome second. Alex.–Athan.* 766 + [1] pp. [DP: 1840 (title page)].

*Tome troisième. Athan.–Bolom.* 775 pp. [DP: 1841 (title page)].

*Tome quatrième. Bolon.–Caistre.* 768 pp. [DP: 1842 (title page)].

*Tome cinquième. Cait-Bey.–Catherine.* 768 pp. [DP: 1843 (title page)].

*Tome sixième. Catherine.–Charles II.* 768 pp. [DP: 1843 (title page)].

*Tome septième. Charles III.–Circulation.* 768 pp. [DP: 1844 (title page)].

*Tome huitième. Circulation.–Communication.* 792 pp. [DP: 1845 (title page)].

*Tome neuvième. Communion.–Czvittinger.* 852 pp. [DP: 1846 (title page)].

*Tome dixième. D–Église.* 848 pp. [DP: 1846 (title page)].

*Tome onzième. Églogaire.–Fernandez.* 747 pp. [DP: 1846 (title page)].

*Tome douxième. Fernandez–Histérotomie.* 804 pp. [DP: 1847 (title page)].

*Tome treizième. I.–Llywelyn.* 672 pp. [DP: 1847 (title page)].

*Tome quatorze. Nécessité.–Nysten.* 900 pp. [DP: 1847 (title page)].

*Tome quinzième. O.–Pnyx.* 819 pp. [DP: 1847 (title page)].

*Tome seizième. Po.–Rzewuski.* 883 pp. [DP: 1848 (title page)].

*Tome dix-septième. Saale.–Thersite.* 1080 pp. [DP: 1848 (title page)].

*Tome dix-huitième. Thomas (Saint).–Tradition.* 768 pp. [DP: 1848 (title page)].

*Supplément. Tome premier.* iv + 772 pp. [DP: n.d. (title page)].

*Supplément. Tome deuxième.* 768 pp. [DP: n.d. (title page)].

*Supplément. Tome troisième.* 804 pp. [DP: n.d. (title page)].

The first 18 volumes of this encyclopedia were published under the direction of Jean Baptiste Glaire [1798–1879] and viscount Joseph-Alexis Walsh [1782–1860], the three supplements under Joseph Chantrel [1818–1884] and abbot Jean-Baptiste-Siffroy Orse [1810–1874].

The title of this work varied throughout its publication. The one reported here is for the first printing of the first volume. This volume was also issued with the title page dated 1840 and reading “*Encyclopédie catholique*, *répertoire universel et raisonné des sciences, des lettres, des arts et des métiers, formant une bibliothèque universelle, avec la biographie des hommes célèbres; ornée de plus de 3000 gravures dans le texte et renfermant le résumé de plus de dix mille ouvrages ; publiée avec la collaboration des hommes les plus éminents dans les sciences, les lettres, les arts, etc., sous la direction, collaboration et révision de M. l’Abbé Glaire, de M. le V^te^ Walsh, et d’un comité d’orthodoxie*” [GB]. The three supplements were issued by the same publisher under the title “*Supplément a l’Encyclopédie catholique publié sous la direction de M. Chantrel et de M. l’Abbé Orse avec la collaboration d’hommes spéciaux dans les lettres, les sciences et les arts, contenant les progrès nouveaux, les découvertes récentes dans les sciences, les lettres et les arts, la biographies des hommes illustres morts depuis la publication de l’Encyclopédie Catholique jusqu’à nos jours, etc. Ce supplément est le complément indispensable à l’Encyclopédie Catholique, publiée en 18 vol. in 4^o^, sous la direction de M. l’Abbé Glaire et de M. le vicomte Walsh.*”

The first 18 volumes were issued in 152 livraisons (Anonymous 1849: 304) from 7 August 1839 (*Semeur*) to 1848 and the three supplements in 24 livraisons (Anonymous 1859f: 580) from 18 April 1857 (*Bibl Fr*) to 10 December 1859 (*Bibl Fr*). Dating of all livraisons is desired.

The work includes many listings of family-group and generic names of insects, including Coleoptera. Some of them are signed by initials. Type-species are designated for some genera although they are probably not first typifications.

***Nouveau dictionnaire classique d’histoire naturelle***, *ou répertoire universel par ordre alphabétique, des sciences naturelles et physiques, rédigé par une société de naturalistes. 2^e^ édition, revue et corrigée avec soin par M.B.S., ancien professeur*. [47 text-volumes + 47-atlas volumes of 15 plates each]. 1844–1847. Cosson, Paris. (12mo) GB (partial)

*I* [A–ALU]. x + [1] + 474 pp. [DP: 1844 (title page), 9 March 1844 (*Bibl Fr*), 20 March 1844 (*Monit Lib*), 23 March 1844 (*Lit Ztg*)].

*II* [ALU–APP]. 472 pp. [DP: 1844 (title page), 23 March 1844 (*Bibl Fr*), 6 April 1844 (*Lit Ztg*)].

*III* [APP–BAL]. 469 pp. [DP: 1844 (title page), 6 April 1844 (*Bibl Fr*)].

*IV* [BAL–BOS]. 470 pp. [DP: 1844 (title page), 27 April 1844 (*Bibl Fr*)].

*V* [BOT–CAM]. 495 pp. [DP: 1844 (title page), 18 May 1844 (*Bibl Fr*), 1 June 1844 (*Monit Lib*)].

*VI* [CAM–CHA]. 482 pp. [DP: 1844 (title page), 22 June 1844 (*Bibl Fr*), 12 July 1844 (*Leip Reper*)].

*VII* [CHA–CLE]. 499 pp. [DP: 1844 (title page), 17 August 1844 (*Bibl Fr*), 1 September 1844 (*Monit Lib*)].

*VIII* [CLE–COU]. 503 pp. [DP: 1844 (title page), 31 August 1844 (*Bibl Fr*), 10 September 1844 (*Monit Lib*)].

*IX* [COU–DAM]. 485 pp. [DP: 1844 (title page), 28 September 1844 (*Bibl Fr*), 10 October 1844 (*Monit Lib*)].

*X* [DAM–DUF]. 503 pp. [DP: 1844 (title page), 2 November 1844 (*Bibl Fr*), 20 November 1844 (*Monit Lib*)].

*XI* [DUF–EPI]. 486 pp. [DP: 1845 (title page), 3 May 1845 (*Bibl Fr*)].

*XII* [EPI–FIA]. 504 pp. [DP: 1845 (title page), 5 July 1845 (*Bibl Fr*)].

*XIII* [FIA–GAR]. 499 pp. [DP: 1845 (title page), 5 July 1845 (*Bibl Fr*)].

*XIV* [GAR–GOB]. 489 pp. [DP: 1845 (title page), 5 July 1845 (*Bibl Fr*)].

*XV* [GOB–HEL]. 499 pp. [DP: 1845 (title page), 30 August 1845 (*Bibl Fr*)].

*XVI* [HEL–HYD]. 504 pp. [DP: 1845 (title page), 30 August 1845 (*Bibl Fr*)].

*XVII* [HYD–INS]. 497 pp. [DP: 1845 (title page), 4 October 1845 (*Bibl Fr*), 9 October 1845 (*Echo Monde Sav*), 15 October 1845 (*Lit Ztg*)].

*XVIII* [INS–LAG]. 489 pp. [DP: 1845 (title page), 25 October 1845 (*Bibl Fr*)].

*XIX* [LAG–LIL]. 501 pp. [DP: 1845 (title page), 25 October 1845 (*Bibl Fr*)].

*XX* [LIL–MAG]. 468 pp. (*n.v.*).

*XXI* [MAG–MAR]. 496 pp. (*n.v.*).

*XXII* [MAR–MEN]. 479 pp. (*n.v.*).

*XXIII* [MEN–MET]. 515 pp. [DP: 1845 (title page)].

*XXIV* [MET–MON]. 556 pp. [DP: 1845 (title page)].

*XXV* [MON–MYR]. 496 pp. [DP: 1846 (title page), 24 October 1846 (*Bibl Fr*)].

*XXVI* [MYR–NEZ]. 499 pp. [DP: 1846 (title page), 24 October 1846 (*Bibl Fr*)].

*XXVII* [NIA–NYS]. 496 pp. [DP: 1846 (title page), 24 October 1846 (*Bibl Fr*)].

*XXVIII* [OAS–OIS]. 492 pp. [DP: 1846 (title page), 24 October 1846 (*Bibl Fr*)].

*XXIX* [OIS–ORI]. 493 pp. [DP: 1846 (title page), 24 October 1846 (*Bibl Fr*)].

*XXX* [ORM–OVO]. 584 pp. [DP: 1846 (title page), 24 October 1846 (*Bibl Fr*)].

*XXXI* [OVO–PAP]. 530 pp. [DP: 1846 (title page), 31 October 1846 (*Bibl Fr*)].

*XXXII* [PAP–PAS]. 486 pp. [DP: 1846 (title page), 31 October 1846 (*Bibl Fr*)].

*XXXIII* [PAS–PER]. 539 pp. [DP: 1846 (title page), 31 October 1846 (*Bibl Fr*)].

*XXXIV* [PER–PHT]. 503 pp. [DP: 1846 (title page), 31 October 1846 (*Bibl Fr*)].

*XXXV* [PHT–PHY]. 528 pp. [DP: 1846 (title page), 31 October 1846 (*Bibl Fr*)].

*XXXVI* [PHY–PIM]. 503 pp. [DP: 1846 (title page), 31 October 1846 (*Bibl Fr*)].

*XXXVII* [PIM–POG]. 504 pp. [DP: 1846 (title page), 31 October 1846 (*Bibl Fr*)].

*XXXVIII* [POI–PON]. 502 pp. [DP: 1846 (title page), 28 November 1846 (*Bibl Fr*)].

*XXXIX* [PON–QUI]. 504 pp. [DP: 1846 (title page), 28 November 1846 (*Bibl Fr*)].

*XL* [RAB–SAL]. 540 pp. [DP: 1846 (title page), 28 November 1846 (*Bibl Fr*)].

*XLI* [SAN–SIL]. 501 pp. [DP: 1846 (title page), 27 March 1847 (*Bibl Fr*)].

*XLII* [SIL–SQU]. 430 pp. [DP: 1846 (title page), 27 March 1847 (*Bibl Fr*)].

*XLIII* [SQU–TAU]. 427 pp. [DP: 1846 (title page), 27 March 1847 (*Bibl Fr*)].

*XLIV* [TAX–TES]. 420 pp. [DP: 1846 (title page), 3 April 1847 (*Bibl Fr*)].

*XLV* [TES–TRO]. 429 pp. [DP: 1846 (title page), 3 April 1847 (*Bibl Fr*)].

*XLVI* [TRO–ZIR]. 427 pp. [DP: 1846 (title page), 17 April 1847 (*Bibl Fr*), 28 April 1847 (*Lit Ztg*)].

*XLVII* [ZIZ–ZYG]; *Supplément [ACA–VIO*]; *Table*; *Table d’atlas.* 188 + 110 + 36 pp. [DP: 17 April 1847 (*Bibl Fr*), 28 April 1847 (*Lit Ztg*)] (*n.v.*).

Jean Baptiste Geneviève Marcellin Baron de Bory de Saint-Vincent was the editor of this dictionary. According to Sherborn (1922a: xliv), this work “seems to be a mere resetting, with an occasional foot-note” of the *Dictionnaire pittoresque d’histoire naturelle et des phénomènes de la nature* [*q.v.*].

***Nouveau dictionnaire d’histoire naturelle***, *appliquée aux arts, principalement à l’agriculture et à l’économie rurale et domestique: par une société de naturalistes et d’agriculteurs: avec des figures tirées des trois règnes de la nature*. [24 volumes]. 1802–1804. Déterville, Paris. (8vo) GB

*Tome I* [A–APA]. lxiv + 512 pp. [DP: “An XI–1803” (title page), 23 October–21 November 1802 (*J Lit Fr*), 29 November 1802 (*Observ Spec*), 3 December 1802 (Patrin 1803: 524), 6 December 1802 (*J Typogr Bibl*)].

*Tome II* [APA–BAR]. 567 pp. [DP: “An XI–1803” (title page), 23 October–21 November 1802 (*J Lit Fr*), 29 November 1802 (*Observ Spec*), 3 December 1802 (Patrin 1803: 524), 6 December 1802 (*J Typogr Bibl*)].

*Tome III* [BAR–BYT]. 581 pp. [DP: “An XI–1803” (title page), 23 October–21 November 1802 (*J Lit Fr*), 29 November 1802 (*Observ Spec*), 3 December 1802 (Patrin 1803: 524), 6 December 1802 (*J Typogr Bibl*)].

*Tome IV* [CAA–CHA]. 575 pp. [DP: “An XI– 1803” (title page), 4 February 1803 (*J Typogr Bibl*), 21 January–19 February 1803 (*J Lit Fr*; *J Phys*), 3 March 1803 (Patrin 1803: 524)].

*Tome V* [CHA–COC]. 590 pp. [DP: “An XI–1803” (title page), 4 February 1803 (*J Typogr Bibl*), 21 January–19 February 1803 (*J Lit Fr*; *J Phys*), 3 March 1803 (Patrin 1803: 524)].

*Tome VI* [COC–CUB]. 583 pp. [DP: “An XI–1803” (title page), 4 February 1803 (*J Typogr Bibl*), 21 January–19 February 1803 (*J Lit Fr*; *J Phys*), 3 March 1803 (Patrin 1803: 524)].

*Tome VII* [CUC–ENH]. 575 pp. [DP^121^: “An XI–1803” (title page), 14 April 1803 (*J Typogr Bibl*), 21 April–20 May 1803 (*J Lit Fr*)].

*Tome VIII* [EJO–FOT]. 590 pp. [DP: “An XI–1803” (title page), 14 April 1803 (*J Typogr Bibl*), 21 April–20 May 1803 (*J Lit Fr*)].

*Tome IX* [FOU–GOR]. 576 pp. [DP: “An XI–1803” (title page), 14 April 1803 (*J Typogr Bibl*), 21 April–20 May 1803 (*J Lit Fr*)].

*Tome X* [GOT–HIN]. 576 pp. [DP: “An XI–1803” (title page), 6 June 1803 (*J Débats*), 13 June 1803 (*J Typogr Bibl*), 20 June–19 July 1803 (*J Lit Fr*)].

*Tome XI* [HIP–IMP]. 584 pp. [DP: “An XI–1803” (title page), 6 June 1803 (*J Débats*), 13 June 1803 (*J Typogr Bibl*), 20 June–19 July 1803 (*J Lit Fr*)].

*Tome XII* [INA–LAT]. 566 pp. [DP: “An XI–1803” (title page), 6 June 1803 (*J Débats*), 13 June 1803 (*J Typogr Bibl*), 20 June–19 July 1803 (*J Lit Fr*)].

*Tome XIII* [LAU–MAL]. 576 pp. [DP: “An XI–1803” (title page), 20 July 1803 (Patrin 1803: 524), 12 August 1803 (*J Typogr Bibl*), 19 August–17 September 1803 (*J Lit Fr*)].

*Tome XIV* [MAL–MOI]. 576 pp. [DP: “An XI–1803” (title page), 20 July 1803 (Patrin 1803: 524), 12 August 1803 (*J Typogr Bibl*), 19 August–17 September 1803 (*J Lit Fr*)].

*Tome XV* [MOI–NZI]. 580 pp. [DP: “An XI–1803” (title page), 20 July 1803 (Patrin 1803: 524), 12 August 1803 (*J Typogr Bibl*), 19 August–17 September 1803 (*J Lit Fr*)].

*Tome XVI* [OBÉ–PAN]. 575 pp. [DP: “An XI–1803” (title page), 7 October 1803 (*J Débats*), 24 September–23 October 1803 (*J Lit Fr*; *J Phys*), 8 November 1803 (*J Typogr Bibl*)].

*Tome XVII* [PAN–PIL]. 574 pp. [DP: “An XI–1803” (title page), 7 October 1803 (*J Débats*), 24 September–23 October 1803 (*J Lit Fr*; *J Phys*), 8 November 1803 (*J Typogr Bibl*)].

*Tome XVIII* [PIM–PYX]. 595 pp. [DP: “An XI–1803” (title page), 7 October 1803 (*J Débats*), 24 September–23 October 1803 (*J Lit Fr*; *J Phys*), 8 November 1803 (*J Typogr Bibl*), 21 January 1804 (*Neue Intell Lit Kunst*)].

*Tome XIX* [QOT–RYZ]. 578 pp. [DP: “An XI–1803” (title page), 22 December 1803 (*J Typogr Bibl*), 23 December 1803–21 January 1804 (*J Lit Fr*)].

*Tome XX* [SAA–SOI]. 576 pp. [DP: “An XI–1803” (title page), 22 December 1803 (*J Typogr Bibl*), 23 December 1803–21 January 1804 (*J Lit Fr*)].

*Tome XXI* [SOL–THE]. 571 pp. [DP: “An XI–1803” (title page), 22 December 1803 (*J Typogr Bibl*), 23 December 1803–21 January 1804 (*J Lit Fr*)].

*Tome XXII* [TEI–VAL]. ii + 583 pp. [DP: “An XII–1804” (title page), 22 January–20 February 1804 (*J Phys*), 7 March 1804 (*J Typogr Bibl*), 16 March 1804 (*J Débats*), 21 February–21 March 1804 (*J Lit Fr*), 20 April 1804 (*J Paris*)].

*Tome XXIII* [VAL–ZYZ]. 567 pp. [DP: “An XII–1804” (title page), 22 January–20 February 1804 (*J Phys*), 7 March 1804 (*J Typogr Bibl*), 16 March 1804 (*J Débats*), 21 February–21 March 1804 (*J Lit Fr*), 20 April 1804 (*J Paris*)].

*Tome XXIV*. 84 (Addition d’articles connus pendant l’impression de ce dictionnaire; Table de noms Latins) + 85 (Avis de l’éditeur; Explication et développemens des caractères) + 238 (Tableau méthodique d’histoire naturelle) + 18 (Tableau alphabétique des figures) + 34 (Liste alphabétique des souscripteurs) pp. [DP: An XII – 1804 (title page), 22 January–20 February 1804 (*J Phys*), 7 March 1804 (*J Typogr Bibl*), 16 March 1804 (*J Débats*), 21 February–21 March 1804 (*J Lit Fr*), 20 April 1804 (*J Paris*)].

Two hundred and four plates in 4to were issued along with the livraisons.

Guillaume-Antoine Olivier (using the intial “O.”) and Pierre André Latreille (using the initial “L.”) authored the entries on the insects. New beetles taxa are described in some of the volumes.

This dictionary was reissued, probably by an Italian editor, under the same title but with the title pages dated 1839 and the plates copied from the original edition (Guédès 1978: 46).

***Nouveau dictionnaire d’histoire naturelle*** *appliquée aux arts, à l’agriculture, à l’économie rurale et domestique, à la médecine, etc. par une société de naturalistes et d’agriculteurs: nouvelle édition presqu’entièrement refondue et considérablement augmentée; avec des figures tirées des trois règnes de la nature.* [36 volumes]. 1816–1819. Déterville, Paris. (8vo) McG, GAL (all but vols 16, 19, 22)

*Tome I* [ABA–ANI]. 3 + [1] + lxxix + 552 pp. + 8 pls. [DP: 1816 (title page), 14 September 1816, November 1816 (*J Sav*)].

*Tome II* [ANI–ASE]. [2] + 592 pp. + 10 pls. [DP: 1816 (title page), 14 September 1816, November 1816 (*J Sav*)].

*Tome III* [ASI–BOE]. [2] + 560 pp. + 10 pls. [DP: 1816 (title page), 14 September 1816, November 1816 (*J Sav*)].

*Tome IV* [BOE–CAL]. [2] + 602 pp. + 7 pls. [DP: 1816 (title page), 14 December 1816, January 1817 (*J Sav*)].

*Tome V* [CAL–CEZ]. [2] + 614 pp. + 9 pls. [DP: 1816 (title page), 14 December 1816, January 1817 (*J Sav*)].

*Tome VI* [CHA–CHO]. [2] + 570 pp. + 8 pls. [DP: 1816 (title page), 14 December 1816, January 1817 (*J Sav*)].

*Tome VII* [CHO–COR]. [2] + 586 pp. + 9 pls. [DP: 1817 (title page), 15 March 1817, 5 April 1817 (*Constit*), 13 April 1817 (*J Débats*)].

*Tome VIII* [COR–CUN]. [2] + 602 pp. + 10 pls. [DP: 1817 (title page), 15 March 1817, 5 April 1817 (*Constit*), 13 April 1817 (*J Débats*)].

*Tome IX* [CUN–DZW]. [2] + 624 pp. + 6 pls. [DP: 1817 (title page), 15 March 1817, 5 April 1817 (*Constit*), 13 April 1817 (*J Débats*)].

*Tome X* [EAL–EZE]. [2] + 591 pp. + 6 pls. [DP: 1817 (title page), 21 June 1817].

*Tome XI* [FAB–FOR]. [2] + 602 pp. + 6 pls. [DP: 1817 (title page), 21 June 1817].

*Tome XII* [FOR–GEN]. [2] + 608 pp. + 8 pls. [DP: 1817 (title page), 21 June 1817].

*Tome XIII* [GEN–GUE]. [2] + 586 pp. + 7 pls. [DP: 1817 (title page), 13 September 1817, November 1817 (*J Sav*)].

*Tome XIV* [GUE–HOM]. 627 pp. + 7 pls. [DP: 1817 (title page), 13 September 1817, November 1817 (*J Sav*)].

*Tome XV* [HOM–HYS]. [2] + 550 pp. + 6 pls. [DP: 1817 (title page), 13 September 1817, November 1817 (*J Sav*)].

*Tome XVI* [IAS–JYN]. 595 pp. + 8 pls. [DP: 1817 (title page), 27 December 1817].

*Tome XVII* [KAA–LIG]. 622 pp. + 6 pls. [DP: 1817 (title page), 27 December 1817].

*Tome XVIII* [LIG–MAM]. 542 pp. + 6 pls. [DP: 1817 (title page), 27 December 1817].

*Tome XIX* [MAM–MED]. 619 pp. + 6 pls. [DP: 1818 (title page), 30 May 1818].

*Tome XX* [MED–MIN]. 586 pp. + 9 pls. [DP: 1818 (title page), 30 May 1818].

*Tome XXI* [MIN–MOZ]. 612 pp. + 5 pls. [DP: 1818 (title page), 30 May 1818].

*Tome XXII* [MU–NIL]. 618 pp. + 6 pls. [DP: 1818 (title page), 5 September 1818].

*Tome XXIII* [NIL–ORC]. 612 pp. + 6 pls. [DP: 1818 (title page), 5 September 1818].

*Tome XXIV* [ORC–PAS]. 577 pp. + 8 pls. [DP: 1818 (title page), 5 September 1818].

*Tome XXV* [PAS–PHO]. 610 pp. + 7 pls. [DP: 1817 (title page)^122^, 26 December 1818].

*Tome XXVI* [PHO–PLA]. 584 pp. + 8 pls. [DP: 1818 (title page), 26 December 1818].

*Tome XXVII* [PLA–POR]. 586 pp. + 5 pls. [DP: 1818 (title page), 26 December 1818].

*Tome XXVIII* [POR–RAL]. 570 pp. + 12 pls. [DP: 1819 (title page), 1 May 1819].

*Tome XXIX* [RAM–RYZ]. 576 pp. + 7 pls. [DP: 1819 (title page), 1 May 1819].

*Tome XXX* [SA–SEN]. 592 pp. + 6 pls. [DP: 1819 (title page), 1 May 1819].

*Tome XXXI* [SEO–SPE]. 577 pp. + 7 pls. [DP: 1819 (title page), 11 September 1819].

*Tome XXXII* [SPH–TAZ]. 595 pp. + 8 pls. [DP: 1819 (title page), 11 September 1819].

*Tome XXXIII* [TCH–THE]. 568 pp. + 5 pls. [DP: 1819 (title page), 11 September 1819].

*Tome XXXIV* [THE–TSU]. 578 pp. + 9 pls. [DP: 1819 (title page), 11 December 1819].

*Tome XXXV* [TUA–VIM]. 572 pp. + 5 pls. [DP: 1819 (title page), 11 December 1819].

*Tome XXXVI* [VIN–ZYZ]. 544 pp. + 5 pls. [DP: 1819 (title page), 11 December 1819].

The publication dates are from Stafleu and Cowan (1976: 636) unless otherwise noted. The entomological entries were written by Guillaume-Antoine Olivier (O.L.) and Pierre André Latreille (L.). All the names on the plates are in vernacular form. Sherborn (1932b: cxxxvii) mentioned that apparently two settings of this dictionary have been issued “as several variants in specific names are known.”

***Onomatologia historiae natvralis completa*** *oder vollständiges Lexicon das alle Benennungen der Kunstwörter der Naturgeschichte nach ihrem ganzen Umfang erkläret und den reichen Schatz der ganzen Natur durch deutliche und richtige Beschreibungen des nützlichen und sonderbaren von allen Thieren, Pflanzen und Mineralien sowohl vor Aerzte als andere Liebhaber in sich fasst zu allgemeinem Gebrauch von einer Gesellschaft naturforschender Aerzte nach den richtigsten Urkunden zusammengetragen.* [7 volumes]. 1758–1777. Gaum [vols 1–2] / August Lebrecht Stettin [vols 3–7], Ulm, Frankfurt und Leipzig. (8vo) MDZ, GB

[*Erster Band*. A–AF]. [6 (D.P.F. Gmelins *Vorrede*)] + [8 (*Vorrede der Verfasser*, dated 15 March 1758] + [6] + 840 columns. [DP: 1758 (title page). The title of this volume is “*Onomatologia medica completa sev Onomatologia historiae natvralis*...”

*Zweiter Band mit einer Vorrede versehen von Herrn Jacob Christian Schäffer* [AF–CL]. [43] pp. + 896 colums. [DP: 1761 (title page; page [1] dated 20 February 1761)].

*Dritter Band mit einer Vorrede versehen von denen Verfassern* [CO–FU]. [11] pp. + 1004 columns. [DP: 1766 (title page), 4 September 1767 (*Jena Ztg*)]. The titles of this and followings volumes are identical to that of volume 2 except that the word “*Pflanzen*” is dropped.

*Vierter Band mit einer Vorrede versehen von den Verfassern* [GA–LY]. [11] pp. + 918 columns. [DP: 1773 (title page; *Vorrede* dated 24 November 1772)].

*Fünfter Band mit einer Vorrede versehen von den Verfassern* [MA–OX]. [10] pp. + 894 colums + [1] p. [DP: 1775 (title page; *Vorrede* dated 8 April 1775)] .

*Sechster Band mit einer Vorrede versehen von den Verfassern* [PA–SC]. [10] pp. + 952 colums + [1] p. [DP: 1775 (title page; *Vorrede* dated 14 December 1775)].

*Siebender und lezter Band mit einer Vorrede versehen von den Verfassern* [SC–ZY]. [6] pp. + 904 columns + [58 (Register)] pp. [DP: 1777 (title page; *Vorrede* dated 2 January 1777), 20 April 1777^123^].

The editor of this dictionary is not mentioned as such and the entries are not signed. Engelmann (1846: 115) credited volumes 1–4 to Philipp Friedrich Gmelin [1721–1768] and volumes 5–7 to Gottlieb Friedrich Christmann [1752–1836]. All known insect genera are described following by the descriptions of the species. The species listed in the first two volumes are not binominal. The entire work has been placed on the *Official Index of Rejected and Invalid Works in Zoological Nomenclature* (Direction 32, ICZN 1956a).

***Ph. Funke’s und G.H.C. Lippold’s neuestes Natur- und Kunstlexicon***, *enthaltend: die meisten, insbesondere aber die gemeinnützigsten Gegenstände aus der Naturgeschichte, Naturlehre, Chemie, Technologie und Oeconomie. Zum bequemen Gebrauche für Jedermann nach den bisher gemachten Entdeckungen, Erfahrungen, Erfindungen und Beobachtungen aus dem Gebiethe der oben erwähnten Wissenschaften, von einem Vereine mehrerer Gelehrten neu bearbeitet, vermehrt und verbessert.* [10 volumes]. 1824–1827. Hirschfeld [vols 1–3] / Kaulfuss und Krammer [vols 4–10], Wien. (8vo) MDZ, GB (part)

*Erster Band. (Mit drey Kupfertafeln.)* [AAL–BYS]. vi + 552 + [14] pp. [DP: 1824 (title page; *Vorrede* dated April 1824)].

*Zweyter Band. (Mit drey Kupfertafeln.)* [CAB–EYW]. 532 + [16] pp. + 3 pls. [DP: 1824 (title page)].

*Dritter Band. (Mit drey Kupfertafeln.)* [FAB–HAU]. 560 + [12] pp. + 3 pls. [DP: 1825 (title page)].

*Vierter Band. (Mit drey Kupfertafeln.)* [HAU–LAU]. 560 + [15] pp. + 3 pls. [DP: 1825 (title page)].

*Fünfter Band. (Mit drey Kupfertafeln.)* [LAU–MUS]. 605 + [10] pp. [DP: 1825 (title page)].

*Sechster Band. (Mit drey Kupfertafeln.)* [MUS–QUO]. 529 + [11] pp. + 3 pls. [DP: 1825 (title page)].

*Siebenter Band. (Mit drey Kupfertafeln.)* [RAA–SEE]. 576 + [14] pp. + 3 pls. [DP: 1825 (title page)].

*Achter Band. (Mit drey Kupfertafeln.)* [SEE–TOR]. 561 + [13] pp. [DP: 1826 (title page)].

*Neunter Band. (Mit drey Kupfertafeln.)* [TOR–WUR]. [2] + 548 + [12] pp. + 3 pls. [DP: 1826 (title page; *Vorwort* dated 10 August 1826)].

*Zehnter Band. (Mit zwey Kupfertafeln.)* [XAN–ZYP]. [2] + 356 pp. + 2 pls. [DP: 1827 (title page; *Vorwort* dated March 1827)]. This volume includes the “*Nomenclator latinus physiographico-systematicus*” (pp. 177–338) and “*Adpendix ad Nomenclatorem latinum physiographico-systematicum*” (pp. 339–356) given a list of species treated in the different volumes.

The editor is this encyclopedia is mentioned in the *Vorwort* of the ninth volume which is signed “Aloys Hofmann [?–1827], Redacteur vorliegenden Lexicon.” Authorship of the various entries are not indicated. The original edition was issued in Weimar in three volumes, 1801–1804, by Georg Heinrich Christian Lippold [1767–1841] and Carl Philipp Funke [1752–1807] under the title “*Neues Natur- und Kunstlexicon, enthaltend die wichtigsten und gemeinnützigsten Gegenstände aus der Naturgeschichte, Naturlehre, Chemie und Technologie. Zum bequemen Gebrauch insonderheit auch für Ungelehrte und für gebildete Frauenzimmer ausgearbeitet von G.H.C. Lippold, und herausgegeben von C.Ph. Funke.*”

A four-volume atlas, totaling 109 plates, was issued in Wien, 1825–1827, under the title “*Naturhistorischer Atlas zu Ph. Funke’s und G.H.C. Lippold’s neu umgearbeitetem und vervollkommnetem Natur- und Kunstlexicon.*” (Nissen 1969: 205).

***Universal-Lexikon der Gegenwart und Vergangenheit*** *oder neuestes encyclopädisches Wörterbuch der Wissenschaften, Künste und Gewerbe bearbeitet von mehr als 220 Gelehrten herausgegeben von H.A. Pierer. Zweite, völlig umgearbeitete Auflage. (Dritte Ausgabe.).* [34 volumes]. 1840–1847. H.A. Pierer, Altenburg. (8vo) GB

*Erster Band. A–Amirola.* lxxiv + 480 pp. [1840 (title page; *Vorwort* dated May 1840)]. *Hefte* 1–3: (pp. lxxiv + 1–240) 5 June 1840 (*Allg Bibl Deutsch*); *Hefte* 4–6: (pp. 241–480) 17 July 1840 (*Allg Bibl Deutsch*).

*Zweiter Band. Amis–Assyrius.* 478 pp. [1840 (title page)]. *Hefte* 7–12: (pp. 1–478) 5 February 1841 (*Allg Bibl Deutsch*).

*Dritter Band. Ast–Batteriewurst.* 478 pp. [1840 (title page)]. *Hefte* 13–18: (pp. 1–478) 5 February 1841 (*Allg Bibl Deutsch*).

*Vierter Band. Battersea–Blokade.* 478 pp. [1841 (title page)]. *Hefte* 19–24: (pp. 1–478) 4 June 1841 (*Allg Bibl Deutsch*).

*Fünfter Band. Blomberg–Buigen.* 478 pp. [1841 (title page)]. *Hefte* 25–30: (pp. 1–478) 1 October 1841 (*Allg Bibl Deutsch*).

*Sechster Band. Buil–Choctaws.* 478 pp. [1841 (title page)]. *Hefte* 31–36: (pp. 1–478) 17 December 1841 (*Allg Bibl Deutsch*).

*Siebenter Band. Choczim–Czwittinger.* 462 pp. [1841 (title page)]. *Hefte* 37–42: (pp. 1–462) 14 January 1842 (*Allg Bibl Deutsch*).

*Achter Band. D–Doit.* 462 pp. [1841 (title page)]. *Hefte* 43–48: 15 January–17 August 1842.

*Neunter Band. Dok–Entenzunge.* 478 pp. [1842 (title page)]. *Hefte* 49–54: 15 January–17 August 1842.

*Zehnter Band. Enter–Flüvogel.* 478 pp. [1842 (title page)]. *Hefte* 55–60: (pp. 1–478) 18 August 1842 (*Wöchentl Lit Anz*).

*Eilfter Band. Flug–Gefechtslehre.* 478 pp. [1842 (title page)]. *Hefte* 61–66: (pp. 1–478) 27 October 1842 (*Wöchentl Lit Anz*).

*Zwölfter Band. Gefege–Grey.* 438 pp. [1842 (title page)]. *Hefte* 67–72 (pp. 1–438) 15 December 1842 (*Wöchentl Lit Anz*).

*Dreizehnter Band. Griäsowetz–Hebriden.* 478 pp. [1843 (title page)]. *Hefte* 73–78: (pp. 1–478) February 1843 (*Allg Bibl Deutsch*).

*Vierzehnter Band. Hebron–Hyutahy.* 470 pp. [1843 (title page)]. *Hefte* 79–84: (pp. 1–470) May 1843 (*Allg Bibl Deutsch*).

*Funfzehnter Band. I–Karkor.* 478 pp. [1843 (title page)]. *Hefte* 85–87: (pp. 1–224) July 1843 (*Allg Bibl Deutsch*); *Hefte* 88–90: (pp. 225–478) August 1843 (*Allg Bibl Deutsch*).

*Sechzehnter Band. Karl–Krönungsmünzen.* 478 pp. [1843 (title page)]. *Hefte* 91–93: (pp. 1–240) September 1843 (*Allg Bibl Deutsch*); *Hefte* 94–96: (pp. 241–478) October 1843 (*Allg Bibl Deutsch*).

*Siebzehnter Band. Kröpelin–Linnoux.* 462 pp. [1843 (title page)]. *Hefte* 97–102: (pp. 1–462) November 1842 (*Allg Bibl Deutsch*).

*Achtzehnter Band. Linociera–Maroniten.* 462 pp. [1843 (title page)]. *Hefte* 103–105: (pp. 1–240) December 1843 (*Allg Bibl Deutsch*); *Hefte* 106–108: (pp. 241–462) January 1844 (*Allg Bibl Deutsch*).

*Neunzehnter Band. Maronneger–Morfling.* 494 pp. [1843 (title page)]. *Hefte* 109–114: (pp. 1–494) February 1844 (*Allg Bibl Deutsch*).

*Zwanzigster Band. Morgä–Niemand.* 498 pp. [1844 (title page)]. *Hefte* 115–117: (pp. 1–240) April 1844 (*Allg Bibl Deutsch*); *Hefte* 118–120: (pp. 241–498) June 1844 (*Allg Bibl Deutsch*).

*Einundzwanzigster Band. Niemann–Ozzek.* 510 pp. [1844 (title page)]. *Hefte* 121–123: (pp. 1–224) July 1844 (*Allg Bibl Deutsch*); *Hefte* 124–126: (pp. 225–510) September 1844 (*Allg Bibl Deutsch*).

*Zweiundzwanzigster Band. P–Pfrimm.* 454 pp. [1844 (title page)]. *Hefte* 127–129: (pp. 1–224) October 1844 (*Allg Bibl Deutsch*); *Hefte* 130–132: (pp. 225–454) November 1844 (*Allg Bibl Deutsch*).

*Dreiundzwanzigster Band. Pfropf–Preussisch-Roth.* 510 pp. [1844 (title page)]. *Hefte* 133–138: (pp. 1–510) 23 January 1845 (*Allg Bibl Deutsch*).

*Vierundzwanzigster Band. Preussisch-russischer Krieg gegen Frankreich 1806-1807–Reluitionsrecht.* 446 pp. [1844 (title page)]. *Hefte* 139–144: (pp. 1–446) 23 January 1845 (*Allg Bibl Deutsch*).

*Fünfundzwanzigster Band. Rema–Russisische Hornmusik.* 492 pp. [1844 (title page)]. *Hefte* 145–150: (pp. 1–492) 24 January–4 June 1845.

*Sechsundzwanzigster Band. Russische Kirche–Schamvielen.* 462 pp. [1845 (title page)]. *Hefte* 151–153: (pp. 1–240) 5 June 1845 (*Allg Bibl Deutsch*); *Hefte* 154–156: (pp. 244–462) 3 July 1845 (*Allg Bibl Deutsch*).

*Siebenundzwanzigster Band. Schan–Schwedt.* 474 pp. [1845 (title page)]. *Hefte* 157–162: (pp. 1–474) 18 September 1845 (*Allg Bibl Deutsch*).

*Achtundzwanzigster Band. Schwefel–Skirren.* 470 pp. [1845 (title page)]. *Hefte* 163–168: (pp. 1–470) 23 October 1845 (*Allg Bibl Deutsch*).

*Neunundzwanzigster Band. Skirrhös–Stchutschin.* 446 pp. [1845 (title page)]. *Hefte* 169–174: (pp. 1–446) 2 January 1846 (*Allg Bibl Deutsch*).

*Dreissigster Band. Steamboats–Tauroa.* 446 pp. [1845 (title page)]. *Hefte* 175–180: (pp. 1–446) 12 March 1846 (*Allg Bibl Deutsch*).

*Einunddreissigster Band. Taurobolion–Tromm.* 510 pp. [1845 (title page)]. *Hefte* 181–186: (pp. 1–510) 16 April 1846 (*Allg Bibl Deutsch*).

*Zweiunddreissigster Band. Trommel–Vergrösserungswörter.* 510 pp. [1846 (title page)]. *Hefte* 187–192: (pp. 1–510) 6 August 1846 (*Allg Bibl Deutsch*).

*Dreiunddreissigster Band. Vergründen–Westergau.* 510 pp. [1846 (title page)]. *Hefte* 193–198: (pp. 1–510) 3 December 1846 (*Allg Bibl Deutsch*).

*Vierunddreissigster Band. Westerhäubchen–Zzubin und Nachtrag.* viii + 711 + [1] pp. [1846 (title page; *Nachwort* dated March 1847)]. *Hefte* 199–204: (pp. 1–480) 18 March 1847 (*Allg Bibl Deutsch*); *Hefte* 205–207: (pp. 481–711) 6 May 1847 (*Allg Bibl Deutsch*).

This second edition (third printing) was issued under the direction of the publisher Heinrich August Pierer [1794–1850]. Most of the entries on Coleoptera are not informative but some, usually signed “Wr” (someone named Winkler), are detailed and could be of interest. The title has remained unchanged through this edition except that starting with volume 16 “*220 Gelehrten*” is changed to “*300 Gelehrten*.”

The first edition of this encyclopedia was issued in 26 volumes, 1824–1837, under the title “*Encyclopädisches Wörterbuch der Wissenschaften, Künste und Gewerbe, bearbeitet von mehreren Gelehrten*” [HATH, in part]. A second printing of the first edition was published, 1835–1837, under the title “*Universal-Lexikon oder vollständiges encyclopädisches Wörterbuch herausgegeben von H.A. Pierer*” [HATH]. A third edition came out of the press in 17 volumes, 1849–1852, under the title of the seond edition. Four subsequent editions will be issued after Pierer’s death under the title “*Pierer’s Universal-Lexikon*…”.

***Wörterbuch der Naturgeschichte***, *dem gegenwärtigen Stande der Botanik, Mineralogie und Zoologie angemesse.* [11 volumes]. 1824–1837. Landes-Industrie-Comptoir, Weimar. (4to) GB

*Erster Band. A–Bir.* [DP: 1825 (title page)]. *Heft* 1: (pp. 1–288 + pls 1–10) 9 May 1824 (Evenhuis 2015a: 54), 26 June 1824 (*Intell Ztg Welt*); *Heft* 2: (pp. 289–560 + [5] + pls 11–20) January 1825 (Evenhuis 2015a: 54), 15 February 1825 (*Intell Ztg Welt*).

*Zweiter Band. Bir–Chai.* [DP: 1825 (title page)]. *Heft* 1: (pp. 1–256 + pls 21–30) 17 May 1825 (Evenhuis 2015a: 54); *Heft* 2: (pp. 257–468 + pls 31–40) 2 October 1825 (Evenhuis 2015a: 54), 1 December 1825 (*Leip Ztg*).

*Dritter Band. Cha–Cro.* [DP: 1826 (title page)]. *Heft* 1: (pp. 1–288 + pls 41–50) 15 April 1826 (Evenhuis 2015a: 54), 6 May 1826 (*Bibl Deutsch*); *Heft* 2: (pp. 289–560 + pls 51–60) 19 September 1826 (Evenhuis 2015a: 54), 7 November 1826 (*Intell Ztg Welt*), 8 November 1826 (*Bibl Deutsch*).

*Vierter Band. Cro.– Eisen*. [DP: 1828 (title page)]. *Heft* 1: (pp. 1–288 + pls 61–70) 6 May 1827 (Evenhuis 2015a: 54), 9 June 1827 (*Bibl Deutsch*), 23 June 1827 (*Intell Ztg Welt*); *Heft* 2: (pp. 289–580 + pls 71–80) 12 December 1827 (*Bibl Deutsch*), 13 December 1827 (Evenhuis 2015a: 54), 18 December 1827 (*Intell Ztg Welt*).

*Fünster Band. Eisen–Garidella.* [DP: 1829 (title page)]. *Heft* 1: (pp. 1–288 + pls 81–90) 14 June 1828 (*Bibl Deutsch*), 22 July 1828 (Evenhuis 2015a: 54); *Heft* 2: (pp. 289–544) April 1829 (*Allg Monats Deutsch*), 9 May 1829 (*Bibl Deutsch*), 23 June 1829 (Evenhuis 2015a: 54).

*Sechster Band. Gariofilata–Herinaceus.* [DP: 1829 (title page)]. *Heft* 1: (pp. 1–272) 20 June 1829 (*Bibl Deutsch*), July 1829 (Evenhuis 2015a: 54), 4 August 1829 (*Intell Ztg Welt*); *Heft* 2: (pp. 273–512) 12 January 1830 (*Bibl Deutsch*), 16 March 1830 (Evenhuis 2015a: 54).

*Siebenter Band. Herion–Justica.* [DP: 1831 (title page)]. *Heft* 1: (pp. 1–256 + pls 91–100) November 1830 (Evenhuis 2015a: 54), 24 December 1830 (*Bibl Deutsch*), 27 December 1830 (*Beil Allg Ztg*); *Heft* 2: (pp. 257–512) February 1831 (Evenhuis 2015a: 54), 9 April 1831 (*Bibl Deutsch*), 7 June 1831 (*Intell Ztg Welt*).

*Achter Band. Justicia–Lepuropetalon.* [DP: 1832 (title page)]. *Heft* 1: (pp. 1–256) 19 September 1831 (*Bibl Deutsch*), 2 October 1831 (Evenhuis 2015a: 54); *Heft* 2: (pp. 257–542) 3 April 1832 (Evenhuis 2015a: 54).

*Neunter Band. Lepus–Mauhlia.* [DP: 1833 (title page)]. *Heft* 1: (pp. 1–256) 7 January 1833 (*Bibl Deutsch*); *Heft* 2: (pp. 257–574) 6 October 1833 (Evenhuis 2015a: 54), 9 November 1833 (*Bibl Deutsch*).

*Zehnter Band. Maulin– Myzoxyle.* [DP: 1836 (title page)]. *Heft* 1: (pp. 1–256) 12 April 1835 (Evenhuis 2015a: 54); *Heft* 2: (pp. 257–582) July 1836 (Evenhuis 2015a: 54), 12 August 1836 (*Allg Bibl Deutsch*), 17 August 1836 (*Lit Ztg*), September 1836 (*Intell Allg Lit Ztg*).

*Elften Bandes. Erste Hälfte. Naats–Oceanides.* 272 pp. [DP: 1837 (title page), 10 March 1837 (*Allg Bibl Deutsch*), November 1837 (Evenhuis 2015a: 54)].

This unfinished dictionnary was issued in 21 parts (*Hefte*) under the supervision of Ludwig Friedrich von Froriep (Evenhuis 2015a: 51). Evenhuis (2015) mentioned that the work contains many new nomenclatural acts for both botany and zoology, including type species designations. Unfortunately, none of the entries are authored. As pointed by Evenhuis (1995), the new nomenclatural acts in this work should be credited to Froriep.

A new edition (*n.v.*) was started in 1837 but soon abandoned. Only four parts were issued and recorded in *Literarische Zeitung* on 22 March 1837 (*Band* 1, *Heft* 1), 10 May 1837 (*Band* 1, *Heft* 2) and 30 August 1837 (*Band* 2, *Hefte* 1–2).

## Cross-index to works^130^

A catalogue of British Coleoptera; *see* Crotch, G.R.

A catalogue [of] British insects; *see* Morris, F.O.

A catalogue of British insects; *see* Forster, J.R.

A catalogue of Coleoptera from the Japanese Archipelago; *see* Lewis, G.

A catalogue of Indian medicinal plants and drugs; *see* Fleming, J.

A catalogue of the animals of North America; *see* Forster, J.R.

A catalogue of the lucanoid Coleoptera; *see* Hope, F.W.

A classified index and synopsis of the animal kingdom; *see* Griffith, E.

A general natural history; *see* Hill, J.

A general system of nature; *see* Turton, W.

A guide to an arrangement of British insects; *see* Curtis, J.

A handbook of the Coleoptera; *see* Cox, H.E.

A history of Egyptian mummies; *see* Hope, F.W.

A history of the fossil insects in the Secondary rocks of England; *see* Brodie, P.B.

A list of the Australian longicorns; *see* Pascoe, F.P.

A list of the described longicornia of Australia and Tasmania; *see* Pascoe, F.P.

A manual of British Coleoptera; *see* Stephens, J.F.

A manual of natural history, for the use of travellers; *see* Adams, A.

A monograph of Christmas Island (Indian Ocean); *see* Waterhouse, C.O.

A monograph of the coleopterous families Corylophidae and Sphaeriidae; *see* Matthews, A.

A naturalist in the Transvaal; *see* Distant, W.L.; Gahan, C.J.

A naturalist’s wanderings in the eastern Archipelago; *see* Janson, O.E.; Waterhouse, C.O.

A nomenclature of British entomology; *see* Samouelle, G.

A rearrangement of the nomenclature and synonymy of those species of British Coleoptera; *see* Dawson, J.F.

A report on the insects of Massachusetts; *see* Harris, T.W.

A revision of the coleopterous family Coccinellidae; *see* Crotch, G.R.

A systematic catalogue of British insects; *see* Stephens, J.F.

A treatise on insects, general and systematic; *see* Wilson, J.

Abbildungen auslaendischer Insecten; *see* Thon, T.

Abbildungen und Beschreibungen merkwürdiger naturgeschichtlicher Gegenstände; *see* Wolf, J.

Abbildungen zu Karl Illiger’s Uebersetzung von Olivier’s Entomologie; *see* Sturm, J.

Abgekürzte Geschichte der Insecten; *see* Sulzer, J.H.

Abhandlungen über verschiedene Gegenstände der Naturgeschichte; *see* Schröter, J.S.

Abhandlungen von Insecten; *see* Schäffer, J.C.G. von.

Ad cognitonem specierum Fennicarum generis Mycetopori symbolae; *see* Mäklin, F.W.

Adatok Erdély bogárfaunájához; *see* Ormay, S.

Adephagous and clavicorn Coleoptera from the Tertiary; *see* Scudder, S.H.

Agriculture of New-York; *see* Emmons, E.

Allgemeine Naturgeschichte; *see* Schubert, G.H.

Allgemeine Naturgeschichte für alle Stände; *see* Oken, L.

Alterum supplementum Coleoptera Europae; *see* Villa, A.

American entomology; *see* Say, T.

Amphicoma et Eulasia insectorum coleopterorum genera; *see* Truqui, E.

An epitome of the natural history of the insects of China; *see* Donovan, E.

An epitome of the natural history of the insects of India; *see* Donovan, E.

An epitome of the natural history of the insects of New Holland; *see* Donovan, E.

An essay on the genus Hydroscapha; *see* Matthews, A.

An introduction to entomology; *see* Kirby, W.

An introduction to the modern classification of insects; *see* Westwood, J.O.

Analecta entomologica; *see* Dalman, J.W.; Schaum, H.R.

Analyse de la nature; *see* Rafinesque, C.S.

Analytische Zoologie; *see* Froriep, L.F. von.

Anfangsgründe der Naturgeschichte; *see* Esmarch, H.P.C.; Leske, N.G.

Anfangsgründe der Thiergeschichte; *see* Lenz, J.G.

Anhang zur Bestimmungs-Tabellen der Carabidae; *see* Reitter, E.

Animal kingdom, arranged after its organization; *see* Carpenter, W.B.

Animalium specierum in classes; *see* Linnaeus, C.

Animaux nouveaux ou rares; *see* Lucas, P.H.

Anleitung zur Kenntniss des Thierreichs; *see* Fiedler, K.W.

Annual Report of the Chief of Engineers; *see* LeConte, J.L.

Annulosa Javanica; *see* Macleay, W.S.

Annvs II. Historico-natvralis; *see* Scopoli, J.A.

Annvs V. Historico-natvralis; *see* Scopoli, J.A.

Appendix ad C.J. Schönherr Synonymiam Insectorum; *see* Schönherr, C.J.

Appendix ad catalogum insectorum; *see* Megerle von Mühlfeld, J.K.

Appendix to the narrative of a second voyage in search of a North-West passage; *see* Curtis, J.

Apuntes para la geografía y fauna entomológicas de Mataró; *see* Salvañá y Comas, J.M.

Aradvármegye és Arad szabad királyi város természetrajzi leirása; *see* Simonkai, L.

Arcana entomologica; *see* Westwood, J.O.

Arcana naturae; *see* Thomson, J.L.

Archives de l’histoire des insectes; *see* Fuessly, J.K.

Archives entomologiques; *see* Thomson, J.L.

Archives of entomology; *see* Fuessly, J.K.

Arctic searching expedition; *see* White, A.

Ausführliche Naturgeschichte des Thier-, Pflanzen- und Mineralreichs; *see* Schilling, P. S.

Baiersche Reise; *see* Schrank, F.

Balneis infantvm dissertatio adnexa Bvprestis descriptione; *see* Hotz, J.

Begrenzung der deutschen Necrophoren-Arten; *see* Michow, H.P.G.

Beitrag zur Käferfauna Ostfrieslands; *see* Wessel, A.W.

Beitrag zur Kenntniss der Coleopteren-Fauna der Balearen; *see* Schaufuss, L.W.

Beiträge zu der Insekten-Geschichte; *see* Scriba, L.G.

Beiträge zu einem natürlichen System der Coleopteren; *see* Preller, C.H.

Beiträge zur baierischen Insektenfaune; *see* Beck, J.M.

Beiträge zur Fauna von Aschaffenburg und Umgegend; *see* Fröhlich, C.A.A.T

Beiträge zur Insekten-Fauna Europas; *see* Rosenhauer, W.G.

Beiträge zur Insektenfauna Galiziens; *see* Nowicki, M.S.

Beiträge zur Kenntniss der kaukasischen Käferfauna; *see* Schneider, O.; Weise, J.

Bemerkungen, Berichtigungen und Zusätze zu Illiger’s Zusätzten; *see* Megerle von Mühlfeld, J.K.

Bericht über eine auf Madagascar veranstaltete Sammlung von Insecten; *see* Klug, J.C.F.

Beschreibung derjenigen Insecten; *see* Harrer, G.A.

Beschreibung einer neuen Käferart; *see* Nowicki, M.S.

Beschreibung neuer Käferarten; *see* Nowicki, M.S.

Beschreibung zweyer Decaden neuer und wenig bekannter Carabicinen; *see* Palliardi, A.A.

Beschreibvngen zv des Herrn D. Iacob Christian Schaeffers; *see* Harrer, G.A.

Bestimmungs-Tabelle der Borkenkäfer (Scolytidae); *see* Reitter, E.

Bestimmungs-Tabelle der Coleopteren-Familie der Cleriden; *see* Reitter, E.

Bestimmungs-Tabelle der Dytiscidae und Gyrinidae; *see* Seidlitz, G.

Bestimmungs-Tabelle der europäischen Coleopteren [Cantharidae]; *see* Procházka, J.

Bestimmungs-Tabelle der europäischen Coleopteren [Carabini]; *see* Reitter, E.

Bestimmungs-Tabelle der europäischen Coleopteren [Drilini]; *see* Reitter, E.

Bestimmungs-Tabelle der europäischen Coleopteren [Harpalini & Licinini]; *see* Reitter, E.

Bestimmungs-Tabelle der europäischen Coleopteren [Hylophilidae]; *see* Pic, M.

Bestimmungs-Tabelle der europäischen Coleopteren [Nitidulidae]; *see* Reitter, E.

Bestimmungs-Tabelle der europäischen Coleopteren [Scaritini]; *see* Fleischer, A.

Bestimmungs-Tabelle der europäischen Coleopteren [Zonitidae]; *see* Escherich, K.L.

Bestimmungs-Tabelle der europäischen Curculionidae; *see* Reitter, E.

Bestimmungs-Tabelle der Lucaniden und coprophagen Lamellicornen; *see* Reitter, E.

Bestimmungs-Tabelle der Melolonthidae aus der europäischen Fauna; *see* Reitter, E.

Bestimmungs-Tabelle der Tenebrioniden-Abtheilungen; *see* Reitter, E.

Bestimmungs-Tabelle der unechten Pimeliden; *see* Reitter, E.

Bestimmungs-Tabellen der europäischen Coleopteren [Bruchidae]; *see* Reitter, E.

Bestimmungs-Tabellen der europäischen Coleopteren [Coccinellidae]; *see* Weise, J.

Bestimmungs-Tabellen der europäischen Coleopteren [Coryssomerini & Baridiini]; *see* Reitter, E.

Bestimmungs-Tabellen der europäischen Coleopteren [Cryptorrhynchiden]; *see* Meyer, P.

Bestimmungs-Tabellen der europäischen Coleopteren [Cucujidae, &c.]; *see* Reitter, E.

Bestimmungs-Tabellen der europäischen Coleopteren [Erotylidae & Cryptophagidae]; *see* Reitter, E.

Bestimmungs-Tabellen der europäischen Coleopteren [Hydrophilini]; *see* Kuwert, A.F.

Bestimmungs-Tabellen der europäischen Coleopteren [Meloidae]; *see* Reitter, E.

Bestimmungs-Tabellen der europäischen Coleopteren [Necrophaga]; *see* Reitter, E.

Bestimmungs-Tabellen der europäischen Coleopteren [Phalacridae]; *see* Flach, K.

Bestimmungs-Tabellen der europäischen Coleopteren [Scaphidiidae, &c.]; *see* Reitter, E.

Bestimmungs-Tabellen der europäischen Coleopteren [Sphaeridiini & Helophorini]; *see* Kuwert, A.F.

Beytrag zu einer Monographie der Pselaphen; *see* Schmidt-Göbel, H.M.

Beyträge zur Naturgeschichte; *see* Schrank, F.

Bidrag till kännedom om insekternas geografiska utbredning i norden; *see* Mäklin, F.W.

Bidrag till kännedom om såkallade vikarierande former bland Coleoptera i norden; *see* Mäklin, F.W.

Bidrag till Tschuktsch-halföns Insektfauna; *see* Sahlberg, J.R.

Biologia Centrali-Americana; *see* Baly, J.S.; Bates, H.W.; Blandford, W.F.H.; Champion, G.C.; Gorham, H.S.; Horn, G.H.; Jacoby, M.; Lewis, G.; Matthews, A.; Sharp, D.; Waterhouse, C.O.

Bosquejo geográfico é histórico-natural del Archipiélago Filipino; *see* Jordana y Morera, R.

Bouwstoffen voor eene Fauna van Nederland; *see* Snellen van Vollenhoven, S.C.

Brachelytrorum species Agri Halensis; *see* Runde, W.H.

British beetles: an introduction to the study of our indigenous Coleoptera; *see* Rye, E.C.

British beetles. Transferred from Curtis’s British entomology; *see* Janson, E.W.

British Coleoptera delineated; *see* Shuckard, W.E.

British entomology; *see* Curtis, J.

British miscellany; *see* Sowerby, J.

Broscosoma, carabidum genus novum; *see* Putzeys, J.A.AH.

Broscosoma und Laricobius; *see* Rosenhauer, W.G.

Brouci. Soustavný popis brouků ve střední Evropě; *see* Klika, J.

Buprestidae; *see* Hope, F.W.

C.J. Schoenherr, genera et species curculionidum; *see* Jekel, H.

Cabinet cyclopaedia; *see* Swainson, W.

Cabinet of Oriental entomology; *see* Westwood, J.O.

Calendario entomologico; *see* Giorna, G.

Cambridge natural history; *see* Sharp, D.

Carabe d’Agassiz; *see* Barthélemy Lapommeraye, C.-J.

Carol. Pet. Thunberg. Characteres generum insectorum; *see* Meyer, F.A.A.

Caroli a Linné Systema Naturae; *see* Gmelin, J.F.; Beckmann, J.

Caroli Lib. Bar. De Geer… Genera et species insectorvm e generosissimi avctoris; *see* Retzius, A.J.

Caroli Linnaei entomologia, Faunae Suecicae; *see* Villers, C.J. de

Caroli Linnaei, Sueci, doctoris medicinae, systema naturae; *see* Fée, A.L.A.

Catalog der Coleopteren von Sibirien; *see* Heyden, L.F.J.D. von.

Catalog der Kaefer-Sammlung; *see* Sturm, J.

Catalog der preussischen Käfer; *see* Lentz, F.L.

Catalog der schweizerischen Coleopteren; *see* Bremi-Wolf, J.J.

Catalog meiner Insecten-Sammlung; *see* Sturm, J.

Cataloghi degli uccelli e degli insetti delle provincie di Padova e Venezia; *see* Contarini, N.B.

Cataloghi sistematici e descrittivi degli oggetti di storia naturale; *see* Cristofori, G. de.

Catalogi Coleopterorum Europae; *see* Stein, J.P.E.F.

Catálogo metódico de los Coleópteros de Menorca; *see* Cardona y Orfila, F.

Catálogo métódico y razonado de los coleópteros; *see* Cuní y Martorell, M.

Catalogo sinonimico e topografico dei Coleotteri d’Italia; *see* Bertolini, S.

Catálogo sistemático de toda la fauna de Filipinas; *see* Elera, C. de.

Catálogos sinonímicos de los insectos encontrados en Cataluña; *see* Martorell y Peña, M.

Catalogue de la collection de cicindélètes; *see* Chaudoir, M. de.

Catalogue de la collection de coléoptères de M. le Baron Dejean; *see* Dejean, P.F.M.A.

Catalogue de la collection entomologique. Classe des Insectes. Ordre des Coléoptères; *see* Milne-Edwards, H.

Catalogue des cétonides de la collection du Muséum d’Histoire Naturelle; *see* Blanchard, C.É.

Catalogue des coléoptères carabiques de la tribu des carabides; *see* Géhin, J.J.B.

Catalogue des coléoptères carnassiers aquatiques; *see* Van den Branden, C.

Catalogue des coléoptères de Belgique; *see* Kerremans, C.

Catalogue des coléoptères de France; *see* Gozis, M.G.P. des; Grenier, A.J.F.

Catalogue des coléoptères de l’Algérie; *see* Reiche, L.J.

Catalogue des coléoptères de l’Alsace; *see* Wencker, J.A.

Catalogue des coléoptères de la collection d’Auguste Dejean; *see* Dejean, P.F.M.A.

Catalogue des coléoptères de la collection de J.-B. Géhin; *see* Géhin, J.J.B.

Catalogue des coléoptères de la collection de M. le Comte Dejean; *see* Dejean, P.F.M.A.

Catalogue des coléoptères d’Europe; *see* Marseul, S.A. de

Catalogue des coléoptères d’Europe et du bassin de la Méditerranée; *see* Marseul, S.A. de

Catalogue des coléoptères gallo-rhénans; *see* Fauvel, C.A.A.

Catalogue des espèces d’insectes coléoptères recueillies par M. F. de Saulcy; *see* Reiche, L.J.

Catalogue des insectes du Portugal; *see* Oliveira, M.P.

Catalogue méthodique des élatérides connus en 1890; *see* Candèze, E.C.A.

Catalogue of British Coleoptera; *see* Crotch, G.R.; Sharp, D.; Waterhouse, G.R.

Catalogue [of] British insects; *see* Morris, F.O.

Catalogue of British insects; *see* Forster, J.R.

Catalogue of Coleoptera from the Japanese Archipelago; *see* Lewis, G.

Catalogue of coleopterous insects in the collection of the British Museum; *see* White, A.

Catalogue of Halticidae in the collection of the British Museum; *see* Clark, H.

Catalogue of Hispidae in the collection of the British Museum; *see* Baly, J.S.

Catalogue of Indian medicinal plants and drugs; *see* Fleming, J.

Catalogue of insects of Pennsylvania; *see* Melsheimer, F.V.

Catalogue of Phytophaga; *see* Clark, H.

Catalogue of the animals of North America; *see* Forster, J.R.

Catalogue of the Coleoptera of Scotland; *see* Murray, A.D.

Catalogue of the coleopterous insects in the collection of the British Museum; *see* Boheman, K.H.

Catalogue of the coleopterous insects of Madeira; *see* Wollaston, T.V.

Catalogue of the coleopterous insects of the Canaries; *see* Wollaston, T.V.

Catalogue of the described Coleoptera of Australia; *see* Masters, G.

Catalogue of the described Coleoptera of the United States; *see* Melsheimer, F.E.

Catalogue of the lucanoid Coleoptera; *see* Hope, F.W.

Catalogue of the species contained in the genus Buprestis; *see* Saunders, E.

Catalogue raisonné des coléoptères de Saone-&-Loire; *see* Fauconnet, L.

Catalogue raisonné des coléoptères du nord de l’Afrique; *see* Bedel, L.E.M.

Catalogue raisonné des objets de zoologie; *see* Ménétriés, E.

Catalogue synonymique des coléoptères d’Europe et d’Algérie; *see* Gaubil, J.

Catalogue synonymique et systématique des coléoptères de la tribu des carabides; *see* Géhin, J.J.B.

Catalogue systématique des Cicindelidae; *see* Fleutiaux, E.J.-B.

Catalogus buprestidarum synonymicus et systematicus; *see* Saunders, E.

Catalogus coleopterorum Europae, Caucasi et Armeniae rossicae; *see* Heyden, L.F.J.D. von.

Catalogus coleopterorum Europae et Caucasi; *see* Heyden, L.F.J.D. von.

Catalogus coleopterorum Europae; *see* Dohrn, C.A.; Schaum, H.R.; Stein, J.P.E.F.

Catalogus coleopterorum Haliciae; *see* Łomnicki, A.M.

Catalogus coleopterorum hucusque descriptorum synonymicus et systematicus; *see* Gemminger, M.

Catalogus coleopterorum in Sibiria orientali; *see* Fischer von Waldheim, J.G.

Catalogus coleopterorum lucanoidum; *see* Parry, F.J.S.

Catalogus musei zoologici; *see* Lichtenstein, A.A.H.

Catalogus systematicus coleopterorum; *see* Voet, J.E.

Catalogus van eene uitmuntende Verzameling; *see* Houttuyn, M.

Cenni zoologici; *see* Costa, A.

Centuria insectorum rariorum; *see* Linnaeus, C.

Centurie zoologique; *see* Lesson, R.P.

Čeští brouci; *see* Kliment, J.

Ceylon: an account of the island; *see* Walker, F.

Check list of the Coleoptera of America; *see* Crotch, G.R.

Civil and natural history of Jamaica; *see* Browne, P.

Class Insecta arranged by the Baron Cuvier; *see* Griffith, E.

Classification of the Coleoptera of North America; *see* LeConte, J.L.

Classified index and synopsis of the animal kingdom; *see* Griffith, E.

Cognitonem specierum Fennicarum generis Mycetopori symbolae; *see* Mäklin, F.W.

Coleoptera. A Magyar birodalom állatvilága; *see* Kuthy, D.

Coleoptera Atlantidum; *see* Wollaston, T.V.

Coleoptera chilensia; *see* Fairmaire, L.

Coleoptera Europae dupleta; *see* Galeazzi, G.; Villa, A.

Coleoptera Hesperidum; *see* Wollaston, T.V.

Coleoptera Jekeliana adjecta eleutheratorum bibliotheca; *see* Jekel, H.

Coleoptera microptera Brunsvicensia; *see* Gravenhorst, J.L.C.

Coleoptera Neerlandica; *see* Everts, E.J.G.

Coleoptera och Hemiptera, insamlade af Vega-expeditionens; *see* Sahlberg, J.R.

Coleoptera of Kansas and eastern New Mexico; *see* LeConte, J.L.

Coleoptera of Suffolk; *see* Morley, C.

Coleoptera of the British Islands; *see* Fowler, W.W.

Coleoptera Sanctae-Helenae; *see* Wollaston, T.V.

Coleoptera und Lepidoptera; *see* Dahl, G.

Coleopteren. Die Käfer Deutsch-Ost-Afrikas; *see* Kolbe, H.J.

Coleopteren-Studien; *see* Daniel, K.

Coléoptères du Mexique; *see* Chevrolat, L.A.A.

Coleopteri Italici. Genus novum Leptomastax; *see* Pirazzoli, O.

Coleopteris novis ac rarioribus minusve cognitis provinciae Novocomi; *see* Comolli, A.

Coleopteris, quae Oscarus et Alfredus Brehm in Africa; *see* Apetz, J.H.G.

Coleopterist’s manual; *see* Hope, F.W.

Coleopterorum novitates; *see* Oberthür, R.

Coleopterorum species novae aut minus cognitae; *see* Germar, E.F.

Complete writings of Thomas Say on the entomology of North America; *see* LeConte, J.L.

Concimazione del terreno vegetale per opera di alcuni Lamellicorni; *see* Mingazzini, P.

Consideraciones filosófico-naturales sobre los insectos; *see* Diaz y Lizana, R.

Considérations générales sur la classe des insectes; *see* Duméril, A.-M.-C.

Considérations générales sur l’ordre naturel des animaux; *see* Latreille, P.A.

Contribucion a la entomologia Cubana; *see* Gundlach, J.C.

Contributions to the descriptive and systematic coleopterology; *see* Casey, T.L.

Corrispondenza zoologica; *see* Costa, O.G.

Cours d’histoire naturelle; *see* Van der Stegen de Putte, J.F.P.

Creacion. Historia natural; *see* Taschenberg, E.L.; Vilanova y Piera, J.

Curculionidum dispositio methodica; *see* Schönherr, C.J.

Curculioniti dell’agro Pavese; *see* Prada, T.

Cuvier’s animal kingdom, arranged according to its organization; *see* Westwood, J.O.

Danske atlas eller Konge-Riget Dannemark; *see* Pontoppidan, E.L.

Danske Barkbiller; *see* Løvendal, E.A.

Das thierische Leben und sein Formen; *see* Zenker, J.C.

Das Thierreich; *see* Kneifl, R.

Das Thierreich eingetheilt nach dem Bau der Thiere; *see* Schinz, H.R.

Das zweckmässige Fangen, Tödten und Aufbewahren der Käfer; *see* Hoffmeister, P.

De balneis infantvm dissertatio adnexa Bvprestis descriptione; *see* Hotz, J.

De coleopteris novis ac rarioribus minusve cognitis provinciae Novocomi; *see* Comolli, A.

De coleopteris, quae Oscarus et Alfredus Brehm in Africa; *see* Apetz, J.H.G.

De Danske Barkbiller; *see* Løvendal, E.A.

De insectis venenatis agri Ticinensis; *see* Pensa, A.

De insectorum in America meridionali; *see* Perty, J.A.M.

De insectorum systemate naturali; *see* Burmeister, H.C.C.

De nederlandsche insecten; *see* Oudemans, J.T.

De quibusdam coleopteris agri Ticinensis; *see* Betta, V.

De quibusdam coleopteris Italiae; *see* Aragona, L.

Delectus animalium articulatorum; *see* Perty, J.A.M.

Deliciae florae et faunae Insubricae; *see* Scopoli, J.A.

Den danske atlas eller Konge-Riget Dannemark; *see* Pontoppidan, E.L.

Der Käferfreund; *see* Fleischer, H.; Reichenbach, A.B.

Der Käfersammler; *see* Hofmann, E.

Der Plauische Grund bei Dresden; *see* Block, P.L.H. von.

Der Société entomologique de Belgique zu Brüssel; *see* Schaufuss, L.W.

Der Trichterwickler; *see* Wasmann, E.

Des Ritters Carl von Linné Königlich schwedischen Leibarztes; *see* Müller, P.L.S.

Description de six nouvelles espèces de coléoptères hétéromères; *see* Allard, E.

Description des cols, ou passages des Alpes; *see* Jurine, L.

Description des dascillides du Bassin du Léman; *see* Tournier, H.

Description of a rare Scarabaeus from Potosi; *see* Francillon, J.

Description physique et naturelle de l’ile de Crète; *see* Raulin, V.

Descriptiones animalium avium, amphibiorum, piscium, insectorum, vermium; *see* Forsskål, P.

Descriptiones qvorvmdam Capensivm insectorvm; *see* Wulfen, F.X.F. von.

Descriptions de longicornes de Syrie; *see* Pic, M.

Descriptions of new genera and species of Phytophaga; *see* Baly, J.S.

Descriptions of new species of curculionites; *see* Say, T.

Descriptions of new species of North American insects; *see* Say, T.

Deutsche Käferwelt; *see* Schenkling, C.G.

Deutschlands Fauna; *see* Gillmeister, C.J.F.; Sturm, J.; Sturm, J.H.C.F.

Deutschlands Insectenfaune; *see* Panzer, G.W.F.

Deutschlands Petrefacten; *see* Giebel, C.G.

Diagnosen neuer blinder Käfer; *see* Dieck, G.

Dictionnaire vétérinaire, et des animaux domestiques; *see* Buchoz, P.J.

Die Begrenzung der deutschen Necrophoren-Arten; *see* Michow, H.P.G.

Die deutsche Käferwelt; *see* Schenkling, C.G.

Die europäischen Borkenkäfer; *see* Eichhoff, W.J.

Die exotischen Käfer in Wort und Bild; *see* Heyne, A.

Die Fauna und Flora des südwestlichen Caspi-Gebietes; *see* Leder, H.

Die Forst- und Baumzuchtschädlichen Borkenkäfer; *see* Ferrari, J.A.

Die Forst-Insecten; *see* Ratzeburg, J.T.C.

Die Forstkäfer; *see* Thiersch, E.L.

Die Gattungen der deutschen Kaefer-Fauna; *see* Redtenbacher, L.

Die Genera und Species meiner Cetonidensammlung; *see* Schoch, G.

Die Insecten-Doubletten; *see* Gistel, J. von N.F.X.

Die Insekten, Krebs- und Spinnenthiere; *see* Thon, T.

Die Insektenfauna der Tertiärgebilde; *see* Heer, O.

Die Insektenwelt; *see* Karsch, A.

Die Insel Cypern; *see* Kotschy, K.G.T.

Die Kaefer; *see* Ackermann, C.C.

Die Kaefer der Schweiz; *see* Heer, O.

Die Kaefer Russlands; *see* Motschulsky, V. de

Die Käfer der Mark Brandenburg; *see* Erichson, W.F.

Die Käfer der Steiermark; *see* Brancsik, K.

Die Käfer der Umgegend Aschaffenburgs; *see* Oechsner, G.

Die Käfer Deutschlands; *see* Gutfleisch, V.

Die Käfer Europa’s; *see* Schilsky, F.J.

Die Käfer Europas; *see* Kraatz, G.; Küster, H.C.

Die Käfer Siebenbürgens; *see* Fuss, K.A.

Die Käfer von Hamburg und Umgegend; *see* Preller, C.H.

Die Käfer von Mitteleuropa; *see* Ganglbauer, L.

Die Käfer von Tirol; *see* Gredler, V.M.

Die Käfer Westfalens; *see* Westhoff, F.

Die Kennzeichen der Insekten; *see* Sulzer, J.H.

Die kleinen Feinde der Landwirthschaft; *see* Nördlinger, H. von.

Die Lauf- und Schwimmkäfer Erlangens; *see* Rosenhauer, W.G.

Die mittleren Hochländer des nördlichen Deutsch-Ost-Afrika; *see* Kolbe, H.J.

Die Mysterien der europäischen Insectenwelt; *see* Gistel, J. von N.F.X.

Die nützlichen und schädlichen Forstkäfer für Forstbeamte; *see* Altmann, L.

Die otiorhynchiden; *see* Seidlitz, G.C.M. von.

Die schädlichen Forst- und Obstbaum-Insekten; *see* Henschel, G.

Die schweizerischen Käfergattungen; *see* Labram, J.D.

Die Thiere Andalusiens; *see* Rosenhauer, W.G.

Die Thierwelt Deutschlands und der Schweiz; *see* Calwer, C.G.

Die Urwelt der Schweiz; *see* Heer, O.

Die verbreitetsten Käfer Deutschlands; *see* Wünsche, O.

Discoveries in Australia; *see* White, A.

Dispvtatio inavgvralis de coccionellae natvra; *see* Linck, J.W.

Dispvtatio physico-medica inavgvralis, de insectis Coleopteris Danicis; *see* Detharding, G.C.

Dissertatio academica novas coleopterorum Fennicorum; *see* Sahlberg, R.F.

Dissertatio entomologica; *see* Linnaeus, C.

Dissertatio entomologica novas insectorum species; *see* Thunberg, C.P.

Dissertatio entomologica sistens insecta Svecica; *see* Thunberg, C.P.

Dissertatio entomologica. Insecta Fennica enumerans; *see* Sahlberg, C.R.

Dissertatio historico-naturalis ignotas insectorum species continens; *see* Quensel, C.

Dissertatio inauguralis enumerans genera Coleopterorum in Duftschmid; *see* Kliemstein, J.

Dissertatio inavgvralis medica exhibens catalogvm insectorvm Coleopterorvm; *see* Lehmann, J.C.

Dissertatio inauguralis medica sistens coleopterorum species agri Halensis; *see* Nicolai, E.A.

Dissertatio inauguralis sistens enumerationem systematicam curculionidum; *see* Krackowizer, J.C.S.

Dissertatio inauguralis zoologica de Pselaphis faunae Pragensis; *see* Schmidt-Göbel, H.M.

Dissertatio inauguralis zoologica sistens enumerationem coleopterorum; *see* Fischer, L.H.

Dissertatio inauguralis zoologico-medica; *see* Czenpiński, P.

Dissertatio medica, de Melöe-vesicatorio; *see* Linnaeus, C.

Doscientos coleópteros mas de Menorca; *see* Cardona y Orfila, F.

Doubletten-Verzeichniss von Senegallensischen Insecten; *see* Klug, J.C.F.

Drury’s Abbildungen und Beschreibungen exotischer Insecten; *see* Panzer, G.W.F.

Durch Afrika von Ost nach West; *see* Kaeseberg, K.

Durch Massailand zur Nilquelle; *see* Ganglbauer, L.

Edinburgh Encyclopaedia; *see* Leach, W.E.

Einige Cerambyciden der grossherzoglichen Sammlung zu Darmstadt; *see* Kaup, J.J.

Einleitung in die nähere Kenntnis der Insectenlehre; *see* Schmiedlein, G.B.

Élatérides nouveaux; *see* Candèze, E.C.A.

Elementa entomologica; *see* Schäffer, J.C.G. von.

Elementarbuch der Insektenkunde vorzüglich der Käfer; *see* Malinowsky, von.

Elementary text-book of entomology; *see* Kirby, W.F.

Elementi di storia naturale degli animali; *see* Pini, E.

Elements of British entomology; *see* Shuckard, W.E.

Elements of natural history; *see* Stark, J.

Elements of the natural history; *see* Stewart, C.

Elenco sistematico dei coleotteri; *see* Halbherr, B.

Enchiridion historiae naturali inserviens; *see* Forster, J.R.

Encyclopédie d’histoire naturelle; *see* Desmarest, E.A.S.L.

Encyclopédie méthodique; *see* Latreille, P.A.; Olivier, G.-A.

Endomycici recitati; *see* Gorham, H.S.

English entomologist; *see* Martyn, T.

Ensayo para la monografia de los coleópteros melóidos; *see* Górriz y Muñoz, R.J.

Entomographia imperii Russici; *see* Fischer von Waldheim, J.G.

Entomographien; *see* Erichson, W.F.; Eschscholtz, J.F. von; Gerstaecker, C.E.A.

Entomologia; *see* Brünnich, M.T.

Entomologia Britannica; *see* Marsham, T.

Entomologia Carniolica; *see* Scopoli, J.A.

Entomologia degli stagni; *see* Bracciforti, A.

Entomologia Edinensis; *see* Wilson, J.

Entomologia Parisiensis; *see* Fourcroy, A.F. de.

Entomologia systematica; *see* Fabricius, J.C.

Entomologia Vicentina; *see* Disconzi, F.

Entomologiae neapolitanae specimen primum; *see* Cirillo, D.M.L.

Entomological cabinet; *see* Samouelle, G.

Entomologie helvétique; *see* Clairville, J.P. de.

Entomologie, ou histoire naturelle des insectes; *see* Olivier, G.-A.

Entomologie, ou l’histoire naturelle des insectes; *see* Aucher-Éloy, P.M.R.

Entomologische Bemerkungen; *see* Hausmann, J.F.L.

Entomologische Beyträge; *see* Schellenberg, J.R.

Entomologische Beyträge zu des Ritter Linné zwölften Ausgabe des Natursystems; *see* Goeze, J.A.E.

Entomologische Hefte; *see* Hoffmann, J.J.J.

Entomologische Monographieen; *see* Klug, J.C.F.

Entomologische Reise nach dem südlichen Spanien; *see* Heyden, L.F.J.D. von.

Entomologische Versuche; *see* Creutzer, C.

Entomologisches Bilderbuch für junge Insektensammler; *see* Dunker, J.H.A.

Entomologisches Taschenbuch; *see* Andersch, J.D.; Brahm, N.J.; Hoppe, D.H.

Entomologist’s useful compendium; *see* Samouelle, G.

Entwurf der Insectenwissenschaft; *see* Steiner, J.F.R.

Enumeratio carabicorum Ticinensium; *see* Cadolini, L.

Enumeratio coleopterorum agri Monacensis; *see* Gistel, J. von N.F.X.

Enumeratio coleopterorum circa Heidelbergam; *see* Maehler, F.J.

Enumeratio insectorum in museo Gust. Joh. Billberg; *see* Billberg, G.J.

Enumeratio insectorum Norvegicorum; *see* Siebke, J.H.S.

Énumération des buprestides; *see* Kerremans, C.

Enumération des carabiques et hydrocanthares; *see* Chaudoir, M. de.

Envmeratio insectorvm Avstriae indigenorum; *see* Schrank, F.

Envmeratio insectorvm elevtheratorvm Capitis Bonae Spei; *see* Goldfuss, G.A.

Envmeratio insectorvm elytratorvm circa Erlangam; *see* Hoppe, D.H.

Ephemerides entomologicae; *see* Dalman, J.W.

Epitome entomologiae Fabricianae; *see* Borkhausen, M.B.

Epitome entomologiae systematicae secundum Fabricium; *see* Hentsch, G.F.

Epitome of the natural history of the insects of China; *see* Donovan, E.

Epitome of the natural history of the insects of India; *see* Donovan, E.

Epitome of the natural history of the insects of New Holland; *see* Donovan, E.

Ergebnisse entomologischer Excursionen im Gebiete Schässburgs; *see* Petri, K.

Erste Aufzählung der bis in Sachsen entdeckten Insekten; *see* Ludwig, C.F.

Esplorazione delle regioni equatoriali; *see* Osculati, G.

Essai d’une classification de la famille des cérambycides; *see* Thomson, J.L.

Essai monographique et iconographique de la tribu des cossyphides; *see* Brême, F. de.

Essai monographique sur la famille des throscides; *see* Bonvouloir, H.A. de.

Essai monographique sur le genre Pimelia; *see* Sénac, H.

Essai monographique sur les cisides; *see* Abeille de Perrin, E.E.A.

Essai monographique sur les clérites; *see* Spinola, M.

Essai pour servir a l’histoire des animaux du Midi de la France; *see* Serres, P.T.M. de

Essai sur la géographie physique, le climat et l’histoire naturelle du département du Doubs; *see* Girod-Chantrans, J.

Essai sur l’entomologie du Département du Puy-de-Dome; *see* Baudet-Lafarge, M.J.

Essai sur les collaptérides de la tribu des Molurites; *see* Solier, A.J.J.

Essai sur les insectes vésicants; *see* Ferrer, L.

Essais entomologiques; *see* Hummel, A.-D.

Essay on the genus Hydroscapha; *see* Matthews, A.

Étude sur les insectes vésicants; *see* Aubert, L.J.A.

Etudes entomologiques; *see* Laporte, F.L.; Levrat, J.N.B.G.; Motschulsky, V. de

Études sur les coléoptères cavernicoles; *see* Abeille de Perrin, E.E.A.

Eucnemis, insectorum genus monographice; *see* Mannerheim, C.G. von.

Europäische Fauna; *see* Donndorff, J.A.

Europäische Höhlenfauna; *see* Hamann, O.

Europäischen Borkenkäfer; *see* Eichhoff, W.J.

Excursion entomologique dans les montagnes de la vallée d’Ossau; *see* Dufour, L.J.M.

Exotischen Käfer in Wort und Bild; *see* Heyne, A.

Expédition scientifique de Morée; *see* Brullé, G.A.

Exploration and survey of the valley of the Great Salt Lake; *see* Haldeman, S.S.

Exploration of Mount Kina Balu; *see* Bates, H.W.; Gorham, H.S.

Exploration scientifique de l’Algérie; *see* Lucas, P.H.

Exploration scientifique de la Tunisie; *see* Bedel, L.E.M.; Lefèvre, E.

Extrait du cours de zoologie; *see* Lamarck, J.B.P.A.

Fabricia Entomologica; *see* Jekel, H.

Familles naturelles du règne animal; *see* Latreille, P.A.

Fauna Austriaca. Die Käfer; *see* Redtenbacher, L.

Fauna Austriae; *see* Duftschmid, C.E.

Fauna Baltica. Die Käfer; *see* Seidlitz, G.C.M. von.

Fauna Boreali-Americana; *see* Kirby, W.

Fauna coleopterorum Helvetica; *see* Heer, O.; Stierlin, W.G.

Fauna del Regno di Napoli; *see* Costa, A.

Fauna der Vorwelt mit steter Berücksichtigung der lebenden Thiere; *see* Giebel, C.G.A.

Fauna Etrusca; *see* Rossi, P.

Fauna Hawaiiensis; *see* Perkins, R.C.L.; Sharp, D.

Fauna insectorum Europae; *see* Ahrens, J.F.A.; Germar, E.F.

Fauna insectorum Lapponica; *see* Zetterstedt, J.W.

Fauna kornjašah trojedne kraljevine; *see* Schlosser, J.C.

Fauna Ratisbonensis; *see* Herrich-Schäffer, G.A.

Fauna Salentina; *see* Costa, G.

Fauna Svecica. Insecta; *see* Paykull, G.

Fauna Svecica sistens animalia Sveciae regni; *see* Linnaeus, C.

Fauna Transsylvanica. Die Kaefer; *see* Seidlitz, G.C.M. von.

Fauna und Flora des südwestlichen Caspi-Gebietes; *see* Leder, H.

Faunae insectorum Americes borealis; *see* Panzer, G.W.F.

Faunae insectorum Germanicae; *see* Geyer, K.; Herrich-Schäffer, G.A.; Panzer, G.W.F.

Faune analytique des coléoptères de France; *see* Fauconnet, L.

Faune avignonaise; *see* Fabre, J.-H.C.

Faune de France contenant la description de toutes les espèces indigènes; *see* Acloque, A.N.C.

Faune de la Moselle; *see* Fournel, D.H.L.

Faune des coléoptères du Bassin de la Seine; *see* Bedel, L.E.M.

Faune des invertébrés de Maine-et-Loire; *see* Millet de la Turtaudière, P.-A.

Faune entomologique de l’Andalousie; *see* Rambur, P.-J.

Faune entomologique des environs de Paris; *see* Boisduval, J.B.A.D. de.

Faune entomologique française; *see* Fairmaire, L.

Faune et flore des pays Çomalis (Afrique orientale); *see* Fairmaire, L.

Faune française; *see* Audinet-Serville, J.-G.

Faune gallo-rhénane; *see* Bourgeois, J.; Buysson, H.J.C. du; Fauvel, C.A.A.

Faune parisienne, insectes; *see* Walckenaer, C.A.

Faunula coleopterorum Birmaniae; *see* Schmidt-Göbel, H.M.

Favna Boica; *see* Schrank, F.

Favna Etrvsca; *see* Hellwig, J.C.L.

Favna Groenlandica; *see* Fabricius, O.

Favna insectorvm Fridrichsdalina; *see* Müller, O.F.

Favnae Ingricae; *see* Cederhjelm, J.

Fest-Schrift zur Feier des fünfzigjährigen Bestehens des Vereins; *see* Reitter, E.

Florae Italicae prodromus; *see* Turra, A.

Forst-Insecten; *see* Ratzeburg, J.T.C.

Forstliches und forstnaturwissenschaftliches Conversations-Lexicon; *see* Hartig, G.L.

Forstinsektologie; *see* Bechstein, J.M.

Forstkäfer; *see* Thiersch, E.L.

Forst- und Baumzuchtschädlichen Borkenkäfer; *see* Ferrari, J.A.

Forstzoologie; *see* Altum, J.B.T.

Förteckning öfver Skandinaviens, Danmarks och Finlands Coleoptera; *see* Grill, C.E.

Fundamenta entomologiae; *see* Linnaeus, C.

Fundamenta entomologiae: or, an introduction to the knowledge of insects; *see* Curtis, W.

Fur seals and fur-seal islands; *see* Linell, M.L.

Gattungen der deutschen Kaefer-Fauna; *see* Redtenbacher, L.

Gemeinnützige Naturgeschichte; *see* Lenz, H.O.

Gemeinnützliche Naturgeschichte der giftigen Insecten; *see* Meyer, F.A.A.

Genera crustaceorum et insectorum; *see* Latreille, P.A.

Genera des coléoptères d’Europe; *see* Fairmaire, L.; Jacquelin du Val, P.N.C.

Genera des coléoptères de France; *see* Fauconnet, L.

Genera des insectes; *see* Guérin-Méneville, F.É.

Genera dyticeorum; *see* Erichson, W.F.

Genera et species curculionidum; *see* Schönherr, C.J.

Genera et species staphylinorum; *see* Erichson, W.F.

Genera insectorum Linnaei et Fabricii; *see* Römer, J.J.

Genera insectorum of Linnaeus; *see* Barbut, J.

Genera insectorvm; *see* Fabricius, J.C.

Genera of British Coleoptera; *see* Curtis, J.

Genera og species af Danmarks Eleutherata; *see* Schiødte, J.M.C.

Genera quaedam insectorum; *see* Burmeister, H.C.C.

Genera und Species meiner Cetonidensammlung; *see* Schoch, G.

General natural history; *see* Hill, J.

General system of nature; *see* Turton, W.

General zoology or systematic natural history; *see* Shaw, G.

Geodephaga Britannica; *see* Dawson, J.F.

Géognosie des terrains tertiaires; *see* Serres, P.T.M. de.

Geographisch-physikalische und naturhistorische Beschreibung; *see* Georgi, J.G.

Geographische, naturhistorische und technologische Beschreibung; *see* Weigel, J.A.V.

Geographische Naturkunde von Kurhessen; *see* Schwaab, W.

Geology and mineralogy considered with reference to natural theology; *see* Buckland, W.

Geology of Oxford and The Valley of The Thames; *see* Phillips, J.

Glanures entomologiques; *see* Jacquelin du Val, P.N.C.

Gróf Széchenyi Béla Keletázsiai utjának tudományos Eredménye; *see* Frivaldszky, J.

Grundlage zur Fauna Steyermark’s dargestellt durch das Coleoptern-Verzeichniss; *see* Grimmer, K.H.B.

Grundlage zur Kenntniss der Käfer Oberschlesiens; *see* Kelch, J.A.

Grundlage zur Kenntniss der um Sondershausen vorkommenden Käfer; *see* Göbel, F.

Gründliche Anweisung; *see* Hahn, C.W.

Grundriss der Zoologie; *see* Goldfuss, G.A.

Guide du naturaliste dans les trois regnes; *see* Van der Stegen de Putte, J.F.P.

Guide to an arrangement of British insects; *see* Curtis, J.

Handboek der dierkunde; *see* Hoeven, J. van der.

Handbook of the Coleoptera; *see* Cox, H.E.

Handbuch der deutschen Thiergeschichte für Schulen; *see* Anonymous.

Handbuch der Entomologie; *see* Burmeister, H.C.C.

Handbuch der Landeskunde Siebenbürgens; *see* Bielz, E.A.

Handbuch der Naturgeschichte; *see* Blumenbach, J.F.; Burmeister, H.C.C.

Handbuch der Naturgeschichte der drei Reiche; *see* Gräfe, H.G.A.

Handbuch der Naturgeschichte des Thierreichs; *see* Wilbrand, J.B.

Handbuch der Naturgeschichte für die Jugend; *see* Wilmsen, F.P.

Handbuch der Zoologie; *see* Döbner, E.P.; Fischer, S.C.; Gerstaecker, C.E.A.; Goldfuss, G.A.; Wiegmann, A.F.A.

Handbuch für Käfer-Sammler; *see* Bau, A.

Hernstein in Niederösterreich; *see* Beck, G.

Histoire abrégée des insectes; *see* Geoffroy, E.L.

Histoire d’un petit crustacé; *see* Joly, N.

Histoire des coléoptères de France; *see* Sériziat, C.V.É.

Histoire des insectes, traitant de leurs moeurs et de leurs métamorphoses; *see* Blanchard, C.É.

Histoire naturelle de la France. Coléoptères; *see* Fairmaire, L.

Histoire naturelle des animaux sans vertèbres; *see* Deshayes, G.P.; Lamarck, J.B.P.A.

Histoire naturelle des coléoptères de France; *see* Belon, P.M.J.; Foudras, A.C.M.E.; Mulsant, M.E.; Rey, C.

Histoire naturelle des fourmis; *see* Latreille, P.A.

Histoire naturelle des Iles Canaries; *see* Brullé, G.A.

Histoire naturelle des insectes; *see* Brullé, G.A.; Chapuis, F.; Lacordaire, J.T.; Tigny, F.M.G.T. de

Histoire naturelle des insectes coléoptères; *see* Laporte, F.L.

Histoire naturelle des principales productions de l’Europe méridionale; *see* Risso, J.A.

Histoire naturelle du Département des Pyrénées-Orientales; *see* Companyo, B.-J.-L.

Histoire naturelle du Jorat et de ses environs; *see* Razumovsky, G.K.

Histoire naturelle du Jura et des départements voisins; *see* Étienne, J.A.C.

Histoire naturelle et iconographie des insectes coléoptères; *see* Gory, H.L.; Laporte, F.L.

Histoire naturelle et iconographie des insectes coléoptères d’Europe; *see* Latreille, P.A.

Histoire naturelle, générale et particulière, des crustacés et des insectes; *see* Latreille, P.A.

Histoire physique, naturelle et politique de Madagascar; *see* Alluaud, C.; Künckel d’Herculais, J.P.A.

Histoire physique, politique et naturelle de l’Ile de Cuba; *see* Jacquelin du Val, P.N.C.

Historia fisica politica y natural de la Isla de Cuba; *see* Jacquelin du Val, P.N.C.

Historia fisica y politica de Chile; *see* Blanchard, C.É.; Solier, A.J.J.; Spinola, M.

Historia naturalis curculionum Sveciae; *see* Bonsdorff, G. von.

History of Egyptian mummies; *see* Hope, F.W.

History of Glanville’s Wootton; *see* Dale, C.W.

History of the fossil insects in the Secondary rocks of England; *see* Brodie, P.B.

Horae entomologicae; *see* Charpentier, T. de.; Macleay, W.S.

Hydrocanthares de la Russie; *see* Motschulsky, V. de

Icones insectorvm circa Ratisbonam; *see* Schäffer, J.C.G. von.

Icones insectorvm praesertim Rossiae Sibiriaeqve; *see* Pallas, P.S.

Iconographie des insectes; *see* Milne-Edwards, H.

Iconographie du règne animal de G. Cuvier; *see* Guérin-Méneville, F.É.

Iconographie et histoire naturelle des coléoptères d’Europe; *see* Aubé, C.N.; Dejean, P.F.M.A.

Il libro dei Coleotteri; *see* Griffini, A.

Iles Açores; *see* Tarnier, F.

Illustratio iconographica insectorum; *see* Coquebert, A.J.

Illustrations de zoologie; *see* Lesson, R.P.

Illustrations of British entomology; *see* Stephens, J.F.

Illustrations of exotic entomology; *see* Westwood, J.O.

Illustrations of natural history; *see* Drury, D.

Illustrations of the annulosa of South Africa; *see* Macleay, W.S.

Illustrations of the botany and other branches of the natural history; *see* Westwood, J.O.

Illustrations of the Linnaean genera of insects; *see* Wood, W.

Illustrations of typical specimens of Coleoptera; *see* Waterhouse, C.O.

In faunam insectorum Rossicam symbola; *see* Sahlberg, R.F.

Index alphabeticus in J.C. Fabricii Entomologiam Systematicam; *see* Fabricius, J.C.

Index alphabeticus in J.C. Fabricii Supplementum Entomologiae Systematicae; *see* Fabricius, J.C.

Index entomologicvs sistens omnes insectorum species; *see* Panzer, G.W.F.

Informe oficial de la comision científica; *see* Berg, F.W.K.

Insecta Caffraria; *see* Boheman, K.H.

Insecta Lapponica; *see* Zetterstedt, J.W.

Insecta Maderensia; *see* Wollaston, T.V.

Insecta musei Graecensis; *see* Poda von Neuhaus, N.

Insecta Saundersiana [Buprestidae]; *see* Saunders, E.

Insecta Saundersiana [Curculionides]; *see* Jekel, H.

Insecta Senarum in agro suburbano; *see* Dei, G.A.A.C.

Insecta Suecica; *see* Gyllenhal, L.

Insecten-Doubletten; *see* Gistel, J. von N.F.X.

Insectes; *see* Girard, M.J.A.

Insectes coléoptères du département des Alpes-Maritimes; *see* Peragallo, A.B.H.

Insectes recueillis en Afrique et en Amérique; *see* Palisot de Beauvois, A.–M.-F.-J.

Insectes vésicants; *see* Beauregard, H.E.

Insectis venenatis agri Ticinensis; *see* Pensa, A.

Insectorum in America meridionali; *see* Perty, J.A.M.

Insectorum systemate naturali; *see* Burmeister, H.C.C.

Insectos de Costa Rica; *see* Tristán, J.F.

Insects; *see* Sharp, D.

Insects abroad; *see* Wood, J.G.

Insects at home; *see* Wood, J.G.

Insekten der Schweiz; *see* Labram, J.D.

Insekten, Krebs- und Spinnenthiere; *see* Thon, T.

Insektenfauna der Tertiärgebilde; *see* Heer, O.

Insektenkalender für Sammler und Oekonomen; *see* Brahm, N.J.

Insektenwelt; *see* Karsch, A.

Insel Cypern; *see* Kotschy, K.G.T.

Institutiones entomologicae; *see* Petagna, V.

Institutions of entomology; *see* Yeats, T.P.

Introductio in oryctographiam, et zoologiam Aragoniæ; *see* Asso y del Río, I.J.C. de.

Introduction to entomology; *see* Kirby, W.

Introduction to the modern classification of insects; *see* Westwood, J.O.

Introdvctio ad historiam natvralem; *see* Scopoli, J.A.

Invertebrados de Costa Rica. I. Coleópteros; *see* Pittier, H.F.

Iter per Poseganam Sclavoniae; *see* Piller, M.

Jahrbücher der Insektenkunde; *see* Klug, J.C.F.

Johann Euseb Voets Beschreibungen und Abbildungen hartschaaligter Insecten; *see* Panzer, G.W.F.

Journals of two expeditions of discovery in north-west and western Australia; *see* White, A.

Kaefer; *see* Ackermann, C.C.

Kaefer der Schweiz; *see* Heer, O.

Kaefer Russlands; *see* Motschulsky, V. de

Käfer der Mark Brandenburg; *see* Erichson, W.F.

Käfer der Steiermark; *see* Brancsik, K.

Käfer der Umgegend Aschaffenburgs; *see* Oechsner, G.

Käfer Deutschlands; *see* Gutfleisch, V.

Käfer Europa’s; *see* Schilsky, F.J.

Käfer Europas; *see* Kraatz, G.; Küster, H.C.

Käfer Siebenbürgens; *see* Fuss, K.A.

Käfer von Hamburg und Umgegend; *see* Preller, C.H.

Käfer von Mitteleuropa; *see* Ganglbauer, L.

Käfer von Tirol; *see* Gredler, V.M.

Käfer Westfalens; *see* Westhoff, F.

Käferbuch; *see* Berge, K.F.W.; Calwer, C.G.

Käfer-Büchlein; *see* Gebauer, C.A.

Käferfauna für Nord- und Mitteldeutschland; *see* Bach, M.

Käfer-Fauna Hildesheims; *see* Wilken, C.

Käferfreund; *see* Fleischer, H.; Reichenbach, A.B.

Käfersammler; *see* Hofmann, E.

Kalocsa környékének bogárfaunája; *see* Speiser, F.

Kaschmir und das Reich der Siek; *see* Kollar, V.

Kennzeichen der Insekten; *see* Sulzer, J.H.

Kleinen Feinde der Landwirthschaft; *see* Nördlinger, H. von.

Kleiner Beytrag zur Entomologie; *see* Trost, P.

Kongliga Svenska Fregatten Eugenies Resa; *see* Boheman, K.H.

Kort underrättelse om Skandinaviska insekters; *see* Dahlbom, A.G.

Kritische Revision der Insektenfaune Deutschlands; *see* Panzer, G.W.F.

Kritisches Verzeichniss der myrmekophilen und termitophilen Arthropoden; *see* Wasmann, E.

Kurze Einleitung zur Kenntniss der Insekten; *see* Herbst, J.F.W.

Kurzer Abriss der Entomologie; *see* Altmann, L.

L’entomologia degli stagni; *see* Bracciforti, A.

L’entomologie, ou l’histoire naturelle des insectes; *see* Aucher-Éloy, P.M.R.

La concimazione del terreno vegetale per opera di alcuni Lamellicorni; *see* Mingazzini, P.

La creacion. Historia natural; *see* Taschenberg, E.L.; Vilanova y Piera, J.

Lake Superior; *see* LeConte, J.L.

Lamellicornia melitophila; *see* Schoch, G.

Landshutische Nebenstunden zur Erweiterung der Naturgeschichte; *see* Schrank, F.

Latreille’s Natürliche Familien des Thierreichs; *see* Berthold, A.A.

Lauf- und Schwimmkäfer Erlangens; *see* Rosenhauer, W.G.

Le guide du naturaliste dans les trois regnes; *see* Van der Stegen de Putte, J.F.P.

Le petit entomologiste collecteur au nord de Paris; *see* Maillard, J.

Le règne animal distribué d’après son organisation; *see* Audouin, J.-V.; Latreille, P.A.

Lebensweise von Forstkerfen; *see* Nördlinger, H. von.

Leçons d’anatomie comparée de G. Cuvier; *see* Duméril, A.-M.-C.

Lehrbuch der mitteleuropäischen Forstinsektenkunde; *see* Judeich, J.F.

Lehrbuch der Naturgeschichte; *see* Burmeister, H.C.C.; Oken, L.

Lehrbuch der Zoologie; *see* Krassow, C.R.A. von; Schulz, J.H.; Thienemann, F.A.L.; Voigt, F.S.

Lehrbuch des Forstschutzes; *see* Nördlinger, H. von.

Les insectes; *see* Girard, M.J.A.

Les insectes coléoptères du département des Alpes-Maritimes; *see* Peragallo, A.B.H.

Les insectes vésicants; *see* Beauregard, H.E.

Lettre adressée au nom de la Société impériale des Naturalistes de Moscou; *see* Fischer von Waldheim, J.G.

Lettres à Julie sur l’entomologie; *see* Mulsant, M.E.

Libro dei Coleotteri; *see* Griffini, A.

Life in the wilderness; *see* White, A.

Lijst der in Nederland voorkomende schildvleugelige Insecten; *see* Everts, E.J.G.

Lijst der Nederlandsche Coleoptera; *see* Everts, E.J.G.

List of all the Coleoptera described A.D. 1758–1821; *see* Crotch, G.R.

List of British Curculionidae; *see* Walton, J.

List of Coccinellidae; *see* Crotch, G.R.

List of Coleoptera collected by J.K. Lord; *see* Walker, F.

List of the Australian longicorns; *see* Pascoe, F.P.

List of the Coleoptera of America; *see* Henshaw, S.

List of the Coleoptera of North America; *see* LeConte, J.L.

List of the coleopterous insects in the collection of the British Museum; *see* Smith, F.

List of the described longicornia of Australia and Tasmania; *see* Pascoe, F.P.

Los tres reinos de la naturaleza; *see* Galdo López de Neira, M.M.J. de

Mantissa insectorum exibens species nuper in Etruria; *see* Rossi, P.

Mantissa insectorvm; *see* Fabricius, J.C.

Mantissa plantarum; *see* Linnaeus, C.

Mantissa secunda familiae Curculionidum; *see* Schönherr, C.J.

Mantissae insectorvm iconibvs illvstratae species novas; *see* Reich, G.C.

Manual of British Coleoptera; *see* Stephens, J.F.

Manual of natural history, for the use of travellers; *see* Adams, A.

Manual of the New Zealand Coleoptera; *see* Broun, T.

Manuali Hoepli [Coleotteri italiani]; *see* Griffini, A.

Manuel de la faune de Belgique; *see* Lameere, A.A.L.

Manuel d’entomologie; *see* Boitard, P.

Matabele land and the Victoria Falls; *see* Westwood, J.O.

Matériaux pour la faune entomologique de la province de Limbourg; *see* Preudhomme de Borre, C.F.P.A.

Matériaux pour servir à l’étude des longicornes; *see* Pic, M.

Matériaux pour servir à la faune des coléoptères de France; *see* Grenier, A.J.F.

Med. Dr. Joh. Wilh. Helfer’s hinterlassene Sammlungen; *see* Schmidt-Göbel, H.M.

Medizinische Zoologie; *see* Brandt, J.F. von.

Mélanges entomologiques; *see* Perroud, B.-P.

Mélanges entomologiques sur les insectes du Portugal; *see* Oliveira, M.P.

Meletemata entomologica; *see* Kolenati, F.A.

Mémoire sur la famille des clérites; *see* Chevrolat, L.A.A.

Mémoires pour servir a l’histoire des insectes; *see* DeGeer, C.

Mémoires pour servir a l’histoire naturelle de la Provence; *see* Bernard, P.-J.

Memoranda relating to coleopterous insects; *see* Dillwyn, L.W.

Memoria sobre los insectos epispásticos; *see* Amor y Mayor, F.

Memorias de la comision del mapa geológico de España; *see* Graells y de la Agüera, M.

Memorias sobre la historia natural de la Isla de Cuba; *see* Poey, F.

Mission from Cape Coast Castle to Ashantee; *see* Leach, W.E.

Mission scientifique du Cap Horn; *see* Fairmaire, L.

Mittleren Hochländer des nördlichen Deutsch-Ost-Afrika; *see* Kolbe, H.J.

Monograph of Christmas Island; *see* Waterhouse, C.O.

Monograph of the coleopterous families Corylophidae and Sphaeriidae; *see* Matthews, A.

Monographia amaroidum; *see* Zimmermann, K.C.A.

Monographia apum Angliae; *see* Kirby, W.

Monographia cantharidum et malachiorum Sveciae; *see* Fallén, C.F.

Monographia caraborum Sueciae; *see* Paykull, G.

Monographia cassididarum; *see* Boheman, K.H.

Monographia chlamydum; *see* Kollar, V.

Monographia coleopterorvm micropterorvm; *see* Gravenhorst, J.L.C.

Monographia curculionum Sueciae; *see* Paykull, G.

Monographia histeroidum; *see* Paykull, G.

Monographia mylabridum; *see* Billberg, G.J.

Monographia pselaphidarum et scydmaenidarum Britanniae; *see* Denny, H.

Monographia pselaphorvm; *see* Reichenbach, H.G.L.

Monographia staphylinorum Sueciae; *see* Paykull, G.

Monographie de la famille des eucnémides; *see* Bonvouloir, H.A. de.

Monographie de quelques genres de coléoptères hétéromères; *see* Brême, F. de.

Monographie der Carabiden; *see* Zimmermann, K.C.A.

Monographie der Curculioniden-Gattung Peritelus; *see* Seidlitz, G.C.M. von.

Monographie der europäischen Arten der Gattung Meloë; *see* Katter, F.C.A.

Monographie der paläarktischen Cicindelen; *see* Horn, H.W.W.

Monographie der Passaliden; *see* Kaup, J.J.

Monographie des Anthicus; *see* LaFerté-Sénectère, F.T.

Monographie des cétoines et genres voisins; *see* Gory, H.L.

Monographie des cicindélides; *see* Thomson, J.L.

Monographie des Clivina et genres voisins; *see* Putzeys, J.A.AH.

Monographie des coccinellides; *see* Mulsant, M.E.

Monographie des coléoptères subpentamères; *see* Lacordaire, J.T.

Monographie des élatérides; *see* Candèze, E.C.A.

Monographie des erotyliens; *see* Lacordaire, J.T.

Monographie des mylabrides; *see* Marseul, S.A. de.

Monographie des passales; *see* Percheron, A.R.

Monographie des platypides; *see* Chapuis, F.

Monographie du genre Clytus; *see* Laporte, F.L.

Monographie du genre Sisyphe; *see* Gory, H.L.

Mormolyce novum coleopterorum genus; *see* Hagenbach, J.J.

Munus rectoris in Academia Christiana Albertina; *see* Wiedemann, C.R.W.

Musée entomologique illustré; *see* Rothschild, J.

Musée scientifique; *see* Thomson, J.L.

Museum Demidoff; *see* Fischer von Waldheim, J.G.

Museum historiae naturalis Universitatis Caesareae Mosquensis; *see* Fischer von Waldheim, J.G.

Museum N. G. Leskeanum; *see* Zschach, J.J.

Museum naturalium Academiae Upsaliensis; *see* Thunberg, C.P.

Museum S:ae R:ae M:tis Ludovicae Ulricae Reginae; *see* Linnaeus, C.

Musevm Geversianvm; *see* Meuschen, F.C.

Musevm Gronovianvm; *see* Meuschen, F.C.

Mysterien der europäischen Insectenwelt; *see* Gistel, J. von N.F.X.

Nachtrag zu den Gattungen und Arten meiner Cetoniden-Sammlung; *see* Schoch, G.

Nachträge für dessen Geographisch-physikalische und naturhistorische Beschreibung; *see* Georgi, J.G.

Nachträge zu Ratzeburg’s Forstinsekten; *see* Nördlinger, H. von.

Narrative of a survey of the intertropical and western coasts of Australia; *see* Macleay, W.S.

Narrative of an expedition to the source of St. Peter’s River; *see* Say, T.

Narrative of the voyage of H.M.S. Rattlesnake; *see* White, A.

Natural history of British insects; *see* Donovan, E.

Natural history of the Azores; *see* Crotch, G.R.

Natural history of Tutbury; *see* Brown, E.

Naturalist in the Transvaal; *see* Distant, W.L.; Gahan, C.J.

Naturalist in Vancouver Island and British Columbia; *see* Walker, F.

Naturalist’s library. Beetles; *see* Duncan, J.

Naturalist’s repository; *see* Donovan, E.

Naturalist’s wanderings in the eastern Archipelago; *see* Janson, O.E.; Waterhouse, C.O.

Naturgeschichte der in Deutschland einheimischen Käfer; *see* Fricken, H.W.M. von.

Naturgeschichte der Insecten; *see* Glaser, L.

Naturgeschichte der Insecten Deutschlands; *see* Erichson, W.F.; Kiesenwetter, E.A.H. von; Kraatz, G.; Reitter, E.; Schaum, H.R.; Seidlitz, G.C.M. von; Weise, J.

Naturgeschichte der Insekten; *see* Bouché, P.F.; Suckow, F.W.L.

Naturgeschichte der schädlichen Nadelholz-Insecten; *see* Zinke, G.G.

Naturgeschichte der schädlichen und nützlichen Garten-Inskecten; *see* Bouché, P.F.

Naturgeschichte des Thierreichs; *see* Gistel, J. von N.F.X.

Naturgeschichte im Auszuge des Linnéischen-Systems; *see* Esper, E.J.C.

Naturgeschichte schädlicher Thiere; *see* Zinke, G.G.

Naturhistorische Briefe; *see* Schrank, F.

Naturhistorische Reise durch einen Theil Schwedens; *see* Weber, F.

Naturhistorische und ökonomische Briefe über das Donaumoor; *see* Schrank, F.

Natursystem aller bekannten in- und ausländischen Insekten; *see* Herbst, J.F.W.; Jablonsky, C.G.

Naturwissenschaftliche Reise nach Mossambique; *see* Gerstaecker, C.E.A.; Klug, J.C.F.

Natuurkundige verhandelingen van de Hollandsche maatschappij; *see* Bennet, J.A.

Natuurlijke historie; *see* Ritsema, C.

Natuurlyke historie; *see* Houttuyn, M.

Nederlandsche insecten; *see* Oudemans, J.T.

Neue Beyträge zur Insectenkunde; *see* Knoch, A.W.

Neues Verzeichniss der Preussischen Käfer; *see* Lentz, F.L.

Neuestes und vollständigstes Handbuch der Naturgeschichte für Lehrer und Lernende; *see* Gistel, J. von N.F.X.

New species of North American Coleoptera; *see* LeConte, J.L.

New species of North American insects; *see* Say, T.

Nicolai Josephi Jacquin collectanea ad botanicam; *see* Host, N.T.

Nicolai Josephi Jacquin miscellanea austriaca; *see* Well, J.J. von.

Nomenclator entomologicus; *see* Herrich-Schäffer, G.A.; Schneider, D.H.

Nomenclator entomologicus secundum Entomologiam Systematicam; *see* Weber, F.

Nomenclator über die in den Röselschen Insekten; *see* Schwarz, C.

Nomenclator zoologicus; *see* Agassiz, J.L.R.; Marschall, A.F.; Scudder, S.H.

Nomenclatoris zoologici; *see* Agassiz, J.L.R.

Nomenclatur und Beschreibung der Insecten; *see* Bergsträsser, J.A.B.

Nomenclature of British entomology; *see* Samouelle, G.

Nomenclature of British insects; *see* Stephens, J.F.

Nomenclature of coleopterous insects in the collection of the British Museum; *see* Smith, F.; White, A.

Notas para la fáuna Gallega; *see* López Seoane, V.

Note d’un viaggio nella Persia e nelle Indie orientali; *see* Osculati, G.

Notes and descriptions of a few injurious farm; *see* Ormerod, E.A.

Notes sur l’île de la Réunion (Bourbon); *see* Deyrolle, A.

Notice sur l’entomologie du Bourbonnais; *see* Desbrochers des Loges, J.

Nouvelles illustrations de zoologie; *see* Brown, P.

Nouvelles lettres pour servir à l’histoire des insectes de la tribu des carabides; *see* Géhin, J.J.B.

Novae species insectorum; *see* Forster, J.R.

Novae species insectorvm; *see* Schreber, J.C.D. von.

Nützlichen und schädlichen Forstkäfer für Forstbeamte; *see* Altmann, L.

Observationes entomologicae; *see* Fallén, C.F.; Heer, O.; Weber, F.

Observationes nonnullae in Coleoptera Indiae Orientalis; *see* Perty, J.A.M.

Observations; *see* Chaudoir, M. de.

Observations entomologiques; *see* Bonelli, F.A.

Observations on marine vermes, insects; *see* Martin, M.

Olivier’s Entomologie oder Naturgeschichte der Insekten; *see* Illiger, J.C.W.

Opatum insecti genus; *see* Thunberg, C.P.

Opera, historiam naturalem spectantia; *see* Petiver, J.

Opuscula entomologica; *see* Thomson, C.G.

Opvscvla entomologica; *see* Schäffer, J.C.G. von.

Opuscules entomologiques (coléoptères); *see* Desbrochers des Loges, J.

Opuscules entomologiques; *see* Mulsant, M.E.

Orange insects; *see* Ashmead, W.H.

Ordnance survey of the Peninsula of Sinai; *see* Crotch, G.R.

Otiorhynchiden; *see* Seidlitz, G.C.M. von.

Otros cien coleópteros de Menorca; *see* Cardona y Orfila, F.

Outlines of the natural history of Great Britain and Ireland; *see* Berkenhout, J.

Pandora et flora Rybyensis; *see* Linnaeus, C.

Periculum entomographicum; *see* Sahlberg, C.R.

Periculum entomologicum; *see* Thunberg, C.P.

Petit entomologiste collecteur au nord de Paris; *see* Maillard, J.

Petite faune analytique des coléoptères français; *see* Houlbert, C.V.

Petite faune entomologique du Canada; *see* Provancher, L.

Philosophia entomologica; *see* Fabricius, J.C.

Philosophie entomologique; *see* Saint-Amans, J.F.B. de

Philosophy of zoology; *see* Fleming, J.

Physis; *see* Thomson, J.L.

Plauische Grund bei Dresden; *see* Block, P.L.H. von.

Pleroma zu den Mysterien der europäischen Insektenwelt; *see* Gistel, J. von N.F.X.

Postscriptum ad clivinidarum monographiam; *see* Putzeys, J.A.AH.

Practische Anleitung zum Bestimmen der Käfer; *see* Schoch, G.

Praktische Insekten-Kunde; *see* Taschenberg, E.L.

Précis d’un nouvel arrangement de la famille des brachélytres; *see* Mannerheim, C.G. von.

Précis des caractères génériques des insectes; *see* Latreille, P.A.

Preis-Verzeichniss vorräthiger Insectendoubletten; *see* Klug, J.C.F.

Prémices entomologiques; *see* Putzeys, J.A.AH.

Preussische Käfer für die sammelnde Jugend; *see* Lentz, F.L.

Primae lineae cognitionis insectorum; *see* Schluga, J.B.

Principes fondamentaux de somiologie; *see* Rafinesque, C.S.

Prodromus faunae coleopterorum Lundensis; *see* Munck af Rosenschöld, E.

Prodromus insectologiae Siaellandicae; *see* Brünnich, M.T.

Promenade de Dieppe aux montagnes d’Écosse; *see* Nodier, C.

Preussische Käfer; *see* Fritzen, R.

Pselaphiden Siam’s; *see* Schaufuss, L.W.

Quaedam genera et species coleopterorum Archiducatus Austriae; *see* Redtenbacher, W.

Quer durch Klein-Asien in den Bulghar-Dagh; *see* Bodemeyer, A.R.E. von.

Quibusdam coleopteris agri Ticinensis; *see* Betta, V.

Quibusdam coleopteris Italiae; *see* Aragona, L.

Rassegna delle specie della famiglia dei Milabridi; *see* Baudi di Selve, F.

Rearrangement of the nomenclature and synonymy of those species of British Coleoptera; *see* Dawson, J.F.

Récapitulation des Lathridiidae de l’Amérique méridionale; *see* Belon, P.M.J.

Recherche de l’espèce typique de quelques anciens genres; *see* Gozis, M.G.P. des.

Recherches sur la faune de Madagascar; *see* Snellen van Vollenhoven, S.C.

Recueil d’observations de zoologie et d’anatomie comparée; *see* Latreille, P.A.

Règne animal disposé en tableaux méthodiques; *see* Comte, A.J.

Règne animal distribué d’après son organisation; *see* Audouin, J.-V.; Latreille, P.A.

Reise der Österreichischen Fregatte Novara; *see* Redtenbacher, L.

Reise durch das Altai-Gebirge; *see* Gebler, F.A. von.

Reise durch die La Plata-Staaten; *see* Burmeister, H.C.C.

Reise durch die Wueste Atacama; *see* Philippi, R.A.

Reise durch einen Theil Preussens; *see* Baczko, L.F.J.A. von.

Reisen durch Oeland und Gothland; *see* Linnaeus, C.

Reise durch Sachsen in Rüksicht; *see* Leske, N.G.

Reise durch Tyrol, Oberitalien und Piemont; *see* Waltl, J.

Reise durch verschiedene Provinzen des russischen Reichs; *see* Pallas, P.S.

Reise in den äussersten Norden und Osten Sibiriens; *see* Ménétriés, E.

Reise in Griechenland, Unteregypten; *see* Redtenbacher, L.

Reise nach Dalmatien; *see* Germar, E.F.

Reise nach Norwegen; *see* Fabricius, J.C.

Reise nach Palästina; *see* Hasselquist, F.

Reise nach Persien und dem Lande der Kurden; *see* Hampe, C.

Reise um die Erde durch Nord-Asien; *see* Klug, J.C.F.

Reise von Tripolis nach der Oase Kufra; *see* Karsch, F.A.F.

Reisen in Britisch-Guiana; *see* Erichson, W.F.

Reisen in der Regentschaft Algier; *see* Erichson, W.F.

Reisen in Ost-Afrika; *see* Gerstaecker, C.E.A.

Reisen in verschiedne Provinzen des Königreichs Neapel; *see* Salis-Marschlins, C.U. von.

Reisen und Forschungen im Amur-Lande; *see* Motschulsky, V. de

Report on the geology, mineralogy, botany, and zoology of Massachusetts; *see* Harris, T.W.

Report on the insects of Massachusetts; *see* Harris, T.W.

Report on the work of the Horn scientific expedition; *see* Blackburn, T.; Sloane, T.G.

Report upon geographical and geological explorations and surveys; *see* Ulke, H.

Report upon insects collected on the survey; *see* LeConte, J.L.

Report upon new species of Coleoptera; *see* LeConte, J.L.

Résumé d’entomologie, ou histoire naturelle des animaux articulés; *see* Milne-Edwards, H.

Révision de la famille des cicindélides; *see* Lacordaire, J.T.

Revision der Cicindeliden; *see* Horn, H.W.W.

Revision der europäischen Otiorhynchus-Arten; *see* Stierlin, W.G.

Revision der Tenebrioniden der alten Welt; *see* Kraatz, G.

Révision des curculionides byrsopsides; *see* Allard, E.

Revision of the coleopterous family Coccinellidae; *see* Crotch, G.R.

Revision of the Stenini of America; *see* Casey, T.L.

Revista crítica de las especies españolas del género Percus; *see* Pérez Arcas, L.

Rhipiphoridum coleopterorum familiae dispositio systematica; *see* Gerstaecker, C.E.A.

Ricerche entomologiche sopra i monti Partenii; *see* Costa, A.

Ritters Carl von Linné Königlich schwedischen Leibarztes; *see* Müller, P.L.S.

Saggio sulla storia naturale del Chili; *see* Molina, J.I.

Sammlung naturhistorischer und physikalischer Aufsäze; *see* Schrank, F.

Sammlung physikalischer Aufsätze; *see* Preyssler, J.D.E.

Sammlungen aus der Naturgeschichte; *see* Börner, I.K.H.

Sarawak; its inhabitants and productions; *see* White, A.

Schädlichen Forst- und Obstbaum-Insekten; *see* Henschel, G.

Schweizerischen Käfergattungen; *see* Labram, J.D.

Scientific results of the second Yarkand mission; *see* Baly, J.S.; Bates, F.; Bates, H.W.; Janson, O.E.; Sharp, D.

Silpharum monographiae particula prima; *see* Schmidt, R.C.E.

Singulorum generum curculionidum; *see* Labram, J.D.

Skandinaviens Coleoptera; *see* Thomson, C.G.

Skandinaviens insecter; *see* Thomson, C.G.

Sketch of the natural history of Yarmouth; *see* Paget, C.J.

Société entomologique de Belgique zu Brüssel; *see* Schaufuss, L.W.

Some account of the fauna and flora of Sinai; *see* Hart, H.C.

Sopra il Macronychus quadrituberculatus del Müller; *see* Contarini, N.B.

South Australia illustrated; *see* Angas, G.F.

Souvenirs d’un voyage dans l’Inde; *see* Guérin-Méneville, F.É.

Souvenirs d’un voyage en Allemagne; *see* Mulsant, M.E.

Species des cicindélides; *see* Dokhtouroff, V.S.

Species des coléoptères trimères sécuripalpes; *see* Mulsant, M.E.

Species et iconographie générique des animaux articulés; *see* Guérin-Méneville, F.É.

Species général des coléoptères; *see* Dejean, P.F.M.A.

Species général des hydrocanthares et gyriniens; *see* Aubé, C.N.

Species insectorvm; *see* Fabricius, J.C.

Spécimen Fabricia Entomologica; *see* Jekel, H.

Specimen faunae subterraneae; *see* Schiødte, J.M.C.

Specimen insectorum ulterioris Calabriae; *see* Petagna, V.

Spicilegia zoologica; *see* Pallas, P.S.

St. Helena; *see* Melliss, J.C.

Standard natural history; *see* Dimmock, G.

Statistics of the state of Georgia; *see* LeConte, J.E.

Statistique du département de l’Hérault; *see* Serres, P.T.M. de

Steiermark’s Coleoptern; *see* Grimmer, K.H.B.

Storia naturale degli animali invertebrati; *see* Baldassini, F.

Story of the rear column of the Emin Pasha relief expedition; *see* Bates, H.W.

Student’s list of British Coleoptera; *see* Pascoe, F.P.

Sugli insetti piu nocivi alla agricoltura; *see* Gené, C.G.

Supplément à la monographie des lathridiens de France; *see* Belon, P.M.J.

Supplement to the check list of the Coleoptera of America; *see* Austin, E.P.

Supplement to the fourth, fifth, and sixth editions of the Encyclopaedia Britannica; *see* Fleming, J.

Supplementa faunae insectorum Europae; *see* Richter, C.F.W.

Supplementary appendix to travels amongst the Great Andes; *see* Bates, H.W.; Gorham, H.S.; Jacoby, M.; Olliff, A.S.; Sharp, D.

Supplementum Coleoptera Europae dupletorum; *see* Villa, A.

Supplementum entomologiae systematicae; *see* Fabricius, J.C.

Symbola faunae insectorum Helvetiae; *see* Hagenbach, J.J.

Symbolae ad monographiam Scydmaenorum; *see* Schaum, H.R.

Symbolae ad monographiam Staphylinorum; *see* Nordmann, A. von.

Symbolae physicae; *see* Klug, J.C.F.

Synonymia insectorum; *see* Schönherr, C.J.

Synopsis coleopterorum Europae; *see* Crotch, G.R.

Synopsis der drei Naturreiche; *see* Leunis, J.

Synopsis des scolytides; *see* Chapuis, F.

Synopsis of the natural history of Great-Britain and Ireland; *see* Berkenhout, J.

System der Materia medica; *see* Pfaff, C.H.

Systema cerambycidarum; *see* Thomson, J.L.

Systema elevtheratorvm; *see* Fabricius, J.C.

Systema entomologiae; *see* Fabricius, J.C.

Systema insectorum; *see* Gistel, J. von N.F.X.

Systema naturae per regna tria naturae; *see* Linnaeus, C.

Systematic catalogue of British insects; *see* Stephens, J.F.

Systematische Nomenclatur; *see* Panzer, G.W.F.

Systematische Uebersicht der Käfer um München; *see* Gemminger, M.

Systematische Uebersicht des Thierreichs; *see* Rossmässler, E.A.

Systematisches und alphabetisches Verzeichnis [Cicindelidae]; *see* Heyne, A.

Systematisches Verzeichnis der Käfer Deutschlands; *see* Schilsky, F.J.

Systematisches Verzeichniss der in der Umgegend Erlangens beobachteten Thiere; *see* Küster, H.C.

Systematisches Verzeichniss der Käfer Deutschlands; *see* Bach, M.

Tableau élémentaire de l’histoire naturelle des animaux; *see* Cuvier, G.L.C.F.D.

Tableau encyclopédique et méthodique des trois règnes de la nature; *see* Latreille, P.A.

Tableau méthodique des insectes; *see* Latreille, P.A.

Tableaux synoptiques de zoognosie; *see* Fischer von Waldheim, J.G.

Tagebuch der Reise durch verschiedene Provinzen des russischen Reiches; *see* Lepekhin, I.I.

Taschenbuch für Insectenfreunde; *see* Schmiedlein, G.B.

Tentamen conspectus cantharidiarum; *see* Fischer, J.B.

Tentamen dispositionis generum et specierum coleoptrorum pseudotrimerorum; *see* Redtenbacher, L.

Tentamen monographiae Byrrhorum; *see* Steffahny, G.E.

Tentamen monographiae generis Meloes; *see* Meyer, F.A.A.

Tertiary Rhynchophorus Coleoptera; *see* Scudder, S.H.

The animal kingdom, arranged after its organization; *see* Carpenter, W.B.

The British Coleoptera delineated; *see* Shuckard, W.E.

The British miscellany; *see* Sowerby, J.

The cabinet cyclopaedia; *see* Swainson, W.

The cabinet of Oriental entomology; *see* Westwood, J.O.

The Cambridge natural history; *see* Sharp, D.

The civil and natural history of Jamaica; *see* Browne, P.

The class Insecta arranged by the Baron Cuvier; *see* Griffith, E.

The Coleoptera of Kansas and eastern New Mexico; *see* LeConte, J.L.

The Coleoptera of Suffolk; *see* Morley, C.

The Coleoptera of the British Islands; *see* Fowler, W.W.

The coleopterist’s manual; *see* Hope, F.W.

The complete writings of Thomas Say on the entomology of North America; *see* LeConte, J.L.

The Edinburgh Encyclopaedia; *see* Leach, W.E.

The English entomologist; *see* Martyn, T.

The entomological cabinet; *see* Samouelle, G.

The entomologist’s useful compendium; *see* Samouelle, G.

The fur seals and fur-seal islands; *see* Linell, M.L.

The genera insectorum of Linnaeus; *see* Barbut, J.

The genera of British Coleoptera; *see* Curtis, J.

The history of Glanville’s Wootton; *see* Dale, C.W.

The natural history of British insects; *see* Donovan, E.

The natural history of Tutbury; *see* Brown, E.

The naturalist in Vancouver Island and British Columbia; *see* Walker, F.

The naturalist’s library. Beetles; *see* Duncan, J.

The nomenclature of British insects; *see* Stephens, J.F.

The philosophy of zoology; *see* Fleming, J.

The standard natural history; *see* Dimmock, G.

The student’s list of British Coleoptera; *see* Pascoe, F.P.

The Yorkshire Lias; *see* Blake, J.F.

The zoological miscellany; *see* Leach, W.E.

The zoology of the voyage of H.M.S. Erebus & Terror; *see* White, A.

Thesaurus entomologicus oxoniensis; *see* Westwood, J.O.

Thiere Andalusiens; *see* Rosenhauer, W.G.

Thierische Leben und sein Formen; *see* Zenker, J.C.

Thierreich; *see* Kneifl, R.

Thierreich eingetheilt nach dem Bau der Thiere; *see* Schinz, H.R.

Thierwelt Deutschlands und der Schweiz; *see* Calwer, C.G.

Third supplement to the list of Coleoptera of America; *see* Henshaw, S.

Through New Guinea and the cannibal countries; *see* Jordan, H.-E.K.

Through unknown African countries; *see* Jordan, H.-E.K.; Shipp, J.W.

Topographiae natvralis Lipsiensis; *see* Schwägrichen, C.F.

Traité de paléontologie; *see* Pictet, F.J.

Travels in Hungary; *see* Townson, R.

Travels in New Zealand; *see* White, A.

Travels in the interior of southern Africa; *see* Burchell, W.J.

Travels through Sweden, Finland, and Lapland; *see* Acerbi, J.

Treatise on insects, general and systematic; *see* Wilson, J.

Tres reinos de la naturaleza; *see* Galdo López de Neira, M.M.J. de

Trichopterygia illustrata et descripta; *see* Matthews, A.

Trichterwickler; *see* Wasmann, E.

Typi buprestidarum; *see* Thomson, J.L.

Typi cetonidarum suivis de typi monommidarum et de typi nilionidarum; *see* Thomson, J.L.

Über die Cerambyciden des Harzes; *see* Leimbach, A.L.G.

Ueber den Charakter der Insecten-Fauna von Süd-Persien; *see* Kollar, V.

Ueber die fossilen Calosomen; *see* Heer, O.

Ueber eine neue Familie, Sippe und Gattung aus der Ordnung der Käfer; *see* Gistel, J. von N.F.X.

Uebersicht der cerambyciden Münchens; *see* Kriechbaumer, J.

Ujabb adatok Erdély bogárfaunájához; *see* Ormay, S.

Unterhaltungen aus der Naturgeschichte; *see* Wilhelm, G.T.

Unterhaltungen in der Naturgeschichte aller Arten Insekten; *see* Scheuereck, F.A.

Urwelt der Schweiz; *see* Heer, O.

Vacuna; *see* Gistel, J. von N.F.X.

Variétés; *see* Pic, M.

Venezia e le sue lagune; *see* Contarini, N.B.

Verbreitetsten Käfer Deutschlands; *see* Wünsche, O.

Vergleichende Uebersicht des Linneischen und einiger neuern zoologischen Systeme; *see* Gravenhorst, J.L.C.

Vergleichende Zoologie; *see* Gravenhorst, J.L.C.

Vermischte physicalisch-botanisch-oeconomische Abhandlungen; *see* Gleditsch, J.G.

Versuch einer Anleitung, zur Kenntniss und Geschichte der Thiere; *see* Batsch, A.J.G.K.

Versuch einer Einführung in das Studium der Koleoptern; *see* Imhoff, L.

Versuch einer Käfer-Fauna Krains; *see* Siegel, M.

Versuch einer medicinischen Topographie und Statistik der Haupt- und Residenz-Stadt Dresden; *see* Meyer, E.J.J.

Versuch einer medicinischen Topographie von Berlin; *see* Formey, J.L.

Versuch einer Naturgeschichte von Livland; *see* Fischer, J.B.

Versuch eines neuen Entwurfs der Thiergeschichte; *see* Eberhard, J.P.

Verzeichnis der ihm bekannten schweizerischen Insecten; *see* Fuessly, J.K.

Verzeichnis der in der Umgebung Arnstadts vorkommenden Käfer; *see* Jung, H.

Verzeichnis der in näheren Umgebung Sonderburgs; *see* Wüstnei, W.H.C.F.

Verzeichnis meiner Insecten-Sammlung; *see* Sturm, J.

Verzeichniss aller in Europa vorkommenden Geschlechter der Insecten; *see* Hofmann, C.E.

Verzeichniss böhmischer Insekten; *see* Preyssler, J.D.E.

Verzeichniss der bis jetzt, vornehmlich in der Umgegend von Riga; *see* Precht, K.H.

Verzeichniss der im Gebiete des Aller-Vereins; *see* Wahnschaffe, C.W.M.

Verzeichniss der Kaefer; *see* Roth von Schreckenstein, F.J.A.

Verzeichniss der Käfer Deutschlands; *see* Kraatz, G.

Verzeichniss der Käfer Preussens; *see* Illiger, J.C.W.

Verzeichniss der Lamellicornia melitophila; *see* Schaum, H.R.

Verzeichniss der Lucaniden; *see* Felsche, C.

Verzeichniss europäischer Käfer; *see* Anonymous

Verzeichniss meiner Insecten-Sammlung; *see* Sturm, J.

Verzeichniss und Beschreibung der Tyroler-Insecten; *see* Laicharting, J.N. von.

Verzeichniss verkäuflicher Doubletten der entomologischen Sammlung; *see* Klug, J.C.F.

Vivarium naturae or the naturalist’s miscellany; *see* Shaw, G.

Vollständige Geschichte des Borkenkäfers; *see* Dallinger, F.X.

Vollständige Naturgeschichte der schädlichen Forstinsekten; *see* Bechstein, J.M.

Voyage a Madagascar au couronnement de Radama II; *see* Coquerel, J.C.

Voyage à Méroé; *see* Latreille, P.A.

Voyage au Pôle Sud et dans l’Océanie; *see* Blanchard, C.É.

Voyage autour du monde [L’Uranie et la Physicienne]; *see* Quoy, J.R.C.

Voyage autour du monde [La Bonite]; *see* Desmarest, E.A.S.L.

Voyage autour du monde [La Coquille]; *see* Guérin-Méneville, F.É.

Voyage autour du monde [La Favorite]; *see* Guérin-Méneville, F.É.

Voyage aux Indes-Orientales; *see* Guérin-Méneville, F.É.

Voyage dans l’Afrique australe; *see* Delegorgue, L.-A.-J.

Voyage dans l’Amérique méridionale; *see* Blanchard, C.É.; Brullé, G.A.

Voyage dans l’Inde, par Victor Jacquemont; *see* Blanchard, C.É.

Voyage de découvertes de l’Astrolabe; *see* Boisduval, J.B.A.D. de.

Voyage en Abyssinie; *see* Guérin-Méneville, F.É.

Voyage en Abyssinie dans les provinces du Tigré; *see* Reiche, L.J.

Voyage en Barbarie; *see* Poiret, J.L.M.

Yorkshire Lias; *see* Blake, J.F.

Zoognosia tabulis synopticis illustrata; *see* Fischer von Waldheim, J.G.

Zoologia specialis quam expositis animalibus tum vivis; *see* Eichwald, K.E.I. von.

Zoologiae Danicae; *see* Müller, O.F.

Zoologiae fundamenta; *see* Brünnich, M.T.

Zoological classification; *see* Pascoe, F.P.

Zoological miscellany; *see* Leach, W.E.

Zoological results based on material from New Britain; *see* Sharp, D.

Zoologie analytique; *see* Duméril, A.-M.-C.

Zoologie classique; *see* Pouchet, F.-A.

Zoologie descriptive; *see* Rendu, L.-V.

Zoologische Briefe; *see* Vogt, C.C.

Zoologischer Atlas; Eschscholtz, J.F. von.

Zoology of the voyage of H.M.S. Erebus & Terror; *see* White, A.

Zoophylacii Gronoviani; *see* Gronov, L.T.

Zum Rudolph-See und Stephanie-See; *see* Ganglbauer, L.

Zur fauna der Nieder-Elbe; *see* Endrulat, B.F.J.

Zur Naturgeschichte der Gattung Calandra; *see* Burmeister, H.C.C.

Zweckmässige Fangen, Tödten und Aufbewahren der Käfer; *see* Hoffmeister, P.

## Footnotes

1 To avoid confusion, places of birth and death are listed with their current country.

2 The work of Horn et al. (1990a, b) has been particularly useful for the whereabouts of collections.

3 In old works, the term “publisher” could signify the author, printer or bookseller (*libraire* in French) who underwrote the costs of publication.

4 Dates found on wrappers for works issued in parts are listed but it should be noted that they could be in error because printers sometimes used old wrappers.

5 A few booklets were found online but not connected to a particular digital library and are identified as “Online” in this bibliography; the links are not provided because some are not available anymore.

6 The city seat is listed in brackets unless its name appears in the society name. Society names are those used at the time of the notices; some are know today under different names.

7 There are no dates, besides the year, associated with this journal. However, it was published in 12 issues which is assumed to correspond to each month of the year. Many *Hefte* seen of this journal include unnumbered pages (usually two or three) placed at the beginning and/or the end of the *Heft* where many notifications of books are listed. Apparently these pages are not present on all copies (Neal L. Evenhuis, personal communication).

8 This volume was listed in a catalogue of books presented at the Michaelmesse Book Fairs in Leipzig in 1790 (Anonymous 1790: 229) which was held on 3 October 1790 (Evenhuis 2014: 4).

9 Some sources date Acloque’s death in 1908, others in 1941.

10 Some sources give Altum’s birth on 31 January 1824, others on 31 December 1824.

11 The date of 8 July 1842 listed by Evenhuis (1997a: 179) and likely taken from Sherborn (1922b: 555) is a lapsus for 8 December 1842.

12 In its July 1800 issue, the magazine included the first part of a catalogue of the books at the Leipzig Easter Fair of 1800 (pp. 76–88) where Baczko’s book is listed; the fair was held on 4 May 1800 (Evenhuis 2014a: 4).

13 The collection was studied by Fauvel (1886) who noted that it was in bad condition.

14 This volume was listed in a catalogue of books presented at the Ostermesse Book Fairs in Leipzig in 1804 (Anonymous 1804: 19) which was held on 22 April 1804 (Evenhuis 2014: 4).

15 Plates 15–18 belong to the second Jahrgang.

16 According to Sherborn and Griffin (1934: 132), plates 4 and 19 were also issued in livraisons 41 and 71 respectively (may be cancels) and there were no record for plates 15 and 25.

17 The atlas volume containing the insects reads besides the title “Tome neuvième. Atlas zoologique (mammifères, oiseaux, reptiles, poissons, mollusques, polypiers, foraminifères, crustacés et insectes.)” and is dated 1847.

18 Some sources (e.g., Darby 2016) mentioned that Blandford was born on 28 December 1874. This is unlikely considering that his first paper, based on collecting he made in August 1881, was published in 1883.

19 In his review of Clairville’s second volume of Entomologie helvétique, published in the April 1810 issue of the Magasin Encyclopédique, Latreille mentioned that Bonelli had just published his first memoir on the beetles of the family “des Carabes.”

20 These two pages are the “Rapport fait a l’Académie des sciences par MM. Duméril, et Geoffroy-Saint-Hilaire, sur les tableaux méthodiques du Règne Animal de M. Achille Comte.” The second page is dated 18 June 1832.

21 The second livraison was noted in *Le Bibliologue* for 31 January 1833. However, considering that the livraison was noticed much later in the *Bibliographie de la France*, the notification may simply have been an advertisement from the publishing house.

22 The book was received by the *Société Entomologique de France* during the year 1866 and the first three months of 1867. Therefore it must have been published between 1 January and 31 March 1867 if the date on the title page is correct.

23 The plate includes also the illustration of another species, unnamed on the plate but named *Stenocorus obscurus* in the text.

24 The *Bibliographie de la France* recorded Duméril’s volume without the plates on 17 May 1823 and with the plates on 18 October 1823.

25 The entry in *Verzeichniss der Bücher, Landkarten* gives the year of publication as “Berlin 1829 (1830)” which probably indicates that *Hefte* 2 and 3 are dated 1829 but were published in 1830.

26
Evenhuis (1997a: 247) noted that the *Allgemeines Verzeichniss neuer Bücher* recorded volumes 1 and 2 on March 1781. In fact, only volume 1 was noted; the second one was noticed the following year. Considering the recording dates found in this research, I doubt that Fabricius’ tomes 1 and 2 of this work were issued at the same date in 1781.

27 The date of 20 May 1798, taken from the *Göttingische gelehrte Anzeigen*, provided by Evenhuis (1997a: 248) could not be confirmed.

28 Linnaeus used the name *Buceros* in 1758 for a genus of birds.

29 The date of April–May 1778 provided by Stafleu and Cowan (1976: 838) seems a pure guess based on the date of the *Vorbericht*.

30
Hagen’s (1862: 247) statement that Francillon was a physician in London is inexact (Cowan 1986: 139).

31 According to Jäggli (1958: 199), some copies of this work have additional plates.

32 In most bibliographies seen during this study, including Hagen (1862: 5) and Evenhuis (1997a: 53), the second fascicle is credited to August Ahrens. However the title page of the fascicle, which is often missing, reads “ *Fauna Insectorum Europae cura E.F. Germar. Fasciculus secundus.*”

33 I have seen advertisements (April 1814 issue of *Leip Lit Zeit* and *Allg Lit Zeit*) mentioning that fascicles II and III of Ahrens’ work will be published for the 1814 Easter Book Fairs in Leipzig, which was held on 1 May 1814 (Evenhuis 2014a: 4). However, the third fascicle was issued only in 1817 and I have not been able to confirm that the second fascicle was indeed available at the Book Fair.

34 This fascicle has a second title page, dated 1837, which reads “Insectorum protogaeae specimen sistens insecta carbonum fossilium.”

35 Guérin chnaged his name to Guérin-Méneville in 1836 (see Low et al. 2013: 101–102).

36
Guérin-Méneville (1838: 271) listed plate 17 twice by error, one should had been plate 19. Musgrave (1958: 75) corrected the error.

37 The entry in the *Bibliographie de la France* listed livraison 7 as livraison 6 by error. The sixth livraison was recorded on 1 December 1832.

38 The date of 13 June 1835 for the second livraison, taken from the *Annales de la Société Entomologique de France*, listed by Evenhuis (1997a: 335) is incorrect.

39 The *Société de Géographie* received the 28 ^th^ and last livraison of the work on 5 April 1839 which, according to Sherborn and Woodward (1906: 336), probably contains all the text of part 2 of the second volume.

40 This date is from a separate page inserted in the copy on GAL, entitled “Avis très-essentiel” and dated 1 February 1843, where it is mentioned that the first livraison is on sale.

41
Sherborn (1922a: lxiii) noted that parts 1–3 were issued “Feb. & end of Mar. 1843”

42 The date of 12 August 1844, taken from the *Revue Zoologique par la Société Cuvierienne*, mentioned by Evenhuis (1997a: 333) is incorrect; the permanent secretary Flourens presented a copy of the text at the Society on 26 August 1844.

43 This volume was listed in *The Christian Remembrancer* (vol. 9, p. 404) as having been published at the Leipzig Easter Fair of 1827, which was held on 6 May 1827 (Evenhuis 2014: 4).

44 The plates bound in at the end of the volume have *Tafel* I and II printed on one folded leaf and *Tafel* III and IV printed on the second folded leaf.

45 The date of September–December 1841, taken from *The American Journal of Science and Arts*, reported by Evenhuis (1997a: 345) could not be confirmed. The journal recorded Harris’ work in its January–March 1842 issue.

46 The date of 3 August 1799, taken from the *Göttingische gelehrte Anzeigen*, mentioned by Evenhuis (1997a: 359) for the publication of volume 8 pertains in fact to volume 7.

47 Based on the entries in *Allgemeine Bibliographie für Deutschland* and *Bibliographische Blätter der Press-Zeitung* for the year 1840, *Heft* 178 was issued before *Hefte* 176 and 177.

48 The announcement is appended to volume 4 of *Zeitschrift für die Entomologie* in GB.

49 The Entomological Society of London reported in their *Journal of Proceeding* (p. lxxxix) the acquisition of the three volumes of *The coleopterist’s manual* by Hope in the “additions to the library, from December 31, 1836, to September 30, 1840.” This may be an error for the first two volumes only considering the date of publication reported by Low (1853) for the third volume and the notice of “new publications, from April 6, to May 6, 1841” registered in the *Bent’s Literary Advertiser*.

50 The bottom of page 17 is dated March 1898 which is probably an error for April 1898 considering the date at the bottom of page 3.

51
Lyal (2011: 87) listed plates 1–33 with volume 6, part 1, Supplement (his table 30) while the text pages are listed in table 29.

52 These notes are at the British Museum (Natural History). Sherborn seems to have seen wrappers for most if not all the livraisons. Unfortunately, the date on some of the wrappers may have been incorrect. For exemple, at the request of Kraatz, Deyrolle who was the publisher of Jacquelin du Val’s *Manuel entomologique* assured him that the first livraison of the Staphylinidae (i.e., livraison 45 of the entire work) was issued in May 1857 (Kraatz 1859: vii).

53 The wrapper of livraison 48 (pp. 33–40) is dated 1855 (Sherborn’s notes; Tottenham 1949: 360) but this is an error by the printer for 1857.

54 It appears that the family treatments in the catalogue were issued at the same time or shortly after the publication of the family in the book itself. Therefore the dates attached to the new species here are those found during this study for their respective families in the book.

55 Jacquelin du Val mentioned in his catalogue on page 184 that the catalogue section of the Tenebrionidae was published shortly after the second edition of the *Catalogus Coleopterorum Europae* of Schaum issued early 1862. Therefore publication of the tenebrionid part (pp. 169–184) of Jacquelin du Val’s catalogue was published in 1862 and the date of 1862 should be attributed to *Tribolium confusum*.

56 The Academy of Sciences of Philadelphia received livraisons 78–80 on 22 December 1857, not on 17 June 1857 as noted by Evenhuis (1997b: 679).

57 Several pages in this work have a date at the bottom but these seem to refer to the date when the text was written.

58 The copy I have seen had only six plates (1–5 and 8).

59 The footnote on page 83 written by Klug suggests that Wilhelm Ferdinand Erichson is the author of this section.

60 Two notifications seen, one on 7 September 1842 (*Lit Zeit*) and the other for July–December 1842 (*Verz Bücher Landkarten*), mentioning the fourth *Band* (*Hefte* 61–80) probably refer to the publication of the title page rather than the whole volume.

61 The first 12 *Hefte* were noticed in Schaum’s (1849: 128) review of the entomological literature for 1848.

62 It is possible that the printer used the same wrapper as for the first livraison since the numbering of the livraison on the wrapper is handwritten. Therefore the date of 1834 for the second livraison is questionable.

63 Their source for the dates is derived from the “Inter Documentation Company micro-edition.”

64 In fact, it seems apparent that Engelmann confused this work with the *Histoire naturelle et iconographie des insectes coléoptères* of Laporte and Gory published in livraisons from 1835 to 1841.

65
Evenhuis (1997b: 437) reported that Latreille presented his book at the *Académie des Sciences* on 13 January 1797. In fact, it was presented by Cuvier on 11 pluviôse an 5 (see Anonymous 1910a: 165) which corresponds to 30 January 1797.

66 The date of January 1805, taken from the *Magazin Encyclopédique*, listed by Evenhuis (1997a: 381) could not be confirmed. In fact in their issue of 5 Germinal an 13 (= 26 March 1805), the *Journal Typographique et Bibliographique* reported (p. 270) that the first livraison of the *Recueil d’observations de zoologie et d’anatomie comparée* will be issued in May. The livraison was noticed only in June 1807 by the *Magazin Encyclopédique*.

67 The date of 22 November 1811, taken from the *Bibliographie de la France*, listed by Evenhuis (1997a: 381) is incorrect; the *Bibliographie* did not mention a second edition but simply that the first six livraisons were issued. The second edition was presented by Humboldt to the *Académie des Sciences* on 3 August 1812.

68 The date is incorrect because the printer used wrappers of the second livraison. There are several references in the text to the first volume of Dejean’s *Species général des Coléoptères* published in September 1825.

69 The number of plates indicated is different for some volumes.

70 Plates 8 and 9 were first issued in the 1831 edition published in New York.

71 The copy on ARC has a subsequent title page, dated 1830, which reads “ *The Edinburgh Encyclopaedia… In eightheen volumes. Volume IX.*” The original title page of the first part of volume 8 was not seen during this study.

72 The *Bibliographie de la France* recorded livraison 1 (2 sheets, no plates) on 20 March 1830, livraison 2 (2 sheets and 5 plates) on 2 October 1830, livraison 3 (one sheet and 5 plates) on 29 January 1831, livraisons 6–8 (3 sheets and 15 plates) on 9 July 1831 and livraisons 13–16 (31/4 sheets and 20 plates) on 19 May 1832. No entries were found for livraisons 4–5 and 9–12.

73 Regarding the proper name of this author, see Duris (1993: 13–16).

74 The date of May 1821, taken from the *Edinburgh Magazine*, listed by Evenhuis (1997b: 507) is probably too early. The book was listed under “Works preparing for publication.” A similar announcement for the month of May was issued in *Blackwood’s Edinburgh Magazine* and *The Gentleman’s Magazine*.

75 The date listed “Nov. 1843” is obviously a lapsus for Nov. 1863.

76 The date of January–June 1851 taken from the *Bibliotheca Historico-Naturalis, Physico-Chemica et Mathematica* reported by Evenhuis (1997b: 543) is a lapsus for January–June 1852.

77 Motschulsky presented another copy of his “Sixième année” at the meeting of 24 March 1858 of the Société Impériale des Naturalistes de Moscou as noted by Griffin (1936: 257).

78 The date of 13 June 1860 cited in Bouchard et al. (2011: 771) is incorrect and refers to the “Catalogue des insectes rapportés des environs du fleuve Amour…” published in the Bulletin de la Société Impériale des Naturalistes de Moscou 32: 487–507.

79 The date listed on the title page is “1863–Février 1863” but this is an error, probably for “1863–Février 1864” since one of the article of the volume (pp. 405–599) was presented at the *Société Linnéenne de Lyon* on 11 January 1864.

80 The date listed is “Octobre 1878” but this may be a typographic error for “Octobre 1879” since the last article of the volume (pp. 323–325) was presented at the *Société Linnéenne de Lyon* on 11 November 1878.

81
*Le Naturaliste* recorded on 1 November 1882 (p. 168), in their section “Livres nouveaux,” the “Catalogue des insectes de Portugal” de Manoel Paulino de Oliveira without further comments. Possibly this notification refers to the first part (pp. 1–112) of the publication.

82 The first two livraisons of plates, containing 24 plates, was noticed on February 1789 *(J Phys*).

83 The date of 23 October 1811, taken from the *Mercure de France*, provided by Evenhuis (1997a: 227–228) could not be corroborated.

84 The date of 14 December 1811 listed by Evenhuis (1997b: 588) is a lapsus for 14 December 1812.

85 In it January 1818 issue, the *Journal des Savans* (p. 63) mentioned that livraisons 11 and 12 will be published next March.

86 The eighth volume of the translation, entitled “ *Voyages du Professeur Pallas, dans plusieurs provinces de l’empire de Russie et dans l’Asie septentrionale; traduits de l’allemand par le C. Gauthier de La Peyronie. Nouvelle édition. Appendix, contenant les descriptions des animaux et des végétaux observés dans les Voyages du Professeur Pallas, et cités ou mentionnés dans les volumes précédens; avec des notes et observations par le C. Lamarck. Tome huitième*” contains remarks by Lamarck, some of which are taxonomically important. The eight volumes were issued simultaneously in An II [= 22 September 1793–21 September 1794]. They were noticed on January 1795 (*Esprit J*) suggesting they were issued in 1794.

87 The dates of 1786 and 1787 for the second and third *Hefte* respectively could not be corroborated in this study.

88 The title page is dated “1864–Février 1864” but this is a lapsus probably for “1864–Février 1865” since one paper (pp. 258–288) included in the volume was presented to the society on 14 November 1864.

89 Considering the review date in the *Revue Zoologique*, the date on the title page is likely incorrect.

90 The notice in the *Feuilleton du Journal de la Librairie* mentioned the sale of two livraisons, but this is likely a lapsus for one livraison.

91
Hagen (1863: 68) dated Reiche’s section 1849 but since the year 1850 is listed on page 256, Sherborn and Woodward (1901b: 161) assumed it was reasonable to accept 1850 as the publication date. Schaum (1851: 153) mentioned that Reiche’s section on entomology was certainly not published before 1850, and may even have been issued later. Reiche’s section was cited by in Mulsant’s *Species des coléoptères trimères sécuripalpes* [q.v.] which was recorded on 19 December 1850 establishing that the entomology section of Reiche was published at that time. The three volumes and the atlas of Ferret et Galinier’s voyage were received by the *Société de Géographie* in their sessions of 7 and 21 April 1851.

92 Authorship of this volume is disputed. Some authors (e.g., Blackwelder 1952: 472, Zanetti 1987: 455 and Newton and Thayer 1992: 77) cited Rey alone as the author, others (e.g., Locard 1882: 304; Herman 2001: 3974; Bouchard et al. 2011: 775) credited the work to Mulsant and Rey. The journal issue has no author listed but the separate has Rey alone. The new species in the text are credited to both Mulsant and Rey. According to Belon (1895: 261), the writing of all joint publications involving Mulsant and Rey was done by Rey alone [“il est juste d’ajouter que le mérite de leur rédaction appartient exclusivement à Rey”].

93 This volume was listed in a catalogue of books presented at the Michaelmesse Book Fairs in Leipzig in 1764 (Anonymous 1764: 516) which was held on 1 October 1764 (Evenhuis 2014: 4).

94 This volume was listed in a catalogue of books presented at the Ostermesse Book Fairs in Leipzig in 1766 (Anonymous 1766: 700) which was held on 20 April 1766 (Evenhuis 2014: 4).

95 This volume was listed in a catalogue of books presented at the Ostermesse Book Fairs in Leipzig in 1777 (Anonymous 1777b: 65) which was held on 20 April 1777 (Evenhuis 2014: 4).

96 The date of 8 September 1759, from the *Göttingische Gelehrte Anzeigen*, listed by Evenhuis (1997b: 710) could not be corroborated.

97 The date of 22 August 1771 provided by Evenhuis (1997b: 720) from Beckmann’s *Physikalisch-ökonomische Bibliothek* could not be corroborated in this study.

98 The date listed in the *Vorbemerkung* for this *Heft* is incorrect. The wrapper is dated 1895 and the *Heft* has been recorded on May 1895 (*Nat Nov*), 8 June 1895 (*Academy*), July 1895 (*Ent Nachr* 21: 222), 25 August 1895 (*Wien Ent Zeit* 14: 260) and November 1895 (*Polybiblion*).

99 The *Société Entomologique de France* received pages 113–144 on 14 May 1890.

100
*Naturae Novitates* recorded pages 353–384 on May 1894 and pages 385–426 on August 1894.

101 Hagen stated under this work “Die Ankündigung vom 22. Septbr. 1796 kl. 8. pg. 2 mit Panzer. Fn. Germ. Heft 41 ausgegeben.”

102 Pages 45–48 were skipped.

103 Some of the dates of publication recorded by Evenhuis (1997b: 772), taken from Hayek (1989: 90), are in fact dates listed in the work itself (“latest date” in Hayek’s table 1) and are not actual dates of publication.

104 The work was apparently published in livraisons starting in 1766. “La premiere partie” [the first part] was noticed, without further qualification, in April 1767 by the *Gazette Littéraire de l’Europe*. According to Hagen (1857b: 406), based on Beckmann (1776: 105), signatures A–E + plates 1–24 of volume 1 and signature A + plates 1–8 of volume 2 were published by 1776 and, based on Cobres (1782: 390), signatures A–L + plates 1–48 of volume 1 and signatures A–C + plates 1–24 of volume 2 were issued by 1781.

105 The statement of Gaedike and Smetana (1984: 280) that Friedrich Weber was born in 1852 and not 1781 as mentioned by Horn and Schenkling (1929: 1300) is possibly due to a confusion with the German physician Friedrich August Weber born on 24 January 1853.

106 The work was noticed in the January–June 1805 issue of *Verzeichniss neuer Bücher* (later, *Verzeichniss der Bücher, Landkarten &c*) as “Weigel, J.A.V., Beschreibung von Schlesien, 10r Bd. Fauna Silesiaca, gr. 8. Berlin, Himburg. I thl. 8 gr.” (p. 162). Considering the date of the *Vorrede*, it is possible that this notification refers to another printing without the *Vorrede*. Unfortunately, the July–December 1805 issue of the journal was not available.

107
Stafleu and Cowan (1979: 410) believed the book was issued late 1781 or early 1782.

108 At the meeting of the Entomological Society of London on 7 July 1873 it was announced that “Prof. Westwood sent copies of two parts of his forthcoming ‘Thesaurus Entomologicus Oxoniensis.’ These parts were probably the first two. Westwood presented a copy of the second part to the Society on 1 February 1875, after he presented parts 3 and 4 on 7 December 1874.

109 The letter is bound with the *Insecta Maderensia* volume in the library.

110 Some sources date Zinke’s death in 1813, others in 1849.

111 This volume was listed in a catalogue of books presented at the Ostermesse Book Fairs in Leipzig in 1798 (Anonymous 1799b: 192) which was held on 29 April 1798 (Evenhuis 2014: 4).

112 The date of 5 March 1818, taken from the *Göttingische gelehrte Anzeigen*, mentioned by Evenhuis (1997a: 234) refers to a sample issue (*Probeheft*), not to the first volume.

113 The date of 28 September 1822, taken from the “ *Journal de Paris*” (n.v.), listed by Evenhuis (1997a: 111) for the publication of the second volume is doubtful considering the date on the title page.

114 The date of 15 September 1823, taken from the “ *Moniteur Universel*” (n.v.), listed by Evenhuis (1997a: 111) for the publication of the fourth volume is doubtful considering the date on the title page.

115 The date of 13 August 1824, taken from the “ *Moniteur Universel*” (n.v.), listed by Evenhuis (1997a: 111) for the publication of the sixth volume is doubtful considering the date on the title page.

116 The date of 26 April 1826 from “ *Le Constitutionnel, Journal du Commerce, Politique et Littéraire*” mentioned by Evenhuis (1994: 483; 1997a : 111) could not be confirmed in this study.

117 Only a few copies of volume 6 were issued (Woodward 1903: 458).

118 The date of 16 December 1817 reported by Sayre (1959: 71) and other bibliographers is a lapsus for 6 December 1817.

119 The date of 19 January 1823 listed by Evenhuis (1997a: 182) from the “ *Moniteur Universel*” is incorrect. The journal simply noted on this date that three volumes of the *Dictionnaire des sciences naturelles* were recently published (probably referring to tomes 22–25). Tome 26 was noticed in the journal on 16 June 1823.

120 The date of 1 August 1823 listed by Evenhuis (1997a: 182) from the “ *Gazette de France*” (*n.v.*) is suspect considering Cassini’s date.

121 The date of April 1828 mentioned by Evenhuis (1997a: 183) is incorrect. The date of receipt from the *Bibliographie de la France* confirmed the date of 1829 on the title page. Volume 54 was issued after volumes 55–58 (Cassini 1834: 160).

122 The collation is difficult to establish because the number of pages per livraison varied considerably, being generally of 32, 48, 56 or 64 pages (Iredale 1937: 34; Evenhuis 1990: 220).

123 The date of 4 March 1803 for tomes 7–9 provided without qualification by Evenhuis (1997a: 197) could not be corroborated in this study.

130 Titles starting with an article are also listed without it.

## Appendixes

### Appendix 1^131^. List of Coleoptera illustrated on the plates of ‘*Le règne animal distribué d’après son organisation... Atlas [I*]’ (Audouin, 1836–1849)

Species placed in square brackets indicate that the name of these species is not on the plate itself but in the explanatory caption only; usually in these cases only a structure of the species is illustrated.

**Plate 16** [DP: August 1839]: 1. *Manticora tuberculata*; 2. *Megacephala senegalensis*; 3. *Oxycheila tristis*; 4. *Cicindela campestris*. **Plate 17** [DP: March 1839]: 1. *Euprosopus quadrinotatus*; 2. [*Dromica coarctata*]; 3. *Ctenostoma trinotatum*; 4. *Therates labiata*; 5. *Collyris longicollis*; 6. *Tricondyla aptera*. **Plate 18** [DP: May 1845]: 1. *Anthia decemguttata* Fabr.; 2. *Graphipterus luctuosus* Dej.; 3. [*Aptinus displosor* L.]; 4. *Brachinus crepitans* Fabr.; 5. *Casnonia rugicollis* Dej.; 6. [*Leptotrachelus dorsalis* Fabr.]; 7. *Odacantha melanura* Fabr.; 8. *Zuphium olens* Fabr.; 9. *Polistichus fasciolatus* Fabr.; 10. *Helluo costatus* Bonelli]. **Plate 19** [DP: May 1845]: 1. *Drypta emarginata* Fabr.; 2. *Trichognathus marginipennis* Latr.; 3. *Galerita cyanipennis* Dej.; 4. [*Cordistes acuminatus* Oliv.]; 5. [*Ctenodactylus chevrolatii* Dej.]; 6. *Agra cayennensis* Oliv.; 7. *Cymindis humeralis* Fabr.; 8. [*Calleida fasciata* Dej.]; 9. [*Demetrias elongatulus* Dej.]; 10. *Dromius agilis* Fabr.; 11. *Lebia cyanocephala* Lin.; 11^c^. *Lebia crux-minor* Fabr.; 12. [*Plochionus bonfilsii* Dej.]. **Plate 20** [DP: August 1841]: 1. *Orthogonius acrogonus* Wiedem.; 2. *Coptodera emarginata* Dej.; 3. *Enceladus gigas* Latr.; 4. *Siagona rufipes* Latr.; 5. [*Carenum cyaneum* Fab.]; 6. *Pasimachus depressus* Fab.; 7. [*Acanthoscelis ruficornis* Fab.]; 8. *Scarites pyracmon* Bon.; 9. *Oxystomus cylindricus* Dej. **Plate 21** [DP: February 1842]: 1. *Clivina fossor* Lin.; 2. [*Dyschirius gibbus* Fab.]; 3. *Morio monilicornis* Lat.; 4. [*Ozaena laevigata* Dej.]; 5. *Ditomus calydonius* Fab.; 6. [*Apotomus rufus* Oliv.]; 7. *Acinopus megacephalus* Fab.; 8. [*Daptus vittatus* Gebl.]; 9. *Harpalus aeneus* Lin.; 10. *Ophonus obscurus* Fab.; 11. [*Stenolophus vaporariorum* Lin.]; 12. *Acupalpus meridianus* Lin. **Plate 22** [DP: February 1842]: 1. *Zabrus gibbus* Fab.; 2. *Pogonus pallidipennis* Dej.; 3. *Tetragonoderus quadrum* Fab.; 4. *Amara eurynota* Illig.; 5. *Feronia cuprea* Lin.; 6. *Myas chalybaeus* Palliardi; 7. [*Cephalotes vulgaris* Bon.]; 8. *Stomis pumicatus* Panz. **Plate 22bis** [DP: November 1841]: 1. *Catascopus hardwickii* Kirby; 2. *Mormolyce phyllodes* Hagenb.; 3. *Sphodrus leucophthalmus* Lin.; 4. - [*Sphodrus*] *terricola*^132^ Payk.; 5. *Calathus melanocephalus* Fab.; 6. [*Taphria vivalis* Illig.]. **Plate 23** [DP: November 1841]: 1. [*Dolichus flavicornis* Fabr.]; 2. [*Platynus complanatus* Bon.]; 3. *Agonum marginatum* Fab.; 4. *Anchomenus prasinus* Fab.; 5. [*Callistus lunatus* Fab.]; 6. *Oodes helopioides* Fab.; 7. *Chlaenius velutinus* Duftsch.; 8. [*Epomis circumscriptus* Duftsch.]; 9. [*Rembus politus* Fab.]; 10. *Dicaelus violaceus* Bon.; 11. *Licinus cassidaeus* Fab.; 12. *Badister bipustulatus* Fab. **Plate 24** [DP: November 1841]: 1. *Pelecium cyanipes* Kirby; 2. *Panagaeus crux-major* Fab.; 3. *Loricera pilicornis* Fab.; 4. *Patrobus rufipes* Fab.; 5. [*Pamborus alternans* Latr.]; 6. *Cychrus rostratus* Fab.; 7. [*Scaphinotus elevatus* Fabr.]; 8. [*Sphaeroderus lecontei* Dej.]; 9 [as 8]. *Tefflus megerlei* Fab.; 10. [*Procerus scabrosus* Fabr.]; 11. [*Procrustes coriaceus* Lin.]; 12. *Carabus auratus* Lin. **Plate 25** [DP: November 1841]: 1. *Calosoma sycophanta* Lin.; 2. *Pogonophorus spinibarbis* Fab.; 3. *Nebria arenaria* Lat.; 4. *Omophron limbatum* Fab.; 5. [*Pelophila borealis* Fab.]; 6. [*Blethisa multipunctata* Fab.]; 7. *Elaphrus riparius* Lin.; 8. *Notiophilus aquaticus* Lin.; 9. *Bembidion flavipes* Lin.; 10. [*Trechus rubens* Clairv.]. **Plate 26** [DP: February 1842]: 1. *Dytiscus latissimus* Lin.; 2. *Colymbetes striatus* Lin.; 3. *Hygrobia hermanni* Fab.; 4. *Hydroporus depressus* Fab.; 5. *Noterus crassicornis* Fab.; 6. *Haliplus impressus* Fab.; 7. *Gyrinus natator* Lin. **Plate 27** [DP: February 1842]: 1. *Oxyporus rufus* Lin.; 2. *Astrapaeus ulmi* Oliv.; 3. *Staphylinus hirtus* Lin.; 4. [*Staphylinus olens*: larve, nymphe (a)]; 5. [*Xantholinus fulgidus* Gravenh.]; 6. *Lathrobium fulvipenne* Fab.; 7. *Paederus riparius* Panz.; 8. [*Stilicus orbiculatus* Latr.]; 9. [*Evaesthetus scaber* Gravenh.]; 10. *Stenus biguttatus* Lin.; 11. *Osorius incisicruris* Latr. **Plate 28** [DP: 8 July 1842]: 1. *Oxytelus tricornis* Grav.; 2. *Zirophorus scoriaceus* Germ.; 3. [*Prognatha quadricornis* Kirby]; 4. [*Coprophilus rugosus* Gravenh.]; 5. *Omalium rivulare* Grav.; 6. *Lesteva dichroa* Grav.; 7. [*Micropeplus sulcatus* Gyllenhall]; 8. *Proteinus brachypterus* Fab.; 9. *Aleochara fuscipes* Grav.; 10. *Lomechusa paradoxa* Grav.; 11. *Tachinus subterraneus* Fab.; 12. [*Tachyporus marginatus* Fabric.]. **Plate 29** [DP: 8 July 1842]: 1. *Buprestis gigas* Lin.; 2. *Trachys minuta* Lin.; 3. *Aphanisticus emarginatus* Fab.; 4. *Melasis flabellicornis* Fab.; 5. *Galba fasciata* Blanch.; 6. *Eucnemis capucinus* Ahrens. **Plate 30** [DP: 8 July 1842]: 1. [*Adelocera caliginosa* Guer.]; 2. *Lissomus equestris* Fab.; 3. [*Lissomus punctulatus*^133^ Dalm.]; 4. *Chelonarium ornatum* Klug.; 5. *Throscus dermestoides* Lin.; 6. *Cerophytum elateroides* Latr.; 7. [*Cryptostoma rufithorax* Perty]; 8. [*Nematodes filum* Mannerheim]; 9. *Hemirhipus lineatus* Fab.; 10. *Ctenicera haematodes* Fab.; 11. *Elater germanus* Lin.; 12. [*Campylus linearis* Linné]; 13. [*Phyllocerus flavipennis* Lepeletier Saint-Fargeau et Serville]. **Plate 31** [DP: 8 July 1842]: 1. [*Physodactylus henningii* Fisch.]; 2. *Cebrio gigas* Fab. mâle; 3. *Cebrio gigas* Fab. femelle; 4. [*Anelastes druryi*]; 5. *Callirhipis orientalis* Lap.; 6. *Rhipicera marginata* Latr.; 7. [*Ptilodactyla humeralis* Blanchard]; 8. *Dascillus cervinus* Fab.; 9. [*Elodes pallidus* Fabr.]; 10. *Scyrtes hemisphaericus* Fab.; 11. *Nycteus haemorrhoidalis* Germ. 12. [*Eubria palustris* Germar]. **Plate 32** [DP: December 1842]: 1. *Lycus latissimus* Fab.; 2. *Dictyoptera sanguinea* Lin.; 3. [*Omalisus suturalis* Fabr.]; 4. [*Amydetes plumicornis* Latr.]; 5. *Lampyris noctiluca* Lin. [mâle]; 6. [*Lampyris noctiluca* Linn. femelle]; 7. *Drilus flavescens* Fab. [mâle]; 8. [*Drilus flavescens* Fab. femelle]; 9. [*Drilus flavescens* Fab. larve]; 10. *Telephorus fuscus* Lin.; 11. [*Telephorus fuscus* Lin. larve]; 12. [*Telephorus fuscus* Lin. nymphe]; 13. [*Silis rubricollis* Latr.]; 14. [*Malthinus biguttatus* Fabr.]; 15. *Malachius aeneus* Lin.; 16. *Dasytes caeruleus* Fab.; 17. [*Zygia oblonga* Fab.]; 18. *Melyris viridis* Fab. **Plate 33** [DP: December 1842]: 1. [*Cylidrus cyaneus* Fabr.]; 2. *Tillus elongatus* Lin.; 3. [*Priocera hopei*]; 4. [*Axina analis* Kirby]; 5. [*Eurypus rubens* Kirb.]; 6. *Thanasimus formicarius* Lin.; 7. *Opilo mollis* Lin.; 8. *Clerus alvearius* Fab.; 9. *Necrobia violacea* Lin.; 10. [*Enoplium serraticorne* Oliv.]; 11. *Ptinus fur* Lin.; 12. *Gibbium scotias* Fab. **Plate 34** [DP: December 1842]: 1. *Ptilinus pectinicornis* Fab.; 2. [*Xyletinus pectinatus* Fabr.]; 3. [*Dorcatoma dresdense* Herbst]; 4. *Anobium tessellatum* Fab.; 5. *Atractocerus brevicornis* Lin.; 6. *Hylecaetus dermestoides* Lin.; 7. *Lymexylon navale* Fab.; 8. *Cupes capitata* Lin.; 9. [*Rhysodes exaratus* Dalm.]; 10. *Mastigus palpalis* Latr.; 11. [*Scydmaenus helwigii* Latr.]. **Plate 35** [DP: December 1842]: 1. *Hololepta plana* Fab.; 2. *Hister cadaverinus* Payk.; 3. *Necrophorus vespillo* Lin.; 4. *Necrodes littoralis* Fab.; 5. *Silpha thoracica* Lin.; 6. [*Silpha laevigata* Fab.]; 7. [*Agyrtes castaneus* Gyllenh.]; 8. *Scaphidium quadrimaculatum* Lin.; 9. *Choleva sericea* Spence; 10. [Choleva (Myloechus) oblonga Latr.]. **Plate 36** [DP: 11 May 1843]: 1. [*Colobicus hirtus* Fabr.]; 2. *Thymalus limbatus* Fabr.; 3. [*Ips quadrimaculata* Fabr.]; 4. *Nitidula aenea* Fabr.; 5. [*Cercus pedicularius* Lin.]; 6. *Byturus tomentosus* Fabr.; 7. *Dacne humeralis* Fabr.; 8. [*Cryptophagus cellaris* Fab.]; 9. [*Aspidiphorus orbiculatus* Gyll.]; 10. *Dermestes lardarius* Linn.; 11. *Megatoma pellio* Linn.; 12. [*Limnichus sericeus* Duftschmid]; 13. [*Attagenus serra* Fab.]; 14. [*Trogoderma versicolor* Creutz.]; 15. *Anthrenus museorum* Linn.; 16. [*Globicornis nigripes*]. **Plate 37** [DP: 11 May 1843]: 1. *Nosodendron fasciculare* Fabr.; 2. *Byrrhus pilula* Lin.; 3. *Heterocerus marginatus* Fab.; 4. *Potamophilus acuminatus* Fabr.; 5. *Dryops prolifericornis* Fabr.; 6. *Elmis aeneus* Müller; 7. *Macronychus quadrituberculatus* Müller; 8. [*Georissus pygmaeus* Fabr.]. **Plate 38** [DP: 10 February 1843]: 1. *Elophorus aquaticus* Fab.; 2. *Hydrochus elongatus* Fab.; 3. *Octhebius riparius* Illig.; 4. *Spercheus emarginatus* Fab.; 5. *Hydrophilus piceus* Fab.; 6. [*Hydrobius melanocephalus* Fabr.]; 7. *Berosus luridus* Fab.; 8. *Sphaeridium scarabaeoides* Linn. **Plate 39** [DP: 10 February 1843]: 1. *Ateuchus sacer* Lin.; 2. *Gymnopleurus pilularius* Fab.; 3. *Sisyphus schaefferi* Fab.; 4. [*Circellium bacchus* Fabr.]; 5. *Coprobius smaragdulus* Fab.; 6. [*Choeridium subaeneum* Dejean]; 7. [*Eurysternus foedus* Guer.]; 8. *Oniticellus flavipes* Fab.; 9. *Onthophagus taurus* Lin. **Plate 39bis** [DP: 10 February 1843]: 1. *Onitis olivieri* Illig.; 2. *Phanaeus ensifer* Germ.; 3. *Copris lunaris* Linné; 4. *Aphodius fimetarius* Linné; 5. [*Psammodius sulcicollis* Gyllenhall]; 6. *Aegialia arenaria* Gyllenh.; 7. *Chiron digitatum* Fabr. **Plate 40** [DP: 10 February 1843]: *Lethrus cephalotes* Fabr.; 2. *Geotrupes stercorarius* Linn.; 3. [*Ochodoeus chrysomelinus* Fabr.]; 4. [*Athyraeus fulvescens* Blanchard]; 5. *Bolboceras mobilicornis* Fabr.; 6. *Hybosorus arator* Fabr.; 8. [*Acanthocerus orbignyi* Blanchard]; 9. *Trox sabulosus* Fabr.; 10. *Oryctes nasicornis* Linn.; 11. *Agacephala latreillei* Dej. **Plate 40bis** [DP: July 1841]: *Scarabaeus hercules* Lin. **Plate 41** [DP: 11 May 1843]: 1. *Phileurus didymus* Fab.; 2. *Hexodon reticulatum* Oliv.; 3. *Cyclocephala geminata* Fab.; 4. *Chrysophora chrysochlora* Latr.; 5. *Rutela lineola* Fab.; 6. *Macraspis clavata* Fab. **Plate 42** [DP: July 1843]: 1. *Pachypus excavatus* Fab.; 3. *Anoplognatha latreillei* Sch.; 4. *Leucothyreus aeneicollis* Dej.; 5. [*Apogonia rauca* Fabr.]; 6. *Geniates barbata* Kirb.; 7. *Melolontha vulgaris* Lin.; 8. *Rhizotrogus solstitialis* Fab.; 9. *Ceraspis pruinosa* Less. et Gerv. **Plate 43** [DP: July 1843]: 1. *Areoda lanigera* Fab.; 2. [*Serica ruricola* Fabr.]; 3. *Diphucephala sericea* Kirby; 4. *Macrodactylus subspinosus* Fab.; 5. *Plectris tomentosa* Lep. et Serv.; 6. *Popilia bipunctata* Fab.; 7. *Euchlora vitis* Fab.; 8. *Anisoplia horticola* Fab.; 9. [*Lepisia rupicola* Fabr.]. **Plate 44** [DP: 10 August 1843]: 1. *Dicrania rubricollis* Lep. et Serv.; 2. *Hoplia farinosa* Fabr.; 3. [*Monochelus gonagra* Fabr.]; 4. [*Glaphyre serratulae* Fabr.]; 5. *Amphicoma parreyssii* Brullé; 6. *Anthipna abdominalis* Latr.; 7. *Chasmatopterus villosulus* Illig.; 8. [*Chasme decora* Fabr.]; 9. [*Dichelus dentipes* Fabr.]; 10. [*Lepitrix lineatus* Fabr.]; 11. *Pachycnema crassipes* Fabr.; 12. *Anisonyx crinitus* Fabr. **Plate 45** [DP: July 1843]: 1. *Trichius fasciatus* Lin.; 2. *Platygenia barbata* Schoenh.; 3. [*Chremastochilus hottentota* Gory et Perch.]; 4. *Goliathus giganteus* Lamark; 5. *Gymnetis nitida* Fab.; 6. *Cetonia aurata* Lin. **Plate 45bis** [DP: 10 August 1843]: 1. *Sinodendron cylindricum* Lin.; 2. [*Aesalus scarabaeoides* Fabr.]; 3. *Lamprima aenea* Fabr.; 4. [*Ryssonotus nebulosus* Kirby]; 5. [*Pholidotus humboldtii* Dalman]; 6. *Lucanus cervus* Lin.; 7. *Platycerus caraboides* Fabr.; 8. *Syndesus cornutus* Fabr.; 9. *Passalus interruptus* Fabr. **Plate 46** [DP: July 1841]: 1. *Pimelia bipunctata* Fab.; 2. [*Trachyderma hispida* Fab.]; 3. *Cryptochile maculata* Fab.; 4. *Erodius bilineatus* Oliv.; 5. [*Zophosis quadrilineata* Oliv.]; 6. *Nyctelia nodosa* Germ.; 7. [*Hegeter striatus* Latr.]; 8. *Tentyria orbiculata* Fab.; 9. *Akis punctata* Fab.; 10. [*Elenophorus collaris* Fabr.]; 11. *Eurichora ciliata* Thunb.; 12. [*Adelostoma sulcatum* Dup.]. **Plate 47**[DP: July 1841]: 1. *Tagenia angustata* Herbst; 2. [*Psammeticus costatus* Guer.]; 3. *Scaurus tristis* Oliv.; 4. *Scotobius pilularius* Germ.; 5. *Sepidium cristatum* Fab.; 6. [*Trachynotus reticulatus* Fab.]; 7. *Moluris striata* Fab.; 8. [*Oxura setosa* Kirby]; 9. [*Acanthomera gratilla* Herbst]; 10. *Misolampus hoffmanseggii* Lat.; 11. *Blaps mortisaga* Lin. **Plate 48** [DP: January 1844]: 1. [*Gonopus tibialis* Latr.]; 2. *Heteroscelis dentipes* Fabr.; 3. *Machla serrata* Fabr.; 4. *Scotinus crenicollis* Kirby; 5. *Asida grisea* Lin.; 6. *Pedinus femoralis* Fabr.; 7. [Pedinus (Opatrinus) clathratus Fabr.]; 8. [Pedinus (Dendarus) tristis Rossi]; 9. [Pedinus (Eurynotus) muricatus Kirby]; 10. *Isocerus ferrugineus* Fabr.; 11. [*Blapstinus punctatus* Schoenh.]; 12. *Platyscelis gages* Fisch. **Plate 49** [DP: January 1844]: 1. *Crypticus glaber* Fabr.; 2. *Opatrum sabulosum* Lin.; 3. [*Corticus celtis* Germ.]; 4. *Orthocerus muticus* Lin.; 5. *Chiroscelis digitata* Fabr.; 6. [*Toxicum quadricorne* Fabr.]; 7. [*Boros corticalis* Gyllenh.]; 8. *Calcar elongatus* Herbst; 9. *Upis ceramboides* Fabr.; 10. *Tenebrio molitor* Lin.; 11. *Heterotarsus tenebrioides* Guer. **Plate 50** [DP: March 1844]: 1. *Phaleria cadaverina* Fabr.; 2. *Diaperis boleti* Lin.; 3. *Hypophlaeus castaneus* Fabr.; 4. [*Trachyscelis aphodioides* Latr.]; 5. *Leiodes castaneus* Payk.; 6. [*Tetratoma fungorum* Fabr.]; 7^134^. *Eledona crenata* Fabr.; 8. *Cossyphus hoffmanseggii* Herbst; 9. [*Heloeus brownii* Kirby]; 10. *Nilio villosus* Fabr. **Plate 51** [DP: March 1844]: 1. *Epitragus fuscus* Latr.; 2. [*Cnodalum atrum* Dej. (inédit)]; 3. *Campsia cuprea* Dej. [inédit]; 4. *Spheniscus erotyloides* Kirby; 5. [*Acanthopus dentipes* Panz.]; 6. [*Amarygmus cuprarius* Weber]; 7. *Sphaerotus curvipes* Kirby; 8. *Adelium abbreviatum* Latr.; 9. *Helops caraboides* Panz.; 10. *Laena pimelia* Fabr. **Plate 52** [DP: May 1844]: 1. *Stenotrachelus aeneus* Payk.; 2. [*Strongylium chalconotum*? Kirby (*Soerangodes cicatricosus* Dej.)]; 3. *Pytho depressus* Lin.; 4. *Listronychus equestris* Fabr.; 5. *Cistela sulphurea* Lin.; 6. [*Mycetochares morio* Fabric.]; 7. *Allecula flavo-pubescens* Blanch.; 8. [*Orchesia micans* Fabr.]; 9. *Eustrophus dermestoides* Fabr.; 10. [*Hallomenus affinis* Fabr.]; 11. *Dircaea quadriguttata* Fabr. **Plate 53** [DP: September 1844]: 1. *Melandrya caraboides* Lin.; 2. [*Hypulus bifasciatus* Gyllenh.]; 3. *Serropalpus striatus*; 4. [*Conopalpus flavicollis* Gyllenh.]; 5. *Nothus praeustus* Oliv.; 6. *Calopus serraticornis* Lin.; 7. [*Sparedrus testaceus* Schoenh.]; 8. [*Dytilus ruficollis* Fabr.]; 9. *Oedemera coerulea*; 10. [*Stenostoma rostratum* Fabric.]; 11. *Mycterus curculioides* Fab.; 12. *Rhinosimus roboris* Fab. **Plate 53bis** [DP: September 1844]: 1. *Lagria hirta* Lin.; 2. *Statyra agroides* Lep. et Serv.; 3. [*Dendroides ruficollis* Dej.]; 4. *Pyrochroa rubens* Fab.; 5. *Rhipiphorus bimaculatus* Fab.; 6. *Myodites subdipterus* Fab.; 7. [*Pelecotoma mosquense* Fischer]; 8. *Mordella fasciata* Fab.; 9. *Anaspis frontalis* Fab. **Plate 54** [DP: August 1841]: 1. *Scraptia fusca* Latr.; 2. *Steropes caspius* Stev.; 3. *Notoxus monoceros* Lin.; 4. *Horia maculata* Fabr.; 5. *Cerocoma schaefferi* Lin.; 6. [*Hycleus argentatus* Fab.]; 7. *Mylabris cichorii* Lin.; 8. [*Lydus algiricus* Fabr.]; 9. *Oenas afer* Fab. **Plate 55** [DP: January 1841]: 1. *Meloe proscarabaeus* Lin.; 2. *Tetraonyx sex-guttata* Klug; 3. *Cantharis vesicatoria* Lin.; 4. *Zonitis praeusta* Fab.; 5. *Nemognathus chrysomelinus* Fab.; 6. [*Gnathium francilloni*]; 7. *Sitaris humeralis* Fab. **Plate 56** [DP: November 1843]: 1. *Anthribus latirostris* Fabr.; 2. *Bruchus pisi* Lin.; 3. *Rhaebus gebleri* Fisch.; 5. *Apoderus coryli* Lin.; 6. *Attelabus curculionoides* Lin.; 7. *Rhynchites bacchus* Herbst; 8. *Apion pomonae* Fab.; 9. *Rhinotia haemoptera* Kirby. 10. [*Eurhinus acanthopterus* Boisd.]; 11. [*Tubicenus rhynchitoides* Dej. (*Auletes tubicenus* Schoenh.)]. **Plate 57** [DP: November 1844]: 1. *Brentus italicus*^135^ Fab.; 2. *Ulocerus squalidus* Dalm.; 3. *Cyclas formicarius* Oliv.; 4. *Brachycerus obesus* Oliv.; 5. [*Cyclomus simus* Schoenh.]; 6. *Curculio imperialis* Lin.; 7. *Leptocerus rivulosus* Germ.; 8. [*Phyllobius argentatus* Linné]; 9. *Otiorhynchus ligustici* Lin. **Plate 58** [DP: September 1844]: 1. *Omias brunnipes* Oliv.; 2. [*Pachyrhynchus moniliferus* Esch.]; 3. [*Thylacites hispidus* Schoenh.]; 4. *Hyphantus baccifer* Germ.; 5. *Myniops variolosus* Schoenh.; 6. *Liparus germanus* Fabr.; 7. *Coniatus tamarisci*^136^ Fab.; 8. [*Hylobius abietis* Fabr.]; 9. *Cleonus cruciatus* Illig.; 10. *Lixus paraplecticus* Lin.; 11. [*Tamnophilus violaceus*]. **Plate 59** [DP: December 1844]: 1. [*Bagous binodulus* Herbst]; 2. [Brachypus (Brachonyx) indigena Herbst]; 3. *Balaninus nucum* Lin.; 4. [*Rhynchaenus bimaculatus* Fabr.]; 5. [*Sibynes viscariae* Linné]; 6. [*Myorhinus pulverulentus*]; 7. [Myorhinus (Styphlus) setiger Germ.]; 8. *Cionus scrophulariae* Lin.; 9. *Orchestes alni* Lin.; 10. *Amerhinus dufresnii* Kirby; 11. *Baridius chloris* Fab.; 12. [*Camptorhynchus cyaneus* Schoenh.]; 13. [*Centrinus semiluctuosus* Blanchard]; 14. *Zygops strix* Oliv.; 15. *Ceutorhynchus echii* Fab. **Plate 60** [DP: December 1844]: 1. [*Hydaticus velatus* Schoenh.]; 2. [*Orobitis globosus* Fab.]; 3. *Cryptorhynchus lapathi* Lin.; 4. *Tylodes ptinoides* Gyll.; 5. *Anchonus suillus* Fab.; 6. *Rhina barbirostris* Oliv.; 7. *Calandra palmarum* Lin.; 8. *Cossonus linearis* Fab.; 9. *Dryophthorus limexylon* Fab. **Plate 61** [DP: November 1843]: 1. [*Hylurgus ater* Fabr.]; 2. *Hylesinus piniperda* Fabr.; 3. *Scolytus destructor* Lin.; 4. *Camptocerus aeneipennis* Fabr.; 5. [*Phlaeotribus oleae*]; 6. [*Tomicus typographus* Lin.]; 7. *Platypus cylindricus* Oliv.; 8. *Paussus aethiops* Blanch.; 9. [*Cerapterus latipes* Swed.]; 10. *Bostrichus capucinus* Lin.; 11. *Psoa dubia* Rossi. **Plate 62** [DP: November 1843]: 1. *Cistela boleti* Fabr.; 2. [*Nemosoma elongatum*]; 3. [*Synchita juglandis* Fabr.]; 4. *Cerylon histeroides* Latr.; 5. [*Rhizophagus dispar* Fabr.]; 6. *Monotoma picipes* Herbst; 7. *Lyctus canaliculatus* Fabr.; 8. [*Diodesma subterranea* Dej.]; 9. *Bitoma crenata* Fabr.; 10. *Colydium sulcatum* Fabr.; 11. *Mycetophagus quadrimaculatus* Fabr.; 12. [*Triphyllus bifasciatus* Fabr.]. **Plate 63** [DP: October 1843]: 1. [*Dasycerus sulcatus* Brongn.]; 2. [*Merix rugosa* Latr.]; 3. *Latridius porcatus* Herbst; 4. *Sylvanus unidentatus* Fabr.; 5. *Trogossita mauritanica* Lin.; 6. *Prostomis mandibularis* Fabr.; 7. *Passandra fasciata* Guer.; 8. *Cucujus depressus* Fabr.; 9. [*Dendrophagus crenatus* Payk.]; 10. *Uleiota flavipes* Fabr. **Plate 64** [DP: August 1841]: 1. *Parandra maxillosa* Lin.; 2. *Spondylis buprestoides* Lin.; 3. *Prionus cervicornis*^137^ Lin.; 4. - [*Prionus*] *coriarius* Lin. **Plate 65** [DP: 10 August 1843]: 1. [*Lissonotus purpuratus* Germ.]; 2. *Megaderus stigma* Lin.; 3. *Dorcacerus barbatus* Oliv.; 4. *Trachyderes succinctus* Lin.; 5. [*Lophonocerus barbicornis* Fabr.]; 6. [*Ctenodes geniculatus* Klug]; 7. *Phoenicocerus dejeanii* Lat.; 8. *Callichroma moschata* Lin. **Plate 66** [DP: October 1843]: 1. *Acanthopterus kaehleri*^138^ Fab. var.; 2. *Acanthopterus alpinus*^139^ Lin.; 3. *Cerambyx heros* Lin.; 4. [*Cerambyx scopulicornis*^140^ Kirby]; 5. *Callidium arietis* Lin.; 6. *Callidium sanguineum* Lin.; 7. *Obrium cantharinum* Lin. **Plate 66bis** [DP: October 1843]: 1. *Rhinotragus trizonatus* Blanch.; 2. *Stenopterus rufus* Lin.; 3. *Necydalis major* Lin.; 4. *Distichocerus fuliginosus* Blanch.; 5. *Tmesisternus septempunctatus* Boisd.; 6. *Tragocerus spencei* Hope. **Plate 67** [DP: January 1841]: 1. *Leptocera scripta* Lin.; 2. *Acrocinus longimanus* Lin.; 3. *Acanthocinus aedilis* Fab.; 4. *Tetraopes varicornis* Klug. **Plate 68** [DP: January 1841]: 1. *Monochamus sutor* Lin.; 2. *Lamia textor* Lin.; 3. *Dorcadion fuliginator* Lin.; 4. [*Parmena pilosa*]; 5. *Gnoma giraffa* Fab.; 6. [*Adesmus luctuosus* Dej.]; 7. [*Apomecyna histrio* Fabr.]; 8. *Colobothea picta* Perty; 9. *Saperda carcharias* Lin. **Plate 69** [DP: January 1841]: 1. *Desmocerus cyaneus* Fab.; 2. *Vesperus solieri* Lin. [Dej.]; 3. *Rhagium mordax* Fab.; 4. [*Rhamnusium salicis* Fab.]; 5. *Toxotus meridianus* Fab.; 6. *Stenoderus ceramboides* Kirb.; 7. *Leptura tomentosa* Lin. **Plate 70** [DP: August 1840]: 1. *Megalopus subfasciatus* Germ.; 2. *Sagra boisduvalii* Dej.; 3. *Orsodacna cerasi* Fab.; 4. *Psammaechus bipunctatus* Fab.; 5. *Donacia sagittariae* Lin.; 6. [*Hoemonia equiseti* Fab.]; 7. [*Petauristes crassipes* Oliv.]; 8. *Crioceris merdigera* Lin. **Plate 71** [DP: July 1841]: 1. *Auchenia subspinosa* Fab.; 2. *Megascelis vittata* Fab.; 3. *Alurnus marginatus* Fab.; 4. *Hispa atra* Lin.; 5. [*Chalepus spinipes* Fabr.]; 6. [*Imatidium fasciatum* Fabr.]; 7. *Cassida equestris* Lin. **Plate 72** [DP: August 1841]: 1. *Clythra quadripunctata* Lin.; 2. *Chlamys monstrosa* Fab.; 3. *Lamprosoma bicolor* Kirby; 4. *Crytocephalus sericeus* Lin.; 5. [*Choragus scheppardi* Kirby]; 6. [*Euryope rubra* Lat.]; 7. *Eumolpus vitis* Fab.; 8. *Colaspis barbara* Fab.; 9. *Podontia quatuordecimpunctata* Fab. **Plate 73** [DP: January 1844]: 1. *Phyllocharis cyanitarsis* Fabr.; 2. *Doryphora pustulata* Fabr.; 3. [*Paropsis marmorea* Oliv.]; 4. *Timarcha tenebricosa* Fabr.; 5. *Chrysomela cerealis* Lin.; 6. [*Phaedon cochleariae*]; 7. *Prasocuris phellandrii* Fabr.; 8. [*Adorium bipunctatum* Fabr.]; 9. [*Luperus rufipes* Fabr.]; 10. *Galleruca calmariensis* Lin.; 11. *Altica oleracea* Lin.; 12. [*Oedionychis decemguttata* Fabr.]; 13. [*Psylliodes affinis* Payk.]; 14. [*Longitarsus sisymbrii* Fabr.]. **Plate 74** [DP: March 1844]: 1. *Erotylus histrio* Lin.; 2. *Aegithus marginatus* Fabr.; 3. *Triplax russica* Fabr.; 4. *Tritoma bipustulatum* Oliv.; 5. *Languria scapularis* Chev.; 6. *Phalacrus corruscus* Payk.; 7. *Agathidium seminulum* Fabr. **Plate 74bis** [DP: May 1844]: 1. *Eumorphus marginatus* Fabr.; 2. *Daspsa* [sic] *trimaculata* Cast.; 3. *Endomychus coccineus* Fabr.; 4. *Lycoperdina bovistae* Fabr.; 5. *Lithophilus connatus* Fabr.; 6. *Coccinella septem-punctata* Lin.; 7. *Clypeaster lateralis* Gyll. **Plate 75** [DP: May 1844]: 1. *Chennium bituberculatum* Lin.; 2. [*Dionyx dejeanii* Latr.]; 3. *Pselaphus heisei* Herbst; 4. *Bythinus curtisii* Leach; 5. [*Arcophagus glabricollis* Reich., Leach]; 6. *Ctenistes palpalis* Reichenb.; 7. *Bryaxis longicornis* Leach; 8. *Claviger foveolatus* Muller.

### Appendix 2. List of Coleoptera illustrated on the plates of ‘*Voyage au Pôle Sud... Zoologie Atlas*’ (Blanchard, 1842–1854)

**Plate 1** [DP: 1 December 1842]: 1. *Cicindela insularis* Blanch.; 2. *Cicindela boyerii* Nob.; 3. *Cicindela sanguineo maculata* Nob.; 4. *Cicindela variolosa* Nob.; 5. *Cicindela vitiensis* Nob.; 6. *Cicindela montravelii* Nob.; 7. *Cymindis australis* Nob.; 8. *Lebia binotata* Nob.; 9. *Onypterygia irina* Nob.; 10. *Omalodera limbata* Nob.; 11. *Omalodera discoidalis* Nob.; 12. *Metius splendidus* Guér.; 13. *Cascellus niger* Nob.; 14. *Oodes fuscitarsis* Nob.; 15. *Anchomenus atratus* Nob.; 16. *Sphenopalpus parallelus* Nob. **Plate 2** [DP: 2 February 1843]: 1. *Pristonychus castaneus* Nob.; 2. *Pristonychus brevis* Nob.; 3. *Calathus rubromarginatus* Nob.; 4. *Omaseus elongatus* Nob.; 5. *Omaseus sylvaticus* Nob.; 6. *Argutor pantomelas* Nob.; 7. *Agonum erythropus* Nob.; 8. *Argutor piceus* Nob.; 9. *Paecilus luridus* Nob.; 10. *Platycaelus depressus* Nob.; 11. *Cratogaster sulcatus* Nob.; 12. *Platysma magellanica* Nob.; 13. *Abax australasiae* Guér.; 14. *Mecodema sculpturatum* Nob.; 15. *Oopterus plicaticollis* Nob.; 16. *Ocnera clivinoïdes* Guér. **Plate 3** [DP: 4 May 1843]: 1. *Antarctia lata* Guér.; 2. *Asida complanata* Nob.; 3. *Asida chalybea* Nob.; 4. *Asida glauca* Nob.; 5. *Promecoderus gibbosus* Guér.; 6. *Tetraodes laevis* Nob.; 7. *Carabus chiliensis* Eschsch.; 8. *Carabus suturalis* Fab.; 9. *Carabus suturalis* Fab.; 10. *Carabus suturalis* Fab.; 11. *Carabus suturalis* Fab.; 12. *Acupalpus testaceipes* Nob.; 13. *Acupalpus nigronitidus* Nob.; 14. *Anochilia erythroderes* Nob.; 15. *Threchus testaceus* Nob.; 16. *Dromius fossulatus* Nob. **Plate 4** [DP: 26 August 1843]: 1. *Hydaticus scriptus* (Nob.); 2. *Hydroporus mixtus* (Nob.); 3. *Hydroporus quadrivittatus* (Nob.); 4. *Agabus lugubris* (Nob.); 5. *Dineutes janthinus* (Nob.); 6. *Phillydrus atlanticus* (Nob.); 7. *Byrrhus australis* (Nob.), 8. *Nitidula latens* (Nob.); 9. *Cryptophagus rufescens* (Nob.); 10. *Saprinus viridicupreus* (Nob.); 11. *Leptochirus samoensis* (Nob.); 12. *Paederus peregrinus* (Erich.); 13. *Tillus bipartitus* (Nob.); 14. *Opilus zonatus* (Nob.); 15. *Hydnocera nitens* (Newm.); 16. *Pilus fatuus* (Newm.). **Plate 5** [DP: 19 July 1844]: 1. *Trichodes herbaceus* Nob.; 2. *Telephorus insularis* Nob.; 3. [*Telephorus*] *flavifemoralis* Nob.; 4. [*Telephorus*] *schrefferi* Guer.; 5. *Telephorus ruficollis* Nob.; 6. *Chauliognathus magellanicus* Nob.; 7. [*Chauliognathus*] *bioculatus* Nob.; 8. *Silis maculicollis* Nob.; 9. *Lycus dorycus* Boisd.; 10. [*Lycus*] *latipes* Nob.; 11. [*Lycus*] *rufo-sternalis* Nob.; 12. [*Lycus*] *rufipennis* Nob.; 13. *Lycus flavicans* Nob.; 14. [*Lycus*] *pilosicornis* Nob.; 15. *Luciola plagiata* Nob.; 16. *Prionophorus bicolor* Nob. **Plate 6** [DP: 20 December 1844]: 1. *Luciola limbata* Nob.; 2. *Lycus melanurus* Nob.; 3. *Callirhipis bicolor* Nob.; 4. *Callirhypis dejeanii* Latr.; 5. *Callirhipis castaneus* Nob.; 6. *Monocrepidius cinereus* Nob.; 7. *Agrypnus nigroplagiatus* Nob.; 8. *Lacon triste* Nob.; 9. *Corymbites ramifer* Eschsch.; 10. *Elater nitido-fuscus* Nob.; 11. *Agriotes australasiae* Nob.; 12. *Agriotes magellanicus* Nob.; 13. *Agriotes quadripunctatus* Nob.; 14. *Eucnemis concolor* Nob.; 15. *Colobogaster viridi-punctatus* Nob.; 16. *Buprestis aurantiaco-adspersa* Nob. **Plate 7** [DP: 20 December 1844]: 1. *Parastasia nigro-maculata* Nob.; 2. *Parastasia rufo-limbata* Nob.; 3. *Polyphyllum rubrescens* Nob.; 4. *Calocnemis castaneus* Nob.; 5. *Euchirus longimanus* Lin.; 6. *Heteronychus sanctae-helenae* Nob.; 7. *Onthophagus flavo-lineatus* Nob.; 8. *Onthophagus umbraculatus* Nob.; 9. *Onthophagus armatus*. Mâle. Nob.; 10. *Onthophagus armatus*. fem. 11. *Onthophagus viridi-obscurus* Nob.; 12. *Onthophagus cupreo-viridis* Nob.; 13. *Onthophagus parvus* Nob.; 14. *Aphodius australasiae*. **Plate 8** [DP: 5 November 1846]: 1. *Anoplognathus opalinus* (Nob.); 2. *Anomala maculicollis* (Nob.); 3. *Anomala pallidipennis* (Nob.); 4. *Anomala fuscoviridis* (Nob.); 5. *Odontria striata* (White); 6. *Ancylonycha irrorata* (Nob.); 7. *Adoretus mutabilis* (Fabr.); 8. *Serica tasmanica* (Nob.); 9. *Brachyphylla magellanica* (Nob.); 10. *Listronyx melanocephala* (Guer.); 11. *Phyllochloenia rufescens* (Nob.); 12. *Heteronyx castaneus* (Nob.); 13. *Heteronyx pilosus* (Nob.); 14. *Liparetrus vestitus* (Nob.); 15. *Logisticus obscurus* (Nob.); 16. *Liparetrus convexus* (Mac Leay); 17. *Hymenoplia villigera* (Nob.); 18. *Omaloplia rubricollis* (Nob.); 19. *Omaloplia vittigera* (Nob.); 20. *Sericesthis rufipennis* (Boisd.). **Plate 9** [DP: 6 July 1846]: 1. *Scarabaeus atlas*. Mâle. (Fabr.); 2. *Scarabaeus phidias* Mâle. (Nob.); 3. *Scotias psylloides* Femelle. (Nob.); 4. *Cetonia flamulla* (Nob.); 5. *Cetonia glauca* (Nob.); 6. *Lomaptera virens* (Nob.); 7. *Lomaptera urvillei* (Burm.); 8. *Lomaptera rugata* (Nob.); 9. *Anacamphthorina ignipes* (Nob.); 10. *Lucanus concolor* (Nob.); 11. *Lucanus fulvolimbatus* Mâle (Nob.); 12. *Lamia formosa* Femelle (Nob.); 13. *Lucanus zelandicus* (Nob.); 14. *Figulus insularis* (Nob.). **Plate 10** [DP: 5 November 1846]: 1. *Nyctelia multicristata* (Nob.); 2. *Praocis reflexicollis* (Nob.); 3. *Praocis depressa* (Guer.); 4. *Cilibe elongatus* (De Brème). 5. *Cilibe elongatus* (De Brême); 6. *Cilibe granulosus* (De Brème); 7. *Cilibe phosphugoides* (White); 8. *Pterohelaeus insularis* (De Brème); 9. *Emalodera obesa* (Guer.); 10. *Opatrum aequatoriale* (Nob.); 11. *Osphya aeneipennis* var minor (Nob.); 12. *Osphya molluccanum* (Nob.); 13. *Opatrum denticolle* (Nob.); 14. *Osphya longum* (Nob.); 15. *Osphya villiger* (Nob.); 16. *Epilasium incisum* (Nob.). **Plate 11** [DP: 6 April 1847]: 1. *Opatrum fasciolatum* (Nob.); 2. *Bolitophagus amicorum* (Nob.); 3. *Bolitophagus anguliferus* (Nob.); 4. *Uloma insularis* (Nob.) Mâle; 5. *Uloma insularis* (Nob.); Fem.; 6. *Uloma laevicostata* (Nob.); 7. *Zophobas zelandicus* (Nob.); 8. *Opatrinus laticollis* (Nob.); 9. *Tenebrio humilis* (Erichs.); 10. *Tenebrio nigerrimus* (Dej.); 11. *Nyctobates aequatorialis* (Nob.); 12. *Dolphus globipennis* (Nob.); 13. *Helops oblongiusculus* (Nob.); 14. *Helops sulcicollis* (Boisd.); 15. *Tanychilus metallicus* (White); 16. *Rygmodus pedinoides* (White); 17. *Pseudhelops tuberculatus* (Guer.); 18. *Adelium cisteloides* (Erichs.); 19. *Oplocephala chalcea* (Nob.) Mâle; 20. *Orthorinus cylindrirostris* (Nob.) Fem. **Plate 12** [DP: 5 November 1846]: 1. *Platydema oblonga* (Nob.); 2. *Strongylium rugosum* (Nob.); 3. *Epilampus violaceus* (Nob.); 4. *Amarygmus fulgidi-tessellatus* (Nob.); 5. *Amarygmus tuberculiger* (Nob.); 6. *Amarygmus ruficrurus* (Nob.); 7. *Amarygmus columbinus* (Nob.); 8. *Calodemum viride* (Nob.); 9. *Lagria rufescens* (Latr.); 10. *Lucanus concolor* (Nob.); 11. *Laena pulchella* (Guer.); 12. *Lagria dimidiata* (Nob.); 13. *Pelecotoides murinus* (Nob.); 14. *Rhipiphorus bi-guttatus* (Nob.); 15. *Mordella bi-maculata* (Guer.); 16. *Mordella plurinotata* (Nob.); 17. *Zonitis anguliferus* (Nob.); 18. *Zonitis anguliferus* (Nob.) var. **Plate 13** [DP: 6 July 1846]: 1. *Anthribus semiluctuosus* (Nob.) Mâle; 2. *Adesmia sinaitica* (Nob.) Femelle; 3. *Anthribus albolineatus* (Nob.); 4. *Anthribus arciferus* (Nob.); 5. *Apoderus cygneus* (Fabr.) Femelle; 6. *Belus fascicularis* (Nob.); 7. *Belus cinereus* (Nob.); 8. *Pachyrhynchus biplagiatus* (Guer.); 9. *Elytrurus durvilleii* (Nob.); 10. *Eupholus petitii* (Guer.); 11. *Geonemus insignis* (Guer.); 12. *Geonemus plagiatus* (Nob.); 13. *Geonemus sordidus* (Nob.); 14. *Geonemus angustus* (Nob.); 15. *Geonemus azureipes* (Nob.); 16. *Geonemus zonatus* (Nob.); 17. *Ryssocarpus cinereus* (Nob.); 18. *Cylydrorhinus tessellatus* (Guer.); 19. *Colymbetes lineatus* (Nob.); 20. *Callideriphus clathratus* (Nob.). **Plate 14** [DP: 6 April 1847]: 1. *Acalles pallens* (Nob.); 2. *Rhinaria pusio* (Schoenh.); 3. *Elytrogonus varicosus* (Nob.); 4. *Cryptorhynchus clathratus* (Nob.); 5. *Cryptorhynchus sirius* (Erichs.); 6. *Euthyrinus tessellatus* (Nob.); 7. *Aterpus culthratus* (Fabr.); 8. *Gonipterus exaratus* (Schoenh.); 9. *Pselophax sulcatus* (White); 10. *Listroderes magellanicus* (Nob.); 11. *Gromilus insularis* (Nob.); 12. *Platyurus brevicornis* (Nob.); 13. *Cionus ferrugatus* (Nob.); 14. *Mecopus annulipes* (Nob.); 15. *Mecopus brevipennis* (Nob.); 16. *Rhyephenes immaculatus* (Nob.); 17. *Alcides notatus* (Nob.); 18. *Alcides australis* (Boisd.); 19. *Alcides albolituratus* (Nob.); 20. *Alcides albocinctus* (Chevrol.). **Plate 15** [DP: 5 November 1846]: 1. *Elytrogonus griseus* (Guer.); 2. *Erotylus oblongo-guttatus* Var; 3. *Otiorhynchus nodulosus* (Nob.); 4. *Otiorhynchus rotundipennis* (Nob.); 5. *Trigonops coerulescens* (Nob.); 6. *Coeleutetes cinerascens* (Nob.); 7. *Coptorhynchus ostentatus* (Schoenh.); 8. *Coptorhynchus ternatensis* (Guer.); 9. *Isomerinthus guttiger* (Nob.); 10. *Isomerinthus tessellatus* (Nob.); 11. *Isomerinthus elegans* (Guer.); 12. *Isomerinthus quadrilineatus* (Nob.); 13. *Isomerinthus elongatus* (Nob.); 14. *Isomerinthus lineolatus* (Nob.); 15. *Isomerinthus rufipes* (Nob.); 16. *Isomerinthus caudatus* (Guer.); 17. *Psomeles oblongus* (Nob.); 18. *Psomeles plagiatus* (Nob.); 19. *Sphoerorhinus villosus* (Guer.); 20. *Millocerus pruinosus* (Nob.). **Plate 16** [DP: 6 July 1846]: 1. *Microplophorus magellanicus* (Nob.) Mâle; 2. *Molorchus minor* (Nob.) Fem.; 3. *Phoracantha allapsa* (Newm.); 4. *Phoracantha fuscescens* (Nob.); 5. *Tesseromma undatum* (Newm.); 6. *Clytus aureicollis* (Nob.); 7. *Clytus trizonatus* (Nob.); 8. *Callidium fulvomaculatum* (Nob.); 9. *Cerambyx maculaticollis* (Nob.); 10. *Crinotarsus plagiatus* (Nob.); 11. *Tmesisternus rugosicollis* (Nob.); 12. *Tmesisternus biarciferus* (Nob.); 13. *Tasgius rufipes* (Nob.); 14. *Tmesisternus hieroglyphicus* (Nob.); 15. *Tmesisternus trivittatus* (Guer.); 16. *Tmesisternus trivittatus* (Guer.) Var.; 17. *Tryptocerus politus* (Nob.); 18. *Tmesisternus marmoratus* (Nob.); 19. *Tmesisternus sulcatipennis* (Nob.); 20. *Tmesisternus obsoletus* (Nob.). **Plate 17** [DP: 6 July 1846]: 1. *Coptomma variegatum* (Fabr.); 2. *Cylindrepomus nigrofasciatus* (Nob.); 3. *Callidiomorphus depressus* (Nob.); 4. *Callidium zelandicum* (Nob.); 5. *Monochamusrusticator* (Fabr.); 6. *Monochamus holotephrus* (Boisd.); 7. *Monochamus fulvo-irroratus* (Nob.); 8. *Gnoma albotessellata* (Nob.); 9. *Gnoma acuminipennis* (Nob.); 10. *Heteroclytomorpha quadrinotata* (Nob.); 11. *Prioneta albosignata* (Nob.); 12. *Acanthoderus pubescens* (Nob.); 13. *Hesperophanes obscurus* (Fabr.); 14. *Leiopus sinuatofasciatus* (Nob.); 15. *Saperda semiluctuosa* (Nob.); 16. *Saperda pantherina* (Nob.); 17. *Glaea viridinotata* (Nob.); 18. *Hatlia gracilis* (Nob.); 19. *Stenoderus grammicus* (Newm.); 20. *Macrones elongaticeps* (Nob.). **Plate 18** [DP: 6 July 1846]: 1. *Crioceris papuana* (Lacord.); 2. *Crioceris dichroa* (Nob.); 3. *Crioceris semilimbata* (Nob.); 4. *Crioceris nigrozonata* (Nob.); 5. *Botryonopa speciosa* (Dej.); 6. *Promecotheca coeruleipennis* (Nob.); 7. *Cephaloleia janthina* (Nob.); 8. *Hoplionota moluccana* (Nob.); 9. *Aspidimorpha miliaris* (Fab.) var; 10. *Aspidimorpha elevata* (Fab.) var; 11. *Aspidimorpha adhaerens* (Fab.); 12. *Aspidimorpha punctum* (Fab.) var; 13. *Aspidimorpha astrolabiana* (Nob.); 14. *Aspidimorpha philippinensis* (Reich.); 15. *Altica impressa* (Nob.); 16. *Aspidimorpha trivittata* (Fab.) var; 17. *Cassida angulifera* (Nob.); 18. *Phyllocharis cyanides* (Fab.); 19. *Phyllocharis immaculicollis* (Nob.); 20. *Rhaphidopalpa circumdata* (Nob.). **Plate 19** [DP: 6 July 1846]: 1. *Phyllocharis klugii* (Mac Leay); 2. *Phyllocharis splendidus* (D’Urv.); 3. *Phyllocharis splendens* (Guer.); 4. *Eumolpus vittatus* (Reiche); 5. *Eumolpus viridi-aeneus* (Reiche); 6. *Chalcolampra convexa* (Reiche); 7. *Adorium sexfasciatum* (Nob.); 8. *Adorium linteatum* (Nob.); 9. *Adorium quadrinotatum* (Nob.); 10. *Adorium sumatrense* (Nob.); 11. *Adorium concolor* (Nob.); 12. *Adorium limbatum* (Nob.); 13. *Adorium nigrfiventre* (Nob.); 14. *Adorium dimidiatum* (Nob.); 15. *Adorium dichroum* (Nob.); 16. *Adorium rubrum* (Nob.); 17. *Adorium bifasciatum* (Reiche); 18. *Adorium margineguttatum* (Nob.); 19. *Rhaphidopalpa rubro-zonata* (Nob.); 20. *Rhaphidopalpa melanopus* (Nob.).

### Appendix 3. List of Coleoptera illustrated on the plates of ‘*British entomology*’ (Curtis, 1824–1839)

Following the species name illustrated on the plate, the type species given by Curtis for the genus is listed in square brackets. The dates of publication of the plates are indicated in square brackets.

**Plate 1** [1 January 1824]: *Cicindela sylvicola* [*Cicindela campestris* Linn.]. **Plate 6** [1 February 1824]: *Nebria livida* [*Carabus complanatus* Linn.]. **Plate 11** [1 March 1824]: *Molorchus minor* [*Necydalis umbellatarum* Linn.]. **Plate 15** [1 April 1824]: *Omaseus aterrimus* [*Carabus aterrimus* Fab.]. **Plate 19** [1 May 1824]: *Rhipiphorus paradoxus* [*Mordella paradoxa* Linn.]. **Plate 23** [1 June 1824]: *Siagonum quadricorne* [*Siagonum quadricorne* K.]; **Plate 27** [1 July 1824]: *Aphodius villosus* [*Scarabaeus fossor* Linn.]. **Plate 31** [1 August 1824]: *Buprestis nitidula* [*Buprestis nitidula*]. **Plate 35** [1 September 1824]: *Crytocephalus bipustulatus* [*Chrysomela sericea* Linn.]. **Plate 39** [1 October 1824]: *Thymalus limbatus* [*Cassida limbata* Fab.]. **Plate 43** [1 November 1824]: *Scolytus destructor* [*Bostrichus scolytus* Fab.]. **Plate 44** [1 November 1824]: *Clerus alvearius* [*Attelabus apiarius* Linn.]. **Plate 47** [1 December 1824]: *Pogonus burrellii* [*Carabus chalceus* Marsh.]. **Plate 51** [1 January 1825]: *Platypus cylindrus* [*Platypus cylindrus* Herbst]. **Plate 52** [1 January 1825]: *Onthophagus taurus* [*Scarabaeus nuchicornis* Linn.]. **Plate 55** [1 February 1825]: *Melasis buprestoides* [*Elater buprestoides* Linn.]. **Plate 59** [1 March 1825]: *Cossonus tardii* [*Curculio linearis* Fab.]. **Plate 63** [1 April 1825]: *Acilius caliginosus* [*Dytiscus sulcatus* Linn.]^141^. **Plate 67** [1 May 1825]: *Agrilus chryseis* [*Buprestis viridis* Linn.]. **Plate 71** [1 June 1825]: *Necrophorus germanicus* [*Silpha humator* Linn.]. **Plate 75** [1 July 1825]: *Licinus depressus* [*Carabus cassideus* Fab.]. **Plate 79** [1 August 1825]: *Gyrinus bicolor* [*Gyrinus natator* Linn.]. **Plate 80** [1 August 1825]: *Parnus impressus* [*Parnus prolifericornis* Fab.]. **Plate 83** [1 September 1825]: *Chlaenius sulcicollis* [*Carabus nigricornis* Fab.]. **Plate 87** [1 October 1825]: *Lebia turcica* [*Carabus crux-minor* Linn.]. **Plate 91** [1 November 1825]: *Obrium cantharinum* [*Cerambyx cantharinus* Linn.]. **Plate 95** [1 December 1825]: *Hydaticus cinereus* [*Dytiscus transversalis* Fab.]. **Plate 99** [1 January 1826]: *Dyticus dimidiatus* [*Dytiscus marginalis* Linn.]. **Plate 103** [1 February 1826]: *Helobia gyllenhalii* [*Carabus brevicollis* Fab.]. **Plate 104** [1 February 1826]: *Hylurgus piniperda* [*Dermestes piniperda* Linn.]. **Plate 107** [1 March 1826]: *Dianous coerulescens* [*Stenus coerulescens* Gyll.]. **Plate 108** [1 March 1826]: *Paederus fuscipes* [*Staphylinus riparius* Linn.]. **Plate 111** [1 April 1826]: *Chrysomela adonidis* [*Chrysomela haemoptera* Linn.]. **Plate 112** [1 April 1826]: *Zonitis testacea* [*Zonitis praeusta* Fab.]. **Plate 115** [1 May 1826]: *Achenium depressum* [*Lathrobium depressum* Grav.]. **Plate 116** [1 May 1826]: *Hypera fasciculosa* [*Curculio punctatus* Fab.]. **Plate 119** [1 June 1826]: *Demetrias monostigma* [*Carabus atricapillus* Linn.]. **Plate 123** [1 July 1826]: *Tetratoma ancora* [*Tetratoma fungorum* Fab.]. **Plate 127** [1 August 1826]: *Cassida salicorniae* [*Cassida viridis* Fab.]. **Plate 131** [1 September 1826]: *Dendrophilus sheppardi* [*Hister punctatus* Ent. Heft.]. **Plate 135** [1 October 1826]: *Byrrhus dennii* [*Byrrhus pilula* Linn.]. **Plate 139** [1 November 1826]: *Badister cephalotes* [*Carabus bipustulatus* Fab.]. **Plate 143** [1 December 1826]: *Bledius skrimshirii* [*Staphylinus tricornis* Payk.]. **Plate 144** [1 December 1826]: *Cacidula scutellata* [*Chrysomela pectoralis* Fab.]. **Plate 148** [1 January 1827]: *Blaps obtusa* [*Tenebrio mortisagus* Linn.]. **Plate 149** [1 January 1827]: *Cicones carpini* [*Cicones carpini* Nob.]. **Plate 151** [1 February 1827]: *Cybister roeselii* [*Dytiscus lateralis* Fab.]. **Plate 155** [1 March 1827]: *Melandrya canaliculata* [*Chrysomela caraboides* Linn.]. **Plate 156** [1 March 1827]: *Mycetophagus piceus* [*Chrysomela 4-pustulata* Linn.]. **Plate 159** [1 April 1827]: *Hydrophilus caraboides* [*Dytiscus caraboides* Linn.]. **Plate 160** [1 April 1827]: *Cryptophagus populi* [*Chryptophagus populi* Gyll.]. **Plate 163** [1 May 1827]: *Throscus obtusus* [*Elater dermestoides* Linn.]. **Plate 164** [1 May 1827]: *Stenus kirbii* [*Stenus juno* Payk.]. **Plate 167** [1 June 1827]: *Malachius bispinosus* [*Cantharis bipustulatus* Linn.]. **Plate 168** [1 June 1827]: *Rugilus fragilis* [*Paederus orbiculatus* Fab.]. **Plate 171** [1 July 1827]: *Steropus concinnus* [*Carabus madidus* Linn.]. **Plate 172** [1 July 1827]: *Lamia nubila* [*Cerambyx textor* Linn.]. **Plate 175** [1 August 1827]: *Clivina collaris* [*Tenebrio fossor* Linn.]. **Plate 176** [1 August 1827]: *Leistus fulvibarbis* [*Carabus spinibarbis* Fab.]. **Plate 179** [1 September 1827]: *Elaphrus uliginosus* [*Cicindela riparia* Linn.]. **Plate 180** [1 September 1827]: *Callistus lunatus* [*Carabus lunatus* Fab.]. **Plate 183** [1 October 1827]: *Agonum austriacum* [*Carabus marginatus* Linn.]. **Plate 184** [1 October 1827]: *Calathus latus* [*Carabus cisteloides* Ill.]. **Plate 187** [1 November 1827]: *Poecilus lepidus* [*Carabus cupreus* Linn.]. **Plate 188** [1 November 1827]: *Zabrus obesus* [*Carabus gibbus* Fab.]. **Plate 191** [1 December 1827]: *Ophonus germanus* [*Carabus germanus* Linn.]. **Plate 192** [1 December 1827]: *Patrobus alpinus* [*Carabus rufipes* Fab.]. **Plate 196** [1 January 1828]: *Pterostichus elongatus* [*Carabus fasciato-punctatus* Fab.]. **Plate 197** [1 January 1828]: *Orchesia fasciata* [*Dircaea micans* Fab.]. **Plate 199** [1 February 1828]: *Clytus quadripunctatus* [*Cerambyx arietis* Linn.]. **Plate 200** [1 February 1828]: *Cillenum laterale* [*Cillenum laterale* Leach]. **Plate 203** [1 March 1828]: *Aëpus fulvescens* [*Aëpus fulvescens* Leach]. **Plate 204** [1 March 1828]: *Micropeplus tesserula* [*Staphylinus porcatus* Payk.]. **Plate 207** [1 April 1828]: *Colymbetes consobrinus* [*Dytiscus striatus* Linn.]. **Plate 208** [1 April 1828]: *Coccinella ocellata* [*Coccinella septem-punctata* Linn.]. **Plate 211** [1 May 1828]: *Apion difformis* [*Curculio frumentarius* Linn.]. **Plate 212** [1 May 1828]: *Magdalis carbonarius* [*Curculio cerasi* Linn.]. **Plate 215** [1 June 1828]: *Telephorus cyaneus* [*Cantharis fusca* Linn.]. **Plate 216** [1 June 1828]: *Dascillus cervinus* [*Chrysomela cervina* Linn.]. **Plate 219** [1 July 1828]: *Monochamus sartor* [*Cerambyx sutor* Linn.]. **Plate 220** [1 July 1828]: *Onthophilus sulcatus* [*Hister striatus* Fab.]. **Plate 223** [1 August 1828]: *Polistichus fasciolatus* [*Carabus fasciolatus* Rossi]. **Plate 224** [1 August 1828]: *Heterocerus obsoletus* [*Heterocerus marginatus* Bosc.]. **Plate 227** [1 September 1828]: *Odacantha melanura* [*Attelabus melanurus* Linn.]. **Plate 228** [1 September 1828]: *Syntomium nigroaeneum* [*Syntomium nigroaeneum* Nob.]. **Plate 231** [1 October 1828]: *Dromius spilotus* [*Carabus 4-maculatus* Linn.]. **Plate 232** [1 October 1828]: *Mezium sulcatum* [*Ptinus sulcatus* Fab.]. **Plate 235** [1 November 1828]: *Tarus basalis* [*Carabus humeralis* Fab.]. **Plate 236** [1 November 1828]: *Noterus sparsus* [*Dytiscus crassicornis* Fab.]. **Plate 239** [1 Decmber 1828]: *Hydröus piceus* [*Dytiscus piceus* Linn.]. **Plate 240** [1 December 1828]: *Berosus aericeps* [*Dytiscus luridus* Linn.]. **Plate 243** [1 January 1829]: *Hydrobius chalconotus* [*Dytiscus fuscipes* Linn.]. **Plate 244** [1 January 1829]: *Megatoma serra* [*Dermestes undatus* Linn.]. **Plate 246** [1 February 1829]: *Nosodendron fasciculare* [*Byrrhus fascicularis* Oliv.]. **Plate 247** [1 February 1829]: *Attagenus trifasciatus* [*Dermestes pellio* Linn.]. **Plate 250** [1 March 1829]: *Ochthebius hybernicus* [*Hydrophilus impressus* Marsh.]. **Plate 251** [1 March 1829]: *Leiodes cinnamomea* [*Tetratoma cinnamomea* Panz.]. **Plate 254** [1 April 1829]: *Notiophilus rufipes* [*Cicindela aquatica* Linn.]. **Plate 255** [1 April 1829]: *Hypulus biflexuosus* [*Hypulus quercinus* Payk.]. **Plate 258** [1 May 1829]: *Psammodius sulcicollis* [*Aphodius sulcicollis* Ill.]. **Plate 259** [1 May 1829]: *Bolboceras mobilicornis* [*Scarabaeus mobilicornis* Fab.]. **Plate 262** [1 June 1829]: *Aphanisticus pusillus* [*Buprestis pusilla* Oliv.]. **Plate 263** [1 June 1829]: *Lycus minutus* [*Lampyris sanguineus* Linn.]. **Plate 266** [1 July 1829]: *Geotrupes laevis* [*Scarabaeus stercorarius* Linn.]. **Plate 267** [1 July 1829]: *Tillus unifasciatus* [*Chrysomela elongata* Linn.]. **Plate 270** [1 August 1829]: *Opilus fasciatus* [*Attelabus mollis* Linn.]. **Plate 271** [1 August 1829]: *Apate capucinus* [*Dermestes capucina* Linn.]. **Plate 274** [1 September 1829]: *Platycerus caraboides* [*Lucanus caraboides* Linn.]. **Plate 275** [1 September 1829]: *Saperda atkinsoni* [*Cerambyx scalaris* Linn.]. **Plate 278** [1 October 1829]: *Polydrusus speciosus* [*Curculio cervinus* Linn.]. **Plate 279** [1 October 1829]: *Meloe brevicollis* [*Meloe proscarabaeus* Linn.]. **Plate 282** [1 November 1829]: *Lamprias cyanocephalus* [*Carabus cyanocephalus* Linn.]. **Plate 283** [1 November 1829]: *Bitoma crenata* [*Lyctus crenatus* Fab.]. **Plate 286** [1 December 1829]: *Trichius variabilis* [*Scarabaeus variabilis* Linn.]. **Plate 287** [1 December 1829]: *Masoreus luxatus* [*Trechus laticollis* Sturm]. **Plate 291** [1 January 1830]: *Enicocerus gibsoni* [*Enicocerus gibsoni* Curt.]. **Plate 292** [1 January 1830]: *Mononychus pseudacori* [*Rhynchaenus pseudacori* Fab.]. **Plate 294** [1 February 1830]: *Elmis volckmari* [*Dytiscus volckmari* Panz.]. **Plate 295** [1 February 1830]: *Callidium striatum* [*Cerambyx bajulus* Linn.]. **Plate 298** [1 March 1830]: *Helops pallidus* [*Helops caraboides* Panz.]. **Plate 299** [1 March 1830]: *Xylophilus oculatus* [*Anthicus oculatus* Panz.]. **Plate 302** [1 April 1830]: *Pelophila borealis* [*Carabus borealis* Payk.]. **Plate 303** [1 April 1830]: *Lesteva leachii* [*Staphylinus caraboides* Linn.]. **Plate 306** [1 May 1830]: *Ips quadripunctata* [*Dermestes ferrugineus* Linn.]. **Plate 307** [1 May 1830]: *Hydraena testacea* [*Hydraena riparia* Kug.]. **Plate 310** [1 June 1830]: *Blemus micros* [*Bembidium paludosum* Gyll.]. **Plate 311** [1 June 1830]: *Latridius elongatus* [*Ips transversus* Oliv.]. **Plate 314** [1 July 1830]: *Sarrotrium muticum* [*Hispa mutica* Linn.]. **Plate 315** [1 July 1830]: *Bryaxis sulcicollis* [*Staphylinus sanguineus* Linn.]. **Plate 318** [1 August 1830]: *Macroplea equiseti* [*Donacia zosterae* Fab.]. **Plate 319** [1 August 1830]: *Opatrum tibiale* [*Silpha sabulosa* Linn.]. **Plate 322** [1 September 1830]: *Cafius fucicola* [*Staphylinus xantholoma* Grav.]. **Plate 323** [1 September 1830]: *Crioceris puncticollis* [*Chrysomela asparagi* Linn.]. **Plate 326** [1 October 1830]: *Blethisa multipunctata* [*Carabus multipunctatus* Linn.]. **Plate 327** [1 October 1830]: *Nemosoma elongatum* [*Dermestes elongatus* Linn.]. **Plate 330** [1 November 1830]: *Calosoma sycophanta* [*Carabus sycophanta* Linn.]. **Plate 331** [1 November 1830]: *Tenebrio obscurus* [*Tenebrio molitor* Linn.]. **Plate 334** [1 December 1830]: *Necrodes littoralis* [*Silpha littoralis* Linn.]. **Plate 335** [1 December 1830]: *Simplocaria semistriata* [*Byrrhus semistriatus* Ill.]. **Plate 339** [1 January 1831]: *Strongylus imperialis* [*Nitidula strigata* Fab.]. **Plate 340** [1 January 1831]: *Sitaris humeralis* [*Necydalis humeralis* Fab.]. **Plate 342** [1 February 1831]: *Gibbium scotias* [*Ptinus scotias* Fab.]. **Plate 343** [1 February 1831]: *Hydroporus davisii* [*Dytiscus depressus* Fab.]. **Plate 346** [1 March 1831]: *Leiochiton readii* [*Scarites arcticus* Payk.]. **Plate 347** [1 March 18131]: *Oomorphus concolor* [*Byrrhus concolor* Sturm]. **Plate 350** [1 April 1831]: *Necrobia ruficollis* [*Dermestes ruficollis* Fab.]. **Plate 351** [1 April 1831]: *Corynetes violaceus* [*Dermestes violaceus* Linn.]. **Plate 354** [1 May 1831]: *Dyschirius inermis* [*Scarites thoracicus* Panz.]. **Plate 355** [1 May 1831]: *Lycoperdina bovistae* [*Endomychus bovistae* Fab.]. **Plate 358** [1 June 1831]: *Diaperis boleti* [*Chrysomela boleti* Linn.]. **Plate 359** [1 June 1831]: *Hydrochus elongatus* [*Elophorus elongatus* Fab.]. **Plate 362** [1 July 1831]: *Leptura apicalis* [*Leptura elongata* DeGeer]. **Plate 363** [1 July 1831]: *Uloma fagi* [*Tenebrio mauritanicus* Fab.]. **Plate 366** [1 August 1831]: *Adimonia quadrimaculata* [*Chrysomela halensis* Linn.]. **Plate 370** [1 September 1831]: *Luperus brassicae* [*Chrysomela flavipes* Linn.]. **Plate 371** [1 September 1831]: *Galeruca viburni* [*Chrysomela tanaceti* Linn.]. **Plate 374** [1 October 1831]: *Cetonia stictica* [*Scarabaeus auratus* Linn.]. **Plate 375** [1 October 1831]: *Serrocerus pectinatus* [*Ptilinus pectinatus* Fab.]. **Plate 379** [1 November 1831]: *Scaphidium quadrimaculatum* [*Scaphidium quadrimaculatum* Oliv.]. **Plate 382** [1 December 1831]: *Lymexylon navale* [*Cantharis navalis* Linn.]. **Plate 387** [1 January 1832]: *Anobium pertinax* [*Ptinus tessellatus* Linn.]. **Plate 390** [1 February 1832]: *Oedemera sanguinicollis* [*Necydalis caerulea* Linn.]. **Plate 394** [1 March 1832]: *Spercheus emarginatus* [*Spercheus emarginatus* Fab.]. **Plate 398** [1 April 1832]: *Thanasimus formicarius* [*Attelabus formicarius* Linn.]. **Plate 402** [1 May 1832]: *Cis bidentatus* [*Anobium boleti* Fab.]. **Plate 406** [1 June 1832]: *Melolontha fullo* [*Scarabaeus melolontha* Linn.]. **Plate 410** [1 July 1832]: *Lomechusa dentata* [*Lomechusa dentata* Grav.]. **Plate 414** [1 August 1832]: *Copris lunaris* [*Scarabaeus lunaris* Linn.]. **Plate 418** [1 September 1832]: *Oxyporus maxillosus* [*Staphylinus rufus* Linn.]. **Plate 422** [1 October 1832]: *Arcopagus puncticollis* [*Pselaphus bulbifer* Reich.]. **Plate 426** [1 November 1832]: *Cychrus rostratus* [*Tenebrio rostratus* Linn.]. **Plate 430** [1 December 1832]: *Hypophlaeus bicolor* [*Hypophlaeus castaneus* Fab.]. **Plate 435** [1 January 1833]: *Cardiapus mathewsii* [*Cardiapus mathewsii* Curt.]. **Plate 438** [1 February 1833]: *Tasgius rufipes* [*Astrapaeus rufipes* Lat.]. **Plate 443** [1 March 1833]: *Callicerus spencii* [*Callicerus obscurus* Grav.]. **Plate 446** [1 April 1833]: *Carabus exasperatus* [*Carabus violaceus* Linn.]. **Plate 450** [1 May 1833]: *Aspidiphorus orbiculatus* [*Nitidula orbiculata* Gyl.]. **Plate 454** [1 June 1833]: *Drypta emarginata* [*Drypta emarginata* Fab.]. **Plate 458** [1 July 1833]: *Harpalus ruficeps* [*Carabus ruficornis* Linn.]. **Plate 462** [1 August 1833]: *Falagria thoracica* [*Staphylinus sulcatus* Oliv.]. **Plate 466** [1 September 1833]: *Elophorus fennicus* [*Silpha aquatica* Linn.]. **Plate 470** [1 October 1833]: *Hister quadrimaculatus* [*Hister unicolor* Linn.]. **Plate 474** [1 November 1833]: *Hallomenus flexuosus* [*Dircaea humeralis* Fab.]. **Plate 478** [1 December 1833]: *Synodendron cylindricum* [*Scarabaeus cylindricus* Linn.]. **Plate 483** [1 January 1834]: *Mordella abdominalis* [*Mordella fasciata* Fab.]. **Plate 486** [1 February 1834]: *Macrocnema unimaculata* [*Chrysomela hyoscyami* Linn.]. **Plate 490** [1 March 1834]: *Lucanus cervus* [*Lucanus cervus* Linn.]. **Plate 494** [1 April 1834]: *Donacia typhae* [*Donacia crassipes* Fab.]. **Plate 498** [1 May 1834]: *Tritoma bipustulatum* [–]. **Plate 502** [1 June 1834]: *Mycetaea hirta* [*Silpha hirta* Mars.]. **Plate 506** [1 July 1834]: *Helodes beccabungae* [*Chrysomela phellandrii* Linn.]. **Plate 510** [1 August 1834]: *Cucujus spartii* [*Cantharis sanguinolenta* Linn.]. **Plate 514** [1 September 1834]: *Homalota dimidiata* [*Aleochara plana* Gyll.]. **Plate 518** [1 October 1834]: *Sphaeridium quadrimaculatum* [*Dermestes scarabaeoides* Linn.]. **Plate 522** [1 November 1834]: *Hylesinus scaber* [*Hylesinus crenatus* Fab.]. **Plate 526** [1 December 1834]: *Anisoplia suturalis* [*Scarabaeus horticola* Linn.]. **Plate 531** [1 January 1835]: *Hygrotus decoratus* [*Dytiscus inaequalis* Fab.]. **Plate 534** [1 February 1835]: *Emus hirtus* [*Staphylinus hirtus* Linn.]. **Plate 538** [1 March 1835]: *Nothus bipunctatus* [*Cantharis bipunctatus* Fab.]. **Plate 542** [1 April 1835]: *Lixus angustatus* [*Curculio angustatus* Fab.]. **Plate 546** [1 May 1835]: *Antherophagus similis* [*Tenebrio pallens* Linn.]. **Plate 550** [1 June 1835]: *Acalles roboris* [*Curculio ptinoides* Marsh.]. **Plate 554** [1 July 1835]: *Brachinus sclopeta* [*Carabus crepitans* Linn.]. **Plate 558** [1 August 1835]: *Pachyrhinus comari* [*Curculio comari* Herbst]. **Plate 562** [1 September 1835]: *Anthonomus pomorum* [*Curculio pomorum* Linn.]. **Plate 566** [1 October 1835]: *Catops dissimulator* [*Helops chrysomeloides* Panz.]. **Plate 570** [1 November 1835]: *Endomychus coccineus* [*Chrysomela coccinea* Linn.]. **Plate 574** [1 December 1835]: *Trox sabulosus* [*Scarabaeus sabulosus* Linn.]. **Plate 579** [1 January 1836]: *Rhyzophagus bipustulatus* [*Lyctus ferrugineus* Payk.]. **Plate 582** [1 February 1836]: *Clytra tridentata* [*Chrysomela 4-punctata* Linn.]. **Plate 586** [1 March 1836]: *Bolitophagus agricola* [*Bolitophagus agricola* Fab.]. **Plate 590** [1 April 1836]: *Pyrochroa coccinea* [*Pyrochroa rubens* Fab.]. **Plate 594** [1 May 1836]: *Cistela ceramboides* [*Chrysomela ceramboides* Linn.]. **Plate 598** [1 June 1836]: *Lagria hirta* [*Chrysomela hirta* Linn.]. **Plate 602** [1 July 1836]: *Elodes pini* [*Cyphon lividus* Fab.]. **Plate 606** [1 August 1836]: *Paramecosoma bicolor* [*Prionophorus bicolor* Curt.]. **Plate 610** [1 September 1836]: *Philonthus marginatus* [*Staphylinus splendens* Fab.]. **Plate 614** [1 October 1836]: *Holoparamecus depressus* [*Hydroporus depressus* Curt.]. **Plate 618** [1 November 1836]: *Byturus tomentosus* [*Dermestes tomentosus* Fab.]. **Plate 622** [1 December 1836]: *Omophlus armeriae* [*Cistela picipes* Fab.]. **Plate 627** [1 January 1837]: *Gymnaetron graminis* [*Curculio beccabungae* Linn.]. **Plate 630** [1 February 1837]: *Altica ochripes* [*Chrysomela nemorum* Linn.]. **Plate 634** [1 March 1837]: *Erirhinus aethiops* [*Curculio acridulus* Linn.]. **Plate 638** [1 April 1837]: *Quedius lateralis* [*Staphylinus tristis* Grav.]. **Plate 642** [1 May 1837]: *Rhynchites similis* [*Curculio betulae* Linn.]. **Plate 646** [1 June 1837]: *Ptinus sexpunctatus* [*Ptinus fur* Linn.]. **Plate 650** [1 July 1837]: *Lathrobium terminatum* [*Staphylinus elongatus* Linn.]. **Plate 654** [1 August 1837]: *Hylecoetus dermestoides* [–]. **Plate 658** [1 September 1837]: *Cantharis vesicatoria* [*Meloe vesicatorius* Linn.]. **Plate 662** [1 October 1837]: *Sphaeriestes foveolatus* [*Dermestes ater* Payk.]. **Plate 666** [1 November 1837]: *Argutor longicollis* [*Carabus vernalis* Fab.]. **Plate 670** [1 December 1837]: *Ceutorhynchus geranii* [*Curculio geranii* Payk.]. **Plate 675** [1 January 1838]: *Nitidula colon* [*Silpha grisea* Linn.]. **Plate 678** [1 February 1838]: *Orchestes waltoni* [*Curculio alni* Linn.]. **Plate 682** [1 March 1838]: *Dermestes lardarius* [*Dermestes lardarius* Linn.]. **Plate 686** [1 April 1838]: *Trachys minuta* [*Buprestis minuta* Linn.]. **Plate 690** [1 May 1838]: *Otiorhynchus maurus* [*Curculio tenebricosus* Herb.]. **Plate 694** [1 June 1838]: *Elater aterrimus* [*Elater cupreus* Fab.]. **Plate 698** [1 July 1838]: *Lampyris noctiluca* [*Lampyris noctiluca* Linn.]. **Plate 702** [1 August 1838]: *Typhaea fumata* [*Dermestes fumatus* Linn.]. **Plate 706** [1 September 1838]: *Triplax aenea* [*Silpha russica* Linn.]. **Plate 710** [1 October 1838]: *Attelabus curculionoides* [*Attelabus curculionoides* Linn.]. **Plate 714** [1 November 1838]: *Anthicus tibialis* [*Meloe antherinus* Linn.]. **Plate 718** [1 December 1838]: *Phytosus spinifer* [*Phytosus spinifer* Rudd.]. **Plate 723** [1 January 1839]: *Platyrhinus latirostris* [*Anthribus latirostris* Fab.]. **Plate 726** [1 February 1839]: *Anthribus albinus* [*Curculio albinus* Linn.]. **Plate 730** [1 March 1839]: *Haliplus ferrugineus* [*Dytiscus ferrugineus* Payk.]. **Plate 734** [1 April 1839]: *Trogosita mauritanica* [*Tenebrio mauritanica* Linn.]. **Plate 738** [1 May 1839]: *Aromia moschata* [*Cerambyx moschatus* Linn.]. **Plate 742** [1 June 1839]: *Silpha opaca* [*Silpha obscura* Linn.]. **Plate 746** [1 July 1839]: *Prionus coriarius* [*Cerambyx coriarius* Linn.]. **Plate 750** [1 August 1839]: *Rhagium inquisitor* [*Leptura inquisitor* Linn.]. **Plate 754** [1 September 1839]: *Bruchus ater* [*Bruchus pisi* Linn.]. **Plate 758** [1 October 1839]: *Staphylinus pubescens* [*Staphylinus murinus* Linn.]. **Plate 762** [1 November 1839]: *Tachyporus littoreus* [*Tachyporus pubescens* Gyll.]. **Plate 766** [1 December 1839]: *Baris analis* [*Rhynchaenus artemisiae* Fab.]

### Appendix 4. List of new Coleoptera taxa described in ‘*Insectes recueillis en Afrique et en Amérique*’ (Palisot de Beauvois, 1805-1821)

**Livraison 1** [DP: 14 October 1805]: *Passalus pentaphyllus* (p. 2); *Lucanus sublaevis* (p. 3); *Scarabaeus diana* (p. 4); *Scarabaeus mucronatus* (p. 5); *Melasis picea* (p. 7); *Elater ramicornis* (p. 10); *Elater elegans* [unecessary replacement name for *Elater caecus* Fabricius] (p. 10); *Elater beniniensis* (p. 11); *Elater lepidotus* (p. 11); *Copris nitens* (p. 24); *Copris viridis* (p. 24). **Livr. 2** [DP: 31 December 1805]: *Copris albicornis* (p. 25); *Copris obtectus* (p. 25); *Cetonia taenia* (p. 28); *Cetonia stigma* (p. 28); *Cetonia viridi-cyanea* (p. 29); *Melolontha angustata* (p. 30). **Livr. 3** [DP: 31 May 1806]: *Scarabaeus owariensis* (p. 41); *Scarabaeus truncatus* (p. 41); *Scarabaeus quadrituberculatus* (p. 42); *Buprestis semistriata* (p. 43); *Buprestis trilineata* (p. 44); *Buprestis chrysochlora* (p. 44); *Buprestis rugosa* (p. 45). **Livr. 4** [DP: 15 June 1807]: *Trichius scaber* (p. 58); *Trichius variegatus* (p. 59); *Trichius seticollis* (p. 59); *Trichius squamiger* (p. 60). **Livr. 5** [DP: 30 September 1807]: *Scarabaeus quadri-foveatus* (p. 74); *Scarabaeus oblongus* (p. 74); *Scarabaeus nasicornis americanus*^142^ (p. 75). *Elater bi-foveatus* (p. 78); *Elater elongatus* (p. 78); *Elater subspinosus* (p. 78); *Elater castanei-thorax* (p. 79). **Livr. 6** [DP: 3 July 1809]: *Scarabaeus boscii* (p. 89); *Scarabaeus splendens* (p. 89); 3. *Scarabaeus ferrugineus* (p. 90); *Scarabaeus rubeolus* (p. 90); *Scarabaeus tumefactus* (p. 91); *Copris striatulus* (p. 92). **Livr. 7** [DP: 26 August 1811]: *Scarabaeus complanatus* (p. 102); *Scarabaeus bituberculatus* (p. 103); *Scarabaeus humeralis* (p. 103); *Scarabaeus dubius* (p. 104); *Scarabaeus antillarum* (p. 104); *Scarabaeus obtusus* (p. 104); *Scarabaeus elongatus* (p. 104); *Copris subaeneus* (p. 105); *Scarites marginatus* (p. 106); *Scarites depressus* (p. 106); *Scarites sublaevis* (p. 107); *Scarites georgiae* (p. 107); *Scarites quadrimaculatus* (p. 107); *Harpalus dubius* (p. 108); *Harpalus viridi-aeneus* (p. 108). **Livr. 8** [DP: 14 December 1812]: *Helops contractus* (p. 121); *Helops tenebrioïdes* (p. 121); *Helops taeniatus* (p. 121); *Helops americanus* (p. 122); *Helops quadricolor* (p. 123); *Helops beniniensis* (p. 124); *Trogossita* ? *angustata* (p. 125); *Trogossita marginata* (p. 125); *Trogossita maxillosa* (p. 125); *Trogossita sublaevis* (p. 126); *Trogossita mutica* (p. 126); *Trogossita depressior* (p. 126); *Trogossita quadridens* (p. 126); *Trogossita subnigra* (p. 127). **Livr. 9** [DP: 26 August 1817]: *Scarabaeus tityus* (p. 137); *Helops tristis* (p. 137); *Helops sinuatus* (p. 139); *Lagria testacea* (p. 141); *Lagria violacea* (p. 141); *Opatrum nebulosum* (p. 142); *Opatrum pubescens* (p. 142); *Opatrum beniniense* (p. 143); *Phalaria owariensis* (p. 144). **Livr. 10** [DP: 27 October 1817]: *Sphaeridium pallidum* (p. 157); *Sphaeridium crenatum* (p. 158); *Sphaeridium laeve* (p. 158); *Sphaeridium unistriatum* (p. 158); *Sphaeridium africanum* (p. 159); *Agathidium uniscoïdes* (p. 160); *Nitidula maculata* (p. 160); *Nitidula afra*^143^ (p. 161); *Nitidula dermestoïdes* (p. 161); *Helops caroliniensis* (p. 162); *Helops saperdoïdes* (p. 162); *Tenebrio sublaevis* (p. 163); *Tenebrio femoratus* (p. 163); *Tenebrio minimus* (p. 164); *Tenebrio elongatus* (p. 164). **Livr. 11** [DP: 9 November 1818]: *Melolontha* ? *paradoxa* (p. 173); *Trox ovatus* (p. 175); *Trox tuberculatus* (p. 175); *Trox scabrosus* (p. 175); *Trox unistriatus* (p. 175); *Trox serrulatus* (p. 176); *Hister cylindricus* (p. 178); *Hister 4-dentatus* (p. 178); *Hister complanatus* (p. 179); *Hister latipes* (p. 179); *Hister subhemisphaericus* (p. 180); *Hister interruptus* (p. 180); *Hister regularis* (p. 180); *Sinodendron americanum* (p. 192). **Livr. 12** [DP: 31 December 1818]: *Copris tricolor* (p. 197); *Copris carnifex* (p. 198).

### Appendix 5. List of Coleoptera illustrated on the plates of ‘*Voyage dans l’Amérique méridionale*’ (Blanchard, 1835–1847)

**Plate 1** [DP: 1835]: 1. *Megacephala bifasciata* Br.; 2. *Megacephala cruciata* Br.; 3. *Megacephala spixii* Br.; 4. *Oxycheila labiata* Br.; 5. *Cicindela 4-punctata* Br.; 6. *Cicindela speculifera* Br.; 7. *Cicindela ramosa* Br.; 8. *Cicindela intricata* Br.; 9. *Agra erythrocera* Br.; 10. *Agra klugii* Br. **Plate 2** [DP: 7 November 1836]: 1. *Feronia insignis* Br.; 2. *Feronia unistriata* Dejean; 3. *Feronia cancellata* Br.; 4. *Feronia nobilis* Br.; 5. *Feronia lateralis* Br.; 6. *Oodes pallipes* Br.; 7. *Calleida festiva* Br.; 8. *Calleida tristis* Br.; 9. *Lebia concinna* Br.; 10. *Cymindis aptinoides* Br. [= *Dromius aptinoides* in the text on p. 12]. **Plate 3** [DP: 1836]: 1. *Pogonus bicolor* Br.; 2. *Baripus rivalis* Germ.; 3. *Platynus elegans* Br.; 4. *Brachinus obliquus* Br. [= *Brachinus insignis* Br. in the text on p. 19]; 5. *Brachygnathus pyropterus* Br.; 6. *Chlaenius villosulus* Br.; 7. *Feronia currens* Br.; 8. *Pelecium violaceum* Br.; 9. *Scarites brevicornis* Br.; 10. *Clivina xanthopus* Br. **Plate 4** [DP: 3 October 1836]: 1. *Hydrophilus smaragdinus* Br.; 2. *Hydrophilus palpalis* Br.; 3. *Hydrophilus latus* Br.; 4. *Hydrophilus lepidus* Br.; 5. *Hydrophilus limbatus* Br.; 6. *Hispa dorsalis* Br.; 7. *Dyticus submarginatus* Br. [= *Dyticus glaucus* Br. in the text]; 8. *Hydroporus latipes* Br.; 9. *Gyrinus depressus* Br.; 10. *Gyrinus ellipticus* Br. **Plate 5** [DP: 1836]: 1. *Nitidula m-rubrum* Br. [= *Strongylus m-rubrum* in the text on p. 65]; 2. *Necydalis humeralis* Br. [= *Strongylus humeralis* in the text on p. 66]; 3. *Necrophorus didymus* Br.; 4. *Silpha discicollis* Br.; 5. *Silpha apicalis* Br.; 6. *Staphylinus auricomus* Br.; 7. *Scarites interruptus* Br.; 8. *Silpha chrysopterus* Br.; 9. *Lathrobium majus* Br. [= *Pinophilus majus* in the text on p. 86]; 10. *Sterculia fulgens* Br. [= *Sterculia splendens* Blanch. in the text on p. 83]. **Plate 6** [DP: 8 November 1841]: 1. *Clerus miniaceus* Blanch.; 2. *Cryptocephalus trifasciatus* Blanch. [= *Clerus triplagiatus* Blanch. in the text on p. 90]; 3. *Clerus nigriventris* Blanch.; 4. *Clerus minutus* Blanch.; 5. *Tillus abdominalis* Blanch.; 6. *Epiclines gayi* Guérin; 7. *Eurymetopum fulvipes* Blanch.; 8. *Enoplium maculatum* Blanch.; 9. *Enoplium cyaneo-maculatum* Blanch.; 10. *Dasytes atromaculatus* Blanch. **Plate 7** [DP: 8 November 1841]: 1. *Lamprocera flavo-fasciata* Blanch.; 2. *Lamprocera flavo-quadrata* Blanch.; 3. *Lucidota elongata* Blanch.; 4. *Vesta cincticollis* Blanch.; 5. *Vesta gratiosa* Blanch.; 6. *Megalophthalmus gentilis* Blanch.; 7. *Megalophthalmus obsoletus* Blanch.; 8. *Amydetes praeusta* Blanch.; 9. *Phengodes orbygnyi* Blanch.; 10. *Scyrtes ligneus* Blanch. **Plate 8** [DP: 8 November 1841]: 1. *Chelonarium haemorrhoum* Perty; 2. *Chelonarium ornatum* Klug; 3. *Pterotarsus rugosus* Blanch.; 4. *Galba fasciata* Blanch. [= *Galbodema fasciata* in the text on p. 146]; 5. *Alaus flammula* Blanch.; 6. *Hemirhipus fascicularis* Fab.; 7. *Physorhinus flaviceps* Perty; 8. *Monocrepidius flavo-vittatus* Blanch.; 9. *Aphanobius ruficollis* Blanch. [= *Hemicrepidus ruficollis* in the text on p. 132]; 10. *Cyathodera longicornis* Blanch. **Plate 9** [DP: 22 May 1841]: 1. *Buprestis rudicollis* Blanch.; 2. *Buprestis caesia* Blanch.; 3. *Coeculus americanus* Blanch.; 4. *Colobogaster resplendens* Blanch.; 5. *Chrysobothris denticollis* Blanch. [= *Chrysobothris emarginaticollis* Blanch. in the text on p. 148]; 6. *Chrysobothris myia* Gory; 7. *Ptosima planata* Lap. et Gory; 8. *Zemina orbignyi* Lap. et Gory; 9. *Zemina okea* Blanch.; 10. *Agrilus piscis* Blanch. **Plate 10** [DP: 8 November 1841]: 1. *Anomiopsis aelianus* Blanch.; 2. *Megathopa violacea* Blanch.; 3. *Megathopa auricollis* Blanch.; 4. *Hyboma cupreicolle* Blanch.; 5. *Coprobius gemmatus* Blanch. [= *Canthon gemmatum* Blanch in the text on p. 160]; 6. *Tetraaechma sanguineo-signata* Blanch. [= *Tetraaechma sanguineomaculata* Blanch. in the text on p. 168]; 7. *Phanaeus melibaeus* Blanch.; 8. *Onthophagus clypeatus* Blanch.; 9. *Athyreus fulvescens* Blanch.; 10. *Sphaerelytrus nigerrimus* Blanch. **Plate 11** [DP: 1842]: 1. *Cratocnemus niger* Blanch.; 2. *Megaceras rugosus* Blanch.; 3. *Coelosis hippocrates* Blanch.; 4. *Anomala ebenina* Blanch.; 5. *Cyclocephala erythrodera* Blanch.; 6. *Coccinella villosa* Blanch.; 7. *Rutela plicata* Gory [= *Rutela smaraglina* var. *plicata* in the text on p. 192]; 8. *Rutela elongata* Blanch.; 9. *Leucothyreus marginaticollis* Blanch.; 10. *Philochlaenia virescens* Blanch. **Plate 12** [DP: 17 June 1842]: 1. *Gymnetis flavo-marginata* Blanch.; 2. *Gymnetis touchardii* Blanch.; 3. *Gymnetis rufilatera* Gory et Perch. [= *Gymnetis rufilatris* in the text on p. 193]; 4. *Gymnetis albo-sparsa* Blanch.; 5. *Gymnetis pantherina* Blanch.; 6. *Gymnetis miniata* Blanch.; 7. *Orthognathus albo-fuscus* Blanch.; 8. *Lucanus caelatus* Blanch.; 9. *Lucanus rubro-vittatus* Blanch. [= *Lucanus vittatus* in the text on p. 194]; 10. *Lucanus cucullatus* Blanch. **Plate 13** [DP: 17 June 1842]: 1. *Geoborus costatus* Blanch.; 2. *Geoborus lividipennis* Blanch.; 3. *Cacicus americanus* Sol.; 4. *Emalodera crenaticostata* Blanch.; *5. Scotobius punctatellus* Blanch.; 6. *Scotobius pilularius* Germ. [= *Scotobius pillularius* in the text on p. 195]; 7. *Cerostena cribrata* Blanch.; 8. *Auladera gibba* Blanch.; 9. *Nyctelia latissima* Blanch.; 10. *Nyctelia plicata* Blanch. **Plate 14** [DP: 1842]: 1. *Nyctelia reticulata* Blanch.; 2. *Nemognatha immaculata* Blanch.; 3. *Nyctelia oblonga* Blanch.; 4. *Nyctelia decora* Burm. et Erichs.; 5. *Nyctelia elegans* Blanch.; 6. *Cosmonota angustata* Blanch.; 7. *Cerambyx unicolor* Blanch.; 8. *Heliophugus sulcatus* Guer.; 9. *Phobelius crenatus* Blanch.; 10. *Anaedus punctatissimus* Dej. **Plate 15** [DP: n.d.]^144^: 1. *Isotoma emarginaticollis* Blanch.; 2. *Statyra unicolor* Blanch.; 3. *Allecula pallida* Blanch.; 4. *Prostenus violaceus* Blanch.; 5. *Mordella luteoguttata* Blanch.; 6. *Meloe miniaceomaculata* Blanch.; 7. *Pyrota vittigera* Blanch.; 8. *Lytta rubriceps* Blanch.; 9. *Lytta nigropunctata* Blanch.; 10. *Nacerdes linearis* Blanch. **Plate 16** [DP: 1842]: 1. *Ptychoderes bicallosus* Blanch.; 2. *Stenocerus nigro-tessellatus* Blanch.; 3. *Arhenodes perforatus* Blanch.; 4. *Cyphus decimpunctatus* Fabr. var. [= *Cyphus sedecimpunctatus* Linn. in the text on p. 201]; 5. *Cydianerus chevrolatii* Blanch.; 6. *Oxyops signatus* Blanch.; 7. *Hadromerus aureus* Blanch.; 8. *Heilipus mixtus* Blanch.; 9. *Centrinus flavipennis* Blanch.; 10. *Centrinus semiluctuosus* Blanch. **Plate 17** [DP: 1843]: 1. *Pycnopus bufo* Say; 2. *Naupactus sulphureo-signatus* Blanch.; 3. *Naupactus glaucivittatus* Blanch.; 4. *Naupactus rubricollis* Blanch.; 5. *Phytonomus ochraceus* Blanch.; 6. *Sternechus costatus* Blanch. [= *Sternechus guerini* Schoenh.]; 7. *Baridius interpunctatus* Germ.; 8. *Baridius flexuosus* Blanch.; 9. *Diorymerus lancifer* Guer.; 10. *Ryssomatus polycoccus* Schoenh. **Plate 18** [DP: 1843]: 1. *Naupactus nodicollis* Schoenh.; 2. *Heilipus biplagiatus* Blanch.; 3. *Baridius quadrinotatus* Blanch.; 4. *Sipalus sphacelatus* Schoenh.; 5. *Serropalpus striatus* Schoenh.; 6. *Sipalus luteosignatus* Blanc.; 7. *Rhyncophorus nitidus* Schoenh. [= *Rhyncophorus nitidipennis* Schoenh. in the text on p. 204]; 8. *Sphaenophorus crassus* Blanc.; 9. *Sphaenophorus rubro-tessellatus* Blanch.; 10. *Cossonus bipunctatus* Blanch. **Plate 19** [DP: 1842]: 1. *Phloeotrupes coelatus* Blanch.; 2. *Apate fossulata* Blanch.; 3. *Apate serrata* Blanch.; 4. *Psoa rufipes* Blanch.; 5. *Psoa gracilis* Blanch.; 6. *Trogossita colossus* Lepel. et Serv.; 7. *Trogossita ebenina* Blanch.; 8. *Trogossita fulgidivittata* Blanch.; 9. *Passandra rubrolineata* Blanch.; 10. *Passandra concolor* Blanch. **Plate 20** [DP: 1841]: 1. *Sypilus orbignyi* Guer.; 2. *Anoploderma bicolor* Guer.; 3. *Torneutes pallidipennis* ♂ Reich.; 4. *Torneutes pallidipennis* ♀ Reich.; 5. *Navosoma triste* Blanch.; 6. *Macrotoma melitae-eques* Blanch.; 7. *Cheloderus childreni* Gray; 8. *Poecilopeplus intricatus* Blanch. [= *Poecilosoma intricatus* in the text on p. 207]; 9. *Poecilosoma rufipenne* Dej. **Plate 21** [DP: 17 June 1842]: 1. *Pteroplatus annulipes* Blanch.; 2. *Eriosoma lanaris* Blanch.; 3. *Coccoderus tuberculatus* Buquet; 4. *Coccoderus tristis* Blanch.; 5. *Eburia speciosa* Blanch.; 6. *Eburia vittata* Blanch.; 7. *Eburia formosa* Blanch.; 8. *Eburia gratiosa*; 9. *Tricophorus interogationis* Bl. [*Trichophorus interrogationis* in the text on p. 208]; 10. *Phymatioderus bizonatus* Bl. **Plate 22** [DP: 1842]: 1. *Orion lachesis* Blanch.; 2. *Criodion eburioides* Blanch.; 3. *Grammicosomum flavofasciatum* Blanch.; 4. *Miopteryx spiniger* Blanch.; 5. *Cosmisoma formosa* Blanch.; 6. *Trypanidius andicola* Dej.; 7. *Hypsioma gemmata* Blanch.; 8. *Compsosoma concretum* Dej.; 9. *Hoplistocerus refulgens* Blanch.; 10. *Hemilophus spectabilis* Blanch. **Plate 23** [DP: 1842]: 1. *Megalopus atrofasciatus* Blanch.; 2. *Megalopus erythrosoma* Blanch.; 3. *Megalopus aurantiacus* Blanch.; 4. *Megalopus terminalis* Blanch.; 5. *Alurnus quadrimaculatus* Guer.; 6. *Alurnus d’orbignyi* Guer. [= *Alurnus orbignyi* in the text on p. 211]; 7. *Amphicoma apicalis* Guer.; 8. *Scelaenopla angusticostata* Blanch.; 9. *Odontota nigriceps* Blanch.; 10. *Odontota crenata* Blanch. **Plate 24** [DP: 1842]: 1. *Cephaloleia miniacea* Blanch.; 2. *Omocera aureicornis* Blanch.; 3. *Discomorpha tenebrosa* Blanch.; 4. *Cyrtonota pallidoguttata* Blanch.; 5. *Chelymorpha pantherina* Blanch.; 6. *Dorynota parallela* Blanch.; 7. *Deloyala sanguinolenta* Blanch.; 8. *Caelomera azureofusciata* Blanch.; 9. *Platynocera murina* Blanch.; 10. *Colaspis acuminipennis* Blanch. **Plate 25** [DP: n.d.]^140^: 1. *Doryphora aurantiaco-maculata* Blanch.; 2. *Doryphora margaritae* Blanch.; 3. *Doryphora flavo-zonata* Blanch.; 4. *Doryphora interrupta* Blanch.; 5. *Doryphora glaucivittata* Blanch.; 6. *Chlamys ruficollis* Blanch.; 7. *Erotylus hexagrammus* Lacord.; 8. *Erotylus multiguttatus* Lacord.; 9. *Brachyphaenus perlepidus* Lacord. [= *Brachysphaenus perlepidus* in the text on p. 214]; 10. *Epilachna v-pallidum* Blanch.

### Appendix 6. List of Coleoptera illustrated on the plates of ‘*Entomographia imperii Russici… Volumen I*’ (Fischer von Waldheim, 1820–1822)

All the plates should be dated 1820.

**Plate 1**: 1. *Cicindela lunulata* Ol[iver]; 2. *Cicindela chiloleuca* m[ihi]; 3. *Cicindela tricolor* Ad[ams]; 4. *Cicindela violacea* F[abricius]; 5. *Cicindela gracilis* Pall[as]; 6. *Cicindela fischeri* Ad[ams]; 7. Cicindela var. hybridae. **Plate 2**: *Carabus tauricus* Pall[as]; *Carabus scabrosus* Oliv[er]. **Plate 3**: 2. *Carabus puschkini* Ad[ams]; 3. *Carabus gebleri* [Fischer]; 4. *Carabus drescheri* [Fischer]; 5. *Carabus henningii* [Fischer]. **Plate 4**: 6. *Carabus maurus* Ad[ams]; 7. *Carabus excellens* F[abricius]; 8. *Carabus schönherri* [Fischer]; 9. *Carabus kruberi* [Fischer]; 10. *Carabus latreille* [Fischer]. **Plate 5**: 1. *Ditylus helopioides* [Fischer]; 2. *Ditylus rufus* Fischer; *Pedilus fuscus* Fischer. **Plate 6**: 1. *Nebria metallica* Eschscholtz; 2. *Nebria gregaria* Eschosch[oltz]; 3. *Nebria catenulata* Gebler; 4. *Nebria brevicollis* Latreille; 5-6. *Nebria livida* Latr[eille]. **Plate 7**: 1. *Cychrus marginatus* Esch[scholtz]; 2. *Cychrus rostratus* F[abricius]; *Callisthenes panderi* Fisch[er]; 11. *Carabus baccivorus* Esch[scholz]; 12. *Carabus chamissonis* Esch[scholz]. **Plate 8**: 13. *Carabus cribellatus* Ad[ams]; 14. *Carabus scrobiculatus* Ad[ams]; 15. *Carabus cribratus* Boeb[er]; 16. *Carabus perforatus* Fisch[er]; 17. *Carabus exaratus* Stev[en]; 18. *Carabus variolosus* Fisch[er]. **Plate 9**: 19. *Carabus vietinghovii* Ad[ams]; 20. *Carabus halysidotus* Ill[iger]; 21. *Carabus regalis* Böb[er]; 22. *Carabus cuprinus* B[öber]; 23. *Carabus aeruginosus* B[öber]; 24. *Carabus aereus* B[öber]. **Plate 10**: 25. *Carabus conciliator* [Fischer]; 26. *Carabus maeander* [Fischer]; 27. *Carabus chrysochloris* Fisch[er]; 28. *Carabus campestris* Stev[en]; 29. *Carabus sibiricus* Böb[er]; 30. *Carabus böberi* Ad[ams]. **Plate 11**: 31. *Carabus estreicheri* Bess[er]; 32. *Carabus goldegkii* Meg[erle]; 33-35. *Carabus* [*goldegkii*] var Ej.; 36. *Carabus besseri* Ziegl[er]; 37. *Carabus erythropus* Ziegl[er]. **Plate 12**: 1. *Cymindis lateralis* [Fischer]; 2. *Cymindis binotata* [Fischer]; 3. *Cymindis fusula* [Fischer]; 4. *Cymindis vittata* [Fischer]; 1. *Anomoeus dorsalis* [Fischer]; 2. *Anomoeus cruciatus* Fisch[er]; 1. *Zuphium olens* [Latreille]; 2. *Zuphium fasciolatum* Latr[eille]. **Plate 13**: 1. *Lethrus cephalotes* F[abricius]; 2. *Lethrus scoparius* m[ihi]; 3. *Lethrus longimanus* [Fischer]; 1-2. *Ateuchus tmolus* m[ihi]; 3. *Ateuchus volvens* F[abricius] [*Ateuchus geoffroyi* Fisch[er] in the text on p. 204]; 4. *Ateuchus flagellatus* [Fabricius]; 4. *Ateuchus serratus* m[ihi]. **Plate 14**: 1. *Pimelia gigantea* [Fischer]; 2. *Pimelia verrucosa* [Fischer]; 3. *Pimelia nodosa* [Fischer]; 4. *Pimelia imbricata* [Fischer]; 5. *Pimelia hirta* [Fischer]; 6. *Pimelia anomala* [Fischer]; 7. *Diesia quadridentata* [Fischer]; 8. *Diesia sexdentata* [Fischer]. **Plate 15**: 1. *Platyope granulata* [Fabricius]; 2. *Platyope leucographa* [Pallas]; 3. *Platyope proctoleuca* m[ihi]; 4. *Pimelia cephalotes* Pall[as]; 5. *Pimelia subglobosa* F[abricius]; 6. *Hedyphanes coerulescens* Fisch[er]; 7. *Akis acuminata* F[ischer]; 8. *Akis limbata* m[ihi]; 9. *Akis gibba* m[ihi] (9). **Plate 16**: 1. *Blaps gigas* m[ihi] F[ischer]; 2. *Blaps seriata* m[ihi]; 3. *Blaps fatidica* Cr[eutzer]; 4. *Blaps acuminata* Gebl[er]; 5. *Blaps attenuata* m[ihi]; 6. *Blaps marginata* m[ihi]; 7. *Blaps halophila* m[ihi]; 8. *Tagona acuminata* [Fischer]; 9. *Tagona macrophthalmus* Fisch[er]. **Plate 17**: 3. *Cincindela* α. β. γ. varietas tricoloris [Adams]; 7. *Cicindela distans* m[ihi]; 8. *Cicindela sinuata* Schn[eid]; 9. *Cicindela lactea* ? Pall[as]; 10. *Cicindela zwickii* m[ihi].

### Appendix 7. List of Coleoptera illustrated on the plates of ‘*Entomographia imperii Russici… Volumen II*’ (Fischer von Waldheim, 1823–1824)

All the plates should be dated 1823.

**Plate 18**: 1-2. *Ceratophyus dispar* F.; 3-4. *Ceratophyus fischeri* Zwick; 5. *Odontaeus mobilicornis* Meg. **Plate 19**: 1. *Pterostichus adstrictus* Eschsch.; 2. *Agonum molle* Esch.; 3. *Agonum elongatum* Dejean; 4. *Platysma fossifrons* Esch.; 5. *Platysma foveocollis* Esch.; 6. *Poecilus ventricosus* Esch.; 7. *Poecilus pinguedinis* E.; 8. *Poecilus rugosus* Gebl.; 9. *Poecilus lepidus* Bess. **Plate 20**: 1. *Platyscelis hypolithus* Pall.; 2-3. *Platyscelis melas* m[ihi]; 4. *Platyscelis rugifrons* Gebl.; 5. *Platyscelis gages* m[ihi]; 6. *Erodius ferrugineus*; 7. *Erodius pygmaeus* m[ihi]; 8. *Gnathosia glabra* m[ihi]. **Plate 21**: 1. *Buprestis variolaris* Pall.; 2. *Buprestis exarata* m[ihi]; 3. *Buprestis diadema* m[ihi]; 4. *Buprestis sulcata* m[ihi]; 5. *Buprestis acuminata* Pall.; 6. *Buprestis alni* Meg.; 7. *Buprestis tenebrionis* F.; 8. *Buprestis sibirica* F.; 9. *Buprestis flavomacutata* F. **Plate 22**: 1. *Pytho depressus* F.; 2. *Phaleria bicolor* Stev. [*Phaleria chrysomelina* Rossi in the text on p. 199]; 3. *Uloma cornuta* Meg.; 4. *Helops arboreus* Stev.; 5. *Helops gracilis* Ziegl.; 6. *Steropus caspius* Stev.; 7. *Pelmatopus hummellii* Fisch.; 8. *Laena pulchella* Ziegl. **Plate 23**: 1. *Elater cruciatus* F.; 2. *Elater depressus* Meg.; 3. *Elater volhynensis* Ziegl. [*Elater volhyniensis* Ziegler in the text on p. 202]; 4. *Elater volgensis* m[ihi]; 5–7. *Elater quadripustulatus* m[ihi]; 8. *Elater laticollis* m[ihi]; 9. *Elater sericeus* Gebl. **Plate 24**: 1. *Elater erythropus* m[ihi]; 2. *Elater humeralis* m[ihi]; 3. *Elater scrutator* Gyll.; 4. *Elater trifasciatus* F.; 5. *Elater vittatus* F.; 6. *Elater strictus* m[ihi]; 7-8. *Campylus mesomelas*; 9. *Campylus denticollis*. **Plate 25**: 1. *Hister quadrimaculatus* F.; 2. *Hister distans* m[ihi]; 3. *Hister scapularis* m[ihi] [*Hister humeralis* Fischer in the text on p. 205]; 4. *Hister sinuatus* F.; 5. *Hister personatus* m[ihi]; 6. *Hister fimetarius* F.; 7. *Hister interruptus* Payk.; 8. *Hister biguttatus* Stev.; 9. *Hister externus* m[ihi]. **Plate 26**: 1. *Cephalotes vulgaris* Bon.; 2. *Cephalotes pictus* m[ihi] [*Daptus pictus* Fischer in the text on p. 36]; 3. *Scarites laevigatus* F. [*Scarites volgensis* Steven in the text on p. 31]; 4. *Scarites interruptus* m[ihi]; 5. *Clivina thoracica* F.; 6–7. *Clivina arenaria* F.; 8. *Clivina gibba* F.; 9. *Ophonus discophorus* m[ihi]. **Plate 27**: 1. *Sisyphus schäfferi* F.; 2. *Sisyphus tauscheri* m[ihi]; 3. *Ateuchus sacer* F.; 4. *Ateuchus typhon* m[ihi]; 5. *Ateuchus infirmus* m[ihi]; 6. *Ateuchus laticollis* F. [*Ateuchus nudifrons* Fischer in the text on p. 211]. **Plate 28**: 1–2. *Melolontha fullo* F.; 3. *Melolontha hololeuca* Pall.; 4. *Melolontha anketeri* H.; 5. *Melolontha volgensis* m[ihi]; 6. *Melolontha henningii* Gebl.; 7. *Melolontha ochracea* Kn.; 8. *Melolontha porosa* m[ihi]; 9. *Melolontha pilosa* F. **Plate 29**: 1. *Carabus obsoletus*; 2. *Carabus concretus*; 3. *Carabus haeres* Fischer; 4. *Carabus mingens* F.; 5. *Carabus gastridulus* Fischer; 6. *Carabus leachii* Gebl.; 7. *Carabus modestus* Fischer. **Plate 30**: 1. *Procerus dejeanii* Steven; 2. *Cechenus fischeri*; 3. *Cechenus regularis* Stev. **Plate 31**: 1. *Gematis nigrifrons* Stev.; 2. *Gematis obscura* F.; 3. *Gematis oblonga* F.; 4. *Anisoplia agricola* F.; 5. *Anisoplia lineolata* Dejean; 6. *Anisoplia zwickii* Fischer; 7. *Anisoplia deserticola* Fischer; 8. *Anisoplia horticola* F. **Plate 32**: *Stomphax crucirostris* m[ihi]; *Platycerus caraboides*; *Platycerus rufipes* F.; *Lucanus parallelipipedus* F. **Plate 33**: 1. *Plectes iberus* Stev.; 2. *Plectes nothus* Ad.; 3. *Plectes osseticus* Ad.; 4. *Plectes deplanatus* Stev. **Plate 34**: 1. *Carabus maeoticus* Stev. [*Carabus maeoris* Steven in the text on pp. 86 and 218]; 2. *Carabus calleyi* Fisch.; 3. *Carabus bessarabicus* Stev.; 4. *Carabus striolatus* St.; 5. *Carabus errans* St.; 6. *Carabus bosphoranus* St. **Plate 35**: 1. *Carabus varians* Stev.; 2. *Carabus semistriatus* Stev.; 3. *Carabus cumanus* St.; 4. *Carabus decolor* St.; 5. *Carabus smaragdinus* Fisch.; 6. *Carabus adoxus* St.; 7. *Carabus eremita*; 8. *Carabus hummelii*; 9. *Carabus spasskianus* Fr. **Plate 36**: 1. *Sphodrus longicollis* Stev.; 2. *Sphodrus cimmerius* Stev.; 3. *Sphodrus terricola* Oliv.; 4. *Sphodrus tilesii* Böb.; 5. *Sphodrus angusticollis* m[ihi]; 6. *Sphodrus dauricus* m[ihi]; 7. *Sphodrus inaequalis*; 8. *Sphodrus rufitarsis* m[ihi]; 9. *Sphodrus gigas* m[ihi]. **Plate 37**: 1. *Cymindis homagrica* Duft.; 2. *Cymindis lineata* Stev.; 3. *Cymindis palliata* Stev.; 4. *Cymindis ornata* Stev.; 5. *Cymindis bivittata* Stev.; 6. *Cymindis axillaris* Duft.; 7. *Cymindis humeralis*; 8. *Myosodus ordinatus* St.; 9. *Myosodus regularis* St. **Plate 38**: 1. *Ctenopus melanogaster* Fischer; 2. *Rhipiphorus biguttatus* F. [*Rhipiphorus bimaculatus* F. in the text on p. 222]; 3. *Mordella variegata* F. [*Mordella fasciata* Fabrici. in the text on p. 222]; 4. *Mordella atomaria* F.; 5. *Anaspis frontalis* F.; 6. *Anaspis lateralis* F.; 7. *Anaspis thoracica* F.; 8. *Anaspis flava* L.; 9. *Pelecotoma latreillei* Fischer. **Plate 39**: 11. *Cicindela germanica* F.; 12. *Cicindela angustata* m[ihi]; 13. *Cicindela leucophthalma* m[ihi]; 14. *Cicindela soluta* Ziegl.; 15. *Cicindela elegans* m[ihi]; 16. *Cicindela maritima* Dej. **Plate 40**: 1. *Mylabris speciosa* Pall.; 2. *Mylabris fasciato-punctata* Ad. [*Mylabris adamsii* in the text on p.224]; 3. *Mylabris melanura* P.; 4. *Mylabris sibirica* Gebl.; 5. *Mylabris maculata* Billb.; 6. *Mylabris rufipes* Bess.; 7. *Mylabris frohlovii* G. [*Mylabris frolovii* Gebler in the text on p. 226]; 8. *Mylabris bivulnera* F.; 9. *Mylabris quadrisignata* G.; 10. *Mylabris marginata* m[ihi] [*Mylabris confluens* Fischer in the text on p. 227]. **Plate 41**: 1-2. *Cerocoma schaefferi* F. [*Cerocoma schreberi* Fabricius in the text on p. 227]; 3–4. *Cerocoma stevenii* m[ihi]; 5–6. *Lydus trimaculatus* F.; 7–8. *Lydus quadrisignatus* m[ihi]. **Plate 42**: 1. *Lytta myagri* Z.; 2–3. *Lytta syriaca* F.; 4. *Lytta chalybea* F.; 5. *Lytta collaris* Ol.; 6. *Lytta megalocephala* B.; 7. *Lytta erthrocephala* B.; 8–9. *Lytta dubia* Ol. **Plate 43**: 1. *Lytta bicolor* m[ihi] [*Lytta dichroa* Fischer in the text on p. 230]; 2–3. *Lytta vesicatoria* F.; 4-7. *Lytta fischeri* Gebl. **Plate 44**: 1. *Cleonis granulata*; 2. *Cleonis marginata*; 3. *Cleonis leucoptera*; 4. *Cleonis foveolata*; 5. *Cleonis hololeuca* Pall.; 6. *Cleonis leucophylla*; 7. *Cleonis frontata*; 8. *Cleonis fossulata*; 9. *Cleonis tricarinata*. **Plate 45**: 1. *Carabus aemulus* m[ihi]; 2. *Carabus cyaneus* F.; 3. *Carabus loschnikowii* Gebl.; 4–5. *Carabus scabriusculus* Ol.; 6. *Carabus linnaei* Meg.; 7. *Carabus candisatus* Duft.; 8. *Carabus andrzejuscii* m[ihi] [*Carabus andrejovskii* Fischer in the text on p. 234 but *andrzejuscii* on p. 99]. **Plate 46**: 1. *Cychrus torulosus* Fisch.; 2. *Cychrus angusticollis* Esch.; 3. *Cychrus aenus* Stev.; 4. *Cychrus attenuatus* F.; 5. *Carabus imperialis* Gebler; 6. *Daptus pictus* Fisch.; 7. *Daptus vittatus* Gebl.; 8. *Daptus chloroticus* Gebler. **Plate 47**: 1. *Rhaebus gebleri* Fisch.; 2. *Donacia macrocnemia* Fischer; 3. *Rhyssodes bicolor* Fischer [*Argopus bicolor* Fischer in the text on pp. 183 and 235]; 4. *Ryssodes nigritarsis* Gebler [*Argopus nigritarsis* Gebler in the text on pp. 185 and 235]. **Plate 48**: 1. *Cerambyx moschatus* F.; 2. *Cerambyx ambrosiacus* Böb.; 3–4. *Cerambyx thoracius* Fisch.; 5. *Cerambyx chlorophanus* Fisch.; 6. *Callichroma alpinum* F. **Plate 49**: 1. *Purpuricenus koehleri* F.; 2. *Purpuricenus wredii* Fisch.; 3–4. *Purpuricenus ephippium* Steven; 5–6. *Purpuricenus elaeagni* Stev.; 7–8. *Purpuricenus halodendri* Pall. **Plate 50**: 1–2. *Dorcadion glycyrrhizae* Pall.; 3–4. *Dorcadion politum* Böb.; 5. *Dorcadion morio* F.; 6. *Dorcadion pigrum* Schön.; 7. *Dorcadion erythropterum* Fisch. [*Dorcadion erythropteron* Fischer in the text on p. 240]; 8. *Dorcadion involvens* Fisch.; 9–10. *Dorcadion humerale* Gebl.

### Appendix 8. List of Coleoptera illustrated on the plates of ‘*Voyage autour du monde...* La Coquille, *pendant les années 1822, 1823, 1824 et 1825....*’ (Guérin-Méneville, 1830–1831)

**Plate 1** [DP: 1 September 1830]: 1. *Cicindela decem-guttata* Fabr.; 2. *Cicindela urvillii* Guér. [spelled *durvillei* in the text on p. 58]; 3. *Cicindela tortuosa* Dej.; 4. *Cicindela tuberculata* Fabr.; 5. *Cicindela latreillei* Guér.; 6. *Therates basalis* d’Urv.; 7. *Cymindis australis* Dej.; 8. *Lebia posticalis* Guér.; 9. *Antarctia blanda* Dej.; 10. *Antarctia nitida* Guér.; 11. *Geobaenus australasiae* Guér.; 12. *Acupalpus piceus* Guér.; 13. *Antarctia flavipes* Dej.; 14. *Antarctia malachitica* Dej.; 15. *Trechus soledadinus* Guér.; 16. *Trechus audouinii* Guér.; 17. *Leia jacksoniensis* Guér. [Bembidium (Leia) jacksoniensis in the text on p. 61]; 18. *Dytiscus flavocinctus* Guér.; 19. *Colymbetes lineatus* Guér.; 20. *Gyrinus striolatus* Guér.; 21. *Staphilinus fulgipennis* Guér. [*Staphylinus apicicornis* Guér. in the text on p. 62]; 22. *Paederus cyanipennis* Guér.; 23. *Paederus australis* Guér.; 24. *Aleochara haemorhoïdalis* Guér. [spelled *haemorrhoïdalis* in the text on p. 63]. **Plate 2** [DP: 25 November 1830]: 1. *Buprestis bella* Guér.; 2. *Buprestis auro-foveatus* Guér.; 3. *Galba marmorata* Guér. [*Pterotarsus marmoratus* Guér. in the text on p. 68]; 4. *Callirhypis dejeanii* Latr.; 5. *Telephorus dilaticornis* Guér.; 6. *Cordylocera antennata* Guér. [*Tylocerus antennatus* Guér. in the text on p. 77]; 7. *Adelocera grisea* Guér. [*Adelocera caliginosa* Guér. in the text on p. 68]; 8. *Cladophorus ruficollis* Guér.; 9. *Cladophorus dimidiatus* Guér.; 10. *Laius cyaneus* Guér. **Plate 3** [DP: 7 March 1831]: 1. *Scarabaeus latipes* Guér.; 2. *Scarabaeus aegeon* Fabr.; 3. *Oryctomorphus bimaculatus* Guér.; 4. *Brachysternus prasinus* Guér.; 5. *Serica marmorea* Guér. [*Pseudoserica marmorea* Guér. in the text on p. 87]; 6. *Liogenys castaneus* Guér.; 7. *Ceraspis peruvianus* Guér.; 8. *Pachycerus castaneipennis* Guér. [*Hadrocerus castaneipennis* Guér. in the text on p. 83]; 9. *Heteronyx australis* Guér.; 10. *Liparetrus discipennis* Guér.; 11. *Cetonia papua* Guér.; 12. *Cetonia taciturna* Guér. **Plate 4** [DP: 25 July 1831]: 1. *Proacis rufipes* Esch.; 2. *Nyctozoilus obesus* Guér.; 3. *Nycterinus thoracicus* Esch.; 4. *Ammophorus peruvianus* Guér.; 5. *Tenebrio costatus* Guér.; 6. *Heliofugus arenosus* Guér.; 7. *Nyctopetus tenebrioïdes* Guér.; 8. *Psammeticus costatus* Guér.; 9. *Phitophilus helopioïdes* Guér. [*Phytophilus helopioïdes* in the text on p. 100]; 10. *Opatrum complanatum* Guér.; 11. *Adelium dilaticollis* Guér.; 12. *Blapstinus striatus* Guér. [*Opatrinus striatus* Guér. in the text on p. 99]. **Plate 5** [DP: 15 June 1831]: 1. *Amarygmus mutabilis* Guér.; 2. *Amarygmus cupreus* Guér.; 3. *Helops coerulescens* Guér.; 4. *Cyphonotus dromedarius* Guér. [*Cyphonotus dromadarius* in the text on p. 103]; 5. *Lagria pulchella* Guér.; 6. *Lagria castanea* Guér.; 7. *Helaeus ovatus* Guér.; 8. *Notoxus quadrimaculatus* Guér.; 9. *Oedemera sericea* Guér. [*Oedemera livida* Oliv. in the text on p. 107]; 10. *Cistela pilosula* Guér.; 11. *Cistela luteola* Guér.; 12. *Meloe chiliensis* Guér. **Plate 6** [DP: 7 March 1831]: 1. *Aterpus superciliosus* Latr.; 2. *Geonemus striato-punctatus* Guér.; 3. *Geonemus geoffroyii* Guér. [*Eupholuse geoffroyi* in the text on p. 115]; 4. *Geonemus cuvierii* Guér. [*Eupholus cuvierii* in the text on p. 118]; 5. *Cryptorhynchus striga* Latr. [*Arachnopus striga* Guér. in the text on p. 128]; 6–7. *Rhynolaccus formicarius* Latr.^145^; 8. *Cryptorhynchus humeralis* Latr. [*Tylodes humeralis* Guér. in the text on p. 124]; 9. *Cryptorhynchus lateralis* Guér. [*Tylodes lateralis* in the text on p. 126]; 10. *Pachyrhynchus aeneus* Guér.; 11. *Brenthus bicolor* Guér.; 12. *Brenthus angustatus* Guér. [*Leptorhynchus angustatus* in the text on p. 111]; 13. Tête du *Brenthus nova-guineensis* Guér. **Plate 7** [DP: 13 October 1831]: 1. *Prionus limae* Guér.; 2. *Saperda lefebvrii* Guér.; 3. *Tragocerus bifasciatus* Guér.; 4. *Lamia rubropunctata* Guér. [*Saperda rubro-punctata* in the text on p. 137]; 5. *Saperda venusta* Guér.; 6. *Lamia orbignyi* Guér.; 7. *Lamia albicincta* Guér. [*Saperda albo-cincta* in the text on p. 137]; 8. *Lamia granulosa* Guér.; 9. *Lamia rugosula* Guér.; 10. *Gnoma affinis* Guér.; 11. *Gnoma giraffa* Guér.; 12. *Tmesisternus trivittatus* Guér. [*Tmesisternus trivitatus* in the text on p. 130]; 13. *Tmesisternus marmoratus* Guér.

### Appendix 9. List of Coleoptera illustrated on the plates of ‘*Exploration scientifique de l’Algérie… Histoire naturelle des animaux articulés. Deuxième partie*’ (Lucas, 1846–1849)

**Plate 1** [DP: 28 March–15 August 1846]: 1. *Cicindela maroccana* Fabr.; 2. *Cicindela sardoa* Dej.; 3. *Cicindela littoralis* Fabr.; 4. *Cicindela ritchii* Vig.; 5. *Cicindela nitidula* Dej.; 7. *Cymindis dilaticollis* Luc.; 8. *Cymindis sitifensis* Luc.; 9. *Cymindis leucophthalma* Luc. **Plate 2** [DP: 28 March–15 August 1846]: 1. *Cymindis mauritanica* Dej.; 3. *Dromius insignis* Luc.; 4. *Dromius cruciferus* Luc.; 5. *Dromius striatipennis* Luc.; 6. *Dromius mauritanicus* Luc.; 7. *Dromius levipennis* Luc.; 8. *Dromius albomaculatus* Luc.; 10. *Singilis mauritanica* Luc. **Plate 3** [DP: 28 March–15 August 1846]. 1. *Cymindis levistriata* Luc.; 2. *Cymindis marginata* Luc.; 3. *Cymindis gaubilii* Luc.; 4. *Zuphium numidicum* Luc.; 5. *Lebia fulvicollis* Fabr.; 6. *Lebia numidica* Luc.; 7. *Brachynus barbarus* Luc.; 8. *Brachynus fimbriolatus* Luc. **Plate 4** [DP: 28 March–15 August 1846]: 1. *Graphipterus exclamationis* Fabr.; 2. *Graphipterus luctuosus* Dej.; 3. *Siagona gerardii* Buq.; 4. *Scarites collinus* Ramb.; 6. *Dischirius algiricus* Putz.; 7. *Dischirius obsoletus* Putz.; 8. *Carterus rufipes* Luc.; 9. *Ditomus dilaticollis* Luc. **Plate 5** [DP: 28 March–15 August 1846]: 1. *Ditomus opacus* Erichs.; 2. *Ditomus ruficornis* Luc.; 3. *Carabus morbillosus* Fabr.; 4. *Carabus numida* Lap.; 5. *Carabus maillei* Sol.; 6. *Carabus rugosus* Fabr. **Plate 6** [DP: 28 March–15 August 1846]: 2. *Nebria andalusia* Ramb.; 3. *Nebria rubicunda* Quens.; 5. *Chlaenius auricollis* Géné; 6. *Chlaenius varvasii* Lap.; 7. *Chlaenius aeratus* Quens.; 8. *Oodes mauritanicus* Luc.; 9. *Oodes abaxoides* Luc.; 10. *Pristonychus algirinus* Gory. **Plate 7** [DP: 28 March–15 August 1846]: 1. *Pristonychus sardous* Luc.; 2. *Pristonychus barbarus* Luc.; 4. *Calathus opacus* Luc.; 5. *Anchomenus algirinus* Buq.; 7. *Anchomenus numidicus* Luc.; 8. *Anchomenus fulgidicollis* Erichs.; 9. *Olisthopus puncticollis* Luc.; 10. *Poecilus barbarus* Luc. **Plate 8** [DP: 28 March–15 August 1846]: 1. *Poecilus coarctatus* Luc.; 2. *Poecilus numidicus* Luc.; 3. *Omazeus tingitanus* Luc.; 4. *Omazeus distinctus* Luc.; 5. *Poecilus lineatus* Sol.; 6. *Zabrus distinctus* Luc.; 8. *Masoreus testaceus* Luc.; 10. *Harpalus mauritanicus* Gaub. **Plate 9** [DP: 28 March–15 August 1846]: 1. *Acinopus lepeletieri* Luc.; 2. *Acinopus mauritanicus* Luc.; 3. *Acinopus elongatus* Luc.; 4. *Acinopus gutturosus* Buq.; 5. *Anisodactylus dejeanii* Buq.; 7. *Stenolophus abdominalis* Géné; 9. *Acupalpus flavipennis* Luc.; 10. *Acupalpus marginatus* Luc. **Plate 10** [DP: 28 March–15 August 1846]: 3. *Bembidium algiricum* Luc.; 4. *Bembidium numidicum* Luc.; 5. *Bembidium gibberosum* Luc.; 6. *Bembidium dives* Luc.; 7. *Bembidium mauritanicum* Luc.; 8. *Bembidium andreae* Gyllenh.; 9. *Bembidium vicinum* Luc.; 10. *Bembidium pulchellum* Luc. **Plate 11** [DP: 15 August 1846]: 1. *Cybister immarginatus* Fabr.; 2. *Cybister bivulnerus* Aubé; 3. *Cybister senegalensis* Aubé; 4. *Hydroporus confusus* Luc.; 5. *Hydroporus ferrugineus* Luc.; 7. *Aleochara scutellaris* Luc.; 9. *Myrmedonia tristis* Luc.; 10. *Bolithocara humeralis* Luc.; 11. *Homalota pallipes* Luc.; 12. *Xantholinus rufipes* Luc. **Plate 12** [DP: 15 August–November 1846]: 1. *Xantholinus ruficollis* Luc.; 2. *Ocypus nigrinus* Luc.; 3. *Ocypus planipennis* Aubé; 4. *Philonthus sparsus* Luc.; 5. *Euryporus aeneiventris* Luc.; 6. *Quedius pallipes* Luc.; 7. *Achenium hoemorrhoidale* Luc.; 8. *Achenium distinctum* Luc.; 9. *Lathrobium anale* Luc.; 10. *Lathrobium albipes* Luc. **Plate 13** [DP: 15 August 1846]: 1. *Lithocaris minuta* Luc.; 2. *Stilicus ruficornis* Luc.; 3. *Paederus caligatus* Erichs.; 4. *Stenus aeneus* Luc.; 5. *Stenus modestus* Luc.; 6. *Stenus obscurus* Luc.; 7. *Platysthetus longicornis* Luc.; 8. *Scydmaenus schaumii* Luc.; 9. *Scydmaenus angustatus* Luc.; 10. *Bryaxis heterocera* Aubé. **Plate 14** [DP: 28 March–15 August 1846]: 1. *Julodis sitifensis* Luc.; 3. *Acmoeodera mauritanica* Luc.; 4. *Acmoeodera tristis* Luc.; 5. *Acmoeodera melanosoma* Luc.; 6. *Acmoeodera multipunctata* Luc.; 7. *Acmoeodera rubromaculata* Luc.; 8. *Acmoeodera vicina* Luc.; 9. *Acmoeodera flavopunctata* Luc.; 10. *Acmoeodera flavonotata* Luc.; 11. *Acmoeodera flavovittata* Luc. **Plate 15** [DP: 28 March–15 August 1846]: 1. *Acmoeodera sexpustulata* de Cast et Gory; 2. *Acmoeodera trifoveolata* Luc.; 3. *Acmoeodera coarctata* Luc.; 4. *Acmoeodera rufomarginata* Luc.; 6. *Buprestis mauritanica* Luc.; 7. *Buprestis douei* Luc.; 8. *Buprestis levaillantii* Luc.; 10. *Sphenoptera vittaticollis* Luc.; 12. *Anthaxia rugicollis* Luc.; 13. *Anthaxia fulgidipennis* Luc. **Plate 16** [DP: 15 August–November 1846]: 1. *Anthaxia luctuosa* Luc.; 2. *Anthaxia chlorocephala* Luc.; 3. *Coroebus fulgidicollis* Luc.; 4. *Aphanisticus pygmaeus* Luc.; 5. *Aphanisticus angustatus* Luc.; 6. *Cratonychus mauritanicus* Luc.; 9. *Cardiophorus sex.maculatus* Luc.; 14. *Dolopius marginipennis* Luc.; 15. *Adrastus bicolor* Luc.; 16. *Anelastes barbarus* Luc. **Plate 17** [DP: before 15 May 1847]: 1. *Oophorus algirinus* Luc.; 2. *Cebrio barbarus* Luc.; 3. *Cebrio dimidiatus* Luc.; 4. *Cebrio melanocephalus* Luc.; 5. *Cebrio attenuatus* Luc.; 6 *Cebrio guyonii* Guér.; 7. *Drilus mauritanicus* Luc.; 8. *Drilus mauritanicus* (femelle du); 9. *Drilus mauritanicus* (larre du); 10. *Drilus mauritanicus* (nymphe du). **Plate 18** [DP: 15 August–November 1846]: 1. *Telephorus scutellaris* Luc.; 2. *Telephorus mauritanicus* Luc.; 3. *Telephorus fossulatus* Luc.; 4. *Telephorus geniculatus* Luc.; 5. *Malthinus longipennis* Luc.; 6. *Malthinus pulchellus* Luc.; 7. *Malachius insignis* Buq.; 8. *Malachius marginicollis* Luc.; 9. *Malachius mauritanicus* Luc.; 10. *Malachius angusticollis* Luc. **Plate 19** [DP: 15 August 1846]: 1. *Malachius affinis* Luc.; 2. *Malachius maculicollis* Luc.; 3. *Malachius tristis* Luc.; 4. *Dasytes variegatus* Luc.; 5. *Dasytes mauritanicus* Luc.; 6. *Dasytes cupreus* Luc.; 7. *Dasytes algiricus* Luc.; 8. *Dasytes pectinicornis* Luc.; 9. *Dasytes armatus* Luc.; 10. *Dasytes distinctus* Luc. **Plate 20** [DP: 15 August 1846]: 1. *Melyris rubripes* Luc.; 3. *Opilus dorsalis* Luc.; 4. *Ptinus rufus* Luc.; 5. *Ptinus fossulatus* Luc.; 6. *Ptinus mauritanicus* Luc.; 7. *Ptinus rotundicollis* Luc.; 8. *Ptinus carinatus* Luc.; 9. *Ptinus gibbicollis* Luc.; 10. *Ptinus obesus* Luc.; 11. *Ptinus hirticollis* Luc. **Plate 21** [DP: 28 March–15 August 1846]: 1. *Silpha puncticollis* Luc.; 2. *Silpha tuberculata* Luc.; 3. *Catops rufipennis* Luc.; 4. *Catops marginicollis* Luc.; 5. *Colon pubescens* Luc.; 6. *Cercus bicolor* Luc.; 7. *Cercus barbarus* Luc.; 8. *Epurea nigrita* Luc.; 9. *Myrmecobius agilis* Luc.; 10. *Thorictus mauritanicus* Luc. **Plate 22** [DP: 28 March–15 August 1846]: 1. *Thorictus germari* Luc.; 2. *Thorictus puncticollis* Luc.; 3. *Aulachocheilus chevrolatii* Luc.; 4. *Cryptophagus angustatus* Luc.; 5. *Cryptophagus laticollis* Luc.; 6. *Cryptophagus* ? *gibberosus* Luc.; 7. Cryptophagus ? maurus Luc.; 8. *Hister amplicollis* Erichs.; 9. *Saprinus mauritanicus* Luc.; 10. *Platysoma algiricum* Luc. **Plate 23** [DP: 15 August 1846]: 1. *Parnus algiricus* Luc.; 2. *Georyssus costatus* de Cast.; 3. *Hydrophilus inermis* Luc.; 5. *Ateuchus cicatricosus* Dej.; 6. *Ateuchus puncticollis* Latr.; 8. *Onitis chevrolatii* Luc.; 9. *Onthophagus maurus* Luc.; 10. *Onthophagus analis* Luc.; 11. *Aphodius cribricillis* Luc.; 13. *Aphodius hirtipennis* Luc. **Plate 24** [DP: 28 March–15 August 1846]: 1. *Aphodius affinis* Luc.; 2. *Otophorus scolytoides* Luc.; 3. *Rhyssenus algiricus* Luc.; 4. *Thorectes rotundatus* Luc.; 5. *Thorectes puncticollis* Luc.; 6. *Geobius tricornis* Luc.; 7. *Melolontha mauritanica* Luc.; 8. *Elaphocera mauritanica* Ramb.; 9. *Elaphocera barbara* Ramb.; 10. *Elaphocera numidica* Ramb. **Plate 25** [DP: 15 August 1846]: 2. *Rhizotrogus magagnoscii* Guér.; 3. *Rhizotrogus tusculus* Buq.; 4. *Rhizotrogus amphytus* Buq.; 5. *Rhizotrogus gerardii* Buq.; 6. *Rhizotrogus inflatus* Buq.; 7. *Rhizotrogus numidicus* Luc.; 8. *Hymenoplia cinctipennis* Luc.; 9. *Brachyphilla barbara* Luc.; 10. *Hoplia sulphurea* Chevr.; 11. *Glaphyrus viridicollis* Luc. **Plate 26** [DP: 28 March–15 August 1846]: 1. *Trachyderma angustata* Sol.; 2. *Pachyscelis crinita* Sol.; 3. *Pimelia latreillii* Sol.; 4. *Pimelia barbara* Sol.; 5. *Pimelia dejeanii* Sol.; 6. *Pimelia cribripennis* Sol.; 7. *Pimelia simplex* Sol.; 8. *Pimelia boyeri* Sol.; 9. *Pimelia duponti* Sol.; 10. *Pimelia maura* Sol. **Plate 27** [DP: 28 March–15 August 1846]: 1. *Megagenius friolii* Sol.; 2. *Adesmia microcephala* Sol.; 3. *Adesmia douei* Luc.; 4. *Adesmia biskrensis* Luc.; 5. *Adesmia faremontii* Luc.; 6. *Erodius nitidicollis* Sol.; 7. *Zophosis personata* Erichs.; 8. *Morica octocostata* Sol.; 9. *Akis algeriana* Sol.; 10. *Akis barbara* Sol. **Plate 28** [DP: 28 March–15 August 1846]: 1. *Tentyria bipunctata* Sol.; 2. *Tentyria affinis* Luc.; 3. *Tentyria solieri* Luc.; 4. *Pachychila punctulata* Luc.; 5. *Pachychila sabulosa* Luc.; 6. *Pachychila germari* Sol.; 7. *Tagenia webbii* Luc.; 8. *Tagenia algirica* Luc.; 9. *Scaurus dubius* Sol.; 10. *Scaurus punctatus* Sch. **Plate 29** [DP: before 15 May 1847]: 1. *Asida complanata* Luc.; 2. *Asida sinuaticollis* Sol.; 3. *Asida lapidaria* Luc.; 4. *Pachypterus mauritanicus* Luc.; 5. *Philax moreletii* Luc.; 6. *Dendarus rotundicollis* Luc.; 7. *Crypticus obesus* Luc.; 8. *Opatrum emarginatum* Luc.; 9. *Opatrum perplexum* Luc.; 10. *Opatrum algiricum* Luc. **Plate 30** [DP: 15 August–November 1846]: 1. *Opatrum pulchellum* Luc.; 2. *Opatrum lilliputanum* Luc.; 3. *Trachyscelis rufus* Latr.; 4. *Cossyphus hoffmanseggii* Herbst; 5. *Cossyphus ovatus* de Brême; 6. *Cataphronetis levaillantii* Luc.; 7. *Hypophloeus angustus* Luc.; 8. *Hypophloeus suberis* Luc.; 9. *Boros tagenioides* Luc.; 10. *Boros* ? *rufipes* Luc. **Plate 31** [DP: 15 August 1846]: 1. *Helops insignis* Luc.; 2. *Helops heteromorpha* Luc.; 4. *Helops villosipennis* Luc.; 5. *Helops tuberculipennis* Luc.; 6. *Helops nitidicollis* Luc.; 7. *Helops parvulus* Luc.; 8. *Cistela melanophthalma* Luc.; 10. *Omophlus erythrogaster* Luc.; 11. *Megischia nigripennis* Fabr.; 12. *Megischia erythrocephala* Sol. **Plate 32** [DP: November 1847]: 1. *Eutrapela suturalis* Luc.; 2. *Lagria viridipennis* Fabr.; 3. *Notoxus mauritanicus* de Laferté; 4. *Notoxus numidicus* Luc.; 5. *Anthicus insignis* Luc.; 6. *Anthicus vittatus* Luc.; 7. *Anthicus quadrimaculatus* Luc.; 8. *Anthicus fumosus* Luc.; 9. *Evaniocera boryi* Luc.; 10. *Mordella decora* Luc. **Plate 33** [DP: 15 August 1846]: 1. *Meloe aenea* de Casteln.; 2. *Meloe affinis* Luc.; 3. *Meloe maculifrons* Luc.; 4. *Meloe plicatipennis* Luc.; 5. *Meloe nana* Luc.; 6. *Cerocoma vahlii* Fabr.; 7. *Mylabris interrupta* Oliv.; 8. *Mylabris circumflexa* Chevr.; 9. *Mylabris rubripennis* Chev.; 10. *Mylabris maura* Chevr. **Plate 34** [DP: 28 March–15 August 1846]: 1. *Mylabris praeusta* (var.) Fabr.; 2. *Mylabris affinis* Luc.; 3. *Cantharis segretum* Fabr.; 4. *Cantharis viridissima* Luc.; 5. *Cantharis scutellata* de Casteln.; 6. *Cantharis cirtana* Luc.; 8. *Nemognathus chrysomelinus* Fabr.; 9. *Oedemera viridana* Luc.; 10. *Oedemera brabara* Fabr.; 11. *Oedemera tibialis* Luc. **Plate 35** [DP: 15 August–November 1846]: 1. *Bruchus flavescens* Luc.; 2. *Bruchus plumbeus* Luc.; 4. *Brachytarsus pantherinus* Luc.; 5. *Apion albopilosum* Luc.; 6. *Brachycerus semituberculatus* Luc.; 8. *Brachycerus corrosus* Schoenh. 9. *Brachycerus scutellaris* Luc.; 10. *Thylacites variegatus* Luc.; 11. *Eusomus affinis* Luc.; 12. *Polydrosus pallipes* Luc. **Plate 36** [DP: 15 August 1846]: 1. *Cleonus leucomelas* Luc.; 2. *Cleonus margaratiferus* Luc.; 3. *Pachycerus rugosus* Luc.; 4. *Rhytirhinus variegatus* Luc.; 5. *Rhytirhinus humilis* Luc.; 6. *Rhytirhinus annulipes* Luc.; 7. *Rhytirhinus impressicollis* Luc.; 8. *Rhytirhinus horridus* Luc.; 9. *Otiorhynchus corticalis* Luc.; 10. Otiorhynchus ? metallescens Luc. **Plate 37** [DP: before 15 May 1847]: 1. *Nastus albopunctatus* Luc.; 2. *Nastus albomarginatus* Luc.; 3. *Lixus wagneri* Luc.; 4. *Lixus affinis* Luc.; 5. *Lixus bimaculatus* Luc.; 6. *Lixus brevicaudatus* Luc.; 7. *Lixus coarctatus* Luc.; 9. *Larinus albicans* Luc.; 10. *Larinus nanus* Luc.; 11. *Tychius fuscolineatus* Luc. **Plate 38** [DP: 15 August–November 1846]: 1. *Tychius carinicollis* Luc.; 2. *Sybines sellatus* Luc.; 3. *Baridius pulchellus* Luc.; 4. *Acalles barbarus* Luc.; 5. *Acalles punctaticollis* Luc.; 6. *Acalles impressicollis* Luc.; 7. *Ceutorhynchus flavomarginatus* Luc.; 8. *Gymnoetron crassirostris* Luc.; 9. *Gymnoetron vulpes* Luc.; 10. *Nanophyes durioei* Luc. **Plate 39** [DP: 31 December 1848]: 1. *Bostrichus* ? *dactyliperda* Panz.; 2. *Hypoborus ficus* Erichs.; 3. *Xylopertha appendiculata* Luc.; 4. *Bostrichus nigriventris* Luc.; 5. *Apate francisca* Fabr. (larve de l’). **Plate 40** [DP: 15 August–November 1846]: 1. *Xylopertha humeralis* Luc.; 2. *Cis cribratus* Luc.; 3. *Cis flavipes* Luc.; 4. *Cis punctulatus* Luc.; 5. *Psammoecius boudieri* Luc.; 6. *Rhyzophagus unicolor* Luc.; 7. *Laemophloeus nigricollis* Luc.; 8. *Laemophloeus rufipes* Luc.; 9. *Laemophloeus suberis* Luc.; 10. *Laemophloeus elongatulus* Luc. **Plate 41** [DP: before 15 May 1847]: 1. *Macrotoma myardi* Mulst.; 2. *Ergates faber* Linn.; 3. *Cerambyx mirbeckii* Luc.; 4. *Cerambyx nerii* Erichs.; 5. *Cerambyx levaillantii* Luc.; 7. *Purpuricenus dumerilii* Luc.; 8. *Purpuricenus barbarus* Luc.; 9. *Aromia rosarum* Luc.; 10. *Hesperophanes affinis* Luc. **Plate 42** [DP: before 15 May 1847]: 1. *Clytus glaucus* Fabr.; 2. *Clytus sexguttatus* Luc.; 3. *Stenopterus mauritanicus* Luc.; 4. *Parmena algerica* de Casteln.; 5. *Steneida troberti* Mulst.; 6. *Agapanthia cynaroe* Germ.; 7. *Agapanthia lixoides* Luc.; 8. *Oberea maculicollis* Luc.; 9. *Oberea mauritanica* Luc.; 10. *Phytoecia guerinii* de Brême. **Plate 43** [DP: before 15 May 1847]: 1. *Phytoecia warnieri* Luc.; 2. *Phytoecia cirtana* Luc.; 3. *Phytoecia rubricollis* Luc.; 4. *Phytoecia flavipes* Fabr. 5. *Phytoecia azurea* Sch.; 6. *Phytoecia erythrocnema* Luc.; 7. *Phytoecia malachytica* Luc.; 8. *Phytoecia molybdoena* Dalm.; 9. *Leptura oblongomaculata* Buq.; 10. *Leptura melas* Luc. **Plate 44** [DP: before 15 May 1847]: 1. *Hispa numida* Guér.; 2. *Hispa algeriana* Guér.; 3. *Hispa atra* Fabr.; 4. *Cassida algirica* Bohém.; 5. *Cassida herbea* Bohém.; 7. *Adimonia violacea* Luc.; 8. *Galleruca sublineata* Luc.; 9. *Galleruca foveicollis* Luc.; 10. *Luperus flavipennis* Luc.; 11. *Altica lythri* Aubé. **Plate 45** [DP: before 15 May 1847]: 1. *Altica punctipennis* Luc.; 2. *Altica impressa* Fabr.; 3. *Altica ruficollis* Luc.; 5. *Timarcha punica* Luc.; 6. *Timarcha endora* Buqt.; 7. *Chrysomela afra* Erichs.; 8. *Chrysomela crassipes* Luc.; 9. *Chrysomela erythromera* Luc.; 10. *Chrysomela gaubilii* Luc.; 11. *Chrysomela chloris* Luc. **Plate 46** [DP: 15 August–November 1846]: 1. *Helodes distincta* Luc.; 2. *Helodes vicina* Luc.; 3. *Colaspidea nitida* Luc.; 4. *Colaspidea pulchella* Luc.; 5. *Colaspidea signatipennis* Luc.; 6. *Pseudocolaspis setosa* Luc.; 7. *Pachnephorus cylindricus* Luc.; 8. *Labidostomis rubripennis* Luc.; 9. *Labidostomis hybrida* Luc.; 10. *Labidostomis forcipifera* Luc. **Plate 47** [DP: 15 August–November 1846]: 1. *Cyaniris unicolor* Luc.; 3. *Lachnoea straminipennis* Luc.; 4. *Lachnoea puncticollis* Luc.; *Macrolenes dispar* Luc.; 6. *Smaragdina gratiosa* Luc.; 7. *Cryptocephalus cicatricosus* Luc.; *Cryptocephalus gravidus* Luc.; 10. *Phalacrus striatipennis* Luc.; 12. *Micraspis phalerata* Luc.; 13. *Dapsa barbara* Luc.

### Appendix 10. List of Coleoptera illustrated on the plates of ‘*Histoire physique, naturelle et politique de Madagascar. Histoire naturelle des coléoptères. Tome II. – Atlas*’ (Künckel d’Herculais, 1887–1891)

Plates 1–25 were issued in 1888, plates 26–54 in [1891].

**Plate 1**: *Euchroea auripigmenta*; 2. *Euchroea multiguttata*; 3. *Euchroea histrionica*; 4. *Euchroea clementi*; 5. *Euchroea abdominalis*; 6. *Euchroea episcopalis*; 7.8. *Euchroea aurora*; 9,10. *Euchroea caelestis*; 11. *Euchroea desmarestii*; 12. *Pantolia flavomarginata*. **Plate 2**: 1. *Pantolia rubrofasciata*; 2. *Pantolia ebenina*; 3. *Pantolia striata*; 4. *Dirrhina iris*; 5. *Pogonotarsus plumiger*; 6. *Pantolia vescoi*; 7,8. *Coptomia costata*; 9. *Coptomia bipunctata*; 10,11,12. *Coptomia mauritiana*. **Plate 3**: 1. *Coptomia opalina*; 2. *Choleva sericea*; 3. *Coptomia sexmaculata*; 4. *Cleonis granulata*; 5,6. *Cyriodera tuberculicollis* ♂ et ♀; 7. *Celidota stephensi*; 8. *Euryoma argentea*; 9. *Parachilia collata*; 10. *Anochilia lenocinia*; 11. *Anochilia conjuncta*; 12. *Anochilia ornata*; 13. *Anochilia cingulata*. **Plate 4**: 1. *Anochilia princeps*; 2. *Anochilia scapularis*; 3,4,5,6,7. *Asyda laevigata*; 8. *Anochilia stupida*; 9. *Anochilia villosula*; 10. *Argutor bicolor*; 11. *Anochilia pratensis*; 12. *Anochilia cultrata*; 13. *Pantolia scapha*. **Plate 5**: *Parachilia leroyi*; 2. *Pycnopus bufo*; 3. *Pycnopus melanocala*; 4. *Pycnopus rubripes*; 5. *Pycnopus guerini*; 6. *Euchilia sulcata*; 7. *Euchilia quadrata*; 8. *Bricoptis variolosa*; 9. *Anochilia punctatissima*; 10. *Epixanthis stella*; 11. *Euchilia maculitarsis*; 12. *Euchilia 9-punctata*; 13. *Stenotarsia vermiculata*. **Plate 6**: 1. *Stenotarsia crocata*; 2. *Stenotarsia velutina*; 3. *Stenotarsia scapulata*; 4. *Liostraca iota*; 5. *Liostraca bina*; 6. *Chromoptilia diversipes*; 7. *Doryscelis calcarata*; 8,9. *Bothrorhina reflexa*; 10. *Bothrorhina ochreata*; 11. *Anochilia republicana*; 12. id. var. **Plate 7**: 1,2. *Anochilia erythroderes*; 3. *Oxythyrea argentifer*; 4,5. *Oxythyrea amabilis*; 6. *Oxythyrea vandana*; 7. *Glycyphana versicolor*; 8. *Cetonia goudoti*; 9. *Heterophana deyrollei*; 10,11. *Heterophana villosula*; 12,13. *Heterophana craticula*; 14,15. *Heterophana canaliculata*; 16. *Callipechis flavipes*. **Plate 8**: 1. *Coptomia nigriceps*; 2. *Callipechis lucida*; 3. *Callipechis crucigera*; 4. *Callipechis fulgida*; 5. *Cleonis marginata*; 6. *Callipechis tarsalis*; 7. *Callipechis crassa*; 8. *Euchilia costifer*; 9. *Euchilia picipes*; 10. *Anochilia pulchripes*; 11. *Anochilia cowani*; 12. *Anochilia lineata*; 13. *Anochilia versicolor*. **Plate 9**: 1. *Bothrorrhina reflexa*, var.; 2 et 3. *Bothrorrhina radama* ♂ et ♀; 4. *Linotarsia discoidalis*; 5. *Linotarsia picta*; 6. *Liostraca bella*; 7. *Rhyncocephala hildebrandti*; 8. *Euchilia puncticollis*; 9. *Euchilia cupricollis*; 10. Anochilia (Coquerelia) republicana, var.; 11. *Pantolia rufobasalis*; 12. *Coptomia laevis*; 13. *Curculio mutabilis*; 14. *Colliuris modesta*; 15. *Coptomia apicalis*; 16. *Coptomia uniformis*; 17. *Cercyon elegans*. **Plate 10**: 1. *Encya commersoni*; 2. *Encya bisignata*; 3. *Encya betanimena*; 4. *Encya apicalis*; 5. *Encya variipennis*; 6. *Encya mucronata*; 7. *Empecta piligera*; 8. *Proagosternus niveus*; 9. *Proagosternus niveus* var. *ochraceus*; 10. *Proagosternus antanala*; 11. *Tricholepis niveopilosa*. **Plate 11**: 1. *Encya nebulosa*; 2. *Encya ornatipennis*; 3. *Encya nigra*; 4. *Encya dubia*; 5. *Empecta micantipennis*; 6. *Encya maculipennis*; 7. *Encya villosa*; 8. *Proagosternus lamellifer*; 9. *Pelecotoma latreillei*; 10. *Tricholepis lactea*; 11. *Hoplochelus rhixotrogoides*; 12. *Enthora chlorodera*. **Plate 12**: 1 et 2. *Emphania metallica*; 3. *Enaria melanictera*; 4. *Emphania antanala*; 5. *Trigonostomum mucoreum*; 6. *Adoretus aenescens*; 7. *Adoretus vagepunctatus*; 8. *Adoretus curtus*; 9. *Adoretus costalis*; 10. *Aphodius villosus*; 11. *Adoretus carinatus*; 12. *Adoretus convexipennis*; 13. *Acinopus elongatus*; 14. *Adoretus giganteus*; 15. *Adoretus latissimus*; 16. *Anthicus tibialis*. **Plate 13**: 1. *Hoplia marmorea*; 2. *Harpalus bicolor*; 3. *Harpalus pilosa*; 4. *Harpalus ciribrella*; 5. *Harpalus bicallosa*; 6. *Harpalus irrorata*; 7. *Hoplia hova*; 8. *Harpalus nigrescens*; 9. *Hypsioma gemmata*; 10. *Harpalus debilis*; 11. *Harpalus nodipennis*; 12. *Harpalus oculata*; 13. *Harpalus tuberculata*; 14. *Harpalus fasciculata*; 15. *Dicentrines pumilus*; 16. *Dacne femoralis*. **Plate 14**: 1. *Hoplia fulva*; 2. *Hoplia luctuosa*; 3. *Hoplia lactea*; 4. *Hoplia striata*; 5. *Hoplia nigra*; 6. *Hoplia sinuata*; 7. *Hoplia margaritacea*; 8. *Hoplia retusa*; 9. *Dicentrines lineaticollis*; 10. *Dicentrines lineatus*; 11. *Microplus insignicollis*; 12. *Microplus cinerascens*; 13. *Microplus castaneus*; 14. *Monocrepidius vittatus*; 15. *Megaceras rugosus*; 16. *Microplus brevis*. **Plate 15**: 1. *Hopleidos lineopictus*; 2. *Hopliopsis fulvovestitus*; 3. *Serica castanea*; 4. *Serica setosipennis*; 5. *Serica setosicollis*; 6. *Serica irrorata*; 7. *Serica lucidula*; 8. *Serica cinnamomea*; 9. *Serica fuliginosa*; 10. *Serica mutans*; 11. *Serica grossa*; 12. *Silpha laevigata*; 13. *Serica tesellata*; 14. *Serica conspurcata*; 15. *Serica maculata*; 16. *Stenolophus micans*; 17. *Serica geminata*; 18. *Serica affinis*. **Plate 16**: 1. *Empecta obsoleta*; 2. *Empecta cuprea*; 3. *Empecta squamifera*; 4. *Empecta gracilis*; 5. *Empecta cinerea*; 6. *Empecta betanimena*; 7. *Trigonostoma bivittatum*; 8. *Adoretus strigatus*; 9. *Adoretus hystrix*; 10. *Anthicus vittatus*; 11. *Enthora chlorodera*; 12. *Pachycolus douinii*; 13. *Phaeocrous madagascariensis*; 14. *Phaeocrous madagascariensis*; 15. *Lonchotus muticus*; 16. *Hoplia hova*; 17. *Hoplia madecassa*; 18. *Omaloplia virescens*; 19. *Omaloplia gracilis*; 20. *Ozaena laevigata*; 21. *Omaloplia unicolor*. **Plate 17**: 1. *Ateuchus radama*; 2 et 3. *Onthophagus elegans* ♂ et ♀; 4. *Onthophagus hinnulus*; 5 et 6. *Onthophagus maitso* ♂ et ♀; 7. *Onthophagus auratus*; 8. *Onthophagus variegatus*; 9. *Ometis pictus*; 10, 11 et 12. *Oniticellus marsyas* ♂ et ♀; 13. *Oniticellus caeruleus*; 14 et 15. *Oniticellus undatus* ♂ et ♀; 16. *Oniticellus quadripunctatus*; 17. *Oniticellus clouei*. **Plate 18**: 1. *Epilissus hova*; 2. *Epilissus viridis*; 3. *Epilissus brunnipes*; 4. *Epilissus clypeatus*; 5. *Epilissus rubromaculatus*; 6. *Epilissus vicinus*; 7. *Epilissus histeroides*; 8. *Epilissus globulosus*; 9. *Epilissus minutus*; 10. *Epilissus fulgens*; 11. *Epilissus madagascariensis*; 12. *Elmis aeneus*; 13. *Epilissus striatus*; 14. *Erodius pygmaeus*; 15. *Epilissus ater*; 16. *Epilissus prasinus*; 17. *Epilissus bimaculatus*; 18. *Epilissus splendens*. **Plate 19**: 1 et 2. *Oryctes pyrrhus* seu Radama ♂ et ♀; 3 et 4. *Oryctes simiar* ♂ et ♀; 5 et 6. *Oryctes ranavalo* ♂ et ♀; 7 et 8. *Oryctes insularis* ♂ et ♀; 9 et 10. *Oryctes augias* ♂ et ♀. **Plate 20**: 1 et 2. *Oryctes colonicus* ♂ et ♀; 3. *Oryctes fibekabeka*; 4. *Temnorhyncus galeatus*; 5 et 6. *Lonchotus crassus*; 7. *Heteronychus plebejus*; 8. *Heteronychus rusticus*; 9 et 10. *Trionychus mainty* ♂ et ♀; 11. *Hexodon rotundatum*; 12. *Hister unicolor*; 13. *Hexodon hopei*; 14. *Hexodon montandoni*. **Plate 21**: 1. *Hexodon reticulatum*; 2. *Temnorhynchus truncatus*; 3. *Temnorhynchus fairmairei*; 4. *Temnorhynchus coquereli*; 5. *Anodon coquereli*; 6 et 7. *Cladognathus serricornis*; 8. *Nigidius madagascariensis*; 9. *Figulus anthracinus*; 10. *Figulus striatus*; 11. *Leptaulax morbillosus*; 12. *Leptaulax approximatus*; 13. *Solenocyclus exaratus*. **Plate 22**: 1. *Epilissus violaceus*; 2. *Epilissus nitidus*; 3. *Aphodius moestus*; 4. *Psammodius laticeps*; 5 et 6. *Orphnus nitidulus* ♂ et ♀; 7 et 8. *Orphnus coquereli* ♂ et ♀; 9 et 10. *Orphnus goudoti* ♂ et ♀; 11. *Orphnus madecassus*; 12. *Ochodaeus maxillosus*; 13. *Orphnus cannellinus*; 14. *Hybosorus illigeri*; 15. *Trox madagascariensis*; 16. *Heteronychus rugifrons*; 17. *Heteronychus minutus*; 18. *Heteronychus parvus*; 19. *Philodrosus cyaneus*. **Plate 23**: 1. *Pogonostoma viride*; 2. *Pogonostoma brunnipes*; 3. *Pogonostoma pubescens*; 4. *Pogonostoma anthracinum*; 5. *Pogonostoma chalybaeum*; 6. *Platynus elegans*; 7. *Pogonostoma spinipenne*; 8. *Pogonostoma pusillum*; 9. *Pogonostoma sericeum*; 10. *Pogonostoma gratiosum*; 11. *Pogonostoma coeruleum*; 12. *Pogonostoma cyanescens*. **Plate 24**: 1. *Cicindela minuta*; 2. *Cetonia cyanea*: 3 et 4. *Cicindela maheva*; 5. *Cicindela dumolini*; 6. *Cicindela trinularis*; 7. *Cicindela abbreviata*; 8. var. *circumducta*; 9. *Cicindela perplexa*; 10. *Curculio equestris*; 11. *Cicindela soa*; 12. *Cicindela fimbriata*; 13. *Megalomma adonis*; 14. *Peridexia mirabilis*. **Plate 25**: 1. *Cicindela viridicyanea*; 2. *Cicindela semipicta*; 3. *Cicindela aberrans*; 4. *Colpodes madagascariensis*; 5. *Cicindela hova*; 6. *Cicindela radama*; 7. *Calosoma grandidieri*; 8. *Megalomma rugicollis*; 9. *Megalomma marginatum*; 10. *Peridexia frontalis*; 11. *Tetragonoderus pallidus* ?; 12. *Coptodera miltomera*; 13. *Nycteis madagasciariensis*; 14. *Nebria brevicollis*; 15. *Somoplatus pusilla*; 16. *Hemiteles interruptus*; 17. *Harpalus rufus*; 18. *Morio parallelus*; 19. *Calosoma grandidieri*. **Plate 26**: 1. *Omophron madagascariensis*; 2. *Hexagonia cephalotes*; 3. *Drypta iris*; 4. *Drypta madecassa*; 5. *Eunostus latreillei*; 6. *Pheropsophus abbreviatus*; 7. *Pheropsophus abbreviatus* var.; 8. *Pheropsophus quadrimaculatus*; 9 et 10. *Pheropsophus goudoti*; 11. *Phaeocrous madagascariensis*; 12. *Callida fastuosa*; 13. *Callida jucunda*; 14. *Demetrias dissimilis*; 15. *Dromius nigerrimus*; 16. *Lebia fusca*; 17. *Lamia formosa*. **Plate 27**: 1. *Belonognatha pustulata*; 2. *Belonognatha signatipennis*; 3. *Thyreopterus anchomenoides*; 4. *Trechus laticollis*; 5. *Thyreopterus inermis*; 6. *Thyreopterus longipennis*; 7. *Tabanus striatus*; 8. *Tropopsis unicolor*; 9. *Trogossita sublaevis*; 10. *Thyreopterus spinosus*; 11. *Thyreopterus armatus*; 12. *Thyreopterus armatus* var.; 13. *Sphaerostylus ditomoides*; 14. *Sphaerostylus goryi*. **Plate 28**: 1. *Crepidopterus raffrayi*; 2. *Dioryche interpunctata*; 3. *Colpodes madagascariensis*; 4. *Oodes deplanatus*; 5. *Drimostoma ebeninum*; 6. *Drimostoma anthracinum*; 7. *Distrigus bipustulatus*; 8. *Melanodes atratus*; 9. *Tryptocerus politus*; 10. *Casnonia coerulans*; 11. *Odacantha nosibeana*; 12. *Chlaenius unicolor*; 13. *Euleptus geniculatus*; 14. *Coptodera miltomera*; 15. *Thyreopterus femoratus*; 16. *Thyreopterus impressiusculus*; 17. *Thyreopterus tetraspilotus*; 18. *Galerita madecassa*; 19. *Nycteis semipicea*; 20. *Abacetus corvinus*; 21. *Microchila picea*; 22. *Stenolophus iridescens*; 23. *Pheropsophus fulviventris*. **Plate 29**: 1. *Dyscherus costatus*; 2. *Dyscherus tricostis*; 3. *Crepidopterus pipitzii*; 4. *Storthodontus depressus*; 5. *Storthodontus nimrod*; 6. *Storthodontus coquereli*; 7. *Crepidopterus cribripennis*; 8. *Cetonia goudoti*; 9. *Scarites madagascariensis*; 10. *Scarites pluto*; 11. *Dyscherus rapax*. **Plate 30**: 1. *Oodes atratus*; 2. *Eudema nigrita*; 3. *Eudema festivum*; 4. *Chlaenius attenuatus*; 5. *Chlaenius indutus*; 6. *Chlaenius cribricollis*; 7. *Chlaenius pubifer*; 8. *Chlaenius radama*; 9. *Cerambyx unicolor*; 10. *Chlaenius rufomarginatus*; 11. *Chlaenius subovatus*; 12. *Chlaenius poricollis*; 13. *Chlaenius ranavalo*; 14. *Chlaenius hova*. **Plate 31**: 1. *Eucamptognathus lafertei*; 2. *Eucamptognathus purpurascens*; 3. *Eucamptognathus chevrolati*; 4. *Eucamptognathus spectabilis*; 5. *Eucamptognathus spectabilis* var.; 6. *Abax africanus*; 7. *Homalosoma laevicolle*; 8 et 9. *Homalosoma striatocolle* ♂ et ♀; 10. *Homalosoma tricostatum*; 11. *Chlaenius lugens*. **Plate 32**: 1. *Silpha metallescens*; 2. *Alindria sedilloti*; 3 et 4. *Alindria spectabilis*; 5. *Alindria cyanicornis*; 6. *Hectarthrum goudoti*; 7. *Rhysodes parumcostatus*; 8. *Rhysodes tubericeps*; 9. *Rhysodes canaliculatus*; 10. *Rhysodes montanus*; 11. *Pycnomerus cribricollis*; 12. *Peltis pusillus*; 13. *Oethina pubescens*; 14. *Lordites breviusculus*; 15. *Rechodes coquereli*; 16. *Rechodes fungosus*. **Plate 33**: 1. *Cyllaus scapularis*; 2. *Cyllaus apicalis*; 3. *Carpophilus truncatus*; 4. *Stelidota clavicornis*; 5. *Lordites costulatus* ♀; 6. *Latridius elongatus*; 7. *Iria nigritula*; 8. *Pachycephala chlorotica*; 9. *Rechodes humbloti*; 10. *Cerylon amplicolle*; 11. *Cerylon brevicolle*; 12. *Pseudino fragilis*; 13. *Pseudino coquerelii*; 14. *Psammochoedius spinicollis*; 15. *Potamophilus oxypterus*; 16. *Hydrethus dermostoides*. **Plate 34**: 1. *Cybister tripunctatus*; 2 et 3. *Cybister desjardinsi*; 4 et 5. *Cybister hova* ♂ et ♀; 6 et 7. *Colpodes madagascariensis* ♂ et ♀; 8. *Cybister senegalensis* ♂; 9. *Hydaticus signatipennis*; 10. *Hydaticus leander*; 11. *Hydaticus exclamationis* var.; 12. *Hydaticus madagascariensis*; 13. *Hydaticus petiti*. **Plate 35**: 1. *Lycoreus madagascariensis*; 2. *Lycoreus corpulentus*; 3. *Lycoreus triocellatus*; 4. *Lycoreus cyclops* var.; 5. *Lycoreus goudoti*; 6. *Lycoreus regalis*; 7. *Piezophyllus macrocerus*; 8. *Adelocera inflata*; 9. *Adelocera dorsalis*; 10. *Lacon leprosus*; 11. *Lacon leprosus* var. **Plate 36**: 1. *Lacon turbidus*; 2. *Liparetrus vestitus*; 3. *Lacon albopictus*; 4. *Tylotarsus cinctipes*; 5. *Tylotarsus armatus*; 6. *Melanoxanthus melanocephalus*; 7. *Drasterius tessellatus*; 8. *Melantho klugi*; 9. *Ctenicera nobilis*; 10. *Ctenicera nobilis* var. a; 11. *Ctenicera insignis*; 12 et 13. *Cephalodendron indigaceum* ♂ et ♀; 14. *Curculio virescens*. **Plate 37**: 1. *Lycoreus dux*; 2. *Lycoreus cyclops*; 3. *Melantho klugi*; 4. *Adelocera aterrima*; 5. *Adelocera madida*; 6. *Lacon ornatellus*; 7. *Lacon irroratus*; 8. *Lacon goudoti*; 9. *Liparetrus vestitus* var. *tumidicollis*; 10. *Tylotarsus aculeatus*; 11. *Tylotarsus mucoreus*; 12. *Tylotarsus albisparsus*; 13. *Tylotarsus pulvereus*; 14. *Tetralobus insularis*; 15. *Tetralobus grandidieri*. **Plate 38**: 1. *Chalcophoropsis quadrifoveolata*; 2. *Polybothris zivetta*; 3. *Polybothris impressipennis*; 4. *Polybothris aeneomaculata*; 5. *Polybothris circumdata*; 6. *Polybothris sumptuosa* ♀; 7. *Polybothris convexa*; 8. *Polybothris cyanimaculifer*; 9 et 10. *Polybothris navicularis* ♂ et ♀; 11. *Polybothris hova*. **Plate 39**: 1. *Polybothris aureopilosa*; 2. *Polybothris viridicollis*; 3. *Polybothris dilatata*; 4. *Polybothris goryi*; 5. *Polybothris ochreata*; 6. *Polybothris obtusa*; 7. *Polybothris analis*; 8. *Polybothris coquereli*; 9. *Polybothris aeneostictica*; 10. *Icaria alata*. **Plate 40**: 1. *Polybothris elegans*; 2. *Polybothris luczoti*; 3. *Polybothris scapularis*; 4. *Polybothris lelieuri*; 5. *Polybothris aureopilosa goudoti* var.; 6. *Polybothris sumptuosa* ♂; 7. *Polybothris scenica*; 8. *Polybothris amorpha*. **Plate 41**: 1. *Polybothris bernieri*; 2. *Polybothris maculiventris*; 3. *Polybothris chalcochrysea*; 4. *Polybothris colliciata*; 5. *Phyllocharis klugii*; 6. *Polybothris auriventris*; 7. *Polybothris emarginata*; 8. *Polybothris sparsuta*; 9. *Polybothris lafertei*; 10. *Polybothris nitidiventris*; 11. *Steraspis hercules*. **Plate 42**: 1. *Polybothris auropicta*; 2. *Polybothris emarginaticollis*; 3. *Polybothris rotundata*; 4. *Polybothris circularis*; 5. *Polybothris cassidoides*; 6. *Polybothris coccinella*; 7. *Polybothris lamina*; 8. *Polybothris alboplagiata*; 9. *Polybothris mucronata*; 10. *Polybothris solea*; 11. *Polybothris complanata*. **Plate 43**: 1. *Polybothris pyropyga*; 2. *Polybothris cyaneoviridis*; 3. *Polybothris viridicornis*; 4. *Polybothris viriditarsis*; 5. *Polybothris aeneomaculata* var.; 6. *Polybothris obscurella*; 7. *Polybothris puncticollis*; 8. *Polybothris moesta*; 9. *Polybothris quadrispilota*; 10. *Polybothris ruficauda*; 11. *Polybothris botrypiga*; 12. *Polybothris obscura*; 13. *Polybothris nossibiana*. **Plate 44**: 1. *Polybothris proserpina*; 2. *Polybothris sternalis*; 3. *Polybothris infrasplendens*; 4. *Polybothris pisciformis*; 5. *Polybothris subimpressa*; 6. *Polybothris aurocyanea*; 7. *Polybothris xanthostica*; 8. *Polybothris paraffinis*; 9. *Polybothris sycophanta*; 10. *Polybothris navicularis*; 11. *Polybothris luteomaculata*; 12. *Polybothris superba*. **Plate 45**: 1 et 2. *Macrotoma jejuna* ♂ et ♀; 3. *Macrotoma crassa*; 4. *Macrotoma laevis*; 5 et 6. *Platygnathus octangularis* ♂ et ♀; 7 et 8. *Closterus flabellicornis* ♂ et ♀. **Plate 46**: 1. *Hoploderes spinipennis*; 2. *Hoploderes grandidieri*; 3. *Tragocephala jucunda*; 4. *Tragocephala jucunda* var. *madagascariensis*; 5. *Tragocephala nobilis* var.; 6. *Tragocephala variegata*; 7. *Tragocephala variegata* var.; 8. *Tragocephala chevrolati*; 9. *Callimation venustum*; 10. *Protorrhopala elegans*. **Plate 47**: 1 et 2. *Macrotoma palmata* ♂ et ♀ var.; 3. *Leptocera coadunata*; 4. *Leptocera sericeovittata*; 5. *Xystrocera globosa*; 6 et 7. *Ceresium simplex* ♂ et ♀; 8. *Apharsatus fallaciosus*; 9. *Arrhytmus rugosipennis*; 10. *Logisticus rostratus*. **Plate 48**: 1. *Nethinius fulvipes*; 2. *Nethinius fulvipes* var.; 3. *Nethinius sanguinicollis*; 4. *Nethinius pallidipes*; 5. *Nethinius fulvescens*; 6. *Nesogena obscuripes*; 7. *Nethinius dimidiatipes*; 8. *Phelocalocera peregrina*; 9. *Charidamus hypargyreus*; 10. *Teraschema menalcas*; 11. *Philematium femorale*. **Plate 49**: 1 et 2. *Toxotinus sericeus* ♂ et ♀; 3. *Artelida crinipes*; 4. *Mastodontodera testacipes*; 5. *Mastodontodera coccinea*; 6. *Mastodontodera nodicollis*; 7. *Mastodontodera nodicollis* var *lateralis*; 8. *Mastodontodera transversalis*; 9. *Sagridola quinquemaculata*; 10 et 11. *Sagridola maculosa* ♀ et ♂. **Plate 50**: 1 et 2. *Lophoptera tridentata* ♂ et ♀; 3. *Protorrhopala sexnotata*; 4. *Praonetha obsoleta*; 5. *Micracantha madecassa*; 6. *Dioristus albolateralis*; 7. *Sternotomis thomsoni*; 8. *Sternotomis maculata*; 9. *Sternotomis cornutor*; 10. *Batocera rubus*. **Plate 51**: 1. *Leptocera lineatocollis*; 2. *Sulenus humeralis*; 3. *Eumimetes sparsus*; 4. *Eumimetes sparsus* var.; 5. *Eumimetes sexpunctatus*; 6. *Coptops bidens* var. *liturata*; 7. *Leucographus variegatus*; 8. *Leucographus albovarius*; 9. *Phymatosterna lacteoguttata*; 10. *Goephanes luctuosus*; 11. *Phryneta marmorea*; 12. *Atybe planti*. **Plate 52**: 1. *Leptocera lineatopunctata*; 2. *Leucographus rufofemorata*; 3. *Leucographus flavovittata*; 4. *Leucographus pulchra*; 5. *Arrythmus pallimembris*; 6. *Aedoeus geniculatus*; 7. *Sagridola flavicollis*; 8. *Anthribola femorata*; 9. *Zygocera albovirgulata*; 10. *Tomobrachyta nigroplagiata*; 11. *Logisticus latesulcatus*; 12. *Logisticus sesquivittatus*. **Plate 53**: 1. *Coptops bidens*; 2. *Coptops bidens* var. *chloroticus*; 3. *Opsamates dimidiatus*; 4. *Antigenes funebris*; 5. *Musius 4.nodosus*; 6. *Hoploderes rugicollis*; 7. *Ranova pictipes*; 8. *Leucographus variegatus* var. *pyramidalis*; 9. *Oopsis guttulata*; 10. *Exocentrus madecassus*; 11. *Closterus major*; 12. *Artelida asperata*; 13. *Artelida aurosericea*. **Plate 54**: 1. *Logisticus obscurus*; 2. *Lixus angustatus*; 3. *Logisticus simplex*; 4. *Logisticus modestus*; 5. *Mastodera jansoni*; 6. *Mastodera vidua*; 7. *Dysmalhosoma picipes*; 8. *Diadelia biplagiata*; 9. *Rhaphidopsis pulchra*; 10. *Tragocephala nobilis*; 11. *Lasiocercis fasciata*; 12. *Ancylistes bicuspis*.

### Appendix 11. List of Coleoptera illustrated on the plates of ‘*Expédition scientifique de Morée*’ (Brullé, 1832–1836)

Dates of publication for the plates are taken from the *Annales de la Société Entomologique de France* when known.

**Plate 33** [DP: n.d.]: *Cicindela oliviera* Br.; *Brachinus graecus* Dej.; *Brachinus nigricornis* Gebl.; *Procerus duponchelii* Dej.; *Zabrus graecus* Dej.; *Zabrus puncticollis* Br.; *Zabrus fontenayi* Sol.; *Zabrus ammophilus* Stev.; *Myas rugicollis* Br.; *Pristonychus elegans* Br.; *Accinopus minutus* Br.; *Calathus ovalis* Dej. **Plate 34** [DP: n.d.]: *Ditomus obscurus* Stev.; *Epomis dejeani* Sol.; *Ditomus robustus* Pareyss.; *Chlaenius gracilis* Sol.; *Olisthopus graecus* Br.; *Dinodes maillei* Sol.; *Agonum sordidum* Pareyss.; *Peryphus elongatus* Dej.; *Calathus graecus* Dej.; *Gyrinus dejeani* Br.; *Colymbetes dilatatus* Br.; *Gyrinus graecus* Br. **Plate 35** [DP: n.d.]: *Buprestis lapidaria* Br.; *Buprestis bory* Br.; *Buprestis laportea* Br.; *Elater* (*Menalotus* Eschscholtz) *fuscatocollis* Br.; *Elater* (*Menalotus* Eschs.) *villosus* Br.; *Elater* (*Dicronychus* Eschs.) *obesus* Br.; *Elater* (*Dicronychus* Eschs.) *messenius* Br.; *Elater* (*Cardiostes*. Eschs.) *ruficruris* Br.; *Elater* (*Agriotes* Eschs.) *rufipalpis* Br.; *Elater* (*Agriotes* Eschs.) *punctulatus* Br.; *Lampyris antiqua* Br.; *Lampyris zencheri* Germ. **Plate 36** [DP: n.d.]: *Telephorus discicollis* Br.; *Telephorus tibialis* Br.; *Telephorus bicolor* Br.; *Telephorus decolorans* Br.; *Telephorus sulcicollis* Br.; *Telephorus nigritarsis* Br.; *Telephorus femoralis* Br.; *Malachius dilaticornis* Germ.; *Malachius labiatus* Br.; *Hister graecus* Br.; *Hister godet* Br.; *Hister semi-aeneus* Br. **Plate 37** [DP: n.d.]: *Dasytes cribrarius* Br.; *Dasytes caelatus* Br.; *Dasytes serratus* Br.; *Dasytes striatulus* Br.; *Dasytes melanostoma* Br.; *Dasytes fuscipes* Br.; *Clerus lepidus* Br.; *Clerus favarius* Br.; *Clerus obliquatus* Br.; *Clerus quadripustulatus* Br.; *Silpha gibba* Br.; *Silpha orientalis* Br. **Plate 38** [DP: 8 August 1833]: *Ateuchus pius* Illig.; *Ateuchus affinis* Br.; *Ateuchus vetusus* Br.; *Onitis steven* Br.; *Onitis furcifer* Ross.; *Melolontha boryi* Br.; *Onthophagus ruficapillus* Br.; *Onthophagus nitidicollis* Br.; *Aphodius cribrarius* Br.; *Aphodius quadri-signatus* Br.; *Rhisotrogus vernalis* Br.; *Melolontha pilosa* Fab. **Plate 39** [DP: January–April 1832]: *Anisoplia arenaria* Br.; *Anisoplia flavipennis* Br.; *Anisoplia straminea* Br.; *Anisoplia hirtella* Br.; *Anisoplia lineolata* Br.; *Amphicoma chrysonota* Br.; *Amphicoma anemonina* Br.; *Amphicoma scutellata* Br.; *Amphicoma apicalis* Br.; *Amphicoma hirsuta* Br.; *Amphicoma humeralis* Br. **Plate 40** [DP: 1 April 1835]: *Pimelia quadricollis* Br.; *Pimelia graeca* Br.; *Akis* elongata Br.; *Heliodromus rotundatus* Br.; *Heliodromus angulatus* Br.; *Tentyria quadricollis* Br.; *Blaps producta* Br.; *Phylax gravidus* Br.; *Opatroides punctulatus* Br.; *Scaurus elegans* Br.; *Helops azureus* Br.; *Helops mori* Br.; *Gnaptor spinimanus*. **Plate 41** [DP: 1 April 1835]: *Cistela quadricollis* Br.; *Cistela armillata* Br.; *Cistela curvipes* Br.; *Oedemera femorata* Br.; *Cerocoma muhlfeldi* Gyll.; *Cantharis dives* Br.; *Cantharis vittata* Br.; *Meloe rugulosa* Br.; *Meloe cyanella* Br. var. *pannonica*; *Meloe cyanella* Br. var. *coerulans*; *Cantharis vesicatoria*; *Lytta dubia*. **Plate 42** [DP: 8 August 1833]: *Brachycerus ovatus* Br.; *Polydrosus armipes* Schönh.; *Phyllobius seladonius* Br.; *Tachyerges fulvitarsis* Br.; *Baridius angustus* Br.; *Phytonomus auro-lineatus* Br.; *Centorynchus scutellaris* Br.; *Liparus punctipennis* Br.; *Liparus graecus* Br.; *Larinus subcostatus* Br.; *Tychius elegans* Br.; *Otiorynchus impressipennis* Br. **Plate 43** [DP: 6 August 1834]: *Purpuricenus boryi* Br.; *Dorcadion virleti* Br.; *Dorcadion femoratum* Br.; *Saperda duponchelii* Br.; *Saperda flavescens* Br.; *Saperda baccueti* Br.; *Leptura bisignata* Br.; *Callidium sericeum* Fab.; *Leptura rufa* Br.; *Clytus scalaris* Br.; *Clytus nigripes* Br.; *Clytus bobelayei* Br. **Plate 44** [DP: 6 August 1834]: *Stenopterus gracilis* Br.; *Cassida inquinata* Br.; *Clythra maculicollis* Br.; *Clythra chalybaeicornis* Br.; *Clythra tibialis* Br.; *Chrysomela vernalis* Br.; *Chrysomela lepida* Br.; *Chrysomela flavo-cincta* Br.; *Galeruca elongata* Br.; *Coccinella distincta* Br.; *Coccinella pygmaea* Br.

### Appendix 12. List of Coleoptera illustrated on the plates of ‘*Iconographie du règne animal*’ (Guérin-Méneville, 1829–1837)

**Plate 3** [DP: 21 March 1829]: 1. *Therates basalis* d’Urv.; 2. *Megacephala carolina* Latr.; 3. *Trycondyla aptera* Latr.; 4. *Clenostoma ichneumoneum* Dej.; 5. *Colliuris modesta* Dej.; 6. *Manticora maxillosa* Latr.; 7. *Cicindela tenuipes* Guér.; 8. *Oxycheila tristis* Dej. **Plate 4** [DP: 21 March 1829]: 1. *Anthia 10 guttata* Latr.; 2. *Graphipterus multiguttatus* Latr.; 3. *Casnonia senegalensis* St.Farg. et Serv.; 4. *Brachinus jurinei* Dej.; 5. *Trichognathus marginatus* Latr.; 6. *Galerita americana* Latr.; 7. *Zuphium olens* Latr.; 8. *Helluo costatus* Latr.; 9. *Dripta ruficollis* Dej.; 10. *Agra splendida* Latr.; 11. *Lebia flavomaculata* Guér. **Plate 5** [DP: 21 March 1829]: 1. *Siagona europaea* Dej.; 2. *Oxystomus sancti-hilarii* Lat.; 3. *Scapterus guerini* Dej.; 4. *Enceladus gigas* Bon.; 5. *Apotomus rufus* Latr.; 6. *Dischirius thoracicus* Latr.; 7. *Morio simplex* Dej.; 8. *Acanthocelis ruficornis* Lat.; 9. *Ozaena rogerii* Dej.; 10. *Ditomus violaceus* Latr.; 11. *Ditomus calydonius* Latr.; 12. *Cyclosomus flexuosus* Latr. **Plate 6** [DP: 18 July 1829]: 1. *Harpalus tricolor* Guér.; 2. *Trigonotoma viridicollis* Dej.; 3. *Feronia navarica* Lat.; 4. *Feronia melanaria* Lat.; 5. *Cephalotes rufipes* Lat.; 6. *Patrobus rufipes*; 7. *Mormolyce phyllodes* Hag.; 8. *Zabrus gibbus* Lat.; 9. *Sphodrus terricola* Lat.; 10. *Feronia hottentota* Lat.; 11. *Licinus agricola*; 12. *Loricera pilicornis* Lat.; 13. *Cynthia abaxoïdes* Lat.; 14. *Panagoeus fulgipennis* Lat.; 15. *Omophron suturalis* Guér. **Plate 7** [DP: 18 July 1829]: 1. *Sphoeroderus nitidicollis* Chevrol.; 2. *Calosoma rufipenne* Dej.; 3. *Cychrus italicus* Bonelli; 4. *Pamborus alternans* Lat.; 5. *Pogonophorus*; 6. *Pelophila*; 7. *Carabus rutilans* Lat.; 8. *Pelecium cyanipes* Kirby; 9. *Masoroeus luxatus* Dej.; 10. *Bembidion*. **Plate 8** [DP: 12 September 1829]: 1. *Dytiscus lherminieri* Chevr.; 2. [*Dytiscus*] *serricornis* Payk.; 3. *Colymbetes*; 4. *Hydroporus planus* Latr.; 5. *Hygrobia hermanni* Latr.; 6. *Noterus crassicornis* Clairo.; 7. *Haliplus elevatus*; 8. *Gyrinus sulcatus* Dej. **Plate 9** [DP: 12 September 1829]: 1. *Oxyporus rufus* Lin.; 2. *Astrapoeus ulmineus* Oliv.; 3. *Staphylinus tataricus* Fisc.; 4. *Lathrobium elongatum* Lin.; 5. *Paederus ruficollis* Fab.; 6. *Procirrus lefeburi* Lat.; 7. *Evaesthetus*. 8. *Stenus biguttatus* Lin.; 9. *Stilicus*; 10. *Oxytelus tricornis* Fab.; 11. *Osorius brasiliensis* Guér.; 12. *Zyrophorus striatus* Leach. **Plate 10** [DP: 12 September 1829]: 1. *Prognatha rufipenne* Lat.; 2. *Coprophilus rugosus* Grav.; 3. *Lesteva dichroa* Latr.; 4. *Micropeplus maillei* Dej.; 5. *Aleochara canaliculata* Fab.; 6. *Lomechusa paradoxa* Grav.; 7. *Omalium blattoïdes* Grav.; 8. *Tachinus atricapillus* Fab.; 9. *Tachiporus marginatus* Grav. **Plate 11** [DP: 21 November 1829]: 1. *Buprestis bicolor* Lat.; 2. *Buprestis gigas* Fab.; 3. *Buprestis rubripennis* Guér.; 4. *Buprestis lalandii* Guér.; 5. *Aphanisticus emarginatus* Lat.; 6. *Trachys cruentata* Fab.; 7. *Melasis buprestoïde* Oliv. **Plate 12** [DP: 21 November 1829]: 1. *Eucnemis capucinus* Man.; 2. *Pterotarsus histrio* Latr.; 3. *Galba marmorata* Guér.; 4. *Adelocera chabannii* Guér.; 5. *Pachyderes ruficollis* Guér.; 6. *Cerophytum elateroïdes* Latr.; 7. *Throscus dermestoïdes* Latr.; 8. *Chelonarium undatum* Latr.; 9. *Cryptostoma denticornis* Fabr.; 10. *Lobaederus monilicornis* Guér.; 11. *Nematodes filum* Latr.; 12. *Hemirrhipus flabellicornis* Latr.; 13. *Ctenicera haematodes* Latr.; 14. *Elater plagiatus* Germ.; 15. *Campylus denticollis* Fischer; 16. *Phyllocerus flavipennis* Dej. **Plate 13** [DP: 3 April 1830]: 1. *Physodactylus henningii* Fisch.; 2. *Cebrio fuscus* Gory; 3. *Cebrio gigas*; 4. *Anelastes drurii* Kirby; 5. *Callirhipis goryi* Guérin; 6. *Callirhypis dejeanii* Lat.; 7. *Rhipicera cyanea* Guér.; 8. *Rhipicera*; 9. *Ptilodactyla elaterina* Illig.; 10. *Elodes pallidus* Lat.; 11. *Scyrtes*; 12. *Eubria palustris* Germ. **Plate 14** [DP: November–December 1829]: 1. *Lycus latissimus* Fab.; 2. *Dictyoptera sanguinea* Latr.; 3. *Omalisus suturalis* Geoff.; 4. *Drilus flavescens*; 5. [*Drilus flavescens*]; 6. *Drilus ruficollis* Dej.; 7. *Lampyris savignyi* Kirby; 8. *Amydetes* Hoff.; 9. *Cladophorus ruficollis* Guér.; 10. *Silis tricolor* Guér.; 11. *Malthinus biguttatus* Oliv.; 12. *Cordylocera antennata* Guér. **Plate 15** [DP: January 1835]: 1. *Malachius ruficollis* Fab.; 2. *Dasytes trifasciatus* Guér.; 3. *Zygia oblonga* F.; 4. *Melyris viridis*; 5. *Melyris abdominalis* F.; 6. *Pelecophora nigrolinata* Guér.; 7. *Cylidrus buquetii* Guér.; 8. *Tillus rubricollis* Guér.; 9. *Tillus unifasciatus* F.; 10. *Priocera*; 11. *Axiana*; 12. *Eurypus*; 13. *Thanasimus bombycinus* Chev.; 14. *Thanasimus formicarius* F.; 15. *Opilo*; 16. *Clerus olivierii* Chevr.; 17. *Clerus alvearius* F.; 18. *Necrobia violacea* L.; 19. *Necrobia ruficollis* F.; 20. *Enoplium viridipenne* Kirby. **Plate 16** [DP: January 1835]: 1. *Ptinus italicus* Chev.; 2. *Ptilinus serraticornis* Oliv.; 3. *Xyletinus pallens* Stev.; 3b–e. *Xyletinus pectinatus* Fab.; 4. *Ochina sanguinicollis* Ziegl.; 5. *Dorcatoma rubens* Chevr.; 6. *Gibbium scotias* Oliv.; 7. *Anobium pertinax* L.; 8. *Atractocerus molorchoides* Guér.; 9. *Hylecoetus javanus* Chevr.; 10. *Lymexylon navale* Oliv.; 11. *Cupes capitata* Fab.; 12. *Rhysodes costatus* Chevr. **Plate 17** [DP: January 1835]: 1. *Mastigus fuscus* Klug; 2. *Scydmoenus helwegii* Lat.; 3. *Scydmoenus hirticollis* Gyl.; 4. *Hololepta 4-dentata* F.; 5. *Hister mandibularis* Chev.; 6. *Hister 4-maculatus* L.; 7. *Sphoerites glabratus* F.; 8. *Necrophorus maritimus* Eschsch.; 9. *Necrophorus germanicus* L.; 10. *Silpha granigera* Chev.; 11. *Necrodes littoralis* L.; 12. *Necrophilus hydrophiloides* Eschsch.; 13. *Agyrtes castaneus* F.; 14. *Scaphidium nigripes* Chev.; 15. *Scaphidium 4-maculatum* Oliv. **Plate 18** [DP: January 1835]: 1. *Colobicus marginatus* Lat.; 2. *Thymalus marginicollis* Chev.; 3. *Thymalus limbatus* F.; 4. *Ips fasciata* Oliv.; 5. *Ips 4-punctata* Herbst; 6. *Nitidula peruviana* Guér.; 7. *Nitidula imperialis* F.; 8. *Cercus pulicarius* Lat.; 9. *Byturus tomentosus* F.; 10. *Dacne femoralis* Chevr.; 11. *Cryptophagus nigripennis* Payk.; 12. *Cryptophagus populi* Payk. **Plate 19** [DP: January 1835]: 1. *Aspidiphorus orbiculatus* Gyl.; 2. *Dermestes carnivorus* Fab.; 3. *Megatoma undata* Lat. F.; 4. *Megatoma trifasciata* F.; 5. *Attagenus serra* F.; 6. *Anthrenus capensis* F.; 7. *Nosodendron fasciculare* Ol.; 8. *Byrrhus alpinus* Gory; 9. *Byrrhus dennii* Kirby; 10. *Trinodes hirtus* F.; 11. *Heterocerus marginatus* Fab.; 12. *Byrrhus concolor* Sturm. **Plate 20** [DP: January 1835]: 1. *Potamophilus orientalis* Gory; 2. *Dryops prolifericornis* Fab.; 3. *Elmis volckmari* Panz.; 4. *Macronychus 4-tuberculatus* Mul.; 5. *Georissus pygmaeus* Gyl.; 6. *Elophorus nubilus* Fab.; 7. *Elophorus fennicus* Payk.; 8. *Hydrochus elongatus* Fab.; 9. *Ochthebius hybernicus* Curt.; 10. *Hydroena testacea* Curt.; 11. *Sperchoeus sulcatus* Gory; 12. *Spercheus emarginatus* Fab.; 13. *Globaria nitida* Guér.; 14. *Hydrophilus spinipennis* Gory; 15. *Sphoeridium dimidiatum* Gory. **Plate 21** [DP: 25 September 1830]: 1. *Ateuchus aegyptiorum* Latr.; 2. *Ateuchus sacer* Latr.; 3. *Circellium hemisphericum* Latr.; 4. *Coprobius viridis* Latr.; 5. *Eurysternus foedus* Guér.; 6. *Ontophagus rarus* Guér.; 7. *Ontophagus vacca* Latr.; 8. *Phaneus imperator* Chevrol.; 9. *Oniticellus formosus* Chevr.; 10. *Copris bellator* Chevr.; 11. *Aphodius bipunctatus* Fabr. **Plate 22** [DP: 25 September 1830]: 1. *Aegialia cornifrons* Guér.; 2. *Chiron grandis* Gory; 3. *Lethrus eversmannii* Falderman; 4. *Lethrus cephalotes* Fabr.; 5. *Geotrupes blakburnii* Fabr.; 6. *Ochodeus rufus* Guér.; 7. *Athyreus castaneus* Guér.; 8. *Bolboceras fulvus* Gory; 9. *Trox aeger* Guér.; 10. *Hybosorus arator* Fabr. **Plate 23** [DP: 25 September 1830]: 1. *Oryctes chevrolatii* Guér.; 2. *Agacephala furcata* Guer.; 3. *Scarabaeus mentor* Guer.; 4. *Scarabaeus gedeon* Fabr.; 5. *Phileurus cribratus* Chevr.; 6. *Hexodon reticulatus* Olivier; 7. *Cyclocephala frontalis* Chevr.; 8. *Cyclocephala geminata* Fabr. **Plate 24** [DP: 25 September 1830]: 1. *Chrysophora chrysochlora* Latr.; 2. *Rutela nitidissima* Guér.; 3. *Macraspis splendida* Fab.; 4. *Chasmodia brunnea* Serv. et St.Farg.; 5. *Ometis pictus* Guér.; 6. *Pachypus excavatus* Fabr.; 7. *Amblyteres geminatus* Mack.Leay. **Plate 24bis** [DP: 26 February 1831]: 1. *Leucothyreus nitidicollis* Guér.; 2. *Anoplognathus latreillei* Sch.; 3. *Geniates barbatus* Kirby; 4. *Apogonia gemellata* Kirby; 5. *Melolontha flavida* Gory; 6. *Melolontha vulgaris*; 7. *Rhisotrogus pini* Latr.; 8. *Ceraspis decora* Gory; 9. *Ceraspis albida* Serv.; 10. *Areoda kirbii* Mac.Leay; 11. *Serica flavimana* Gory; 12. *Serica variabilis* Lat.; 13. *Diphucephala furcata* Guér.; 14. *Macrodactylus suturalis* Chevr. **Plate 25** [DP: 15 January 1831]: 1. *Plectris tomentosa* Lep. et Serv.; 2. *Popilia nitidicollis* Gory; 3. *Anisoplia suturalis* Guér.; 4. *Euchlora viridana* Guér.; 5. *Lepisia rupicola* Serv.; 6. *Dicrania velutina* Gory; 7. *Hoplia farinosa* Fabr.; 8. *Dichelus dentipes* Serv. **Plate 25bis** [DP: 26 February 1831]: 1. *Glaphyrus rufipennis* Gory; 2. *Amphicoma bombyliformis* Pall.; 3. *Amphicoma lasserii*; 4. *Anthipna abdominalis* Esch.; 5. *Chasmopterus hirtulus* Sllig.; 6. *Pachycnemus crassipes* Serv.; 7. *Lepitrix abbreviatus* Serv.; 8. *Monochelus gonager* Serv.; 9. *Anisonyx nasua* Wied. **Plate 26** [DP: 15 January 1831]: 1. *Cremastocheilus hirtus* Gory et Perch.; 2. *Cremastocheilus elongatus* Oliv.; 3. *Trichius zebra* Oliv.; 4. *Trichius fasciatus* F.; 5. *Goliath micans* G. et P. Oliv.; 6. *Platygenia zaïrica* Mack.Leay; 7. *Cetonia baxii* G. et P.; 8. *Cetonia aurata* F.; 9. *Gymnetis nervosa* G. et P.; 10. *Macronota aegregia* G. et P. **Plate 27** [DP: 15 January 1831]: 1. *Sinodendron cylindricum* L. Fab.; 2. *Oesalus scaraboeides* Fab.; 3. *Lucanus cinnamomeus* Guér.; 4. *Platycerus auriculatus* Gory; 5. *Lamprima aenea* Latr.; 6. *Pholidotus humboldii* Sch.; 7. *Passalus pentaphyllus* Palis-Bauv.; 8. *Passalus interruptus* Fab. **Plate 28** [DP: 15 January 1831]: 1. *Pimelia vestita* Gory; 2. *Pimelia sericea*; 3. *Erodius gibbus* Fabr.; 4. *Zophosis testudinarius* Oliv.; 5. *Nyctelia luczotii* Chevr.; 5c–d. *Nyctelia brunnipes* Latr.; 6. *Hegeter tagenioïdes* Gory; 7. *Tentyria punctipennis* Lefebvre; 8. *Akis goryi* Guér.; 9. *Elenophorus americanus* Lacord.; 10. *Eurychora rugosula* Guér.; 11. *Eurichora ciliata*; 12. *Adelostoma rugosa* Gory. **Plate 28bis** [DP: 16 April 1831]: 1. *Tagenia orientalis* Gory; 2. *Psammeticus costatus* Guér.; 3. *Scaurus rugosus* Latr.; 4. *Scotobius granosus* Lacord.; 5. *Sepidium vestitum* Gory; 6. *Trachynotus vittatus* Latr.; 7. *Moluris luteipes* Guér. **Plate 29** [DP: 16 April 1831]: 1. *Oxura setosa* Kirby; 2. *Acanthomera gratilla* Guér. Herbst; 3. *Misolampus hoffmansegii* Latr.; 4. *Blaps mortisaga* Fab.; 5. *Blaps sulcata* Fab.; 6. *Gonopus tibialis* Latr.; 7. *Anomalipes dentipes* Lat. Fab.; 8. *Machla villosa* Herbst; 9. *Scotinus brasiliensis* Gory; 10. *Asyda laevigata* Fab.; 11. *Opatrinus chlatratus* Dej.; 12. *Heliophilus hispanicus* Dej.; 13. *Pedinus gibbosus* Gory; 14. *Bapstinus punctatus* Sch.; 15. *Platyscelis gages* Fisch. **Plate 30** [DP: 16 April 1831]: 1. *Crypticus gibbulus* Sch.; 2. *Opatrum elongatum* Guér.; 3. *Corticus celtis* Germ.; 4. *Orthocerus muticus* Lat.; 5. *Chiroscelis bifenestrata* Lam.; 6. *Toxicum curvicorne* Chev.; 7. *Boros thoracicus* Gyl.; 8. *Calcar elongatus* Herbst; 9. *Upis ceramboïdes* Fab.; 10. *Tenebrio molitor* Lin.; 11. *Heterotarsus tenebrioïdes* Guér.; 12. *Tenebrio obscurus* Fab. **Plate 31** [DP: 23 March 1833]: 1. *Diaperis bipustulata* Brul. Lap.; 2. *Hyppophloeus castaneus* Fab.; 3. *Trachyscelis aphodioïdes* Lat.; 4. *Leiodes cinnamomea* Panz.; 5. *Tetratoma fungorum* Fab.; 6. *Eledona cornuta* Fab.; 7. *Cossyphus moniliferus* Chevr.; 8. *Nilio lanatus* Germ.; 9. *Epitragus lineatus* Chevr.; 10. *Cnodalon atrum* Chevr.; 11. *Spheniscus pictus* Chevr. **Plate 32** [DP: October 1834]: 1. *Amarygmus cuprinus* Esch.; 2. *Sphaerotus curvipes* Kirby; 3. *Helops suturalis* Germ.; 4. *Helops lanipes* F.; 5. *Loena pimelia* F.; 6. *Stenotrachelus aeneus* Latr.; 7. *Strongylium serraticorne* Chevr.; 8. *Pitho depressus* F.; 9. *Cistela serrata* Chevr.; 10. *Hallomenus humeralis* Latr.; 11. *Orchesia micans* Latr. **Plate 33** [DP: October 1834]: 1. *Dircaea discolor* Fab.; 2. *Melandrya rufipes* Chevr.; 3. *Serropalpus striatus* Latr.; 4. *Conopalpus flavicolle* Gyl.; 5. *Calopus serraticornis* F.; 6. *Dytilus laevis* Fisch.; 7. *Aedemera podagrariae* F.; 8. *Stenostoma rostratum* Charp.; 9. *Mycterus pulverulentus* Chevr.; 10. *Rhinomacer attelaboïdes* F.; 11. *Rhinosimus ruficollis* F. **Plate 34** [DP: October 1834]: 1. *Lagria gigas* Guér.; 2. *Statira caraboïdes* Guér.; 3. *Pyrochroa coccinea* Fab.; 4. *Ripiphorus rufipennis* Chevr.; 5. *Myodites americanus* Guér.; 6. *Pelecotoma frivaldjskii* Sturm; 7. *Mordella picta* Chevr.; 8. *Scraptia dubia* Oliv.; 9. *Notoxus fasciatus* Chevr.; 10. *Cissites testaceus* Latr. **Plate 35** [DP: October 1834]: 1. *Cerocoma schaefferi* Lin.; 2. *Hycloeus 10guttatus* Chevr.; 3. *Aritnoema 12punctata* Chevr.; 4. *Mylabris myops* Chevr.; 5. *Oenas affer* F.; 6. *Meloe cordillierae* Chevr.; 7. *Meloe brevicollis* Panz.; 8. *Tetraonyx ventralis* Chevr.; 9. *Cantharis sulcifrons* Chevr.; 10. *Cantharis vessicatoria*; 11. *Zonitis puncticollis* Chevr.; 12. *Nemognatha chrysomelina* F.; 13. *Leptopalpus chevrolatii* Guér.; 14. *Gnathium flavicolle* Chevr.; 15. *Sitaris humeralis* Fab. **Plate 36** [DP: 6 July 1833]: 1. *Bruchus marginellus* Fabr.; 2. *Rhaebus gebleri* Fisch.; 3. *Anthribus garnotii* Guer.; 4. *Attelabus falcatus* Guer.; 5. *Rhinotia hoemoptera* Kirby; 6. *Eurhinus conicus* Guer.; 7. *Brentus italicus* Latr.; 8. *Ceocephalus furcillatus* Sch.; 9. *Ulocerus cinereus* Latr.; 10. *Cylas longicollis* Chevr. **Plate 37** [DP: 6 July 1833]: 1. *Brachycerus oculatus* Chevr.; 2. *Cyclomus coronatus* Sch.; 3. *Cyphus illustris* Chevr.; 4. *Cyphus dives* ? Illig.; 5. *Leptocerus macilentus* Chevr.; 6. *Pachyrhynchus profanus* Esch.; 7. *Syzygops cyclops* Sch.; 8. *Rhytirrhinus informis* Chevr.; 9. *Cleonus guttatus* Chevr.; 10. *Lixus vittiger* Godet; 11. *Chlorophanus viridis* Sch. **Plate 38** [DP: 6 July 1833]: 1. *Loemosaccus chevrolatii* Guer.; 2. *Bagous binodosus* Gyll.; 3. *Brachonyx indigena* Gyll.; 4. *Balaninus nucum* Lin.; 5. *Heilipus peplus* Sch.; 6. *Alcides preustus* Guer.; 7. *Myorhinus albolineatus*; 8. *Cionus pulverosus* Pareys; 9. *Tachygonus horridus* Chevr. **Plate 39** [DP: September 1833]: 1. *Cholus flavo-fasciatus* Chevr.; 2. *Camptorhynchus flattuarius* Germ.; 3. *Centrinus curvirostris* Chevr.; 4. *Zygobs rubricollis* Chevr.; 5. *Ceutorhynchus sii* Chevr.; 6. *Hydaticus comarii* Sch.; 7. *Diorymerus altus* Germ.; 8. *Mecopus trilineatus* Guer.; 9. *Gorgus bispinosus* Chevr.; 10. *Tylodes ptinioides* Gyl. **Plate 39bis** [DP: September 1833]: 1. *Anchonus suillus* Fab.; 2. *Rhina barbirostris* Fab.; 3. *Calandra guerini* Chevr.; 4. *Calandra taitense* Guer.; 5. *Belorhynus acutus* Guer.; 6. *Cercidocerus nigrolateralis* Guer.; 7. *Cossonus ephippiger* Guer.; 8. *Dryophtorus lymexilon* Fab.; 9. *Trigonotarsus calandroides* Guer. **Plate 40** [DP: 21 March 1835]: 1. *Scolytus flavicornis* Chev.; 2. *Hylurgus piniperda*; 3. *Camptocerus aeneipennis* Ol.; 4. *Phloiotribus oleae* Lat.; 5. *Tomicus bispinus* Meg.; 6. *Platypus poeyi* Guér.; 7. *Platypus cylindrus* Fab.; 8. *Paussus curvicornis* Chev.; 9. *Paussus microcephalus*; 10. *P.* et *Pentaplatarthrus*; 11. *Platyrhopalus mellei* West.; 12. *Cerapterus latipes* West.; 13. *Bostrichus dufourii* Lat.; 14. *Cis inaequidens* Chev.; 15. *Nemosoma elongatum* L.; 16. *Synchita undata* Guér. **Plate 41** [DP: September 1837]: 1. *Rhyzophagus ephippiger* Guér.; 2. *Monotoma conicicollis* Chevr.; 3. *Lyctus glycirrhizae* Chevr.; 4. *Diodesma subterranea* Dej.; 5. *Bitoma unicolor* Guér.; 6. *Bitoma crenata* Herbst; 7. *Biphyllus fagi* Chevr.; 8. *Dasycerus sulcatus* Brong.; 9. *Latridius lilliputanus* Villa; 10. *Latridius elongatus* Curtis; 11. *Sylvanus zimmermanni* Guér.; 12. *Trogossita crenicollis* Guér.; 13. *Prostomis mandibularis* Fab.; 14. *Passandra brasiliensis* Chevr. **Plate 42** [DP: 21 May 1831]: 1. *Cucujus mandibularis* Gory; 2. *Cucujus depressus* Fab.; 3. *Brontes spinicollis* Gory; 4. *Brontes flavipes* Fab.; 5. *Dendrophagus crenatus* Payk.; 6. *Spondylus buprestoides* Fab.; 7. *Parandra lineola* Gory; 8. *Prionus desmarestii* Guér.; 9. *Anacolus sanguineus* Serv.; 10. *Prionapterus staphilinus* Guér. **Plate 43** [DP: 21 May 1831]: 1. *Lissonotus unifasciatus* Gory; 2. *Megaderus stigma* Fab.; 3. *Trachyderes nigrofasciatus* Gory; 4. *Trachyderes succinctus* Fab.; 5. *Lophonocerus barbicornis* Oliv.; 6. *Callichroma speciosa* Gory; 7. *Acanthopterus tripunctatus* Gory; 8. *Acanthopterus budensis* Goeze; 9. *Cerambyx heros* Fab.; 10. Cerambyx (Callichroma) hemipterus Oliv. **Plate 44** [DP: 21 May 1831]: 1. *Cerambyx rufipennis* Gory; 2. *Cerambyx speculifer* Gory; 3. *Cerambyx hirtipes* Oliv.; 4. *Callidium insubrecum* Ziégler; 5. *Certallum ruficolle* Fab.; 6. *Obrium ferrugineum* Fab.; 7. *Rhinotragus coccineus* Gory; 8. *Necydalis major* Lin.; 9. *Stenopterus elegans* Klug. **Plate 45** [DP: April–June 1831]: 1. *Acrocinus trochlearis* Gory; 2. *Lamia aurocincta* Gory; 3. *Tetraopes dimidiata* Gory; 4. *Saperda albicans* Guér.; 5. *Saperda atkinsoni* Curtis; 6. *Distichocera maculicollis* Kirby; 7. *Tmesisternus bizonulatus* Guér.; 8. *Tragocerus bidentatus* Donov.; 9. *Leptocera bilineata* Gory. **Plate 46** [DP: April–June 1831]: 1. *Desmocerus cyaneus* Fab.; 2. *Vesperus graecus* Guér.; 3. *Rhagium bifasciatum* Fab.; 4. *Rhamnusium salicis* Fab.; 5. *Toxotus meridianus* Fab.; 6. *Pachyta laportii* Guér.; 7. *Stenoderus ceramboïdes* Kirby; 8. *Leptura annulata* Gory. **Plate 47** [DP: December 1833]: 1. *Megalopus unifasciatus*? Gory; 2. *Sagra cyanea* Dalm.; 3. *Sagra splendida* F.; 4. *Orsodacna violacea* Chevr.; 5. *Psammaechus bipunctatus* F. Boudier; 6. *Donacia fennica* Gyll.; 7. *Donacia sagittariae* Fab.; 8. *Haemonia zosterae* Gyll.; 9. *Petauristes crassipes* Oliv.; 10. *Crioceris dorycus* Guér.; 11. *Auchenia betulae* Fab.; 12. *Megascelis prasina* Chevr. **Plate 48** [DP: December 1833]: 1. *Alurnus corallinus* Vigors; 2. *Oxycephala cornigera* Guér.; 3. *Hispa fabricii* Guér.; 4. *Chalepus spinipes* Fab.; 5. *Cassida* (*Cyclosoma* Chevr.) *longicornis* Fab.; 6. *Cassida discors* Fabr.; 7. *Cryptocephalus speciosus* Guér.; 8. *Clythra* Percheron Gory; 9. *Chlamys cuprea* Klug; 10. *Lamprosoma corrusca* Gory; 11. *Choragus scheppardi* Kirby; 12. *Euryope 4-maculata* Oliv.; 13. *Eumolpus cyaneus* Fab. **Plate 49** [DP: December 1833]: 1. *Colaspis illustris* Chevr.; 2. *Podontia affinis* Germ.; 3. *Phyllocharis bicincta* Guér.; 4. *Phyllocharis splendens* Guér.; 5. *Doryphora multipunctata* Chevr.; 6. *Doryphora adunca* Chevr.; 7. *Paropsis 8-lineata* Gory; 8. *Timarcha balearica* Gory; 9. *Chrysomela humeralis* Gory; 10. *Chrysomela sanguinolenta* L.; 11. *Phaedon cyanopterus* Guér.; 12. *Prasocuris hanoveriana* Fab. **Plate 49bis** [DP: December 1834]: 1. *Adorium basale* Guér.; 2. *Adorium bipunctatum* Oliv.; 3. *Galeruca dimidiata* Guér.; 4. *Galeruca viburni* Payk.; 5. *Galeruca 4 maculata* L.; 6. *Luperus cinctellus* Chevr.; 7. *Luperus brassicae* Panz.; 8. *Octogonotes 4-lineatus* Chevr.; 9. *Octogonotes thoracicus* Bosc; 10. *Oedionychis figuratus* Chevr.; 11. *Psylliodes anglica* Chevr.; 12. *Dibolia borealis* Chevr.; 13. *Altica chevrolatii* Guér.; 14. *Longitarsus dorsalis* Fab. **Plate 50** [DP: December 1834]: 1. *Erotylus bengalensis* Guér.; 2. *Aegythus surinamensis* Fab.; 3. *Triplax brunnipes* Chevr.; 4. *Triplax nigripennis* Fab.; 5. *Languria africana* Chevr.; 6. *Phalacrus granulatus* Guér.; 7. *Eumorphus hamatus* Guér.; 8. *Dapsa trimaculata* Megerle; 9. *Endomychus tibialis* Chevr.; 10. *Lycoperdina bovistae* Fab.; 11. *Lycoperdina lata* Chevr.; 12. *Lithophilus ruficollis* Dahl. **Plate 51** [DP: January 1835]: 1. *Coccinella furcifera* Guér.; 2. *Coccinella ocellata* Lin.; 3. *Cacidula litura* Fab.; 4. *Scymnus 4-nottatus* Illig.; 5. *Clypeaster pusillus* Gyl.; 6. *Metopias curculionoïdes* Gory; 7. *Chennium*; 8. *Dionix*; 9. *Bythinus*; 10. *Pselaphus heisei* Herbst; 11. *Bryaxis lefebvrii* Aubé; 12. *Bryaxis longicornis* Leach; 13. *Bryaxis antennata* Aubé; 14. *Euplectus kirbii* Denny; 15. *Euplectus nanus* Reich.; 16. *Claviger foveolatus* Mull.; 17. *Ptillium fasciculare* Herbst.
